# Alternative splicing drives a dynamic transcriptomic response during *Acanthamoeba castellanii* programmed cell death

**DOI:** 10.15698/mic2025.08.858

**Published:** 2025-08-26

**Authors:** Jesús Gómez-Montalvo, Zisis Koutsogiannis, Sutherland K Maciver, Alvaro de Obeso Fernández del Valle

**Affiliations:** 1 Tecnologico de Monterrey, Escuela de Ingeniería y Ciencias, Ave. Eugenio Garza Sada 2501, 64849, Monterrey, N.L, Mexico.; 2 Centre for Discovery Brain Sciences, Edinburgh Medical School, Biomedical Sciences, University of Edinburgh, Hugh Robson Building, George Square, Edinburgh, EH8 9XD, Scotland, UK.

**Keywords:** Acanthamoeba castellanii, programmed cell death, alternative splicing, intron retention, transcriptomics

## Abstract

Programmed cell death (PCD) in unicellular organisms is not well characterized. This study investigated the transcriptomic response of *Acanthamoeba castellanii* to G418-induced PCD, focusing on the role of alternative splicing (AS). RNA sequencing revealed extensive transcriptional changes, affecting approximately 70% of annotated genes over six hours of treatment. This analysis also highlighted significant alterations in pathways related to cell cycle, proteolysis, and RNA splicing. Analysis of AS events identified 18,748 differentially spliced events, predominantly intron retention (IR). Interestingly, retained introns displayed a 3′ bias in untreated cells, a pattern that shifted towards uniform distribution throughout the gene body during PCD. Additionally, we characterized retained introns during trophozoite stage and during PCD of the amoeba. Correlational analysis revealed a significant negative correlation between IR and transcript levels, suggesting a complex interplay between transcriptional and post-transcriptional regulation. The predominance of IR, coupled with its dynamic positional shift during PCD, points to a novel regulatory mechanism in *A. castellanii* PCD. These findings provide insights into the molecular mechanisms underlying PCD in this organism, potentially identifying new therapeutic targets and allowing us a better understanding of such process in *A. castellanii*, a facultative human pathogen.

## Abbreviations

A3SS - alternative 3’ splice sites

A5SS - alternative 5’ splice sites,

ACD - accidental cell death,

AS - alternative splicing,

DE - differentially expressed,

GO - gene ontology,

IR - intron retention,

MXE - mutually exclusive exons,

NMD - nonsense-mediated decay,

PCD - programmed cell death,

SE - exon skipping.

## INTRODUCTION

Cell death occurs when cells lose the ability to maintain their major biological function and is generally categorized into two types, accidental cell death (ACD) and regulated cell death (Programmed cell death or PCD) 1. Contrary to uncontrolled and unregulated ACD, PCD is strictly controlled by signaling cascades in which effector molecules are actively interacting with each other. In higher eukaryotes cell death is very common and tightly regulated and influenced by a variety of signaling cascades and pathways that coordinate from developmental to stress-related signals, to ensure proper cellular function, and tissue integrity 1,2. These pathways include, but are not limited to, autophagy, oxidative stress and the proteasome-ubiquitin system 3.

PCD in unicellular organisms is perhaps counterintuitive, but evidence of this process has been documented in various protozoan unicellular organisms, including *Saccharomyces* 4,5, slime molds such as *Dictyostelium* 6, parasitic protozoans including *Plasmodium* 7, *Trypanosoma* 8,9,10,11*, Leishmania* 12 and *Entamoeba* 13. In parallel, different forms of PCD have been also observed in prokaryotes 14,15,16.

The free-living amoeba* Acanthamoeba *has been reported to undergo a form of regulated cell death 17,18,19, although the mechanism that controls and orchestrates this phenomenon is yet not fully understood. Even though *Acanthamoeba* PCD shares characteristics with higher eukaryotes PCD, implying a common origin, these are not results of the same biological effectors. Important enzymes such as caspases and the Bcl-2 family genes that strictly regulate cell death in the latter have not been identified in protists thus far.

Various compounds have been studied for their ability to induce PCD in *Acanthamoeba*. Doxorubicin causes apoptotic-like characteristics such as cell shrinkage and membrane blebbing 20. Caffeine and maslinic acid can trigger PCD during the encystment stage of *Acanthamoeba*, acting as inhibitors of encystation-involved glycogen phosphorylase 21. Common drugs used against *Acanthamoeba* infections, such as polyhexamethylene biguanide and chloroquine, are also capable of inducing PCD 18,21. Other compounds and microorganisms capable of inducing PCD in *Acanthamoeba* include statins, voriconazole, pitavastatin nanoparticles, oleic acid, olive leaf extracts, *Salmonella choleraesuis*, and *Streptomyces sanyensis* 17,18,22,23,24,25,26. We have previously shown that the aminoglycoside G418 induces PCD in *Acanthamoeba* through elevation of intracellular calcium and cytochrome c translocation, although there is no evidence of genomic DNA breakdown in the early stages 19. In other protists, such as *Tetrahymena thermophila*, G418 inhibited protein synthesis at an early stage of peptide elongation 27. Furthermore, since G418 is known to elicit PCD in other amoebae, such as *Entamoeba histolytica* 13, this compound could be used to expand on the observations regarding the biology of PCD across amoebae and possibly other protists.

Transcriptomics is a promising area for studying PCD, with alternative splicing (AS) being one of the least studied aspects that can shed light on the processes occurring during PCD 28. AS encompasses various processes through which organisms create diverse transcripts, proteins, and phenotypes 29. AS events include intron retention (IR), exon skipping (SE), alternative 5′ (A5SS) and 3′ (A3SS) splice sites, and mutually exclusive exons (MXE) 29,30. Although IR has been widely studied in other organisms, such as plants or mammals, it has been poorly investigated in most protozoans. For *Acanthamoeba*, there have been few reports of IR. A previous study of 65 eukaryotic species reported IR and SE in *Acanthamoeba* 31. Additionally, IR has been shown to occur during *A. castellanii* encystment 32. In other organisms, IR has been related to PCD through tumor suppressor inactivation 33, and PCD has been reported to be enriched in IR clusters 34. Other types of AS have been poorly studied in *Acanthamoeba*, and only broadly described 31.

The *A. castellanii* Neff strain genome has a size of 42.02 Mb and has been sequenced (ENSEMBL assembly: GCA000313135v1) 35,36,37. It contains 14,977 reported protein-coding genes 35,36,38. Each protein-coding gene has an average of 6.2 introns 36,39.

Understanding the molecular pathways and triggers involved in PCD in *Acanthamoeba* is crucial for elucidating host-parasite interactions and developing effective therapeutic strategies against *Acanthamoeba*-related infections. By unraveling the mechanisms through which *A. castellanii* initiates PCD, researchers can gain insights into potential targets for therapeutic interventions aimed at disrupting the parasite's survival strategies and enhancing host immune responses against infection. In this study, we conducted RNA-seq of trophozoites treated for 1, 3 and 6 hours with the aminoglycoside G418. We explored changes in transcript levels and the occurrence of AS events to gain deeper understanding of the molecular mechanisms taking place during *Acanthamoeba* PCD.

## RESULTS

### G418 treatment prompts extensive changes in gene expression

The *A. castellanii* Neff strain genome (ENSEMBL assembly: GCA000313135v1) was used to map the sequencing reads. Quality control analysis revealed that most reads were uniquely mapped (Figure S1A) and were primarily associated with exons (Figure S1B), supporting the high quality and reliability of the mapping step.

**Figure 1:**
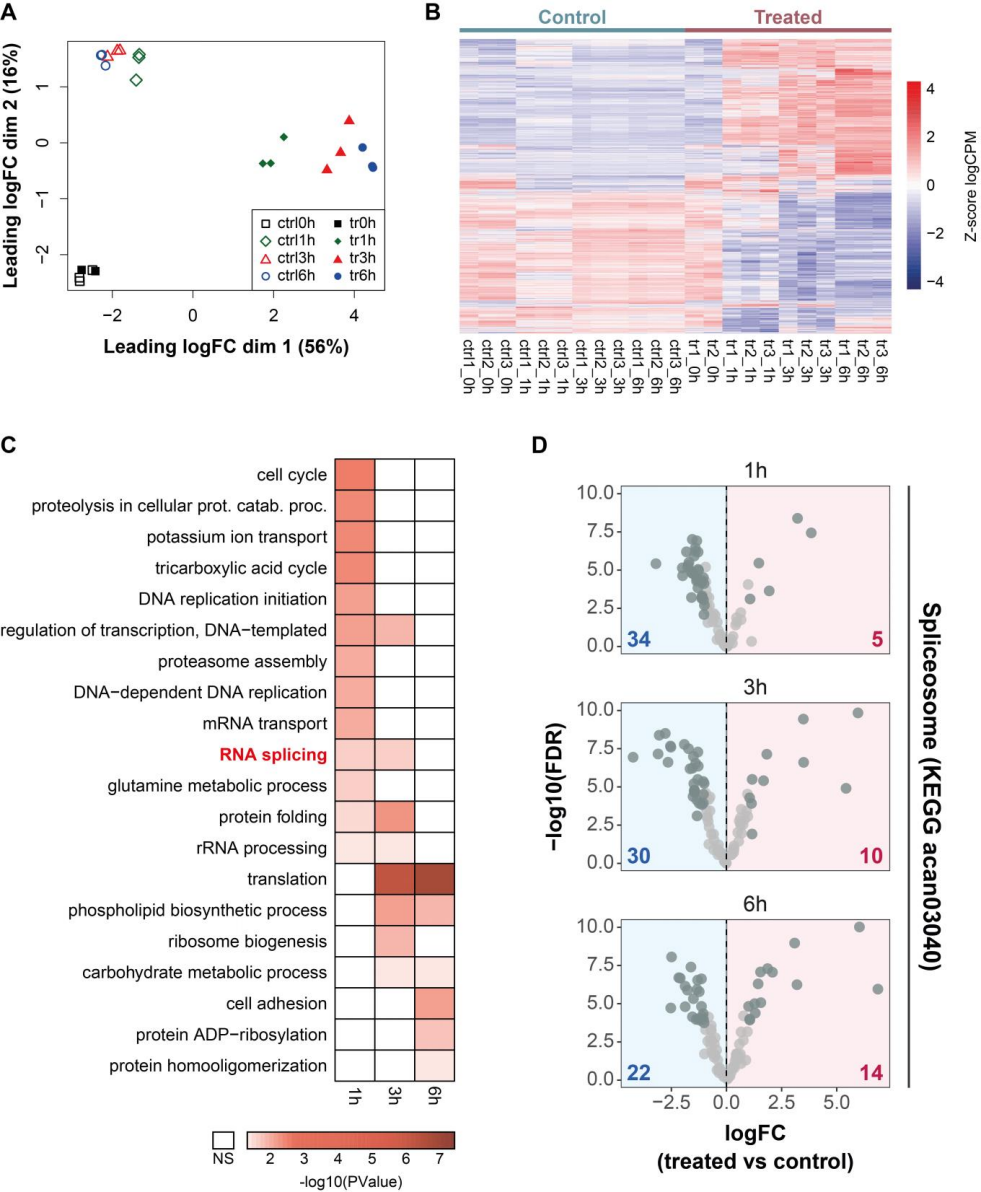
FIGURE 1: A dynamic transcriptional program occurs during G418-induced *A. castellanii* programmed cell death. **(A) **MDS plot displaying clustering of control (empty shapes) and treated (filled shapes) samples at different timepoints. **(B) **Heatmap displaying the expression level of the 10818 differentially expressed (DE) genes identified across all treatment times. Gene expression levels are shown as Z-score-normalized log2CPM (Counts Per Million) values. **(C)** Gene ontology analysis of the DE genes in **B**. Colored boxes in the heatmap indicate significant terms (PValue < 0.05, Fisher’s exact test). NS, Not Significant. **(D)** Volcano plots showing changes in the expression of 88 spliceosome genes. The number of differentially expressed spliceosome genes (dark gray dots, |logFC| > 1 and FDR < 0.05) is indicated at the bottom of each plot.

Multidimensional scaling (MDS) analysis for gene expression clearly separated treated from untreated samples along the first dimension (**Figure 1A**), except for 0 h samples, which clustered together. In fact, comparison between 0 h treated and control samples did not reveal changes in transcript levels of any genes. We found that the number of differentially expressed (DE) genes (|logFC| > 1 and FDR < 0.05) increased with treatment time (**Figure 1B**). Overall, G418 treatment altered the expression of 10,818 genes or approximately 70% of all annotated *A. castellanii* genes.

Gene ontology (GO) analysis showed that genes altered after G418 treatment were significantly enriched in processes such as cell cycle, proteolysis, proteasome assembly, and cell adhesion (**Figure 1C**). Manual inspection of our lists of DE genes revealed that the ACA1_087710 gene, which is annotated as an ICElike protease (Caspase) p20 domain containing protein and commonly referred to as *A. castellanii* metacaspase 40, displayed increased expression with treatment time (Figure S2). In addition, we analyzed the expression of genes involved in pathways and processes known to influence PCD, such as autophagy, the proteasome, the ubiquitin system, and oxidative stress. To explore the latter, we analyzed genes associated with oxidative phosphorylation. We found that G418 treatment altered the expression of genes in all these processes (Figure S3, left), with the proteasome and oxidative phosphorylation being mainly downregulated and the ubiquitin system showing an almost equal number of up- and downregulated genes.

RNA splicing was revealed to be an altered process during *Acanthamoeba* PCD (**Figure 1C**, highlighted in red). In fact, when checking in detail the expression of spliceosome genes we found that many of them were altered (**Figure 1D**). Specifically, after 1 and 3 hours of treatment most spliceosome genes showing significant changes decreased their expression and it was until 6 hours of treatment that various of these genes increased their expression levels. For instance, pre-mRNA splicing factors annotated as PRP proteins, such as PRP3 (ACA1_264860), PRP16 (ACA1_369140), and PRP38 (ACA1_062870), displayed altered transcript levels following G418 treatment. PRP16 transcript levels were downregulated after 1 h of treatment (logFC = -1.34), whereas PRP3 and PRP38 showed increased expression only after 6 h (logFC = 1.002 and 1.43, respectively). DEAH box RNA helicases, such as ACA1_338770 and ACA1_224690, were among the top upregulated spliceosome genes after 6 h (logFC = 6.87 and 3.1, respectively). On the other hand, genes encoding the U6 snRNA-associated proteins LSm4 (ACA1_097170) and LSm8 (ACA1_290470) displayed decreased transcript levels after 3 h and 6 h, respectively (logFC = -1.12 and -1.02). Together, these results suggest that altered expression of spliceosome components is part of the transcriptional program of *Acanthamoeba* PCD.

### AS events occur during *A. castellanii* PCD

**Figure 2:**
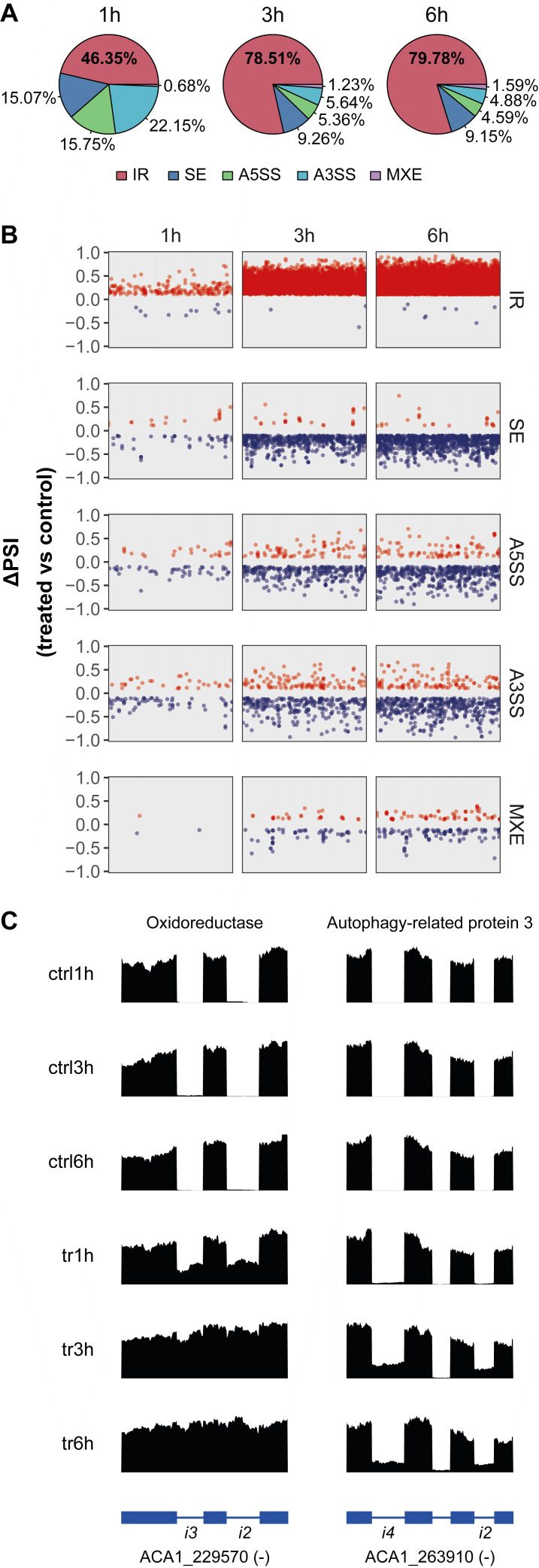
FIGURE 2: Alternative Splicing (AS) events abound during *Acanthamoeba* programmed cell death. **(A)** Pie charts displaying the proportion of AS events identified after 1h, 3h and 6h of treatment. **(B) **The figure shows a summary of the AS analysis. Each dot represents one event. Red dots are upregulated while blue dots represent downregulated events. The horizontal distribution is only to facilitate the visualization of the events, which are grouped by gene on the X-axis. The Y-axis represents the level of change for each event, where a positive or negative value means that the event was either more or less included in the final mature transcript, respectively. For instance, a positive value of 0.5 means that the event was included in 50% more transcripts. No dots are found from -0.1 and 0.1 since the events within this range were not considered significant. In the case of IR, the ΔIR ratio calculated using the IRFinder algorithm is shown. PSI, Percent Spliced In. **(C)** RNA-seq tracks showing increased IR in ACA1_229570 (Oxidoreductase) and ACA1_263910 (Autophagy-related protein 3). Exons are shown as boxes and introns as lines. Introns with increased retention are labeled. The minus (-) sign next to the gene identifier indicates that the gene is located in the reverse strand. Note the absence of read density within the introns in the control (ctrl) samples and the increased abundance of intron reads in the treated (tr) samples. RNA-seq tracks were generated with SparK 66.

Based on our findings that RNA splicing genes are altered during G418 treatment (**Figure 1C, D**), we sought to determine whether AS events take place during *A. castellanii* PCD. Across all treatment times we detected 18,748 differential AS events (including IR, SE, A5SS, A3SS and MXE) in 5,006 genes, which represents around 35% of all annotated *Acanthamoeba* genes (a single gene may retain more than one intron or present two or more AS events). IR accounted for approximately 80% of all AS events, thus representing the predominant type of AS during *Acanthamoeba* PCD (**Figure 2A**). We found that the number of AS events increased with treatment time (**Figure 2B**). Tables containing significantly altered IR and other AS events for each treatment time can be found in the supplemental materials. One of the top genes displaying increased IR during *Acanthamoeba* PCD was ACA1_229570, which is annotated as an oxidoreductase. We observed that two introns of this gene were retained in ~40% of all transcripts following 1 h of treatment. Notably, the proportion of transcripts retaining these introns increased to ~80-90% after 3 h and 6 h of treatment (**Figure 2C**, left). This same behavior was observed for two introns in the ACA1_263910 gene (autophagy-related protein 3), which did not show retention even after 1 h of treatment but were retained in ~25% of transcripts following 3 h and 6 h of treatment (**Figure 2C**, right).

The *A. castellanii *annotation (ENSEMBL v37) reports only one transcript variant per gene, which represents the canonical transcript variant. In the context of SE, the canonical transcript variant is taken as reference. The red dots in **Figure 2B** indicate included exons, while the blue dots represent skipped exons whose exclusion has not been previously reported. Most differentially skipped exons showed decreased inclusion (blue dots), indicating that novel, unannotated transcript variants lacking these exons are expressed during *Acanthamoeba* PCD. For A5SS and A3SS splice sites, and MXE there was no overall bias towards increased or decreased inclusion levels, instead, the changes were bidirectional (**Figure 2B**).

**Figure 3:**
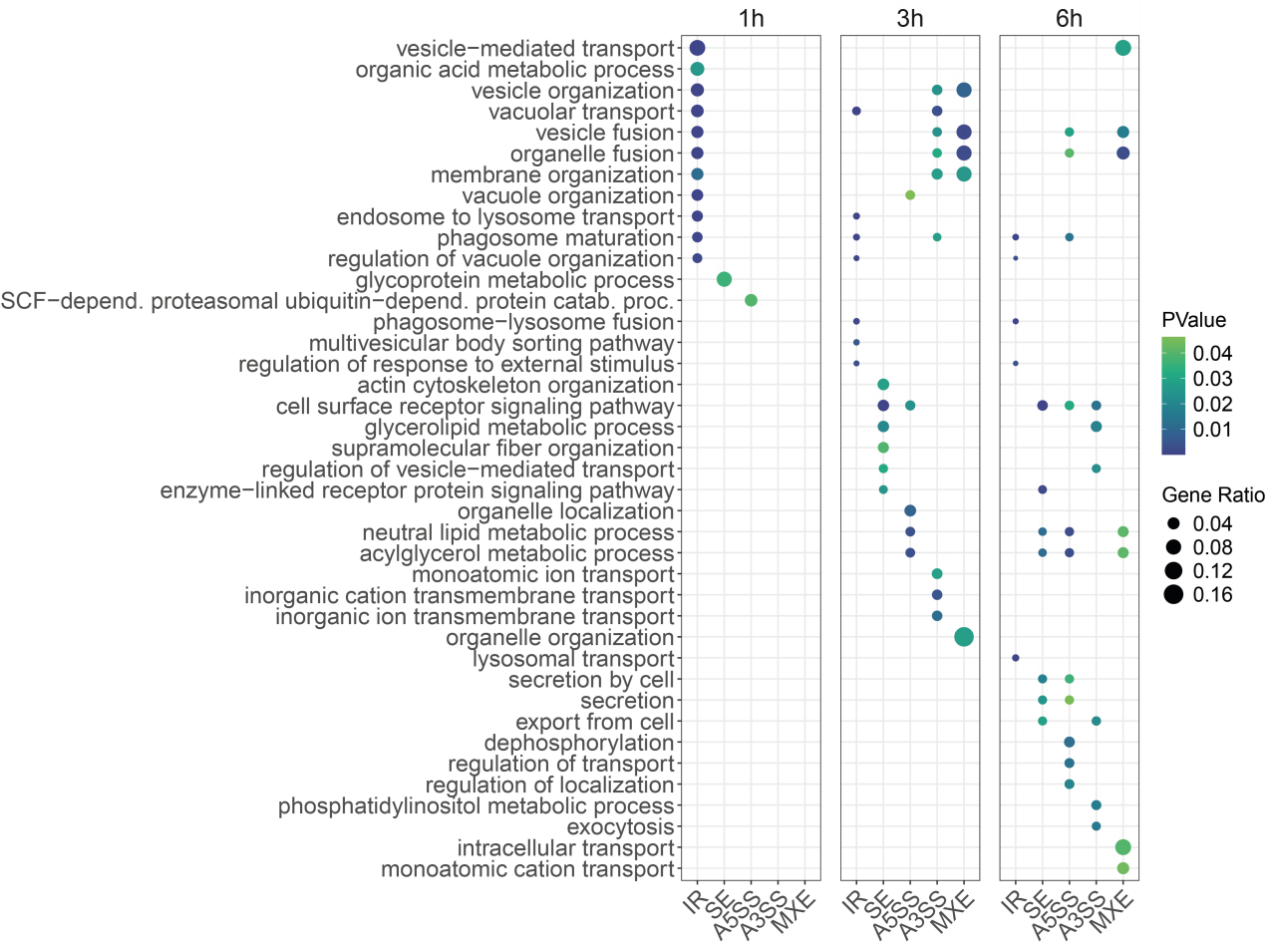
FIGURE 3: Gene ontology for AS events during programmed cell death of *Acanthamoeba*. The figure shows the significant GO terms that were enriched among genes displaying AS events. The size of the circle represents the *Gene Ratio*, which refers to the proportion of genes displaying AS associated with a given GO term. Therefore, a larger circle represents a larger representation of genes. The color scale refers to the PValue as stated by the legend.

After observing the large number of genes displaying AS, we performed a GO analysis to identify biological processes potentially affected by AS (**Figure 3**). We observed that IR is nearly the only type of AS event for which significant enrichment of GO terms is found at 1 h. Several cellular processes are affected by post-transcriptional regulation (AS) which include processes related to transport, organelle and membrane structure and organization. Of note, phagosome-related GO terms were only enriched among differential IR events during *Acanthamoeba* PCD. We then explored the occurrence of AS in genes involved in pathways known to influence PCD, including autophagy, the proteasome, the ubiquitin system, and oxidative stress (for which oxidative phosphorylation genes were used as a proxy). Increased IR was found among genes in all these pathways (**Figure 2C** and S3). On the other hand, decreased inclusion of alternative exons (SE) was observed in autophagy, oxidative phosphorylation and ubiquitin system genes. In the case of A5SS and A3SS, decreased inclusion of alternative splice sites was also mainly observed. MXE events were rare in these pathways, with only one event detected in ACA1_141980, a gene associated with the ubiquitin system. Altogether, these findings indicate that AS occurs in both PCD-related pathways and others not traditionally associated with cell death, suggesting that AS may play a broader role in shaping the transcriptomic landscape of PCD.

**Figure 4:**
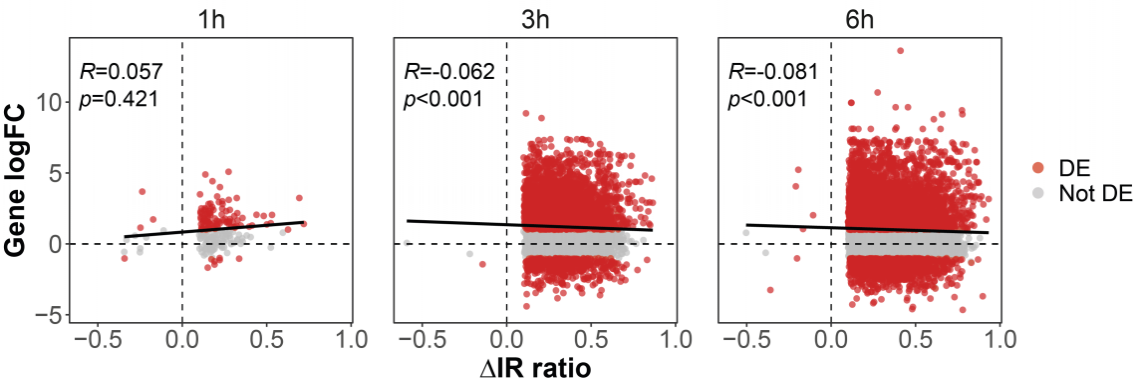
FIGURE 4: Relationship between intron retention (IR) and transcript levels during programmed cell death in *A. castellanii*. Each dot represents a differentially retained intron (a single gene could be retaining two introns and therefore be represented by two dots). Red dots represent genes that are differentially expressed (DE). The line across each graph represents the spearman correlation, while the *R* represents the correlation value and *p* the PValue. On the Y-axis, logFC of transcript levels is shown, while the X-axis represents changes in IR levels.

Since increased IR has been associated with decreased transcript levels and vice versa 41,42, we compared changes in IR against changes in transcript levels. Results (**Figure 4**) show that a large fraction of genes with IR are also differentially expressed after 1, 3 and 6 hours of G418 treatment. We further conducted a Spearman correlation analysis between changes in IR and transcript levels. There is a statistically significant negative correlation between transcript levels and IR after 3 and 6 hours. However, after 1 hour of treatment, the statistical analysis did not reveal any significant correlation. Additionally, we found that there are many genes that are not differentially expressed but display altered IR levels (**Figure 4**, gray dots; proportion of genes with altered IR levels but stable transcript levels at 1 h = 47%, 3 h = 45%, 6 h = 46%). This group of genes could play a role during *Acanthamoeba* PCD at the post-transcriptional level.

### Retained introns in *Acanthamoeba* display specific characteristics

**Figure 5:**
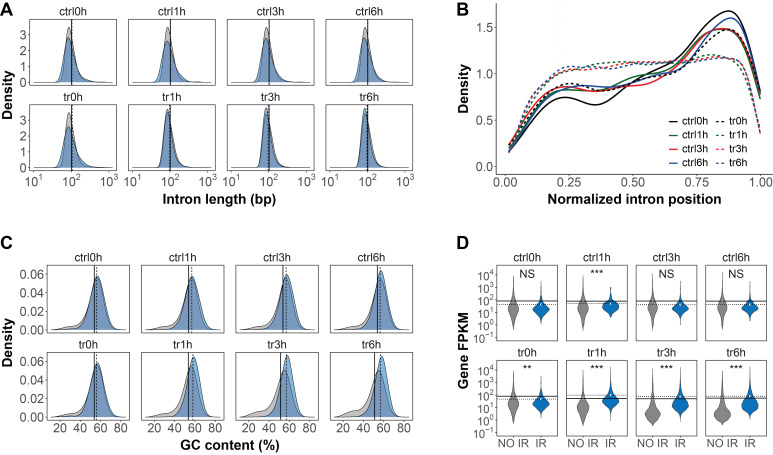
FIGURE 5: * Acanthamoeba* retained introns possess specific characteristics. **(A)** Distribution of the lengths of retained (blue) and constitutively spliced (gray) introns. **(B)** Normalized position of retained introns within genes. On the X-axis, 0 and 1 represent the 5′ and 3′ end of genes, respectively. Note the 3′ bias of retained introns in control (ctrl) and 0h treated (tr0h) samples. **(C)** Distribution of the GC content in retained (blue) and constitutively spliced (gray) introns. **(D)** Transcript levels of genes with non-retained (NO IR) and retained (IR) introns. For **A**, **C**, and **D**, the dotted and solid lines represent the mean values for IR and NO IR, respectively. Significance was tested using the Wilcoxon Rank Sum Test. *p < 0.05, **p < 0.005, ***p < 0.0005. NS, Not Significant.

Since IR accounted as the predominant type of AS during *Acanthamoeba* PCD, we sought to determine whether retained introns possess specific characteristics. We did not observe an obvious difference between the length of retained and constitutively spliced introns (**Figure 5A**). However, retained introns were mainly found towards the 3′ of genes in all control and 0 h treated samples (**Figure 5B**, solid lines and tr0h dotted line). Interestingly, this 3′ bias was not observed in 1 h, 3 h, and 6 h treated samples, in which retained introns were detected almost uniformly throughout the gene body (**Figure 5B**, dotted lines). In untreated *Acanthamoeba* trophozoites, retained introns were found to have slightly higher GC content, compared to constitutively spliced introns (**Figure 5C**, upper panel). This difference became even more noticeable during PCD, in which introns with higher GC content are preferentially retained (**Figure 5C**, bottom panel).

Genes expressing IR transcripts displayed, on average, lower transcript levels than genes with non-retained introns in untreated *Acanthamoeba* trophozoites and 0 h treated samples (**Figure 5D**, upper panel and tr0h). However, during PCD there was a shift towards increased expression levels of IR genes (**Figure 5D**, bottom panel).

## DISCUSSION

PCD in *A. castellanii* involves regulated AS events that follow specific patterns, indicating a coordinated biological process. Our results show various mechanisms and timing of gene regulation occurring during PCD, both at transcriptional and post-transcriptional levels. These regulations lead to alterations in key cellular processes, including changes in mRNA transport, transcription regulation, splicing, ribosome biogenesis, and organelle organization. Importantly, AS introduces greater protein isoform diversity 43, which could facilitate adaptation and phenotypic diversity, even if many AS events lead to non-functional variants 29. We cannot categorically discount that sequencing techniques, AS software and even annotation might be causing this difference.

*Acanthamoeba* exhibits a higher percentage of AS genes (35%) during PCD than what has been previously reported for other protists like *Dictyostelium discoideum* (2.2%), *Phaeodactylum tricornutum* (17.5%), *Phytophthora infestans* (14.9%), *Plasmodium falciparum* (2.9%), *Pythium ultimum* (7.9%) and *Toxoplasma gondii* (24.3%) 44. This increased complexity in splicing of *Acanthamoeba* may be attributed to the rich diversity of protists and the evolutionary relationship between Amoebozoa, to which it belongs, and Opisthokonts which include animals and fungi. However, occurrence of AS events is not necessarily high for all Amoebozoans as illustrated by *D. discoideum*. Another consideration related to the high prevalence of AS events in *Acanthamoeba* is the high GC content. According to NCBI, *Acanthamoeba* reference genome has a GC content of 59% (Submitted GenBank Assembly: GCA_000826485.1), while* D. discoideum *has 22.5% (GCF_000004695.1). Other protists with similarly low GC content include *Entamoeba histolytica* (30.5%; GCA_917563895.1), *Naegleria fowleri* (37%; GCF_008403515.1) and *Balamuthia mandrillaris* (47%; GCA_031835245.2). The higher GC content might explain the abundance of alternative splicing, as splicing sites tend to be GC enriched 45.

Our observations indicate that both IR and SE events increase during PCD in *Acanthamoeba*. This suggests a controlled alteration of post-transcriptional mechanisms that generate a more diverse transcriptome. Interestingly, IR and SE have opposite effects on RNA processing: while IR leads to less "cutting" of transcripts, SE involves more "cutting." Ultimately, PCD results in a higher number of introns and a lower number of included exons in the transcriptome. This coordinated behavior of AS events seems to indicate a regulated mechanism at work rather than a random breakdown of cellular functions.

Previously, IR was considered a process with no physiological effect, often leading only to reduced gene expression via nonsense-mediated decay (NMD), it has been now implicated in various biological processes, including cell differentiation in multicellular organisms 46. Many genomic regions associated with NMD are highly conserved among vertebrates and are crucial for maintaining splicing homeostasis. Investigating these aspects in protists like *Acanthamoeba* may yield valuable evolutionary insights, while considering the differences between organisms such as the lack of caspases in protists that are relevant to PCD in vertebrates 36,43,47.

Moreover, the interplay between alternative splicing and nonsense-mediated decay (AS-NMD) may regulate gene expression following transcription, impacting PCD in *Acanthamoeba* 47. Thus, understanding these mechanisms is crucial for elucidating the underlying processes of cell death. It is essential to consider that AS events can lead to apparent differential gene expression without necessarily affecting the phenotype. An increase in transcripts might be offset by non-functional RNAs produced by AS, potentially masking critical physiological differences. A significant number of hypothetical proteins exist within the annotated *Acanthamoeba* genome, as indicated by this study and prior analyses (AmoebaDB). These discrepancies might help explain the differences in transcriptomic analysis that can be found for example to other studies such as the ones published by Bernard *et al*. 48, de Obeso Fernandez del Valle 49, Scheckhuber 50 and Ahmed *et al*. 51, As more RNA-seq studies emerge, improvement in genome annotation and characterization of genes is anticipated, increasing our understanding of various hidden processes.

*Acanthamoeba* retained introns show characteristics previously observed in other organisms. For instance, retained introns in *Acanthamoeba* possess higher GC content and display 3′ bias, similar to what has been observed in vertebrates 52. It is important to note that *Acanthamoeba* already has a high GC content 53. However, the length of retained introns in *Acanthamoeba* does not follow the trend observed in other kingdoms, e.g., plants and animals, in which retained introns have been found to be shorter, compared to non-retained introns 52,54.

Multiple cellular processes influenced by AS events in *Acanthamoeba*, including vesicle transport, membrane organization, and cytoskeletal dynamics, support the notion that these splicing events are integral to the cell death process. The criteria for defining cell death involve loss of membrane integrity, cytoplasmic collapse, and nuclear degradation 2,55. The GO analysis of AS events aligns with these processes, contrasting with differential gene expression observations.

*Acanthamoeba* infections are difficult to treat, and current approaches involve agents that are toxic or fail to eradicate cysts, which leads to relapse. The understanding and manipulation of PCD phenomena could lead to new, more targeted and efficient therapeutic strategies 19. PCD has been targeted for therapeutic purposes to combat malaria with mefloquine to induce PCD in *Plasmodium falciparum* 56. Other fields that have used PCD as therapeutic target include cancer 57 and neurodegenerative disease 58. A deeper understanding of AS events during PCD might offer critical insights that could guide the development of effective treatments to combat *Acanthamoeba* infections.

In conclusion, this study underscores the significance of considering AS events in understanding PCD in *Acanthamoeba*. The coordinated changes in splicing patterns imply a complex regulatory mechanism that plays a crucial role of these post-transcriptional changes during cell death. Relying solely on differential gene expression analyses may not provide a complete picture of the underlying molecular mechanisms of PCD. Future research should aim to characterize the functional implications of these splicing events and explore their potential as therapeutic targets against *Acanthamoeba*-related infections. Additionally, incorporating proteomic studies will enhance our understanding of this process and its interconnections with other biological events, such as encystation, looking at uncoupling of the transcriptome, and looking at multi-omics approaches 48.

## MATERIALS AND METHODS

### *Acanthamoeba* cultures

*Acanthamoeba castellanii* strain GS-336, closely related to the Neff strain (ATCC 30010), served as the experimental model 19,59. The relationship to the Neff strain was established previously by phylogeny analysis of the 18S gene 19. The *Acanthamoeba* cultures were grown in axenic medium composed of Bacto tryptone (14.3 g L^−1^), yeast extract (7.15 g L^−1^), glucose (15.4 g L^−1^), Na_2_HPO_4_ (0.51 g L^−1^), and KH_2_PO_4_ (0.486 g L^−1^) adjusted to pH 6.5.

### PCD Induction

To induce PCD, trophozoites were cultured in a 6-well plate. Upon reaching confluency, the cells were exposed to Neff's saline buffer supplemented with 75 μg/mL of G418 and incubated at 37°C (to maintain relevance of the pathological context) for a period ranging from 0 to 6 hours. Post-incubation, the amoebae were extracted from the wells and centrifuged at 500 g for 5 minutes. Pellet of amoebae was washed with Neff's saline buffer twice and was subsequently utilized for RNA extraction. Two distinct groups were established for comparison: one undergoing the specified treatment and the other serving as an untreated time-matched control 60. Samples were incubated for 0, 1, 3 and 6 hours (referred to as 0 h, 1 h, 3 h and 6 h, respectively). Experiments were performed in triplicates.

### RNA sequencing and data processing

RNA extraction was carried out utilizing the RNeasy Mini Kit (QIAGEN) in accordance with the manufacturer's protocols. Subsequently, the quality and purity of RNA were evaluated employing the QUBIT RNA BR Assay Kit (Thermo Fisher Scientific). For the generation of cDNA libraries, an automated TruSeq mRNAseq (next-generation shotgun sequencing) workflow was implemented using total RNA as the starting material. The sequencing process was conducted utilizing a HiSeq-4000 instrument (2 x 75 nt) through Edinburgh Genomics.

Eight groups in total were sequenced in triplicate. The groups consisted of 0, 1, 3 and 6 hours for control and G418 treatment. All comparisons for differential gene expression or AS events were done between treatment and control of the same timepoint.

*A. castellanii* Neff strain reference genome (assembly: GCA000313135v1) was obtained from ENSEMBL Protists. Raw RNA-seq reads quality was assessed using FastQC. STAR was used to index the genome and align the reads 61.

Gene counts were obtained using featureCounts together with the *A. castellanii* ENSEMBL gene annotation v37. Multidimensional scaling analysis was conducted using edgeR 62. We identified one of the 0h treated samples as an outlier (Figure S1C), which was discarded for all the analysis reported in this study.

### Differential expression and intron retention analysis

Differential gene expression analysis was conducted using edgeR 62. Differentially expressed (DE) genes were considered as those showing |logFC| > 1 and False Discovery Rate (FDR) < 0.05.

IRFinder 63 was used to identify retained introns. The IRFinder reference was generated using the *A. castellanii* ENSEMBL gene annotation v37. IRFinder uses the IR ratio metric to estimate the proportion of transcripts retaining introns. IR was assessed in 82,571 introns of 12,990 genes. Introns were considered as retained if they showed *Coverage* >= 0.8, *IntronDepth* >= 5, *SpliceExact* >= 5, and *IRratio* > 0.1. This IR ratio threshold was used to keep introns that were estimated to be retained in at least 10% of the transcripts. Only retained introns from genes showing Fragments Per Kilobase of transcript per Million reads mapped (FPKM) > 1 were kept for further analysis.

Differential IR analysis was conducted using the Generalized Linear Model approach with DESeq2 64. Differentially retained introns were considered as those showing |ΔIR ratio| > 0.1 and adjusted PValue < 0.05.

For comparing intron features, e.g., intron length and GC content, constitutively spliced introns were considered as those showing *IntronDepth* < 5 and *IRratio* < 0.01.

### Alternative splicing analysis

rMATS 65 was used to identify other types of AS, e.g., exon skipping (SE), alternative 5′ (A5SS) and 3′ (A3SS) splice sites, and mutually exclusive exons (MXE). Since the *A. castellanii* ENSEMBL v37 reports only one transcript variant per gene, rMATS was run using the *--novelSS* parameter. The maser tool was used to filter the rMATS output. AS events with at least 10 average reads were kept. Differential AS events were identified as those with |ΔPSI| > 0.1 and FDR < 0.05. Only differential AS events identified in genes with FPKM > 1 were kept for further analysis. RNA-seq tracks were generated with SparK 66.

### Gene ontology

Biological processes enriched among genes with altered transcript levels, and genes showing differential IR and AS events were identified using DAVID (https://david.ncifcrf.gov/) 67. Significant gene ontology (GO) terms were identified as those showing PValue < 0.05.

## CONFLICT OF INTEREST

The authors declare no competing interest.

## SUPPLEMENTAL MATERIAL

Click here for supplemental data file.

All supplemental data for this article are available online at www.microbialcell.com/researcharticles/2025a-gomez-montalvo-microbial-cell/.

## References

[1] Tang D, Kang R, Berghe T Vanden, Vandenabeele P, Kroemer G (2019). The molecular machinery of regulated cell death.. Cell Res.

[2] Kroemer G, Galluzzi L, Vandenabeele P, Abrams J, Alnemri ES, Baehrecke EH, Blagosklonny M V, El-Deiry WS, Golstein P, Green DR, Hengartner M, Knight RA, Kumar S, Lipton SA, Malorni W, Nuñez G, Peter ME, Tschopp J, Yuan J, Piacentini M, Zhivotovsky B, Melino G (2009). Classification of cell death: recommenda-tions of the Nomenclature Committee on Cell Death 2009.. Cell Death Differ.

[3] Wójcik C (2002). Regulation of apoptosis by the ubiquitin and proteasome pathway.. J Cell Mol Med.

[4] Ludovico P, Sousa MJ, Silva MT, Leão C, Côrte-Real M (2001). Saccharomy-ces cerevisiae commits to a programmed cell death process in response to acetic acid.. Microbiology.

[5] Madeo F, Fröhlich E, Ligr M, Grey M, Sigrist SJ, Wolf DH, Fröhlich K-U (1999). Oxygen Stress: A Regulator of Apoptosis in Yeast.. J Cell Biol.

[6] Cornillon S, Foa C, Davoust J, Buonavista N, Gross JD, Golstein P (1994). Programmed cell death in Dictyostelium.. J Cell Sci.

[7] Al-Olayan EM, Williams GT, Hurd H (2002). Apoptosis in the malaria proto-zoan, Plasmodium berghei: a possible mechanism for limiting intensity of infec-tion in the mosquito.. Int J Parasitol.

[8] Welburn SC, Dale C, Ellis D, Beecroft R, Pearson TW (1996). Apoptosis in procyclic Trypanosoma brucei rhodesiense in vitro.. Cell Death Differ.

[9] Jiménez-Ruiz A, Alzate JF, MacLeod ET, Lüder CGK, Fasel N, Hurd H (2010). Apoptotic markers in protozoan parasites.. Parasit Vectors.

[10] Nguewa PA, Fuertes MA, Valladares B, Alonso C, Pérez JM (2004). Programmed cell death in trypanosomatids: a way to maximize their biological fitness?. Trends Parasitol.

[11] Duszenko M, Figarella K, Macleod ET, Welburn SC (2006). Death of a trypanosome: a selfish altruism.. Trends Parasitol.

[12] Lee N, Bertholet S, Debrabant A, Muller J, Duncan R, Nakhasi HL (2002). Programmed cell death in the unicellular protozoan parasite Leishmania.. Cell Death Differ.

[13] Villalba JD, Gómez C, Medel O, Sánchez V, Carrero JC, Shibayama M, Ishiwara DGP (2007). Programmed cell death in Entamoeba histolytica induced by the aminoglycoside G418.. Microbiology.

[14] Kelly C Rice, Kenneth W B (2008). Molecular Control of Bacterial Death and Lysis.. Microbiol Mol Biol Rev.

[15] Chaloupka J, Vinter V (1996). Programmed cell death in bacteria.. Folia Microbiol.

[16] Zheng W, Rasmussen U, Zheng S, Bao X, Chen B, Gao Y, Guan X, Larsson J, Bergman B (2013). Multiple Modes of Cell Death Discovered in a Prokaryotic (Cyanobacterial) Endosymbiont.. PLoS One.

[17] Feng Y, Hsiao Y-H, Chen H-L, Chu C, Tang P, Chiu C-H (2009). Apoptosis-like cell death induced by Salmonella in Acanthamoeba rhysodes.. Genomics.

[18] Martín-Navarro CM, López-Arencibia A, Sifaoui I, Reyes-Batlle M, Valladares B, Martínez-Carretero E, Piñero JE, Maciver SK, Lorenzo-Morales J (2015). Statins and Voriconazole Induce Programmed Cell Death in Acanthamoeba castellanii.. Antimicrob Agents Chemother.

[19] Koutsogiannis Z, MacLeod ET, Maciver SK (2019). G418 induces pro-grammed cell death in Acanthamoeba through the elevation of intracellular calcium and cytochrome c translocation.. Parasitol Res.

[20] Baig AM, Lalani S, Khan NA (2017). Apoptosis in Acanthamoeba castel-lanii belonging to the T4 genotype.. J Basic Microbiol.

[21] Martín-Navarro CM, López-Arencibia A, Sifaoui I, Reyes-Batlle M, Fouque E, Osuna A, Valladares B, Piñero JE, Héchard Y, Maciver SK, Lorenzo-Morales J (2017). Amoebicidal Activity of Caffeine and Maslinic Acid by the Induction of Programmed Cell Death in Acanthamoeba.. Antimicrob Agents Chemother.

[22] Lorenzo-Morales J, Martín-Navarro CM, López-Arencibia A, Arnalich-Montiel F, Piñero JE, Valladares B (2013). Acanthamoeba keratitis: an emerging disease gathering importance worldwide?. Trends Parasitol.

[23] Cartuche L, Sifaoui I, Cruz D, Reyes-Batlle M, López-Arencibia A, Javier Fernández J, Díaz-Marrero AR, Piñero JE, Lorenzo-Morales J (2019). Stauro-sporine from Streptomyces sanyensis activates Programmed Cell Death in Acan-thamoeba via the mitochondrial pathway and presents low in vitro cytotoxicity levels in a macrophage cell line.. Sci Rep.

[24] Sifaoui I, López-Arencibia A, Martín-Navarro CM, Reyes-Batlle M, Wagner C, Chiboub O, Mejri M, Valladares B, Abderrabba M, Piñero JE, Lorenzo-Morales J (2017). Programmed cell death in Acanthamoeba castellanii Neff induced by several molecules present in olive leaf extracts.. PLoS One.

[25] Wu D, Qiao K, Feng M, Fu Y, Cai J, Deng Y, Tachibana H, Cheng X (2018). Apoptosis of Acanthamoeba castellanii Trophozoites Induced by Oleic Acid.. J Eukaryot Microbiol.

[26] Sifaoui I, Díaz-Rodríguez P, Rodríguez-Expósito RL, Reyes-Batlle M, López-Arencibia A, Salazar Villatoro L, Castelan-Ramírez I, Omaña-Molina M, Oliva A, Piñero JE, Lorenzo-Morales J (2022). Pitavastatin loaded nanoparticles: A suitable ophthalmic treatment for Acanthamoeba Keratitis inducing cell death and autophagy in Acanthamoeba polyphaga.. Eur J Pharm Biopharm.

[27] Eustice DC, Wilhelm JM (1984). Mechanisms of action of aminoglycoside antibiotics in eucaryotic protein synthesis.. Antimicrob Agents Chemother.

[28] Lin J-C, Tsao M-F, Lin Y-J (2016). Differential impacts of alternative splic-ing networks on apoptosis.. Int J Mol Sci.

[29] Wright CJ, Smith CWJ, Jiggins CD (2022). Alternative splicing as a source of phenotypic diversity.. Nat Rev Genet.

[30] Marasco LE, Kornblihtt AR (2023). The physiology of alternative splicing.. Nat Rev Mol Cell Biol.

[31] Grau-Bové X, Ruiz-Trillo I, Irimia M (2018). Origin of exon skipping-rich transcriptomes in animals driven by evolution of gene architecture.. Genome Biol.

[32] de Obeso Fernández del Valle A, Gómez-Montalvo J, Maciver SK (2022). Acanthamoeba castellanii exhibits intron retention during encystment.. Parasitol Res.

[33] Jung H, Lee D, Lee J, Park D, Kim YJ, Park W-Y, Hong D, Park PJ, Lee E (2015). Intron retention is a widespread mechanism of tumor-suppressor inacti-vation.. Nat Genet.

[34] Pimentel H, Parra M, Gee SL, Mohandas N, Pachter L, Conboy JG (2016). A dynamic intron retention program enriched in RNA processing genes regulates gene expression during terminal erythropoiesis.. Nucleic Acids Res.

[35] Aurrecoechea C, Barreto A, Brestelli J, Brunk BP, Caler E V, Fischer S, Gajria B, Gao X, Gingle A, Grant G, Harb OS, Heiges M, Iodice J, Kissinger JC, Kraemer ET, Li W, Nayak V, Pennington C, Pinney DF, Pitts B, Roos DS, Srinivasamoorthy G, Stoeckert CJ, Treatman C, Wang H (2011). AmoebaDB and MicrosporidiaDB: functional genomic resources for Amoebozoa and Microsporidia species.. Nucleic Acids Res.

[36] Clarke M (2013). Genome of Acanthamoeba castellanii highlights exten-sive lateral gene transfer and early evolution of tyrosine kinase signaling.. Genome Biol.

[37] Matthey-Doret C, Colp MJ, Escoll P, Thierry A, Moreau P, Curtis B, Sahr T, Sarrasin M, Gray MW, Lang BF, Archibald JM, Buchrieser C, Koszul R (2022). Chromosome-scale assemblies of Acanthamoeba castellanii genomes provide insights into Legionella pneumophila infection-related chromatin reorganization.. Genome Res.

[38] Amos B (2022). VEuPathDB: the eukaryotic pathogen, vector and host bioinformatics resource center.. Nucleic Acids Res.

[39] Roy SW (2006). Intron-rich ancestors.. Trends Genet.

[40] Saheb E, Trzyna W, Bush J (2014). Caspase-like proteins: Acanthamoeba castellanii metacaspase and Dictyostelium discoideum paracaspase, what are their functions?. J Biosci.

[41] Yao J, Ding D, Li X, Shen T, Fu H, Zhong H, Wei G, Ni T (2020). Prevalent intron retention fine-tunes gene expression and contributes to cellular senes-cence.. Aging Cell.

[42] Ni T, Yang W, Han M, Zhang Y, Shen T, Nie H, Zhou Z, Dai Y, Yang Y, Liu P, Cui K, Zeng Z, Tian Y, Zhou B, Wei G, Zhao K, Peng W, Zhu J (2016). Global intron retention mediated gene regulation during CD4+ T cell activation.. Nucleic Acids Res.

[43] McGlincy NJ, Smith CWJ (2008). Alternative splicing resulting in non-sense-mediated mRNA decay: what is the meaning of nonsense?. Trends Biochem Sci.

[44] Chen L, Bush SJ, Tovar-Corona JM, Castillo-Morales A, Urrutia AO (2014). Correcting for Differential Transcript Coverage Reveals a Strong Relationship between Alternative Splicing and Organism Complexity.. Mol Biol Evol.

[45] Zhang J, Kuo CCJ, Chen L (2011). GC content around splice sites affects splicing through pre-mRNA secondary structures.. BMC Genomics.

[46] Monteuuis G, Wong JJL, Bailey CG, Schmitz U, Rasko JEJ (2019). The changing paradigm of intron retention: regulation, ramifications and recipes.. Nucleic Acids Res.

[47] Ni JZ, Grate L, Donohue JP, Preston C, Nobida N, O’Brien G, Shiue L, Clark TA, Blume JE, Ares M (2007). Ultraconserved elements are associated with homeostatic control of splicing regulators by alternative splicing and nonsense-mediated decay.. Genes Dev.

[48] Bernard C, Locard-Paulet M, Noël C, Duchateau M, Giai Gianetto Q, Moumen B, Rattei T, Hechard Y, Jensen LJ, Matondo M, Samba-Louaka A (2022). A time-resolved multi-omics atlas of Acanthamoeba castellanii encystment.. Nat Commun.

[49] de Obeso Fernández del Valle A, Scheckhuber CQ, Chavaro-Pérez DA, Ortega-Barragán E, Maciver SK (2023). mRNA Sequencing Reveals Upregulation of Glutathione S-Transferase Genes during Acanthamoeba Encystation.. Microorganisms.

[50] Scheckhuber CQ, Damián Ferrara R, Gómez-Montalvo J, Maciver SK, de Obeso Fernández del Valle A (2024). Oxidase enzyme genes are differentially expressed during Acanthamoeba castellanii encystment.. Parasitol Res.

[51] Ahmed U, Ong S-K, Tan KO, Khan KM, Khan NA, Siddiqui R, Alawfi BS, Anwar A (2024). Alpha-Mangostin and its nano-conjugates induced programmed cell death in Acanthamoeba castellanii belonging to the T4 genotype.. Int Microbiol.

[52] Schmitz U, Pinello N, Jia F, Alasmari S, Ritchie W, Keightley M-C, Shini S, Lieschke GJ, Wong JJ-L, Rasko JEJ (2017). Intron retention enhances gene regulatory complexity in vertebrates.. Genome Biol.

[53] Adam KMG, Blewett DA, Flamm WG (1969). The DNA of Acanthamoeba spp. a method for extraction and its characterization.. J Protozool.

[54] Zhang G, Guo G, Hu X, Zhang Y, Li Q, Li R, Zhuang R, Lu Z, He Z, Fang X, Chen L, Tian W, Tao Y, Kristiansen K, Zhang X, Li S, Yang H, Wang J, Wang J (2010). Deep RNA sequencing at single base-pair resolution reveals high complexity of the rice transcriptome.. Genome Res.

[55] Galluzzi L (2018). Molecular mechanisms of cell death: recommenda-tions of the Nomenclature Committee on Cell Death 2018.. Cell Death Differ.

[56] Gunjan S, Singh SK, Sharma T, Dwivedi H, Chauhan BS, Imran Siddiqi M, Tripathi R (2016). Mefloquine induces ROS mediated programmed cell death in malaria parasite: Plasmodium.. Apoptosis.

[57] Luobin L, Wanxin H, Yingxin G, Qinzhou Z, Zefeng L, Danyang W, Huaqin L (2024). Nanomedicine-induced programmed cell death in cancer therapy: mech-anisms and perspectives.. Cell Death Discov.

[58] Vila M, Przedborski S (2003). Targeting programmed cell death in neuro-degenerative diseases.. Nat Rev Neurosci.

[59] de Obeso Fernández del Valle A (2018). Protein secretion and encystation in Acanthamoeba.. PhD Thesis, The University of Edinburgh.

[60] Koutsogiannis Z (2019). Acanthamoeba programmed cell death.. PhD, University of Edinburgh.

[61] Dobin A, Davis CA, Schlesinger F, Drenkow J, Zaleski C, Jha S, Batut P, Chais-son M, Gingeras TR (2013). STAR: Ultrafast universal RNA-seq aligner.. Bioin-formatics.

[62] Robinson MD, Oshlack A (2010). A scaling normalization method for differential expression analysis of RNA-seq data.. Genome Biol.

[63] Middleton R, Gao D, Thomas A, Singh B, Au A, Wong JJ-L, Bomane A, Cosson B, Eyras E, Rasko JEJ, Ritchie W (2017). IRFinder: assessing the impact of intron retention on mammalian gene expression.. Genome Biol.

[64] Love MI, Huber W, Anders S (2014). Moderated estimation of fold change and dispersion for RNA-seq data with DESeq2.. Genome Biol.

[65] Shen S, Park JW, Lu Z, Lin L, Henry MD, Wu YN, Zhou Q, Xing Y (2014). rMATS: Robust and flexible detection of differential alternative splicing from replicate RNA-Seq data.. Proc Natl Acad Sci U S A.

[66] Kurtenbach S, William Harbour J (2019). SparK: A publication-quality NGS visualization tool.. bioRxiv.

[67] Huang DW, Sherman BT, Lempicki RA (2009). Systematic and integrative analysis of large gene lists using DAVID bioinformatics resources.. Nat Protoc.

