# Supplementary material for: Alternative splicing drives a dynamic transcriptomic response during Acanthamoeba castellanii programmed cell death
Source: Microb Cell. 2025 Aug 26;12:231–41. doi: 10.15698/mic2025.08.858 (PMC12404691; doi:10.15698/mic2025.08.858)
Supplement: Supplementary file 1 [file mic-12-231-s01.pdf]

Supplemental Figures

A

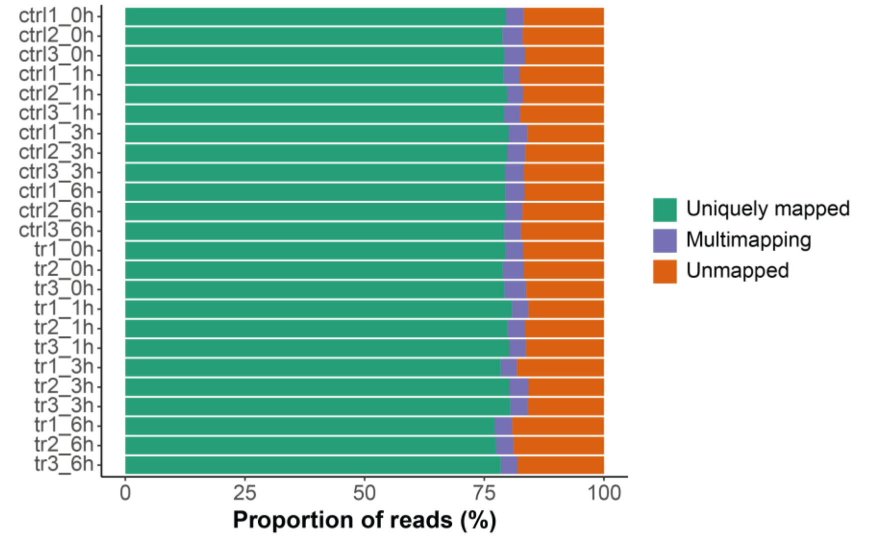
ctrl1_0h
ctrl2_0h
ctrl3_0h
ctrl1_1h
ctrl2_1h
ctrl3_1h
ctrl1_3h
ctrl2_3h
ctrl3_3h
ctrl1_6h
ctrl2_6h
ctrl3_6h
tr1_0h
tr2_0h
tr3_0h
tr1_1h
tr2_1h
tr3_1h
tr1_3h
tr2_3h
tr3_3h
tr1_6h
tr2_6h
tr3_6h
0
25
50
75
100
Proportion of reads (%)
Uniquely mapped
Multimapping
Unmapped

B

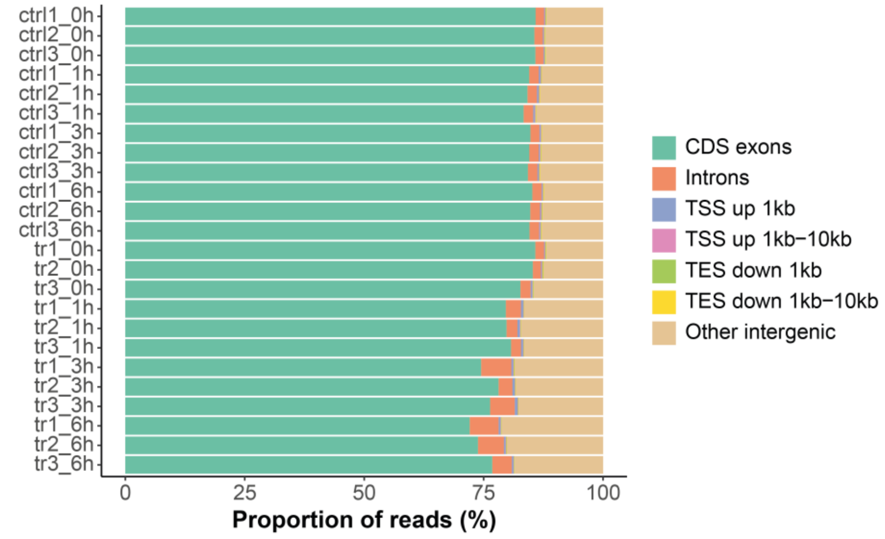
ctrl1_0h
ctrl2_0h
ctrl3_0h
ctrl1_1h
ctrl2_1h
ctrl3_1h
ctrl1_3h
ctrl2_3h
ctrl3_3h
ctrl1_6h
ctrl2_6h
ctrl3_6h
tr1_0h
tr2_0h
tr3_0h
tr1_1h
tr2_1h
tr3_1h
tr1_3h
tr2_3h
tr3_3h
tr1_6h
tr2_6h
tr3_6h
0
25
50
75
100
Proportion of reads (%)
CDS exons
Introns
TSS up 1kb
TSS up 1kb–10kb
TES down 1kb
TES down 1kb–10kb
Other intergenic

C

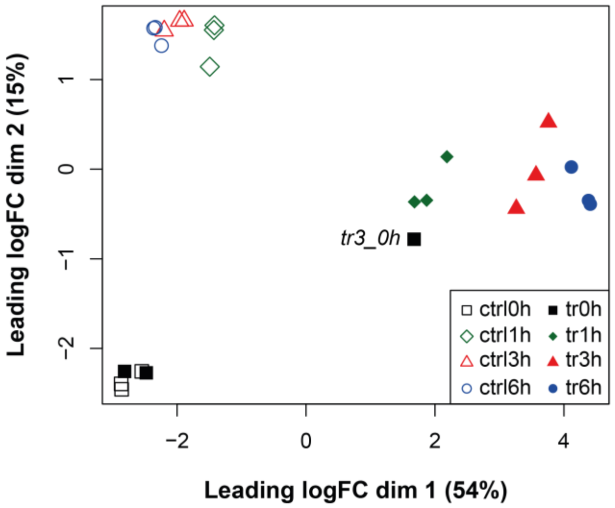
Leading logFC dim 2 (15%)
1
0
−1
−2
tr3_0h
ctrl0h
tr0h
ctrl1h
tr1h
ctrl3h
tr3h
ctrl6h
tr6h
−2
0
2
4
Leading logFC dim 1 (54%)

Figure S1. Quality control of RNA-seq data. (A) Proportion of uniquely mapped, multimapping and unmapped reads after mapping against the *A. castellanii* Neff strain genome (assembly: GCA000313135v1). Note that most reads (~80%) were uniquely mapped reads. **(B)** Proportion of reads assigned to distinct types of genomic features. Note that most reads (75-80%) were assigned to exons. TSS, transcription start site. TES, transcription end site. **(C)** Preliminary MDS plot highlighting an outlier tr0h sample (tr3_0h), which was discarded for differential gene expression, IR and AS analyses.

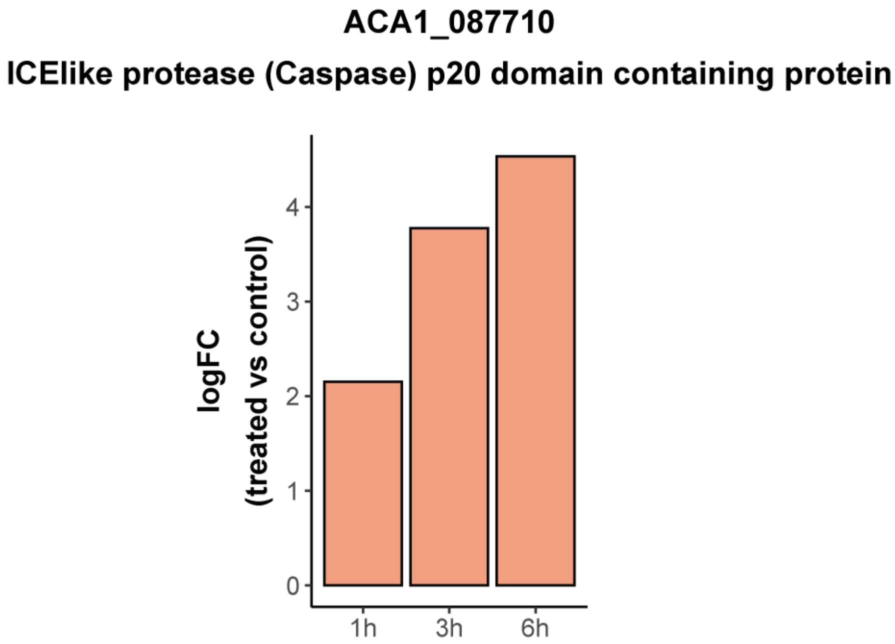

Figure S2. Expression analysis of *Acanthamoeba castellanii* metacaspase. Bar plot showing increased transcript levels of ACA1_087710, which is annotated as an ICElike protease (Caspase) p20 domain containing protein and is usually referred to as *A. castellanii* metacaspase. The Y-axis displays the logFC values (treated vs control) for the treatment times 1h, 3h, and 6h.

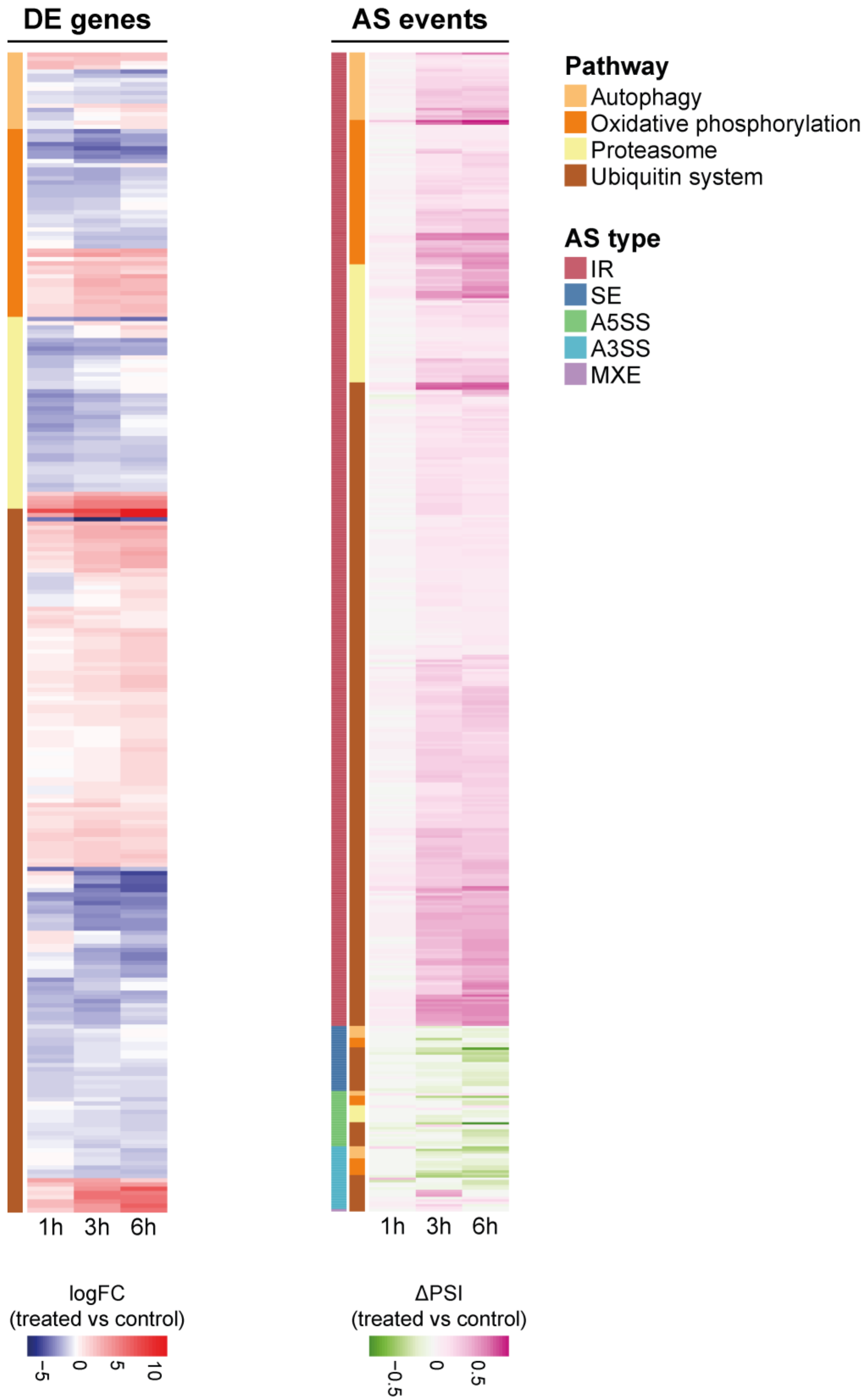

Figure S3. Expression and AS analyses of genes involved in pathways known to influence PCD. The left heatmap shows changes in transcript levels, expressed as logFC, of differentially expressed (DE) genes, while the right heatmap shows changes of differential AS events using the ΔPSI metric. In the case of IR, the ΔIR ratio calculated using the IRFinder algorithm is shown. Lists

of genes involved in pathways known to influence PCD, such as autophagy (acan04136), the proteasome (acan04136), the ubiquitin system (acan03051), and oxidative stress (which was explored by analyzing oxidative phosphorylation genes, acan00190) were retrieved from KEGG. Only significant DE genes or AS events identified in these pathways are shown.

Supplemental Tables

Supplemental Table 1. Differentially retained introns.

Time	IntronID	Geneid	Description	IRratio.ctrl	IRratio.tr	deltaIRratio	padj
1h	ACA1_375930/clean/scf7180000084685:5984-6061:-	ACA1_375930	SUN domain-containing protein [Source:UniProtKB/TrEMBL;Acc:L8HIE1]	0.139897667	0.859447	0.719549333	3.87E-10
1h	ACA1_380210/clean/scf7180000084712:32261-32346:-	ACA1_380210	DNA-(apurinic or apyrimidinic site) lyase [Source:UniProtKB/TrEMBL;Acc:L8GQH4]	0.111930333	0.804947667	0.693017333	4.49E-07
1h	ACA1_255760/clean/scf7180000084768:563824-563934:+	ACA1_255760	Acid phosphatase PHOa, putative [Source:UniProtKB/TrEMBL;Acc:L8HB67]	0.080117033	0.706225333	0.6261083	1.70E-09
1h	ACA1_207830/clean/scf7180000084757:82133-82256:+	ACA1_207830	GRAM domain-containing protein [Source:UniProtKB/TrEMBL;Acc:L8H0N0]	0.096777533	0.692390667	0.595613133	3.60E-07
1h	ACA1_095540/clean/scf7180000084645:841615-841689:-	ACA1_095540	F-box domain-containing protein [Source:UniProtKB/TrEMBL;Acc:L8GJ76]	0.127878	0.659488667	0.531610667	7.32E-05
1h	ACA1_260960/clean/scf7180000084769:565433-565581:-	ACA1_260960	Glutathione transferase family protein [Source:UniProtKB/TrEMBL;Acc:L8GFR3]	0.0295663	0.553839667	0.524273367	1.22E-13
1h	ACA1_382630/clean/scf7180000084721:88438-88554:-	ACA1_382630	LBP / BPI / CETP family, Cterminal domain containing protein [Source:UniProtKB/TrEMBL;Acc:L8GVZ7]	0.264145667	0.788115333	0.523969667	3.96E-08
1h	ACA1_074840/clean/scf7180000084608:99888-99963:+	ACA1_074840	Lecithin:cholesterol acyltransferase [Source:UniProtKB/TrEMBL;Acc:L8HF90]	0.122936367	0.623696	0.500759633	0.000240741
1h	ACA1_182190/clean/scf7180000084745:150193-150310:-	ACA1_182190	hypothetical protein	0.179485667	0.661758333	0.482272667	6.73E-05
1h	ACA1_020870/clean/scf7180000084095:29634-29720:+	ACA1_020870	Large conductance mechanosensitive channel protein [Source:UniProtKB/TrEMBL;Acc:L8GWH9]	0.133143667	0.572715667	0.439572	0.000148446
1h	ACA1_229570/clean/scf7180000084761:280946-281030:-	ACA1_229570	Oxidoreductase, short chain dehydrogenase/reductase superfamily protein [Source:UniProtKB/TrEMBL;Acc:L8H814]	0.016281067	0.429974	0.413692933	0.000270858
1h	ACA1_058280/clean/scf7180000084599:286913-287040:-	ACA1_058280	OGFr_N domain-containing protein [Source:UniProtKB/TrEMBL;Acc:L8GW80]	0.031049033	0.435887667	0.404838633	8.63E-07
1h	ACA1_177720/clean/scf7180000084743:114016-114083:-	ACA1_177720	PARP-type domain-containing protein [Source:UniProtKB/TrEMBL;Acc:L8GU65]	0.0217839	0.417801667	0.396017767	9.01E-05
1h	ACA1_175760/clean/scf7180000084741:1053649-1053722:-	ACA1_175760	Phosphoglycerate mutase family domain containing protein [Source:UniProtKB/TrEMBL;Acc:L8HH51]	0.1060176	0.487807	0.3817894	1.35E-05
1h	ACA1_382580/clean/scf7180000084721:77714-77840:+	ACA1_382580	LBP / BPI / CETP family, Cterminal domain containing protein [Source:UniProtKB/TrEMBL;Acc:L8GVZ1]	0.127876333	0.485809333	0.357933	5.06E-05
1h	ACA1_323310/clean/scf7180000084468:853-932:+	ACA1_323310	hypothetical protein	0.1370041	0.493553	0.3565489	0.038736329
1h	ACA1_329650/clean/scf7180000084562:37527-37589:-	ACA1_329650	hypothetical protein	0.063263867	0.418657	0.355393133	7.88E-05
1h	ACA1_229570/clean/scf7180000084761:280817-280885:-	ACA1_229570	Oxidoreductase, short chain dehydrogenase/reductase superfamily protein [Source:UniProtKB/TrEMBL;Acc:L8H814]	0.000249688	0.346106267	0.345856579	9.21E-05
1h	ACA1_366740/clean/scf7180000084641:493509-493608:+	ACA1_366740	Lactamase_B domain-containing protein [Source:UniProtKB/TrEMBL;Acc:L8GM47]	0.033466233	0.379303333	0.3458371	1.08E-06
1h	ACA1_364780/clean/scf7180000084641:196969-197195:-	ACA1_364780	Lung seven transmembrane receptor protein [Source:UniProtKB/TrEMBL;Acc:L8GLJ8]	0.138568	0.475318	0.33675	0.00016648
1h	ACA1_153010/clean/scf7180000084720:53815-53877:-	ACA1_153010	hypothetical protein	0.316863667	0.65228	0.335416333	0.000646572
1h	ACA1_358270/clean/scf7180000084742:2410-2534:-	ACA1_358270	Pyr_redox_2 domain-containing protein [Source:UniProtKB/TrEMBL;Acc:L8GL60]	0.006230533	0.333929667	0.327699133	0.001440429
1h	ACA1_255030/clean/scf7180000084768:453728-453822:-	ACA1_255030	hypothetical protein	0.013221133	0.339394333	0.3261732	0.000964258
1h	ACA1_363760/clean/scf7180000084641:8771-8846:-	ACA1_363760	Adenosine specific kinase [Source:UniProtKB/TrEMBL;Acc:L8GP09]	0.0506845	0.376601333	0.325916833	0.004702537
1h	ACA1_262350/clean/scf7180000084770:35433-35507:+	ACA1_262350	Flavinbinding monooxygenase-like subfamily protein [Source:UniProtKB/TrEMBL;Acc:L8H1V5]	0.095756267	0.419072667	0.3233164	0.006183517
1h	ACA1_373900/clean/scf7180000084679:186898-186962:+	ACA1_373900	Methyltransferase domain containing protein [Source:UniProtKB/TrEMBL;Acc:L8GHT5]	0.04667725	0.369234333	0.322557083	0.004707466
1h	ACA1_065830/clean/scf7180000084599:1919740-1919822:-	ACA1_065830	PH domain-containing protein [Source:UniProtKB/TrEMBL;Acc:L8GYZ9]	0.117294654	0.439342333	0.322047679	0.028444318
1h	ACA1_151500/clean/scf7180000084719:446-532:+	ACA1_151500	Ras subfamily protein [Source:UniProtKB/TrEMBL;Acc:L8GGW7]	0.1681857	0.480073	0.3118873	0.015489939
1h	ACA1_177720/clean/scf7180000084743:113679-113788:-	ACA1_177720	PARP-type domain-containing protein [Source:UniProtKB/TrEMBL;Acc:L8GU65]	0.113455533	0.423074333	0.3096188	0.001296718
1h	ACA1_167050/clean/scf7180000084736:63077-63163:-	ACA1_167050	Ras subfamily protein [Source:UniProtKB/TrEMBL;Acc:L8H0S4]	0.0620228	0.369575333	0.307552533	0.00311678
1h	ACA1_271580/clean/scf7180000084771:78428-78551:-	ACA1_271580	Protein phosphatase 1, regulatory subunit 7, putative [Source:UniProtKB/TrEMBL;Acc:L8GPQ8]	0.062891267	0.365288333	0.302397067	0.007230347
1h	ACA1_036890/clean/scf7180000084567:6275-6340:+	ACA1_036890	Ras subfamily protein [Source:UniProtKB/TrEMBL;Acc:L8H0T2]	0.092391767	0.391915667	0.2995239	0.000186635
1h	ACA1_271330/clean/scf7180000084771:21289-21464:+	ACA1_271330	hypothetical protein	0.071237367	0.3694	0.298162633	0.001700826
1h	ACA1_366150/clean/scf7180000084641:363755-363867:-	ACA1_366150	tRNA methyltransferase, putative [Source:UniProtKB/TrEMBL;Acc:L8GMF0]	0.0181219	0.307969667	0.289847767	0.000218613
1h	ACA1_027000/clean/scf7180000084464:15453-15542:-	ACA1_027000	SCP family extracellular, putative [Source:UniProtKB/TrEMBL;Acc:L8GHL6]	0.000967163	0.290369333	0.28940217	2.02E-05
1h	ACA1_289660/clean/scf7180000084777:439305-439384:+	ACA1_289660	Churchill domain containing 1, putative [Source:UniProtKB/TrEMBL;Acc:L8HKL9]	0.130170033	0.417981667	0.287811633	0.001151412
1h	ACA1_069380/clean/scf7180000084603:424419-424538:+	ACA1_069380	LNS2 domain-containing protein [Source:UniProtKB/TrEMBL;Acc:L8HCT1]	0.060599767	0.345387	0.284787233	0.003460471
1h	ACA1_025540/clean/scf7180000084448:7569-7646:+	ACA1_025540	hypothetical protein	0.0862332	0.368710333	0.282477133	0.00178538
1h	ACA1_207320/clean/scf7180000084757:52794-52950:+	ACA1_207320	Acyltransferase [Source:UniProtKB/TrEMBL;Acc:L8GY38]	0.031750533	0.313707667	0.281957133	0.005984385
1h	ACA1_384710/clean/scf7180000084730:90624-90741:+	ACA1_384710	Transmembrane receptor family protein [Source:UniProtKB/TrEMBL;Acc:L8HBG4]	0.019212673	0.301065667	0.281852993	0.000911895
1h	ACA1_033640/clean/scf7180000084559:64826-64905:+	ACA1_033640	Metallophos domain-containing protein [Source:UniProtKB/TrEMBL;Acc:L8GJ60]	0.021147633	0.300509667	0.279362033	8.86E-05
1h	ACA1_053360/clean/scf7180000084594:268950-269094:+	ACA1_053360	RING-type domain-containing protein [Source:UniProtKB/TrEMBL;Acc:L8H7D5]	0.077730033	0.355956667	0.278226633	0.008575794
1h	ACA1_036350/clean/scf7180000084565:84116-84193:+	ACA1_036350	hypothetical protein	0.032719433	0.308856667	0.276137233	0.000294005
1h	ACA1_116740/clean/scf7180000084669:839211-839288:+	ACA1_116740	Methyltransf_2 domain-containing protein [Source:UniProtKB/TrEMBL;Acc:L8H4R1]	0.0525602	0.327986	0.2754258	0.011524073
1h	ACA1_359270/clean/scf7180000084742:70646-70701:-	ACA1_359270	RAB family member (Rab7), putative [Source:UniProtKB/TrEMBL;Acc:L8GLL4]	0.029823567	0.3050327	0.275209133	0.000379985
1h	ACA1_270200/clean/scf7180000084770:1076880-1076967:-	ACA1_270200	FYVE-type domain-containing protein [Source:UniProtKB/TrEMBL;Acc:L8H522]	0.0042735	0.278419	0.2741455	0.026046638
1h	ACA1_087650/clean/scf7180000084639:90740-90831:-	ACA1_087650	CULLIN_2 domain-containing protein [Source:UniProtKB/TrEMBL;Acc:L8GUJ4]	0.082867367	0.353864	0.270996633	0.049701796
1h	ACA1_260950/clean/scf7180000084769:563733-563821:-	ACA1_260950	Ribonuclease 25A protein [Source:UniProtKB/TrEMBL;Acc:L8GG37]	0.0786186	0.344474667	0.265856067	0.001485193
1h	ACA1_174080/clean/scf7180000084741:602739-602887:-	ACA1_174080	t-SNARE coiled-coil homology domain-containing protein [Source:UniProtKB/TrEMBL;Acc:L8HGR7]	0.279602	0.544625667	0.265023667	0.03356012
1h	ACA1_075030/clean/scf7180000084608:131508-131570:+	ACA1_075030	Serine carboxypeptidase [Source:UniProtKB/TrEMBL;Acc:L8HFJ6]	0.045416567	0.306336667	0.2609201	2.02E-05
1h	ACA1_341100/clean/scf7180000084620:51340-51404:-	ACA1_341100	MFS domain-containing protein [Source:UniProtKB/TrEMBL;Acc:L8GR49]	0.157861033	0.418354667	0.260493633	0.03439599
1h	ACA1_175410/clean/scf7180000084741:949459-949562:-	ACA1_175410	Peptidase M28, putative [Source:UniProtKB/TrEMBL;Acc:L8HKC9]	0.015044483	0.273840667	0.258796183	0.001931574
1h	ACA1_390570/clean/scf7180000084740:74970-75038:-	ACA1_390570	Acetyl/propionyl CoA carboxylase, beta subunit, putative [Source:UniProtKB/TrEMBL;Acc:L8GRH2]	0.085760033	0.344130333	0.2583703	0.007056787
1h	ACA1_101530/clean/scf7180000084649:87651-87736:-	ACA1_101530	6phosphogluconate dehydrogenase NAD-binding domain containing protein [Source:UniProtKB/TrEMBL;Acc:L8GGL6]	0.151490667	0.409401	0.257910333	0.005896833
1h	ACA1_382380/clean/scf7180000084721:45368-45448:+	ACA1_382380	WD_REPEATS_REGION domain-containing protein [Source:UniProtKB/TrEMBL;Acc:L8GUA6]	0.004694833	0.258389667	0.253694833	0.00023515
1h	ACA1_048880/clean/scf7180000084583:10024-10100:+	ACA1_048880	LIM zinc-binding domain-containing protein [Source:UniProtKB/TrEMBL;Acc:L8HKD0]	0.094553467	0.346081067	0.2515276	0.02319076
1h	ACA1_315600/clean/scf7180000083939:3645-3749:-	ACA1_315600	hypothetical protein	0.0168972	0.264672333	0.247775133	3.60E-07
1h	ACA1_343290/clean/scf7180000084638:15395-15453:+	ACA1_343290	hypothetical protein	0.112074733	0.358830667	0.246755933	0.001842118
1h	ACA1_092000/clean/scf7180000084645:244529-244654:-	ACA1_092000	hypothetical protein	0.04263909	0.284280667	0.241641577	0.011524073
1h	ACA1_382920/clean/scf7180000084721:118628-118727:-	ACA1_382920	Ras subfamily protein [Source:UniProtKB/TrEMBL;Acc:L8GV31]	0.047941367	0.28674	0.238798633	0.006183517
1h	ACA1_287660/clean/scf7180000084777:56714-56803:+	ACA1_287660	Pentapeptide repeatcontaining protein [Source:UniProtKB/TrEMBL;Acc:L8HIP0]	0.068523767	0.306829	0.238305233	0.000196831
1h	ACA1_280570/clean/scf7180000084776:497885-497985:+	ACA1_280570	Znfinger in Ran binding protein and others domain containing protein [Source:UniProtKB/TrEMBL;Acc:L8H8F5]	0.0821515	0.320019333	0.237867833	0.018051754
1h	ACA1_199630/clean/scf7180000084753:282051-282138:-	ACA1_199630	PRAI domain-containing protein [Source:UniProtKB/TrEMBL;Acc:L8H3A6]	0.098893467	0.336616333	0.237722867	0.005896833
1h	ACA1_057460/clean/scf7180000084599:147412-147512:-	ACA1_057460	hypothetical protein	0.027339783	0.264371333	0.23703155	0.030326479
1h	ACA1_263890/clean/scf7180000084770:127106-127201:-	ACA1_263890	Methyltransf_11 domain-containing protein [Source:UniProtKB/TrEMBL;Acc:L8H151]	0.663383	0.899815333	0.236432333	0.007817724
1h	ACA1_002500/clean/scf7180000082931:8966-9042:+	ACA1_002500	hypothetical protein	0.271974	0.506611667	0.234637667	0.02118103
1h	ACA1_203980/clean/scf7180000084754:243272-243361:+	ACA1_203980	hypothetical protein	0.023849067	0.2584693	0.234620233	0.000218613
1h	ACA1_391420/clean/scf7180000084740:169174-169273:+	ACA1_391420	hypothetical protein	0.00312925	0.236792633	0.233663383	3.91E-05
1h	ACA1_044920/clean/scf7180000084576:59578-59775:-	ACA1_044920	hypothetical protein	0.028394033	0.259043	0.230648967	8.56E-05
1h	ACA1_097540/clean/scf7180000084645:1277344-1277416:-	ACA1_097540	Serine/threonine kinase [Source:UniProtKB/TrEMBL;Acc:L8GK27]	0.013087	0.236432033	0.223345033	0.002130783
1h	ACA1_309360/clean/scf7180000083758:11988-12070:-	ACA1_309360	hypothetical protein	0.001153403	0.224349067	0.223195663	0.006123257
1h	ACA1_329650/clean/scf7180000084562:37997-38061:-	ACA1_329650	hypothetical protein	0.0049707	0.227581233	0.222610533	4.59E-05
1h	ACA1_373540/clean/scf7180000084679:96177-96301:-	ACA1_373540	hypothetical protein	0.034372467	0.25611	0.221737533	0.008308229
1h	ACA1_349660/clean/scf7180000084690:31077-31713:+	ACA1_349660	BAT1 protein [Source:UniProtKB/TrEMBL;Acc:L8GJC9]	0.093203733	0.314447333	0.2212436	0.007637688
1h	ACA1_053340/clean/scf7180000084594:265973-266111:-	ACA1_053340	Dynein light intermediate chain [Source:UniProtKB/TrEMBL;Acc:L8H840]	0.063985467	0.284996333	0.221010867	0.00508973
1h	ACA1_296250/clean/scf7180000084777:1306313-1306402:+	ACA1_296250	Rasrelated protein Rab-32, putative [Source:UniProtKB/TrEMBL;Acc:L8HJZ6]	0.0532768	0.271622267	0.218345467	0.015290563
1h	ACA1_103630/clean/scf7180000084653:47179-47296:+	ACA1_103630	Peroxidase [Source:UniProtKB/TrEMBL;Acc:L8GCR8]	0.000864403	0.217689667	0.216825263	0.00083667
1h	ACA1_266800/clean/scf7180000084770:716880-716965:+	ACA1_266800	Protein kinase domain containing protein [Source:UniProtKB/TrEMBL;Acc:L8H3W0]	0.079894567	0.293068333	0.213173767	0.002339739
1h	ACA1_189640/clean/scf7180000084748:71362-71558:-	ACA1_189640	Bifunctional aspartate kinase/diaminopimelate decarboxylase protein [Source:UniProtKB/TrEMBL;Acc:L8GEY3]	0.0402223	0.250422333	0.210200033	0.000727696
1h	ACA1_052600/clean/scf7180000084594:121647-121712:+	ACA1_052600	hypothetical protein	0.0561443	0.265932333	0.209788033	0.030294142
1h	ACA1_006200/clean/scf7180000083029:69118-69193:-	ACA1_006200	hypothetical protein	0.01878257	0.225619733	0.206837163	0.028517598
1h	ACA1_296570/clean/scf7180000084777:1386723-1386820:+	ACA1_296570	TRANSMEMBRANE PROTEIN, putative [Source:UniProtKB/TrEMBL;Acc:L8HIX9]	0.078405633	0.283990333	0.2055847	0.028914868
1h	ACA1_154690/clean/scf7180000084722:78274-78372:+	ACA1_154690	unspecified product	0.012047377	0.216967933	0.204920557	0.002483901
1h	ACA1_098950/clean/scf7180000084646:73861-73927:+	ACA1_098950	hypothetical protein	0.0218566	0.2264709	0.2046143	0.007773343
1h	ACA1_341080/clean/scf7180000084620:46511-46581:+	ACA1_341080	RING-type domain-containing protein [Source:UniProtKB/TrEMBL;Acc:L8GS99]	0.090455	0.294706667	0.204251667	0.02319076
1h	ACA1_216320/clean/scf7180000084758:467384-467533:+	ACA1_216320	Homogentisate 1,2dioxygenase [Source:UniProtKB/TrEMBL;Acc:L8GQ83]	0.01526652	0.215891367	0.200624847	0.002297927
1h	ACA1_283300/clean/scf7180000084776:810856-810964:-	ACA1_283300	DEP domain-containing protein [Source:UniProtKB/TrEMBL;Acc:L8H8T3]	0.1327643	0.331923333	0.199159033	0.047992287
1h	ACA1_193110/clean/scf7180000084749:185314-185482:-	ACA1_193110	hypothetical protein	0.1059345	0.304521	0.1985865	0.03701896
1h	ACA1_095680/clean/scf7180000084645:878735-878801:+	ACA1_095680	hypothetical protein	0.016202341	0.211617	0.195414659	0.000132474
1h	ACA1_048110/clean/scf7180000084582:24254-24512:-	ACA1_048110	Serine carboxypeptidase s28 protein [Source:UniProtKB/TrEMBL;Acc:L8GD90]	0.0398989	0.232939333	0.193040433	0.006290775
1h	ACA1_227080/clean/scf7180000084761:94749-94826:+	ACA1_227080	Uracil-DNA glycosylase [Source:UniProtKB/TrEMBL;Acc:L8HA23]	0.015951433	0.208941067	0.192989633	0.000879059
1h	ACA1_174780/clean/scf7180000084741:795639-795802:-	ACA1_174780	VanY domain-containing protein [Source:UniProtKB/TrEMBL;Acc:L8HK80]	0.167787667	0.359810667	0.192023	0.035830184
1h	ACA1_363780/clean/scf7180000084641:13637-13723:+	ACA1_363780	Vtype ATPase, D subunit [Source:UniProtKB/TrEMBL;Acc:L8GM99]	0.002435177	0.194277067	0.19184189	2.42E-07
1h	ACA1_106720/clean/scf7180000084660:109896-110061:-	ACA1_106720	CID domain-containing protein [Source:UniProtKB/TrEMBL;Acc:L8GQ94]	0.0660501	0.257768667	0.191718567	0.026891046
1h	ACA1_187740/clean/scf7180000084747:87434-87552:-	ACA1_187740	hypothetical protein	0.1371592	0.328786667	0.191627467	0.026225881
1h	ACA1_272960/clean/scf7180000084773:5189-5354:-	ACA1_272960	Ras-GAP domain-containing protein [Source:UniProtKB/TrEMBL;Acc:L8GFH7]	0.0302028	0.221525733	0.191322933	0.005724428
1h	ACA1_076410/clean/scf7180000084612:66157-66239:-	ACA1_076410	hypothetical protein	0.022847433	0.213987667	0.191140233	2.02E-05
1h	ACA1_101080/clean/scf7180000084649:35292-35381:-	ACA1_101080	Purple acid phosphatase [Source:UniProtKB/TrEMBL;Acc:L8GGG9]	0.013242873	0.204351333	0.19110846	6.22E-05
1h	ACA1_295350/clean/scf7180000084777:1110414-1110529:-	ACA1_295350	RNA_bind domain-containing protein [Source:UniProtKB/TrEMBL;Acc:L8HJR1]	0.076986667	0.267954	0.190967333	0.025424168
1h	ACA1_185700/clean/scf7180000084746:114487-114556:+	ACA1_185700	EF hand domain containing protein [Source:UniProtKB/TrEMBL;Acc:L8H786]	0.0512075	0.2386287	0.1874212	0.013103317
1h	ACA1_396930/clean/scf7180000084759:151004-151197:+	ACA1_396930	hypothetical protein	0.0160488	0.201666667	0.185617867	0.0011877
1h	ACA1_320100/clean/scf7180000084352:7362-7452:+	ACA1_320100	VWFA domain-containing protein [Source:UniProtKB/TrEMBL;Acc:L8H729]	0.01390052	0.198816333	0.184915813	0.000189227
1h	ACA1_122370/clean/scf7180000084682:135028-135112:-	ACA1_122370	Short chain oxidoreductase [Source:UniProtKB/TrEMBL;Acc:L8GEE8]	0.105485333	0.28893	0.183444667	0.032522133
1h	ACA1_142590/clean/scf7180000084708:191916-192020:+	ACA1_142590	unspecified product	0.042299853	0.224846	0.182546147	0.00158843
1h	ACA1_043190/clean/scf7180000084573:137070-137138:-	ACA1_043190	hypothetical protein	0.027234	0.208741	0.181507	0.000323806
1h	ACA1_287570/clean/scf7180000084777:38466-38543:+	ACA1_287570	hypothetical protein	0.0412458	0.220171	0.1789252	0.005473996
1h	ACA1_385700/clean/scf7180000084730:241652-241741:+	ACA1_385700	F-box domain-containing protein [Source:UniProtKB/TrEMBL;Acc:L8HAP4]	0.000212608	0.179082147	0.178869539	0.011689812
1h	ACA1_142680/clean/scf7180000084708:218869-219110:+	ACA1_142680	unspecified product	0.033705951	0.2115488	0.177842849	0.001821731
1h	ACA1_230310/clean/scf7180000084761:342605-342687:+	ACA1_230310	PAS domain-containing protein [Source:UniProtKB/TrEMBL;Acc:L8HBA3]	0.024757267	0.202437767	0.1776805	0.009394093
1h	ACA1_255900/clean/scf7180000084768:579178-579284:+	ACA1_255900	DEP domain-containing protein [Source:UniProtKB/TrEMBL;Acc:L8HA90]	0.0601444	0.236667933	0.176523533	0.003418007
1h	ACA1_107530/clean/scf7180000084660:216036-216160:-	ACA1_107530	UBP-type domain-containing protein [Source:UniProtKB/TrEMBL;Acc:L8GMW8]	0.00510783	0.181370833	0.176263003	3.37E-05
1h	ACA1_144440/clean/scf7180000084709:244650-244859:+	ACA1_144440	SNF2 family Nterminal domain containing protein [Source:UniProtKB/TrEMBL;Acc:L8HFN1]	0.036675733	0.2122289	0.175553167	0.000699727
1h	ACA1_278800/clean/scf7180000084776:242961-243063:+	ACA1_278800	LIM zinc-binding domain-containing protein [Source:UniProtKB/TrEMBL;Acc:L8H5V5]	0.150940533	0.324779	0.173838467	0.027350435
1h	ACA1_144930/clean/scf7180000084710:41854-41980:-	ACA1_144930	N-acetyltransferase domain-containing protein [Source:UniProtKB/TrEMBL;Acc:L8GCT6]	0.101927533	0.2745634	0.172635867	0.041425824
1h	ACA1_310500/clean/scf7180000083773:11761-11822:+	ACA1_310500	hypothetical protein	0.0047619	0.177339	0.1725771	0.000173988
1h	ACA1_206370/clean/scf7180000084757:17262-17334:+	ACA1_206370	MOSC domain-containing protein [Source:UniProtKB/TrEMBL;Acc:L8GY21]	0.003627527	0.1738747	0.170247173	2.34E-05
1h	ACA1_382900/clean/scf7180000084721:112168-112290:-	ACA1_382900	ADPribosylation factor, putative [Source:UniProtKB/TrEMBL;Acc:L8GUW5]	0.027296133	0.196185333	0.1688892	0.000823162
1h	ACA1_014620/clean/scf7180000083772:1382-1486:-	ACA1_014620	hypothetical protein	0.0186948	0.1860403	0.1673455	0.00589889
1h	ACA1_017740/clean/scf7180000083876:7199-7289:+	ACA1_017740	5'-3' exoribonuclease 1 [Source:UniProtKB/TrEMBL;Acc:L8GMR4]	0.01213801	0.179156067	0.167018057	0.002586138
1h	ACA1_252210/clean/scf7180000084768:146713-146801:+	ACA1_252210	Protein kinase domain-containing protein [Source:UniProtKB/TrEMBL;Acc:L8HAM0]	0.0171932	0.184042533	0.166849333	0.008308229
1h	ACA1_031270/clean/scf7180000084506:13798-13878:+	ACA1_031270	hypothetical protein	0.0187266	0.185108667	0.166382067	0.015890625
1h	ACA1_105970/clean/scf7180000084660:40538-40615:-	ACA1_105970	PhytanoylCoA dioxygenase family protein [Source:UniProtKB/TrEMBL;Acc:L8GQ69]	0.008845237	0.175098667	0.16625343	2.56E-05
1h	ACA1_180600/clean/scf7180000084744:62961-63079:+	ACA1_180600	hypothetical protein	0.039870433	0.2043648	0.164494367	0.000563943
1h	ACA1_377830/clean/scf7180000084711:127015-127081:+	ACA1_377830	UBIQUITIN_CONJUGAT_2 domain-containing protein [Source:UniProtKB/TrEMBL;Acc:L8GSV2]	0.035397467	0.199441333	0.164043867	0.026421608
1h	ACA1_065970/clean/scf7180000084599:1963947-1964010:-	ACA1_065970	Usp domain-containing protein [Source:UniProtKB/TrEMBL;Acc:L8H061]	0.024634533	0.187433433	0.1627989	0.005579221
1h	ACA1_363760/clean/scf7180000084641:8925-8996:-	ACA1_363760	Adenosine specific kinase [Source:UniProtKB/TrEMBL;Acc:L8GP09]	0.0419404	0.202654467	0.160714067	0.022049146
1h	ACA1_214850/clean/scf7180000084758:245642-245741:+	ACA1_214850	LIM zinc-binding domain-containing protein [Source:UniProtKB/TrEMBL;Acc:L8GSG1]	0.072599433	0.231011	0.158411567	0.024422122
1h	ACA1_271330/clean/scf7180000084771:20879-20960:+	ACA1_271330	hypothetical protein	0.042074633	0.199907233	0.1578326	0.02962858
1h	ACA1_183960/clean/scf7180000084745:528043-528144:-	ACA1_183960	Phosphoesterase family protein [Source:UniProtKB/TrEMBL;Acc:L8H8F6]	0.02709902	0.183576433	0.156477413	0.001419613
1h	ACA1_016360/clean/scf7180000083820:5823-5920:+	ACA1_016360	DUF4392 domain-containing protein [Source:UniProtKB/TrEMBL;Acc:L8GQ34]	0.017994457	0.174458033	0.156463577	0.000469445
1h	ACA1_130560/clean/scf7180000084698:108441-108510:+	ACA1_130560	Glucosyl hydrolase family protein [Source:UniProtKB/TrEMBL;Acc:L8GRU3]	0	0.155995767	0.155995767	0.008464102
1h	ACA1_189640/clean/scf7180000084748:70409-70499:-	ACA1_189640	Bifunctional aspartate kinase/diaminopimelate decarboxylase protein [Source:UniProtKB/TrEMBL;Acc:L8GEY3]	0.00902257	0.164754267	0.155731697	0.000935516
1h	ACA1_382920/clean/scf7180000084721:119288-119370:-	ACA1_382920	Ras subfamily protein [Source:UniProtKB/TrEMBL;Acc:L8GV31]	0.0311637	0.186785767	0.155622067	0.01059235
1h	ACA1_189640/clean/scf7180000084748:70712-70803:-	ACA1_189640	Bifunctional aspartate kinase/diaminopimelate decarboxylase protein [Source:UniProtKB/TrEMBL;Acc:L8GEY3]	0.00768302	0.162598667	0.154915647	0.000754008
1h	ACA1_295880/clean/scf7180000084777:1250622-1250693:+	ACA1_295880	Malic enzyme [Source:UniProtKB/TrEMBL;Acc:L8HM80]	0.008379673	0.1607474	0.152367727	0.00589889
1h	ACA1_102790/clean/scf7180000084652:28296-28369:-	ACA1_102790	hypothetical protein	0.0340025	0.186319	0.1523165	0.006613393
1h	ACA1_173140/clean/scf7180000084741:410239-410321:+	ACA1_173140	Deacetylase sirtuin-type domain-containing protein [Source:UniProtKB/TrEMBL;Acc:L8HJX9]	0.011403803	0.163676633	0.15227283	0.001419613

1h	ACA1_271330/clean/scf7180000084771:21570-21663:+	ACA1_271330	hypothetical protein	0.028617303	0.1803406	0.151723297	0.018835188
1h	ACA1_208140/clean/scf7180000084757:113957-114035:-	ACA1_208140	ER lumen proteinretaining receptor-like protein [Source:UniProtKB/TrEMBL;Acc:L8GYA7]	0.0627453	0.213545667	0.150800367	0.025764638
1h	ACA1_156030/clean/scf7180000084723:6173-6256:+	ACA1_156030	hypothetical protein	0.016666667	0.165461967	0.1487953	0.020389469
1h	ACA1_053670/clean/scf7180000084594:313216-313411:+	ACA1_053670	CYP, putative [Source:UniProtKB/TrEMBL;Acc:L8H7F1]	0.0212447	0.168598667	0.147353967	0.000196944
1h	ACA1_325170/clean/scf7180000084475:15371-15565:-	ACA1_325170	MHD domain-containing protein [Source:UniProtKB/TrEMBL;Acc:L8GPP2]	0.001661794	0.1487165	0.147054706	0.000183426
1h	ACA1_037190/clean/scf7180000084567:80442-80529:+	ACA1_037190	Aminotran_5 domain-containing protein [Source:UniProtKB/TrEMBL;Acc:L8H0V4]	0.068364933	0.215351333	0.1469864	0.034482543
1h	ACA1_383560/clean/scf7180000084721:208395-208461:-	ACA1_383560	TBC domain containing protein [Source:UniProtKB/TrEMBL;Acc:L8GW44]	0.02612068	0.172376867	0.146256187	0.030288999
1h	ACA1_149540/clean/scf7180000084715:176960-177057:+	ACA1_149540	VPS37 C-terminal domain-containing protein [Source:UniProtKB/TrEMBL;Acc:L8HEF0]	0.034117833	0.178715567	0.144597733	0.018493862
1h	ACA1_175510/clean/scf7180000084741:974008-974097:+	ACA1_175510	Xylose isomerase [Source:UniProtKB/TrEMBL;Acc:L8HKE1]	0.007071983	0.150350833	0.14327885	4.14E-05
1h	ACA1_106710/clean/scf7180000084660:106517-106629:+	ACA1_106710	TPR_REGION domain-containing protein [Source:UniProtKB/TrEMBL;Acc:L8GMK9]	0.069934933	0.211622267	0.141687333	0.02319076
1h	ACA1_246780/clean/scf7180000084763:580874-580955:-	ACA1_246780	Ras subfamily protein [Source:UniProtKB/TrEMBL;Acc:L8GK82]	0.012417367	0.154090933	0.141673567	0.000298785
1h	ACA1_018760/clean/scf7180000083912:11268-11348:+	ACA1_018760	Cell cycle control protein [Source:UniProtKB/TrEMBL;Acc:L8HJG5]	0.013246603	0.1543503	0.141103697	0.000323806
1h	ACA1_060250/clean/scf7180000084599:626597-626697:-	ACA1_060250	Alpha-carbonic anhydrase domain-containing protein [Source:UniProtKB/TrEMBL;Acc:L8GXK3]	0.0211022	0.161332467	0.140230267	0.001524301
1h	ACA1_069320/clean/scf7180000084603:409362-409454:+	ACA1_069320	Eukaryotic initiation factor, putative [Source:UniProtKB/TrEMBL;Acc:L8HF00]	0.025321833	0.16554	0.140218167	7.32E-05
1h	ACA1_200100/clean/scf7180000084753:390262-390353:-	ACA1_200100	hypothetical protein	0.044602667	0.1844948	0.139892133	0.032168457
1h	ACA1_108220/clean/scf7180000084661:17262-17336:+	ACA1_108220	hypothetical protein	0.021297017	0.160414367	0.13911735	0.019124414
1h	ACA1_296110/clean/scf7180000084777:1298314-1298423:-	ACA1_296110	MORN repeat variantcontaining protein [Source:UniProtKB/TrEMBL;Acc:L8HLH8]	0.001352681	0.1395158	0.138163119	3.60E-07
1h	ACA1_159850/clean/scf7180000084724:393288-393380:+	ACA1_159850	hypothetical protein	0.0310352	0.168626533	0.137591333	0.009364913
1h	ACA1_152930/clean/scf7180000084720:36638-36735:-	ACA1_152930	PG_binding_1 domain-containing protein [Source:UniProtKB/TrEMBL;Acc:L8HF34]	0.00010214	0.137094633	0.136992494	0.000686462
1h	ACA1_380340/clean/scf7180000084712:41224-41301:-	ACA1_380340	Leucine rich repeat-containing protein [Source:UniProtKB/TrEMBL;Acc:L8GNJ3]	0.0104396	0.147257467	0.136817867	0.011476926
1h	ACA1_208430/clean/scf7180000084757:190256-190365:+	ACA1_208430	hypothetical protein	0.012275267	0.1486152	0.136339933	0.023031319
1h	ACA1_272960/clean/scf7180000084773:2315-2374:-	ACA1_272960	Ras-GAP domain-containing protein [Source:UniProtKB/TrEMBL;Acc:L8GFH7]	0.0498503	0.185062	0.1352117	0.008464102
1h	ACA1_037190/clean/scf7180000084567:79804-79879:+	ACA1_037190	Aminotran_5 domain-containing protein [Source:UniProtKB/TrEMBL;Acc:L8H0V4]	0.051673267	0.186664667	0.1349914	0.012791591
1h	ACA1_230540/clean/scf7180000084761:374608-374681:+	ACA1_230540	hypothetical protein	0.0159826	0.150729667	0.134747067	0.007230347
1h	ACA1_108210/clean/scf7180000084661:13246-13334:-	ACA1_108210	PH domain containing protein [Source:UniProtKB/TrEMBL;Acc:L8GD50]	0.009561633	0.143611567	0.134049933	0.005942411
1h	ACA1_012990/clean/scf7180000083742:17999-18072:+	ACA1_012990	ATP-dependent DNA helicase [Source:UniProtKB/TrEMBL;Acc:L8H692]	0	0.132989433	0.132989433	0.007720273
1h	ACA1_080580/clean/scf7180000084618:39260-39370:+	ACA1_080580	t-SNARE coiled-coil homology domain-containing protein [Source:UniProtKB/TrEMBL;Acc:L8HBP6]	0.037954133	0.170635867	0.132681733	0.019679819
1h	ACA1_396930/clean/scf7180000084759:151602-151744:+	ACA1_396930	hypothetical protein	0.004877917	0.135612	0.130734083	0.000823162
1h	ACA1_320790/clean/scf7180000084416:23487-23555:-	ACA1_320790	RUN domain-containing protein [Source:UniProtKB/TrEMBL;Acc:L8GTR9]	0.040628633	0.171183367	0.130554733	0.017066315
1h	ACA1_174110/clean/scf7180000084741:614339-614452:+	ACA1_174110	hypothetical protein	0.043006467	0.172122333	0.129115867	0.008464102
1h	ACA1_216300/clean/scf7180000084758:461568-461772:+	ACA1_216300	Ras family protein [Source:UniProtKB/TrEMBL;Acc:L8GR60]	0.026247217	0.154210733	0.127963517	0.026952567
1h	ACA1_144360/clean/scf7180000084709:215192-215302:+	ACA1_144360	Repressor of RNA polymerase III transcription [Source:UniProtKB/TrEMBL;Acc:L8HFX0]	0.00936268	0.134933967	0.125571287	0.000911895
1h	ACA1_362600/clean/scf7180000084624:110081-110141:+	ACA1_362600	hypothetical protein	0.0547767	0.179601367	0.124824667	0.040121377
1h	ACA1_287660/clean/scf7180000084777:57007-57073:+	ACA1_287660	Pentapeptide repeatcontaining protein [Source:UniProtKB/TrEMBL;Acc:L8HIP0]	0.005067133	0.129638067	0.124570933	0.000935516
1h	ACA1_151330/clean/scf7180000084716:104444-104535:-	ACA1_151330	Purple acid phosphatase [Source:UniProtKB/TrEMBL;Acc:L8H0Q3]	0.012655377	0.134254367	0.12159899	0.001722535
1h	ACA1_278910/clean/scf7180000084776:273908-273990:-	ACA1_278910	hypothetical protein	0.024140133	0.142557533	0.1184174	0.017989562
1h	ACA1_098400/clean/scf7180000084646:3005-3124:+	ACA1_098400	MFS domain-containing protein [Source:UniProtKB/TrEMBL;Acc:L8H5Q6]	0.091809733	0.209199	0.117389267	0.040045096
1h	ACA1_305550/clean/scf7180000083609:19660-19739:+	ACA1_305550	Folate gamma-glutamyl hydrolase [Source:UniProtKB/TrEMBL;Acc:L8GKY1]	0.006231493	0.1221614	0.115929907	0.000120577
1h	ACA1_261270/clean/scf7180000084769:625859-625926:+	ACA1_261270	Methyltransferase domain containing protein [Source:UniProtKB/TrEMBL;Acc:L8GG55]	0.0479001	0.163715667	0.115815567	0.015048504
1h	ACA1_053290/clean/scf7180000084594:249958-250104:+	ACA1_053290	V-type proton ATPase proteolipid subunit [Source:UniProtKB/TrEMBL;Acc:L8H836]	0.002105607	0.11613026	0.114024653	9.74E-06
1h	ACA1_325150/clean/scf7180000084475:10629-10700:+	ACA1_325150	Rab7/RabGfamily small GTPase [Source:UniProtKB/TrEMBL;Acc:L8GPH2]	0.013040573	0.126896667	0.113856093	0.002427861
1h	ACA1_296560/clean/scf7180000084777:1382605-1382716:-	ACA1_296560	CitMHS domain-containing protein [Source:UniProtKB/TrEMBL;Acc:L8HLL2]	0.014744423	0.127928233	0.11318381	0.028914868
1h	ACA1_322090/clean/scf7180000084459:3302-3408:-	ACA1_322090	Myosin head (Motor domain) domain containing protein [Source:UniProtKB/TrEMBL;Acc:L8GN06]	0.012748627	0.125891167	0.11314254	0.002620414
1h	ACA1_246780/clean/scf7180000084763:580698-580795:-	ACA1_246780	Ras subfamily protein [Source:UniProtKB/TrEMBL;Acc:L8GK82]	0.00467577	0.1178145	0.11313873	0.000362621
1h	ACA1_075020/clean/scf7180000084608:128325-128392:-	ACA1_075020	Rab7, putative [Source:UniProtKB/TrEMBL;Acc:L8HEH5]	0.013178133	0.1246906	0.111512467	0.000132474
1h	ACA1_164510/clean/scf7180000084729:233223-233319:+	ACA1_164510	Leucine rich repeat domain containing protein [Source:UniProtKB/TrEMBL;Acc:L8GU14]	0.0003367	0.1108138	0.1104771	0.015048504
1h	ACA1_235920/clean/scf7180000084762:385520-385599:-	ACA1_235920	RRM domain-containing protein [Source:UniProtKB/TrEMBL;Acc:L8H0M8]	0.005931208	0.115238367	0.109307158	0.003437436
1h	ACA1_278000/clean/scf7180000084776:150636-150704:+	ACA1_278000	Carrier superfamily protein [Source:UniProtKB/TrEMBL;Acc:L8H5S3]	0.010463297	0.119013333	0.108550037	0.005509718
1h	ACA1_191480/clean/scf7180000084749:673-766:-	ACA1_191480	CS domain containing protein [Source:UniProtKB/TrEMBL;Acc:L8GMZ3]	0.00136892	0.109881677	0.108512757	0.001041666
1h	ACA1_201470/clean/scf7180000084753:559589-559680:-	ACA1_201470	Rasrelated protein Rab-2B, putative [Source:UniProtKB/TrEMBL;Acc:L8H2N2]	0.006711197	0.114684633	0.107973437	0.000661032
1h	ACA1_338180/clean/scf7180000084604:48884-48941:-	ACA1_338180	hypothetical protein	0.008663296	0.115951	0.107287704	0.000186267
1h	ACA1_376730/clean/scf7180000084685:147951-148022:-	ACA1_376730	Glutamate dehydrogenase [Source:UniProtKB/TrEMBL;Acc:L8HFB5]	0.000458142	0.107433157	0.106975015	7.32E-05
1h	ACA1_063740/clean/scf7180000084599:1492697-1492779:-	ACA1_063740	Transmembrane protein [Source:UniProtKB/TrEMBL;Acc:L8GXB1]	0.011446733	0.1165257	0.105078967	0.020741227
1h	ACA1_035580/clean/scf7180000084564:93699-93769:+	ACA1_035580	hypothetical protein	0.039168867	0.141940867	0.102772	0.011956106
1h	ACA1_107440/clean/scf7180000084660:197117-197262:+	ACA1_107440	Purple acid phosphatase [Source:UniProtKB/TrEMBL;Acc:L8GNJ7]	0.00554139	0.106502633	0.100961243	0.000323806
1h	ACA1_357160/clean/scf7180000084735:11827-11943:-	ACA1_357160	Actin subfamily protein [Source:UniProtKB/TrEMBL;Acc:L8HDB7]	0.178499	0.0678281	-0.1106709	0.027203781
1h	ACA1_080550/clean/scf7180000084618:31513-31572:+	ACA1_080550	FAD binding domain containing protein [Source:UniProtKB/TrEMBL;Acc:L8HBU6]	0.309999333	0.137663967	-0.172335367	0.041036117
1h	ACA1_333280/clean/scf7180000084581:30055-30249:-	ACA1_333280	Gpatch domain containing protein [Source:UniProtKB/TrEMBL;Acc:L8HIC3]	0.419273	0.205757	-0.213516	0.026855199
1h	ACA1_252570/clean/scf7180000084768:184026-184101:+	ACA1_252570	CBS domain containing protein [Source:UniProtKB/TrEMBL;Acc:L8HAN6]	0.37444	0.1374363	-0.2370037	0.036450983
1h	ACA1_265500/clean/scf7180000084770:495229-495291:-	ACA1_265500	F-box domain-containing protein [Source:UniProtKB/TrEMBL;Acc:L8H275]	0.403977333	0.156361333	-0.247616	0.001524301
1h	ACA1_094060/clean/scf7180000084645:688716-688846:+	ACA1_094060	START domain-containing protein [Source:UniProtKB/TrEMBL;Acc:L8GJ20]	0.415722667	0.167048667	-0.248674	0.049893191
1h	ACA1_388760/clean/scf7180000084733:159727-159780:+	ACA1_388760	hypothetical protein	0.825137667	0.574206	-0.250931667	0.039212998
1h	ACA1_360600/clean/scf7180000084622:168353-168462:+	ACA1_360600	hypothetical protein	0.311013333	0.031112267	-0.279901067	3.10E-06
1h	ACA1_178730/clean/scf7180000084743:271136-271233:+	ACA1_178730	Histidine acid phosphatase superfamily protein [Source:UniProtKB/TrEMBL;Acc:L8GSM6]	0.376185667	0.045821467	-0.3303642	0.017303641
1h	ACA1_232850/clean/scf7180000084762:86755-86898:+	ACA1_232850	hypothetical protein	0.384522	0.042515167	-0.342006833	0.008464102
1h	ACA1_107630/clean/scf7180000084660:235791-235901:+	ACA1_107630	DNA-(apurinic or apyrimidinic site) lyase [Source:UniProtKB/TrEMBL;Acc:L8GMX8]	0.361762667	0.0179754	-0.343787267	0.009682335
3h	ACA1_129340/clean/scf7180000084698:6522-6619:+	ACA1_129340	Methyltransf_11 domain-containing protein [Source:UniProtKB/TrEMBL;Acc:L8GNW8]	0.011494267	0.876197	0.864702733	5.53E-09
3h	ACA1_368140/clean/scf7180000084664:65474-65569:-	ACA1_368140	Glutamine amidotransferase type-2 domain-containing protein [Source:UniProtKB/TrEMBL;Acc:L8H040]	0.0128205	0.871806	0.8589855	2.22E-08
3h	ACA1_382630/clean/scf7180000084721:88438-88554:-	ACA1_382630	LBP / BPI / CETP family, Cterminal domain containing protein [Source:UniProtKB/TrEMBL;Acc:L8GVZ7]	0.0686254	0.917814667	0.849189267	1.34E-26
3h	ACA1_229570/clean/scf7180000084761:280946-281030:-	ACA1_229570	Oxidoreductase, short chain dehydrogenase/reductase superfamily protein [Source:UniProtKB/TrEMBL;Acc:L8H814]	0.007202563	0.85314	0.845937437	3.05E-11
3h	ACA1_177720/clean/scf7180000084743:113679-113788:-	ACA1_177720	PARP-type domain-containing protein [Source:UniProtKB/TrEMBL;Acc:L8GU65]	0.0334527	0.876458667	0.843005967	6.75E-16
3h	ACA1_255760/clean/scf7180000084768:563824-563934:+	ACA1_255760	Acid phosphatase PHOa, putative [Source:UniProtKB/TrEMBL;Acc:L8HB67]	0.083819067	0.922105	0.838285933	1.13E-16
3h	ACA1_237890/clean/scf7180000084763:39673-39763:+	ACA1_237890	SCP2 sterol transfer family protein [Source:UniProtKB/TrEMBL;Acc:L8GM48]	0.117193733	0.948682667	0.831488933	3.50E-22
3h	ACA1_129340/clean/scf7180000084698:6027-6113:+	ACA1_129340	Methyltransf_11 domain-containing protein [Source:UniProtKB/TrEMBL;Acc:L8GNW8]	0.002262787	0.829787667	0.82752488	6.08E-11
3h	ACA1_255030/clean/scf7180000084768:453728-453822:-	ACA1_255030	hypothetical protein	0.00268976	0.822413	0.81972324	7.69E-12
3h	ACA1_243850/clean/scf7180000084763:363141-363235:+	ACA1_243850	Ras-GAP domain-containing protein [Source:UniProtKB/TrEMBL;Acc:L8GJZ5]	0.014492767	0.833456667	0.8189639	3.52E-07
3h	ACA1_382580/clean/scf7180000084721:77714-77840:+	ACA1_382580	LBP / BPI / CETP family, Cterminal domain containing protein [Source:UniProtKB/TrEMBL;Acc:L8GVZ1]	0.053039033	0.862504333	0.8094653	2.95E-26
3h	ACA1_278900/clean/scf7180000084776:271265-271352:+	ACA1_278900	ANK_REP_REGION domain-containing protein [Source:UniProtKB/TrEMBL;Acc:L8H5W6]	0.01272392	0.821187667	0.808463747	1.06E-13
3h	ACA1_071580/clean/scf7180000084603:997734-997799:-	ACA1_071580	GOLD domain-containing protein [Source:UniProtKB/TrEMBL;Acc:L8HGH0]	0.004362577	0.806044667	0.80168209	9.06E-12
3h	ACA1_074840/clean/scf7180000084608:99888-99963:+	ACA1_074840	Lecithin:cholesterol acyltransferase [Source:UniProtKB/TrEMBL;Acc:L8HF90]	0.003117163	0.797852333	0.79473517	1.44E-11
3h	ACA1_072500/clean/scf7180000084603:1217166-1217259:-	ACA1_072500	F-box domain-containing protein [Source:UniProtKB/TrEMBL;Acc:L8HFX9]	0.007843133	0.799204333	0.7913612	6.40E-10
3h	ACA1_025540/clean/scf7180000084448:7569-7646:+	ACA1_025540	hypothetical protein	0.050401767	0.840816667	0.7904149	4.81E-11
3h	ACA1_287360/clean/scf7180000084777:16846-17004:-	ACA1_287360	Acid phosphatase [Source:UniProtKB/TrEMBL;Acc:L8HIM1]	0.121344533	0.895913333	0.7745688	5.12E-10
3h	ACA1_072500/clean/scf7180000084603:1216961-1217048:-	ACA1_072500	F-box domain-containing protein [Source:UniProtKB/TrEMBL;Acc:L8HFX9]	0	0.767543	0.767543	2.73E-08
3h	ACA1_389110/clean/scf7180000084733:198465-198568:+	ACA1_389110	hypothetical protein	0.016481143	0.782837667	0.766356523	1.32E-15
3h	ACA1_358270/clean/scf7180000084742:2410-2534:-	ACA1_358270	Pyr_redox_2 domain-containing protein [Source:UniProtKB/TrEMBL;Acc:L8GL60]	0.047637667	0.811169333	0.763531667	7.50E-07
3h	ACA1_072500/clean/scf7180000084603:1218524-1218611:-	ACA1_072500	F-box domain-containing protein [Source:UniProtKB/TrEMBL;Acc:L8HFX9]	0.019747887	0.780830333	0.761082447	1.05E-07
3h	ACA1_229570/clean/scf7180000084761:280817-280885:-	ACA1_229570	Oxidoreductase, short chain dehydrogenase/reductase superfamily protein [Source:UniProtKB/TrEMBL;Acc:L8H814]	0.02104859	0.779741667	0.758693077	1.70E-10
3h	ACA1_287790/clean/scf7180000084777:79994-80085:-	ACA1_287790	hypothetical protein	0.018393073	0.776514333	0.75812126	3.83E-13
3h	ACA1_323520/clean/scf7180000084468:41224-41298:-	ACA1_323520	Inosineuridine preferring nucleoside hydrolase [Source:UniProtKB/TrEMBL;Acc:L8H334]	0.018730533	0.765696333	0.7469658	6.08E-11
3h	ACA1_024200/clean/scf7180000084441:15696-15765:-	ACA1_024200	WD_REPEATS_REGION domain-containing protein [Source:UniProtKB/TrEMBL;Acc:L8GKS0]	0	0.742075333	0.742075333	5.81E-09
3h	ACA1_108580/clean/scf7180000084661:50794-50882:-	ACA1_108580	hypothetical protein	0.077430467	0.818342333	0.740911867	0.000265338
3h	ACA1_170960/clean/scf7180000084741:21940-22142:+	ACA1_170960	hypothetical protein	0.008513543	0.747162	0.738648457	3.98E-09
3h	ACA1_183210/clean/scf7180000084745:357210-357300:-	ACA1_183210	Thioredoxin domain-containing protein [Source:UniProtKB/TrEMBL;Acc:L8H812]	0.069307	0.802637	0.73333	0.001515248
3h	ACA1_271330/clean/scf7180000084771:21289-21464:+	ACA1_271330	hypothetical protein	0.065841133	0.794945	0.729103867	2.68E-07
3h	ACA1_230520/clean/scf7180000084761:371650-371715:-	ACA1_230520	Hypothetical protein	0.01673672	0.743667667	0.726930947	1.20E-12
3h	ACA1_072500/clean/scf7180000084603:1216853-1216923:-	ACA1_072500	F-box domain-containing protein [Source:UniProtKB/TrEMBL;Acc:L8HFX9]	0.011444933	0.737592667	0.726147733	2.78E-07
3h	ACA1_383660/clean/scf7180000084721:239911-240002:+	ACA1_383660	tRNA (guanine-N(7)-)-methyltransferase non-catalytic subunit [Source:UniProtKB/TrEMBL;Acc:L8GW53]	0	0.725335667	0.725335667	1.07E-10
3h	ACA1_071170/clean/scf7180000084603:880211-880318:+	ACA1_071170	RhoGAP domain containing protein [Source:UniProtKB/TrEMBL;Acc:L8HGC8]	0	0.7253	0.7253	1.11E-10
3h	ACA1_383660/clean/scf7180000084721:240604-240676:+	ACA1_383660	tRNA (guanine-N(7)-)-methyltransferase non-catalytic subunit [Source:UniProtKB/TrEMBL;Acc:L8GW53]	0.032967033	0.757409333	0.7244423	2.46E-10
3h	ACA1_278900/clean/scf7180000084776:271851-271938:+	ACA1_278900	ANK_REP_REGION domain-containing protein [Source:UniProtKB/TrEMBL;Acc:L8H5W6]	0	0.721700333	0.721700333	1.35E-11
3h	ACA1_088960/clean/scf7180000084639:274567-274634:-	ACA1_088960	Beta-lactamase domain-containing protein [Source:UniProtKB/TrEMBL;Acc:L8GU04]	0	0.721333667	0.721333667	2.98E-07
3h	ACA1_092590/clean/scf7180000084645:361504-361580:-	ACA1_092590	Leucine rich repeat domain containing protein [Source:UniProtKB/TrEMBL;Acc:L8GKK8]	0.019607833	0.736105667	0.716497833	6.10E-07
3h	ACA1_383660/clean/scf7180000084721:239758-239835:+	ACA1_383660	tRNA (guanine-N(7)-)-methyltransferase non-catalytic subunit [Source:UniProtKB/TrEMBL;Acc:L8GW53]	0.0452366	0.760820667	0.715584067	4.21E-11
3h	ACA1_287790/clean/scf7180000084777:80215-80303:-	ACA1_287790	hypothetical protein	0.0167442	0.730307333	0.713563133	1.28E-11
3h	ACA1_206620/clean/scf7180000084757:24821-24907:+	ACA1_206620	MCM domain-containing protein [Source:UniProtKB/TrEMBL;Acc:L8GY26]	0.033669067	0.746859667	0.7131906	1.65E-09
3h	ACA1_367300/clean/scf7180000084641:592784-592893:+	ACA1_367300	NTP_transferase domain-containing protein [Source:UniProtKB/TrEMBL;Acc:L8GMA8]	0.0084839	0.720061667	0.711577767	2.86E-09
3h	ACA1_020870/clean/scf7180000084095:29634-29720:+	ACA1_020870	Large conductance mechanosensitive channel protein [Source:UniProtKB/TrEMBL;Acc:L8GWH9]	0.193751333	0.904210333	0.710459	9.02E-11
3h	ACA1_306850/clean/scf7180000083668:20973-21053:+	ACA1_306850	RanBPMrelated, putative [Source:UniProtKB/TrEMBL;Acc:L8GNY2]	0.060814733	0.771244	0.710429267	2.17E-09
3h	ACA1_178580/clean/scf7180000084743:262261-262374:-	ACA1_178580	F-box domain containing protein [Source:UniProtKB/TrEMBL;Acc:L8GSM2]	0.290462333	1	0.709537667	5.02E-06
3h	ACA1_232650/clean/scf7180000084762:55213-55340:-	ACA1_232650	AMP-binding domain-containing protein [Source:UniProtKB/TrEMBL;Acc:L8H2D5]	0.00258793	0.712097667	0.709509737	1.46E-13
3h	ACA1_177720/clean/scf7180000084743:114016-114083:-	ACA1_177720	PARP-type domain-containing protein [Source:UniProtKB/TrEMBL;Acc:L8GU65]	0.001403837	0.708137	0.706733163	8.70E-12
3h	ACA1_379390/clean/scf7180000084711:347066-347179:-	ACA1_379390	hypothetical protein	0.297527	1	0.702473	0.004384276
3h	ACA1_384360/clean/scf7180000084730:32826-32913:-	ACA1_384360	Signal recognition particle, putative [Source:UniProtKB/TrEMBL;Acc:L8HAH5]	0.00430693	0.705813333	0.701506403	3.31E-14
3h	ACA1_103750/clean/scf7180000084653:52237-52308:+	ACA1_103750	Fascin domain-containing protein [Source:UniProtKB/TrEMBL;Acc:L8GD37]	0.027538467	0.725954667	0.6984162	2.50E-08
3h	ACA1_396120/clean/scf7180000084759:23911-24033:-	ACA1_396120	Rasrelated protein Rap-1b, putative [Source:UniProtKB/TrEMBL;Acc:L8HC60]	0.025327067	0.719316667	0.6939896	2.66E-11
3h	ACA1_146440/clean/scf7180000084713:26105-26178:+	ACA1_146440	Methyltransf_11 domain-containing protein [Source:UniProtKB/TrEMBL;Acc:L8GU12]	0.032685767	0.726334	0.693648233	4.20E-12
3h	ACA1_077020/clean/scf7180000084612:215408-215503:+	ACA1_077020	Propionate-CoA synthetase and ligase [Source:UniProtKB/TrEMBL;Acc:L8GLY7]	0.143856333	0.836707667	0.692851333	4.70E-06
3h	ACA1_257320/clean/scf7180000084769:150623-150712:-	ACA1_257320	PNP_phzG_C domain-containing protein [Source:UniProtKB/TrEMBL;Acc:L8GEX9]	0.0742214	0.767039	0.6928176	4.43E-09
3h	ACA1_309630/clean/scf7180000083763:5485-5601:+	ACA1_309630	DUF1768 domain-containing protein [Source:UniProtKB/TrEMBL;Acc:L8GIA7]	0.1099791	0.801708333	0.691729233	4.86E-07
3h	ACA1_260060/clean/scf7180000084769:413592-413689:+	ACA1_260060	WD_REPEATS_REGION domain-containing protein [Source:UniProtKB/TrEMBL;Acc:L8GF95]	0.025688067	0.715783333	0.690095267	1.83E-12
3h	ACA1_265380/clean/scf7180000084770:455003-455067:-	ACA1_265380	Phosphatidylinositol glycan anchor biosynthesis, class S, putative [Source:UniProtKB/TrEMBL;Acc:L8H3M4]	0.00204499	0.691428	0.68938301	4.85E-09
3h	ACA1_296570/clean/scf7180000084777:1386723-1386820:+	ACA1_296570	TRANSMEMBRANE PROTEIN, putative [Source:UniProtKB/TrEMBL;Acc:L8HIX9]	0.035853767	0.725105	0.689251233	3.93E-09
3h	ACA1_027000/clean/scf7180000084464:15453-15542:-	ACA1_027000	SCP family extracellular, putative [Source:UniProtKB/TrEMBL;Acc:L8GHL6]	0.011795859	0.699721	0.687925141	3.83E-12
3h	ACA1_175470/clean/scf7180000084741:966321-966380:-	ACA1_175470	Endo/exonuclease/phosphatase domain-containing protein [Source:UniProtKB/TrEMBL;Acc:L8HI27]	0.0953089	0.782459	0.6871501	4.20E-06
3h	ACA1_048880/clean/scf7180000084583:10024-10100:+	ACA1_048880	LIM zinc-binding domain-containing protein [Source:UniProtKB/TrEMBL;Acc:L8HKD0]	0.068962933	0.754991667	0.686028733	6.47E-09
3h	ACA1_216300/clean/scf7180000084758:461568-461772:+	ACA1_216300	Ras family protein [Source:UniProtKB/TrEMBL;Acc:L8GR60]	0.023338253	0.708580667	0.685242413	1.36E-07
3h	ACA1_071170/clean/scf7180000084603:879314-879414:+	ACA1_071170	RhoGAP domain containing protein [Source:UniProtKB/TrEMBL;Acc:L8HGC8]	0	0.684614667	0.684614667	1.20E-09
3h	ACA1_247950/clean/scf7180000084766:1669-1749:-	ACA1_247950	hypothetical protein	0.038662067	0.723183	0.684520933	1.64E-10
3h	ACA1_224310/clean/scf7180000084760:166379-166473:+	ACA1_224310	PH domain-containing protein [Source:UniProtKB/TrEMBL;Acc:L8GTW7]	0.005546443	0.689316667	0.683770223	2.20E-10
3h	ACA1_088960/clean/scf7180000084639:274764-274836:-	ACA1_088960	Beta-lactamase domain-containing protein [Source:UniProtKB/TrEMBL;Acc:L8GU04]	0.040995867	0.724725333	0.683729467	1.40E-06
3h	ACA1_185700/clean/scf7180000084746:114487-114556:+	ACA1_185700	EF hand domain containing protein [Source:UniProtKB/TrEMBL;Acc:L8H786]	0.0326877	0.714770667	0.682082967	1.35E-10
3h	ACA1_259480/clean/scf7180000084769:349054-349195:-	ACA1_259480	EnoylCoA hydratase/isomerase [Source:UniProtKB/TrEMBL;Acc:L8GFL3]	0.208071667	0.889890667	0.681819	2.77E-08
3h	ACA1_362550/clean/scf7180000084624:92352-92416:+	ACA1_362550	Aldedh domain-containing protein [Source:UniProtKB/TrEMBL;Acc:L8GHV5]	0.000978303	0.682544667	0.681566364	2.36E-20
3h	ACA1_359270/clean/scf7180000084742:70646-70701:-	ACA1_359270	RAB family member (Rab7), putative [Source:UniProtKB/TrEMBL;Acc:L8GLL4]	0.0305593	0.711773	0.6812137	3.11E-09
3h	ACA1_069380/clean/scf7180000084603:424419-424538:+	ACA1_069380	LNS2 domain-containing protein [Source:UniProtKB/TrEMBL;Acc:L8HCT1]	0.044137367	0.724788333	0.680650967	2.00E-09
3h	ACA1_159850/clean/scf7180000084724:393288-393380:+	ACA1_159850	hypothetical protein	0.009056	0.689466667	0.680410667	6.69E-13
3h	ACA1_012040/clean/scf7180000083652:9387-9539:+	ACA1_012040	LIM domain containing protein [Source:UniProtKB/TrEMBL;Acc:L8GI34]	0.026180867	0.703835333	0.677654467	4.90E-13

3h	ACA1_082750/clean/scf7180000084625:9008-9105:+	ACA1_082750	N-acetyltransferase domain-containing protein [Source:UniProtKB/TrEMBL;Acc:L8GSP4]	0.024708487	0.702001	0.677292513	2.21E-10
3h	ACA1_066420/clean/scf7180000084600:1002-1072:+	ACA1_066420	hypothetical protein	0.219658	0.896639667	0.676981667	1.66E-09
3h	ACA1_289710/clean/scf7180000084777:446308-446373:-	ACA1_289710	hypothetical protein	0.01950425	0.696401	0.67689675	2.33E-07
3h	ACA1_193130/clean/scf7180000084749:193111-193220:-	ACA1_193130	Metallophos domain-containing protein [Source:UniProtKB/TrEMBL;Acc:L8GN68]	0.17685	0.851588667	0.674738667	1.60E-09
3h	ACA1_058500/clean/scf7180000084599:334235-334349:-	ACA1_058500	RNA_ligase domain-containing protein [Source:UniProtKB/TrEMBL;Acc:L8GVF6]	0.0255708	0.700085667	0.674514867	0.00065299
3h	ACA1_383580/clean/scf7180000084721:215085-215168:-	ACA1_383580	Sulfate adenylyltransferase [Source:UniProtKB/TrEMBL;Acc:L8GUZ7]	0.1078306	0.782011	0.6741804	2.01E-07
3h	ACA1_329650/clean/scf7180000084562:37527-37589:-	ACA1_329650	hypothetical protein	0.117628667	0.790931	0.673302333	1.88E-09
3h	ACA1_267000/clean/scf7180000084770:772955-773031:+	ACA1_267000	Camp-dependent protein kinase catalytic subunit family protein [Source:UniProtKB/TrEMBL;Acc:L8H3Y8]	0.0076031	0.678715667	0.671112567	2.29E-08
3h	ACA1_394600/clean/scf7180000084756:117156-117236:-	ACA1_394600	GRIP domain-containing protein [Source:UniProtKB/TrEMBL;Acc:L8H1Q7]	0.156019667	0.826588	0.670568333	8.40E-09
3h	ACA1_265380/clean/scf7180000084770:455635-455721:-	ACA1_265380	Phosphatidylinositol glycan anchor biosynthesis, class S, putative [Source:UniProtKB/TrEMBL;Acc:L8H3M4]	0.026112493	0.695809	0.669696507	4.68E-10
3h	ACA1_260060/clean/scf7180000084769:413925-414056:+	ACA1_260060	WD_REPEATS_REGION domain-containing protein [Source:UniProtKB/TrEMBL;Acc:L8GF95]	0.024725667	0.691564	0.666838333	1.19E-10
3h	ACA1_072960/clean/scf7180000084603:1346287-1346398:+	ACA1_072960	F-box domain-containing protein [Source:UniProtKB/TrEMBL;Acc:L8HEK9]	0.190767	0.857216667	0.666449667	3.29E-08
3h	ACA1_012980/clean/scf7180000083742:16382-16462:-	ACA1_012980	Endo/exonuclease/phosphatase domain-containing protein [Source:UniProtKB/TrEMBL;Acc:L8H4W5]	0.035505433	0.70078	0.665274567	1.85E-07
3h	ACA1_358270/clean/scf7180000084742:2611-2696:-	ACA1_358270	Pyr_redox_2 domain-containing protein [Source:UniProtKB/TrEMBL;Acc:L8GL60]	0.014696367	0.679409333	0.664712967	1.12E-07
3h	ACA1_381100/clean/scf7180000084712:144628-144684:+	ACA1_381100	hypothetical protein	0.1071192	0.771645	0.6645258	6.28E-07
3h	ACA1_226190/clean/scf7180000084761:39137-39205:+	ACA1_226190	C2H2-type domain-containing protein [Source:UniProtKB/TrEMBL;Acc:L8H9Z3]	0.0050505	0.668910667	0.663860167	7.63E-09
3h	ACA1_167050/clean/scf7180000084736:63077-63163:-	ACA1_167050	Ras subfamily protein [Source:UniProtKB/TrEMBL;Acc:L8H0S4]	0.1156701	0.779334	0.6636639	2.59E-06
3h	ACA1_385640/clean/scf7180000084730:221743-221864:+	ACA1_385640	hypothetical protein	0.00170856	0.665298	0.66358944	7.96E-10
3h	ACA1_048880/clean/scf7180000084583:9878-9936:+	ACA1_048880	LIM zinc-binding domain-containing protein [Source:UniProtKB/TrEMBL;Acc:L8HKD0]	0.074839467	0.737690667	0.6628512	1.39E-08
3h	ACA1_373900/clean/scf7180000084679:186898-186962:+	ACA1_373900	Methyltransferase domain containing protein [Source:UniProtKB/TrEMBL;Acc:L8GHT5]	0.073099533	0.735615333	0.6625158	0.0011647
3h	ACA1_038160/clean/scf7180000084568:85780-85892:-	ACA1_038160	Protein kinase domain-containing protein [Source:UniProtKB/TrEMBL;Acc:L8GN38]	0.138642	0.800128667	0.661486667	7.15E-05
3h	ACA1_384720/clean/scf7180000084730:92713-92807:+	ACA1_384720	Vault_2 domain-containing protein [Source:UniProtKB/TrEMBL;Acc:L8H933]	0.145942	0.806984333	0.661042333	1.89E-08
3h	ACA1_153200/clean/scf7180000084720:84587-84756:+	ACA1_153200	RBR-type E3 ubiquitin transferase [Source:UniProtKB/TrEMBL;Acc:L8HIB3]	0.241986333	0.901909667	0.659923333	2.36E-05
3h	ACA1_277500/clean/scf7180000084776:65335-65391:-	ACA1_277500	DBR1 domain-containing protein [Source:UniProtKB/TrEMBL;Acc:L8H8N5]	0.114285667	0.774009	0.659723333	0.000533575
3h	ACA1_053310/clean/scf7180000084594:260499-260586:+	ACA1_053310	Serine/threonine kinase [Source:UniProtKB/TrEMBL;Acc:L8H7D1]	0	0.65965	0.65965	1.48E-05
3h	ACA1_389330/clean/scf7180000084733:231518-231619:+	ACA1_389330	Nicotinamide riboside kinase 1 family protein [Source:UniProtKB/TrEMBL;Acc:L8GDW0]	0.003542947	0.662946	0.659403053	1.99E-09
3h	ACA1_278900/clean/scf7180000084776:272601-272705:+	ACA1_278900	ANK_REP_REGION domain-containing protein [Source:UniProtKB/TrEMBL;Acc:L8H5W6]	0.003205127	0.662575	0.659369873	5.12E-10
3h	ACA1_383610/clean/scf7180000084721:228305-228408:+	ACA1_383610	PAKA subfamily protein kinase [Source:UniProtKB/TrEMBL;Acc:L8GW48]	0.022724633	0.681646667	0.658922033	1.16E-10
3h	ACA1_184090/clean/scf7180000084745:556617-556723:-	ACA1_184090	hypothetical protein	0.0435294	0.701248667	0.657719267	4.17E-10
3h	ACA1_296250/clean/scf7180000084777:1306313-1306402:+	ACA1_296250	Rasrelated protein Rab-32, putative [Source:UniProtKB/TrEMBL;Acc:L8HJZ6]	0.01385188	0.67089	0.65703812	5.02E-08
3h	ACA1_101530/clean/scf7180000084649:87651-87736:-	ACA1_101530	6phosphogluconate dehydrogenase NAD-binding domain containing protein [Source:UniProtKB/TrEMBL;Acc:L8GGL6]	0.079324367	0.736313667	0.6569893	3.11E-10
3h	ACA1_290180/clean/scf7180000084777:547112-547176:+	ACA1_290180	hypothetical protein	0.231349667	0.887878667	0.656529	5.89E-05
3h	ACA1_388690/clean/scf7180000084733:148660-148720:+	ACA1_388690	hypothetical protein	0.002898083	0.658919333	0.65602125	8.69E-08
3h	ACA1_325110/clean/scf7180000084475:4281-4355:-	ACA1_325110	3'-5' exonuclease domain-containing protein [Source:UniProtKB/TrEMBL;Acc:L8GRY3]	0.00098912	0.656640667	0.655651547	1.87E-08
3h	ACA1_180600/clean/scf7180000084744:62961-63079:+	ACA1_180600	hypothetical protein	0.014986867	0.66967	0.654683133	8.90E-13
3h	ACA1_136140/clean/scf7180000084702:137628-137729:+	ACA1_136140	Homeobox domain-containing protein [Source:UniProtKB/TrEMBL;Acc:L8GF88]	0.020440133	0.67505	0.654609867	1.02E-11
3h	ACA1_266800/clean/scf7180000084770:716880-716965:+	ACA1_266800	Protein kinase domain containing protein [Source:UniProtKB/TrEMBL;Acc:L8H3W0]	0.0654444	0.719078	0.6536336	8.12E-10
3h	ACA1_220160/clean/scf7180000084758:878165-878344:-	ACA1_220160	Hypothetical protein	0.120741303	0.773930667	0.653189363	1.13E-06
3h	ACA1_329650/clean/scf7180000084562:37997-38061:-	ACA1_329650	hypothetical protein	0.003074529	0.655354333	0.652279804	3.55E-12
3h	ACA1_349640/clean/scf7180000084690:23912-23996:+	ACA1_349640	HEAT repeat domain containing protein [Source:UniProtKB/TrEMBL;Acc:L8GKB3]	0.002715723	0.654674667	0.651958943	2.00E-08
3h	ACA1_203980/clean/scf7180000084754:243272-243361:+	ACA1_203980	hypothetical protein	0.0287966	0.679322	0.6505254	2.29E-11
3h	ACA1_103170/clean/scf7180000084653:11967-12070:+	ACA1_103170	CBS domain containing protein [Source:UniProtKB/TrEMBL;Acc:L8GCP7]	0.0308642	0.681284667	0.650420467	5.99E-06
3h	ACA1_101080/clean/scf7180000084649:35292-35381:-	ACA1_101080	Purple acid phosphatase [Source:UniProtKB/TrEMBL;Acc:L8GGG9]	0.006694133	0.656931667	0.650237533	4.19E-15
3h	ACA1_193180/clean/scf7180000084749:206163-206277:+	ACA1_193180	Serine incorporator 3, putative [Source:UniProtKB/TrEMBL;Acc:L8GN74]	0.000879832	0.650503667	0.649623835	2.30E-11
3h	ACA1_224290/clean/scf7180000084760:164552-164652:-	ACA1_224290	CIA30 domain-containing protein [Source:UniProtKB/TrEMBL;Acc:L8GVE8]	0.027671267	0.676683667	0.6490124	7.81E-12
3h	ACA1_390570/clean/scf7180000084740:74970-75038:-	ACA1_390570	Acetyl/propionyl CoA carboxylase, beta subunit, putative [Source:UniProtKB/TrEMBL;Acc:L8GRH2]	0.115383867	0.764360667	0.6489768	1.48E-08
3h	ACA1_271330/clean/scf7180000084771:21570-21663:+	ACA1_271330	hypothetical protein	0.006214097	0.653455667	0.64724157	4.35E-08
3h	ACA1_084920/clean/scf7180000084632:35230-35297:+	ACA1_084920	Phospholipase, putative [Source:UniProtKB/TrEMBL;Acc:L8HJC3]	0.031046467	0.678153667	0.6471072	6.36E-09
3h	ACA1_243850/clean/scf7180000084763:360846-360971:+	ACA1_243850	Ras-GAP domain-containing protein [Source:UniProtKB/TrEMBL;Acc:L8GJZ5]	0.001181567	0.648159667	0.6469781	1.81E-06
3h	ACA1_360980/clean/scf7180000084622:214115-214203:+	ACA1_360980	hypothetical protein	0.070168967	0.716839333	0.646670367	3.98E-06
3h	ACA1_213080/clean/scf7180000084758:173868-174005:+	ACA1_213080	hypothetical protein	0.049473509	0.695957333	0.646483825	3.07E-07
3h	ACA1_199500/clean/scf7180000084753:247986-248059:+	ACA1_199500	1acylglycerophosphocholine O-acyltransferase 1, putative [Source:UniProtKB/TrEMBL;Acc:L8H4L3]	0	0.646164	0.646164	1.77E-06
3h	ACA1_193130/clean/scf7180000084749:192862-192958:-	ACA1_193130	Metallophos domain-containing protein [Source:UniProtKB/TrEMBL;Acc:L8GN68]	0.0347419	0.680333333	0.645591433	9.62E-09
3h	ACA1_372610/clean/scf7180000084679:25349-25429:-	ACA1_372610	Asp_Arg_Hydrox domain-containing protein [Source:UniProtKB/TrEMBL;Acc:L8GH09]	0.03866302	0.684041667	0.645378647	1.31E-07
3h	ACA1_012300/clean/scf7180000083740:10522-10589:-	ACA1_012300	hypothetical protein	0.012741933	0.657203	0.644461067	4.25E-07
3h	ACA1_111960/clean/scf7180000084669:74591-74682:-	ACA1_111960	Ornithine cyclodeaminase [Source:UniProtKB/TrEMBL;Acc:L8H5A8]	0.01293808	0.656281667	0.643343587	9.46E-13
3h	ACA1_053310/clean/scf7180000084594:257766-257854:+	ACA1_053310	Serine/threonine kinase [Source:UniProtKB/TrEMBL;Acc:L8H7D1]	0	0.64312	0.64312	0.000532829
3h	ACA1_072500/clean/scf7180000084603:1217354-1217428:-	ACA1_072500	F-box domain-containing protein [Source:UniProtKB/TrEMBL;Acc:L8HFX9]	0.014171277	0.657258667	0.64308739	1.29E-05
3h	ACA1_349640/clean/scf7180000084690:22625-22703:+	ACA1_349640	HEAT repeat domain containing protein [Source:UniProtKB/TrEMBL;Acc:L8GKB3]	0.005292743	0.646847333	0.64155459	5.45E-07
3h	ACA1_186870/clean/scf7180000084746:214665-214801:+	ACA1_186870	Protein kinase domain-containing protein [Source:UniProtKB/TrEMBL;Acc:L8H6J9]	0.240884	0.882200333	0.641316333	2.14E-06
3h	ACA1_040210/clean/scf7180000084571:13868-13936:+	ACA1_040210	Mitochondrial uncoupling protein [Source:UniProtKB/TrEMBL;Acc:L8GGI9]	0.01797827	0.658826333	0.640848063	4.00E-11
3h	ACA1_297090/clean/scf7180000084777:1454490-1454573:-	ACA1_297090	Kelch repeatcontaining protein [Source:UniProtKB/TrEMBL;Acc:L8HK57]	0.016223243	0.656043667	0.639820423	0.000115857
3h	ACA1_105970/clean/scf7180000084660:40538-40615:-	ACA1_105970	PhytanoylCoA dioxygenase family protein [Source:UniProtKB/TrEMBL;Acc:L8GQ69]	0.007525673	0.646810667	0.639284993	6.30E-13
3h	ACA1_389110/clean/scf7180000084733:198080-198168:+	ACA1_389110	hypothetical protein	0.008166847	0.647344333	0.639177487	5.13E-13
3h	ACA1_343310/clean/scf7180000084638:23565-23680:-	ACA1_343310	EF-hand domain-containing protein [Source:UniProtKB/TrEMBL;Acc:L8H9D8]	0.004016067	0.643171667	0.6391556	1.91E-08
3h	ACA1_175700/clean/scf7180000084741:1038479-1038550:-	ACA1_175700	Frizzled/Smoothened family membrane region protein [Source:UniProtKB/TrEMBL;Acc:L8HH45]	0.003060477	0.642048333	0.638987857	5.17E-06
3h	ACA1_323430/clean/scf7180000084468:21313-21415:+	ACA1_323430	SnoaL family polyketide cyclase [Source:UniProtKB/TrEMBL;Acc:L8H0W6]	0.003299643	0.642126	0.638826357	2.67E-18
3h	ACA1_044920/clean/scf7180000084576:59578-59775:-	ACA1_044920	hypothetical protein	0.020342667	0.657685333	0.637342667	2.38E-13
3h	ACA1_070000/clean/scf7180000084603:617418-617515:+	ACA1_070000	hypothetical protein	0.010919177	0.648011333	0.637092157	2.06E-11
3h	ACA1_218690/clean/scf7180000084758:691064-691176:+	ACA1_218690	1,2-dihydroxy-3-keto-5-methylthiopentene dioxygenase [Source:UniProtKB/TrEMBL;Acc:L8GPW7]	0.003861343	0.640666667	0.636805323	1.62E-13
3h	ACA1_020120/clean/scf7180000084037:8300-8377:+	ACA1_020120	Peptidase S53 domain-containing protein [Source:UniProtKB/TrEMBL;Acc:L8GL48]	0.168865667	0.805581	0.636715333	6.76E-05
3h	ACA1_237860/clean/scf7180000084763:32994-33094:+	ACA1_237860	Glycerophosphodiester phosphodiesterase family protein [Source:UniProtKB/TrEMBL;Acc:L8GKN2]	0.195412	0.832060333	0.636648333	1.48E-06
3h	ACA1_062280/clean/scf7180000084599:1156724-1156817:-	ACA1_062280	hypothetical protein	0.02410577	0.660384333	0.636278563	5.27E-06
3h	ACA1_054020/clean/scf7180000084594:373447-373553:-	ACA1_054020	hypothetical protein	0.012485077	0.648549	0.636063923	1.23E-08
3h	ACA1_289590/clean/scf7180000084777:427931-428013:+	ACA1_289590	hypothetical protein	0.003404033	0.639401333	0.6359973	1.03E-07
3h	ACA1_383610/clean/scf7180000084721:225990-226087:+	ACA1_383610	PAKA subfamily protein kinase [Source:UniProtKB/TrEMBL;Acc:L8GW48]	0.006642337	0.642481667	0.63583933	3.13E-11
3h	ACA1_323430/clean/scf7180000084468:21892-21996:+	ACA1_323430	SnoaL family polyketide cyclase [Source:UniProtKB/TrEMBL;Acc:L8H0W6]	0.00398097	0.639500333	0.635519363	3.13E-18
3h	ACA1_376590/clean/scf7180000084685:113811-113940:+	ACA1_376590	C2 domain-containing protein [Source:UniProtKB/TrEMBL;Acc:L8HIH7]	0.049822833	0.685159	0.635336167	7.02E-07
3h	ACA1_204850/clean/scf7180000084754:329493-329569:+	ACA1_204850	Beta_helix domain-containing protein [Source:UniProtKB/TrEMBL;Acc:L8GWA3]	0.246890333	0.881118333	0.634228	0.000437889
3h	ACA1_379080/clean/scf7180000084711:275891-275963:+	ACA1_379080	GATA-type domain-containing protein [Source:UniProtKB/TrEMBL;Acc:L8GS28]	0.231911333	0.865929	0.634017667	2.92E-07
3h	ACA1_175760/clean/scf7180000084741:1053649-1053722:-	ACA1_175760	Phosphoglycerate mutase family domain containing protein [Source:UniProtKB/TrEMBL;Acc:L8HH51]	0.1063947	0.739997333	0.633602633	4.81E-07
3h	ACA1_142610/clean/scf7180000084708:199733-199829:-	ACA1_142610	Myblike DNA-binding domain containing protein [Source:UniProtKB/TrEMBL;Acc:L8HCT3]	0.039993767	0.673589333	0.633595567	2.22E-08
3h	ACA1_024240/clean/scf7180000084441:23674-23764:-	ACA1_024240	Yip1 domain containing protein [Source:UniProtKB/TrEMBL;Acc:L8GN09]	0.005446137	0.638437	0.632990863	6.35E-12
3h	ACA1_271720/clean/scf7180000084771:92521-92594:-	ACA1_271720	Ras-GAP domain-containing protein [Source:UniProtKB/TrEMBL;Acc:L8GR76]	0.017647033	0.649522	0.631874967	3.23E-09
3h	ACA1_184330/clean/scf7180000084745:619706-619791:-	ACA1_184330	SAGA-associated factor 11 [Source:UniProtKB/TrEMBL;Acc:L8H8C5]	0.0594656	0.691191667	0.631726067	2.26E-06
3h	ACA1_070500/clean/scf7180000084603:702737-702888:-	ACA1_070500	Ras subfamily protein [Source:UniProtKB/TrEMBL;Acc:L8HDY4]	0.018297157	0.649888667	0.63159151	3.28E-09
3h	ACA1_103150/clean/scf7180000084653:7967-8040:+	ACA1_103150	hypothetical protein	0.0213687	0.652654667	0.631285967	8.60E-09
3h	ACA1_243850/clean/scf7180000084763:362810-362936:+	ACA1_243850	Ras-GAP domain-containing protein [Source:UniProtKB/TrEMBL;Acc:L8GJZ5]	0.007942567	0.638586	0.630643433	3.32E-06
3h	ACA1_290250/clean/scf7180000084777:569198-569268:+	ACA1_290250	hypothetical protein	0.00963191	0.639091	0.62945909	2.89E-07
3h	ACA1_274900/clean/scf7180000084773:135398-135494:-	ACA1_274900	2,3diketo-5-methylthio-1-phosphopentane phosphatase subfamily protein [Source:UniProtKB/TrEMBL;Acc:L8GFW5]	0.0294912	0.658376667	0.628885467	5.47E-09
3h	ACA1_091690/clean/scf7180000084645:181591-181680:+	ACA1_091690	PolyA_pol domain-containing protein [Source:UniProtKB/TrEMBL;Acc:L8GIJ9]	0.188618667	0.817251	0.628632333	0.00014811
3h	ACA1_058280/clean/scf7180000084599:287200-287292:-	ACA1_058280	OGFr_N domain-containing protein [Source:UniProtKB/TrEMBL;Acc:L8GW80]	0.0794871	0.707886667	0.628399567	1.87E-07
3h	ACA1_074140/clean/scf7180000084605:34786-34885:+	ACA1_074140	Transporter, major facilitator subfamily protein [Source:UniProtKB/TrEMBL;Acc:L8HK94]	0.00225549	0.630592333	0.628336843	2.70E-10
3h	ACA1_376660/clean/scf7180000084685:138094-138162:-	ACA1_376660	hypothetical protein	0.048313233	0.676426667	0.628113433	3.22E-10
3h	ACA1_127640/clean/scf7180000084696:40081-40159:+	ACA1_127640	GP-PDE domain-containing protein [Source:UniProtKB/TrEMBL;Acc:L8GEW1]	0.002392147	0.628754333	0.626362187	7.62E-09
3h	ACA1_206620/clean/scf7180000084757:23968-24047:+	ACA1_206620	MCM domain-containing protein [Source:UniProtKB/TrEMBL;Acc:L8GY26]	0	0.625832333	0.625832333	1.16E-08
3h	ACA1_113870/clean/scf7180000084669:392445-392548:+	ACA1_113870	CUB domain containing protein [Source:UniProtKB/TrEMBL;Acc:L8H6F7]	0.018531833	0.643354	0.624822167	1.70E-08
3h	ACA1_051550/clean/scf7180000084592:37118-37210:-	ACA1_051550	Phenylalanine hydroxylase [Source:UniProtKB/TrEMBL;Acc:L8GPS8]	0.001795737	0.626414667	0.62461893	5.94E-14
3h	ACA1_182400/clean/scf7180000084745:205613-205733:+	ACA1_182400	Integral membrane protein [Source:UniProtKB/TrEMBL;Acc:L8H9C3]	0.0423709	0.666943	0.6245721	8.07E-10
3h	ACA1_144440/clean/scf7180000084709:244650-244859:+	ACA1_144440	SNF2 family Nterminal domain containing protein [Source:UniProtKB/TrEMBL;Acc:L8HFN1]	0.0183279	0.642178	0.6238501	2.77E-10
3h	ACA1_172910/clean/scf7180000084741:354932-355040:-	ACA1_172910	Chloride channel protein [Source:UniProtKB/TrEMBL;Acc:L8HGK6]	0.028753133	0.652552667	0.623799533	1.79E-07
3h	ACA1_093770/clean/scf7180000084645:642070-642184:+	ACA1_093770	hypothetical protein	0.224224667	0.847667667	0.623443	5.15E-05
3h	ACA1_323370/clean/scf7180000084468:12780-12836:-	ACA1_323370	SHSP domain-containing protein [Source:UniProtKB/TrEMBL;Acc:L8H320]	0.0138889	0.635902667	0.622013767	3.48E-05
3h	ACA1_364720/clean/scf7180000084641:184376-184457:-	ACA1_364720	Sas10/Utp3/C1D family protein [Source:UniProtKB/TrEMBL;Acc:L8GM85]	0.004843993	0.626406667	0.621562673	7.16E-09
3h	ACA1_131580/clean/scf7180000084699:90988-91066:-	ACA1_131580	Glycerol-3-phosphate dehydrogenase [NAD(+)] [Source:UniProtKB/TrEMBL;Acc:L8GM84]	0.0153355	0.636778333	0.621442833	3.72E-11
3h	ACA1_016010/clean/scf7180000083800:13409-13521:-	ACA1_016010	hypothetical protein	0.01653529	0.637146333	0.620611043	6.97E-07
3h	ACA1_360100/clean/scf7180000084622:49229-49325:+	ACA1_360100	hypothetical protein	0.0373449	0.657749333	0.620404433	1.70E-06
3h	ACA1_028670/clean/scf7180000084473:5229-5378:+	ACA1_028670	Serine/threonine-protein phosphatase [Source:UniProtKB/TrEMBL;Acc:L8GWF5]	0.000427332	0.620121333	0.619694002	7.38E-14
3h	ACA1_108830/clean/scf7180000084661:93343-93434:+	ACA1_108830	Terpene cyclase/mutase family member [Source:UniProtKB/TrEMBL;Acc:L8GD76]	0.011499333	0.630653667	0.619154333	1.89E-11
3h	ACA1_097960/clean/scf7180000084645:1363284-1363366:-	ACA1_097960	Ribosomal protein L33, putative [Source:UniProtKB/TrEMBL;Acc:L8GJX1]	0.013552697	0.632437667	0.61888497	4.75E-12
3h	ACA1_383700/clean/scf7180000084721:249139-249217:+	ACA1_383700	Endo/exonuclease/phosphatase domain-containing protein [Source:UniProtKB/TrEMBL;Acc:L8GV68]	0.016980653	0.635394667	0.618414013	5.83E-10
3h	ACA1_295880/clean/scf7180000084777:1250622-1250693:+	ACA1_295880	Malic enzyme [Source:UniProtKB/TrEMBL;Acc:L8HM80]	0.004959357	0.623371	0.618411643	8.20E-08
3h	ACA1_341080/clean/scf7180000084620:46511-46581:+	ACA1_341080	RING-type domain-containing protein [Source:UniProtKB/TrEMBL;Acc:L8GS99]	0.0759393	0.694263333	0.618324033	3.22E-06
3h	ACA1_193130/clean/scf7180000084749:192717-192794:-	ACA1_193130	Metallophos domain-containing protein [Source:UniProtKB/TrEMBL;Acc:L8GN68]	0.185421667	0.803669333	0.618247667	9.84E-08
3h	ACA1_358270/clean/scf7180000084742:2246-2350:-	ACA1_358270	Pyr_redox_2 domain-containing protein [Source:UniProtKB/TrEMBL;Acc:L8GL60]	0.0051034	0.62301	0.6179066	3.55E-07
3h	ACA1_116720/clean/scf7180000084669:833657-833739:+	ACA1_116720	J domain-containing protein [Source:UniProtKB/TrEMBL;Acc:L8H4V8]	0.01025785	0.628083667	0.617825817	5.68E-11
3h	ACA1_282440/clean/scf7180000084776:680878-680960:+	ACA1_282440	hypothetical protein	0.006424837	0.624176333	0.617751497	2.38E-07
3h	ACA1_175420/clean/scf7180000084741:951239-951332:+	ACA1_175420	hypothetical protein	0.056624633	0.674055	0.617430367	0.000113611
3h	ACA1_370300/clean/scf7180000084664:490213-490274:-	ACA1_370300	hypothetical protein	0.011869633	0.628875667	0.617006033	2.76E-10
3h	ACA1_264550/clean/scf7180000084770:319403-319497:+	ACA1_264550	Cys/Met metabolism PLPdependent enzyme superfamily protein [Source:UniProtKB/TrEMBL;Acc:L8H496]	0.036024267	0.652786333	0.616762067	1.52E-09
3h	ACA1_322230/clean/scf7180000084459:36794-36854:-	ACA1_322230	UvrD/REP helicase subfamily protein [Source:UniProtKB/TrEMBL;Acc:L8GM54]	0.299919	0.915611667	0.615692667	0.000191929
3h	ACA1_389330/clean/scf7180000084733:231682-231815:+	ACA1_389330	Nicotinamide riboside kinase 1 family protein [Source:UniProtKB/TrEMBL;Acc:L8GDW0]	0.001375963	0.616769333	0.61539337	3.96E-10
3h	ACA1_343290/clean/scf7180000084638:15395-15453:+	ACA1_343290	hypothetical protein	0.121042033	0.736198667	0.615156633	2.52E-07
3h	ACA1_278900/clean/scf7180000084776:272854-272964:+	ACA1_278900	ANK_REP_REGION domain-containing protein [Source:UniProtKB/TrEMBL;Acc:L8H5W6]	0.011286	0.625609333	0.614323333	2.62E-11
3h	ACA1_216320/clean/scf7180000084758:467384-467533:+	ACA1_216320	Homogentisate 1,2dioxygenase [Source:UniProtKB/TrEMBL;Acc:L8GQ83]	0.00465805	0.618835667	0.614177617	1.96E-12
3h	ACA1_063950/clean/scf7180000084599:1542302-1542376:+	ACA1_063950	Fbox domain containing protein [Source:UniProtKB/TrEMBL;Acc:L8GZW1]	0.007278033	0.620736333	0.6134583	1.32E-06
3h	ACA1_072500/clean/scf7180000084603:1217655-1217737:-	ACA1_072500	F-box domain-containing protein [Source:UniProtKB/TrEMBL;Acc:L8HFX9]	0.158691233	0.771719667	0.613028433	9.43E-06
3h	ACA1_129350/clean/scf7180000084698:8929-9053:+	ACA1_129350	Fatty acid hydroxylase domain-containing protein [Source:UniProtKB/TrEMBL;Acc:L8GPA5]	0.014088247	0.627082333	0.612994087	4.87E-11
3h	ACA1_073410/clean/scf7180000084603:1412452-1412531:-	ACA1_073410	UBX domain-containing protein [Source:UniProtKB/TrEMBL;Acc:L8HEN7]	0.0058846	0.618651	0.6127664	1.35E-07
3h	ACA1_059480/clean/scf7180000084599:453454-453547:+	ACA1_059480	hypothetical protein	0	0.612738333	0.612738333	3.66E-06
3h	ACA1_178310/clean/scf7180000084743:209503-209626:-	ACA1_178310	Ubiquitin-like domain-containing protein [Source:UniProtKB/TrEMBL;Acc:L8GTE8]	0.278299	0.890933667	0.612634667	0.000126955
3h	ACA1_282390/clean/scf7180000084776:667442-667517:-	ACA1_282390	SNF2 family Nterminal domain containing protein [Source:UniProtKB/TrEMBL;Acc:L8H6E7]	0.047001467	0.659582	0.612580533	5.43E-08
3h	ACA1_035640/clean/scf7180000084564:103091-103162:-	ACA1_035640	Beta-lactamase domain-containing protein [Source:UniProtKB/TrEMBL;Acc:L8H9P2]	0	0.612482	0.612482	0.015474762
3h	ACA1_064490/clean/scf7180000084599:1628218-1628296:+	ACA1_064490	TYR_PHOSPHATASE_2 domain-containing protein [Source:UniProtKB/TrEMBL;Acc:L8GXK2]	0.0509532	0.662785	0.6118318	6.29E-05
3h	ACA1_282700/clean/scf7180000084776:719212-719286:+	ACA1_282700	EF hand domain containing protein [Source:UniProtKB/TrEMBL;Acc:L8H6I5]	0.031111167	0.642793	0.611681833	8.50E-10
3h	ACA1_106710/clean/scf7180000084660:106517-106629:+	ACA1_106710	TPR_REGION domain-containing protein [Source:UniProtKB/TrEMBL;Acc:L8GMK9]	0.0322504	0.643732	0.6114816	9.33E-11
3h	ACA1_036890/clean/scf7180000084567:6275-6340:+	ACA1_036890	Ras subfamily protein [Source:UniProtKB/TrEMBL;Acc:L8H0T2]	0.061687233	0.673140333	0.6114531	3.87E-09
3h	ACA1_382920/clean/scf7180000084721:118628-118727:-	ACA1_382920	Ras subfamily protein [Source:UniProtKB/TrEMBL;Acc:L8GV31]	0.0503299	0.661642333	0.611312433	5.18E-06

3h	ACA1_266230/clean/scf7180000084770:638288-638370:+	ACA1_266230	Dilute domain-containing protein [Source:UniProtKB/TrEMBL;Acc:L8H2D0]	0.016666667	0.627901333	0.611234667	8.13E-05
3h	ACA1_069190/clean/scf7180000084603:378149-378217:+	ACA1_069190	PfkB domain-containing protein [Source:UniProtKB/TrEMBL;Acc:L8HDR4]	0.011035767	0.621737333	0.610701567	7.91E-09
3h	ACA1_224860/clean/scf7180000084760:258809-258888:+	ACA1_224860	Myosin head (Motor domain) domain containing protein [Source:UniProtKB/TrEMBL;Acc:L8GSY9]	0.057333333	0.667832	0.610498667	0.000395587
3h	ACA1_002780/clean/scf7180000082937:9977-10056:-	ACA1_002780	RRM domain-containing protein [Source:UniProtKB/TrEMBL;Acc:L8GGZ7]	0.0673159	0.677725	0.6104091	1.07E-06
3h	ACA1_144930/clean/scf7180000084710:41854-41980:-	ACA1_144930	N-acetyltransferase domain-containing protein [Source:UniProtKB/TrEMBL;Acc:L8GCT6]	0.0962379	0.706542	0.6103041	8.81E-08
3h	ACA1_349640/clean/scf7180000084690:21715-21810:+	ACA1_349640	HEAT repeat domain containing protein [Source:UniProtKB/TrEMBL;Acc:L8GKB3]	0	0.610178	0.610178	1.31E-08
3h	ACA1_245500/clean/scf7180000084763:496838-496903:+	ACA1_245500	Fe2OG dioxygenase domain-containing protein [Source:UniProtKB/TrEMBL;Acc:L8GL61]	0.00833403	0.618264	0.60992997	1.96E-08
3h	ACA1_230310/clean/scf7180000084761:342605-342687:+	ACA1_230310	PAS domain-containing protein [Source:UniProtKB/TrEMBL;Acc:L8HBA3]	0.032704933	0.642398667	0.609693733	5.28E-08
3h	ACA1_174180/clean/scf7180000084741:633511-633628:+	ACA1_174180	hypothetical protein	0.018730333	0.628415667	0.609685333	3.67E-09
3h	ACA1_243160/clean/scf7180000084763:324384-324442:+	ACA1_243160	Glyco_trans_2-like domain-containing protein [Source:UniProtKB/TrEMBL;Acc:L8GMD7]	0.21332	0.822009333	0.608689333	1.18E-07
3h	ACA1_193230/clean/scf7180000084749:212733-212797:+	ACA1_193230	Macro domain-containing protein [Source:UniProtKB/TrEMBL;Acc:L8GN79]	0.008014745	0.616695	0.608680255	3.26E-09
3h	ACA1_183740/clean/scf7180000084745:493531-493615:+	ACA1_183740	hypothetical protein	0.04365964	0.652117333	0.608457693	2.70E-06
3h	ACA1_376270/clean/scf7180000084685:67757-67830:+	ACA1_376270	Phospho-2-dehydro-3-deoxyheptonate aldolase [Source:UniProtKB/TrEMBL;Acc:L8HFZ7]	0.00275482	0.610843333	0.608088513	1.86E-08
3h	ACA1_107270/clean/scf7180000084660:166714-166793:-	ACA1_107270	AAA domain-containing protein [Source:UniProtKB/TrEMBL;Acc:L8GQA9]	0.029532367	0.637397	0.607864633	2.39E-08
3h	ACA1_006200/clean/scf7180000083029:69118-69193:-	ACA1_006200	hypothetical protein	0.032990913	0.637267333	0.60427642	2.67E-06
3h	ACA1_199500/clean/scf7180000084753:247190-247293:+	ACA1_199500	1acylglycerophosphocholine O-acyltransferase 1, putative [Source:UniProtKB/TrEMBL;Acc:L8H4L3]	0	0.603772333	0.603772333	0.000105007
3h	ACA1_287570/clean/scf7180000084777:38724-38821:+	ACA1_287570	hypothetical protein	0.0335948	0.637123667	0.603528867	1.96E-10
3h	ACA1_074980/clean/scf7180000084608:123191-123290:+	ACA1_074980	Rab-GAP TBC domain-containing protein [Source:UniProtKB/TrEMBL;Acc:L8HFJ1]	0.116424433	0.719322	0.602897567	3.92E-07
3h	ACA1_295560/clean/scf7180000084777:1172523-1172637:-	ACA1_295560	hypothetical protein	0.0296735	0.632468	0.6027945	4.78E-11
3h	ACA1_295300/clean/scf7180000084777:1101617-1101773:-	ACA1_295300	Calponin domain containing protein [Source:UniProtKB/TrEMBL;Acc:L8HJQ6]	0.031837967	0.634457333	0.602619367	2.77E-08
3h	ACA1_273110/clean/scf7180000084773:16644-16718:+	ACA1_273110	hypothetical protein	0.170943333	0.773089333	0.602146	0.001458683
3h	ACA1_070970/clean/scf7180000084603:820037-820286:-	ACA1_070970	BTB domain-containing protein [Source:UniProtKB/TrEMBL;Acc:L8HGA8]	0.2086	0.810109	0.601509	0.000463105
3h	ACA1_129340/clean/scf7180000084698:6133-6209:+	ACA1_129340	Methyltransf_11 domain-containing protein [Source:UniProtKB/TrEMBL;Acc:L8GNW8]	0.000258799	0.601463333	0.601204534	3.53E-08
3h	ACA1_035640/clean/scf7180000084564:102338-102416:-	ACA1_035640	Beta-lactamase domain-containing protein [Source:UniProtKB/TrEMBL;Acc:L8H9P2]	0	0.601057333	0.601057333	0.002534498
3h	ACA1_360980/clean/scf7180000084622:214564-214645:+	ACA1_360980	hypothetical protein	0.015584823	0.615645333	0.60006051	2.33E-06
3h	ACA1_300860/clean/scf7180000083147:9476-9599:+	ACA1_300860	TFIIB-type domain-containing protein [Source:UniProtKB/TrEMBL;Acc:L8HHA5]	0.291169	0.890903667	0.599734667	3.16E-06
3h	ACA1_174780/clean/scf7180000084741:795639-795802:-	ACA1_174780	VanY domain-containing protein [Source:UniProtKB/TrEMBL;Acc:L8HK80]	0.069354467	0.668722333	0.599367867	1.58E-05
3h	ACA1_272600/clean/scf7180000084772:75686-75789:-	ACA1_272600	SAM domain (Sterile alpha motif) domain containing protein [Source:UniProtKB/TrEMBL;Acc:L8HG53]	0.345730333	0.944424333	0.598694	1.65E-08
3h	ACA1_183740/clean/scf7180000084745:494022-494084:+	ACA1_183740	hypothetical protein	0.019666033	0.618312333	0.5986463	3.71E-07
3h	ACA1_113550/clean/scf7180000084669:315629-315811:-	ACA1_113550	hypothetical protein	0.1341753	0.732743333	0.598568033	1.60E-06
3h	ACA1_287570/clean/scf7180000084777:38466-38543:+	ACA1_287570	hypothetical protein	0.0353619	0.633503667	0.598141767	4.92E-08
3h	ACA1_095060/clean/scf7180000084645:804428-804665:-	ACA1_095060	Sidoreflexin [Source:UniProtKB/TrEMBL;Acc:L8GL14]	0.00815857	0.605944333	0.597785763	5.79E-08
3h	ACA1_265380/clean/scf7180000084770:454661-454742:-	ACA1_265380	Phosphatidylinositol glycan anchor biosynthesis, class S, putative [Source:UniProtKB/TrEMBL;Acc:L8H3M4]	0.010372433	0.608108333	0.5977359	2.23E-08
3h	ACA1_384710/clean/scf7180000084730:90624-90741:+	ACA1_384710	Transmembrane receptor family protein [Source:UniProtKB/TrEMBL;Acc:L8HBG4]	0.024227633	0.621836667	0.597609033	3.99E-08
3h	ACA1_278900/clean/scf7180000084776:272443-272532:+	ACA1_278900	ANK_REP_REGION domain-containing protein [Source:UniProtKB/TrEMBL;Acc:L8H5W6]	0.00510039	0.602695333	0.597594943	2.43E-09
3h	ACA1_320790/clean/scf7180000084416:23487-23555:-	ACA1_320790	RUN domain-containing protein [Source:UniProtKB/TrEMBL;Acc:L8GTR9]	0.01809101	0.615614	0.59752299	7.08E-10
3h	ACA1_384800/clean/scf7180000084730:107650-107733:+	ACA1_384800	hypothetical protein	0.033995823	0.630936	0.596940177	8.20E-08
3h	ACA1_125820/clean/scf7180000084691:80438-80512:+	ACA1_125820	hypothetical protein	0.083396667	0.680322	0.596925333	1.17E-05
3h	ACA1_309340/clean/scf7180000083758:6222-6285:+	ACA1_309340	Protein tyrosine phosphataselike protein, PTPLA [Source:UniProtKB/TrEMBL;Acc:L8GR24]	0.029289577	0.625932333	0.596642757	3.03E-10
3h	ACA1_157810/clean/scf7180000084724:122628-122729:-	ACA1_157810	M20_dimer domain-containing protein [Source:UniProtKB/TrEMBL;Acc:L8H958]	0	0.596637667	0.596637667	2.75E-10
3h	ACA1_071130/clean/scf7180000084603:867717-867865:-	ACA1_071130	hypothetical protein	0.225540667	0.822052	0.596511333	1.24E-05
3h	ACA1_360290/clean/scf7180000084622:83456-83534:+	ACA1_360290	Singlestrand binding protein family [Source:UniProtKB/TrEMBL;Acc:L8HBV6]	0.002981234	0.598766333	0.5957851	5.80E-11
3h	ACA1_355980/clean/scf7180000084728:39846-39939:+	ACA1_355980	Aa_trans domain-containing protein [Source:UniProtKB/TrEMBL;Acc:L8H7L7]	0.005162903	0.600768	0.595605097	2.18E-08
3h	ACA1_088960/clean/scf7180000084639:275276-275368:-	ACA1_088960	Beta-lactamase domain-containing protein [Source:UniProtKB/TrEMBL;Acc:L8GU04]	0.006229333	0.601349	0.595119667	3.19E-06
3h	ACA1_307820/clean/scf7180000083687:20646-20742:-	ACA1_307820	BSD domain-containing protein [Source:UniProtKB/TrEMBL;Acc:L8HBM7]	0.017094167	0.612181667	0.5950875	2.72E-10
3h	ACA1_069120/clean/scf7180000084603:361014-361103:-	ACA1_069120	hypothetical protein	0	0.594943667	0.594943667	1.57E-07
3h	ACA1_296290/clean/scf7180000084777:1314854-1314932:+	ACA1_296290	Ras-GAP domain-containing protein [Source:UniProtKB/TrEMBL;Acc:L8HMB5]	0	0.594865333	0.594865333	7.23E-08
3h	ACA1_116460/clean/scf7180000084669:759601-759666:-	ACA1_116460	Queuosine salvage protein [Source:UniProtKB/TrEMBL;Acc:L8H4T6]	0.052390533	0.647132333	0.5947418	1.37E-08
3h	ACA1_197880/clean/scf7180000084753:46052-46119:-	ACA1_197880	NTP_transf_2 domain-containing protein [Source:UniProtKB/TrEMBL;Acc:L8H4G5]	0.1948662	0.789560667	0.594694467	5.99E-05
3h	ACA1_175300/clean/scf7180000084741:918255-918344:+	ACA1_175300	Zinc knuckle domain containing protein [Source:UniProtKB/TrEMBL;Acc:L8HKB8]	0.0318346	0.626416333	0.594581733	1.63E-05
3h	ACA1_192670/clean/scf7180000084749:153312-153432:+	ACA1_192670	F-box domain-containing protein [Source:UniProtKB/TrEMBL;Acc:L8GNQ3]	0.322901333	0.917137333	0.594236	0.004925184
3h	ACA1_218620/clean/scf7180000084758:679392-679454:-	ACA1_218620	Carboxylic ester hydrolase [Source:UniProtKB/TrEMBL;Acc:L8GRD3]	0.002946451	0.596984	0.594037549	6.66E-09
3h	ACA1_069640/clean/scf7180000084603:498628-498734:-	ACA1_069640	Thioesterase family domain containing protein [Source:UniProtKB/TrEMBL;Acc:L8HCV4]	0	0.593225667	0.593225667	3.88E-06
3h	ACA1_156480/clean/scf7180000084723:44699-44791:+	ACA1_156480	PHB domain-containing protein [Source:UniProtKB/TrEMBL;Acc:L8GK25]	0.00437857	0.597501	0.59312243	7.07E-14
3h	ACA1_263870/clean/scf7180000084770:123783-123880:-	ACA1_263870	MAGE domain-containing protein [Source:UniProtKB/TrEMBL;Acc:L8H1Y1]	0.077030867	0.669612667	0.5925818	0.000100795
3h	ACA1_206620/clean/scf7180000084757:24950-25053:+	ACA1_206620	MCM domain-containing protein [Source:UniProtKB/TrEMBL;Acc:L8GY26]	0.0289706	0.621110667	0.592140067	6.21E-07
3h	ACA1_149530/clean/scf7180000084715:176214-176308:-	ACA1_149530	Elongation factor Tu GTP binding domain containing protein [Source:UniProtKB/TrEMBL;Acc:L8HC62]	0.024248733	0.615855667	0.591606933	7.05E-07
3h	ACA1_237860/clean/scf7180000084763:33222-33358:+	ACA1_237860	Glycerophosphodiester phosphodiesterase family protein [Source:UniProtKB/TrEMBL;Acc:L8GKN2]	0.106712733	0.698082667	0.591369933	8.05E-05
3h	ACA1_323490/clean/scf7180000084468:36325-36414:+	ACA1_323490	DH domain-containing protein [Source:UniProtKB/TrEMBL;Acc:L8H1L1]	0.0796115	0.670788333	0.591176833	6.91E-07
3h	ACA1_091560/clean/scf7180000084645:149699-149763:+	ACA1_091560	UBP-type domain-containing protein [Source:UniProtKB/TrEMBL;Acc:L8GKE3]	0.0852092	0.676361667	0.591152467	2.21E-05
3h	ACA1_261470/clean/scf7180000084769:688960-689043:+	ACA1_261470	hypothetical protein	0.141712633	0.732467333	0.5907547	1.47E-05
3h	ACA1_378130/clean/scf7180000084711:193436-193554:-	ACA1_378130	hypothetical protein	0.003973767	0.594588333	0.590614567	1.86E-08
3h	ACA1_174080/clean/scf7180000084741:602739-602887:-	ACA1_174080	t-SNARE coiled-coil homology domain-containing protein [Source:UniProtKB/TrEMBL;Acc:L8HGR7]	0.230182	0.819980333	0.589798333	7.85E-05
3h	ACA1_046600/clean/scf7180000084577:150912-151000:+	ACA1_046600	FSH1 domain-containing protein [Source:UniProtKB/TrEMBL;Acc:L8H9S5]	0.372210667	0.961756667	0.589546	2.03E-05
3h	ACA1_110510/clean/scf7180000084663:142565-142636:+	ACA1_110510	HEAT repeat domain containing protein [Source:UniProtKB/TrEMBL;Acc:L8HIL2]	0.002593117	0.592086333	0.589493217	1.72E-07
3h	ACA1_031400/clean/scf7180000084506:20052-20122:-	ACA1_031400	hypothetical protein	0.068376033	0.657207667	0.588831633	0.000128224
3h	ACA1_098910/clean/scf7180000084646:61019-61121:-	ACA1_098910	OGFr_N domain-containing protein [Source:UniProtKB/TrEMBL;Acc:L8H4R8]	0.016218697	0.605017333	0.588798637	2.21E-08
3h	ACA1_071430/clean/scf7180000084603:952109-952216:+	ACA1_071430	EngB-type G domain-containing protein [Source:UniProtKB/TrEMBL;Acc:L8HGF2]	0.042541403	0.63092	0.588378597	2.22E-06
3h	ACA1_333230/clean/scf7180000084581:23705-23764:-	ACA1_333230	ANK_REP_REGION domain-containing protein [Source:UniProtKB/TrEMBL;Acc:L8HIB9]	0.339263667	0.927524667	0.588261	4.21E-08
3h	ACA1_106600/clean/scf7180000084660:103308-103427:-	ACA1_106600	Protein kinase domain-containing protein [Source:UniProtKB/TrEMBL;Acc:L8GN97]	0.161805333	0.75	0.588194667	0.024070101
3h	ACA1_332590/clean/scf7180000084579:42865-42943:-	ACA1_332590	GH16 domain-containing protein [Source:UniProtKB/TrEMBL;Acc:L8GKV3]	0.006191883	0.594234333	0.58804245	4.64E-15
3h	ACA1_175300/clean/scf7180000084741:916822-916909:+	ACA1_175300	Zinc knuckle domain containing protein [Source:UniProtKB/TrEMBL;Acc:L8HKB8]	0	0.587843333	0.587843333	1.37E-06
3h	ACA1_295300/clean/scf7180000084777:1097893-1097990:-	ACA1_295300	Calponin domain containing protein [Source:UniProtKB/TrEMBL;Acc:L8HJQ6]	0.021963457	0.609577333	0.587613877	1.26E-07
3h	ACA1_215970/clean/scf7180000084758:418277-418362:-	ACA1_215970	EGFlike domain containing protein [Source:UniProtKB/TrEMBL;Acc:L8GQ73]	0	0.587538	0.587538	9.63E-07
3h	ACA1_018780/clean/scf7180000083912:15861-15963:-	ACA1_018780	AAA domain-containing protein [Source:UniProtKB/TrEMBL;Acc:L8HMV6]	0.011580013	0.598953333	0.58737332	1.50E-09
3h	ACA1_098900/clean/scf7180000084646:58872-58937:-	ACA1_098900	Putative transcriptional regulator cudA [Source:UniProtKB/TrEMBL;Acc:L8H713]	0.147999	0.734053	0.586054	1.87E-05
3h	ACA1_275720/clean/scf7180000084774:92727-92792:+	ACA1_275720	Enolase, Cterminal TIM barrel domain containing protein [Source:UniProtKB/TrEMBL;Acc:L8GRZ9]	0.003259073	0.589163	0.585903927	4.55E-15
3h	ACA1_252210/clean/scf7180000084768:146713-146801:+	ACA1_252210	Protein kinase domain-containing protein [Source:UniProtKB/TrEMBL;Acc:L8HAM0]	0.0200878	0.605615	0.5855272	1.87E-07
3h	ACA1_265940/clean/scf7180000084770:551456-551543:+	ACA1_265940	hypothetical protein	0.024962133	0.610422667	0.585460533	7.51E-11
3h	ACA1_171370/clean/scf7180000084741:88541-88616:+	ACA1_171370	hypothetical protein	0.050571367	0.636021667	0.5854503	8.40E-09
3h	ACA1_014620/clean/scf7180000083772:1382-1486:-	ACA1_014620	hypothetical protein	0.014067983	0.599499333	0.58543135	2.75E-10
3h	ACA1_170960/clean/scf7180000084741:23403-23513:+	ACA1_170960	hypothetical protein	0.008658	0.593915667	0.585257667	4.69E-08
3h	ACA1_158780/clean/scf7180000084724:254613-254702:-	ACA1_158780	Lipase_3 domain-containing protein [Source:UniProtKB/TrEMBL;Acc:L8HBK1]	0	0.584707	0.584707	3.60E-08
3h	ACA1_385720/clean/scf7180000084730:250366-250427:+	ACA1_385720	Protein kinase domain containing protein [Source:UniProtKB/TrEMBL;Acc:L8H9D4]	0.0315585	0.616172	0.5846135	1.15E-08
3h	ACA1_062330/clean/scf7180000084599:1166502-1166593:+	ACA1_062330	U-box domain-containing protein [Source:UniProtKB/TrEMBL;Acc:L8GZF7]	0.008684033	0.592992	0.584307967	0.000844309
3h	ACA1_163490/clean/scf7180000084729:48425-48479:-	ACA1_163490	hypothetical protein	0.022598033	0.606617667	0.584019633	3.32E-07
3h	ACA1_193130/clean/scf7180000084749:192351-192441:-	ACA1_193130	Metallophos domain-containing protein [Source:UniProtKB/TrEMBL;Acc:L8GN68]	0.1082504	0.691540667	0.583290267	2.97E-07
3h	ACA1_062330/clean/scf7180000084599:1165955-1166020:+	ACA1_062330	U-box domain-containing protein [Source:UniProtKB/TrEMBL;Acc:L8GZF7]	0.187922667	0.771143667	0.583221	0.001567412
3h	ACA1_158030/clean/scf7180000084724:186383-186453:-	ACA1_158030	Doublecortin domain-containing protein [Source:UniProtKB/TrEMBL;Acc:L8H983]	0.01312462	0.596164	0.58303938	3.36E-11
3h	ACA1_039340/clean/scf7180000084569:29189-29258:-	ACA1_039340	hypothetical protein	0.021887294	0.604550667	0.582663372	5.93E-06
3h	ACA1_255900/clean/scf7180000084768:579178-579284:+	ACA1_255900	DEP domain-containing protein [Source:UniProtKB/TrEMBL;Acc:L8HA90]	0.039398933	0.621900667	0.582501733	2.85E-12
3h	ACA1_133170/clean/scf7180000084701:14570-14642:+	ACA1_133170	Threonine synthase [Source:UniProtKB/TrEMBL;Acc:L8H3M8]	0.006835143	0.589248333	0.58241319	4.58E-09
3h	ACA1_363780/clean/scf7180000084641:13637-13723:+	ACA1_363780	Vtype ATPase, D subunit [Source:UniProtKB/TrEMBL;Acc:L8GM99]	0.004372617	0.586518667	0.58214605	1.33E-15
3h	ACA1_031910/clean/scf7180000084523:35061-35162:-	ACA1_031910	hypothetical protein	0.082921033	0.665042667	0.582121633	2.66E-07
3h	ACA1_279570/clean/scf7180000084776:417259-417335:+	ACA1_279570	Elp3 domain-containing protein [Source:UniProtKB/TrEMBL;Acc:L8H759]	0.081517767	0.663314667	0.5817969	9.09E-05
3h	ACA1_174820/clean/scf7180000084741:805494-805556:+	ACA1_174820	hypothetical protein	0.030199567	0.611705333	0.581505767	2.66E-09
3h	ACA1_379630/clean/scf7180000084712:11567-11662:-	ACA1_379630	Fbox domain containing protein [Source:UniProtKB/TrEMBL;Acc:L8GMS8]	0.014861967	0.595922333	0.581060367	4.84E-08
3h	ACA1_289660/clean/scf7180000084777:439305-439384:+	ACA1_289660	Churchill domain containing 1, putative [Source:UniProtKB/TrEMBL;Acc:L8HKL9]	0.141101433	0.722144333	0.5810429	1.52E-05
3h	ACA1_389460/clean/scf7180000084733:260868-260949:-	ACA1_389460	hypothetical protein	0.0165389	0.597189667	0.580650767	1.72E-07
3h	ACA1_154550/clean/scf7180000084722:64577-64674:+	ACA1_154550	unspecified product	0.000487741	0.581119667	0.580631926	1.89E-11
3h	ACA1_189830/clean/scf7180000084748:97740-97796:+	ACA1_189830	Partner of sld five, psf2 protein [Source:UniProtKB/TrEMBL;Acc:L8GE47]	0.028381667	0.608968	0.580586333	9.65E-05
3h	ACA1_274930/clean/scf7180000084773:142362-142465:-	ACA1_274930	Histone deacetylaserelated, putative [Source:UniProtKB/TrEMBL;Acc:L8GFN9]	0.130111867	0.710687	0.580575133	6.34E-07
3h	ACA1_053310/clean/scf7180000084594:258548-258680:+	ACA1_053310	Serine/threonine kinase [Source:UniProtKB/TrEMBL;Acc:L8H7D1]	0.0750386	0.655498333	0.580459733	0.00088344
3h	ACA1_020770/clean/scf7180000084095:2994-3071:+	ACA1_020770	Multisensor hybrid histidine kinase [Source:UniProtKB/TrEMBL;Acc:L8GWG6]	0.0445602	0.624488	0.5799278	8.23E-08
3h	ACA1_035640/clean/scf7180000084564:102553-102618:-	ACA1_035640	Beta-lactamase domain-containing protein [Source:UniProtKB/TrEMBL;Acc:L8H9P2]	0	0.579468333	0.579468333	0.004189009
3h	ACA1_384780/clean/scf7180000084730:101879-101987:+	ACA1_384780	MIT domain containing protein [Source:UniProtKB/TrEMBL;Acc:L8H937]	0.147674867	0.726948667	0.5792738	1.25E-07
3h	ACA1_146600/clean/scf7180000084713:68166-68264:-	ACA1_146600	Protein kinase domain containing protein [Source:UniProtKB/TrEMBL;Acc:L8GWQ5]	0.00263269	0.581543667	0.578910977	3.52E-12
3h	ACA1_088960/clean/scf7180000084639:275091-275161:-	ACA1_088960	Beta-lactamase domain-containing protein [Source:UniProtKB/TrEMBL;Acc:L8GU04]	0	0.578141	0.578141	1.06E-06
3h	ACA1_063310/clean/scf7180000084599:1380387-1380452:+	ACA1_063310	Phospholipid/glycerol acyltransferase, putative [Source:UniProtKB/TrEMBL;Acc:L8GX94]	0.115861467	0.693263667	0.5774022	1.81E-06
3h	ACA1_196310/clean/scf7180000084752:55582-55663:+	ACA1_196310	SET domain-containing protein [Source:UniProtKB/TrEMBL;Acc:L8HDZ8]	0.035096033	0.61235	0.577253967	1.66E-08
3h	ACA1_284250/clean/scf7180000084776:910885-910979:+	ACA1_284250	hypothetical protein	0.070588233	0.647781667	0.577193433	0.04602507
3h	ACA1_138500/clean/scf7180000084703:153574-153798:-	ACA1_138500	UBIQUITIN_CONJUGAT_2 domain-containing protein [Source:UniProtKB/TrEMBL;Acc:L8H0X9]	0.060433267	0.637425	0.576991733	8.27E-07
3h	ACA1_224400/clean/scf7180000084760:192704-192861:+	ACA1_224400	F_actin_bund_C domain-containing protein [Source:UniProtKB/TrEMBL;Acc:L8GVG0]	0.007199087	0.584115	0.576915913	6.28E-15
3h	ACA1_118750/clean/scf7180000084671:18638-18714:-	ACA1_118750	Threonylcarbamoyl-AMP synthase [Source:UniProtKB/TrEMBL;Acc:L8GLU7]	0.01639465	0.593284333	0.576889683	2.71E-06
3h	ACA1_071190/clean/scf7180000084603:886799-886897:+	ACA1_071190	RNA_ligase domain-containing protein [Source:UniProtKB/TrEMBL;Acc:L8HFI2]	0	0.576599667	0.576599667	0.000360462
3h	ACA1_048110/clean/scf7180000084582:24254-24512:-	ACA1_048110	Serine carboxypeptidase s28 protein [Source:UniProtKB/TrEMBL;Acc:L8GD90]	0.025974267	0.602533333	0.576559067	4.70E-09
3h	ACA1_089240/clean/scf7180000084639:346398-346461:+	ACA1_089240	Ubiquitin_4 domain-containing protein [Source:UniProtKB/TrEMBL;Acc:L8GX75]	0.271467333	0.847909667	0.576442333	3.50E-05
3h	ACA1_182190/clean/scf7180000084745:150193-150310:-	ACA1_182190	hypothetical protein	0.095119733	0.671502333	0.5763826	3.03E-05
3h	ACA1_215600/clean/scf7180000084758:347256-347381:-	ACA1_215600	hypothetical protein	0.149857333	0.726238333	0.576381	0.029727913
3h	ACA1_096430/clean/scf7180000084645:1009673-1009757:-	ACA1_096430	Aminotran_1_2 domain-containing protein [Source:UniProtKB/TrEMBL;Acc:L8GIY6]	0.004312787	0.580453333	0.576140547	8.20E-08
3h	ACA1_128500/clean/scf7180000084697:73280-73366:-	ACA1_128500	UBIQUITIN_CONJUGAT_2 domain-containing protein [Source:UniProtKB/TrEMBL;Acc:L8GWJ5]	0.0101573	0.585950667	0.575793367	4.19E-10
3h	ACA1_087900/clean/scf7180000084639:127577-127668:-	ACA1_087900	hypothetical protein	0.038220067	0.613864333	0.575644267	2.15E-05
3h	ACA1_060650/clean/scf7180000084599:724928-725019:+	ACA1_060650	Protein kinase domain-containing protein [Source:UniProtKB/TrEMBL;Acc:L8GXP5]	0.0168867	0.592290333	0.575403633	8.02E-07
3h	ACA1_325610/clean/scf7180000084495:50432-50509:-	ACA1_325610	Protein kinase domain containing protein [Source:UniProtKB/TrEMBL;Acc:L8H900]	0.012638153	0.588032	0.575393847	2.22E-08
3h	ACA1_099740/clean/scf7180000084647:19101-19234:-	ACA1_099740	COesterase domain-containing protein [Source:UniProtKB/TrEMBL;Acc:L8HMJ7]	0.028048867	0.603417	0.575368133	9.30E-07
3h	ACA1_379460/clean/scf7180000084711:369864-369947:-	ACA1_379460	F-box domain-containing protein [Source:UniProtKB/TrEMBL;Acc:L8GRH3]	0.350224	0.925480667	0.575256667	0.00035957
3h	ACA1_040740/clean/scf7180000084572:72323-72412:+	ACA1_040740	SAM-dependent MTase TRM10-type domain-containing protein [Source:UniProtKB/TrEMBL;Acc:L8H1W9]	0.001461187	0.576559667	0.57509848	1.38E-07
3h	ACA1_036350/clean/scf7180000084565:84116-84193:+	ACA1_036350	hypothetical protein	0.0575097	0.632415667	0.574905967	4.15E-06
3h	ACA1_364750/clean/scf7180000084641:190220-190277:-	ACA1_364750	UBP-type domain-containing protein [Source:UniProtKB/TrEMBL;Acc:L8GLN3]	0.022806	0.597604333	0.574798333	1.35E-10
3h	ACA1_193170/clean/scf7180000084749:204307-204453:-	ACA1_193170	JmjC domain-containing protein [Source:UniProtKB/TrEMBL;Acc:L8GNQ7]	0.016603933	0.59136	0.574756067	4.13E-10
3h	ACA1_175700/clean/scf7180000084741:1038084-1038176:-	ACA1_175700	Frizzled/Smoothened family membrane region protein [Source:UniProtKB/TrEMBL;Acc:L8HH45]	0.00615966	0.580882667	0.574723007	2.80E-07
3h	ACA1_362250/clean/scf7180000084624:36792-36861:+	ACA1_362250	hypothetical protein	0.069151367	0.643814333	0.574662967	9.29E-06
3h	ACA1_388780/clean/scf7180000084733:169440-169528:+	ACA1_388780	EGFlike domain containing protein [Source:UniProtKB/TrEMBL;Acc:L8GDT8]	0.004208237	0.578705667	0.57449743	1.37E-09
3h	ACA1_088880/clean/scf7180000084639:253579-253653:-	ACA1_088880	OGFr_N domain-containing protein [Source:UniProtKB/TrEMBL;Acc:L8GUP7]	0.02335126	0.597839667	0.574488407	0.000226176
3h	ACA1_142140/clean/scf7180000084708:94875-94970:+	ACA1_142140	unspecified product	0.101154367	0.675308333	0.574153967	1.70E-05
3h	ACA1_071970/clean/scf7180000084603:1118900-1118980:+	ACA1_071970	Carn_acyltransf domain-containing protein [Source:UniProtKB/TrEMBL;Acc:L8HEI6]	0	0.573638667	0.573638667	1.06E-09
3h	ACA1_216300/clean/scf7180000084758:461860-462045:+	ACA1_216300	Ras family protein [Source:UniProtKB/TrEMBL;Acc:L8GR60]	0.008605767	0.582192	0.573586233	2.19E-06
3h	ACA1_291340/clean/scf7180000084777:749586-749657:-	ACA1_291340	Ubiquitin-like domain-containing protein [Source:UniProtKB/TrEMBL;Acc:L8HLN7]	0.010132683	0.583606	0.573473317	4.52E-10

3h	ACA1_170210/clean/scf7180000084739:72847-72936:-	ACA1_170210	hypothetical protein	0.027538837	0.600906667	0.57336783	0.000246469
3h	ACA1_289740/clean/scf7180000084777:453772-453865:+	ACA1_289740	Rho-GAP domain-containing protein [Source:UniProtKB/TrEMBL;Acc:L8HLF4]	0.014171033	0.587501	0.573329967	2.61E-06
3h	ACA1_299810/clean/scf7180000083079:11487-11570:+	ACA1_299810	hypothetical protein	0.005858573	0.578998	0.573139427	8.44E-08
3h	ACA1_116190/clean/scf7180000084669:722919-722978:-	ACA1_116190	Protein kinase domain-containing protein [Source:UniProtKB/TrEMBL;Acc:L8H600]	0.011904767	0.584484333	0.572579567	3.34E-05
3h	ACA1_063690/clean/scf7180000084599:1475674-1475729:-	ACA1_063690	Amidase domain-containing protein [Source:UniProtKB/TrEMBL;Acc:L8GXA6]	0.0794748	0.651657	0.5721822	2.60E-07
3h	ACA1_103630/clean/scf7180000084653:47179-47296:+	ACA1_103630	Peroxidase [Source:UniProtKB/TrEMBL;Acc:L8GCR8]	0.000907718	0.572306333	0.571398616	3.61E-17
3h	ACA1_182400/clean/scf7180000084745:205223-205407:+	ACA1_182400	Integral membrane protein [Source:UniProtKB/TrEMBL;Acc:L8H9C3]	0.011629757	0.58295	0.571320243	2.14E-11
3h	ACA1_173350/clean/scf7180000084741:468908-469026:-	ACA1_173350	Carrier superfamily protein [Source:UniProtKB/TrEMBL;Acc:L8HHM8]	0.000968591	0.572243	0.571274409	2.01E-11
3h	ACA1_279550/clean/scf7180000084776:412850-412950:-	ACA1_279550	CBS domain containing protein [Source:UniProtKB/TrEMBL;Acc:L8H8C1]	0.0330853	0.604116	0.5710307	1.85E-07
3h	ACA1_295320/clean/scf7180000084777:1104376-1104491:+	ACA1_295320	hypothetical protein	0.026364733	0.597134667	0.570769933	4.46E-08
3h	ACA1_391420/clean/scf7180000084740:169174-169273:+	ACA1_391420	hypothetical protein	0.01187462	0.582589333	0.570714713	8.26E-10
3h	ACA1_088880/clean/scf7180000084639:253988-254051:-	ACA1_088880	OGFr_N domain-containing protein [Source:UniProtKB/TrEMBL;Acc:L8GUP7]	0.052525267	0.623139667	0.5706144	0.003148223
3h	ACA1_186900/clean/scf7180000084746:219860-219973:-	ACA1_186900	Synapsin, ATP binding domain containing protein [Source:UniProtKB/TrEMBL;Acc:L8H7B4]	0.008411733	0.578966667	0.570554933	7.48E-06
3h	ACA1_260200/clean/scf7180000084769:455569-455647:-	ACA1_260200	RNA recognition motif domain containing protein [Source:UniProtKB/TrEMBL;Acc:L8GHK6]	0.016403903	0.586500667	0.570096763	1.12E-09
3h	ACA1_169580/clean/scf7180000084739:44606-44690:+	ACA1_169580	hypothetical protein	0.006495487	0.576396333	0.569900846	8.21E-08
3h	ACA1_380340/clean/scf7180000084712:41224-41301:-	ACA1_380340	Leucine rich repeat-containing protein [Source:UniProtKB/TrEMBL;Acc:L8GNJ3]	0.007395343	0.577023	0.569627657	1.84E-07
3h	ACA1_138520/clean/scf7180000084703:160269-160390:+	ACA1_138520	Phospholipase B-like [Source:UniProtKB/TrEMBL;Acc:L8GZK2]	0.003527313	0.573057667	0.569530353	8.97E-09
3h	ACA1_287590/clean/scf7180000084777:41331-41396:+	ACA1_287590	hypothetical protein	0.0279356	0.596819333	0.568883733	9.26E-07
3h	ACA1_325170/clean/scf7180000084475:15371-15565:-	ACA1_325170	MHD domain-containing protein [Source:UniProtKB/TrEMBL;Acc:L8GPP2]	0.002943879	0.571471333	0.568527455	3.11E-10
3h	ACA1_246100/clean/scf7180000084763:536023-536097:-	ACA1_246100	ANK_REP_REGION domain-containing protein [Source:UniProtKB/TrEMBL;Acc:L8GKB9]	0.308712333	0.877068333	0.568356	0.000405132
3h	ACA1_251640/clean/scf7180000084768:98382-98495:+	ACA1_251640	CBS domain-containing protein [Source:UniProtKB/TrEMBL;Acc:L8HAQ8]	0	0.568083333	0.568083333	5.65E-09
3h	ACA1_024200/clean/scf7180000084441:15525-15618:-	ACA1_024200	WD_REPEATS_REGION domain-containing protein [Source:UniProtKB/TrEMBL;Acc:L8GKS0]	0	0.568061667	0.568061667	3.09E-07
3h	ACA1_341070/clean/scf7180000084620:45270-45348:-	ACA1_341070	Peroxisomal membrane 22 kDa family protein [Source:UniProtKB/TrEMBL;Acc:L8GRB3]	0.00352592	0.571492667	0.567966747	5.68E-09
3h	ACA1_173330/clean/scf7180000084741:465227-465294:+	ACA1_173330	Fbox domain containing protein [Source:UniProtKB/TrEMBL;Acc:L8HHQ0]	0.195952333	0.763784333	0.567832	1.21E-05
3h	ACA1_096440/clean/scf7180000084645:1011284-1011382:+	ACA1_096440	RhoGAP domain containing protein [Source:UniProtKB/TrEMBL;Acc:L8GJR5]	0.012111613	0.579755333	0.56764372	2.77E-08
3h	ACA1_074100/clean/scf7180000084605:23993-24088:+	ACA1_074100	Fungal Zn(2)-Cys(6) binuclear cluster domain containing protein [Source:UniProtKB/TrEMBL;Acc:L8HLU8]	0.057729067	0.624867333	0.567138267	9.13E-08
3h	ACA1_154690/clean/scf7180000084722:78274-78372:+	ACA1_154690	unspecified product	5.12E-05	0.567137667	0.567086487	3.33E-10
3h	ACA1_271330/clean/scf7180000084771:20879-20960:+	ACA1_271330	hypothetical protein	0.0765215	0.643501	0.5669795	5.38E-05
3h	ACA1_172880/clean/scf7180000084741:348146-348217:+	ACA1_172880	Protein kinase domain-containing protein [Source:UniProtKB/TrEMBL;Acc:L8HJV9]	0.036029633	0.602956	0.566926367	8.96E-08
3h	ACA1_173790/clean/scf7180000084741:520286-520366:+	ACA1_173790	Nicotinamide-nucleotide adenylyltransferase [Source:UniProtKB/TrEMBL;Acc:L8HK06]	0.018726633	0.585624667	0.566898033	9.18E-08
3h	ACA1_163990/clean/scf7180000084729:126982-127047:-	ACA1_163990	Glycosyl hydrolases family 32 superfamily protein [Source:UniProtKB/TrEMBL;Acc:L8GRF2]	0.026394233	0.592543	0.566148767	7.53E-06
3h	ACA1_383660/clean/scf7180000084721:240089-240200:+	ACA1_383660	tRNA (guanine-N(7)-)-methyltransferase non-catalytic subunit [Source:UniProtKB/TrEMBL;Acc:L8GW53]	0.014976367	0.581119	0.566142633	2.12E-08
3h	ACA1_017740/clean/scf7180000083876:7199-7289:+	ACA1_017740	5'-3' exoribonuclease 1 [Source:UniProtKB/TrEMBL;Acc:L8GMR4]	0.008358059	0.574209333	0.565851274	1.50E-12
3h	ACA1_113870/clean/scf7180000084669:392641-392775:+	ACA1_113870	CUB domain containing protein [Source:UniProtKB/TrEMBL;Acc:L8H6F7]	0.006800127	0.572099667	0.56529954	6.59E-08
3h	ACA1_371760/clean/scf7180000084664:600814-600921:-	ACA1_371760	PH domain-containing protein [Source:UniProtKB/TrEMBL;Acc:L8H1R1]	0.001041386	0.566311667	0.565270281	1.59E-08
3h	ACA1_074140/clean/scf7180000084605:36353-36439:+	ACA1_074140	Transporter, major facilitator subfamily protein [Source:UniProtKB/TrEMBL;Acc:L8HK94]	0.015288217	0.580034667	0.56474645	3.41E-10
3h	ACA1_330400/clean/scf7180000084566:52801-52937:-	ACA1_330400	SURF1-like protein [Source:UniProtKB/TrEMBL;Acc:L8GJV9]	0.01019858	0.574664	0.56446542	8.36E-11
3h	ACA1_103160/clean/scf7180000084653:9668-9738:+	ACA1_103160	PALP domain-containing protein [Source:UniProtKB/TrEMBL;Acc:L8GDL9]	0.010154307	0.574408	0.564253693	2.89E-09
3h	ACA1_200010/clean/scf7180000084753:368354-368532:+	ACA1_200010	Protein kinase domain containing protein [Source:UniProtKB/TrEMBL;Acc:L8H3C5]	0.005882207	0.569825333	0.563943127	1.16E-08
3h	ACA1_069730/clean/scf7180000084603:538907-538984:+	ACA1_069730	Lysozyme, putative [Source:UniProtKB/TrEMBL;Acc:L8HF46]	0.003746057	0.567561	0.563814943	6.23E-12
3h	ACA1_183210/clean/scf7180000084745:357657-357771:-	ACA1_183210	Thioredoxin domain-containing protein [Source:UniProtKB/TrEMBL;Acc:L8H812]	0.111111	0.674909	0.563798	0.012490627
3h	ACA1_400570/clean/scf7180000084775:198113-198199:-	ACA1_400570	Ubiquitin domain containing protein [Source:UniProtKB/TrEMBL;Acc:L8GGV7]	0.004390506	0.567913	0.563522494	5.97E-12
3h	ACA1_383660/clean/scf7180000084721:240313-240397:+	ACA1_383660	tRNA (guanine-N(7)-)-methyltransferase non-catalytic subunit [Source:UniProtKB/TrEMBL;Acc:L8GW53]	0.001723873	0.564722667	0.562998793	5.68E-10
3h	ACA1_103160/clean/scf7180000084653:10002-10093:+	ACA1_103160	PALP domain-containing protein [Source:UniProtKB/TrEMBL;Acc:L8GDL9]	0.00451657	0.567484333	0.562967763	1.05E-09
3h	ACA1_170770/clean/scf7180000084741:556-670:-	ACA1_170770	UBP-type domain-containing protein [Source:UniProtKB/TrEMBL;Acc:L8HJG8]	0.010643083	0.573395	0.562751917	9.04E-11
3h	ACA1_367290/clean/scf7180000084641:591222-591322:-	ACA1_367290	hypothetical protein	0.001031852	0.563768667	0.562736815	5.98E-10
3h	ACA1_283310/clean/scf7180000084776:814168-814262:-	ACA1_283310	SAM domain-containing protein [Source:UniProtKB/TrEMBL;Acc:L8H6L5]	0.039888633	0.602536333	0.5626477	4.23E-06
3h	ACA1_070620/clean/scf7180000084603:749045-749133:+	ACA1_070620	Myotubularin phosphatase domain-containing protein [Source:UniProtKB/TrEMBL;Acc:L8HFC4]	0.015118833	0.577755333	0.5626365	7.09E-11
3h	ACA1_208140/clean/scf7180000084757:113957-114035:-	ACA1_208140	ER lumen proteinretaining receptor-like protein [Source:UniProtKB/TrEMBL;Acc:L8GYA7]	0.042839667	0.605425667	0.562586	1.41E-07
3h	ACA1_184160/clean/scf7180000084745:573081-573213:+	ACA1_184160	AAA domain-containing protein [Source:UniProtKB/TrEMBL;Acc:L8H8H4]	0.00488283	0.567323667	0.562440837	8.37E-13
3h	ACA1_075030/clean/scf7180000084608:131508-131570:+	ACA1_075030	Serine carboxypeptidase [Source:UniProtKB/TrEMBL;Acc:L8HFJ6]	0.074645167	0.636896667	0.5622515	3.76E-08
3h	ACA1_384050/clean/scf7180000084730:11492-11587:+	ACA1_384050	hypothetical protein	0.0236493	0.585742667	0.562093367	3.80E-08
3h	ACA1_092000/clean/scf7180000084645:244529-244654:-	ACA1_092000	hypothetical protein	0.016393433	0.578462333	0.5620689	6.09E-08
3h	ACA1_396260/clean/scf7180000084759:65295-65402:+	ACA1_396260	hypothetical protein	0.020881433	0.582865667	0.561984233	2.14E-08
3h	ACA1_120250/clean/scf7180000084675:11997-12066:+	ACA1_120250	Doublecortin domain-containing protein [Source:UniProtKB/TrEMBL;Acc:L8GVW9]	0.025395883	0.587271667	0.561875783	8.70E-11
3h	ACA1_045250/clean/scf7180000084576:93674-93744:+	ACA1_045250	hypothetical protein	0.177485333	0.739268333	0.561783	5.38E-06
3h	ACA1_189450/clean/scf7180000084748:43020-43103:-	ACA1_189450	Chloride channel protein [Source:UniProtKB/TrEMBL;Acc:L8GEJ2]	0.0197996	0.581465333	0.561665733	1.06E-09
3h	ACA1_295880/clean/scf7180000084777:1250924-1251027:+	ACA1_295880	Malic enzyme [Source:UniProtKB/TrEMBL;Acc:L8HM80]	0.001072168	0.562721	0.561648832	1.05E-08
3h	ACA1_107530/clean/scf7180000084660:215836-215931:-	ACA1_107530	UBP-type domain-containing protein [Source:UniProtKB/TrEMBL;Acc:L8GMW8]	0.19433	0.755900667	0.561570667	2.76E-05
3h	ACA1_071170/clean/scf7180000084603:881893-881972:+	ACA1_071170	RhoGAP domain containing protein [Source:UniProtKB/TrEMBL;Acc:L8HGC8]	0	0.561259	0.561259	2.74E-07
3h	ACA1_214990/clean/scf7180000084758:286387-286463:-	ACA1_214990	hypothetical protein	0.106937533	0.668109667	0.561172133	0.004503757
3h	ACA1_264460/clean/scf7180000084770:293954-294030:-	ACA1_264460	GTPbinding protein [Source:UniProtKB/TrEMBL;Acc:L8H231]	0.011893653	0.572992667	0.561099013	5.60E-08
3h	ACA1_012970/clean/scf7180000083742:13466-13572:+	ACA1_012970	CBS domain containing protein [Source:UniProtKB/TrEMBL;Acc:L8H720]	0.0451726	0.606059	0.5608864	1.17E-05
3h	ACA1_073230/clean/scf7180000084603:1376453-1376536:-	ACA1_073230	EHN domain-containing protein [Source:UniProtKB/TrEMBL;Acc:L8HDT9]	0.01860667	0.579204667	0.560597997	1.73E-07
3h	ACA1_274930/clean/scf7180000084773:142635-142725:-	ACA1_274930	Histone deacetylaserelated, putative [Source:UniProtKB/TrEMBL;Acc:L8GFN9]	0.01870869	0.579229667	0.560520977	7.46E-10
3h	ACA1_265560/clean/scf7180000084770:510918-511000:+	ACA1_265560	RFX-type winged-helix domain-containing protein [Source:UniProtKB/TrEMBL;Acc:L8H4F0]	0.005079747	0.565460333	0.560380587	1.10E-07
3h	ACA1_198340/clean/scf7180000084753:82983-83064:-	ACA1_198340	Pept_C1 domain-containing protein [Source:UniProtKB/TrEMBL;Acc:L8H2C3]	0.002531647	0.562866667	0.56033502	3.83E-12
3h	ACA1_264180/clean/scf7180000084770:206798-206889:+	ACA1_264180	Oxidoreductase, short chain dehydrogenase/reductase superfamily protein [Source:UniProtKB/TrEMBL;Acc:L8H208]	0.143991333	0.704250667	0.560259333	2.15E-05
3h	ACA1_141960/clean/scf7180000084708:61214-61304:-	ACA1_141960	unspecified product	0.010021403	0.570042333	0.56002093	1.02E-07
3h	ACA1_252610/clean/scf7180000084768:189232-189315:-	ACA1_252610	ADPribosylglycohydrolase superfamily protein [Source:UniProtKB/TrEMBL;Acc:L8HD40]	0.007806923	0.567818667	0.560011743	2.76E-10
3h	ACA1_395500/clean/scf7180000084756:195233-195321:-	ACA1_395500	Rho-GAP domain-containing protein [Source:UniProtKB/TrEMBL;Acc:L8GZP8]	0.004460577	0.564371	0.559910423	2.47E-08
3h	ACA1_165580/clean/scf7180000084732:55681-55784:-	ACA1_165580	BTB/POZ domain containing protein [Source:UniProtKB/TrEMBL;Acc:L8HI24]	0.0515842	0.611319667	0.559735467	2.14E-05
3h	ACA1_252800/clean/scf7180000084768:220260-220346:+	ACA1_252800	Dual specificity phosphatase, catalytic domain containing protein [Source:UniProtKB/TrEMBL;Acc:L8H9X0]	0.047619067	0.607333333	0.559714267	7.12E-05
3h	ACA1_061680/clean/scf7180000084599:1010082-1010167:+	ACA1_061680	PH domain containing protein [Source:UniProtKB/TrEMBL;Acc:L8GXZ1]	0.01437063	0.574006333	0.559635703	8.41E-07
3h	ACA1_012330/clean/scf7180000083740:16392-16483:+	ACA1_012330	WD_REPEATS_REGION domain-containing protein [Source:UniProtKB/TrEMBL;Acc:L8HCD7]	0.003583483	0.563044333	0.55946085	7.97E-09
3h	ACA1_372660/clean/scf7180000084679:40513-40594:-	ACA1_372660	hypothetical protein	0.023311372	0.582368667	0.559057295	0.000505574
3h	ACA1_389110/clean/scf7180000084733:197830-197923:+	ACA1_389110	hypothetical protein	0.001308545	0.560316667	0.559008121	3.05E-12
3h	ACA1_224310/clean/scf7180000084760:167219-167307:+	ACA1_224310	PH domain-containing protein [Source:UniProtKB/TrEMBL;Acc:L8GTW7]	0.003003003	0.561881	0.558877997	1.08E-08
3h	ACA1_107530/clean/scf7180000084660:216036-216160:-	ACA1_107530	UBP-type domain-containing protein [Source:UniProtKB/TrEMBL;Acc:L8GMW8]	0.0085186	0.567068667	0.558550067	9.48E-11
3h	ACA1_024420/clean/scf7180000084442:23168-23242:+	ACA1_024420	TatD DNase domain containing 1, putative [Source:UniProtKB/TrEMBL;Acc:L8H9Z1]	0.291218667	0.849560333	0.558341667	0.000168205
3h	ACA1_385640/clean/scf7180000084730:222419-222536:+	ACA1_385640	hypothetical protein	0.009889153	0.568088	0.558198847	6.09E-07
3h	ACA1_069730/clean/scf7180000084603:538676-538805:+	ACA1_069730	Lysozyme, putative [Source:UniProtKB/TrEMBL;Acc:L8HF46]	0.018065233	0.575933333	0.5578681	6.20E-12
3h	ACA1_116990/clean/scf7180000084669:886068-886156:+	ACA1_116990	Carboxypeptidase [Source:UniProtKB/TrEMBL;Acc:L8H6Y6]	0.008439973	0.566219333	0.55777936	1.09E-10
3h	ACA1_186200/clean/scf7180000084746:170750-170827:+	ACA1_186200	ATPase/histidine kinase/DNA gyrase B/HSP90 domain containing protein [Source:UniProtKB/TrEMBL;Acc:L8H4A4]	0.00257566	0.559833	0.55725734	2.27E-05
3h	ACA1_035640/clean/scf7180000084564:103277-103356:-	ACA1_035640	Beta-lactamase domain-containing protein [Source:UniProtKB/TrEMBL;Acc:L8H9P2]	0	0.557113667	0.557113667	0.004802628
3h	ACA1_119430/clean/scf7180000084672:66464-66597:+	ACA1_119430	RRM domain-containing protein [Source:UniProtKB/TrEMBL;Acc:L8HKC8]	0.001883167	0.558993333	0.557110166	2.47E-09
3h	ACA1_027810/clean/scf7180000084471:24520-24598:+	ACA1_027810	YTH domain-containing protein [Source:UniProtKB/TrEMBL;Acc:L8HJ39]	0.004830933	0.561881	0.557050067	7.68E-07
3h	ACA1_042070/clean/scf7180000084573:53936-54010:+	ACA1_042070	Dual specificity protein kinase [Source:UniProtKB/TrEMBL;Acc:L8GW91]	0.1310173	0.688022	0.5570047	4.49E-05
3h	ACA1_208100/clean/scf7180000084757:101353-101427:+	ACA1_208100	hypothetical protein	0.002700589	0.559445333	0.556744744	4.76E-08
3h	ACA1_341100/clean/scf7180000084620:51340-51404:-	ACA1_341100	MFS domain-containing protein [Source:UniProtKB/TrEMBL;Acc:L8GR49]	0.202464	0.758285	0.555821	3.64E-05
3h	ACA1_182400/clean/scf7180000084745:204326-204539:+	ACA1_182400	Integral membrane protein [Source:UniProtKB/TrEMBL;Acc:L8H9C3]	0.186093667	0.741529	0.555435333	1.08E-05
3h	ACA1_002780/clean/scf7180000082937:9646-9732:-	ACA1_002780	RRM domain-containing protein [Source:UniProtKB/TrEMBL;Acc:L8GGZ7]	0.029124867	0.584032	0.554907133	1.88E-06
3h	ACA1_271580/clean/scf7180000084771:78428-78551:-	ACA1_271580	Protein phosphatase 1, regulatory subunit 7, putative [Source:UniProtKB/TrEMBL;Acc:L8GPQ8]	0.046985767	0.601863667	0.5548779	2.86E-08
3h	ACA1_360090/clean/scf7180000084622:48555-48653:-	ACA1_360090	hypothetical protein	0.0406421	0.595354	0.5547119	4.47E-06
3h	ACA1_271330/clean/scf7180000084771:21073-21211:+	ACA1_271330	hypothetical protein	0.00058165	0.555094667	0.554513017	7.58E-09
3h	ACA1_322380/clean/scf7180000084463:11695-11773:-	ACA1_322380	hypothetical protein	0.011904767	0.566079333	0.554174567	1.44E-06
3h	ACA1_173790/clean/scf7180000084741:520699-520797:+	ACA1_173790	Nicotinamide-nucleotide adenylyltransferase [Source:UniProtKB/TrEMBL;Acc:L8HK06]	0.01081114	0.564935333	0.554124193	2.58E-08
3h	ACA1_154690/clean/scf7180000084722:77713-77806:+	ACA1_154690	unspecified product	0.063661733	0.617758667	0.554096933	1.39E-06
3h	ACA1_230530/clean/scf7180000084761:373234-373308:+	ACA1_230530	S(Hydroxymethyl)glutathione dehydrogenase/class III alcohol dehydrogenase [Source:UniProtKB/TrEMBL;Acc:L8H8X2]	0.003878677	0.557937667	0.55405899	2.22E-15
3h	ACA1_156050/clean/scf7180000084723:13246-13325:+	ACA1_156050	Armadillo/betacatenin-like repeat domain containing protein [Source:UniProtKB/TrEMBL;Acc:L8GIE3]	0	0.553902667	0.553902667	0.040852295
3h	ACA1_197610/clean/scf7180000084753:30084-30206:-	ACA1_197610	hypothetical protein	0.166309	0.720173	0.553864	0.00036503
3h	ACA1_178060/clean/scf7180000084743:162467-162555:-	ACA1_178060	Dipeptidylpeptidase 9, putative [Source:UniProtKB/TrEMBL;Acc:L8GTD8]	0.00612182	0.559933333	0.553811513	3.68E-08
3h	ACA1_259980/clean/scf7180000084769:398355-398534:+	ACA1_259980	DUF4042 domain-containing protein [Source:UniProtKB/TrEMBL;Acc:L8GFM1]	0.267234667	0.820953333	0.553718667	0.000267793
3h	ACA1_385700/clean/scf7180000084730:241652-241741:+	ACA1_385700	F-box domain-containing protein [Source:UniProtKB/TrEMBL;Acc:L8HAP4]	0.013332467	0.566161	0.552828533	0.004725935
3h	ACA1_108210/clean/scf7180000084661:13246-13334:-	ACA1_108210	PH domain containing protein [Source:UniProtKB/TrEMBL;Acc:L8GD50]	0.0121788	0.564880333	0.552701533	5.92E-06
3h	ACA1_123350/clean/scf7180000084686:16397-16486:-	ACA1_123350	hypothetical protein	0.023553263	0.576144	0.552590737	2.56E-09
3h	ACA1_384790/clean/scf7180000084730:103919-103995:-	ACA1_384790	Cellulase domain-containing protein [Source:UniProtKB/TrEMBL;Acc:L8HAJ9]	0.025532367	0.578121333	0.552588967	2.55E-09
3h	ACA1_171870/clean/scf7180000084741:182777-183056:-	ACA1_171870	Leucine rich repeat domain containing protein [Source:UniProtKB/TrEMBL;Acc:L8HIV4]	0.072653533	0.625115333	0.5524618	0.000571292
3h	ACA1_183970/clean/scf7180000084745:533533-533601:-	ACA1_183970	hypothetical protein	0.17854344	0.730673	0.55212956	0.004732434
3h	ACA1_274870/clean/scf7180000084773:131144-131219:-	ACA1_274870	Leucine rich repeat domain containing protein [Source:UniProtKB/TrEMBL;Acc:L8GFN3]	0.034425233	0.586136667	0.551711433	3.25E-05
3h	ACA1_251640/clean/scf7180000084768:98620-98709:+	ACA1_251640	CBS domain-containing protein [Source:UniProtKB/TrEMBL;Acc:L8HAQ8]	0	0.551711333	0.551711333	8.47E-09
3h	ACA1_367180/clean/scf7180000084641:562268-562320:-	ACA1_367180	Profilin [Source:UniProtKB/TrEMBL;Acc:L8GM22]	0	0.551372	0.551372	3.60E-08
3h	ACA1_077120/clean/scf7180000084612:240145-240249:-	ACA1_077120	DH domain-containing protein [Source:UniProtKB/TrEMBL;Acc:L8GM77]	0.064219167	0.615571	0.551351833	1.68E-06
3h	ACA1_092000/clean/scf7180000084645:243958-244035:-	ACA1_092000	hypothetical protein	0.047866533	0.599189667	0.551323133	3.63E-07
3h	ACA1_394730/clean/scf7180000084756:131130-131192:-	ACA1_394730	Putative tyrosineprotein phosphatase oca1, putative [Source:UniProtKB/TrEMBL;Acc:L8H2T3]	0.0306138	0.581655333	0.551041533	8.35E-08
3h	ACA1_072660/clean/scf7180000084603:1247629-1247710:+	ACA1_072660	hypothetical protein	0	0.550923667	0.550923667	0.004248862
3h	ACA1_114470/clean/scf7180000084669:482446-482528:+	ACA1_114470	C2 domain containing protein [Source:UniProtKB/TrEMBL;Acc:L8H5S6]	0.139272333	0.689759	0.550486667	0.009703453
3h	ACA1_156050/clean/scf7180000084723:14447-14504:+	ACA1_156050	Armadillo/betacatenin-like repeat domain containing protein [Source:UniProtKB/TrEMBL;Acc:L8GIE3]	0	0.55028	0.55028	0.012142207
3h	ACA1_170940/clean/scf7180000084741:19208-19275:+	ACA1_170940	Proteasome subunit alpha type [Source:UniProtKB/TrEMBL;Acc:L8HH61]	0.00295996	0.553237333	0.550277373	3.46E-16
3h	ACA1_385810/clean/scf7180000084730:270939-271053:+	ACA1_385810	Rap-GAP domain-containing protein [Source:UniProtKB/TrEMBL;Acc:L8HAQ1]	0.007870893	0.557740333	0.54986944	3.75E-09
3h	ACA1_158440/clean/scf7180000084724:218990-219268:+	ACA1_158440	Coiled-coil domain containing 53, putative [Source:UniProtKB/TrEMBL;Acc:L8HA48]	0.068135167	0.617782667	0.5496475	1.00E-06
3h	ACA1_131240/clean/scf7180000084699:37234-37314:+	ACA1_131240	Alpha-galactosidase [Source:UniProtKB/TrEMBL;Acc:L8GN31]	0.0802267	0.629783667	0.549556967	1.15E-07
3h	ACA1_070820/clean/scf7180000084603:769554-769636:+	ACA1_070820	hypothetical protein	0.013542313	0.562880667	0.549338353	1.59E-05
3h	ACA1_333220/clean/scf7180000084581:22910-22983:+	ACA1_333220	hypothetical protein	0.0136492	0.562551333	0.548902133	1.55E-08
3h	ACA1_199630/clean/scf7180000084753:282051-282138:-	ACA1_199630	PRAI domain-containing protein [Source:UniProtKB/TrEMBL;Acc:L8H3A6]	0.102753667	0.650864667	0.548111	3.37E-06
3h	ACA1_378010/clean/scf7180000084711:163311-163380:+	ACA1_378010	JmjC domain-containing protein [Source:UniProtKB/TrEMBL;Acc:L8GRT4]	0.416722	0.964747	0.548025	2.44E-06
3h	ACA1_106720/clean/scf7180000084660:109896-110061:-	ACA1_106720	CID domain-containing protein [Source:UniProtKB/TrEMBL;Acc:L8GQ94]	0.0723963	0.620174667	0.547778367	1.48E-05
3h	ACA1_070980/clean/scf7180000084603:821585-821647:-	ACA1_070980	hypothetical protein	0.027907333	0.575069667	0.547162333	1.19E-08
3h	ACA1_255530/clean/scf7180000084768:534087-534180:-	ACA1_255530	Carrier superfamily protein [Source:UniProtKB/TrEMBL;Acc:L8HB08]	0.034493267	0.581528333	0.547035067	3.50E-07
3h	ACA1_060920/clean/scf7180000084599:781530-781651:-	ACA1_060920	MFS domain-containing protein [Source:UniProtKB/TrEMBL;Acc:L8GVT5]	0.0329477	0.579535667	0.546587967	7.30E-06
3h	ACA1_108260/clean/scf7180000084661:24195-24262:-	ACA1_108260	Prenylcys_lyase domain-containing protein [Source:UniProtKB/TrEMBL;Acc:L8GD56]	0.015873	0.562453	0.54658	1.74E-06
3h	ACA1_326190/clean/scf7180000084504:50397-50502:+	ACA1_326190	F-box domain-containing protein [Source:UniProtKB/TrEMBL;Acc:L8HH77]	0.0250405	0.571346	0.5463055	0.000191026
3h	ACA1_270200/clean/scf7180000084770:1076880-1076967:-	ACA1_270200	FYVE-type domain-containing protein [Source:UniProtKB/TrEMBL;Acc:L8H522]	0.0212766	0.567440333	0.546163733	0.003385837
3h	ACA1_089760/clean/scf7180000084639:376902-377006:-	ACA1_089760	Betaglucuronidase, putative [Source:UniProtKB/TrEMBL;Acc:L8GVR6]	0.0410498	0.585923	0.5448732	0.025788815
3h	ACA1_247250/clean/scf7180000084763:655678-655746:-	ACA1_247250	MBD domain-containing protein [Source:UniProtKB/TrEMBL;Acc:L8GMS1]	0.00582798	0.550691333	0.544863353	3.73E-07
3h	ACA1_206370/clean/scf7180000084757:17262-17334:+	ACA1_206370	MOSC domain-containing protein [Source:UniProtKB/TrEMBL;Acc:L8GY21]	0.003648607	0.548493333	0.544844727	1.13E-09
3h	ACA1_061670/clean/scf7180000084599:1002349-1002427:-	ACA1_061670	SSD domain-containing protein [Source:UniProtKB/TrEMBL;Acc:L8GWV2]	0	0.544804667	0.544804667	4.92E-07
3h	ACA1_225900/clean/scf7180000084761:17997-18117:+	ACA1_225900	General transcription factor IIH subunit 4 [Source:UniProtKB/TrEMBL;Acc:L8H8M7]	0.317044333	0.861561667	0.544517333	0.001246429

3h	ACA1_228760/clean/scf7180000084761:208247-208349:+	ACA1_228760	Mitochondrial carrier protein [Source:UniProtKB/TrEMBL;Acc:L8H8X8]	0.012959327	0.557337667	0.54437834	3.58E-07
3h	ACA1_068040/clean/scf7180000084603:85068-85143:+	ACA1_068040	START domain-containing protein [Source:UniProtKB/TrEMBL;Acc:L8HDG1]	0.008607713	0.552733667	0.544125953	1.45E-06
3h	ACA1_230540/clean/scf7180000084761:374608-374681:+	ACA1_230540	hypothetical protein	0.012364003	0.556156333	0.54379233	3.03E-11
3h	ACA1_210540/clean/scf7180000084758:52571-52660:-	ACA1_210540	ABC transporter, ATPbinding domain containing protein [Source:UniProtKB/TrEMBL;Acc:L8GPZ1]	0.124966333	0.668748	0.543781667	0.010077074
3h	ACA1_393390/clean/scf7180000084756:54963-55084:+	ACA1_393390	Exostosin domain-containing protein [Source:UniProtKB/TrEMBL;Acc:L8H1N1]	0.001046177	0.544776667	0.54373049	6.46E-08
3h	ACA1_061150/clean/scf7180000084599:837026-837153:-	ACA1_061150	UBA/TSN domain containing protein [Source:UniProtKB/TrEMBL;Acc:L8GWQ2]	0.00290158	0.546059	0.54315742	6.17E-07
3h	ACA1_131000/clean/scf7180000084698:147497-147588:-	ACA1_131000	Nudix hydrolase domain-containing protein [Source:UniProtKB/TrEMBL;Acc:L8GPF6]	0.006098203	0.549153667	0.543055463	1.54E-07
3h	ACA1_074140/clean/scf7180000084605:35972-36043:+	ACA1_074140	Transporter, major facilitator subfamily protein [Source:UniProtKB/TrEMBL;Acc:L8HK94]	0.014724867	0.557765	0.543040133	4.32E-11
3h	ACA1_153630/clean/scf7180000084720:142691-142766:+	ACA1_153630	OTU domain-containing protein [Source:UniProtKB/TrEMBL;Acc:L8HHG7]	5.83E-05	0.543084	0.543025735	7.97E-10
3h	ACA1_108220/clean/scf7180000084661:17262-17336:+	ACA1_108220	hypothetical protein	0.014654557	0.557555333	0.542900777	5.21E-06
3h	ACA1_067470/clean/scf7180000084601:34845-34913:-	ACA1_067470	hypothetical protein	0.0183403	0.561125333	0.542785033	3.11E-09
3h	ACA1_277480/clean/scf7180000084776:61383-61451:-	ACA1_277480	DegPtype protease [Source:UniProtKB/TrEMBL;Acc:L8H5P1]	0.111426367	0.654152667	0.5427263	0.000183794
3h	ACA1_301720/clean/scf7180000083222:28463-28525:+	ACA1_301720	Amidohydro-rel domain-containing protein [Source:UniProtKB/TrEMBL;Acc:L8HKQ5]	0.016849497	0.55948	0.542630503	3.15E-11
3h	ACA1_282540/clean/scf7180000084776:706575-706665:-	ACA1_282540	Ribonuclease [Source:UniProtKB/TrEMBL;Acc:L8H8Q0]	0.00636201	0.548814	0.54245199	5.45E-08
3h	ACA1_343310/clean/scf7180000084638:23405-23505:-	ACA1_343310	EF-hand domain-containing protein [Source:UniProtKB/TrEMBL;Acc:L8H9D8]	0.01247722	0.554922	0.54244478	3.50E-06
3h	ACA1_229570/clean/scf7180000084761:280212-280297:-	ACA1_229570	Oxidoreductase, short chain dehydrogenase/reductase superfamily protein [Source:UniProtKB/TrEMBL;Acc:L8H814]	0.000582518	0.542864	0.542281482	1.41E-07
3h	ACA1_145180/clean/scf7180000084710:54542-54614:-	ACA1_145180	F-box domain-containing protein [Source:UniProtKB/TrEMBL;Acc:L8GCU3]	0.007246367	0.549524333	0.542277967	1.28E-05
3h	ACA1_114910/clean/scf7180000084669:582408-582519:-	ACA1_114910	C2 domain containing protein [Source:UniProtKB/TrEMBL;Acc:L8H6P3]	0.0538586	0.596106667	0.542248067	0.000107516
3h	ACA1_185180/clean/scf7180000084746:81927-82011:-	ACA1_185180	LIM zinc-binding domain-containing protein [Source:UniProtKB/TrEMBL;Acc:L8H575]	0.001365461	0.543453667	0.542088206	8.53E-11
3h	ACA1_138470/clean/scf7180000084703:146878-146960:+	ACA1_138470	Ribosomal_L11 domain-containing protein [Source:UniProtKB/TrEMBL;Acc:L8GZJ5]	0.0080771	0.550157667	0.542080567	4.04E-07
3h	ACA1_064400/clean/scf7180000084599:1613130-1613210:-	ACA1_064400	Geranylgeranyl transferase type-2 subunit beta [Source:UniProtKB/TrEMBL;Acc:L8GWR5]	0.041393567	0.583224	0.541830433	1.17E-07
3h	ACA1_252770/clean/scf7180000084768:206448-206539:-	ACA1_252770	Peripla_BP_6 domain-containing protein [Source:UniProtKB/TrEMBL;Acc:L8HAP5]	0.00188888	0.543586667	0.541697787	6.72E-09
3h	ACA1_360020/clean/scf7180000084622:35106-35195:-	ACA1_360020	Mannosyltransferase [Source:UniProtKB/TrEMBL;Acc:L8HCL9]	0.00297619	0.543807333	0.540831143	5.04E-08
3h	ACA1_103160/clean/scf7180000084653:9834-9913:+	ACA1_103160	PALP domain-containing protein [Source:UniProtKB/TrEMBL;Acc:L8GDL9]	0.00752658	0.548098667	0.540572087	1.57E-09
3h	ACA1_162120/clean/scf7180000084726:33650-33739:+	ACA1_162120	hypothetical protein	0.003970633	0.544341333	0.5403707	3.96E-05
3h	ACA1_289750/clean/scf7180000084777:456537-456620:+	ACA1_289750	Antioxidant, AhpC/TSA superfamily protein [Source:UniProtKB/TrEMBL;Acc:L8HJ25]	0.071908433	0.612017333	0.5401089	8.92E-07
3h	ACA1_173790/clean/scf7180000084741:520498-520597:+	ACA1_173790	Nicotinamide-nucleotide adenylyltransferase [Source:UniProtKB/TrEMBL;Acc:L8HK06]	0.01224017	0.552245667	0.540005497	8.23E-08
3h	ACA1_025560/clean/scf7180000084448:14754-14846:-	ACA1_025560	hypothetical protein	0.027777767	0.567688333	0.539910567	0.000618684
3h	ACA1_149210/clean/scf7180000084715:99334-99415:-	ACA1_149210	hypothetical protein	0	0.539688667	0.539688667	1.72E-07
3h	ACA1_243850/clean/scf7180000084763:361546-361624:+	ACA1_243850	Ras-GAP domain-containing protein [Source:UniProtKB/TrEMBL;Acc:L8GJZ5]	0	0.539610333	0.539610333	0.000621372
3h	ACA1_385640/clean/scf7180000084730:221950-222051:+	ACA1_385640	hypothetical protein	0.024809733	0.564318667	0.539508933	5.39E-08
3h	ACA1_212240/clean/scf7180000084758:140808-140882:+	ACA1_212240	Esterase/lipase ( ), putative [Source:UniProtKB/TrEMBL;Acc:L8GQU1]	0.017858303	0.557252	0.539393697	2.07E-08
3h	ACA1_035640/clean/scf7180000084564:101627-101695:-	ACA1_035640	Beta-lactamase domain-containing protein [Source:UniProtKB/TrEMBL;Acc:L8H9P2]	0.055555667	0.594754	0.539198333	0.008430352
3h	ACA1_396170/clean/scf7180000084759:44228-44310:-	ACA1_396170	Phosphatidylserine decarboxylase proenzyme 2 [Source:UniProtKB/TrEMBL;Acc:L8HEJ4]	0.044589567	0.583592667	0.5390031	1.81E-07
3h	ACA1_140510/clean/scf7180000084707:77675-77777:+	ACA1_140510	Fbox domain containing protein [Source:UniProtKB/TrEMBL;Acc:L8GGG0]	0.009202482	0.548060667	0.538858184	5.84E-10
3h	ACA1_167050/clean/scf7180000084736:63243-63304:-	ACA1_167050	Ras subfamily protein [Source:UniProtKB/TrEMBL;Acc:L8H0S4]	0.011904767	0.550746	0.538841233	4.56E-06
3h	ACA1_176150/clean/scf7180000084741:1135023-1135115:+	ACA1_176150	PhytanoylCoA dioxygenase (PhyH) superfamily protein [Source:UniProtKB/TrEMBL;Acc:L8HI91]	0.0198017	0.558627667	0.538825967	2.53E-07
3h	ACA1_209860/clean/scf7180000084757:339630-339718:-	ACA1_209860	Protein kinase domain-containing protein [Source:UniProtKB/TrEMBL;Acc:L8GYD8]	0.006728573	0.545464	0.538735427	2.47E-10
3h	ACA1_227080/clean/scf7180000084761:94749-94826:+	ACA1_227080	Uracil-DNA glycosylase [Source:UniProtKB/TrEMBL;Acc:L8HA23]	0.00413185	0.542789667	0.538657817	2.67E-07
3h	ACA1_089980/clean/scf7180000084639:410662-410733:-	ACA1_089980	hypothetical protein	0.018609733	0.557176667	0.538566933	2.38E-05
3h	ACA1_116850/clean/scf7180000084669:857749-857846:+	ACA1_116850	JmjC domain-containing protein [Source:UniProtKB/TrEMBL;Acc:L8H4R9]	0.003721	0.542145333	0.538424333	3.95E-07
3h	ACA1_230310/clean/scf7180000084761:343386-343487:+	ACA1_230310	PAS domain-containing protein [Source:UniProtKB/TrEMBL;Acc:L8HBA3]	0.013692373	0.551814667	0.538122293	9.74E-08
3h	ACA1_185180/clean/scf7180000084746:81730-81836:-	ACA1_185180	LIM zinc-binding domain-containing protein [Source:UniProtKB/TrEMBL;Acc:L8H575]	0.002038743	0.540116667	0.538077924	9.56E-10
3h	ACA1_156030/clean/scf7180000084723:9117-9204:+	ACA1_156030	hypothetical protein	0.043211767	0.580918333	0.537706567	6.58E-06
3h	ACA1_101470/clean/scf7180000084649:69416-69477:+	ACA1_101470	Dynactin 4 isoform 2, putative [Source:UniProtKB/TrEMBL;Acc:L8GIW5]	0.025163867	0.562831	0.537667133	4.50E-08
3h	ACA1_164170/clean/scf7180000084729:178665-178743:-	ACA1_164170	Rieske domain-containing protein [Source:UniProtKB/TrEMBL;Acc:L8GSJ9]	0.009009	0.546646	0.537637	1.16E-06
3h	ACA1_130600/clean/scf7180000084698:120315-120379:-	ACA1_130600	hypothetical protein	0.0271007	0.564654333	0.537553633	6.15E-07
3h	ACA1_157940/clean/scf7180000084724:155453-155550:-	ACA1_157940	Histidine acid phosphatase superfamily protein [Source:UniProtKB/TrEMBL;Acc:L8H9Z4]	0.006133237	0.543540333	0.537407097	5.62E-11
3h	ACA1_278920/clean/scf7180000084776:275183-275245:+	ACA1_278920	ABC1 domain-containing protein [Source:UniProtKB/TrEMBL;Acc:L8H8Y2]	0.007259533	0.544132333	0.5368728	3.79E-05
3h	ACA1_274900/clean/scf7180000084773:135770-135844:-	ACA1_274900	2,3diketo-5-methylthio-1-phosphopentane phosphatase subfamily protein [Source:UniProtKB/TrEMBL;Acc:L8GFW5]	0.032951833	0.569706333	0.5367545	1.85E-07
3h	ACA1_172050/clean/scf7180000084741:232236-232297:-	ACA1_172050	hypothetical protein	0.0130866	0.54982	0.5367334	2.58E-05
3h	ACA1_065910/clean/scf7180000084599:1945841-1945919:+	ACA1_065910	hypothetical protein	0.077759167	0.614398667	0.5366395	0.000266093
3h	ACA1_306870/clean/scf7180000083668:22707-22798:+	ACA1_306870	hypothetical protein	0.126858667	0.663192333	0.536333667	1.09E-05
3h	ACA1_309360/clean/scf7180000083758:11988-12070:-	ACA1_309360	hypothetical protein	0.030303033	0.566309667	0.536006633	0.00085182
3h	ACA1_080550/clean/scf7180000084618:31513-31572:+	ACA1_080550	FAD binding domain containing protein [Source:UniProtKB/TrEMBL;Acc:L8HBU6]	0.086477867	0.622268	0.535790133	6.56E-05
3h	ACA1_183960/clean/scf7180000084745:528043-528144:-	ACA1_183960	Phosphoesterase family protein [Source:UniProtKB/TrEMBL;Acc:L8H8F6]	0.017659467	0.553026333	0.535366867	2.95E-10
3h	ACA1_097520/clean/scf7180000084645:1266668-1266744:-	ACA1_097520	hypothetical protein	0.005260287	0.540547	0.535286713	3.28E-11
3h	ACA1_382900/clean/scf7180000084721:112168-112290:-	ACA1_382900	ADPribosylation factor, putative [Source:UniProtKB/TrEMBL;Acc:L8GUW5]	0.026538167	0.561765667	0.5352275	1.89E-09
3h	ACA1_040640/clean/scf7180000084572:33164-33262:+	ACA1_040640	MFS domain-containing protein [Source:UniProtKB/TrEMBL;Acc:L8H1W1]	0.024456533	0.559564667	0.535108133	1.06E-06
3h	ACA1_074140/clean/scf7180000084605:35012-35116:+	ACA1_074140	Transporter, major facilitator subfamily protein [Source:UniProtKB/TrEMBL;Acc:L8HK94]	0.000220425	0.535056	0.534835575	2.68E-11
3h	ACA1_037450/clean/scf7180000084567:93782-93845:+	ACA1_037450	NTPase_I-T domain-containing protein [Source:UniProtKB/TrEMBL;Acc:L8H395]	0.159921	0.694698	0.534777	2.49E-05
3h	ACA1_095220/clean/scf7180000084645:815066-815222:-	ACA1_095220	Protein kinase domain-containing protein [Source:UniProtKB/TrEMBL;Acc:L8GIR4]	0.103357267	0.637518	0.534160733	0.000166017
3h	ACA1_108830/clean/scf7180000084661:93198-93283:+	ACA1_108830	Terpene cyclase/mutase family member [Source:UniProtKB/TrEMBL;Acc:L8GD76]	0.006474587	0.540566	0.534091413	6.19E-11
3h	ACA1_326090/clean/scf7180000084504:19588-19712:-	ACA1_326090	hypothetical protein	0.002285473	0.536292667	0.534007194	5.42E-10
3h	ACA1_002620/clean/scf7180000082935:5438-5505:+	ACA1_002620	Fbox domain containing protein [Source:UniProtKB/TrEMBL;Acc:L8HLR9]	0.040552233	0.574549667	0.533997433	1.30E-08
3h	ACA1_063340/clean/scf7180000084599:1390889-1390971:+	ACA1_063340	Dynein intermediate chain, cytosolic, putative [Source:UniProtKB/TrEMBL;Acc:L8GX48]	0.002849003	0.536843	0.533993997	1.16E-08
3h	ACA1_091570/clean/scf7180000084645:152139-152227:+	ACA1_091570	Superoxide dismutase [Source:UniProtKB/TrEMBL;Acc:L8GI64]	0.055068767	0.588721333	0.533652567	3.70E-06
3h	ACA1_041960/clean/scf7180000084573:27222-27304:+	ACA1_041960	Osmotically inducible protein C, putative [Source:UniProtKB/TrEMBL;Acc:L8GUI8]	0.013109107	0.546454667	0.53334556	1.25E-09
3h	ACA1_272530/clean/scf7180000084772:55711-55787:+	ACA1_272530	VWFA domain-containing protein [Source:UniProtKB/TrEMBL;Acc:L8HIL5]	0	0.533145	0.533145	3.68E-09
3h	ACA1_112000/clean/scf7180000084669:80448-80542:+	ACA1_112000	Rho-GAP domain-containing protein [Source:UniProtKB/TrEMBL;Acc:L8H3W7]	0.005198243	0.538086	0.532887757	1.54E-07
3h	ACA1_167050/clean/scf7180000084736:61957-62045:-	ACA1_167050	Ras subfamily protein [Source:UniProtKB/TrEMBL;Acc:L8H0S4]	0.075813633	0.608409333	0.5325957	1.31E-05
3h	ACA1_155460/clean/scf7180000084722:162605-162681:-	ACA1_155460	unspecified product	0.008973417	0.541293667	0.53232025	9.49E-06
3h	ACA1_140730/clean/scf7180000084707:109513-109699:-	ACA1_140730	Kelch repeat protein [Source:UniProtKB/TrEMBL;Acc:L8GGS1]	0.1114641	0.64364	0.5321759	0.00023624
3h	ACA1_123630/clean/scf7180000084686:46779-46850:-	ACA1_123630	hypothetical protein	0.0769353	0.608934	0.5319987	2.47E-05
3h	ACA1_170960/clean/scf7180000084741:23197-23344:+	ACA1_170960	hypothetical protein	0.029842667	0.561623667	0.531781	3.04E-06
3h	ACA1_037780/clean/scf7180000084568:24668-24779:+	ACA1_037780	RhoGEF domain containing protein [Source:UniProtKB/TrEMBL;Acc:L8GN17]	0.00858316	0.540235333	0.531652173	9.36E-08
3h	ACA1_279050/clean/scf7180000084776:301937-302029:-	ACA1_279050	PCI domain-containing protein [Source:UniProtKB/TrEMBL;Acc:L8H5Y4]	0.006953797	0.538403	0.531449203	4.48E-12
3h	ACA1_253360/clean/scf7180000084768:284669-284796:+	ACA1_253360	TP_methylase domain-containing protein [Source:UniProtKB/TrEMBL;Acc:L8HAX0]	0.022418067	0.553689667	0.5312716	1.16E-08
3h	ACA1_388770/clean/scf7180000084733:165843-165909:+	ACA1_388770	Poly(A) polymerase central domain containing protein [Source:UniProtKB/TrEMBL;Acc:L8GG84]	0.192801333	0.723347333	0.530546	0.000874315
3h	ACA1_110100/clean/scf7180000084663:63149-63339:-	ACA1_110100	Protein kinase [Source:UniProtKB/TrEMBL;Acc:L8HK05]	0.010192117	0.540566	0.530373883	2.33E-06
3h	ACA1_264550/clean/scf7180000084770:318285-318375:+	ACA1_264550	Cys/Met metabolism PLPdependent enzyme superfamily protein [Source:UniProtKB/TrEMBL;Acc:L8H496]	0.048596167	0.578902667	0.5303065	4.74E-07
3h	ACA1_077100/clean/scf7180000084612:235899-235985:-	ACA1_077100	Rab1/RabDfamily small GTPase [Source:UniProtKB/TrEMBL;Acc:L8GNN9]	0.018151967	0.548156667	0.5300047	1.72E-07
3h	ACA1_323520/clean/scf7180000084468:41386-41442:-	ACA1_323520	Inosineuridine preferring nucleoside hydrolase [Source:UniProtKB/TrEMBL;Acc:L8H334]	0.0227441	0.552713333	0.529969233	8.27E-07
3h	ACA1_391320/clean/scf7180000084740:152440-152522:-	ACA1_391320	Rab-GAP TBC domain-containing protein [Source:UniProtKB/TrEMBL;Acc:L8GNV7]	0.012283753	0.542089333	0.52980558	4.17E-08
3h	ACA1_263890/clean/scf7180000084770:127106-127201:-	ACA1_263890	Methyltransf_11 domain-containing protein [Source:UniProtKB/TrEMBL;Acc:L8H151]	0.427979	0.957720333	0.529741333	3.05E-11
3h	ACA1_142340/clean/scf7180000084708:141607-141725:+	ACA1_142340	unspecified product	0.0631581	0.592512667	0.529354567	5.50E-06
3h	ACA1_037190/clean/scf7180000084567:80442-80529:+	ACA1_037190	Aminotran_5 domain-containing protein [Source:UniProtKB/TrEMBL;Acc:L8H0V4]	0.0405687	0.569774	0.5292053	7.26E-09
3h	ACA1_018950/clean/scf7180000083912:33190-33281:-	ACA1_018950	Lipoprotein, putative [Source:UniProtKB/TrEMBL;Acc:L8HM57]	0.0148148	0.543607667	0.528792867	1.83E-05
3h	ACA1_063340/clean/scf7180000084599:1391167-1391249:+	ACA1_063340	Dynein intermediate chain, cytosolic, putative [Source:UniProtKB/TrEMBL;Acc:L8GX48]	0.001801063	0.530431333	0.52863027	4.40E-10
3h	ACA1_309500/clean/scf7180000083762:8572-8655:+	ACA1_309500	Pyridine nucleotidedisulfide oxidoreductase domain containing protein [Source:UniProtKB/TrEMBL;Acc:L8GI23]	0.002179207	0.530626667	0.52844746	1.86E-10
3h	ACA1_174040/clean/scf7180000084741:585390-585506:-	ACA1_174040	Protein kinase domain-containing protein [Source:UniProtKB/TrEMBL;Acc:L8HK27]	0.005712757	0.533997333	0.528284577	5.13E-08
3h	ACA1_232420/clean/scf7180000084762:22374-22498:+	ACA1_232420	TPR_REGION domain-containing protein [Source:UniProtKB/TrEMBL;Acc:L8H3B0]	0.072947	0.600995	0.528048	1.40E-06
3h	ACA1_210400/clean/scf7180000084758:39920-40009:-	ACA1_210400	PPM-type phosphatase domain-containing protein [Source:UniProtKB/TrEMBL;Acc:L8GQQ0]	0.016853527	0.544775667	0.52792214	2.39E-08
3h	ACA1_321440/clean/scf7180000084439:30739-30833:+	ACA1_321440	hypothetical protein	0.001226577	0.528998	0.527771423	2.94E-10
3h	ACA1_392390/clean/scf7180000084740:269368-269474:+	ACA1_392390	Rfwd1prov protein [Source:UniProtKB/TrEMBL;Acc:L8GPG4]	0.00481485	0.532161	0.52734615	1.01E-06
3h	ACA1_383560/clean/scf7180000084721:211551-211637:-	ACA1_383560	TBC domain containing protein [Source:UniProtKB/TrEMBL;Acc:L8GW44]	0.007246639	0.534005333	0.526758694	4.43E-06
3h	ACA1_063780/clean/scf7180000084599:1500678-1500777:+	ACA1_063780	Nucleotidyltransferase domain containing protein [Source:UniProtKB/TrEMBL;Acc:L8GWN7]	0.003267973	0.529636333	0.52636836	6.64E-08
3h	ACA1_397180/clean/scf7180000084759:165084-165169:-	ACA1_397180	hypothetical protein	0.001230013	0.527178	0.525947987	3.05E-06
3h	ACA1_199750/clean/scf7180000084753:319007-319075:-	ACA1_199750	hypothetical protein	0.007229613	0.532667	0.525437387	6.14E-09
3h	ACA1_067410/clean/scf7180000084601:17627-17716:-	ACA1_067410	F-box domain-containing protein [Source:UniProtKB/TrEMBL;Acc:L8GTP9]	0.038824333	0.563989333	0.525165	4.81E-05
3h	ACA1_396220/clean/scf7180000084759:56800-56883:+	ACA1_396220	Acyl-coenzyme A oxidase [Source:UniProtKB/TrEMBL;Acc:L8HEJ7]	0.000253627	0.525385	0.525131373	5.13E-11
3h	ACA1_183960/clean/scf7180000084745:527800-527901:-	ACA1_183960	Phosphoesterase family protein [Source:UniProtKB/TrEMBL;Acc:L8H8F6]	0.022280367	0.546959333	0.524678967	3.00E-08
3h	ACA1_218390/clean/scf7180000084758:651851-651986:-	ACA1_218390	Glycosyl hydrolase family 20, catalytic domain containing protein [Source:UniProtKB/TrEMBL;Acc:L8GQD2]	0	0.524661	0.524661	6.93E-08
3h	ACA1_072370/clean/scf7180000084603:1212767-1212852:-	ACA1_072370	Leucine rich repeat domain containing protein [Source:UniProtKB/TrEMBL;Acc:L8HEP0]	0.004939933	0.529290333	0.5243504	1.55E-05
3h	ACA1_144100/clean/scf7180000084709:150917-151010:-	ACA1_144100	hypothetical protein	0.0480668	0.571960667	0.523893867	4.32E-08
3h	ACA1_197490/clean/scf7180000084753:25481-25566:+	ACA1_197490	hypothetical protein	0.0267538	0.550579	0.5238252	2.98E-07
3h	ACA1_287950/clean/scf7180000084777:131726-131837:-	ACA1_287950	hypothetical protein	0.010940213	0.534442667	0.523502453	9.41E-07
3h	ACA1_182400/clean/scf7180000084745:204872-204961:+	ACA1_182400	Integral membrane protein [Source:UniProtKB/TrEMBL;Acc:L8H9C3]	0.00446285	0.527924	0.52346115	1.43E-10
3h	ACA1_351920/clean/scf7180000084695:35007-35076:+	ACA1_351920	VWFA domain-containing protein [Source:UniProtKB/TrEMBL;Acc:L8GE58]	0.048122667	0.571505667	0.523383	1.04E-06
3h	ACA1_066040/clean/scf7180000084599:1980510-1980578:-	ACA1_066040	hypothetical protein	0.004255333	0.527637	0.523381667	0.000366738
3h	ACA1_182370/clean/scf7180000084745:198115-198265:-	ACA1_182370	ANK_REP_REGION domain-containing protein [Source:UniProtKB/TrEMBL;Acc:L8H846]	0.018286067	0.541523333	0.523237267	1.38E-09
3h	ACA1_261270/clean/scf7180000084769:625859-625926:+	ACA1_261270	Methyltransferase domain containing protein [Source:UniProtKB/TrEMBL;Acc:L8GG55]	0.0634112	0.586630667	0.523219467	2.07E-08
3h	ACA1_209860/clean/scf7180000084757:341281-341374:-	ACA1_209860	Protein kinase domain-containing protein [Source:UniProtKB/TrEMBL;Acc:L8GYD8]	0.01463481	0.537811667	0.523176857	1.99E-07
3h	ACA1_106290/clean/scf7180000084660:76899-76971:+	ACA1_106290	WD_REPEATS_REGION domain-containing protein [Source:UniProtKB/TrEMBL;Acc:L8GNF9]	0.156809	0.67997	0.523161	0.000488332
3h	ACA1_204620/clean/scf7180000084754:323173-323245:+	ACA1_204620	hypothetical protein	0.023696767	0.546754	0.523057233	2.17E-05
3h	ACA1_390770/clean/scf7180000084740:90705-90770:-	ACA1_390770	DephosphoCoA kinase [Source:UniProtKB/TrEMBL;Acc:L8GP39]	0.233893333	0.756923333	0.52303	0.000100542
3h	ACA1_140770/clean/scf7180000084707:117224-117327:+	ACA1_140770	ATPase, AAA domain containing protein [Source:UniProtKB/TrEMBL;Acc:L8GJ22]	0.015504403	0.538386667	0.522882263	6.33E-07
3h	ACA1_174540/clean/scf7180000084741:732266-732350:-	ACA1_174540	Fucosylgalactoside 3-alpha-galactosyltransferase [Source:UniProtKB/TrEMBL;Acc:L8HJC5]	0.0504674	0.573252667	0.522785267	9.96E-06
3h	ACA1_034150/clean/scf7180000084561:35650-35723:-	ACA1_034150	hypothetical protein	0.031414867	0.554111	0.522696133	1.86E-07
3h	ACA1_052350/clean/scf7180000084594:51561-51635:-	ACA1_052350	SKA2 domain-containing protein [Source:UniProtKB/TrEMBL;Acc:L8H7V1]	0.076066533	0.5986	0.522533467	0.000845597
3h	ACA1_297530/clean/scf7180000082850:1045-1126:-	ACA1_297530	hypothetical protein	0.0197296	0.542225	0.5224954	1.82E-08
3h	ACA1_152970/clean/scf7180000084720:43703-43833:-	ACA1_152970	SET family protein [Source:UniProtKB/TrEMBL;Acc:L8HHE2]	0.0250123	0.547474333	0.522462033	2.29E-09
3h	ACA1_118750/clean/scf7180000084671:18436-18544:-	ACA1_118750	Threonylcarbamoyl-AMP synthase [Source:UniProtKB/TrEMBL;Acc:L8GLU7]	0.035818833	0.557851667	0.522032833	0.000101079
3h	ACA1_071240/clean/scf7180000084603:895763-895894:-	ACA1_071240	Transporter, major facilitator subfamily protein [Source:UniProtKB/TrEMBL;Acc:L8HFI7]	0.007971167	0.529966667	0.5219955	5.44E-07
3h	ACA1_139830/clean/scf7180000084707:6998-7101:-	ACA1_139830	Eukaryotic translation initiation factor 3 subunit L [Source:UniProtKB/TrEMBL;Acc:L8GIZ4]	0.000454259	0.522272667	0.521818407	9.04E-11
3h	ACA1_271720/clean/scf7180000084771:91574-91677:-	ACA1_271720	Ras-GAP domain-containing protein [Source:UniProtKB/TrEMBL;Acc:L8GR76]	0.005619558	0.527094667	0.521475108	7.98E-07
3h	ACA1_136170/clean/scf7180000084702:144381-144480:+	ACA1_136170	Cofilin/tropomyosin-type actin-binding protein [Source:UniProtKB/TrEMBL;Acc:L8GGT0]	0.00880307	0.529452	0.52064893	4.68E-10
3h	ACA1_298660/clean/scf7180000082992:15906-16011:+	ACA1_298660	Bromo domain-containing protein [Source:UniProtKB/TrEMBL;Acc:L8H1K3]	0	0.520568333	0.520568333	4.85E-09
3h	ACA1_192970/clean/scf7180000084749:173824-173907:-	ACA1_192970	Basic helixloop-helix (BHLH) family protein [Source:UniProtKB/TrEMBL;Acc:L8GNQ5]	0.106582267	0.627059	0.520476733	0.000800927
3h	ACA1_038340/clean/scf7180000084568:125612-125702:-	ACA1_038340	Ras protein activator like 1, putative [Source:UniProtKB/TrEMBL;Acc:L8GLF6]	0.0138889	0.534346333	0.520457433	0.000141934
3h	ACA1_114840/clean/scf7180000084669:559497-559603:+	ACA1_114840	Serine/threonine-protein phosphatase [Source:UniProtKB/TrEMBL;Acc:L8H3P4]	0.0357821	0.556223333	0.520441233	4.67E-06
3h	ACA1_097540/clean/scf7180000084645:1277344-1277416:-	ACA1_097540	Serine/threonine kinase [Source:UniProtKB/TrEMBL;Acc:L8GK27]	0.00793227	0.528294	0.52036173	3.89E-07
3h	ACA1_023890/clean/scf7180000084436:31989-32101:-	ACA1_023890	SAM domain-containing protein [Source:UniProtKB/TrEMBL;Acc:L8GSK7]	0.016171263	0.536458333	0.52028707	1.50E-09
3h	ACA1_336100/clean/scf7180000084593:24873-24958:-	ACA1_336100	Ribosomal protein L12, putative [Source:UniProtKB/TrEMBL;Acc:L8H1R0]	0.0050505	0.525257667	0.520207167	3.43E-05
3h	ACA1_154840/clean/scf7180000084722:94082-94159:+	ACA1_154840	Protein kinase domain-containing protein [Source:UniProtKB/TrEMBL;Acc:L8H1C2]	0.112366033	0.632497333	0.5201313	3.18E-05
3h	ACA1_024220/clean/scf7180000084441:20702-20769:+	ACA1_024220	Purinergic receptor, putative [Source:UniProtKB/TrEMBL;Acc:L8GLB7]	0.003780267	0.523469	0.519688733	2.66E-11
3h	ACA1_374110/clean/scf7180000084679:227556-227651:+	ACA1_374110	Protein kinase domain-containing protein [Source:UniProtKB/TrEMBL;Acc:L8GHL2]	0.011858083	0.531452	0.519593917	3.00E-07
3h	ACA1_252200/clean/scf7180000084768:142514-142612:+	ACA1_252200	Saposin, putative [Source:UniProtKB/TrEMBL;Acc:L8HD22]	0.015639067	0.535006333	0.519367267	5.62E-10

3h	ACA1_331800/clean/scf7180000084578:33444-33574:-	ACA1_331800	hypothetical protein	0	0.519167	0.519167	1.15E-06
3h	ACA1_027030/clean/scf7180000084464:27743-27840:-	ACA1_027030	C2 domain-containing protein [Source:UniProtKB/TrEMBL;Acc:L8GJ80]	0.014112686	0.533218	0.519105314	0.000249353
3h	ACA1_207320/clean/scf7180000084757:53528-53597:+	ACA1_207320	Acyltransferase [Source:UniProtKB/TrEMBL;Acc:L8GY38]	0.023883043	0.542957	0.519073957	0.000240519
3h	ACA1_063800/clean/scf7180000084599:1507318-1507419:-	ACA1_063800	B30.2/SPRY domain-containing protein [Source:UniProtKB/TrEMBL;Acc:L8GZV1]	0.006679853	0.525414333	0.51873448	1.41E-07
3h	ACA1_069190/clean/scf7180000084603:379154-379241:+	ACA1_069190	PfkB domain-containing protein [Source:UniProtKB/TrEMBL;Acc:L8HDR4]	0.036927367	0.555558333	0.518630967	1.54E-07
3h	ACA1_272530/clean/scf7180000084772:54571-54739:+	ACA1_272530	VWFA domain-containing protein [Source:UniProtKB/TrEMBL;Acc:L8HIL5]	0.031440533	0.549847	0.518406467	2.73E-06
3h	ACA1_258230/clean/scf7180000084769:203429-203488:-	ACA1_258230	DNA ligase [Source:UniProtKB/TrEMBL;Acc:L8GF13]	0.002472657	0.520560333	0.518087677	3.84E-07
3h	ACA1_165600/clean/scf7180000084732:61708-61785:-	ACA1_165600	VWFA domain-containing protein [Source:UniProtKB/TrEMBL;Acc:L8HGQ3]	0.015286513	0.533197	0.517910487	4.59E-07
3h	ACA1_279360/clean/scf7180000084776:371693-371765:-	ACA1_279360	hypothetical protein	0.004290867	0.522142	0.517851133	1.05E-06
3h	ACA1_264230/clean/scf7180000084770:216003-216090:+	ACA1_264230	ATP/GTPbinding-like protein [Source:UniProtKB/TrEMBL;Acc:L8H212]	0.012869573	0.530576	0.517706427	6.68E-08
3h	ACA1_250310/clean/scf7180000084767:47140-47204:-	ACA1_250310	Serine/threonine protein kinase [Source:UniProtKB/TrEMBL;Acc:L8HD37]	0.029434533	0.546826333	0.5173918	2.07E-05
3h	ACA1_018140/clean/scf7180000083885:16722-16811:-	ACA1_018140	Leucine-tRNA ligase [Source:UniProtKB/TrEMBL;Acc:L8GL00]	0.014795833	0.532092333	0.5172965	1.18E-05
3h	ACA1_174050/clean/scf7180000084741:588559-588720:-	ACA1_174050	hypothetical protein	0.00578173	0.522903333	0.517121603	5.09E-07
3h	ACA1_271290/clean/scf7180000084771:12743-12808:-	ACA1_271290	hypothetical protein	0.011733177	0.528654667	0.51692149	6.51E-11
3h	ACA1_138470/clean/scf7180000084703:147254-147322:+	ACA1_138470	Ribosomal_L11 domain-containing protein [Source:UniProtKB/TrEMBL;Acc:L8GZJ5]	0.0657704	0.582675	0.5169046	0.000283014
3h	ACA1_015490/clean/scf7180000083799:3872-3948:+	ACA1_015490	hypothetical protein	0.010274323	0.527073333	0.51679901	3.16E-07
3h	ACA1_091900/clean/scf7180000084645:223935-224032:-	ACA1_091900	Acylphosphatase [Source:UniProtKB/TrEMBL;Acc:L8GHY8]	0.018176143	0.534936333	0.51676019	5.59E-06
3h	ACA1_095260/clean/scf7180000084645:824660-824750:-	ACA1_095260	F-box domain-containing protein [Source:UniProtKB/TrEMBL;Acc:L8GL23]	0.004109477	0.520679	0.516569523	2.54E-07
3h	ACA1_171860/clean/scf7180000084741:179773-179861:+	ACA1_171860	POT family protein [Source:UniProtKB/TrEMBL;Acc:L8HHD5]	0	0.516478667	0.516478667	3.35E-07
3h	ACA1_265420/clean/scf7180000084770:465875-465937:+	ACA1_265420	hypothetical protein	0.042781967	0.558846333	0.516064367	0.000124895
3h	ACA1_269380/clean/scf7180000084770:991502-991635:+	ACA1_269380	hypothetical protein	0.0285307	0.544505667	0.515974967	2.24E-06
3h	ACA1_279550/clean/scf7180000084776:412669-412785:-	ACA1_279550	CBS domain containing protein [Source:UniProtKB/TrEMBL;Acc:L8H8C1]	0.018721867	0.534628	0.515906133	2.38E-07
3h	ACA1_094910/clean/scf7180000084645:789055-789264:-	ACA1_094910	Deoxyhypusine synthase [Source:UniProtKB/TrEMBL;Acc:L8GL08]	0.018698947	0.534361667	0.51566272	1.31E-06
3h	ACA1_200200/clean/scf7180000084753:413201-413324:-	ACA1_200200	Asp_Arg_Hydrox domain-containing protein [Source:UniProtKB/TrEMBL;Acc:L8H4R6]	0.013140477	0.528394	0.515253523	1.28E-07
3h	ACA1_219060/clean/scf7180000084758:708330-708474:-	ACA1_219060	DWNN domain containing protein [Source:UniProtKB/TrEMBL;Acc:L8GSX0]	0.152823	0.667679667	0.514856667	0.003139662
3h	ACA1_223350/clean/scf7180000084760:138588-138666:+	ACA1_223350	F-box domain-containing protein [Source:UniProtKB/TrEMBL;Acc:L8GSX8]	0.0411811	0.555640667	0.514459567	6.86E-07
3h	ACA1_078020/clean/scf7180000084614:73985-74101:-	ACA1_078020	AlbA_2 domain-containing protein [Source:UniProtKB/TrEMBL;Acc:L8GR16]	0.105338467	0.619716	0.514377533	0.000925769
3h	ACA1_328770/clean/scf7180000084560:26515-26601:-	ACA1_328770	Aldo_ket_red domain-containing protein [Source:UniProtKB/TrEMBL;Acc:L8GQZ7]	0.003151513	0.51703	0.513878487	1.24E-08
3h	ACA1_301860/clean/scf7180000083222:43938-44030:+	ACA1_301860	Phosphatidylinositol 3-and 4-kinase [Source:UniProtKB/TrEMBL;Acc:L8HHB0]	0.010449383	0.52425	0.513800617	3.18E-12
3h	ACA1_377980/clean/scf7180000084711:155850-155930:+	ACA1_377980	Ubiquitin-like domain-containing protein [Source:UniProtKB/TrEMBL;Acc:L8GRZ4]	0.003538677	0.517162667	0.51362399	8.81E-09
3h	ACA1_269320/clean/scf7180000084770:971302-971371:-	ACA1_269320	eIF2 gamma, putative [Source:UniProtKB/TrEMBL;Acc:L8H4Y2]	0.029720267	0.543247333	0.513527067	2.90E-08
3h	ACA1_097210/clean/scf7180000084645:1184782-1184948:-	ACA1_097210	Ubiquitin-like domain-containing protein [Source:UniProtKB/TrEMBL;Acc:L8GJ53]	0.026497133	0.540003667	0.513506533	6.99E-06
3h	ACA1_208320/clean/scf7180000084757:161025-161086:-	ACA1_208320	ADPribosylglycohydrolase superfamily protein [Source:UniProtKB/TrEMBL;Acc:L8GXF3]	0.020911933	0.534191333	0.5132794	0.008821377
3h	ACA1_301560/clean/scf7180000083222:9836-9899:+	ACA1_301560	PH domain-containing protein [Source:UniProtKB/TrEMBL;Acc:L8HID1]	0.0923733	0.605388333	0.513015033	0.000686569
3h	ACA1_296550/clean/scf7180000084777:1380790-1380880:+	ACA1_296550	hypothetical protein	0.006674997	0.519649333	0.512974337	1.68E-06
3h	ACA1_364620/clean/scf7180000084641:158594-158680:-	ACA1_364620	Palmitoyl protein thioesterase [Source:UniProtKB/TrEMBL;Acc:L8GM75]	0.035087667	0.547951667	0.512864	4.53E-05
3h	ACA1_106330/clean/scf7180000084660:86277-86363:-	ACA1_106330	Methyltransf_11 domain-containing protein [Source:UniProtKB/TrEMBL;Acc:L8GMT5]	0.016294673	0.529065333	0.51277066	7.75E-08
3h	ACA1_189640/clean/scf7180000084748:71362-71558:-	ACA1_189640	Bifunctional aspartate kinase/diaminopimelate decarboxylase protein [Source:UniProtKB/TrEMBL;Acc:L8GEY3]	0.025913	0.538439	0.512526	1.98E-08
3h	ACA1_149380/clean/scf7180000084715:139069-139180:+	ACA1_149380	Ribose 5phosphate isomerase A, putative [Source:UniProtKB/TrEMBL;Acc:L8HC48]	0.029397967	0.541816667	0.5124187	1.24E-07
3h	ACA1_054970/clean/scf7180000084594:615862-615946:-	ACA1_054970	FYVE-type domain-containing protein [Source:UniProtKB/TrEMBL;Acc:L8H8G2]	0.001866367	0.514073333	0.512206967	1.89E-05
3h	ACA1_341100/clean/scf7180000084620:50566-50665:-	ACA1_341100	MFS domain-containing protein [Source:UniProtKB/TrEMBL;Acc:L8GR49]	0.005565743	0.517467	0.511901257	7.73E-09
3h	ACA1_088900/clean/scf7180000084639:256770-256856:-	ACA1_088900	hypothetical protein	0.0034547	0.515202	0.5117473	1.47E-05
3h	ACA1_306850/clean/scf7180000083668:20589-20677:+	ACA1_306850	RanBPMrelated, putative [Source:UniProtKB/TrEMBL;Acc:L8GNY2]	0.003720493	0.515234	0.511513507	1.73E-08
3h	ACA1_173170/clean/scf7180000084741:418037-418176:-	ACA1_173170	SNF2 family Nterminal domain containing protein [Source:UniProtKB/TrEMBL;Acc:L8HGM5]	0.001246933	0.512638333	0.5113914	2.44E-08
3h	ACA1_142580/clean/scf7180000084708:186949-187071:-	ACA1_142580	unspecified product	0.0872114	0.598385667	0.511174267	3.04E-05
3h	ACA1_380750/clean/scf7180000084712:80815-80899:-	ACA1_380750	hypothetical protein	0.035366633	0.546057667	0.510691033	1.93E-06
3h	ACA1_192720/clean/scf7180000084749:165506-165635:-	ACA1_192720	ANK_REP_REGION domain-containing protein [Source:UniProtKB/TrEMBL;Acc:L8GNQ4]	0.260865333	0.771433333	0.510568	0.019014615
3h	ACA1_199500/clean/scf7180000084753:248169-248278:+	ACA1_199500	1acylglycerophosphocholine O-acyltransferase 1, putative [Source:UniProtKB/TrEMBL;Acc:L8H4L3]	0.005929967	0.515979333	0.510049367	0.000696052
3h	ACA1_230520/clean/scf7180000084761:371251-371345:-	ACA1_230520	Hypothetical protein	0.003383887	0.513366667	0.509982779	3.00E-08
3h	ACA1_203000/clean/scf7180000084754:114012-114109:+	ACA1_203000	PGM1_C domain-containing protein [Source:UniProtKB/TrEMBL;Acc:L8GW29]	0.014921167	0.524428	0.509506833	3.82E-05
3h	ACA1_156030/clean/scf7180000084723:6173-6256:+	ACA1_156030	hypothetical protein	0	0.509504667	0.509504667	7.80E-08
3h	ACA1_264270/clean/scf7180000084770:226686-226775:-	ACA1_264270	Oxidoreductase, short chain dehydrogenase/reductase family protein [Source:UniProtKB/TrEMBL;Acc:L8H474]	0.009426103	0.518897667	0.509471563	3.43E-08
3h	ACA1_245510/clean/scf7180000084763:498620-498736:+	ACA1_245510	SAM domain (Sterile alpha motif) domain containing protein [Source:UniProtKB/TrEMBL;Acc:L8GKU3]	0.000376933	0.509732	0.509355067	1.44E-06
3h	ACA1_035420/clean/scf7180000084564:45465-45575:-	ACA1_035420	Carrier superfamily protein [Source:UniProtKB/TrEMBL;Acc:L8HAE9]	0.008532843	0.517836	0.509303157	0.001188257
3h	ACA1_277590/clean/scf7180000084776:82511-82638:-	ACA1_277590	Peptidase_S66 domain-containing protein [Source:UniProtKB/TrEMBL;Acc:L8H6R2]	0.0209656	0.530184	0.5092184	2.68E-11
3h	ACA1_201010/clean/scf7180000084753:462387-462451:+	ACA1_201010	Fe2OG dioxygenase domain-containing protein [Source:UniProtKB/TrEMBL;Acc:L8H2K6]	0.103152667	0.612256	0.509103333	0.000446636
3h	ACA1_145180/clean/scf7180000084710:53858-53921:-	ACA1_145180	F-box domain-containing protein [Source:UniProtKB/TrEMBL;Acc:L8GCU3]	0.044389733	0.553445	0.509055267	0.000273988
3h	ACA1_054370/clean/scf7180000084594:474673-474777:-	ACA1_054370	Fe2OG dioxygenase domain-containing protein [Source:UniProtKB/TrEMBL;Acc:L8H8A8]	0.193422	0.702403667	0.508981667	6.14E-05
3h	ACA1_063800/clean/scf7180000084599:1506577-1506664:-	ACA1_063800	B30.2/SPRY domain-containing protein [Source:UniProtKB/TrEMBL;Acc:L8GZV1]	0.01568589	0.524324667	0.508638777	6.47E-06
3h	ACA1_218470/clean/scf7180000084758:663155-663259:-	ACA1_218470	Rasrelated protein Rab-21, putative [Source:UniProtKB/TrEMBL;Acc:L8GRC8]	0.00762423	0.515891333	0.508267103	2.46E-10
3h	ACA1_069910/clean/scf7180000084603:597875-597981:+	ACA1_069910	Citrin, putative [Source:UniProtKB/TrEMBL;Acc:L8HG30]	0.0297334	0.537799333	0.508065933	1.89E-07
3h	ACA1_072690/clean/scf7180000084603:1254859-1254947:+	ACA1_072690	Signal peptide peptidase [Source:UniProtKB/TrEMBL;Acc:L8HGU4]	0.012131077	0.520001667	0.50787059	8.92E-07
3h	ACA1_167050/clean/scf7180000084736:61752-61810:-	ACA1_167050	Ras subfamily protein [Source:UniProtKB/TrEMBL;Acc:L8H0S4]	0.0547814	0.562253333	0.507471933	2.24E-05
3h	ACA1_043190/clean/scf7180000084573:137070-137138:-	ACA1_043190	hypothetical protein	0.035997367	0.543406	0.507408633	1.38E-09
3h	ACA1_203940/clean/scf7180000084754:227645-227726:+	ACA1_203940	hypothetical protein	0.0954884	0.602868	0.5073796	1.08E-05
3h	ACA1_245940/clean/scf7180000084763:529382-529479:+	ACA1_245940	hypothetical protein	0.016257477	0.523439	0.507181523	1.39E-07
3h	ACA1_082750/clean/scf7180000084625:9212-9308:+	ACA1_082750	N-acetyltransferase domain-containing protein [Source:UniProtKB/TrEMBL;Acc:L8GSP4]	0.0530757	0.560257	0.5071813	8.78E-08
3h	ACA1_384530/clean/scf7180000084730:41638-41784:+	ACA1_384530	hypothetical protein	0.009493967	0.516611667	0.5071177	1.68E-07
3h	ACA1_252170/clean/scf7180000084768:136887-136950:-	ACA1_252170	Ras subfamily protein [Source:UniProtKB/TrEMBL;Acc:L8HC26]	0.029906633	0.536927333	0.5070207	5.20E-07
3h	ACA1_113560/clean/scf7180000084669:321431-321591:-	ACA1_113560	PH domain containing protein [Source:UniProtKB/TrEMBL;Acc:L8H6B7]	0.035268733	0.54216	0.506891267	8.54E-08
3h	ACA1_174620/clean/scf7180000084741:750292-750421:-	ACA1_174620	Late embryogenesis abundant protein [Source:UniProtKB/TrEMBL;Acc:L8HK68]	0.001938803	0.508790667	0.506851863	8.85E-08
3h	ACA1_100470/clean/scf7180000084648:91887-91949:-	ACA1_100470	ABC transmembrane type-1 domain-containing protein [Source:UniProtKB/TrEMBL;Acc:L8GJH4]	0.0520957	0.558908333	0.506812633	0.007794252
3h	ACA1_237880/clean/scf7180000084763:38355-38479:-	ACA1_237880	ICAT domain-containing protein [Source:UniProtKB/TrEMBL;Acc:L8GJP4]	0.007205817	0.513494	0.506288183	2.53E-11
3h	ACA1_278100/clean/scf7180000084776:171536-171633:+	ACA1_278100	F-box domain-containing protein [Source:UniProtKB/TrEMBL;Acc:L8H5T0]	0.172211533	0.678415333	0.5062038	6.11E-05
3h	ACA1_093570/clean/scf7180000084645:582645-582733:-	ACA1_093570	hypothetical protein	0.0241153	0.530111333	0.505996033	4.57E-06
3h	ACA1_273880/clean/scf7180000084773:77231-77320:-	ACA1_273880	Peptidase_S8 domain-containing protein [Source:UniProtKB/TrEMBL;Acc:L8GI59]	0.032032567	0.537953667	0.5059211	2.78E-05
3h	ACA1_123630/clean/scf7180000084686:46279-46384:-	ACA1_123630	hypothetical protein	0.120403333	0.626139333	0.505736	5.69E-05
3h	ACA1_290910/clean/scf7180000084777:699306-699418:+	ACA1_290910	ATPase ASNA1 homolog [Source:UniProtKB/TrEMBL;Acc:L8HKV9]	0.009714937	0.515396667	0.50568173	1.22E-09
3h	ACA1_173790/clean/scf7180000084741:519853-519992:+	ACA1_173790	Nicotinamide-nucleotide adenylyltransferase [Source:UniProtKB/TrEMBL;Acc:L8HK06]	0.001009573	0.506366	0.505356427	2.96E-08
3h	ACA1_245500/clean/scf7180000084763:496605-496699:+	ACA1_245500	Fe2OG dioxygenase domain-containing protein [Source:UniProtKB/TrEMBL;Acc:L8GL61]	0.003628833	0.508913333	0.5052845	1.56E-06
3h	ACA1_046390/clean/scf7180000084577:84544-84655:-	ACA1_046390	hypothetical protein	0.03089254	0.536146667	0.505254127	7.51E-05
3h	ACA1_116270/clean/scf7180000084669:743933-744008:+	ACA1_116270	G_PROTEIN_RECEP_F1_2 domain-containing protein [Source:UniProtKB/TrEMBL;Acc:L8H6T9]	0	0.505155567	0.505155567	3.01E-07
3h	ACA1_157850/clean/scf7180000084724:129437-129512:-	ACA1_157850	Endo/exonuclease/phosphatase domain-containing protein [Source:UniProtKB/TrEMBL;Acc:L8HBE3]	0.0396638	0.544685667	0.505021867	7.69E-06
3h	ACA1_216300/clean/scf7180000084758:463728-463951:+	ACA1_216300	Ras family protein [Source:UniProtKB/TrEMBL;Acc:L8GR60]	0.000663777	0.505637333	0.504973557	8.61E-07
3h	ACA1_130600/clean/scf7180000084698:119851-119924:-	ACA1_130600	hypothetical protein	0.020848177	0.525508333	0.504660157	8.26E-06
3h	ACA1_384800/clean/scf7180000084730:106158-106257:+	ACA1_384800	hypothetical protein	0.01515739	0.519747333	0.504589943	7.34E-07
3h	ACA1_321180/clean/scf7180000084437:7343-7447:+	ACA1_321180	PH domain containing protein [Source:UniProtKB/TrEMBL;Acc:L8GIK9]	0.017819867	0.522365333	0.504545467	1.98E-06
3h	ACA1_255500/clean/scf7180000084768:515873-516003:+	ACA1_255500	Peptidase C83 domain-containing protein [Source:UniProtKB/TrEMBL;Acc:L8HB52]	0.014044037	0.518500333	0.504456297	1.53E-06
3h	ACA1_289870/clean/scf7180000084777:484343-484438:+	ACA1_289870	Helicase ATP-binding domain-containing protein [Source:UniProtKB/TrEMBL;Acc:L8HI29]	0.009803933	0.513906	0.504102067	3.43E-05
3h	ACA1_140610/clean/scf7180000084707:100951-101047:-	ACA1_140610	Poly [ADP-ribose] polymerase [Source:UniProtKB/TrEMBL;Acc:L8GGG7]	0	0.504051033	0.504051033	0.002139342
3h	ACA1_305680/clean/scf7180000083609:29380-29471:+	ACA1_305680	hypothetical protein	0.013614117	0.517559333	0.503945217	1.23E-10
3h	ACA1_069380/clean/scf7180000084603:425300-425412:+	ACA1_069380	LNS2 domain-containing protein [Source:UniProtKB/TrEMBL;Acc:L8HCT1]	0.00664134	0.510553	0.50391166	1.05E-07
3h	ACA1_031650/clean/scf7180000084522:39142-39243:-	ACA1_031650	Endo/exonuclease/phosphatase domain-containing protein [Source:UniProtKB/TrEMBL;Acc:L8GSN6]	0.064607	0.568458333	0.503851333	0.002036815
3h	ACA1_380820/clean/scf7180000084712:93466-93535:+	ACA1_380820	tRNA-binding domain-containing protein [Source:UniProtKB/TrEMBL;Acc:L8GMV0]	0.006331637	0.510166	0.503834363	4.21E-11
3h	ACA1_085170/clean/scf7180000084632:63626-63722:+	ACA1_085170	TPR_REGION domain-containing protein [Source:UniProtKB/TrEMBL;Acc:L8HM05]	0.001821493	0.50561	0.503788507	6.05E-08
3h	ACA1_360290/clean/scf7180000084622:83775-83991:+	ACA1_360290	Singlestrand binding protein family [Source:UniProtKB/TrEMBL;Acc:L8HBV6]	0.0188007	0.522458	0.5036573	5.39E-08
3h	ACA1_064860/clean/scf7180000084599:1714844-1714936:+	ACA1_064860	OTU domain-containing protein [Source:UniProtKB/TrEMBL;Acc:L8GWU4]	0.020525367	0.524178	0.503652633	4.89E-06
3h	ACA1_372060/clean/scf7180000084664:623722-623795:-	ACA1_372060	Aldo_ket_red domain-containing protein [Source:UniProtKB/TrEMBL;Acc:L8GYH3]	0.010699867	0.514323333	0.503623467	0.000117282
3h	ACA1_130580/clean/scf7180000084698:115749-115836:-	ACA1_130580	CID domain-containing protein [Source:UniProtKB/TrEMBL;Acc:L8GQC8]	0.00257732	0.506060667	0.503483347	2.14E-07
3h	ACA1_098230/clean/scf7180000084645:1433747-1433832:-	ACA1_098230	Leucine rich repeat domain containing protein [Source:UniProtKB/TrEMBL;Acc:L8GLR4]	0.060955667	0.564364	0.503408333	0.000594874
3h	ACA1_145780/clean/scf7180000084710:145785-145846:+	ACA1_145780	A_deaminase domain-containing protein [Source:UniProtKB/TrEMBL;Acc:L8GCK4]	0.0758113	0.579057	0.5032457	9.28E-06
3h	ACA1_024240/clean/scf7180000084441:23869-23950:-	ACA1_024240	Yip1 domain containing protein [Source:UniProtKB/TrEMBL;Acc:L8GN09]	0.000465503	0.5036	0.503134497	1.21E-10
3h	ACA1_089980/clean/scf7180000084639:410886-410951:-	ACA1_089980	hypothetical protein	0	0.503039	0.503039	7.99E-06
3h	ACA1_349640/clean/scf7180000084690:21914-22010:+	ACA1_349640	HEAT repeat domain containing protein [Source:UniProtKB/TrEMBL;Acc:L8GKB3]	0	0.502971333	0.502971333	1.96E-07
3h	ACA1_187290/clean/scf7180000084747:21323-21434:-	ACA1_187290	hypothetical protein	0.024712533	0.527673	0.502960467	2.60E-06
3h	ACA1_314960/clean/scf7180000083887:661-723:-	ACA1_314960	F-box domain-containing protein [Source:UniProtKB/TrEMBL;Acc:L8GZL8]	0.100507133	0.602851	0.502343867	6.20E-05
3h	ACA1_398890/clean/scf7180000084759:240075-240161:+	ACA1_398890	hypothetical protein	0.00933267	0.511648	0.50231533	6.59E-08
3h	ACA1_209820/clean/scf7180000084757:333216-333335:-	ACA1_209820	hypothetical protein	0.0315657	0.53331	0.5017443	2.72E-07
3h	ACA1_002620/clean/scf7180000082935:5797-5865:+	ACA1_002620	Fbox domain containing protein [Source:UniProtKB/TrEMBL;Acc:L8HLR9]	0.000376729	0.501958	0.501581271	9.00E-10
3h	ACA1_025410/clean/scf7180000084446:14306-14377:+	ACA1_025410	Serine/threonine protein kinase [Source:UniProtKB/TrEMBL;Acc:L8HGY2]	0.017183367	0.518664667	0.5014813	7.49E-07
3h	ACA1_320790/clean/scf7180000084416:23173-23251:-	ACA1_320790	RUN domain-containing protein [Source:UniProtKB/TrEMBL;Acc:L8GTR9]	0.00725268	0.508624333	0.501371653	1.01E-08
3h	ACA1_002770/clean/scf7180000082937:7507-7597:+	ACA1_002770	Fbox domain containing protein [Source:UniProtKB/TrEMBL;Acc:L8GJ90]	0.028940733	0.530284667	0.501343933	1.14E-07
3h	ACA1_069340/clean/scf7180000084603:413563-413630:-	ACA1_069340	hypothetical protein	0.042413867	0.543619667	0.5012058	2.69E-06
3h	ACA1_142370/clean/scf7180000084708:153137-153244:-	ACA1_142370	Serine hydroxymethyltransferase [Source:UniProtKB/TrEMBL;Acc:L8HDT1]	0.000673221	0.501734667	0.501061446	2.25E-12
3h	ACA1_385640/clean/scf7180000084730:221547-221655:+	ACA1_385640	hypothetical protein	0.003226113	0.504214333	0.50098822	4.37E-07
3h	ACA1_306690/clean/scf7180000083668:5730-5853:-	ACA1_306690	Gammasecretase subunit APH-1B, putative [Source:UniProtKB/TrEMBL;Acc:L8GQE8]	0.000969294	0.501947667	0.500978373	5.83E-10
3h	ACA1_044720/clean/scf7180000084576:37692-37786:-	ACA1_044720	F-box domain-containing protein [Source:UniProtKB/TrEMBL;Acc:L8GZR9]	0.0392533	0.540102	0.5008487	5.37E-06
3h	ACA1_257430/clean/scf7180000084769:152223-152293:-	ACA1_257430	Pyridoxamine 5'phosphate oxidase family superfamily protein [Source:UniProtKB/TrEMBL;Acc:L8GFR0]	0.02585416	0.5263301	0.50047594	1.34E-05
3h	ACA1_320100/clean/scf7180000084352:7362-7452:+	ACA1_320100	VWFA domain-containing protein [Source:UniProtKB/TrEMBL;Acc:L8H729]	0.006496632	0.506833	0.500336368	4.93E-06
3h	ACA1_053310/clean/scf7180000084594:261120-261214:+	ACA1_053310	Serine/threonine kinase [Source:UniProtKB/TrEMBL;Acc:L8H7D1]	0	0.500252333	0.500252333	9.95E-08
3h	ACA1_108260/clean/scf7180000084661:23165-23238:-	ACA1_108260	Prenylcys_lyase domain-containing protein [Source:UniProtKB/TrEMBL;Acc:L8GD56]	0.180002	0.679844667	0.499842667	0.000111999
3h	ACA1_071240/clean/scf7180000084603:895983-896129:-	ACA1_071240	Transporter, major facilitator subfamily protein [Source:UniProtKB/TrEMBL;Acc:L8HFI7]	0.00260802	0.502361667	0.499753647	1.47E-06
3h	ACA1_261080/clean/scf7180000084769:596122-596226:-	ACA1_261080	MIT domain-containing protein [Source:UniProtKB/TrEMBL;Acc:L8GF47]	0.171697	0.671412667	0.499715667	4.06E-05
3h	ACA1_091390/clean/scf7180000084645:106149-106243:+	ACA1_091390	Ras family GTPase [Source:UniProtKB/TrEMBL;Acc:L8GIH3]	0.00485949	0.5045734	0.49971391	6.40E-13
3h	ACA1_193140/clean/scf7180000084749:195334-195457:+	ACA1_193140	Metallophosphoesterase [Source:UniProtKB/TrEMBL;Acc:L8GQV5]	0.00119161	0.500728	0.49953639	1.11E-07
3h	ACA1_221030/clean/scf7180000084758:982682-982774:-	ACA1_221030	DUF1992 domain-containing protein [Source:UniProtKB/TrEMBL;Acc:L8GQX4]	0.008317987	0.507841	0.499523013	4.40E-06
3h	ACA1_012040/clean/scf7180000083652:9146-9265:+	ACA1_012040	LIM domain containing protein [Source:UniProtKB/TrEMBL;Acc:L8GI34]	0.0104337	0.509774333	0.499340633	2.87E-10
3h	ACA1_063950/clean/scf7180000084599:1542815-1542906:+	ACA1_063950	Fbox domain containing protein [Source:UniProtKB/TrEMBL;Acc:L8GZW1]	0	0.499007	0.499007	5.17E-07
3h	ACA1_367390/clean/scf7180000084641:619598-619664:+	ACA1_367390	FAD-binding PCMH-type domain-containing protein [Source:UniProtKB/TrEMBL;Acc:L8GM36]	0.1420894	0.640863	0.4987736	0.001290804
3h	ACA1_145180/clean/scf7180000084710:53681-53750:-	ACA1_145180	F-box domain-containing protein [Source:UniProtKB/TrEMBL;Acc:L8GCU3]	0.0074074	0.50598	0.4985726	1.02E-05
3h	ACA1_295270/clean/scf7180000084777:1088796-1088877:-	ACA1_295270	Rhodanese domain-containing protein [Source:UniProtKB/TrEMBL;Acc:L8HJQ3]	0.060830433	0.559355333	0.4985249	3.42E-06
3h	ACA1_184420/clean/scf7180000084745:640192-640285:+	ACA1_184420	Cathepsin Llike proteinase [Source:UniProtKB/TrEMBL;Acc:L8H8J9]	0.004948973	0.502798	0.497849027	6.03E-15
3h	ACA1_385700/clean/scf7180000084730:241285-241343:+	ACA1_385700	F-box domain-containing protein [Source:UniProtKB/TrEMBL;Acc:L8HAP4]	0.076861667	0.574448	0.497586333	0.005648264
3h	ACA1_067410/clean/scf7180000084601:20340-20437:-	ACA1_067410	F-box domain-containing protein [Source:UniProtKB/TrEMBL;Acc:L8GTP9]	0.012127233	0.509692667	0.497565433	6.75E-07
3h	ACA1_068360/clean/scf7180000084603:181458-181558:+	ACA1_068360	Dual specificity protein kinase [Source:UniProtKB/TrEMBL;Acc:L8HDE4]	0.011817642	0.509208	0.497390358	3.26E-09
3h	ACA1_093490/clean/scf7180000084645:558740-558823:+	ACA1_093490	Arf-GAP domain-containing protein [Source:UniProtKB/TrEMBL;Acc:L8GJ65]	0.00246151	0.499751	0.49728949	4.51E-09
3h	ACA1_229570/clean/scf7180000084761:280569-280672:-	ACA1_229570	Oxidoreductase, short chain dehydrogenase/reductase superfamily protein [Source:UniProtKB/TrEMBL;Acc:L8H814]	0.026291867	0.523449667	0.4971578	6.08E-06
3h	ACA1_391330/clean/scf7180000084740:153902-154145:-	ACA1_391330	hypothetical protein	0.040738333	0.537797667	0.497059333	7.60E-08

3h	ACA1_247140/clean/scf7180000084763:629097-629192:+	ACA1_247140	hypothetical protein	0.008506423	0.505348	0.496841577	3.87E-07
3h	ACA1_251790/clean/scf7180000084768:119067-119139:-	ACA1_251790	Cellulase (Glycosyl hydrolase family 5) subfamily protein [Source:UniProtKB/TrEMBL;Acc:L8HAR2]	0.012416353	0.509170333	0.49675398	4.78E-09
3h	ACA1_041970/clean/scf7180000084573:28614-28699:+	ACA1_041970	hypothetical protein	0	0.496345333	0.496345333	0.000253982
3h	ACA1_129350/clean/scf7180000084698:9563-9631:+	ACA1_129350	Fatty acid hydroxylase domain-containing protein [Source:UniProtKB/TrEMBL;Acc:L8GPA5]	0.0147887	0.511072	0.4962833	3.57E-10
3h	ACA1_076720/clean/scf7180000084612:121950-122024:-	ACA1_076720	Fbox domain containing protein [Source:UniProtKB/TrEMBL;Acc:L8GLV4]	0.01596267	0.512152333	0.496189663	1.35E-06
3h	ACA1_068360/clean/scf7180000084603:180971-181059:+	ACA1_068360	Dual specificity protein kinase [Source:UniProtKB/TrEMBL;Acc:L8HDE4]	0.020229367	0.516279	0.496049633	9.56E-09
3h	ACA1_155810/clean/scf7180000084722:245570-245665:+	ACA1_155810	Fructose6-phosphate-2-kinase, putative [Source:UniProtKB/TrEMBL;Acc:L8H1J5]	0.089088167	0.585008333	0.495920167	1.51E-05
3h	ACA1_370080/clean/scf7180000084664:435113-435201:-	ACA1_370080	ATPdependent RNA helicase Ddx1, putative [Source:UniProtKB/TrEMBL;Acc:L8GZ36]	0	0.495874	0.495874	1.52E-06
3h	ACA1_258970/clean/scf7180000084769:330568-330717:-	ACA1_258970	hypothetical protein	0.225596667	0.721428333	0.495831667	6.95E-05
3h	ACA1_101490/clean/scf7180000084649:73433-73537:-	ACA1_101490	Methyltransf_11 domain-containing protein [Source:UniProtKB/TrEMBL;Acc:L8GH92]	0.384928333	0.880503	0.495574667	0.002638207
3h	ACA1_053360/clean/scf7180000084594:269298-269383:+	ACA1_053360	RING-type domain-containing protein [Source:UniProtKB/TrEMBL;Acc:L8H7D5]	0.0159026	0.511325667	0.495423067	3.13E-06
3h	ACA1_195240/clean/scf7180000084751:82355-82410:+	ACA1_195240	CBS domain containing protein [Source:UniProtKB/TrEMBL;Acc:L8H5D8]	0.025620073	0.520878333	0.49525826	2.25E-08
3h	ACA1_149540/clean/scf7180000084715:176960-177057:+	ACA1_149540	VPS37 C-terminal domain-containing protein [Source:UniProtKB/TrEMBL;Acc:L8HEF0]	0.019429657	0.514426667	0.49499701	2.70E-09
3h	ACA1_012960/clean/scf7180000083742:12499-12579:-	ACA1_012960	hypothetical protein	0.0618649	0.556599333	0.494734433	2.37E-06
3h	ACA1_287920/clean/scf7180000084777:125484-125555:-	ACA1_287920	Leucinerich repeat protein lrrA, putative [Source:UniProtKB/TrEMBL;Acc:L8HL59]	0.009710997	0.504395	0.494684003	1.38E-09
3h	ACA1_371710/clean/scf7180000084664:586749-586829:-	ACA1_371710	Fbox domain containing protein [Source:UniProtKB/TrEMBL;Acc:L8GZD1]	0.0388393	0.533277333	0.494438033	1.04E-06
3h	ACA1_025560/clean/scf7180000084448:13829-13904:-	ACA1_025560	hypothetical protein	0.130921	0.625329333	0.494408333	0.004755773
3h	ACA1_061670/clean/scf7180000084599:1001738-1001828:-	ACA1_061670	SSD domain-containing protein [Source:UniProtKB/TrEMBL;Acc:L8GWV2]	0	0.494362	0.494362	2.36E-06
3h	ACA1_173060/clean/scf7180000084741:394438-394536:-	ACA1_173060	SNARE associated Golgi protein [Source:UniProtKB/TrEMBL;Acc:L8HGL7]	0.0083494	0.502648333	0.494298933	3.00E-05
3h	ACA1_175480/clean/scf7180000084741:966948-967026:-	ACA1_175480	PQ loop repeat-containing protein [Source:UniProtKB/TrEMBL;Acc:L8HJK6]	0.139370333	0.633498	0.494127667	0.000149001
3h	ACA1_273100/clean/scf7180000084773:15170-15262:-	ACA1_273100	EF-hand domain-containing protein [Source:UniProtKB/TrEMBL;Acc:L8GG54]	0.0168397	0.510888	0.4940483	0.00510451
3h	ACA1_296570/clean/scf7180000084777:1386317-1386385:+	ACA1_296570	TRANSMEMBRANE PROTEIN, putative [Source:UniProtKB/TrEMBL;Acc:L8HIX9]	0.029144103	0.523104333	0.49396023	3.35E-07
3h	ACA1_110060/clean/scf7180000084663:50405-50517:-	ACA1_110060	Initiation factor, subunit 2 family protein [Source:UniProtKB/TrEMBL;Acc:L8HHD7]	0.000286216	0.494104333	0.493818118	8.07E-10
3h	ACA1_060250/clean/scf7180000084599:626783-626906:-	ACA1_060250	Alpha-carbonic anhydrase domain-containing protein [Source:UniProtKB/TrEMBL;Acc:L8GXK3]	0.018692367	0.512452	0.493759633	2.45E-09
3h	ACA1_143580/clean/scf7180000084709:40489-40576:-	ACA1_143580	Med12 domain-containing protein [Source:UniProtKB/TrEMBL;Acc:L8HH02]	0.007314147	0.501013333	0.493699187	5.07E-09
3h	ACA1_200240/clean/scf7180000084753:419661-419755:-	ACA1_200240	Phospholipase D active site domain containing protein [Source:UniProtKB/TrEMBL;Acc:L8H3B9]	0	0.493578667	0.493578667	4.60E-08
3h	ACA1_198480/clean/scf7180000084753:124168-124308:+	ACA1_198480	Transcription factor III family protein [Source:UniProtKB/TrEMBL;Acc:L8H324]	0.026644733	0.520173	0.493528267	3.15E-05
3h	ACA1_035370/clean/scf7180000084564:36712-36831:-	ACA1_035370	Protein kinase domain-containing protein [Source:UniProtKB/TrEMBL;Acc:L8HAE6]	0.01407887	0.507604	0.49352513	5.06E-07
3h	ACA1_235090/clean/scf7180000084762:336006-336100:-	ACA1_235090	Lysophospholipaselike protein [Source:UniProtKB/TrEMBL;Acc:L8H3L9]	0.021349833	0.514858	0.493508167	3.45E-09
3h	ACA1_060250/clean/scf7180000084599:626597-626697:-	ACA1_060250	Alpha-carbonic anhydrase domain-containing protein [Source:UniProtKB/TrEMBL;Acc:L8GXK3]	0.032967667	0.526440667	0.493473	1.58E-08
3h	ACA1_310800/clean/scf7180000083780:17627-17703:-	ACA1_310800	EamA domain-containing protein [Source:UniProtKB/TrEMBL;Acc:L8GVY7]	0.022321433	0.515683	0.493361567	2.92E-05
3h	ACA1_360290/clean/scf7180000084622:83356-83419:+	ACA1_360290	Singlestrand binding protein family [Source:UniProtKB/TrEMBL;Acc:L8HBV6]	0.0126023	0.5059	0.4932977	6.37E-09
3h	ACA1_289970/clean/scf7180000084777:515400-515524:+	ACA1_289970	FHA domain-containing protein [Source:UniProtKB/TrEMBL;Acc:L8HKP4]	0.027323337	0.520552333	0.493228997	1.96E-06
3h	ACA1_360980/clean/scf7180000084622:214758-214843:+	ACA1_360980	hypothetical protein	0.0313084	0.524474333	0.493165933	0.00030555
3h	ACA1_264680/clean/scf7180000084770:356495-356592:+	ACA1_264680	Protein kinase domain-containing protein [Source:UniProtKB/TrEMBL;Acc:L8H1B3]	0.0095817	0.502511	0.4929293	3.50E-09
3h	ACA1_054330/clean/scf7180000084594:469621-469674:-	ACA1_054330	Hbxip, putative [Source:UniProtKB/TrEMBL;Acc:L8H639]	0.007691463	0.500592667	0.492901203	2.76E-10
3h	ACA1_035640/clean/scf7180000084564:101470-101553:-	ACA1_035640	Beta-lactamase domain-containing protein [Source:UniProtKB/TrEMBL;Acc:L8H9P2]	0.086400533	0.579167	0.492766467	0.013396807
3h	ACA1_025560/clean/scf7180000084448:14272-14352:-	ACA1_025560	hypothetical protein	0.024169133	0.516660667	0.492491533	0.002617523
3h	ACA1_057460/clean/scf7180000084599:147412-147512:-	ACA1_057460	hypothetical protein	0.0169455	0.509407	0.4924615	3.49E-05
3h	ACA1_293730/clean/scf7180000084777:968202-968295:+	ACA1_293730	PB1 domain containing protein [Source:UniProtKB/TrEMBL;Acc:L8HJM7]	0.00440068	0.496760667	0.492359987	1.02E-14
3h	ACA1_073240/clean/scf7180000084603:1378734-1378834:-	ACA1_073240	hypothetical protein	0.004335633	0.496598667	0.492263033	2.76E-05
3h	ACA1_024630/clean/scf7180000084444:1644-1733:+	ACA1_024630	hypothetical protein	0.003875967	0.496080333	0.492204367	9.40E-08
3h	ACA1_325150/clean/scf7180000084475:10629-10700:+	ACA1_325150	Rab7/RabGfamily small GTPase [Source:UniProtKB/TrEMBL;Acc:L8GPH2]	0.0111165	0.503256333	0.492139833	6.81E-11
3h	ACA1_123890/clean/scf7180000084686:79747-79854:-	ACA1_123890	hypothetical protein	0.139897333	0.631846333	0.491949	0.002437423
3h	ACA1_230490/clean/scf7180000084761:359460-359580:+	ACA1_230490	RFX-type winged-helix domain-containing protein [Source:UniProtKB/TrEMBL;Acc:L8HAD4]	0.003969253	0.495885	0.491915747	6.64E-08
3h	ACA1_247260/clean/scf7180000084763:657894-658013:+	ACA1_247260	SET domain-containing protein [Source:UniProtKB/TrEMBL;Acc:L8GKH1]	0.003881855	0.495592333	0.491710478	5.58E-12
3h	ACA1_000330/clean/scf7180000082823:14852-14934:-	ACA1_000330	Myotubularin phosphatase domain-containing protein [Source:UniProtKB/TrEMBL;Acc:L8H1Z4]	0.039410367	0.531038667	0.4916283	0.000110499
3h	ACA1_107540/clean/scf7180000084660:217615-217705:+	ACA1_107540	hypothetical protein	0.0261789	0.517628333	0.491449433	0.00015049
3h	ACA1_059530/clean/scf7180000084599:462217-462292:-	ACA1_059530	hypothetical protein	0.006171167	0.497544333	0.491373167	1.83E-08
3h	ACA1_331240/clean/scf7180000084575:10130-10195:-	ACA1_331240	Fe2OG dioxygenase domain-containing protein [Source:UniProtKB/TrEMBL;Acc:L8GVM3]	0.1062146	0.597449333	0.491234733	8.74E-05
3h	ACA1_326880/clean/scf7180000084510:63541-63624:-	ACA1_326880	hypothetical protein	0.005940117	0.496696	0.490755883	5.37E-06
3h	ACA1_165580/clean/scf7180000084732:56587-56750:-	ACA1_165580	BTB/POZ domain containing protein [Source:UniProtKB/TrEMBL;Acc:L8HI24]	0	0.490734667	0.490734667	1.21E-05
3h	ACA1_029390/clean/scf7180000084476:5503-5597:-	ACA1_029390	WD repeat protein [Source:UniProtKB/TrEMBL;Acc:L8GZ57]	0.0393283	0.529669333	0.490341033	3.12E-07
3h	ACA1_088790/clean/scf7180000084639:229172-229240:-	ACA1_088790	hypothetical protein	0.006524333	0.496769333	0.490245	9.47E-05
3h	ACA1_264460/clean/scf7180000084770:294313-294427:-	ACA1_264460	GTPbinding protein [Source:UniProtKB/TrEMBL;Acc:L8H231]	0.072966333	0.563006	0.490039667	1.49E-05
3h	ACA1_264850/clean/scf7180000084770:389582-389654:+	ACA1_264850	MaoC-like domain-containing protein [Source:UniProtKB/TrEMBL;Acc:L8H4C0]	0.0585522	0.548582667	0.490030467	1.12E-06
3h	ACA1_222680/clean/scf7180000084760:52025-52118:-	ACA1_222680	FAD-binding FR-type domain-containing protein [Source:UniProtKB/TrEMBL;Acc:L8GST4]	0.029386067	0.519319	0.489932933	3.00E-07
3h	ACA1_024410/clean/scf7180000084442:17621-17719:+	ACA1_024410	Serine/threonineprotein kinase [Source:UniProtKB/TrEMBL;Acc:L8H7W5]	0.004359873	0.494233333	0.48987346	5.19E-09
3h	ACA1_178710/clean/scf7180000084743:267426-267491:-	ACA1_178710	ADPribosylation factor related protein 1, putative [Source:UniProtKB/TrEMBL;Acc:L8GTG3]	0.004166667	0.4939514	0.489784733	1.07E-06
3h	ACA1_323490/clean/scf7180000084468:35894-35977:+	ACA1_323490	DH domain-containing protein [Source:UniProtKB/TrEMBL;Acc:L8H1L1]	0.031010033	0.520721667	0.489711633	3.26E-08
3h	ACA1_193140/clean/scf7180000084749:195982-196056:+	ACA1_193140	Metallophosphoesterase [Source:UniProtKB/TrEMBL;Acc:L8GQV5]	0	0.489558333	0.489558333	1.86E-06
3h	ACA1_050360/clean/scf7180000084589:77953-78031:-	ACA1_050360	Metalloenzyme domain-containing protein [Source:UniProtKB/TrEMBL;Acc:L8H565]	0.006575303	0.496033667	0.489458363	1.51E-10
3h	ACA1_296570/clean/scf7180000084777:1386553-1386632:+	ACA1_296570	TRANSMEMBRANE PROTEIN, putative [Source:UniProtKB/TrEMBL;Acc:L8HIX9]	0.009918723	0.499287333	0.48936861	1.46E-07
3h	ACA1_101520/clean/scf7180000084649:84188-84292:-	ACA1_101520	ANK_REP_REGION domain-containing protein [Source:UniProtKB/TrEMBL;Acc:L8GIW9]	0.011261533	0.500579	0.489317467	4.97E-09
3h	ACA1_357160/clean/scf7180000084735:10478-10574:-	ACA1_357160	Actin subfamily protein [Source:UniProtKB/TrEMBL;Acc:L8HDB7]	0.137488667	0.626753333	0.489264667	4.35E-06
3h	ACA1_103860/clean/scf7180000084653:54119-54193:-	ACA1_103860	Kinase [Source:UniProtKB/TrEMBL;Acc:L8GE29]	0.010441767	0.499694333	0.489252567	1.17E-06
3h	ACA1_298630/clean/scf7180000082992:5660-5755:-	ACA1_298630	Vacuolar protein sorting 16, putative [Source:UniProtKB/TrEMBL;Acc:L8H081]	0	0.489187	0.489187	1.30E-09
3h	ACA1_182860/clean/scf7180000084745:267307-267418:-	ACA1_182860	Ankyrin repeat protein, putative [Source:UniProtKB/TrEMBL;Acc:L8H7X7]	0.083920533	0.57301	0.489089467	5.85E-06
3h	ACA1_142130/clean/scf7180000084708:90404-90467:+	ACA1_142130	unspecified product	0	0.489082	0.489082	6.82E-11
3h	ACA1_382360/clean/scf7180000084721:37875-37972:+	ACA1_382360	F-box domain-containing protein [Source:UniProtKB/TrEMBL;Acc:L8GV11]	0.030451033	0.519513	0.489061967	3.01E-06
3h	ACA1_127620/clean/scf7180000084696:36745-36966:+	ACA1_127620	Fe2OG dioxygenase domain-containing protein [Source:UniProtKB/TrEMBL;Acc:L8GGE8]	0.014852393	0.503757667	0.488905273	0.000534333
3h	ACA1_107160/clean/scf7180000084660:161645-161783:+	ACA1_107160	hypothetical protein	0.009898143	0.49852	0.488621857	1.65E-07
3h	ACA1_095070/clean/scf7180000084645:806721-806844:+	ACA1_095070	hypothetical protein	0.007953678	0.496474333	0.488520655	7.02E-08
3h	ACA1_116270/clean/scf7180000084669:744118-744229:+	ACA1_116270	G_PROTEIN_RECEP_F1_2 domain-containing protein [Source:UniProtKB/TrEMBL;Acc:L8H6T9]	0.0270836	0.515587667	0.488504067	8.64E-07
3h	ACA1_141540/clean/scf7180000084708:14516-14602:+	ACA1_141540	Beta_helix domain-containing protein [Source:UniProtKB/TrEMBL;Acc:L8HBA1]	0.048597867	0.537034333	0.488436467	1.25E-05
3h	ACA1_272260/clean/scf7180000084772:42979-43079:-	ACA1_272260	M20_dimer domain-containing protein [Source:UniProtKB/TrEMBL;Acc:L8HGE8]	0.005854523	0.494110667	0.488256143	2.55E-09
3h	ACA1_106270/clean/scf7180000084660:67485-67557:-	ACA1_106270	ZT_dimer domain-containing protein [Source:UniProtKB/TrEMBL;Acc:L8GQ80]	0.005803513	0.493981667	0.488178153	5.67E-06
3h	ACA1_123880/clean/scf7180000084686:78302-78433:-	ACA1_123880	hypothetical protein	0.0346215	0.522598	0.4879765	1.30E-06
3h	ACA1_015510/clean/scf7180000083799:10370-10488:-	ACA1_015510	Replication factora protein 1 (Rpa1) subfamily protein [Source:UniProtKB/TrEMBL;Acc:L8GZQ4]	0.01700314	0.504978	0.48797486	2.53E-07
3h	ACA1_388480/clean/scf7180000084733:123002-123087:-	ACA1_388480	Cofilin/tropomyosin-type actin-binding protein [Source:UniProtKB/TrEMBL;Acc:L8GEM8]	0.026843473	0.514809	0.487965527	1.26E-07
3h	ACA1_141960/clean/scf7180000084708:61745-61814:-	ACA1_141960	unspecified product	0.040049467	0.527977333	0.487927867	4.23E-06
3h	ACA1_203620/clean/scf7180000084754:172093-172181:+	ACA1_203620	Cullin domain-containing protein [Source:UniProtKB/TrEMBL;Acc:L8GUK9]	0.048320567	0.536052	0.487731433	2.04E-05
3h	ACA1_290650/clean/scf7180000084777:646292-646368:+	ACA1_290650	DENN domain-containing protein [Source:UniProtKB/TrEMBL;Acc:L8HI74]	0.008294467	0.495843	0.487548533	1.49E-05
3h	ACA1_264270/clean/scf7180000084770:226332-226432:-	ACA1_264270	Oxidoreductase, short chain dehydrogenase/reductase family protein [Source:UniProtKB/TrEMBL;Acc:L8H474]	0.033202233	0.520635333	0.4874331	1.92E-08
3h	ACA1_395500/clean/scf7180000084756:194703-194776:-	ACA1_395500	Rho-GAP domain-containing protein [Source:UniProtKB/TrEMBL;Acc:L8GZP8]	0.0181995	0.505583333	0.487383833	4.06E-06
3h	ACA1_234070/clean/scf7180000084762:193401-193511:+	ACA1_234070	ELL complex EAP30 subunit [Source:UniProtKB/TrEMBL;Acc:L8H0D3]	0.004610947	0.491877667	0.48726672	3.44E-07
3h	ACA1_068390/clean/scf7180000084603:186577-186659:-	ACA1_068390	hypothetical protein	0.024763667	0.512024	0.487260333	8.14E-05
3h	ACA1_070900/clean/scf7180000084603:797146-797200:+	ACA1_070900	MMtag domain-containing protein [Source:UniProtKB/TrEMBL;Acc:L8HD49]	0.018872933	0.506031	0.487158067	1.22E-06
3h	ACA1_342290/clean/scf7180000084633:13782-13878:+	ACA1_342290	hypothetical protein	0.069128233	0.55593	0.486801767	5.40E-05
3h	ACA1_269730/clean/scf7180000084770:1027056-1027219:+	ACA1_269730	Metallophos domain-containing protein [Source:UniProtKB/TrEMBL;Acc:L8H506]	0.0217648	0.508478333	0.486713533	0.010967218
3h	ACA1_041780/clean/scf7180000084573:7363-7442:+	ACA1_041780	hypothetical protein	0.020687067	0.507359333	0.486672267	1.58E-05
3h	ACA1_088960/clean/scf7180000084639:274349-274431:-	ACA1_088960	Beta-lactamase domain-containing protein [Source:UniProtKB/TrEMBL;Acc:L8GU04]	0.021505367	0.50798	0.486474633	2.47E-05
3h	ACA1_189450/clean/scf7180000084748:41176-41260:-	ACA1_189450	Chloride channel protein [Source:UniProtKB/TrEMBL;Acc:L8GEJ2]	0.00060887	0.487054667	0.486445797	2.33E-08
3h	ACA1_156050/clean/scf7180000084723:14255-14318:+	ACA1_156050	Armadillo/betacatenin-like repeat domain containing protein [Source:UniProtKB/TrEMBL;Acc:L8GIE3]	0	0.486316667	0.486316667	0.004464791
3h	ACA1_070410/clean/scf7180000084603:669575-669737:-	ACA1_070410	L-type lectin-like domain-containing protein [Source:UniProtKB/TrEMBL;Acc:L8HFA0]	0.088377667	0.574417333	0.486039667	2.34E-05
3h	ACA1_099100/clean/scf7180000084646:91129-91197:+	ACA1_099100	PH domain-containing protein [Source:UniProtKB/TrEMBL;Acc:L8H5M3]	0.01187214	0.497841333	0.485969193	2.09E-06
3h	ACA1_144880/clean/scf7180000084710:32797-32889:-	ACA1_144880	hypothetical protein	3.66E-05	0.485961667	0.485925049	4.24E-09
3h	ACA1_281320/clean/scf7180000084776:587004-587087:-	ACA1_281320	Sel1 repeatcontaining protein [Source:UniProtKB/TrEMBL;Acc:L8H8J4]	0	0.485896333	0.485896333	5.47E-07
3h	ACA1_035600/clean/scf7180000084564:97530-97598:+	ACA1_035600	Cupin_2 domain-containing protein [Source:UniProtKB/TrEMBL;Acc:L8HAL8]	0.009692257	0.495404333	0.485712077	0.000125864
3h	ACA1_024200/clean/scf7180000084441:14171-14278:-	ACA1_024200	WD_REPEATS_REGION domain-containing protein [Source:UniProtKB/TrEMBL;Acc:L8GKS0]	0.018898733	0.504499	0.485600267	3.38E-06
3h	ACA1_237840/clean/scf7180000084763:27251-27322:-	ACA1_237840	hypothetical protein	0.059075333	0.544655	0.485579667	2.06E-07
3h	ACA1_061980/clean/scf7180000084599:1093017-1093086:+	ACA1_061980	F-box domain-containing protein [Source:UniProtKB/TrEMBL;Acc:L8GZC3]	0.0212982	0.506822333	0.485524133	0.000109205
3h	ACA1_290510/clean/scf7180000084777:609159-609232:-	ACA1_290510	ABC transporter domain-containing protein [Source:UniProtKB/TrEMBL;Acc:L8HLJ2]	0	0.485506667	0.485506667	1.24E-08
3h	ACA1_191150/clean/scf7180000084748:186806-186874:+	ACA1_191150	Triosephosphate isomerase [Source:UniProtKB/TrEMBL;Acc:L8GE90]	0.000807103	0.486159	0.485351897	6.19E-11
3h	ACA1_382380/clean/scf7180000084721:45368-45448:+	ACA1_382380	WD_REPEATS_REGION domain-containing protein [Source:UniProtKB/TrEMBL;Acc:L8GUA6]	0.003039027	0.488333	0.485293973	4.92E-07
3h	ACA1_038350/clean/scf7180000084568:134164-134226:-	ACA1_038350	Fbox domain containing protein [Source:UniProtKB/TrEMBL;Acc:L8GKR6]	0.042769	0.527567	0.484798	3.93E-05
3h	ACA1_235700/clean/scf7180000084762:359324-359468:-	ACA1_235700	TLDc domain-containing protein [Source:UniProtKB/TrEMBL;Acc:L8H1D8]	0.0936563	0.578350333	0.484694033	0.000511317
3h	ACA1_043190/clean/scf7180000084573:136795-136870:-	ACA1_043190	hypothetical protein	0.019804033	0.504458	0.484653967	5.85E-09
3h	ACA1_341560/clean/scf7180000084628:4069-4138:+	ACA1_341560	CBS domain containing protein [Source:UniProtKB/TrEMBL;Acc:L8GL09]	0.090872867	0.575445	0.484572133	4.19E-05
3h	ACA1_246780/clean/scf7180000084763:580698-580795:-	ACA1_246780	Ras subfamily protein [Source:UniProtKB/TrEMBL;Acc:L8GK82]	0.00233126	0.486497	0.48416574	8.62E-11
3h	ACA1_087880/clean/scf7180000084639:122870-122957:-	ACA1_087880	Beta-amylase [Source:UniProtKB/TrEMBL;Acc:L8GVJ7]	0.002109703	0.486200333	0.48409063	1.76E-06
3h	ACA1_280530/clean/scf7180000084776:490147-490220:-	ACA1_280530	hypothetical protein	0.048109767	0.531975333	0.483865567	4.06E-06
3h	ACA1_114500/clean/scf7180000084669:486959-487042:-	ACA1_114500	Macro domain-containing protein [Source:UniProtKB/TrEMBL;Acc:L8H6J5]	0.00643987	0.490299333	0.483859463	1.65E-09
3h	ACA1_092640/clean/scf7180000084645:373459-373558:-	ACA1_092640	Amidase domain-containing protein [Source:UniProtKB/TrEMBL;Acc:L8GKL1]	0.01044963	0.494221333	0.483771703	5.01E-09
3h	ACA1_109740/clean/scf7180000084663:16713-16832:-	ACA1_109740	Myblike DNA-binding domain containing protein [Source:UniProtKB/TrEMBL;Acc:L8HJY9]	0	0.483655333	0.483655333	3.75E-08
3h	ACA1_178250/clean/scf7180000084743:190407-190567:-	ACA1_178250	hypothetical protein	0.006110507	0.489621667	0.48351116	1.76E-05
3h	ACA1_068950/clean/scf7180000084603:317063-317141:+	ACA1_068950	hypothetical protein	0.003828017	0.48715	0.483321983	1.79E-08
3h	ACA1_152930/clean/scf7180000084720:36638-36735:-	ACA1_152930	PG_binding_1 domain-containing protein [Source:UniProtKB/TrEMBL;Acc:L8HF34]	0	0.483102	0.483102	6.64E-09
3h	ACA1_027030/clean/scf7180000084464:26788-26853:-	ACA1_027030	C2 domain-containing protein [Source:UniProtKB/TrEMBL;Acc:L8GJ80]	0.026342053	0.509310333	0.48296828	1.40E-06
3h	ACA1_377830/clean/scf7180000084711:127015-127081:+	ACA1_377830	UBIQUITIN_CONJUGAT_2 domain-containing protein [Source:UniProtKB/TrEMBL;Acc:L8GSV2]	0.0460145	0.528980333	0.482965833	7.63E-08
3h	ACA1_069340/clean/scf7180000084603:414272-414368:-	ACA1_069340	hypothetical protein	0.008490977	0.491349667	0.48285869	2.04E-08
3h	ACA1_127640/clean/scf7180000084696:39639-39757:+	ACA1_127640	GP-PDE domain-containing protein [Source:UniProtKB/TrEMBL;Acc:L8GEW1]	0.032678133	0.515526333	0.4828482	1.14E-06
3h	ACA1_048230/clean/scf7180000084582:61311-61425:+	ACA1_048230	Protein kinase domain-containing protein [Source:UniProtKB/TrEMBL;Acc:L8GE93]	0.0476805	0.530517667	0.482837167	0.000156581
3h	ACA1_220590/clean/scf7180000084758:926356-926426:+	ACA1_220590	hypothetical protein	0	0.482695	0.482695	0.007620149
3h	ACA1_066020/clean/scf7180000084599:1974254-1974352:+	ACA1_066020	CBS domain containing protein [Source:UniProtKB/TrEMBL;Acc:L8H067]	0.001695873	0.484230667	0.482534793	8.92E-05
3h	ACA1_230510/clean/scf7180000084761:368278-368344:-	ACA1_230510	hypothetical protein	0.0391097	0.521642	0.4825323	2.27E-06
3h	ACA1_106290/clean/scf7180000084660:72437-72509:+	ACA1_106290	WD_REPEATS_REGION domain-containing protein [Source:UniProtKB/TrEMBL;Acc:L8GNF9]	0	0.482494667	0.482494667	4.29E-07
3h	ACA1_054840/clean/scf7180000084594:589274-589344:-	ACA1_054840	Cytochrome p450 superfamily protein [Source:UniProtKB/TrEMBL;Acc:L8H7M8]	0.0941137	0.576590333	0.482476633	5.82E-05
3h	ACA1_206620/clean/scf7180000084757:25811-25922:+	ACA1_206620	MCM domain-containing protein [Source:UniProtKB/TrEMBL;Acc:L8GY26]	0.014999413	0.497275667	0.482276253	4.69E-07
3h	ACA1_281330/clean/scf7180000084776:590285-590363:-	ACA1_281330	Protein kinase domain containing protein [Source:UniProtKB/TrEMBL;Acc:L8H6A8]	0.021740267	0.503884	0.482143733	9.30E-06
3h	ACA1_323530/clean/scf7180000084468:42919-42985:+	ACA1_323530	Fbox domain containing protein [Source:UniProtKB/TrEMBL;Acc:L8H0X5]	0.029295333	0.511402333	0.482107	0.000124151
3h	ACA1_214850/clean/scf7180000084758:245642-245741:+	ACA1_214850	LIM zinc-binding domain-containing protein [Source:UniProtKB/TrEMBL;Acc:L8GSG1]	0.050807467	0.532822667	0.4820152	1.39E-06
3h	ACA1_123630/clean/scf7180000084686:46489-46581:-	ACA1_123630	hypothetical protein	0.232619	0.714617667	0.481998667	0.000347924
3h	ACA1_139560/clean/scf7180000084705:96489-96592:-	ACA1_139560	YDG domain-containing protein [Source:UniProtKB/TrEMBL;Acc:L8GN24]	0.137402333	0.619241	0.481838667	0.004475176
3h	ACA1_024200/clean/scf7180000084441:15012-15094:-	ACA1_024200	WD_REPEATS_REGION domain-containing protein [Source:UniProtKB/TrEMBL;Acc:L8GKS0]	0	0.481415	0.481415	1.10E-06
3h	ACA1_253100/clean/scf7180000084768:240633-240703:-	ACA1_253100	MORN repeatcontaining protein [Source:UniProtKB/TrEMBL;Acc:L8H9X8]	0.002329287	0.483577	0.481247713	8.54E-08
3h	ACA1_048090/clean/scf7180000084582:18556-18635:+	ACA1_048090	hypothetical protein	0.009207767	0.490073667	0.4808659	7.80E-06
3h	ACA1_174000/clean/scf7180000084741:569158-569235:-	ACA1_174000	hypothetical protein	0.015957657	0.496784333	0.480826677	1.07E-08

3h	ACA1_200100/clean/scf7180000084753:390262-390353:-	ACA1_200100	hypothetical protein	0.022911983	0.503597333	0.48068535	6.15E-07
3h	ACA1_307840/clean/scf7180000083687:22944-23037:+	ACA1_307840	TehB domain-containing protein [Source:UniProtKB/TrEMBL;Acc:L8HBJ4]	0.0242071	0.504823667	0.480616567	1.42E-05
3h	ACA1_095060/clean/scf7180000084645:804206-804358:-	ACA1_095060	Sidoreflexin [Source:UniProtKB/TrEMBL;Acc:L8GL14]	0.001351222	0.481654	0.480302778	1.33E-08
3h	ACA1_072990/clean/scf7180000084603:1350925-1350996:-	ACA1_072990	hypothetical protein	0.011494267	0.491765333	0.480271067	3.37E-05
3h	ACA1_184390/clean/scf7180000084745:633021-633123:+	ACA1_184390	hypothetical protein	0.006512733	0.486777333	0.4802646	4.42E-06
3h	ACA1_296560/clean/scf7180000084777:1382605-1382716:-	ACA1_296560	CitMHS domain-containing protein [Source:UniProtKB/TrEMBL;Acc:L8HLL2]	0.020942087	0.501195	0.480252913	1.59E-07
3h	ACA1_024200/clean/scf7180000084441:15319-15408:-	ACA1_024200	WD_REPEATS_REGION domain-containing protein [Source:UniProtKB/TrEMBL;Acc:L8GKS0]	0	0.480214	0.480214	1.55E-06
3h	ACA1_114600/clean/scf7180000084669:519964-520122:+	ACA1_114600	hypothetical protein	0.0250166	0.505223667	0.480207067	8.30E-08
3h	ACA1_186860/clean/scf7180000084746:211879-211967:+	ACA1_186860	GRASP55_65 domain-containing protein [Source:UniProtKB/TrEMBL;Acc:L8H559]	0.003436433	0.48364	0.480203567	1.44E-07
3h	ACA1_062020/clean/scf7180000084599:1103533-1103653:+	ACA1_062020	Major vault protein alpha, putative [Source:UniProtKB/TrEMBL;Acc:L8GWT3]	0.00222284	0.482307	0.48008416	1.72E-07
3h	ACA1_054020/clean/scf7180000084594:374853-374928:-	ACA1_054020	hypothetical protein	7.39E-05	0.480142333	0.480068385	2.46E-11
3h	ACA1_216380/clean/scf7180000084758:483140-483253:+	ACA1_216380	Magnesium transporter [Source:UniProtKB/TrEMBL;Acc:L8GSQ2]	0.001759882	0.481770667	0.480010785	9.79E-10
3h	ACA1_039340/clean/scf7180000084569:28955-29070:-	ACA1_039340	hypothetical protein	0.02809981	0.508019	0.47991919	5.17E-05
3h	ACA1_064340/clean/scf7180000084599:1595826-1595930:-	ACA1_064340	VASt domain-containing protein [Source:UniProtKB/TrEMBL;Acc:L8GWR3]	0	0.479780333	0.479780333	8.85E-07
3h	ACA1_146470/clean/scf7180000084713:29945-30011:-	ACA1_146470	MORN repeatcontaining protein [Source:UniProtKB/TrEMBL;Acc:L8GV81]	0.0153343	0.494948667	0.479614367	8.93E-10
3h	ACA1_191520/clean/scf7180000084749:10693-10860:-	ACA1_191520	GDP-Man:Man(3)GlcNAc(2)-PP-Dol alpha-1,2-mannosyltransferase [Source:UniProtKB/TrEMBL;Acc:L8GNP5]	0.002851903	0.482133	0.479281097	3.69E-06
3h	ACA1_133550/clean/scf7180000084701:79089-79146:+	ACA1_133550	Lactamase_B domain-containing protein [Source:UniProtKB/TrEMBL;Acc:L8H3M7]	0.085148867	0.564095333	0.478946467	0.000133729
3h	ACA1_175300/clean/scf7180000084741:914599-914661:+	ACA1_175300	Zinc knuckle domain containing protein [Source:UniProtKB/TrEMBL;Acc:L8HKB8]	0	0.478845667	0.478845667	0.000340278
3h	ACA1_180160/clean/scf7180000084744:34572-34703:+	ACA1_180160	hypothetical protein	0.00707617	0.485914	0.47883783	1.12E-06
3h	ACA1_054250/clean/scf7180000084594:443992-444080:+	ACA1_054250	G_PROTEIN_RECEP_F2_4 domain-containing protein [Source:UniProtKB/TrEMBL;Acc:L8H5A3]	0.0656002	0.544341	0.4787408	0.000317531
3h	ACA1_287660/clean/scf7180000084777:57258-57335:+	ACA1_287660	Pentapeptide repeatcontaining protein [Source:UniProtKB/TrEMBL;Acc:L8HIP0]	0.003191713	0.481840333	0.47864862	1.98E-08
3h	ACA1_204530/clean/scf7180000084754:305340-305423:-	ACA1_204530	F-box domain-containing protein [Source:UniProtKB/TrEMBL;Acc:L8GTM3]	0.021367533	0.499956667	0.478589133	0.000847286
3h	ACA1_077090/clean/scf7180000084612:235055-235248:-	ACA1_077090	Transmembrane 9 superfamily member [Source:UniProtKB/TrEMBL;Acc:L8GLA1]	0.000623777	0.479071867	0.47844809	5.53E-10
3h	ACA1_178260/clean/scf7180000084743:193114-193265:+	ACA1_178260	Cytochrome P450, putative [Source:UniProtKB/TrEMBL;Acc:L8GTE4]	0.010356857	0.488690333	0.478333477	7.52E-09
3h	ACA1_063830/clean/scf7180000084599:1514650-1514755:+	ACA1_063830	hypothetical protein	0.007574947	0.485842333	0.478267387	1.70E-05
3h	ACA1_322160/clean/scf7180000084459:21575-21642:+	ACA1_322160	AB hydrolase-1 domain-containing protein [Source:UniProtKB/TrEMBL;Acc:L8GMT9]	0.243118333	0.721201667	0.478083333	0.001116997
3h	ACA1_260180/clean/scf7180000084769:452536-452630:+	ACA1_260180	Nitric oxide synthase-interacting protein homolog [Source:UniProtKB/TrEMBL;Acc:L8GFN7]	0.01434264	0.492414	0.47807136	3.43E-07
3h	ACA1_349660/clean/scf7180000084690:31077-31713:+	ACA1_349660	BAT1 protein [Source:UniProtKB/TrEMBL;Acc:L8GJC9]	0.122199433	0.600250333	0.4780509	4.15E-05
3h	ACA1_061010/clean/scf7180000084599:798761-798970:-	ACA1_061010	zf-Tim10_DDP domain-containing protein [Source:UniProtKB/TrEMBL;Acc:L8GXS4]	0.00799081	0.485980667	0.477989857	5.48E-08
3h	ACA1_024220/clean/scf7180000084441:21653-21736:+	ACA1_024220	Purinergic receptor, putative [Source:UniProtKB/TrEMBL;Acc:L8GLB7]	0.00844513	0.486421	0.47797587	4.46E-11
3h	ACA1_189340/clean/scf7180000084748:11515-11605:-	ACA1_189340	Dyptype peroxidase superfamily protein [Source:UniProtKB/TrEMBL;Acc:L8GDU2]	0.002198986	0.480157333	0.477958348	2.02E-12
3h	ACA1_264440/clean/scf7180000084770:287226-287290:+	ACA1_264440	Sulfurtransferase [Source:UniProtKB/TrEMBL;Acc:L8H202]	0.102965667	0.580898667	0.477933	8.35E-05
3h	ACA1_267810/clean/scf7180000084770:872973-873051:+	ACA1_267810	hypothetical protein	0.0370242	0.514884667	0.477860467	2.41E-06
3h	ACA1_287660/clean/scf7180000084777:56714-56803:+	ACA1_287660	Pentapeptide repeatcontaining protein [Source:UniProtKB/TrEMBL;Acc:L8HIP0]	0.088107767	0.565823	0.477715233	0.000184264
3h	ACA1_223040/clean/scf7180000084760:89378-89447:-	ACA1_223040	Protein kinase domain-containing protein [Source:UniProtKB/TrEMBL;Acc:L8GTT7]	0.025609067	0.503263	0.477653933	5.06E-06
3h	ACA1_378280/clean/scf7180000084711:229167-229294:+	ACA1_378280	hypothetical protein	0.080667433	0.558168333	0.4775009	7.52E-05
3h	ACA1_174040/clean/scf7180000084741:586167-586249:-	ACA1_174040	Protein kinase domain-containing protein [Source:UniProtKB/TrEMBL;Acc:L8HK27]	0	0.477426	0.477426	6.31E-08
3h	ACA1_282720/clean/scf7180000084776:724598-724666:-	ACA1_282720	Leucine rich repeat domain containing protein [Source:UniProtKB/TrEMBL;Acc:L8H9L8]	0.063734333	0.541124	0.477389667	0.000467559
3h	ACA1_068740/clean/scf7180000084603:268724-268797:+	ACA1_068740	tRNAdihydrouridine synthase A, putative [Source:UniProtKB/TrEMBL;Acc:L8HDM1]	0.0261294	0.503491333	0.477361933	0.000474183
3h	ACA1_141880/clean/scf7180000084708:42168-42260:+	ACA1_141880	RNA editing ligase [Source:UniProtKB/TrEMBL;Acc:L8HCM1]	0.008395633	0.485698	0.477302367	0.000151937
3h	ACA1_389460/clean/scf7180000084733:260501-260585:-	ACA1_389460	hypothetical protein	0.004986	0.482127	0.477141	2.16E-06
3h	ACA1_264710/clean/scf7180000084770:363441-363511:-	ACA1_264710	Peptidase_S8 domain-containing protein [Source:UniProtKB/TrEMBL;Acc:L8H251]	0.005252277	0.482086	0.476833723	8.20E-08
3h	ACA1_136780/clean/scf7180000084703:7077-7152:+	ACA1_136780	hypothetical protein	0.141852667	0.618588333	0.476735667	0.000796657
3h	ACA1_301240/clean/scf7180000083203:7657-7747:+	ACA1_301240	Usp domain-containing protein [Source:UniProtKB/TrEMBL;Acc:L8H1P7]	0.00659865	0.483263	0.47666435	1.53E-08
3h	ACA1_040450/clean/scf7180000084572:6990-7080:+	ACA1_040450	Carrier superfamily protein [Source:UniProtKB/TrEMBL;Acc:L8H0I1]	0.0489198	0.525563	0.4766432	8.80E-05
3h	ACA1_234460/clean/scf7180000084762:289735-289845:+	ACA1_234460	hypothetical protein	0.010680293	0.486811333	0.47613104	6.08E-11
3h	ACA1_040210/clean/scf7180000084571:14350-14437:+	ACA1_040210	Mitochondrial uncoupling protein [Source:UniProtKB/TrEMBL;Acc:L8GGI9]	0.006771177	0.482883667	0.47611249	3.50E-06
3h	ACA1_192960/clean/scf7180000084749:172629-172757:-	ACA1_192960	Multiple Ankyrin repeat domain containing protein [Source:UniProtKB/TrEMBL;Acc:L8GPD9]	0.005873423	0.481955333	0.47608191	2.24E-06
3h	ACA1_116020/clean/scf7180000084669:707018-707120:+	ACA1_116020	Rho-GAP domain-containing protein [Source:UniProtKB/TrEMBL;Acc:L8H6T0]	0.009674433	0.4856801	0.476005667	5.26E-07
3h	ACA1_117260/clean/scf7180000084669:964542-964615:+	ACA1_117260	hypothetical protein	0.322089667	0.797647333	0.475557667	0.001758363
3h	ACA1_279760/clean/scf7180000084776:441821-441902:-	ACA1_279760	PWI domain containing protein [Source:UniProtKB/TrEMBL;Acc:L8H642]	0.000295011	0.475701667	0.475406655	9.91E-10
3h	ACA1_264380/clean/scf7180000084770:255988-256087:-	ACA1_264380	S-formylglutathione hydrolase [Source:UniProtKB/TrEMBL;Acc:L8H1Z7]	0.00051534	0.475912667	0.475397327	3.15E-11
3h	ACA1_378130/clean/scf7180000084711:192570-192662:-	ACA1_378130	hypothetical protein	0.028165567	0.503491667	0.4753261	4.43E-05
3h	ACA1_197850/clean/scf7180000084753:39614-39697:-	ACA1_197850	HAD hydrolase, family IIA subfamily protein [Source:UniProtKB/TrEMBL;Acc:L8H335]	0.01827317	0.493546667	0.475273497	4.56E-07
3h	ACA1_113440/clean/scf7180000084669:277101-277221:+	ACA1_113440	hypothetical protein	0.00376848	0.478616667	0.474848187	4.76E-10
3h	ACA1_087900/clean/scf7180000084639:127387-127464:-	ACA1_087900	hypothetical protein	0.021302967	0.496149667	0.4748467	0.000167578
3h	ACA1_230530/clean/scf7180000084761:372175-372254:+	ACA1_230530	S(Hydroxymethyl)glutathione dehydrogenase/class III alcohol dehydrogenase [Source:UniProtKB/TrEMBL;Acc:L8H8X2]	0.002546297	0.476993667	0.47444737	1.02E-15
3h	ACA1_372660/clean/scf7180000084679:41403-41491:-	ACA1_372660	hypothetical protein	0.140763667	0.615205667	0.474442	0.007735266
3h	ACA1_018780/clean/scf7180000083912:16536-16630:-	ACA1_018780	AAA domain-containing protein [Source:UniProtKB/TrEMBL;Acc:L8HMV6]	0.013899097	0.488229	0.474329903	3.46E-07
3h	ACA1_034150/clean/scf7180000084561:36366-36471:-	ACA1_034150	hypothetical protein	0.007533915	0.481606	0.474072085	2.43E-06
3h	ACA1_323430/clean/scf7180000084468:21742-21820:+	ACA1_323430	SnoaL family polyketide cyclase [Source:UniProtKB/TrEMBL;Acc:L8H0W6]	0.000773542	0.474725	0.473951458	4.13E-12
3h	ACA1_025980/clean/scf7180000084456:1499-1568:-	ACA1_025980	hypothetical protein	0.040652467	0.514486667	0.4738342	5.67E-07
3h	ACA1_164920/clean/scf7180000084729:312405-312467:-	ACA1_164920	DJ-1_PfpI domain-containing protein [Source:UniProtKB/TrEMBL;Acc:L8GRN3]	0.005733383	0.479485667	0.473752283	4.40E-10
3h	ACA1_388440/clean/scf7180000084733:111608-111670:+	ACA1_388440	hypothetical protein	0.015802683	0.489492	0.473689317	1.46E-06
3h	ACA1_172940/clean/scf7180000084741:366868-367025:+	ACA1_172940	Protein kinase domain-containing protein [Source:UniProtKB/TrEMBL;Acc:L8HHJ2]	0.004819667	0.478443667	0.473624	0.000223553
3h	ACA1_224310/clean/scf7180000084760:166536-166616:+	ACA1_224310	PH domain-containing protein [Source:UniProtKB/TrEMBL;Acc:L8GTW7]	0.010087597	0.483468	0.473380403	3.19E-09
3h	ACA1_064490/clean/scf7180000084599:1628383-1628451:+	ACA1_064490	TYR_PHOSPHATASE_2 domain-containing protein [Source:UniProtKB/TrEMBL;Acc:L8GXK2]	0.063157267	0.536440333	0.473283067	0.000551478
3h	ACA1_215970/clean/scf7180000084758:418506-418598:-	ACA1_215970	EGFlike domain containing protein [Source:UniProtKB/TrEMBL;Acc:L8GQ73]	0.017749533	0.490954333	0.4732048	0.000632431
3h	ACA1_396240/clean/scf7180000084759:62195-62280:+	ACA1_396240	Transcription factor e2f/dimerization partner (Tdp) domain containing protein [Source:UniProtKB/TrEMBL;Acc:L8HDJ6]	0.009359923	0.482405667	0.473045743	7.46E-09
3h	ACA1_385370/clean/scf7180000084730:150991-151075:-	ACA1_385370	Drf_GBD domain-containing protein [Source:UniProtKB/TrEMBL;Acc:L8HBI2]	0.005369544	0.478373667	0.473004123	3.39E-06
3h	ACA1_150950/clean/scf7180000084716:34574-34656:+	ACA1_150950	Exocyst complex component Sec6 [Source:UniProtKB/TrEMBL;Acc:L8H215]	0.002273197	0.475228	0.472954803	1.12E-06
3h	ACA1_189410/clean/scf7180000084748:30838-30930:+	ACA1_189410	BTB/POZ domain containing protein [Source:UniProtKB/TrEMBL;Acc:L8GGG2]	0.035061433	0.507912	0.472850567	2.00E-05
3h	ACA1_127640/clean/scf7180000084696:39029-39136:+	ACA1_127640	GP-PDE domain-containing protein [Source:UniProtKB/TrEMBL;Acc:L8GEW1]	0.001080861	0.473926	0.472845139	2.41E-07
3h	ACA1_189640/clean/scf7180000084748:70712-70803:-	ACA1_189640	Bifunctional aspartate kinase/diaminopimelate decarboxylase protein [Source:UniProtKB/TrEMBL;Acc:L8GEY3]	0.0231216	0.495197	0.4720754	8.40E-09
3h	ACA1_074520/clean/scf7180000084608:30026-30107:+	ACA1_074520	SH3 domain containing protein [Source:UniProtKB/TrEMBL;Acc:L8HFD8]	0.046459067	0.518529333	0.472070267	1.53E-08
3h	ACA1_282190/clean/scf7180000084776:622680-622783:-	ACA1_282190	Synapsin, ATP binding domain containing protein [Source:UniProtKB/TrEMBL;Acc:L8H7E1]	0.00746738	0.479483667	0.472016287	2.28E-07
3h	ACA1_106720/clean/scf7180000084660:110842-110956:-	ACA1_106720	CID domain-containing protein [Source:UniProtKB/TrEMBL;Acc:L8GQ94]	0.136231	0.608231333	0.472000333	5.53E-05
3h	ACA1_096820/clean/scf7180000084645:1108374-1108494:-	ACA1_096820	hypothetical protein	0.002411303	0.4744033	0.471991997	4.18E-08
3h	ACA1_267170/clean/scf7180000084770:813552-813652:+	ACA1_267170	START domain-containing protein [Source:UniProtKB/TrEMBL;Acc:L8H406]	0.014569023	0.486531333	0.47196231	9.52E-10
3h	ACA1_313850/clean/scf7180000083835:964-1035:+	ACA1_313850	PAS, putative [Source:UniProtKB/TrEMBL;Acc:L8H077]	0.085567	0.557372667	0.471805667	0.004622172
3h	ACA1_264290/clean/scf7180000084770:229995-230073:-	ACA1_264290	Geranylgeranyl diphosphate synthase 1, putative [Source:UniProtKB/TrEMBL;Acc:L8H3G3]	0.004906777	0.476418333	0.471511557	3.39E-07
3h	ACA1_279380/clean/scf7180000084776:374375-374432:+	ACA1_279380	4HBT domain-containing protein [Source:UniProtKB/TrEMBL;Acc:L8H926]	0.182275667	0.653715	0.471439333	0.000467026
3h	ACA1_187380/clean/scf7180000084747:48354-48455:+	ACA1_187380	RING-type domain-containing protein [Source:UniProtKB/TrEMBL;Acc:L8GSD0]	6.54E-05	0.471465667	0.471400269	2.16E-07
3h	ACA1_027030/clean/scf7180000084464:27235-27352:-	ACA1_027030	C2 domain-containing protein [Source:UniProtKB/TrEMBL;Acc:L8GJ80]	0.025596667	0.496975	0.471378333	1.78E-06
3h	ACA1_150950/clean/scf7180000084716:33128-33270:+	ACA1_150950	Exocyst complex component Sec6 [Source:UniProtKB/TrEMBL;Acc:L8H215]	0.0523969	0.523772667	0.471375767	1.20E-05
3h	ACA1_203070/clean/scf7180000084754:135341-135436:+	ACA1_203070	Fbox domain containing protein [Source:UniProtKB/TrEMBL;Acc:L8GUJ5]	0.015800773	0.487143333	0.47134256	1.11E-07
3h	ACA1_123890/clean/scf7180000084686:80636-80715:-	ACA1_123890	hypothetical protein	0.062237133	0.533451333	0.4712142	0.000836815
3h	ACA1_202680/clean/scf7180000084754:74546-74607:-	ACA1_202680	DENN (AEX3) domain containing protein [Source:UniProtKB/TrEMBL;Acc:L8GTF2]	0.00141013	0.472307	0.47089687	7.36E-08
3h	ACA1_046390/clean/scf7180000084577:84893-85000:-	ACA1_046390	hypothetical protein	0.001571453	0.472449333	0.47087788	5.14E-06
3h	ACA1_072940/clean/scf7180000084603:1338242-1338337:+	ACA1_072940	Protein kinase domain-containing protein [Source:UniProtKB/TrEMBL;Acc:L8HET0]	0.007246367	0.478122667	0.4708763	8.90E-06
3h	ACA1_046590/clean/scf7180000084577:149263-149343:+	ACA1_046590	EnoylCoA hydratase/isomerase, putative [Source:UniProtKB/TrEMBL;Acc:L8H8U0]	0.011980093	0.482805667	0.470825573	2.91E-07
3h	ACA1_301860/clean/scf7180000083222:44246-44344:+	ACA1_301860	Phosphatidylinositol 3-and 4-kinase [Source:UniProtKB/TrEMBL;Acc:L8HHB0]	0.004347833	0.474914	0.470566167	8.33E-10
3h	ACA1_169100/clean/scf7180000084739:22157-22247:+	ACA1_169100	Dual specificity phosphatase, catalytic domain containing protein [Source:UniProtKB/TrEMBL;Acc:L8HG79]	0.01057572	0.481041333	0.470465613	7.47E-10
3h	ACA1_368230/clean/scf7180000084664:85448-85533:-	ACA1_368230	C2 domain-containing protein [Source:UniProtKB/TrEMBL;Acc:L8GYW6]	0	0.470246667	0.470246667	3.80E-05
3h	ACA1_234260/clean/scf7180000084762:255364-255470:+	ACA1_234260	DH domain-containing protein [Source:UniProtKB/TrEMBL;Acc:L8H2M0]	0.018309373	0.488525	0.470215627	5.69E-07
3h	ACA1_176060/clean/scf7180000084741:1116941-1117028:+	ACA1_176060	DAGKc domain-containing protein [Source:UniProtKB/TrEMBL;Acc:L8HJR7]	0.07356	0.543713667	0.470153667	2.79E-06
3h	ACA1_061500/clean/scf7180000084599:944837-944916:+	ACA1_061500	Ras-GAP domain-containing protein [Source:UniProtKB/TrEMBL;Acc:L8GWM5]	0.00211119	0.472259333	0.470148144	4.76E-08
3h	ACA1_287660/clean/scf7180000084777:57007-57073:+	ACA1_287660	Pentapeptide repeatcontaining protein [Source:UniProtKB/TrEMBL;Acc:L8HIP0]	0.005964063	0.476058667	0.470094603	4.97E-09
3h	ACA1_252560/clean/scf7180000084768:181722-181799:+	ACA1_252560	hypothetical protein	0.0405092	0.510493333	0.469984133	4.95E-05
3h	ACA1_252460/clean/scf7180000084768:156364-156440:-	ACA1_252460	Isovaleryl Coenzyme A dehydrogenase [Source:UniProtKB/TrEMBL;Acc:L8HD26]	0	0.469894667	0.469894667	1.15E-11
3h	ACA1_233870/clean/scf7180000084762:173852-173957:-	ACA1_233870	hypothetical protein	0.005348047	0.474935667	0.46958762	2.34E-06
3h	ACA1_116740/clean/scf7180000084669:839406-839489:+	ACA1_116740	Methyltransf_2 domain-containing protein [Source:UniProtKB/TrEMBL;Acc:L8H4R1]	0.064383567	0.533864	0.469480433	0.000162225
3h	ACA1_031790/clean/scf7180000084523:3510-3590:+	ACA1_031790	Rap-GAP domain-containing protein [Source:UniProtKB/TrEMBL;Acc:L8H1P5]	0.0258502	0.495065333	0.469215133	7.81E-06
3h	ACA1_183960/clean/scf7180000084745:528936-529022:-	ACA1_183960	Phosphoesterase family protein [Source:UniProtKB/TrEMBL;Acc:L8H8F6]	0.002674216	0.471883333	0.469209117	1.37E-08
3h	ACA1_098950/clean/scf7180000084646:73861-73927:+	ACA1_098950	hypothetical protein	0.011055717	0.479879667	0.46882395	1.86E-08
3h	ACA1_061680/clean/scf7180000084599:1009569-1009660:+	ACA1_061680	PH domain containing protein [Source:UniProtKB/TrEMBL;Acc:L8GXZ1]	0.006528522	0.475264	0.468735478	3.12E-06
3h	ACA1_071970/clean/scf7180000084603:1118651-1118742:+	ACA1_071970	Carn_acyltransf domain-containing protein [Source:UniProtKB/TrEMBL;Acc:L8HEI6]	0.00672693	0.475454	0.46872707	3.90E-07
3h	ACA1_091960/clean/scf7180000084645:236987-237087:-	ACA1_091960	hypothetical protein	0.019874633	0.488357	0.468482367	2.43E-05
3h	ACA1_246780/clean/scf7180000084763:580874-580955:-	ACA1_246780	Ras subfamily protein [Source:UniProtKB/TrEMBL;Acc:L8GK82]	0.0230361	0.491506333	0.468470233	5.50E-11
3h	ACA1_332500/clean/scf7180000084579:30639-30752:-	ACA1_332500	hypothetical protein	0.00191689	0.470292333	0.468375443	2.79E-06
3h	ACA1_107450/clean/scf7180000084660:199711-199784:-	ACA1_107450	PB1 domain-containing protein [Source:UniProtKB/TrEMBL;Acc:L8GNC1]	0.010346333	0.478705	0.468358667	0.00015832
3h	ACA1_063740/clean/scf7180000084599:1493710-1493799:-	ACA1_063740	Transmembrane protein [Source:UniProtKB/TrEMBL;Acc:L8GXB1]	0.007619033	0.475972333	0.4683533	4.82E-06
3h	ACA1_031950/clean/scf7180000084523:39720-39829:+	ACA1_031950	CBS domain containing protein [Source:UniProtKB/TrEMBL;Acc:L8H414]	0.027187967	0.495417	0.468229033	0.000697892
3h	ACA1_091560/clean/scf7180000084645:149389-149458:+	ACA1_091560	UBP-type domain-containing protein [Source:UniProtKB/TrEMBL;Acc:L8GKE3]	0.1396109	0.607770333	0.468159433	0.001194387
3h	ACA1_200210/clean/scf7180000084753:414446-414510:+	ACA1_200210	hypothetical protein	0.019255853	0.487317667	0.468061813	6.06E-08
3h	ACA1_112000/clean/scf7180000084669:80725-80858:+	ACA1_112000	Rho-GAP domain-containing protein [Source:UniProtKB/TrEMBL;Acc:L8H3W7]	0.000125417	0.468129	0.468003583	1.04E-08
3h	ACA1_140610/clean/scf7180000084707:96789-96876:-	ACA1_140610	Poly [ADP-ribose] polymerase [Source:UniProtKB/TrEMBL;Acc:L8GGG7]	0.096868067	0.564325667	0.4674576	0.001283948
3h	ACA1_300860/clean/scf7180000083147:9958-10040:+	ACA1_300860	TFIIB-type domain-containing protein [Source:UniProtKB/TrEMBL;Acc:L8HHA5]	0.076840467	0.544208667	0.4673682	2.42E-05
3h	ACA1_400210/clean/scf7180000084775:120658-120909:-	ACA1_400210	Non-specific serine/threonine protein kinase [Source:UniProtKB/TrEMBL;Acc:L8GIE6]	0.006014497	0.473298	0.467283503	4.74E-08
3h	ACA1_179960/clean/scf7180000084744:9775-9950:-	ACA1_179960	Protein kinase domain/MORN repeatcontaining protein [Source:UniProtKB/TrEMBL;Acc:L8GC88]	0.001988334	0.468901333	0.466912999	1.20E-08
3h	ACA1_367890/clean/scf7180000084664:9160-9276:-	ACA1_367890	hypothetical protein	0.047738167	0.514615333	0.466877167	4.34E-06
3h	ACA1_062120/clean/scf7180000084599:1124119-1124212:+	ACA1_062120	RING-type domain-containing protein [Source:UniProtKB/TrEMBL;Acc:L8GWU5]	0.013149337	0.479796667	0.46664733	5.78E-07
3h	ACA1_266140/clean/scf7180000084770:612202-612275:-	ACA1_266140	CBAH domain-containing protein [Source:UniProtKB/TrEMBL;Acc:L8H3S3]	0.00976749	0.476310667	0.466543177	1.43E-09
3h	ACA1_391680/clean/scf7180000084740:242626-242740:-	ACA1_391680	Squalene and phytoene synthases superfamily protein [Source:UniProtKB/TrEMBL;Acc:L8GRM8]	0.026954707	0.493495667	0.46654096	4.55E-05
3h	ACA1_170640/clean/scf7180000084739:99812-99895:+	ACA1_170640	Protein kinase domain-containing protein [Source:UniProtKB/TrEMBL;Acc:L8HIR2]	0.014082433	0.480385333	0.4663029	2.86E-05
3h	ACA1_136190/clean/scf7180000084702:150505-150600:+	ACA1_136190	SER_THR_PHOSPHATASE domain-containing protein [Source:UniProtKB/TrEMBL;Acc:L8GF93]	0.027719267	0.493969667	0.4662504	1.01E-06
3h	ACA1_384860/clean/scf7180000084730:117424-117503:+	ACA1_384860	Isochorismatase domain-containing protein [Source:UniProtKB/TrEMBL;Acc:L8H995]	0.003697447	0.469894667	0.46619722	1.89E-11
3h	ACA1_271290/clean/scf7180000084771:12075-12196:-	ACA1_271290	hypothetical protein	0.009872037	0.476024333	0.466152297	2.41E-10
3h	ACA1_164680/clean/scf7180000084729:248155-248375:-	ACA1_164680	ANK_REP_REGION domain-containing protein [Source:UniProtKB/TrEMBL;Acc:L8GSN8]	0.011738317	0.477874367	0.46613605	4.67E-07
3h	ACA1_188010/clean/scf7180000084747:103555-103652:+	ACA1_188010	Importin 13, putative [Source:UniProtKB/TrEMBL;Acc:L8GRR2]	0.004334367	0.470375333	0.466040967	5.09E-06
3h	ACA1_085210/clean/scf7180000084632:74539-74613:+	ACA1_085210	ThiF domain-containing protein [Source:UniProtKB/TrEMBL;Acc:L8HKE8]	0.01183576	0.477824333	0.465988573	2.22E-10
3h	ACA1_215970/clean/scf7180000084758:421221-421292:-	ACA1_215970	EGFlike domain containing protein [Source:UniProtKB/TrEMBL;Acc:L8GQ73]	0.017543867	0.483484333	0.465940467	0.000123333
3h	ACA1_287960/clean/scf7180000084777:134226-134309:-	ACA1_287960	SET domain-containing protein [Source:UniProtKB/TrEMBL;Acc:L8HIS3]	0.005306387	0.471235	0.465928613	6.22E-05
3h	ACA1_185180/clean/scf7180000084746:82109-82187:-	ACA1_185180	LIM zinc-binding domain-containing protein [Source:UniProtKB/TrEMBL;Acc:L8H575]	0.004329853	0.470243333	0.46591348	1.69E-09
3h	ACA1_219750/clean/scf7180000084758:799849-799936:+	ACA1_219750	FAD_binding_3 domain-containing protein [Source:UniProtKB/TrEMBL;Acc:L8GPZ6]	0.001701493	0.467525	0.465823507	8.54E-08
3h	ACA1_060930/clean/scf7180000084599:782725-782872:+	ACA1_060930	RFX-type winged-helix domain-containing protein [Source:UniProtKB/TrEMBL;Acc:L8GWH8]	0.035765067	0.501476233	0.465711167	7.79E-06
3h	ACA1_191150/clean/scf7180000084748:186959-187031:+	ACA1_191150	Triosephosphate isomerase [Source:UniProtKB/TrEMBL;Acc:L8GE90]	0.002906797	0.468589	0.465682203	2.66E-10
3h	ACA1_170960/clean/scf7180000084741:25616-25727:+	ACA1_170960	hypothetical protein	0.028048033	0.493706333	0.4656583	2.00E-06

3h	ACA1_199940/clean/scf7180000084753:351334-351426:+	ACA1_199940	hypothetical protein	0.115600333	0.581116333	0.465516	0.003367359
3h	ACA1_291600/clean/scf7180000084777:784568-784671:-	ACA1_291600	Macro domain-containing protein [Source:UniProtKB/TrEMBL;Acc:L8HJG2]	0.008913287	0.474312	0.465398713	2.88E-07
3h	ACA1_074110/clean/scf7180000084605:26973-27054:-	ACA1_074110	Peptidase [Source:UniProtKB/TrEMBL;Acc:L8HJ70]	0.004138498	0.469518333	0.465379835	1.08E-07
3h	ACA1_122710/clean/scf7180000084682:151823-151897:-	ACA1_122710	hypothetical protein	0.01829227	0.483627333	0.465335063	8.78E-08
3h	ACA1_199520/clean/scf7180000084753:254433-254519:+	ACA1_199520	hypothetical protein	0.008702487	0.474031333	0.465328847	5.18E-08
3h	ACA1_062410/clean/scf7180000084599:1180401-1180476:-	ACA1_062410	Vacuolar fusion protein ccz1 family protein [Source:UniProtKB/TrEMBL;Acc:L8GY76]	0.021023527	0.486351	0.465327473	2.81E-06
3h	ACA1_055390/clean/scf7180000084594:693398-693471:-	ACA1_055390	hypothetical protein	0.0177448	0.483004667	0.465259867	0.000906215
3h	ACA1_011270/clean/scf7180000083623:4881-4950:-	ACA1_011270	FYVE-type domain-containing protein [Source:UniProtKB/TrEMBL;Acc:L8GQN9]	0.075800733	0.541037	0.465236267	0.006745661
3h	ACA1_180150/clean/scf7180000084744:32787-32927:-	ACA1_180150	Protein kinase domain-containing protein [Source:UniProtKB/TrEMBL;Acc:L8GDP6]	0.014203227	0.479411333	0.465208107	2.22E-08
3h	ACA1_039450/clean/scf7180000084569:31765-31840:-	ACA1_039450	SAP domain-containing protein [Source:UniProtKB/TrEMBL;Acc:L8HI99]	0.017766683	0.482774333	0.46500765	0.000220512
3h	ACA1_065040/clean/scf7180000084599:1758677-1758747:-	ACA1_065040	SIT4 phosphatase-associated protein [Source:UniProtKB/TrEMBL;Acc:L8GXP9]	0.005527173	0.470424333	0.46489716	9.01E-07
3h	ACA1_043610/clean/scf7180000084573:183936-184024:-	ACA1_043610	FYVE-type domain-containing protein [Source:UniProtKB/TrEMBL;Acc:L8GV86]	0.055427233	0.520299333	0.4648721	0.00032545
3h	ACA1_396730/clean/scf7180000084759:110114-110211:+	ACA1_396730	Conserved oligomeric Golgi complex subunit 6 [Source:UniProtKB/TrEMBL;Acc:L8HC92]	0.146952	0.61166	0.464708	0.000317924
3h	ACA1_234060/clean/scf7180000084762:191723-191789:-	ACA1_234060	SPRY domain containing protein [Source:UniProtKB/TrEMBL;Acc:L8H2J5]	0.038233967	0.502805	0.464571033	8.56E-06
3h	ACA1_113870/clean/scf7180000084669:392225-392331:+	ACA1_113870	CUB domain containing protein [Source:UniProtKB/TrEMBL;Acc:L8H6F7]	0.000602773	0.465171933	0.46456916	1.28E-07
3h	ACA1_300850/clean/scf7180000083147:7438-7517:+	ACA1_300850	WD_REPEATS_REGION domain-containing protein [Source:UniProtKB/TrEMBL;Acc:L8HF88]	0.013623492	0.478142667	0.464519175	5.52E-07
3h	ACA1_154230/clean/scf7180000084722:28173-28267:+	ACA1_154230	hypothetical protein	0.011347273	0.475746333	0.46439906	1.40E-12
3h	ACA1_048870/clean/scf7180000084583:6274-6347:-	ACA1_048870	hypothetical protein	0.023204277	0.487555	0.464350723	1.10E-06
3h	ACA1_193110/clean/scf7180000084749:185314-185482:-	ACA1_193110	hypothetical protein	0.0561401	0.520276667	0.464136567	6.65E-07
3h	ACA1_325610/clean/scf7180000084495:50077-50181:-	ACA1_325610	Protein kinase domain containing protein [Source:UniProtKB/TrEMBL;Acc:L8H900]	0.008359903	0.472414333	0.46405443	2.60E-06
3h	ACA1_116740/clean/scf7180000084669:839211-839288:+	ACA1_116740	Methyltransf_2 domain-containing protein [Source:UniProtKB/TrEMBL;Acc:L8H4R1]	0.0650368	0.528860667	0.463823867	0.000455882
3h	ACA1_011280/clean/scf7180000083623:8206-8281:+	ACA1_011280	AAA domain-containing protein [Source:UniProtKB/TrEMBL;Acc:L8GP61]	0.001102427	0.464793667	0.46369124	1.86E-07
3h	ACA1_365710/clean/scf7180000084641:299364-299447:-	ACA1_365710	Protein kinase domain-containing protein [Source:UniProtKB/TrEMBL;Acc:L8GPB8]	0.020246633	0.483889333	0.4636427	4.75E-07
3h	ACA1_122370/clean/scf7180000084682:135028-135112:-	ACA1_122370	Short chain oxidoreductase [Source:UniProtKB/TrEMBL;Acc:L8GEE8]	0.0881449	0.551768333	0.463623433	6.48E-06
3h	ACA1_066750/clean/scf7180000084600:38578-38661:-	ACA1_066750	F-box domain-containing protein [Source:UniProtKB/TrEMBL;Acc:L8GNT1]	0.02693834	0.490531667	0.463593327	0.000641954
3h	ACA1_146440/clean/scf7180000084713:25533-25631:+	ACA1_146440	Methyltransf_11 domain-containing protein [Source:UniProtKB/TrEMBL;Acc:L8GU12]	0.075045733	0.538634	0.463588267	0.000144712
3h	ACA1_197890/clean/scf7180000084753:47808-47914:+	ACA1_197890	hypothetical protein	0.0037679	0.467255667	0.463487767	2.06E-07
3h	ACA1_140730/clean/scf7180000084707:108943-109027:-	ACA1_140730	Kelch repeat protein [Source:UniProtKB/TrEMBL;Acc:L8GGS1]	0.0083981	0.471853333	0.463455233	1.89E-06
3h	ACA1_042070/clean/scf7180000084573:53726-53833:+	ACA1_042070	Dual specificity protein kinase [Source:UniProtKB/TrEMBL;Acc:L8GW91]	0.1122398	0.575663	0.4634232	0.000124413
3h	ACA1_260890/clean/scf7180000084769:548509-548616:+	ACA1_260890	F-box domain-containing protein [Source:UniProtKB/TrEMBL;Acc:L8GFC7]	0.008904653	0.472322333	0.46341768	1.86E-05
3h	ACA1_309500/clean/scf7180000083762:10938-11013:+	ACA1_309500	Pyridine nucleotidedisulfide oxidoreductase domain containing protein [Source:UniProtKB/TrEMBL;Acc:L8GI23]	0.028983633	0.492365667	0.463382033	3.15E-09
3h	ACA1_129920/clean/scf7180000084698:49667-49722:+	ACA1_129920	SET domain-containing protein [Source:UniProtKB/TrEMBL;Acc:L8GPI9]	0.158470667	0.621766333	0.463295667	0.001180819
3h	ACA1_296490/clean/scf7180000084777:1360507-1360647:-	ACA1_296490	Succinate--CoA ligase [ADP-forming] subunit beta, mitochondrial [Source:UniProtKB/TrEMBL;Acc:L8HMD9]	0.002882762	0.466116333	0.463233571	6.76E-11
3h	ACA1_089960/clean/scf7180000084639:403854-403923:-	ACA1_089960	Dual specificity protein kinase [Source:UniProtKB/TrEMBL;Acc:L8GVS6]	0.017855167	0.481014333	0.463159167	6.68E-07
3h	ACA1_007740/clean/scf7180000083166:5829-5922:-	ACA1_007740	RasGEF domain containing protein [Source:UniProtKB/TrEMBL;Acc:L8HBV5]	0.249947	0.712982333	0.463035333	0.002864476
3h	ACA1_106000/clean/scf7180000084660:52967-53058:+	ACA1_106000	HEAT repeat domain containing protein [Source:UniProtKB/TrEMBL;Acc:L8GN71]	0.009349577	0.472157	0.462807423	2.16E-08
3h	ACA1_288630/clean/scf7180000084777:253003-253094:-	ACA1_288630	Mediator of RNA polymerase II transcription subunit 20 [Source:UniProtKB/TrEMBL;Acc:L8HIV7]	0.092992167	0.555619	0.462626833	9.85E-05
3h	ACA1_133480/clean/scf7180000084701:64308-64391:+	ACA1_133480	hypothetical protein	0.015216123	0.477667667	0.462451543	3.91E-07
3h	ACA1_054460/clean/scf7180000084594:500282-500388:-	ACA1_054460	Cytochrome b5 heme-binding domain-containing protein [Source:UniProtKB/TrEMBL;Acc:L8H6D5]	0.002138027	0.464530333	0.462392307	2.33E-14
3h	ACA1_389430/clean/scf7180000084733:253302-253384:+	ACA1_389430	hypothetical protein	0.0898203	0.552032667	0.462212367	4.16E-05
3h	ACA1_164920/clean/scf7180000084729:311774-311845:-	ACA1_164920	DJ-1_PfpI domain-containing protein [Source:UniProtKB/TrEMBL;Acc:L8GRN3]	0.01361096	0.475811333	0.462200373	1.48E-09
3h	ACA1_106940/clean/scf7180000084660:151224-151352:-	ACA1_106940	TPT domain-containing protein [Source:UniProtKB/TrEMBL;Acc:L8GNI5]	0.007565313	0.469687667	0.462122353	1.16E-07
3h	ACA1_204620/clean/scf7180000084754:322549-322759:+	ACA1_204620	hypothetical protein	0.131481067	0.593267333	0.461786267	0.005443429
3h	ACA1_233000/clean/scf7180000084762:98659-98763:-	ACA1_233000	Fatty acid betaoxidation-related protein [Source:UniProtKB/TrEMBL;Acc:L8H2F3]	0.012108033	0.473880333	0.4617723	5.59E-12
3h	ACA1_210530/clean/scf7180000084758:48860-48958:-	ACA1_210530	4Fe-4S ferredoxin-type domain-containing protein [Source:UniProtKB/TrEMBL;Acc:L8GS75]	0.000678493	0.462224667	0.461546173	2.37E-07
3h	ACA1_247260/clean/scf7180000084763:659906-659981:+	ACA1_247260	SET domain-containing protein [Source:UniProtKB/TrEMBL;Acc:L8GKH1]	0.00514126	0.466672333	0.461531073	1.21E-10
3h	ACA1_162500/clean/scf7180000084726:49146-49243:-	ACA1_162500	31.5 kDa midgut protein [Source:UniProtKB/TrEMBL;Acc:L8GYK7]	0.00394143	0.465459333	0.461517903	2.06E-07
3h	ACA1_043320/clean/scf7180000084573:164658-164730:+	ACA1_043320	Ubiquitin-like domain-containing protein [Source:UniProtKB/TrEMBL;Acc:L8GXV7]	0	0.461474333	0.461474333	0.038638102
3h	ACA1_288590/clean/scf7180000084777:247151-247243:+	ACA1_288590	RING-type domain-containing protein [Source:UniProtKB/TrEMBL;Acc:L8HKF4]	0.0406305	0.502030333	0.461399833	4.87E-05
3h	ACA1_191700/clean/scf7180000084749:33276-33365:-	ACA1_191700	Aldo_ket_red domain-containing protein [Source:UniProtKB/TrEMBL;Acc:L8GNA1]	0.043922767	0.505062	0.461139233	0.000348536
3h	ACA1_033610/clean/scf7180000084559:58270-58352:+	ACA1_033610	Sortilin [Source:UniProtKB/TrEMBL;Acc:L8GHJ0]	0.002369903	0.463021	0.460651097	7.53E-08
3h	ACA1_313620/clean/scf7180000083822:14470-14562:-	ACA1_313620	DUF4461 domain-containing protein [Source:UniProtKB/TrEMBL;Acc:L8GLZ5]	0.009370053	0.470006	0.460635947	5.13E-06
3h	ACA1_096400/clean/scf7180000084645:1004659-1004734:+	ACA1_096400	Methyltransf_11 domain-containing protein [Source:UniProtKB/TrEMBL;Acc:L8GJF1]	0.006420357	0.466936667	0.46051631	8.83E-09
3h	ACA1_321060/clean/scf7180000084435:30853-31235:-	ACA1_321060	hypothetical protein	0.209085667	0.669601333	0.460515667	0.001136225
3h	ACA1_264440/clean/scf7180000084770:287523-287589:+	ACA1_264440	Sulfurtransferase [Source:UniProtKB/TrEMBL;Acc:L8H202]	0.015766033	0.476147333	0.4603813	1.10E-07
3h	ACA1_066030/clean/scf7180000084599:1976375-1976451:-	ACA1_066030	hypothetical protein	0	0.460373667	0.460373667	2.01E-08
3h	ACA1_063970/clean/scf7180000084599:1547310-1547404:-	ACA1_063970	SH3 domain containing protein [Source:UniProtKB/TrEMBL;Acc:L8GYN6]	0.001551173	0.461304	0.459752827	1.65E-09
3h	ACA1_024020/clean/scf7180000084440:5771-5842:-	ACA1_024020	RhoGAP domain containing protein [Source:UniProtKB/TrEMBL;Acc:L8GJP9]	0	0.459637	0.459637	4.33E-09
3h	ACA1_011280/clean/scf7180000083623:7698-7768:+	ACA1_011280	AAA domain-containing protein [Source:UniProtKB/TrEMBL;Acc:L8GP61]	0.0057483	0.465356	0.4596077	4.76E-07
3h	ACA1_193130/clean/scf7180000084749:193343-193451:-	ACA1_193130	Metallophos domain-containing protein [Source:UniProtKB/TrEMBL;Acc:L8GN68]	0.002148453	0.461752333	0.45960388	4.09E-08
3h	ACA1_048870/clean/scf7180000084583:6870-6969:-	ACA1_048870	hypothetical protein	0.0183252	0.477879	0.4595538	2.80E-07
3h	ACA1_018120/clean/scf7180000083885:10839-10910:+	ACA1_018120	hypothetical protein	0	0.459515667	0.459515667	1.04E-07
3h	ACA1_109730/clean/scf7180000084663:11630-11733:-	ACA1_109730	Replication factor C subunit 1 [Source:UniProtKB/TrEMBL;Acc:L8HIF4]	0.004869067	0.46429	0.459420933	3.70E-07
3h	ACA1_091750/clean/scf7180000084645:196695-196827:-	ACA1_091750	hypothetical protein	0.01050795	0.469758667	0.459250717	7.06E-07
3h	ACA1_045680/clean/scf7180000084576:174319-174407:-	ACA1_045680	hypothetical protein	0.021971433	0.481167	0.459195567	0.000345598
3h	ACA1_123220/clean/scf7180000084686:6285-6394:-	ACA1_123220	hypothetical protein	0.004975133	0.464119	0.459143867	9.33E-06
3h	ACA1_170640/clean/scf7180000084739:98781-98870:+	ACA1_170640	Protein kinase domain-containing protein [Source:UniProtKB/TrEMBL;Acc:L8HIR2]	0.00624725	0.4653892	0.45914195	4.41E-05
3h	ACA1_140770/clean/scf7180000084707:115771-115842:+	ACA1_140770	ATPase, AAA domain containing protein [Source:UniProtKB/TrEMBL;Acc:L8GJ22]	0.00285144	0.461933	0.45908156	4.38E-07
3h	ACA1_359700/clean/scf7180000084622:5468-5539:-	ACA1_359700	Thioredoxin domain-containing protein [Source:UniProtKB/TrEMBL;Acc:L8HCQ3]	0.006961617	0.465840667	0.45887905	8.70E-12
3h	ACA1_259480/clean/scf7180000084769:348887-349024:-	ACA1_259480	EnoylCoA hydratase/isomerase [Source:UniProtKB/TrEMBL;Acc:L8GFL3]	0.001485714	0.460057667	0.458571952	6.65E-11
3h	ACA1_146440/clean/scf7180000084713:25343-25426:+	ACA1_146440	Methyltransf_11 domain-containing protein [Source:UniProtKB/TrEMBL;Acc:L8GU12]	0.032605233	0.491087333	0.4584821	1.08E-05
3h	ACA1_306840/clean/scf7180000083668:17533-17628:+	ACA1_306840	RTP1_C1 domain-containing protein [Source:UniProtKB/TrEMBL;Acc:L8GQF6]	0	0.458242667	0.458242667	5.26E-06
3h	ACA1_103600/clean/scf7180000084653:42540-42629:+	ACA1_103600	BTB domain-containing protein [Source:UniProtKB/TrEMBL;Acc:L8GD31]	0.008688663	0.466710667	0.458022003	2.51E-06
3h	ACA1_121250/clean/scf7180000084682:12450-12541:-	ACA1_121250	hypothetical protein	0.012417603	0.470176333	0.45775873	3.04E-06
3h	ACA1_376720/clean/scf7180000084685:145825-145947:-	ACA1_376720	LIM zinc-binding domain-containing protein [Source:UniProtKB/TrEMBL;Acc:L8HHL9]	0.00495931	0.462710667	0.457751357	1.72E-13
3h	ACA1_380930/clean/scf7180000084712:118788-118863:-	ACA1_380930	hypothetical protein	0.005929423	0.463501667	0.457572243	2.49E-07
3h	ACA1_065830/clean/scf7180000084599:1920051-1920128:-	ACA1_065830	PH domain-containing protein [Source:UniProtKB/TrEMBL;Acc:L8GYZ9]	0.01667	0.474022333	0.457352333	5.97E-06
3h	ACA1_208120/clean/scf7180000084757:108039-108131:+	ACA1_208120	hypothetical protein	0.00485479	0.461873	0.45701821	8.86E-14
3h	ACA1_227780/clean/scf7180000084761:135981-136077:+	ACA1_227780	NAD(P)-bd_dom domain-containing protein [Source:UniProtKB/TrEMBL;Acc:L8H8P3]	0.016862433	0.473662233	0.4567998	2.81E-05
3h	ACA1_037040/clean/scf7180000084567:38954-39016:-	ACA1_037040	hypothetical protein	0.010902227	0.467669	0.456766773	8.70E-06
3h	ACA1_384860/clean/scf7180000084730:117276-117365:+	ACA1_384860	Isochorismatase domain-containing protein [Source:UniProtKB/TrEMBL;Acc:L8H995]	0.00341274	0.460036	0.45662326	4.21E-11
3h	ACA1_310760/clean/scf7180000083780:7543-7608:+	ACA1_310760	hypothetical protein	0.071114133	0.52731	0.456195867	0.000785872
3h	ACA1_191490/clean/scf7180000084749:2550-2612:-	ACA1_191490	RTR1-type domain-containing protein [Source:UniProtKB/TrEMBL;Acc:L8GQP2]	0.007423894	0.463595333	0.45617144	9.59E-09
3h	ACA1_123220/clean/scf7180000084686:6076-6188:-	ACA1_123220	hypothetical protein	0.006367147	0.462402333	0.456035187	9.66E-06
3h	ACA1_265960/clean/scf7180000084770:554280-554391:-	ACA1_265960	Mac domain-containing protein [Source:UniProtKB/TrEMBL;Acc:L8H4H0]	0.0129707	0.468954	0.4559833	1.71E-07
3h	ACA1_369600/clean/scf7180000084664:343551-343618:+	ACA1_369600	PH domain containing protein [Source:UniProtKB/TrEMBL;Acc:L8GZ46]	0.019681777	0.475398333	0.455716557	2.56E-06
3h	ACA1_224950/clean/scf7180000084760:290473-290588:-	ACA1_224950	ANK_REP_REGION domain-containing protein [Source:UniProtKB/TrEMBL;Acc:L8GSF6]	0.019940537	0.475597333	0.455656797	0.000585524
3h	ACA1_048880/clean/scf7180000084583:10197-10268:+	ACA1_048880	LIM zinc-binding domain-containing protein [Source:UniProtKB/TrEMBL;Acc:L8HKD0]	0.0703971	0.526029667	0.455632567	2.84E-06
3h	ACA1_164650/clean/scf7180000084729:242723-242801:+	ACA1_164650	ATPdependent DNA helicase, RecQ subfamily protein [Source:UniProtKB/TrEMBL;Acc:L8GRL0]	0.033540067	0.489130333	0.455590267	0.000119051
3h	ACA1_101470/clean/scf7180000084649:69592-69696:+	ACA1_101470	Dynactin 4 isoform 2, putative [Source:UniProtKB/TrEMBL;Acc:L8GIW5]	0.017004483	0.472579	0.455574517	4.66E-08
3h	ACA1_143760/clean/scf7180000084709:91761-91824:-	ACA1_143760	hypothetical protein	0	0.455555667	0.455555667	7.01E-09
3h	ACA1_396260/clean/scf7180000084759:66490-66584:+	ACA1_396260	hypothetical protein	0.012255623	0.467749333	0.45549371	9.69E-07
3h	ACA1_046700/clean/scf7180000084577:171986-172131:+	ACA1_046700	Translocase of inner mitochondrial membrane 17, putative [Source:UniProtKB/TrEMBL;Acc:L8H9T6]	0.00906779	0.464506	0.45543821	3.07E-08
3h	ACA1_199500/clean/scf7180000084753:248790-248907:+	ACA1_199500	1acylglycerophosphocholine O-acyltransferase 1, putative [Source:UniProtKB/TrEMBL;Acc:L8H4L3]	0.041198837	0.496569667	0.45537083	0.001752885
3h	ACA1_036890/clean/scf7180000084567:7021-7111:+	ACA1_036890	Ras subfamily protein [Source:UniProtKB/TrEMBL;Acc:L8H0T2]	0.048001567	0.503264667	0.4552631	4.76E-06
3h	ACA1_171280/clean/scf7180000084741:63075-63177:+	ACA1_171280	Nicotinate phosphoribosyltransferase [Source:UniProtKB/TrEMBL;Acc:L8HHA8]	0.045228833	0.500402333	0.4551735	0.000531475
3h	ACA1_364280/clean/scf7180000084641:70499-70610:+	ACA1_364280	Sporulation proteinrelated, putative [Source:UniProtKB/TrEMBL;Acc:L8GLJ0]	0.00661578	0.461780667	0.455164887	9.20E-10
3h	ACA1_164900/clean/scf7180000084729:305191-305285:-	ACA1_164900	Bromo domain-containing protein [Source:UniProtKB/TrEMBL;Acc:L8GSQ5]	0.014655873	0.469676333	0.45502046	2.46E-08
3h	ACA1_063740/clean/scf7180000084599:1492697-1492779:-	ACA1_063740	Transmembrane protein [Source:UniProtKB/TrEMBL;Acc:L8GXB1]	0.017472167	0.472306333	0.454834167	3.05E-06
3h	ACA1_195840/clean/scf7180000084752:33617-33721:+	ACA1_195840	EGFlike domain containing protein [Source:UniProtKB/TrEMBL;Acc:L8HET8]	0.025491083	0.480277667	0.454786583	6.93E-05
3h	ACA1_013340/clean/scf7180000083750:1388-1489:+	ACA1_013340	PH domain-containing protein [Source:UniProtKB/TrEMBL;Acc:L8HGJ7]	0.002126233	0.456897667	0.454771433	1.15E-09
3h	ACA1_117270/clean/scf7180000084669:966766-966849:+	ACA1_117270	hypothetical protein	0.003133157	0.457852	0.454718843	1.57E-07
3h	ACA1_053290/clean/scf7180000084594:249958-250104:+	ACA1_053290	V-type proton ATPase proteolipid subunit [Source:UniProtKB/TrEMBL;Acc:L8H836]	0.003924657	0.458513333	0.454588677	5.28E-22
3h	ACA1_385390/clean/scf7180000084730:162929-163029:+	ACA1_385390	Ubiquitinconjugating enzyme subfamily protein [Source:UniProtKB/TrEMBL;Acc:L8HAL6]	0.00227199	0.456818667	0.454546677	1.03E-05
3h	ACA1_237860/clean/scf7180000084763:33904-34103:+	ACA1_237860	Glycerophosphodiester phosphodiesterase family protein [Source:UniProtKB/TrEMBL;Acc:L8GKN2]	0.025802367	0.480317	0.454514633	1.33E-06
3h	ACA1_103170/clean/scf7180000084653:13209-13280:+	ACA1_103170	CBS domain containing protein [Source:UniProtKB/TrEMBL;Acc:L8GCP7]	0.123548333	0.577943333	0.454395	0.000249393
3h	ACA1_145170/clean/scf7180000084710:52939-53062:+	ACA1_145170	Carrier superfamily protein [Source:UniProtKB/TrEMBL;Acc:L8GFD9]	0.026910367	0.481269667	0.4543593	1.24E-06
3h	ACA1_386950/clean/scf7180000084730:381406-381485:-	ACA1_386950	hypothetical protein	0.0239239	0.478276	0.4543521	1.21E-05
3h	ACA1_174440/clean/scf7180000084741:709874-709977:-	ACA1_174440	Zinc knuckle domain containing protein [Source:UniProtKB/TrEMBL;Acc:L8HJB4]	0.13366	0.587935667	0.454275667	0.000331016
3h	ACA1_384570/clean/scf7180000084730:53394-53484:-	ACA1_384570	Gelsolin repeat-containing protein [Source:UniProtKB/TrEMBL;Acc:L8H918]	0.01236955	0.466562	0.45419245	1.72E-08
3h	ACA1_122360/clean/scf7180000084682:131950-132030:+	ACA1_122360	PH domain-containing protein [Source:UniProtKB/TrEMBL;Acc:L8GF24]	0.005649733	0.459831667	0.454181933	3.17E-06
3h	ACA1_071690/clean/scf7180000084603:1028965-1029025:+	ACA1_071690	Vacuolar protein sorting factor 4, putative [Source:UniProtKB/TrEMBL;Acc:L8HE96]	0.00536963	0.459405	0.45403537	1.30E-07
3h	ACA1_383560/clean/scf7180000084721:208395-208461:-	ACA1_383560	TBC domain containing protein [Source:UniProtKB/TrEMBL;Acc:L8GW44]	0.022834867	0.476598333	0.453763467	6.94E-06
3h	ACA1_353190/clean/scf7180000084717:13097-13169:-	ACA1_353190	Cytochrome p450 superfamily protein [Source:UniProtKB/TrEMBL;Acc:L8GGB2]	0.01001727	0.463763333	0.453746063	1.79E-09
3h	ACA1_368780/clean/scf7180000084664:160564-160633:-	ACA1_368780	CMP/dCMP-type deaminase domain-containing protein [Source:UniProtKB/TrEMBL;Acc:L8H1B4]	0.003682533	0.45732	0.453637467	9.65E-07
3h	ACA1_072710/clean/scf7180000084603:1272012-1272093:+	ACA1_072710	ABC transporter domain-containing protein [Source:UniProtKB/TrEMBL;Acc:L8HFZ6]	0.03360052	0.487172	0.45357148	1.31E-05
3h	ACA1_164720/clean/scf7180000084729:258583-258663:+	ACA1_164720	Autophagy-related protein 9 [Source:UniProtKB/TrEMBL;Acc:L8GU30]	0.033089167	0.486632667	0.4535435	2.39E-05
3h	ACA1_015560/clean/scf7180000083799:20616-20698:+	ACA1_015560	hypothetical protein	0	0.453507	0.453507	2.41E-10
3h	ACA1_108260/clean/scf7180000084661:22710-22796:-	ACA1_108260	Prenylcys_lyase domain-containing protein [Source:UniProtKB/TrEMBL;Acc:L8GD56]	0.053396567	0.506895	0.453498433	7.59E-05
3h	ACA1_323460/clean/scf7180000084468:25874-25972:-	ACA1_323460	DEP domain-containing protein [Source:UniProtKB/TrEMBL;Acc:L8H1P0]	0.001210567	0.454690667	0.4534801	4.91E-08
3h	ACA1_164660/clean/scf7180000084729:245424-245533:-	ACA1_164660	Protein tyrosine phosphatase, dual specificity, putative [Source:UniProtKB/TrEMBL;Acc:L8GU25]	0.008841907	0.462197667	0.45335576	9.23E-10
3h	ACA1_095690/clean/scf7180000084645:882871-882945:+	ACA1_095690	hypothetical protein	0.00105241	0.454315333	0.453262923	7.68E-07
3h	ACA1_135650/clean/scf7180000084702:99776-99849:-	ACA1_135650	MYND finger domain containing protein [Source:UniProtKB/TrEMBL;Acc:L8GGQ8]	0.1280083	0.581209333	0.453201033	0.000494789
3h	ACA1_385410/clean/scf7180000084730:165251-165371:+	ACA1_385410	TRANSKETOLASE_1 domain-containing protein [Source:UniProtKB/TrEMBL;Acc:L8H9A8]	0.001293396	0.454456667	0.453163271	1.64E-10
3h	ACA1_338180/clean/scf7180000084604:48884-48941:-	ACA1_338180	hypothetical protein	0.004828157	0.457961	0.453132843	2.36E-13
3h	ACA1_234790/clean/scf7180000084762:318697-318794:-	ACA1_234790	PPM-type phosphatase domain-containing protein [Source:UniProtKB/TrEMBL;Acc:L8H3L1]	0.114857267	0.567861667	0.4530044	0.000902242
3h	ACA1_182400/clean/scf7180000084745:204671-204814:+	ACA1_182400	Integral membrane protein [Source:UniProtKB/TrEMBL;Acc:L8H9C3]	0.0538974	0.506821333	0.452923933	4.85E-07
3h	ACA1_364680/clean/scf7180000084641:176138-176258:+	ACA1_364680	BTB domain-containing protein [Source:UniProtKB/TrEMBL;Acc:L8GLI9]	0.024940433	0.477822667	0.452882233	3.29E-05
3h	ACA1_045090/clean/scf7180000084576:77398-77513:-	ACA1_045090	F-box domain-containing protein [Source:UniProtKB/TrEMBL;Acc:L8GYZ3]	0.007152367	0.460016667	0.4528643	8.63E-07
3h	ACA1_197300/clean/scf7180000084753:918-994:-	ACA1_197300	hypothetical protein	0.0629867	0.515677333	0.452690633	0.000112806
3h	ACA1_337990/clean/scf7180000084604:22182-22278:-	ACA1_337990	DJ-1_PfpI domain-containing protein [Source:UniProtKB/TrEMBL;Acc:L8GCZ7]	0.005630973	0.458213667	0.452582693	4.05E-09
3h	ACA1_012330/clean/scf7180000083740:16931-17020:+	ACA1_012330	WD_REPEATS_REGION domain-containing protein [Source:UniProtKB/TrEMBL;Acc:L8HCD7]	0	0.45228	0.45228	8.57E-10
3h	ACA1_195840/clean/scf7180000084752:32366-32440:+	ACA1_195840	EGFlike domain containing protein [Source:UniProtKB/TrEMBL;Acc:L8HET8]	0.009203333	0.461457667	0.452254333	4.07E-06
3h	ACA1_070820/clean/scf7180000084603:769327-769472:+	ACA1_070820	hypothetical protein	0.0094467	0.461617667	0.452170967	6.22E-06
3h	ACA1_023890/clean/scf7180000084436:31824-31923:-	ACA1_023890	SAM domain-containing protein [Source:UniProtKB/TrEMBL;Acc:L8GSK7]	0.018613523	0.470739667	0.452126143	4.07E-08
3h	ACA1_074900/clean/scf7180000084608:110930-110994:-	ACA1_074900	E3 SUMOprotein ligase NSE2, putative [Source:UniProtKB/TrEMBL;Acc:L8HGT6]	0.005649733	0.457713	0.452063267	1.68E-06
3h	ACA1_182110/clean/scf7180000084745:128244-128342:+	ACA1_182110	MethylcrotonoylCoA carboxylase beta chain, mitochondrial, putative [Source:UniProtKB/TrEMBL;Acc:L8H820]	0.00503419	0.456986	0.45195181	1.89E-08
3h	ACA1_138490/clean/scf7180000084703:151741-151805:-	ACA1_138490	Tyrosine-protein phosphatase domain-containing protein [Source:UniProtKB/TrEMBL;Acc:L8GZM7]	0.01113309	0.462919667	0.451786577	2.68E-06

3h	ACA1_376730/clean/scf7180000084685:147951-148022:-	ACA1_376730	Glutamate dehydrogenase [Source:UniProtKB/TrEMBL;Acc:L8HFB5]	0.001801453	0.453533667	0.451732214	4.31E-14
3h	ACA1_380580/clean/scf7180000084712:55638-55732:-	ACA1_380580	Tim17/Tim22/Tim23 family protein [Source:UniProtKB/TrEMBL;Acc:L8GP12]	0.002643727	0.454349667	0.45170594	1.02E-09
3h	ACA1_397180/clean/scf7180000084759:165490-165573:-	ACA1_397180	hypothetical protein	0.00165221	0.453321667	0.451669457	2.55E-06
3h	ACA1_363800/clean/scf7180000084641:20540-20630:-	ACA1_363800	ABC transporter domain-containing protein [Source:UniProtKB/TrEMBL;Acc:L8GM21]	0.057703133	0.509097667	0.451394533	1.19E-05
3h	ACA1_053340/clean/scf7180000084594:265973-266111:-	ACA1_053340	Dynein light intermediate chain [Source:UniProtKB/TrEMBL;Acc:L8H840]	0.0625354	0.513642	0.4511066	8.42E-07
3h	ACA1_358270/clean/scf7180000084742:1842-1914:-	ACA1_358270	Pyr_redox_2 domain-containing protein [Source:UniProtKB/TrEMBL;Acc:L8GL60]	0	0.45102	0.45102	6.53E-07
3h	ACA1_059990/clean/scf7180000084599:558807-558894:+	ACA1_059990	Actine, putative [Source:UniProtKB/TrEMBL;Acc:L8GWG1]	0.157324667	0.608332333	0.451007667	0.002106558
3h	ACA1_391330/clean/scf7180000084740:153748-153821:-	ACA1_391330	hypothetical protein	0.00498903	0.455865667	0.450876637	1.64E-07
3h	ACA1_256450/clean/scf7180000084769:33137-33225:+	ACA1_256450	Poly [ADP-ribose] polymerase [Source:UniProtKB/TrEMBL;Acc:L8GH34]	0.033333333	0.483953667	0.450620333	0.000425576
3h	ACA1_069380/clean/scf7180000084603:425840-425951:+	ACA1_069380	LNS2 domain-containing protein [Source:UniProtKB/TrEMBL;Acc:L8HCT1]	0.01048417	0.460998333	0.450514163	1.52E-06
3h	ACA1_216300/clean/scf7180000084758:461309-461450:+	ACA1_216300	Ras family protein [Source:UniProtKB/TrEMBL;Acc:L8GR60]	0.0050505	0.4555	0.4504495	4.26E-06
3h	ACA1_002430/clean/scf7180000082931:156-243:-	ACA1_002430	G domain-containing protein [Source:UniProtKB/TrEMBL;Acc:L8GY39]	0.004507751	0.454805667	0.450297916	5.89E-06
3h	ACA1_380970/clean/scf7180000084712:130020-130098:+	ACA1_380970	ANK_REP_REGION domain-containing protein [Source:UniProtKB/TrEMBL;Acc:L8GMX1]	0.007246367	0.457435667	0.4501893	1.84E-05
3h	ACA1_060240/clean/scf7180000084599:626066-626270:-	ACA1_060240	hypothetical protein	0.045362967	0.495415	0.450052033	5.44E-06
3h	ACA1_097640/clean/scf7180000084645:1304418-1304521:+	ACA1_097640	Fbox domain containing protein [Source:UniProtKB/TrEMBL;Acc:L8GK52]	0.0633978	0.513424333	0.450026533	5.78E-05
3h	ACA1_145180/clean/scf7180000084710:53470-53557:-	ACA1_145180	F-box domain-containing protein [Source:UniProtKB/TrEMBL;Acc:L8GCU3]	0.030069	0.480085667	0.450016667	0.000484604
3h	ACA1_210530/clean/scf7180000084758:47739-47843:-	ACA1_210530	4Fe-4S ferredoxin-type domain-containing protein [Source:UniProtKB/TrEMBL;Acc:L8GS75]	0.007762281	0.457399	0.449636719	5.53E-06
3h	ACA1_183100/clean/scf7180000084745:333891-333979:-	ACA1_183100	hypothetical protein	0.026306867	0.475880667	0.4495738	1.03E-07
3h	ACA1_053270/clean/scf7180000084594:240196-240304:+	ACA1_053270	Nudix hydrolase domain-containing protein [Source:UniProtKB/TrEMBL;Acc:L8H547]	0.003401367	0.452922067	0.4495207	4.31E-06
3h	ACA1_297750/clean/scf7180000082853:1384-1500:-	ACA1_297750	14_3_3 domain-containing protein [Source:UniProtKB/TrEMBL;Acc:L8HE45]	0.045727433	0.494975333	0.4492479	0.02962482
3h	ACA1_071580/clean/scf7180000084603:998552-998623:-	ACA1_071580	GOLD domain-containing protein [Source:UniProtKB/TrEMBL;Acc:L8HGH0]	0.001920283	0.451041667	0.449121383	1.56E-07
3h	ACA1_184110/clean/scf7180000084745:561552-561644:+	ACA1_184110	hypothetical protein	0.0128205	0.461865333	0.449044833	0.00010509
3h	ACA1_055950/clean/scf7180000084594:790994-791091:+	ACA1_055950	Serine/threonine protein kinase [Source:UniProtKB/TrEMBL;Acc:L8H5M2]	0.026324	0.475054333	0.448730333	1.07E-06
3h	ACA1_180010/clean/scf7180000084744:17416-17601:-	ACA1_180010	hypothetical protein	0.029168633	0.477735	0.448566367	2.73E-07
3h	ACA1_073000/clean/scf7180000084603:1352541-1352615:+	ACA1_073000	Mis12 domain containing protein [Source:UniProtKB/TrEMBL;Acc:L8HGW7]	0.019925633	0.468445333	0.4485197	2.73E-05
3h	ACA1_216300/clean/scf7180000084758:464725-464883:+	ACA1_216300	Ras family protein [Source:UniProtKB/TrEMBL;Acc:L8GR60]	0.067712967	0.516062	0.448349033	0.000263004
3h	ACA1_112100/clean/scf7180000084669:110220-110322:+	ACA1_112100	BTB/POZ domain containing protein [Source:UniProtKB/TrEMBL;Acc:L8H3Y0]	0	0.448332333	0.448332333	9.55E-07
3h	ACA1_200220/clean/scf7180000084753:416908-416993:+	ACA1_200220	FBPase domain-containing protein [Source:UniProtKB/TrEMBL;Acc:L8H3E2]	0.002532803	0.450610333	0.44807753	2.25E-06
3h	ACA1_024220/clean/scf7180000084441:21145-21228:+	ACA1_024220	Purinergic receptor, putative [Source:UniProtKB/TrEMBL;Acc:L8GLB7]	0.012582077	0.460461	0.447878923	2.24E-09
3h	ACA1_279800/clean/scf7180000084776:449696-449950:+	ACA1_279800	Peroxisomal biogenesis factor 11c, putative	0.015145567	0.462875	0.447729433	1.20E-07
3h	ACA1_060920/clean/scf7180000084599:780517-780569:-	ACA1_060920	MFS domain-containing protein [Source:UniProtKB/TrEMBL;Acc:L8GVT5]	0.0713991	0.519098333	0.447699233	0.001350993
3h	ACA1_153060/clean/scf7180000084720:73978-74066:-	ACA1_153060	Ras-GAP domain-containing protein [Source:UniProtKB/TrEMBL;Acc:L8HFU5]	0.01110147	0.458716667	0.447615197	5.02E-08
3h	ACA1_349640/clean/scf7180000084690:23441-23527:+	ACA1_349640	HEAT repeat domain containing protein [Source:UniProtKB/TrEMBL;Acc:L8GKB3]	0	0.447517333	0.447517333	1.18E-07
3h	ACA1_360070/clean/scf7180000084622:41510-41584:-	ACA1_360070	Thr_synth_N domain-containing protein [Source:UniProtKB/TrEMBL;Acc:L8HCM4]	0	0.44748	0.44748	2.08E-08
3h	ACA1_153600/clean/scf7180000084720:136063-136179:-	ACA1_153600	tRNA pseudouridine synthase [Source:UniProtKB/TrEMBL;Acc:L8HG78]	0.174381	0.621816	0.447435	0.007584603
3h	ACA1_153210/clean/scf7180000084720:90197-90290:-	ACA1_153210	Solute carrier family protein [Source:UniProtKB/TrEMBL;Acc:L8HFV0]	0	0.447402	0.447402	1.26E-06
3h	ACA1_026960/clean/scf7180000084464:210-308:-	ACA1_026960	FtsX domain-containing protein [Source:UniProtKB/TrEMBL;Acc:L8GHC2]	0.005263293	0.452563333	0.44730004	3.61E-09
3h	ACA1_265560/clean/scf7180000084770:509388-509464:+	ACA1_265560	RFX-type winged-helix domain-containing protein [Source:UniProtKB/TrEMBL;Acc:L8H4F0]	0	0.447097	0.447097	6.83E-09
3h	ACA1_199790/clean/scf7180000084753:329824-329949:+	ACA1_199790	hypothetical protein	0.0244625	0.471414	0.4469515	3.08E-05
3h	ACA1_220200/clean/scf7180000084758:895601-895675:+	ACA1_220200	Aminopeptidase [Source:UniProtKB/TrEMBL;Acc:L8GT26]	0.000827549	0.447721	0.446893451	3.81E-10
3h	ACA1_365090/clean/scf7180000084641:234290-234365:+	ACA1_365090	ADPribosylglycohydrolase superfamily protein [Source:UniProtKB/TrEMBL;Acc:L8GP97]	0.166099667	0.612885	0.446785333	0.000930737
3h	ACA1_058700/clean/scf7180000084599:360866-360937:+	ACA1_058700	hypothetical protein	0.005557783	0.452253	0.446695217	1.90E-08
3h	ACA1_087720/clean/scf7180000084639:105672-105743:+	ACA1_087720	Dihydrodipicolinate synthetase [Source:UniProtKB/TrEMBL;Acc:L8GVJ3]	0.001272533	0.447881	0.446608467	1.24E-06
3h	ACA1_119240/clean/scf7180000084672:49968-50078:+	ACA1_119240	Aspartate aminotransferase [Source:UniProtKB/TrEMBL;Acc:L8HKA6]	0.003309977	0.449724667	0.44641469	5.94E-14
3h	ACA1_027610/clean/scf7180000084469:11042-11116:+	ACA1_027610	hypothetical protein	0.01075926	0.457001667	0.446242407	8.11E-05
3h	ACA1_007760/clean/scf7180000083166:21352-21463:+	ACA1_007760	PH domain containing protein [Source:UniProtKB/TrEMBL;Acc:L8HAY1]	0.024688233	0.470876667	0.446188433	9.74E-06
3h	ACA1_400560/clean/scf7180000084775:194180-194258:+	ACA1_400560	hypothetical protein	0.000449573	0.446587333	0.44613776	1.59E-08
3h	ACA1_128500/clean/scf7180000084697:73459-73584:-	ACA1_128500	UBIQUITIN_CONJUGAT_2 domain-containing protein [Source:UniProtKB/TrEMBL;Acc:L8GWJ5]	0.00709303	0.453145	0.44605197	2.13E-07
3h	ACA1_089330/clean/scf7180000084639:370548-370655:-	ACA1_089330	Glyco_hydro_2_C domain-containing protein [Source:UniProtKB/TrEMBL;Acc:L8GUL5]	0	0.445995	0.445995	1.87E-05
3h	ACA1_376140/clean/scf7180000084685:34127-34211:-	ACA1_376140	AcetylCoA acetyltransferase, mitochondrial, putative [Source:UniProtKB/TrEMBL;Acc:L8HG98]	0	0.445824333	0.445824333	2.92E-07
3h	ACA1_045630/clean/scf7180000084576:165810-165880:-	ACA1_045630	Deacetylase sirtuin-type domain-containing protein [Source:UniProtKB/TrEMBL;Acc:L8GZU7]	0.017835367	0.463551333	0.445715967	0.000107758
3h	ACA1_103170/clean/scf7180000084653:11803-11882:+	ACA1_103170	CBS domain containing protein [Source:UniProtKB/TrEMBL;Acc:L8GCP7]	0.015653767	0.461332	0.445678233	4.02E-05
3h	ACA1_113490/clean/scf7180000084669:294158-294362:-	ACA1_113490	SAM domain-containing protein [Source:UniProtKB/TrEMBL;Acc:L8H3A5]	0.135202333	0.580767	0.445564667	0.000114035
3h	ACA1_068620/clean/scf7180000084603:240766-240843:-	ACA1_068620	hypothetical protein	0	0.445485333	0.445485333	3.08E-08
3h	ACA1_153680/clean/scf7180000084720:153573-153655:-	ACA1_153680	Amz2 protein [Source:UniProtKB/TrEMBL;Acc:L8HHH0]	0.0116175	0.456994333	0.445376833	0.003468133
3h	ACA1_151250/clean/scf7180000084716:81709-81861:+	ACA1_151250	Glycine cleavage Tprotein (Aminomethyl transferase) domain containing protein [Source:UniProtKB/TrEMBL;Acc:L8H0R3]	0.00614166	0.451444	0.44530234	1.74E-06
3h	ACA1_270940/clean/scf7180000084770:1172125-1172191:-	ACA1_270940	hypothetical protein	0.070282567	0.515574	0.445291433	0.000861211
3h	ACA1_333170/clean/scf7180000084581:9655-9714:-	ACA1_333170	Ras-GEF domain-containing protein [Source:UniProtKB/TrEMBL;Acc:L8HGS6]	0.000904567	0.446008333	0.445103767	0.0029252
3h	ACA1_015490/clean/scf7180000083799:3028-3128:+	ACA1_015490	hypothetical protein	0.011954663	0.457046667	0.445092003	1.13E-05
3h	ACA1_189640/clean/scf7180000084748:70409-70499:-	ACA1_189640	Bifunctional aspartate kinase/diaminopimelate decarboxylase protein [Source:UniProtKB/TrEMBL;Acc:L8GEY3]	0.00716963	0.452224667	0.445055037	8.40E-09
3h	ACA1_091960/clean/scf7180000084645:236286-236374:-	ACA1_091960	hypothetical protein	0.0311308	0.476128667	0.444997867	1.72E-05
3h	ACA1_277560/clean/scf7180000084776:74362-74440:-	ACA1_277560	F-box domain-containing protein [Source:UniProtKB/TrEMBL;Acc:L8H6G6]	0.041119233	0.486114333	0.4449951	1.40E-05
3h	ACA1_181950/clean/scf7180000084745:90330-90428:+	ACA1_181950	hypothetical protein	0.04567134	0.490638333	0.444966993	0.000273057
3h	ACA1_279800/clean/scf7180000084776:450441-450513:+	ACA1_279800	Peroxisomal biogenesis factor 11c, putative	0	0.444777667	0.444777667	5.58E-09
3h	ACA1_208430/clean/scf7180000084757:190256-190365:+	ACA1_208430	hypothetical protein	0.034442373	0.479101	0.444658627	8.22E-06
3h	ACA1_254920/clean/scf7180000084768:447370-447468:+	ACA1_254920	von Willebrand factor type A domain containing protein [Source:UniProtKB/TrEMBL;Acc:L8HA47]	0.001826333	0.446484	0.444657667	1.20E-07
3h	ACA1_054710/clean/scf7180000084594:559063-559161:-	ACA1_054710	Ras family protein [Source:UniProtKB/TrEMBL;Acc:L8H6F4]	0.006145157	0.450792667	0.44464751	2.57E-08
3h	ACA1_230500/clean/scf7180000084761:364851-364958:+	ACA1_230500	Ammonium transporter [Source:UniProtKB/TrEMBL;Acc:L8H844]	0.035468267	0.480030667	0.4445624	1.64E-05
3h	ACA1_152950/clean/scf7180000084720:40695-40788:-	ACA1_152950	UBIQUITIN_CONJUGAT_2 domain-containing protein [Source:UniProtKB/TrEMBL;Acc:L8HI96]	0.00662803	0.451138667	0.444510637	5.49E-12
3h	ACA1_115400/clean/scf7180000084669:624959-625077:+	ACA1_115400	Protoporphyrinogen oxidase [Source:UniProtKB/TrEMBL;Acc:L8H4N9]	0.0457286	0.490166	0.4444374	5.82E-05
3h	ACA1_026390/clean/scf7180000084456:9941-10035:-	ACA1_026390	EFhand domain containing protein [Source:UniProtKB/TrEMBL;Acc:L8GMZ4]	0.002727123	0.447133333	0.44440621	1.25E-09
3h	ACA1_012310/clean/scf7180000083740:11493-11577:+	ACA1_012310	Biogenesis of lysosome related family protein [Source:UniProtKB/TrEMBL;Acc:L8HEJ0]	0.01065317	0.454981	0.44432783	3.67E-09
3h	ACA1_335940/clean/scf7180000084593:8135-8206:-	ACA1_335940	RNA recognition motif domain containing protein [Source:UniProtKB/TrEMBL;Acc:L8H3Y6]	0.014959507	0.459278333	0.444318827	4.85E-07
3h	ACA1_184420/clean/scf7180000084745:638985-639079:+	ACA1_184420	Cathepsin Llike proteinase [Source:UniProtKB/TrEMBL;Acc:L8H8J9]	0.000232919	0.444435667	0.444202748	3.39E-11
3h	ACA1_282440/clean/scf7180000084776:681534-681599:+	ACA1_282440	hypothetical protein	0.0144766	0.458562	0.4440854	1.57E-05
3h	ACA1_068360/clean/scf7180000084603:179230-179333:+	ACA1_068360	Dual specificity protein kinase [Source:UniProtKB/TrEMBL;Acc:L8HDE4]	2.52E-05	0.44408	0.444054849	3.28E-11
3h	ACA1_090240/clean/scf7180000084639:464648-464742:-	ACA1_090240	Magnesiumdependent phosphatase-1, putative [Source:UniProtKB/TrEMBL;Acc:L8GXB9]	0.024704733	0.468731333	0.4440266	6.68E-06
3h	ACA1_154730/clean/scf7180000084722:90693-90788:+	ACA1_154730	Protein kinase domain-containing protein [Source:UniProtKB/TrEMBL;Acc:L8H011]	0.085478467	0.529279	0.443800533	0.000245737
3h	ACA1_286170/clean/scf7180000084776:1051777-1051856:+	ACA1_286170	Peptidase_S9 domain-containing protein [Source:UniProtKB/TrEMBL;Acc:L8H7U8]	0.00107527	0.444875667	0.443800397	8.42E-08
3h	ACA1_234100/clean/scf7180000084762:199826-199959:+	ACA1_234100	G_PROTEIN_RECEP_F2_4 domain-containing protein [Source:UniProtKB/TrEMBL;Acc:L8H173]	0	0.443794333	0.443794333	2.12E-07
3h	ACA1_053950/clean/scf7180000084594:359446-359627:-	ACA1_053950	Nudix hydrolase domain-containing protein [Source:UniProtKB/TrEMBL;Acc:L8H877]	0.085469533	0.529112	0.443642467	5.80E-05
3h	ACA1_053850/clean/scf7180000084594:334939-335036:+	ACA1_053850	ATP11 protein [Source:UniProtKB/TrEMBL;Acc:L8H868]	0.106509267	0.55	0.443490733	0.0106584
3h	ACA1_151330/clean/scf7180000084716:106350-106466:-	ACA1_151330	Purple acid phosphatase [Source:UniProtKB/TrEMBL;Acc:L8H0Q3]	0.002978629	0.446383333	0.443404704	7.56E-08
3h	ACA1_239550/clean/scf7180000084763:165718-165828:-	ACA1_239550	Cytidine and deoxycytidylate deaminase zincbinding region subfamily protein [Source:UniProtKB/TrEMBL;Acc:L8GKS6]	0.012440557	0.455844667	0.44340411	4.18E-09
3h	ACA1_068390/clean/scf7180000084603:186759-186845:-	ACA1_068390	hypothetical protein	0.03978432	0.4831434	0.44335908	0.000594874
3h	ACA1_178060/clean/scf7180000084743:162886-162965:-	ACA1_178060	Dipeptidylpeptidase 9, putative [Source:UniProtKB/TrEMBL;Acc:L8GTD8]	0.000360127	0.443698667	0.44333854	4.86E-08
3h	ACA1_253360/clean/scf7180000084768:284886-284969:+	ACA1_253360	TP_methylase domain-containing protein [Source:UniProtKB/TrEMBL;Acc:L8HAX0]	0	0.443301	0.443301	1.36E-08
3h	ACA1_183970/clean/scf7180000084745:533357-533440:-	ACA1_183970	hypothetical protein	0	0.443292667	0.443292667	0.002247345
3h	ACA1_173640/clean/scf7180000084741:500503-500596:+	ACA1_173640	ZnF_Rad18 domain-containing protein [Source:UniProtKB/TrEMBL;Acc:L8HK04]	0.02614649	0.469342333	0.443195843	7.03E-05
3h	ACA1_181910/clean/scf7180000084745:72043-72122:-	ACA1_181910	CrcB family protein [Source:UniProtKB/TrEMBL;Acc:L8H804]	0.006410267	0.449506667	0.4430964	4.13E-06
3h	ACA1_045250/clean/scf7180000084576:93796-93895:+	ACA1_045250	hypothetical protein	0.046152767	0.489037667	0.4428849	0.000294536
3h	ACA1_066530/clean/scf7180000084600:6667-6730:-	ACA1_066530	Bac_rhamnosid_C domain-containing protein [Source:UniProtKB/TrEMBL;Acc:L8GPR7]	0.075422633	0.518299333	0.4428767	0.000285932
3h	ACA1_174690/clean/scf7180000084741:764862-764944:+	ACA1_174690	Deacetylase sirtuin-type domain-containing protein [Source:UniProtKB/TrEMBL;Acc:L8HHW7]	0.0114848	0.454338333	0.442853533	8.93E-08
3h	ACA1_072010/clean/scf7180000084603:1125928-1126075:-	ACA1_072010	hypothetical protein	0.028600073	0.471442333	0.44284226	1.79E-05
3h	ACA1_052440/clean/scf7180000084594:76734-76797:-	ACA1_052440	GPI mannosyltransferase 1 [Source:UniProtKB/TrEMBL;Acc:L8H760]	0.026074117	0.468813333	0.442739217	8.59E-06
3h	ACA1_366940/clean/scf7180000084641:519853-519932:-	ACA1_366940	BTB/POZ domain containing protein [Source:UniProtKB/TrEMBL;Acc:L8GPM1]	0.004485567	0.4471424	0.442656833	0.000198908
3h	ACA1_234600/clean/scf7180000084762:303604-303693:+	ACA1_234600	ANK_REP_REGION domain-containing protein [Source:UniProtKB/TrEMBL;Acc:L8H1A6]	0.011423887	0.454064333	0.442640447	9.65E-08
3h	ACA1_292880/clean/scf7180000084777:924577-924698:-	ACA1_292880	RhoGEF domain containing protein [Source:UniProtKB/TrEMBL;Acc:L8HJL4]	0.022774333	0.465048667	0.442274333	2.65E-05
3h	ACA1_200050/clean/scf7180000084753:381969-382055:-	ACA1_200050	Poly [ADP-ribose] polymerase [Source:UniProtKB/TrEMBL;Acc:L8H4Q2]	0.006250397	0.448522333	0.442271937	4.68E-07
3h	ACA1_170970/clean/scf7180000084741:30730-30855:-	ACA1_170970	Protein kinase domain-containing protein [Source:UniProtKB/TrEMBL;Acc:L8HH98]	0.002521323	0.444690333	0.44216901	4.92E-15
3h	ACA1_396920/clean/scf7180000084759:149436-149523:-	ACA1_396920	Guanylate kinase-like domain-containing protein [Source:UniProtKB/TrEMBL;Acc:L8HEM5]	0.004806063	0.446779333	0.44197327	5.54E-13
3h	ACA1_279280/clean/scf7180000084776:346662-346742:-	ACA1_279280	Lcarnitine dehydratase/bile acid-inducible protein F, putative [Source:UniProtKB/TrEMBL;Acc:L8H915]	0.013471233	0.455346	0.441874767	8.49E-07
3h	ACA1_055560/clean/scf7180000084594:728891-728955:-	ACA1_055560	Protein C16orf58 family protein [Source:UniProtKB/TrEMBL;Acc:L8H6L0]	0	0.441853	0.441853	0.000249353
3h	ACA1_030740/clean/scf7180000084505:17981-18085:-	ACA1_030740	Znfinger in Ran binding protein [Source:UniProtKB/TrEMBL;Acc:L8HIE7]	0.005322677	0.447112667	0.44178999	5.26E-07
3h	ACA1_367410/clean/scf7180000084641:628287-628346:-	ACA1_367410	Phosphodiesterase [Source:UniProtKB/TrEMBL;Acc:L8GMB7]	0.0430482	0.484701	0.4416528	0.000289396
3h	ACA1_229570/clean/scf7180000084761:281105-281197:-	ACA1_229570	Oxidoreductase, short chain dehydrogenase/reductase superfamily protein [Source:UniProtKB/TrEMBL;Acc:L8H814]	0.000283366	0.441819333	0.441535967	2.77E-08
3h	ACA1_062680/clean/scf7180000084599:1271935-1272031:+	ACA1_062680	hypothetical protein	0.0165631	0.458000933	0.441437833	8.20E-08
3h	ACA1_025560/clean/scf7180000084448:14048-14122:-	ACA1_025560	hypothetical protein	0.047619	0.488992267	0.441373267	0.002097428
3h	ACA1_250180/clean/scf7180000084767:36436-36575:-	ACA1_250180	RhoGEF domain containing protein [Source:UniProtKB/TrEMBL;Acc:L8HC96]	0.033361363	0.474595667	0.441234303	8.75E-07
3h	ACA1_137790/clean/scf7180000084703:72675-72755:-	ACA1_137790	IBR domain-containing protein [Source:UniProtKB/TrEMBL;Acc:L8GZJ6]	0.019111533	0.460293333	0.4411818	0.002247345
3h	ACA1_234250/clean/scf7180000084762:249378-249492:-	ACA1_234250	Protein kinase domain-containing protein [Source:UniProtKB/TrEMBL;Acc:L8H190]	0.064823367	0.505964	0.441140633	1.16E-05
3h	ACA1_186870/clean/scf7180000084746:214400-214497:+	ACA1_186870	Protein kinase domain-containing protein [Source:UniProtKB/TrEMBL;Acc:L8H6J9]	0.000258054	0.441384567	0.441126513	1.15E-08
3h	ACA1_323370/clean/scf7180000084468:12393-12455:-	ACA1_323370	SHSP domain-containing protein [Source:UniProtKB/TrEMBL;Acc:L8H320]	0.017660033	0.458639	0.440978967	0.002894154
3h	ACA1_389130/clean/scf7180000084733:207304-207388:+	ACA1_389130	BTB/POZ domain containing protein [Source:UniProtKB/TrEMBL;Acc:L8GDV1]	0	0.440690667	0.440690667	4.72E-10
3h	ACA1_191700/clean/scf7180000084749:33707-33798:-	ACA1_191700	Aldo_ket_red domain-containing protein [Source:UniProtKB/TrEMBL;Acc:L8GNA1]	0.021244933	0.461925	0.440680067	4.66E-06
3h	ACA1_380780/clean/scf7180000084712:88004-88081:-	ACA1_380780	hypothetical protein	0.002298547	0.44272	0.440421453	2.21E-08
3h	ACA1_216320/clean/scf7180000084758:466814-466955:+	ACA1_216320	Homogentisate 1,2dioxygenase [Source:UniProtKB/TrEMBL;Acc:L8GQ83]	0.001157347	0.441549	0.440391653	1.19E-09
3h	ACA1_016360/clean/scf7180000083820:5823-5920:+	ACA1_016360	DUF4392 domain-containing protein [Source:UniProtKB/TrEMBL;Acc:L8GQ34]	0.013457187	0.453566667	0.44010948	4.24E-09
3h	ACA1_203750/clean/scf7180000084754:204756-204852:-	ACA1_203750	PH domain-containing protein [Source:UniProtKB/TrEMBL;Acc:L8GTJ3]	0.005163358	0.445233667	0.440070308	2.90E-07
3h	ACA1_380350/clean/scf7180000084712:46445-46508:+	ACA1_380350	Membrane protein, putative [Source:UniProtKB/TrEMBL;Acc:L8GMT8]	0.051757233	0.491661	0.439903767	4.34E-06
3h	ACA1_024200/clean/scf7180000084441:14633-14719:-	ACA1_024200	WD_REPEATS_REGION domain-containing protein [Source:UniProtKB/TrEMBL;Acc:L8GKS0]	0.036171367	0.475939667	0.4397683	2.56E-05
3h	ACA1_035580/clean/scf7180000084564:93699-93769:+	ACA1_035580	hypothetical protein	0.027670733	0.467345667	0.439674933	6.44E-07
3h	ACA1_289290/clean/scf7180000084777:408613-408695:-	ACA1_289290	EamA domain-containing protein [Source:UniProtKB/TrEMBL;Acc:L8HLD7]	0.0171125	0.456611333	0.439498833	2.22E-05
3h	ACA1_034030/clean/scf7180000084561:9347-9470:+	ACA1_034030	RNA recognition motif domain containing protein [Source:UniProtKB/TrEMBL;Acc:L8GTW3]	0.029973933	0.469458667	0.439484733	9.13E-06
3h	ACA1_323370/clean/scf7180000084468:11999-12065:-	ACA1_323370	SHSP domain-containing protein [Source:UniProtKB/TrEMBL;Acc:L8H320]	0.007751933	0.447186667	0.439434733	0.003309609
3h	ACA1_163660/clean/scf7180000084729:65575-65663:-	ACA1_163660	hypothetical protein	0.00216151	0.441338333	0.439176823	6.53E-10
3h	ACA1_091570/clean/scf7180000084645:151618-151698:+	ACA1_091570	Superoxide dismutase [Source:UniProtKB/TrEMBL;Acc:L8GI64]	0.010921947	0.450095333	0.439173387	1.58E-05
3h	ACA1_034300/clean/scf7180000084563:6500-6560:+	ACA1_034300	DUF1084 domain-containing protein [Source:UniProtKB/TrEMBL;Acc:L8HCY8]	0	0.438912333	0.438912333	5.90E-07
3h	ACA1_069380/clean/scf7180000084603:424176-424254:+	ACA1_069380	LNS2 domain-containing protein [Source:UniProtKB/TrEMBL;Acc:L8HCT1]	0.000652317	0.439561667	0.43890935	1.01E-07
3h	ACA1_095680/clean/scf7180000084645:878735-878801:+	ACA1_095680	hypothetical protein	0.061118	0.499956667	0.438838667	1.00E-05
3h	ACA1_037900/clean/scf7180000084568:53753-53841:-	ACA1_037900	Nudix hydrolase domain-containing protein [Source:UniProtKB/TrEMBL;Acc:L8GKU1]	0.014522767	0.453267667	0.4387449	0.019014615
3h	ACA1_074850/clean/scf7180000084608:103944-104027:-	ACA1_074850	S5 DRBM domain-containing protein [Source:UniProtKB/TrEMBL;Acc:L8HGT0]	0.037711333	0.476314333	0.438603	0.004127836
3h	ACA1_384870/clean/scf7180000084730:128421-128511:+	ACA1_384870	Protein kinase domain containing protein [Source:UniProtKB/TrEMBL;Acc:L8HBH4]	0.01196782	0.450505667	0.438537847	1.84E-06
3h	ACA1_164900/clean/scf7180000084729:304911-305007:-	ACA1_164900	Bromo domain-containing protein [Source:UniProtKB/TrEMBL;Acc:L8GSQ5]	0.047987667	0.486515333	0.438527667	2.08E-07
3h	ACA1_385400/clean/scf7180000084730:164152-164234:-	ACA1_385400	Rieske domain-containing protein [Source:UniProtKB/TrEMBL;Acc:L8H8D5]	0.158388333	0.596864333	0.438476	0.000957253
3h	ACA1_269320/clean/scf7180000084770:971980-972071:-	ACA1_269320	eIF2 gamma, putative [Source:UniProtKB/TrEMBL;Acc:L8H4Y2]	0.01319387	0.451637	0.43844313	1.34E-08
3h	ACA1_272530/clean/scf7180000084772:55901-55976:+	ACA1_272530	VWFA domain-containing protein [Source:UniProtKB/TrEMBL;Acc:L8HIL5]	0.003003003	0.441087333	0.43808433	2.39E-06
3h	ACA1_116740/clean/scf7180000084669:839044-839133:+	ACA1_116740	Methyltransf_2 domain-containing protein [Source:UniProtKB/TrEMBL;Acc:L8H4R1]	0.002918003	0.440964333	0.43804633	3.47E-06

3h	ACA1_373930/clean/scf7180000084679:192749-192884:-	ACA1_373930	hypothetical protein	0.00183619	0.439812667	0.437976477	6.09E-07
3h	ACA1_063620/clean/scf7180000084599:1462782-1462872:-	ACA1_063620	Hydroxysteroid dehydrogenase, putative [Source:UniProtKB/TrEMBL;Acc:L8GYL0]	0.00241546	0.440267333	0.437851873	3.59E-08
3h	ACA1_069100/clean/scf7180000084603:355575-355666:+	ACA1_069100	Lactamase_B domain-containing protein [Source:UniProtKB/TrEMBL;Acc:L8HFU9]	0.153606	0.591390667	0.437784667	0.001887236
3h	ACA1_057820/clean/scf7180000084599:186668-186780:-	ACA1_057820	Dual specificity phosphatase, putative [Source:UniProtKB/TrEMBL;Acc:L8GYG8]	0.004461593	0.441895333	0.43743374	0.000186831
3h	ACA1_146620/clean/scf7180000084713:76879-76983:-	ACA1_146620	Membrane protein [Source:UniProtKB/TrEMBL;Acc:L8GVA1]	0.268460333	0.70585	0.437389667	0.002014053
3h	ACA1_276410/clean/scf7180000084774:168454-168580:+	ACA1_276410	Phosphate transporter [Source:UniProtKB/TrEMBL;Acc:L8GTH7]	0.036141527	0.473366667	0.43722514	2.76E-05
3h	ACA1_126140/clean/scf7180000084693:30481-30602:-	ACA1_126140	HA domain-containing protein [Source:UniProtKB/TrEMBL;Acc:L8HAG8]	0.019107233	0.456229333	0.4371221	1.59E-05
3h	ACA1_063800/clean/scf7180000084599:1506142-1506202:-	ACA1_063800	B30.2/SPRY domain-containing protein [Source:UniProtKB/TrEMBL;Acc:L8GZV1]	0.0510311	0.487685333	0.436654233	0.000154432
3h	ACA1_102790/clean/scf7180000084652:28296-28369:-	ACA1_102790	hypothetical protein	0.016142343	0.452785	0.436642657	2.43E-05
3h	ACA1_362720/clean/scf7180000084624:159751-159841:-	ACA1_362720	Neutral ceramidase [Source:UniProtKB/TrEMBL;Acc:L8GFC5]	0.004867935	0.441502667	0.436634732	1.86E-07
3h	ACA1_367310/clean/scf7180000084641:595398-595498:-	ACA1_367310	CCHC-type domain-containing protein [Source:UniProtKB/TrEMBL;Acc:L8GMZ2]	0.015000733	0.451545	0.436544267	0.001929889
3h	ACA1_230570/clean/scf7180000084761:380667-380763:-	ACA1_230570	DUF4139 domain-containing protein [Source:UniProtKB/TrEMBL;Acc:L8H928]	0.006349981	0.442888	0.436538019	3.09E-07
3h	ACA1_138420/clean/scf7180000084703:133941-134041:-	ACA1_138420	hypothetical protein	0.025924107	0.462271333	0.436347227	6.82E-06
3h	ACA1_142680/clean/scf7180000084708:218869-219110:+	ACA1_142680	unspecified product	0.0251651	0.461504	0.4363389	1.48E-07
3h	ACA1_255710/clean/scf7180000084768:549330-549481:+	ACA1_255710	Rho-GAP domain-containing protein [Source:UniProtKB/TrEMBL;Acc:L8HB62]	0.0664193	0.502745667	0.436326367	0.000906907
3h	ACA1_040710/clean/scf7180000084572:56753-56854:+	ACA1_040710	AAA domain-containing protein [Source:UniProtKB/TrEMBL;Acc:L8H0J4]	0.003812223	0.440076	0.436263777	3.22E-08
3h	ACA1_178270/clean/scf7180000084743:194715-194799:+	ACA1_178270	Rasrelated protein O-RAL, putative [Source:UniProtKB/TrEMBL;Acc:L8GU86]	0.0245651	0.460731	0.4361659	1.53E-07
3h	ACA1_096590/clean/scf7180000084645:1054694-1054778:-	ACA1_096590	Usp domain-containing protein [Source:UniProtKB/TrEMBL;Acc:L8GJ01]	0.067938267	0.504033	0.436094733	0.003607493
3h	ACA1_202770/clean/scf7180000084754:106110-106195:-	ACA1_202770	TPR_REGION domain-containing protein [Source:UniProtKB/TrEMBL;Acc:L8GSW6]	0.054961767	0.491011	0.436049233	7.16E-05
3h	ACA1_369480/clean/scf7180000084664:310081-310164:-	ACA1_369480	SMP-LTD domain-containing protein [Source:UniProtKB/TrEMBL;Acc:L8GYZ6]	0.011646355	0.447633667	0.435987312	1.65E-06
3h	ACA1_109140/clean/scf7180000084661:130568-130668:+	ACA1_109140	F-box domain-containing protein [Source:UniProtKB/TrEMBL;Acc:L8GD84]	0.008086087	0.443851	0.435764913	1.52E-05
3h	ACA1_015560/clean/scf7180000083799:20968-21049:+	ACA1_015560	hypothetical protein	0.0160592	0.451805233	0.435746033	2.47E-07
3h	ACA1_062040/clean/scf7180000084599:1108773-1108849:-	ACA1_062040	PPR repeat-containing protein [Source:UniProtKB/TrEMBL;Acc:L8GWY3]	0.0107527	0.445867667	0.435114967	2.65E-05
3h	ACA1_037190/clean/scf7180000084567:79804-79879:+	ACA1_037190	Aminotran_5 domain-containing protein [Source:UniProtKB/TrEMBL;Acc:L8H0V4]	0.055348233	0.490299	0.434950767	3.96E-06
3h	ACA1_012980/clean/scf7180000083742:15602-15666:-	ACA1_012980	Endo/exonuclease/phosphatase domain-containing protein [Source:UniProtKB/TrEMBL;Acc:L8H4W5]	0.006666667	0.441460667	0.434794	8.30E-06
3h	ACA1_054480/clean/scf7180000084594:508737-508808:+	ACA1_054480	Pribosyltran domain-containing protein [Source:UniProtKB/TrEMBL;Acc:L8H652]	0.033932547	0.468709333	0.434776787	3.68E-05
3h	ACA1_088880/clean/scf7180000084639:253841-253918:-	ACA1_088880	OGFr_N domain-containing protein [Source:UniProtKB/TrEMBL;Acc:L8GUP7]	0.049450567	0.484217	0.434766433	0.008591968
3h	ACA1_103150/clean/scf7180000084653:8166-8250:+	ACA1_103150	hypothetical protein	0.0313792	0.466051667	0.434672467	3.03E-06
3h	ACA1_035640/clean/scf7180000084564:103520-103627:-	ACA1_035640	Beta-lactamase domain-containing protein [Source:UniProtKB/TrEMBL;Acc:L8H9P2]	0	0.434537667	0.434537667	0.017258824
3h	ACA1_063870/clean/scf7180000084599:1522068-1522158:+	ACA1_063870	Metallophos domain-containing protein [Source:UniProtKB/TrEMBL;Acc:L8GYM9]	0.02062066	0.455033	0.43441234	5.38E-06
3h	ACA1_183190/clean/scf7180000084745:352781-352887:-	ACA1_183190	TYR_PHOSPHATASE_2 domain-containing protein [Source:UniProtKB/TrEMBL;Acc:L8H893]	0.0288301	0.463237333	0.434407233	1.24E-07
3h	ACA1_028680/clean/scf7180000084473:9343-9451:+	ACA1_028680	Protein kinase domain-containing protein [Source:UniProtKB/TrEMBL;Acc:L8GU00]	0.03435182	0.468748333	0.434396513	7.58E-06
3h	ACA1_074850/clean/scf7180000084608:103799-103861:-	ACA1_074850	S5 DRBM domain-containing protein [Source:UniProtKB/TrEMBL;Acc:L8HGT0]	0	0.43437	0.43437	2.39E-06
3h	ACA1_224390/clean/scf7180000084760:191867-192021:-	ACA1_224390	hypothetical protein	0.00635983	0.440691	0.43433117	7.25E-07
3h	ACA1_144360/clean/scf7180000084709:215192-215302:+	ACA1_144360	Repressor of RNA polymerase III transcription [Source:UniProtKB/TrEMBL;Acc:L8HFX0]	0.01414339	0.448418667	0.434275277	3.12E-10
3h	ACA1_015560/clean/scf7180000083799:20456-20518:+	ACA1_015560	hypothetical protein	0.00118914	0.435296333	0.434107193	6.69E-09
3h	ACA1_165790/clean/scf7180000084732:83464-83565:-	ACA1_165790	hypothetical protein	0.016557667	0.450657	0.434099333	2.63E-08
3h	ACA1_390600/clean/scf7180000084740:79999-80082:-	ACA1_390600	Metallophos domain-containing protein [Source:UniProtKB/TrEMBL;Acc:L8GNV1]	0.021812067	0.4558091	0.433997033	0.000489279
3h	ACA1_063460/clean/scf7180000084599:1414375-1414433:-	ACA1_063460	AAA domain-containing protein [Source:UniProtKB/TrEMBL;Acc:L8GXD1]	0.021208013	0.455202333	0.43399432	9.30E-08
3h	ACA1_123360/clean/scf7180000084686:18125-18421:+	ACA1_123360	Zn(2)-C6 fungal-type domain-containing protein [Source:UniProtKB/TrEMBL;Acc:L8HF21]	0.00650586	0.440488667	0.433982807	5.29E-07
3h	ACA1_061250/clean/scf7180000084599:866748-866812:+	ACA1_061250	Peptidyl-prolyl cis-trans isomerase [Source:UniProtKB/TrEMBL;Acc:L8GWR2]	0.000983307	0.434963667	0.433980359	1.06E-09
3h	ACA1_090230/clean/scf7180000084639:462541-462641:+	ACA1_090230	Chromatin associated protein [Source:UniProtKB/TrEMBL;Acc:L8GUQ1]	0.00746576	0.441311	0.43384524	1.88E-08
3h	ACA1_267340/clean/scf7180000084770:859690-859749:-	ACA1_267340	CBS domain containing protein [Source:UniProtKB/TrEMBL;Acc:L8H2L9]	0.006666667	0.439996	0.433329333	4.21E-05
3h	ACA1_015480/clean/scf7180000083799:2123-2230:+	ACA1_015480	hypothetical protein	0.040194333	0.473476	0.433281667	0.000147433
3h	ACA1_119140/clean/scf7180000084672:19998-20114:-	ACA1_119140	hypothetical protein	0.008429133	0.441675667	0.433246533	0.00015923
3h	ACA1_184780/clean/scf7180000084746:61444-61560:+	ACA1_184780	Protein YIPF [Source:UniProtKB/TrEMBL;Acc:L8H566]	0.003526537	0.436750233	0.433223696	1.19E-07
3h	ACA1_378010/clean/scf7180000084711:163514-163603:+	ACA1_378010	JmjC domain-containing protein [Source:UniProtKB/TrEMBL;Acc:L8GRT4]	0.561466	0.994613	0.433147	5.09E-05
3h	ACA1_206030/clean/scf7180000084755:66654-66730:+	ACA1_206030	Abnormal long morphology protein [Source:UniProtKB/TrEMBL;Acc:L8H4K0]	0.004975133	0.438041667	0.433066533	8.87E-06
3h	ACA1_057520/clean/scf7180000084599:158538-158606:+	ACA1_057520	NTP_transf_2 domain-containing protein [Source:UniProtKB/TrEMBL;Acc:L8GYG1]	0.002934577	0.435769	0.432834423	2.87E-06
3h	ACA1_042040/clean/scf7180000084573:44498-44576:-	ACA1_042040	RAI1 domain-containing protein [Source:UniProtKB/TrEMBL;Acc:L8GV91]	0.042455307	0.475220333	0.432765027	5.13E-05
3h	ACA1_040690/clean/scf7180000084572:48081-48160:+	ACA1_040690	Multisensor hybrid histidine kinase [Source:UniProtKB/TrEMBL;Acc:L8H1W6]	0.085147267	0.517682	0.432534733	0.000278449
3h	ACA1_065700/clean/scf7180000084599:1874573-1874676:+	ACA1_065700	Protein kinase domain-containing protein [Source:UniProtKB/TrEMBL;Acc:L8H039]	0.00579053	0.438298333	0.432507803	4.81E-07
3h	ACA1_234290/clean/scf7180000084762:262833-262945:-	ACA1_234290	Deacetylase sirtuin-type domain-containing protein [Source:UniProtKB/TrEMBL;Acc:L8H3J1]	0.036374	0.468821333	0.432447333	2.27E-06
3h	ACA1_174900/clean/scf7180000084741:826211-826287:-	ACA1_174900	Rasrelated protein Rab-32, putative [Source:UniProtKB/TrEMBL;Acc:L8HHY7]	0.004574847	0.436574667	0.43199982	1.31E-12
3h	ACA1_301690/clean/scf7180000083222:19137-19205:-	ACA1_301690	MoCF_biosynth domain-containing protein [Source:UniProtKB/TrEMBL;Acc:L8HJW0]	0.006792571	0.438772	0.431979429	5.79E-10
3h	ACA1_336100/clean/scf7180000084593:24490-24579:-	ACA1_336100	Ribosomal protein L12, putative [Source:UniProtKB/TrEMBL;Acc:L8H1R0]	0.009908347	0.441776333	0.431867987	0.000271612
3h	ACA1_380780/clean/scf7180000084712:87267-87363:-	ACA1_380780	hypothetical protein	0.010132177	0.441966667	0.43183449	2.37E-07
3h	ACA1_076990/clean/scf7180000084612:209772-209852:-	ACA1_076990	Aminotran_5 domain-containing protein [Source:UniProtKB/TrEMBL;Acc:L8GNM9]	0.00561043	0.437442333	0.431831903	3.36E-10
3h	ACA1_290190/clean/scf7180000084777:548007-548067:+	ACA1_290190	SCF ubiquitin ligase complex protein SKP1a, putative [Source:UniProtKB/TrEMBL;Acc:L8HJ88]	0.032535833	0.464361	0.431825167	1.26E-07
3h	ACA1_243850/clean/scf7180000084763:362555-362616:+	ACA1_243850	Ras-GAP domain-containing protein [Source:UniProtKB/TrEMBL;Acc:L8GJZ5]	0	0.431719333	0.431719333	0.000246776
3h	ACA1_061500/clean/scf7180000084599:945591-945680:+	ACA1_061500	Ras-GAP domain-containing protein [Source:UniProtKB/TrEMBL;Acc:L8GWM5]	0.00301485	0.434688667	0.431673817	1.10E-07
3h	ACA1_046730/clean/scf7180000084577:175120-175236:-	ACA1_046730	SPRY domain containing protein [Source:UniProtKB/TrEMBL;Acc:L8HB40]	0.0162786	0.447894333	0.431615733	2.17E-07
3h	ACA1_109730/clean/scf7180000084663:13183-13260:-	ACA1_109730	Replication factor C subunit 1 [Source:UniProtKB/TrEMBL;Acc:L8HIF4]	0.002769353	0.434363667	0.431594313	2.42E-07
3h	ACA1_360980/clean/scf7180000084622:214412-214483:+	ACA1_360980	hypothetical protein	0	0.431521633	0.431521633	2.83E-06
3h	ACA1_140730/clean/scf7180000084707:109354-109458:-	ACA1_140730	Kelch repeat protein [Source:UniProtKB/TrEMBL;Acc:L8GGS1]	0.01662174	0.448103333	0.431481593	3.02E-06
3h	ACA1_140610/clean/scf7180000084707:102916-102996:-	ACA1_140610	Poly [ADP-ribose] polymerase [Source:UniProtKB/TrEMBL;Acc:L8GGG7]	0.007113067	0.438504667	0.4313916	0.000637974
3h	ACA1_359020/clean/scf7180000084742:51981-52065:+	ACA1_359020	HpcH/HpaI aldolase/citrate lyase subfamily protein [Source:UniProtKB/TrEMBL;Acc:L8GLJ7]	0.004611737	0.435834667	0.43122293	4.66E-07
3h	ACA1_062350/clean/scf7180000084599:1169339-1169434:+	ACA1_062350	ADF-H domain-containing protein [Source:UniProtKB/TrEMBL;Acc:L8GY68]	0.00485292	0.436040333	0.431187413	9.28E-09
3h	ACA1_215900/clean/scf7180000084758:400405-400524:+	ACA1_215900	RAP1A, member of RAS oncogene family [Source:UniProtKB/TrEMBL;Acc:L8GR43]	0.00314385	0.434229333	0.431085483	1.61E-13
3h	ACA1_063560/clean/scf7180000084599:1449613-1449716:+	ACA1_063560	hypothetical protein	0.001643033	0.432723667	0.431080633	2.82E-08
3h	ACA1_038360/clean/scf7180000084568:134795-134894:+	ACA1_038360	Kelch repeatcontaining protein [Source:UniProtKB/TrEMBL;Acc:L8GN55]	0.0035056	0.434558333	0.431052733	1.08E-10
3h	ACA1_188690/clean/scf7180000084747:192460-192537:-	ACA1_188690	PhytanoylCoA dioxygenase domain containing 1 (Predicted), putative [Source:UniProtKB/TrEMBL;Acc:L8GUY3]	0.101894233	0.532938333	0.4310441	0.002169295
3h	ACA1_271570/clean/scf7180000084771:75525-75627:-	ACA1_271570	Protein kinase domain-containing protein [Source:UniProtKB/TrEMBL;Acc:L8GP11]	0.006009257	0.436904333	0.430895077	1.25E-09
3h	ACA1_199490/clean/scf7180000084753:243507-243587:+	ACA1_199490	ATPdependent RNA helicase DBP5, putative [Source:UniProtKB/TrEMBL;Acc:L8H357]	0.003082507	0.433964333	0.430881827	7.39E-09
3h	ACA1_183740/clean/scf7180000084745:493341-493409:+	ACA1_183740	hypothetical protein	0.008156453	0.438803333	0.43064688	1.77E-05
3h	ACA1_185320/clean/scf7180000084746:93897-93963:+	ACA1_185320	hypothetical protein	0.001092897	0.431718	0.430625103	4.18E-09
3h	ACA1_305550/clean/scf7180000083609:19660-19739:+	ACA1_305550	Folate gamma-glutamyl hydrolase [Source:UniProtKB/TrEMBL;Acc:L8GKY1]	0.011369743	0.441951	0.430581257	1.19E-10
3h	ACA1_221050/clean/scf7180000084758:987080-987154:-	ACA1_221050	hypothetical protein	0.009007123	0.439584667	0.430577543	1.12E-05
3h	ACA1_279050/clean/scf7180000084776:301443-301573:-	ACA1_279050	PCI domain-containing protein [Source:UniProtKB/TrEMBL;Acc:L8H5Y4]	0.006337853	0.436896	0.430558147	2.56E-09
3h	ACA1_228670/clean/scf7180000084761:188026-188136:+	ACA1_228670	hypothetical protein	0.025056333	0.455525333	0.430469	4.95E-06
3h	ACA1_128840/clean/scf7180000084697:108026-108092:+	ACA1_128840	CMP/dCMP-type deaminase domain-containing protein [Source:UniProtKB/TrEMBL;Acc:L8GVK0]	0.016992267	0.44739	0.430397733	5.50E-05
3h	ACA1_272960/clean/scf7180000084773:4513-4572:-	ACA1_272960	Ras-GAP domain-containing protein [Source:UniProtKB/TrEMBL;Acc:L8GFH7]	0	0.430229333	0.430229333	1.16E-08
3h	ACA1_119170/clean/scf7180000084672:31357-31436:+	ACA1_119170	hypothetical protein	0.044928133	0.475124	0.430195867	0.000171805
3h	ACA1_052610/clean/scf7180000084594:123358-123437:+	ACA1_052610	hypothetical protein	0.0327171	0.462902033	0.430184933	4.34E-06
3h	ACA1_331590/clean/scf7180000084578:7864-7965:+	ACA1_331590	ATPdependent DNA helicase, RecQ subfamily protein [Source:UniProtKB/TrEMBL;Acc:L8HBM3]	0.006172833	0.436277333	0.4301045	1.18E-05
3h	ACA1_372660/clean/scf7180000084679:41225-41299:-	ACA1_372660	hypothetical protein	0	0.430038667	0.430038667	0.000187147
3h	ACA1_290480/clean/scf7180000084777:599741-599824:+	ACA1_290480	AminoacyltRNA hydrolase [Source:UniProtKB/TrEMBL;Acc:L8HI62]	0.009005877	0.4389962	0.429990323	0.000264934
3h	ACA1_054420/clean/scf7180000084594:489284-489375:+	ACA1_054420	N-formylglutamate amidohydrolase [Source:UniProtKB/TrEMBL;Acc:L8H8B3]	0.00042812	0.430324333	0.429896213	6.86E-05
3h	ACA1_278860/clean/scf7180000084776:259282-259393:-	ACA1_278860	Glycosylphosphatidylinositol anchor attachment 1 protein [Source:UniProtKB/TrEMBL;Acc:L8H6Z3]	0	0.429828	0.429828	8.76E-08
3h	ACA1_062680/clean/scf7180000084599:1272290-1272416:+	ACA1_062680	hypothetical protein	0.026012267	0.4558297	0.429817433	3.03E-06
3h	ACA1_175830/clean/scf7180000084741:1070881-1070965:-	ACA1_175830	Mago nashi protein [Source:UniProtKB/TrEMBL;Acc:L8HKH2]	0.005406907	0.435020333	0.429613427	3.64E-09
3h	ACA1_296970/clean/scf7180000084777:1451620-1451693:-	ACA1_296970	Kelch domain containing 1, putative [Source:UniProtKB/TrEMBL;Acc:L8HK47]	0	0.429527067	0.429527067	0.000349586
3h	ACA1_069880/clean/scf7180000084603:585889-585970:-	ACA1_069880	hypothetical protein	0.00409261	0.433598	0.42950539	1.92E-07
3h	ACA1_272960/clean/scf7180000084773:5189-5354:-	ACA1_272960	Ras-GAP domain-containing protein [Source:UniProtKB/TrEMBL;Acc:L8GFH7]	0.012832027	0.442176667	0.42934464	2.38E-07
3h	ACA1_054710/clean/scf7180000084594:559354-559408:-	ACA1_054710	Ras family protein [Source:UniProtKB/TrEMBL;Acc:L8H6F4]	0.003327587	0.432664	0.429336413	1.65E-08
3h	ACA1_380820/clean/scf7180000084712:93901-93958:+	ACA1_380820	tRNA-binding domain-containing protein [Source:UniProtKB/TrEMBL;Acc:L8GMV0]	0.036331	0.465473667	0.429142667	4.99E-08
3h	ACA1_309340/clean/scf7180000083758:6371-6447:+	ACA1_309340	Protein tyrosine phosphataselike protein, PTPLA [Source:UniProtKB/TrEMBL;Acc:L8GR24]	0.00526953	0.434272333	0.429002803	1.50E-07
3h	ACA1_074770/clean/scf7180000084608:82846-82935:-	ACA1_074770	Vps52 / Sac2 family protein [Source:UniProtKB/TrEMBL;Acc:L8HFG7]	0	0.428684	0.428684	7.53E-08
3h	ACA1_107630/clean/scf7180000084660:236310-236422:+	ACA1_107630	DNA-(apurinic or apyrimidinic site) lyase [Source:UniProtKB/TrEMBL;Acc:L8GMX8]	0.350681	0.779043333	0.428362333	0.017722881
3h	ACA1_259480/clean/scf7180000084769:348383-348468:-	ACA1_259480	EnoylCoA hydratase/isomerase [Source:UniProtKB/TrEMBL;Acc:L8GFL3]	0.117402667	0.545731	0.428328333	4.80E-05
3h	ACA1_271590/clean/scf7180000084771:80389-80516:+	ACA1_271590	G_PROTEIN_RECEP_F2_4 domain-containing protein [Source:UniProtKB/TrEMBL;Acc:L8GNS7]	0.171778333	0.600034667	0.428256333	0.00120094
3h	ACA1_360210/clean/scf7180000084622:69456-69542:+	ACA1_360210	Fbox domain containing protein [Source:UniProtKB/TrEMBL;Acc:L8HF19]	0.01787517	0.445649667	0.427774497	4.53E-05
3h	ACA1_377270/clean/scf7180000084711:18337-18459:+	ACA1_377270	RING-type domain-containing protein [Source:UniProtKB/TrEMBL;Acc:L8GSR8]	0.00943834	0.436997667	0.427559327	3.45E-07
3h	ACA1_329450/clean/scf7180000084562:13493-13567:-	ACA1_329450	hypothetical protein	0	0.427329533	0.427329533	6.10E-08
3h	ACA1_203070/clean/scf7180000084754:134949-135029:+	ACA1_203070	Fbox domain containing protein [Source:UniProtKB/TrEMBL;Acc:L8GUJ5]	0	0.427309	0.427309	2.92E-08
3h	ACA1_173130/clean/scf7180000084741:408761-408847:-	ACA1_173130	hypothetical protein	0.076736033	0.503800667	0.427064633	0.004174653
3h	ACA1_174110/clean/scf7180000084741:614339-614452:+	ACA1_174110	hypothetical protein	0.072661633	0.499558	0.426896367	1.61E-05
3h	ACA1_343290/clean/scf7180000084638:15622-15699:+	ACA1_343290	hypothetical protein	0.025147733	0.451987	0.426839267	2.32E-07
3h	ACA1_204520/clean/scf7180000084754:303203-303287:-	ACA1_204520	hypothetical protein	0	0.426582	0.426582	0.016475166
3h	ACA1_024220/clean/scf7180000084441:20479-20587:+	ACA1_024220	Purinergic receptor, putative [Source:UniProtKB/TrEMBL;Acc:L8GLB7]	0.0303989	0.456947	0.4265481	9.81E-06
3h	ACA1_220170/clean/scf7180000084758:881997-882104:+	ACA1_220170	RSL1D1 protein [Source:UniProtKB/TrEMBL;Acc:L8GRK5]	0	0.426532333	0.426532333	1.02E-05
3h	ACA1_269380/clean/scf7180000084770:991275-991377:+	ACA1_269380	hypothetical protein	0.021783767	0.448216333	0.426432567	9.48E-06
3h	ACA1_198700/clean/scf7180000084753:154317-154378:-	ACA1_198700	ABC transporter domain-containing protein [Source:UniProtKB/TrEMBL;Acc:L8H2D9]	0.017792	0.444206333	0.426414333	4.76E-08
3h	ACA1_330350/clean/scf7180000084566:44131-44210:-	ACA1_330350	SEP domain containing protein [Source:UniProtKB/TrEMBL;Acc:L8GJV6]	0.004724117	0.430997667	0.42627355	4.83E-09
3h	ACA1_184420/clean/scf7180000084745:639596-639680:+	ACA1_184420	Cathepsin Llike proteinase [Source:UniProtKB/TrEMBL;Acc:L8H8J9]	0.004245183	0.430423	0.426177817	1.09E-10
3h	ACA1_166940/clean/scf7180000084736:32834-32936:-	ACA1_166940	Ras-GAP domain-containing protein [Source:UniProtKB/TrEMBL;Acc:L8H0R6]	0.002835123	0.428827333	0.42599221	6.41E-07
3h	ACA1_332300/clean/scf7180000084579:9249-9386:+	ACA1_332300	hypothetical protein	0.050779667	0.476689333	0.425909667	0.008157802
3h	ACA1_076410/clean/scf7180000084612:66157-66239:-	ACA1_076410	hypothetical protein	0.0330944	0.458994	0.4258996	3.38E-07
3h	ACA1_298780/clean/scf7180000082992:23912-23988:+	ACA1_298780	LIM domain containing protein [Source:UniProtKB/TrEMBL;Acc:L8H085]	0.0195047	0.445373	0.4258683	9.04E-08
3h	ACA1_331110/clean/scf7180000084575:3232-3336:-	ACA1_331110	RAP1A, member of RAS oncogene family [Source:UniProtKB/TrEMBL;Acc:L8GU20]	0.013265133	0.438956333	0.4256912	1.45E-08
3h	ACA1_307560/clean/scf7180000083680:38070-38177:-	ACA1_307560	WD domain, G-beta repeat-containing protein [Source:UniProtKB/TrEMBL;Acc:L8H741]	0	0.425615	0.425615	7.66E-07
3h	ACA1_113850/clean/scf7180000084669:387282-387362:-	ACA1_113850	PMSR domain-containing protein [Source:UniProtKB/TrEMBL;Acc:L8H3F4]	0.001978666	0.427579333	0.425600668	9.46E-13
3h	ACA1_306840/clean/scf7180000083668:16847-16972:+	ACA1_306840	RTP1_C1 domain-containing protein [Source:UniProtKB/TrEMBL;Acc:L8GQF6]	0	0.425579667	0.425579667	2.94E-05
3h	ACA1_279550/clean/scf7180000084776:412272-412359:-	ACA1_279550	CBS domain containing protein [Source:UniProtKB/TrEMBL;Acc:L8H8C1]	0.010930147	0.436426667	0.42549652	3.41E-06
3h	ACA1_288290/clean/scf7180000084777:161048-161128:+	ACA1_288290	SH3 domain-containing protein [Source:UniProtKB/TrEMBL;Acc:L8HKD1]	0.012256433	0.437658667	0.425402233	5.56E-07
3h	ACA1_203020/clean/scf7180000084754:123698-123792:-	ACA1_203020	Protein pns1, putative [Source:UniProtKB/TrEMBL;Acc:L8GUJ1]	0.007750087	0.4327674	0.425017313	1.49E-07
3h	ACA1_133350/clean/scf7180000084701:37041-37138:-	ACA1_133350	PlsC domain-containing protein [Source:UniProtKB/TrEMBL;Acc:L8H3L0]	0.004876267	0.429702333	0.424826067	8.72E-07
3h	ACA1_157530/clean/scf7180000084724:75409-75582:-	ACA1_157530	hypothetical protein	0.013402617	0.437838667	0.42443605	1.38E-08
3h	ACA1_179970/clean/scf7180000084744:11109-11306:+	ACA1_179970	PPM-type phosphatase domain-containing protein [Source:UniProtKB/TrEMBL;Acc:L8GF73]	0.075216867	0.499648667	0.4244318	2.86E-06
3h	ACA1_062650/clean/scf7180000084599:1264261-1264341:+	ACA1_062650	hypothetical protein	0	0.424311333	0.424311333	8.80E-07
3h	ACA1_279550/clean/scf7180000084776:413027-413129:-	ACA1_279550	CBS domain containing protein [Source:UniProtKB/TrEMBL;Acc:L8H8C1]	0.01067786	0.434949	0.42427114	4.33E-06
3h	ACA1_057940/clean/scf7180000084599:214420-214484:+	ACA1_057940	Alpha-mannosidase [Source:UniProtKB/TrEMBL;Acc:L8GX61]	5.49E-05	0.4242205	0.424165596	2.43E-06
3h	ACA1_315050/clean/scf7180000083887:24482-24590:-	ACA1_315050	Chain a, 4ank: a designed ankyrin repeat protein [Source:UniProtKB/TrEMBL;Acc:L8GYU2]	0.01388784	0.437900667	0.424012827	1.81E-07
3h	ACA1_039450/clean/scf7180000084569:31058-31129:-	ACA1_039450	SAP domain-containing protein [Source:UniProtKB/TrEMBL;Acc:L8HI99]	0	0.423892667	0.423892667	5.68E-06
3h	ACA1_083640/clean/scf7180000084629:7780-7874:-	ACA1_083640	Elongation factor 1alpha [Source:UniProtKB/TrEMBL;Acc:L8GYH2]	0.001973087	0.425796333	0.423823247	3.05E-11
3h	ACA1_328770/clean/scf7180000084560:25823-25892:-	ACA1_328770	Aldo_ket_red domain-containing protein [Source:UniProtKB/TrEMBL;Acc:L8GQZ7]	0.003861567	0.427416333	0.423554767	2.14E-14
3h	ACA1_074110/clean/scf7180000084605:26556-26636:-	ACA1_074110	Peptidase [Source:UniProtKB/TrEMBL;Acc:L8HJ70]	0.006318523	0.429806	0.423487477	5.39E-08
3h	ACA1_088960/clean/scf7180000084639:273666-273747:-	ACA1_088960	Beta-lactamase domain-containing protein [Source:UniProtKB/TrEMBL;Acc:L8GU04]	0.005946153	0.4293594	0.423413247	3.08E-05

3h	ACA1_022080/clean/scf7180000084264:12378-12499:+	ACA1_022080	Ankyrin repeat RhoGAP domain containing protein [Source:UniProtKB/TrEMBL;Acc:L8GC83]	0.022805533	0.446129333	0.4233238	0.000202349
3h	ACA1_143770/clean/scf7180000084709:94219-94280:+	ACA1_143770	WD repeatcontaining protein 40A, putative [Source:UniProtKB/TrEMBL;Acc:L8HFH4]	0.381817	0.805042667	0.423225667	0.000938359
3h	ACA1_184600/clean/scf7180000084746:3175-3235:-	ACA1_184600	Alpha-N-acetylglucosaminidase family protein [Source:UniProtKB/TrEMBL;Acc:L8H6D3]	0.002235983	0.425439333	0.42320335	6.85E-05
3h	ACA1_064430/clean/scf7180000084599:1618145-1618232:+	ACA1_064430	hypothetical protein	0.006477593	0.429479667	0.423002073	5.02E-08
3h	ACA1_264330/clean/scf7180000084770:240681-240784:+	ACA1_264330	Abhydrolase_3 domain-containing protein [Source:UniProtKB/TrEMBL;Acc:L8H479]	0.012349426	0.435172667	0.42282324	5.20E-07
3h	ACA1_218390/clean/scf7180000084758:649638-649732:-	ACA1_218390	Glycosyl hydrolase family 20, catalytic domain containing protein [Source:UniProtKB/TrEMBL;Acc:L8GQD2]	0.012068667	0.434869	0.422800333	1.02E-05
3h	ACA1_098880/clean/scf7180000084646:54103-54180:-	ACA1_098880	UDENN domain-containing protein [Source:UniProtKB/TrEMBL;Acc:L8H7R0]	0	0.422616333	0.422616333	9.12E-09
3h	ACA1_399880/clean/scf7180000084775:55725-55835:+	ACA1_399880	Rab-GAP TBC domain-containing protein [Source:UniProtKB/TrEMBL;Acc:L8GGF5]	0.00536557	0.427963	0.42259743	4.27E-06
3h	ACA1_215990/clean/scf7180000084758:424786-424859:+	ACA1_215990	hypothetical protein	0.000299222	0.422813333	0.422514111	9.36E-07
3h	ACA1_287760/clean/scf7180000084777:72896-72958:-	ACA1_287760	JmjC domain-containing protein [Source:UniProtKB/TrEMBL;Acc:L8HIQ1]	0.026282833	0.448739	0.422456167	0.034626212
3h	ACA1_252210/clean/scf7180000084768:144673-144770:+	ACA1_252210	Protein kinase domain-containing protein [Source:UniProtKB/TrEMBL;Acc:L8HAM0]	0.038135367	0.460552	0.422416633	5.40E-05
3h	ACA1_038180/clean/scf7180000084568:91146-91232:+	ACA1_038180	FHA domain containing protein [Source:UniProtKB/TrEMBL;Acc:L8GLQ3]	0.020145767	0.442543333	0.422397567	3.54E-06
3h	ACA1_097790/clean/scf7180000084645:1340064-1340125:-	ACA1_097790	DUF4209 domain-containing protein [Source:UniProtKB/TrEMBL;Acc:L8GJC1]	0.021169367	0.44333	0.422160633	0.000966246
3h	ACA1_352370/clean/scf7180000084704:2343-2421:+	ACA1_352370	Oxidoreductase, short chain dehydrogenase/reductase superfamily protein [Source:UniProtKB/TrEMBL;Acc:L8GTQ2]	0.0113304	0.433466333	0.422135933	2.99E-05
3h	ACA1_066870/clean/scf7180000084600:67042-67112:-	ACA1_066870	Glucose-6-phosphate isomerase [Source:UniProtKB/TrEMBL;Acc:L8GRA3]	0.001451187	0.423536667	0.42208548	1.65E-09
3h	ACA1_175240/clean/scf7180000084741:899197-899283:+	ACA1_175240	Rab32 protein, putative [Source:UniProtKB/TrEMBL;Acc:L8HI25]	0.011665937	0.433662667	0.42199673	1.36E-06
3h	ACA1_290250/clean/scf7180000084777:569774-569844:+	ACA1_290250	hypothetical protein	0	0.421943	0.421943	1.03E-05
3h	ACA1_199500/clean/scf7180000084753:248627-248734:+	ACA1_199500	1acylglycerophosphocholine O-acyltransferase 1, putative [Source:UniProtKB/TrEMBL;Acc:L8H4L3]	0.052890772	0.474712667	0.421821895	0.000656141
3h	ACA1_052260/clean/scf7180000084594:27136-27237:-	ACA1_052260	Leucine rich repeat domain containing protein [Source:UniProtKB/TrEMBL;Acc:L8H5N7]	0.002264013	0.424043	0.421778987	4.27E-06
3h	ACA1_325260/clean/scf7180000084475:40178-40271:+	ACA1_325260	Peptidylprolyl isomerase [Source:UniProtKB/TrEMBL;Acc:L8GPH9]	0.001747303	0.423456	0.421708697	3.01E-09
3h	ACA1_182190/clean/scf7180000084745:150598-150674:-	ACA1_182190	hypothetical protein	0.039710733	0.461039333	0.4213286	0.000256915
3h	ACA1_184270/clean/scf7180000084745:600306-600377:+	ACA1_184270	Ankyrin repeatcontaining protein [Source:UniProtKB/TrEMBL;Acc:L8HAN7]	0.03381384	0.455135	0.42132116	9.23E-06
3h	ACA1_115650/clean/scf7180000084669:637106-637175:+	ACA1_115650	hypothetical protein	0.0309891	0.452143333	0.421154233	6.42E-05
3h	ACA1_157940/clean/scf7180000084724:155695-155765:-	ACA1_157940	Histidine acid phosphatase superfamily protein [Source:UniProtKB/TrEMBL;Acc:L8H9Z4]	0.004152583	0.425292	0.421139417	4.43E-10
3h	ACA1_289770/clean/scf7180000084777:461570-461672:-	ACA1_289770	Type I phosphodiesterase/nucleotide pyrophosphatase family protein [Source:UniProtKB/TrEMBL;Acc:L8HI22]	0.032828467	0.453863667	0.4210352	2.46E-06
3h	ACA1_359690/clean/scf7180000084622:1607-1753:-	ACA1_359690	Rab-GAP TBC domain-containing protein [Source:UniProtKB/TrEMBL;Acc:L8HBR2]	0.033601833	0.454554	0.420952167	2.57E-06
3h	ACA1_216320/clean/scf7180000084758:467825-467908:+	ACA1_216320	Homogentisate 1,2dioxygenase [Source:UniProtKB/TrEMBL;Acc:L8GQ83]	0.0048704	0.425773667	0.420903267	1.48E-11
3h	ACA1_046390/clean/scf7180000084577:84248-84359:-	ACA1_046390	hypothetical protein	0.016113833	0.436949333	0.4208355	6.77E-05
3h	ACA1_074070/clean/scf7180000084605:16230-16468:-	ACA1_074070	PAS domain-containing protein [Source:UniProtKB/TrEMBL;Acc:L8HKA1]	0.021207183	0.4417947	0.420587517	8.02E-06
3h	ACA1_064570/clean/scf7180000084599:1645492-1645560:+	ACA1_064570	Ras subfamily protein [Source:UniProtKB/TrEMBL;Acc:L8GXF9]	0.009279612	0.429781333	0.420501721	2.26E-05
3h	ACA1_289800/clean/scf7180000084777:466602-466662:-	ACA1_289800	J domain-containing protein [Source:UniProtKB/TrEMBL;Acc:L8HJ30]	0.061460833	0.481549	0.420088167	3.51E-05
3h	ACA1_235890/clean/scf7180000084762:379526-379614:+	ACA1_235890	hypothetical protein	0.003460033	0.423526	0.420065967	1.48E-06
3h	ACA1_145900/clean/scf7180000084710:184177-184249:+	ACA1_145900	GSDH domain-containing protein [Source:UniProtKB/TrEMBL;Acc:L8GDY5]	0.017211333	0.437104667	0.419893333	2.37E-05
3h	ACA1_074110/clean/scf7180000084605:25398-25493:-	ACA1_074110	Peptidase [Source:UniProtKB/TrEMBL;Acc:L8HJ70]	0.015835837	0.435613667	0.41977783	3.78E-07
3h	ACA1_113440/clean/scf7180000084669:275010-275200:+	ACA1_113440	hypothetical protein	0.005176897	0.424913333	0.419736437	1.40E-09
3h	ACA1_377450/clean/scf7180000084711:66989-67065:+	ACA1_377450	hypothetical protein	0.014376783	0.433981	0.419604217	7.49E-07
3h	ACA1_096520/clean/scf7180000084645:1033373-1033467:-	ACA1_096520	Cytochrome p450 superfamily protein [Source:UniProtKB/TrEMBL;Acc:L8GLA2]	0	0.419601667	0.419601667	0.000967347
3h	ACA1_228910/clean/scf7180000084761:221754-221849:+	ACA1_228910	hypothetical protein	0.005400967	0.424838667	0.4194377	5.65E-07
3h	ACA1_002430/clean/scf7180000082931:1142-1228:-	ACA1_002430	G domain-containing protein [Source:UniProtKB/TrEMBL;Acc:L8GY39]	0.00491932	0.424350667	0.419431347	1.75E-05
3h	ACA1_123760/clean/scf7180000084686:55557-55645:+	ACA1_123760	Protein kinase domain-containing protein [Source:UniProtKB/TrEMBL;Acc:L8HF31]	0.195221467	0.614586333	0.419364867	0.002649314
3h	ACA1_100600/clean/scf7180000084648:127558-127643:+	ACA1_100600	AAA_28 domain-containing protein [Source:UniProtKB/TrEMBL;Acc:L8GKH3]	0.004906497	0.42424	0.419333503	4.53E-07
3h	ACA1_269280/clean/scf7180000084770:964272-964433:-	ACA1_269280	RNA methyltransferase protein [Source:UniProtKB/TrEMBL;Acc:L8H2P1]	0.014015767	0.433291	0.419275233	8.04E-08
3h	ACA1_298630/clean/scf7180000082992:2946-3045:-	ACA1_298630	Vacuolar protein sorting 16, putative [Source:UniProtKB/TrEMBL;Acc:L8H081]	0.004024567	0.42327	0.419245433	2.47E-07
3h	ACA1_316750/clean/scf7180000083984:3226-3295:+	ACA1_316750	Peptidase, S8/S53 subfamily protein [Source:UniProtKB/TrEMBL;Acc:L8GLP0]	0	0.419225	0.419225	2.89E-05
3h	ACA1_269730/clean/scf7180000084770:1028102-1028227:+	ACA1_269730	Metallophos domain-containing protein [Source:UniProtKB/TrEMBL;Acc:L8H506]	0.038336533	0.457490667	0.419154133	0.019572366
3h	ACA1_091940/clean/scf7180000084645:234122-234192:-	ACA1_091940	Metallopeptidase [Source:UniProtKB/TrEMBL;Acc:L8GIL4]	0.001450853	0.420538667	0.419087813	7.00E-08
3h	ACA1_264550/clean/scf7180000084770:319649-319732:+	ACA1_264550	Cys/Met metabolism PLPdependent enzyme superfamily protein [Source:UniProtKB/TrEMBL;Acc:L8H496]	0.002804057	0.421871	0.419066943	1.03E-07
3h	ACA1_218620/clean/scf7180000084758:679705-679786:-	ACA1_218620	Carboxylic ester hydrolase [Source:UniProtKB/TrEMBL;Acc:L8GRD3]	0.00909207	0.428049333	0.418957263	3.26E-08
3h	ACA1_053470/clean/scf7180000084594:286488-286567:-	ACA1_053470	hypothetical protein	0.018839993	0.437736333	0.41889634	5.59E-06
3h	ACA1_366940/clean/scf7180000084641:520374-520490:-	ACA1_366940	BTB/POZ domain containing protein [Source:UniProtKB/TrEMBL;Acc:L8GPM1]	0.0934972	0.512358333	0.418861133	0.002397045
3h	ACA1_199010/clean/scf7180000084753:192086-192182:+	ACA1_199010	JmjC domain-containing protein [Source:UniProtKB/TrEMBL;Acc:L8H2E5]	0.006843243	0.425282667	0.418439423	5.22E-08
3h	ACA1_174040/clean/scf7180000084741:585590-585728:-	ACA1_174040	Protein kinase domain-containing protein [Source:UniProtKB/TrEMBL;Acc:L8HK27]	0.00044212	0.418673	0.41823088	9.44E-08
3h	ACA1_208430/clean/scf7180000084757:189819-189937:+	ACA1_208430	hypothetical protein	0.0245852	0.442810333	0.418225133	4.60E-05
3h	ACA1_176030/clean/scf7180000084741:1109438-1109561:-	ACA1_176030	ATPase, AAA domain containing protein [Source:UniProtKB/TrEMBL;Acc:L8HI90]	0.0133954	0.431570333	0.418174933	6.31E-05
3h	ACA1_292880/clean/scf7180000084777:924787-924890:-	ACA1_292880	RhoGEF domain containing protein [Source:UniProtKB/TrEMBL;Acc:L8HJL4]	0.00979121	0.427670333	0.417879123	2.44E-05
3h	ACA1_070900/clean/scf7180000084603:797496-797640:+	ACA1_070900	MMtag domain-containing protein [Source:UniProtKB/TrEMBL;Acc:L8HD49]	0.012097347	0.429946333	0.417848987	9.23E-05
3h	ACA1_218620/clean/scf7180000084758:680745-680883:-	ACA1_218620	Carboxylic ester hydrolase [Source:UniProtKB/TrEMBL;Acc:L8GRD3]	0.003401189	0.421231333	0.417830144	4.13E-07
3h	ACA1_263610/clean/scf7180000084770:104388-104476:+	ACA1_263610	hypothetical protein	0.014454693	0.431918333	0.41746364	3.82E-08
3h	ACA1_103150/clean/scf7180000084653:7755-7852:+	ACA1_103150	hypothetical protein	0.0209515	0.437930333	0.416978833	3.35E-07
3h	ACA1_377830/clean/scf7180000084711:127178-127253:+	ACA1_377830	UBIQUITIN_CONJUGAT_2 domain-containing protein [Source:UniProtKB/TrEMBL;Acc:L8GSV2]	0.043605433	0.460538667	0.416933233	2.79E-06
3h	ACA1_095960/clean/scf7180000084645:920020-920105:-	ACA1_095960	hypothetical protein	0.002469137	0.419276	0.416806863	6.21E-07
3h	ACA1_248600/clean/scf7180000084766:93354-93479:+	ACA1_248600	DUF4114 domain-containing protein [Source:UniProtKB/TrEMBL;Acc:L8GXW7]	0.015063667	0.431803	0.416739333	1.49E-08
3h	ACA1_173160/clean/scf7180000084741:414877-414975:+	ACA1_173160	hypothetical protein	0.03959123	0.456279	0.41668777	3.59E-05
3h	ACA1_098920/clean/scf7180000084646:64080-64186:-	ACA1_098920	DH domain-containing protein [Source:UniProtKB/TrEMBL;Acc:L8H5S9]	0	0.416556667	0.416556667	1.31E-08
3h	ACA1_315050/clean/scf7180000083887:23635-23738:-	ACA1_315050	Chain a, 4ank: a designed ankyrin repeat protein [Source:UniProtKB/TrEMBL;Acc:L8GYU2]	0.063661367	0.480162333	0.416500967	0.000283166
3h	ACA1_325600/clean/scf7180000084495:42202-42303:-	ACA1_325600	Protein kinase domain containing protein [Source:UniProtKB/TrEMBL;Acc:L8HB78]	0.00347063	0.419878	0.41640737	1.48E-08
3h	ACA1_319100/clean/scf7180000084239:14464-14523:+	ACA1_319100	hypothetical protein	0.107819933	0.524221	0.416401067	0.001434414
3h	ACA1_234290/clean/scf7180000084762:261484-261542:-	ACA1_234290	Deacetylase sirtuin-type domain-containing protein [Source:UniProtKB/TrEMBL;Acc:L8H3J1]	0.211761333	0.628040333	0.416279	0.002828053
3h	ACA1_265340/clean/scf7180000084770:445180-445278:+	ACA1_265340	Protein kinase domain containing protein [Source:UniProtKB/TrEMBL;Acc:L8H258]	0	0.416153	0.416153	1.61E-07
3h	ACA1_121840/clean/scf7180000084682:109160-109230:+	ACA1_121840	Protein kinase domain-containing protein [Source:UniProtKB/TrEMBL;Acc:L8GFF3]	0.003436433	0.419560567	0.416124133	3.24E-07
3h	ACA1_389080/clean/scf7180000084733:193049-193150:-	ACA1_389080	Fbox domain containing protein [Source:UniProtKB/TrEMBL;Acc:L8GDU7]	0.019818433	0.435709	0.415890567	0.000269984
3h	ACA1_297750/clean/scf7180000082853:1097-1230:-	ACA1_297750	14_3_3 domain-containing protein [Source:UniProtKB/TrEMBL;Acc:L8HE45]	0	0.415882	0.415882	0.027654063
3h	ACA1_100560/clean/scf7180000084648:114503-114581:+	ACA1_100560	hypothetical protein	0.045957667	0.461800667	0.415843	0.000101119
3h	ACA1_393010/clean/scf7180000084756:26612-26709:+	ACA1_393010	AA_TRNA_LIGASE_II domain-containing protein [Source:UniProtKB/TrEMBL;Acc:L8H0A5]	0.003335817	0.419122667	0.41578685	4.46E-08
3h	ACA1_199640/clean/scf7180000084753:286627-286768:-	ACA1_199640	RFX-type winged-helix domain-containing protein [Source:UniProtKB/TrEMBL;Acc:L8H5G1]	0.030420167	0.446190333	0.415770167	2.02E-05
3h	ACA1_169220/clean/scf7180000084739:30735-30818:+	ACA1_169220	ANK_REP_REGION domain-containing protein [Source:UniProtKB/TrEMBL;Acc:L8HFG6]	0.000701017	0.416411	0.415709983	3.49E-08
3h	ACA1_373540/clean/scf7180000084679:96177-96301:-	ACA1_373540	hypothetical protein	0.0580031	0.473694	0.4156909	3.79E-05
3h	ACA1_114500/clean/scf7180000084669:487494-487582:-	ACA1_114500	Macro domain-containing protein [Source:UniProtKB/TrEMBL;Acc:L8H6J5]	0.00210207	0.417544	0.41544193	1.64E-08
3h	ACA1_188290/clean/scf7180000084747:146175-146239:+	ACA1_188290	IPT/TIG domain-containing protein [Source:UniProtKB/TrEMBL;Acc:L8GSG4]	0.00834077	0.423703833	0.415363063	1.83E-07
3h	ACA1_156050/clean/scf7180000084723:13874-13934:+	ACA1_156050	Armadillo/betacatenin-like repeat domain containing protein [Source:UniProtKB/TrEMBL;Acc:L8GIE3]	0.111111	0.526441667	0.415330667	0.039183039
3h	ACA1_064540/clean/scf7180000084599:1638097-1638240:-	ACA1_064540	hypothetical protein	0.030393967	0.44569	0.415296033	3.33E-05
3h	ACA1_369190/clean/scf7180000084664:268491-268582:+	ACA1_369190	PCI domain-containing protein [Source:UniProtKB/TrEMBL;Acc:L8GZ19]	0.000636643	0.415922233	0.41528559	3.31E-10
3h	ACA1_100460/clean/scf7180000084648:88176-88233:-	ACA1_100460	ABC transporter [Source:UniProtKB/TrEMBL;Acc:L8GK53]	0	0.415164333	0.415164333	0.000262617
3h	ACA1_278330/clean/scf7180000084776:197150-197238:+	ACA1_278330	WH1 domain containing protein [Source:UniProtKB/TrEMBL;Acc:L8H6L6]	0.023663033	0.438727	0.415063967	7.82E-05
3h	ACA1_072730/clean/scf7180000084603:1278745-1278848:+	ACA1_072730	hypothetical protein	0.003581937	0.418621	0.415039063	0.000241759
3h	ACA1_267250/clean/scf7180000084770:837628-837712:+	ACA1_267250	Oxidoreductase, short chain dehydrogenase/reductase superfamily protein [Source:UniProtKB/TrEMBL;Acc:L8H4S7]	0.026519733	0.441514	0.414994267	2.17E-08
3h	ACA1_216330/clean/scf7180000084758:470543-470701:+	ACA1_216330	hypothetical protein	0.0652011	0.480074333	0.414873233	6.29E-05
3h	ACA1_240070/clean/scf7180000084763:194838-194958:-	ACA1_240070	Carboxylic ester hydrolase [Source:UniProtKB/TrEMBL;Acc:L8GJU3]	0.016074267	0.430794667	0.4147204	1.47E-08
3h	ACA1_091930/clean/scf7180000084645:231444-231532:-	ACA1_091930	hypothetical protein	0.026548333	0.441202333	0.414654	3.85E-06
3h	ACA1_376170/clean/scf7180000084685:40580-40657:-	ACA1_376170	Aa_trans domain-containing protein [Source:UniProtKB/TrEMBL;Acc:L8HFY8]	0.006506353	0.421002667	0.414496314	4.32E-06
3h	ACA1_071130/clean/scf7180000084603:867985-868066:-	ACA1_071130	hypothetical protein	0.012493333	0.426789667	0.414296333	0.000145234
3h	ACA1_123350/clean/scf7180000084686:16563-16641:-	ACA1_123350	hypothetical protein	0.00296331	0.417107667	0.414144357	9.20E-08
3h	ACA1_184890/clean/scf7180000084746:64236-64321:-	ACA1_184890	hypothetical protein	0.044488633	0.458423667	0.413935033	0.000191396
3h	ACA1_325160/clean/scf7180000084475:13430-13504:+	ACA1_325160	Rab7/RabGfamily small GTPase [Source:UniProtKB/TrEMBL;Acc:L8GRY8]	0.025166567	0.439062	0.413895433	4.18E-08
3h	ACA1_012260/clean/scf7180000083740:2627-2698:+	ACA1_012260	hypothetical protein	0.006361593	0.420216	0.413854407	4.17E-10
3h	ACA1_257950/clean/scf7180000084769:185729-185812:-	ACA1_257950	hypothetical protein	0.00125023	0.415095	0.41384477	7.38E-08
3h	ACA1_163930/clean/scf7180000084729:115954-116010:+	ACA1_163930	Peptidase_M14 domain-containing protein [Source:UniProtKB/TrEMBL;Acc:L8GQX3]	0.013901037	0.427680333	0.413779297	1.49E-08
3h	ACA1_072260/clean/scf7180000084603:1181931-1182001:-	ACA1_072260	PNPLA domain-containing protein [Source:UniProtKB/TrEMBL;Acc:L8HDN5]	0.050865167	0.464595	0.413729833	9.01E-05
3h	ACA1_063540/clean/scf7180000084599:1446534-1446604:+	ACA1_063540	hypothetical protein	0.000111074	0.413749	0.413637926	1.34E-09
3h	ACA1_239240/clean/scf7180000084763:147302-147388:+	ACA1_239240	DH domain-containing protein [Source:UniProtKB/TrEMBL;Acc:L8GKG2]	0.006218957	0.419789333	0.413570377	9.97E-06
3h	ACA1_063750/clean/scf7180000084599:1495816-1495887:+	ACA1_063750	TauD domain-containing protein [Source:UniProtKB/TrEMBL;Acc:L8GZU6]	0.05163132	0.465141	0.41350968	0.006599484
3h	ACA1_285540/clean/scf7180000084776:992201-992294:-	ACA1_285540	GPS domain-containing protein [Source:UniProtKB/TrEMBL;Acc:L8H7T4]	0	0.413480667	0.413480667	1.91E-05
3h	ACA1_281330/clean/scf7180000084776:590958-591045:-	ACA1_281330	Protein kinase domain containing protein [Source:UniProtKB/TrEMBL;Acc:L8H6A8]	0	0.413336	0.413336	1.84E-06
3h	ACA1_189820/clean/scf7180000084748:96180-96262:-	ACA1_189820	Protein bcp1, putative [Source:UniProtKB/TrEMBL;Acc:L8GGI0]	0.013438967	0.426611	0.413172033	0.000161991
3h	ACA1_217140/clean/scf7180000084758:525585-525697:+	ACA1_217140	hypothetical protein	0.000690327	0.413831667	0.41314134	5.06E-09
3h	ACA1_333370/clean/scf7180000084581:60408-60479:-	ACA1_333370	LIM domain containing protein [Source:UniProtKB/TrEMBL;Acc:L8HGV2]	0.016129033	0.429201333	0.4130723	2.19E-05
3h	ACA1_182840/clean/scf7180000084745:262855-263104:-	ACA1_182840	MATE efflux family subfamily protein [Source:UniProtKB/TrEMBL;Acc:L8H859]	0.00368007	0.416745667	0.413065597	0.000207407
3h	ACA1_244080/clean/scf7180000084763:371339-371402:+	ACA1_244080	PCI domain-containing protein [Source:UniProtKB/TrEMBL;Acc:L8GL06]	0.005114673	0.418170667	0.413055993	1.65E-09
3h	ACA1_201400/clean/scf7180000084753:543628-543713:+	ACA1_201400	CBS domain containing protein [Source:UniProtKB/TrEMBL;Acc:L8H3G1]	0.012885	0.425887333	0.413002333	6.59E-08
3h	ACA1_144000/clean/scf7180000084709:123119-123192:+	ACA1_144000	RhoGEF domain containing protein [Source:UniProtKB/TrEMBL;Acc:L8HFT1]	0.006039363	0.418869333	0.41282997	1.03E-05
3h	ACA1_054370/clean/scf7180000084594:474981-475067:-	ACA1_054370	Fe2OG dioxygenase domain-containing protein [Source:UniProtKB/TrEMBL;Acc:L8H8A8]	0.461397	0.874148	0.412751	0.000293469
3h	ACA1_282260/clean/scf7180000084776:640135-640196:-	ACA1_282260	R3H domain containing protein [Source:UniProtKB/TrEMBL;Acc:L8H768]	0.0281303	0.440781533	0.412651233	0.00024087
3h	ACA1_291600/clean/scf7180000084777:783503-783632:-	ACA1_291600	Macro domain-containing protein [Source:UniProtKB/TrEMBL;Acc:L8HJG2]	0.0055892	0.418106667	0.412517467	1.49E-06
3h	ACA1_387890/clean/scf7180000084733:83111-83182:+	ACA1_387890	PH domain containing protein [Source:UniProtKB/TrEMBL;Acc:L8GE85]	0	0.412301	0.412301	7.12E-10
3h	ACA1_181620/clean/scf7180000084745:28784-28865:+	ACA1_181620	hypothetical protein	0	0.412282667	0.412282667	4.83E-06
3h	ACA1_341060/clean/scf7180000084620:41362-41440:-	ACA1_341060	RhoGAP domain containing protein [Source:UniProtKB/TrEMBL;Acc:L8GTM9]	0.047389933	0.459665	0.412275067	1.69E-06
3h	ACA1_268930/clean/scf7180000084770:944267-944367:+	ACA1_268930	PWI domain-containing protein [Source:UniProtKB/TrEMBL;Acc:L8H2N5]	0.003816213	0.415967	0.412150787	1.57E-06
3h	ACA1_164920/clean/scf7180000084729:312227-312291:-	ACA1_164920	DJ-1_PfpI domain-containing protein [Source:UniProtKB/TrEMBL;Acc:L8GRN3]	0.024320833	0.436459	0.412138167	4.69E-08
3h	ACA1_096400/clean/scf7180000084645:1005006-1005063:+	ACA1_096400	Methyltransf_11 domain-containing protein [Source:UniProtKB/TrEMBL;Acc:L8GJF1]	0.002793073	0.414772333	0.41197926	3.49E-08
3h	ACA1_110930/clean/scf7180000084666:16863-16947:-	ACA1_110930	Nudix hydrolase domain-containing protein [Source:UniProtKB/TrEMBL;Acc:L8H9Z8]	0	0.411874333	0.411874333	9.03E-07
3h	ACA1_057790/clean/scf7180000084599:179391-179504:+	ACA1_057790	Proteasome subunit beta [Source:UniProtKB/TrEMBL;Acc:L8GX50]	0.0034048	0.415227333	0.411822533	1.28E-12
3h	ACA1_050200/clean/scf7180000084589:54929-55015:+	ACA1_050200	Mammalian PTEN tumor suppressorlike, putative [Source:UniProtKB/TrEMBL;Acc:L8H3Q5]	0.0299551	0.441693333	0.411738233	5.28E-06
3h	ACA1_321930/clean/scf7180000084457:17839-17945:+	ACA1_321930	Transporter, major intrinsic protein (MIP) superfamily protein [Source:UniProtKB/TrEMBL;Acc:L8HCR2]	0.002374933	0.414107267	0.411732333	1.42E-05
3h	ACA1_319980/clean/scf7180000084259:22116-22235:-	ACA1_319980	PDEase domain-containing protein [Source:UniProtKB/TrEMBL;Acc:L8HLY7]	0.0305545	0.442220667	0.411666167	1.21E-05
3h	ACA1_150720/clean/scf7180000084716:26078-26203:-	ACA1_150720	TPR_REGION domain-containing protein [Source:UniProtKB/TrEMBL;Acc:L8H0N3]	0.232779333	0.644441333	0.411662	0.012001814
3h	ACA1_360980/clean/scf7180000084622:215036-215125:+	ACA1_360980	hypothetical protein	0.006953337	0.418475333	0.411521997	4.11E-06
3h	ACA1_400220/clean/scf7180000084775:122387-122477:-	ACA1_400220	Abhydrolase_2 domain-containing protein [Source:UniProtKB/TrEMBL;Acc:L8GG24]	0.006967227	0.418436333	0.411469107	6.38E-09
3h	ACA1_376490/clean/scf7180000084685:90440-90523:-	ACA1_376490	TIP41like family protein [Source:UniProtKB/TrEMBL;Acc:L8HIG8]	0.02756058	0.439028667	0.411468087	8.03E-06
3h	ACA1_288660/clean/scf7180000084777:255931-255996:+	ACA1_288660	hypothetical protein	0.005249333	0.416482667	0.411233333	4.71E-07
3h	ACA1_295340/clean/scf7180000084777:1108539-1108622:-	ACA1_295340	Arl5family small GTPase [Source:UniProtKB/TrEMBL;Acc:L8HIM0]	0.012856867	0.423947667	0.4110908	1.63E-10
3h	ACA1_167050/clean/scf7180000084736:63402-63471:-	ACA1_167050	Ras subfamily protein [Source:UniProtKB/TrEMBL;Acc:L8H0S4]	0.044500133	0.455559667	0.411059533	0.000103253
3h	ACA1_396130/clean/scf7180000084759:24941-25060:-	ACA1_396130	Prefoldin, alpha subunit [Source:UniProtKB/TrEMBL;Acc:L8HDI9]	0.01615651	0.427212667	0.411056157	1.44E-10
3h	ACA1_113490/clean/scf7180000084669:295569-295693:-	ACA1_113490	SAM domain-containing protein [Source:UniProtKB/TrEMBL;Acc:L8H3A5]	0.011566571	0.422576067	0.411009495	1.60E-07
3h	ACA1_234790/clean/scf7180000084762:317398-317491:-	ACA1_234790	PPM-type phosphatase domain-containing protein [Source:UniProtKB/TrEMBL;Acc:L8H3L1]	0.033642167	0.444602533	0.410960367	0.00251613
3h	ACA1_068230/clean/scf7180000084603:141492-141580:-	ACA1_068230	DAGKc domain-containing protein [Source:UniProtKB/TrEMBL;Acc:L8HCH8]	0.023185067	0.434050667	0.4108656	7.79E-06
3h	ACA1_246780/clean/scf7180000084763:580522-580609:-	ACA1_246780	Ras subfamily protein [Source:UniProtKB/TrEMBL;Acc:L8GK82]	0.0343298	0.445117333	0.410787533	6.44E-10
3h	ACA1_207360/clean/scf7180000084757:61352-61449:-	ACA1_207360	Tr-type G domain-containing protein [Source:UniProtKB/TrEMBL;Acc:L8GXB6]	0.002889933	0.413671	0.410781067	1.33E-06
3h	ACA1_091910/clean/scf7180000084645:226226-226297:-	ACA1_091910	NIPSNAP family protein [Source:UniProtKB/TrEMBL;Acc:L8GKH2]	0.0068243	0.417474	0.4106497	1.31E-12

3h	ACA1_009910/clean/scf7180000083468:7688-7767:+	ACA1_009910	tRNA-synt_1c domain-containing protein [Source:UniProtKB/TrEMBL;Acc:L8GDZ9]	0.013151533	0.423785	0.410633467	3.80E-05
3h	ACA1_048870/clean/scf7180000084583:6450-6544:-	ACA1_048870	hypothetical protein	0.0347709	0.445373667	0.410602767	1.97E-05
3h	ACA1_320100/clean/scf7180000084352:7150-7238:+	ACA1_320100	VWFA domain-containing protein [Source:UniProtKB/TrEMBL;Acc:L8H729]	0.00981822	0.420272667	0.410454447	0.000151807
3h	ACA1_236050/clean/scf7180000084762:390192-390305:+	ACA1_236050	hypothetical protein	0.021987367	0.4324	0.410412633	0.001223183
3h	ACA1_112030/clean/scf7180000084669:88904-89063:-	ACA1_112030	COMM domain-containing protein [Source:UniProtKB/TrEMBL;Acc:L8H411]	0.00272777	0.413025667	0.410297897	8.60E-08
3h	ACA1_197450/clean/scf7180000084753:11462-11536:-	ACA1_197450	MCD domain-containing protein [Source:UniProtKB/TrEMBL;Acc:L8H321]	0.016564433	0.426386	0.409821567	7.86E-07
3h	ACA1_385410/clean/scf7180000084730:165472-165643:+	ACA1_385410	TRANSKETOLASE_1 domain-containing protein [Source:UniProtKB/TrEMBL;Acc:L8H9A8]	0.022537533	0.432105667	0.409568133	1.85E-07
3h	ACA1_280690/clean/scf7180000084776:525576-525659:+	ACA1_280690	AP_endonuc_2 domain-containing protein [Source:UniProtKB/TrEMBL;Acc:L8H794]	0.0209469	0.430422	0.4094751	1.99E-06
3h	ACA1_294840/clean/scf7180000084777:1046068-1046186:+	ACA1_294840	Transcription elongation factor SII, hS-II-T1 isoform 8, putative [Source:UniProtKB/TrEMBL;Acc:L8HIK4]	0.131048333	0.540483333	0.409435	6.90E-05
3h	ACA1_133590/clean/scf7180000084701:90285-90358:-	ACA1_133590	hypothetical protein	0.029630643	0.438995667	0.409365023	0.000335657
3h	ACA1_144070/clean/scf7180000084709:142464-142609:-	ACA1_144070	Methyltrans_SAM domain-containing protein [Source:UniProtKB/TrEMBL;Acc:L8HHX9]	0.032753667	0.441955667	0.409202	6.39E-07
3h	ACA1_232420/clean/scf7180000084762:22625-22739:+	ACA1_232420	TPR_REGION domain-containing protein [Source:UniProtKB/TrEMBL;Acc:L8H3B0]	0.032246067	0.441433333	0.409187267	4.97E-07
3h	ACA1_197890/clean/scf7180000084753:48477-48554:+	ACA1_197890	hypothetical protein	0.004901967	0.414044667	0.4091427	8.27E-07
3h	ACA1_080580/clean/scf7180000084618:39260-39370:+	ACA1_080580	t-SNARE coiled-coil homology domain-containing protein [Source:UniProtKB/TrEMBL;Acc:L8HBP6]	0.044895067	0.453925667	0.4090306	3.32E-06
3h	ACA1_191490/clean/scf7180000084749:2272-2373:-	ACA1_191490	RTR1-type domain-containing protein [Source:UniProtKB/TrEMBL;Acc:L8GQP2]	0.008614893	0.417583667	0.408968773	3.43E-08
3h	ACA1_155740/clean/scf7180000084722:233024-233111:+	ACA1_155740	Ras subfamily protein [Source:UniProtKB/TrEMBL;Acc:L8H2N0]	0.002379307	0.410989667	0.40861036	3.19E-07
3h	ACA1_323460/clean/scf7180000084468:25142-25256:-	ACA1_323460	DEP domain-containing protein [Source:UniProtKB/TrEMBL;Acc:L8H1P0]	0.037449633	0.445993	0.408543367	6.25E-06
3h	ACA1_073230/clean/scf7180000084603:1376077-1376161:-	ACA1_073230	EHN domain-containing protein [Source:UniProtKB/TrEMBL;Acc:L8HDT9]	0.050743767	0.459262	0.408518233	1.40E-05
3h	ACA1_119410/clean/scf7180000084672:63718-63916:+	ACA1_119410	PCI domain-containing protein [Source:UniProtKB/TrEMBL;Acc:L8HLX4]	0.011358753	0.419702667	0.408343913	4.34E-08
3h	ACA1_369480/clean/scf7180000084664:310357-310435:-	ACA1_369480	SMP-LTD domain-containing protein [Source:UniProtKB/TrEMBL;Acc:L8GYZ6]	0.0190232	0.427332	0.4083088	1.42E-06
3h	ACA1_184260/clean/scf7180000084745:596192-596270:-	ACA1_184260	Phytanoyl-CoA dioxygenase [Source:UniProtKB/TrEMBL;Acc:L8H8I4]	0.034196037	0.442299	0.408102963	2.74E-05
3h	ACA1_234280/clean/scf7180000084762:259084-259148:+	ACA1_234280	hypothetical protein	0.0846197	0.492661667	0.408041967	2.31E-05
3h	ACA1_285540/clean/scf7180000084776:994717-994808:-	ACA1_285540	GPS domain-containing protein [Source:UniProtKB/TrEMBL;Acc:L8H7T4]	0.008130067	0.4161425	0.408012433	0.000171834
3h	ACA1_306840/clean/scf7180000083668:18316-18415:+	ACA1_306840	RTP1_C1 domain-containing protein [Source:UniProtKB/TrEMBL;Acc:L8GQF6]	0.007887887	0.415840667	0.40795278	0.000172926
3h	ACA1_123760/clean/scf7180000084686:56024-56116:+	ACA1_123760	Protein kinase domain-containing protein [Source:UniProtKB/TrEMBL;Acc:L8HF31]	0.01468169	0.422586	0.40790431	7.59E-06
3h	ACA1_246770/clean/scf7180000084763:578592-578670:+	ACA1_246770	Metallophos domain-containing protein [Source:UniProtKB/TrEMBL;Acc:L8GKY3]	0.006206563	0.414033	0.407826437	4.70E-08
3h	ACA1_070980/clean/scf7180000084603:821233-821347:-	ACA1_070980	hypothetical protein	0.031684567	0.439451333	0.407766767	2.83E-07
3h	ACA1_027030/clean/scf7180000084464:26564-26656:-	ACA1_027030	C2 domain-containing protein [Source:UniProtKB/TrEMBL;Acc:L8GJ80]	0.094694267	0.502371667	0.4076774	0.000449107
3h	ACA1_048110/clean/scf7180000084582:24054-24142:-	ACA1_048110	Serine carboxypeptidase s28 protein [Source:UniProtKB/TrEMBL;Acc:L8GD90]	0.00455201	0.412123333	0.407571323	4.15E-10
3h	ACA1_352400/clean/scf7180000084704:9409-9548:-	ACA1_352400	Fn3_like domain-containing protein [Source:UniProtKB/TrEMBL;Acc:L8GTX5]	0.018241167	0.425806333	0.407565167	3.06E-09
3h	ACA1_131910/clean/scf7180000084699:171925-172008:-	ACA1_131910	EF hand domain containing protein [Source:UniProtKB/TrEMBL;Acc:L8GMI5]	0.00402597	0.411536	0.40751003	8.96E-10
3h	ACA1_174690/clean/scf7180000084741:763572-763645:+	ACA1_174690	Deacetylase sirtuin-type domain-containing protein [Source:UniProtKB/TrEMBL;Acc:L8HHW7]	0.00330033	0.4108031	0.40750277	9.79E-07
3h	ACA1_264610/clean/scf7180000084770:336586-336677:-	ACA1_264610	Peptidase_M28 domain-containing protein [Source:UniProtKB/TrEMBL;Acc:L8H243]	0.004713047	0.412138333	0.407425287	1.35E-10
3h	ACA1_197850/clean/scf7180000084753:40430-40513:-	ACA1_197850	HAD hydrolase, family IIA subfamily protein [Source:UniProtKB/TrEMBL;Acc:L8H335]	0.061096533	0.468430667	0.407334133	0.000102031
3h	ACA1_063610/clean/scf7180000084599:1459832-1459965:-	ACA1_063610	Vacuolar protein sorting 55 protein [Source:UniProtKB/TrEMBL;Acc:L8GXE6]	0.038589767	0.445819667	0.4072299	4.85E-05
3h	ACA1_326170/clean/scf7180000084504:46208-46289:+	ACA1_326170	SAM domain-containing protein [Source:UniProtKB/TrEMBL;Acc:L8HIK1]	0.037649367	0.444774333	0.407124967	3.26E-05
3h	ACA1_169220/clean/scf7180000084739:31092-31182:+	ACA1_169220	ANK_REP_REGION domain-containing protein [Source:UniProtKB/TrEMBL;Acc:L8HFG6]	0.00650635	0.413382	0.40687565	7.15E-07
3h	ACA1_097190/clean/scf7180000084645:1180754-1180820:-	ACA1_097190	JmjC domain-containing protein [Source:UniProtKB/TrEMBL;Acc:L8GIZ2]	0.0540741	0.460835667	0.406761567	0.000370132
3h	ACA1_116900/clean/scf7180000084669:868835-868951:+	ACA1_116900	UBA/TSN domain containing protein [Source:UniProtKB/TrEMBL;Acc:L8H4S4]	0.040291933	0.446861667	0.406569733	6.07E-05
3h	ACA1_061500/clean/scf7180000084599:942490-942596:+	ACA1_061500	Ras-GAP domain-containing protein [Source:UniProtKB/TrEMBL;Acc:L8GWM5]	0.00366152	0.410214667	0.406553147	3.92E-07
3h	ACA1_377260/clean/scf7180000084711:15077-15158:+	ACA1_377260	PH domain containing protein [Source:UniProtKB/TrEMBL;Acc:L8GRU5]	0.0202497	0.426772667	0.406522967	7.97E-06
3h	ACA1_385650/clean/scf7180000084730:226379-226465:+	ACA1_385650	Leucine rich repeat domain containing protein [Source:UniProtKB/TrEMBL;Acc:L8HAN9]	0.008531383	0.414822667	0.406291283	2.83E-05
3h	ACA1_095060/clean/scf7180000084645:803777-803882:-	ACA1_095060	Sidoreflexin [Source:UniProtKB/TrEMBL;Acc:L8GL14]	0	0.406159333	0.406159333	1.88E-07
3h	ACA1_140770/clean/scf7180000084707:115399-115490:+	ACA1_140770	ATPase, AAA domain containing protein [Source:UniProtKB/TrEMBL;Acc:L8GJ22]	0.01883578	0.424879667	0.406043887	7.30E-05
3h	ACA1_261080/clean/scf7180000084769:597263-597345:-	ACA1_261080	MIT domain-containing protein [Source:UniProtKB/TrEMBL;Acc:L8GF47]	0.00049805	0.406530667	0.406032617	2.18E-08
3h	ACA1_060530/clean/scf7180000084599:702515-702621:+	ACA1_060530	TPR_REGION domain-containing protein [Source:UniProtKB/TrEMBL;Acc:L8GYY8]	0.04137855	0.447347667	0.405969117	0.002719527
3h	ACA1_232460/clean/scf7180000084762:31294-31396:+	ACA1_232460	Protein kinase domain-containing protein [Source:UniProtKB/TrEMBL;Acc:L8H0X8]	0.0127954	0.4185	0.4057046	8.66E-06
3h	ACA1_174040/clean/scf7180000084741:581343-581475:-	ACA1_174040	Protein kinase domain-containing protein [Source:UniProtKB/TrEMBL;Acc:L8HK27]	0.018416933	0.423907667	0.405490733	4.50E-05
3h	ACA1_227730/clean/scf7180000084761:126064-126165:+	ACA1_227730	FYVE zinc finger domain containing protein [Source:UniProtKB/TrEMBL;Acc:L8H8M9]	0.001648197	0.407093	0.405444803	1.54E-08
3h	ACA1_264850/clean/scf7180000084770:389766-389857:+	ACA1_264850	MaoC-like domain-containing protein [Source:UniProtKB/TrEMBL;Acc:L8H4C0]	0.054726633	0.460020333	0.4052937	8.04E-06
3h	ACA1_226550/clean/scf7180000084761:50597-50687:+	ACA1_226550	Histidine ammonia-lyase [Source:UniProtKB/TrEMBL;Acc:L8H7Q1]	0.003058103	0.408238567	0.405180463	1.60E-06
3h	ACA1_278000/clean/scf7180000084776:150636-150704:+	ACA1_278000	Carrier superfamily protein [Source:UniProtKB/TrEMBL;Acc:L8H5S3]	0.013874117	0.418661333	0.404787217	0.000114069
3h	ACA1_400260/clean/scf7180000084775:131345-131416:+	ACA1_400260	Ranlike small GTPase [Source:UniProtKB/TrEMBL;Acc:L8GIF4]	0	0.404783333	0.404783333	5.46E-05
3h	ACA1_267250/clean/scf7180000084770:837110-837189:+	ACA1_267250	Oxidoreductase, short chain dehydrogenase/reductase superfamily protein [Source:UniProtKB/TrEMBL;Acc:L8H4S7]	0.013401053	0.417632667	0.404231613	1.33E-06
3h	ACA1_015480/clean/scf7180000083799:1937-2030:+	ACA1_015480	hypothetical protein	0.0669506	0.471136333	0.404185733	7.65E-05
3h	ACA1_140770/clean/scf7180000084707:116793-116863:+	ACA1_140770	ATPase, AAA domain containing protein [Source:UniProtKB/TrEMBL;Acc:L8GJ22]	0.025950667	0.430124667	0.404174	3.63E-05
3h	ACA1_031600/clean/scf7180000084522:23098-23182:-	ACA1_031600	Leucine rich repeat-containing protein [Source:UniProtKB/TrEMBL;Acc:L8GSM8]	0.027471567	0.431574667	0.4041031	1.50E-05
3h	ACA1_141880/clean/scf7180000084708:41471-41568:+	ACA1_141880	RNA editing ligase [Source:UniProtKB/TrEMBL;Acc:L8HCM1]	0.009656697	0.413635333	0.403978637	3.92E-05
3h	ACA1_192070/clean/scf7180000084749:93937-94059:+	ACA1_192070	hypothetical protein	0.007815463	0.411689667	0.403874203	1.33E-08
3h	ACA1_382920/clean/scf7180000084721:119288-119370:-	ACA1_382920	Ras subfamily protein [Source:UniProtKB/TrEMBL;Acc:L8GV31]	0.144991067	0.548787667	0.4037966	0.005632832
3h	ACA1_022330/clean/scf7180000084293:9476-9582:+	ACA1_022330	Carbamoylphosphate synthase L chain, ATP-binding, putative [Source:UniProtKB/TrEMBL;Acc:L8GNZ7]	0.002938713	0.406718	0.403779287	1.58E-10
3h	ACA1_097620/clean/scf7180000084645:1298532-1298633:-	ACA1_097620	hypothetical protein	0.000354107	0.404087667	0.40373356	6.71E-07
3h	ACA1_183970/clean/scf7180000084745:532391-532483:-	ACA1_183970	hypothetical protein	0	0.403594667	0.403594667	0.003454355
3h	ACA1_275670/clean/scf7180000084774:82349-82491:-	ACA1_275670	unspecified product	0.026082433	0.429643667	0.403561233	0.000115143
3h	ACA1_277560/clean/scf7180000084776:74553-74614:-	ACA1_277560	F-box domain-containing protein [Source:UniProtKB/TrEMBL;Acc:L8H6G6]	0.0603152	0.463866333	0.403551133	0.000290581
3h	ACA1_325170/clean/scf7180000084475:14416-14537:-	ACA1_325170	MHD domain-containing protein [Source:UniProtKB/TrEMBL;Acc:L8GPP2]	0.004361863	0.407832	0.403470137	7.47E-10
3h	ACA1_368170/clean/scf7180000084664:71184-71347:+	ACA1_368170	Actin-related protein 2/3 complex subunit 5 [Source:UniProtKB/TrEMBL;Acc:L8H192]	0.004096573	0.4073203	0.403223727	7.80E-13
3h	ACA1_237810/clean/scf7180000084763:21450-21610:-	ACA1_237810	hypothetical protein	0.00779702	0.410888	0.40309098	1.51E-06
3h	ACA1_298630/clean/scf7180000082992:3177-3286:-	ACA1_298630	Vacuolar protein sorting 16, putative [Source:UniProtKB/TrEMBL;Acc:L8H081]	0.000163424	0.403217333	0.403053909	4.72E-10
3h	ACA1_161750/clean/scf7180000084726:19838-19940:-	ACA1_161750	hypothetical protein	0.014728196	0.417686	0.402957804	0.000172611
3h	ACA1_283220/clean/scf7180000084776:791864-791958:+	ACA1_283220	Calponin domain containing protein [Source:UniProtKB/TrEMBL;Acc:L8H7M9]	0.000233263	0.403103033	0.40286977	0.001095989
3h	ACA1_320550/clean/scf7180000084416:9471-9549:-	ACA1_320550	Proline iminopeptidase [Source:UniProtKB/TrEMBL;Acc:L8GWD8]	0.025735867	0.428596667	0.4028608	3.96E-10
3h	ACA1_097600/clean/scf7180000084645:1292967-1293075:+	ACA1_097600	Peroxidase [Source:UniProtKB/TrEMBL;Acc:L8GJT1]	0.000589036	0.4034415	0.402852464	3.36E-10
3h	ACA1_322160/clean/scf7180000084459:22615-22689:+	ACA1_322160	AB hydrolase-1 domain-containing protein [Source:UniProtKB/TrEMBL;Acc:L8GMT9]	0.009504287	0.412228	0.402723713	9.32E-09
3h	ACA1_183910/clean/scf7180000084745:519064-519161:+	ACA1_183910	Glycerate kinase subfamily protein [Source:UniProtKB/TrEMBL;Acc:L8H8F1]	0.128858733	0.531568667	0.402709933	0.001419842
3h	ACA1_068440/clean/scf7180000084603:195541-195605:+	ACA1_068440	F-box domain-containing protein [Source:UniProtKB/TrEMBL;Acc:L8HDJ5]	0.059573387	0.462148	0.402574613	0.000993688
3h	ACA1_387360/clean/scf7180000084733:52962-53050:-	ACA1_387360	hypothetical protein	0.031134893	0.4337041	0.402569207	0.004609596
3h	ACA1_296390/clean/scf7180000084777:1333866-1333968:+	ACA1_296390	hypothetical protein	0.013206103	0.415722	0.402515897	4.53E-07
3h	ACA1_275430/clean/scf7180000084774:17039-17149:-	ACA1_275430	unspecified product	0.0042735	0.406750333	0.402476833	6.42E-07
3h	ACA1_225960/clean/scf7180000084761:31320-31402:+	ACA1_225960	EF hand domain containing protein [Source:UniProtKB/TrEMBL;Acc:L8H8N3]	0.001937983	0.404294667	0.402356683	5.75E-08
3h	ACA1_175510/clean/scf7180000084741:974008-974097:+	ACA1_175510	Xylose isomerase [Source:UniProtKB/TrEMBL;Acc:L8HKE1]	0.016166133	0.418506667	0.402340533	2.94E-10
3h	ACA1_177470/clean/scf7180000084743:73730-73798:+	ACA1_177470	Nudix hydrolase domain-containing protein [Source:UniProtKB/TrEMBL;Acc:L8GSI6]	0.009548223	0.411860667	0.402312443	2.24E-07
3h	ACA1_125100/clean/scf7180000084691:30240-30322:-	ACA1_125100	hypothetical protein	0.011792497	0.414076333	0.402283837	3.88E-06
3h	ACA1_153200/clean/scf7180000084720:85325-85413:+	ACA1_153200	RBR-type E3 ubiquitin transferase [Source:UniProtKB/TrEMBL;Acc:L8HIB3]	0.000701017	0.402951233	0.402250217	1.27E-06
3h	ACA1_321060/clean/scf7180000084435:31332-31425:-	ACA1_321060	hypothetical protein	0.016386633	0.418576667	0.402190033	3.00E-07
3h	ACA1_090130/clean/scf7180000084639:443637-443700:+	ACA1_090130	hypothetical protein	0.0339046	0.436015	0.4021104	2.10E-05
3h	ACA1_199500/clean/scf7180000084753:248443-248513:+	ACA1_199500	1acylglycerophosphocholine O-acyltransferase 1, putative [Source:UniProtKB/TrEMBL;Acc:L8H4L3]	0.0107527	0.412802333	0.402049633	0.00226266
3h	ACA1_369220/clean/scf7180000084664:274857-274921:-	ACA1_369220	RBR-type E3 ubiquitin transferase [Source:UniProtKB/TrEMBL;Acc:L8GYY2]	0.015696633	0.417717667	0.402021033	1.50E-05
3h	ACA1_183740/clean/scf7180000084745:493681-493758:+	ACA1_183740	hypothetical protein	0.094440733	0.496295	0.401854267	0.001565421
3h	ACA1_127620/clean/scf7180000084696:36254-36326:+	ACA1_127620	Fe2OG dioxygenase domain-containing protein [Source:UniProtKB/TrEMBL;Acc:L8GGE8]	0.02153121	0.423380667	0.401849457	0.000991257
3h	ACA1_261980/clean/scf7180000084770:12567-12661:+	ACA1_261980	Defective in cullin neddylation protein [Source:UniProtKB/TrEMBL;Acc:L8H1R9]	0.041136833	0.442879333	0.4017425	4.48E-06
3h	ACA1_173170/clean/scf7180000084741:416679-416746:-	ACA1_173170	SNF2 family Nterminal domain containing protein [Source:UniProtKB/TrEMBL;Acc:L8HGM5]	0.003853567	0.405399333	0.401545767	6.25E-06
3h	ACA1_006180/clean/scf7180000083029:64793-64863:-	ACA1_006180	Multicopper oxidase, type 2 [Source:UniProtKB/TrEMBL;Acc:L8HG21]	0.027777767	0.429269667	0.4014919	0.001009875
3h	ACA1_315060/clean/scf7180000083887:26780-26897:-	ACA1_315060	AAA domain-containing protein [Source:UniProtKB/TrEMBL;Acc:L8GZM9]	0.000588795	0.401919033	0.401330238	3.81E-11
3h	ACA1_037730/clean/scf7180000084568:9598-9669:+	ACA1_037730	Transmembrane protein, putative [Source:UniProtKB/TrEMBL;Acc:L8GN13]	0	0.401325333	0.401325333	0.026805398
3h	ACA1_146570/clean/scf7180000084713:58210-58274:+	ACA1_146570	Ceramidase [Source:UniProtKB/TrEMBL;Acc:L8GV94]	0.006944433	0.408226333	0.4012819	9.32E-05
3h	ACA1_282270/clean/scf7180000084776:641318-641382:-	ACA1_282270	hypothetical protein	0	0.4012288	0.4012288	8.46E-06
3h	ACA1_062020/clean/scf7180000084599:1103824-1103933:+	ACA1_062020	Major vault protein alpha, putative [Source:UniProtKB/TrEMBL;Acc:L8GWT3]	0.0047619	0.405819333	0.401057433	1.26E-05
3h	ACA1_369090/clean/scf7180000084664:240241-240305:-	ACA1_369090	2hydroxyacyl-CoA lyase 1, putative [Source:UniProtKB/TrEMBL;Acc:L8GZ11]	0.003236247	0.404232333	0.400996087	2.75E-06
3h	ACA1_068440/clean/scf7180000084603:195393-195464:+	ACA1_068440	F-box domain-containing protein [Source:UniProtKB/TrEMBL;Acc:L8HDJ5]	0.099833	0.500672	0.400839	0.002478495
3h	ACA1_228930/clean/scf7180000084761:227992-228096:-	ACA1_228930	Vta1 domain-containing protein [Source:UniProtKB/TrEMBL;Acc:L8H8T2]	0.0040824	0.404842333	0.400759933	2.21E-09
3h	ACA1_163650/clean/scf7180000084729:64641-64736:+	ACA1_163650	hypothetical protein	0.041836995	0.442528	0.400691005	0.000449514
3h	ACA1_297530/clean/scf7180000082850:1266-1335:-	ACA1_297530	hypothetical protein	0	0.400625	0.400625	1.89E-08
3h	ACA1_368140/clean/scf7180000084664:66043-66118:-	ACA1_368140	Glutamine amidotransferase type-2 domain-containing protein [Source:UniProtKB/TrEMBL;Acc:L8H040]	0	0.400541	0.400541	0.000526851
3h	ACA1_269670/clean/scf7180000084770:1012991-1013062:-	ACA1_269670	Tetratricopeptide repeat domain containing protein [Source:UniProtKB/TrEMBL;Acc:L8H2T8]	0	0.400521333	0.400521333	2.27E-06
3h	ACA1_131530/clean/scf7180000084699:79466-79581:+	ACA1_131530	Nudix hydrolase domain-containing protein [Source:UniProtKB/TrEMBL;Acc:L8GM80]	0.025995787	0.426333	0.400337213	6.95E-05
3h	ACA1_284210/clean/scf7180000084776:891613-891897:-	ACA1_284210	Oxoglutarate carrier, putative [Source:UniProtKB/TrEMBL;Acc:L8H8V6]	0.018567207	0.418904333	0.400337127	1.20E-07
3h	ACA1_273770/clean/scf7180000084773:52999-53074:-	ACA1_273770	hypothetical protein	0.011479883	0.411779	0.400299117	1.96E-12
3h	ACA1_287810/clean/scf7180000084777:85064-85137:+	ACA1_287810	HemolysinIII related subfamily protein [Source:UniProtKB/TrEMBL;Acc:L8HIR3]	0.008855017	0.409137	0.400281983	3.20E-05
3h	ACA1_083640/clean/scf7180000084629:7550-7683:-	ACA1_083640	Elongation factor 1alpha [Source:UniProtKB/TrEMBL;Acc:L8GYH2]	0.003573482	0.403845	0.400271518	5.46E-09
3h	ACA1_131570/clean/scf7180000084699:89928-90029:-	ACA1_131570	Transmembrane proteins 14c protein [Source:UniProtKB/TrEMBL;Acc:L8H2Q4]	0.010972847	0.411210667	0.40023782	9.64E-09
3h	ACA1_061500/clean/scf7180000084599:943300-943400:+	ACA1_061500	Ras-GAP domain-containing protein [Source:UniProtKB/TrEMBL;Acc:L8GWM5]	0.000443853	0.4006351	0.400191247	3.69E-08
3h	ACA1_255670/clean/scf7180000084768:542882-543003:+	ACA1_255670	Aminotran_1_2 domain-containing protein [Source:UniProtKB/TrEMBL;Acc:L8HDH4]	0.009412903	0.409323333	0.39991043	8.26E-11
3h	ACA1_076260/clean/scf7180000084612:44735-44874:+	ACA1_076260	LanC lantibiotic synthetase [Source:UniProtKB/TrEMBL;Acc:L8GM15]	0.600324333	1	0.399675667	0.000200532
3h	ACA1_112830/clean/scf7180000084669:227776-227917:-	ACA1_112830	RFX-type winged-helix domain-containing protein [Source:UniProtKB/TrEMBL;Acc:L8H5H1]	0.013984917	0.413490333	0.399505417	0.000308348
3h	ACA1_112100/clean/scf7180000084669:109259-109345:+	ACA1_112100	BTB/POZ domain containing protein [Source:UniProtKB/TrEMBL;Acc:L8H3Y0]	0.020061633	0.419544667	0.399483033	4.52E-05
3h	ACA1_237270/clean/scf7180000084762:500332-500412:+	ACA1_237270	RNA 2'phosphotransferase, Tpt1/KptA domain containing protein [Source:UniProtKB/TrEMBL;Acc:L8H0V2]	0.01025197	0.409726333	0.399474363	1.94E-05
3h	ACA1_378170/clean/scf7180000084711:201115-201176:-	ACA1_378170	G_PROTEIN_RECEP_F1_2 domain-containing protein [Source:UniProtKB/TrEMBL;Acc:L8GRC2]	0	0.399472667	0.399472667	6.29E-07
3h	ACA1_163650/clean/scf7180000084729:63783-63891:+	ACA1_163650	hypothetical protein	0.001660563	0.401038167	0.399377604	7.66E-06
3h	ACA1_055410/clean/scf7180000084594:699015-699086:+	ACA1_055410	Protein kinase domain-containing protein [Source:UniProtKB/TrEMBL;Acc:L8H6J6]	0.008744943	0.407746	0.399001057	0.000100448
3h	ACA1_260050/clean/scf7180000084769:412181-412270:+	ACA1_260050	WD_REPEATS_REGION domain-containing protein [Source:UniProtKB/TrEMBL;Acc:L8GHJ4]	0.00600147	0.404738	0.39873653	8.26E-11
3h	ACA1_295300/clean/scf7180000084777:1097247-1097363:-	ACA1_295300	Calponin domain containing protein [Source:UniProtKB/TrEMBL;Acc:L8HJQ6]	0.0520327	0.450712667	0.398679967	1.50E-05
3h	ACA1_124920/clean/scf7180000084691:12892-13001:-	ACA1_124920	hypothetical protein	0.0095745	0.408052	0.3984775	2.10E-05
3h	ACA1_262400/clean/scf7180000084770:43369-43469:+	ACA1_262400	Probable tRNA N6-adenosine threonylcarbamoyltransferase [Source:UniProtKB/TrEMBL;Acc:L8H1V9]	0.016660167	0.415095667	0.3984355	1.95E-07
3h	ACA1_367310/clean/scf7180000084641:597334-597403:-	ACA1_367310	CCHC-type domain-containing protein [Source:UniProtKB/TrEMBL;Acc:L8GMZ2]	0.0299093	0.428223	0.3983137	0.031082221
3h	ACA1_235920/clean/scf7180000084762:385520-385599:-	ACA1_235920	RRM domain-containing protein [Source:UniProtKB/TrEMBL;Acc:L8H0M8]	0.01298278	0.411278	0.39829522	1.65E-07
3h	ACA1_245510/clean/scf7180000084763:498860-499095:+	ACA1_245510	SAM domain (Sterile alpha motif) domain containing protein [Source:UniProtKB/TrEMBL;Acc:L8GKU3]	0.048396967	0.446681333	0.398284367	0.00043576
3h	ACA1_236630/clean/scf7180000084762:462944-463028:-	ACA1_236630	PX domain-containing protein [Source:UniProtKB/TrEMBL;Acc:L8H1G0]	0.061723533	0.459964667	0.398241133	0.000547881
3h	ACA1_236630/clean/scf7180000084762:463130-463222:-	ACA1_236630	PX domain-containing protein [Source:UniProtKB/TrEMBL;Acc:L8H1G0]	0.012538933	0.410743	0.398204067	3.59E-05
3h	ACA1_282990/clean/scf7180000084776:759373-759441:+	ACA1_282990	hypothetical protein	0.015932833	0.414104667	0.398171833	2.85E-05
3h	ACA1_192960/clean/scf7180000084749:172383-172480:-	ACA1_192960	Multiple Ankyrin repeat domain containing protein [Source:UniProtKB/TrEMBL;Acc:L8GPD9]	0.000382703	0.398317667	0.397934963	2.36E-08
3h	ACA1_275430/clean/scf7180000084774:16807-16903:-	ACA1_275430	unspecified product	0.000467133	0.398358333	0.3978912	5.40E-08
3h	ACA1_158500/clean/scf7180000084724:239620-239729:+	ACA1_158500	Nudix hydrolase domain-containing protein [Source:UniProtKB/TrEMBL;Acc:L8HA53]	0.003640033	0.401510333	0.3978703	2.20E-06
3h	ACA1_164910/clean/scf7180000084729:310627-310734:-	ACA1_164910	hypothetical protein	0.00041308	0.398110167	0.397697086	1.23E-10
3h	ACA1_174040/clean/scf7180000084741:582067-582231:-	ACA1_174040	Protein kinase domain-containing protein [Source:UniProtKB/TrEMBL;Acc:L8HK27]	0.0865244	0.484167667	0.397643267	0.00016764
3h	ACA1_171960/clean/scf7180000084741:209672-209738:-	ACA1_171960	F-box domain-containing protein [Source:UniProtKB/TrEMBL;Acc:L8HHE3]	0.00319706	0.400588333	0.397391273	1.53E-06
3h	ACA1_175420/clean/scf7180000084741:951379-951465:+	ACA1_175420	hypothetical protein	0.038850033	0.436092667	0.397242633	0.001710605
3h	ACA1_382920/clean/scf7180000084721:120592-120664:-	ACA1_382920	Ras subfamily protein [Source:UniProtKB/TrEMBL;Acc:L8GV31]	0.026934	0.424147333	0.397213333	0.000224366

3h	ACA1_234780/clean/scf7180000084762:315778-315882:-	ACA1_234780	hypothetical protein	0.01197222	0.4089668	0.39699458	1.32E-06
3h	ACA1_318540/clean/scf7180000084158:19585-19665:+	ACA1_318540	SPX domain containing protein [Source:UniProtKB/TrEMBL;Acc:L8GH73]	0.013990017	0.410825333	0.396835317	3.58E-07
3h	ACA1_206620/clean/scf7180000084757:25192-25300:+	ACA1_206620	MCM domain-containing protein [Source:UniProtKB/TrEMBL;Acc:L8GY26]	0	0.396760333	0.396760333	2.47E-07
3h	ACA1_243850/clean/scf7180000084763:360729-360801:+	ACA1_243850	Ras-GAP domain-containing protein [Source:UniProtKB/TrEMBL;Acc:L8GJZ5]	0.032653067	0.429331	0.396677933	0.003748519
3h	ACA1_109740/clean/scf7180000084663:16277-16353:-	ACA1_109740	Myblike DNA-binding domain containing protein [Source:UniProtKB/TrEMBL;Acc:L8HJY9]	0	0.396568	0.396568	1.12E-07
3h	ACA1_026960/clean/scf7180000084464:4996-5073:-	ACA1_026960	FtsX domain-containing protein [Source:UniProtKB/TrEMBL;Acc:L8GHC2]	0	0.396384333	0.396384333	8.00E-10
3h	ACA1_260270/clean/scf7180000084769:469224-469318:+	ACA1_260270	Proteasome subunit alpha type [Source:UniProtKB/TrEMBL;Acc:L8GG17]	0.006658577	0.402914667	0.39625609	5.24E-13
3h	ACA1_111100/clean/scf7180000084666:33325-33426:+	ACA1_111100	hypothetical protein	0.003333333	0.399390333	0.396057	3.70E-07
3h	ACA1_095270/clean/scf7180000084645:825557-825678:-	ACA1_095270	Thioredoxin-like_fold domain-containing protein [Source:UniProtKB/TrEMBL;Acc:L8GIR9]	0.003401388	0.399381667	0.395980279	7.85E-08
3h	ACA1_009890/clean/scf7180000083468:804-909:-	ACA1_009890	TPR_REGION domain-containing protein [Source:UniProtKB/TrEMBL;Acc:L8GDE6]	0.005567663	0.40146	0.395892337	1.25E-08
3h	ACA1_182120/clean/scf7180000084745:133465-133567:+	ACA1_182120	WD repeat domain 5B, putative [Source:UniProtKB/TrEMBL;Acc:L8HA46]	0.008449153	0.404329	0.395879847	2.06E-08
3h	ACA1_216300/clean/scf7180000084758:462686-462827:+	ACA1_216300	Ras family protein [Source:UniProtKB/TrEMBL;Acc:L8GR60]	0.0366081	0.432434	0.3958259	0.000749189
3h	ACA1_129970/clean/scf7180000084698:60113-60219:+	ACA1_129970	Cation transport domain containing protein [Source:UniProtKB/TrEMBL;Acc:L8GPJ3]	0.102455967	0.498239	0.395783033	0.000260334
3h	ACA1_020880/clean/scf7180000084095:30828-30926:+	ACA1_020880	Protozoan/cyanobacterial globin family subfamily protein [Source:UniProtKB/TrEMBL;Acc:L8GU16]	0.018271067	0.414008333	0.395737267	3.68E-07
3h	ACA1_237770/clean/scf7180000084763:10256-10371:+	ACA1_237770	PH domain-containing protein [Source:UniProtKB/TrEMBL;Acc:L8GKB5]	0.007607203	0.403275667	0.395668463	2.80E-05
3h	ACA1_331370/clean/scf7180000084575:18605-18688:-	ACA1_331370	Succinylglutamate desuccinylase/aspartoacylase subfamily protein [Source:UniProtKB/TrEMBL;Acc:L8GSG2]	0.008671167	0.404326333	0.395655167	5.26E-06
3h	ACA1_331800/clean/scf7180000084578:34207-34302:-	ACA1_331800	hypothetical protein	0.042764867	0.438363667	0.3955988	0.000557269
3h	ACA1_293710/clean/scf7180000084777:964803-964913:+	ACA1_293710	F-box domain-containing protein [Source:UniProtKB/TrEMBL;Acc:L8HJM5]	0.033511267	0.429035333	0.395524067	1.55E-06
3h	ACA1_274410/clean/scf7180000084773:103464-103584:+	ACA1_274410	Trafficking protein particle complex subunit [Source:UniProtKB/TrEMBL;Acc:L8GFV3]	0.015197683	0.410610333	0.39541265	1.58E-08
3h	ACA1_389130/clean/scf7180000084733:208785-208858:+	ACA1_389130	BTB/POZ domain containing protein [Source:UniProtKB/TrEMBL;Acc:L8GDV1]	0.01027044	0.405464467	0.395194027	2.40E-08
3h	ACA1_059470/clean/scf7180000084599:452241-452329:+	ACA1_059470	G-patch domain-containing protein [Source:UniProtKB/TrEMBL;Acc:L8GW60]	0.013164123	0.408338333	0.39517421	1.97E-05
3h	ACA1_159160/clean/scf7180000084724:349201-349286:-	ACA1_159160	hypothetical protein	0.037594	0.432584333	0.394990333	0.000391324
3h	ACA1_152960/clean/scf7180000084720:41370-41458:+	ACA1_152960	Malate dehydrogenase [Source:UniProtKB/TrEMBL;Acc:L8HFT9]	0.001448744	0.396344667	0.394895923	4.47E-19
3h	ACA1_352370/clean/scf7180000084704:2699-2777:+	ACA1_352370	Oxidoreductase, short chain dehydrogenase/reductase superfamily protein [Source:UniProtKB/TrEMBL;Acc:L8GTQ2]	0.048487873	0.443374	0.394886127	0.000447048
3h	ACA1_136770/clean/scf7180000084703:5790-5874:+	ACA1_136770	hypothetical protein	0.2697	0.664583	0.394883	0.00966763
3h	ACA1_158170/clean/scf7180000084724:201936-202068:-	ACA1_158170	Pescadillo homolog [Source:UniProtKB/TrEMBL;Acc:L8HBG6]	0.014445283	0.409297667	0.394852383	1.05E-06
3h	ACA1_081440/clean/scf7180000084618:110361-110456:-	ACA1_081440	Alg14 family oligosaccharide biosynthesis protein [Source:UniProtKB/TrEMBL;Acc:L8HD51]	0.03264769	0.427474	0.39482631	1.04E-07
3h	ACA1_341080/clean/scf7180000084620:46699-46785:+	ACA1_341080	RING-type domain-containing protein [Source:UniProtKB/TrEMBL;Acc:L8GS99]	0.032221667	0.427035333	0.394813667	5.90E-06
3h	ACA1_066880/clean/scf7180000084600:69990-70100:+	ACA1_066880	hypothetical protein	0.019893797	0.414474	0.394580203	5.53E-05
3h	ACA1_034160/clean/scf7180000084561:37460-37550:+	ACA1_034160	FERM domain-containing protein [Source:UniProtKB/TrEMBL;Acc:L8GV43]	0.019024733	0.413474667	0.394449933	1.05E-06
3h	ACA1_285650/clean/scf7180000084776:1019584-1019664:-	ACA1_285650	G-patch domain-containing protein [Source:UniProtKB/TrEMBL;Acc:L8H9W2]	0.016094733	0.410535333	0.3944406	0.000104486
3h	ACA1_398880/clean/scf7180000084759:238990-239064:-	ACA1_398880	RNA_ligase domain-containing protein [Source:UniProtKB/TrEMBL;Acc:L8HCD5]	0.015503867	0.409887333	0.394383467	0.000614046
3h	ACA1_035310/clean/scf7180000084564:17774-17825:+	ACA1_035310	AMP-binding domain-containing protein [Source:UniProtKB/TrEMBL;Acc:L8HCU7]	0.0252644	0.419593667	0.394329267	0.000325711
3h	ACA1_252450/clean/scf7180000084768:153620-153701:+	ACA1_252450	L1P family protein [Source:UniProtKB/TrEMBL;Acc:L8HAS4]	0.009093334	0.403053333	0.393959999	5.20E-07
3h	ACA1_214850/clean/scf7180000084758:245772-245864:+	ACA1_214850	LIM zinc-binding domain-containing protein [Source:UniProtKB/TrEMBL;Acc:L8GSG1]	0.00944003	0.403399667	0.393959637	7.86E-08
3h	ACA1_219050/clean/scf7180000084758:705438-705531:-	ACA1_219050	hypothetical protein	0.024185203	0.418100667	0.393915463	3.95E-07
3h	ACA1_144000/clean/scf7180000084709:125968-126052:+	ACA1_144000	RhoGEF domain containing protein [Source:UniProtKB/TrEMBL;Acc:L8HFT1]	0.04162129	0.435465333	0.393844043	0.000216957
3h	ACA1_052600/clean/scf7180000084594:121647-121712:+	ACA1_052600	hypothetical protein	0.059803367	0.453615333	0.393811967	0.002032018
3h	ACA1_333220/clean/scf7180000084581:20760-20852:+	ACA1_333220	hypothetical protein	0.00113717	0.394881667	0.393744497	1.48E-06
3h	ACA1_298630/clean/scf7180000082992:2726-2837:-	ACA1_298630	Vacuolar protein sorting 16, putative [Source:UniProtKB/TrEMBL;Acc:L8H081]	0.016624233	0.410207	0.393582767	6.10E-07
3h	ACA1_042850/clean/scf7180000084573:106349-106462:+	ACA1_042850	hypothetical protein	0	0.393459667	0.393459667	2.16E-07
3h	ACA1_306840/clean/scf7180000083668:19565-19636:+	ACA1_306840	RTP1_C1 domain-containing protein [Source:UniProtKB/TrEMBL;Acc:L8GQF6]	0.008456287	0.4018113	0.393355013	0.000922637
3h	ACA1_396750/clean/scf7180000084759:115266-115437:+	ACA1_396750	Glutaredoxin, putative [Source:UniProtKB/TrEMBL;Acc:L8HBE6]	0.097120233	0.490422667	0.393302433	0.002673517
3h	ACA1_139400/clean/scf7180000084705:73757-73922:+	ACA1_139400	WD domain, Gbeta repeat-containing protein [Source:UniProtKB/TrEMBL;Acc:L8GNA0]	0.025677533	0.418856	0.393178467	5.01E-05
3h	ACA1_075300/clean/scf7180000084608:172722-172821:+	ACA1_075300	Sec1 family domain containing protein [Source:UniProtKB/TrEMBL;Acc:L8HHN4]	0.0468469	0.439973667	0.393126767	0.000151194
3h	ACA1_069260/clean/scf7180000084603:391656-391730:-	ACA1_069260	hypothetical protein	0.395760667	0.788779	0.393018333	0.027140018
3h	ACA1_264270/clean/scf7180000084770:227768-227864:-	ACA1_264270	Oxidoreductase, short chain dehydrogenase/reductase family protein [Source:UniProtKB/TrEMBL;Acc:L8H474]	0.007379633	0.400323667	0.392944033	4.54E-06
3h	ACA1_115860/clean/scf7180000084669:689277-689367:-	ACA1_115860	NOT2 / NOT3 / NOT5 family protein [Source:UniProtKB/TrEMBL;Acc:L8H4R3]	0.0167286	0.409641	0.3929124	3.91E-07
3h	ACA1_188380/clean/scf7180000084747:168440-168533:-	ACA1_188380	Fbox domain containing protein [Source:UniProtKB/TrEMBL;Acc:L8GSC5]	0.014562257	0.4074188	0.392856543	6.01E-07
3h	ACA1_216440/clean/scf7180000084758:507618-507870:+	ACA1_216440	PPM-type phosphatase domain-containing protein [Source:UniProtKB/TrEMBL;Acc:L8GQI6]	0.050591967	0.443297	0.392705033	9.36E-06
3h	ACA1_140770/clean/scf7180000084707:117409-117499:+	ACA1_140770	ATPase, AAA domain containing protein [Source:UniProtKB/TrEMBL;Acc:L8GJ22]	0.098435733	0.491085667	0.392649933	0.003830853
3h	ACA1_097190/clean/scf7180000084645:1182101-1182239:-	ACA1_097190	JmjC domain-containing protein [Source:UniProtKB/TrEMBL;Acc:L8GIZ2]	0.016437767	0.409021333	0.392583567	6.50E-05
3h	ACA1_200560/clean/scf7180000084753:448510-448602:-	ACA1_200560	Calcium binding atopyrelated autoantigen 1, putative [Source:UniProtKB/TrEMBL;Acc:L8H3D2]	0.012988893	0.405500333	0.39251144	4.37E-07
3h	ACA1_200110/clean/scf7180000084753:394258-394423:+	ACA1_200110	Peptidase S53 domain-containing protein [Source:UniProtKB/TrEMBL;Acc:L8H2I7]	0.0215311	0.414017	0.3924859	3.59E-05
3h	ACA1_107160/clean/scf7180000084660:161296-161454:+	ACA1_107160	hypothetical protein	0.001920926	0.3942558	0.392334874	1.08E-08
3h	ACA1_240180/clean/scf7180000084763:203096-203202:+	ACA1_240180	WD_REPEATS_REGION domain-containing protein [Source:UniProtKB/TrEMBL;Acc:L8GM97]	0.031116467	0.423386333	0.392269867	0.00020913
3h	ACA1_178230/clean/scf7180000084743:183875-183989:+	ACA1_178230	Transducin family protein [Source:UniProtKB/TrEMBL;Acc:L8GSK8]	0.067359033	0.459498333	0.3921393	2.39E-05
3h	ACA1_329650/clean/scf7180000084562:38124-38216:-	ACA1_329650	hypothetical protein	0.00366529	0.395793	0.39212771	1.92E-09
3h	ACA1_115860/clean/scf7180000084669:690605-690703:-	ACA1_115860	NOT2 / NOT3 / NOT5 family protein [Source:UniProtKB/TrEMBL;Acc:L8H4R3]	0	0.392063667	0.392063667	3.49E-08
3h	ACA1_175520/clean/scf7180000084741:984104-984175:-	ACA1_175520	PH domain-containing protein [Source:UniProtKB/TrEMBL;Acc:L8HI32]	0	0.391995	0.391995	3.57E-05
3h	ACA1_051680/clean/scf7180000084592:48175-48250:+	ACA1_051680	Ras subfamily protein [Source:UniProtKB/TrEMBL;Acc:L8GPK3]	0.015873	0.407836667	0.391963667	0.000272596
3h	ACA1_140780/clean/scf7180000084707:119537-119623:+	ACA1_140780	Zinc carboxypeptidase superfamily protein [Source:UniProtKB/TrEMBL;Acc:L8GGS5]	0.002851257	0.394740333	0.391889077	7.79E-08
3h	ACA1_279550/clean/scf7180000084776:412516-412590:-	ACA1_279550	CBS domain containing protein [Source:UniProtKB/TrEMBL;Acc:L8H8C1]	0.001916508	0.393791867	0.391875359	1.39E-06
3h	ACA1_088860/clean/scf7180000084639:249725-249829:+	ACA1_088860	Leucine rich repeat domain containing protein [Source:UniProtKB/TrEMBL;Acc:L8GUH8]	0	0.391834667	0.391834667	1.79E-06
3h	ACA1_204500/clean/scf7180000084754:293440-293535:+	ACA1_204500	hypothetical protein	0.002875367	0.394327	0.391451633	3.39E-07
3h	ACA1_142640/clean/scf7180000084708:210198-210335:-	ACA1_142640	unspecified product	0.212146667	0.603516	0.391369333	0.011818625
3h	ACA1_323110/clean/scf7180000084467:4257-4360:+	ACA1_323110	hypothetical protein	0	0.391361533	0.391361533	4.03E-06
3h	ACA1_188710/clean/scf7180000084747:195856-195965:+	ACA1_188710	SNARE associated Golgi protein [Source:UniProtKB/TrEMBL;Acc:L8GTI0]	0.030787	0.421991667	0.391204667	4.36E-06
3h	ACA1_077120/clean/scf7180000084612:240326-240404:-	ACA1_077120	DH domain-containing protein [Source:UniProtKB/TrEMBL;Acc:L8GM77]	0.000329613	0.391514667	0.391185053	5.58E-09
3h	ACA1_074070/clean/scf7180000084605:15812-15919:-	ACA1_074070	PAS domain-containing protein [Source:UniProtKB/TrEMBL;Acc:L8HKA1]	0.014973893	0.406035333	0.39106144	2.00E-05
3h	ACA1_061500/clean/scf7180000084599:943535-943652:+	ACA1_061500	Ras-GAP domain-containing protein [Source:UniProtKB/TrEMBL;Acc:L8GWM5]	0.004308737	0.395326733	0.391017997	1.79E-06
3h	ACA1_025980/clean/scf7180000084456:1823-1924:-	ACA1_025980	hypothetical protein	0.005534887	0.396419667	0.39088478	1.06E-06
3h	ACA1_393010/clean/scf7180000084756:26923-27009:+	ACA1_393010	AA_TRNA_LIGASE_II domain-containing protein [Source:UniProtKB/TrEMBL;Acc:L8H0A5]	0.014595867	0.405236333	0.390640467	9.29E-08
3h	ACA1_399930/clean/scf7180000084775:70212-70322:+	ACA1_399930	Ras subfamily protein [Source:UniProtKB/TrEMBL;Acc:L8GGR5]	0.030003167	0.420518067	0.3905149	0.007086687
3h	ACA1_155100/clean/scf7180000084722:105294-105350:+	ACA1_155100	EF-hand domain-containing protein [Source:UniProtKB/TrEMBL;Acc:L8GZ97]	0.003805857	0.394303	0.390497143	4.37E-13
3h	ACA1_307250/clean/scf7180000083680:1829-1943:-	ACA1_307250	Mss4 protein [Source:UniProtKB/TrEMBL;Acc:L8H520]	0.007501877	0.39786	0.390358123	7.95E-08
3h	ACA1_369720/clean/scf7180000084664:367484-367579:-	ACA1_369720	Ras subfamily protein [Source:UniProtKB/TrEMBL;Acc:L8GY91]	0.01929392	0.409539333	0.390245413	0.00168121
3h	ACA1_278320/clean/scf7180000084776:196179-196318:+	ACA1_278320	N-acetyltransferase domain-containing protein [Source:UniProtKB/TrEMBL;Acc:L8H8U2]	0.102862533	0.493022667	0.390160133	0.000600038
3h	ACA1_193130/clean/scf7180000084749:193543-193641:-	ACA1_193130	Metallophos domain-containing protein [Source:UniProtKB/TrEMBL;Acc:L8GN68]	0.225571	0.615711333	0.390140333	0.006184093
3h	ACA1_145850/clean/scf7180000084710:168067-168142:-	ACA1_145850	O-methyltransferase, putative [Source:UniProtKB/TrEMBL;Acc:L8GFG9]	0.008979857	0.398918667	0.38993881	5.49E-07
3h	ACA1_034600/clean/scf7180000084563:85402-85477:+	ACA1_034600	hypothetical protein	0.0486031	0.438423667	0.389820567	0.000268875
3h	ACA1_198970/clean/scf7180000084753:180606-180664:-	ACA1_198970	Viral Atype inclusion protein, putative [Source:UniProtKB/TrEMBL;Acc:L8H376]	0	0.389722	0.389722	1.20E-06
3h	ACA1_176030/clean/scf7180000084741:1109151-1109222:-	ACA1_176030	ATPase, AAA domain containing protein [Source:UniProtKB/TrEMBL;Acc:L8HI90]	0.000711237	0.390336767	0.38962553	0.000278987
3h	ACA1_379850/clean/scf7180000084712:15456-15570:-	ACA1_379850	Isochorismatase domain containing protein 2, mitochondrial, putative [Source:UniProtKB/TrEMBL;Acc:L8GN12]	0.000329741	0.3899101	0.389580359	1.16E-10
3h	ACA1_068360/clean/scf7180000084603:181269-181348:+	ACA1_068360	Dual specificity protein kinase [Source:UniProtKB/TrEMBL;Acc:L8HDE4]	0.005040537	0.394601667	0.38956113	8.57E-10
3h	ACA1_081100/clean/scf7180000084618:90457-90538:-	ACA1_081100	Prephenate dehydratase [Source:UniProtKB/TrEMBL;Acc:L8HE69]	0.007857847	0.397353667	0.38949582	1.81E-06
3h	ACA1_253970/clean/scf7180000084768:354638-354691:-	ACA1_253970	EGFlike domain containing protein [Source:UniProtKB/TrEMBL;Acc:L8HAZ9]	0.44604	0.83532	0.38928	0.002315031
3h	ACA1_176030/clean/scf7180000084741:1110789-1110905:-	ACA1_176030	ATPase, AAA domain containing protein [Source:UniProtKB/TrEMBL;Acc:L8HI90]	0.009009	0.397691167	0.388682167	0.00025905
3h	ACA1_329850/clean/scf7180000084566:11472-11581:-	ACA1_329850	hypothetical protein	0.053835267	0.442506	0.388670733	0.000467026
3h	ACA1_173010/clean/scf7180000084741:386169-386233:-	ACA1_173010	Vtype ATPase, A subunit [Source:UniProtKB/TrEMBL;Acc:L8HGL3]	0	0.388412	0.388412	0.000161508
3h	ACA1_182300/clean/scf7180000084745:176763-176837:+	ACA1_182300	hypothetical protein	0.004083441	0.392447	0.388363559	5.82E-07
3h	ACA1_285540/clean/scf7180000084776:995351-995423:-	ACA1_285540	GPS domain-containing protein [Source:UniProtKB/TrEMBL;Acc:L8H7T4]	0	0.3882157	0.3882157	6.69E-05
3h	ACA1_107610/clean/scf7180000084660:229994-230110:+	ACA1_107610	Aminotransferase, class III superfamily protein [Source:UniProtKB/TrEMBL;Acc:L8GMP2]	0.009949133	0.3980572	0.388108067	0.000720372
3h	ACA1_392550/clean/scf7180000084740:292133-292241:+	ACA1_392550	Helicase C-terminal domain-containing protein [Source:UniProtKB/TrEMBL;Acc:L8GRN9]	0.30768	0.695621333	0.387941333	0.009574839
3h	ACA1_219420/clean/scf7180000084758:732284-732384:+	ACA1_219420	Fbox domain containing protein [Source:UniProtKB/TrEMBL;Acc:L8GQP8]	0.02968242	0.417454333	0.387771913	8.06E-05
3h	ACA1_126940/clean/scf7180000084696:5030-5098:+	ACA1_126940	hypothetical protein	0.011139667	0.3989	0.387760333	2.74E-06
3h	ACA1_296250/clean/scf7180000084777:1306540-1306676:+	ACA1_296250	Rasrelated protein Rab-32, putative [Source:UniProtKB/TrEMBL;Acc:L8HJZ6]	0.007976193	0.395562667	0.387586473	1.02E-05
3h	ACA1_154230/clean/scf7180000084722:28525-28653:+	ACA1_154230	hypothetical protein	0.010595653	0.398129333	0.38753368	6.21E-10
3h	ACA1_224860/clean/scf7180000084760:257532-257630:+	ACA1_224860	Myosin head (Motor domain) domain containing protein [Source:UniProtKB/TrEMBL;Acc:L8GSY9]	0	0.387508667	0.387508667	0.004200664
3h	ACA1_244760/clean/scf7180000084763:411555-411655:-	ACA1_244760	CitMHS domain-containing protein [Source:UniProtKB/TrEMBL;Acc:L8GMI1]	0.002912683	0.390325	0.387412317	1.44E-11
3h	ACA1_289690/clean/scf7180000084777:443645-443723:-	ACA1_289690	hypothetical protein	0.001233193	0.388534667	0.387301473	4.61E-07
3h	ACA1_012020/clean/scf7180000083652:3504-3611:-	ACA1_012020	hypothetical protein	0.001960187	0.389211	0.387250813	1.03E-07
3h	ACA1_152950/clean/scf7180000084720:40493-40555:-	ACA1_152950	UBIQUITIN_CONJUGAT_2 domain-containing protein [Source:UniProtKB/TrEMBL;Acc:L8HI96]	0.0112317	0.398060333	0.386828633	3.36E-11
3h	ACA1_054230/clean/scf7180000084594:437846-437925:+	ACA1_054230	CDPalcohol phosphatidyltransferase superfamily protein [Source:UniProtKB/TrEMBL;Acc:L8H630]	0.005475903	0.392196667	0.386720763	5.07E-08
3h	ACA1_068510/clean/scf7180000084603:219358-219482:-	ACA1_068510	Tyrosine phosphatase [Source:UniProtKB/TrEMBL;Acc:L8HDF9]	0.056732667	0.443402333	0.386669667	4.33E-05
3h	ACA1_145850/clean/scf7180000084710:167925-168007:-	ACA1_145850	O-methyltransferase, putative [Source:UniProtKB/TrEMBL;Acc:L8GFG9]	0.000606533	0.387094	0.386487467	9.28E-09
3h	ACA1_189450/clean/scf7180000084748:38429-38512:-	ACA1_189450	Chloride channel protein [Source:UniProtKB/TrEMBL;Acc:L8GEJ2]	0.0107744	0.397059333	0.386284933	2.55E-06
3h	ACA1_060490/clean/scf7180000084599:693948-694041:-	ACA1_060490	Flavinbinding monooxygenase-like subfamily protein [Source:UniProtKB/TrEMBL;Acc:L8GWK2]	0.084277767	0.470478	0.386200233	0.000227952
3h	ACA1_348970/clean/scf7180000084689:22092-22185:+	ACA1_348970	UBA domain-containing protein [Source:UniProtKB/TrEMBL;Acc:L8GWL7]	0.02042573	0.406548133	0.386122403	9.38E-05
3h	ACA1_299380/clean/scf7180000083027:14025-14093:+	ACA1_299380	AAA_11 domain-containing protein [Source:UniProtKB/TrEMBL;Acc:L8GS61]	0.0152952	0.401342667	0.386047467	1.09E-08
3h	ACA1_031780/clean/scf7180000084523:804-904:-	ACA1_031780	Lactamase_B domain-containing protein [Source:UniProtKB/TrEMBL;Acc:L8H103]	0.0234906	0.4095373	0.3860467	0.001054077
3h	ACA1_218390/clean/scf7180000084758:650712-650826:-	ACA1_218390	Glycosyl hydrolase family 20, catalytic domain containing protein [Source:UniProtKB/TrEMBL;Acc:L8GQD2]	0.006020897	0.392009	0.385988103	2.84E-06
3h	ACA1_025540/clean/scf7180000084448:7399-7495:+	ACA1_025540	hypothetical protein	0.005836897	0.391673	0.385836103	2.14E-05
3h	ACA1_144300/clean/scf7180000084709:198671-198801:-	ACA1_144300	RRM domain-containing protein [Source:UniProtKB/TrEMBL;Acc:L8HEU9]	0.003025173	0.388799333	0.38577416	3.50E-08
3h	ACA1_112820/clean/scf7180000084669:223584-223643:-	ACA1_112820	PeptidyltRNA hydrolase [Source:UniProtKB/TrEMBL;Acc:L8H433]	0.020748733	0.406454667	0.385705933	9.65E-09
3h	ACA1_288440/clean/scf7180000084777:206799-206860:-	ACA1_288440	DH domain-containing protein [Source:UniProtKB/TrEMBL;Acc:L8HKE3]	0.060444167	0.446138	0.385693833	0.001558392
3h	ACA1_240070/clean/scf7180000084763:196311-196389:-	ACA1_240070	Carboxylic ester hydrolase [Source:UniProtKB/TrEMBL;Acc:L8GJU3]	0.001250553	0.386835	0.385584447	1.91E-08
3h	ACA1_234110/clean/scf7180000084762:203365-203463:+	ACA1_234110	Sulfurtransferase [Source:UniProtKB/TrEMBL;Acc:L8H2J9]	0.027228333	0.412763667	0.385535333	5.13E-09
3h	ACA1_240060/clean/scf7180000084763:191820-191899:-	ACA1_240060	COesterase domain-containing protein [Source:UniProtKB/TrEMBL;Acc:L8GKI1]	0.003019383	0.388528667	0.385509283	7.37E-07
3h	ACA1_109780/clean/scf7180000084663:24917-25076:-	ACA1_109780	AAA domain-containing protein [Source:UniProtKB/TrEMBL;Acc:L8HIF9]	0.0307941	0.416262667	0.385468567	5.41E-06
3h	ACA1_061140/clean/scf7180000084599:833415-833692:-	ACA1_061140	WD_REPEATS_REGION domain-containing protein [Source:UniProtKB/TrEMBL;Acc:L8GZ38]	0.0174594	0.402916667	0.385457267	1.47E-05
3h	ACA1_048880/clean/scf7180000084583:10471-10596:+	ACA1_048880	LIM zinc-binding domain-containing protein [Source:UniProtKB/TrEMBL;Acc:L8HKD0]	0.139518	0.524795667	0.385277667	0.001865716
3h	ACA1_027160/clean/scf7180000084465:2956-3045:+	ACA1_027160	SSD domain-containing protein [Source:UniProtKB/TrEMBL;Acc:L8GH18]	0.000755613	0.3859531	0.385197487	1.89E-07
3h	ACA1_142140/clean/scf7180000084708:94197-94282:+	ACA1_142140	unspecified product	0.007631878	0.392828333	0.385196456	3.40E-06
3h	ACA1_166080/clean/scf7180000084732:114580-114659:+	ACA1_166080	hypothetical protein	0.000673674	0.385849	0.385175326	3.64E-09
3h	ACA1_184690/clean/scf7180000084746:39404-39481:-	ACA1_184690	Growth hormoneinducible transmembrane protein, putative [Source:UniProtKB/TrEMBL;Acc:L8H763]	0.004691143	0.389858333	0.38516719	2.10E-07
3h	ACA1_259490/clean/scf7180000084769:353911-354003:+	ACA1_259490	TPR_REGION domain-containing protein [Source:UniProtKB/TrEMBL;Acc:L8GEZ4]	0.0195545	0.404672667	0.385118167	1.35E-06
3h	ACA1_173140/clean/scf7180000084741:410239-410321:+	ACA1_173140	Deacetylase sirtuin-type domain-containing protein [Source:UniProtKB/TrEMBL;Acc:L8HJX9]	0.027969	0.412705667	0.384736667	1.15E-05
3h	ACA1_031270/clean/scf7180000084506:13798-13878:+	ACA1_031270	hypothetical protein	0.020164753	0.404815667	0.384650913	5.90E-06
3h	ACA1_019960/clean/scf7180000084033:1032-1099:+	ACA1_019960	SWIRM domain containing protein [Source:UniProtKB/TrEMBL;Acc:L8GUS1]	0.0077127	0.392264467	0.384551767	5.32E-07
3h	ACA1_375990/clean/scf7180000084685:22859-22941:-	ACA1_375990	AcetylCoA acetyltransferase, mitochondrial, putative [Source:UniProtKB/TrEMBL;Acc:L8HFY3]	0.001588813	0.385899	0.384310187	1.43E-06
3h	ACA1_266760/clean/scf7180000084770:707055-707160:+	ACA1_266760	hypothetical protein	0.008617243	0.392867667	0.384250423	1.53E-06
3h	ACA1_366940/clean/scf7180000084641:520029-520114:-	ACA1_366940	BTB/POZ domain containing protein [Source:UniProtKB/TrEMBL;Acc:L8GPM1]	0	0.384192	0.384192	4.80E-06
3h	ACA1_038180/clean/scf7180000084568:90704-90794:+	ACA1_038180	FHA domain containing protein [Source:UniProtKB/TrEMBL;Acc:L8GLQ3]	0.017730933	0.401892	0.384161067	6.57E-06
3h	ACA1_066150/clean/scf7180000084599:2010567-2010667:+	ACA1_066150	Myosin head (Motor domain) domain containing protein [Source:UniProtKB/TrEMBL;Acc:L8GX00]	0.03663	0.420630667	0.384000667	0.006253264
3h	ACA1_279570/clean/scf7180000084776:416051-416118:+	ACA1_279570	Elp3 domain-containing protein [Source:UniProtKB/TrEMBL;Acc:L8H759]	0.193339	0.577307	0.383968	0.001758363
3h	ACA1_063800/clean/scf7180000084599:1506266-1506367:-	ACA1_063800	B30.2/SPRY domain-containing protein [Source:UniProtKB/TrEMBL;Acc:L8GZV1]	0.024888833	0.408795667	0.383906833	2.11E-05
3h	ACA1_310500/clean/scf7180000083773:11761-11822:+	ACA1_310500	hypothetical protein	0	0.383894833	0.383894833	6.62E-08

3h	ACA1_055570/clean/scf7180000084594:731044-731168:-	ACA1_055570	hypothetical protein	0.0042735	0.388137667	0.383864167	2.41E-05
3h	ACA1_107530/clean/scf7180000084660:215648-215770:-	ACA1_107530	UBP-type domain-containing protein [Source:UniProtKB/TrEMBL;Acc:L8GMW8]	0.0231466	0.406912	0.3837654	2.78E-07
3h	ACA1_057940/clean/scf7180000084599:214671-214751:+	ACA1_057940	Alpha-mannosidase [Source:UniProtKB/TrEMBL;Acc:L8GX61]	0.003975696	0.387673333	0.383697638	1.11E-07
3h	ACA1_125990/clean/scf7180000084693:16902-17000:-	ACA1_125990	hypothetical protein	0.022857167	0.406552667	0.3836955	2.60E-06
3h	ACA1_192990/clean/scf7180000084749:176784-176908:-	ACA1_192990	PH domain-containing protein [Source:UniProtKB/TrEMBL;Acc:L8GQU9]	0.092125067	0.475818333	0.383693267	0.00021435
3h	ACA1_229530/clean/scf7180000084761:269073-269175:+	ACA1_229530	Diacylglycerol kinase [Source:UniProtKB/TrEMBL;Acc:L8H8Z8]	0.000418823	0.384053333	0.38363451	1.49E-09
3h	ACA1_289740/clean/scf7180000084777:453935-454019:+	ACA1_289740	Rho-GAP domain-containing protein [Source:UniProtKB/TrEMBL;Acc:L8HLF4]	0.0056277	0.389181	0.3835533	4.51E-06
3h	ACA1_365090/clean/scf7180000084641:234844-234924:+	ACA1_365090	ADPribosylglycohydrolase superfamily protein [Source:UniProtKB/TrEMBL;Acc:L8GP97]	0.142337333	0.525862667	0.383525333	0.009119307
3h	ACA1_364750/clean/scf7180000084641:189597-189670:-	ACA1_364750	UBP-type domain-containing protein [Source:UniProtKB/TrEMBL;Acc:L8GLN3]	0.014656253	0.398072333	0.38341608	4.76E-09
3h	ACA1_275600/clean/scf7180000084774:49944-50015:+	ACA1_275600	unspecified product	0.118082667	0.501398	0.383315333	0.000432534
3h	ACA1_031600/clean/scf7180000084522:22241-22308:-	ACA1_031600	Leucine rich repeat-containing protein [Source:UniProtKB/TrEMBL;Acc:L8GSM8]	0.001048426	0.384222667	0.383174241	2.29E-07
3h	ACA1_006180/clean/scf7180000083029:64917-64982:-	ACA1_006180	Multicopper oxidase, type 2 [Source:UniProtKB/TrEMBL;Acc:L8HG21]	0.2458917	0.62868	0.3827883	0.024200398
3h	ACA1_240060/clean/scf7180000084763:191650-191731:-	ACA1_240060	COesterase domain-containing protein [Source:UniProtKB/TrEMBL;Acc:L8GKI1]	0.00115601	0.38394	0.38278399	2.31E-09
3h	ACA1_091900/clean/scf7180000084645:224221-224305:-	ACA1_091900	Acylphosphatase [Source:UniProtKB/TrEMBL;Acc:L8GHY8]	0.204246667	0.586999667	0.382753	0.003791308
3h	ACA1_251300/clean/scf7180000084768:54521-54618:-	ACA1_251300	J domain-containing protein [Source:UniProtKB/TrEMBL;Acc:L8H9R3]	0.00583995	0.388535333	0.382695383	6.50E-07
3h	ACA1_044940/clean/scf7180000084576:66966-67063:-	ACA1_044940	BTB domain-containing protein [Source:UniProtKB/TrEMBL;Acc:L8GYY6]	0.001759013	0.3843566	0.382597587	5.04E-06
3h	ACA1_368860/clean/scf7180000084664:177530-177653:+	ACA1_368860	Usp domain-containing protein [Source:UniProtKB/TrEMBL;Acc:L8GY34]	0.049991	0.432518	0.382527	1.52E-05
3h	ACA1_385760/clean/scf7180000084730:257493-257632:-	ACA1_385760	MSP domain-containing protein [Source:UniProtKB/TrEMBL;Acc:L8H8G8]	0.131079667	0.513603	0.382523333	0.001326057
3h	ACA1_272530/clean/scf7180000084772:55234-55314:+	ACA1_272530	VWFA domain-containing protein [Source:UniProtKB/TrEMBL;Acc:L8HIL5]	0	0.382486867	0.382486867	8.41E-08
3h	ACA1_040720/clean/scf7180000084572:61279-61361:+	ACA1_040720	Adaptin_N domain-containing protein [Source:UniProtKB/TrEMBL;Acc:L8H2X2]	0.00788657	0.390239	0.38235243	2.36E-07
3h	ACA1_054930/clean/scf7180000084594:607466-607531:+	ACA1_054930	Zinc finger, zz type domain containing protein [Source:UniProtKB/TrEMBL;Acc:L8H689]	0.002603197	0.384763667	0.38216047	1.67E-08
3h	ACA1_392150/clean/scf7180000084740:262748-262807:-	ACA1_392150	AAA domain-containing protein [Source:UniProtKB/TrEMBL;Acc:L8GQ70]	0.00524114	0.387333667	0.382092527	1.09E-07
3h	ACA1_039450/clean/scf7180000084569:30872-30956:-	ACA1_039450	SAP domain-containing protein [Source:UniProtKB/TrEMBL;Acc:L8HI99]	0.000835947	0.382885633	0.382049687	1.52E-05
3h	ACA1_314110/clean/scf7180000083842:3057-3128:-	ACA1_314110	DH domain-containing protein [Source:UniProtKB/TrEMBL;Acc:L8GGY1]	0.014976633	0.397024333	0.3820477	9.39E-06
3h	ACA1_323430/clean/scf7180000084468:21543-21624:+	ACA1_323430	SnoaL family polyketide cyclase [Source:UniProtKB/TrEMBL;Acc:L8H0W6]	0.009211023	0.391206	0.381994977	1.21E-10
3h	ACA1_389130/clean/scf7180000084733:209238-209313:+	ACA1_389130	BTB/POZ domain containing protein [Source:UniProtKB/TrEMBL;Acc:L8GDV1]	0.0237431	0.405717667	0.381974567	3.19E-07
3h	ACA1_369710/clean/scf7180000084664:365819-365928:+	ACA1_369710	Hypothetical protein	0.052584373	0.434537667	0.381953293	0.000339715
3h	ACA1_388780/clean/scf7180000084733:169656-169730:+	ACA1_388780	EGFlike domain containing protein [Source:UniProtKB/TrEMBL;Acc:L8GDT8]	0.001016537	0.382831333	0.381814796	4.57E-09
3h	ACA1_325100/clean/scf7180000084475:966-1052:+	ACA1_325100	Calponin domain containing protein [Source:UniProtKB/TrEMBL;Acc:L8GPG8]	0.000986193	0.382742	0.381755807	6.01E-09
3h	ACA1_237440/clean/scf7180000084762:511003-511057:-	ACA1_237440	Peptidyl-prolyl cis-trans isomerase [Source:UniProtKB/TrEMBL;Acc:L8H0V6]	0.010596944	0.392266333	0.381669389	6.41E-07
3h	ACA1_173170/clean/scf7180000084741:421408-421487:-	ACA1_173170	SNF2 family Nterminal domain containing protein [Source:UniProtKB/TrEMBL;Acc:L8HGM5]	0.000540967	0.382205	0.381664033	4.02E-07
3h	ACA1_164660/clean/scf7180000084729:245014-245087:-	ACA1_164660	Protein tyrosine phosphatase, dual specificity, putative [Source:UniProtKB/TrEMBL;Acc:L8GU25]	0.011430283	0.392999	0.381568717	2.00E-09
3h	ACA1_309360/clean/scf7180000083758:12370-12449:-	ACA1_309360	hypothetical protein	0	0.381419	0.381419	0.000201605
3h	ACA1_033590/clean/scf7180000084559:48303-48395:-	ACA1_033590	Carn_acyltransf domain-containing protein [Source:UniProtKB/TrEMBL;Acc:L8GJ57]	0.01366163	0.394787667	0.381126037	1.15E-06
3h	ACA1_019960/clean/scf7180000084033:2900-3001:+	ACA1_019960	SWIRM domain containing protein [Source:UniProtKB/TrEMBL;Acc:L8GUS1]	0.0909558	0.472059	0.3811032	0.000245375
3h	ACA1_382640/clean/scf7180000084721:93099-93253:+	ACA1_382640	von Willebrand factor type A domain containing protein [Source:UniProtKB/TrEMBL;Acc:L8GUB9]	0.005564	0.386515767	0.380951767	4.14E-05
3h	ACA1_078010/clean/scf7180000084614:70609-70756:-	ACA1_078010	hypothetical protein	0.009933323	0.39082	0.380886677	1.43E-08
3h	ACA1_287870/clean/scf7180000084777:112568-112652:+	ACA1_287870	Domain found in dishevelled, egl10, and pleckstrin domain containing protein [Source:UniProtKB/TrEMBL;Acc:L8HL57]	0.00609197	0.386885667	0.380793697	9.86E-06
3h	ACA1_229490/clean/scf7180000084761:259611-259691:+	ACA1_229490	hypothetical protein	0.002441197	0.383209667	0.38076847	4.76E-08
3h	ACA1_109740/clean/scf7180000084663:16399-16579:-	ACA1_109740	Myblike DNA-binding domain containing protein [Source:UniProtKB/TrEMBL;Acc:L8HJY9]	0	0.380626667	0.380626667	2.25E-07
3h	ACA1_130550/clean/scf7180000084698:106137-106229:-	ACA1_130550	WH2 domain-containing protein [Source:UniProtKB/TrEMBL;Acc:L8GPE2]	0.000961097	0.381571333	0.380610237	7.53E-08
3h	ACA1_019960/clean/scf7180000084033:2110-2213:+	ACA1_019960	SWIRM domain containing protein [Source:UniProtKB/TrEMBL;Acc:L8GUS1]	0.036335867	0.416891667	0.3805558	2.40E-05
3h	ACA1_364370/clean/scf7180000084641:93751-93833:+	ACA1_364370	Glutathione transferase family protein [Source:UniProtKB/TrEMBL;Acc:L8GLF8]	0.017580783	0.398058667	0.380477883	6.20E-07
3h	ACA1_283050/clean/scf7180000084776:773943-774023:+	ACA1_283050	Eukaryotic translation initiation factor 4e1a-binding protein [Source:UniProtKB/TrEMBL;Acc:L8H8S1]	0.002869477	0.383108367	0.38023889	4.19E-13
3h	ACA1_378160/clean/scf7180000084711:199652-199767:-	ACA1_378160	Ubiquitin carboxyl-terminal hydrolase [Source:UniProtKB/TrEMBL;Acc:L8GSX2]	0.001460705	0.3814066	0.379945895	1.66E-09
3h	ACA1_112850/clean/scf7180000084669:232911-232999:+	ACA1_112850	Glyco_transf_20 domain-containing protein [Source:UniProtKB/TrEMBL;Acc:L8H469]	5.62E-05	0.379907	0.379850779	2.47E-06
3h	ACA1_399880/clean/scf7180000084775:55516-55596:+	ACA1_399880	Rab-GAP TBC domain-containing protein [Source:UniProtKB/TrEMBL;Acc:L8GGF5]	0.00941033	0.38924	0.37982967	1.30E-05
3h	ACA1_364510/clean/scf7180000084641:126333-126389:-	ACA1_364510	Kelch repeatcontaining protein [Source:UniProtKB/TrEMBL;Acc:L8GM67]	0.172296333	0.551924667	0.379628333	0.002853266
3h	ACA1_267340/clean/scf7180000084770:859813-859892:-	ACA1_267340	CBS domain containing protein [Source:UniProtKB/TrEMBL;Acc:L8H2L9]	0.011550267	0.391043333	0.379493067	0.000101727
3h	ACA1_027030/clean/scf7180000084464:27978-28086:-	ACA1_027030	C2 domain-containing protein [Source:UniProtKB/TrEMBL;Acc:L8GJ80]	0.005804963	0.3851449	0.379339937	0.00018073
3h	ACA1_109710/clean/scf7180000084663:5198-5319:+	ACA1_109710	FYVE zinc finger domain containing protein [Source:UniProtKB/TrEMBL;Acc:L8HIF7]	0.032123567	0.411415333	0.379291767	1.35E-05
3h	ACA1_360270/clean/scf7180000084622:80565-80632:+	ACA1_360270	hypothetical protein	0.009803933	0.389073	0.379269067	3.70E-05
3h	ACA1_063540/clean/scf7180000084599:1446998-1447082:+	ACA1_063540	hypothetical protein	0.006528753	0.385626867	0.379098113	2.45E-08
3h	ACA1_310750/clean/scf7180000083780:5265-5743:+	ACA1_310750	RFX-type winged-helix domain-containing protein [Source:UniProtKB/TrEMBL;Acc:L8GVY4]	0.141567667	0.520433667	0.378866	0.006131725
3h	ACA1_130560/clean/scf7180000084698:108441-108510:+	ACA1_130560	Glucosyl hydrolase family protein [Source:UniProtKB/TrEMBL;Acc:L8GRU3]	0.001926767	0.380777	0.378850233	1.93E-07
3h	ACA1_367360/clean/scf7180000084641:613564-613660:-	ACA1_367360	TPR_REGION domain-containing protein [Source:UniProtKB/TrEMBL;Acc:L8GMB3]	0.001572637	0.380371967	0.37879933	7.79E-08
3h	ACA1_121260/clean/scf7180000084682:17542-17609:+	ACA1_121260	Ankyrin repeat containing serine/threonine kinase [Source:UniProtKB/TrEMBL;Acc:L8GEX5]	0.000398247	0.379059	0.378660753	1.28E-07
3h	ACA1_114500/clean/scf7180000084669:487740-487839:-	ACA1_114500	Macro domain-containing protein [Source:UniProtKB/TrEMBL;Acc:L8H6J5]	0.002367162	0.380996333	0.378629171	8.18E-09
3h	ACA1_071580/clean/scf7180000084603:997551-997630:-	ACA1_071580	GOLD domain-containing protein [Source:UniProtKB/TrEMBL;Acc:L8HGH0]	0.00388205	0.38247	0.37858795	5.83E-06
3h	ACA1_207340/clean/scf7180000084757:56213-56330:+	ACA1_207340	t-SNARE coiled-coil homology domain-containing protein [Source:UniProtKB/TrEMBL;Acc:L8GY87]	0.0029319	0.381509	0.3785771	1.08E-06
3h	ACA1_336860/clean/scf7180000084596:6782-6880:-	ACA1_336860	Glutathione reductase [Source:UniProtKB/TrEMBL;Acc:L8GUD1]	0.0051747	0.383744333	0.378569633	6.69E-09
3h	ACA1_077300/clean/scf7180000084612:291119-291227:+	ACA1_077300	Serine/threonine kinase [Source:UniProtKB/TrEMBL;Acc:L8GP03]	0	0.378497333	0.378497333	2.49E-06
3h	ACA1_362670/clean/scf7180000084624:135081-135169:+	ACA1_362670	Rieske domain-containing protein [Source:UniProtKB/TrEMBL;Acc:L8GHW3]	0	0.37847	0.37847	3.07E-09
3h	ACA1_374010/clean/scf7180000084679:203888-203956:-	ACA1_374010	Peptidase M15D, vanX Dala-D-ala dipeptidase [Source:UniProtKB/TrEMBL;Acc:L8GHK1]	0.0108344	0.389188667	0.378354267	0.000140189
3h	ACA1_142090/clean/scf7180000084708:85673-85755:+	ACA1_142090	rRNA metabolism protein, SBDS family [Source:UniProtKB/TrEMBL;Acc:L8HCP3]	0.008347063	0.386671667	0.378324603	1.41E-07
3h	ACA1_095270/clean/scf7180000084645:825850-825963:-	ACA1_095270	Thioredoxin-like_fold domain-containing protein [Source:UniProtKB/TrEMBL;Acc:L8GIR9]	0.002775587	0.381096567	0.37832098	5.25E-07
3h	ACA1_231690/clean/scf7180000084761:445154-445228:-	ACA1_231690	hypothetical protein	0.0261696	0.404392	0.3782224	4.50E-05
3h	ACA1_100440/clean/scf7180000084648:77937-78021:-	ACA1_100440	Cytochrome p450 superfamily protein [Source:UniProtKB/TrEMBL;Acc:L8GKG1]	0.008597003	0.386752967	0.378155963	4.75E-07
3h	ACA1_217230/clean/scf7180000084758:546452-546551:+	ACA1_217230	RasGEF domain containing protein [Source:UniProtKB/TrEMBL;Acc:L8GSS2]	0.002785517	0.380787333	0.378001817	5.08E-05
3h	ACA1_265380/clean/scf7180000084770:455492-455573:-	ACA1_265380	Phosphatidylinositol glycan anchor biosynthesis, class S, putative [Source:UniProtKB/TrEMBL;Acc:L8H3M4]	0.012834233	0.390696	0.377861767	2.48E-06
3h	ACA1_233070/clean/scf7180000084762:118783-118908:+	ACA1_233070	hypothetical protein	0.011323067	0.388888333	0.377565267	5.40E-07
3h	ACA1_343730/clean/scf7180000084638:30718-30800:+	ACA1_343730	hypothetical protein	0.083627133	0.461146667	0.377519533	0.000283855
3h	ACA1_063800/clean/scf7180000084599:1507134-1507219:-	ACA1_063800	B30.2/SPRY domain-containing protein [Source:UniProtKB/TrEMBL;Acc:L8GZV1]	0.002737573	0.380151667	0.377414093	1.98E-06
3h	ACA1_113540/clean/scf7180000084669:314829-314913:-	ACA1_113540	SCP domain-containing protein [Source:UniProtKB/TrEMBL;Acc:L8H3B1]	0.003481767	0.380882	0.377400233	1.85E-07
3h	ACA1_268430/clean/scf7180000084770:905997-906114:-	ACA1_268430	SAM domain (Sterile alpha motif) domain containing protein [Source:UniProtKB/TrEMBL;Acc:L8H2M3]	0.294474333	0.671683667	0.377209333	0.018879746
3h	ACA1_279530/clean/scf7180000084776:409658-409719:+	ACA1_279530	Rab4, putative [Source:UniProtKB/TrEMBL;Acc:L8H946]	0.003615237	0.380821667	0.37720643	2.61E-08
3h	ACA1_360980/clean/scf7180000084622:215526-215659:+	ACA1_360980	hypothetical protein	0.0092696	0.386376667	0.377107067	8.77E-05
3h	ACA1_065970/clean/scf7180000084599:1963947-1964010:-	ACA1_065970	Usp domain-containing protein [Source:UniProtKB/TrEMBL;Acc:L8H061]	0.009448527	0.386548	0.377099473	9.13E-08
3h	ACA1_065450/clean/scf7180000084599:1856637-1856725:-	ACA1_065450	Cation_ATPase_N domain-containing protein [Source:UniProtKB/TrEMBL;Acc:L8H037]	0	0.377020867	0.377020867	0.02577576
3h	ACA1_172940/clean/scf7180000084741:367963-368045:+	ACA1_172940	Protein kinase domain-containing protein [Source:UniProtKB/TrEMBL;Acc:L8HHJ2]	0	0.377019667	0.377019667	0.002365877
3h	ACA1_321760/clean/scf7180000084455:14116-14197:-	ACA1_321760	Haloacid dehalogenaselike hydrolase domain containing protein [Source:UniProtKB/TrEMBL;Acc:L8GRZ2]	0.008254233	0.385211	0.376956767	0.000317252
3h	ACA1_301700/clean/scf7180000083222:20653-20783:-	ACA1_301700	hypothetical protein	0.02514293	0.4020832	0.37694027	2.41E-05
3h	ACA1_052440/clean/scf7180000084594:75891-75961:-	ACA1_052440	GPI mannosyltransferase 1 [Source:UniProtKB/TrEMBL;Acc:L8H760]	0.114249467	0.491075667	0.3768262	0.001302016
3h	ACA1_260830/clean/scf7180000084769:534492-534561:+	ACA1_260830	hypothetical protein	0.014625767	0.391370333	0.376744567	1.03E-06
3h	ACA1_113440/clean/scf7180000084669:276420-276530:+	ACA1_113440	hypothetical protein	0.000503623	0.377121	0.376617377	1.88E-09
3h	ACA1_051680/clean/scf7180000084592:49001-49082:+	ACA1_051680	Ras subfamily protein [Source:UniProtKB/TrEMBL;Acc:L8GPK3]	0.048998733	0.425401333	0.3764026	0.000345028
3h	ACA1_127620/clean/scf7180000084696:36475-36564:+	ACA1_127620	Fe2OG dioxygenase domain-containing protein [Source:UniProtKB/TrEMBL;Acc:L8GGE8]	0.031120767	0.407493867	0.3763731	0.005012462
3h	ACA1_247140/clean/scf7180000084763:629505-629573:+	ACA1_247140	hypothetical protein	0.006883	0.383087333	0.376204333	1.32E-05
3h	ACA1_110920/clean/scf7180000084666:15332-15396:-	ACA1_110920	F-box domain-containing protein [Source:UniProtKB/TrEMBL;Acc:L8H7X4]	0.042980883	0.418990667	0.376009783	0.000911793
3h	ACA1_372580/clean/scf7180000084679:17056-17225:-	ACA1_372580	Lipase [Source:UniProtKB/TrEMBL;Acc:L8GHE3]	0.021812333	0.397776333	0.375964	4.30E-06
3h	ACA1_369480/clean/scf7180000084664:309917-309996:-	ACA1_369480	SMP-LTD domain-containing protein [Source:UniProtKB/TrEMBL;Acc:L8GYZ6]	0.0082886	0.384181667	0.375893067	2.31E-06
3h	ACA1_133510/clean/scf7180000084701:72695-72772:+	ACA1_133510	Glutathione peroxidase [Source:UniProtKB/TrEMBL;Acc:L8H527]	0.000376477	0.376268	0.375891523	6.77E-11
3h	ACA1_011270/clean/scf7180000083623:5055-5144:-	ACA1_011270	FYVE-type domain-containing protein [Source:UniProtKB/TrEMBL;Acc:L8GQN9]	0.0731329	0.448987	0.3758541	0.034549077
3h	ACA1_215750/clean/scf7180000084758:387576-387695:+	ACA1_215750	CBS domain containing protein [Source:UniProtKB/TrEMBL;Acc:L8GR40]	0.0253586	0.401164333	0.375805733	3.03E-07
3h	ACA1_066580/clean/scf7180000084600:18347-18438:+	ACA1_066580	E3 ubiquitin-protein ligase [Source:UniProtKB/TrEMBL;Acc:L8GP23]	0	0.375736667	0.375736667	4.16E-07
3h	ACA1_243590/clean/scf7180000084763:351163-351245:+	ACA1_243590	hypothetical protein	0.00455736	0.380290333	0.375732973	1.32E-06
3h	ACA1_233420/clean/scf7180000084762:138350-138427:+	ACA1_233420	hypothetical protein	0.00725745	0.382883967	0.375626517	1.69E-09
3h	ACA1_051820/clean/scf7180000084592:58958-59027:+	ACA1_051820	Ubiquitin-like domain-containing protein [Source:UniProtKB/TrEMBL;Acc:L8GP24]	0.00478173	0.380396633	0.375614903	5.11E-12
3h	ACA1_189360/clean/scf7180000084748:17086-17158:-	ACA1_189360	Proline dehydrogenase [Source:UniProtKB/TrEMBL;Acc:L8GGF7]	0	0.375542333	0.375542333	0.001809975
3h	ACA1_057110/clean/scf7180000084599:47217-47277:-	ACA1_057110	unspecified product	0	0.375529	0.375529	0.000304132
3h	ACA1_366610/clean/scf7180000084641:462725-462820:+	ACA1_366610	DNAbinding protein HU superfamily protein [Source:UniProtKB/TrEMBL;Acc:L8GMK2]	0.00425832	0.379774667	0.375516347	1.23E-10
3h	ACA1_070640/clean/scf7180000084603:752528-752690:+	ACA1_070640	RNA recognition motif domain containing protein [Source:UniProtKB/TrEMBL;Acc:L8HE36]	0.009742442	0.385258667	0.375516225	8.41E-08
3h	ACA1_144920/clean/scf7180000084710:40845-40941:-	ACA1_144920	IgA Peptidase M64 protein [Source:UniProtKB/TrEMBL;Acc:L8GFD4]	0.00586042	0.381318	0.37545758	5.88E-09
3h	ACA1_126680/clean/scf7180000084693:76811-76893:+	ACA1_126680	Ras family protein [Source:UniProtKB/TrEMBL;Acc:L8HAC7]	0.00337501	0.3788102	0.37543519	1.22E-12
3h	ACA1_290510/clean/scf7180000084777:607109-607202:-	ACA1_290510	ABC transporter domain-containing protein [Source:UniProtKB/TrEMBL;Acc:L8HLJ2]	0.016828833	0.392195933	0.3753671	1.32E-06
3h	ACA1_144440/clean/scf7180000084709:244330-244447:+	ACA1_144440	SNF2 family Nterminal domain containing protein [Source:UniProtKB/TrEMBL;Acc:L8HFN1]	0.011698415	0.387010667	0.375312252	8.21E-08
3h	ACA1_329480/clean/scf7180000084562:20383-20443:-	ACA1_329480	hypothetical protein	0.1015356	0.476845	0.3753094	0.004818443
3h	ACA1_236630/clean/scf7180000084762:463307-463395:-	ACA1_236630	PX domain-containing protein [Source:UniProtKB/TrEMBL;Acc:L8H1G0]	0.01495493	0.3902347	0.37527977	0.000220109
3h	ACA1_084820/clean/scf7180000084632:8597-8702:+	ACA1_084820	RhoGEF domain containing protein [Source:UniProtKB/TrEMBL;Acc:L8HMR3]	0.001559693	0.376824	0.375264307	2.89E-07
3h	ACA1_282440/clean/scf7180000084776:680488-680556:+	ACA1_282440	hypothetical protein	0.018367767	0.393591	0.375223233	4.89E-06
3h	ACA1_066890/clean/scf7180000084600:71023-71321:+	ACA1_066890	RFX-type winged-helix domain-containing protein [Source:UniProtKB/TrEMBL;Acc:L8GPT8]	0.0248418	0.400038667	0.375196867	0.000463256
3h	ACA1_012990/clean/scf7180000083742:17999-18072:+	ACA1_012990	ATP-dependent DNA helicase [Source:UniProtKB/TrEMBL;Acc:L8H692]	0	0.374935333	0.374935333	1.47E-05
3h	ACA1_117270/clean/scf7180000084669:967092-967189:+	ACA1_117270	hypothetical protein	0.013967887	0.388769333	0.374801447	1.10E-06
3h	ACA1_389320/clean/scf7180000084733:229801-229875:+	ACA1_389320	BTB domain-containing protein [Source:UniProtKB/TrEMBL;Acc:M0QSM2]	0.000141985	0.3748899	0.374747915	5.18E-08
3h	ACA1_054090/clean/scf7180000084594:398950-399051:-	ACA1_054090	Fbox domain containing protein [Source:UniProtKB/TrEMBL;Acc:L8H592]	0.099180067	0.473909333	0.374729267	0.031630208
3h	ACA1_261600/clean/scf7180000084769:720783-720879:-	ACA1_261600	RING-type domain-containing protein [Source:UniProtKB/TrEMBL;Acc:L8GG78]	0.009782237	0.3843831	0.374600863	4.68E-07
3h	ACA1_377320/clean/scf7180000084711:25648-25716:+	ACA1_377320	SPRY domain containing protein [Source:UniProtKB/TrEMBL;Acc:L8GSS3]	0	0.374531333	0.374531333	9.20E-10
3h	ACA1_193320/clean/scf7180000084749:231312-231402:-	ACA1_193320	hypothetical protein	0	0.374336533	0.374336533	0.014357979
3h	ACA1_063540/clean/scf7180000084599:1446742-1446808:+	ACA1_063540	hypothetical protein	0.002288591	0.376518867	0.374230276	2.08E-08
3h	ACA1_193530/clean/scf7180000084749:253770-253864:+	ACA1_193530	RRM domain-containing protein [Source:UniProtKB/TrEMBL;Acc:L8GN98]	0.016358833	0.390565667	0.374206833	0.000188547
3h	ACA1_376220/clean/scf7180000084685:57232-57317:-	ACA1_376220	Breast carcinoma amplified sequence 2 (Bcas2) protein [Source:UniProtKB/TrEMBL;Acc:L8HFZ3]	0.00656882	0.380735667	0.374166847	2.44E-08
3h	ACA1_321930/clean/scf7180000084457:17621-17707:+	ACA1_321930	Transporter, major intrinsic protein (MIP) superfamily protein [Source:UniProtKB/TrEMBL;Acc:L8HCR2]	0	0.374149	0.374149	2.37E-06
3h	ACA1_377770/clean/scf7180000084711:116415-116530:+	ACA1_377770	WDrepeat protein pop3, putative [Source:UniProtKB/TrEMBL;Acc:L8GRX7]	0.005617967	0.379648767	0.3740308	1.82E-07
3h	ACA1_042090/clean/scf7180000084573:55521-55587:+	ACA1_042090	hypothetical protein	0	0.373954667	0.373954667	0.048247834
3h	ACA1_114590/clean/scf7180000084669:517662-517781:-	ACA1_114590	Proteasome activator protein [Source:UniProtKB/TrEMBL;Acc:L8H6L8]	0.01530526	0.389169	0.37386374	1.70E-07
3h	ACA1_288340/clean/scf7180000084777:173904-173984:+	ACA1_288340	Conditioned medium factor, putative [Source:UniProtKB/TrEMBL;Acc:L8HKD6]	0.003482656	0.377281333	0.373798678	7.83E-12
3h	ACA1_144860/clean/scf7180000084710:27645-27722:+	ACA1_144860	PH domain containing protein [Source:UniProtKB/TrEMBL;Acc:L8GCF4]	0.006410267	0.380150667	0.3737404	0.000136762
3h	ACA1_256380/clean/scf7180000084769:14878-14950:-	ACA1_256380	UBA/TS-N domain containing protein [Source:UniProtKB/TrEMBL;Acc:L8GF49]	0.008376257	0.381949333	0.373573077	2.47E-05
3h	ACA1_144110/clean/scf7180000084709:152423-152513:+	ACA1_144110	Erythronate4-phosphate dehydrogenase domain containing protein [Source:UniProtKB/TrEMBL;Acc:L8HFU3]	0.02114444	0.394715667	0.373571227	7.08E-06
3h	ACA1_352370/clean/scf7180000084704:3070-3179:+	ACA1_352370	Oxidoreductase, short chain dehydrogenase/reductase superfamily protein [Source:UniProtKB/TrEMBL;Acc:L8GTQ2]	0.062125867	0.435578333	0.373452467	0.002974473
3h	ACA1_038110/clean/scf7180000084568:74645-74704:-	ACA1_038110	Glycosyltransferase family 8 protein [Source:UniProtKB/TrEMBL;Acc:L8GN34]	0.002192983	0.375467333	0.37327435	3.69E-08
3h	ACA1_323310/clean/scf7180000084468:572-652:+	ACA1_323310	hypothetical protein	0.012842	0.385962467	0.373120467	0.000103976
3h	ACA1_378170/clean/scf7180000084711:201471-201558:-	ACA1_378170	G_PROTEIN_RECEP_F1_2 domain-containing protein [Source:UniProtKB/TrEMBL;Acc:L8GRC2]	0.00376322	0.376855367	0.373092147	5.04E-06
3h	ACA1_146620/clean/scf7180000084713:76617-76692:-	ACA1_146620	Membrane protein [Source:UniProtKB/TrEMBL;Acc:L8GVA1]	0.003525881	0.376553333	0.373027452	2.65E-07
3h	ACA1_116630/clean/scf7180000084669:815788-815895:+	ACA1_116630	BPL/LPL catalytic domain-containing protein [Source:UniProtKB/TrEMBL;Acc:L8H6V8]	0.020946483	0.393888667	0.372942183	6.99E-06
3h	ACA1_176060/clean/scf7180000084741:1116541-1116622:+	ACA1_176060	DAGKc domain-containing protein [Source:UniProtKB/TrEMBL;Acc:L8HJR7]	0.041765567	0.414499	0.372733433	3.12E-06
3h	ACA1_088880/clean/scf7180000084639:253226-253307:-	ACA1_088880	OGFr_N domain-containing protein [Source:UniProtKB/TrEMBL;Acc:L8GUP7]	0.0138889	0.3865936	0.3727047	0.003639422
3h	ACA1_384850/clean/scf7180000084730:115706-115816:-	ACA1_384850	Arrestin_C domain-containing protein [Source:UniProtKB/TrEMBL;Acc:L8H8B9]	0.00672657	0.379419033	0.372692463	5.17E-06

3h	ACA1_156720/clean/scf7180000084723:58738-58837:+	ACA1_156720	INTRADIOL_DIOXYGENAS domain-containing protein [Source:UniProtKB/TrEMBL;Acc:L8GI57]	0.010117953	0.382791667	0.372673713	4.51E-06
3h	ACA1_043650/clean/scf7180000084573:193413-193506:-	ACA1_043650	Poly(Adpribose) glycohydrolase [Source:UniProtKB/TrEMBL;Acc:L8GUS0]	0	0.372649333	0.372649333	2.91E-06
3h	ACA1_365710/clean/scf7180000084641:301242-301326:-	ACA1_365710	Protein kinase domain-containing protein [Source:UniProtKB/TrEMBL;Acc:L8GPB8]	0.001775297	0.374307	0.372531703	2.18E-07
3h	ACA1_320810/clean/scf7180000084416:26650-26800:+	ACA1_320810	PGM1_C domain-containing protein [Source:UniProtKB/TrEMBL;Acc:L8GTZ1]	0.000172028	0.372661	0.372488972	1.38E-05
3h	ACA1_107440/clean/scf7180000084660:198377-198487:+	ACA1_107440	Purple acid phosphatase [Source:UniProtKB/TrEMBL;Acc:L8GNJ7]	0.010867147	0.383352667	0.37248552	1.15E-09
3h	ACA1_092590/clean/scf7180000084645:361078-361159:-	ACA1_092590	Leucine rich repeat domain containing protein [Source:UniProtKB/TrEMBL;Acc:L8GKK8]	0.070786833	0.443196667	0.372409833	0.003046486
3h	ACA1_240360/clean/scf7180000084763:227124-227204:+	ACA1_240360	hypothetical protein	0.008614967	0.380988	0.372373033	5.24E-05
3h	ACA1_282210/clean/scf7180000084776:627570-627638:+	ACA1_282210	Betacarotene 15,15'-monooxygenase 1, putative [Source:UniProtKB/TrEMBL;Acc:L8H762]	0.0170774	0.389399667	0.372322267	8.79E-07
3h	ACA1_155110/clean/scf7180000084722:106621-106713:-	ACA1_155110	Cyclin, Nterminal domain containing protein [Source:UniProtKB/TrEMBL;Acc:L8H002]	0.074141633	0.446455333	0.3723137	1.69E-05
3h	ACA1_061150/clean/scf7180000084599:836185-836257:-	ACA1_061150	UBA/TSN domain containing protein [Source:UniProtKB/TrEMBL;Acc:L8GWQ2]	0.01757817	0.389756667	0.372178497	8.55E-06
3h	ACA1_121240/clean/scf7180000084682:9279-9362:-	ACA1_121240	Phosphatidylinositol Nacetylglucosaminyltransferase subunit c, putative [Source:UniProtKB/TrEMBL;Acc:L8GEH1]	0.032439333	0.404530667	0.372091333	0.000126955
3h	ACA1_096430/clean/scf7180000084645:1009160-1009262:-	ACA1_096430	Aminotran_1_2 domain-containing protein [Source:UniProtKB/TrEMBL;Acc:L8GIY6]	0.057821067	0.429897667	0.3720766	0.000371344
3h	ACA1_395480/clean/scf7180000084756:188708-188784:-	ACA1_395480	Copine subfamily protein [Source:UniProtKB/TrEMBL;Acc:L8H0H7]	0.023691133	0.395700333	0.3720092	0.000448158
3h	ACA1_276410/clean/scf7180000084774:167647-167841:+	ACA1_276410	Phosphate transporter [Source:UniProtKB/TrEMBL;Acc:L8GTH7]	0.007447887	0.379400667	0.37195278	1.05E-05
3h	ACA1_220570/clean/scf7180000084758:924151-924223:+	ACA1_220570	Cyclopropyl isomeraselike protein [Source:UniProtKB/TrEMBL;Acc:L8GT38]	0.002057613	0.374005667	0.371948053	1.23E-07
3h	ACA1_272960/clean/scf7180000084773:3498-3584:-	ACA1_272960	Ras-GAP domain-containing protein [Source:UniProtKB/TrEMBL;Acc:L8GFH7]	0.006708853	0.3786235	0.371914647	4.75E-07
3h	ACA1_252780/clean/scf7180000084768:208290-208539:-	ACA1_252780	Sel1 repeatcontaining protein [Source:UniProtKB/TrEMBL;Acc:L8HC46]	0.064564433	0.436473333	0.3719089	0.014321415
3h	ACA1_214610/clean/scf7180000084758:234403-234478:-	ACA1_214610	Aminotran_1_2 domain-containing protein [Source:UniProtKB/TrEMBL;Acc:L8GQ78]	0.011735633	0.383604667	0.371869033	2.15E-05
3h	ACA1_096520/clean/scf7180000084645:1032991-1033146:-	ACA1_096520	Cytochrome p450 superfamily protein [Source:UniProtKB/TrEMBL;Acc:L8GLA2]	0.0236337	0.395430333	0.371796633	0.001885842
3h	ACA1_187780/clean/scf7180000084747:93736-93812:-	ACA1_187780	Corrinoid adenosyltransferase [Source:UniProtKB/TrEMBL;Acc:L8GUU3]	0.00472731	0.376442167	0.371714857	1.15E-06
3h	ACA1_042040/clean/scf7180000084573:44825-44929:-	ACA1_042040	RAI1 domain-containing protein [Source:UniProtKB/TrEMBL;Acc:L8GV91]	0.0399878	0.411609	0.3716212	0.00058357
3h	ACA1_263870/clean/scf7180000084770:123350-123468:-	ACA1_263870	MAGE domain-containing protein [Source:UniProtKB/TrEMBL;Acc:L8H1Y1]	0.0333826	0.404887667	0.371505067	1.32E-05
3h	ACA1_172230/clean/scf7180000084741:280114-280212:+	ACA1_172230	Serine/threonine-protein phosphatase 2A activator [Source:UniProtKB/TrEMBL;Acc:L8HIY6]	0.016982903	0.388399667	0.371416763	4.63E-07
3h	ACA1_228660/clean/scf7180000084761:186466-186574:-	ACA1_228660	DNA-directed RNA polymerase III subunit RPC6 [Source:UniProtKB/TrEMBL;Acc:L8H8X0]	0.008721073	0.3801376	0.371416527	6.26E-05
3h	ACA1_153650/clean/scf7180000084720:149191-149261:+	ACA1_153650	Pterin4-alpha-carbinolamine dehydratase [Source:UniProtKB/TrEMBL;Acc:L8HG82]	0.021422167	0.392702	0.371279833	4.46E-08
3h	ACA1_110910/clean/scf7180000084666:14209-14286:-	ACA1_110910	HEAT repeat-containing protein [Source:UniProtKB/TrEMBL;Acc:L8H6V7]	0.150713	0.521819	0.371106	0.002724201
3h	ACA1_058280/clean/scf7180000084599:286913-287040:-	ACA1_058280	OGFr_N domain-containing protein [Source:UniProtKB/TrEMBL;Acc:L8GW80]	0.016993783	0.387970333	0.37097655	4.92E-07
3h	ACA1_261310/clean/scf7180000084769:634047-634119:+	ACA1_261310	Phytanoyl-CoA dioxygenase family protein [Source:UniProtKB/TrEMBL;Acc:L8GHP7]	0.007755967	0.378446367	0.3706904	0.000109085
3h	ACA1_260950/clean/scf7180000084769:559980-560080:-	ACA1_260950	Ribonuclease 25A protein [Source:UniProtKB/TrEMBL;Acc:L8GG37]	0.035663933	0.406277667	0.370613733	3.14E-05
3h	ACA1_063920/clean/scf7180000084599:1536485-1536594:-	ACA1_063920	hypothetical protein	0.00490504	0.375512	0.37060696	1.07E-06
3h	ACA1_329820/clean/scf7180000084566:3372-3461:-	ACA1_329820	Transporter, major facilitator subfamily protein [Source:UniProtKB/TrEMBL;Acc:L8GHX2]	0.0240585	0.394653333	0.370594833	1.19E-07
3h	ACA1_212820/clean/scf7180000084758:158781-158861:-	ACA1_212820	hypothetical protein	0.024882433	0.395457833	0.3705754	1.73E-05
3h	ACA1_264290/clean/scf7180000084770:230333-230425:-	ACA1_264290	Geranylgeranyl diphosphate synthase 1, putative [Source:UniProtKB/TrEMBL;Acc:L8H3G3]	0.00308642	0.373660667	0.370574247	2.09E-07
3h	ACA1_066000/clean/scf7180000084599:1971115-1971228:+	ACA1_066000	Arp2/3 complex 34 kDa subunit [Source:UniProtKB/TrEMBL;Acc:L8GWZ2]	0.01213939	0.382696	0.37055661	5.13E-13
3h	ACA1_288290/clean/scf7180000084777:160883-160972:+	ACA1_288290	SH3 domain-containing protein [Source:UniProtKB/TrEMBL;Acc:L8HKD1]	0.01002911	0.380418333	0.370389223	6.48E-06
3h	ACA1_252540/clean/scf7180000084768:176270-176389:-	ACA1_252540	hypothetical protein	0.0278816	0.3979	0.3700184	1.86E-07
3h	ACA1_278060/clean/scf7180000084776:164731-164790:-	ACA1_278060	hypothetical protein	0.009590863	0.379417333	0.36982647	9.95E-08
3h	ACA1_152930/clean/scf7180000084720:34858-34927:-	ACA1_152930	PG_binding_1 domain-containing protein [Source:UniProtKB/TrEMBL;Acc:L8HF34]	0.023026167	0.392788667	0.3697625	1.32E-05
3h	ACA1_366940/clean/scf7180000084641:518913-519036:-	ACA1_366940	BTB/POZ domain containing protein [Source:UniProtKB/TrEMBL;Acc:L8GPM1]	0.147535667	0.517197333	0.369661667	0.04633616
3h	ACA1_174300/clean/scf7180000084741:664726-664785:+	ACA1_174300	hypothetical protein	0.026236733	0.395889333	0.3696526	0.001530937
3h	ACA1_320950/clean/scf7180000084435:1169-1253:-	ACA1_320950	SET domain containing protein [Source:UniProtKB/TrEMBL;Acc:L8GPN6]	0.0907622	0.460411667	0.369649467	0.004090198
3h	ACA1_015510/clean/scf7180000083799:8363-8443:-	ACA1_015510	Replication factora protein 1 (Rpa1) subfamily protein [Source:UniProtKB/TrEMBL;Acc:L8GZQ4]	0.0619614	0.431598333	0.369636933	5.42E-05
3h	ACA1_366530/clean/scf7180000084641:447336-447447:-	ACA1_366530	hypothetical protein	0.049140167	0.418768	0.369627833	0.001415613
3h	ACA1_259490/clean/scf7180000084769:354404-354629:+	ACA1_259490	TPR_REGION domain-containing protein [Source:UniProtKB/TrEMBL;Acc:L8GEZ4]	0.057300633	0.426894333	0.3695937	5.63E-05
3h	ACA1_051920/clean/scf7180000084592:86344-86429:+	ACA1_051920	SMP-LTD domain-containing protein [Source:UniProtKB/TrEMBL;Acc:L8GP37]	0.000764527	0.370356	0.369591473	5.52E-07
3h	ACA1_048650/clean/scf7180000084582:187916-188042:+	ACA1_048650	Hsp40, putative [Source:UniProtKB/TrEMBL;Acc:L8GDD9]	0.043569767	0.413151667	0.3695819	0.000197188
3h	ACA1_107440/clean/scf7180000084660:197117-197262:+	ACA1_107440	Purple acid phosphatase [Source:UniProtKB/TrEMBL;Acc:L8GNJ7]	0.006704482	0.376210333	0.369505851	3.96E-08
3h	ACA1_184780/clean/scf7180000084746:61250-61367:+	ACA1_184780	Protein YIPF [Source:UniProtKB/TrEMBL;Acc:L8H566]	0.002270363	0.371769333	0.36949897	3.36E-08
3h	ACA1_252880/clean/scf7180000084768:233963-234086:-	ACA1_252880	hypothetical protein	0.030135787	0.399622333	0.369486547	3.11E-06
3h	ACA1_174170/clean/scf7180000084741:632983-633100:-	ACA1_174170	Glutathione Stransferase, C-terminal domain containing protein [Source:UniProtKB/TrEMBL;Acc:L8HHR9]	0.016467667	0.385818667	0.369351	9.56E-10
3h	ACA1_146600/clean/scf7180000084713:64470-64559:-	ACA1_146600	Protein kinase domain containing protein [Source:UniProtKB/TrEMBL;Acc:L8GWQ5]	0.000603774	0.369902667	0.369298892	6.12E-12
3h	ACA1_106820/clean/scf7180000084660:140689-140833:-	ACA1_106820	AAA domain-containing protein [Source:UniProtKB/TrEMBL;Acc:L8GQA4]	0.0670757	0.436338667	0.369262967	0.000102411
3h	ACA1_149980/clean/scf7180000084715:261687-261752:-	ACA1_149980	Telo_bind domain-containing protein [Source:UniProtKB/TrEMBL;Acc:L8HDH1]	0.010783573	0.379892333	0.36910876	0.000544398
3h	ACA1_396270/clean/scf7180000084759:68685-68794:+	ACA1_396270	hypothetical protein	0.020833333	0.389427	0.368593667	0.000463256
3h	ACA1_272960/clean/scf7180000084773:2315-2374:-	ACA1_272960	Ras-GAP domain-containing protein [Source:UniProtKB/TrEMBL;Acc:L8GFH7]	0.037035133	0.405541	0.368505867	6.22E-06
3h	ACA1_307560/clean/scf7180000083680:36743-36850:-	ACA1_307560	WD domain, G-beta repeat-containing protein [Source:UniProtKB/TrEMBL;Acc:L8H741]	0.015805933	0.384262	0.368456067	0.000175422
3h	ACA1_027500/clean/scf7180000084469:6658-6734:-	ACA1_027500	hypothetical protein	0.000880623	0.369154333	0.36827371	2.73E-07
3h	ACA1_162120/clean/scf7180000084726:34976-35075:+	ACA1_162120	hypothetical protein	0.007005011	0.375234	0.368228989	0.000102063
3h	ACA1_172250/clean/scf7180000084741:283363-283457:-	ACA1_172250	Leucine rich repeat domain containing protein [Source:UniProtKB/TrEMBL;Acc:L8HHK1]	0.221351	0.589431333	0.368080333	0.006443081
3h	ACA1_015560/clean/scf7180000083799:20788-20875:+	ACA1_015560	hypothetical protein	0.0093564	0.377339667	0.367983267	1.84E-07
3h	ACA1_163650/clean/scf7180000084729:62982-63059:+	ACA1_163650	hypothetical protein	0	0.367868933	0.367868933	9.25E-06
3h	ACA1_075040/clean/scf7180000084608:133788-133862:-	ACA1_075040	RhoGEF domain containing protein [Source:UniProtKB/TrEMBL;Acc:L8HHL5]	0.004243237	0.372097333	0.367854097	1.69E-06
3h	ACA1_377500/clean/scf7180000084711:76511-76598:+	ACA1_377500	Peptidase family c78 protein [Source:UniProtKB/TrEMBL;Acc:L8GU78]	0.0448145	0.412398	0.3675835	0.000776443
3h	ACA1_061860/clean/scf7180000084599:1065715-1065789:+	ACA1_061860	ATP_transf domain-containing protein [Source:UniProtKB/TrEMBL;Acc:L8GW40]	0	0.367571033	0.367571033	7.98E-06
3h	ACA1_277740/clean/scf7180000084776:90718-90790:+	ACA1_277740	MORN repeat-containing protein [Source:UniProtKB/TrEMBL;Acc:L8H6R9]	0.000447427	0.367907333	0.367459907	8.41E-05
3h	ACA1_152960/clean/scf7180000084720:41676-41745:+	ACA1_152960	Malate dehydrogenase [Source:UniProtKB/TrEMBL;Acc:L8HFT9]	0.001869921	0.369308333	0.367438413	6.24E-16
3h	ACA1_210530/clean/scf7180000084758:49657-49753:-	ACA1_210530	4Fe-4S ferredoxin-type domain-containing protein [Source:UniProtKB/TrEMBL;Acc:L8GS75]	0.007145813	0.374236333	0.36709052	6.88E-06
3h	ACA1_132620/clean/scf7180000084700:46169-46261:-	ACA1_132620	RING-CH-type domain-containing protein [Source:UniProtKB/TrEMBL;Acc:L8GV25]	0.001072577	0.368088667	0.36701609	1.23E-05
3h	ACA1_131880/clean/scf7180000084699:167255-167327:-	ACA1_131880	N-acetyltransferase domain-containing protein [Source:UniProtKB/TrEMBL;Acc:L8GMY7]	0.001327887	0.368314667	0.366986779	2.79E-08
3h	ACA1_064870/clean/scf7180000084599:1716198-1716273:-	ACA1_064870	Ubiquitin-like domain-containing protein [Source:UniProtKB/TrEMBL;Acc:L8GXI8]	0.003409733	0.370253667	0.366843933	8.73E-11
3h	ACA1_072790/clean/scf7180000084603:1297669-1297749:-	ACA1_072790	AB hydrolase-1 domain-containing protein [Source:UniProtKB/TrEMBL;Acc:L8HGV1]	0.0272397	0.3940361	0.3667964	0.000916791
3h	ACA1_399880/clean/scf7180000084775:58072-58207:+	ACA1_399880	Rab-GAP TBC domain-containing protein [Source:UniProtKB/TrEMBL;Acc:L8GGF5]	0.026351067	0.393125333	0.366774267	7.52E-05
3h	ACA1_087910/clean/scf7180000084639:128810-128956:+	ACA1_087910	hypothetical protein	0.0504208	0.417188	0.3667672	0.000455088
3h	ACA1_266230/clean/scf7180000084770:637429-637519:+	ACA1_266230	Dilute domain-containing protein [Source:UniProtKB/TrEMBL;Acc:L8H2D0]	0.015873	0.382636667	0.366763667	0.000146251
3h	ACA1_381120/clean/scf7180000084712:147842-147924:+	ACA1_381120	Maleylacetoacetate isomerase [Source:UniProtKB/TrEMBL;Acc:L8GMX6]	0.033451367	0.400133667	0.3666823	1.17E-06
3h	ACA1_346610/clean/scf7180000084658:29753-29845:+	ACA1_346610	DH domain-containing protein [Source:UniProtKB/TrEMBL;Acc:L8GHN5]	0.150859333	0.517440667	0.366581333	0.023228424
3h	ACA1_226180/clean/scf7180000084761:36205-36297:+	ACA1_226180	5'nucleotidase [Source:UniProtKB/TrEMBL;Acc:L8H8H8]	0.004532983	0.3710931	0.366560117	0.000579211
3h	ACA1_229230/clean/scf7180000084761:247497-247594:-	ACA1_229230	Mitochondrial carrier protein [Source:UniProtKB/TrEMBL;Acc:L8HB72]	0.009962417	0.37645	0.366487583	1.95E-09
3h	ACA1_087940/clean/scf7180000084639:137047-137107:+	ACA1_087940	ANK_REP_REGION domain-containing protein [Source:UniProtKB/TrEMBL;Acc:L8GTX2]	0.023679433	0.390133667	0.366454233	0.001529341
3h	ACA1_211500/clean/scf7180000084758:89745-89807:+	ACA1_211500	RAB5B protein, putative [Source:UniProtKB/TrEMBL;Acc:L8GS95]	0.094463433	0.460841667	0.366378233	0.002712306
3h	ACA1_063820/clean/scf7180000084599:1513607-1513701:+	ACA1_063820	VASt domain-containing protein [Source:UniProtKB/TrEMBL;Acc:L8GYM6]	0.027062933	0.393438	0.366375067	5.90E-05
3h	ACA1_375990/clean/scf7180000084685:23065-23280:-	ACA1_375990	AcetylCoA acetyltransferase, mitochondrial, putative [Source:UniProtKB/TrEMBL;Acc:L8HFY3]	0.000223614	0.366447333	0.36622372	2.66E-07
3h	ACA1_264730/clean/scf7180000084770:368997-369082:-	ACA1_264730	hypothetical protein	0.037315433	0.403505	0.366189567	0.000104055
3h	ACA1_064550/clean/scf7180000084599:1640903-1640992:-	ACA1_064550	60S ribosomal export protein NMD3 [Source:UniProtKB/TrEMBL;Acc:L8GYR2]	0.007727977	0.373868	0.366140023	4.99E-07
3h	ACA1_170640/clean/scf7180000084739:99094-99160:+	ACA1_170640	Protein kinase domain-containing protein [Source:UniProtKB/TrEMBL;Acc:L8HIR2]	0.005464467	0.371591167	0.3661267	6.81E-05
3h	ACA1_116020/clean/scf7180000084669:707386-707482:+	ACA1_116020	Rho-GAP domain-containing protein [Source:UniProtKB/TrEMBL;Acc:L8H6T0]	0.00192447	0.3679131	0.36598863	6.31E-07
3h	ACA1_063910/clean/scf7180000084599:1535400-1535495:+	ACA1_063910	Tho complex subunit 7 protein [Source:UniProtKB/TrEMBL;Acc:L8GXH2]	0.019128363	0.385100967	0.365972603	5.81E-06
3h	ACA1_347660/clean/scf7180000084667:6260-6317:+	ACA1_347660	Fructose-2,6-bisphosphate 2-phosphatase [Source:UniProtKB/TrEMBL;Acc:L8GM28]	0.01360793	0.379465667	0.365857737	2.60E-05
3h	ACA1_272530/clean/scf7180000084772:56353-56433:+	ACA1_272530	VWFA domain-containing protein [Source:UniProtKB/TrEMBL;Acc:L8HIL5]	0.002465977	0.368313567	0.36584759	7.31E-07
3h	ACA1_387910/clean/scf7180000084733:88763-88873:-	ACA1_387910	P/Homo B domain-containing protein [Source:UniProtKB/TrEMBL;Acc:L8GG58]	0.00478581	0.370532867	0.365747057	9.48E-06
3h	ACA1_053270/clean/scf7180000084594:240479-240550:+	ACA1_053270	Nudix hydrolase domain-containing protein [Source:UniProtKB/TrEMBL;Acc:L8H547]	0.008797353	0.374473333	0.36567598	3.12E-05
3h	ACA1_321760/clean/scf7180000084455:14414-14492:-	ACA1_321760	Haloacid dehalogenaselike hydrolase domain containing protein [Source:UniProtKB/TrEMBL;Acc:L8GRZ2]	0.026300567	0.391872667	0.3655721	0.001268429
3h	ACA1_096520/clean/scf7180000084645:1034256-1034328:-	ACA1_096520	Cytochrome p450 superfamily protein [Source:UniProtKB/TrEMBL;Acc:L8GLA2]	0	0.365549333	0.365549333	0.004400459
3h	ACA1_037190/clean/scf7180000084567:79051-79140:+	ACA1_037190	Aminotran_5 domain-containing protein [Source:UniProtKB/TrEMBL;Acc:L8H0V4]	0.005180367	0.370686667	0.3655063	5.60E-08
3h	ACA1_146500/clean/scf7180000084713:33657-33725:-	ACA1_146500	DH domain-containing protein [Source:UniProtKB/TrEMBL;Acc:L8GWP3]	0.01490777	0.380368667	0.365460897	1.67E-05
3h	ACA1_336860/clean/scf7180000084596:7456-7564:-	ACA1_336860	Glutathione reductase [Source:UniProtKB/TrEMBL;Acc:L8GUD1]	0.004468017	0.369782667	0.36531465	1.89E-08
3h	ACA1_050200/clean/scf7180000084589:53819-53916:+	ACA1_050200	Mammalian PTEN tumor suppressorlike, putative [Source:UniProtKB/TrEMBL;Acc:L8H3Q5]	0.020833333	0.386132267	0.365298933	0.000888425
3h	ACA1_363800/clean/scf7180000084641:20926-21105:-	ACA1_363800	ABC transporter domain-containing protein [Source:UniProtKB/TrEMBL;Acc:L8GM21]	0.072039633	0.437264	0.365224367	3.23E-05
3h	ACA1_210530/clean/scf7180000084758:50540-50608:-	ACA1_210530	4Fe-4S ferredoxin-type domain-containing protein [Source:UniProtKB/TrEMBL;Acc:L8GS75]	0.00934379	0.374490667	0.365146877	3.81E-05
3h	ACA1_296110/clean/scf7180000084777:1298314-1298423:-	ACA1_296110	MORN repeat variantcontaining protein [Source:UniProtKB/TrEMBL;Acc:L8HLH8]	0.001441713	0.366573833	0.36513212	1.06E-17
3h	ACA1_264380/clean/scf7180000084770:256205-256288:-	ACA1_264380	S-formylglutathione hydrolase [Source:UniProtKB/TrEMBL;Acc:L8H1Z7]	3.20E-05	0.36502	0.36498796	4.64E-10
3h	ACA1_321030/clean/scf7180000084435:22889-22989:-	ACA1_321030	hypothetical protein	0.000747101	0.36561	0.364862899	8.62E-13
3h	ACA1_150720/clean/scf7180000084716:24138-24300:-	ACA1_150720	TPR_REGION domain-containing protein [Source:UniProtKB/TrEMBL;Acc:L8H0N3]	0.000482087	0.365331467	0.36484938	5.83E-09
3h	ACA1_388490/clean/scf7180000084733:125693-125768:+	ACA1_388490	ANK_REP_REGION domain-containing protein [Source:UniProtKB/TrEMBL;Acc:L8GEA1]	0.017862347	0.382705267	0.36484292	0.00038735
3h	ACA1_219870/clean/scf7180000084758:828738-828824:-	ACA1_219870	Lysophospholipase 3 (Lysosomal phospholipase A2) isoform 7, putative [Source:UniProtKB/TrEMBL;Acc:L8GT04]	0.027746803	0.392512667	0.364765863	7.08E-06
3h	ACA1_096600/clean/scf7180000084645:1058449-1058537:+	ACA1_096600	CUE domain containing protein [Source:UniProtKB/TrEMBL;Acc:L8GJT0]	0.000590854	0.3653319	0.364741046	8.04E-08
3h	ACA1_080550/clean/scf7180000084618:32715-32818:+	ACA1_080550	FAD binding domain containing protein [Source:UniProtKB/TrEMBL;Acc:L8HBU6]	0.0599648	0.424620667	0.364655867	0.000853653
3h	ACA1_110060/clean/scf7180000084663:50654-50726:-	ACA1_110060	Initiation factor, subunit 2 family protein [Source:UniProtKB/TrEMBL;Acc:L8HHD7]	0.001386873	0.36601	0.364623127	1.78E-07
3h	ACA1_258670/clean/scf7180000084769:300030-300151:-	ACA1_258670	DUF1084 domain-containing protein [Source:UniProtKB/TrEMBL;Acc:L8GFJ7]	0.0055447	0.370151667	0.364606967	1.76E-06
3h	ACA1_015490/clean/scf7180000083799:4080-4159:+	ACA1_015490	hypothetical protein	0.053882733	0.418392667	0.364509933	0.001428336
3h	ACA1_176120/clean/scf7180000084741:1127266-1127347:+	ACA1_176120	Armadillo repeat containing 7 isoform 1 family protein [Source:UniProtKB/TrEMBL;Acc:L8HH78]	0.015625	0.380124933	0.364499933	4.62E-05
3h	ACA1_245470/clean/scf7180000084763:493953-494009:+	ACA1_245470	4F5 domain-containing protein [Source:UniProtKB/TrEMBL;Acc:L8GK44]	0.002254773	0.366698233	0.36444346	2.36E-20
3h	ACA1_092390/clean/scf7180000084645:333051-333107:+	ACA1_092390	CBS domain containing protein [Source:UniProtKB/TrEMBL;Acc:L8GIP6]	0.006666667	0.371058333	0.364391667	0.000168593
3h	ACA1_115040/clean/scf7180000084669:594335-594423:-	ACA1_115040	hypothetical protein	0.1346301	0.499000667	0.364370567	0.000477085
3h	ACA1_248650/clean/scf7180000084766:103047-103124:+	ACA1_248650	Dehydrogenase/reductase SDR family member 12, putative [Source:UniProtKB/TrEMBL;Acc:L8GXX2]	0.012345667	0.376711	0.364365333	0.000156001
3h	ACA1_389450/clean/scf7180000084733:259021-259117:+	ACA1_389450	hypothetical protein	0.02316	0.387516667	0.364356667	1.19E-08
3h	ACA1_238450/clean/scf7180000084763:96471-96564:-	ACA1_238450	TLDc domain-containing protein [Source:UniProtKB/TrEMBL;Acc:L8GM61]	0.010326733	0.374611367	0.364284633	1.24E-06
3h	ACA1_011280/clean/scf7180000083623:8404-8499:+	ACA1_011280	AAA domain-containing protein [Source:UniProtKB/TrEMBL;Acc:L8GP61]	0.00118574	0.365347333	0.364161593	2.72E-07
3h	ACA1_031950/clean/scf7180000084523:40789-40866:+	ACA1_031950	CBS domain containing protein [Source:UniProtKB/TrEMBL;Acc:L8H414]	0.072618367	0.436766667	0.3641483	0.019110219
3h	ACA1_298630/clean/scf7180000082992:5428-5589:-	ACA1_298630	Vacuolar protein sorting 16, putative [Source:UniProtKB/TrEMBL;Acc:L8H081]	0.000142713	0.364122633	0.36397992	1.90E-08
3h	ACA1_279440/clean/scf7180000084776:384455-384538:+	ACA1_279440	Hypothetical protein	0.011162307	0.375057233	0.363894927	6.45E-05
3h	ACA1_323310/clean/scf7180000084468:1691-1771:+	ACA1_323310	hypothetical protein	0.018231067	0.382029	0.363797933	0.000121018
3h	ACA1_097640/clean/scf7180000084645:1303348-1303443:+	ACA1_097640	Fbox domain containing protein [Source:UniProtKB/TrEMBL;Acc:L8GK52]	0.001267017	0.3650357	0.363768683	3.11E-06
3h	ACA1_269310/clean/scf7180000084770:969133-969221:-	ACA1_269310	RING-type domain-containing protein [Source:UniProtKB/TrEMBL;Acc:L8H2R5]	0	0.363479333	0.363479333	1.91E-08
3h	ACA1_264000/clean/scf7180000084770:149917-150017:-	ACA1_264000	Glutamine amidotransferase type-1 domain-containing protein [Source:UniProtKB/TrEMBL;Acc:L8H1W5]	0.023166633	0.386619667	0.363453033	5.79E-07
3h	ACA1_035560/clean/scf7180000084564:90049-90117:-	ACA1_035560	Short chain dehydrogenase/reductase family oxidoreductase [Source:UniProtKB/TrEMBL;Acc:L8HCW5]	0.00140147	0.364802833	0.363401363	2.83E-07
3h	ACA1_366940/clean/scf7180000084641:520165-520263:-	ACA1_366940	BTB/POZ domain containing protein [Source:UniProtKB/TrEMBL;Acc:L8GPM1]	0.00306499	0.3663441	0.36327911	0.000177496
3h	ACA1_073440/clean/scf7180000084603:1420076-1420149:+	ACA1_073440	MFS domain-containing protein [Source:UniProtKB/TrEMBL;Acc:L8HEW4]	0.033173867	0.396348	0.363174133	0.000485301
3h	ACA1_109130/clean/scf7180000084661:125288-125381:+	ACA1_109130	Serine/threonine kinase [Source:UniProtKB/TrEMBL;Acc:L8GDM5]	0.007392077	0.370477333	0.363085257	9.54E-07
3h	ACA1_114510/clean/scf7180000084669:490590-490687:+	ACA1_114510	F-box domain-containing protein [Source:UniProtKB/TrEMBL;Acc:L8H4E4]	0.033730433	0.396775667	0.363045233	0.000673532
3h	ACA1_174100/clean/scf7180000084741:612490-612574:-	ACA1_174100	Protein kinase domain containing protein [Source:UniProtKB/TrEMBL;Acc:L8HK30]	0.004124823	0.3671588	0.363033977	4.49E-07
3h	ACA1_333220/clean/scf7180000084581:21044-21173:+	ACA1_333220	hypothetical protein	0.013829073	0.376776	0.362946927	3.06E-06
3h	ACA1_366370/clean/scf7180000084641:403127-403225:+	ACA1_366370	2OG-FeII_Oxy_2 domain-containing protein [Source:UniProtKB/TrEMBL;Acc:L8GLS4]	0.008749747	0.371463	0.362713253	2.80E-08
3h	ACA1_045250/clean/scf7180000084576:94242-94324:+	ACA1_045250	hypothetical protein	0.008333333	0.371041333	0.362708	5.07E-06
3h	ACA1_280520/clean/scf7180000084776:487728-487796:-	ACA1_280520	C2 domain containing protein [Source:UniProtKB/TrEMBL;Acc:L8H660]	0.009307797	0.371933667	0.36262587	4.53E-06
3h	ACA1_007760/clean/scf7180000083166:21649-21712:+	ACA1_007760	PH domain containing protein [Source:UniProtKB/TrEMBL;Acc:L8HAY1]	0.002083333	0.364626667	0.362543333	9.01E-07
3h	ACA1_255750/clean/scf7180000084768:561918-562089:+	ACA1_255750	hypothetical protein	0.238348	0.600856667	0.362508667	0.002126192
3h	ACA1_203780/clean/scf7180000084754:212209-212287:+	ACA1_203780	hypothetical protein	0.00390511	0.3664068	0.36250169	3.10E-06

3h	ACA1_178530/clean/scf7180000084743:254103-254234:-	ACA1_178530	Dual specificity phosphatase, catalytic domain containing protein [Source:UniProtKB/TrEMBL;Acc:L8GSL7]	0	0.362408667	0.362408667	0.001074545
3h	ACA1_233880/clean/scf7180000084762:176370-176486:+	ACA1_233880	Purple acid phosphatase [Source:UniProtKB/TrEMBL;Acc:L8H124]	0.010557687	0.372938767	0.36238108	1.86E-07
3h	ACA1_133320/clean/scf7180000084701:31138-31230:+	ACA1_133320	Homeobox domain-containing protein [Source:UniProtKB/TrEMBL;Acc:L8H2R6]	0.004285019	0.366557733	0.362272715	3.45E-07
3h	ACA1_278910/clean/scf7180000084776:273908-273990:-	ACA1_278910	hypothetical protein	0.053253667	0.415497067	0.3622434	0.000378416
3h	ACA1_112100/clean/scf7180000084669:111897-111958:+	ACA1_112100	BTB/POZ domain containing protein [Source:UniProtKB/TrEMBL;Acc:L8H3Y0]	0.066941933	0.429165333	0.3622234	0.004128097
3h	ACA1_391200/clean/scf7180000084740:147426-147508:-	ACA1_391200	LmbE family protein [Source:UniProtKB/TrEMBL;Acc:L8GPC9]	0.005411009	0.367607667	0.362196658	2.23E-08
3h	ACA1_048870/clean/scf7180000084583:6674-6807:-	ACA1_048870	hypothetical protein	0.079938533	0.442117333	0.3621788	0.000877481
3h	ACA1_036520/clean/scf7180000084565:128822-128929:+	ACA1_036520	WD domain, G-beta repeat-containing protein [Source:UniProtKB/TrEMBL;Acc:L8HBN8]	0.006587322	0.368758	0.362170678	2.62E-06
3h	ACA1_191150/clean/scf7180000084748:187164-187231:+	ACA1_191150	Triosephosphate isomerase [Source:UniProtKB/TrEMBL;Acc:L8GE90]	0.007986713	0.370036667	0.362049953	4.36E-09
3h	ACA1_175860/clean/scf7180000084741:1077326-1077407:-	ACA1_175860	von Willebrand factor type A domain containing protein [Source:UniProtKB/TrEMBL;Acc:L8HH59]	0.042606667	0.404369333	0.361762667	0.022723777
3h	ACA1_398880/clean/scf7180000084759:239147-239255:-	ACA1_398880	RNA_ligase domain-containing protein [Source:UniProtKB/TrEMBL;Acc:L8HCD5]	0.000783087	0.362537867	0.36175478	3.42E-05
3h	ACA1_236350/clean/scf7180000084762:443297-443380:+	ACA1_236350	Peptidase_M28 domain-containing protein [Source:UniProtKB/TrEMBL;Acc:L8H1H1]	0.003512283	0.365156667	0.361644383	2.96E-10
3h	ACA1_298650/clean/scf7180000082992:10581-10681:+	ACA1_298650	hypothetical protein	0.005959983	0.367577333	0.36161735	2.68E-05
3h	ACA1_216040/clean/scf7180000084758:440784-440910:-	ACA1_216040	Protein kinase domain-containing protein [Source:UniProtKB/TrEMBL;Acc:L8GQH1]	0.019620267	0.381174333	0.361554067	3.97E-06
3h	ACA1_107630/clean/scf7180000084660:235791-235901:+	ACA1_107630	DNA-(apurinic or apyrimidinic site) lyase [Source:UniProtKB/TrEMBL;Acc:L8GMX8]	0.175667333	0.537199667	0.361532333	0.017675618
3h	ACA1_096820/clean/scf7180000084645:1108122-1108225:-	ACA1_096820	hypothetical protein	0.01281998	0.374256	0.36143602	1.19E-07
3h	ACA1_246520/clean/scf7180000084763:567380-567436:+	ACA1_246520	Tetratricopeptide repeat domain containing protein [Source:UniProtKB/TrEMBL;Acc:L8GKX6]	0.23263	0.5940335	0.3614035	0.007706644
3h	ACA1_052260/clean/scf7180000084594:24689-24763:-	ACA1_052260	Leucine rich repeat domain containing protein [Source:UniProtKB/TrEMBL;Acc:L8H5N7]	0.1064195	0.467704333	0.361284833	0.011882052
3h	ACA1_211500/clean/scf7180000084758:89959-90037:+	ACA1_211500	RAB5B protein, putative [Source:UniProtKB/TrEMBL;Acc:L8GS95]	0.01525359	0.376538	0.36128441	0.000526788
3h	ACA1_289830/clean/scf7180000084777:474997-475067:+	ACA1_289830	WD_REPEATS_REGION domain-containing protein [Source:UniProtKB/TrEMBL;Acc:L8HJ65]	0.0163106	0.3775277	0.3612171	6.73E-06
3h	ACA1_358790/clean/scf7180000084742:31721-31800:-	ACA1_358790	hypothetical protein	0.00954857	0.370608	0.36105943	2.58E-07
3h	ACA1_191480/clean/scf7180000084749:673-766:-	ACA1_191480	CS domain containing protein [Source:UniProtKB/TrEMBL;Acc:L8GMZ3]	0.001280518	0.362276433	0.360995915	1.85E-10
3h	ACA1_133200/clean/scf7180000084701:20338-20414:-	ACA1_133200	Protein kinase domain containing protein [Source:UniProtKB/TrEMBL;Acc:L8H3K5]	0.016541607	0.377475	0.360933393	5.42E-06
3h	ACA1_107160/clean/scf7180000084660:158503-158640:+	ACA1_107160	hypothetical protein	0	0.360882167	0.360882167	1.10E-08
3h	ACA1_009890/clean/scf7180000083468:1586-1695:-	ACA1_009890	TPR_REGION domain-containing protein [Source:UniProtKB/TrEMBL;Acc:L8GDE6]	1.00E-04	0.360976567	0.360876602	7.63E-09
3h	ACA1_217150/clean/scf7180000084758:528171-528251:-	ACA1_217150	Protein kinase domain-containing protein [Source:UniProtKB/TrEMBL;Acc:L8GR83]	0.013633453	0.3740449	0.360411447	0.000103864
3h	ACA1_149410/clean/scf7180000084715:147065-147173:+	ACA1_149410	Lectin_legB domain-containing protein [Source:UniProtKB/TrEMBL;Acc:L8HDD6]	0.012105407	0.372431833	0.360326426	5.89E-06
3h	ACA1_013340/clean/scf7180000083750:2040-2117:+	ACA1_013340	PH domain-containing protein [Source:UniProtKB/TrEMBL;Acc:L8HGJ7]	0.0220684	0.382283	0.3602146	1.22E-07
3h	ACA1_300890/clean/scf7180000083147:17584-17679:-	ACA1_300890	hypothetical protein	0.00759435	0.367802	0.36020765	1.86E-08
3h	ACA1_313600/clean/scf7180000083822:8657-8767:-	ACA1_313600	Peptidyl-prolyl cis-trans isomerase [Source:UniProtKB/TrEMBL;Acc:L8GNF0]	0.0245608	0.384709733	0.360148933	5.54E-06
3h	ACA1_052830/clean/scf7180000084594:192203-192306:-	ACA1_052830	Aldehyde oxidase and xanthine dehydrogenase, molybdopterin binding domain containing protein [Source:UniProtKB/TrEMBL;Acc:L8H821]	0.002091637	0.362101667	0.36001003	5.00E-06
3h	ACA1_348980/clean/scf7180000084689:23268-23352:+	ACA1_348980	3hydroxyanthranilate 3,4-dioxygenase [Source:UniProtKB/TrEMBL;Acc:L8GU67]	0.005385927	0.36539	0.360004073	1.22E-06
3h	ACA1_059470/clean/scf7180000084599:451576-451644:+	ACA1_059470	G-patch domain-containing protein [Source:UniProtKB/TrEMBL;Acc:L8GW60]	0.002495543	0.362472667	0.359977123	9.53E-07
3h	ACA1_324040/clean/scf7180000084470:12975-13048:-	ACA1_324040	Carrier superfamily protein [Source:UniProtKB/TrEMBL;Acc:L8HE29]	0.006032187	0.365989667	0.35995748	1.99E-08
3h	ACA1_309500/clean/scf7180000083762:9901-9988:+	ACA1_309500	Pyridine nucleotidedisulfide oxidoreductase domain containing protein [Source:UniProtKB/TrEMBL;Acc:L8GI23]	0.0167589	0.376689	0.3599301	8.30E-08
3h	ACA1_171960/clean/scf7180000084741:209246-209326:-	ACA1_171960	F-box domain-containing protein [Source:UniProtKB/TrEMBL;Acc:L8HHE3]	0.059342633	0.419227333	0.3598847	0.000484904
3h	ACA1_134400/clean/scf7180000084702:29591-29670:+	ACA1_134400	C2 NT-type domain-containing protein [Source:UniProtKB/TrEMBL;Acc:L8GF46]	0	0.359732333	0.359732333	7.92E-08
3h	ACA1_125950/clean/scf7180000084693:6024-6098:+	ACA1_125950	AB hydrolase-1 domain-containing protein [Source:UniProtKB/TrEMBL;Acc:L8HCR9]	0.000191373	0.359842	0.359650627	8.32E-08
3h	ACA1_142590/clean/scf7180000084708:193402-193539:+	ACA1_142590	unspecified product	0.00250245	0.362079	0.35957655	4.49E-07
3h	ACA1_065450/clean/scf7180000084599:1854162-1854227:-	ACA1_065450	Cation_ATPase_N domain-containing protein [Source:UniProtKB/TrEMBL;Acc:L8H037]	0.0431849	0.402690667	0.359505767	0.014195038
3h	ACA1_089800/clean/scf7180000084639:385256-385337:-	ACA1_089800	Protein kinase domain-containing protein [Source:UniProtKB/TrEMBL;Acc:L8GUU1]	0.00378704	0.363243433	0.359456393	1.53E-08
3h	ACA1_380340/clean/scf7180000084712:40874-40992:-	ACA1_380340	Leucine rich repeat-containing protein [Source:UniProtKB/TrEMBL;Acc:L8GNJ3]	0.0293211	0.388713667	0.359392567	1.70E-05
3h	ACA1_012020/clean/scf7180000083652:3751-3838:-	ACA1_012020	hypothetical protein	0.01143013	0.370532333	0.359102203	2.73E-07
3h	ACA1_140600/clean/scf7180000084707:95129-95256:-	ACA1_140600	Transmembrane protein 34 family protein [Source:UniProtKB/TrEMBL;Acc:L8GH57]	0.01406936	0.373158667	0.359089307	3.29E-06
3h	ACA1_245660/clean/scf7180000084763:516351-516489:-	ACA1_245660	hypothetical protein	0.046859433	0.405912667	0.359053233	0.001351742
3h	ACA1_135140/clean/scf7180000084702:73263-73337:+	ACA1_135140	hypothetical protein	0.060541433	0.419422067	0.358880633	0.004363672
3h	ACA1_280610/clean/scf7180000084776:505838-505911:+	ACA1_280610	C2H2-type domain-containing protein [Source:UniProtKB/TrEMBL;Acc:L8H702]	0.022721757	0.381372	0.358650243	0.000849016
3h	ACA1_096400/clean/scf7180000084645:1005120-1005220:+	ACA1_096400	Methyltransf_11 domain-containing protein [Source:UniProtKB/TrEMBL;Acc:L8GJF1]	0.003546957	0.362124667	0.35857771	1.60E-07
3h	ACA1_153680/clean/scf7180000084720:154241-154297:-	ACA1_153680	Amz2 protein [Source:UniProtKB/TrEMBL;Acc:L8HHH0]	0	0.358480567	0.358480567	0.007919052
3h	ACA1_234610/clean/scf7180000084762:304894-304968:-	ACA1_234610	Peptidyl-prolyl cis-trans isomerase [Source:UniProtKB/TrEMBL;Acc:L8H2P2]	0.003596345	0.361717667	0.358121322	2.87E-12
3h	ACA1_282960/clean/scf7180000084776:753966-754055:-	ACA1_282960	Peptidyl-prolyl cis-trans isomerase [Source:UniProtKB/TrEMBL;Acc:L8H7L6]	0.0866291	0.444698667	0.358069567	0.000226371
3h	ACA1_353620/clean/scf7180000084717:46741-46845:-	ACA1_353620	RhoGAP domain containing protein [Source:UniProtKB/TrEMBL;Acc:L8GGS6]	0.055804833	0.413779567	0.357974733	0.000397037
3h	ACA1_162370/clean/scf7180000084726:43032-43115:-	ACA1_162370	Protein kinase domain-containing protein [Source:UniProtKB/TrEMBL;Acc:L8GYN7]	0.01103124	0.368791233	0.357759993	1.86E-07
3h	ACA1_279420/clean/scf7180000084776:381866-381972:+	ACA1_279420	BAR domain containing protein [Source:UniProtKB/TrEMBL;Acc:L8H745]	0.003179057	0.360883	0.357703943	1.10E-07
3h	ACA1_389490/clean/scf7180000084733:266985-267069:-	ACA1_389490	RRM domain-containing protein [Source:UniProtKB/TrEMBL;Acc:L8GES5]	0.002064486	0.359655667	0.35759118	8.58E-09
3h	ACA1_060260/clean/scf7180000084599:630467-630638:-	ACA1_060260	RFX-type winged-helix domain-containing protein [Source:UniProtKB/TrEMBL;Acc:L8GVN2]	0.451976	0.809324667	0.357348667	0.048612615
3h	ACA1_298650/clean/scf7180000082992:12188-12277:+	ACA1_298650	hypothetical protein	0.022463833	0.3797279	0.357264067	2.74E-05
3h	ACA1_210540/clean/scf7180000084758:52370-52434:-	ACA1_210540	ABC transporter, ATPbinding domain containing protein [Source:UniProtKB/TrEMBL;Acc:L8GPZ1]	0.037089833	0.394346333	0.3572565	0.017215733
3h	ACA1_060920/clean/scf7180000084599:779077-779184:-	ACA1_060920	MFS domain-containing protein [Source:UniProtKB/TrEMBL;Acc:L8GVT5]	0.01731935	0.3745538	0.35723445	0.000806798
3h	ACA1_188220/clean/scf7180000084747:127507-127579:+	ACA1_188220	hypothetical protein	0.0444689	0.401695333	0.357226433	0.000127448
3h	ACA1_058720/clean/scf7180000084599:363582-363681:-	ACA1_058720	hypothetical protein	0.007031407	0.364117	0.357085593	8.40E-09
3h	ACA1_381570/clean/scf7180000084712:183636-183712:+	ACA1_381570	Ras-GAP domain-containing protein [Source:UniProtKB/TrEMBL;Acc:L8GMY5]	0.021047933	0.37803	0.356982067	2.25E-06
3h	ACA1_371710/clean/scf7180000084664:587403-587473:-	ACA1_371710	Fbox domain containing protein [Source:UniProtKB/TrEMBL;Acc:L8GZD1]	0.015499757	0.3722527	0.356752943	2.54E-05
3h	ACA1_362600/clean/scf7180000084624:110081-110141:+	ACA1_362600	hypothetical protein	0.0454683	0.402017667	0.356549367	0.001789401
3h	ACA1_087950/clean/scf7180000084639:140095-140174:+	ACA1_087950	Thg1 domain-containing protein [Source:UniProtKB/TrEMBL;Acc:L8GUE5]	0.010416667	0.3668422	0.356425533	5.87E-05
3h	ACA1_379350/clean/scf7180000084711:338051-338142:+	ACA1_379350	hypothetical protein	0	0.356402333	0.356402333	0.000113611
3h	ACA1_272600/clean/scf7180000084772:73163-73252:-	ACA1_272600	SAM domain (Sterile alpha motif) domain containing protein [Source:UniProtKB/TrEMBL;Acc:L8HG53]	0.009690557	0.366083333	0.356392777	8.17E-06
3h	ACA1_006200/clean/scf7180000083029:68953-69040:-	ACA1_006200	hypothetical protein	0.014331237	0.3707139	0.356382663	0.000151807
3h	ACA1_315060/clean/scf7180000083887:26134-26237:-	ACA1_315060	AAA domain-containing protein [Source:UniProtKB/TrEMBL;Acc:L8GZM9]	0.00114123	0.357482	0.35634077	8.81E-12
3h	ACA1_283300/clean/scf7180000084776:811096-811185:-	ACA1_283300	DEP domain-containing protein [Source:UniProtKB/TrEMBL;Acc:L8H8T3]	0.001885126	0.358220333	0.356335208	3.32E-07
3h	ACA1_051460/clean/scf7180000084592:18415-18494:-	ACA1_051460	hypothetical protein	0	0.3562635	0.3562635	0.015747874
3h	ACA1_053300/clean/scf7180000084594:252494-252585:+	ACA1_053300	Prolyl oligopeptidase [Source:UniProtKB/TrEMBL;Acc:L8H5Y2]	0.002673797	0.358903667	0.35622987	5.01E-10
3h	ACA1_187780/clean/scf7180000084747:94180-94277:-	ACA1_187780	Corrinoid adenosyltransferase [Source:UniProtKB/TrEMBL;Acc:L8GUU3]	0.004436523	0.360632667	0.356196143	3.25E-07
3h	ACA1_133350/clean/scf7180000084701:36463-36575:-	ACA1_133350	PlsC domain-containing protein [Source:UniProtKB/TrEMBL;Acc:L8H3L0]	0.000712283	0.356770333	0.35605805	1.71E-08
3h	ACA1_321440/clean/scf7180000084439:31178-31266:+	ACA1_321440	hypothetical protein	0.007435303	0.363491333	0.35605603	7.45E-08
3h	ACA1_110430/clean/scf7180000084663:122185-122266:-	ACA1_110430	HECT domain-containing protein [Source:UniProtKB/TrEMBL;Acc:L8HHG5]	0.00923664	0.36529	0.35605336	2.09E-07
3h	ACA1_187470/clean/scf7180000084747:73317-73370:-	ACA1_187470	hypothetical protein	0.044657867	0.400003	0.355345133	7.27E-05
3h	ACA1_221620/clean/scf7180000084760:6813-6911:-	ACA1_221620	Purple acid phosphatase [Source:UniProtKB/TrEMBL;Acc:L8GV66]	0.008243107	0.363585	0.355341893	1.12E-07
3h	ACA1_266120/clean/scf7180000084770:606223-606428:+	ACA1_266120	SAM domain-containing protein [Source:UniProtKB/TrEMBL;Acc:L8H4I6]	0.234959667	0.590146	0.355186333	0.002095361
3h	ACA1_390540/clean/scf7180000084740:65784-65852:+	ACA1_390540	Phosphoesterase family protein [Source:UniProtKB/TrEMBL;Acc:L8GNV0]	0.009072323	0.364231333	0.35515901	3.93E-05
3h	ACA1_172880/clean/scf7180000084741:347992-348066:+	ACA1_172880	Protein kinase domain-containing protein [Source:UniProtKB/TrEMBL;Acc:L8HJV9]	0.004737574	0.359864667	0.355127092	6.13E-06
3h	ACA1_167020/clean/scf7180000084736:57765-57856:+	ACA1_167020	EF-hand domain-containing protein [Source:UniProtKB/TrEMBL;Acc:L8H0U0]	0.011411207	0.366512667	0.35510146	5.07E-06
3h	ACA1_171280/clean/scf7180000084741:62616-63006:+	ACA1_171280	Nicotinate phosphoribosyltransferase [Source:UniProtKB/TrEMBL;Acc:L8HHA8]	0.037146567	0.392171333	0.355024767	0.002325575
3h	ACA1_368150/clean/scf7180000084664:67691-67881:-	ACA1_368150	Synaptobrevin protein [Source:UniProtKB/TrEMBL;Acc:L8GY05]	0.01770912	0.372645333	0.354936213	5.21E-06
3h	ACA1_027490/clean/scf7180000084469:2834-2917:-	ACA1_027490	Calcium-transporting ATPase [Source:UniProtKB/TrEMBL;Acc:L8H4Z2]	0	0.354724667	0.354724667	3.60E-09
3h	ACA1_366090/clean/scf7180000084641:345652-345716:-	ACA1_366090	RNA polymerase III subunit C3 [Source:UniProtKB/TrEMBL;Acc:L8GML8]	0.022315967	0.376976	0.354660033	0.000146283
3h	ACA1_096950/clean/scf7180000084645:1123502-1123575:+	ACA1_096950	RNA recognition motif domain containing protein [Source:UniProtKB/TrEMBL;Acc:L8GJ25]	0.002824388	0.357453267	0.354628878	6.62E-08
3h	ACA1_116760/clean/scf7180000084669:842665-842761:+	ACA1_116760	hypothetical protein	0.005169747	0.359766	0.354596253	8.48E-08
3h	ACA1_343310/clean/scf7180000084638:23188-23272:-	ACA1_343310	EF-hand domain-containing protein [Source:UniProtKB/TrEMBL;Acc:L8H9D8]	0.0523796	0.4068519	0.3544723	0.000324579
3h	ACA1_002610/clean/scf7180000082935:2862-2923:+	ACA1_002610	hypothetical protein	0.044968667	0.399353333	0.354384667	8.50E-05
3h	ACA1_043610/clean/scf7180000084573:184100-184177:-	ACA1_043610	FYVE-type domain-containing protein [Source:UniProtKB/TrEMBL;Acc:L8GV86]	0.00269179	0.356870567	0.354178777	0.000120542
3h	ACA1_210400/clean/scf7180000084758:40088-40184:-	ACA1_210400	PPM-type phosphatase domain-containing protein [Source:UniProtKB/TrEMBL;Acc:L8GQQ0]	0.00820926	0.362225667	0.354016407	2.88E-07
3h	ACA1_368700/clean/scf7180000084664:140703-140789:-	ACA1_368700	hypothetical protein	0.009168167	0.363095033	0.353926867	2.28E-05
3h	ACA1_386950/clean/scf7180000084730:381619-381724:-	ACA1_386950	hypothetical protein	0.024691367	0.378542	0.353850633	0.000159173
3h	ACA1_226760/clean/scf7180000084761:73349-73460:+	ACA1_226760	hypothetical protein	0.037654833	0.391501	0.353846167	0.001290804
3h	ACA1_051550/clean/scf7180000084592:35889-35957:-	ACA1_051550	Phenylalanine hydroxylase [Source:UniProtKB/TrEMBL;Acc:L8GPS8]	0.008983883	0.362682333	0.35369845	6.40E-09
3h	ACA1_158940/clean/scf7180000084724:301524-301617:+	ACA1_158940	Oxidoreductase [Source:UniProtKB/TrEMBL;Acc:L8HBL7]	0.009991227	0.363631133	0.353639907	2.65E-06
3h	ACA1_323530/clean/scf7180000084468:43087-43186:+	ACA1_323530	Fbox domain containing protein [Source:UniProtKB/TrEMBL;Acc:L8H0X5]	0.003004597	0.3566109	0.353606303	6.29E-05
3h	ACA1_074550/clean/scf7180000084608:42050-42122:-	ACA1_074550	zf-C2HC5 domain-containing protein [Source:UniProtKB/TrEMBL;Acc:L8HGP3]	0.040441167	0.393924667	0.3534835	0.009631851
3h	ACA1_230570/clean/scf7180000084761:379321-379410:-	ACA1_230570	DUF4139 domain-containing protein [Source:UniProtKB/TrEMBL;Acc:L8H928]	0.002481377	0.355829	0.353347623	3.57E-07
3h	ACA1_077300/clean/scf7180000084612:287216-287304:+	ACA1_077300	Serine/threonine kinase [Source:UniProtKB/TrEMBL;Acc:L8GP03]	0.020464733	0.373726667	0.353261933	0.001923265
3h	ACA1_183160/clean/scf7180000084745:348245-348329:+	ACA1_183160	Cytochrome p450 superfamily protein [Source:UniProtKB/TrEMBL;Acc:L8H807]	0.0590796	0.412332667	0.353253067	0.005150974
3h	ACA1_170290/clean/scf7180000084739:85997-86093:+	ACA1_170290	tRNA_edit domain-containing protein [Source:UniProtKB/TrEMBL;Acc:L8HIQ9]	0.169378667	0.522527333	0.353148667	0.021658392
3h	ACA1_052510/clean/scf7180000084594:92523-92612:+	ACA1_052510	PQ loop repeat family protein [Source:UniProtKB/TrEMBL;Acc:L8H5Y5]	0.008887503	0.361864	0.352976497	6.41E-08
3h	ACA1_103160/clean/scf7180000084653:10249-10339:+	ACA1_103160	PALP domain-containing protein [Source:UniProtKB/TrEMBL;Acc:L8GDL9]	0.00258398	0.355351	0.35276702	9.21E-08
3h	ACA1_183530/clean/scf7180000084745:440996-441100:-	ACA1_183530	Clu domain-containing protein [Source:UniProtKB/TrEMBL;Acc:L8H9K5]	0	0.352727	0.352727	2.47E-07
3h	ACA1_055410/clean/scf7180000084594:698739-698859:+	ACA1_055410	Protein kinase domain-containing protein [Source:UniProtKB/TrEMBL;Acc:L8H6J6]	0.0028514	0.355425	0.3525736	7.98E-06
3h	ACA1_236650/clean/scf7180000084762:466578-466803:+	ACA1_236650	Calciumbinding mitochondrial carrier protein [Source:UniProtKB/TrEMBL;Acc:L8H1J4]	0.008938687	0.361346667	0.35240798	8.69E-09
3h	ACA1_142370/clean/scf7180000084708:151634-151726:-	ACA1_142370	Serine hydroxymethyltransferase [Source:UniProtKB/TrEMBL;Acc:L8HDT1]	0.004120643	0.356425	0.352304357	8.71E-10
3h	ACA1_279570/clean/scf7180000084776:416623-416704:+	ACA1_279570	Elp3 domain-containing protein [Source:UniProtKB/TrEMBL;Acc:L8H759]	0.00631679	0.358489667	0.352172877	0.000559564
3h	ACA1_138450/clean/scf7180000084703:142002-142149:-	ACA1_138450	S1/P1 Nuclease [Source:UniProtKB/TrEMBL;Acc:L8H0X4]	0.001270276	0.3533467	0.352076424	1.34E-07
3h	ACA1_037070/clean/scf7180000084567:47522-47626:-	ACA1_037070	DLH domain-containing protein [Source:UniProtKB/TrEMBL;Acc:L8H284]	0.013793193	0.365841	0.352047807	4.11E-07
3h	ACA1_281240/clean/scf7180000084776:570117-570226:+	ACA1_281240	Dual specificity protein kinase shka, putative [Source:UniProtKB/TrEMBL;Acc:L8H7B2]	0.0149756	0.367	0.3520244	5.39E-08
3h	ACA1_071180/clean/scf7180000084603:884784-884890:-	ACA1_071180	hypothetical protein	0.012569127	0.3643869	0.351817773	2.77E-05
3h	ACA1_053890/clean/scf7180000084594:345261-345378:-	ACA1_053890	Aconitate hydratase, mitochondrial [Source:UniProtKB/TrEMBL;Acc:L8H690]	0.001087099	0.352718667	0.351631567	1.74E-08
3h	ACA1_217360/clean/scf7180000084758:576296-576457:-	ACA1_217360	Thioredoxin domain-containing protein [Source:UniProtKB/TrEMBL;Acc:L8GRA1]	0.010700757	0.362122333	0.351421577	7.17E-08
3h	ACA1_400560/clean/scf7180000084775:195585-195664:+	ACA1_400560	hypothetical protein	0.0685913	0.419738	0.3511467	0.000804975
3h	ACA1_295690/clean/scf7180000084777:1195605-1195700:-	ACA1_295690	NTP_transferase domain-containing protein [Source:UniProtKB/TrEMBL;Acc:L8HLD2]	0.004846533	0.355931333	0.3510848	1.44E-08
3h	ACA1_182150/clean/scf7180000084745:141640-141726:+	ACA1_182150	hypothetical protein	0.031850967	0.382922867	0.3510719	0.011723679
3h	ACA1_210530/clean/scf7180000084758:46876-46972:-	ACA1_210530	4Fe-4S ferredoxin-type domain-containing protein [Source:UniProtKB/TrEMBL;Acc:L8GS75]	0.0552546	0.406307333	0.351052733	0.00029315
3h	ACA1_093770/clean/scf7180000084645:642884-643002:+	ACA1_093770	hypothetical protein	0.0074074	0.358458033	0.351050633	0.000511687
3h	ACA1_199750/clean/scf7180000084753:317340-317480:-	ACA1_199750	hypothetical protein	0.030640777	0.381668333	0.351027557	1.76E-06
3h	ACA1_140530/clean/scf7180000084707:81723-81859:-	ACA1_140530	F-box domain containing protein [Source:UniProtKB/TrEMBL;Acc:L8GGR1]	0.0626271	0.413627667	0.351000567	0.000982707
3h	ACA1_170820/clean/scf7180000084741:13828-13934:-	ACA1_170820	hypothetical protein	0.00922496	0.360191333	0.350966374	1.77E-08
3h	ACA1_129350/clean/scf7180000084698:9343-9407:+	ACA1_129350	Fatty acid hydroxylase domain-containing protein [Source:UniProtKB/TrEMBL;Acc:L8GPA5]	0.005747133	0.356654667	0.350907533	7.58E-08
3h	ACA1_309640/clean/scf7180000083763:7789-7904:-	ACA1_309640	hypothetical protein	0.00248552	0.353134	0.35064848	9.32E-07
3h	ACA1_057940/clean/scf7180000084599:212853-212941:+	ACA1_057940	Alpha-mannosidase [Source:UniProtKB/TrEMBL;Acc:L8GX61]	0.003821283	0.3544072	0.350585917	1.12E-06
3h	ACA1_012020/clean/scf7180000083652:3336-3399:-	ACA1_012020	hypothetical protein	0.026215933	0.376665333	0.3504494	1.18E-06
3h	ACA1_027500/clean/scf7180000084469:6883-6956:-	ACA1_027500	hypothetical protein	0	0.350410667	0.350410667	6.70E-07
3h	ACA1_240180/clean/scf7180000084763:201250-201329:+	ACA1_240180	WD_REPEATS_REGION domain-containing protein [Source:UniProtKB/TrEMBL;Acc:L8GM97]	0	0.350369167	0.350369167	3.35E-06
3h	ACA1_176050/clean/scf7180000084741:1115030-1115110:-	ACA1_176050	Poly [ADP-ribose] polymerase [Source:UniProtKB/TrEMBL;Acc:L8HI82]	0.009216473	0.359499	0.350282527	1.95E-08
3h	ACA1_315600/clean/scf7180000083939:3645-3749:-	ACA1_315600	hypothetical protein	0.003249873	0.353519667	0.350269793	6.40E-09
3h	ACA1_051840/clean/scf7180000084592:60925-61005:-	ACA1_051840	RNA recognition motif domain containing protein [Source:UniProtKB/TrEMBL;Acc:L8GS29]	0.021120787	0.371347067	0.35022628	8.43E-06
3h	ACA1_037450/clean/scf7180000084567:93445-93506:+	ACA1_037450	NTPase_I-T domain-containing protein [Source:UniProtKB/TrEMBL;Acc:L8H395]	0.009250453	0.359413667	0.350163213	3.94E-08
3h	ACA1_060380/clean/scf7180000084599:665803-665878:-	ACA1_060380	ATG13 domain-containing protein [Source:UniProtKB/TrEMBL;Acc:L8GYW8]	0.001519297	0.351589333	0.350070037	2.14E-07
3h	ACA1_061570/clean/scf7180000084599:963989-964069:-	ACA1_061570	hypothetical protein	0.009104103	0.359109733	0.35000563	2.79E-07
3h	ACA1_163930/clean/scf7180000084729:115700-115771:+	ACA1_163930	Peptidase_M14 domain-containing protein [Source:UniProtKB/TrEMBL;Acc:L8GQX3]	0.003508767	0.353504767	0.349996	2.30E-07
3h	ACA1_155670/clean/scf7180000084722:212842-212956:+	ACA1_155670	SPRY domain containing protein [Source:UniProtKB/TrEMBL;Acc:L8H063]	0.00470969	0.354685333	0.349975644	1.07E-06

3h	ACA1_191530/clean/scf7180000084749:12297-12400:+	ACA1_191530	Pex2_Pex12 domain-containing protein [Source:UniProtKB/TrEMBL;Acc:L8GMZ9]	0.03131207	0.381119667	0.349807597	1.32E-05
3h	ACA1_089130/clean/scf7180000084639:327523-327592:+	ACA1_089130	Beta-lactamase domain-containing protein [Source:UniProtKB/TrEMBL;Acc:L8GX66]	0.001741853	0.351452667	0.349710813	3.12E-07
3h	ACA1_061040/clean/scf7180000084599:805649-805716:+	ACA1_061040	Oxidoreductase family, NADbinding Rossmann fold domain containing protein [Source:UniProtKB/TrEMBL;Acc:L8GZ28]	0.073677267	0.423331	0.349653733	0.000133512
3h	ACA1_171940/clean/scf7180000084741:204878-204961:-	ACA1_171940	hypothetical protein	0.025101744	0.374715667	0.349613923	0.00047882
3h	ACA1_295390/clean/scf7180000084777:1124678-1124794:-	ACA1_295390	Aamy domain-containing protein [Source:UniProtKB/TrEMBL;Acc:L8HIM5]	0.001387167	0.350835	0.349447833	1.10E-08
3h	ACA1_097280/clean/scf7180000084645:1208545-1208609:-	ACA1_097280	hypothetical protein	0.004475963	0.353773667	0.349297703	0.000328
3h	ACA1_018760/clean/scf7180000083912:11268-11348:+	ACA1_018760	Cell cycle control protein [Source:UniProtKB/TrEMBL;Acc:L8HJG5]	0.020456967	0.369746	0.349289033	1.29E-07
3h	ACA1_164910/clean/scf7180000084729:309590-309661:-	ACA1_164910	hypothetical protein	0.031454133	0.380456667	0.349002533	2.72E-06
3h	ACA1_331800/clean/scf7180000084578:33219-33350:-	ACA1_331800	hypothetical protein	0	0.348959333	0.348959333	1.09E-05
3h	ACA1_044720/clean/scf7180000084576:37539-37613:-	ACA1_044720	F-box domain-containing protein [Source:UniProtKB/TrEMBL;Acc:L8GZR9]	0.230686333	0.579571	0.348884667	0.009888304
3h	ACA1_035640/clean/scf7180000084564:101858-101972:-	ACA1_035640	Beta-lactamase domain-containing protein [Source:UniProtKB/TrEMBL;Acc:L8H9P2]	0.071428533	0.420241833	0.3488133	0.02683473
3h	ACA1_228760/clean/scf7180000084761:207487-207604:+	ACA1_228760	Mitochondrial carrier protein [Source:UniProtKB/TrEMBL;Acc:L8H8X8]	0.009107167	0.357745667	0.3486385	0.000449059
3h	ACA1_221650/clean/scf7180000084760:13550-13639:+	ACA1_221650	R3H domain containing protein [Source:UniProtKB/TrEMBL;Acc:L8GS35]	0.004830933	0.353465333	0.3486344	5.36E-05
3h	ACA1_107160/clean/scf7180000084660:158305-158417:+	ACA1_107160	hypothetical protein	0.002911207	0.351503633	0.348592427	4.31E-07
3h	ACA1_053410/clean/scf7180000084594:277550-277620:-	ACA1_053410	hypothetical protein	0.0323609	0.380952	0.3485911	3.23E-05
3h	ACA1_329700/clean/scf7180000084562:53182-53258:+	ACA1_329700	Electron transfer flavoprotein subunit alpha [Source:UniProtKB/TrEMBL;Acc:L8GI91]	0.001820296	0.350355333	0.348535037	1.45E-08
3h	ACA1_278070/clean/scf7180000084776:166103-166171:-	ACA1_278070	RBR-type E3 ubiquitin transferase [Source:UniProtKB/TrEMBL;Acc:L8H8S7]	0.0664162	0.414856333	0.348440133	0.000241302
3h	ACA1_063290/clean/scf7180000084599:1372534-1372628:-	ACA1_063290	hypothetical protein	0.008585817	0.357022333	0.348436517	0.000111156
3h	ACA1_268750/clean/scf7180000084770:911892-912048:+	ACA1_268750	Thioredoxin domain-containing protein [Source:UniProtKB/TrEMBL;Acc:L8H1X3]	0.00234931	0.350779667	0.348430357	2.45E-07
3h	ACA1_287760/clean/scf7180000084777:72663-72737:-	ACA1_287760	JmjC domain-containing protein [Source:UniProtKB/TrEMBL;Acc:L8HIQ1]	0.0828304	0.431221667	0.348391267	0.00860116
3h	ACA1_167040/clean/scf7180000084736:61130-61247:+	ACA1_167040	Elongation of fatty acids protein [Source:UniProtKB/TrEMBL;Acc:L8GZZ6]	0.013324582	0.361307667	0.347983084	1.53E-06
3h	ACA1_012980/clean/scf7180000083742:15968-16052:-	ACA1_012980	Endo/exonuclease/phosphatase domain-containing protein [Source:UniProtKB/TrEMBL;Acc:L8H4W5]	0.027750267	0.375645333	0.347895067	6.85E-05
3h	ACA1_268920/clean/scf7180000084770:942148-942262:-	ACA1_268920	Transket_pyr domain-containing protein [Source:UniProtKB/TrEMBL;Acc:L8H4W8]	0.003375055	0.351041	0.347665945	4.79E-07
3h	ACA1_111870/clean/scf7180000084669:50339-50419:-	ACA1_111870	SNF2 family Nterminal domain containing protein [Source:UniProtKB/TrEMBL;Acc:L8H2Z3]	0.024469377	0.372023333	0.347553957	5.43E-07
3h	ACA1_117320/clean/scf7180000084669:976028-976106:-	ACA1_117320	FAD binding domain containing protein [Source:UniProtKB/TrEMBL;Acc:L8H415]	0.001370849	0.348907667	0.347536817	1.38E-05
3h	ACA1_144430/clean/scf7180000084709:240217-240384:-	ACA1_144430	CASP_C domain-containing protein [Source:UniProtKB/TrEMBL;Acc:L8HI28]	0.033811233	0.381259167	0.347447933	8.65E-06
3h	ACA1_075020/clean/scf7180000084608:128325-128392:-	ACA1_075020	Rab7, putative [Source:UniProtKB/TrEMBL;Acc:L8HEH5]	0.014969567	0.362375667	0.3474061	8.08E-10
3h	ACA1_264070/clean/scf7180000084770:171249-171356:+	ACA1_264070	COPI_C domain-containing protein [Source:UniProtKB/TrEMBL;Acc:L8H460]	0.005208333	0.352607567	0.347399233	7.15E-06
3h	ACA1_156700/clean/scf7180000084723:55893-55976:+	ACA1_156700	Proteintyrosine phosphatase [Source:UniProtKB/TrEMBL;Acc:L8GIF7]	0.004765967	0.352141	0.347375033	5.13E-06
3h	ACA1_191880/clean/scf7180000084749:63946-64068:+	ACA1_191880	Calponin domain containing protein [Source:UniProtKB/TrEMBL;Acc:L8GP96]	0.003869543	0.351038	0.347168457	3.70E-09
3h	ACA1_119270/clean/scf7180000084672:55895-55970:+	ACA1_119270	Raclike protein [Source:UniProtKB/TrEMBL;Acc:L8HJ94]	0.003032527	0.3501802	0.347147673	3.50E-11
3h	ACA1_235050/clean/scf7180000084762:328035-328180:+	ACA1_235050	RNA ligase, putative [Source:UniProtKB/TrEMBL;Acc:L8H1C3]	0.00800999	0.3550021	0.34699211	0.000246912
3h	ACA1_264270/clean/scf7180000084770:227620-227701:-	ACA1_264270	Oxidoreductase, short chain dehydrogenase/reductase family protein [Source:UniProtKB/TrEMBL;Acc:L8H474]	0.006272407	0.353171333	0.346898927	1.95E-06
3h	ACA1_011490/clean/scf7180000083641:7396-7465:+	ACA1_011490	DH domain-containing protein [Source:UniProtKB/TrEMBL;Acc:L8GZK7]	0.006847337	0.353727667	0.34688033	8.65E-06
3h	ACA1_097960/clean/scf7180000084645:1363072-1363152:-	ACA1_097960	Ribosomal protein L33, putative [Source:UniProtKB/TrEMBL;Acc:L8GJX1]	0.010741533	0.357611667	0.346870133	1.43E-07
3h	ACA1_071180/clean/scf7180000084603:885454-885644:-	ACA1_071180	hypothetical protein	0.058195333	0.4049285	0.346733167	0.002458294
3h	ACA1_152930/clean/scf7180000084720:38286-38375:-	ACA1_152930	PG_binding_1 domain-containing protein [Source:UniProtKB/TrEMBL;Acc:L8HF34]	0	0.346455	0.346455	3.94E-07
3h	ACA1_106800/clean/scf7180000084660:133536-133692:+	ACA1_106800	EF hand domain containing protein [Source:UniProtKB/TrEMBL;Acc:L8GNA6]	0.012725042	0.359135333	0.346410291	3.12E-08
3h	ACA1_116850/clean/scf7180000084669:861119-861213:+	ACA1_116850	JmjC domain-containing protein [Source:UniProtKB/TrEMBL;Acc:L8H4R9]	0.024510233	0.370919	0.346408767	3.53E-05
3h	ACA1_277880/clean/scf7180000084776:118255-118323:+	ACA1_277880	RBR-type E3 ubiquitin transferase [Source:UniProtKB/TrEMBL;Acc:L8H6I3]	0.002226847	0.348614333	0.346387487	4.25E-07
3h	ACA1_133450/clean/scf7180000084701:59613-59703:+	ACA1_133450	HECT domain-containing protein [Source:UniProtKB/TrEMBL;Acc:L8H3L8]	0.012024927	0.358342567	0.346317639	0.000111176
3h	ACA1_106330/clean/scf7180000084660:85538-85649:-	ACA1_106330	Methyltransf_11 domain-containing protein [Source:UniProtKB/TrEMBL;Acc:L8GMT5]	0.006734633	0.352868333	0.3461337	2.52E-06
3h	ACA1_391560/clean/scf7180000084740:204144-204267:-	ACA1_391560	Ras guanine nucleotide exchange factor, putative [Source:UniProtKB/TrEMBL;Acc:L8GP78]	0.034815367	0.380947667	0.3461323	3.22E-06
3h	ACA1_234000/clean/scf7180000084762:183633-183732:+	ACA1_234000	Purple acid phosphatase [Source:UniProtKB/TrEMBL;Acc:L8H162]	0.00298379	0.348921933	0.345938143	4.11E-10
3h	ACA1_015330/clean/scf7180000083794:7289-7370:-	ACA1_015330	CBS domain containing protein [Source:UniProtKB/TrEMBL;Acc:L8GGC6]	0.011178553	0.357079867	0.345901313	1.49E-05
3h	ACA1_320790/clean/scf7180000084416:22658-22828:-	ACA1_320790	RUN domain-containing protein [Source:UniProtKB/TrEMBL;Acc:L8GTR9]	0.00237407	0.348271667	0.345897597	8.72E-07
3h	ACA1_110140/clean/scf7180000084663:73668-73797:-	ACA1_110140	hypothetical protein	0.01053479	0.356352	0.34581721	1.11E-07
3h	ACA1_396130/clean/scf7180000084759:25092-25192:-	ACA1_396130	Prefoldin, alpha subunit [Source:UniProtKB/TrEMBL;Acc:L8HDI9]	0.032485	0.378105333	0.345620333	8.33E-08
3h	ACA1_216020/clean/scf7180000084758:433339-433460:-	ACA1_216020	CudA family protein [Source:UniProtKB/TrEMBL;Acc:L8GQ76]	0.004748733	0.3503679	0.345619167	4.71E-07
3h	ACA1_365020/clean/scf7180000084641:203401-203547:+	ACA1_365020	hypothetical protein	0.056003233	0.401250333	0.3452471	0.000304964
3h	ACA1_277590/clean/scf7180000084776:83167-83265:-	ACA1_277590	Peptidase_S66 domain-containing protein [Source:UniProtKB/TrEMBL;Acc:L8H6R2]	0.00519483	0.350358333	0.345163503	1.19E-10
3h	ACA1_126030/clean/scf7180000084693:24813-24898:-	ACA1_126030	CD36 family protein [Source:UniProtKB/TrEMBL;Acc:L8H9J7]	0.0019805	0.347090667	0.345110167	2.14E-06
3h	ACA1_211790/clean/scf7180000084758:114621-114717:+	ACA1_211790	RhoGEF domain containing protein [Source:UniProtKB/TrEMBL;Acc:L8GQS6]	0.0959065	0.440872333	0.344965833	0.003241055
3h	ACA1_099100/clean/scf7180000084646:89827-89916:+	ACA1_099100	PH domain-containing protein [Source:UniProtKB/TrEMBL;Acc:L8H5M3]	0.006314353	0.351209867	0.344895513	1.61E-05
3h	ACA1_216420/clean/scf7180000084758:496886-497134:-	ACA1_216420	C2 domain-containing protein [Source:UniProtKB/TrEMBL;Acc:L8GQ92]	0.0753065	0.420197	0.3448905	0.001854386
3h	ACA1_064540/clean/scf7180000084599:1637914-1638018:-	ACA1_064540	hypothetical protein	0.0261481	0.371004667	0.344856567	0.000176227
3h	ACA1_019190/clean/scf7180000083937:12672-12762:+	ACA1_019190	Abhydrolase_3 domain-containing protein [Source:UniProtKB/TrEMBL;Acc:L8HI58]	0.008431653	0.353261467	0.344829813	2.07E-07
3h	ACA1_227730/clean/scf7180000084761:126816-126895:+	ACA1_227730	FYVE zinc finger domain containing protein [Source:UniProtKB/TrEMBL;Acc:L8H8M9]	0.072892733	0.417633333	0.3447406	0.000330921
3h	ACA1_264440/clean/scf7180000084770:287964-288021:+	ACA1_264440	Sulfurtransferase [Source:UniProtKB/TrEMBL;Acc:L8H202]	0.0619879	0.406587667	0.344599767	0.000217798
3h	ACA1_053270/clean/scf7180000084594:239782-239855:+	ACA1_053270	Nudix hydrolase domain-containing protein [Source:UniProtKB/TrEMBL;Acc:L8H547]	0.00855384	0.3529495	0.34439566	1.41E-05
3h	ACA1_400300/clean/scf7180000084775:142790-142876:+	ACA1_400300	UBIQUITIN_CONJUGAT_2 domain-containing protein [Source:UniProtKB/TrEMBL;Acc:L8GGI3]	0.011494267	0.355847567	0.3443533	0.001451547
3h	ACA1_125790/clean/scf7180000084691:73086-73179:-	ACA1_125790	Nuclear protein E33 isoform a isoform 3, putative [Source:UniProtKB/TrEMBL;Acc:L8GNY7]	0.020980223	0.365316667	0.344336443	2.39E-06
3h	ACA1_027390/clean/scf7180000084465:11019-11106:-	ACA1_027390	hypothetical protein	0.014757103	0.359064333	0.34430723	5.15E-09
3h	ACA1_012330/clean/scf7180000083740:19290-19375:+	ACA1_012330	WD_REPEATS_REGION domain-containing protein [Source:UniProtKB/TrEMBL;Acc:L8HCD7]	0	0.344188333	0.344188333	8.70E-09
3h	ACA1_075370/clean/scf7180000084608:189036-189127:+	ACA1_075370	Amine oxidase [Source:UniProtKB/TrEMBL;Acc:L8HGX7]	0	0.344159333	0.344159333	1.53E-06
3h	ACA1_157910/clean/scf7180000084724:148872-149004:+	ACA1_157910	Leucine rich repeat domain containing protein [Source:UniProtKB/TrEMBL;Acc:L8H970]	0.019921967	0.364059233	0.344137267	0.00064282
3h	ACA1_279310/clean/scf7180000084776:352867-352937:+	ACA1_279310	hypothetical protein	0.001242866	0.345370667	0.3441278	7.41E-06
3h	ACA1_244850/clean/scf7180000084763:438630-438712:+	ACA1_244850	EF-hand domain-containing protein [Source:UniProtKB/TrEMBL;Acc:L8GK26]	0.046023933	0.390151667	0.344127733	0.000266093
3h	ACA1_023890/clean/scf7180000084436:33024-33091:-	ACA1_023890	SAM domain-containing protein [Source:UniProtKB/TrEMBL;Acc:L8GSK7]	0.003067457	0.347084	0.344016543	5.62E-07
3h	ACA1_248410/clean/scf7180000084766:59382-59451:-	ACA1_248410	Alpha2-macroglobulin domain containing protein [Source:UniProtKB/TrEMBL;Acc:L8GY08]	0.007485247	0.351348667	0.34386342	9.13E-06
3h	ACA1_399230/clean/scf7180000084759:277602-277689:+	ACA1_399230	Synaptobrevin protein [Source:UniProtKB/TrEMBL;Acc:L8HCF2]	0.0555272	0.3993105	0.3437833	0.000131212
3h	ACA1_174780/clean/scf7180000084741:795928-796073:-	ACA1_174780	VanY domain-containing protein [Source:UniProtKB/TrEMBL;Acc:L8HK80]	0.043326467	0.387105	0.343778533	1.77E-05
3h	ACA1_063740/clean/scf7180000084599:1492988-1493075:-	ACA1_063740	Transmembrane protein [Source:UniProtKB/TrEMBL;Acc:L8GXB1]	0.004306127	0.348067333	0.343761207	2.47E-05
3h	ACA1_164750/clean/scf7180000084729:265255-265346:-	ACA1_164750	Carbonic anhydrase [Source:UniProtKB/TrEMBL;Acc:L8GR38]	0.00236594	0.345792333	0.343426393	6.00E-10
3h	ACA1_158550/clean/scf7180000084724:249111-249213:+	ACA1_158550	Enoyl-CoA hydratase, putative [Source:UniProtKB/TrEMBL;Acc:L8HA73]	0.101563033	0.444979	0.343415967	0.000569301
3h	ACA1_198250/clean/scf7180000084753:66075-66160:+	ACA1_198250	hypothetical protein	0.0506967	0.394036	0.3433393	0.000847117
3h	ACA1_107160/clean/scf7180000084660:157873-157970:+	ACA1_107160	hypothetical protein	9.82E-05	0.3433651	0.343266887	8.38E-09
3h	ACA1_374430/clean/scf7180000084679:296537-296632:-	ACA1_374430	Cyclin N-terminal domain-containing protein [Source:UniProtKB/TrEMBL;Acc:L8GGZ8]	0.0140171	0.357255	0.3432379	1.44E-05
3h	ACA1_149030/clean/scf7180000084715:53559-53640:+	ACA1_149030	Thioredoxin domain-containing protein [Source:UniProtKB/TrEMBL;Acc:L8HEB1]	0.00076648	0.343941	0.34317452	3.43E-07
3h	ACA1_368140/clean/scf7180000084664:66756-66834:-	ACA1_368140	Glutamine amidotransferase type-2 domain-containing protein [Source:UniProtKB/TrEMBL;Acc:L8H040]	0	0.342753	0.342753	0.015380556
3h	ACA1_216420/clean/scf7180000084758:494622-494748:-	ACA1_216420	C2 domain-containing protein [Source:UniProtKB/TrEMBL;Acc:L8GQ92]	0.0562022	0.398908667	0.342706467	9.30E-05
3h	ACA1_329680/clean/scf7180000084562:49904-50004:-	ACA1_329680	Glyco_hydro_15 domain-containing protein [Source:UniProtKB/TrEMBL;Acc:L8GHW7]	0.002701243	0.345266	0.342564757	1.46E-06
3h	ACA1_091960/clean/scf7180000084645:237349-237463:-	ACA1_091960	hypothetical protein	0.015316617	0.35782	0.342503383	7.32E-05
3h	ACA1_045640/clean/scf7180000084576:167658-167776:+	ACA1_045640	hypothetical protein	0.004748546	0.347246667	0.342498121	8.60E-07
3h	ACA1_383100/clean/scf7180000084721:140987-141061:+	ACA1_383100	Glutamine amidotransferase type-2 domain-containing protein [Source:UniProtKB/TrEMBL;Acc:L8GUD3]	0.005607567	0.348022333	0.342414766	1.86E-05
3h	ACA1_020120/clean/scf7180000084037:7739-7792:+	ACA1_020120	Peptidase S53 domain-containing protein [Source:UniProtKB/TrEMBL;Acc:L8GL48]	0.152108	0.49443	0.342322	0.007116638
3h	ACA1_050400/clean/scf7180000084589:86032-86100:+	ACA1_050400	Usp domain-containing protein [Source:UniProtKB/TrEMBL;Acc:L8H3R6]	0.034831467	0.377137	0.342305533	2.39E-05
3h	ACA1_291910/clean/scf7180000084777:849427-849528:-	ACA1_291910	RING-CH-type domain-containing protein [Source:UniProtKB/TrEMBL;Acc:L8HJI4]	0.018594833	0.360833	0.342238167	0.000228259
3h	ACA1_150720/clean/scf7180000084716:24813-24943:-	ACA1_150720	TPR_REGION domain-containing protein [Source:UniProtKB/TrEMBL;Acc:L8H0N3]	0.003631095	0.3458622	0.342231105	8.49E-08
3h	ACA1_126460/clean/scf7180000084693:72672-72751:+	ACA1_126460	hypothetical protein	0	0.342139233	0.342139233	2.87E-07
3h	ACA1_258610/clean/scf7180000084769:281479-281566:+	ACA1_258610	hypothetical protein	0.017543867	0.359596667	0.3420528	0.009015092
3h	ACA1_036530/clean/scf7180000084565:131943-132054:+	ACA1_036530	CRT-like domain-containing protein [Source:UniProtKB/TrEMBL;Acc:L8HCN5]	0.000525423	0.342519733	0.341994311	2.26E-08
3h	ACA1_063460/clean/scf7180000084599:1414231-1414296:-	ACA1_063460	AAA domain-containing protein [Source:UniProtKB/TrEMBL;Acc:L8GXD1]	0.021453967	0.363377667	0.3419237	4.65E-07
3h	ACA1_369710/clean/scf7180000084664:366147-366252:+	ACA1_369710	Hypothetical protein	0.025	0.366521433	0.341521433	0.001337604
3h	ACA1_282190/clean/scf7180000084776:623109-623222:-	ACA1_282190	Synapsin, ATP binding domain containing protein [Source:UniProtKB/TrEMBL;Acc:L8H7E1]	0	0.341392333	0.341392333	9.95E-08
3h	ACA1_161520/clean/scf7180000084726:11545-11675:-	ACA1_161520	Protein kinase domain-containing protein [Source:UniProtKB/TrEMBL;Acc:L8GZW0]	0.01880913	0.360168	0.34135887	2.27E-05
3h	ACA1_269280/clean/scf7180000084770:964523-964604:-	ACA1_269280	RNA methyltransferase protein [Source:UniProtKB/TrEMBL;Acc:L8H2P1]	0	0.341293333	0.341293333	1.43E-08
3h	ACA1_167020/clean/scf7180000084736:57078-57146:+	ACA1_167020	EF-hand domain-containing protein [Source:UniProtKB/TrEMBL;Acc:L8H0U0]	0.009947437	0.351058	0.341110563	1.30E-05
3h	ACA1_034530/clean/scf7180000084563:64713-64799:+	ACA1_034530	hypothetical protein	0.006107653	0.347204667	0.341097013	1.50E-05
3h	ACA1_082160/clean/scf7180000084623:16694-16788:+	ACA1_082160	Nudix hydrolase domain-containing protein [Source:UniProtKB/TrEMBL;Acc:L8GNB9]	0.000803213	0.341825	0.341021787	1.73E-07
3h	ACA1_250030/clean/scf7180000084767:27167-27233:-	ACA1_250030	C2 NT-type domain-containing protein [Source:UniProtKB/TrEMBL;Acc:L8HC94]	0.00959938	0.350527333	0.340927953	2.01E-05
3h	ACA1_144350/clean/scf7180000084709:212705-212787:+	ACA1_144350	CN hydrolase domain-containing protein [Source:UniProtKB/TrEMBL;Acc:L8HEV4]	0.0582597	0.399176667	0.340916967	0.000145143
3h	ACA1_261980/clean/scf7180000084770:12145-12224:+	ACA1_261980	Defective in cullin neddylation protein [Source:UniProtKB/TrEMBL;Acc:L8H1R9]	0.019649833	0.360417667	0.340767833	1.84E-05
3h	ACA1_325210/clean/scf7180000084475:30781-30892:+	ACA1_325210	CitMHS domain-containing protein [Source:UniProtKB/TrEMBL;Acc:L8GPH6]	0.004672997	0.345423333	0.340750337	1.33E-06
3h	ACA1_061700/clean/scf7180000084599:1013822-1013921:+	ACA1_061700	Amine oxidase family protein [Source:UniProtKB/TrEMBL;Acc:L8GWP7]	0.016521767	0.357214833	0.340693067	1.09E-05
3h	ACA1_285210/clean/scf7180000084776:961182-961325:-	ACA1_285210	Protein kinase domain containing protein [Source:UniProtKB/TrEMBL;Acc:L8H7H7]	0.010333699	0.350980433	0.340646735	2.25E-06
3h	ACA1_143940/clean/scf7180000084709:109794-109910:-	ACA1_143940	VWFA domain-containing protein [Source:UniProtKB/TrEMBL;Acc:L8HEQ6]	0.050280333	0.390852067	0.340571733	0.00023272
3h	ACA1_112030/clean/scf7180000084669:89103-89186:-	ACA1_112030	COMM domain-containing protein [Source:UniProtKB/TrEMBL;Acc:L8H411]	0.002083333	0.342616333	0.340533	1.46E-07
3h	ACA1_186060/clean/scf7180000084746:160727-160849:-	ACA1_186060	Dual specificity phosphatase, catalytic domain containing protein [Source:UniProtKB/TrEMBL;Acc:L8H5A1]	0.020636233	0.361108833	0.3404726	0.000112436
3h	ACA1_129370/clean/scf7180000084698:11680-11785:+	ACA1_129370	GRAM domain-containing protein [Source:UniProtKB/TrEMBL;Acc:L8GPH7]	0.004737513	0.3450029	0.340265387	9.00E-07
3h	ACA1_063500/clean/scf7180000084599:1432881-1432973:+	ACA1_063500	U-box domain-containing protein [Source:UniProtKB/TrEMBL;Acc:L8GZS8]	0	0.340121667	0.340121667	4.24E-08
3h	ACA1_313850/clean/scf7180000083835:1187-1275:+	ACA1_313850	PAS, putative [Source:UniProtKB/TrEMBL;Acc:L8H077]	0	0.340077333	0.340077333	0.005756556
3h	ACA1_069760/clean/scf7180000084603:548260-548331:-	ACA1_069760	VWFA domain-containing protein [Source:UniProtKB/TrEMBL;Acc:L8HG18]	0	0.340059667	0.340059667	0.000271289
3h	ACA1_224310/clean/scf7180000084760:166677-166744:+	ACA1_224310	PH domain-containing protein [Source:UniProtKB/TrEMBL;Acc:L8GTW7]	0.002929143	0.342764	0.339834857	6.97E-07
3h	ACA1_023850/clean/scf7180000084436:21635-21707:-	ACA1_023850	Protein kinase domain-containing protein [Source:UniProtKB/TrEMBL;Acc:L8GTK1]	0.02474569	0.364391333	0.339645643	2.96E-05
3h	ACA1_161750/clean/scf7180000084726:20030-20183:-	ACA1_161750	hypothetical protein	0.00616693	0.345649733	0.339482803	0.000231873
3h	ACA1_119010/clean/scf7180000084672:9202-9305:+	ACA1_119010	Ras-GEF domain-containing protein [Source:UniProtKB/TrEMBL;Acc:L8HJ80]	0.025641033	0.364991	0.339349967	0.038649378
3h	ACA1_210520/clean/scf7180000084758:44074-44214:+	ACA1_210520	SANT domain-containing protein [Source:UniProtKB/TrEMBL;Acc:L8GPP0]	0.058587	0.397914667	0.339327667	0.000998513
3h	ACA1_149210/clean/scf7180000084715:98277-98386:-	ACA1_149210	hypothetical protein	0.015683233	0.354966667	0.339283433	5.15E-05
3h	ACA1_285540/clean/scf7180000084776:990418-990492:-	ACA1_285540	GPS domain-containing protein [Source:UniProtKB/TrEMBL;Acc:L8H7T4]	0	0.3392711	0.3392711	1.71E-05
3h	ACA1_281820/clean/scf7180000084776:608132-608195:+	ACA1_281820	BPI2 domain-containing protein [Source:UniProtKB/TrEMBL;Acc:L8H8K5]	0.008100713	0.346800067	0.338699353	5.07E-06
3h	ACA1_295580/clean/scf7180000084777:1177642-1177749:+	ACA1_295580	Solute carrier family protein [Source:UniProtKB/TrEMBL;Acc:L8HJT6]	0.009901	0.34853	0.338629	6.88E-06
3h	ACA1_290900/clean/scf7180000084777:697494-697589:-	ACA1_290900	Fbox domain containing protein [Source:UniProtKB/TrEMBL;Acc:L8HJB9]	0.012948107	0.351563667	0.33861556	4.20E-06
3h	ACA1_055450/clean/scf7180000084594:708264-708348:+	ACA1_055450	SBF2 domain-containing protein [Source:UniProtKB/TrEMBL;Acc:L8H5J1]	0.0556555	0.39421	0.3385545	1.70E-05
3h	ACA1_053350/clean/scf7180000084594:267829-267951:+	ACA1_053350	Histone domain-containing protein [Source:UniProtKB/TrEMBL;Acc:L8H5Y7]	0.018462067	0.356949333	0.338487267	3.46E-05
3h	ACA1_290440/clean/scf7180000084777:593300-593391:-	ACA1_290440	hypothetical protein	0.011994167	0.350448667	0.3384545	6.27E-05
3h	ACA1_375930/clean/scf7180000084685:5713-5834:-	ACA1_375930	SUN domain-containing protein [Source:UniProtKB/TrEMBL;Acc:L8HIE1]	0	0.338408333	0.338408333	1.03E-05
3h	ACA1_067770/clean/scf7180000084603:12864-12944:+	ACA1_067770	CBS domain containing protein [Source:UniProtKB/TrEMBL;Acc:L8HEJ6]	0.0175484	0.355887333	0.338338933	1.08E-06
3h	ACA1_325500/clean/scf7180000084495:21736-21872:+	ACA1_325500	Usp domain-containing protein [Source:UniProtKB/TrEMBL;Acc:L8HB68]	0.017879033	0.356169333	0.3382903	3.47E-07
3h	ACA1_133350/clean/scf7180000084701:36873-36966:-	ACA1_133350	PlsC domain-containing protein [Source:UniProtKB/TrEMBL;Acc:L8H3L0]	0.002347417	0.340538333	0.338190917	1.78E-06
3h	ACA1_069640/clean/scf7180000084603:498390-498517:-	ACA1_069640	Thioesterase family domain containing protein [Source:UniProtKB/TrEMBL;Acc:L8HCV4]	0.00273996	0.340606733	0.337866773	1.24E-05
3h	ACA1_245660/clean/scf7180000084763:515695-515807:-	ACA1_245660	hypothetical protein	0.050314567	0.388019333	0.337704767	0.000780979
3h	ACA1_031650/clean/scf7180000084522:39718-39797:-	ACA1_031650	Endo/exonuclease/phosphatase domain-containing protein [Source:UniProtKB/TrEMBL;Acc:L8GSN6]	0.0138889	0.351532	0.3376431	0.0028748
3h	ACA1_368770/clean/scf7180000084664:157495-157572:-	ACA1_368770	Glyco_transf_20 domain-containing protein [Source:UniProtKB/TrEMBL;Acc:L8GYU8]	0.091790333	0.429429667	0.337639333	0.002345742
3h	ACA1_153010/clean/scf7180000084720:53815-53877:-	ACA1_153010	hypothetical protein	0.334234667	0.671837	0.337602333	0.005490548
3h	ACA1_306850/clean/scf7180000083668:20789-20860:+	ACA1_306850	RanBPMrelated, putative [Source:UniProtKB/TrEMBL;Acc:L8GNY2]	0.0507147	0.388045	0.3373303	4.24E-05
3h	ACA1_201470/clean/scf7180000084753:559589-559680:-	ACA1_201470	Rasrelated protein Rab-2B, putative [Source:UniProtKB/TrEMBL;Acc:L8H2N2]	0.013388173	0.350586333	0.33719816	1.09E-07
3h	ACA1_140220/clean/scf7180000084707:45898-46032:-	ACA1_140220	hypothetical protein	0.009624267	0.346778	0.337153733	1.55E-05

3h	ACA1_138060/clean/scf7180000084703:83840-83979:-	ACA1_138060	hypothetical protein	0.002564103	0.339460333	0.33689623	2.73E-06
3h	ACA1_051680/clean/scf7180000084592:48486-48559:+	ACA1_051680	Ras subfamily protein [Source:UniProtKB/TrEMBL;Acc:L8GPK3]	0.0395882	0.376437667	0.336849467	0.001129541
3h	ACA1_038180/clean/scf7180000084568:90497-90571:+	ACA1_038180	FHA domain containing protein [Source:UniProtKB/TrEMBL;Acc:L8GLQ3]	0.035789733	0.372611333	0.3368216	5.24E-05
3h	ACA1_230470/clean/scf7180000084761:356650-356728:+	ACA1_230470	hypothetical protein	0	0.336789733	0.336789733	0.001355048
3h	ACA1_285540/clean/scf7180000084776:992445-992515:-	ACA1_285540	GPS domain-containing protein [Source:UniProtKB/TrEMBL;Acc:L8H7T4]	0.00275482	0.33954	0.33678518	1.25E-05
3h	ACA1_064240/clean/scf7180000084599:1567024-1567106:+	ACA1_064240	NAD(P)-bd_dom domain-containing protein [Source:UniProtKB/TrEMBL;Acc:L8GXD4]	0.00673553	0.343496	0.33676047	2.72E-10
3h	ACA1_320550/clean/scf7180000084416:10044-10177:-	ACA1_320550	Proline iminopeptidase [Source:UniProtKB/TrEMBL;Acc:L8GWD8]	0.004775203	0.341448667	0.336673463	3.93E-12
3h	ACA1_087720/clean/scf7180000084639:105307-105395:+	ACA1_087720	Dihydrodipicolinate synthetase [Source:UniProtKB/TrEMBL;Acc:L8GVJ3]	0.000328407	0.336805	0.336476593	5.81E-05
3h	ACA1_113650/clean/scf7180000084669:343300-343384:+	ACA1_113650	hypothetical protein	0.009561823	0.346012667	0.336450843	4.39E-08
3h	ACA1_352370/clean/scf7180000084704:3253-3320:+	ACA1_352370	Oxidoreductase, short chain dehydrogenase/reductase superfamily protein [Source:UniProtKB/TrEMBL;Acc:L8GTQ2]	0.0238918	0.360291667	0.336399867	0.000584317
3h	ACA1_179970/clean/scf7180000084744:12240-12338:+	ACA1_179970	PPM-type phosphatase domain-containing protein [Source:UniProtKB/TrEMBL;Acc:L8GF73]	0.004529617	0.340898333	0.336368717	1.81E-08
3h	ACA1_331800/clean/scf7180000084578:33940-34061:-	ACA1_331800	hypothetical protein	0.0040855	0.340434333	0.336348833	0.00017258
3h	ACA1_288810/clean/scf7180000084777:292516-292614:-	ACA1_288810	Leucine rich repeat domain containing protein [Source:UniProtKB/TrEMBL;Acc:L8HIZ3]	0.059967933	0.396133667	0.336165733	0.02342758
3h	ACA1_119410/clean/scf7180000084672:64174-64236:+	ACA1_119410	PCI domain-containing protein [Source:UniProtKB/TrEMBL;Acc:L8HLX4]	0.003923061	0.340079533	0.336156472	2.25E-08
3h	ACA1_107400/clean/scf7180000084660:184195-184281:-	ACA1_107400	Nucleoside diphosphate, putative [Source:UniProtKB/TrEMBL;Acc:L8GNB6]	0.0238075	0.359884333	0.336076833	3.97E-05
3h	ACA1_013670/clean/scf7180000083751:19865-19948:+	ACA1_013670	HMA domain-containing protein [Source:UniProtKB/TrEMBL;Acc:L8HHA3]	0.013992593	0.3500105	0.336017907	1.49E-08
3h	ACA1_037740/clean/scf7180000084568:12656-12765:-	ACA1_037740	hypothetical protein	0.0035461	0.339550667	0.336004567	4.13E-06
3h	ACA1_253150/clean/scf7180000084768:252136-252208:+	ACA1_253150	RAB8D, putative [Source:UniProtKB/TrEMBL;Acc:L8H9Y3]	0.006704447	0.342614	0.335909553	2.39E-06
3h	ACA1_218440/clean/scf7180000084758:659643-659765:+	ACA1_218440	RNA_pol_L_2 domain-containing protein [Source:UniProtKB/TrEMBL;Acc:L8GQD6]	0.013184633	0.349025667	0.335841033	1.17E-08
3h	ACA1_220710/clean/scf7180000084758:950349-950440:+	ACA1_220710	Malate dehydrogenase [Source:UniProtKB/TrEMBL;Acc:L8GQP0]	0.00041848	0.336243	0.33582452	7.09E-11
3h	ACA1_002750/clean/scf7180000082937:4567-4630:-	ACA1_002750	F-box domain-containing protein [Source:UniProtKB/TrEMBL;Acc:L8GHD2]	0.017473857	0.3532226	0.335748743	2.44E-05
3h	ACA1_100600/clean/scf7180000084648:127384-127462:+	ACA1_100600	AAA_28 domain-containing protein [Source:UniProtKB/TrEMBL;Acc:L8GKH3]	0.004593637	0.340315667	0.33572203	5.80E-07
3h	ACA1_280610/clean/scf7180000084776:506654-506767:+	ACA1_280610	C2H2-type domain-containing protein [Source:UniProtKB/TrEMBL;Acc:L8H702]	0.009113033	0.3447454	0.335632367	0.001114233
3h	ACA1_238630/clean/scf7180000084763:109469-109558:-	ACA1_238630	Phosphogluconate dehydrogenase (Decarboxylating), NAD binding domain containing protein [Source:UniProtKB/TrEMBL;Acc:L8GKQ5]	0.102481233	0.438109667	0.335628433	0.012821511
3h	ACA1_190280/clean/scf7180000084748:146909-146991:+	ACA1_190280	Ubiquitinyl hydrolase 1 [Source:UniProtKB/TrEMBL;Acc:L8GGJ9]	0.004637358	0.340238	0.335600642	6.40E-08
3h	ACA1_266370/clean/scf7180000084770:671565-671664:+	ACA1_266370	Methyltransf_11 domain-containing protein [Source:UniProtKB/TrEMBL;Acc:L8H4L5]	0.093262933	0.428724667	0.335461733	0.00174057
3h	ACA1_158440/clean/scf7180000084724:219616-219782:+	ACA1_158440	Coiled-coil domain containing 53, putative [Source:UniProtKB/TrEMBL;Acc:L8HA48]	0.010982673	0.346257933	0.33527526	8.03E-08
3h	ACA1_060990/clean/scf7180000084599:793412-793495:-	ACA1_060990	Purple acid phosphatase [Source:UniProtKB/TrEMBL;Acc:L8GZ22]	0.053792267	0.388979	0.335186733	8.47E-05
3h	ACA1_081440/clean/scf7180000084618:110143-110229:-	ACA1_081440	Alg14 family oligosaccharide biosynthesis protein [Source:UniProtKB/TrEMBL;Acc:L8HD51]	0.00831951	0.343435	0.33511549	9.31E-08
3h	ACA1_094110/clean/scf7180000084645:694650-694712:+	ACA1_094110	hypothetical protein	0.007266417	0.342342667	0.33507625	3.59E-10
3h	ACA1_060380/clean/scf7180000084599:663950-664033:-	ACA1_060380	ATG13 domain-containing protein [Source:UniProtKB/TrEMBL;Acc:L8GYW8]	0.060310833	0.395252667	0.334941833	0.000965078
3h	ACA1_173060/clean/scf7180000084741:394159-394234:-	ACA1_173060	SNARE associated Golgi protein [Source:UniProtKB/TrEMBL;Acc:L8HGL7]	0.0172175	0.352115667	0.334898167	0.000507152
3h	ACA1_323200/clean/scf7180000084467:29736-29809:-	ACA1_323200	ShKT domain-containing protein [Source:UniProtKB/TrEMBL;Acc:L8GFA8]	0	0.334823333	0.334823333	2.79E-06
3h	ACA1_097270/clean/scf7180000084645:1203414-1203483:-	ACA1_097270	Serine/threonine protein kinase [Source:UniProtKB/TrEMBL;Acc:L8GJZ3]	0	0.334820333	0.334820333	6.12E-06
3h	ACA1_365090/clean/scf7180000084641:233838-233921:+	ACA1_365090	ADPribosylglycohydrolase superfamily protein [Source:UniProtKB/TrEMBL;Acc:L8GP97]	0.045496633	0.380256667	0.334760033	5.94E-05
3h	ACA1_252450/clean/scf7180000084768:154080-154183:+	ACA1_252450	L1P family protein [Source:UniProtKB/TrEMBL;Acc:L8HAS4]	0.034928533	0.369563	0.334634467	5.50E-06
3h	ACA1_173980/clean/scf7180000084741:565611-565737:-	ACA1_173980	OTU domain-containing protein [Source:UniProtKB/TrEMBL;Acc:L8HHS8]	0.007415203	0.342017	0.334601797	5.56E-05
3h	ACA1_399930/clean/scf7180000084775:70426-70537:+	ACA1_399930	Ras subfamily protein [Source:UniProtKB/TrEMBL;Acc:L8GGR5]	0.01196133	0.346411467	0.334450137	0.000539918
3h	ACA1_296120/clean/scf7180000084777:1300741-1300872:+	ACA1_296120	hypothetical protein	0.02054835	0.354988667	0.334440317	0.001299189
3h	ACA1_068700/clean/scf7180000084603:257668-257768:+	ACA1_068700	Purple acid phosphatase [Source:UniProtKB/TrEMBL;Acc:L8HFR9]	0.000127456	0.334411367	0.334283911	1.62E-10
3h	ACA1_256650/clean/scf7180000084769:72278-72358:+	ACA1_256650	von Willebrand factor type A domain containing protein [Source:UniProtKB/TrEMBL;Acc:L8GH56]	0	0.334223267	0.334223267	0.000120573
3h	ACA1_062850/clean/scf7180000084599:1287408-1287498:+	ACA1_062850	WD_REPEATS_REGION domain-containing protein [Source:UniProtKB/TrEMBL;Acc:L8GZK3]	0.005617397	0.339766	0.334148603	9.65E-09
3h	ACA1_277750/clean/scf7180000084776:92166-92253:-	ACA1_277750	Raslike protein 1, putative [Source:UniProtKB/TrEMBL;Acc:L8H8P6]	0.003681743	0.337810033	0.33412829	1.68E-16
3h	ACA1_333270/clean/scf7180000084581:29286-29344:-	ACA1_333270	hypothetical protein	0	0.334115333	0.334115333	0.002602919
3h	ACA1_025680/clean/scf7180000084451:4613-4683:-	ACA1_025680	hypothetical protein	0.00080822	0.334873667	0.334065447	5.19E-09
3h	ACA1_063760/clean/scf7180000084599:1497103-1497186:+	ACA1_063760	PH domain-containing protein [Source:UniProtKB/TrEMBL;Acc:L8GXG1]	0.010580227	0.344356333	0.333776107	4.32E-06
3h	ACA1_078020/clean/scf7180000084614:75167-75268:-	ACA1_078020	AlbA_2 domain-containing protein [Source:UniProtKB/TrEMBL;Acc:L8GR16]	0	0.333740667	0.333740667	0.001410713
3h	ACA1_325150/clean/scf7180000084475:11025-11137:+	ACA1_325150	Rab7/RabGfamily small GTPase [Source:UniProtKB/TrEMBL;Acc:L8GPH2]	0.004739653	0.3384492	0.333709547	2.40E-08
3h	ACA1_306840/clean/scf7180000083668:19063-19166:+	ACA1_306840	RTP1_C1 domain-containing protein [Source:UniProtKB/TrEMBL;Acc:L8GQF6]	0.007575767	0.341263733	0.333687967	0.000432077
3h	ACA1_280550/clean/scf7180000084776:491824-491973:-	ACA1_280550	RSNARE, VAMP71-family [Source:UniProtKB/TrEMBL;Acc:L8H6Z7]	0.010485357	0.344002667	0.33351731	2.18E-07
3h	ACA1_381640/clean/scf7180000084712:202051-202132:+	ACA1_381640	hypothetical protein	0.049632333	0.383145333	0.333513	0.044833589
3h	ACA1_269670/clean/scf7180000084770:1012454-1012542:-	ACA1_269670	Tetratricopeptide repeat domain containing protein [Source:UniProtKB/TrEMBL;Acc:L8H2T8]	0.003786967	0.337240667	0.3334537	2.24E-06
3h	ACA1_225890/clean/scf7180000084761:14059-14132:+	ACA1_225890	hypothetical protein	0.0049075	0.338289967	0.333382467	0.000534759
3h	ACA1_355980/clean/scf7180000084728:41075-41175:+	ACA1_355980	Aa_trans domain-containing protein [Source:UniProtKB/TrEMBL;Acc:L8H7L7]	0.016470667	0.349809533	0.333338867	1.21E-05
3h	ACA1_325610/clean/scf7180000084495:50712-50792:-	ACA1_325610	Protein kinase domain containing protein [Source:UniProtKB/TrEMBL;Acc:L8H900]	0	0.333316	0.333316	4.46E-07
3h	ACA1_261280/clean/scf7180000084769:627114-627185:-	ACA1_261280	hypothetical protein	0.126391267	0.459669333	0.333278067	0.015608456
3h	ACA1_071210/clean/scf7180000084603:889848-889923:-	ACA1_071210	AAC-rich mRNA clone AAC4 protein [Source:UniProtKB/TrEMBL;Acc:L8HE90]	0.032441567	0.365652667	0.3332111	4.95E-05
3h	ACA1_252210/clean/scf7180000084768:145823-145907:+	ACA1_252210	Protein kinase domain-containing protein [Source:UniProtKB/TrEMBL;Acc:L8HAM0]	0.004019404	0.337214333	0.333194929	2.96E-05
3h	ACA1_208430/clean/scf7180000084757:191078-191175:+	ACA1_208430	hypothetical protein	0.098458	0.431574333	0.333116333	0.002587227
3h	ACA1_149000/clean/scf7180000084715:48078-48159:+	ACA1_149000	Deacetylase sirtuin-type domain-containing protein [Source:UniProtKB/TrEMBL;Acc:L8HD96]	0.446021667	0.779075	0.333053333	0.020009629
3h	ACA1_060930/clean/scf7180000084599:782291-782460:+	ACA1_060930	RFX-type winged-helix domain-containing protein [Source:UniProtKB/TrEMBL;Acc:L8GWH8]	0.042069233	0.374851233	0.332782	0.000258361
3h	ACA1_128500/clean/scf7180000084697:73108-73227:-	ACA1_128500	UBIQUITIN_CONJUGAT_2 domain-containing protein [Source:UniProtKB/TrEMBL;Acc:L8GWJ5]	0.01258411	0.345244667	0.332660557	2.37E-07
3h	ACA1_288310/clean/scf7180000084777:166872-166972:-	ACA1_288310	hypothetical protein	0.001328497	0.333973667	0.33264517	6.99E-07
3h	ACA1_063460/clean/scf7180000084599:1414064-1414133:-	ACA1_063460	AAA domain-containing protein [Source:UniProtKB/TrEMBL;Acc:L8GXD1]	0.0228074	0.355333667	0.332526267	1.23E-06
3h	ACA1_092600/clean/scf7180000084645:363984-364061:-	ACA1_092600	NLPC_P60 domain-containing protein [Source:UniProtKB/TrEMBL;Acc:L8GIC1]	0.005274356	0.337718833	0.332444477	2.03E-07
3h	ACA1_015210/clean/scf7180000083783:10085-10194:-	ACA1_015210	MYND-type domain-containing protein [Source:UniProtKB/TrEMBL;Acc:L8GGA6]	0.0266393	0.359031333	0.332392033	3.63E-05
3h	ACA1_291770/clean/scf7180000084777:834596-834683:-	ACA1_291770	Alpha-type protein kinase domain-containing protein [Source:UniProtKB/TrEMBL;Acc:L8HL18]	0.01318335	0.345532333	0.332348983	8.38E-05
3h	ACA1_173830/clean/scf7180000084741:530355-530464:+	ACA1_173830	Methylisocitrate lyase, putative [Source:UniProtKB/TrEMBL;Acc:L8HHR5]	0.017176253	0.349462333	0.33228608	3.72E-06
3h	ACA1_247140/clean/scf7180000084763:630109-630175:+	ACA1_247140	hypothetical protein	0.016629867	0.348880333	0.332250467	2.44E-05
3h	ACA1_178540/clean/scf7180000084743:255602-255681:-	ACA1_178540	Ubiquitin carboxyl-terminal hydrolase [Source:UniProtKB/TrEMBL;Acc:L8GT72]	0.045514367	0.377734	0.332219633	5.29E-05
3h	ACA1_036890/clean/scf7180000084567:6541-6603:+	ACA1_036890	Ras subfamily protein [Source:UniProtKB/TrEMBL;Acc:L8H0T2]	0.030781647	0.362659333	0.331877687	5.00E-05
3h	ACA1_382620/clean/scf7180000084721:86613-86695:+	ACA1_382620	SERPIN domain-containing protein [Source:UniProtKB/TrEMBL;Acc:L8GV22]	0.007909433	0.339725333	0.3318159	1.32E-06
3h	ACA1_243400/clean/scf7180000084763:333500-333579:+	ACA1_243400	hypothetical protein	0.072725567	0.404534333	0.331808767	0.000568144
3h	ACA1_035420/clean/scf7180000084564:46156-46285:-	ACA1_035420	Carrier superfamily protein [Source:UniProtKB/TrEMBL;Acc:L8HAE9]	0.042705167	0.374475	0.331769833	0.000478727
3h	ACA1_192610/clean/scf7180000084749:139452-139594:+	ACA1_192610	Hypothetical protein	0.077401467	0.409012	0.331610533	0.002127521
3h	ACA1_206370/clean/scf7180000084757:17397-17471:+	ACA1_206370	MOSC domain-containing protein [Source:UniProtKB/TrEMBL;Acc:L8GY21]	0.008377687	0.339948	0.331570313	1.13E-06
3h	ACA1_280610/clean/scf7180000084776:507473-507561:+	ACA1_280610	C2H2-type domain-containing protein [Source:UniProtKB/TrEMBL;Acc:L8H702]	0	0.331540833	0.331540833	0.000392127
3h	ACA1_068350/clean/scf7180000084603:178101-178173:+	ACA1_068350	hypothetical protein	0.002633397	0.334146567	0.33151317	1.79E-08
3h	ACA1_163990/clean/scf7180000084729:125047-125106:-	ACA1_163990	Glycosyl hydrolases family 32 superfamily protein [Source:UniProtKB/TrEMBL;Acc:L8GRF2]	0.063518533	0.394651667	0.331133133	0.00051599
3h	ACA1_232830/clean/scf7180000084762:84122-84216:-	ACA1_232830	Surfeit locus protein 5 [Source:UniProtKB/TrEMBL;Acc:L8H3C8]	0.001949317	0.333045667	0.33109635	2.43E-07
3h	ACA1_167020/clean/scf7180000084736:56119-56216:+	ACA1_167020	EF-hand domain-containing protein [Source:UniProtKB/TrEMBL;Acc:L8H0U0]	0.00375944	0.334740033	0.330980593	3.76E-07
3h	ACA1_170940/clean/scf7180000084741:19596-19670:+	ACA1_170940	Proteasome subunit alpha type [Source:UniProtKB/TrEMBL;Acc:L8HH61]	0.000901186	0.3318027	0.330901514	5.49E-12
3h	ACA1_290100/clean/scf7180000084777:524496-524576:+	ACA1_290100	Kri1_C domain-containing protein [Source:UniProtKB/TrEMBL;Acc:L8HLH0]	0.00983338	0.340687433	0.330854053	4.04E-05
3h	ACA1_040750/clean/scf7180000084572:74681-74777:+	ACA1_040750	Transporter, permease [Source:UniProtKB/TrEMBL;Acc:L8GZS4]	0.000234536	0.3310669	0.330832364	3.85E-06
3h	ACA1_293710/clean/scf7180000084777:965243-965355:+	ACA1_293710	F-box domain-containing protein [Source:UniProtKB/TrEMBL;Acc:L8HJM5]	0.019225867	0.349978667	0.3307528	1.19E-05
3h	ACA1_198480/clean/scf7180000084753:125119-125235:+	ACA1_198480	Transcription factor III family protein [Source:UniProtKB/TrEMBL;Acc:L8H324]	0.042595333	0.373247667	0.330652333	0.000371764
3h	ACA1_237860/clean/scf7180000084763:33441-33566:+	ACA1_237860	Glycerophosphodiester phosphodiesterase family protein [Source:UniProtKB/TrEMBL;Acc:L8GKN2]	0.000287394	0.3309322	0.330644806	2.37E-08
3h	ACA1_262100/clean/scf7180000084770:14286-14357:+	ACA1_262100	JmjC domain-containing protein [Source:UniProtKB/TrEMBL;Acc:L8H1V0]	0.005023647	0.335536	0.330512353	5.88E-06
3h	ACA1_301690/clean/scf7180000083222:18938-19055:-	ACA1_301690	MoCF_biosynth domain-containing protein [Source:UniProtKB/TrEMBL;Acc:L8HJW0]	0.001963487	0.332370333	0.330406847	4.76E-08
3h	ACA1_039190/clean/scf7180000084569:18227-18322:+	ACA1_039190	ANK_REP_REGION domain-containing protein [Source:UniProtKB/TrEMBL;Acc:L8HGQ0]	0.028571433	0.358973667	0.330402233	0.001769585
3h	ACA1_189640/clean/scf7180000084748:70191-70262:-	ACA1_189640	Bifunctional aspartate kinase/diaminopimelate decarboxylase protein [Source:UniProtKB/TrEMBL;Acc:L8GEY3]	0.003965147	0.3343275	0.330362353	1.10E-06
3h	ACA1_261390/clean/scf7180000084769:657038-657158:+	ACA1_261390	BTB domain-containing protein [Source:UniProtKB/TrEMBL;Acc:L8GFT7]	0.014284677	0.344620233	0.330335557	1.03E-05
3h	ACA1_259490/clean/scf7180000084769:353122-353209:+	ACA1_259490	TPR_REGION domain-containing protein [Source:UniProtKB/TrEMBL;Acc:L8GEZ4]	0	0.330303333	0.330303333	3.78E-07
3h	ACA1_180160/clean/scf7180000084744:34838-35013:+	ACA1_180160	hypothetical protein	0.018380497	0.348671633	0.330291137	2.40E-05
3h	ACA1_060370/clean/scf7180000084599:661403-661631:-	ACA1_060370	hypothetical protein	0.133009533	0.463282667	0.330273133	0.004168327
3h	ACA1_169710/clean/scf7180000084739:56275-56355:+	ACA1_169710	SHSP domain-containing protein [Source:UniProtKB/TrEMBL;Acc:L8HHU0]	0.008205043	0.338278333	0.33007329	1.17E-07
3h	ACA1_117080/clean/scf7180000084669:908378-908452:+	ACA1_117080	PALP domain-containing protein [Source:UniProtKB/TrEMBL;Acc:L8H4Y6]	0.0035983	0.333614	0.3300157	0.000202994
3h	ACA1_217150/clean/scf7180000084758:527957-528044:-	ACA1_217150	Protein kinase domain-containing protein [Source:UniProtKB/TrEMBL;Acc:L8GR83]	0.053208733	0.383213767	0.330005033	0.001619449
3h	ACA1_253320/clean/scf7180000084768:272650-272717:+	ACA1_253320	hypothetical protein	0.0074074	0.337298	0.3298906	2.66E-05
3h	ACA1_117240/clean/scf7180000084669:956730-956816:-	ACA1_117240	Non-specific serine/threonine protein kinase [Source:UniProtKB/TrEMBL;Acc:L8H707]	0	0.329855333	0.329855333	4.75E-07
3h	ACA1_100610/clean/scf7180000084648:131645-131728:-	ACA1_100610	Serine/threonine kinase [Source:UniProtKB/TrEMBL;Acc:L8GK64]	0.007171167	0.336999	0.329827833	2.30E-06
3h	ACA1_015310/clean/scf7180000083794:3600-3675:-	ACA1_015310	RhoGEF domain-containing protein [Source:UniProtKB/TrEMBL;Acc:L8GHA4]	0	0.329774633	0.329774633	5.82E-05
3h	ACA1_367290/clean/scf7180000084641:589789-589890:-	ACA1_367290	hypothetical protein	0.002020672	0.331751333	0.329730661	4.02E-08
3h	ACA1_254920/clean/scf7180000084768:451692-451820:+	ACA1_254920	von Willebrand factor type A domain containing protein [Source:UniProtKB/TrEMBL;Acc:L8HA47]	0.009561573	0.33923	0.329668427	1.45E-08
3h	ACA1_171960/clean/scf7180000084741:208318-208384:-	ACA1_171960	F-box domain-containing protein [Source:UniProtKB/TrEMBL;Acc:L8HHE3]	0.0463282	0.375685	0.3293568	0.000191564
3h	ACA1_209120/clean/scf7180000084757:276405-276512:+	ACA1_209120	Leucine rich repeat domain containing protein [Source:UniProtKB/TrEMBL;Acc:L8GYC0]	0.00888215	0.33823	0.32934785	9.32E-06
3h	ACA1_255900/clean/scf7180000084768:576990-577198:+	ACA1_255900	DEP domain-containing protein [Source:UniProtKB/TrEMBL;Acc:L8HA90]	0.000618247	0.32987	0.329251753	6.97E-10
3h	ACA1_010610/clean/scf7180000083497:7736-7815:-	ACA1_010610	Fbox domain and Ankyrin repeat containing protein [Source:UniProtKB/TrEMBL;Acc:L8GTH9]	0.004005333	0.333141333	0.329136	6.97E-06
3h	ACA1_320820/clean/scf7180000084416:29287-29395:+	ACA1_320820	hypothetical protein	0.013287733	0.342419667	0.329131933	7.43E-14
3h	ACA1_018110/clean/scf7180000083885:9899-9971:-	ACA1_018110	hypothetical protein	0.0292169	0.358153667	0.328936767	3.58E-05
3h	ACA1_201480/clean/scf7180000084753:560737-560807:+	ACA1_201480	PKS_ER domain-containing protein [Source:UniProtKB/TrEMBL;Acc:L8H3J6]	0.0192206	0.348147333	0.328926733	7.26E-06
3h	ACA1_339740/clean/scf7180000084619:15983-16085:+	ACA1_339740	Serine/threonine protein kinase [Source:UniProtKB/TrEMBL;Acc:L8GLZ6]	0	0.328844667	0.328844667	4.58E-07
3h	ACA1_116200/clean/scf7180000084669:725438-725519:+	ACA1_116200	WASH_WAHD domain-containing protein [Source:UniProtKB/TrEMBL;Acc:L8H3U1]	0.058565567	0.387368333	0.328802767	0.000417693
3h	ACA1_368080/clean/scf7180000084664:48062-48125:-	ACA1_368080	hypothetical protein	0	0.328768333	0.328768333	0.0021101
3h	ACA1_292890/clean/scf7180000084777:929125-929280:-	ACA1_292890	Ribosomal_S17_N domain-containing protein [Source:UniProtKB/TrEMBL;Acc:L8HL46]	0.002607107	0.331226333	0.328619227	5.80E-21
3h	ACA1_275430/clean/scf7180000084774:15857-15948:-	ACA1_275430	unspecified product	0.012222233	0.340818	0.328595767	5.42E-05
3h	ACA1_278160/clean/scf7180000084776:181450-181523:-	ACA1_278160	hypothetical protein	0.041286567	0.369860333	0.328573767	6.90E-06
3h	ACA1_074080/clean/scf7180000084605:18489-18575:-	ACA1_074080	hypothetical protein	0.00662351	0.3351218	0.32849829	4.89E-06
3h	ACA1_308770/clean/scf7180000083743:16896-16961:-	ACA1_308770	Fumarylacetoacetase [Source:UniProtKB/TrEMBL;Acc:L8HKF9]	0.00096371	0.329332333	0.328368623	1.32E-11
3h	ACA1_044720/clean/scf7180000084576:36793-36894:-	ACA1_044720	F-box domain-containing protein [Source:UniProtKB/TrEMBL;Acc:L8GZR9]	0.00616504	0.334503	0.32833796	1.24E-06
3h	ACA1_229540/clean/scf7180000084761:271858-271980:-	ACA1_229540	HD phosphohydrolase, putative [Source:UniProtKB/TrEMBL;Acc:L8HB82]	0.128201	0.456537	0.328336	0.002663929
3h	ACA1_164760/clean/scf7180000084729:267583-267653:+	ACA1_164760	hypothetical protein	0.0112837	0.339526333	0.328242633	2.45E-06
3h	ACA1_247250/clean/scf7180000084763:656124-656212:-	ACA1_247250	MBD domain-containing protein [Source:UniProtKB/TrEMBL;Acc:L8GMS1]	0.00428396	0.332525	0.32824104	3.72E-06
3h	ACA1_169100/clean/scf7180000084739:22012-22080:+	ACA1_169100	Dual specificity phosphatase, catalytic domain containing protein [Source:UniProtKB/TrEMBL;Acc:L8HG79]	0.00869155	0.336876667	0.328185117	6.61E-09
3h	ACA1_364280/clean/scf7180000084641:71890-71968:+	ACA1_364280	Sporulation proteinrelated, putative [Source:UniProtKB/TrEMBL;Acc:L8GLJ0]	0.032834533	0.3610141	0.328179567	3.19E-05
3h	ACA1_085170/clean/scf7180000084632:62727-62809:+	ACA1_085170	TPR_REGION domain-containing protein [Source:UniProtKB/TrEMBL;Acc:L8HM05]	0.0015873	0.329707	0.3281197	1.66E-07
3h	ACA1_091930/clean/scf7180000084645:230656-230729:-	ACA1_091930	hypothetical protein	0.336881333	0.664991667	0.328110333	0.013441753
3h	ACA1_138930/clean/scf7180000084705:26756-26823:+	ACA1_138930	Protein kinase domain containing protein [Source:UniProtKB/TrEMBL;Acc:L8GQ12]	0.11882	0.446865	0.328045	0.010631439
3h	ACA1_067770/clean/scf7180000084603:12696-12777:+	ACA1_067770	CBS domain containing protein [Source:UniProtKB/TrEMBL;Acc:L8HEJ6]	0.01751881	0.345511667	0.327992857	1.32E-06
3h	ACA1_072660/clean/scf7180000084603:1246556-1246616:+	ACA1_072660	hypothetical protein	0.047619	0.375600333	0.327981333	0.036417083
3h	ACA1_179970/clean/scf7180000084744:11779-11912:+	ACA1_179970	PPM-type phosphatase domain-containing protein [Source:UniProtKB/TrEMBL;Acc:L8GF73]	0.242324333	0.570303667	0.327979333	0.009505217
3h	ACA1_103170/clean/scf7180000084653:12172-12259:+	ACA1_103170	CBS domain containing protein [Source:UniProtKB/TrEMBL;Acc:L8GCP7]	0.039842733	0.367681	0.327838267	0.002019729
3h	ACA1_216300/clean/scf7180000084758:463495-463632:+	ACA1_216300	Ras family protein [Source:UniProtKB/TrEMBL;Acc:L8GR60]	0.008492567	0.336239767	0.3277472	0.00023152
3h	ACA1_266830/clean/scf7180000084770:725067-725153:-	ACA1_266830	ATP-dependent DNA helicase [Source:UniProtKB/TrEMBL;Acc:L8H4N3]	0.229127	0.556863	0.327736	0.034738603
3h	ACA1_089040/clean/scf7180000084639:304817-304890:+	ACA1_089040	UBA/TS-N domain containing protein [Source:UniProtKB/TrEMBL;Acc:L8GUR2]	0.00196992	0.329596	0.32762608	1.72E-07
3h	ACA1_221060/clean/scf7180000084758:989055-989126:-	ACA1_221060	Leucine rich repeat domain containing protein [Source:UniProtKB/TrEMBL;Acc:L8GQQ4]	0.010533153	0.338069933	0.32753678	0.001283812
3h	ACA1_036530/clean/scf7180000084565:132655-132768:+	ACA1_036530	CRT-like domain-containing protein [Source:UniProtKB/TrEMBL;Acc:L8HCN5]	0.0294245	0.356933333	0.327508833	1.06E-06
3h	ACA1_025540/clean/scf7180000084448:8002-8083:+	ACA1_025540	hypothetical protein	0.013119037	0.340480333	0.327361297	2.39E-05

3h	ACA1_258290/clean/scf7180000084769:221792-221855:+	ACA1_258290	hypothetical protein	0.050478967	0.377805	0.327326033	0.000211595
3h	ACA1_024200/clean/scf7180000084441:14349-14438:-	ACA1_024200	WD_REPEATS_REGION domain-containing protein [Source:UniProtKB/TrEMBL;Acc:L8GKS0]	0	0.327275333	0.327275333	7.13E-06
3h	ACA1_365010/clean/scf7180000084641:202533-202597:-	ACA1_365010	hypothetical protein	0.204823	0.532035	0.327212	0.007190955
3h	ACA1_111550/clean/scf7180000084669:16161-16253:-	ACA1_111550	alpha-1,2-Mannosidase [Source:UniProtKB/TrEMBL;Acc:L8H589]	0.015542967	0.3426455	0.327102533	0.000302468
3h	ACA1_088090/clean/scf7180000084639:156656-156714:-	ACA1_088090	Retinol dehydrogenase 14 (All-trans and 9-cis) family protein [Source:UniProtKB/TrEMBL;Acc:L8GTX6]	0.019360267	0.346433333	0.327073067	0.001209188
3h	ACA1_171870/clean/scf7180000084741:182200-182269:-	ACA1_171870	Leucine rich repeat domain containing protein [Source:UniProtKB/TrEMBL;Acc:L8HIV4]	0.028317033	0.355373867	0.327056833	0.016738889
3h	ACA1_312180/clean/scf7180000083781:14136-14240:-	ACA1_312180	Fn3_like domain-containing protein [Source:UniProtKB/TrEMBL;Acc:L8GRI1]	0.005068723	0.332021367	0.326952643	2.33E-08
3h	ACA1_143290/clean/scf7180000084709:21969-22097:-	ACA1_143290	F_actin_bund_C domain-containing protein [Source:UniProtKB/TrEMBL;Acc:L8HEL3]	0.012484067	0.339413	0.326928933	1.66E-08
3h	ACA1_307820/clean/scf7180000083687:20308-20395:-	ACA1_307820	BSD domain-containing protein [Source:UniProtKB/TrEMBL;Acc:L8HBM7]	0.00204499	0.328820667	0.326775677	1.41E-07
3h	ACA1_149410/clean/scf7180000084715:144991-145103:+	ACA1_149410	Lectin_legB domain-containing protein [Source:UniProtKB/TrEMBL;Acc:L8HDD6]	0.00329337	0.330050333	0.326756963	8.32E-06
3h	ACA1_370300/clean/scf7180000084664:490379-490456:-	ACA1_370300	hypothetical protein	0	0.326697367	0.326697367	2.68E-09
3h	ACA1_221620/clean/scf7180000084760:6445-6539:-	ACA1_221620	Purple acid phosphatase [Source:UniProtKB/TrEMBL;Acc:L8GV66]	0.001579684	0.328271633	0.326691949	6.59E-08
3h	ACA1_187740/clean/scf7180000084747:87434-87552:-	ACA1_187740	hypothetical protein	0.188031	0.514643	0.326612	0.002644747
3h	ACA1_041920/clean/scf7180000084573:20123-20225:+	ACA1_041920	Deoxyribonuclease II, putative [Source:UniProtKB/TrEMBL;Acc:L8GV23]	0.00182452	0.328428	0.32660348	4.04E-08
3h	ACA1_204500/clean/scf7180000084754:298431-298516:+	ACA1_204500	hypothetical protein	0.01581302	0.342306333	0.326493313	1.09E-06
3h	ACA1_267280/clean/scf7180000084770:842495-842635:+	ACA1_267280	Coatomer subunit gamma [Source:UniProtKB/TrEMBL;Acc:L8H1U2]	0.00187266	0.328256667	0.326384007	7.79E-08
3h	ACA1_138520/clean/scf7180000084703:160596-160691:+	ACA1_138520	Phospholipase B-like [Source:UniProtKB/TrEMBL;Acc:L8GZK2]	0.000595693	0.326914	0.326318307	7.17E-09
3h	ACA1_155670/clean/scf7180000084722:212265-212357:+	ACA1_155670	SPRY domain containing protein [Source:UniProtKB/TrEMBL;Acc:L8H063]	0.005956803	0.332275	0.326318197	7.72E-07
3h	ACA1_058840/clean/scf7180000084599:392263-392344:-	ACA1_058840	HATPase_c domain-containing protein [Source:UniProtKB/TrEMBL;Acc:L8GXB7]	0.002582144	0.328868333	0.32628619	9.74E-12
3h	ACA1_269270/clean/scf7180000084770:962462-962615:+	ACA1_269270	Protein phosphatase [Source:UniProtKB/TrEMBL;Acc:L8H4X6]	0.022033987	0.34822	0.326186013	3.52E-05
3h	ACA1_209860/clean/scf7180000084757:340620-340752:-	ACA1_209860	Protein kinase domain-containing protein [Source:UniProtKB/TrEMBL;Acc:L8GYD8]	0.031166933	0.357263	0.326096067	1.33E-05
3h	ACA1_139840/clean/scf7180000084707:9627-9741:+	ACA1_139840	Receptor mediated endocytosis family member (Rme1), putative [Source:UniProtKB/TrEMBL;Acc:L8GGP3]	0.00419324	0.330235767	0.326042527	8.37E-09
3h	ACA1_071580/clean/scf7180000084603:998403-998482:-	ACA1_071580	GOLD domain-containing protein [Source:UniProtKB/TrEMBL;Acc:L8HGH0]	0	0.326001	0.326001	4.11E-07
3h	ACA1_074670/clean/scf7180000084608:61534-61607:-	ACA1_074670	Arginase [Source:UniProtKB/TrEMBL;Acc:L8HFF6]	0.0216408	0.347606333	0.325965533	2.26E-06
3h	ACA1_233410/clean/scf7180000084762:136540-136622:+	ACA1_233410	NFX1type zinc finger-containing protein 1, putative [Source:UniProtKB/TrEMBL;Acc:L8H2G5]	0.0199791	0.345845667	0.325866567	1.55E-07
3h	ACA1_156030/clean/scf7180000084723:5781-5867:+	ACA1_156030	hypothetical protein	0	0.3257961	0.3257961	7.13E-06
3h	ACA1_332300/clean/scf7180000084579:8773-8897:+	ACA1_332300	hypothetical protein	0	0.3257068	0.3257068	7.15E-05
3h	ACA1_227720/clean/scf7180000084761:124284-124482:-	ACA1_227720	hypothetical protein	0.029452133	0.355041	0.325588867	9.77E-08
3h	ACA1_066870/clean/scf7180000084600:67221-67435:-	ACA1_066870	Glucose-6-phosphate isomerase [Source:UniProtKB/TrEMBL;Acc:L8GRA3]	0.0013038	0.326793	0.3254892	8.20E-08
3h	ACA1_380810/clean/scf7180000084712:92486-92591:-	ACA1_380810	Ribosomal_L11 domain-containing protein [Source:UniProtKB/TrEMBL;Acc:L8GNK9]	0.0464899	0.371966	0.3254761	0.000847641
3h	ACA1_071440/clean/scf7180000084603:953480-953643:-	ACA1_071440	Nascent polypeptide-associated complex subunit beta [Source:UniProtKB/TrEMBL;Acc:L8HE68]	0.00897973	0.334405333	0.325425603	8.49E-10
3h	ACA1_301230/clean/scf7180000083203:5881-6002:-	ACA1_301230	hypothetical protein	0.012673227	0.337865667	0.32519244	9.32E-05
3h	ACA1_171960/clean/scf7180000084741:209021-209110:-	ACA1_171960	F-box domain-containing protein [Source:UniProtKB/TrEMBL;Acc:L8HHE3]	0.001177263	0.326342	0.325164737	2.06E-06
3h	ACA1_036340/clean/scf7180000084565:81580-81680:+	ACA1_036340	Serine hydroxymethyltransferase [Source:UniProtKB/TrEMBL;Acc:L8HBL5]	0.00209745	0.327203333	0.325105883	1.34E-08
3h	ACA1_322090/clean/scf7180000084459:3302-3408:-	ACA1_322090	Myosin head (Motor domain) domain containing protein [Source:UniProtKB/TrEMBL;Acc:L8GN06]	0.0169106	0.341997	0.3250864	1.51E-06
3h	ACA1_060260/clean/scf7180000084599:630262-630400:-	ACA1_060260	RFX-type winged-helix domain-containing protein [Source:UniProtKB/TrEMBL;Acc:L8GVN2]	0.036958967	0.361945333	0.324986367	0.004103177
3h	ACA1_038530/clean/scf7180000084568:172332-172417:-	ACA1_038530	AMP-binding domain-containing protein [Source:UniProtKB/TrEMBL;Acc:L8GLT0]	0	0.324911	0.324911	0.000184176
3h	ACA1_165210/clean/scf7180000084732:8585-8677:-	ACA1_165210	Ras subfamily protein [Source:UniProtKB/TrEMBL;Acc:L8HGF1]	0.001720867	0.326578067	0.3248572	2.23E-08
3h	ACA1_127620/clean/scf7180000084696:35972-36040:+	ACA1_127620	Fe2OG dioxygenase domain-containing protein [Source:UniProtKB/TrEMBL;Acc:L8GGE8]	0.003169163	0.3279781	0.324808937	9.38E-05
3h	ACA1_234240/clean/scf7180000084762:242604-242724:+	ACA1_234240	Xpo1 domain-containing protein [Source:UniProtKB/TrEMBL;Acc:L8H3I6]	0.01483886	0.339506667	0.324667807	2.07E-05
3h	ACA1_096180/clean/scf7180000084645:957617-957724:+	ACA1_096180	BHLH domain-containing protein [Source:UniProtKB/TrEMBL;Acc:L8GJN8]	0.005649733	0.3303007	0.324650967	8.95E-05
3h	ACA1_230570/clean/scf7180000084761:379563-379648:-	ACA1_230570	DUF4139 domain-containing protein [Source:UniProtKB/TrEMBL;Acc:L8H928]	0.005399781	0.330038667	0.324638886	1.71E-06
3h	ACA1_264380/clean/scf7180000084770:255387-255477:-	ACA1_264380	S-formylglutathione hydrolase [Source:UniProtKB/TrEMBL;Acc:L8H1Z7]	0.003333857	0.3279092	0.324575343	3.49E-08
3h	ACA1_209120/clean/scf7180000084757:276070-276146:+	ACA1_209120	Leucine rich repeat domain containing protein [Source:UniProtKB/TrEMBL;Acc:L8GYC0]	0.010799953	0.335361	0.324561047	2.74E-05
3h	ACA1_052690/clean/scf7180000084594:152920-152987:+	ACA1_052690	Rho-GAP domain-containing protein [Source:UniProtKB/TrEMBL;Acc:L8H797]	0.086990767	0.411493333	0.324502567	0.01081427
3h	ACA1_144250/clean/scf7180000084709:185569-185706:-	ACA1_144250	SH2 domain containing protein [Source:UniProtKB/TrEMBL;Acc:L8HEU2]	0.001159413	0.325634967	0.324475554	1.43E-07
3h	ACA1_167330/clean/scf7180000084737:6585-6708:+	ACA1_167330	hypothetical protein	0.1301304	0.454559333	0.324428933	0.010144477
3h	ACA1_214220/clean/scf7180000084758:199152-199233:-	ACA1_214220	hypothetical protein	0.1969697	0.521371667	0.324401967	0.020152317
3h	ACA1_291910/clean/scf7180000084777:849656-849732:-	ACA1_291910	RING-CH-type domain-containing protein [Source:UniProtKB/TrEMBL;Acc:L8HJI4]	0.0191572	0.343447833	0.324290633	0.000451477
3h	ACA1_264230/clean/scf7180000084770:215636-215713:+	ACA1_264230	ATP/GTPbinding-like protein [Source:UniProtKB/TrEMBL;Acc:L8H212]	0.003408883	0.327678333	0.32426945	1.40E-05
3h	ACA1_252490/clean/scf7180000084768:161575-161673:-	ACA1_252490	Protein kinase domain-containing protein [Source:UniProtKB/TrEMBL;Acc:L8H9V3]	0.006283686	0.330536667	0.32425298	5.97E-06
3h	ACA1_382390/clean/scf7180000084721:50822-50900:+	ACA1_382390	GH10 domain-containing protein [Source:UniProtKB/TrEMBL;Acc:L8GUU4]	0.018518533	0.342742	0.324223467	0.020412902
3h	ACA1_330350/clean/scf7180000084566:44409-44484:-	ACA1_330350	SEP domain containing protein [Source:UniProtKB/TrEMBL;Acc:L8GJV6]	0.005889517	0.330049	0.324159483	4.78E-08
3h	ACA1_085170/clean/scf7180000084632:62185-62267:+	ACA1_085170	TPR_REGION domain-containing protein [Source:UniProtKB/TrEMBL;Acc:L8HM05]	0	0.3241078	0.3241078	1.45E-08
3h	ACA1_195840/clean/scf7180000084752:33298-33389:+	ACA1_195840	EGFlike domain containing protein [Source:UniProtKB/TrEMBL;Acc:L8HET8]	0.002624673	0.326729	0.324104327	1.04E-05
3h	ACA1_320790/clean/scf7180000084416:21487-21767:-	ACA1_320790	RUN domain-containing protein [Source:UniProtKB/TrEMBL;Acc:L8GTR9]	0.116743067	0.440841333	0.324098267	0.000903931
3h	ACA1_389550/clean/scf7180000084733:282088-282162:+	ACA1_389550	hypothetical protein	0.083687533	0.407784	0.324096467	0.003436494
3h	ACA1_320100/clean/scf7180000084352:6987-7049:+	ACA1_320100	VWFA domain-containing protein [Source:UniProtKB/TrEMBL;Acc:L8H729]	0.000912947	0.324896133	0.323983187	1.71E-05
3h	ACA1_389690/clean/scf7180000084733:309282-309376:-	ACA1_389690	RBR-type E3 ubiquitin transferase [Source:UniProtKB/TrEMBL;Acc:L8GEU0]	0.009670877	0.3336458	0.323974923	1.14E-06
3h	ACA1_092030/clean/scf7180000084645:251922-252002:-	ACA1_092030	Nudix hydrolase domain-containing protein [Source:UniProtKB/TrEMBL;Acc:L8GIW6]	0.0084361	0.332405667	0.323969567	0.0004072
3h	ACA1_351920/clean/scf7180000084695:34008-34082:+	ACA1_351920	VWFA domain-containing protein [Source:UniProtKB/TrEMBL;Acc:L8GE58]	0.027356323	0.351250667	0.323894343	0.000211738
3h	ACA1_048850/clean/scf7180000084583:685-776:-	ACA1_048850	hypothetical protein	0.0119239	0.3358	0.3238761	6.91E-07
3h	ACA1_108210/clean/scf7180000084661:12839-12929:-	ACA1_108210	PH domain containing protein [Source:UniProtKB/TrEMBL;Acc:L8GD50]	0.018783527	0.3426453	0.323861773	0.000903453
3h	ACA1_246490/clean/scf7180000084763:555005-555103:-	ACA1_246490	Aldo_ket_red domain-containing protein [Source:UniProtKB/TrEMBL;Acc:L8GMP1]	0.0249371	0.348687	0.3237499	0.003844195
3h	ACA1_157260/clean/scf7180000084724:24782-24899:+	ACA1_157260	hypothetical protein	0.020035133	0.343590167	0.323555033	1.20E-05
3h	ACA1_006110/clean/scf7180000083029:43274-43520:+	ACA1_006110	GGACT domain-containing protein [Source:UniProtKB/TrEMBL;Acc:L8HGX8]	0.158720033	0.482131	0.323410967	0.007814348
3h	ACA1_292830/clean/scf7180000084777:910756-910822:+	ACA1_292830	CULLIN_2 domain-containing protein [Source:UniProtKB/TrEMBL;Acc:L8HJK9]	0.004026833	0.327417667	0.323390833	1.61E-06
3h	ACA1_288630/clean/scf7180000084777:253569-253646:-	ACA1_288630	Mediator of RNA polymerase II transcription subunit 20 [Source:UniProtKB/TrEMBL;Acc:L8HIV7]	0.0168774	0.340164267	0.323286867	0.000170683
3h	ACA1_175760/clean/scf7180000084741:1053427-1053544:-	ACA1_175760	Phosphoglycerate mutase family domain containing protein [Source:UniProtKB/TrEMBL;Acc:L8HH51]	0.044156033	0.367432667	0.323276633	0.000239905
3h	ACA1_377930/clean/scf7180000084711:146238-146345:-	ACA1_377930	hypothetical protein	0.026456567	0.349652	0.323195433	0.000166593
3h	ACA1_050510/clean/scf7180000084589:109705-109774:+	ACA1_050510	4HBT domain-containing protein [Source:UniProtKB/TrEMBL;Acc:L8H578]	0.008026777	0.330970367	0.32294359	5.55E-07
3h	ACA1_012300/clean/scf7180000083740:10372-10445:-	ACA1_012300	hypothetical protein	0.012376	0.335115267	0.322739267	8.99E-05
3h	ACA1_224860/clean/scf7180000084760:256434-256511:+	ACA1_224860	Myosin head (Motor domain) domain containing protein [Source:UniProtKB/TrEMBL;Acc:L8GSY9]	0	0.322710667	0.322710667	0.001085697
3h	ACA1_028150/clean/scf7180000084472:8636-8711:-	ACA1_028150	hypothetical protein	0.010662183	0.3333617	0.322699517	6.68E-07
3h	ACA1_060920/clean/scf7180000084599:780888-780993:-	ACA1_060920	MFS domain-containing protein [Source:UniProtKB/TrEMBL;Acc:L8GVT5]	0	0.3226184	0.3226184	1.63E-05
3h	ACA1_080550/clean/scf7180000084618:32435-32524:+	ACA1_080550	FAD binding domain containing protein [Source:UniProtKB/TrEMBL;Acc:L8HBU6]	0.024146093	0.346713	0.322566907	2.88E-05
3h	ACA1_174180/clean/scf7180000084741:633984-634065:+	ACA1_174180	hypothetical protein	0.012279337	0.334799667	0.32252033	3.87E-05
3h	ACA1_389150/clean/scf7180000084733:212333-212451:+	ACA1_389150	Tasp1 protein [Source:UniProtKB/TrEMBL;Acc:L8GEC7]	0.018649333	0.3411524	0.322503067	4.23E-05
3h	ACA1_379060/clean/scf7180000084711:272363-272453:+	ACA1_379060	Protein kinase domain-containing protein [Source:UniProtKB/TrEMBL;Acc:L8GRW8]	0.007018827	0.3293809	0.322362073	2.27E-08
3h	ACA1_173890/clean/scf7180000084741:541886-541987:-	ACA1_173890	Ubiquitin carboxyl-terminal hydrolase [Source:UniProtKB/TrEMBL;Acc:L8HK15]	0.005112007	0.327470667	0.32235866	5.54E-07
3h	ACA1_368560/clean/scf7180000084664:128461-128572:-	ACA1_368560	Protein kinase domain-containing protein [Source:UniProtKB/TrEMBL;Acc:L8GY18]	0.027946867	0.350286333	0.322339467	4.21E-06
3h	ACA1_072810/clean/scf7180000084603:1302045-1302194:+	ACA1_072810	Aha1_N domain-containing protein [Source:UniProtKB/TrEMBL;Acc:L8HG06]	0.002767803	0.325033533	0.32226573	8.62E-13
3h	ACA1_349650/clean/scf7180000084690:28511-28611:-	ACA1_349650	Glyco_trans_2-like domain-containing protein [Source:UniProtKB/TrEMBL;Acc:L8GK12]	0	0.322260333	0.322260333	5.97E-06
3h	ACA1_158940/clean/scf7180000084724:301716-301854:+	ACA1_158940	Oxidoreductase [Source:UniProtKB/TrEMBL;Acc:L8HBL7]	0.01048109	0.332702367	0.322221277	3.69E-06
3h	ACA1_179970/clean/scf7180000084744:12494-12615:+	ACA1_179970	PPM-type phosphatase domain-containing protein [Source:UniProtKB/TrEMBL;Acc:L8GF73]	0.015169073	0.3373	0.322130927	1.47E-07
3h	ACA1_386360/clean/scf7180000084730:334456-334539:+	ACA1_386360	WH2 domain-containing protein [Source:UniProtKB/TrEMBL;Acc:L8HAS1]	0.007745823	0.329832	0.322086177	6.43E-06
3h	ACA1_272960/clean/scf7180000084773:3944-4025:-	ACA1_272960	Ras-GAP domain-containing protein [Source:UniProtKB/TrEMBL;Acc:L8GFH7]	0	0.322069433	0.322069433	1.73E-07
3h	ACA1_295260/clean/scf7180000084777:1086620-1086842:+	ACA1_295260	CTP:ethanolaminephosphate cytidylyltransferase [Source:UniProtKB/TrEMBL;Acc:L8HM00]	0.031986667	0.354034	0.322047333	5.22E-05
3h	ACA1_198970/clean/scf7180000084753:181198-181282:-	ACA1_198970	Viral Atype inclusion protein, putative [Source:UniProtKB/TrEMBL;Acc:L8H376]	0.000236911	0.322272667	0.322035756	2.57E-06
3h	ACA1_361100/clean/scf7180000084622:242045-242142:-	ACA1_361100	TLDc domain-containing protein [Source:UniProtKB/TrEMBL;Acc:L8HE93]	0.007396107	0.329242167	0.32184606	1.96E-05
3h	ACA1_031870/clean/scf7180000084523:24541-24631:-	ACA1_031870	TYR_PHOSPHATASE_2 domain-containing protein [Source:UniProtKB/TrEMBL;Acc:L8H378]	0.001557633	0.323328333	0.3217707	1.63E-08
3h	ACA1_045270/clean/scf7180000084576:102853-102968:+	ACA1_045270	Vacuolar protein sorting-associated protein 28 homolog [Source:UniProtKB/TrEMBL;Acc:L8H286]	0.00965656	0.331224967	0.321568407	5.72E-06
3h	ACA1_184600/clean/scf7180000084746:5215-5293:-	ACA1_184600	Alpha-N-acetylglucosaminidase family protein [Source:UniProtKB/TrEMBL;Acc:L8H6D3]	0	0.321552333	0.321552333	8.25E-06
3h	ACA1_064270/clean/scf7180000084599:1574123-1574231:-	ACA1_064270	Dihydroxybiphenyl dioxygenase, putative [Source:UniProtKB/TrEMBL;Acc:L8GYP3]	0.011516277	0.333065333	0.321549057	6.33E-06
3h	ACA1_233420/clean/scf7180000084762:137601-137680:+	ACA1_233420	hypothetical protein	0.002569307	0.324113	0.321543693	4.21E-11
3h	ACA1_236340/clean/scf7180000084762:441442-441554:-	ACA1_236340	hypothetical protein	0.011242023	0.332603733	0.32136171	6.24E-09
3h	ACA1_238440/clean/scf7180000084763:95336-95452:+	ACA1_238440	UDG domain-containing protein [Source:UniProtKB/TrEMBL;Acc:L8GJQ7]	0.018967837	0.3401514	0.321183563	0.000282634
3h	ACA1_395410/clean/scf7180000084756:172095-172176:-	ACA1_395410	hypothetical protein	0	0.320964	0.320964	1.17E-05
3h	ACA1_287850/clean/scf7180000084777:96448-96521:-	ACA1_287850	RING-type domain-containing protein [Source:UniProtKB/TrEMBL;Acc:L8HHQ7]	0	0.320936	0.320936	2.97E-07
3h	ACA1_057240/clean/scf7180000084599:95296-95387:-	ACA1_057240	unspecified product	0.008130067	0.328982767	0.3208527	0.000241302
3h	ACA1_216390/clean/scf7180000084758:487487-487548:-	ACA1_216390	MI domain-containing protein [Source:UniProtKB/TrEMBL;Acc:L8GQI1]	0.041505433	0.362239	0.320733567	1.39E-05
3h	ACA1_163660/clean/scf7180000084729:65265-65343:-	ACA1_163660	hypothetical protein	0.00606173	0.3267309	0.32066917	6.59E-08
3h	ACA1_166910/clean/scf7180000084736:26499-26559:-	ACA1_166910	Amine oxidase family protein [Source:UniProtKB/TrEMBL;Acc:L8H242]	0.003108003	0.3237024	0.320594397	0.000101789
3h	ACA1_138810/clean/scf7180000084705:14869-14968:-	ACA1_138810	Signal transducer and activator of transcription [Source:UniProtKB/TrEMBL;Acc:L8GN02]	0.022747933	0.343160667	0.320412733	3.16E-06
3h	ACA1_331780/clean/scf7180000084578:26527-26614:-	ACA1_331780	BRCA1 C Terminus (BRCT) domain containing protein [Source:UniProtKB/TrEMBL;Acc:L8HE43]	0.05787691	0.378130667	0.320253757	0.000319562
3h	ACA1_179970/clean/scf7180000084744:11975-12149:+	ACA1_179970	PPM-type phosphatase domain-containing protein [Source:UniProtKB/TrEMBL;Acc:L8GF73]	0.0749953	0.395209333	0.320214033	0.000241302
3h	ACA1_033770/clean/scf7180000084559:129571-129655:+	ACA1_033770	RING-type domain-containing protein [Source:UniProtKB/TrEMBL;Acc:L8GJ68]	0.044034333	0.36424	0.320205667	0.000113828
3h	ACA1_208460/clean/scf7180000084757:197801-197910:-	ACA1_208460	Abhydro_lipase domain-containing protein [Source:UniProtKB/TrEMBL;Acc:L8GZL5]	0.014133223	0.334332667	0.320199443	8.42E-05
3h	ACA1_158440/clean/scf7180000084724:219391-219486:+	ACA1_158440	Coiled-coil domain containing 53, putative [Source:UniProtKB/TrEMBL;Acc:L8HA48]	0.01014013	0.329980767	0.319840637	1.37E-06
3h	ACA1_133320/clean/scf7180000084701:31451-31523:+	ACA1_133320	Homeobox domain-containing protein [Source:UniProtKB/TrEMBL;Acc:L8H2R6]	0.004989217	0.324792933	0.319803717	3.64E-06
3h	ACA1_184120/clean/scf7180000084745:565534-565605:-	ACA1_184120	hypothetical protein	0.00488832	0.324553667	0.319665347	4.01E-07
3h	ACA1_093740/clean/scf7180000084645:628418-628563:-	ACA1_093740	Serine/threonine protein kinase [Source:UniProtKB/TrEMBL;Acc:L8GJ93]	0	0.319565067	0.319565067	3.19E-08
3h	ACA1_184670/clean/scf7180000084746:30388-30467:+	ACA1_184670	B30.2/SPRY domain-containing protein [Source:UniProtKB/TrEMBL;Acc:L8H461]	0	0.319549833	0.319549833	6.11E-06
3h	ACA1_258270/clean/scf7180000084769:219009-219104:+	ACA1_258270	Ras subfamily protein [Source:UniProtKB/TrEMBL;Acc:L8GHB6]	0.009187707	0.328548667	0.31936096	1.68E-07
3h	ACA1_220060/clean/scf7180000084758:852998-853072:+	ACA1_220060	SAP domain-containing protein [Source:UniProtKB/TrEMBL;Acc:L8GQ17]	0	0.319139	0.319139	2.27E-10
3h	ACA1_107440/clean/scf7180000084660:196719-196824:+	ACA1_107440	Purple acid phosphatase [Source:UniProtKB/TrEMBL;Acc:L8GNJ7]	0.001017143	0.320146367	0.319129223	9.10E-09
3h	ACA1_254880/clean/scf7180000084768:429768-429906:-	ACA1_254880	Ankyrin repeatcontaining protein [Source:UniProtKB/TrEMBL;Acc:L8HB25]	0.0503773	0.369445667	0.319068367	0.002824815
3h	ACA1_091560/clean/scf7180000084645:151025-151111:+	ACA1_091560	UBP-type domain-containing protein [Source:UniProtKB/TrEMBL;Acc:L8GKE3]	0.02272838	0.3417414	0.31901302	0.002520568
3h	ACA1_219740/clean/scf7180000084758:797789-797876:+	ACA1_219740	Glucosamine 6-phosphate N-acetyltransferase [Source:UniProtKB/TrEMBL;Acc:L8GRH7]	0.027731667	0.346672	0.318940333	1.39E-05
3h	ACA1_006200/clean/scf7180000083029:68588-68653:-	ACA1_006200	hypothetical protein	0.020696187	0.339433667	0.31873748	0.000218435
3h	ACA1_211500/clean/scf7180000084758:89330-89418:+	ACA1_211500	RAB5B protein, putative [Source:UniProtKB/TrEMBL;Acc:L8GS95]	0.0044969	0.323199667	0.318702767	6.61E-05
3h	ACA1_188180/clean/scf7180000084747:121324-121412:-	ACA1_188180	IU_nuc_hydro domain-containing protein [Source:UniProtKB/TrEMBL;Acc:L8GUV3]	0	0.318643733	0.318643733	0.038902642
3h	ACA1_297530/clean/scf7180000082850:1426-1494:-	ACA1_297530	hypothetical protein	0.0079087	0.326436967	0.318528267	2.04E-06
3h	ACA1_145790/clean/scf7180000084710:147713-147805:+	ACA1_145790	hypothetical protein	0.030658067	0.349183	0.318524933	1.95E-05
3h	ACA1_216020/clean/scf7180000084758:434481-434585:-	ACA1_216020	CudA family protein [Source:UniProtKB/TrEMBL;Acc:L8GQ76]	0.000689607	0.319147267	0.31845766	4.50E-08
3h	ACA1_368670/clean/scf7180000084664:133824-133904:-	ACA1_368670	Mitochondrial GTPase 1 [Source:UniProtKB/TrEMBL;Acc:L8GYT9]	0.085853333	0.404201667	0.318348333	0.017392245
3h	ACA1_232860/clean/scf7180000084762:87626-87757:+	ACA1_232860	hypothetical protein	0.119683633	0.437940667	0.318257033	0.001526
3h	ACA1_288770/clean/scf7180000084777:285800-285877:-	ACA1_288770	Leucine rich repeat domain containing protein [Source:UniProtKB/TrEMBL;Acc:L8HLA9]	0	0.318136667	0.318136667	1.95E-06
3h	ACA1_295270/clean/scf7180000084777:1087863-1087956:-	ACA1_295270	Rhodanese domain-containing protein [Source:UniProtKB/TrEMBL;Acc:L8HJQ3]	0.009372487	0.327462567	0.31809008	7.29E-06
3h	ACA1_370300/clean/scf7180000084664:491066-491172:-	ACA1_370300	hypothetical protein	0.001870323	0.319574967	0.317704643	3.58E-07
3h	ACA1_040470/clean/scf7180000084572:10895-10970:+	ACA1_040470	Leuk-A4-hydro_C domain-containing protein [Source:UniProtKB/TrEMBL;Acc:L8H0K2]	0.012323313	0.330021	0.317697687	2.70E-08
3h	ACA1_169710/clean/scf7180000084739:56999-57086:+	ACA1_169710	SHSP domain-containing protein [Source:UniProtKB/TrEMBL;Acc:L8HHU0]	0.013881187	0.331563333	0.317682147	2.58E-06
3h	ACA1_052590/clean/scf7180000084594:117536-117595:+	ACA1_052590	PeptidyltRNA hydrolase PTH2 domain containing protein [Source:UniProtKB/TrEMBL;Acc:L8H778]	0.004919287	0.322479667	0.31756038	3.77E-08
3h	ACA1_091140/clean/scf7180000084645:66200-66314:-	ACA1_091140	BTB/POZ domain containing protein [Source:UniProtKB/TrEMBL;Acc:L8GIG3]	0.000355747	0.317896	0.317540253	2.90E-07
3h	ACA1_378490/clean/scf7180000084711:230233-230313:-	ACA1_378490	hypothetical protein	0.030134333	0.347477333	0.317343	3.87E-05
3h	ACA1_191150/clean/scf7180000084748:186629-186685:+	ACA1_191150	Triosephosphate isomerase [Source:UniProtKB/TrEMBL;Acc:L8GE90]	0.000958457	0.318299333	0.317340876	4.46E-09
3h	ACA1_063230/clean/scf7180000084599:1362150-1362242:-	ACA1_063230	Flagellar associated protein [Source:UniProtKB/TrEMBL;Acc:L8GWG8]	0.00723246	0.323864333	0.316631873	1.73E-05
3h	ACA1_093350/clean/scf7180000084645:525501-525579:+	ACA1_093350	Fbox domain containing protein [Source:UniProtKB/TrEMBL;Acc:L8GIV6]	0.0084656	0.325077333	0.316611733	4.07E-05

3h	ACA1_209860/clean/scf7180000084757:340311-340396:-	ACA1_209860	Protein kinase domain-containing protein [Source:UniProtKB/TrEMBL;Acc:L8GYD8]	0.0170517	0.333641333	0.316589633	1.74E-07
3h	ACA1_387910/clean/scf7180000084733:87498-87572:-	ACA1_387910	P/Homo B domain-containing protein [Source:UniProtKB/TrEMBL;Acc:L8GG58]	0	0.316555667	0.316555667	6.46E-08
3h	ACA1_116020/clean/scf7180000084669:706526-706635:+	ACA1_116020	Rho-GAP domain-containing protein [Source:UniProtKB/TrEMBL;Acc:L8H6T0]	0.005348117	0.321840567	0.31649245	6.20E-07
3h	ACA1_290560/clean/scf7180000084777:626298-626371:+	ACA1_290560	Peptidase_M14 domain-containing protein [Source:UniProtKB/TrEMBL;Acc:L8HLJ4]	0.002184607	0.318664033	0.316479427	7.63E-09
3h	ACA1_382280/clean/scf7180000084721:20240-20324:-	ACA1_382280	Aminotran_1_2 domain-containing protein [Source:UniProtKB/TrEMBL;Acc:L8GUT4]	0.010095467	0.326210067	0.3161146	0.000138644
3h	ACA1_012970/clean/scf7180000083742:13707-13796:+	ACA1_012970	CBS domain containing protein [Source:UniProtKB/TrEMBL;Acc:L8H720]	0.027879177	0.343843	0.315963823	0.000595751
3h	ACA1_002490/clean/scf7180000082931:7570-7674:-	ACA1_002490	AA_TRNA_LIGASE_II_ALA domain-containing protein [Source:UniProtKB/TrEMBL;Acc:L8GVN5]	0.00450207	0.320320033	0.315817963	2.66E-06
3h	ACA1_089960/clean/scf7180000084639:405072-405145:-	ACA1_089960	Dual specificity protein kinase [Source:UniProtKB/TrEMBL;Acc:L8GVS6]	0.00094697	0.316703633	0.315756663	1.19E-07
3h	ACA1_336380/clean/scf7180000084593:56143-56247:-	ACA1_336380	RasGEF domain containing protein [Source:UniProtKB/TrEMBL;Acc:L8H1N6]	0.004571381	0.320308667	0.315737286	2.42E-07
3h	ACA1_107160/clean/scf7180000084660:158109-158239:+	ACA1_107160	hypothetical protein	0	0.315721133	0.315721133	2.05E-08
3h	ACA1_189600/clean/scf7180000084748:62792-62864:-	ACA1_189600	hypothetical protein	0.021369393	0.337067333	0.31569794	2.43E-07
3h	ACA1_189360/clean/scf7180000084748:16941-17012:-	ACA1_189360	Proline dehydrogenase [Source:UniProtKB/TrEMBL;Acc:L8GGF7]	0	0.3156026	0.3156026	0.006472415
3h	ACA1_394730/clean/scf7180000084756:130776-130857:-	ACA1_394730	Putative tyrosineprotein phosphatase oca1, putative [Source:UniProtKB/TrEMBL;Acc:L8H2T3]	0.000958383	0.316474567	0.315516183	3.68E-08
3h	ACA1_277500/clean/scf7180000084776:64999-65080:-	ACA1_277500	DBR1 domain-containing protein [Source:UniProtKB/TrEMBL;Acc:L8H8N5]	0.001336007	0.3168191	0.315483093	0.01133922
3h	ACA1_192020/clean/scf7180000084749:74755-74851:+	ACA1_192020	Cystathionine gammasynthase [Source:UniProtKB/TrEMBL;Acc:L8GNB3]	0.001376373	0.316781333	0.31540496	8.62E-13
3h	ACA1_372060/clean/scf7180000084664:624290-624376:-	ACA1_372060	Aldo_ket_red domain-containing protein [Source:UniProtKB/TrEMBL;Acc:L8GYH3]	0.029970033	0.345243667	0.315273633	0.013340472
3h	ACA1_069110/clean/scf7180000084603:357652-357745:+	ACA1_069110	Ras small GTPbinding protein [Source:UniProtKB/TrEMBL;Acc:L8HDL4]	0.015725967	0.330979167	0.3152532	9.50E-06
3h	ACA1_370300/clean/scf7180000084664:489711-489779:-	ACA1_370300	hypothetical protein	0.01183605	0.327033	0.31519695	5.20E-07
3h	ACA1_065830/clean/scf7180000084599:1919901-1919974:-	ACA1_065830	PH domain-containing protein [Source:UniProtKB/TrEMBL;Acc:L8GYZ9]	0.02090243	0.3360669	0.31516447	0.000197768
3h	ACA1_274880/clean/scf7180000084773:132806-132883:+	ACA1_274880	AB hydrolase-1 domain-containing protein [Source:UniProtKB/TrEMBL;Acc:L8GI88]	0.055721967	0.370859	0.315137033	0.002348515
3h	ACA1_266230/clean/scf7180000084770:638959-639034:+	ACA1_266230	Dilute domain-containing protein [Source:UniProtKB/TrEMBL;Acc:L8H2D0]	0.070962667	0.386031333	0.315068667	0.02032483
3h	ACA1_227080/clean/scf7180000084761:95748-95849:+	ACA1_227080	Uracil-DNA glycosylase [Source:UniProtKB/TrEMBL;Acc:L8HA23]	0.017574667	0.332579	0.315004333	0.00029315
3h	ACA1_208500/clean/scf7180000084757:210179-210290:+	ACA1_208500	Aldedh domain-containing protein [Source:UniProtKB/TrEMBL;Acc:L8GYD7]	0.000216986	0.315101	0.314884014	7.28E-07
3h	ACA1_199700/clean/scf7180000084753:301171-301408:+	ACA1_199700	RFX DNAbinding domain containing protein [Source:UniProtKB/TrEMBL;Acc:L8H5G4]	0.021997493	0.336766667	0.314769173	0.000423267
3h	ACA1_368760/clean/scf7180000084664:155315-155383:-	ACA1_368760	hypothetical protein	0.0464501	0.361209667	0.314759567	0.007640979
3h	ACA1_215600/clean/scf7180000084758:345624-345694:-	ACA1_215600	hypothetical protein	0.074074	0.388794	0.31472	0.04879339
3h	ACA1_020880/clean/scf7180000084095:31114-31212:+	ACA1_020880	Protozoan/cyanobacterial globin family subfamily protein [Source:UniProtKB/TrEMBL;Acc:L8GU16]	0.030249033	0.344924333	0.3146753	6.03E-05
3h	ACA1_200000/clean/scf7180000084753:365781-365870:-	ACA1_200000	LAO/AO transport system ATPase [Source:UniProtKB/TrEMBL;Acc:L8H2I2]	0.014188797	0.328857667	0.31466887	2.77E-05
3h	ACA1_024630/clean/scf7180000084444:3113-3184:+	ACA1_024630	hypothetical protein	0.031906533	0.346454	0.314547467	5.50E-05
3h	ACA1_283190/clean/scf7180000084776:783803-783869:+	ACA1_283190	hypothetical protein	0	0.3144984	0.3144984	0.000453195
3h	ACA1_057400/clean/scf7180000084599:135093-135206:+	ACA1_057400	Leucine rich repeat domain containing protein [Source:UniProtKB/TrEMBL;Acc:L8GVU1]	0.022467733	0.336669267	0.314201533	0.000633219
3h	ACA1_140780/clean/scf7180000084707:121320-121400:+	ACA1_140780	Zinc carboxypeptidase superfamily protein [Source:UniProtKB/TrEMBL;Acc:L8GGS5]	0.017335933	0.331325333	0.3139894	4.36E-07
3h	ACA1_173960/clean/scf7180000084741:560552-560655:+	ACA1_173960	AB hydrolase-1 domain-containing protein [Source:UniProtKB/TrEMBL;Acc:L8HJ69]	0.010636303	0.324583	0.313946697	3.39E-05
3h	ACA1_051910/clean/scf7180000084592:83176-83288:-	ACA1_051910	Twocomponent hybrid sensor and regulator kinase [Source:UniProtKB/TrEMBL;Acc:L8GQL7]	0.00245151	0.316369667	0.313918157	0.00154091
3h	ACA1_074100/clean/scf7180000084605:24405-24516:+	ACA1_074100	Fungal Zn(2)-Cys(6) binuclear cluster domain containing protein [Source:UniProtKB/TrEMBL;Acc:L8HLU8]	0.01016689	0.323962167	0.313795277	1.77E-05
3h	ACA1_306840/clean/scf7180000083668:18142-18222:+	ACA1_306840	RTP1_C1 domain-containing protein [Source:UniProtKB/TrEMBL;Acc:L8GQF6]	0	0.313759633	0.313759633	0.000128546
3h	ACA1_020160/clean/scf7180000084037:19922-19986:-	ACA1_020160	Sec16_C domain-containing protein [Source:UniProtKB/TrEMBL;Acc:L8GLH3]	0.00147615	0.3151501	0.31367395	3.63E-08
3h	ACA1_270330/clean/scf7180000084770:1113474-1113555:-	ACA1_270330	hypothetical protein	0.047096467	0.360694667	0.3135982	0.013706033
3h	ACA1_113540/clean/scf7180000084669:313580-313658:-	ACA1_113540	SCP domain-containing protein [Source:UniProtKB/TrEMBL;Acc:L8H3B1]	0.01591916	0.3295011	0.31358194	1.19E-05
3h	ACA1_113600/clean/scf7180000084669:328866-328928:+	ACA1_113600	CBS domain containing protein [Source:UniProtKB/TrEMBL;Acc:L8H4A7]	0.005720673	0.319207667	0.313486993	1.10E-05
3h	ACA1_068080/clean/scf7180000084603:100227-100300:+	ACA1_068080	PlsC domain-containing protein [Source:UniProtKB/TrEMBL;Acc:L8HCG7]	0.007083033	0.320429667	0.313346633	3.32E-08
3h	ACA1_384870/clean/scf7180000084730:127561-127634:+	ACA1_384870	Protein kinase domain containing protein [Source:UniProtKB/TrEMBL;Acc:L8HBH4]	0.000522467	0.313819933	0.313297467	8.30E-07
3h	ACA1_063610/clean/scf7180000084599:1459658-1459741:-	ACA1_063610	Vacuolar protein sorting 55 protein [Source:UniProtKB/TrEMBL;Acc:L8GXE6]	0.024463067	0.337686133	0.313223067	0.000176206
3h	ACA1_338000/clean/scf7180000084604:23377-23439:-	ACA1_338000	hypothetical protein	0.000673202	0.313801133	0.313127932	3.33E-10
3h	ACA1_052780/clean/scf7180000084594:178097-178173:-	ACA1_052780	F-box domain-containing protein [Source:UniProtKB/TrEMBL;Acc:L8H818]	0.000795018	0.313825667	0.313030649	4.58E-06
3h	ACA1_222650/clean/scf7180000084760:44553-44681:-	ACA1_222650	hypothetical protein	0.02813088	0.341086	0.31295512	2.98E-05
3h	ACA1_156480/clean/scf7180000084723:44530-44606:+	ACA1_156480	PHB domain-containing protein [Source:UniProtKB/TrEMBL;Acc:L8GK25]	0.00255298	0.315489433	0.312936453	1.68E-09
3h	ACA1_054100/clean/scf7180000084594:401841-401968:+	ACA1_054100	Diacylglycerol kinase [Source:UniProtKB/TrEMBL;Acc:L8H6A6]	0	0.3128287	0.3128287	1.56E-05
3h	ACA1_054040/clean/scf7180000084594:380163-380256:-	ACA1_054040	F-box domain-containing protein [Source:UniProtKB/TrEMBL;Acc:L8H6A3]	0.000156397	0.312946333	0.312789937	6.53E-07
3h	ACA1_236090/clean/scf7180000084762:402247-402314:-	ACA1_236090	PUL domain-containing protein [Source:UniProtKB/TrEMBL;Acc:L8H3Q0]	0.097324467	0.409856433	0.312531967	0.02921499
3h	ACA1_219480/clean/scf7180000084758:747663-747773:+	ACA1_219480	hypothetical protein	0.00138889	0.313690633	0.312301743	1.89E-07
3h	ACA1_366940/clean/scf7180000084641:520870-520961:-	ACA1_366940	BTB/POZ domain containing protein [Source:UniProtKB/TrEMBL;Acc:L8GPM1]	0	0.312249333	0.312249333	4.88E-05
3h	ACA1_382370/clean/scf7180000084721:42250-42320:-	ACA1_382370	P-type domain-containing protein [Source:UniProtKB/TrEMBL;Acc:L8GVY3]	0.051350267	0.363592	0.312241733	4.61E-05
3h	ACA1_189600/clean/scf7180000084748:62669-62743:-	ACA1_189600	hypothetical protein	0.00066539	0.3126705	0.31200511	6.46E-09
3h	ACA1_320840/clean/scf7180000084416:33068-33130:+	ACA1_320840	HSP91 (Heat shock protein 91), putative [Source:UniProtKB/TrEMBL;Acc:L8GTS5]	0.000812788	0.3127973	0.311984512	2.43E-17
3h	ACA1_052710/clean/scf7180000084594:158163-158302:+	ACA1_052710	acidPPc domain-containing protein [Source:UniProtKB/TrEMBL;Acc:L8H519]	0.036365433	0.348343	0.311977567	1.81E-05
3h	ACA1_063260/clean/scf7180000084599:1367950-1368039:+	ACA1_063260	Short chain dehydrogenase/reductase family oxidoreductase [Source:UniProtKB/TrEMBL;Acc:L8GZN5]	0.029515933	0.341270667	0.311754733	0.000133015
3h	ACA1_173670/clean/scf7180000084741:512273-512370:+	ACA1_173670	DEP domain-containing protein [Source:UniProtKB/TrEMBL;Acc:L8HGP4]	0.005817933	0.317557333	0.3117394	0.010529685
3h	ACA1_061350/clean/scf7180000084599:889801-889882:+	ACA1_061350	PlsC domain-containing protein [Source:UniProtKB/TrEMBL;Acc:L8GWS1]	0.090178733	0.401910333	0.3117316	0.002898819
3h	ACA1_307550/clean/scf7180000083680:33230-33299:+	ACA1_307550	Fbox domain containing protein [Source:UniProtKB/TrEMBL;Acc:L8H529]	0.0095238	0.3212502	0.3117264	0.010032832
3h	ACA1_224390/clean/scf7180000084760:189277-189404:-	ACA1_224390	hypothetical protein	0.055214967	0.3669371	0.311722133	0.000660581
3h	ACA1_339740/clean/scf7180000084619:17500-17590:+	ACA1_339740	Serine/threonine protein kinase [Source:UniProtKB/TrEMBL;Acc:L8GLZ6]	0.002849003	0.314488567	0.311639563	1.75E-05
3h	ACA1_400220/clean/scf7180000084775:121956-122032:-	ACA1_400220	Abhydrolase_2 domain-containing protein [Source:UniProtKB/TrEMBL;Acc:L8GG24]	0.023944133	0.335451667	0.311507533	2.73E-07
3h	ACA1_384550/clean/scf7180000084730:45806-45908:+	ACA1_384550	GTPase, putative [Source:UniProtKB/TrEMBL;Acc:L8H969]	0.01258593	0.323842467	0.311256537	8.50E-06
3h	ACA1_145740/clean/scf7180000084710:137045-137117:+	ACA1_145740	CBS domain containing protein [Source:UniProtKB/TrEMBL;Acc:L8GFG0]	0.014730633	0.325878	0.311147367	0.003309609
3h	ACA1_091710/clean/scf7180000084645:187300-187383:-	ACA1_091710	PAS domain Sbox domain containing protein [Source:UniProtKB/TrEMBL;Acc:L8GKF5]	0.00167522	0.3126792	0.31100398	6.39E-07
3h	ACA1_164510/clean/scf7180000084729:234870-234939:+	ACA1_164510	Leucine rich repeat domain containing protein [Source:UniProtKB/TrEMBL;Acc:L8GU14]	0.055738367	0.366683667	0.3109453	8.56E-05
3h	ACA1_096600/clean/scf7180000084645:1059470-1059550:+	ACA1_096600	CUE domain containing protein [Source:UniProtKB/TrEMBL;Acc:L8GJT0]	0.003892663	0.314633	0.310740337	3.82E-08
3h	ACA1_279270/clean/scf7180000084776:344789-344860:+	ACA1_279270	RNA_ligase domain-containing protein [Source:UniProtKB/TrEMBL;Acc:L8H728]	0.00144569	0.312152	0.31070631	1.68E-07
3h	ACA1_389130/clean/scf7180000084733:207141-207241:+	ACA1_389130	BTB/POZ domain containing protein [Source:UniProtKB/TrEMBL;Acc:L8GDV1]	0	0.310653633	0.310653633	2.62E-08
3h	ACA1_063910/clean/scf7180000084599:1535012-1535091:+	ACA1_063910	Tho complex subunit 7 protein [Source:UniProtKB/TrEMBL;Acc:L8GXH2]	0.000472947	0.3110712	0.310598253	2.20E-07
3h	ACA1_368190/clean/scf7180000084664:74697-74764:+	ACA1_368190	hypothetical protein	0.00331497	0.313799533	0.310484563	2.65E-08
3h	ACA1_215970/clean/scf7180000084758:418669-418750:-	ACA1_215970	EGFlike domain containing protein [Source:UniProtKB/TrEMBL;Acc:L8GQ73]	0.111111	0.421565	0.310454	0.009082117
3h	ACA1_330410/clean/scf7180000084566:53858-53947:+	ACA1_330410	hypothetical protein	0.00491586	0.315339667	0.310423806	1.36E-06
3h	ACA1_390570/clean/scf7180000084740:74520-74583:-	ACA1_390570	Acetyl/propionyl CoA carboxylase, beta subunit, putative [Source:UniProtKB/TrEMBL;Acc:L8GRH2]	0.00161744	0.312002667	0.310385227	3.32E-06
3h	ACA1_069710/clean/scf7180000084603:533620-533695:+	ACA1_069710	Serine/threonine kinase [Source:UniProtKB/TrEMBL;Acc:L8HG11]	0.016666667	0.326970667	0.310304	6.25E-05
3h	ACA1_268930/clean/scf7180000084770:944457-944617:+	ACA1_268930	PWI domain-containing protein [Source:UniProtKB/TrEMBL;Acc:L8H2N5]	0.051006967	0.361287667	0.3102807	0.000465781
3h	ACA1_175870/clean/scf7180000084741:1080567-1080666:-	ACA1_175870	Zinc finger, C3HC4 type (RING finger) domain containing protein [Source:UniProtKB/TrEMBL;Acc:L8HI76]	0.015468567	0.3257311	0.310262533	0.000406266
3h	ACA1_086070/clean/scf7180000084636:21556-21640:-	ACA1_086070	Serine/threonine kinase [Source:UniProtKB/TrEMBL;Acc:L8HJ13]	0.036842833	0.347021067	0.310178233	0.000237327
3h	ACA1_250030/clean/scf7180000084767:26625-26710:-	ACA1_250030	C2 NT-type domain-containing protein [Source:UniProtKB/TrEMBL;Acc:L8HC94]	8.38E-05	0.3102573	0.310173464	2.03E-06
3h	ACA1_130580/clean/scf7180000084698:114581-114694:-	ACA1_130580	CID domain-containing protein [Source:UniProtKB/TrEMBL;Acc:L8GQC8]	0.021174933	0.331288667	0.310113733	1.32E-05
3h	ACA1_219870/clean/scf7180000084758:829762-829897:-	ACA1_219870	Lysophospholipase 3 (Lysosomal phospholipase A2) isoform 7, putative [Source:UniProtKB/TrEMBL;Acc:L8GT04]	0.004016067	0.314087333	0.310071267	4.93E-07
3h	ACA1_215010/clean/scf7180000084758:293378-293461:-	ACA1_215010	Inorganic diphosphatase [Source:UniProtKB/TrEMBL;Acc:L8GQA5]	0.00050288	0.310573333	0.310070454	1.20E-10
3h	ACA1_146500/clean/scf7180000084713:35021-35116:-	ACA1_146500	DH domain-containing protein [Source:UniProtKB/TrEMBL;Acc:L8GWP3]	0.005239593	0.315258667	0.310019073	7.05E-06
3h	ACA1_098160/clean/scf7180000084645:1411447-1411532:-	ACA1_098160	ABC transporter, ATPbinding domain containing protein [Source:UniProtKB/TrEMBL;Acc:L8GJY4]	0.00102607	0.3110207	0.30999463	1.50E-06
3h	ACA1_233410/clean/scf7180000084762:136301-136400:+	ACA1_233410	NFX1type zinc finger-containing protein 1, putative [Source:UniProtKB/TrEMBL;Acc:L8H2G5]	0.001403695	0.311358267	0.309954572	9.36E-10
3h	ACA1_031640/clean/scf7180000084522:34712-34805:-	ACA1_031640	PH domain-containing protein [Source:UniProtKB/TrEMBL;Acc:L8GV44]	0.0073859	0.317303367	0.309917467	2.09E-05
3h	ACA1_031780/clean/scf7180000084523:1398-1474:-	ACA1_031780	Lactamase_B domain-containing protein [Source:UniProtKB/TrEMBL;Acc:L8H103]	0.0178076	0.327693067	0.309885467	0.004400459
3h	ACA1_076860/clean/scf7180000084612:168311-168409:+	ACA1_076860	RNA binding motif containing protein [Source:UniProtKB/TrEMBL;Acc:L8GM55]	0.005808323	0.3156751	0.309866777	9.80E-06
3h	ACA1_201510/clean/scf7180000084753:577863-577923:+	ACA1_201510	Ubiquitin fusion degradation protein, putative [Source:UniProtKB/TrEMBL;Acc:L8H4X5]	0.070918267	0.380692	0.309773733	0.000169231
3h	ACA1_278960/clean/scf7180000084776:285690-285777:-	ACA1_278960	Cbl_N2 domain-containing protein [Source:UniProtKB/TrEMBL;Acc:L8H705]	0.01532262	0.325077333	0.309754713	0.002635551
3h	ACA1_237770/clean/scf7180000084763:11940-12056:+	ACA1_237770	PH domain-containing protein [Source:UniProtKB/TrEMBL;Acc:L8GKB5]	0.024016833	0.3337714	0.309754567	7.83E-05
3h	ACA1_182940/clean/scf7180000084745:293616-293687:+	ACA1_182940	Myblike DNA-binding domain containing protein [Source:UniProtKB/TrEMBL;Acc:L8H867]	0	0.309741	0.309741	2.02E-07
3h	ACA1_171230/clean/scf7180000084741:50562-50638:+	ACA1_171230	Arf-GAP domain-containing protein [Source:UniProtKB/TrEMBL;Acc:L8HHA4]	0.004389993	0.314015	0.309625007	1.25E-07
3h	ACA1_287790/clean/scf7180000084777:79642-79717:-	ACA1_287790	hypothetical protein	0.00268743	0.312302	0.30961457	1.68E-06
3h	ACA1_151330/clean/scf7180000084716:105769-105873:-	ACA1_151330	Purple acid phosphatase [Source:UniProtKB/TrEMBL;Acc:L8H0Q3]	0.005695953	0.315124	0.309428047	3.89E-08
3h	ACA1_113900/clean/scf7180000084669:405534-405605:+	ACA1_113900	Leucine rich repeat domain containing protein [Source:UniProtKB/TrEMBL;Acc:L8H3G0]	0.00032113	0.309669267	0.309348136	5.28E-08
3h	ACA1_389830/clean/scf7180000084740:725-812:+	ACA1_389830	Response regulatory domain-containing protein [Source:UniProtKB/TrEMBL;Acc:L8GNU6]	0.011807821	0.321151167	0.309343346	2.87E-05
3h	ACA1_058500/clean/scf7180000084599:335174-335515:-	ACA1_058500	RNA_ligase domain-containing protein [Source:UniProtKB/TrEMBL;Acc:L8GVF6]	0.537797	0.847071	0.309274	0.003513888
3h	ACA1_397190/clean/scf7180000084759:167925-168044:+	ACA1_397190	Metallophos domain-containing protein [Source:UniProtKB/TrEMBL;Acc:L8HDM9]	0.004639413	0.313755667	0.309116253	1.18E-07
3h	ACA1_027000/clean/scf7180000084464:17290-17475:-	ACA1_027000	SCP family extracellular, putative [Source:UniProtKB/TrEMBL;Acc:L8GHL6]	0.004400117	0.313392667	0.30899255	4.17E-07
3h	ACA1_112830/clean/scf7180000084669:225471-225557:-	ACA1_112830	RFX-type winged-helix domain-containing protein [Source:UniProtKB/TrEMBL;Acc:L8H5H1]	0.0648712	0.373855333	0.308984133	0.013977527
3h	ACA1_182120/clean/scf7180000084745:132080-132209:+	ACA1_182120	WD repeat domain 5B, putative [Source:UniProtKB/TrEMBL;Acc:L8HA46]	0.001177547	0.310091667	0.30891412	5.24E-08
3h	ACA1_142310/clean/scf7180000084708:133755-133861:+	ACA1_142310	unspecified product	0.261774	0.570565	0.308791	0.020980244
3h	ACA1_379330/clean/scf7180000084711:330263-330363:-	ACA1_379330	Polynucleotide adenyltransferase [Source:UniProtKB/TrEMBL;Acc:L8GUG7]	0.001733853	0.310469867	0.308736013	0.000799778
3h	ACA1_296550/clean/scf7180000084777:1380372-1380506:+	ACA1_296550	hypothetical protein	0.001005027	0.309657333	0.308652307	2.17E-06
3h	ACA1_002770/clean/scf7180000082937:8154-8223:+	ACA1_002770	Fbox domain containing protein [Source:UniProtKB/TrEMBL;Acc:L8GJ90]	0.0210717	0.329584333	0.308512633	9.86E-06
3h	ACA1_245470/clean/scf7180000084763:493760-493847:+	ACA1_245470	4F5 domain-containing protein [Source:UniProtKB/TrEMBL;Acc:L8GK44]	0.002026473	0.3104387	0.308412227	3.11E-20
3h	ACA1_216440/clean/scf7180000084758:505206-505330:+	ACA1_216440	PPM-type phosphatase domain-containing protein [Source:UniProtKB/TrEMBL;Acc:L8GQI6]	0.247632	0.555957333	0.308325333	0.008506946
3h	ACA1_398440/clean/scf7180000084759:225214-225317:-	ACA1_398440	hypothetical protein	0.047619	0.355789733	0.308170733	0.016625036
3h	ACA1_385120/clean/scf7180000084730:137428-137560:+	ACA1_385120	Non-specific serine/threonine protein kinase [Source:UniProtKB/TrEMBL;Acc:L8HBH8]	0	0.308160333	0.308160333	8.51E-11
3h	ACA1_321550/clean/scf7180000084454:1954-2049:-	ACA1_321550	F-box domain-containing protein [Source:UniProtKB/TrEMBL;Acc:L8GYJ0]	0.001244683	0.309232667	0.307987983	8.49E-08
3h	ACA1_176120/clean/scf7180000084741:1127027-1127092:+	ACA1_176120	Armadillo repeat containing 7 isoform 1 family protein [Source:UniProtKB/TrEMBL;Acc:L8HH78]	0.003856347	0.311795667	0.30793932	1.62E-06
3h	ACA1_097590/clean/scf7180000084645:1291900-1291982:-	ACA1_097590	Amine oxidase [Source:UniProtKB/TrEMBL;Acc:L8GK33]	0.001863637	0.309743033	0.307879397	8.70E-12
3h	ACA1_053370/clean/scf7180000084594:270222-270305:-	ACA1_053370	acidPPc domain-containing protein [Source:UniProtKB/TrEMBL;Acc:L8H555]	0.01727133	0.324987	0.30771567	2.14E-05
3h	ACA1_107450/clean/scf7180000084660:199524-199620:-	ACA1_107450	PB1 domain-containing protein [Source:UniProtKB/TrEMBL;Acc:L8GNC1]	0.023478469	0.331180133	0.307701665	0.017456213
3h	ACA1_224500/clean/scf7180000084760:218475-218568:-	ACA1_224500	Phospholipid-transporting ATPase [Source:UniProtKB/TrEMBL;Acc:L8GVH2]	6.79E-05	0.307485667	0.307417803	6.11E-09
3h	ACA1_025540/clean/scf7180000084448:7249-7326:+	ACA1_025540	hypothetical protein	8.22E-05	0.307490667	0.307408453	4.38E-05
3h	ACA1_329650/clean/scf7180000084562:37827-37914:-	ACA1_329650	hypothetical protein	0.00216076	0.3095615	0.30740074	3.77E-09
3h	ACA1_101530/clean/scf7180000084649:86623-86676:-	ACA1_101530	6phosphogluconate dehydrogenase NAD-binding domain containing protein [Source:UniProtKB/TrEMBL;Acc:L8GGL6]	0.027819167	0.335195333	0.307376167	7.66E-06
3h	ACA1_362650/clean/scf7180000084624:124114-124224:+	ACA1_362650	G-patch domain-containing protein [Source:UniProtKB/TrEMBL;Acc:L8GFY2]	0	0.307259	0.307259	1.80E-06
3h	ACA1_362730/clean/scf7180000084624:161027-161133:-	ACA1_362730	ANK_REP_REGION domain-containing protein [Source:UniProtKB/TrEMBL;Acc:L8GHW9]	0.003576757	0.310789667	0.30721291	5.48E-10
3h	ACA1_199500/clean/scf7180000084753:249005-249140:+	ACA1_199500	1acylglycerophosphocholine O-acyltransferase 1, putative [Source:UniProtKB/TrEMBL;Acc:L8H4L3]	0.049149667	0.356214667	0.307065	0.01159488
3h	ACA1_280610/clean/scf7180000084776:507195-507282:+	ACA1_280610	C2H2-type domain-containing protein [Source:UniProtKB/TrEMBL;Acc:L8H702]	0	0.307050233	0.307050233	4.45E-05
3h	ACA1_096600/clean/scf7180000084645:1058657-1058759:+	ACA1_096600	CUE domain containing protein [Source:UniProtKB/TrEMBL;Acc:L8GJT0]	0	0.307037967	0.307037967	7.31E-09
3h	ACA1_037150/clean/scf7180000084567:68306-68382:+	ACA1_037150	DUF1990 domain-containing protein [Source:UniProtKB/TrEMBL;Acc:L8H384]	0.030000667	0.3369737	0.306973033	6.73E-05
3h	ACA1_117070/clean/scf7180000084669:906406-906497:-	ACA1_117070	Rap-GAP domain-containing protein [Source:UniProtKB/TrEMBL;Acc:L8H3Z5]	0.012688997	0.3196128	0.306923803	0.000348297
3h	ACA1_158530/clean/scf7180000084724:245827-245913:+	ACA1_158530	Glucose-6-phosphate 1-dehydrogenase [Source:UniProtKB/TrEMBL;Acc:L8HBJ1]	0.060113867	0.366976667	0.3068628	0.005475262
3h	ACA1_360060/clean/scf7180000084622:40272-40346:-	ACA1_360060	GHMP_kinases_C domain-containing protein [Source:UniProtKB/TrEMBL;Acc:L8HF07]	0.007109473	0.313971667	0.306862193	1.07E-06
3h	ACA1_240070/clean/scf7180000084763:196478-196549:-	ACA1_240070	Carboxylic ester hydrolase [Source:UniProtKB/TrEMBL;Acc:L8GJU3]	0.00403961	0.310769767	0.306730157	3.89E-07
3h	ACA1_011280/clean/scf7180000083623:9478-9593:+	ACA1_011280	AAA domain-containing protein [Source:UniProtKB/TrEMBL;Acc:L8GP61]	0.006682407	0.313362667	0.30668026	1.83E-06
3h	ACA1_041790/clean/scf7180000084573:12909-12980:+	ACA1_041790	Ornithine/lysine/arginine decarboxylase [Source:UniProtKB/TrEMBL;Acc:L8GV78]	0.041628633	0.348225667	0.306597033	2.43E-05
3h	ACA1_053340/clean/scf7180000084594:266805-266875:-	ACA1_053340	Dynein light intermediate chain [Source:UniProtKB/TrEMBL;Acc:L8H840]	0.003753834	0.310327633	0.306573799	6.14E-07
3h	ACA1_268750/clean/scf7180000084770:912240-912428:+	ACA1_268750	Thioredoxin domain-containing protein [Source:UniProtKB/TrEMBL;Acc:L8H1X3]	0.023893263	0.330304	0.306410737	8.22E-07
3h	ACA1_320840/clean/scf7180000084416:34222-34326:+	ACA1_320840	HSP91 (Heat shock protein 91), putative [Source:UniProtKB/TrEMBL;Acc:L8GTS5]	0.001256895	0.307628133	0.306371238	2.18E-13
3h	ACA1_096820/clean/scf7180000084645:1108663-1108764:-	ACA1_096820	hypothetical protein	0.000302322	0.3066363	0.306333978	6.36E-08
3h	ACA1_318550/clean/scf7180000084158:23927-24008:-	ACA1_318550	HECT domain-containing protein [Source:UniProtKB/TrEMBL;Acc:L8GGI5]	0.005627072	0.311930433	0.306303361	2.20E-07
3h	ACA1_264150/clean/scf7180000084770:201817-201873:-	ACA1_264150	Gammaglutamyltranspeptidase [Source:UniProtKB/TrEMBL;Acc:L8H169]	0	0.306294	0.306294	1.84E-05

3h	ACA1_244870/clean/scf7180000084763:447172-447262:+	ACA1_244870	DNA polymerase [Source:UniProtKB/TrEMBL;Acc:L8GK72]	0.0186346	0.324815333	0.306180733	9.12E-06
3h	ACA1_325560/clean/scf7180000084495:32979-33123:+	ACA1_325560	Solute carrier family protein [Source:UniProtKB/TrEMBL;Acc:L8H8Z4]	0.00430814	0.310441	0.30613286	1.67E-08
3h	ACA1_151100/clean/scf7180000084716:56953-57080:-	ACA1_151100	NFACT-R_1 domain-containing protein [Source:UniProtKB/TrEMBL;Acc:L8H219]	0.011705653	0.317836333	0.30613068	3.70E-05
3h	ACA1_185890/clean/scf7180000084746:138788-138874:+	ACA1_185890	Peptidase S74 domain-containing protein [Source:UniProtKB/TrEMBL;Acc:L8H596]	0.001319467	0.3074382	0.306118733	3.93E-07
3h	ACA1_377830/clean/scf7180000084711:126742-126846:+	ACA1_377830	UBIQUITIN_CONJUGAT_2 domain-containing protein [Source:UniProtKB/TrEMBL;Acc:L8GSV2]	0.000784566	0.306851667	0.3060671	4.37E-09
3h	ACA1_109130/clean/scf7180000084661:127105-127197:+	ACA1_109130	Serine/threonine kinase [Source:UniProtKB/TrEMBL;Acc:L8GDM5]	0.014198677	0.320258667	0.30605999	1.95E-06
3h	ACA1_146600/clean/scf7180000084713:65025-65116:-	ACA1_146600	Protein kinase domain containing protein [Source:UniProtKB/TrEMBL;Acc:L8GWQ5]	0.00146788	0.307494	0.30602612	3.15E-11
3h	ACA1_352400/clean/scf7180000084704:9684-9767:-	ACA1_352400	Fn3_like domain-containing protein [Source:UniProtKB/TrEMBL;Acc:L8GTX5]	9.32E-05	0.305926	0.305832838	3.38E-10
3h	ACA1_123640/clean/scf7180000084686:49453-49539:+	ACA1_123640	hypothetical protein	0.0366868	0.342346	0.3056592	0.000283781
3h	ACA1_210520/clean/scf7180000084758:44833-44899:+	ACA1_210520	SANT domain-containing protein [Source:UniProtKB/TrEMBL;Acc:L8GPP0]	0.005291	0.310923067	0.305632067	4.61E-05
3h	ACA1_140730/clean/scf7180000084707:108602-108685:-	ACA1_140730	Kelch repeat protein [Source:UniProtKB/TrEMBL;Acc:L8GGS1]	0.002666667	0.308296833	0.305630167	1.67E-05
3h	ACA1_198970/clean/scf7180000084753:181478-181554:-	ACA1_198970	Viral Atype inclusion protein, putative [Source:UniProtKB/TrEMBL;Acc:L8H376]	0	0.305565033	0.305565033	2.51E-05
3h	ACA1_076210/clean/scf7180000084612:26292-26379:-	ACA1_076210	hypothetical protein	0.002967023	0.308443667	0.305476643	1.06E-07
3h	ACA1_216420/clean/scf7180000084758:497448-497572:-	ACA1_216420	C2 domain-containing protein [Source:UniProtKB/TrEMBL;Acc:L8GQ92]	0.0222491	0.327683333	0.305434233	2.78E-05
3h	ACA1_378040/clean/scf7180000084711:174686-174777:-	ACA1_378040	RING-CH-type domain-containing protein [Source:UniProtKB/TrEMBL;Acc:L8GRZ8]	0	0.3053932	0.3053932	2.56E-07
3h	ACA1_029510/clean/scf7180000084477:871-950:-	ACA1_029510	Leucine rich repeat containing protein [Source:UniProtKB/TrEMBL;Acc:L8H9E3]	0	0.3053711	0.3053711	2.76E-06
3h	ACA1_171880/clean/scf7180000084741:183873-183962:-	ACA1_171880	F-box domain-containing protein [Source:UniProtKB/TrEMBL;Acc:L8HGG2]	0.024553567	0.329894	0.305340433	0.00221505
3h	ACA1_280510/clean/scf7180000084776:486423-486517:-	ACA1_280510	Peptidase A1 domain-containing protein [Source:UniProtKB/TrEMBL;Acc:L8H8F0]	0.012828367	0.317931	0.305102633	2.32E-05
3h	ACA1_147520/clean/scf7180000084714:30769-30881:+	ACA1_147520	RanBP2-type domain-containing protein [Source:UniProtKB/TrEMBL;Acc:L8GKJ7]	0.006155167	0.311256	0.305100833	0.000195413
3h	ACA1_042040/clean/scf7180000084573:44273-44356:-	ACA1_042040	RAI1 domain-containing protein [Source:UniProtKB/TrEMBL;Acc:L8GV91]	0.010460833	0.3154497	0.304988867	0.000343116
3h	ACA1_251800/clean/scf7180000084768:122196-122338:+	ACA1_251800	WD domain, Gbeta repeat-containing protein [Source:UniProtKB/TrEMBL;Acc:L8HD10]	0.002903773	0.3077304	0.304826627	4.97E-05
3h	ACA1_164760/clean/scf7180000084729:269004-269067:+	ACA1_164760	hypothetical protein	0.035348467	0.340096667	0.3047482	7.63E-06
3h	ACA1_070900/clean/scf7180000084603:797775-797923:+	ACA1_070900	MMtag domain-containing protein [Source:UniProtKB/TrEMBL;Acc:L8HD49]	0.069735433	0.374391	0.304655567	0.001623815
3h	ACA1_034300/clean/scf7180000084563:6907-7004:+	ACA1_034300	DUF1084 domain-containing protein [Source:UniProtKB/TrEMBL;Acc:L8HCY8]	0.024047467	0.3286475	0.304600033	0.000110621
3h	ACA1_037160/clean/scf7180000084567:68867-68985:-	ACA1_037160	Leucine rich repeat domain containing protein [Source:UniProtKB/TrEMBL;Acc:L8H0X7]	0.011154227	0.315639333	0.304485107	8.27E-07
3h	ACA1_156030/clean/scf7180000084723:7948-8031:+	ACA1_156030	hypothetical protein	0.008333333	0.3127955	0.304462167	7.27E-05
3h	ACA1_370140/clean/scf7180000084664:448611-448699:+	ACA1_370140	RFX-type winged-helix domain-containing protein [Source:UniProtKB/TrEMBL;Acc:L8GZ40]	0.014695357	0.319088333	0.304392977	1.15E-06
3h	ACA1_106750/clean/scf7180000084660:115907-115995:+	ACA1_106750	Ubiquinone biosynthesis protein AarF family protein, putative [Source:UniProtKB/TrEMBL;Acc:L8GNA2]	0.026761033	0.331149667	0.304388633	2.61E-05
3h	ACA1_233880/clean/scf7180000084762:177067-177221:+	ACA1_233880	Purple acid phosphatase [Source:UniProtKB/TrEMBL;Acc:L8H124]	0.003573337	0.307958033	0.304384697	1.17E-08
3h	ACA1_052820/clean/scf7180000084594:189025-189126:-	ACA1_052820	hypothetical protein	0.0454026	0.349768	0.3043654	0.000109059
3h	ACA1_366310/clean/scf7180000084641:389531-389642:+	ACA1_366310	Reticulon-like protein [Source:UniProtKB/TrEMBL;Acc:L8GMG7]	0.011517367	0.315646067	0.3041287	5.24E-08
3h	ACA1_091340/clean/scf7180000084645:94393-94473:+	ACA1_091340	UDENN domain-containing protein [Source:UniProtKB/TrEMBL;Acc:L8GIG9]	0.040055964	0.344002	0.303946036	0.00223941
3h	ACA1_107640/clean/scf7180000084660:238691-238901:+	ACA1_107640	Signal transduction histidine kinase [Source:UniProtKB/TrEMBL;Acc:L8GNL7]	0.015488807	0.319421733	0.303932927	5.23E-05
3h	ACA1_362790/clean/scf7180000084624:182483-182743:-	ACA1_362790	Protein kinase domain containing protein [Source:UniProtKB/TrEMBL;Acc:L8GFL4]	0.056629467	0.360500333	0.303870867	0.007046696
3h	ACA1_129940/clean/scf7180000084698:52867-52973:+	ACA1_129940	LisH domain-containing protein [Source:UniProtKB/TrEMBL;Acc:L8GNX2]	0.028959967	0.332820667	0.3038607	0.000761052
3h	ACA1_381510/clean/scf7180000084712:167057-167139:+	ACA1_381510	Pyr_redox_2 domain-containing protein [Source:UniProtKB/TrEMBL;Acc:L8GNN7]	0.0504242	0.354272	0.3038478	0.000976415
3h	ACA1_184620/clean/scf7180000084746:10660-10778:+	ACA1_184620	Nudix hydrolase domain-containing protein [Source:UniProtKB/TrEMBL;Acc:L8H551]	0.012152253	0.315983333	0.30383108	5.32E-08
3h	ACA1_306840/clean/scf7180000083668:17866-17944:+	ACA1_306840	RTP1_C1 domain-containing protein [Source:UniProtKB/TrEMBL;Acc:L8GQF6]	0	0.3037998	0.3037998	0.000152488
3h	ACA1_031850/clean/scf7180000084523:21117-21277:-	ACA1_031850	Proteintyrosine phosphatase [Source:UniProtKB/TrEMBL;Acc:L8H408]	0.00388075	0.307655567	0.303774817	1.04E-05
3h	ACA1_368250/clean/scf7180000084664:89808-89880:-	ACA1_368250	BPI2 domain-containing protein [Source:UniProtKB/TrEMBL;Acc:L8GY12]	0.001899713	0.3056342	0.303734487	2.01E-06
3h	ACA1_088090/clean/scf7180000084639:156806-156866:-	ACA1_088090	Retinol dehydrogenase 14 (All-trans and 9-cis) family protein [Source:UniProtKB/TrEMBL;Acc:L8GTX6]	0.007710057	0.311426333	0.303716277	0.000387137
3h	ACA1_206050/clean/scf7180000084755:71084-71165:+	ACA1_206050	ANK_REP_REGION domain-containing protein [Source:UniProtKB/TrEMBL;Acc:L8H7K6]	0.036879433	0.34049	0.303610567	0.000105713
3h	ACA1_002450/clean/scf7180000082931:3186-3246:+	ACA1_002450	eIF-5a domain-containing protein [Source:UniProtKB/TrEMBL;Acc:L8GWN9]	0.056985633	0.360412333	0.3034267	0.004146942
3h	ACA1_054820/clean/scf7180000084594:578865-578942:-	ACA1_054820	Tetratricopeptide repeat domain containing protein [Source:UniProtKB/TrEMBL;Acc:L8H8E7]	0.00067323	0.304032333	0.303359103	3.04E-06
3h	ACA1_100610/clean/scf7180000084648:133838-133937:-	ACA1_100610	Serine/threonine kinase [Source:UniProtKB/TrEMBL;Acc:L8GK64]	0.000592476	0.303905033	0.303312558	4.22E-07
3h	ACA1_399200/clean/scf7180000084759:271147-271248:-	ACA1_399200	hypothetical protein	0.001041667	0.304277333	0.303235667	1.11E-07
3h	ACA1_063730/clean/scf7180000084599:1488428-1488504:-	ACA1_063730	Tetratricopeptide repeat domain containing protein [Source:UniProtKB/TrEMBL;Acc:L8GWN4]	0.108924333	0.411956333	0.303032	0.001725123
3h	ACA1_199610/clean/scf7180000084753:273580-273677:+	ACA1_199610	hypothetical protein	0.0207326	0.323722033	0.302989433	1.86E-05
3h	ACA1_199290/clean/scf7180000084753:217280-217373:+	ACA1_199290	Rho-GAP domain-containing protein [Source:UniProtKB/TrEMBL;Acc:L8H347]	0.002079847	0.305039333	0.302959487	8.26E-06
3h	ACA1_181600/clean/scf7180000084745:25457-25524:+	ACA1_181600	ACB domain-containing protein [Source:UniProtKB/TrEMBL;Acc:L8HA17]	0.00987137	0.312597667	0.302726297	3.89E-08
3h	ACA1_129350/clean/scf7180000084698:9764-9826:+	ACA1_129350	Fatty acid hydroxylase domain-containing protein [Source:UniProtKB/TrEMBL;Acc:L8GPA5]	0.006486911	0.309154667	0.302667755	7.68E-08
3h	ACA1_271500/clean/scf7180000084771:43397-43551:+	ACA1_271500	Leucine rich repeat domain containing protein [Source:UniProtKB/TrEMBL;Acc:L8GNI4]	0.007128833	0.309794033	0.3026652	2.07E-05
3h	ACA1_144200/clean/scf7180000084709:169867-170034:+	ACA1_144200	PH domain-containing protein [Source:UniProtKB/TrEMBL;Acc:L8HET7]	0.0038249	0.3064582	0.3026333	1.63E-10
3h	ACA1_176030/clean/scf7180000084741:1108424-1108510:-	ACA1_176030	ATPase, AAA domain containing protein [Source:UniProtKB/TrEMBL;Acc:L8HI90]	0.0087204	0.311326767	0.302606367	0.00227114
3h	ACA1_189590/clean/scf7180000084748:56075-56133:+	ACA1_189590	Transcriptional regulatorrelated, putative [Source:UniProtKB/TrEMBL;Acc:L8GEX7]	0.008367967	0.310766333	0.302398367	1.86E-07
3h	ACA1_383070/clean/scf7180000084721:135091-135159:+	ACA1_383070	CBS domain containing protein [Source:UniProtKB/TrEMBL;Acc:L8GXI5]	0.01347238	0.315761667	0.302289287	0.000210147
3h	ACA1_054120/clean/scf7180000084594:407275-407419:-	ACA1_054120	Protein kinase domain-containing protein [Source:UniProtKB/TrEMBL;Acc:L8H625]	0.038417167	0.340513333	0.302096167	0.007463279
3h	ACA1_381620/clean/scf7180000084712:198143-198217:+	ACA1_381620	Alpha-galactosidase [Source:UniProtKB/TrEMBL;Acc:L8GMY9]	0.001473907	0.303444133	0.301970227	8.90E-13
3h	ACA1_219740/clean/scf7180000084758:797512-797595:+	ACA1_219740	Glucosamine 6-phosphate N-acetyltransferase [Source:UniProtKB/TrEMBL;Acc:L8GRH7]	0.014103567	0.3160518	0.301948233	8.58E-05
3h	ACA1_277560/clean/scf7180000084776:74974-75066:-	ACA1_277560	F-box domain-containing protein [Source:UniProtKB/TrEMBL;Acc:L8H6G6]	0.00820141	0.310128	0.30192659	0.000153781
3h	ACA1_073230/clean/scf7180000084603:1375727-1375799:-	ACA1_073230	EHN domain-containing protein [Source:UniProtKB/TrEMBL;Acc:L8HDT9]	0.002688173	0.304545567	0.301857393	1.49E-05
3h	ACA1_151100/clean/scf7180000084716:55212-55306:-	ACA1_151100	NFACT-R_1 domain-containing protein [Source:UniProtKB/TrEMBL;Acc:L8H219]	0.000154036	0.301825333	0.301671298	3.52E-07
3h	ACA1_360060/clean/scf7180000084622:40068-40138:-	ACA1_360060	GHMP_kinases_C domain-containing protein [Source:UniProtKB/TrEMBL;Acc:L8HF07]	0.0221538	0.323825	0.3016712	7.61E-06
3h	ACA1_054180/clean/scf7180000084594:420012-420095:+	ACA1_054180	hypothetical protein	0.0235997	0.325260333	0.301660633	1.06E-06
3h	ACA1_229940/clean/scf7180000084761:312821-312872:+	ACA1_229940	Arf GTPase activating protein, putative [Source:UniProtKB/TrEMBL;Acc:L8H819]	0.072670467	0.374274	0.301603533	0.000119535
3h	ACA1_262400/clean/scf7180000084770:43217-43302:+	ACA1_262400	Probable tRNA N6-adenosine threonylcarbamoyltransferase [Source:UniProtKB/TrEMBL;Acc:L8H1V9]	0.000236626	0.301827333	0.301590707	3.23E-09
3h	ACA1_389630/clean/scf7180000084733:299426-299505:+	ACA1_389630	F-box domain-containing protein [Source:UniProtKB/TrEMBL;Acc:L8GDY9]	0.0257587	0.327318633	0.301559933	2.20E-05
3h	ACA1_201160/clean/scf7180000084753:495733-495824:-	ACA1_201160	RasGEF domain containing protein [Source:UniProtKB/TrEMBL;Acc:L8H4V2]	0	0.301538333	0.301538333	2.48E-07
3h	ACA1_051450/clean/scf7180000084592:17304-17397:-	ACA1_051450	TMEM135_C_rich domain-containing protein [Source:UniProtKB/TrEMBL;Acc:L8GQJ0]	0.0726683	0.374081	0.3014127	0.03797319
3h	ACA1_065450/clean/scf7180000084599:1856985-1857067:-	ACA1_065450	Cation_ATPase_N domain-containing protein [Source:UniProtKB/TrEMBL;Acc:L8H037]	0	0.301394067	0.301394067	0.039994555
3h	ACA1_131740/clean/scf7180000084699:112916-112993:+	ACA1_131740	Carrier domain-containing protein [Source:UniProtKB/TrEMBL;Acc:L8GPY2]	0.000641321	0.302015333	0.301374012	2.07E-07
3h	ACA1_144360/clean/scf7180000084709:214827-214999:+	ACA1_144360	Repressor of RNA polymerase III transcription [Source:UniProtKB/TrEMBL;Acc:L8HFX0]	0.005735583	0.306974733	0.30123915	2.66E-09
3h	ACA1_072290/clean/scf7180000084603:1191686-1191771:-	ACA1_072290	Endo/exonuclease/phosphatase domain-containing protein [Source:UniProtKB/TrEMBL;Acc:L8HEG9]	0.146428667	0.447643333	0.301214667	0.026972503
3h	ACA1_380970/clean/scf7180000084712:132951-133038:+	ACA1_380970	ANK_REP_REGION domain-containing protein [Source:UniProtKB/TrEMBL;Acc:L8GMX1]	0.00120373	0.302407667	0.301203937	2.33E-05
3h	ACA1_390940/clean/scf7180000084740:114969-115055:+	ACA1_390940	Zn(2)-C6 fungal-type domain-containing protein [Source:UniProtKB/TrEMBL;Acc:L8GPB9]	0	0.301196267	0.301196267	1.43E-08
3h	ACA1_069620/clean/scf7180000084603:487539-487645:+	ACA1_069620	Poly [ADP-ribose] polymerase [Source:UniProtKB/TrEMBL;Acc:L8HDR5]	0.0624733	0.363644667	0.301171367	0.000636614
3h	ACA1_057360/clean/scf7180000084599:123236-123301:+	ACA1_057360	Mitochondrial inner membrane protease subunit [Source:UniProtKB/TrEMBL;Acc:L8GYF2]	0.012776657	0.313939	0.301162343	0.000505758
3h	ACA1_063890/clean/scf7180000084599:1527245-1527337:+	ACA1_063890	DAO domain-containing protein [Source:UniProtKB/TrEMBL;Acc:L8GXC2]	0.003826827	0.304925467	0.30109864	1.31E-05
3h	ACA1_298630/clean/scf7180000082992:5235-5346:-	ACA1_298630	Vacuolar protein sorting 16, putative [Source:UniProtKB/TrEMBL;Acc:L8H081]	0	0.301059467	0.301059467	4.41E-08
3h	ACA1_116740/clean/scf7180000084669:839696-839776:+	ACA1_116740	Methyltransf_2 domain-containing protein [Source:UniProtKB/TrEMBL;Acc:L8H4R1]	0.041276133	0.342252667	0.300976533	0.001615283
3h	ACA1_285540/clean/scf7180000084776:991795-991866:-	ACA1_285540	GPS domain-containing protein [Source:UniProtKB/TrEMBL;Acc:L8H7T4]	0.002426837	0.303364867	0.30093803	2.25E-05
3h	ACA1_123500/clean/scf7180000084686:32796-32893:+	ACA1_123500	hypothetical protein	0.069792333	0.370722667	0.300930333	0.007910415
3h	ACA1_215970/clean/scf7180000084758:419093-419167:-	ACA1_215970	EGFlike domain containing protein [Source:UniProtKB/TrEMBL;Acc:L8GQ73]	0	0.300755333	0.300755333	0.000292567
3h	ACA1_233020/clean/scf7180000084762:105959-106078:-	ACA1_233020	hypothetical protein	0.009358937	0.310093	0.300734063	1.15E-07
3h	ACA1_209530/clean/scf7180000084757:303998-304079:-	ACA1_209530	Purple acid phosphatase [Source:UniProtKB/TrEMBL;Acc:L8GZQ1]	0.302753333	0.603429667	0.300676333	0.02577576
3h	ACA1_048490/clean/scf7180000084582:123238-123310:+	ACA1_048490	Coatomer subunit alpha [Source:UniProtKB/TrEMBL;Acc:L8GEB3]	0.00348499	0.304119667	0.300634677	2.64E-07
3h	ACA1_044480/clean/scf7180000084576:28308-28377:-	ACA1_044480	Allergen V5/Tpx1 family protein [Source:UniProtKB/TrEMBL;Acc:L8H123]	0.002767043	0.303376333	0.30060929	3.45E-06
3h	ACA1_151100/clean/scf7180000084716:56601-56788:-	ACA1_151100	NFACT-R_1 domain-containing protein [Source:UniProtKB/TrEMBL;Acc:L8H219]	0.005291	0.305895667	0.300604667	3.40E-05
3h	ACA1_382630/clean/scf7180000084721:89493-89603:-	ACA1_382630	LBP / BPI / CETP family, Cterminal domain containing protein [Source:UniProtKB/TrEMBL;Acc:L8GVZ7]	0.003081639	0.3036661	0.300584461	1.15E-09
3h	ACA1_119230/clean/scf7180000084672:48362-48431:+	ACA1_119230	Armadillo/betacatenin-like repeat domain containing protein [Source:UniProtKB/TrEMBL;Acc:L8HMP1]	0.008197661	0.3086173	0.300419639	2.29E-07
3h	ACA1_054180/clean/scf7180000084594:419404-419581:+	ACA1_054180	hypothetical protein	0.00707952	0.307455333	0.300375813	1.07E-06
3h	ACA1_080550/clean/scf7180000084618:30788-30879:+	ACA1_080550	FAD binding domain containing protein [Source:UniProtKB/TrEMBL;Acc:L8HBU6]	0.0140419	0.314290567	0.300248667	5.81E-05
3h	ACA1_383610/clean/scf7180000084721:226599-226735:+	ACA1_383610	PAKA subfamily protein kinase [Source:UniProtKB/TrEMBL;Acc:L8GW48]	0	0.300208333	0.300208333	5.12E-10
3h	ACA1_147520/clean/scf7180000084714:29030-29117:+	ACA1_147520	RanBP2-type domain-containing protein [Source:UniProtKB/TrEMBL;Acc:L8GKJ7]	0.000968993	0.301151667	0.300182673	1.11E-05
3h	ACA1_039640/clean/scf7180000084570:10422-10497:+	ACA1_039640	hypothetical protein	0.0110307	0.311176667	0.300145967	8.55E-06
3h	ACA1_054410/clean/scf7180000084594:486444-486559:-	ACA1_054410	Dual specificity protein kinase [Source:UniProtKB/TrEMBL;Acc:L8H6D1]	0.021189067	0.321288333	0.300099267	7.11E-06
3h	ACA1_310750/clean/scf7180000083780:6657-6735:+	ACA1_310750	RFX-type winged-helix domain-containing protein [Source:UniProtKB/TrEMBL;Acc:L8GVY4]	0	0.2999935	0.2999935	2.44E-05
3h	ACA1_258270/clean/scf7180000084769:216391-216534:+	ACA1_258270	Ras subfamily protein [Source:UniProtKB/TrEMBL;Acc:L8GHB6]	0.00605407	0.306038	0.29998393	1.33E-05
3h	ACA1_044480/clean/scf7180000084576:26833-26935:-	ACA1_044480	Allergen V5/Tpx1 family protein [Source:UniProtKB/TrEMBL;Acc:L8H123]	0	0.299949333	0.299949333	8.99E-08
3h	ACA1_380980/clean/scf7180000084712:139030-139104:-	ACA1_380980	Phosphoinositide 3kinase family, accessory domain (Pik domain) domain containing protein [Source:UniProtKB/TrEMBL;Acc:L8GQK9]	0.002220983	0.301953	0.299732017	5.58E-08
3h	ACA1_283960/clean/scf7180000084776:884502-884610:-	ACA1_283960	hypothetical protein	0.003513613	0.303240833	0.29972722	1.42E-09
3h	ACA1_034530/clean/scf7180000084563:64912-64984:+	ACA1_034530	hypothetical protein	0.006273213	0.305957333	0.29968412	3.24E-05
3h	ACA1_106280/clean/scf7180000084660:68813-68940:-	ACA1_106280	hypothetical protein	0.00756897	0.307074667	0.299505697	1.08E-11
3h	ACA1_189420/clean/scf7180000084748:34009-34110:+	ACA1_189420	Protein disulfideisomerase domain containing protein [Source:UniProtKB/TrEMBL;Acc:L8GE28]	0.0212108	0.320677333	0.299466533	3.65E-06
3h	ACA1_298650/clean/scf7180000082992:10347-10471:+	ACA1_298650	hypothetical protein	0.001495857	0.300950733	0.299454877	4.72E-05
3h	ACA1_058840/clean/scf7180000084599:393402-393492:-	ACA1_058840	HATPase_c domain-containing protein [Source:UniProtKB/TrEMBL;Acc:L8GXB7]	0.004405367	0.303676	0.299270633	9.71E-11
3h	ACA1_189890/clean/scf7180000084748:107335-107408:-	ACA1_189890	Usp domain-containing protein [Source:UniProtKB/TrEMBL;Acc:L8GEZ9]	0.001722988	0.300836667	0.299113679	1.50E-08
3h	ACA1_061700/clean/scf7180000084599:1013449-1013522:+	ACA1_061700	Amine oxidase family protein [Source:UniProtKB/TrEMBL;Acc:L8GWP7]	0.000920303	0.300017833	0.29909753	4.02E-07
3h	ACA1_074110/clean/scf7180000084605:29861-29969:-	ACA1_074110	Peptidase [Source:UniProtKB/TrEMBL;Acc:L8HJ70]	0.00067408	0.299766833	0.299092753	1.17E-07
3h	ACA1_209110/clean/scf7180000084757:273278-273360:+	ACA1_209110	hypothetical protein	0.021366267	0.320440333	0.299074067	0.000262617
3h	ACA1_226710/clean/scf7180000084761:60425-60508:-	ACA1_226710	hypothetical protein	0.009259267	0.3082893	0.299030033	0.013874744
3h	ACA1_178750/clean/scf7180000084743:275863-275953:-	ACA1_178750	PCI domain-containing protein [Source:UniProtKB/TrEMBL;Acc:L8GVV1]	0.001838764	0.300868267	0.299029503	7.27E-08
3h	ACA1_066150/clean/scf7180000084599:2012380-2012448:+	ACA1_066150	Myosin head (Motor domain) domain containing protein [Source:UniProtKB/TrEMBL;Acc:L8GX00]	0	0.298910367	0.298910367	0.004008222
3h	ACA1_072330/clean/scf7180000084603:1199537-1199608:-	ACA1_072330	UBA_e1_C domain-containing protein [Source:UniProtKB/TrEMBL;Acc:L8HGS4]	0.007383893	0.306280333	0.29889644	2.63E-07
3h	ACA1_400300/clean/scf7180000084775:142648-142729:+	ACA1_400300	UBIQUITIN_CONJUGAT_2 domain-containing protein [Source:UniProtKB/TrEMBL;Acc:L8GGI3]	0.015873	0.314743667	0.298870667	0.004088388
3h	ACA1_137270/clean/scf7180000084703:29286-29369:-	ACA1_137270	hypothetical protein	0	0.298841333	0.298841333	0.000222583
3h	ACA1_237850/clean/scf7180000084763:31919-32056:+	ACA1_237850	BTB/POZ domain containing protein [Source:UniProtKB/TrEMBL;Acc:L8GJT8]	0.024926267	0.323754667	0.2988284	3.66E-06
3h	ACA1_296270/clean/scf7180000084777:1308665-1308777:-	ACA1_296270	Deoxyribosephosphate aldolase [Source:UniProtKB/TrEMBL;Acc:L8HIU4]	0.009044683	0.307761	0.298716317	8.76E-08
3h	ACA1_381570/clean/scf7180000084712:184501-184559:+	ACA1_381570	Ras-GAP domain-containing protein [Source:UniProtKB/TrEMBL;Acc:L8GMY5]	0.0491101	0.347719	0.2986089	4.42E-05
3h	ACA1_123220/clean/scf7180000084686:5779-5875:-	ACA1_123220	hypothetical protein	0	0.298604	0.298604	9.11E-06
3h	ACA1_074800/clean/scf7180000084608:86967-87038:-	ACA1_074800	Vesicle transport protein [Source:UniProtKB/TrEMBL;Acc:L8HGS3]	0.087839533	0.386344667	0.298505133	0.001137822
3h	ACA1_282710/clean/scf7180000084776:721287-721373:+	ACA1_282710	Cyclic nucleotide-binding domain-containing protein [Source:UniProtKB/TrEMBL;Acc:L8H7K2]	0.018796847	0.317288	0.298491153	0.003879765
3h	ACA1_074780/clean/scf7180000084608:84988-85053:+	ACA1_074780	Rho-GAP domain-containing protein [Source:UniProtKB/TrEMBL;Acc:L8HHI0]	8.95E-05	0.298492667	0.298403201	2.44E-08
3h	ACA1_164640/clean/scf7180000084729:238935-239007:+	ACA1_164640	hypothetical protein	0.0051748	0.303540433	0.298365633	1.07E-08
3h	ACA1_033610/clean/scf7180000084559:58769-58849:+	ACA1_033610	Sortilin [Source:UniProtKB/TrEMBL;Acc:L8GHJ0]	0	0.298309867	0.298309867	3.50E-08
3h	ACA1_280270/clean/scf7180000084776:469320-469412:-	ACA1_280270	Lipase_GDSL domain-containing protein [Source:UniProtKB/TrEMBL;Acc:L8H655]	0.00226213	0.300515667	0.298253537	3.68E-07
3h	ACA1_183130/clean/scf7180000084745:338433-338503:-	ACA1_183130	PNP_UDP_1 domain-containing protein [Source:UniProtKB/TrEMBL;Acc:L8H767]	0.155396	0.453552667	0.298156667	0.002445319
3h	ACA1_395210/clean/scf7180000084756:155873-155953:-	ACA1_395210	Nonspecific lipidtransfer protein [Source:UniProtKB/TrEMBL;Acc:L8GZN6]	0	0.2981491	0.2981491	2.74E-05
3h	ACA1_287940/clean/scf7180000084777:128708-128798:-	ACA1_287940	Conditioned medium factor, putative [Source:UniProtKB/TrEMBL;Acc:L8HKB9]	0.00263529	0.300563667	0.297928377	4.61E-12
3h	ACA1_349670/clean/scf7180000084690:34494-34588:-	ACA1_349670	hypothetical protein	0.012331311	0.310252333	0.297921022	2.16E-06
3h	ACA1_061490/clean/scf7180000084599:941501-941591:-	ACA1_061490	FHA domain-containing protein [Source:UniProtKB/TrEMBL;Acc:L8GW04]	0	0.2978971	0.2978971	4.72E-07
3h	ACA1_338040/clean/scf7180000084604:33254-33331:+	ACA1_338040	Peptidase S74 domain-containing protein [Source:UniProtKB/TrEMBL;Acc:L8GD03]	0.0224999	0.3203	0.2978001	0.000122579
3h	ACA1_216320/clean/scf7180000084758:466607-466719:+	ACA1_216320	Homogentisate 1,2dioxygenase [Source:UniProtKB/TrEMBL;Acc:L8GQ83]	0.00484654	0.302642	0.29779546	1.20E-08
3h	ACA1_377350/clean/scf7180000084711:40994-41075:+	ACA1_377350	YDG/SRA domain containing protein [Source:UniProtKB/TrEMBL;Acc:L8GU64]	0.017241367	0.314989333	0.297747967	0.000129908
3h	ACA1_262400/clean/scf7180000084770:43571-43671:+	ACA1_262400	Probable tRNA N6-adenosine threonylcarbamoyltransferase [Source:UniProtKB/TrEMBL;Acc:L8H1V9]	0.001803604	0.299212	0.297408396	3.89E-07
3h	ACA1_380750/clean/scf7180000084712:82030-82146:-	ACA1_380750	hypothetical protein	0	0.297382667	0.297382667	4.12E-07
3h	ACA1_174540/clean/scf7180000084741:732033-732145:-	ACA1_174540	Fucosylgalactoside 3-alpha-galactosyltransferase [Source:UniProtKB/TrEMBL;Acc:L8HJC5]	0.0084827	0.305807167	0.297324467	1.57E-05
3h	ACA1_279530/clean/scf7180000084776:410311-410391:+	ACA1_279530	Rab4, putative [Source:UniProtKB/TrEMBL;Acc:L8H946]	0.007106333	0.304303467	0.297197133	1.09E-06

3h	ACA1_322390/clean/scf7180000084463:17898-17985:+	ACA1_322390	DIL domain containing protein [Source:UniProtKB/TrEMBL;Acc:L8HH10]	0.0277366	0.324735	0.2969984	3.60E-05
3h	ACA1_189300/clean/scf7180000084748:823-910:+	ACA1_189300	hypothetical protein	0.028205633	0.325111667	0.296906033	4.13E-06
3h	ACA1_091560/clean/scf7180000084645:149958-150040:+	ACA1_091560	UBP-type domain-containing protein [Source:UniProtKB/TrEMBL;Acc:L8GKE3]	0.02006852	0.316966467	0.296897947	0.002569054
3h	ACA1_035470/clean/scf7180000084564:59220-59289:+	ACA1_035470	Cation exchanger, putative [Source:UniProtKB/TrEMBL;Acc:L8HAF4]	0	0.296802333	0.296802333	1.61E-08
3h	ACA1_367310/clean/scf7180000084641:596383-596464:-	ACA1_367310	CCHC-type domain-containing protein [Source:UniProtKB/TrEMBL;Acc:L8GMZ2]	0.016751367	0.313431767	0.2966804	0.024328893
3h	ACA1_145790/clean/scf7180000084710:147088-147257:+	ACA1_145790	hypothetical protein	0.0049114	0.301585867	0.296674467	3.07E-07
3h	ACA1_064190/clean/scf7180000084599:1553016-1553086:+	ACA1_064190	hypothetical protein	0.00425084	0.3008964	0.29664556	1.55E-10
3h	ACA1_164910/clean/scf7180000084729:308905-308976:-	ACA1_164910	hypothetical protein	0.048595633	0.345191	0.296595367	8.46E-05
3h	ACA1_300890/clean/scf7180000083147:17144-17221:-	ACA1_300890	hypothetical protein	0.004119291	0.300710333	0.296591043	2.87E-08
3h	ACA1_024380/clean/scf7180000084442:8536-8623:-	ACA1_024380	Myosin1, putative [Source:UniProtKB/TrEMBL;Acc:L8H7M2]	0.00098776	0.297481733	0.296493973	8.96E-08
3h	ACA1_289610/clean/scf7180000084777:431001-431070:+	ACA1_289610	Copper transporter [Source:UniProtKB/TrEMBL;Acc:L8HKL4]	0.01146197	0.307950533	0.296488563	8.60E-08
3h	ACA1_113870/clean/scf7180000084669:392913-392999:+	ACA1_113870	CUB domain containing protein [Source:UniProtKB/TrEMBL;Acc:L8H6F7]	0.009367817	0.305723733	0.296355917	1.21E-05
3h	ACA1_269730/clean/scf7180000084770:1029176-1029256:+	ACA1_269730	Metallophos domain-containing protein [Source:UniProtKB/TrEMBL;Acc:L8H506]	0.003301	0.299515267	0.296214267	2.10E-07
3h	ACA1_108220/clean/scf7180000084661:17705-17793:+	ACA1_108220	hypothetical protein	0.049536433	0.3457189	0.296182467	0.008840251
3h	ACA1_068740/clean/scf7180000084603:267386-267468:+	ACA1_068740	tRNAdihydrouridine synthase A, putative [Source:UniProtKB/TrEMBL;Acc:L8HDM1]	0	0.296123467	0.296123467	0.005422283
3h	ACA1_098400/clean/scf7180000084646:3005-3124:+	ACA1_098400	MFS domain-containing protein [Source:UniProtKB/TrEMBL;Acc:L8H5Q6]	0.079888167	0.375980667	0.2960925	0.000725786
3h	ACA1_233070/clean/scf7180000084762:118616-118695:+	ACA1_233070	hypothetical protein	0.026514567	0.322586667	0.2960721	1.74E-05
3h	ACA1_012980/clean/scf7180000083742:15172-15248:-	ACA1_012980	Endo/exonuclease/phosphatase domain-containing protein [Source:UniProtKB/TrEMBL;Acc:L8H4W5]	0.013113927	0.309021	0.295907073	6.24E-05
3h	ACA1_285200/clean/scf7180000084776:956203-956288:-	ACA1_285200	Na_H_Exchanger domain-containing protein [Source:UniProtKB/TrEMBL;Acc:L8H9U2]	0.003116037	0.298961833	0.295845797	8.37E-07
3h	ACA1_158530/clean/scf7180000084724:245296-245369:+	ACA1_158530	Glucose-6-phosphate 1-dehydrogenase [Source:UniProtKB/TrEMBL;Acc:L8HBJ1]	0	0.295832733	0.295832733	0.000737821
3h	ACA1_144880/clean/scf7180000084710:31420-31538:-	ACA1_144880	hypothetical protein	0.0017452	0.297569933	0.295824733	6.07E-05
3h	ACA1_221050/clean/scf7180000084758:986656-986755:-	ACA1_221050	hypothetical protein	0.0320922	0.3278557	0.2957635	0.001056851
3h	ACA1_113560/clean/scf7180000084669:319114-319288:-	ACA1_113560	PH domain containing protein [Source:UniProtKB/TrEMBL;Acc:L8H6B7]	0.015794137	0.311490333	0.295696197	0.000126812
3h	ACA1_149410/clean/scf7180000084715:145848-145989:+	ACA1_149410	Lectin_legB domain-containing protein [Source:UniProtKB/TrEMBL;Acc:L8HDD6]	0.000967837	0.296623367	0.29565553	1.32E-07
3h	ACA1_113860/clean/scf7180000084669:390316-390439:+	ACA1_113860	Saposin B-type domain-containing protein [Source:UniProtKB/TrEMBL;Acc:L8H4D7]	0.00595623	0.301527333	0.295571103	2.82E-07
3h	ACA1_113560/clean/scf7180000084669:322062-322225:-	ACA1_113560	PH domain containing protein [Source:UniProtKB/TrEMBL;Acc:L8H6B7]	0.0570241	0.352474	0.2954499	0.000471763
3h	ACA1_174630/clean/scf7180000084741:751856-751961:+	ACA1_174630	Aminotran_1_2 domain-containing protein [Source:UniProtKB/TrEMBL;Acc:L8HHW2]	0	0.295394333	0.295394333	2.35E-07
3h	ACA1_166080/clean/scf7180000084732:116092-116165:+	ACA1_166080	hypothetical protein	0.0111871	0.306377667	0.295190567	4.15E-07
3h	ACA1_126140/clean/scf7180000084693:28839-28907:-	ACA1_126140	HA domain-containing protein [Source:UniProtKB/TrEMBL;Acc:L8HAG8]	0.006567367	0.3017391	0.295171733	0.00017353
3h	ACA1_133410/clean/scf7180000084701:48569-48649:-	ACA1_133410	EF hand domain containing protein [Source:UniProtKB/TrEMBL;Acc:L8H515]	0.0106618	0.305820967	0.295159167	2.22E-06
3h	ACA1_397190/clean/scf7180000084759:168159-168237:+	ACA1_397190	Metallophos domain-containing protein [Source:UniProtKB/TrEMBL;Acc:L8HDM9]	0.002081407	0.297160933	0.295079527	2.74E-05
3h	ACA1_362610/clean/scf7180000084624:112600-112679:-	ACA1_362610	Leucine rich repeat domain containing protein [Source:UniProtKB/TrEMBL;Acc:L8GFJ8]	0.0125745	0.307592333	0.295017833	5.33E-05
3h	ACA1_188190/clean/scf7180000084747:123733-123800:-	ACA1_188190	Calponin-homology (CH) domain-containing protein [Source:UniProtKB/TrEMBL;Acc:L8GSF5]	0.021612467	0.316621333	0.295008867	3.38E-05
3h	ACA1_315790/clean/scf7180000083943:8870-8950:-	ACA1_315790	Copper transport accessory protein [Source:UniProtKB/TrEMBL;Acc:L8HDX3]	0.016122133	0.311094667	0.294972533	3.43E-05
3h	ACA1_256380/clean/scf7180000084769:13976-14109:-	ACA1_256380	UBA/TS-N domain containing protein [Source:UniProtKB/TrEMBL;Acc:L8GF49]	0.0231008	0.318068333	0.294967533	0.000589323
3h	ACA1_191850/clean/scf7180000084749:57923-57993:-	ACA1_191850	Protein kinase domain-containing protein [Source:UniProtKB/TrEMBL;Acc:L8GN20]	0.024329067	0.319266033	0.294936967	2.20E-05
3h	ACA1_185320/clean/scf7180000084746:92795-92907:+	ACA1_185320	hypothetical protein	0.000180501	0.29507	0.294889499	3.60E-09
3h	ACA1_268540/clean/scf7180000084770:908225-908334:+	ACA1_268540	Ras-GEF domain-containing protein [Source:UniProtKB/TrEMBL;Acc:L8H452]	0.02630848	0.3211337	0.29482522	0.000696052
3h	ACA1_384320/clean/scf7180000084730:27055-27127:+	ACA1_384320	Glycosyl hydrolases family 25 subfamily protein [Source:UniProtKB/TrEMBL;Acc:L8H880]	0	0.294737	0.294737	0.020792243
3h	ACA1_091880/clean/scf7180000084645:220382-220450:+	ACA1_091880	Ubiquitin-like domain-containing protein [Source:UniProtKB/TrEMBL;Acc:L8GIV5]	0.003832733	0.298530433	0.2946977	2.58E-06
3h	ACA1_074110/clean/scf7180000084605:26757-26862:-	ACA1_074110	Peptidase [Source:UniProtKB/TrEMBL;Acc:L8HJ70]	0.00171561	0.296408467	0.294692856	2.43E-07
3h	ACA1_285540/clean/scf7180000084776:990653-990744:-	ACA1_285540	GPS domain-containing protein [Source:UniProtKB/TrEMBL;Acc:L8H7T4]	0.003394433	0.297972633	0.2945782	0.000102468
3h	ACA1_172870/clean/scf7180000084741:343494-343576:-	ACA1_172870	Nadph-dependent carbonyl reductase family protein [Source:UniProtKB/TrEMBL;Acc:L8HHL8]	0.01264786	0.307192333	0.294544473	5.49E-07
3h	ACA1_218390/clean/scf7180000084758:649877-649960:-	ACA1_218390	Glycosyl hydrolase family 20, catalytic domain containing protein [Source:UniProtKB/TrEMBL;Acc:L8GQD2]	0.003663	0.2981634	0.2945004	3.19E-05
3h	ACA1_370080/clean/scf7180000084664:433686-433787:-	ACA1_370080	ATPdependent RNA helicase Ddx1, putative [Source:UniProtKB/TrEMBL;Acc:L8GZ36]	0.000630357	0.295069	0.294438643	4.21E-05
3h	ACA1_372580/clean/scf7180000084679:16554-16631:-	ACA1_372580	Lipase [Source:UniProtKB/TrEMBL;Acc:L8GHE3]	0.029667133	0.3240885	0.294421367	0.000627019
3h	ACA1_019610/clean/scf7180000084011:20000-20098:-	ACA1_019610	FHA domain-containing protein [Source:UniProtKB/TrEMBL;Acc:L8GQM8]	0.0135975	0.3080156	0.2944181	8.13E-05
3h	ACA1_202770/clean/scf7180000084754:106342-106433:-	ACA1_202770	TPR_REGION domain-containing protein [Source:UniProtKB/TrEMBL;Acc:L8GSW6]	0.016136733	0.3105026	0.294365867	2.15E-05
3h	ACA1_121840/clean/scf7180000084682:110630-110689:+	ACA1_121840	Protein kinase domain-containing protein [Source:UniProtKB/TrEMBL;Acc:L8GFF3]	0.311167	0.60553	0.294363	0.009507682
3h	ACA1_250300/clean/scf7180000084767:43942-44026:+	ACA1_250300	Carrier superfamily protein [Source:UniProtKB/TrEMBL;Acc:L8HFF4]	0.000102344	0.294443667	0.294341323	5.17E-06
3h	ACA1_114530/clean/scf7180000084669:499409-499485:+	ACA1_114530	NB-ARC domain containing protein [Source:UniProtKB/TrEMBL;Acc:L8H3K3]	0.009694623	0.303868967	0.294174343	0.000595015
3h	ACA1_264320/clean/scf7180000084770:235487-235565:-	ACA1_264320	PH domain-containing protein [Source:UniProtKB/TrEMBL;Acc:L8H1Z3]	0	0.294151	0.294151	2.82E-06
3h	ACA1_013340/clean/scf7180000083750:376-456:+	ACA1_013340	PH domain-containing protein [Source:UniProtKB/TrEMBL;Acc:L8HGJ7]	0	0.294122167	0.294122167	3.58E-08
3h	ACA1_092200/clean/scf7180000084645:299129-299587:-	ACA1_092200	RFX-type winged-helix domain-containing protein [Source:UniProtKB/TrEMBL;Acc:L8GI10]	0.016588043	0.310684933	0.29409689	0.000696052
3h	ACA1_368920/clean/scf7180000084664:197754-197822:+	ACA1_368920	MFS domain-containing protein [Source:UniProtKB/TrEMBL;Acc:L8GYV9]	0	0.293635	0.293635	4.99E-07
3h	ACA1_218390/clean/scf7180000084758:651633-651713:-	ACA1_218390	Glycosyl hydrolase family 20, catalytic domain containing protein [Source:UniProtKB/TrEMBL;Acc:L8GQD2]	0.00098912	0.2945667	0.29357758	1.81E-06
3h	ACA1_066870/clean/scf7180000084600:68121-68203:-	ACA1_066870	Glucose-6-phosphate isomerase [Source:UniProtKB/TrEMBL;Acc:L8GRA3]	0.00218318	0.295750567	0.293567387	9.30E-08
3h	ACA1_068120/clean/scf7180000084603:111854-111934:+	ACA1_068120	Endo/exonuclease/phosphatase domain-containing protein [Source:UniProtKB/TrEMBL;Acc:L8HEN2]	0	0.2935637	0.2935637	6.98E-06
3h	ACA1_358790/clean/scf7180000084742:41659-41727:-	ACA1_358790	hypothetical protein	0	0.293175333	0.293175333	1.56E-06
3h	ACA1_380580/clean/scf7180000084712:55253-55383:-	ACA1_380580	Tim17/Tim22/Tim23 family protein [Source:UniProtKB/TrEMBL;Acc:L8GP12]	0.00218746	0.295357667	0.293170207	1.87E-08
3h	ACA1_388780/clean/scf7180000084733:167553-167753:+	ACA1_388780	EGFlike domain containing protein [Source:UniProtKB/TrEMBL;Acc:L8GDT8]	0.001795885	0.294862667	0.293066782	4.76E-08
3h	ACA1_235200/clean/scf7180000084762:339042-339118:-	ACA1_235200	SCP domain-containing protein [Source:UniProtKB/TrEMBL;Acc:L8H1C9]	0.001961807	0.2947256	0.292763793	5.49E-07
3h	ACA1_216420/clean/scf7180000084758:494364-494482:-	ACA1_216420	C2 domain-containing protein [Source:UniProtKB/TrEMBL;Acc:L8GQ92]	0.0483609	0.340985	0.2926241	0.00027102
3h	ACA1_025570/clean/scf7180000084448:16070-16149:+	ACA1_025570	hypothetical protein	0.005347277	0.2977893	0.292442023	1.89E-09
3h	ACA1_201470/clean/scf7180000084753:560005-560114:-	ACA1_201470	Rasrelated protein Rab-2B, putative [Source:UniProtKB/TrEMBL;Acc:L8H2N2]	0.002619967	0.295045233	0.292425267	3.38E-07
3h	ACA1_078010/clean/scf7180000084614:70854-71012:-	ACA1_078010	hypothetical protein	0.00175216	0.294034333	0.292282173	8.78E-08
3h	ACA1_355850/clean/scf7180000084728:32484-32571:-	ACA1_355850	Methionine aminopeptidase [Source:UniProtKB/TrEMBL;Acc:L8H6U0]	0.000665821	0.292922733	0.292256912	1.73E-08
3h	ACA1_274880/clean/scf7180000084773:131972-132132:+	ACA1_274880	AB hydrolase-1 domain-containing protein [Source:UniProtKB/TrEMBL;Acc:L8GI88]	0.335885	0.628044	0.292159	0.024221333
3h	ACA1_255900/clean/scf7180000084768:578188-578307:+	ACA1_255900	DEP domain-containing protein [Source:UniProtKB/TrEMBL;Acc:L8HA90]	0.011778907	0.30385	0.292071093	1.69E-09
3h	ACA1_112470/clean/scf7180000084669:159857-159977:-	ACA1_112470	KH domain-containing protein [Source:UniProtKB/TrEMBL;Acc:L8H5E2]	0.00120773	0.2932728	0.29206507	7.26E-06
3h	ACA1_368680/clean/scf7180000084664:135825-135919:-	ACA1_368680	PlsC domain-containing protein [Source:UniProtKB/TrEMBL;Acc:L8H1A8]	0.016071553	0.308017333	0.29194578	1.63E-05
3h	ACA1_271350/clean/scf7180000084771:24780-25011:+	ACA1_271350	hypothetical protein	0.335665333	0.627531333	0.291866	0.013659658
3h	ACA1_361020/clean/scf7180000084622:223753-223883:-	ACA1_361020	Ribosomal L22e protein family [Source:UniProtKB/TrEMBL;Acc:L8HF78]	0.00182529	0.293617667	0.291792377	1.40E-21
3h	ACA1_239090/clean/scf7180000084763:130053-130132:+	ACA1_239090	Hemerythrin domain-containing protein [Source:UniProtKB/TrEMBL;Acc:L8GKF7]	0.002331003	0.2940777	0.291746697	1.27E-06
3h	ACA1_065450/clean/scf7180000084599:1855695-1855769:-	ACA1_065450	Cation_ATPase_N domain-containing protein [Source:UniProtKB/TrEMBL;Acc:L8H037]	0	0.291651567	0.291651567	0.007507083
3h	ACA1_391090/clean/scf7180000084740:127692-127758:-	ACA1_391090	Inorganic diphosphatase [Source:UniProtKB/TrEMBL;Acc:L8GPC3]	0.0238282	0.315407	0.2915788	3.90E-05
3h	ACA1_393010/clean/scf7180000084756:27320-27399:+	ACA1_393010	AA_TRNA_LIGASE_II domain-containing protein [Source:UniProtKB/TrEMBL;Acc:L8H0A5]	0.018577433	0.310099567	0.291522133	3.13E-05
3h	ACA1_036200/clean/scf7180000084565:38125-38217:-	ACA1_036200	hypothetical protein	0.016143323	0.307551667	0.291408343	4.09E-05
3h	ACA1_054970/clean/scf7180000084594:615351-615465:-	ACA1_054970	FYVE-type domain-containing protein [Source:UniProtKB/TrEMBL;Acc:L8H8G2]	0.006730573	0.298079333	0.29134876	0.000194661
3h	ACA1_174580/clean/scf7180000084741:742359-742425:-	ACA1_174580	hypothetical protein	0.000623053	0.291849	0.291225947	3.02E-09
3h	ACA1_296850/clean/scf7180000084777:1416411-1416507:+	ACA1_296850	UBIQUITIN_CONJUGAT_2 domain-containing protein [Source:UniProtKB/TrEMBL;Acc:L8HK35]	0.0274566	0.318616967	0.291160367	0.000123042
3h	ACA1_377500/clean/scf7180000084711:75616-75695:+	ACA1_377500	Peptidase family c78 protein [Source:UniProtKB/TrEMBL;Acc:L8GU78]	0.00464209	0.295727667	0.291085577	2.63E-06
3h	ACA1_384870/clean/scf7180000084730:125241-125367:+	ACA1_384870	Protein kinase domain containing protein [Source:UniProtKB/TrEMBL;Acc:L8HBH4]	0.001430617	0.292376	0.290945383	4.11E-07
3h	ACA1_248670/clean/scf7180000084766:107088-107170:-	ACA1_248670	CBS domain containing protein [Source:UniProtKB/TrEMBL;Acc:L8GY23]	0.00401335	0.294899867	0.290886517	2.54E-07
3h	ACA1_313720/clean/scf7180000083824:1094-1166:+	ACA1_313720	VWFA domain-containing protein [Source:UniProtKB/TrEMBL;Acc:L8GND4]	0.029716133	0.320544333	0.2908282	3.18E-05
3h	ACA1_069150/clean/scf7180000084603:367644-367719:+	ACA1_069150	PH domain containing protein [Source:UniProtKB/TrEMBL;Acc:L8HFV4]	0.05523118	0.346043	0.29081182	0.004282802
3h	ACA1_229520/clean/scf7180000084761:264086-264370:+	ACA1_229520	Protein serine/threonine kinase, putative [Source:UniProtKB/TrEMBL;Acc:L8H813]	0.012044493	0.302852533	0.29080804	7.66E-06
3h	ACA1_379080/clean/scf7180000084711:276786-276865:+	ACA1_379080	GATA-type domain-containing protein [Source:UniProtKB/TrEMBL;Acc:L8GS28]	0.00742599	0.298229233	0.290803243	2.09E-06
3h	ACA1_024390/clean/scf7180000084442:10495-10567:+	ACA1_024390	hypothetical protein	0	0.290775067	0.290775067	2.44E-07
3h	ACA1_200360/clean/scf7180000084753:428775-428907:+	ACA1_200360	Leucine rich repeat domain containing protein [Source:UniProtKB/TrEMBL;Acc:L8H2J7]	0.0351002	0.32587	0.2907698	7.34E-05
3h	ACA1_237810/clean/scf7180000084763:20822-20921:-	ACA1_237810	hypothetical protein	0.008266527	0.299025667	0.29075914	5.71E-06
3h	ACA1_171900/clean/scf7180000084741:194484-194595:+	ACA1_171900	ATPase/histidine kinase/DNA gyrase B/HSP90 domain containing protein [Source:UniProtKB/TrEMBL;Acc:L8HJR6]	0	0.290746033	0.290746033	0.000131894
3h	ACA1_144250/clean/scf7180000084709:182075-182151:-	ACA1_144250	SH2 domain containing protein [Source:UniProtKB/TrEMBL;Acc:L8HEU2]	0.014594897	0.305277367	0.29068247	1.22E-06
3h	ACA1_181910/clean/scf7180000084745:71362-71422:-	ACA1_181910	CrcB family protein [Source:UniProtKB/TrEMBL;Acc:L8H804]	0.02056629	0.311017	0.29045071	0.00060758
3h	ACA1_399880/clean/scf7180000084775:58262-58352:+	ACA1_399880	Rab-GAP TBC domain-containing protein [Source:UniProtKB/TrEMBL;Acc:L8GGF5]	0.01026814	0.300676633	0.290408493	0.000326624
3h	ACA1_130620/clean/scf7180000084698:123820-123918:-	ACA1_130620	Rho-GAP domain-containing protein [Source:UniProtKB/TrEMBL;Acc:L8GPL1]	0.018567343	0.308930033	0.29036269	0.000494789
3h	ACA1_125040/clean/scf7180000084691:21879-21937:-	ACA1_125040	hypothetical protein	0.005712817	0.295980667	0.29026785	7.55E-09
3h	ACA1_100480/clean/scf7180000084648:94586-94659:-	ACA1_100480	Fbox/LRR-repeat protein [Source:UniProtKB/TrEMBL;Acc:L8GLX9]	0	0.2902496	0.2902496	9.46E-05
3h	ACA1_224860/clean/scf7180000084760:261188-261260:+	ACA1_224860	Myosin head (Motor domain) domain containing protein [Source:UniProtKB/TrEMBL;Acc:L8GSY9]	0	0.290231	0.290231	0.002540177
3h	ACA1_071690/clean/scf7180000084603:1030291-1030386:+	ACA1_071690	Vacuolar protein sorting factor 4, putative [Source:UniProtKB/TrEMBL;Acc:L8HE96]	0.003880319	0.294078067	0.290197747	3.10E-07
3h	ACA1_323110/clean/scf7180000084467:4448-4543:+	ACA1_323110	hypothetical protein	0.020093517	0.310104567	0.29001105	0.00096066
3h	ACA1_112170/clean/scf7180000084669:135762-135836:+	ACA1_112170	PPM-type phosphatase domain-containing protein [Source:UniProtKB/TrEMBL;Acc:L8H322]	0.004827847	0.294745033	0.289917187	2.54E-05
3h	ACA1_018770/clean/scf7180000083912:14324-14394:+	ACA1_018770	Signal recognition particle subunit SRP68 [Source:UniProtKB/TrEMBL;Acc:L8HKJ5]	0.011563693	0.30141	0.289846307	8.39E-08
3h	ACA1_066140/clean/scf7180000084599:2004164-2004330:-	ACA1_066140	AP complex subunit sigma [Source:UniProtKB/TrEMBL;Acc:L8GZ32]	0.00664648	0.296402367	0.289755887	2.13E-06
3h	ACA1_215000/clean/scf7180000084758:290302-290444:-	ACA1_215000	GATA-type domain-containing protein [Source:UniProtKB/TrEMBL;Acc:L8GSI2]	0.0045662	0.294289233	0.289723033	0.000136035
3h	ACA1_215970/clean/scf7180000084758:421425-421589:-	ACA1_215970	EGFlike domain containing protein [Source:UniProtKB/TrEMBL;Acc:L8GQ73]	0.052321833	0.342000333	0.2896785	0.007886713
3h	ACA1_142590/clean/scf7180000084708:193123-193240:+	ACA1_142590	unspecified product	0.001766851	0.2913736	0.289606749	1.76E-06
3h	ACA1_158940/clean/scf7180000084724:300646-300716:+	ACA1_158940	Oxidoreductase [Source:UniProtKB/TrEMBL;Acc:L8HBL7]	0.002904623	0.2925078	0.289603177	1.98E-06
3h	ACA1_114590/clean/scf7180000084669:517824-517946:-	ACA1_114590	Proteasome activator protein [Source:UniProtKB/TrEMBL;Acc:L8H6L8]	0.0025305	0.2919908	0.2894603	9.10E-07
3h	ACA1_126180/clean/scf7180000084693:46167-46237:+	ACA1_126180	BTB domain-containing protein [Source:UniProtKB/TrEMBL;Acc:L8HBT3]	2.86E-05	0.289487333	0.289458699	1.25E-08
3h	ACA1_233880/clean/scf7180000084762:177350-177456:+	ACA1_233880	Purple acid phosphatase [Source:UniProtKB/TrEMBL;Acc:L8H124]	0.002986733	0.2924299	0.289443167	2.58E-08
3h	ACA1_257280/clean/scf7180000084769:142460-142547:-	ACA1_257280	Chloride transporter, chloride channel (ClC) subfamily protein [Source:UniProtKB/TrEMBL;Acc:L8GFQ3]	0	0.289427333	0.289427333	1.44E-06
3h	ACA1_171870/clean/scf7180000084741:182475-182555:-	ACA1_171870	Leucine rich repeat domain containing protein [Source:UniProtKB/TrEMBL;Acc:L8HIV4]	0.042179033	0.331463667	0.289284633	0.014314582
3h	ACA1_068700/clean/scf7180000084603:258575-258649:+	ACA1_068700	Purple acid phosphatase [Source:UniProtKB/TrEMBL;Acc:L8HFR9]	0.00244236	0.291709567	0.289267206	2.65E-09
3h	ACA1_184260/clean/scf7180000084745:594176-594326:-	ACA1_184260	Phytanoyl-CoA dioxygenase [Source:UniProtKB/TrEMBL;Acc:L8H8I4]	0.004681677	0.293904	0.289222323	6.18E-05
3h	ACA1_217160/clean/scf7180000084758:532181-532298:+	ACA1_217160	hypothetical protein	0.020155567	0.309213333	0.289057767	4.83E-07
3h	ACA1_012020/clean/scf7180000083652:5780-5918:-	ACA1_012020	hypothetical protein	0.005625827	0.294594767	0.28896894	6.79E-06
3h	ACA1_341110/clean/scf7180000084620:53533-53622:-	ACA1_341110	PPM-type phosphatase domain-containing protein [Source:UniProtKB/TrEMBL;Acc:L8GTN5]	0.002292217	0.291224333	0.288932117	3.23E-05
3h	ACA1_060230/clean/scf7180000084599:623477-623571:+	ACA1_060230	hypothetical protein	0.00869499	0.297548333	0.288853343	1.65E-06
3h	ACA1_069920/clean/scf7180000084603:600765-600853:-	ACA1_069920	Phospholipid-transporting ATPase [Source:UniProtKB/TrEMBL;Acc:L8HDU8]	0.006944433	0.295793667	0.288849233	3.96E-05
3h	ACA1_024200/clean/scf7180000084441:14804-14884:-	ACA1_024200	WD_REPEATS_REGION domain-containing protein [Source:UniProtKB/TrEMBL;Acc:L8GKS0]	0.154256	0.443057	0.288801	0.009722171
3h	ACA1_383710/clean/scf7180000084721:251646-251739:+	ACA1_383710	Glucose-6-phosphate 1-dehydrogenase [Source:UniProtKB/TrEMBL;Acc:L8GW58]	0.010466993	0.299260133	0.28879314	3.03E-07
3h	ACA1_106330/clean/scf7180000084660:87229-87413:-	ACA1_106330	Methyltransf_11 domain-containing protein [Source:UniProtKB/TrEMBL;Acc:L8GMT5]	0.00292517	0.291594333	0.288669163	1.12E-05
3h	ACA1_112660/clean/scf7180000084669:210662-210737:+	ACA1_112660	BTB_2 domain-containing protein [Source:UniProtKB/TrEMBL;Acc:M0QSU3]	0.017515333	0.30616	0.288644667	0.000135434
3h	ACA1_068110/clean/scf7180000084603:110672-110731:+	ACA1_068110	Peptidylprolyl isomerase [Source:UniProtKB/TrEMBL;Acc:L8HDB8]	0.012094703	0.300675667	0.288580963	6.75E-07
3h	ACA1_252540/clean/scf7180000084768:176023-176105:-	ACA1_252540	hypothetical protein	0.01063917	0.299184	0.28854483	8.04E-08
3h	ACA1_064490/clean/scf7180000084599:1628105-1628174:+	ACA1_064490	TYR_PHOSPHATASE_2 domain-containing protein [Source:UniProtKB/TrEMBL;Acc:L8GXK2]	0.0374555	0.325981667	0.288526167	0.003186905
3h	ACA1_264380/clean/scf7180000084770:255714-255791:-	ACA1_264380	S-formylglutathione hydrolase [Source:UniProtKB/TrEMBL;Acc:L8H1Z7]	0.002693865	0.291155967	0.288462102	1.68E-08
3h	ACA1_335940/clean/scf7180000084593:9194-9295:-	ACA1_335940	RNA recognition motif domain containing protein [Source:UniProtKB/TrEMBL;Acc:L8H3Y6]	0.001826597	0.2902863	0.288459703	1.83E-06
3h	ACA1_165790/clean/scf7180000084732:83168-83274:-	ACA1_165790	hypothetical protein	0.012895292	0.301343467	0.288448175	2.44E-06
3h	ACA1_216320/clean/scf7180000084758:468241-468353:+	ACA1_216320	Homogentisate 1,2dioxygenase [Source:UniProtKB/TrEMBL;Acc:L8GQ83]	0.000769823	0.289161767	0.288391943	2.23E-08
3h	ACA1_189590/clean/scf7180000084748:58748-58833:+	ACA1_189590	Transcriptional regulatorrelated, putative [Source:UniProtKB/TrEMBL;Acc:L8GEX7]	0.00323947	0.291603667	0.288364197	3.72E-07
3h	ACA1_396170/clean/scf7180000084759:44563-44644:-	ACA1_396170	Phosphatidylserine decarboxylase proenzyme 2 [Source:UniProtKB/TrEMBL;Acc:L8HEJ4]	0.014597533	0.302871767	0.288274233	8.17E-06
3h	ACA1_255750/clean/scf7180000084768:561071-561239:+	ACA1_255750	hypothetical protein	0.094506033	0.382762	0.288255967	0.001503229
3h	ACA1_027030/clean/scf7180000084464:27590-27664:-	ACA1_027030	C2 domain-containing protein [Source:UniProtKB/TrEMBL;Acc:L8GJ80]	0.006453237	0.294630333	0.288177097	1.48E-05
3h	ACA1_221030/clean/scf7180000084758:982492-982571:-	ACA1_221030	DUF1992 domain-containing protein [Source:UniProtKB/TrEMBL;Acc:L8GQX4]	0.001682287	0.289719333	0.288037047	1.71E-05
3h	ACA1_048870/clean/scf7180000084583:5856-5968:-	ACA1_048870	hypothetical protein	0.045422767	0.333274267	0.2878515	0.001602795
3h	ACA1_155470/clean/scf7180000084722:163461-163564:-	ACA1_155470	unspecified product	0.0745306	0.362367333	0.287836733	0.010021094

3h	ACA1_330540/clean/scf7180000084566:60419-60535:-	ACA1_330540	hypothetical protein	0.00626131	0.2940654	0.28780409	7.69E-07
3h	ACA1_301720/clean/scf7180000083222:28265-28381:+	ACA1_301720	Amidohydro-rel domain-containing protein [Source:UniProtKB/TrEMBL;Acc:L8HKQ5]	0.000571488	0.28836	0.287788512	7.90E-09
3h	ACA1_069270/clean/scf7180000084603:398964-399030:-	ACA1_069270	RhoGEF domain containing protein [Source:UniProtKB/TrEMBL;Acc:L8HEZ6]	0.0061958	0.293933	0.2877372	0.000692381
3h	ACA1_098010/clean/scf7180000084645:1376697-1376809:+	ACA1_098010	Lactamase_B domain-containing protein [Source:UniProtKB/TrEMBL;Acc:L8GJX3]	0.015576333	0.303293	0.287716667	0.007627358
3h	ACA1_289280/clean/scf7180000084777:405528-405609:-	ACA1_289280	RasGEF domain containing protein [Source:UniProtKB/TrEMBL;Acc:L8HJ36]	0.039682533	0.327278333	0.2875958	0.004534807
3h	ACA1_027270/clean/scf7180000084465:8783-8865:-	ACA1_027270	HAD_SAK_1 domain containing protein [Source:UniProtKB/TrEMBL;Acc:L8GEP7]	0	0.287519667	0.287519667	3.32E-07
3h	ACA1_109790/clean/scf7180000084663:28021-28187:+	ACA1_109790	Delta-1-pyrroline-5-carboxylate synthase [Source:UniProtKB/TrEMBL;Acc:L8HJZ5]	0.017379267	0.3048813	0.287502033	0.000133512
3h	ACA1_135650/clean/scf7180000084702:100151-100219:-	ACA1_135650	MYND finger domain containing protein [Source:UniProtKB/TrEMBL;Acc:L8GGQ8]	0.153809433	0.44128	0.287470567	0.020794714
3h	ACA1_038210/clean/scf7180000084568:97245-97329:-	ACA1_038210	hypothetical protein	0.054664767	0.342082	0.287417233	0.002456999
3h	ACA1_057960/clean/scf7180000084599:222582-222660:+	ACA1_057960	BTB/POZ domain containing protein [Source:UniProtKB/TrEMBL;Acc:L8GVX1]	0.007563097	0.2949581	0.287395003	0.000170012
3h	ACA1_046170/clean/scf7180000084577:74141-74213:-	ACA1_046170	Kelch repeat-containing protein [Source:UniProtKB/TrEMBL;Acc:L8HB10]	0.025641033	0.3129141	0.287273067	0.028987858
3h	ACA1_306840/clean/scf7180000083668:16589-16680:+	ACA1_306840	RTP1_C1 domain-containing protein [Source:UniProtKB/TrEMBL;Acc:L8GQF6]	0.011904767	0.2991102	0.287205433	0.001528859
3h	ACA1_073230/clean/scf7180000084603:1375556-1375653:-	ACA1_073230	EHN domain-containing protein [Source:UniProtKB/TrEMBL;Acc:L8HDT9]	0.006785173	0.293990533	0.28720536	9.96E-05
3h	ACA1_181830/clean/scf7180000084745:54986-55134:-	ACA1_181830	PIPK domain containing protein [Source:UniProtKB/TrEMBL;Acc:L8H972]	0	0.287189267	0.287189267	0.003701946
3h	ACA1_157210/clean/scf7180000084724:6544-6677:+	ACA1_157210	hypothetical protein	0.00618032	0.2931705	0.28699018	9.98E-06
3h	ACA1_171510/clean/scf7180000084741:123975-124084:+	ACA1_171510	HemolysinIII related subfamily protein [Source:UniProtKB/TrEMBL;Acc:L8HHB3]	0.01923265	0.306089	0.28685635	0.000663749
3h	ACA1_384590/clean/scf7180000084730:59739-59809:-	ACA1_384590	RAB1B, member RAS oncogene family [Source:UniProtKB/TrEMBL;Acc:L8H896]	0.010002113	0.296839033	0.28683692	8.51E-08
3h	ACA1_040850/clean/scf7180000084572:94245-94318:+	ACA1_040850	Oxidored_q6 domain-containing protein [Source:UniProtKB/TrEMBL;Acc:L8GZT1]	0.007326003	0.294092533	0.28676653	3.40E-10
3h	ACA1_331590/clean/scf7180000084578:8270-8344:+	ACA1_331590	ATPdependent DNA helicase, RecQ subfamily protein [Source:UniProtKB/TrEMBL;Acc:L8HBM3]	0.003314917	0.289967667	0.28665275	2.01E-05
3h	ACA1_094910/clean/scf7180000084645:788068-788240:-	ACA1_094910	Deoxyhypusine synthase [Source:UniProtKB/TrEMBL;Acc:L8GL08]	0.001972185	0.2885339	0.286561715	8.37E-06
3h	ACA1_288840/clean/scf7180000084777:307721-307788:-	ACA1_288840	Saposin family type B, region 1 protein [Source:UniProtKB/TrEMBL;Acc:L8HKH6]	0.007530167	0.294048	0.286517833	8.33E-08
3h	ACA1_158810/clean/scf7180000084724:258771-258859:-	ACA1_158810	Coatomer subunit epsilon [Source:UniProtKB/TrEMBL;Acc:L8HCK7]	0.001990143	0.2884427	0.286452557	4.94E-08
3h	ACA1_065910/clean/scf7180000084599:1944900-1945001:+	ACA1_065910	hypothetical protein	0.048711967	0.335144667	0.2864327	0.023412324
3h	ACA1_201500/clean/scf7180000084753:573617-573731:+	ACA1_201500	Endo/exonuclease/phosphatase domain-containing protein [Source:UniProtKB/TrEMBL;Acc:L8H3H0]	0.039108433	0.325498	0.286389567	0.000223445
3h	ACA1_116750/clean/scf7180000084669:840644-840734:+	ACA1_116750	CAP_C domain-containing protein [Source:UniProtKB/TrEMBL;Acc:L8H632]	0.011158067	0.297539233	0.286381167	4.33E-05
3h	ACA1_060920/clean/scf7180000084599:781141-781237:-	ACA1_060920	MFS domain-containing protein [Source:UniProtKB/TrEMBL;Acc:L8GVT5]	0.003049827	0.2892615	0.286211673	0.000204008
3h	ACA1_110860/clean/scf7180000084666:3841-3908:+	ACA1_110860	SID1 transmembrane family, member 1, putative [Source:UniProtKB/TrEMBL;Acc:L8H6V2]	0.0161737	0.3023789	0.2862052	0.002596941
3h	ACA1_178710/clean/scf7180000084743:267576-267651:-	ACA1_178710	ADPribosylation factor related protein 1, putative [Source:UniProtKB/TrEMBL;Acc:L8GTG3]	0.003787867	0.2899847	0.286196833	1.50E-05
3h	ACA1_382920/clean/scf7180000084721:120334-120416:-	ACA1_382920	Ras subfamily protein [Source:UniProtKB/TrEMBL;Acc:L8GV31]	0.018518533	0.304559933	0.2860414	0.006131449
3h	ACA1_106810/clean/scf7180000084660:135862-136003:-	ACA1_106810	FHA domain containing protein [Source:UniProtKB/TrEMBL;Acc:L8GML7]	0.025824433	0.311649333	0.2858249	2.33E-05
3h	ACA1_137790/clean/scf7180000084703:72417-72483:-	ACA1_137790	IBR domain-containing protein [Source:UniProtKB/TrEMBL;Acc:L8GZJ6]	0.004136733	0.289719267	0.285582533	0.002210706
3h	ACA1_034080/clean/scf7180000084561:18179-18286:-	ACA1_034080	RhoGEF domain containing protein [Source:UniProtKB/TrEMBL;Acc:L8GTW8]	0.0064725	0.291935667	0.285463167	0.000151807
3h	ACA1_322220/clean/scf7180000084459:33690-33832:+	ACA1_322220	hypothetical protein	0.0501283	0.3354629	0.2853346	0.001307956
3h	ACA1_064800/clean/scf7180000084599:1697054-1697114:-	ACA1_064800	Serine/threonine kinase [Source:UniProtKB/TrEMBL;Acc:L8GYT2]	0	0.285271	0.285271	0.001225505
3h	ACA1_204520/clean/scf7180000084754:303775-303886:-	ACA1_204520	hypothetical protein	0.031390133	0.316631967	0.285241833	0.042519365
3h	ACA1_322160/clean/scf7180000084459:22404-22485:+	ACA1_322160	AB hydrolase-1 domain-containing protein [Source:UniProtKB/TrEMBL;Acc:L8GMT9]	0.016597833	0.301783067	0.285185233	3.82E-06
3h	ACA1_310750/clean/scf7180000083780:6473-6541:+	ACA1_310750	RFX-type winged-helix domain-containing protein [Source:UniProtKB/TrEMBL;Acc:L8GVY4]	0.029681707	0.314651233	0.284969527	0.002362727
3h	ACA1_364570/clean/scf7180000084641:145145-145293:+	ACA1_364570	hypothetical protein	0.003436223	0.288351167	0.284914943	1.76E-06
3h	ACA1_066150/clean/scf7180000084599:2010779-2010890:+	ACA1_066150	Myosin head (Motor domain) domain containing protein [Source:UniProtKB/TrEMBL;Acc:L8GX00]	0	0.2847228	0.2847228	0.00134951
3h	ACA1_072790/clean/scf7180000084603:1297232-1297319:-	ACA1_072790	AB hydrolase-1 domain-containing protein [Source:UniProtKB/TrEMBL;Acc:L8HGV1]	0.035695	0.320405667	0.284710667	0.001884228
3h	ACA1_389320/clean/scf7180000084733:228509-228575:+	ACA1_389320	BTB domain-containing protein [Source:UniProtKB/TrEMBL;Acc:M0QSM2]	0.003603367	0.2882941	0.284690733	5.68E-06
3h	ACA1_040690/clean/scf7180000084572:48865-49027:+	ACA1_040690	Multisensor hybrid histidine kinase [Source:UniProtKB/TrEMBL;Acc:L8H1W6]	0.007813475	0.2924332	0.284619725	8.86E-05
3h	ACA1_305550/clean/scf7180000083609:19042-19144:+	ACA1_305550	Folate gamma-glutamyl hydrolase [Source:UniProtKB/TrEMBL;Acc:L8GKY1]	0.000235397	0.284813	0.284577603	4.52E-10
3h	ACA1_361090/clean/scf7180000084622:239577-239678:+	ACA1_361090	hypothetical protein	0.0093464	0.293878333	0.284531933	1.58E-05
3h	ACA1_215950/clean/scf7180000084758:414126-414216:+	ACA1_215950	SLT domain-containing protein [Source:UniProtKB/TrEMBL;Acc:L8GR46]	0.005259737	0.289775333	0.284515597	1.11E-05
3h	ACA1_078990/clean/scf7180000084615:125424-125539:-	ACA1_078990	AcylCoA synthetase, putative [Source:UniProtKB/TrEMBL;Acc:L8GRK9]	0.000615508	0.284850333	0.284234826	4.78E-09
3h	ACA1_150720/clean/scf7180000084716:26273-26352:-	ACA1_150720	TPR_REGION domain-containing protein [Source:UniProtKB/TrEMBL;Acc:L8H0N3]	0.004610104	0.288800033	0.28418993	1.50E-06
3h	ACA1_130580/clean/scf7180000084698:115264-115335:-	ACA1_130580	CID domain-containing protein [Source:UniProtKB/TrEMBL;Acc:L8GQC8]	0.000908267	0.2848458	0.283937533	3.94E-06
3h	ACA1_193320/clean/scf7180000084749:232384-232463:-	ACA1_193320	hypothetical protein	0	0.283914567	0.283914567	0.005880408
3h	ACA1_154200/clean/scf7180000084722:22748-22810:-	ACA1_154200	Peptidase S53 domain-containing protein [Source:UniProtKB/TrEMBL;Acc:L8GZY7]	0.0516172	0.335518667	0.283901467	0.000217431
3h	ACA1_063740/clean/scf7180000084599:1492463-1492533:-	ACA1_063740	Transmembrane protein [Source:UniProtKB/TrEMBL;Acc:L8GXB1]	0.00850157	0.292386333	0.28388763	4.53E-05
3h	ACA1_377770/clean/scf7180000084711:117248-117341:+	ACA1_377770	WDrepeat protein pop3, putative [Source:UniProtKB/TrEMBL;Acc:L8GRX7]	0.008528497	0.2923699	0.283841403	1.47E-05
3h	ACA1_104240/clean/scf7180000084653:73974-74052:-	ACA1_104240	hypothetical protein	0.010454903	0.294123133	0.28366823	5.36E-05
3h	ACA1_002450/clean/scf7180000082931:3054-3125:+	ACA1_002450	eIF-5a domain-containing protein [Source:UniProtKB/TrEMBL;Acc:L8GWN9]	0	0.283600267	0.283600267	3.84E-05
3h	ACA1_297760/clean/scf7180000082853:3740-3815:+	ACA1_297760	hypothetical protein	0.006531263	0.290074667	0.283543403	5.25E-08
3h	ACA1_299380/clean/scf7180000083027:11354-11419:+	ACA1_299380	AAA_11 domain-containing protein [Source:UniProtKB/TrEMBL;Acc:L8GS61]	0.009661137	0.2931986	0.283537463	7.01E-08
3h	ACA1_289800/clean/scf7180000084777:466753-466826:-	ACA1_289800	J domain-containing protein [Source:UniProtKB/TrEMBL;Acc:L8HJ30]	0.0306849	0.314195	0.2835101	0.001929905
3h	ACA1_167030/clean/scf7180000084736:59624-59738:+	ACA1_167030	hypothetical protein	0.029852333	0.313333667	0.283481333	7.59E-05
3h	ACA1_071250/clean/scf7180000084603:898573-898647:-	ACA1_071250	DEAD box RNA helicase, putative [Source:UniProtKB/TrEMBL;Acc:L8HD92]	0.107038033	0.390452	0.283413967	0.003007945
3h	ACA1_396260/clean/scf7180000084759:66015-66111:+	ACA1_396260	hypothetical protein	0.002222223	0.2855829	0.283360677	3.91E-06
3h	ACA1_366940/clean/scf7180000084641:520646-520775:-	ACA1_366940	BTB/POZ domain containing protein [Source:UniProtKB/TrEMBL;Acc:L8GPM1]	0.013605433	0.296859233	0.2832538	0.001623815
3h	ACA1_098910/clean/scf7180000084646:61209-61300:-	ACA1_098910	OGFr_N domain-containing protein [Source:UniProtKB/TrEMBL;Acc:L8H4R8]	0.003032397	0.286218933	0.283186537	1.21E-06
3h	ACA1_152930/clean/scf7180000084720:37455-37525:-	ACA1_152930	PG_binding_1 domain-containing protein [Source:UniProtKB/TrEMBL;Acc:L8HF34]	0	0.283152667	0.283152667	1.28E-06
3h	ACA1_203500/clean/scf7180000084754:168662-168763:-	ACA1_203500	Coatomer subunit delta [Source:UniProtKB/TrEMBL;Acc:L8GW47]	0.002076843	0.285057167	0.282980323	7.03E-07
3h	ACA1_198520/clean/scf7180000084753:132850-132950:-	ACA1_198520	Carrier superfamily protein [Source:UniProtKB/TrEMBL;Acc:L8H5C1]	0.01931604	0.302281	0.28296496	0.000851039
3h	ACA1_384820/clean/scf7180000084730:109702-109767:+	ACA1_384820	hypothetical protein	0.0181402	0.301064333	0.282924133	0.021450737
3h	ACA1_055050/clean/scf7180000084594:640790-640889:+	ACA1_055050	SH3 domain containing protein [Source:UniProtKB/TrEMBL;Acc:L8H5H2]	0.001973804	0.284305533	0.28233173	9.49E-11
3h	ACA1_139150/clean/scf7180000084705:61307-61431:+	ACA1_139150	ENT domain-containing protein [Source:UniProtKB/TrEMBL;Acc:L8GN95]	0.051047267	0.333300667	0.2822534	0.000216988
3h	ACA1_278920/clean/scf7180000084776:277048-277138:+	ACA1_278920	ABC1 domain-containing protein [Source:UniProtKB/TrEMBL;Acc:L8H8Y2]	0.0172418	0.299368633	0.282126833	0.000512648
3h	ACA1_095660/clean/scf7180000084645:872223-872345:+	ACA1_095660	Poly [ADP-ribose] polymerase [Source:UniProtKB/TrEMBL;Acc:L8GL42]	0.00132109	0.2833675	0.28204641	1.01E-06
3h	ACA1_291600/clean/scf7180000084777:783784-783904:-	ACA1_291600	Macro domain-containing protein [Source:UniProtKB/TrEMBL;Acc:L8HJG2]	0.00680673	0.288825567	0.282018837	1.38E-05
3h	ACA1_331110/clean/scf7180000084575:3060-3140:-	ACA1_331110	RAP1A, member of RAS oncogene family [Source:UniProtKB/TrEMBL;Acc:L8GU20]	0.006015512	0.287993833	0.281978321	4.75E-07
3h	ACA1_325560/clean/scf7180000084495:34337-34444:+	ACA1_325560	Solute carrier family protein [Source:UniProtKB/TrEMBL;Acc:L8H8Z4]	0.020207267	0.302082533	0.281875267	4.51E-06
3h	ACA1_269310/clean/scf7180000084770:968920-969004:-	ACA1_269310	RING-type domain-containing protein [Source:UniProtKB/TrEMBL;Acc:L8H2R5]	0.003355153	0.28523	0.281874847	1.16E-06
3h	ACA1_310760/clean/scf7180000083780:7379-7449:+	ACA1_310760	hypothetical protein	0.019736723	0.301428967	0.281692243	0.000477131
3h	ACA1_062620/clean/scf7180000084599:1256640-1256721:-	ACA1_062620	hypothetical protein	0	0.281615	0.281615	2.05E-07
3h	ACA1_285540/clean/scf7180000084776:992689-992793:-	ACA1_285540	GPS domain-containing protein [Source:UniProtKB/TrEMBL;Acc:L8H7T4]	0	0.281441033	0.281441033	0.000116939
3h	ACA1_095670/clean/scf7180000084645:877336-877423:-	ACA1_095670	FAD-binding PCMH-type domain-containing protein [Source:UniProtKB/TrEMBL;Acc:L8GIT7]	0.01868665	0.300126	0.28143935	5.89E-06
3h	ACA1_175240/clean/scf7180000084741:898952-899086:+	ACA1_175240	Rab32 protein, putative [Source:UniProtKB/TrEMBL;Acc:L8HI25]	0.00954122	0.290960633	0.281419413	3.49E-05
3h	ACA1_392520/clean/scf7180000084740:284330-284448:-	ACA1_392520	hypothetical protein	0.0109605	0.292334633	0.281374133	5.73E-05
3h	ACA1_288730/clean/scf7180000084777:273290-273390:-	ACA1_288730	Esterase, PHB depolymerase subfamily protein [Source:UniProtKB/TrEMBL;Acc:L8HIW4]	0.004065033	0.285407667	0.281342633	3.17E-05
3h	ACA1_381510/clean/scf7180000084712:167822-167918:+	ACA1_381510	Pyr_redox_2 domain-containing protein [Source:UniProtKB/TrEMBL;Acc:L8GNN7]	0.015312067	0.296616567	0.2813045	8.88E-06
3h	ACA1_296240/clean/scf7180000084777:1304096-1304182:-	ACA1_296240	hypothetical protein	0.007559697	0.2888336	0.281273903	5.92E-06
3h	ACA1_061870/clean/scf7180000084599:1069341-1069425:+	ACA1_061870	Gluconokinase [Source:UniProtKB/TrEMBL;Acc:L8GWR6]	0.00726015	0.288483533	0.281223384	2.85E-05
3h	ACA1_307520/clean/scf7180000083680:25965-26033:-	ACA1_307520	B5 domain-containing protein [Source:UniProtKB/TrEMBL;Acc:L8H4Y1]	0.00093066	0.282147733	0.281217073	5.86E-08
3h	ACA1_038160/clean/scf7180000084568:86387-86636:-	ACA1_038160	Protein kinase domain-containing protein [Source:UniProtKB/TrEMBL;Acc:L8GN38]	0.019378517	0.300538667	0.28116015	4.93E-05
3h	ACA1_256380/clean/scf7180000084769:15071-15164:-	ACA1_256380	UBA/TS-N domain containing protein [Source:UniProtKB/TrEMBL;Acc:L8GF49]	0.003267973	0.284366667	0.281098693	5.95E-05
3h	ACA1_237860/clean/scf7180000084763:34391-34510:+	ACA1_237860	Glycerophosphodiester phosphodiesterase family protein [Source:UniProtKB/TrEMBL;Acc:L8GKN2]	0.004092777	0.285030233	0.280937456	1.10E-05
3h	ACA1_293890/clean/scf7180000084777:987232-987321:+	ACA1_293890	hypothetical protein	0.017846033	0.298648833	0.2808028	2.91E-05
3h	ACA1_400210/clean/scf7180000084775:120248-120333:-	ACA1_400210	Non-specific serine/threonine protein kinase [Source:UniProtKB/TrEMBL;Acc:L8GIE6]	0.0051282	0.285909067	0.280780867	5.84E-06
3h	ACA1_291470/clean/scf7180000084777:753793-753891:-	ACA1_291470	20S proteasome subunit beta 3, putative [Source:UniProtKB/TrEMBL;Acc:L8HIC0]	0.003404907	0.284140333	0.280735427	3.05E-11
3h	ACA1_281600/clean/scf7180000084776:601736-601815:-	ACA1_281600	PH domain-containing protein [Source:UniProtKB/TrEMBL;Acc:L8H9D9]	0.044444467	0.325157833	0.280713367	0.00915123
3h	ACA1_390820/clean/scf7180000084740:108113-108208:+	ACA1_390820	LIM zinc-binding domain-containing protein [Source:UniProtKB/TrEMBL;Acc:L8GP44]	0.004859616	0.2855581	0.280698484	4.36E-10
3h	ACA1_055490/clean/scf7180000084594:716632-716773:+	ACA1_055490	ThiF domain-containing protein [Source:UniProtKB/TrEMBL;Acc:L8H7S5]	0.002695383	0.2833391	0.280643717	3.87E-05
3h	ACA1_232470/clean/scf7180000084762:35230-35308:-	ACA1_232470	hypothetical protein	0.0448039	0.325384267	0.280580367	0.000855792
3h	ACA1_074930/clean/scf7180000084608:115506-115588:+	ACA1_074930	Calponin domain containing protein [Source:UniProtKB/TrEMBL;Acc:L8HHK0]	0.015830933	0.2962504	0.280419467	3.04E-05
3h	ACA1_183320/clean/scf7180000084745:378693-378798:+	ACA1_183320	hypothetical protein	0.001366303	0.281726733	0.28036043	4.04E-05
3h	ACA1_215030/clean/scf7180000084758:297226-297300:-	ACA1_215030	hypothetical protein	0.105090633	0.385327	0.280236367	0.043677166
3h	ACA1_174090/clean/scf7180000084741:605184-605310:+	ACA1_174090	AB hydrolase-1 domain-containing protein [Source:UniProtKB/TrEMBL;Acc:L8HHT7]	0.010959237	0.2911626	0.280203363	5.92E-08
3h	ACA1_337990/clean/scf7180000084604:22385-22524:-	ACA1_337990	DJ-1_PfpI domain-containing protein [Source:UniProtKB/TrEMBL;Acc:L8GCZ7]	0.00158608	0.2815524	0.27996632	2.67E-07
3h	ACA1_189810/clean/scf7180000084748:90804-90905:-	ACA1_189810	GYF domain-containing protein [Source:UniProtKB/TrEMBL;Acc:L8GDX6]	0.026572257	0.306491333	0.279919077	0.000134091
3h	ACA1_074580/clean/scf7180000084608:48307-48390:+	ACA1_074580	Ras-related protein Rab-7A [Source:UniProtKB/TrEMBL;Acc:L8HHF3]	0.0151502	0.295067467	0.279917267	4.04E-07
3h	ACA1_215000/clean/scf7180000084758:289434-289498:-	ACA1_215000	GATA-type domain-containing protein [Source:UniProtKB/TrEMBL;Acc:L8GSI2]	0.016153467	0.296059333	0.279905867	0.000127849
3h	ACA1_189830/clean/scf7180000084748:97905-97987:+	ACA1_189830	Partner of sld five, psf2 protein [Source:UniProtKB/TrEMBL;Acc:L8GE47]	0.0060606	0.285882167	0.279821567	0.000327333
3h	ACA1_248870/clean/scf7180000084766:139373-139461:-	ACA1_248870	GOLD domain-containing protein [Source:UniProtKB/TrEMBL;Acc:L8GY33]	0.01132293	0.291046733	0.279723803	3.79E-07
3h	ACA1_068700/clean/scf7180000084603:256698-256752:+	ACA1_068700	Purple acid phosphatase [Source:UniProtKB/TrEMBL;Acc:L8HFR9]	0.004859717	0.284496667	0.27963695	3.40E-09
3h	ACA1_099740/clean/scf7180000084647:18906-18996:-	ACA1_099740	COesterase domain-containing protein [Source:UniProtKB/TrEMBL;Acc:L8HMJ7]	0.010383207	0.290015867	0.27963266	6.71E-07
3h	ACA1_266200/clean/scf7180000084770:629414-629600:+	ACA1_266200	PX domain-containing protein [Source:UniProtKB/TrEMBL;Acc:L8H1K2]	0.035734967	0.315299667	0.2795647	0.000230476
3h	ACA1_396290/clean/scf7180000084759:73953-74043:+	ACA1_396290	DUF1084 domain-containing protein [Source:UniProtKB/TrEMBL;Acc:L8HDK0]	0.016371128	0.295928	0.279556872	1.34E-05
3h	ACA1_320550/clean/scf7180000084416:10566-10667:-	ACA1_320550	Proline iminopeptidase [Source:UniProtKB/TrEMBL;Acc:L8GWD8]	0.000360127	0.279751667	0.27939154	1.48E-10
3h	ACA1_146510/clean/scf7180000084713:37409-37488:-	ACA1_146510	hypothetical protein	0.000647187	0.280013333	0.279366147	3.31E-07
3h	ACA1_289760/clean/scf7180000084777:458192-458265:+	ACA1_289760	FFD and TFG box motifscontaining protein [Source:UniProtKB/TrEMBL;Acc:L8HKM6]	0.004659523	0.284015667	0.279356143	1.25E-09
3h	ACA1_362740/clean/scf7180000084624:162494-162573:-	ACA1_362740	Protein kinase domain-containing protein [Source:UniProtKB/TrEMBL;Acc:L8GFK9]	0.013818753	0.293055	0.279236247	7.98E-06
3h	ACA1_320540/clean/scf7180000084416:8164-8333:-	ACA1_320540	Calponin-homology (CH) domain-containing protein [Source:UniProtKB/TrEMBL;Acc:L8GTR4]	0.000855056	0.280019333	0.279164277	2.87E-12
3h	ACA1_255900/clean/scf7180000084768:577549-577670:+	ACA1_255900	DEP domain-containing protein [Source:UniProtKB/TrEMBL;Acc:L8HA90]	0.004406213	0.283547333	0.27914112	1.21E-10
3h	ACA1_289660/clean/scf7180000084777:439502-439570:+	ACA1_289660	Churchill domain containing 1, putative [Source:UniProtKB/TrEMBL;Acc:L8HKL9]	0.015364573	0.294459	0.279094427	3.22E-06
3h	ACA1_360060/clean/scf7180000084622:39717-39787:-	ACA1_360060	GHMP_kinases_C domain-containing protein [Source:UniProtKB/TrEMBL;Acc:L8HF07]	0.00880137	0.287798367	0.278996997	2.44E-05
3h	ACA1_154690/clean/scf7180000084722:78498-78651:+	ACA1_154690	unspecified product	0.011118163	0.289995333	0.27887717	3.40E-05
3h	ACA1_070350/clean/scf7180000084603:649556-649679:-	ACA1_070350	PKS_ER domain-containing protein [Source:UniProtKB/TrEMBL;Acc:L8HDW9]	0.000536097	0.2791742	0.278638103	2.07E-08
3h	ACA1_054370/clean/scf7180000084594:475423-475509:-	ACA1_054370	Fe2OG dioxygenase domain-containing protein [Source:UniProtKB/TrEMBL;Acc:L8H8A8]	0.030532933	0.309163	0.278630067	0.000148539
3h	ACA1_063700/clean/scf7180000084599:1477961-1478026:-	ACA1_063700	Vacuolarsorting protein SNF7, putative [Source:UniProtKB/TrEMBL;Acc:L8GZU3]	0.001280081	0.2798745	0.278594419	5.27E-09
3h	ACA1_144440/clean/scf7180000084709:245211-245380:+	ACA1_144440	SNF2 family Nterminal domain containing protein [Source:UniProtKB/TrEMBL;Acc:L8HFN1]	0.005384999	0.2839081	0.278523101	2.86E-08
3h	ACA1_037100/clean/scf7180000084567:57824-57899:-	ACA1_037100	3methyl-2-oxobutanoate hydroxymethyltransferase [Source:UniProtKB/TrEMBL;Acc:L8H379]	0.00563917	0.284148667	0.278509497	5.03E-06
3h	ACA1_171660/clean/scf7180000084741:137144-137221:+	ACA1_171660	Serine/threonine-protein phosphatase 2A 55 kDa regulatory subunit B [Source:UniProtKB/TrEMBL;Acc:L8HHC4]	0.011002673	0.289425067	0.278422393	5.67E-06
3h	ACA1_273530/clean/scf7180000084773:45176-45273:+	ACA1_273530	hypothetical protein	0.012944467	0.291226	0.278281533	6.68E-05
3h	ACA1_054220/clean/scf7180000084594:434183-434258:-	ACA1_054220	MHCK/EF2 kinase domain containing protein [Source:UniProtKB/TrEMBL;Acc:L8H897]	0.104005433	0.382150333	0.2781449	0.001532213
3h	ACA1_207940/clean/scf7180000084757:87908-88016:+	ACA1_207940	Rab-GAP TBC domain-containing protein [Source:UniProtKB/TrEMBL;Acc:L8GY97]	0.003595117	0.281722	0.278126883	5.04E-06
3h	ACA1_187290/clean/scf7180000084747:20809-20869:-	ACA1_187290	hypothetical protein	0	0.277964667	0.277964667	8.24E-07
3h	ACA1_173630/clean/scf7180000084741:498195-498315:+	ACA1_173630	RhoGEF domain containing protein [Source:UniProtKB/TrEMBL;Acc:L8HHQ8]	0.054337367	0.332301	0.277963633	0.000189228
3h	ACA1_143940/clean/scf7180000084709:111760-111853:-	ACA1_143940	VWFA domain-containing protein [Source:UniProtKB/TrEMBL;Acc:L8HEQ6]	0.00170068	0.279584267	0.277883587	9.89E-07
3h	ACA1_126680/clean/scf7180000084693:76537-76650:+	ACA1_126680	Ras family protein [Source:UniProtKB/TrEMBL;Acc:L8HAC7]	0.002002567	0.279885533	0.277882967	2.22E-10
3h	ACA1_261430/clean/scf7180000084769:675112-675187:+	ACA1_261430	Serine/threonine protein kinase, putative [Source:UniProtKB/TrEMBL;Acc:L8GG67]	0.00141243	0.2792495	0.27783707	1.62E-05
3h	ACA1_058590/clean/scf7180000084599:357646-357714:+	ACA1_058590	Copine VIII, putative [Source:UniProtKB/TrEMBL;Acc:L8GXA8]	0	0.2778113	0.2778113	3.74E-09
3h	ACA1_379300/clean/scf7180000084711:326598-326706:-	ACA1_379300	hypothetical protein	0.00425886	0.282008633	0.277749773	5.21E-06
3h	ACA1_279560/clean/scf7180000084776:415118-415190:-	ACA1_279560	Iron hydrogenase assembly protein [Source:UniProtKB/TrEMBL;Acc:L8H633]	0.058808	0.336557667	0.277749667	0.015501912
3h	ACA1_196090/clean/scf7180000084752:50802-50886:+	ACA1_196090	SAC domain-containing protein [Source:UniProtKB/TrEMBL;Acc:L8HEU4]	0.017583833	0.295268667	0.277684833	1.48E-07
3h	ACA1_380960/clean/scf7180000084712:125677-125748:-	ACA1_380960	ThrE domain-containing protein [Source:UniProtKB/TrEMBL;Acc:L8GNM4]	0.0213533	0.298929233	0.277575933	0.000304964
3h	ACA1_014630/clean/scf7180000083772:3713-3799:-	ACA1_014630	Doublestranded RNA binding motif domain containing protein [Source:UniProtKB/TrEMBL;Acc:L8HFR7]	0	0.277501	0.277501	2.13E-05

3h	ACA1_077920/clean/scf7180000084614:42862-42970:-	ACA1_077920	hypothetical protein	0.008383783	0.285825	0.277441217	9.37E-08
3h	ACA1_171980/clean/scf7180000084741:213092-213166:-	ACA1_171980	Ras subfamily protein [Source:UniProtKB/TrEMBL;Acc:L8HGG9]	0.0945587	0.371967	0.2774083	0.007678897
3h	ACA1_151330/clean/scf7180000084716:104444-104535:-	ACA1_151330	Purple acid phosphatase [Source:UniProtKB/TrEMBL;Acc:L8H0Q3]	0.009652563	0.2870121	0.277359537	1.11E-06
3h	ACA1_325560/clean/scf7180000084495:33835-33944:+	ACA1_325560	Solute carrier family protein [Source:UniProtKB/TrEMBL;Acc:L8H8Z4]	0.00686468	0.284188433	0.277323753	3.44E-07
3h	ACA1_051870/clean/scf7180000084592:69354-69427:+	ACA1_051870	Protein kinase domain-containing protein [Source:UniProtKB/TrEMBL;Acc:L8GP31]	0.020684887	0.297999667	0.27731478	1.10E-05
3h	ACA1_236110/clean/scf7180000084762:408617-408728:-	ACA1_236110	PPM-type phosphatase domain-containing protein [Source:UniProtKB/TrEMBL;Acc:L8H2W4]	0	0.277120833	0.277120833	9.22E-07
3h	ACA1_115710/clean/scf7180000084669:652966-653055:-	ACA1_115710	R3H domain-containing protein [Source:UniProtKB/TrEMBL;Acc:L8H6R6]	0.003416867	0.2804841	0.277067233	0.001962914
3h	ACA1_190120/clean/scf7180000084748:132526-132610:-	ACA1_190120	hypothetical protein	0.001438607	0.278458667	0.27702006	2.50E-06
3h	ACA1_290990/clean/scf7180000084777:714713-714910:-	ACA1_290990	HnRNPL/PTB/hephaestus splicing factor subfamily protein [Source:UniProtKB/TrEMBL;Acc:L8HLM4]	0.016403567	0.293419967	0.2770164	5.71E-06
3h	ACA1_072980/clean/scf7180000084603:1349916-1350024:-	ACA1_072980	Proteasome subunit alpha type [Source:UniProtKB/TrEMBL;Acc:L8HDS9]	0.0012005	0.278202467	0.277001966	2.33E-09
3h	ACA1_295350/clean/scf7180000084777:1112407-1112487:-	ACA1_295350	RNA_bind domain-containing protein [Source:UniProtKB/TrEMBL;Acc:L8HJR1]	0.00857157	0.285419	0.27684743	6.83E-05
3h	ACA1_126140/clean/scf7180000084693:29030-29103:-	ACA1_126140	HA domain-containing protein [Source:UniProtKB/TrEMBL;Acc:L8HAG8]	0.00349289	0.280243	0.27675011	1.98E-05
3h	ACA1_360980/clean/scf7180000084622:215276-215384:+	ACA1_360980	hypothetical protein	0.005280828	0.281998733	0.276717905	2.21E-05
3h	ACA1_373600/clean/scf7180000084679:112435-112541:-	ACA1_373600	G domain-containing protein [Source:UniProtKB/TrEMBL;Acc:L8GJC7]	0.007888133	0.284594567	0.276706433	0.001509423
3h	ACA1_282440/clean/scf7180000084776:681064-681135:+	ACA1_282440	hypothetical protein	0.022271233	0.298970767	0.276699533	0.000349403
3h	ACA1_166940/clean/scf7180000084736:33232-33325:-	ACA1_166940	Ras-GAP domain-containing protein [Source:UniProtKB/TrEMBL;Acc:L8H0R6]	0.00254631	0.278960667	0.276414357	8.51E-07
3h	ACA1_280610/clean/scf7180000084776:504770-504881:+	ACA1_280610	C2H2-type domain-containing protein [Source:UniProtKB/TrEMBL;Acc:L8H702]	0.0173815	0.293733233	0.276351733	0.006474849
3h	ACA1_038430/clean/scf7180000084568:146759-146853:+	ACA1_038430	hypothetical protein	0.002068513	0.278382333	0.27631382	1.79E-08
3h	ACA1_054480/clean/scf7180000084594:509797-509869:+	ACA1_054480	Pribosyltran domain-containing protein [Source:UniProtKB/TrEMBL;Acc:L8H652]	0.024439773	0.300704667	0.276264893	0.000950494
3h	ACA1_298640/clean/scf7180000082992:8592-8697:+	ACA1_298640	V-type proton ATPase subunit C [Source:UniProtKB/TrEMBL;Acc:L8H2N9]	0.001854118	0.277983167	0.276129049	1.15E-09
3h	ACA1_040480/clean/scf7180000084572:16045-16127:+	ACA1_040480	Chloride channel protein [Source:UniProtKB/TrEMBL;Acc:L8H1V6]	0.0348427	0.310714	0.2758713	0.0001591
3h	ACA1_191850/clean/scf7180000084749:59090-59170:-	ACA1_191850	Protein kinase domain-containing protein [Source:UniProtKB/TrEMBL;Acc:L8GN20]	0.1730881	0.448865667	0.275777567	0.022377784
3h	ACA1_270810/clean/scf7180000084770:1162947-1163029:+	ACA1_270810	C2 domain containing protein [Source:UniProtKB/TrEMBL;Acc:L8H2Z8]	0.00241546	0.2780508	0.27563534	1.42E-06
3h	ACA1_073230/clean/scf7180000084603:1376617-1376706:-	ACA1_073230	EHN domain-containing protein [Source:UniProtKB/TrEMBL;Acc:L8HDT9]	0.032897467	0.308367533	0.275470067	0.000339674
3h	ACA1_089980/clean/scf7180000084639:409728-409800:-	ACA1_089980	hypothetical protein	0.00847714	0.283846467	0.275369327	8.18E-05
3h	ACA1_180780/clean/scf7180000084744:79947-80073:-	ACA1_180780	RasGEF domain containing protein [Source:UniProtKB/TrEMBL;Acc:L8GF96]	0	0.275297333	0.275297333	1.15E-08
3h	ACA1_228910/clean/scf7180000084761:222557-222616:+	ACA1_228910	hypothetical protein	0.001461987	0.2767228	0.275260813	7.66E-06
3h	ACA1_261310/clean/scf7180000084769:633748-633847:+	ACA1_261310	Phytanoyl-CoA dioxygenase family protein [Source:UniProtKB/TrEMBL;Acc:L8GHP7]	0.014422577	0.289648267	0.27522569	0.000686569
3h	ACA1_271370/clean/scf7180000084771:37477-37569:+	ACA1_271370	PIPK domain-containing protein [Source:UniProtKB/TrEMBL;Acc:L8GP04]	0.140209	0.415414667	0.275205667	0.015910908
3h	ACA1_046130/clean/scf7180000084577:58140-58233:+	ACA1_046130	Cytochrome p450 superfamily protein [Source:UniProtKB/TrEMBL;Acc:L8H8R3]	0.010953433	0.286065667	0.275112233	1.18E-05
3h	ACA1_175700/clean/scf7180000084741:1039094-1039192:-	ACA1_175700	Frizzled/Smoothened family membrane region protein [Source:UniProtKB/TrEMBL;Acc:L8HH45]	0.0247511	0.299830367	0.275079267	0.000937358
3h	ACA1_031620/clean/scf7180000084522:26885-26972:-	ACA1_031620	Prenylcysteine lyase, putative [Source:UniProtKB/TrEMBL;Acc:L8GS08]	0.136228	0.411301	0.275073	0.012467126
3h	ACA1_184420/clean/scf7180000084745:638437-638589:+	ACA1_184420	Cathepsin Llike proteinase [Source:UniProtKB/TrEMBL;Acc:L8H8J9]	0	0.275021633	0.275021633	2.21E-09
3h	ACA1_148480/clean/scf7180000084715:10674-10768:-	ACA1_148480	I/LWEQ domain containing protein [Source:UniProtKB/TrEMBL;Acc:L8HEA2]	0.000969863	0.2759726	0.275002737	4.95E-08
3h	ACA1_369660/clean/scf7180000084664:350925-350997:-	ACA1_369660	Signal recognition particle subunit SRP72 [Source:UniProtKB/TrEMBL;Acc:L8H0D1]	0.014365531	0.289342333	0.274976802	3.16E-05
3h	ACA1_196310/clean/scf7180000084752:56942-57152:+	ACA1_196310	SET domain-containing protein [Source:UniProtKB/TrEMBL;Acc:L8HDZ8]	0.697408	0.972380333	0.274972333	0.0007405
3h	ACA1_336430/clean/scf7180000084593:74795-74882:-	ACA1_336430	Cysteine protease [Source:UniProtKB/TrEMBL;Acc:L8H1P1]	0.000871113	0.275827667	0.274956553	7.09E-08
3h	ACA1_323140/clean/scf7180000084467:14504-14593:-	ACA1_323140	RFX-type winged-helix domain-containing protein [Source:UniProtKB/TrEMBL;Acc:L8GEG4]	0.002773437	0.277535167	0.27476173	0.00068867
3h	ACA1_048130/clean/scf7180000084582:30103-30266:-	ACA1_048130	Charged multivesicular body protein, putative [Source:UniProtKB/TrEMBL;Acc:L8GDU5]	0.000873671	0.275555333	0.274681662	1.48E-07
3h	ACA1_162370/clean/scf7180000084726:43208-43287:-	ACA1_162370	Protein kinase domain-containing protein [Source:UniProtKB/TrEMBL;Acc:L8GYN7]	0.00154321	0.276179933	0.274636723	1.62E-06
3h	ACA1_210530/clean/scf7180000084758:48664-48756:-	ACA1_210530	4Fe-4S ferredoxin-type domain-containing protein [Source:UniProtKB/TrEMBL;Acc:L8GS75]	0.014934903	0.289341333	0.27440643	6.38E-05
3h	ACA1_379300/clean/scf7180000084711:326415-326507:-	ACA1_379300	hypothetical protein	0.003332267	0.277624	0.274291733	2.39E-06
3h	ACA1_179960/clean/scf7180000084744:8281-8380:-	ACA1_179960	Protein kinase domain/MORN repeatcontaining protein [Source:UniProtKB/TrEMBL;Acc:L8GC88]	0.0344785	0.308741	0.2742625	7.13E-06
3h	ACA1_066150/clean/scf7180000084599:2013202-2013278:+	ACA1_066150	Myosin head (Motor domain) domain containing protein [Source:UniProtKB/TrEMBL;Acc:L8GX00]	0	0.274170667	0.274170667	0.003538096
3h	ACA1_074110/clean/scf7180000084605:25988-26082:-	ACA1_074110	Peptidase [Source:UniProtKB/TrEMBL;Acc:L8HJ70]	0.005267033	0.27922	0.273952967	2.29E-06
3h	ACA1_072590/clean/scf7180000084603:1230128-1230225:-	ACA1_072590	Kynurenine 3-monooxygenase [Source:UniProtKB/TrEMBL;Acc:L8HEI5]	0.003426693	0.2772778	0.273851107	7.74E-06
3h	ACA1_157910/clean/scf7180000084724:149799-149892:+	ACA1_157910	Leucine rich repeat domain containing protein [Source:UniProtKB/TrEMBL;Acc:L8H970]	0.027787967	0.3016228	0.273834833	0.002066758
3h	ACA1_062850/clean/scf7180000084599:1285680-1285750:+	ACA1_062850	WD_REPEATS_REGION domain-containing protein [Source:UniProtKB/TrEMBL;Acc:L8GZK3]	0.00113572	0.274906	0.27377028	1.08E-07
3h	ACA1_198700/clean/scf7180000084753:155247-155326:-	ACA1_198700	ABC transporter domain-containing protein [Source:UniProtKB/TrEMBL;Acc:L8H2D9]	0.005163663	0.278890433	0.27372677	1.07E-07
3h	ACA1_388770/clean/scf7180000084733:164346-164440:+	ACA1_388770	Poly(A) polymerase central domain containing protein [Source:UniProtKB/TrEMBL;Acc:L8GG84]	0.0108848	0.2845987	0.2737139	0.000237064
3h	ACA1_098160/clean/scf7180000084645:1410695-1410761:-	ACA1_098160	ABC transporter, ATPbinding domain containing protein [Source:UniProtKB/TrEMBL;Acc:L8GJY4]	0.001311897	0.274961433	0.273649537	5.57E-08
3h	ACA1_100460/clean/scf7180000084648:88440-88506:-	ACA1_100460	ABC transporter [Source:UniProtKB/TrEMBL;Acc:L8GK53]	0	0.273617333	0.273617333	0.002067767
3h	ACA1_236350/clean/scf7180000084762:443560-443673:+	ACA1_236350	Peptidase_M28 domain-containing protein [Source:UniProtKB/TrEMBL;Acc:L8H1H1]	0.008536247	0.282123	0.273586753	9.22E-09
3h	ACA1_238450/clean/scf7180000084763:95952-96024:-	ACA1_238450	TLDc domain-containing protein [Source:UniProtKB/TrEMBL;Acc:L8GM61]	0.035818267	0.309389933	0.273571667	0.000211626
3h	ACA1_146600/clean/scf7180000084713:65349-65464:-	ACA1_146600	Protein kinase domain containing protein [Source:UniProtKB/TrEMBL;Acc:L8GWQ5]	0.001102602	0.274635333	0.273532732	1.17E-08
3h	ACA1_117130/clean/scf7180000084669:922596-922676:+	ACA1_117130	Eukaryotic initiation factor 4e, putative [Source:UniProtKB/TrEMBL;Acc:L8H4Z1]	0.007987093	0.2814486	0.273461507	2.07E-06
3h	ACA1_031780/clean/scf7180000084523:1694-1844:-	ACA1_031780	Lactamase_B domain-containing protein [Source:UniProtKB/TrEMBL;Acc:L8H103]	0.015278567	0.288472067	0.2731935	0.00316719
3h	ACA1_144200/clean/scf7180000084709:170745-170841:+	ACA1_144200	PH domain-containing protein [Source:UniProtKB/TrEMBL;Acc:L8HET7]	0.004067113	0.2772434	0.273176287	7.37E-10
3h	ACA1_114860/clean/scf7180000084669:567689-567813:-	ACA1_114860	ANK_REP_REGION domain-containing protein [Source:UniProtKB/TrEMBL;Acc:L8H6N8]	0.015778487	0.288918	0.273139513	6.11E-06
3h	ACA1_369850/clean/scf7180000084664:395768-395843:-	ACA1_369850	ATPase with chaperone activity, putative [Source:UniProtKB/TrEMBL;Acc:L8GZ67]	0	0.272960733	0.272960733	3.86E-07
3h	ACA1_133450/clean/scf7180000084701:60189-60275:+	ACA1_133450	HECT domain-containing protein [Source:UniProtKB/TrEMBL;Acc:L8H3L8]	0.0180643	0.291010367	0.272946067	0.000865764
3h	ACA1_101330/clean/scf7180000084649:46882-46979:+	ACA1_101330	t-SNARE coiled-coil homology domain-containing protein [Source:UniProtKB/TrEMBL;Acc:L8GGH6]	0.0194357	0.292330333	0.272894633	0.001317538
3h	ACA1_077300/clean/scf7180000084612:287557-287643:+	ACA1_077300	Serine/threonine kinase [Source:UniProtKB/TrEMBL;Acc:L8GP03]	0.009259267	0.282102567	0.2728433	0.016190266
3h	ACA1_202400/clean/scf7180000084754:37618-37710:+	ACA1_202400	K+ channel protein [Source:UniProtKB/TrEMBL;Acc:M0QSM5]	0.0496927	0.322521	0.2728283	0.000498059
3h	ACA1_144430/clean/scf7180000084709:240637-240732:-	ACA1_144430	CASP_C domain-containing protein [Source:UniProtKB/TrEMBL;Acc:L8HI28]	0.0197745	0.292497233	0.272722733	7.69E-06
3h	ACA1_128490/clean/scf7180000084697:71267-71367:-	ACA1_128490	RPN2_C domain-containing protein [Source:UniProtKB/TrEMBL;Acc:L8GVI7]	0.001771248	0.274493333	0.272722085	8.63E-08
3h	ACA1_164680/clean/scf7180000084729:247960-248059:-	ACA1_164680	ANK_REP_REGION domain-containing protein [Source:UniProtKB/TrEMBL;Acc:L8GSN8]	0.0121711	0.2847668	0.2725957	3.26E-05
3h	ACA1_092030/clean/scf7180000084645:251736-251839:-	ACA1_092030	Nudix hydrolase domain-containing protein [Source:UniProtKB/TrEMBL;Acc:L8GIW6]	0	0.272501767	0.272501767	8.38E-05
3h	ACA1_390520/clean/scf7180000084740:55901-55966:+	ACA1_390520	EnoylCoA hydratase/isomerase family domain containing protein [Source:UniProtKB/TrEMBL;Acc:L8GPA6]	0.0051282	0.277458667	0.272330467	2.36E-06
3h	ACA1_158810/clean/scf7180000084724:259201-259346:-	ACA1_158810	Coatomer subunit epsilon [Source:UniProtKB/TrEMBL;Acc:L8HCK7]	0.013890567	0.286130667	0.2722401	2.63E-07
3h	ACA1_060250/clean/scf7180000084599:627094-627214:-	ACA1_060250	Alpha-carbonic anhydrase domain-containing protein [Source:UniProtKB/TrEMBL;Acc:L8GXK3]	0.005752037	0.277973	0.272220963	1.31E-08
3h	ACA1_107440/clean/scf7180000084660:197703-197817:+	ACA1_107440	Purple acid phosphatase [Source:UniProtKB/TrEMBL;Acc:L8GNJ7]	0.001746237	0.2739071	0.272160863	2.30E-08
3h	ACA1_074160/clean/scf7180000084605:39009-39090:+	ACA1_074160	Aamy domain-containing protein [Source:UniProtKB/TrEMBL;Acc:L8HJ75]	0.00385402	0.275983867	0.272129847	9.41E-07
3h	ACA1_183980/clean/scf7180000084745:535259-535356:-	ACA1_183980	Protein-S-isoprenylcysteine O-methyltransferase [Source:UniProtKB/TrEMBL;Acc:L8H887]	0.020551697	0.2926459	0.272094203	0.000200783
3h	ACA1_130850/clean/scf7180000084698:135236-135333:+	ACA1_130850	AMPbinding enzyme domain containing protein [Source:UniProtKB/TrEMBL;Acc:L8GPF1]	0.005325753	0.277349	0.272023247	5.71E-05
3h	ACA1_289330/clean/scf7180000084777:417144-417218:+	ACA1_289330	U-box domain-containing protein [Source:UniProtKB/TrEMBL;Acc:L8HJ41]	0.054477433	0.326493267	0.272015833	0.002705339
3h	ACA1_273830/clean/scf7180000084773:65230-65320:+	ACA1_273830	DUF4209 domain-containing protein [Source:UniProtKB/TrEMBL;Acc:L8GI54]	0	0.271988567	0.271988567	0.000889647
3h	ACA1_252460/clean/scf7180000084768:155684-155783:-	ACA1_252460	Isovaleryl Coenzyme A dehydrogenase [Source:UniProtKB/TrEMBL;Acc:L8HD26]	0.000516277	0.272404033	0.271887757	2.25E-08
3h	ACA1_383060/clean/scf7180000084721:134017-134123:+	ACA1_383060	hypothetical protein	0.030856633	0.302691667	0.271835033	9.30E-06
3h	ACA1_193520/clean/scf7180000084749:250499-250569:-	ACA1_193520	hypothetical protein	0.014862	0.286661567	0.271799567	0.004224253
3h	ACA1_183240/clean/scf7180000084745:361740-361814:+	ACA1_183240	LysM domain-containing protein [Source:UniProtKB/TrEMBL;Acc:L8H898]	0.00946129	0.281212567	0.271751277	5.19E-06
3h	ACA1_384590/clean/scf7180000084730:59547-59635:-	ACA1_384590	RAB1B, member RAS oncogene family [Source:UniProtKB/TrEMBL;Acc:L8H896]	0.017339667	0.288916367	0.2715767	2.03E-05
3h	ACA1_054820/clean/scf7180000084594:585013-585108:-	ACA1_054820	Tetratricopeptide repeat domain containing protein [Source:UniProtKB/TrEMBL;Acc:L8H8E7]	0	0.271530333	0.271530333	4.48E-06
3h	ACA1_298630/clean/scf7180000082992:4993-5129:-	ACA1_298630	Vacuolar protein sorting 16, putative [Source:UniProtKB/TrEMBL;Acc:L8H081]	0.00309613	0.2745355	0.27143937	4.49E-07
3h	ACA1_246510/clean/scf7180000084763:565324-565389:+	ACA1_246510	PPM-type phosphatase domain-containing protein [Source:UniProtKB/TrEMBL;Acc:L8GL94]	0.000277052	0.271691367	0.271414315	3.16E-07
3h	ACA1_146600/clean/scf7180000084713:66067-66166:-	ACA1_146600	Protein kinase domain containing protein [Source:UniProtKB/TrEMBL;Acc:L8GWQ5]	0.001184987	0.2725067	0.271321713	7.95E-08
3h	ACA1_014870/clean/scf7180000083772:13193-13276:+	ACA1_014870	tRNA phosphotransferase 1 isoform 1, putative [Source:UniProtKB/TrEMBL;Acc:L8HI60]	9.57E-05	0.271395367	0.27129962	4.97E-07
3h	ACA1_069510/clean/scf7180000084603:459844-459916:-	ACA1_069510	hypothetical protein	0.006535933	0.277692667	0.271156733	6.45E-05
3h	ACA1_143070/clean/scf7180000084709:19391-19468:+	ACA1_143070	ATPase, AAA domain containing protein [Source:UniProtKB/TrEMBL;Acc:L8HFD9]	0.002380953	0.273535967	0.271155013	1.38E-07
3h	ACA1_078990/clean/scf7180000084615:123952-124044:-	ACA1_078990	AcylCoA synthetase, putative [Source:UniProtKB/TrEMBL;Acc:L8GRK9]	0.000367823	0.271437367	0.271069544	2.07E-09
3h	ACA1_369600/clean/scf7180000084664:343722-343820:+	ACA1_369600	PH domain containing protein [Source:UniProtKB/TrEMBL;Acc:L8GZ46]	0.003144653	0.2741798	0.271035147	6.30E-05
3h	ACA1_229570/clean/scf7180000084761:280357-280450:-	ACA1_229570	Oxidoreductase, short chain dehydrogenase/reductase superfamily protein [Source:UniProtKB/TrEMBL;Acc:L8H814]	0	0.271020167	0.271020167	1.51E-06
3h	ACA1_380820/clean/scf7180000084712:93336-93404:+	ACA1_380820	tRNA-binding domain-containing protein [Source:UniProtKB/TrEMBL;Acc:L8GMV0]	0.0281586	0.299153433	0.270994833	2.93E-05
3h	ACA1_247140/clean/scf7180000084763:629858-630020:+	ACA1_247140	hypothetical protein	0.129648667	0.400601667	0.270953	0.007639769
3h	ACA1_211140/clean/scf7180000084758:75843-75920:+	ACA1_211140	Ser/Thr protein phosphatase [Source:UniProtKB/TrEMBL;Acc:L8GS86]	0.001746723	0.272673933	0.27092721	0.000132973
3h	ACA1_228910/clean/scf7180000084761:221604-221704:+	ACA1_228910	hypothetical protein	0.000683055	0.271601	0.270917945	5.00E-07
3h	ACA1_142680/clean/scf7180000084708:220477-220594:+	ACA1_142680	unspecified product	0.003468457	0.274297567	0.27082911	1.06E-07
3h	ACA1_080670/clean/scf7180000084618:60100-60180:+	ACA1_080670	Non-specific serine/threonine protein kinase [Source:UniProtKB/TrEMBL;Acc:L8HBV7]	0.00335393	0.274176133	0.270822203	1.37E-08
3h	ACA1_142600/clean/scf7180000084708:198585-198652:+	ACA1_142600	unspecified product	0.024084833	0.294836567	0.270751733	1.25E-05
3h	ACA1_043200/clean/scf7180000084573:138025-138092:-	ACA1_043200	ACB domain-containing protein [Source:UniProtKB/TrEMBL;Acc:L8GUP2]	0.004697933	0.275406	0.270708067	6.66E-08
3h	ACA1_387910/clean/scf7180000084733:86416-86560:-	ACA1_387910	P/Homo B domain-containing protein [Source:UniProtKB/TrEMBL;Acc:L8GG58]	0.0118708	0.2824206	0.2705498	0.000166385
3h	ACA1_222680/clean/scf7180000084760:50554-50617:-	ACA1_222680	FAD-binding FR-type domain-containing protein [Source:UniProtKB/TrEMBL;Acc:L8GST4]	0.011737903	0.282235333	0.27049743	2.74E-05
3h	ACA1_198300/clean/scf7180000084753:76180-76287:-	ACA1_198300	hypothetical protein	0.002624673	0.273082433	0.27045776	1.76E-06
3h	ACA1_377530/clean/scf7180000084711:84039-84116:-	ACA1_377530	Protein kinase domain-containing protein [Source:UniProtKB/TrEMBL;Acc:L8GR81]	0	0.2704125	0.2704125	1.76E-05
3h	ACA1_321380/clean/scf7180000084439:20586-20683:+	ACA1_321380	RasGEF domain containing protein [Source:UniProtKB/TrEMBL;Acc:L8HKN6]	0.0168823	0.28722	0.2703377	4.89E-05
3h	ACA1_057400/clean/scf7180000084599:133357-133469:+	ACA1_057400	Leucine rich repeat domain containing protein [Source:UniProtKB/TrEMBL;Acc:L8GVU1]	0.060739633	0.331038433	0.2702988	0.011007261
3h	ACA1_040840/clean/scf7180000084572:93523-93594:-	ACA1_040840	BRO1 domain-containing protein [Source:UniProtKB/TrEMBL;Acc:L8H1X9]	0.000661047	0.270950633	0.270289587	6.20E-08
3h	ACA1_037490/clean/scf7180000084567:102610-102718:-	ACA1_037490	hypothetical protein	0.021698913	0.291841733	0.27014282	0.001109366
3h	ACA1_227830/clean/scf7180000084761:148464-148549:+	ACA1_227830	PhoPQactivated pathogenicity-related protein-like protein, putative [Source:UniProtKB/TrEMBL;Acc:L8H8Q5]	1.24E-05	0.270149267	0.27013687	1.43E-11
3h	ACA1_116920/clean/scf7180000084669:873673-873763:-	ACA1_116920	hypothetical protein	0	0.270075233	0.270075233	1.85E-07
3h	ACA1_183480/clean/scf7180000084745:425959-426075:-	ACA1_183480	EF hand,SPla/RYanodine receptor (SPRY) domain containing protein [Source:UniProtKB/TrEMBL;Acc:L8H9K0]	0.00696812	0.2770368	0.27006868	1.38E-06
3h	ACA1_138800/clean/scf7180000084705:12684-12782:+	ACA1_138800	hypothetical protein	0.026197533	0.29626	0.270062467	4.78E-06
3h	ACA1_064510/clean/scf7180000084599:1629940-1630010:-	ACA1_064510	Small GTPbinding protein domain containing protein [Source:UniProtKB/TrEMBL;Acc:L8GWS3]	0.072001133	0.342060333	0.2700592	0.000892648
3h	ACA1_378280/clean/scf7180000084711:229457-229530:+	ACA1_378280	hypothetical protein	0.196003333	0.466045333	0.270042	0.032934142
3h	ACA1_228910/clean/scf7180000084761:222724-222807:+	ACA1_228910	hypothetical protein	0.013950767	0.283868333	0.269917567	5.36E-06
3h	ACA1_052390/clean/scf7180000084594:56958-57062:-	ACA1_052390	Nop domain-containing protein [Source:UniProtKB/TrEMBL;Acc:L8H4W4]	0.020424633	0.2901967	0.269772067	0.001329583
3h	ACA1_279740/clean/scf7180000084776:433643-433735:+	ACA1_279740	BATS domain-containing protein [Source:UniProtKB/TrEMBL;Acc:L8H6X4]	0.009358103	0.279097667	0.269739563	0.001483653
3h	ACA1_142840/clean/scf7180000084708:245796-245869:-	ACA1_142840	hypothetical protein	0.018385933	0.288081133	0.2696952	0.000262536
3h	ACA1_322420/clean/scf7180000084463:22720-22833:-	ACA1_322420	hypothetical protein	0.00660373	0.276043	0.26943927	2.22E-05
3h	ACA1_138990/clean/scf7180000084705:50500-50587:+	ACA1_138990	AP-1 complex subunit gamma [Source:UniProtKB/TrEMBL;Acc:L8GMK5]	0.026806933	0.296188333	0.2693814	7.08E-06
3h	ACA1_396930/clean/scf7180000084759:151602-151744:+	ACA1_396930	hypothetical protein	0.00316023	0.27249	0.26932977	5.42E-09
3h	ACA1_234220/clean/scf7180000084762:229096-229177:-	ACA1_234220	hypothetical protein	0.112136	0.381461667	0.269325667	0.005657769
3h	ACA1_144080/clean/scf7180000084709:147575-147640:+	ACA1_144080	hypothetical protein	0.00506073	0.274351333	0.269290603	9.84E-06
3h	ACA1_257990/clean/scf7180000084769:197272-197394:+	ACA1_257990	hypothetical protein	0.003706133	0.2729857	0.269279567	0.00026001
3h	ACA1_149410/clean/scf7180000084715:147881-147969:+	ACA1_149410	Lectin_legB domain-containing protein [Source:UniProtKB/TrEMBL;Acc:L8HDD6]	0.003296473	0.272497867	0.269201393	4.60E-06
3h	ACA1_287820/clean/scf7180000084777:87080-87184:+	ACA1_287820	TRANSKETOLASE_1 domain-containing protein [Source:UniProtKB/TrEMBL;Acc:L8HL41]	0.000656671	0.269840333	0.269183662	1.69E-10
3h	ACA1_098210/clean/scf7180000084645:1430260-1430332:+	ACA1_098210	Peroxidase, putative [Source:UniProtKB/TrEMBL;Acc:L8GJY8]	0	0.269171333	0.269171333	3.15E-06
3h	ACA1_394860/clean/scf7180000084756:140559-140644:+	ACA1_394860	PA domain containing protein [Source:UniProtKB/TrEMBL;Acc:L8GZN2]	0	0.269170333	0.269170333	9.75E-08
3h	ACA1_393390/clean/scf7180000084756:55937-56053:+	ACA1_393390	Exostosin domain-containing protein [Source:UniProtKB/TrEMBL;Acc:L8H1N1]	0.013386913	0.282550967	0.269164053	0.000118529
3h	ACA1_207940/clean/scf7180000084757:88473-88563:+	ACA1_207940	Rab-GAP TBC domain-containing protein [Source:UniProtKB/TrEMBL;Acc:L8GY97]	0.006286053	0.275240667	0.268954613	1.01E-05
3h	ACA1_220060/clean/scf7180000084758:852190-852333:+	ACA1_220060	SAP domain-containing protein [Source:UniProtKB/TrEMBL;Acc:L8GQ17]	0.004765497	0.273645733	0.268880237	3.06E-08
3h	ACA1_399880/clean/scf7180000084775:56003-56114:+	ACA1_399880	Rab-GAP TBC domain-containing protein [Source:UniProtKB/TrEMBL;Acc:L8GGF5]	0.00259711	0.271394033	0.268796923	3.11E-05
3h	ACA1_255900/clean/scf7180000084768:578618-578755:+	ACA1_255900	DEP domain-containing protein [Source:UniProtKB/TrEMBL;Acc:L8HA90]	0.068952633	0.337738667	0.268786033	0.000120395
3h	ACA1_368250/clean/scf7180000084664:90501-90593:-	ACA1_368250	BPI2 domain-containing protein [Source:UniProtKB/TrEMBL;Acc:L8GY12]	0.013464473	0.282246	0.268781527	6.75E-06
3h	ACA1_054190/clean/scf7180000084594:421174-421301:+	ACA1_054190	Rasrelated protein ralB-B, putative [Source:UniProtKB/TrEMBL;Acc:L8H599]	0.015503173	0.2842843	0.268781127	3.75E-07
3h	ACA1_223330/clean/scf7180000084760:131534-131635:+	ACA1_223330	Peptidase_S8 domain-containing protein [Source:UniProtKB/TrEMBL;Acc:L8GSQ9]	0.002956177	0.271633333	0.268677157	1.58E-10
3h	ACA1_040840/clean/scf7180000084572:91030-91097:-	ACA1_040840	BRO1 domain-containing protein [Source:UniProtKB/TrEMBL;Acc:L8H1X9]	0.00140056	0.270035233	0.268634673	1.27E-07
3h	ACA1_214850/clean/scf7180000084758:245998-246062:+	ACA1_214850	LIM zinc-binding domain-containing protein [Source:UniProtKB/TrEMBL;Acc:L8GSG1]	0.008968677	0.277504667	0.26853599	7.00E-08

3h	ACA1_287660/clean/scf7180000084777:57133-57198:+	ACA1_287660	Pentapeptide repeatcontaining protein [Source:UniProtKB/TrEMBL;Acc:L8HIP0]	0.008269003	0.276773667	0.268504663	1.97E-06
3h	ACA1_217280/clean/scf7180000084758:557246-557373:+	ACA1_217280	Protein kinase domain containing protein [Source:UniProtKB/TrEMBL;Acc:L8GQB1]	0.013586947	0.281924033	0.268337087	3.81E-06
3h	ACA1_155710/clean/scf7180000084722:223694-223826:-	ACA1_155710	TAF6_C domain-containing protein [Source:UniProtKB/TrEMBL;Acc:L8GZG8]	0.008555083	0.276883	0.268327917	1.72E-05
3h	ACA1_277400/clean/scf7180000084776:23241-23309:-	ACA1_277400	RFX-type winged-helix domain-containing protein [Source:UniProtKB/TrEMBL;Acc:L8H6F5]	0.018518533	0.286739	0.268220467	0.000514997
3h	ACA1_038280/clean/scf7180000084568:117491-117569:+	ACA1_038280	CPSF_A domain-containing protein [Source:UniProtKB/TrEMBL;Acc:L8GLR0]	0.00132275	0.269531	0.26820825	1.41E-07
3h	ACA1_138810/clean/scf7180000084705:14662-14755:-	ACA1_138810	Signal transducer and activator of transcription [Source:UniProtKB/TrEMBL;Acc:L8GN02]	0.014075053	0.282141667	0.268066613	2.79E-07
3h	ACA1_133450/clean/scf7180000084701:55591-55716:+	ACA1_133450	HECT domain-containing protein [Source:UniProtKB/TrEMBL;Acc:L8H3L8]	0	0.268041133	0.268041133	6.79E-05
3h	ACA1_192100/clean/scf7180000084749:100759-100936:+	ACA1_192100	L51_S25_CI-B8 domain-containing protein [Source:UniProtKB/TrEMBL;Acc:L8GN37]	0.001911041	0.269884067	0.267973026	3.53E-10
3h	ACA1_088960/clean/scf7180000084639:273907-273985:-	ACA1_088960	Beta-lactamase domain-containing protein [Source:UniProtKB/TrEMBL;Acc:L8GU04]	0.005221927	0.2731863	0.267964373	5.88E-05
3h	ACA1_038350/clean/scf7180000084568:131015-131095:-	ACA1_038350	Fbox domain containing protein [Source:UniProtKB/TrEMBL;Acc:L8GKR6]	0.004180755	0.272135333	0.267954579	1.40E-05
3h	ACA1_364570/clean/scf7180000084641:148905-148976:+	ACA1_364570	hypothetical protein	0.018802467	0.286659333	0.267856867	7.52E-05
3h	ACA1_142690/clean/scf7180000084708:222701-222806:+	ACA1_142690	unspecified product	0.0297944	0.2976429	0.2678485	0.000176685
3h	ACA1_274150/clean/scf7180000084773:88418-88575:-	ACA1_274150	6PF2K domain-containing protein [Source:UniProtKB/TrEMBL;Acc:L8GI65]	0.082156367	0.349840667	0.2676843	0.032445602
3h	ACA1_199500/clean/scf7180000084753:247552-247646:+	ACA1_199500	1acylglycerophosphocholine O-acyltransferase 1, putative [Source:UniProtKB/TrEMBL;Acc:L8H4L3]	0.002318033	0.269975667	0.267657633	0.001934338
3h	ACA1_038180/clean/scf7180000084568:90337-90406:+	ACA1_038180	FHA domain containing protein [Source:UniProtKB/TrEMBL;Acc:L8GLQ3]	0.018745023	0.286353	0.267607977	2.49E-05
3h	ACA1_174170/clean/scf7180000084741:632361-632467:-	ACA1_174170	Glutathione Stransferase, C-terminal domain containing protein [Source:UniProtKB/TrEMBL;Acc:L8HHR9]	0.002641277	0.2701671	0.267525823	1.89E-11
3h	ACA1_101530/clean/scf7180000084649:86478-86546:-	ACA1_101530	6phosphogluconate dehydrogenase NAD-binding domain containing protein [Source:UniProtKB/TrEMBL;Acc:L8GGL6]	0.00866109	0.275932	0.26727091	4.10E-07
3h	ACA1_071930/clean/scf7180000084603:1111870-1111938:+	ACA1_071930	hypothetical protein	0.015102	0.28234	0.267238	0.000811323
3h	ACA1_382920/clean/scf7180000084721:120003-120076:-	ACA1_382920	Ras subfamily protein [Source:UniProtKB/TrEMBL;Acc:L8GV31]	0.014492767	0.281700933	0.267208167	0.002468722
3h	ACA1_063300/clean/scf7180000084599:1377784-1377848:-	ACA1_063300	Transporter, small conductance mechanosensitive ion channel (MscS) family protein [Source:UniProtKB/TrEMBL;Acc:L8GZP0]	0.008620963	0.275807233	0.26718627	0.000277681
3h	ACA1_158790/clean/scf7180000084724:256532-256679:+	ACA1_158790	Peroxiredoxin [Source:UniProtKB/TrEMBL;Acc:L8H9D0]	0.001619529	0.2686947	0.267075171	1.64E-08
3h	ACA1_091040/clean/scf7180000084645:46974-47036:-	ACA1_091040	Amidohydro-rel domain-containing protein [Source:UniProtKB/TrEMBL;Acc:L8GIF1]	0	0.267041867	0.267041867	8.79E-06
3h	ACA1_382080/clean/scf7180000084721:1129-1184:-	ACA1_382080	Ubiquitin-like protein ATG12 [Source:UniProtKB/TrEMBL;Acc:L8GUS5]	0.0107527	0.277750333	0.266997633	4.64E-05
3h	ACA1_380350/clean/scf7180000084712:43172-43252:+	ACA1_380350	Membrane protein, putative [Source:UniProtKB/TrEMBL;Acc:L8GMT8]	0.005640787	0.272505333	0.266864547	2.10E-06
3h	ACA1_061700/clean/scf7180000084599:1012685-1012751:+	ACA1_061700	Amine oxidase family protein [Source:UniProtKB/TrEMBL;Acc:L8GWP7]	0.005533933	0.272348667	0.266814733	6.14E-05
3h	ACA1_176030/clean/scf7180000084741:1109725-1109809:-	ACA1_176030	ATPase, AAA domain containing protein [Source:UniProtKB/TrEMBL;Acc:L8HI90]	0	0.2666946	0.2666946	0.000355187
3h	ACA1_399960/clean/scf7180000084775:82524-82615:-	ACA1_399960	WD_REPEATS_REGION domain-containing protein [Source:UniProtKB/TrEMBL;Acc:L8GID8]	0.011141293	0.277704067	0.266562773	0.000100211
3h	ACA1_040480/clean/scf7180000084572:16223-16307:+	ACA1_040480	Chloride channel protein [Source:UniProtKB/TrEMBL;Acc:L8H1V6]	0.026461033	0.292985033	0.266524	0.000179004
3h	ACA1_370300/clean/scf7180000084664:490668-490743:-	ACA1_370300	hypothetical protein	0.00055493	0.267072567	0.266517637	2.92E-08
3h	ACA1_054500/clean/scf7180000084594:514938-515125:-	ACA1_054500	Glycosyl hydrolase family 65 central catalytic domain containing protein [Source:UniProtKB/TrEMBL;Acc:L8H5C3]	0.000507967	0.266953133	0.266445167	5.24E-08
3h	ACA1_310750/clean/scf7180000083780:5895-5971:+	ACA1_310750	RFX-type winged-helix domain-containing protein [Source:UniProtKB/TrEMBL;Acc:L8GVY4]	0.0006488	0.267092067	0.266443267	9.13E-06
3h	ACA1_394860/clean/scf7180000084756:140017-140090:+	ACA1_394860	PA domain containing protein [Source:UniProtKB/TrEMBL;Acc:L8GZN2]	0.00079019	0.267067767	0.266277577	1.95E-07
3h	ACA1_382370/clean/scf7180000084721:40175-40301:-	ACA1_382370	P-type domain-containing protein [Source:UniProtKB/TrEMBL;Acc:L8GVY3]	0.003052557	0.269291667	0.26623911	2.74E-07
3h	ACA1_373690/clean/scf7180000084679:142121-142210:+	ACA1_373690	ADPribosylglycohydrolase superfamily protein [Source:UniProtKB/TrEMBL;Acc:L8GGT7]	0.00419854	0.270315733	0.266117193	0.000152395
3h	ACA1_193190/clean/scf7180000084749:208023-208090:-	ACA1_193190	Sep15_SelM domain-containing protein [Source:UniProtKB/TrEMBL;Acc:L8GQW0]	0.00364541	0.2697557	0.26611029	2.18E-07
3h	ACA1_237870/clean/scf7180000084763:36569-36706:+	ACA1_237870	Methyltransferase [Source:UniProtKB/TrEMBL;Acc:L8GKC5]	0.005663927	0.271698667	0.26603474	1.17E-10
3h	ACA1_173060/clean/scf7180000084741:393395-393484:-	ACA1_173060	SNARE associated Golgi protein [Source:UniProtKB/TrEMBL;Acc:L8HGL7]	0.046120233	0.312127	0.266006767	0.00512169
3h	ACA1_113490/clean/scf7180000084669:293914-294054:-	ACA1_113490	SAM domain-containing protein [Source:UniProtKB/TrEMBL;Acc:L8H3A5]	0.044809467	0.310794967	0.2659855	0.000882393
3h	ACA1_236630/clean/scf7180000084762:464249-464350:-	ACA1_236630	PX domain-containing protein [Source:UniProtKB/TrEMBL;Acc:L8H1G0]	0	0.265862267	0.265862267	2.52E-05
3h	ACA1_295900/clean/scf7180000084777:1256596-1256657:-	ACA1_295900	hypothetical protein	0.016894133	0.282728333	0.2658342	2.10E-05
3h	ACA1_203120/clean/scf7180000084754:148109-148226:+	ACA1_203120	Ras-GEF domain-containing protein [Source:UniProtKB/TrEMBL;Acc:L8GUK0]	0.052849633	0.3185678	0.265718167	0.009873877
3h	ACA1_100670/clean/scf7180000084648:147761-147872:+	ACA1_100670	hypothetical protein	0.003353467	0.269011333	0.265657867	5.00E-06
3h	ACA1_295390/clean/scf7180000084777:1125158-1125265:-	ACA1_295390	Aamy domain-containing protein [Source:UniProtKB/TrEMBL;Acc:L8HIM5]	0.0004494	0.266105067	0.265655667	1.83E-08
3h	ACA1_385360/clean/scf7180000084730:147395-147497:-	ACA1_385360	RasGEF domain containing protein [Source:UniProtKB/TrEMBL;Acc:L8H9A3]	0.015108967	0.280645	0.265536033	3.73E-05
3h	ACA1_228720/clean/scf7180000084761:196202-196282:+	ACA1_228720	BRO1 domain-containing protein [Source:UniProtKB/TrEMBL;Acc:L8HB55]	0.001545597	0.2670777	0.265532103	5.29E-07
3h	ACA1_366150/clean/scf7180000084641:363755-363867:-	ACA1_366150	tRNA methyltransferase, putative [Source:UniProtKB/TrEMBL;Acc:L8GMF0]	0.005291	0.270801333	0.265510333	3.21E-05
3h	ACA1_100090/clean/scf7180000084648:35188-35282:-	ACA1_100090	Fbox domain containing protein [Source:UniProtKB/TrEMBL;Acc:L8GK42]	0.00500448	0.270461833	0.265457353	0.000189027
3h	ACA1_376720/clean/scf7180000084685:145989-146111:-	ACA1_376720	LIM zinc-binding domain-containing protein [Source:UniProtKB/TrEMBL;Acc:L8HHL9]	0.004010237	0.269457533	0.265447297	4.55E-10
3h	ACA1_183970/clean/scf7180000084745:532902-532979:-	ACA1_183970	hypothetical protein	0.020833333	0.286227367	0.265394033	0.034626212
3h	ACA1_219440/clean/scf7180000084758:734913-735017:-	ACA1_219440	Helicase associated domain containing protein [Source:UniProtKB/TrEMBL;Acc:L8GPX8]	0.042592267	0.307951333	0.265359067	0.011160166
3h	ACA1_264200/clean/scf7180000084770:209712-209791:-	ACA1_264200	hypothetical protein	0.004952177	0.2702706	0.265318423	7.89E-05
3h	ACA1_325480/clean/scf7180000084495:17775-17866:+	ACA1_325480	RRM domain-containing protein [Source:UniProtKB/TrEMBL;Acc:L8HC56]	0.068849933	0.334159033	0.2653091	0.005010211
3h	ACA1_052190/clean/scf7180000084594:5216-5307:-	ACA1_052190	G-patch domain-containing protein [Source:UniProtKB/TrEMBL;Acc:L8H7T3]	0.000159738	0.2653787	0.265218962	4.92E-07
3h	ACA1_396260/clean/scf7180000084759:66675-66813:+	ACA1_396260	hypothetical protein	0.0211723	0.286317667	0.265145367	9.87E-05
3h	ACA1_037740/clean/scf7180000084568:13444-13539:-	ACA1_037740	hypothetical protein	0.021674333	0.286701	0.265026667	0.000297053
3h	ACA1_255670/clean/scf7180000084768:542316-542425:+	ACA1_255670	Aminotran_1_2 domain-containing protein [Source:UniProtKB/TrEMBL;Acc:L8HDH4]	0.004856913	0.2698756	0.265018687	1.15E-08
3h	ACA1_173860/clean/scf7180000084741:534951-535166:-	ACA1_173860	Mob4B protein isoform 3, putative [Source:UniProtKB/TrEMBL;Acc:L8HJ60]	0.013597733	0.278555667	0.264957933	2.22E-06
3h	ACA1_064240/clean/scf7180000084599:1566300-1566368:+	ACA1_064240	NAD(P)-bd_dom domain-containing protein [Source:UniProtKB/TrEMBL;Acc:L8GXD4]	0.001426617	0.266362	0.264935383	1.73E-08
3h	ACA1_071690/clean/scf7180000084603:1029325-1029395:+	ACA1_071690	Vacuolar protein sorting factor 4, putative [Source:UniProtKB/TrEMBL;Acc:L8HE96]	0.001373357	0.2662624	0.264889043	5.17E-07
3h	ACA1_007780/clean/scf7180000083166:28143-28309:+	ACA1_007780	V-type proton ATPase subunit G [Source:UniProtKB/TrEMBL;Acc:L8H9M3]	0.00212399	0.267001033	0.264877043	2.31E-09
3h	ACA1_270690/clean/scf7180000084770:1156844-1156939:+	ACA1_270690	Lysine--tRNA ligase [Source:UniProtKB/TrEMBL;Acc:L8H4E2]	0.00183624	0.266703967	0.264867727	2.49E-08
3h	ACA1_057360/clean/scf7180000084599:123788-123875:+	ACA1_057360	Mitochondrial inner membrane protease subunit [Source:UniProtKB/TrEMBL;Acc:L8GYF2]	0.002911877	0.267768667	0.26485679	3.26E-06
3h	ACA1_151100/clean/scf7180000084716:52287-52482:-	ACA1_151100	NFACT-R_1 domain-containing protein [Source:UniProtKB/TrEMBL;Acc:L8H219]	0.00978991	0.274623767	0.264833857	0.000271692
3h	ACA1_109780/clean/scf7180000084663:25393-25562:-	ACA1_109780	AAA domain-containing protein [Source:UniProtKB/TrEMBL;Acc:L8HIF9]	0.006047493	0.27087	0.264822507	5.05E-07
3h	ACA1_322580/clean/scf7180000084466:23648-23757:+	ACA1_322580	Calmodulin, putative [Source:UniProtKB/TrEMBL;Acc:L8HAA1]	0.00654424	0.271307667	0.264763427	5.33E-06
3h	ACA1_112170/clean/scf7180000084669:136236-136339:+	ACA1_112170	PPM-type phosphatase domain-containing protein [Source:UniProtKB/TrEMBL;Acc:L8H322]	0.013854567	0.278427833	0.264573267	6.41E-05
3h	ACA1_157300/clean/scf7180000084724:35095-35178:+	ACA1_157300	TOPEUc domain-containing protein [Source:UniProtKB/TrEMBL;Acc:L8HB99]	0.0368924	0.301444333	0.264551933	0.000247687
3h	ACA1_279360/clean/scf7180000084776:371531-371613:-	ACA1_279360	hypothetical protein	0	0.264483067	0.264483067	1.74E-06
3h	ACA1_062280/clean/scf7180000084599:1156358-1156428:-	ACA1_062280	hypothetical protein	0.0388076	0.303052033	0.264244433	0.001973616
3h	ACA1_058840/clean/scf7180000084599:392653-392745:-	ACA1_058840	HATPase_c domain-containing protein [Source:UniProtKB/TrEMBL;Acc:L8GXB7]	0.002643309	0.266791367	0.264148057	1.27E-09
3h	ACA1_091440/clean/scf7180000084645:124093-124166:+	ACA1_091440	hypothetical protein	0	0.264140333	0.264140333	0.037701165
3h	ACA1_064190/clean/scf7180000084599:1552554-1552640:+	ACA1_064190	hypothetical protein	0.000373123	0.264376067	0.264002943	1.57E-10
3h	ACA1_365840/clean/scf7180000084641:334667-334744:-	ACA1_365840	hypothetical protein	0	0.264002067	0.264002067	0.003114363
3h	ACA1_112100/clean/scf7180000084669:109016-109109:+	ACA1_112100	BTB/POZ domain containing protein [Source:UniProtKB/TrEMBL;Acc:L8H3Y0]	0.000455747	0.264341233	0.263885487	8.72E-05
3h	ACA1_155500/clean/scf7180000084722:176554-176676:-	ACA1_155500	unspecified product	0.013669767	0.277523333	0.263853567	0.000238663
3h	ACA1_073000/clean/scf7180000084603:1352736-1352812:+	ACA1_073000	Mis12 domain containing protein [Source:UniProtKB/TrEMBL;Acc:L8HGW7]	0.03893	0.302776333	0.263846333	0.002003195
3h	ACA1_187340/clean/scf7180000084747:38859-38997:-	ACA1_187340	MFS domain-containing protein [Source:UniProtKB/TrEMBL;Acc:L8GTA9]	0.0108867	0.274666167	0.263779467	0.001244659
3h	ACA1_275630/clean/scf7180000084774:68332-68411:-	ACA1_275630	unspecified product	0.000678712	0.26442	0.263741288	1.77E-07
3h	ACA1_220760/clean/scf7180000084758:962387-962458:-	ACA1_220760	Methyltransf_11 domain-containing protein [Source:UniProtKB/TrEMBL;Acc:L8GQP4]	0.00189595	0.265628	0.26373205	9.52E-10
3h	ACA1_339740/clean/scf7180000084619:15747-15838:+	ACA1_339740	Serine/threonine protein kinase [Source:UniProtKB/TrEMBL;Acc:L8GLZ6]	0	0.263677267	0.263677267	1.81E-06
3h	ACA1_276370/clean/scf7180000084774:157610-157783:-	ACA1_276370	unspecified product	0.005045987	0.2687141	0.263668113	3.72E-06
3h	ACA1_377320/clean/scf7180000084711:27201-27316:+	ACA1_377320	SPRY domain containing protein [Source:UniProtKB/TrEMBL;Acc:L8GSS3]	0.01284784	0.276508067	0.263660227	4.37E-06
3h	ACA1_116140/clean/scf7180000084669:711352-711420:+	ACA1_116140	Dipeptidyl-peptidase family protein [Source:UniProtKB/TrEMBL;Acc:L8H5Z5]	0.004844416	0.268411833	0.263567418	2.42E-06
3h	ACA1_320950/clean/scf7180000084435:756-846:-	ACA1_320950	SET domain containing protein [Source:UniProtKB/TrEMBL;Acc:L8GPN6]	0.011035607	0.274596867	0.26356126	0.001066621
3h	ACA1_307250/clean/scf7180000083680:1413-1548:-	ACA1_307250	Mss4 protein [Source:UniProtKB/TrEMBL;Acc:L8H520]	0.127685667	0.391158333	0.263472667	0.004581454
3h	ACA1_072810/clean/scf7180000084603:1301123-1301213:+	ACA1_072810	Aha1_N domain-containing protein [Source:UniProtKB/TrEMBL;Acc:L8HG06]	0.00092491	0.264340433	0.263415523	3.30E-09
3h	ACA1_157980/clean/scf7180000084724:170889-171008:+	ACA1_157980	Protein kinase domain containing protein [Source:UniProtKB/TrEMBL;Acc:L8HA37]	0.010342953	0.273720067	0.263377113	8.88E-07
3h	ACA1_074160/clean/scf7180000084605:40443-40519:+	ACA1_074160	Aamy domain-containing protein [Source:UniProtKB/TrEMBL;Acc:L8HJ75]	0.012532887	0.275876667	0.26334378	7.64E-07
3h	ACA1_048540/clean/scf7180000084582:141505-141676:+	ACA1_048540	hypothetical protein	0.0620735	0.325389667	0.263316167	0.016191382
3h	ACA1_111060/clean/scf7180000084666:20960-21046:-	ACA1_111060	Xtp3transactivated protein, putative [Source:UniProtKB/TrEMBL;Acc:L8H6W1]	0.006460153	0.269693233	0.26323308	4.44E-06
3h	ACA1_366690/clean/scf7180000084641:480892-480962:+	ACA1_366690	Aspartyl aminopeptidase [Source:UniProtKB/TrEMBL;Acc:L8GM39]	0.002419301	0.265534333	0.263115032	1.24E-07
3h	ACA1_270690/clean/scf7180000084770:1155778-1155852:+	ACA1_270690	Lysine--tRNA ligase [Source:UniProtKB/TrEMBL;Acc:L8H4E2]	0.00074291	0.263735633	0.262992723	4.40E-08
3h	ACA1_265500/clean/scf7180000084770:495735-495826:-	ACA1_265500	F-box domain-containing protein [Source:UniProtKB/TrEMBL;Acc:L8H275]	0	0.262854333	0.262854333	0.000193579
3h	ACA1_264200/clean/scf7180000084770:209942-210022:-	ACA1_264200	hypothetical protein	0.001611383	0.264419167	0.262807783	2.08E-06
3h	ACA1_175380/clean/scf7180000084741:941249-941313:-	ACA1_175380	Adenylosuccinate synthetase [Source:UniProtKB/TrEMBL;Acc:L8HJJ6]	0.023904367	0.286688333	0.262783967	4.06E-07
3h	ACA1_264220/clean/scf7180000084770:212956-213018:+	ACA1_264220	hypothetical protein	0.01124766	0.2739764	0.26272874	4.61E-06
3h	ACA1_174460/clean/scf7180000084741:712418-712531:+	ACA1_174460	RING-type domain-containing protein [Source:UniProtKB/TrEMBL;Acc:L8HHW8]	0.009860677	0.272523833	0.262663157	4.35E-06
3h	ACA1_092610/clean/scf7180000084645:366774-366854:+	ACA1_092610	EF hand domain containing protein [Source:UniProtKB/TrEMBL;Acc:L8GIZ7]	0	0.262607433	0.262607433	7.97E-08
3h	ACA1_072890/clean/scf7180000084603:1324753-1324847:+	ACA1_072890	F-box domain-containing protein [Source:UniProtKB/TrEMBL;Acc:L8HES6]	0	0.26257	0.26257	0.019968959
3h	ACA1_362650/clean/scf7180000084624:123843-123955:+	ACA1_362650	G-patch domain-containing protein [Source:UniProtKB/TrEMBL;Acc:L8GFY2]	0.00204499	0.264567	0.26252201	1.32E-05
3h	ACA1_198700/clean/scf7180000084753:155062-155148:-	ACA1_198700	ABC transporter domain-containing protein [Source:UniProtKB/TrEMBL;Acc:L8H2D9]	0.002918229	0.265439	0.262520771	1.72E-07
3h	ACA1_379130/clean/scf7180000084711:282329-282488:-	ACA1_379130	hypothetical protein	0.047271663	0.309749333	0.26247767	0.021987462
3h	ACA1_227800/clean/scf7180000084761:139645-139715:+	ACA1_227800	hypothetical protein	0.009188063	0.271574333	0.26238627	1.78E-06
3h	ACA1_214610/clean/scf7180000084758:233992-234139:-	ACA1_214610	Aminotran_1_2 domain-containing protein [Source:UniProtKB/TrEMBL;Acc:L8GQ78]	0	0.2622622	0.2622622	4.20E-05
3h	ACA1_218600/clean/scf7180000084758:675968-676073:-	ACA1_218600	Eukaryotic translation initiation factor 3 subunit A [Source:UniProtKB/TrEMBL;Acc:L8GSV9]	0.000641413	0.2628153	0.262173887	6.33E-07
3h	ACA1_190110/clean/scf7180000084748:128905-128973:-	ACA1_190110	ABC transporter domain-containing protein [Source:UniProtKB/TrEMBL;Acc:L8GEM3]	0.004604737	0.266682433	0.262077697	6.09E-07
3h	ACA1_272530/clean/scf7180000084772:55415-55510:+	ACA1_272530	VWFA domain-containing protein [Source:UniProtKB/TrEMBL;Acc:L8HIL5]	0	0.2620352	0.2620352	2.62E-06
3h	ACA1_390790/clean/scf7180000084740:92894-92970:-	ACA1_390790	PKD domain containing protein [Source:UniProtKB/TrEMBL;Acc:L8GPB6]	0.013172757	0.275079	0.261906243	4.74E-07
3h	ACA1_072660/clean/scf7180000084603:1245829-1245895:+	ACA1_072660	hypothetical protein	0	0.261883	0.261883	0.043288652
3h	ACA1_054970/clean/scf7180000084594:615135-615226:-	ACA1_054970	FYVE-type domain-containing protein [Source:UniProtKB/TrEMBL;Acc:L8H8G2]	0.009846917	0.271689	0.261842083	0.00061183
3h	ACA1_169220/clean/scf7180000084739:31631-31698:+	ACA1_169220	ANK_REP_REGION domain-containing protein [Source:UniProtKB/TrEMBL;Acc:L8HFG6]	0.005855242	0.267650333	0.261795091	5.73E-07
3h	ACA1_097540/clean/scf7180000084645:1277523-1277601:-	ACA1_097540	Serine/threonine kinase [Source:UniProtKB/TrEMBL;Acc:L8GK27]	0	0.2617282	0.2617282	2.65E-06
3h	ACA1_325260/clean/scf7180000084475:38988-39065:+	ACA1_325260	Peptidylprolyl isomerase [Source:UniProtKB/TrEMBL;Acc:L8GPH9]	0.000175659	0.2618052	0.261629541	2.47E-09
3h	ACA1_054090/clean/scf7180000084594:399508-399597:-	ACA1_054090	Fbox domain containing protein [Source:UniProtKB/TrEMBL;Acc:L8H592]	0.00904087	0.270537767	0.261496897	0.004287217
3h	ACA1_066040/clean/scf7180000084599:1979837-1979911:-	ACA1_066040	hypothetical protein	0.019437503	0.280762433	0.26132493	0.005151427
3h	ACA1_265460/clean/scf7180000084770:485167-485239:-	ACA1_265460	Dolichyl-diphosphooligosaccharide--protein glycosyltransferase subunit 2 [Source:UniProtKB/TrEMBL;Acc:L8H4E5]	0.000116447	0.2614007	0.261284253	2.44E-10
3h	ACA1_158550/clean/scf7180000084724:248957-249036:+	ACA1_158550	Enoyl-CoA hydratase, putative [Source:UniProtKB/TrEMBL;Acc:L8HA73]	0.003968267	0.2651941	0.261225833	1.32E-05
3h	ACA1_189340/clean/scf7180000084748:10701-10788:-	ACA1_189340	Dyptype peroxidase superfamily protein [Source:UniProtKB/TrEMBL;Acc:L8GDU2]	0.004175993	0.2653723	0.261196307	1.52E-08
3h	ACA1_068040/clean/scf7180000084603:85203-85282:+	ACA1_068040	START domain-containing protein [Source:UniProtKB/TrEMBL;Acc:L8HDG1]	0.00062893	0.261820767	0.261191837	1.67E-05
3h	ACA1_063390/clean/scf7180000084599:1399113-1399332:+	ACA1_063390	Ribosomal_L7Ae domain-containing protein [Source:UniProtKB/TrEMBL;Acc:L8GX53]	0.021913	0.282966	0.261053	6.60E-08
3h	ACA1_053890/clean/scf7180000084594:344928-344996:-	ACA1_053890	Aconitate hydratase, mitochondrial [Source:UniProtKB/TrEMBL;Acc:L8H690]	0.000490917	0.261516333	0.261025417	2.23E-08
3h	ACA1_153600/clean/scf7180000084720:135599-135959:-	ACA1_153600	tRNA pseudouridine synthase [Source:UniProtKB/TrEMBL;Acc:L8HG78]	0.0259267	0.286943267	0.261016567	0.003902565
3h	ACA1_255510/clean/scf7180000084768:519043-519130:-	ACA1_255510	Ephrin_rec_like domain-containing protein [Source:UniProtKB/TrEMBL;Acc:L8HDG9]	0.010153267	0.271079333	0.260926067	1.34E-07
3h	ACA1_189640/clean/scf7180000084748:70855-70931:-	ACA1_189640	Bifunctional aspartate kinase/diaminopimelate decarboxylase protein [Source:UniProtKB/TrEMBL;Acc:L8GEY3]	0.002916815	0.263838733	0.260921919	1.23E-06
3h	ACA1_095260/clean/scf7180000084645:824466-824551:-	ACA1_095260	F-box domain-containing protein [Source:UniProtKB/TrEMBL;Acc:L8GL23]	0.002258497	0.263018333	0.260759837	9.13E-06
3h	ACA1_244370/clean/scf7180000084763:389199-389333:-	ACA1_244370	Protein kinase domain-containing protein [Source:UniProtKB/TrEMBL;Acc:L8GK51]	0.014545753	0.2752277	0.260681947	0.001244509
3h	ACA1_063810/clean/scf7180000084599:1510224-1510314:+	ACA1_063810	DHHC zinc finger domain containing protein [Source:UniProtKB/TrEMBL;Acc:L8GXG4]	0.027747933	0.288397667	0.260649733	0.000658628
3h	ACA1_171380/clean/scf7180000084741:89891-90018:+	ACA1_171380	COMM domain-containing protein [Source:UniProtKB/TrEMBL;Acc:L8HGA6]	0.014848603	0.275396333	0.26054773	1.45E-05
3h	ACA1_114450/clean/scf7180000084669:473707-473816:-	ACA1_114450	SH3 domain containing protein [Source:UniProtKB/TrEMBL;Acc:L8H6I9]	0.00834789	0.268850733	0.260502843	8.54E-08
3h	ACA1_037480/clean/scf7180000084567:98148-98253:-	ACA1_037480	AB hydrolase-1 domain-containing protein [Source:UniProtKB/TrEMBL;Acc:L8H038]	0.054400567	0.314816667	0.2604161	0.009492133
3h	ACA1_002450/clean/scf7180000082931:3577-3659:+	ACA1_002450	eIF-5a domain-containing protein [Source:UniProtKB/TrEMBL;Acc:L8GWN9]	0.037987867	0.298355967	0.2603681	0.008428821
3h	ACA1_074420/clean/scf7180000084608:5682-5779:-	ACA1_074420	Putative N6adenosine-methyltransferase [Source:UniProtKB/TrEMBL;Acc:L8HFC7]	0.00772488	0.268077933	0.260353053	2.98E-06
3h	ACA1_323140/clean/scf7180000084467:14835-15055:-	ACA1_323140	RFX-type winged-helix domain-containing protein [Source:UniProtKB/TrEMBL;Acc:L8GEG4]	0.019171367	0.279428	0.260256633	0.006419373
3h	ACA1_136150/clean/scf7180000084702:140023-140118:-	ACA1_136150	MPN domain-containing protein [Source:UniProtKB/TrEMBL;Acc:L8GEU6]	0.000534727	0.260758667	0.26022394	3.93E-08
3h	ACA1_189360/clean/scf7180000084748:17319-17409:-	ACA1_189360	Proline dehydrogenase [Source:UniProtKB/TrEMBL;Acc:L8GGF7]	0	0.2601785	0.2601785	0.04328369
3h	ACA1_376270/clean/scf7180000084685:68267-68345:+	ACA1_376270	Phospho-2-dehydro-3-deoxyheptonate aldolase [Source:UniProtKB/TrEMBL;Acc:L8HFZ7]	0.018828967	0.27894	0.260111033	0.000126
3h	ACA1_203070/clean/scf7180000084754:135151-135260:+	ACA1_203070	Fbox domain containing protein [Source:UniProtKB/TrEMBL;Acc:L8GUJ5]	0.042230633	0.302302	0.260071367	0.000342092
3h	ACA1_043200/clean/scf7180000084573:137786-137868:-	ACA1_043200	ACB domain-containing protein [Source:UniProtKB/TrEMBL;Acc:L8GUP2]	0.003910827	0.2639598	0.260048973	4.80E-07

3h	ACA1_114530/clean/scf7180000084669:501551-501674:+	ACA1_114530	NB-ARC domain containing protein [Source:UniProtKB/TrEMBL;Acc:L8H3K3]	0.023327992	0.2833356	0.260007608	0.000576106
3h	ACA1_320420/clean/scf7180000084352:15910-15979:-	ACA1_320420	hypothetical protein	0	0.259977667	0.259977667	5.06E-06
3h	ACA1_061160/clean/scf7180000084599:843935-844023:-	ACA1_061160	WD_REPEATS_REGION domain-containing protein [Source:UniProtKB/TrEMBL;Acc:L8GXU0]	0.002012073	0.261974367	0.259962293	2.74E-05
3h	ACA1_287960/clean/scf7180000084777:132896-132974:-	ACA1_287960	SET domain-containing protein [Source:UniProtKB/TrEMBL;Acc:L8HIS3]	0.0196248	0.279558267	0.259933467	0.014598585
3h	ACA1_092000/clean/scf7180000084645:244151-244261:-	ACA1_092000	hypothetical protein	0.014811567	0.274590367	0.2597788	0.000104469
3h	ACA1_211790/clean/scf7180000084758:114935-115029:+	ACA1_211790	RhoGEF domain containing protein [Source:UniProtKB/TrEMBL;Acc:L8GQS6]	0.0645149	0.324154667	0.259639767	0.003905818
3h	ACA1_384860/clean/scf7180000084730:117596-117695:+	ACA1_384860	Isochorismatase domain-containing protein [Source:UniProtKB/TrEMBL;Acc:L8H995]	0.00512778	0.2647346	0.25960682	1.03E-07
3h	ACA1_065210/clean/scf7180000084599:1816441-1816517:+	ACA1_065210	3-hydroxyisobutyryl-CoA hydrolase, mitochondrial [Source:UniProtKB/TrEMBL;Acc:L8GXR1]	0.0035752	0.263120333	0.259545133	5.50E-06
3h	ACA1_153680/clean/scf7180000084720:154388-154474:-	ACA1_153680	Amz2 protein [Source:UniProtKB/TrEMBL;Acc:L8HHH0]	0	0.2593537	0.2593537	0.03050747
3h	ACA1_064550/clean/scf7180000084599:1639689-1639779:-	ACA1_064550	60S ribosomal export protein NMD3 [Source:UniProtKB/TrEMBL;Acc:L8GYR2]	0.01937116	0.278674433	0.259303273	6.30E-05
3h	ACA1_279740/clean/scf7180000084776:433856-433934:+	ACA1_279740	BATS domain-containing protein [Source:UniProtKB/TrEMBL;Acc:L8H6X4]	0.01034859	0.269636833	0.259288243	0.000698767
3h	ACA1_107160/clean/scf7180000084660:158766-158894:+	ACA1_107160	hypothetical protein	0.000141183	0.259314833	0.25917365	2.70E-07
3h	ACA1_089960/clean/scf7180000084639:403482-403556:-	ACA1_089960	Dual specificity protein kinase [Source:UniProtKB/TrEMBL;Acc:L8GVS6]	0.00635934	0.2655038	0.25914446	2.96E-05
3h	ACA1_091940/clean/scf7180000084645:232853-232913:-	ACA1_091940	Metallopeptidase [Source:UniProtKB/TrEMBL;Acc:L8GIL4]	0.036792567	0.295901667	0.2591091	0.000191579
3h	ACA1_040840/clean/scf7180000084572:92717-92813:-	ACA1_040840	BRO1 domain-containing protein [Source:UniProtKB/TrEMBL;Acc:L8H1X9]	0	0.259000467	0.259000467	1.46E-07
3h	ACA1_237790/clean/scf7180000084763:18597-18760:+	ACA1_237790	Protein kinase domain containing protein [Source:UniProtKB/TrEMBL;Acc:L8GM38]	0.001305076	0.260056	0.258750924	1.81E-07
3h	ACA1_382350/clean/scf7180000084721:36494-36557:-	ACA1_382350	RFX-type winged-helix domain-containing protein [Source:UniProtKB/TrEMBL;Acc:L8GXF7]	0.010620067	0.269228367	0.2586083	0.008675561
3h	ACA1_380620/clean/scf7180000084712:64959-65030:+	ACA1_380620	PRK domain-containing protein [Source:UniProtKB/TrEMBL;Acc:L8GQI5]	0.00746572	0.2660205	0.25855478	3.24E-06
3h	ACA1_045390/clean/scf7180000084576:129813-129904:+	ACA1_045390	Protein kinase domain-containing protein [Source:UniProtKB/TrEMBL;Acc:L8GZV5]	0	0.258475133	0.258475133	0.000737638
3h	ACA1_158180/clean/scf7180000084724:206904-207096:+	ACA1_158180	eIF2A domain-containing protein [Source:UniProtKB/TrEMBL;Acc:L8H989]	0.025451	0.283904667	0.258453667	2.44E-05
3h	ACA1_042250/clean/scf7180000084573:69303-69401:-	ACA1_042250	Phosphoglucomutase, putative [Source:UniProtKB/TrEMBL;Acc:L8GV41]	0.021284743	0.279527333	0.25824259	5.36E-05
3h	ACA1_333170/clean/scf7180000084581:10463-10555:-	ACA1_333170	Ras-GEF domain-containing protein [Source:UniProtKB/TrEMBL;Acc:L8HGS6]	0	0.258153633	0.258153633	0.028609437
3h	ACA1_255460/clean/scf7180000084768:506629-506706:-	ACA1_255460	Diacylglycerol acyltransferase protein [Source:UniProtKB/TrEMBL;Acc:L8HB03]	0.0344351	0.292549667	0.258114567	0.000122734
3h	ACA1_108200/clean/scf7180000084661:11050-11111:-	ACA1_108200	hypothetical protein	0	0.257961	0.257961	0.002242518
3h	ACA1_062570/clean/scf7180000084599:1236015-1236088:-	ACA1_062570	Zinc finger, c2h2 type domain containing protein [Source:UniProtKB/TrEMBL;Acc:L8GY95]	0.003615754	0.261555567	0.257939813	2.62E-05
3h	ACA1_035310/clean/scf7180000084564:19335-19433:+	ACA1_035310	AMP-binding domain-containing protein [Source:UniProtKB/TrEMBL;Acc:L8HCU7]	0.0130647	0.270889467	0.257824767	0.001454231
3h	ACA1_319110/clean/scf7180000084239:16035-16129:+	ACA1_319110	KOG2701 domain-containing protein [Source:UniProtKB/TrEMBL;Acc:L8GID5]	0.00188677	0.259632733	0.257745963	1.50E-06
3h	ACA1_246770/clean/scf7180000084763:579475-579593:+	ACA1_246770	Metallophos domain-containing protein [Source:UniProtKB/TrEMBL;Acc:L8GKY3]	0.008825343	0.266536533	0.25771119	1.39E-05
3h	ACA1_220200/clean/scf7180000084758:895970-896047:+	ACA1_220200	Aminopeptidase [Source:UniProtKB/TrEMBL;Acc:L8GT26]	0.001106231	0.258785633	0.257679402	4.21E-08
3h	ACA1_238390/clean/scf7180000084763:81429-81529:-	ACA1_238390	Coronin [Source:UniProtKB/TrEMBL;Acc:L8GJQ2]	0.011507783	0.2690665	0.257558717	3.13E-06
3h	ACA1_192010/clean/scf7180000084749:73702-73983:+	ACA1_192010	PCI domain-containing protein [Source:UniProtKB/TrEMBL;Acc:L8GQR2]	0.003519423	0.261016667	0.257497243	4.23E-08
3h	ACA1_074160/clean/scf7180000084605:40254-40321:+	ACA1_074160	Aamy domain-containing protein [Source:UniProtKB/TrEMBL;Acc:L8HJ75]	0.01065127	0.267976333	0.257325063	1.08E-06
3h	ACA1_237860/clean/scf7180000084763:32780-32877:+	ACA1_237860	Glycerophosphodiester phosphodiesterase family protein [Source:UniProtKB/TrEMBL;Acc:L8GKN2]	0.005150867	0.2624454	0.257294533	4.31E-06
3h	ACA1_399960/clean/scf7180000084775:82715-82790:-	ACA1_399960	WD_REPEATS_REGION domain-containing protein [Source:UniProtKB/TrEMBL;Acc:L8GID8]	0	0.257282767	0.257282767	1.29E-06
3h	ACA1_325470/clean/scf7180000084495:15007-15185:-	ACA1_325470	BTB domain-containing protein [Source:UniProtKB/TrEMBL;Acc:M0QSM7]	0.063703733	0.320962	0.257258267	0.002524629
3h	ACA1_310740/clean/scf7180000083780:2700-2787:-	ACA1_310740	COesterase domain-containing protein [Source:UniProtKB/TrEMBL;Acc:L8GTB6]	0.000160256	0.257208667	0.25704841	0.000252401
3h	ACA1_239940/clean/scf7180000084763:186976-187062:+	ACA1_239940	Dolichol phosphate-mannose biosynthesis regulatory protein [Source:UniProtKB/TrEMBL;Acc:L8GJY5]	0.041585967	0.298575167	0.2569892	0.006151515
3h	ACA1_277720/clean/scf7180000084776:87876-87947:+	ACA1_277720	CBS domain containing protein [Source:UniProtKB/TrEMBL;Acc:L8H7X6]	0.000778817	0.257730633	0.256951817	0.000224718
3h	ACA1_285540/clean/scf7180000084776:993245-993335:-	ACA1_285540	GPS domain-containing protein [Source:UniProtKB/TrEMBL;Acc:L8H7T4]	0.0033648	0.2601768	0.256812	0.000170401
3h	ACA1_013340/clean/scf7180000083750:2258-2321:+	ACA1_013340	PH domain-containing protein [Source:UniProtKB/TrEMBL;Acc:L8HGJ7]	0.0205058	0.2772944	0.2567886	1.87E-05
3h	ACA1_208460/clean/scf7180000084757:197296-197367:-	ACA1_208460	Abhydro_lipase domain-containing protein [Source:UniProtKB/TrEMBL;Acc:L8GZL5]	0.0537075	0.310489	0.2567815	0.006579797
3h	ACA1_266770/clean/scf7180000084770:708924-709051:+	ACA1_266770	Fe2OG dioxygenase domain-containing protein [Source:UniProtKB/TrEMBL;Acc:L8H2F4]	0.021717933	0.2784844	0.256766467	3.51E-05
3h	ACA1_306840/clean/scf7180000083668:18559-18693:+	ACA1_306840	RTP1_C1 domain-containing protein [Source:UniProtKB/TrEMBL;Acc:L8GQF6]	0.003984067	0.260708633	0.256724567	0.001279753
3h	ACA1_170940/clean/scf7180000084741:19010-19079:+	ACA1_170940	Proteasome subunit alpha type [Source:UniProtKB/TrEMBL;Acc:L8HH61]	0.002018166	0.2587193	0.256701134	1.84E-10
3h	ACA1_062150/clean/scf7180000084599:1129701-1129814:-	ACA1_062150	Nacylsphingosine amidohydrolase, putative [Source:UniProtKB/TrEMBL;Acc:L8GY45]	0.001049509	0.257670867	0.256621357	1.02E-07
3h	ACA1_072330/clean/scf7180000084603:1200199-1200290:-	ACA1_072330	UBA_e1_C domain-containing protein [Source:UniProtKB/TrEMBL;Acc:L8HGS4]	0.00442968	0.261047167	0.256617487	6.76E-07
3h	ACA1_358610/clean/scf7180000084742:12864-12929:-	ACA1_358610	hypothetical protein	0.1572081	0.413651333	0.256443233	0.017970051
3h	ACA1_064290/clean/scf7180000084599:1576110-1576188:-	ACA1_064290	hypothetical protein	0.01373324	0.270167667	0.256434427	5.80E-05
3h	ACA1_313860/clean/scf7180000083835:2108-2202:-	ACA1_313860	STAS domain-containing protein [Source:UniProtKB/TrEMBL;Acc:L8H2N6]	0.011553063	0.267939	0.256385937	5.38E-07
3h	ACA1_040440/clean/scf7180000084572:2015-2089:-	ACA1_040440	hypothetical protein	0.01468817	0.271039	0.25635083	4.59E-06
3h	ACA1_389440/clean/scf7180000084733:254660-254802:+	ACA1_389440	Proteasome subunit alpha type [Source:UniProtKB/TrEMBL;Acc:L8GES1]	0.00289775	0.259145433	0.256247683	6.41E-10
3h	ACA1_278980/clean/scf7180000084776:289721-289823:-	ACA1_278980	Extracellular ligandbinding receptor, putative [Source:UniProtKB/TrEMBL;Acc:L8H6Q3]	0.017353247	0.273493833	0.256140586	0.005847016
3h	ACA1_338180/clean/scf7180000084604:49039-49122:-	ACA1_338180	hypothetical protein	0.003348567	0.259444667	0.2560961	7.63E-09
3h	ACA1_132360/clean/scf7180000084700:19749-19812:-	ACA1_132360	Glutamine-tRNA ligase [Source:UniProtKB/TrEMBL;Acc:L8GWR8]	0.001960783	0.258	0.256039217	1.11E-06
3h	ACA1_400300/clean/scf7180000084775:144005-144087:+	ACA1_400300	UBIQUITIN_CONJUGAT_2 domain-containing protein [Source:UniProtKB/TrEMBL;Acc:L8GGI3]	0	0.255954467	0.255954467	0.000594874
3h	ACA1_041920/clean/scf7180000084573:20846-20916:+	ACA1_041920	Deoxyribonuclease II, putative [Source:UniProtKB/TrEMBL;Acc:L8GV23]	0.00117371	0.2570734	0.25589969	1.27E-06
3h	ACA1_287920/clean/scf7180000084777:125694-125783:-	ACA1_287920	Leucinerich repeat protein lrrA, putative [Source:UniProtKB/TrEMBL;Acc:L8HL59]	0.011648553	0.267548233	0.25589968	2.90E-06
3h	ACA1_181830/clean/scf7180000084745:53503-53577:-	ACA1_181830	PIPK domain-containing protein [Source:UniProtKB/TrEMBL;Acc:L8H972]	0.0307549	0.286629	0.2558741	0.007851356
3h	ACA1_313740/clean/scf7180000083824:6465-6557:-	ACA1_313740	F-box domain-containing protein [Source:UniProtKB/TrEMBL;Acc:L8GMG9]	0.002139007	0.257775867	0.25563686	2.90E-06
3h	ACA1_309490/clean/scf7180000083762:6403-6531:+	ACA1_309490	Clat_adaptor_s domain-containing protein [Source:UniProtKB/TrEMBL;Acc:L8GIC8]	0.003428247	0.258745067	0.25531682	1.80E-09
3h	ACA1_143940/clean/scf7180000084709:112194-112289:-	ACA1_143940	VWFA domain-containing protein [Source:UniProtKB/TrEMBL;Acc:L8HEQ6]	0	0.2552791	0.2552791	2.52E-07
3h	ACA1_121830/clean/scf7180000084682:105394-105524:-	ACA1_121830	COesterase domain-containing protein [Source:UniProtKB/TrEMBL;Acc:L8GEL0]	0.002983642	0.258253333	0.255269691	3.50E-05
3h	ACA1_065710/clean/scf7180000084599:1877483-1877564:+	ACA1_065710	GATA-type domain-containing protein [Source:UniProtKB/TrEMBL;Acc:L8GXT1]	0.0794813	0.334672	0.2551907	0.002526185
3h	ACA1_330410/clean/scf7180000084566:54038-54138:+	ACA1_330410	hypothetical protein	0.01303527	0.268046667	0.255011397	8.61E-06
3h	ACA1_221050/clean/scf7180000084758:986157-986242:-	ACA1_221050	hypothetical protein	0.017094033	0.2720978	0.255003767	0.004157941
3h	ACA1_250800/clean/scf7180000084768:30219-30292:+	ACA1_250800	hypothetical protein	0.017351167	0.272255333	0.254904167	1.30E-05
3h	ACA1_066040/clean/scf7180000084599:1979612-1979700:-	ACA1_066040	hypothetical protein	0.024601533	0.279206667	0.254605133	0.038197167
3h	ACA1_253380/clean/scf7180000084768:289028-289095:+	ACA1_253380	EF hand domain containing protein [Source:UniProtKB/TrEMBL;Acc:L8HAR9]	0.01019749	0.264758333	0.254560843	0.001658148
3h	ACA1_093030/clean/scf7180000084645:464755-464851:+	ACA1_093030	G domain-containing protein [Source:UniProtKB/TrEMBL;Acc:L8GJ37]	0.007237797	0.261714167	0.25447637	0.000130229
3h	ACA1_158180/clean/scf7180000084724:204624-204933:+	ACA1_158180	eIF2A domain-containing protein [Source:UniProtKB/TrEMBL;Acc:L8H989]	0.016954233	0.271331667	0.254377433	2.07E-05
3h	ACA1_378170/clean/scf7180000084711:200905-200989:-	ACA1_378170	G_PROTEIN_RECEP_F1_2 domain-containing protein [Source:UniProtKB/TrEMBL;Acc:L8GRC2]	0.001738897	0.2560691	0.254330203	4.76E-06
3h	ACA1_054220/clean/scf7180000084594:433735-433820:-	ACA1_054220	MHCK/EF2 kinase domain containing protein [Source:UniProtKB/TrEMBL;Acc:L8H897]	0.053805967	0.3080877	0.254281733	0.002373863
3h	ACA1_210400/clean/scf7180000084758:39205-39300:-	ACA1_210400	PPM-type phosphatase domain-containing protein [Source:UniProtKB/TrEMBL;Acc:L8GQQ0]	0.007684227	0.261958	0.254273773	1.71E-07
3h	ACA1_236180/clean/scf7180000084762:426748-426844:-	ACA1_236180	hypothetical protein	0.022635883	0.276756833	0.25412095	0.000204693
3h	ACA1_287570/clean/scf7180000084777:38251-38327:+	ACA1_287570	hypothetical protein	0.008670693	0.262595733	0.25392504	1.97E-07
3h	ACA1_024390/clean/scf7180000084442:11270-11360:+	ACA1_024390	hypothetical protein	0.001809277	0.255673967	0.25386469	4.40E-06
3h	ACA1_367910/clean/scf7180000084664:12702-12797:+	ACA1_367910	MFS domain-containing protein [Source:UniProtKB/TrEMBL;Acc:L8H174]	0.00063423	0.254418	0.25378377	7.35E-08
3h	ACA1_237280/clean/scf7180000084762:501482-501559:-	ACA1_237280	2OG-FeII_Oxy_2 domain-containing protein [Source:UniProtKB/TrEMBL;Acc:L8H1I9]	0.456643	0.710344667	0.253701667	0.039606908
3h	ACA1_040770/clean/scf7180000084572:78760-78843:-	ACA1_040770	Helicase Cterminal domain containing protein [Source:UniProtKB/TrEMBL;Acc:L8H2X4]	0.007813133	0.2615142	0.253701067	0.000363214
3h	ACA1_089040/clean/scf7180000084639:304638-304731:+	ACA1_089040	UBA/TS-N domain containing protein [Source:UniProtKB/TrEMBL;Acc:L8GUR2]	0.024452167	0.278149333	0.253697167	2.97E-05
3h	ACA1_094910/clean/scf7180000084645:787544-787604:-	ACA1_094910	Deoxyhypusine synthase [Source:UniProtKB/TrEMBL;Acc:L8GL08]	0	0.253572	0.253572	8.93E-07
3h	ACA1_236660/clean/scf7180000084762:471093-471163:-	ACA1_236660	Fbox domain containing protein [Source:UniProtKB/TrEMBL;Acc:L8H304]	0.005649733	0.2591078	0.253458067	0.000500705
3h	ACA1_095670/clean/scf7180000084645:876126-876212:-	ACA1_095670	FAD-binding PCMH-type domain-containing protein [Source:UniProtKB/TrEMBL;Acc:L8GIT7]	0.005340233	0.258736367	0.253396133	1.91E-05
3h	ACA1_320780/clean/scf7180000084416:20326-20427:+	ACA1_320780	hypothetical protein	0.030913867	0.284226667	0.2533128	4.15E-05
3h	ACA1_211960/clean/scf7180000084758:123031-123087:-	ACA1_211960	hypothetical protein	0.020903033	0.274152333	0.2532493	0.010364214
3h	ACA1_174060/clean/scf7180000084741:592473-592555:+	ACA1_174060	BTB domain-containing protein [Source:UniProtKB/TrEMBL;Acc:M0QSG3]	0.1216692	0.374888667	0.253219467	0.010832143
3h	ACA1_199970/clean/scf7180000084753:361595-361666:-	ACA1_199970	Glycoside hydrolase family protein [Source:UniProtKB/TrEMBL;Acc:L8H5H5]	0.00074789	0.253836767	0.253088877	5.75E-10
3h	ACA1_172230/clean/scf7180000084741:279902-279988:+	ACA1_172230	Serine/threonine-protein phosphatase 2A activator [Source:UniProtKB/TrEMBL;Acc:L8HIY6]	0	0.2530868	0.2530868	1.05E-07
3h	ACA1_329820/clean/scf7180000084566:5717-5824:-	ACA1_329820	Transporter, major facilitator subfamily protein [Source:UniProtKB/TrEMBL;Acc:L8GHX2]	0.003921892	0.256740667	0.252818775	1.05E-07
3h	ACA1_097590/clean/scf7180000084645:1290908-1290980:-	ACA1_097590	Amine oxidase [Source:UniProtKB/TrEMBL;Acc:L8GK33]	0.007323647	0.260140967	0.25281732	7.52E-09
3h	ACA1_013390/clean/scf7180000083750:11683-11776:-	ACA1_013390	Aamy domain-containing protein [Source:UniProtKB/TrEMBL;Acc:L8HGK3]	0.00444407	0.2572308	0.25278673	3.02E-06
3h	ACA1_309340/clean/scf7180000083758:6495-6559:+	ACA1_309340	Protein tyrosine phosphataselike protein, PTPLA [Source:UniProtKB/TrEMBL;Acc:L8GR24]	0	0.252755333	0.252755333	1.08E-07
3h	ACA1_061500/clean/scf7180000084599:944369-944466:+	ACA1_061500	Ras-GAP domain-containing protein [Source:UniProtKB/TrEMBL;Acc:L8GWM5]	0.001227787	0.253964767	0.25273698	5.48E-06
3h	ACA1_310750/clean/scf7180000083780:6854-6919:+	ACA1_310750	RFX-type winged-helix domain-containing protein [Source:UniProtKB/TrEMBL;Acc:L8GVY4]	0.01335323	0.266057	0.25270377	0.000571972
3h	ACA1_210540/clean/scf7180000084758:51911-51998:-	ACA1_210540	ABC transporter, ATPbinding domain containing protein [Source:UniProtKB/TrEMBL;Acc:L8GPZ1]	0	0.252649533	0.252649533	0.003246401
3h	ACA1_269300/clean/scf7180000084770:967672-967832:-	ACA1_269300	hypothetical protein	0.01173874	0.264372	0.25263326	5.35E-07
3h	ACA1_384010/clean/scf7180000084730:3407-3484:-	ACA1_384010	Arf-GAP domain-containing protein [Source:UniProtKB/TrEMBL;Acc:L8HAG1]	0.000941953	0.253434933	0.25249298	2.67E-08
3h	ACA1_282720/clean/scf7180000084776:724864-724932:-	ACA1_282720	Leucine rich repeat domain containing protein [Source:UniProtKB/TrEMBL;Acc:L8H9L8]	0.010979667	0.263436467	0.2524568	0.003029222
3h	ACA1_384820/clean/scf7180000084730:109467-109539:+	ACA1_384820	hypothetical protein	0	0.2522412	0.2522412	0.003342114
3h	ACA1_228790/clean/scf7180000084761:214543-214629:+	ACA1_228790	IgA-specific serine endopeptidase [Source:UniProtKB/TrEMBL;Acc:L8HA86]	0.0073721	0.2595912	0.2522191	0.000127483
3h	ACA1_204500/clean/scf7180000084754:296456-296541:+	ACA1_204500	hypothetical protein	0.004198053	0.256232667	0.252034613	1.48E-06
3h	ACA1_377500/clean/scf7180000084711:77350-77475:+	ACA1_377500	Peptidase family c78 protein [Source:UniProtKB/TrEMBL;Acc:L8GU78]	0.013458433	0.265402367	0.251943933	1.51E-05
3h	ACA1_226710/clean/scf7180000084761:60232-60321:-	ACA1_226710	hypothetical protein	0.025398033	0.277301333	0.2519033	0.023419556
3h	ACA1_034080/clean/scf7180000084561:17049-17170:-	ACA1_034080	RhoGEF domain containing protein [Source:UniProtKB/TrEMBL;Acc:L8GTW8]	0.017584587	0.269432	0.251847413	0.000901052
3h	ACA1_255750/clean/scf7180000084768:560191-560344:+	ACA1_255750	hypothetical protein	0.1120705	0.363878667	0.251808167	0.010428715
3h	ACA1_041960/clean/scf7180000084573:26559-26622:+	ACA1_041960	Osmotically inducible protein C, putative [Source:UniProtKB/TrEMBL;Acc:L8GUI8]	0.0517243	0.303363667	0.251639367	0.000377236
3h	ACA1_024200/clean/scf7180000084441:13737-13849:-	ACA1_024200	WD_REPEATS_REGION domain-containing protein [Source:UniProtKB/TrEMBL;Acc:L8GKS0]	0.029274	0.280844667	0.251570667	0.000585524
3h	ACA1_142840/clean/scf7180000084708:246031-246136:-	ACA1_142840	hypothetical protein	0.0187118	0.270274633	0.251562833	0.001244659
3h	ACA1_381590/clean/scf7180000084712:187425-187513:+	ACA1_381590	PI3K/PI4K domain-containing protein [Source:UniProtKB/TrEMBL;Acc:L8GN80]	0.0045045	0.256032133	0.251527633	0.000386573
3h	ACA1_336430/clean/scf7180000084593:74159-74239:-	ACA1_336430	Cysteine protease [Source:UniProtKB/TrEMBL;Acc:L8H1P1]	0.006511913	0.257932033	0.25142012	5.16E-07
3h	ACA1_388490/clean/scf7180000084733:125549-125622:+	ACA1_388490	ANK_REP_REGION domain-containing protein [Source:UniProtKB/TrEMBL;Acc:L8GEA1]	0.02071698	0.272098467	0.251381487	0.001952478
3h	ACA1_158530/clean/scf7180000084724:243842-243956:+	ACA1_158530	Glucose-6-phosphate 1-dehydrogenase [Source:UniProtKB/TrEMBL;Acc:L8HBJ1]	0	0.251376167	0.251376167	0.000226426
3h	ACA1_024430/clean/scf7180000084442:25766-25842:+	ACA1_024430	hypothetical protein	0.0294482	0.280634233	0.251186033	0.033003483
3h	ACA1_144270/clean/scf7180000084709:189198-189348:+	ACA1_144270	SAC domain-containing protein [Source:UniProtKB/TrEMBL;Acc:L8HI07]	0.003921567	0.255053633	0.251132067	0.000176765
3h	ACA1_322090/clean/scf7180000084459:4402-4470:-	ACA1_322090	Myosin head (Motor domain) domain containing protein [Source:UniProtKB/TrEMBL;Acc:L8GN06]	0.009132433	0.2601194	0.250986967	4.98E-05
3h	ACA1_277560/clean/scf7180000084776:76332-76405:-	ACA1_277560	F-box domain-containing protein [Source:UniProtKB/TrEMBL;Acc:L8H6G6]	0.199051	0.450036333	0.250985333	0.042417008
3h	ACA1_235090/clean/scf7180000084762:335653-335758:-	ACA1_235090	Lysophospholipaselike protein [Source:UniProtKB/TrEMBL;Acc:L8H3L9]	0.002236667	0.2530509	0.250814233	2.03E-06
3h	ACA1_036910/clean/scf7180000084567:10170-10301:+	ACA1_036910	SAM domain-containing protein [Source:UniProtKB/TrEMBL;Acc:L8H0V3]	0.014650843	0.265268	0.250617157	5.16E-06
3h	ACA1_112470/clean/scf7180000084669:159387-159469:-	ACA1_112470	KH domain-containing protein [Source:UniProtKB/TrEMBL;Acc:L8H5E2]	0.0323424	0.282939233	0.250596833	0.001397885
3h	ACA1_026980/clean/scf7180000084464:7697-7824:+	ACA1_026980	hypothetical protein	0.095890533	0.346411333	0.2505208	0.008330589
3h	ACA1_285200/clean/scf7180000084776:956384-956479:-	ACA1_285200	Na_H_Exchanger domain-containing protein [Source:UniProtKB/TrEMBL;Acc:L8H9U2]	0.002960658	0.2532842	0.250323542	2.88E-06
3h	ACA1_108270/clean/scf7180000084661:26215-26314:+	ACA1_108270	Transferrin receptor family dimerization domain containing protein [Source:UniProtKB/TrEMBL;Acc:L8GC39]	0	0.2502895	0.2502895	2.42E-07
3h	ACA1_381570/clean/scf7180000084712:184324-184415:+	ACA1_381570	Ras-GAP domain-containing protein [Source:UniProtKB/TrEMBL;Acc:L8GMY5]	0.015024517	0.265303467	0.25027895	1.23E-05
3h	ACA1_046020/clean/scf7180000084577:32263-32379:-	ACA1_046020	hypothetical protein	0.025641033	0.275844467	0.250203433	0.004716899
3h	ACA1_063220/clean/scf7180000084599:1360115-1360189:+	ACA1_063220	PDZ domain-containing protein [Source:UniProtKB/TrEMBL;Acc:L8GYE7]	0.014612633	0.264742667	0.250130033	0.000351354
3h	ACA1_105960/clean/scf7180000084660:39178-39236:-	ACA1_105960	hypothetical protein	0.032552567	0.282651667	0.2500991	0.000107575
3h	ACA1_191520/clean/scf7180000084749:9601-9697:-	ACA1_191520	GDP-Man:Man(3)GlcNAc(2)-PP-Dol alpha-1,2-mannosyltransferase [Source:UniProtKB/TrEMBL;Acc:L8GNP5]	0	0.250000867	0.250000867	1.17E-05
3h	ACA1_264460/clean/scf7180000084770:294506-294622:-	ACA1_264460	GTPbinding protein [Source:UniProtKB/TrEMBL;Acc:L8H231]	0.001650773	0.2515744	0.249923627	2.44E-05
3h	ACA1_145850/clean/scf7180000084710:168655-168728:-	ACA1_145850	O-methyltransferase, putative [Source:UniProtKB/TrEMBL;Acc:L8GFG9]	0.002115413	0.252037	0.249921587	3.68E-07
3h	ACA1_280550/clean/scf7180000084776:491553-491663:-	ACA1_280550	RSNARE, VAMP71-family [Source:UniProtKB/TrEMBL;Acc:L8H6Z7]	0.029340933	0.279096167	0.249755233	0.001561485
3h	ACA1_119230/clean/scf7180000084672:46717-46802:+	ACA1_119230	Armadillo/betacatenin-like repeat domain containing protein [Source:UniProtKB/TrEMBL;Acc:L8HMP1]	0	0.249633167	0.249633167	1.91E-08
3h	ACA1_111310/clean/scf7180000084669:2486-2561:+	ACA1_111310	hypothetical protein	0.011432097	0.260935	0.249502903	0.000144649
3h	ACA1_092640/clean/scf7180000084645:373302-373378:-	ACA1_092640	Amidase domain-containing protein [Source:UniProtKB/TrEMBL;Acc:L8GKL1]	0.006652917	0.256137	0.249484083	2.83E-06
3h	ACA1_365090/clean/scf7180000084641:234437-234503:+	ACA1_365090	ADPribosylglycohydrolase superfamily protein [Source:UniProtKB/TrEMBL;Acc:L8GP97]	0.0232142	0.272635433	0.249421233	7.64E-05
3h	ACA1_234000/clean/scf7180000084762:181511-181600:+	ACA1_234000	Purple acid phosphatase [Source:UniProtKB/TrEMBL;Acc:L8H162]	8.36E-05	0.249494267	0.249410691	1.05E-10
3h	ACA1_064490/clean/scf7180000084599:1627688-1627764:+	ACA1_064490	TYR_PHOSPHATASE_2 domain-containing protein [Source:UniProtKB/TrEMBL;Acc:L8GXK2]	0.002453333	0.251749333	0.249296	9.89E-05
3h	ACA1_068360/clean/scf7180000084603:179873-180005:+	ACA1_068360	Dual specificity protein kinase [Source:UniProtKB/TrEMBL;Acc:L8HDE4]	0.000173944	0.2494248	0.249250856	8.40E-09
3h	ACA1_237870/clean/scf7180000084763:37297-37437:+	ACA1_237870	Methyltransferase [Source:UniProtKB/TrEMBL;Acc:L8GKC5]	0.005472853	0.254579667	0.249106813	7.57E-09

3h	ACA1_173790/clean/scf7180000084741:520084-520172:+	ACA1_173790	Nicotinamide-nucleotide adenylyltransferase [Source:UniProtKB/TrEMBL;Acc:L8HK06]	0.000633378	0.2497374	0.249104022	4.67E-07
3h	ACA1_156080/clean/scf7180000084723:19248-19325:-	ACA1_156080	Aminoacylase 1, putative [Source:UniProtKB/TrEMBL;Acc:L8GK16]	0.003584233	0.252572	0.248987767	7.66E-06
3h	ACA1_301260/clean/scf7180000083203:11338-11432:-	ACA1_301260	WD_REPEATS_REGION domain-containing protein [Source:UniProtKB/TrEMBL;Acc:L8H0Y0]	0.00535341	0.254229	0.24887559	2.00E-10
3h	ACA1_282880/clean/scf7180000084776:741079-741175:+	ACA1_282880	DUF667 domain-containing protein [Source:UniProtKB/TrEMBL;Acc:L8H7B1]	0.0060606	0.254907933	0.248847333	0.000114546
3h	ACA1_189580/clean/scf7180000084748:52472-52550:-	ACA1_189580	Viral life cyclerelated protein [Source:UniProtKB/TrEMBL;Acc:L8GE33]	0.003291873	0.252109	0.248817127	2.03E-06
3h	ACA1_201450/clean/scf7180000084753:553769-553885:+	ACA1_201450	ADPribosylation factor, putative [Source:UniProtKB/TrEMBL;Acc:L8H3G5]	0.014649867	0.263436233	0.248786367	0.000106894
3h	ACA1_050360/clean/scf7180000084589:78236-78298:-	ACA1_050360	Metalloenzyme domain-containing protein [Source:UniProtKB/TrEMBL;Acc:L8H565]	0	0.248785667	0.248785667	2.77E-08
3h	ACA1_089970/clean/scf7180000084639:406694-406801:-	ACA1_089970	Macro domain-containing protein [Source:UniProtKB/TrEMBL;Acc:L8GU43]	0.013189887	0.2619295	0.248739613	0.000173987
3h	ACA1_264220/clean/scf7180000084770:214215-214375:+	ACA1_264220	hypothetical protein	0.001992773	0.250708067	0.248715293	2.56E-06
3h	ACA1_065450/clean/scf7180000084599:1854586-1854660:-	ACA1_065450	Cation_ATPase_N domain-containing protein [Source:UniProtKB/TrEMBL;Acc:L8H037]	0	0.248582067	0.248582067	0.00752976
3h	ACA1_136150/clean/scf7180000084702:139866-139930:-	ACA1_136150	MPN domain-containing protein [Source:UniProtKB/TrEMBL;Acc:L8GEU6]	0.00232513	0.2508132	0.24848807	3.63E-08
3h	ACA1_228260/clean/scf7180000084761:161774-161877:+	ACA1_228260	RRM domain-containing protein [Source:UniProtKB/TrEMBL;Acc:L8H8W0]	0.054218167	0.302677333	0.248459167	0.000923166
3h	ACA1_216320/clean/scf7180000084758:467095-467234:+	ACA1_216320	Homogentisate 1,2dioxygenase [Source:UniProtKB/TrEMBL;Acc:L8GQ83]	0.000604008	0.249058767	0.248454759	9.74E-08
3h	ACA1_325210/clean/scf7180000084475:31057-31212:+	ACA1_325210	CitMHS domain-containing protein [Source:UniProtKB/TrEMBL;Acc:L8GPH6]	0.011348313	0.259708333	0.24836002	9.55E-06
3h	ACA1_271740/clean/scf7180000084771:96036-96353:+	ACA1_271740	SAM domain (Sterile alpha motif) domain containing protein [Source:UniProtKB/TrEMBL;Acc:L8GPR2]	0.00900884	0.257315333	0.248306493	6.41E-07
3h	ACA1_045070/clean/scf7180000084576:73165-73266:-	ACA1_045070	SAYSvFN domain-containing protein [Source:UniProtKB/TrEMBL;Acc:L8GZT3]	0.005816857	0.254114867	0.24829801	1.39E-06
3h	ACA1_175300/clean/scf7180000084741:916987-917073:+	ACA1_175300	Zinc knuckle domain containing protein [Source:UniProtKB/TrEMBL;Acc:L8HKB8]	0	0.248235833	0.248235833	0.000314475
3h	ACA1_295810/clean/scf7180000084777:1237309-1237448:+	ACA1_295810	RING-type domain-containing protein [Source:UniProtKB/TrEMBL;Acc:L8HIR0]	0.051772367	0.300001333	0.248228967	0.002300069
3h	ACA1_325150/clean/scf7180000084475:11453-11528:+	ACA1_325150	Rab7/RabGfamily small GTPase [Source:UniProtKB/TrEMBL;Acc:L8GPH2]	0.00332551	0.251525867	0.248200357	7.82E-08
3h	ACA1_185040/clean/scf7180000084746:74904-74992:-	ACA1_185040	hypothetical protein	0.024866133	0.272896733	0.2480306	0.000386497
3h	ACA1_131530/clean/scf7180000084699:79171-79272:+	ACA1_131530	Nudix hydrolase domain-containing protein [Source:UniProtKB/TrEMBL;Acc:L8GM80]	0.015854437	0.263832833	0.247978397	0.002313882
3h	ACA1_238590/clean/scf7180000084763:103046-103115:+	ACA1_238590	CBS domain containing protein [Source:UniProtKB/TrEMBL;Acc:L8GKE6]	0.014860067	0.262653267	0.2477932	5.16E-05
3h	ACA1_288000/clean/scf7180000084777:144396-144495:+	ACA1_288000	E2_bind domain-containing protein [Source:UniProtKB/TrEMBL;Acc:L8HHS0]	0.01110195	0.258828167	0.247726217	1.12E-07
3h	ACA1_332820/clean/scf7180000084579:48411-48501:+	ACA1_332820	Sel1 repeatcontaining protein [Source:UniProtKB/TrEMBL;Acc:L8GLV3]	0.027911733	0.275597233	0.2476855	0.007922168
3h	ACA1_216020/clean/scf7180000084758:434268-434371:-	ACA1_216020	CudA family protein [Source:UniProtKB/TrEMBL;Acc:L8GQ76]	0.0161939	0.263854667	0.247660767	3.84E-06
3h	ACA1_165830/clean/scf7180000084732:98918-99008:-	ACA1_165830	Glutathione transferase family protein [Source:UniProtKB/TrEMBL;Acc:L8HGI5]	0.0258069	0.273296967	0.247490067	0.000127002
3h	ACA1_210550/clean/scf7180000084758:56127-56226:-	ACA1_210550	ABC transmembrane type-1 domain-containing protein [Source:UniProtKB/TrEMBL;Acc:L8GQQ5]	0.0128205	0.2602312	0.2474107	0.045296587
3h	ACA1_325600/clean/scf7180000084495:41784-41863:-	ACA1_325600	Protein kinase domain containing protein [Source:UniProtKB/TrEMBL;Acc:L8HB78]	0.004501247	0.251896433	0.247395187	2.68E-06
3h	ACA1_369180/clean/scf7180000084664:267533-267602:+	ACA1_369180	acidPPc domain-containing protein [Source:UniProtKB/TrEMBL;Acc:L8H1E2]	0.006017231	0.253225	0.247207769	2.58E-07
3h	ACA1_020770/clean/scf7180000084095:546-795:+	ACA1_020770	Multisensor hybrid histidine kinase [Source:UniProtKB/TrEMBL;Acc:L8GWG6]	0.006642327	0.253836	0.247193673	0.000168146
3h	ACA1_055400/clean/scf7180000084594:696198-696270:-	ACA1_055400	hypothetical protein	0	0.247080333	0.247080333	0.002810918
3h	ACA1_150570/clean/scf7180000084716:13500-13563:-	ACA1_150570	Rhodanese domain-containing protein [Source:UniProtKB/TrEMBL;Acc:L8H0M9]	0.012652613	0.259671167	0.247018553	6.85E-06
3h	ACA1_006200/clean/scf7180000083029:68348-68436:-	ACA1_006200	hypothetical protein	0.036906133	0.283641433	0.2467353	0.002784356
3h	ACA1_252210/clean/scf7180000084768:146231-146296:+	ACA1_252210	Protein kinase domain-containing protein [Source:UniProtKB/TrEMBL;Acc:L8HAM0]	0.004716967	0.2513104	0.246593433	0.00048827
3h	ACA1_292140/clean/scf7180000084777:860346-860436:-	ACA1_292140	hypothetical protein	0.005452477	0.251989667	0.24653719	0.000109371
3h	ACA1_144920/clean/scf7180000084710:39308-39400:-	ACA1_144920	IgA Peptidase M64 protein [Source:UniProtKB/TrEMBL;Acc:L8GFD4]	0.015096553	0.261531	0.246434447	5.42E-06
3h	ACA1_018960/clean/scf7180000083912:35000-35107:-	ACA1_018960	Papain family cysteine protease subfamily protein [Source:UniProtKB/TrEMBL;Acc:L8HJH5]	0.000849014	0.247182733	0.24633372	1.89E-07
3h	ACA1_173230/clean/scf7180000084741:435358-435445:-	ACA1_173230	hypothetical protein	0.002465043	0.248793667	0.246328623	5.62E-06
3h	ACA1_379260/clean/scf7180000084711:313424-313505:+	ACA1_379260	Coatomer subunit beta [Source:UniProtKB/TrEMBL;Acc:L8GRY6]	0.00262031	0.248933767	0.246313457	1.63E-07
3h	ACA1_285200/clean/scf7180000084776:955236-955321:-	ACA1_285200	Na_H_Exchanger domain-containing protein [Source:UniProtKB/TrEMBL;Acc:L8H9U2]	0.001652919	0.2479455	0.246292581	2.32E-07
3h	ACA1_192020/clean/scf7180000084749:76837-76939:+	ACA1_192020	Cystathionine gammasynthase [Source:UniProtKB/TrEMBL;Acc:L8GNB3]	0.01016329	0.256389667	0.246226377	9.84E-09
3h	ACA1_228690/clean/scf7180000084761:191341-191452:+	ACA1_228690	MORN repeatcontaining protein [Source:UniProtKB/TrEMBL;Acc:L8HA78]	0.024817233	0.271010633	0.2461934	0.001571461
3h	ACA1_309500/clean/scf7180000083762:8986-9083:+	ACA1_309500	Pyridine nucleotidedisulfide oxidoreductase domain containing protein [Source:UniProtKB/TrEMBL;Acc:L8GI23]	0.003006643	0.249064667	0.246058024	5.41E-08
3h	ACA1_142370/clean/scf7180000084708:152902-153020:-	ACA1_142370	Serine hydroxymethyltransferase [Source:UniProtKB/TrEMBL;Acc:L8HDT1]	0.000236769	0.2462318	0.245995031	1.15E-09
3h	ACA1_310620/clean/scf7180000083773:17019-17085:-	ACA1_310620	Protein kinase domain-containing protein [Source:UniProtKB/TrEMBL;Acc:L8H1T9]	0.005196379	0.251029733	0.245833355	6.75E-05
3h	ACA1_178340/clean/scf7180000084743:220927-221063:+	ACA1_178340	RhoGEF domain containing protein [Source:UniProtKB/TrEMBL;Acc:L8GT64]	0.000835643	0.246651567	0.245815923	1.87E-05
3h	ACA1_398190/clean/scf7180000084759:213373-213445:-	ACA1_398190	6-phosphogluconate dehydrogenase, decarboxylating [Source:UniProtKB/TrEMBL;Acc:L8HDP8]	0.0295123	0.2753194	0.2458071	0.007266213
3h	ACA1_089040/clean/scf7180000084639:304449-304525:+	ACA1_089040	UBA/TS-N domain containing protein [Source:UniProtKB/TrEMBL;Acc:L8GUR2]	0.017070193	0.262751333	0.24568114	2.07E-05
3h	ACA1_155270/clean/scf7180000084722:136505-136575:+	ACA1_155270	PhytanoylCoA dioxygenase (PhyH) superfamily protein [Source:UniProtKB/TrEMBL;Acc:L8H032]	0.01749577	0.263067667	0.245571897	0.002488326
3h	ACA1_165570/clean/scf7180000084732:46656-46746:-	ACA1_165570	WW domain-containing protein [Source:UniProtKB/TrEMBL;Acc:L8HGG7]	0.006820117	0.252257667	0.24543755	0.000254938
3h	ACA1_230500/clean/scf7180000084761:363815-363885:+	ACA1_230500	Ammonium transporter [Source:UniProtKB/TrEMBL;Acc:L8H844]	0	0.245334667	0.245334667	2.38E-07
3h	ACA1_165270/clean/scf7180000084732:23130-23231:+	ACA1_165270	hypothetical protein	0.006266737	0.251481	0.245214263	2.09E-05
3h	ACA1_062700/clean/scf7180000084599:1277253-1277319:+	ACA1_062700	ULP_PROTEASE domain-containing protein [Source:UniProtKB/TrEMBL;Acc:L8GZJ7]	0.01724016	0.2624529	0.24521274	0.000230003
3h	ACA1_057240/clean/scf7180000084599:94399-94494:-	ACA1_057240	unspecified product	0	0.2451716	0.2451716	1.85E-05
3h	ACA1_066140/clean/scf7180000084599:2003680-2003891:-	ACA1_066140	AP complex subunit sigma [Source:UniProtKB/TrEMBL;Acc:L8GZ32]	0.01646418	0.261623833	0.245159653	1.28E-05
3h	ACA1_355850/clean/scf7180000084728:31588-31649:-	ACA1_355850	Methionine aminopeptidase [Source:UniProtKB/TrEMBL;Acc:L8H6U0]	0.007038517	0.252136067	0.24509755	3.61E-08
3h	ACA1_260810/clean/scf7180000084769:529502-529582:-	ACA1_260810	MYND finger domain containing protein [Source:UniProtKB/TrEMBL;Acc:L8GHM1]	0.00061511	0.245703	0.24508789	3.95E-06
3h	ACA1_152200/clean/scf7180000084719:34064-34152:-	ACA1_152200	ANK_REP_REGION domain-containing protein [Source:UniProtKB/TrEMBL;Acc:L8GG74]	0.010469933	0.255531	0.245061067	0.028762587
3h	ACA1_163660/clean/scf7180000084729:65732-65826:-	ACA1_163660	hypothetical protein	0.002239043	0.247271833	0.24503279	1.83E-06
3h	ACA1_364760/clean/scf7180000084641:191396-191493:+	ACA1_364760	Endonuclease [Source:UniProtKB/TrEMBL;Acc:L8GMG4]	0.000716071	0.245730067	0.245013995	5.75E-08
3h	ACA1_062620/clean/scf7180000084599:1255130-1255211:-	ACA1_062620	hypothetical protein	0.006092497	0.2510503	0.244957803	1.28E-05
3h	ACA1_228690/clean/scf7180000084761:191037-191131:+	ACA1_228690	MORN repeatcontaining protein [Source:UniProtKB/TrEMBL;Acc:L8HA78]	0.012327553	0.257065667	0.244738113	5.65E-05
3h	ACA1_082860/clean/scf7180000084625:13520-13642:-	ACA1_082860	Peptidase S9, prolyl oligopeptidase active site region, putative [Source:UniProtKB/TrEMBL;Acc:L8GTP0]	0.002814787	0.247544667	0.24472988	2.52E-05
3h	ACA1_009930/clean/scf7180000083468:12139-12227:+	ACA1_009930	Acyl-CoA_dh_1 domain-containing protein [Source:UniProtKB/TrEMBL;Acc:L8GFW8]	0	0.2446655	0.2446655	2.82E-08
3h	ACA1_142390/clean/scf7180000084708:157897-157981:-	ACA1_142390	Acetyltransferase component of pyruvate dehydrogenase complex [Source:UniProtKB/TrEMBL;Acc:L8HCS5]	0.000435418	0.245100067	0.244664649	6.49E-11
3h	ACA1_209780/clean/scf7180000084757:317260-317341:-	ACA1_209780	Glycogen debranching enzyme [Source:UniProtKB/TrEMBL;Acc:L8GZQ5]	0.185386	0.430014667	0.244628667	0.034577936
3h	ACA1_268430/clean/scf7180000084770:905691-905838:-	ACA1_268430	SAM domain (Sterile alpha motif) domain containing protein [Source:UniProtKB/TrEMBL;Acc:L8H2M3]	0.080403767	0.324903533	0.244499767	0.047219246
3h	ACA1_070990/clean/scf7180000084603:822851-822953:+	ACA1_070990	hypothetical protein	0	0.244438	0.244438	1.39E-07
3h	ACA1_142400/clean/scf7180000084708:161044-161182:+	ACA1_142400	Plus3 domain-containing protein [Source:UniProtKB/TrEMBL;Acc:L8HAK6]	0.013300407	0.257722067	0.24442166	0.000284948
3h	ACA1_095060/clean/scf7180000084645:802634-802714:-	ACA1_095060	Sidoreflexin [Source:UniProtKB/TrEMBL;Acc:L8GL14]	0.016990767	0.261397533	0.244406767	0.001517297
3h	ACA1_101310/clean/scf7180000084649:44350-44425:+	ACA1_101310	VIT domain-containing protein [Source:UniProtKB/TrEMBL;Acc:L8GG72]	0.000581733	0.2449782	0.244396467	0.00022308
3h	ACA1_384010/clean/scf7180000084730:3615-3682:-	ACA1_384010	Arf-GAP domain-containing protein [Source:UniProtKB/TrEMBL;Acc:L8HAG1]	0.001999383	0.2463813	0.244381917	1.24E-07
3h	ACA1_245660/clean/scf7180000084763:516751-516846:-	ACA1_245660	hypothetical protein	0.01536484	0.259669533	0.244304693	0.000567994
3h	ACA1_139420/clean/scf7180000084705:83687-83860:+	ACA1_139420	Calcium binding protein 39 (Mo25 protein) isoform 5, putative [Source:UniProtKB/TrEMBL;Acc:L8GMD6]	0.007777913	0.2520461	0.244268187	9.81E-07
3h	ACA1_394150/clean/scf7180000084756:91167-91235:-	ACA1_394150	hypothetical protein	0.016146733	0.260392	0.244245267	0.000315734
3h	ACA1_272960/clean/scf7180000084773:4249-4339:-	ACA1_272960	Ras-GAP domain-containing protein [Source:UniProtKB/TrEMBL;Acc:L8GFH7]	3.50E-05	0.244164633	0.244129623	1.02E-07
3h	ACA1_113680/clean/scf7180000084669:348603-348818:-	ACA1_113680	M20_dimer domain-containing protein [Source:UniProtKB/TrEMBL;Acc:L8H5M1]	0.009586267	0.253656667	0.2440704	3.49E-08
3h	ACA1_199640/clean/scf7180000084753:287020-287304:-	ACA1_199640	RFX-type winged-helix domain-containing protein [Source:UniProtKB/TrEMBL;Acc:L8H5G1]	0.0230468	0.2669714	0.2439246	0.000326232
3h	ACA1_037740/clean/scf7180000084568:13020-13114:-	ACA1_037740	hypothetical protein	0.00459506	0.248492	0.24389694	8.37E-06
3h	ACA1_392270/clean/scf7180000084740:266569-266636:-	ACA1_392270	Carrier superfamily protein [Source:UniProtKB/TrEMBL;Acc:L8GP94]	0.0440494	0.287946133	0.243896733	0.003658577
3h	ACA1_389120/clean/scf7180000084733:203103-203180:+	ACA1_389120	DENN (AEX3) domain containing protein [Source:UniProtKB/TrEMBL;Acc:L8GG93]	0.003836787	0.247695333	0.243858547	6.20E-06
3h	ACA1_382350/clean/scf7180000084721:36281-36386:-	ACA1_382350	RFX-type winged-helix domain-containing protein [Source:UniProtKB/TrEMBL;Acc:L8GXF7]	0.0314355	0.275262333	0.243826833	0.016010805
3h	ACA1_062570/clean/scf7180000084599:1236193-1236300:-	ACA1_062570	Zinc finger, c2h2 type domain containing protein [Source:UniProtKB/TrEMBL;Acc:L8GY95]	0.00387003	0.247611433	0.243741403	3.82E-05
3h	ACA1_171280/clean/scf7180000084741:65302-65599:+	ACA1_171280	Nicotinate phosphoribosyltransferase [Source:UniProtKB/TrEMBL;Acc:L8HHA8]	0.034587367	0.278325	0.243737633	0.014550933
3h	ACA1_392530/clean/scf7180000084740:287728-287811:+	ACA1_392530	tRNA-synt_1e domain-containing protein [Source:UniProtKB/TrEMBL;Acc:L8GNW6]	0.001949317	0.2455648	0.243615483	7.65E-06
3h	ACA1_210540/clean/scf7180000084758:51193-51270:-	ACA1_210540	ABC transporter, ATPbinding domain containing protein [Source:UniProtKB/TrEMBL;Acc:L8GPZ1]	0.010089753	0.253646367	0.243556613	0.004786584
3h	ACA1_111950/clean/scf7180000084669:69586-69872:+	ACA1_111950	Lipid transport family protein [Source:UniProtKB/TrEMBL;Acc:L8H3W2]	0.000494416	0.244047233	0.243552817	4.70E-09
3h	ACA1_396170/clean/scf7180000084759:44380-44443:-	ACA1_396170	Phosphatidylserine decarboxylase proenzyme 2 [Source:UniProtKB/TrEMBL;Acc:L8HEJ4]	0.01256128	0.256106633	0.243545353	8.65E-06
3h	ACA1_264150/clean/scf7180000084770:200414-200486:-	ACA1_264150	Gammaglutamyltranspeptidase [Source:UniProtKB/TrEMBL;Acc:L8H169]	0.004629633	0.248142	0.243512367	0.001211812
3h	ACA1_326120/clean/scf7180000084504:29102-29208:+	ACA1_326120	Glycogen [starch] synthase [Source:UniProtKB/TrEMBL;Acc:L8HIJ6]	0.0005698	0.2440018	0.243432	6.06E-08
3h	ACA1_276460/clean/scf7180000084774:185222-185330:+	ACA1_276460	unspecified product	0.0198514	0.263225667	0.243374267	5.81E-05
3h	ACA1_321910/clean/scf7180000084457:8494-8584:-	ACA1_321910	Importin N-terminal domain-containing protein [Source:UniProtKB/TrEMBL;Acc:L8H9I3]	0.064255233	0.307626333	0.2433711	0.005892571
3h	ACA1_027030/clean/scf7180000084464:27463-27546:-	ACA1_027030	C2 domain-containing protein [Source:UniProtKB/TrEMBL;Acc:L8GJ80]	0.002202571	0.2455133	0.243310729	1.46E-05
3h	ACA1_238410/clean/scf7180000084763:86954-87061:+	ACA1_238410	hypothetical protein	0.004861923	0.2481041	0.243242177	3.26E-07
3h	ACA1_399880/clean/scf7180000084775:57090-57154:+	ACA1_399880	Rab-GAP TBC domain-containing protein [Source:UniProtKB/TrEMBL;Acc:L8GGF5]	0	0.2431591	0.2431591	1.43E-05
3h	ACA1_243850/clean/scf7180000084763:363575-363721:+	ACA1_243850	Ras-GAP domain-containing protein [Source:UniProtKB/TrEMBL;Acc:L8GJZ5]	0	0.242940333	0.242940333	0.007186395
3h	ACA1_033610/clean/scf7180000084559:60882-60970:+	ACA1_033610	Sortilin [Source:UniProtKB/TrEMBL;Acc:L8GHJ0]	0.004428793	0.247280733	0.24285194	2.09E-06
3h	ACA1_358660/clean/scf7180000084742:25344-25435:+	ACA1_358660	SH3 domain containing protein [Source:UniProtKB/TrEMBL;Acc:L8GLJ2]	0.039400167	0.282247967	0.2428478	0.003402011
3h	ACA1_089130/clean/scf7180000084639:327752-327824:+	ACA1_089130	Beta-lactamase domain-containing protein [Source:UniProtKB/TrEMBL;Acc:L8GX66]	0.049658767	0.292428	0.242769233	0.001013681
3h	ACA1_199000/clean/scf7180000084753:189542-189633:-	ACA1_199000	hypothetical protein	0.00077566	0.243525867	0.242750207	4.57E-07
3h	ACA1_035560/clean/scf7180000084564:88600-88686:-	ACA1_035560	Short chain dehydrogenase/reductase family oxidoreductase [Source:UniProtKB/TrEMBL;Acc:L8HCW5]	0.0046195	0.247327667	0.242708167	1.44E-05
3h	ACA1_379060/clean/scf7180000084711:272847-272932:+	ACA1_379060	Protein kinase domain-containing protein [Source:UniProtKB/TrEMBL;Acc:L8GRW8]	0.000754147	0.243333867	0.24257972	4.78E-07
3h	ACA1_073000/clean/scf7180000084603:1352919-1353008:+	ACA1_073000	Mis12 domain containing protein [Source:UniProtKB/TrEMBL;Acc:L8HGW7]	0.105379567	0.347853333	0.242473767	0.011995151
3h	ACA1_203090/clean/scf7180000084754:141553-141661:+	ACA1_203090	Glycosyl hydrolase, family 31 protein [Source:UniProtKB/TrEMBL;Acc:L8GTG9]	0.00119904	0.243616367	0.242417327	1.26E-06
3h	ACA1_325260/clean/scf7180000084475:40561-40625:+	ACA1_325260	Peptidylprolyl isomerase [Source:UniProtKB/TrEMBL;Acc:L8GPH9]	0.004205623	0.246570633	0.24236501	1.21E-08
3h	ACA1_002780/clean/scf7180000082937:10127-10209:-	ACA1_002780	RRM domain-containing protein [Source:UniProtKB/TrEMBL;Acc:L8GGZ7]	0.026130487	0.268432667	0.24230218	0.00457315
3h	ACA1_258610/clean/scf7180000084769:281653-281768:+	ACA1_258610	hypothetical protein	0.006421467	0.248677267	0.2422558	0.014002325
3h	ACA1_385390/clean/scf7180000084730:158370-158442:+	ACA1_385390	Ubiquitinconjugating enzyme subfamily protein [Source:UniProtKB/TrEMBL;Acc:L8HAL6]	0.001358457	0.2434229	0.242064443	8.69E-08
3h	ACA1_065450/clean/scf7180000084599:1854001-1854066:-	ACA1_065450	Cation_ATPase_N domain-containing protein [Source:UniProtKB/TrEMBL;Acc:L8H037]	0	0.242062133	0.242062133	0.007752111
3h	ACA1_290510/clean/scf7180000084777:606867-606945:-	ACA1_290510	ABC transporter domain-containing protein [Source:UniProtKB/TrEMBL;Acc:L8HLJ2]	0.010224507	0.252162367	0.24193786	2.08E-05
3h	ACA1_216320/clean/scf7180000084758:468030-468136:+	ACA1_216320	Homogentisate 1,2dioxygenase [Source:UniProtKB/TrEMBL;Acc:L8GQ83]	0.002906133	0.244695833	0.2417897	3.26E-08
3h	ACA1_271720/clean/scf7180000084771:89219-89315:-	ACA1_271720	Ras-GAP domain-containing protein [Source:UniProtKB/TrEMBL;Acc:L8GR76]	0.023774913	0.265549	0.241774087	4.16E-05
3h	ACA1_175570/clean/scf7180000084741:1000054-1000171:-	ACA1_175570	DH domain-containing protein [Source:UniProtKB/TrEMBL;Acc:L8HI39]	0.028082467	0.269743	0.241660533	7.08E-05
3h	ACA1_376560/clean/scf7180000084685:107515-107600:+	ACA1_376560	Ankyrin repeatcontaining protein [Source:UniProtKB/TrEMBL;Acc:L8HHK7]	0.0365367	0.2781558	0.2416191	0.015919659
3h	ACA1_187780/clean/scf7180000084747:94348-94428:-	ACA1_187780	Corrinoid adenosyltransferase [Source:UniProtKB/TrEMBL;Acc:L8GUU3]	0.003834247	0.2454465	0.241612253	5.06E-06
3h	ACA1_271600/clean/scf7180000084771:81682-81799:-	ACA1_271600	Cation diffusion facilitator family transporter superfamily protein [Source:UniProtKB/TrEMBL;Acc:L8GNJ1]	0.002245847	0.243853333	0.241607487	1.90E-05
3h	ACA1_081100/clean/scf7180000084618:89798-89896:-	ACA1_081100	Prephenate dehydratase [Source:UniProtKB/TrEMBL;Acc:L8HE69]	0.005854766	0.247413333	0.241558567	1.09E-05
3h	ACA1_027770/clean/scf7180000084471:5776-5875:+	ACA1_027770	GATA-type domain-containing protein [Source:UniProtKB/TrEMBL;Acc:L8HK85]	0.037569667	0.2790719	0.241502233	0.003155026
3h	ACA1_092760/clean/scf7180000084645:399460-399552:-	ACA1_092760	hypothetical protein	0	0.241484433	0.241484433	0.000448144
3h	ACA1_054090/clean/scf7180000084594:398387-398478:-	ACA1_054090	Fbox domain containing protein [Source:UniProtKB/TrEMBL;Acc:L8H592]	0.051045067	0.292367567	0.2413225	0.047464842
3h	ACA1_109690/clean/scf7180000084663:1185-1265:+	ACA1_109690	Transmembrane and coiled-coil domains 1 family protein [Source:UniProtKB/TrEMBL;Acc:L8HJY2]	0.003993063	0.245075067	0.241082003	4.11E-08
3h	ACA1_051530/clean/scf7180000084592:29949-30020:-	ACA1_051530	MFS domain-containing protein [Source:UniProtKB/TrEMBL;Acc:L8GPJ8]	0	0.240994367	0.240994367	2.54E-05
3h	ACA1_107530/clean/scf7180000084660:215407-215508:-	ACA1_107530	UBP-type domain-containing protein [Source:UniProtKB/TrEMBL;Acc:L8GMW8]	0.0131812	0.2541465	0.2409653	1.27E-06
3h	ACA1_307820/clean/scf7180000083687:19214-19285:-	ACA1_307820	BSD domain-containing protein [Source:UniProtKB/TrEMBL;Acc:L8HBM7]	0.024265033	0.26519	0.240924967	5.02E-05
3h	ACA1_087970/clean/scf7180000084639:146642-146710:+	ACA1_087970	NACHT domain containing protein [Source:UniProtKB/TrEMBL;Acc:L8GUL2]	0.01061195	0.251522333	0.240910383	6.29E-05
3h	ACA1_166070/clean/scf7180000084732:112050-112168:-	ACA1_166070	Malate dehydrogenase [Source:UniProtKB/TrEMBL;Acc:L8HIY2]	0.000659301	0.241525233	0.240865932	3.53E-08
3h	ACA1_251570/clean/scf7180000084768:87039-87129:-	ACA1_251570	Vesicle-fusing ATPase [Source:UniProtKB/TrEMBL;Acc:L8HAQ4]	0.00739691	0.248207	0.24081009	0.000531862
3h	ACA1_385810/clean/scf7180000084730:270731-270804:+	ACA1_385810	Rap-GAP domain-containing protein [Source:UniProtKB/TrEMBL;Acc:L8HAQ1]	0.007133287	0.247623933	0.240490647	1.79E-05
3h	ACA1_010180/clean/scf7180000083486:7002-7083:+	ACA1_010180	DUF410 family protein [Source:UniProtKB/TrEMBL;Acc:L8HD09]	0.00191947	0.2423436	0.24042413	3.81E-06
3h	ACA1_360020/clean/scf7180000084622:34006-34082:-	ACA1_360020	Mannosyltransferase [Source:UniProtKB/TrEMBL;Acc:L8HCL9]	0	0.240407167	0.240407167	6.63E-07
3h	ACA1_264380/clean/scf7180000084770:255576-255666:-	ACA1_264380	S-formylglutathione hydrolase [Source:UniProtKB/TrEMBL;Acc:L8H1Z7]	0.000946877	0.2412386	0.240291723	9.98E-08
3h	ACA1_323140/clean/scf7180000084467:14239-14333:-	ACA1_323140	RFX-type winged-helix domain-containing protein [Source:UniProtKB/TrEMBL;Acc:L8GEG4]	0	0.2402831	0.2402831	0.00251767
3h	ACA1_255750/clean/scf7180000084768:560812-560965:+	ACA1_255750	hypothetical protein	0.051598933	0.291815667	0.240216733	0.001830126
3h	ACA1_185890/clean/scf7180000084746:140466-140556:+	ACA1_185890	Peptidase S74 domain-containing protein [Source:UniProtKB/TrEMBL;Acc:L8H596]	0.011818293	0.2520348	0.240216507	7.49E-06
3h	ACA1_217280/clean/scf7180000084758:556192-556295:+	ACA1_217280	Protein kinase domain containing protein [Source:UniProtKB/TrEMBL;Acc:L8GQB1]	0.008436673	0.248647167	0.240210493	2.30E-07
3h	ACA1_361100/clean/scf7180000084622:241585-241688:-	ACA1_361100	TLDc domain-containing protein [Source:UniProtKB/TrEMBL;Acc:L8HE93]	0	0.240157167	0.240157167	8.58E-07
3h	ACA1_067380/clean/scf7180000084601:5061-5140:-	ACA1_067380	hypothetical protein	0.020572567	0.260704067	0.2401315	0.003282606
3h	ACA1_024050/clean/scf7180000084440:18351-18457:+	ACA1_024050	Zn fingercontaining protein [Source:UniProtKB/TrEMBL;Acc:L8GHT1]	0	0.240026667	0.240026667	2.25E-06

3h	ACA1_012020/clean/scf7180000083652:5417-5545:-	ACA1_012020	hypothetical protein	0.001103432	0.2411209	0.240017468	3.90E-07
3h	ACA1_091040/clean/scf7180000084645:45578-45654:-	ACA1_091040	Amidohydro-rel domain-containing protein [Source:UniProtKB/TrEMBL;Acc:L8GIF1]	5.61E-05	0.2400222	0.239966093	1.92E-05
3h	ACA1_383630/clean/scf7180000084721:234073-234172:+	ACA1_383630	Peptidylprolyl isomerase [Source:UniProtKB/TrEMBL;Acc:L8GV01]	0.000748586	0.240662367	0.23991378	1.86E-08
3h	ACA1_034090/clean/scf7180000084561:21671-21749:-	ACA1_034090	Protein kinase domain containing protein [Source:UniProtKB/TrEMBL;Acc:L8GWJ8]	0.013574683	0.253452567	0.239877883	1.19E-05
3h	ACA1_376690/clean/scf7180000084685:141469-141621:-	ACA1_376690	Ribosomal_L18e/L15P domain-containing protein [Source:UniProtKB/TrEMBL;Acc:L8HGC7]	0.00803222	0.2479053	0.23987308	8.90E-13
3h	ACA1_064570/clean/scf7180000084599:1645623-1645710:+	ACA1_064570	Ras subfamily protein [Source:UniProtKB/TrEMBL;Acc:L8GXF9]	0.004629633	0.244498833	0.2398692	0.00058357
3h	ACA1_144880/clean/scf7180000084710:31250-31334:-	ACA1_144880	hypothetical protein	0.001510673	0.2413255	0.239814827	2.54E-05
3h	ACA1_183960/clean/scf7180000084745:529517-529589:-	ACA1_183960	Phosphoesterase family protein [Source:UniProtKB/TrEMBL;Acc:L8H8F6]	0.008223267	0.247991967	0.2397687	1.62E-06
3h	ACA1_002500/clean/scf7180000082931:9946-10019:+	ACA1_002500	hypothetical protein	0.000970407	0.240695467	0.23972506	2.02E-08
3h	ACA1_341100/clean/scf7180000084620:50207-50294:-	ACA1_341100	MFS domain-containing protein [Source:UniProtKB/TrEMBL;Acc:L8GR49]	0.014735947	0.254460167	0.23972422	4.03E-05
3h	ACA1_210250/clean/scf7180000084758:16123-16212:-	ACA1_210250	HP domain-containing protein [Source:UniProtKB/TrEMBL;Acc:L8GQP3]	0.00481496	0.244457967	0.239643007	4.07E-06
3h	ACA1_108200/clean/scf7180000084661:11256-11342:-	ACA1_108200	hypothetical protein	0	0.239626333	0.239626333	0.003414749
3h	ACA1_372060/clean/scf7180000084664:624148-624217:-	ACA1_372060	Aldo_ket_red domain-containing protein [Source:UniProtKB/TrEMBL;Acc:L8GYH3]	0.01067527	0.250227	0.23955173	0.005499461
3h	ACA1_126940/clean/scf7180000084696:5229-5308:+	ACA1_126940	hypothetical protein	0.009694853	0.2491906	0.239495747	0.000152323
3h	ACA1_183350/clean/scf7180000084745:383835-383945:+	ACA1_183350	Short chain dehydrogenase/reductase family [Source:UniProtKB/TrEMBL;Acc:L8HAD9]	0.009127413	0.2485726	0.239445187	7.04E-05
3h	ACA1_346040/clean/scf7180000084657:16138-16221:+	ACA1_346040	Ras-related protein Rab-7A [Source:UniProtKB/TrEMBL;Acc:L8HHF3]	0.016586233	0.255973433	0.2393872	7.86E-06
3h	ACA1_183970/clean/scf7180000084745:533863-533922:-	ACA1_183970	hypothetical protein	0.038152667	0.2775141	0.239361433	0.045615866
3h	ACA1_182660/clean/scf7180000084745:228878-229001:+	ACA1_182660	hypothetical protein	0.005555567	0.2445115	0.238955933	0.000585524
3h	ACA1_254430/clean/scf7180000084768:391302-391413:+	ACA1_254430	Macro domain-containing protein [Source:UniProtKB/TrEMBL;Acc:L8HDC4]	0.016005767	0.2548276	0.238821833	5.10E-05
3h	ACA1_236940/clean/scf7180000084762:486531-486645:-	ACA1_236940	hypothetical protein	0.003577107	0.24224	0.238662893	3.79E-07
3h	ACA1_100560/clean/scf7180000084648:113230-113316:+	ACA1_100560	hypothetical protein	0.013252583	0.251901133	0.23864855	8.88E-05
3h	ACA1_024410/clean/scf7180000084442:16299-16378:+	ACA1_024410	Serine/threonineprotein kinase [Source:UniProtKB/TrEMBL;Acc:L8H7W5]	0	0.238633867	0.238633867	1.43E-08
3h	ACA1_268430/clean/scf7180000084770:906362-906474:-	ACA1_268430	SAM domain (Sterile alpha motif) domain containing protein [Source:UniProtKB/TrEMBL;Acc:L8H2M3]	0.070145033	0.308756	0.238610967	0.032969655
3h	ACA1_290280/clean/scf7180000084777:576590-576663:-	ACA1_290280	Exportin-T [Source:UniProtKB/TrEMBL;Acc:L8HI54]	0.004115233	0.242705333	0.2385901	0.000273479
3h	ACA1_380930/clean/scf7180000084712:118345-118450:-	ACA1_380930	hypothetical protein	0.029270833	0.2678268	0.238555967	0.000110877
3h	ACA1_176060/clean/scf7180000084741:1117130-1117249:+	ACA1_176060	DAGKc domain-containing protein [Source:UniProtKB/TrEMBL;Acc:L8HJR7]	0.00596722	0.244474	0.23850678	2.74E-05
3h	ACA1_061930/clean/scf7180000084599:1080384-1080448:-	ACA1_061930	Transporter, major intrinsic protein (MIP) superfamily protein [Source:UniProtKB/TrEMBL;Acc:L8GZB9]	0.0484818	0.286960333	0.238478533	0.001022857
3h	ACA1_378280/clean/scf7180000084711:226830-227005:+	ACA1_378280	hypothetical protein	0.027427247	0.265735233	0.238307987	0.003845428
3h	ACA1_364280/clean/scf7180000084641:70961-71118:+	ACA1_364280	Sporulation proteinrelated, putative [Source:UniProtKB/TrEMBL;Acc:L8GLJ0]	0.002591172	0.240873433	0.238282261	1.43E-08
3h	ACA1_236660/clean/scf7180000084762:469955-470034:-	ACA1_236660	Fbox domain containing protein [Source:UniProtKB/TrEMBL;Acc:L8H304]	0.01006308	0.248295633	0.238232553	0.000691233
3h	ACA1_382920/clean/scf7180000084721:118910-118983:-	ACA1_382920	Ras subfamily protein [Source:UniProtKB/TrEMBL;Acc:L8GV31]	0.006829427	0.244952033	0.238122607	0.000778963
3h	ACA1_076210/clean/scf7180000084612:25919-26023:-	ACA1_076210	hypothetical protein	0.008391083	0.2464053	0.238014217	3.44E-07
3h	ACA1_235680/clean/scf7180000084762:353996-354109:-	ACA1_235680	Rap-GAP domain-containing protein [Source:UniProtKB/TrEMBL;Acc:L8H1A5]	0.0320455	0.269934733	0.237889233	0.000965311
3h	ACA1_174120/clean/scf7180000084741:618207-618269:+	ACA1_174120	hypothetical protein	0.122805067	0.360593667	0.2377886	0.042075555
3h	ACA1_112100/clean/scf7180000084669:111424-111518:+	ACA1_112100	BTB/POZ domain containing protein [Source:UniProtKB/TrEMBL;Acc:L8H3Y0]	0.022410533	0.260159767	0.237749233	0.00174846
3h	ACA1_150570/clean/scf7180000084716:13948-14043:-	ACA1_150570	Rhodanese domain-containing protein [Source:UniProtKB/TrEMBL;Acc:L8H0M9]	0.00246184	0.2401923	0.23773046	6.17E-07
3h	ACA1_280550/clean/scf7180000084776:492064-492127:-	ACA1_280550	RSNARE, VAMP71-family [Source:UniProtKB/TrEMBL;Acc:L8H6Z7]	0.006000533	0.243577467	0.237576934	5.47E-06
3h	ACA1_185590/clean/scf7180000084746:112338-112485:+	ACA1_185590	EF hand domain containing protein [Source:UniProtKB/TrEMBL;Acc:L8H583]	0.001450037	0.2390165	0.237566463	1.93E-06
3h	ACA1_193320/clean/scf7180000084749:231548-231654:-	ACA1_193320	hypothetical protein	0	0.237532267	0.237532267	0.032449963
3h	ACA1_027500/clean/scf7180000084469:5711-5791:-	ACA1_027500	hypothetical protein	0	0.2374677	0.2374677	9.79E-07
3h	ACA1_224850/clean/scf7180000084760:254054-254166:+	ACA1_224850	Galactosebinding domain containing protein [Source:UniProtKB/TrEMBL;Acc:L8GSE7]	0.000284209	0.237634667	0.237350458	2.49E-08
3h	ACA1_164910/clean/scf7180000084729:308734-308817:-	ACA1_164910	hypothetical protein	0.023899233	0.261197067	0.237297833	0.000113611
3h	ACA1_169060/clean/scf7180000084739:13168-13262:-	ACA1_169060	Derlin [Source:UniProtKB/TrEMBL;Acc:L8HHS6]	0.01455372	0.251731833	0.237178113	4.05E-06
3h	ACA1_223790/clean/scf7180000084760:148273-148381:-	ACA1_223790	Saccharopine dehydrogenase (NAD+, Llysine-forming), putative protein	0.002227195	0.239366067	0.237138872	5.78E-07
3h	ACA1_027810/clean/scf7180000084471:23314-23405:+	ACA1_027810	YTH domain-containing protein [Source:UniProtKB/TrEMBL;Acc:L8HJ39]	0.01426967	0.251406333	0.237136663	0.000241816
3h	ACA1_144270/clean/scf7180000084709:191490-191567:+	ACA1_144270	SAC domain-containing protein [Source:UniProtKB/TrEMBL;Acc:L8HI07]	0	0.237019267	0.237019267	1.32E-05
3h	ACA1_279370/clean/scf7180000084776:373497-373608:+	ACA1_279370	Tetratricopeptide repeat domain containing protein [Source:UniProtKB/TrEMBL;Acc:L8H738]	0.028024867	0.265023667	0.2369988	0.000731246
3h	ACA1_128200/clean/scf7180000084697:46067-46151:-	ACA1_128200	CBS domain containing protein [Source:UniProtKB/TrEMBL;Acc:L8GUU2]	0.007053433	0.244026	0.236972567	0.000860974
3h	ACA1_091870/clean/scf7180000084645:219070-219155:-	ACA1_091870	Calmodulin [Source:UniProtKB/TrEMBL;Acc:L8GI85]	0.00488654	0.241706667	0.236820127	5.76E-07
3h	ACA1_278190/clean/scf7180000084776:188412-188575:-	ACA1_278190	MAT1 domain-containing protein [Source:UniProtKB/TrEMBL;Acc:L8H822]	0.022666067	0.2593908	0.236724733	5.23E-05
3h	ACA1_061200/clean/scf7180000084599:852162-852275:+	ACA1_061200	5' nucleotidase family protein [Source:UniProtKB/TrEMBL;Acc:L8GWQ7]	0.00403262	0.240461667	0.236429047	1.04E-07
3h	ACA1_384850/clean/scf7180000084730:115947-116056:-	ACA1_384850	Arrestin_C domain-containing protein [Source:UniProtKB/TrEMBL;Acc:L8H8B9]	0.006600793	0.243009733	0.23640894	2.78E-05
3h	ACA1_058730/clean/scf7180000084599:365546-365626:+	ACA1_058730	Tetratricopeptide repeat domain containing protein [Source:UniProtKB/TrEMBL;Acc:L8GWA7]	0.113763367	0.350141667	0.2363783	0.00668548
3h	ACA1_308650/clean/scf7180000083743:14205-14268:-	ACA1_308650	hypothetical protein	0	0.236373	0.236373	2.44E-05
3h	ACA1_393520/clean/scf7180000084756:61847-61939:-	ACA1_393520	Carbohydrate-binding family 6 protein [Source:UniProtKB/TrEMBL;Acc:L8H2R4]	0.011494267	0.247806667	0.2363124	0.002006743
3h	ACA1_245510/clean/scf7180000084763:500517-500605:+	ACA1_245510	SAM domain (Sterile alpha motif) domain containing protein [Source:UniProtKB/TrEMBL;Acc:L8GKU3]	0.001828373	0.238140667	0.236312293	2.39E-05
3h	ACA1_063250/clean/scf7180000084599:1365496-1365635:-	ACA1_063250	MYND-type domain-containing protein [Source:UniProtKB/TrEMBL;Acc:L8GX85]	0.025044167	0.261145667	0.2361015	7.58E-05
3h	ACA1_159190/clean/scf7180000084724:357570-357632:-	ACA1_159190	hypothetical protein	0.040120233	0.276053667	0.235933433	0.00103947
3h	ACA1_264840/clean/scf7180000084770:386774-386842:-	ACA1_264840	Leucine rich repeat domain containing protein [Source:UniProtKB/TrEMBL;Acc:L8H241]	0.017736333	0.253657133	0.2359208	3.00E-06
3h	ACA1_125950/clean/scf7180000084693:7440-7557:+	ACA1_125950	AB hydrolase-1 domain-containing protein [Source:UniProtKB/TrEMBL;Acc:L8HCR9]	0.0227126	0.258570633	0.235858033	0.001593575
3h	ACA1_224860/clean/scf7180000084760:258576-258676:+	ACA1_224860	Myosin head (Motor domain) domain containing protein [Source:UniProtKB/TrEMBL;Acc:L8GSY9]	0	0.235772733	0.235772733	0.012792796
3h	ACA1_063620/clean/scf7180000084599:1463003-1463063:-	ACA1_063620	Hydroxysteroid dehydrogenase, putative [Source:UniProtKB/TrEMBL;Acc:L8GYL0]	0.000974917	0.236743867	0.23576895	1.82E-07
3h	ACA1_220760/clean/scf7180000084758:962215-962298:-	ACA1_220760	Methyltransf_11 domain-containing protein [Source:UniProtKB/TrEMBL;Acc:L8GQP4]	0.00184454	0.237518	0.23567346	2.06E-09
3h	ACA1_161750/clean/scf7180000084726:20239-20320:-	ACA1_161750	hypothetical protein	0.002086694	0.237643567	0.235556873	0.000127804
3h	ACA1_396930/clean/scf7180000084759:151004-151197:+	ACA1_396930	hypothetical protein	0.01530488	0.250732233	0.235427353	4.26E-05
3h	ACA1_063830/clean/scf7180000084599:1515478-1515547:+	ACA1_063830	hypothetical protein	0.0369709	0.27238	0.2354091	0.00354484
3h	ACA1_195060/clean/scf7180000084751:63894-63982:-	ACA1_195060	hypothetical protein	0.000571981	0.235938	0.235366019	5.88E-09
3h	ACA1_363780/clean/scf7180000084641:12760-12852:+	ACA1_363780	Vtype ATPase, D subunit [Source:UniProtKB/TrEMBL;Acc:L8GM99]	0.0004232	0.235738067	0.235314866	1.07E-08
3h	ACA1_396280/clean/scf7180000084759:71081-71220:-	ACA1_396280	FHA domain-containing protein [Source:UniProtKB/TrEMBL;Acc:L8HC78]	0.014184507	0.249309	0.235124493	3.47E-05
3h	ACA1_214980/clean/scf7180000084758:284495-284566:+	ACA1_214980	HAD hydrolase, family IA, variant 3 subfamily protein [Source:UniProtKB/TrEMBL;Acc:L8GPI3]	0.004856337	0.239977	0.235120663	9.01E-05
3h	ACA1_330890/clean/scf7180000084566:73864-73954:+	ACA1_330890	hypothetical protein	0	0.2349914	0.2349914	8.29E-09
3h	ACA1_329680/clean/scf7180000084562:48523-48633:-	ACA1_329680	Glyco_hydro_15 domain-containing protein [Source:UniProtKB/TrEMBL;Acc:L8GHW7]	0.007575767	0.242549667	0.2349739	0.000285678
3h	ACA1_063780/clean/scf7180000084599:1501252-1501973:+	ACA1_063780	Nucleotidyltransferase domain containing protein [Source:UniProtKB/TrEMBL;Acc:L8GWN7]	0.035735167	0.270460333	0.234725167	0.000485706
3h	ACA1_224410/clean/scf7180000084760:196339-196416:+	ACA1_224410	hypothetical protein	0.098156867	0.332818667	0.2346618	0.017838322
3h	ACA1_103190/clean/scf7180000084653:17287-17372:-	ACA1_103190	RING-type domain-containing protein [Source:UniProtKB/TrEMBL;Acc:L8GD19]	0.001874917	0.236526533	0.234651617	6.13E-06
3h	ACA1_158940/clean/scf7180000084724:300917-301000:+	ACA1_158940	Oxidoreductase [Source:UniProtKB/TrEMBL;Acc:L8HBL7]	0	0.234636333	0.234636333	3.27E-06
3h	ACA1_012310/clean/scf7180000083740:11735-11809:+	ACA1_012310	Biogenesis of lysosome related family protein [Source:UniProtKB/TrEMBL;Acc:L8HEJ0]	0.006537849	0.241130267	0.234592417	7.14E-07
3h	ACA1_215030/clean/scf7180000084758:298667-298753:-	ACA1_215030	hypothetical protein	0.011525433	0.246013333	0.2344879	0.00056739
3h	ACA1_009890/clean/scf7180000083468:244-338:-	ACA1_009890	TPR_REGION domain-containing protein [Source:UniProtKB/TrEMBL;Acc:L8GDE6]	0.0293154	0.263783667	0.234468267	0.000128315
3h	ACA1_087450/clean/scf7180000084639:32564-32636:-	ACA1_087450	Ras subfamily protein [Source:UniProtKB/TrEMBL;Acc:L8GVE7]	0.004545467	0.238925	0.234379533	0.000230383
3h	ACA1_017740/clean/scf7180000083876:7593-7682:+	ACA1_017740	5'-3' exoribonuclease 1 [Source:UniProtKB/TrEMBL;Acc:L8GMR4]	0.002830849	0.237197667	0.234366818	1.08E-07
3h	ACA1_097600/clean/scf7180000084645:1293231-1293503:+	ACA1_097600	Peroxidase [Source:UniProtKB/TrEMBL;Acc:L8GJT1]	0.004152737	0.238483667	0.23433093	1.05E-09
3h	ACA1_278000/clean/scf7180000084776:151758-151867:+	ACA1_278000	Carrier superfamily protein [Source:UniProtKB/TrEMBL;Acc:L8H5S3]	0.004202733	0.238300467	0.234097733	0.00808768
3h	ACA1_267240/clean/scf7180000084770:835609-835730:-	ACA1_267240	UBIQUITIN_CONJUGAT_2 domain-containing protein [Source:UniProtKB/TrEMBL;Acc:L8H2K7]	0.016604067	0.250685433	0.234081367	1.89E-05
3h	ACA1_176140/clean/scf7180000084741:1131684-1131770:+	ACA1_176140	PH domain-containing protein [Source:UniProtKB/TrEMBL;Acc:L8HKL1]	0.004617327	0.238680467	0.23406314	6.98E-08
3h	ACA1_376240/clean/scf7180000084685:62876-62949:+	ACA1_376240	VWFA domain-containing protein [Source:UniProtKB/TrEMBL;Acc:L8HF89]	0.00665867	0.24072	0.23406133	1.45E-08
3h	ACA1_091330/clean/scf7180000084645:88949-89043:-	ACA1_091330	Pyruvate kinase [Source:UniProtKB/TrEMBL;Acc:L8GIR3]	0.086700867	0.320709667	0.2340088	0.016581491
3h	ACA1_064570/clean/scf7180000084599:1645234-1645354:+	ACA1_064570	Ras subfamily protein [Source:UniProtKB/TrEMBL;Acc:L8GXF9]	0	0.2340037	0.2340037	0.000118769
3h	ACA1_271730/clean/scf7180000084771:94324-94476:-	ACA1_271730	t-SNARE coiled-coil homology domain-containing protein [Source:UniProtKB/TrEMBL;Acc:L8GP14]	0.012520757	0.246492467	0.23397171	3.72E-06
3h	ACA1_149150/clean/scf7180000084715:84401-84672:-	ACA1_149150	GATA-type domain-containing protein [Source:UniProtKB/TrEMBL;Acc:L8HBX6]	0.0201928	0.2539995	0.2338067	1.82E-05
3h	ACA1_320790/clean/scf7180000084416:22475-22607:-	ACA1_320790	RUN domain-containing protein [Source:UniProtKB/TrEMBL;Acc:L8GTR9]	0.00013242	0.2338868	0.23375438	4.92E-07
3h	ACA1_251640/clean/scf7180000084768:101379-101457:+	ACA1_251640	CBS domain-containing protein [Source:UniProtKB/TrEMBL;Acc:L8HAQ8]	0.007450867	0.241174567	0.2337237	6.74E-05
3h	ACA1_203950/clean/scf7180000084754:229067-229250:+	ACA1_203950	PAS domain Sbox domain containing protein [Source:UniProtKB/TrEMBL;Acc:L8GTK3]	0.00522294	0.2388193	0.23359636	5.43E-06
3h	ACA1_145850/clean/scf7180000084710:168824-168900:-	ACA1_145850	O-methyltransferase, putative [Source:UniProtKB/TrEMBL;Acc:L8GFG9]	0.00152617	0.235107133	0.233580963	2.10E-05
3h	ACA1_233420/clean/scf7180000084762:138193-138260:+	ACA1_233420	hypothetical protein	0.005652661	0.2392171	0.233564439	1.66E-07
3h	ACA1_189420/clean/scf7180000084748:32169-32357:+	ACA1_189420	Protein disulfideisomerase domain containing protein [Source:UniProtKB/TrEMBL;Acc:L8GE28]	0.002999187	0.2365256	0.233526413	2.20E-07
3h	ACA1_074160/clean/scf7180000084605:38774-38875:+	ACA1_074160	Aamy domain-containing protein [Source:UniProtKB/TrEMBL;Acc:L8HJ75]	0.003423183	0.236920333	0.23349715	5.53E-06
3h	ACA1_172150/clean/scf7180000084741:257238-257310:+	ACA1_172150	Outer membrane autotransporter, putative [Source:UniProtKB/TrEMBL;Acc:L8HHJ4]	0.032303033	0.265694333	0.2333913	0.004354676
3h	ACA1_024220/clean/scf7180000084441:19818-19927:+	ACA1_024220	Purinergic receptor, putative [Source:UniProtKB/TrEMBL;Acc:L8GLB7]	0	0.2333028	0.2333028	2.32E-09
3h	ACA1_074760/clean/scf7180000084608:77793-77865:+	ACA1_074760	MPN domain-containing protein [Source:UniProtKB/TrEMBL;Acc:L8HEE3]	0.000924721	0.2342243	0.233299579	3.93E-08
3h	ACA1_352050/clean/scf7180000084695:42556-42645:+	ACA1_352050	Succinate dehydrogenase [ubiquinone] iron-sulfur subunit, mitochondrial [Source:UniProtKB/TrEMBL;Acc:L8GDM2]	0	0.233261633	0.233261633	0.000310718
3h	ACA1_265340/clean/scf7180000084770:447389-447481:+	ACA1_265340	Protein kinase domain containing protein [Source:UniProtKB/TrEMBL;Acc:L8H258]	0	0.233234333	0.233234333	3.26E-06
3h	ACA1_285540/clean/scf7180000084776:990208-990279:-	ACA1_285540	GPS domain-containing protein [Source:UniProtKB/TrEMBL;Acc:L8H7T4]	0	0.2332341	0.2332341	7.54E-05
3h	ACA1_022710/clean/scf7180000084294:25718-25833:+	ACA1_022710	Vacuolar proton pump subunit B [Source:UniProtKB/TrEMBL;Acc:L8H9V1]	0.000585061	0.233795833	0.233210772	4.26E-17
3h	ACA1_373550/clean/scf7180000084679:97640-97723:-	ACA1_373550	CBS domain containing protein [Source:UniProtKB/TrEMBL;Acc:L8GH26]	0	0.233177333	0.233177333	0.002486607
3h	ACA1_182840/clean/scf7180000084745:261631-261713:-	ACA1_182840	MATE efflux family subfamily protein [Source:UniProtKB/TrEMBL;Acc:L8H859]	0.003760233	0.236838367	0.233078133	0.00025523
3h	ACA1_162370/clean/scf7180000084726:42852-42929:-	ACA1_162370	Protein kinase domain-containing protein [Source:UniProtKB/TrEMBL;Acc:L8GYN7]	0.015680023	0.248694933	0.23301491	5.60E-05
3h	ACA1_384710/clean/scf7180000084730:91658-91743:+	ACA1_384710	Transmembrane receptor family protein [Source:UniProtKB/TrEMBL;Acc:L8HBG4]	0.00523254	0.2381943	0.23296176	1.73E-07
3h	ACA1_290810/clean/scf7180000084777:679686-679795:+	ACA1_290810	SprT-like domain-containing protein [Source:UniProtKB/TrEMBL;Acc:L8HI88]	0.032082833	0.265020333	0.2329375	0.005349594
3h	ACA1_133480/clean/scf7180000084701:64015-64075:+	ACA1_133480	hypothetical protein	0.008691896	0.241599333	0.232907437	2.84E-06
3h	ACA1_230570/clean/scf7180000084761:381092-381163:-	ACA1_230570	DUF4139 domain-containing protein [Source:UniProtKB/TrEMBL;Acc:L8H928]	0	0.232905667	0.232905667	9.30E-07
3h	ACA1_074540/clean/scf7180000084608:38041-38115:+	ACA1_074540	C2 calcium/lipidbinding (CaLB) region-containing protein [Source:UniProtKB/TrEMBL;Acc:L8HF58]	0	0.232894667	0.232894667	9.07E-06
3h	ACA1_151330/clean/scf7180000084716:105292-105400:-	ACA1_151330	Purple acid phosphatase [Source:UniProtKB/TrEMBL;Acc:L8H0Q3]	0.002653753	0.235422033	0.23276828	3.99E-06
3h	ACA1_210390/clean/scf7180000084758:36988-37080:+	ACA1_210390	hypothetical protein	0.004510464	0.2370603	0.232549837	4.72E-07
3h	ACA1_298640/clean/scf7180000082992:7868-7973:+	ACA1_298640	V-type proton ATPase subunit C [Source:UniProtKB/TrEMBL;Acc:L8H2N9]	0.000749209	0.233297267	0.232548057	4.78E-09
3h	ACA1_257980/clean/scf7180000084769:193810-193929:+	ACA1_257980	5oxoprolinase [Source:UniProtKB/TrEMBL;Acc:L8GF06]	0.00855727	0.240944633	0.232387363	5.06E-06
3h	ACA1_169580/clean/scf7180000084739:44873-44959:+	ACA1_169580	hypothetical protein	0.00383025	0.236217533	0.232387283	6.39E-05
3h	ACA1_201170/clean/scf7180000084753:498391-498570:+	ACA1_201170	RWD domain-containing protein [Source:UniProtKB/TrEMBL;Acc:L8H2L8]	0.005710363	0.237994	0.232283637	0.000112003
3h	ACA1_365090/clean/scf7180000084641:235057-235120:+	ACA1_365090	ADPribosylglycohydrolase superfamily protein [Source:UniProtKB/TrEMBL;Acc:L8GP97]	0.035903733	0.26807	0.232166267	0.000315678
3h	ACA1_110060/clean/scf7180000084663:47698-47762:-	ACA1_110060	Initiation factor, subunit 2 family protein [Source:UniProtKB/TrEMBL;Acc:L8HHD7]	0.032596333	0.264757667	0.232161333	3.44E-05
3h	ACA1_067410/clean/scf7180000084601:20097-20177:-	ACA1_067410	F-box domain-containing protein [Source:UniProtKB/TrEMBL;Acc:L8GTP9]	0.001684567	0.233840567	0.232156	4.26E-05
3h	ACA1_087400/clean/scf7180000084639:24026-24114:+	ACA1_087400	Ras subfamily protein [Source:UniProtKB/TrEMBL;Acc:L8GVE2]	0.0448094	0.276837	0.2320276	0.029757136
3h	ACA1_164040/clean/scf7180000084729:145873-145971:-	ACA1_164040	Signal peptide binding domain containing protein [Source:UniProtKB/TrEMBL;Acc:L8GRG0]	0.004789965	0.236801167	0.232011202	0.001136646
3h	ACA1_143940/clean/scf7180000084709:109576-109693:-	ACA1_143940	VWFA domain-containing protein [Source:UniProtKB/TrEMBL;Acc:L8HEQ6]	0.043195713	0.275105967	0.231910253	0.004243104
3h	ACA1_151110/clean/scf7180000084716:63070-63174:+	ACA1_151110	Sec23/Sec24 trunk domain containing protein [Source:UniProtKB/TrEMBL;Acc:L8GZW6]	0.014863087	0.246640867	0.23177778	6.10E-05
3h	ACA1_171900/clean/scf7180000084741:195084-195281:+	ACA1_171900	ATPase/histidine kinase/DNA gyrase B/HSP90 domain containing protein [Source:UniProtKB/TrEMBL;Acc:L8HJR6]	0.032865333	0.2644491	0.231583767	0.009830062
3h	ACA1_175300/clean/scf7180000084741:918536-918632:+	ACA1_175300	Zinc knuckle domain containing protein [Source:UniProtKB/TrEMBL;Acc:L8HKB8]	0.019714333	0.251279	0.231564667	0.002598358
3h	ACA1_228740/clean/scf7180000084761:203945-204056:-	ACA1_228740	hypothetical protein	0.042319067	0.273804433	0.231485367	0.000366215
3h	ACA1_054820/clean/scf7180000084594:583336-583439:-	ACA1_054820	Tetratricopeptide repeat domain containing protein [Source:UniProtKB/TrEMBL;Acc:L8H8E7]	0	0.2314494	0.2314494	2.87E-05
3h	ACA1_088960/clean/scf7180000084639:274127-274211:-	ACA1_088960	Beta-lactamase domain-containing protein [Source:UniProtKB/TrEMBL;Acc:L8GU04]	0.000374953	0.231816367	0.231441413	0.000239322
3h	ACA1_109770/clean/scf7180000084663:21117-21357:+	ACA1_109770	WD40 repeat-containing protein [Source:UniProtKB/TrEMBL;Acc:L8HKT6]	0.00595426	0.237387	0.23143274	6.38E-06
3h	ACA1_171980/clean/scf7180000084741:213427-213522:-	ACA1_171980	Ras subfamily protein [Source:UniProtKB/TrEMBL;Acc:L8HGG9]	0.023336867	0.2547019	0.231365033	0.004555321
3h	ACA1_334660/clean/scf7180000084586:3237-3330:+	ACA1_334660	hypothetical protein	0.004076453	0.235420867	0.231344413	8.91E-08
3h	ACA1_260080/clean/scf7180000084769:419129-419213:-	ACA1_260080	GAMMA CA3 (GAMMA CARBONIC ANHYDRASE 3), putative [Source:UniProtKB/TrEMBL;Acc:L8GFM8]	0.002595803	0.2338716	0.231275797	2.45E-09
3h	ACA1_368190/clean/scf7180000084664:74887-74970:+	ACA1_368190	hypothetical protein	0.003577753	0.2347473	0.231169547	1.73E-07
3h	ACA1_116960/clean/scf7180000084669:882717-882794:+	ACA1_116960	hypothetical protein	0.018357833	0.249326667	0.230968833	9.86E-06
3h	ACA1_275570/clean/scf7180000084774:43520-43585:-	ACA1_275570	unspecified product	0.00379325	0.2344498	0.23065655	3.57E-06
3h	ACA1_279550/clean/scf7180000084776:413156-413262:-	ACA1_279550	CBS domain containing protein [Source:UniProtKB/TrEMBL;Acc:L8H8C1]	0.02145097	0.252084333	0.230633363	0.000159739

3h	ACA1_169760/clean/scf7180000084739:62620-62697:+	ACA1_169760	OGFr_N domain-containing protein [Source:UniProtKB/TrEMBL;Acc:L8HHU5]	0.0238084	0.254378433	0.230570033	0.002970825
3h	ACA1_029510/clean/scf7180000084477:1318-1464:-	ACA1_029510	Leucine rich repeat containing protein [Source:UniProtKB/TrEMBL;Acc:L8H9E3]	0.001845813	0.232280733	0.23043492	0.000100795
3h	ACA1_345120/clean/scf7180000084655:9326-9411:-	ACA1_345120	ANK_REP_REGION domain-containing protein [Source:UniProtKB/TrEMBL;Acc:L8HEX4]	0.020915033	0.251286467	0.230371433	0.00520998
3h	ACA1_325500/clean/scf7180000084495:21457-21542:+	ACA1_325500	Usp domain-containing protein [Source:UniProtKB/TrEMBL;Acc:L8HB68]	0.003163397	0.233462167	0.230298769	2.98E-06
3h	ACA1_100480/clean/scf7180000084648:93704-93778:-	ACA1_100480	Fbox/LRR-repeat protein [Source:UniProtKB/TrEMBL;Acc:L8GLX9]	0	0.2302297	0.2302297	0.000141934
3h	ACA1_027050/clean/scf7180000084464:32148-32219:-	ACA1_027050	hypothetical protein	0.003291	0.233513767	0.230222767	6.35E-07
3h	ACA1_364760/clean/scf7180000084641:192031-192317:+	ACA1_364760	Endonuclease [Source:UniProtKB/TrEMBL;Acc:L8GMG4]	0.006625173	0.2367	0.230074827	5.44E-10
3h	ACA1_260180/clean/scf7180000084769:453080-453182:+	ACA1_260180	Nitric oxide synthase-interacting protein homolog [Source:UniProtKB/TrEMBL;Acc:L8GFN7]	0.0356805	0.265742467	0.230061967	0.001295648
3h	ACA1_001860/clean/scf7180000082867:4468-4561:+	ACA1_001860	hypothetical protein	0.021394353	0.251411333	0.23001698	0.000689829
3h	ACA1_310790/clean/scf7180000083780:15281-15349:+	ACA1_310790	hypothetical protein	0.004991533	0.2349899	0.229998367	2.40E-05
3h	ACA1_166960/clean/scf7180000084736:39283-39384:+	ACA1_166960	Rab-GAP TBC domain-containing protein [Source:UniProtKB/TrEMBL;Acc:L8H338]	0.007817687	0.2378011	0.229983413	5.79E-05
3h	ACA1_063230/clean/scf7180000084599:1361746-1361840:-	ACA1_063230	Flagellar associated protein [Source:UniProtKB/TrEMBL;Acc:L8GWG8]	0.021552367	0.251489533	0.229937167	0.001169466
3h	ACA1_384640/clean/scf7180000084730:71389-71489:-	ACA1_384640	RasGEF domain containing protein [Source:UniProtKB/TrEMBL;Acc:L8H8A0]	0.112719867	0.342567667	0.2298478	0.021705337
3h	ACA1_147520/clean/scf7180000084714:29562-29653:+	ACA1_147520	RanBP2-type domain-containing protein [Source:UniProtKB/TrEMBL;Acc:L8GKJ7]	0.000684463	0.230312667	0.229628203	0.000129725
3h	ACA1_087350/clean/scf7180000084639:12629-12707:+	ACA1_087350	Protein prenyltransferase, putative [Source:UniProtKB/TrEMBL;Acc:L8GVD3]	0	0.229512667	0.229512667	0.000543303
3h	ACA1_061200/clean/scf7180000084599:851578-851674:+	ACA1_061200	5' nucleotidase family protein [Source:UniProtKB/TrEMBL;Acc:L8GWQ7]	0.043787167	0.2732962	0.229509033	0.00051782
3h	ACA1_277380/clean/scf7180000084776:19059-19142:-	ACA1_277380	hypothetical protein	0.01297235	0.242433	0.22946065	0.000405631
3h	ACA1_396220/clean/scf7180000084759:58945-59024:+	ACA1_396220	Acyl-coenzyme A oxidase [Source:UniProtKB/TrEMBL;Acc:L8HEJ7]	0.017151233	0.246611	0.229459767	1.10E-06
3h	ACA1_050360/clean/scf7180000084589:78405-78510:-	ACA1_050360	Metalloenzyme domain-containing protein [Source:UniProtKB/TrEMBL;Acc:L8H565]	0.00787969	0.237242	0.22936231	2.24E-06
3h	ACA1_184610/clean/scf7180000084746:8866-8946:+	ACA1_184610	Protein kinase domain containing protein [Source:UniProtKB/TrEMBL;Acc:L8H456]	0.004166667	0.233417833	0.229251167	4.89E-05
3h	ACA1_074550/clean/scf7180000084608:43049-43119:-	ACA1_074550	zf-C2HC5 domain-containing protein [Source:UniProtKB/TrEMBL;Acc:L8HGP3]	0	0.2292256	0.2292256	0.000784197
3h	ACA1_043190/clean/scf7180000084573:137290-137393:-	ACA1_043190	hypothetical protein	0.01626313	0.245401167	0.229138037	1.48E-06
3h	ACA1_295530/clean/scf7180000084777:1166746-1166838:+	ACA1_295530	RhoGEF domain containing protein [Source:UniProtKB/TrEMBL;Acc:L8HJT0]	0.007563037	0.236693	0.229129963	1.82E-05
3h	ACA1_184380/clean/scf7180000084745:631246-631358:-	ACA1_184380	Galactose binding lectin domain containing protein [Source:UniProtKB/TrEMBL;Acc:L8HAP7]	0.012327927	0.241426	0.229098073	1.74E-06
3h	ACA1_074760/clean/scf7180000084608:78177-78256:+	ACA1_074760	MPN domain-containing protein [Source:UniProtKB/TrEMBL;Acc:L8HEE3]	0.001928931	0.231025267	0.229096336	7.09E-11
3h	ACA1_360170/clean/scf7180000084622:63663-63746:-	ACA1_360170	Ras subfamily protein [Source:UniProtKB/TrEMBL;Acc:L8HCN9]	0	0.229095167	0.229095167	1.99E-07
3h	ACA1_391420/clean/scf7180000084740:169792-169865:+	ACA1_391420	hypothetical protein	0.00154321	0.230479633	0.228936423	2.28E-06
3h	ACA1_061700/clean/scf7180000084599:1013591-1013685:+	ACA1_061700	Amine oxidase family protein [Source:UniProtKB/TrEMBL;Acc:L8GWP7]	0.004193897	0.2331198	0.228925903	6.24E-05
3h	ACA1_253490/clean/scf7180000084768:315380-315439:+	ACA1_253490	hypothetical protein	0.00945663	0.2383523	0.22889567	0.00010943
3h	ACA1_062680/clean/scf7180000084599:1272561-1272678:+	ACA1_062680	hypothetical protein	0.020674333	0.249521333	0.228847	0.000100542
3h	ACA1_066030/clean/scf7180000084599:1976212-1976295:-	ACA1_066030	hypothetical protein	0.007430333	0.236252667	0.228822333	4.31E-05
3h	ACA1_389360/clean/scf7180000084733:237831-237972:-	ACA1_389360	hypothetical protein	0.094120133	0.32279	0.228669867	0.014067736
3h	ACA1_395480/clean/scf7180000084756:189615-189726:-	ACA1_395480	Copine subfamily protein [Source:UniProtKB/TrEMBL;Acc:L8H0H7]	0.017031867	0.245646	0.228614133	0.000140474
3h	ACA1_125070/clean/scf7180000084691:24161-24243:-	ACA1_125070	F-box domain-containing protein [Source:UniProtKB/TrEMBL;Acc:L8GNN0]	0	0.228584667	0.228584667	0.00150366
3h	ACA1_226760/clean/scf7180000084761:72858-72949:+	ACA1_226760	hypothetical protein	0.004766097	0.233244367	0.22847827	0.000463892
3h	ACA1_219760/clean/scf7180000084758:803370-803486:+	ACA1_219760	hypothetical protein	0	0.228417833	0.228417833	4.28E-06
3h	ACA1_163820/clean/scf7180000084729:75983-76079:-	ACA1_163820	MFS domain-containing protein [Source:UniProtKB/TrEMBL;Acc:L8GQW1]	0.0673047	0.2956147	0.22831	0.031268552
3h	ACA1_054820/clean/scf7180000084594:580257-580366:-	ACA1_054820	Tetratricopeptide repeat domain containing protein [Source:UniProtKB/TrEMBL;Acc:L8H8E7]	0.016260167	0.244443233	0.228183067	0.001915397
3h	ACA1_240180/clean/scf7180000084763:203314-203412:+	ACA1_240180	WD_REPEATS_REGION domain-containing protein [Source:UniProtKB/TrEMBL;Acc:L8GM97]	0.0513065	0.279451333	0.228144833	0.003464891
3h	ACA1_011280/clean/scf7180000083623:8736-8822:+	ACA1_011280	AAA domain-containing protein [Source:UniProtKB/TrEMBL;Acc:L8GP61]	0.00425011	0.232371067	0.228120957	2.67E-06
3h	ACA1_063340/clean/scf7180000084599:1391374-1391478:+	ACA1_063340	Dynein intermediate chain, cytosolic, putative [Source:UniProtKB/TrEMBL;Acc:L8GX48]	0.001524513	0.229638	0.228113487	1.04E-07
3h	ACA1_262530/clean/scf7180000084770:55300-55440:+	ACA1_262530	SH3 domain-containing protein [Source:UniProtKB/TrEMBL;Acc:L8H1T4]	0.012949953	0.240928067	0.227978113	1.81E-06
3h	ACA1_285540/clean/scf7180000084776:991050-991118:-	ACA1_285540	GPS domain-containing protein [Source:UniProtKB/TrEMBL;Acc:L8H7T4]	0.016666667	0.2446195	0.227952833	0.006836067
3h	ACA1_369750/clean/scf7180000084664:373493-373566:+	ACA1_369750	RRM domain-containing protein [Source:UniProtKB/TrEMBL;Acc:L8GZ60]	0.02002228	0.247970667	0.227948387	6.76E-05
3h	ACA1_088830/clean/scf7180000084639:241298-241362:-	ACA1_088830	Isochorismatase domain-containing protein [Source:UniProtKB/TrEMBL;Acc:L8GUP3]	0.023238633	0.2511451	0.227906467	0.000672849
3h	ACA1_331780/clean/scf7180000084578:26691-26784:-	ACA1_331780	BRCA1 C Terminus (BRCT) domain containing protein [Source:UniProtKB/TrEMBL;Acc:L8HE43]	0.006606339	0.234446667	0.227840328	2.23E-05
3h	ACA1_056540/clean/scf7180000084597:15291-15388:+	ACA1_056540	B30.2/SPRY domain-containing protein [Source:UniProtKB/TrEMBL;Acc:L8HGP5]	0.041666667	0.269496433	0.227829767	0.046631429
3h	ACA1_329830/clean/scf7180000084566:6955-7068:-	ACA1_329830	hypothetical protein	0.01134822	0.239021533	0.227673313	0.000161098
3h	ACA1_230570/clean/scf7180000084761:379087-379158:-	ACA1_230570	DUF4139 domain-containing protein [Source:UniProtKB/TrEMBL;Acc:L8H928]	0.009394057	0.237065367	0.22767131	9.64E-06
3h	ACA1_399960/clean/scf7180000084775:82256-82354:-	ACA1_399960	WD_REPEATS_REGION domain-containing protein [Source:UniProtKB/TrEMBL;Acc:L8GID8]	0.01753845	0.2450953	0.22755685	0.000156324
3h	ACA1_158530/clean/scf7180000084724:245492-245610:+	ACA1_158530	Glucose-6-phosphate 1-dehydrogenase [Source:UniProtKB/TrEMBL;Acc:L8HBJ1]	0.07630976	0.303757767	0.227448007	0.035802772
3h	ACA1_203070/clean/scf7180000084754:136181-136266:+	ACA1_203070	Fbox domain containing protein [Source:UniProtKB/TrEMBL;Acc:L8GUJ5]	0.013614507	0.241043633	0.227429127	0.000107323
3h	ACA1_291510/clean/scf7180000084777:765863-766043:+	ACA1_291510	Ubiquitin carboxylterminal hydrolase 15, putative [Source:UniProtKB/TrEMBL;Acc:L8HKZ1]	0.019409593	0.246795467	0.227385873	0.000224405
3h	ACA1_068460/clean/scf7180000084603:206309-206423:-	ACA1_068460	Leucinerich repeat kinase [Source:UniProtKB/TrEMBL;Acc:L8HDF4]	0.002283107	0.229565	0.227281893	1.20E-05
3h	ACA1_136780/clean/scf7180000084703:6818-6911:+	ACA1_136780	hypothetical protein	0.0158694	0.243149667	0.227280267	0.000223012
3h	ACA1_052720/clean/scf7180000084594:162593-162692:+	ACA1_052720	hypothetical protein	0.010874833	0.238128267	0.227253433	0.008516336
3h	ACA1_282190/clean/scf7180000084776:622904-623031:-	ACA1_282190	Synapsin, ATP binding domain containing protein [Source:UniProtKB/TrEMBL;Acc:L8H7E1]	0.007211933	0.234129733	0.2269178	0.000113426
3h	ACA1_133480/clean/scf7180000084701:64173-64234:+	ACA1_133480	hypothetical protein	0.009728249	0.2365583	0.226830051	1.01E-05
3h	ACA1_377310/clean/scf7180000084711:24280-24378:-	ACA1_377310	Translocase, putative [Source:UniProtKB/TrEMBL;Acc:L8GRV0]	0	0.226824667	0.226824667	0.005427844
3h	ACA1_127420/clean/scf7180000084696:18737-19121:-	ACA1_127420	Aconitase domain-containing protein [Source:UniProtKB/TrEMBL;Acc:L8GGD8]	0.011223817	0.238019333	0.226795517	3.38E-07
3h	ACA1_111310/clean/scf7180000084669:2756-2851:+	ACA1_111310	hypothetical protein	0.0192901	0.246082	0.2267919	0.001794509
3h	ACA1_291510/clean/scf7180000084777:764425-764495:+	ACA1_291510	Ubiquitin carboxylterminal hydrolase 15, putative [Source:UniProtKB/TrEMBL;Acc:L8HKZ1]	0.001851853	0.228360267	0.226508413	1.75E-06
3h	ACA1_189340/clean/scf7180000084748:10924-11001:-	ACA1_189340	Dyptype peroxidase superfamily protein [Source:UniProtKB/TrEMBL;Acc:L8GDU2]	0.002183543	0.228687867	0.226504324	1.13E-08
3h	ACA1_313730/clean/scf7180000083824:2271-2365:-	ACA1_313730	Glyco_trans_2-like domain-containing protein [Source:UniProtKB/TrEMBL;Acc:L8GN52]	0.004637743	0.231110333	0.22647259	3.37E-10
3h	ACA1_265500/clean/scf7180000084770:496810-496922:-	ACA1_265500	F-box domain-containing protein [Source:UniProtKB/TrEMBL;Acc:L8H275]	0	0.226357967	0.226357967	0.001283948
3h	ACA1_368920/clean/scf7180000084664:198679-198743:+	ACA1_368920	MFS domain-containing protein [Source:UniProtKB/TrEMBL;Acc:L8GYV9]	0.000196895	0.226531067	0.226334172	3.02E-06
3h	ACA1_051490/clean/scf7180000084592:22396-22472:-	ACA1_051490	Cysteine proteinase 5, putative [Source:UniProtKB/TrEMBL;Acc:L8GS11]	0.002904907	0.229082333	0.226177427	3.75E-06
3h	ACA1_381600/clean/scf7180000084712:193812-193897:+	ACA1_381600	Golgi family protein, putative [Source:UniProtKB/TrEMBL;Acc:L8GP66]	0.010160543	0.236334333	0.22617379	2.75E-05
3h	ACA1_144300/clean/scf7180000084709:197239-197314:-	ACA1_144300	RRM domain-containing protein [Source:UniProtKB/TrEMBL;Acc:L8HEU9]	0.021841767	0.24785	0.226008233	7.33E-06
3h	ACA1_260060/clean/scf7180000084769:414551-414652:+	ACA1_260060	WD_REPEATS_REGION domain-containing protein [Source:UniProtKB/TrEMBL;Acc:L8GF95]	0.0488575	0.274671733	0.225814233	0.000477113
3h	ACA1_271600/clean/scf7180000084771:81521-81609:-	ACA1_271600	Cation diffusion facilitator family transporter superfamily protein [Source:UniProtKB/TrEMBL;Acc:L8GNJ1]	0.044416333	0.270206667	0.225790333	0.003993914
3h	ACA1_042210/clean/scf7180000084573:57293-57453:+	ACA1_042210	Signal transducer and activator of transcription [Source:UniProtKB/TrEMBL;Acc:L8GXQ7]	0.005313933	0.231080033	0.2257661	2.90E-05
3h	ACA1_074130/clean/scf7180000084605:32499-32602:+	ACA1_074130	PlsC domain-containing protein [Source:UniProtKB/TrEMBL;Acc:L8HMM5]	0.016943613	0.2426327	0.225689087	0.000177842
3h	ACA1_064570/clean/scf7180000084599:1646653-1646730:+	ACA1_064570	Ras subfamily protein [Source:UniProtKB/TrEMBL;Acc:L8GXF9]	0.0150328	0.240713367	0.225680567	0.000384502
3h	ACA1_051930/clean/scf7180000084592:88419-88533:+	ACA1_051930	hypothetical protein	0.04824793	0.273850067	0.225602137	0.004497291
3h	ACA1_134270/clean/scf7180000084702:23030-23132:-	ACA1_134270	hypothetical protein	0.018225533	0.243796033	0.2255705	0.006385649
3h	ACA1_070890/clean/scf7180000084603:796399-796459:-	ACA1_070890	hypothetical protein	0.113320567	0.338639	0.225318433	0.01233259
3h	ACA1_027050/clean/scf7180000084464:31980-32064:-	ACA1_027050	hypothetical protein	0.007548417	0.2328628	0.225314383	2.44E-06
3h	ACA1_291040/clean/scf7180000084777:724568-724656:+	ACA1_291040	WRKY DNA-binding domain containing protein [Source:UniProtKB/TrEMBL;Acc:L8HLM9]	0.002076257	0.227085133	0.225008877	6.16E-05
3h	ACA1_232630/clean/scf7180000084762:50367-50502:+	ACA1_232630	FYVE-type domain-containing protein [Source:UniProtKB/TrEMBL;Acc:L8H3C0]	0.031972033	0.256957067	0.224985033	0.000551062
3h	ACA1_031950/clean/scf7180000084523:40094-40180:+	ACA1_031950	CBS domain containing protein [Source:UniProtKB/TrEMBL;Acc:L8H414]	0.007189133	0.232156633	0.2249675	0.008582762
3h	ACA1_002500/clean/scf7180000082931:8966-9042:+	ACA1_002500	hypothetical protein	0.237138333	0.462056333	0.224918	0.027214434
3h	ACA1_285540/clean/scf7180000084776:991409-991477:-	ACA1_285540	GPS domain-containing protein [Source:UniProtKB/TrEMBL;Acc:L8H7T4]	0	0.2249043	0.2249043	0.001209188
3h	ACA1_013390/clean/scf7180000083750:12035-12124:-	ACA1_013390	Aamy domain-containing protein [Source:UniProtKB/TrEMBL;Acc:L8HGK3]	0.001240437	0.225936167	0.22469573	1.55E-06
3h	ACA1_326890/clean/scf7180000084510:65625-65706:+	ACA1_326890	WD domain, Gbeta repeat-containing protein [Source:UniProtKB/TrEMBL;Acc:L8HKJ0]	0.015895617	0.240564767	0.22466915	0.001093694
3h	ACA1_385360/clean/scf7180000084730:149473-149566:-	ACA1_385360	RasGEF domain containing protein [Source:UniProtKB/TrEMBL;Acc:L8H9A3]	0.0117495	0.236265467	0.224515967	0.000206962
3h	ACA1_320830/clean/scf7180000084416:30264-30365:+	ACA1_320830	Lipoyl-binding domain-containing protein [Source:UniProtKB/TrEMBL;Acc:L8GT97]	0.000763567	0.22523	0.224466433	1.56E-11
3h	ACA1_362820/clean/scf7180000084624:188858-188981:+	ACA1_362820	hypothetical protein	0.008589267	0.2329936	0.224404333	1.24E-06
3h	ACA1_380970/clean/scf7180000084712:129728-129850:+	ACA1_380970	ANK_REP_REGION domain-containing protein [Source:UniProtKB/TrEMBL;Acc:L8GMX1]	0.001456877	0.225851367	0.22439449	3.96E-05
3h	ACA1_031600/clean/scf7180000084522:20604-20676:-	ACA1_031600	Leucine rich repeat-containing protein [Source:UniProtKB/TrEMBL;Acc:L8GSM8]	0.014447867	0.2388	0.224352133	0.000915577
3h	ACA1_096830/clean/scf7180000084645:1119849-1119971:+	ACA1_096830	Serine/threonine-protein kinase TOR [Source:UniProtKB/TrEMBL;Acc:L8GIV8]	0.021106387	0.245454	0.224347613	0.000518688
3h	ACA1_054820/clean/scf7180000084594:578519-578623:-	ACA1_054820	Tetratricopeptide repeat domain containing protein [Source:UniProtKB/TrEMBL;Acc:L8H8E7]	0.016509327	0.240754933	0.224245607	0.000285777
3h	ACA1_035450/clean/scf7180000084564:51132-51214:-	ACA1_035450	Opposite strand transcription unit to Stag3, putative [Source:UniProtKB/TrEMBL;Acc:L8HAK5]	0.042782967	0.266995667	0.2242127	0.001749794
3h	ACA1_038430/clean/scf7180000084568:147368-147430:+	ACA1_038430	hypothetical protein	0.002192934	0.226316833	0.224123899	8.04E-07
3h	ACA1_091880/clean/scf7180000084645:220745-220833:+	ACA1_091880	Ubiquitin-like domain-containing protein [Source:UniProtKB/TrEMBL;Acc:L8GIV5]	0.004316287	0.2284374	0.224121113	8.14E-07
3h	ACA1_234290/clean/scf7180000084762:262109-262218:-	ACA1_234290	Deacetylase sirtuin-type domain-containing protein [Source:UniProtKB/TrEMBL;Acc:L8H3J1]	0.135943	0.359952	0.224009	0.028727327
3h	ACA1_230310/clean/scf7180000084761:343197-343294:+	ACA1_230310	PAS domain-containing protein [Source:UniProtKB/TrEMBL;Acc:L8HBA3]	0.017984267	0.241905567	0.2239213	0.000870482
3h	ACA1_271500/clean/scf7180000084771:46888-46991:+	ACA1_271500	Leucine rich repeat domain containing protein [Source:UniProtKB/TrEMBL;Acc:L8GNI4]	0.009714833	0.233623567	0.223908733	0.000251306
3h	ACA1_229230/clean/scf7180000084761:247774-247853:-	ACA1_229230	Mitochondrial carrier protein [Source:UniProtKB/TrEMBL;Acc:L8HB72]	0.004460827	0.2282288	0.223767973	3.50E-08
3h	ACA1_220750/clean/scf7180000084758:960718-960834:-	ACA1_220750	Urease accessory protein UreF, putative [Source:UniProtKB/TrEMBL;Acc:L8GQ53]	0.01110523	0.234821567	0.223716337	0.000706532
3h	ACA1_279460/clean/scf7180000084776:388476-388553:+	ACA1_279460	KLRAQ domain-containing protein [Source:UniProtKB/TrEMBL;Acc:L8H620]	0	0.223711167	0.223711167	7.53E-08
3h	ACA1_183310/clean/scf7180000084745:377570-377665:+	ACA1_183310	hypothetical protein	0.022193407	0.245631733	0.223438327	0.000424569
3h	ACA1_078530/clean/scf7180000084615:39057-39142:+	ACA1_078530	START domain-containing protein [Source:UniProtKB/TrEMBL;Acc:L8GUK7]	0.002334533	0.225657867	0.223323333	2.29E-07
3h	ACA1_232800/clean/scf7180000084762:70416-70514:-	ACA1_232800	M20_dimer domain-containing protein [Source:UniProtKB/TrEMBL;Acc:L8H2E1]	0.028854967	0.2521694	0.223314433	0.008670904
3h	ACA1_144360/clean/scf7180000084709:215515-215590:+	ACA1_144360	Repressor of RNA polymerase III transcription [Source:UniProtKB/TrEMBL;Acc:L8HFX0]	0.011077797	0.234179733	0.223101937	1.12E-05
3h	ACA1_100610/clean/scf7180000084648:129276-129364:-	ACA1_100610	Serine/threonine kinase [Source:UniProtKB/TrEMBL;Acc:L8GK64]	0.005186878	0.228282367	0.223095489	1.20E-05
3h	ACA1_064210/clean/scf7180000084599:1560661-1560743:+	ACA1_064210	Phospholipase D active site domain containing protein [Source:UniProtKB/TrEMBL;Acc:L8GXI1]	0.0085034	0.231578	0.2230746	1.06E-06
3h	ACA1_075030/clean/scf7180000084608:129768-129895:+	ACA1_075030	Serine carboxypeptidase [Source:UniProtKB/TrEMBL;Acc:L8HFJ6]	0.002402507	0.225318033	0.222915527	4.06E-06
3h	ACA1_382380/clean/scf7180000084721:46296-46394:+	ACA1_382380	WD_REPEATS_REGION domain-containing protein [Source:UniProtKB/TrEMBL;Acc:L8GUA6]	0.0370154	0.259889367	0.222873967	0.004640419
3h	ACA1_199600/clean/scf7180000084753:272765-272877:+	ACA1_199600	PH domain-containing protein [Source:UniProtKB/TrEMBL;Acc:L8H366]	0.006822207	0.2296469	0.222824693	2.83E-05
3h	ACA1_313730/clean/scf7180000083824:3546-3644:-	ACA1_313730	Glyco_trans_2-like domain-containing protein [Source:UniProtKB/TrEMBL;Acc:L8GN52]	4.33E-05	0.222864133	0.222820861	4.96E-13
3h	ACA1_092200/clean/scf7180000084645:296729-296809:-	ACA1_092200	RFX-type winged-helix domain-containing protein [Source:UniProtKB/TrEMBL;Acc:L8GI10]	0.040073067	0.2628177	0.222744633	0.016010805
3h	ACA1_123760/clean/scf7180000084686:56212-56306:+	ACA1_123760	Protein kinase domain-containing protein [Source:UniProtKB/TrEMBL;Acc:L8HF31]	0.011747233	0.2344767	0.222729467	0.000715105
3h	ACA1_091910/clean/scf7180000084645:224952-225044:-	ACA1_091910	NIPSNAP family protein [Source:UniProtKB/TrEMBL;Acc:L8GKH2]	0.003724907	0.226344333	0.222619427	7.22E-10
3h	ACA1_188190/clean/scf7180000084747:123121-123219:-	ACA1_188190	Calponin-homology (CH) domain-containing protein [Source:UniProtKB/TrEMBL;Acc:L8GSF5]	0.015098223	0.237708633	0.22261041	0.000176374
3h	ACA1_164660/clean/scf7180000084729:244788-244867:-	ACA1_164660	Protein tyrosine phosphatase, dual specificity, putative [Source:UniProtKB/TrEMBL;Acc:L8GU25]	0.008541059	0.231140167	0.222599108	2.48E-07
3h	ACA1_201490/clean/scf7180000084753:570715-570784:+	ACA1_201490	hypothetical protein	0.004060347	0.226639633	0.222579287	2.01E-05
3h	ACA1_035570/clean/scf7180000084564:92560-92962:-	ACA1_035570	RNAbinding protein, putative [Source:UniProtKB/TrEMBL;Acc:L8HAG2]	0.072009	0.294578	0.222569	0.016270292
3h	ACA1_318540/clean/scf7180000084158:21023-21102:+	ACA1_318540	SPX domain containing protein [Source:UniProtKB/TrEMBL;Acc:L8GH73]	0.001221263	0.223759533	0.22253827	3.06E-06
3h	ACA1_152930/clean/scf7180000084720:34320-34445:-	ACA1_152930	PG_binding_1 domain-containing protein [Source:UniProtKB/TrEMBL;Acc:L8HF34]	0.01178458	0.233818567	0.222033987	0.000294354
3h	ACA1_051530/clean/scf7180000084592:29744-29840:-	ACA1_051530	MFS domain-containing protein [Source:UniProtKB/TrEMBL;Acc:L8GPJ8]	0.00575912	0.227749	0.22198988	0.00021
3h	ACA1_106290/clean/scf7180000084660:76595-76709:+	ACA1_106290	WD_REPEATS_REGION domain-containing protein [Source:UniProtKB/TrEMBL;Acc:L8GNF9]	0.00405481	0.226022933	0.221968123	8.72E-05
3h	ACA1_278150/clean/scf7180000084776:180073-180150:-	ACA1_278150	hypothetical protein	0.009285133	0.231200467	0.221915333	8.68E-06
3h	ACA1_275720/clean/scf7180000084774:93371-93447:+	ACA1_275720	Enolase, Cterminal TIM barrel domain containing protein [Source:UniProtKB/TrEMBL;Acc:L8GRZ9]	0.005307691	0.227100333	0.221792642	1.55E-07
3h	ACA1_096600/clean/scf7180000084645:1058057-1058118:+	ACA1_096600	CUE domain containing protein [Source:UniProtKB/TrEMBL;Acc:L8GJT0]	0	0.2216866	0.2216866	4.60E-08
3h	ACA1_095270/clean/scf7180000084645:826308-826378:-	ACA1_095270	Thioredoxin-like_fold domain-containing protein [Source:UniProtKB/TrEMBL;Acc:L8GIR9]	0.003790087	0.225448933	0.221658847	6.22E-06
3h	ACA1_218620/clean/scf7180000084758:679040-679131:-	ACA1_218620	Carboxylic ester hydrolase [Source:UniProtKB/TrEMBL;Acc:L8GRD3]	0.001738944	0.223391733	0.221652789	3.99E-06
3h	ACA1_277780/clean/scf7180000084776:98942-99053:+	ACA1_277780	Ca2+dependent endoplasmic reticulum nucleoside diphosphatase isoform 3, putative [Source:UniProtKB/TrEMBL;Acc:L8H7Y3]	0.00603654	0.227665	0.22162846	0.000247638
3h	ACA1_149410/clean/scf7180000084715:148119-148214:+	ACA1_149410	Lectin_legB domain-containing protein [Source:UniProtKB/TrEMBL;Acc:L8HDD6]	0.01188301	0.233479633	0.221596623	8.65E-05
3h	ACA1_011270/clean/scf7180000083623:6307-6372:-	ACA1_011270	FYVE-type domain-containing protein [Source:UniProtKB/TrEMBL;Acc:L8GQN9]	0	0.221593033	0.221593033	0.038587373
3h	ACA1_072980/clean/scf7180000084603:1350107-1350204:-	ACA1_072980	Proteasome subunit alpha type [Source:UniProtKB/TrEMBL;Acc:L8HDS9]	0.003464027	0.225048867	0.22158484	4.10E-08
3h	ACA1_060920/clean/scf7180000084599:778912-779008:-	ACA1_060920	MFS domain-containing protein [Source:UniProtKB/TrEMBL;Acc:L8GVT5]	0.019439317	0.240999667	0.22156035	0.005521139
3h	ACA1_181250/clean/scf7180000084744:156513-156628:-	ACA1_181250	NEDD8-activating enzyme E1 regulatory subunit [Source:UniProtKB/TrEMBL;Acc:L8GCR5]	0.008080527	0.229597533	0.221517007	4.44E-07
3h	ACA1_166080/clean/scf7180000084732:115216-115293:+	ACA1_166080	hypothetical protein	0	0.221476333	0.221476333	1.12E-07
3h	ACA1_391420/clean/scf7180000084740:169579-169661:+	ACA1_391420	hypothetical protein	0.004758543	0.226119467	0.221360923	1.48E-05
3h	ACA1_002500/clean/scf7180000082931:10553-10612:+	ACA1_002500	hypothetical protein	0.002693187	0.223913667	0.22122048	2.95E-10
3h	ACA1_036270/clean/scf7180000084565:66897-66990:+	ACA1_036270	hypothetical protein	0.000931187	0.222146967	0.22121578	6.73E-05
3h	ACA1_138440/clean/scf7180000084703:140028-140089:-	ACA1_138440	hypothetical protein	0.0270144	0.248201367	0.221186967	0.00204793

3h	ACA1_061900/clean/scf7180000084599:1075231-1075468:-	ACA1_061900	GTP_EFTU_D3 domain-containing protein [Source:UniProtKB/TrEMBL;Acc:L8GY16]	0.115603	0.336771667	0.221168667	0.023617037
3h	ACA1_260030/clean/scf7180000084769:408171-408352:-	ACA1_260030	ApaG domain-containing protein [Source:UniProtKB/TrEMBL;Acc:L8GFM4]	0.0420517	0.263135	0.2210833	0.000404285
3h	ACA1_362720/clean/scf7180000084624:159129-159196:-	ACA1_362720	Neutral ceramidase [Source:UniProtKB/TrEMBL;Acc:L8GFC5]	0.010633873	0.231603133	0.22096926	0.00052319
3h	ACA1_097290/clean/scf7180000084645:1210270-1210475:+	ACA1_097290	hypothetical protein	0.006910987	0.227853367	0.22094238	4.26E-05
3h	ACA1_219480/clean/scf7180000084758:748587-748700:+	ACA1_219480	hypothetical protein	0.003875467	0.224752733	0.220877267	1.48E-05
3h	ACA1_018770/clean/scf7180000083912:14524-14591:+	ACA1_018770	Signal recognition particle subunit SRP68 [Source:UniProtKB/TrEMBL;Acc:L8HKJ5]	0.017295353	0.2381337	0.220838347	1.15E-05
3h	ACA1_065450/clean/scf7180000084599:1855516-1855590:-	ACA1_065450	Cation_ATPase_N domain-containing protein [Source:UniProtKB/TrEMBL;Acc:L8H037]	0	0.220825067	0.220825067	0.00940168
3h	ACA1_037190/clean/scf7180000084567:79244-79301:+	ACA1_037190	Aminotran_5 domain-containing protein [Source:UniProtKB/TrEMBL;Acc:L8H0V4]	0.000642515	0.221373033	0.220730518	1.08E-07
3h	ACA1_040840/clean/scf7180000084572:92100-92165:-	ACA1_040840	BRO1 domain-containing protein [Source:UniProtKB/TrEMBL;Acc:L8H1X9]	0	0.220684267	0.220684267	1.16E-07
3h	ACA1_178340/clean/scf7180000084743:222348-222490:+	ACA1_178340	RhoGEF domain containing protein [Source:UniProtKB/TrEMBL;Acc:L8GT64]	0.001336163	0.222008067	0.220671903	1.15E-05
3h	ACA1_384330/clean/scf7180000084730:28021-28119:+	ACA1_384330	hypothetical protein	0.005290693	0.225855733	0.22056504	1.04E-06
3h	ACA1_382580/clean/scf7180000084721:78043-78117:+	ACA1_382580	LBP / BPI / CETP family, Cterminal domain containing protein [Source:UniProtKB/TrEMBL;Acc:L8GVZ1]	0.005011137	0.2255747	0.220563563	9.93E-09
3h	ACA1_237270/clean/scf7180000084762:500666-500739:+	ACA1_237270	RNA 2'phosphotransferase, Tpt1/KptA domain containing protein [Source:UniProtKB/TrEMBL;Acc:L8H0V2]	0.003333333	0.223622267	0.220288933	0.000282029
3h	ACA1_149170/clean/scf7180000084715:90726-90801:+	ACA1_149170	SelT/selW/selH selenoprotein domain containing protein [Source:UniProtKB/TrEMBL;Acc:L8HB54]	0.014517493	0.234495033	0.21997754	1.02E-05
3h	ACA1_353620/clean/scf7180000084717:46515-46616:-	ACA1_353620	RhoGAP domain containing protein [Source:UniProtKB/TrEMBL;Acc:L8GGS6]	0.031023733	0.250944767	0.219921033	0.000424069
3h	ACA1_257040/clean/scf7180000084769:133041-133131:-	ACA1_257040	MaoC-like domain-containing protein [Source:UniProtKB/TrEMBL;Acc:L8GEM5]	0.003876077	0.223770667	0.21989459	3.90E-09
3h	ACA1_101560/clean/scf7180000084649:96050-96153:+	ACA1_101560	WD_REPEATS_REGION domain-containing protein [Source:UniProtKB/TrEMBL;Acc:L8GGC0]	0.001003463	0.220823333	0.219819871	3.34E-08
3h	ACA1_380810/clean/scf7180000084712:92263-92339:-	ACA1_380810	Ribosomal_L11 domain-containing protein [Source:UniProtKB/TrEMBL;Acc:L8GNK9]	0.0129484	0.232761167	0.219812767	7.43E-05
3h	ACA1_066780/clean/scf7180000084600:43017-43085:-	ACA1_066780	Transcription initiation factor tfiid subunit 13, putative [Source:UniProtKB/TrEMBL;Acc:L8GP29]	0.013241533	0.233021967	0.219780433	3.96E-05
3h	ACA1_035590/clean/scf7180000084564:95022-95120:-	ACA1_035590	ANK_REP_REGION domain-containing protein [Source:UniProtKB/TrEMBL;Acc:L8H9N8]	0.0084183	0.227877967	0.219459667	0.000855792
3h	ACA1_288340/clean/scf7180000084777:174113-174182:+	ACA1_288340	Conditioned medium factor, putative [Source:UniProtKB/TrEMBL;Acc:L8HKD6]	0.006849467	0.226104	0.219254533	3.62E-07
3h	ACA1_373560/clean/scf7180000084679:99170-99270:-	ACA1_373560	hypothetical protein	0.001950517	0.2210697	0.219119183	0.000189885
3h	ACA1_291510/clean/scf7180000084777:763760-763848:+	ACA1_291510	Ubiquitin carboxylterminal hydrolase 15, putative [Source:UniProtKB/TrEMBL;Acc:L8HKZ1]	0.00224467	0.221300067	0.219055397	1.29E-06
3h	ACA1_201470/clean/scf7180000084753:559727-559833:-	ACA1_201470	Rasrelated protein Rab-2B, putative [Source:UniProtKB/TrEMBL;Acc:L8H2N2]	0.021999867	0.240961133	0.218961267	0.000129734
3h	ACA1_097290/clean/scf7180000084645:1209828-1209899:+	ACA1_097290	hypothetical protein	0.002081393	0.221022667	0.218941273	1.43E-05
3h	ACA1_035260/clean/scf7180000084564:7944-8035:-	ACA1_035260	Guanine nucleotidebinding protein G(O), alpha subunit, putative [Source:UniProtKB/TrEMBL;Acc:L8HCU4]	0.003791363	0.222708533	0.21891717	3.41E-07
3h	ACA1_057100/clean/scf7180000084599:44082-44146:-	ACA1_057100	unspecified product	0	0.218832867	0.218832867	0.000173075
3h	ACA1_297540/clean/scf7180000082850:2856-2942:+	ACA1_297540	WD_REPEATS_REGION domain-containing protein [Source:UniProtKB/TrEMBL;Acc:L8GGE0]	0.005952367	0.2247261	0.218773733	0.000241302
3h	ACA1_055390/clean/scf7180000084594:694337-694411:-	ACA1_055390	hypothetical protein	0.026754533	0.245361433	0.2186069	0.03466132
3h	ACA1_097640/clean/scf7180000084645:1303982-1304095:+	ACA1_097640	Fbox domain containing protein [Source:UniProtKB/TrEMBL;Acc:L8GK52]	0.000259853	0.2188463	0.218586447	6.65E-06
3h	ACA1_074140/clean/scf7180000084605:36154-36227:+	ACA1_074140	Transporter, major facilitator subfamily protein [Source:UniProtKB/TrEMBL;Acc:L8HK94]	0.008715933	0.227282	0.218566067	1.06E-06
3h	ACA1_367890/clean/scf7180000084664:8887-8973:-	ACA1_367890	hypothetical protein	0.002878313	0.221416667	0.218538353	5.92E-05
3h	ACA1_174300/clean/scf7180000084741:664482-664561:+	ACA1_174300	hypothetical protein	0.016585067	0.235112767	0.2185277	0.003851537
3h	ACA1_040690/clean/scf7180000084572:50807-50890:+	ACA1_040690	Multisensor hybrid histidine kinase [Source:UniProtKB/TrEMBL;Acc:L8H1W6]	0.017547097	0.2360598	0.218512703	0.001251676
3h	ACA1_115350/clean/scf7180000084669:611939-612014:+	ACA1_115350	hypothetical protein	0.011089843	0.229504	0.218414157	2.13E-06
3h	ACA1_335940/clean/scf7180000084593:9780-9870:-	ACA1_335940	RNA recognition motif domain containing protein [Source:UniProtKB/TrEMBL;Acc:L8H3Y6]	0.010102887	0.228437633	0.218334747	3.21E-05
3h	ACA1_399470/clean/scf7180000084775:10745-10857:-	ACA1_399470	hypothetical protein	0.059413267	0.277731333	0.218318067	0.007551484
3h	ACA1_019350/clean/scf7180000083937:28549-28604:-	ACA1_019350	FGGAP repeat domain containing protein [Source:UniProtKB/TrEMBL;Acc:L8HFR8]	0.005727737	0.224042667	0.21831493	3.87E-06
3h	ACA1_000820/clean/scf7180000082828:13124-13204:+	ACA1_000820	RING-type domain-containing protein [Source:UniProtKB/TrEMBL;Acc:L8H7M3]	0.01843175	0.236567567	0.218135817	0.002140922
3h	ACA1_092610/clean/scf7180000084645:367354-367418:+	ACA1_092610	EF hand domain containing protein [Source:UniProtKB/TrEMBL;Acc:L8GIZ7]	0.00384324	0.2218829	0.21803966	5.58E-06
3h	ACA1_158170/clean/scf7180000084724:201453-201538:-	ACA1_158170	Pescadillo homolog [Source:UniProtKB/TrEMBL;Acc:L8HBG6]	0.0155966	0.2335611	0.2179645	1.13E-05
3h	ACA1_171430/clean/scf7180000084741:103211-103296:+	ACA1_171430	hypothetical protein	0.054491867	0.272297667	0.2178058	0.017782079
3h	ACA1_089970/clean/scf7180000084639:407210-407286:-	ACA1_089970	Macro domain-containing protein [Source:UniProtKB/TrEMBL;Acc:L8GU43]	0.006022013	0.223803433	0.21778142	0.000113435
3h	ACA1_173370/clean/scf7180000084741:472672-472815:+	ACA1_173370	Chromo domain-containing protein [Source:UniProtKB/TrEMBL;Acc:L8HGN8]	0.0345099	0.252186	0.2176761	0.044566789
3h	ACA1_055820/clean/scf7180000084594:775217-775305:+	ACA1_055820	PHD-finger domain containing protein [Source:UniProtKB/TrEMBL;Acc:L8H8L2]	0.040211023	0.257790667	0.217579643	0.015961868
3h	ACA1_069510/clean/scf7180000084603:458991-459062:-	ACA1_069510	hypothetical protein	0	0.217551833	0.217551833	9.67E-06
3h	ACA1_373560/clean/scf7180000084679:100067-100184:-	ACA1_373560	hypothetical protein	0.00779303	0.225244667	0.217451637	0.000906215
3h	ACA1_279580/clean/scf7180000084776:418668-418759:+	ACA1_279580	Interferonrelated developmental regulator 1, putative [Source:UniProtKB/TrEMBL;Acc:L8H954]	0.001531953	0.218921367	0.217389413	0.000916245
3h	ACA1_037900/clean/scf7180000084568:54638-54719:-	ACA1_037900	Nudix hydrolase domain-containing protein [Source:UniProtKB/TrEMBL;Acc:L8GKU1]	0.004091667	0.221070033	0.216978367	0.025118903
3h	ACA1_372040/clean/scf7180000084664:618598-618695:-	ACA1_372040	Zn(2)-C6 fungal-type domain-containing protein [Source:UniProtKB/TrEMBL;Acc:L8GZD9]	0.020848133	0.237813467	0.216965333	0.002900059
3h	ACA1_042250/clean/scf7180000084573:70535-70613:-	ACA1_042250	Phosphoglucomutase, putative [Source:UniProtKB/TrEMBL;Acc:L8GV41]	0.001012323	0.2178923	0.216879977	1.80E-06
3h	ACA1_361100/clean/scf7180000084622:241386-241485:-	ACA1_361100	TLDc domain-containing protein [Source:UniProtKB/TrEMBL;Acc:L8HE93]	0.019501073	0.236350033	0.21684896	0.000273029
3h	ACA1_244370/clean/scf7180000084763:388454-388519:-	ACA1_244370	Protein kinase domain-containing protein [Source:UniProtKB/TrEMBL;Acc:L8GK51]	0	0.216605233	0.216605233	0.000163431
3h	ACA1_362720/clean/scf7180000084624:158460-158523:-	ACA1_362720	Neutral ceramidase [Source:UniProtKB/TrEMBL;Acc:L8GFC5]	0.00607787	0.2226815	0.21660363	1.79E-05
3h	ACA1_074160/clean/scf7180000084605:39260-39355:+	ACA1_074160	Aamy domain-containing protein [Source:UniProtKB/TrEMBL;Acc:L8HJ75]	0.004909957	0.221340533	0.216430577	2.27E-06
3h	ACA1_048860/clean/scf7180000084583:2933-3030:+	ACA1_048860	Aa_trans domain-containing protein [Source:UniProtKB/TrEMBL;Acc:L8HKE2]	0.002487276	0.218850067	0.216362791	3.58E-06
3h	ACA1_051550/clean/scf7180000084592:36675-36761:-	ACA1_051550	Phenylalanine hydroxylase [Source:UniProtKB/TrEMBL;Acc:L8GPS8]	0.002129961	0.218445233	0.216315273	1.89E-07
3h	ACA1_180780/clean/scf7180000084744:79330-79457:-	ACA1_180780	RasGEF domain containing protein [Source:UniProtKB/TrEMBL;Acc:L8GF96]	0.002793937	0.219092467	0.21629853	6.55E-06
3h	ACA1_370300/clean/scf7180000084664:489361-489446:-	ACA1_370300	hypothetical protein	0.00824339	0.224411	0.21616761	5.21E-06
3h	ACA1_181990/clean/scf7180000084745:98102-98208:+	ACA1_181990	hypothetical protein	0.00838214	0.224379	0.21599686	0.000400667
3h	ACA1_257000/clean/scf7180000084769:122084-122137:+	ACA1_257000	3-hydroxyisobutyryl-CoA hydrolase, mitochondrial [Source:UniProtKB/TrEMBL;Acc:L8GH72]	0.059433533	0.275313667	0.215880133	0.007559018
3h	ACA1_061580/clean/scf7180000084599:965956-966064:+	ACA1_061580	BZIP domain-containing protein [Source:UniProtKB/TrEMBL;Acc:L8GXX9]	0.005221937	0.220974667	0.21575273	1.19E-07
3h	ACA1_349640/clean/scf7180000084690:22395-22516:+	ACA1_349640	HEAT repeat domain containing protein [Source:UniProtKB/TrEMBL;Acc:L8GKB3]	0	0.215728667	0.215728667	1.81E-05
3h	ACA1_277880/clean/scf7180000084776:119301-119364:+	ACA1_277880	RBR-type E3 ubiquitin transferase [Source:UniProtKB/TrEMBL;Acc:L8H6I3]	0.014716667	0.230437333	0.215720667	7.64E-05
3h	ACA1_359280/clean/scf7180000084742:71354-71460:+	ACA1_359280	hypothetical protein	0.015246233	0.230832667	0.215586433	0.001614912
3h	ACA1_175370/clean/scf7180000084741:940214-940279:-	ACA1_175370	PKD_channel domain-containing protein [Source:UniProtKB/TrEMBL;Acc:L8HI19]	0.02065894	0.2361432	0.21548426	0.001355726
3h	ACA1_173960/clean/scf7180000084741:559882-559987:+	ACA1_173960	AB hydrolase-1 domain-containing protein [Source:UniProtKB/TrEMBL;Acc:L8HJ69]	0.0260944	0.2415178	0.2154234	0.000561111
3h	ACA1_197850/clean/scf7180000084753:40179-40274:-	ACA1_197850	HAD hydrolase, family IIA subfamily protein [Source:UniProtKB/TrEMBL;Acc:L8H335]	0.002358233	0.217714733	0.2153565	0.000158452
3h	ACA1_131560/clean/scf7180000084699:88889-88960:-	ACA1_131560	hypothetical protein	0.001118567	0.216344133	0.215225567	2.91E-07
3h	ACA1_222780/clean/scf7180000084760:80959-81048:-	ACA1_222780	Metal cation transporter, ZIP subfamily protein [Source:UniProtKB/TrEMBL;Acc:L8GSU8]	0.008771933	0.223968567	0.215196633	0.000131933
3h	ACA1_177430/clean/scf7180000084743:65542-65667:-	ACA1_177430	hypothetical protein	0.00927033	0.224254733	0.214984403	0.000431784
3h	ACA1_289740/clean/scf7180000084777:453435-453535:+	ACA1_289740	Rho-GAP domain-containing protein [Source:UniProtKB/TrEMBL;Acc:L8HLF4]	0.010693187	0.2256396	0.214946413	0.000615502
3h	ACA1_002490/clean/scf7180000082931:7734-7808:-	ACA1_002490	AA_TRNA_LIGASE_II_ALA domain-containing protein [Source:UniProtKB/TrEMBL;Acc:L8GVN5]	0.013964847	0.228886333	0.214921487	1.96E-05
3h	ACA1_264110/clean/scf7180000084770:190607-190692:+	ACA1_264110	Ribosomal protein L3, putative [Source:UniProtKB/TrEMBL;Acc:L8H1X4]	0.001882385	0.216765	0.214882615	1.77E-18
3h	ACA1_173140/clean/scf7180000084741:410426-410498:+	ACA1_173140	Deacetylase sirtuin-type domain-containing protein [Source:UniProtKB/TrEMBL;Acc:L8HJX9]	0.000889214	0.215601833	0.21471262	4.99E-06
3h	ACA1_296240/clean/scf7180000084777:1303889-1303979:-	ACA1_296240	hypothetical protein	0.005509633	0.220216367	0.214706733	5.57E-05
3h	ACA1_279530/clean/scf7180000084776:409910-409983:+	ACA1_279530	Rab4, putative [Source:UniProtKB/TrEMBL;Acc:L8H946]	0.00640947	0.220913967	0.214504497	1.16E-06
3h	ACA1_379060/clean/scf7180000084711:273414-273535:+	ACA1_379060	Protein kinase domain-containing protein [Source:UniProtKB/TrEMBL;Acc:L8GRW8]	0.00694279	0.221440567	0.214497777	3.45E-06
3h	ACA1_062670/clean/scf7180000084599:1269911-1270048:-	ACA1_062670	B30.2/SPRY domain-containing protein [Source:UniProtKB/TrEMBL;Acc:L8GYA5]	0.002899618	0.2173864	0.214486782	1.88E-05
3h	ACA1_215970/clean/scf7180000084758:417444-417528:-	ACA1_215970	EGFlike domain containing protein [Source:UniProtKB/TrEMBL;Acc:L8GQ73]	0.058408033	0.272858933	0.2144509	0.03616779
3h	ACA1_142370/clean/scf7180000084708:152533-152616:-	ACA1_142370	Serine hydroxymethyltransferase [Source:UniProtKB/TrEMBL;Acc:L8HDT1]	0.000664348	0.2151101	0.214445752	3.20E-08
3h	ACA1_068360/clean/scf7180000084603:180385-180458:+	ACA1_068360	Dual specificity protein kinase [Source:UniProtKB/TrEMBL;Acc:L8HDE4]	0.00109649	0.215407333	0.214310843	3.13E-07
3h	ACA1_271500/clean/scf7180000084771:46341-46525:+	ACA1_271500	Leucine rich repeat domain containing protein [Source:UniProtKB/TrEMBL;Acc:L8GNI4]	0.008983963	0.223204567	0.214220603	0.000143386
3h	ACA1_050150/clean/scf7180000084589:42556-42627:+	ACA1_050150	Glutathione Stransferase [Source:UniProtKB/TrEMBL;Acc:L8H557]	0.691004667	0.905064667	0.21406	0.003814479
3h	ACA1_139260/clean/scf7180000084705:64101-64253:-	ACA1_139260	GTPbinding protein [Source:UniProtKB/TrEMBL;Acc:L8GN15]	0.01032549	0.224299867	0.213974377	7.18E-06
3h	ACA1_139260/clean/scf7180000084705:63689-63798:-	ACA1_139260	GTPbinding protein [Source:UniProtKB/TrEMBL;Acc:L8GN15]	0.00402686	0.217887833	0.213860973	2.39E-06
3h	ACA1_358650/clean/scf7180000084742:21550-21625:+	ACA1_358650	Ubiquitin carboxyl-terminal hydrolase [Source:UniProtKB/TrEMBL;Acc:L8GKM0]	0.01231231	0.226079233	0.213766923	4.26E-06
3h	ACA1_123880/clean/scf7180000084686:77497-77610:-	ACA1_123880	hypothetical protein	0.02154066	0.2352415	0.21370084	0.002081819
3h	ACA1_309500/clean/scf7180000083762:10728-10802:+	ACA1_309500	Pyridine nucleotidedisulfide oxidoreductase domain containing protein [Source:UniProtKB/TrEMBL;Acc:L8GI23]	0.006933017	0.220621233	0.213688217	1.54E-08
3h	ACA1_261470/clean/scf7180000084769:689169-689255:+	ACA1_261470	hypothetical protein	0.001418473	0.215101133	0.21368266	3.16E-06
3h	ACA1_055050/clean/scf7180000084594:641714-641811:+	ACA1_055050	SH3 domain containing protein [Source:UniProtKB/TrEMBL;Acc:L8H5H2]	0.007174933	0.2208043	0.213629367	1.00E-10
3h	ACA1_357960/clean/scf7180000084735:31010-31101:+	ACA1_357960	Alternative oxidase isoform A, putative [Source:UniProtKB/TrEMBL;Acc:L8HDC6]	0.00632514	0.219917067	0.213591927	3.39E-07
3h	ACA1_087620/clean/scf7180000084639:79771-79858:-	ACA1_087620	BZIP domain-containing protein [Source:UniProtKB/TrEMBL;Acc:L8GTV0]	0	0.2134433	0.2134433	5.82E-05
3h	ACA1_070030/clean/scf7180000084603:625841-625915:-	ACA1_070030	NAD(P)-bd_dom domain-containing protein [Source:UniProtKB/TrEMBL;Acc:L8HDV9]	0.005158349	0.218570667	0.213412318	2.10E-06
3h	ACA1_373590/clean/scf7180000084679:109405-109488:-	ACA1_373590	hypothetical protein	0.0718024	0.285191667	0.213389267	0.024322516
3h	ACA1_325420/clean/scf7180000084495:3899-3988:+	ACA1_325420	Nicotinate-nucleotide pyrophosphorylase [carboxylating] [Source:UniProtKB/TrEMBL;Acc:L8H9V7]	0.0205503	0.233867333	0.213317033	3.33E-05
3h	ACA1_219870/clean/scf7180000084758:829143-829275:-	ACA1_219870	Lysophospholipase 3 (Lysosomal phospholipase A2) isoform 7, putative [Source:UniProtKB/TrEMBL;Acc:L8GT04]	0.005886487	0.219027767	0.21314128	1.39E-05
3h	ACA1_278150/clean/scf7180000084776:180508-180568:-	ACA1_278150	hypothetical protein	0.00366445	0.2168037	0.21313925	5.71E-08
3h	ACA1_142630/clean/scf7180000084708:205022-205101:+	ACA1_142630	unspecified product	0.008585487	0.221629033	0.213043547	4.80E-06
3h	ACA1_201130/clean/scf7180000084753:486280-486360:+	ACA1_201130	F-box domain-containing protein [Source:UniProtKB/TrEMBL;Acc:L8H5M0]	0.026945033	0.239827333	0.2128823	0.001519766
3h	ACA1_183980/clean/scf7180000084745:535038-535156:-	ACA1_183980	Protein-S-isoprenylcysteine O-methyltransferase [Source:UniProtKB/TrEMBL;Acc:L8H887]	0.017173033	0.229995333	0.2128223	0.000168195
3h	ACA1_077920/clean/scf7180000084614:42579-42709:-	ACA1_077920	hypothetical protein	0.0092584	0.222073667	0.212815267	2.03E-06
3h	ACA1_090220/clean/scf7180000084639:459832-459925:-	ACA1_090220	Rab-GAP TBC domain-containing protein [Source:UniProtKB/TrEMBL;Acc:L8GU66]	0.015781777	0.22859	0.212808223	4.14E-05
3h	ACA1_108270/clean/scf7180000084661:28038-28104:+	ACA1_108270	Transferrin receptor family dimerization domain containing protein [Source:UniProtKB/TrEMBL;Acc:L8GC39]	0.013695717	0.226370967	0.21267525	0.000120199
3h	ACA1_048870/clean/scf7180000084583:5209-5267:-	ACA1_048870	hypothetical protein	0.0803339	0.292898333	0.212564433	0.016241165
3h	ACA1_162500/clean/scf7180000084726:49376-49450:-	ACA1_162500	31.5 kDa midgut protein [Source:UniProtKB/TrEMBL;Acc:L8GYK7]	0.001230013	0.2134509	0.212220887	5.12E-07
3h	ACA1_091330/clean/scf7180000084645:89720-89791:-	ACA1_091330	Pyruvate kinase [Source:UniProtKB/TrEMBL;Acc:L8GIR3]	0.001296007	0.213474233	0.212178227	0.000484629
3h	ACA1_206370/clean/scf7180000084757:17638-17712:+	ACA1_206370	MOSC domain-containing protein [Source:UniProtKB/TrEMBL;Acc:L8GY21]	0.0130088	0.225132433	0.212123633	0.000156324
3h	ACA1_278190/clean/scf7180000084776:187565-187634:-	ACA1_278190	MAT1 domain-containing protein [Source:UniProtKB/TrEMBL;Acc:L8H822]	0.003908409	0.215999833	0.212091424	3.29E-06
3h	ACA1_169060/clean/scf7180000084739:13677-13778:-	ACA1_169060	Derlin [Source:UniProtKB/TrEMBL;Acc:L8HHS6]	0.008865267	0.2209517	0.212086433	1.58E-05
3h	ACA1_362810/clean/scf7180000084624:188096-188170:+	ACA1_362810	Tyrosyl-tRNA synthetase [Source:UniProtKB/TrEMBL;Acc:L8GFZ7]	0.00880012	0.220841033	0.212040913	3.18E-05
3h	ACA1_064240/clean/scf7180000084599:1566001-1566069:+	ACA1_064240	NAD(P)-bd_dom domain-containing protein [Source:UniProtKB/TrEMBL;Acc:L8GXD4]	0.0033501	0.215322867	0.211972767	2.49E-08
3h	ACA1_368900/clean/scf7180000084664:191301-191373:+	ACA1_368900	Glyco_hydro_65m domain-containing protein [Source:UniProtKB/TrEMBL;Acc:L8H074]	0.01292065	0.224737333	0.211816683	0.000464242
3h	ACA1_097640/clean/scf7180000084645:1303621-1303760:+	ACA1_097640	Fbox domain containing protein [Source:UniProtKB/TrEMBL;Acc:L8GK52]	0	0.211742333	0.211742333	8.54E-06
3h	ACA1_296110/clean/scf7180000084777:1299095-1299181:-	ACA1_296110	MORN repeat variantcontaining protein [Source:UniProtKB/TrEMBL;Acc:L8HLH8]	0.001704793	0.2134345	0.211729707	1.15E-12
3h	ACA1_067410/clean/scf7180000084601:18534-18601:-	ACA1_067410	F-box domain-containing protein [Source:UniProtKB/TrEMBL;Acc:L8GTP9]	0.006666667	0.218317633	0.211650967	0.000248115
3h	ACA1_373690/clean/scf7180000084679:142772-142847:+	ACA1_373690	ADPribosylglycohydrolase superfamily protein [Source:UniProtKB/TrEMBL;Acc:L8GGT7]	0.010582	0.2222011	0.2116191	0.006828968
3h	ACA1_385570/clean/scf7180000084730:211764-211846:-	ACA1_385570	Tetratricopeptide repeat domain containing protein [Source:UniProtKB/TrEMBL;Acc:L8H9C1]	0.013480017	0.225070133	0.211590117	0.00160617
3h	ACA1_176060/clean/scf7180000084741:1118135-1118225:+	ACA1_176060	DAGKc domain-containing protein [Source:UniProtKB/TrEMBL;Acc:L8HJR7]	0.01603365	0.227581	0.21154735	4.38E-05
3h	ACA1_189640/clean/scf7180000084748:71603-71672:-	ACA1_189640	Bifunctional aspartate kinase/diaminopimelate decarboxylase protein [Source:UniProtKB/TrEMBL;Acc:L8GEY3]	0.010324663	0.221863	0.211538337	1.01E-05
3h	ACA1_063540/clean/scf7180000084599:1447429-1447541:+	ACA1_063540	hypothetical protein	0.007503171	0.218857367	0.211354196	5.65E-06
3h	ACA1_203090/clean/scf7180000084754:141372-141504:+	ACA1_203090	Glycosyl hydrolase, family 31 protein [Source:UniProtKB/TrEMBL;Acc:L8GTG9]	0.014217987	0.225539333	0.211321347	7.08E-06
3h	ACA1_392520/clean/scf7180000084740:284038-284133:-	ACA1_392520	hypothetical protein	0.016126867	0.2272239	0.211097033	0.000487596
3h	ACA1_322570/clean/scf7180000084466:22732-22820:+	ACA1_322570	Hsp40, putative [Source:UniProtKB/TrEMBL;Acc:L8HCJ2]	0.000943364	0.211907033	0.210963669	8.90E-13
3h	ACA1_396130/clean/scf7180000084759:24761-24866:-	ACA1_396130	Prefoldin, alpha subunit [Source:UniProtKB/TrEMBL;Acc:L8HDI9]	0.006931733	0.217855833	0.2109241	1.09E-08
3h	ACA1_050200/clean/scf7180000084589:54739-54830:+	ACA1_050200	Mammalian PTEN tumor suppressorlike, putative [Source:UniProtKB/TrEMBL;Acc:L8H3Q5]	0.030708767	0.241575733	0.210866967	0.003180311
3h	ACA1_066150/clean/scf7180000084599:2012189-2012260:+	ACA1_066150	Myosin head (Motor domain) domain containing protein [Source:UniProtKB/TrEMBL;Acc:L8GX00]	0	0.210816933	0.210816933	0.008983888
3h	ACA1_379330/clean/scf7180000084711:331016-331105:-	ACA1_379330	Polynucleotide adenyltransferase [Source:UniProtKB/TrEMBL;Acc:L8GUG7]	0.0210597	0.2318247	0.210765	0.019254562
3h	ACA1_174250/clean/scf7180000084741:655187-655277:-	ACA1_174250	RBR-type E3 ubiquitin transferase [Source:UniProtKB/TrEMBL;Acc:L8HHV0]	0.009537813	0.2202794	0.210741587	1.79E-06
3h	ACA1_309330/clean/scf7180000083758:5719-5781:-	ACA1_309330	hypothetical protein	0.017984933	0.228637667	0.210652733	0.000756261
3h	ACA1_291510/clean/scf7180000084777:763580-763659:+	ACA1_291510	Ubiquitin carboxylterminal hydrolase 15, putative [Source:UniProtKB/TrEMBL;Acc:L8HKZ1]	0.000366503	0.211010933	0.21064443	9.20E-08
3h	ACA1_113850/clean/scf7180000084669:387832-387919:-	ACA1_113850	PMSR domain-containing protein [Source:UniProtKB/TrEMBL;Acc:L8H3F4]	0.00059952	0.2111206	0.21052108	2.17E-08
3h	ACA1_246510/clean/scf7180000084763:565003-565114:+	ACA1_246510	PPM-type phosphatase domain-containing protein [Source:UniProtKB/TrEMBL;Acc:L8GL94]	0.00805946	0.218205	0.21014554	4.56E-05
3h	ACA1_182120/clean/scf7180000084745:132340-132406:+	ACA1_182120	WD repeat domain 5B, putative [Source:UniProtKB/TrEMBL;Acc:L8HA46]	0.004398847	0.214500233	0.210101387	7.98E-06
3h	ACA1_261430/clean/scf7180000084769:675335-675421:+	ACA1_261430	Serine/threonine protein kinase, putative [Source:UniProtKB/TrEMBL;Acc:L8GG67]	0.001757372	0.211822433	0.210065061	2.22E-06
3h	ACA1_287850/clean/scf7180000084777:96149-96240:-	ACA1_287850	RING-type domain-containing protein [Source:UniProtKB/TrEMBL;Acc:L8HHQ7]	0	0.209974367	0.209974367	2.95E-05
3h	ACA1_048400/clean/scf7180000084582:77759-77880:+	ACA1_048400	Rieske domain-containing protein [Source:UniProtKB/TrEMBL;Acc:L8GD10]	0.00217651	0.212051533	0.209875023	4.15E-09
3h	ACA1_199060/clean/scf7180000084753:211402-211495:-	ACA1_199060	HECT domain-containing protein [Source:UniProtKB/TrEMBL;Acc:L8H2E7]	0.006454047	0.216203633	0.209749587	0.001685766

3h	ACA1_378620/clean/scf7180000084711:239815-239889:-	ACA1_378620	AAA-ATPase_like domain-containing protein [Source:UniProtKB/TrEMBL;Acc:L8GS16]	0.112100633	0.321679667	0.209579033	0.01922873
3h	ACA1_185320/clean/scf7180000084746:93644-93740:+	ACA1_185320	hypothetical protein	0.002044303	0.211566	0.209521697	9.04E-08
3h	ACA1_116270/clean/scf7180000084669:744545-744627:+	ACA1_116270	G_PROTEIN_RECEP_F1_2 domain-containing protein [Source:UniProtKB/TrEMBL;Acc:L8H6T9]	0.048330833	0.257814867	0.209484033	0.004209373
3h	ACA1_260220/clean/scf7180000084769:460002-460060:+	ACA1_260220	Cytochrome b561 domain-containing protein [Source:UniProtKB/TrEMBL;Acc:L8GG14]	0.051328967	0.260752333	0.209423367	0.001260818
3h	ACA1_321440/clean/scf7180000084439:31391-31467:+	ACA1_321440	hypothetical protein	0.022436367	0.231848467	0.2094121	0.000137883
3h	ACA1_371630/clean/scf7180000084664:566364-566461:+	ACA1_371630	hypothetical protein	0.003484807	0.212791633	0.209306827	4.98E-10
3h	ACA1_377530/clean/scf7180000084711:83512-83616:-	ACA1_377530	Protein kinase domain-containing protein [Source:UniProtKB/TrEMBL;Acc:L8GR81]	0.000761343	0.2099128	0.209151457	1.12E-05
3h	ACA1_116260/clean/scf7180000084669:741428-741522:-	ACA1_116260	Leucine Rich Repeat and BTB/POZ domain containing protein [Source:UniProtKB/TrEMBL;Acc:L8H4T0]	0.00842742	0.217560433	0.209133013	0.002880403
3h	ACA1_236110/clean/scf7180000084762:407408-407485:-	ACA1_236110	PPM-type phosphatase domain-containing protein [Source:UniProtKB/TrEMBL;Acc:L8H2W4]	0.00383651	0.2129574	0.20912089	1.51E-05
3h	ACA1_319980/clean/scf7180000084259:22358-22491:-	ACA1_319980	PDEase domain-containing protein [Source:UniProtKB/TrEMBL;Acc:L8HLY7]	0.025282217	0.2343973	0.209115083	0.002647497
3h	ACA1_374550/clean/scf7180000084679:320974-321062:+	ACA1_374550	BTB/POZ domain containing protein [Source:UniProtKB/TrEMBL;Acc:L8GHB1]	0.033738133	0.242845733	0.2091076	0.002259915
3h	ACA1_113680/clean/scf7180000084669:347553-347623:-	ACA1_113680	M20_dimer domain-containing protein [Source:UniProtKB/TrEMBL;Acc:L8H5M1]	0.0269034	0.235929	0.2090256	2.32E-05
3h	ACA1_074520/clean/scf7180000084608:28892-29038:+	ACA1_074520	SH3 domain containing protein [Source:UniProtKB/TrEMBL;Acc:L8HFD8]	0.00486895	0.213785667	0.208916717	1.23E-05
3h	ACA1_237870/clean/scf7180000084763:36869-36976:+	ACA1_237870	Methyltransferase [Source:UniProtKB/TrEMBL;Acc:L8GKC5]	0.000777471	0.209651933	0.208874462	1.02E-11
3h	ACA1_265970/clean/scf7180000084770:559853-559927:+	ACA1_265970	SAM domain (Sterile alpha motif) domain containing protein [Source:UniProtKB/TrEMBL;Acc:L8H2A8]	0.0246952	0.233563767	0.208868567	0.002259915
3h	ACA1_368250/clean/scf7180000084664:88835-88906:-	ACA1_368250	BPI2 domain-containing protein [Source:UniProtKB/TrEMBL;Acc:L8GY12]	0.007357837	0.216032233	0.208674397	0.000159173
3h	ACA1_333240/clean/scf7180000084581:25082-25161:+	ACA1_333240	hypothetical protein	0	0.2085284	0.2085284	0.009920423
3h	ACA1_380760/clean/scf7180000084712:83742-83819:+	ACA1_380760	Aminotran_5 domain-containing protein [Source:UniProtKB/TrEMBL;Acc:L8GNK4]	0.157784333	0.366213667	0.208429333	0.044038264
3h	ACA1_265380/clean/scf7180000084770:455824-455907:-	ACA1_265380	Phosphatidylinositol glycan anchor biosynthesis, class S, putative [Source:UniProtKB/TrEMBL;Acc:L8H3M4]	0.005767263	0.214166	0.208398737	4.72E-05
3h	ACA1_083640/clean/scf7180000084629:7975-8105:-	ACA1_083640	Elongation factor 1alpha [Source:UniProtKB/TrEMBL;Acc:L8GYH2]	0.000852993	0.208944467	0.208091474	8.73E-07
3h	ACA1_278760/clean/scf7180000084776:238445-238551:+	ACA1_278760	Protein kinase domain-containing protein [Source:UniProtKB/TrEMBL;Acc:L8H6Y1]	0.066428867	0.274476667	0.2080478	0.011274842
3h	ACA1_093010/clean/scf7180000084645:457718-457799:+	ACA1_093010	BNR/Aspbox repeat domain containing protein [Source:UniProtKB/TrEMBL;Acc:L8GKN9]	0.00035229	0.208398767	0.208046477	4.58E-07
3h	ACA1_341100/clean/scf7180000084620:50745-50810:-	ACA1_341100	MFS domain-containing protein [Source:UniProtKB/TrEMBL;Acc:L8GR49]	0.0038322	0.2116457	0.2078135	1.06E-05
3h	ACA1_038160/clean/scf7180000084568:85509-85605:-	ACA1_038160	Protein kinase domain-containing protein [Source:UniProtKB/TrEMBL;Acc:L8GN38]	0.006069597	0.213880633	0.207811037	5.92E-05
3h	ACA1_191480/clean/scf7180000084749:491-576:-	ACA1_191480	CS domain containing protein [Source:UniProtKB/TrEMBL;Acc:L8GMZ3]	0.00062221	0.208420933	0.207798723	1.29E-08
3h	ACA1_254850/clean/scf7180000084768:421982-422073:+	ACA1_254850	Aamy domain-containing protein [Source:UniProtKB/TrEMBL;Acc:L8HAX2]	0	0.2077374	0.2077374	1.18E-06
3h	ACA1_301740/clean/scf7180000083222:32650-32743:-	ACA1_301740	hypothetical protein	0	0.207697733	0.207697733	0.002461883
3h	ACA1_002610/clean/scf7180000082935:3043-3116:+	ACA1_002610	hypothetical protein	0.027604067	0.235090567	0.2074865	0.0002692
3h	ACA1_285200/clean/scf7180000084776:955499-955583:-	ACA1_285200	Na_H_Exchanger domain-containing protein [Source:UniProtKB/TrEMBL;Acc:L8H9U2]	0.001198787	0.2084688	0.207270013	8.57E-07
3h	ACA1_379220/clean/scf7180000084711:303859-303974:-	ACA1_379220	FYVE-type domain-containing protein [Source:UniProtKB/TrEMBL;Acc:L8GUF8]	0	0.207194633	0.207194633	1.97E-05
3h	ACA1_107640/clean/scf7180000084660:240582-240654:+	ACA1_107640	Signal transduction histidine kinase [Source:UniProtKB/TrEMBL;Acc:L8GNL7]	0.031171633	0.238235767	0.207064133	0.000171487
3h	ACA1_226760/clean/scf7180000084761:73056-73194:+	ACA1_226760	hypothetical protein	0.016806767	0.223773	0.206966233	0.011356141
3h	ACA1_054500/clean/scf7180000084594:513494-513598:-	ACA1_054500	Glycosyl hydrolase family 65 central catalytic domain containing protein [Source:UniProtKB/TrEMBL;Acc:L8H5C3]	0.003617683	0.2103583	0.206740617	2.89E-06
3h	ACA1_198520/clean/scf7180000084753:132211-132329:-	ACA1_198520	Carrier superfamily protein [Source:UniProtKB/TrEMBL;Acc:L8H5C1]	0.014418303	0.221083867	0.206665563	0.001404175
3h	ACA1_151330/clean/scf7180000084716:104712-104793:-	ACA1_151330	Purple acid phosphatase [Source:UniProtKB/TrEMBL;Acc:L8H0Q3]	0.00476178	0.2113943	0.20663252	1.22E-06
3h	ACA1_017740/clean/scf7180000083876:4707-4789:+	ACA1_017740	5'-3' exoribonuclease 1 [Source:UniProtKB/TrEMBL;Acc:L8GMR4]	0.000284801	0.206905367	0.206620566	2.40E-08
3h	ACA1_290560/clean/scf7180000084777:625432-625542:+	ACA1_290560	Peptidase_M14 domain-containing protein [Source:UniProtKB/TrEMBL;Acc:L8HLJ4]	0.001422413	0.207849267	0.206426853	6.00E-07
3h	ACA1_002320/clean/scf7180000082924:9794-9877:+	ACA1_002320	Fbox domain containing protein [Source:UniProtKB/TrEMBL;Acc:L8GU55]	0.021741513	0.228094767	0.206353253	0.00016572
3h	ACA1_279600/clean/scf7180000084776:423937-424041:-	ACA1_279600	hypothetical protein	0.017102107	0.223404667	0.20630256	0.000751002
3h	ACA1_027790/clean/scf7180000084471:18879-18962:+	ACA1_027790	Protein SEQ ID from patent family protein [Source:UniProtKB/TrEMBL;Acc:L8HK75]	0.025528967	0.231678633	0.206149667	0.005358949
3h	ACA1_261200/clean/scf7180000084769:603647-603742:+	ACA1_261200	UBIQUITIN_CONJUGAT_2 domain-containing protein [Source:UniProtKB/TrEMBL;Acc:L8GFE3]	0.020343	0.226445333	0.206102333	0.03349657
3h	ACA1_214940/clean/scf7180000084758:268299-268385:-	ACA1_214940	Leucine rich repeat domain containing protein [Source:UniProtKB/TrEMBL;Acc:L8GQ02]	0.003898633	0.2097506	0.205851967	0.000127847
3h	ACA1_087390/clean/scf7180000084639:22496-22610:+	ACA1_087390	ANK_REP_REGION domain-containing protein [Source:UniProtKB/TrEMBL;Acc:L8GUE4]	0.001374297	0.207172867	0.20579857	1.18E-05
3h	ACA1_320840/clean/scf7180000084416:34929-35110:+	ACA1_320840	HSP91 (Heat shock protein 91), putative [Source:UniProtKB/TrEMBL;Acc:L8GTS5]	0.001686877	0.207418767	0.20573189	1.01E-09
3h	ACA1_289280/clean/scf7180000084777:406200-406267:-	ACA1_289280	RasGEF domain containing protein [Source:UniProtKB/TrEMBL;Acc:L8HJ36]	0	0.205677333	0.205677333	0.000449059
3h	ACA1_210540/clean/scf7180000084758:54354-54419:-	ACA1_210540	ABC transporter, ATPbinding domain containing protein [Source:UniProtKB/TrEMBL;Acc:L8GPZ1]	0.005952367	0.211534533	0.205582167	0.010845057
3h	ACA1_257170/clean/scf7180000084769:139618-139712:+	ACA1_257170	hypothetical protein	0.0282325	0.233783333	0.205550833	0.006904212
3h	ACA1_230470/clean/scf7180000084761:356093-356172:+	ACA1_230470	hypothetical protein	0.0233881	0.2289004	0.2055123	0.025586803
3h	ACA1_117330/clean/scf7180000084669:978175-978287:+	ACA1_117330	Hydroxylamine reductase [Source:UniProtKB/TrEMBL;Acc:L8H505]	0.007247873	0.212749967	0.205502093	1.25E-06
3h	ACA1_178240/clean/scf7180000084743:185734-185822:-	ACA1_178240	Fasciclin domain containing protein [Source:UniProtKB/TrEMBL;Acc:L8GT58]	0.024633	0.2299876	0.2053546	0.000119018
3h	ACA1_040720/clean/scf7180000084572:61455-61543:+	ACA1_040720	Adaptin_N domain-containing protein [Source:UniProtKB/TrEMBL;Acc:L8H2X2]	0.005443813	0.210732667	0.205288853	1.11E-05
3h	ACA1_232650/clean/scf7180000084762:54829-54910:-	ACA1_232650	AMP-binding domain-containing protein [Source:UniProtKB/TrEMBL;Acc:L8H2D5]	0.00058275	0.2056173	0.20503455	2.31E-08
3h	ACA1_071840/clean/scf7180000084603:1079679-1079749:+	ACA1_071840	DIL domain containing protein [Source:UniProtKB/TrEMBL;Acc:L8HEB0]	0.010472613	0.215055533	0.20458292	0.000434811
3h	ACA1_221050/clean/scf7180000084758:986819-986899:-	ACA1_221050	hypothetical protein	0.002400173	0.206758667	0.204358493	4.07E-05
3h	ACA1_072810/clean/scf7180000084603:1300841-1300929:+	ACA1_072810	Aha1_N domain-containing protein [Source:UniProtKB/TrEMBL;Acc:L8HG06]	0.000493761	0.204699433	0.204205672	2.63E-09
3h	ACA1_035590/clean/scf7180000084564:95776-95893:-	ACA1_035590	ANK_REP_REGION domain-containing protein [Source:UniProtKB/TrEMBL;Acc:L8H9N8]	0.042190253	0.246363767	0.204173513	0.013940409
3h	ACA1_064190/clean/scf7180000084599:1553501-1553594:+	ACA1_064190	hypothetical protein	0.01037414	0.214536933	0.204162793	7.83E-08
3h	ACA1_237810/clean/scf7180000084763:21205-21326:-	ACA1_237810	hypothetical protein	0.010511947	0.2145414	0.204029453	0.000310425
3h	ACA1_074110/clean/scf7180000084605:26182-26290:-	ACA1_074110	Peptidase [Source:UniProtKB/TrEMBL;Acc:L8HJ70]	0.005834113	0.209664267	0.203830153	1.53E-05
3h	ACA1_108780/clean/scf7180000084661:79955-80047:+	ACA1_108780	Phospholipase D, putative [Source:UniProtKB/TrEMBL;Acc:L8GD73]	0.007933423	0.211385333	0.20345191	3.44E-05
3h	ACA1_399880/clean/scf7180000084775:56220-56317:+	ACA1_399880	Rab-GAP TBC domain-containing protein [Source:UniProtKB/TrEMBL;Acc:L8GGF5]	0	0.203384767	0.203384767	3.19E-05
3h	ACA1_224860/clean/scf7180000084760:260976-261081:+	ACA1_224860	Myosin head (Motor domain) domain containing protein [Source:UniProtKB/TrEMBL;Acc:L8GSY9]	0	0.203380733	0.203380733	0.002834874
3h	ACA1_325260/clean/scf7180000084475:39977-40062:+	ACA1_325260	Peptidylprolyl isomerase [Source:UniProtKB/TrEMBL;Acc:L8GPH9]	0.00105799	0.2043898	0.20333181	1.22E-06
3h	ACA1_270300/clean/scf7180000084770:1105788-1105910:+	ACA1_270300	hypothetical protein	0.00304172	0.206241	0.20319928	4.67E-07
3h	ACA1_164970/clean/scf7180000084729:320298-320364:-	ACA1_164970	hypothetical protein	0.0158342	0.218960333	0.203126133	1.61E-06
3h	ACA1_068070/clean/scf7180000084603:99197-99259:+	ACA1_068070	Small GTPbinding ADP-ribosylation factor [Source:UniProtKB/TrEMBL;Acc:L8HEM7]	0.010548067	0.213671667	0.2031236	0.002189576
3h	ACA1_009930/clean/scf7180000083468:12811-12896:+	ACA1_009930	Acyl-CoA_dh_1 domain-containing protein [Source:UniProtKB/TrEMBL;Acc:L8GFW8]	0.00251001	0.205633233	0.203123223	3.27E-06
3h	ACA1_068080/clean/scf7180000084603:102798-102874:+	ACA1_068080	PlsC domain-containing protein [Source:UniProtKB/TrEMBL;Acc:L8HCG7]	0.011450357	0.214447833	0.202997477	1.97E-06
3h	ACA1_087400/clean/scf7180000084639:24414-24480:+	ACA1_087400	Ras subfamily protein [Source:UniProtKB/TrEMBL;Acc:L8GVE2]	0	0.202993867	0.202993867	0.002070208
3h	ACA1_158790/clean/scf7180000084724:257299-257398:+	ACA1_158790	Peroxiredoxin [Source:UniProtKB/TrEMBL;Acc:L8H9D0]	0.003663777	0.206556233	0.202892457	2.23E-07
3h	ACA1_187470/clean/scf7180000084747:72976-73063:-	ACA1_187470	hypothetical protein	0.0081052	0.2109598	0.2028546	0.000904954
3h	ACA1_091560/clean/scf7180000084645:150319-150426:+	ACA1_091560	UBP-type domain-containing protein [Source:UniProtKB/TrEMBL;Acc:L8GKE3]	0.025641033	0.228416967	0.202775933	0.016252647
3h	ACA1_130550/clean/scf7180000084698:105593-105687:-	ACA1_130550	WH2 domain-containing protein [Source:UniProtKB/TrEMBL;Acc:L8GPE2]	0.01796453	0.2206804	0.20271587	6.88E-05
3h	ACA1_237820/clean/scf7180000084763:24359-24518:-	ACA1_237820	NUDE_C domain-containing protein [Source:UniProtKB/TrEMBL;Acc:L8GKB8]	0.011671537	0.214369533	0.202697997	7.83E-05
3h	ACA1_098920/clean/scf7180000084646:63660-63773:-	ACA1_098920	DH domain-containing protein [Source:UniProtKB/TrEMBL;Acc:L8H5S9]	0	0.202662867	0.202662867	1.03E-07
3h	ACA1_063170/clean/scf7180000084599:1348430-1348493:-	ACA1_063170	hypothetical protein	0.004919397	0.207551333	0.202631937	4.96E-05
3h	ACA1_373600/clean/scf7180000084679:111497-111591:-	ACA1_373600	G domain-containing protein [Source:UniProtKB/TrEMBL;Acc:L8GJC7]	0.022216367	0.224829733	0.202613367	0.00654973
3h	ACA1_389690/clean/scf7180000084733:310399-310514:-	ACA1_389690	RBR-type E3 ubiquitin transferase [Source:UniProtKB/TrEMBL;Acc:L8GEU0]	0.001107303	0.203502367	0.202395063	3.91E-07
3h	ACA1_110290/clean/scf7180000084663:84603-84669:+	ACA1_110290	hypothetical protein	0.0416763	0.243867	0.2021907	0.001169977
3h	ACA1_368430/clean/scf7180000084664:121527-121624:-	ACA1_368430	Carnitine/acylcarnitine translocase, putative [Source:UniProtKB/TrEMBL;Acc:L8GYX3]	0.000447071	0.202580633	0.202133562	2.56E-09
3h	ACA1_369760/clean/scf7180000084664:374324-374432:+	ACA1_369760	hypothetical protein	0.00552243	0.2074978	0.20197537	0.002297501
3h	ACA1_227080/clean/scf7180000084761:94913-95013:+	ACA1_227080	Uracil-DNA glycosylase [Source:UniProtKB/TrEMBL;Acc:L8HA23]	0.003436433	0.205233333	0.2017969	0.000441655
3h	ACA1_275750/clean/scf7180000084774:98538-98656:-	ACA1_275750	unspecified product	0.019912227	0.221574933	0.201662707	0.006827469
3h	ACA1_080580/clean/scf7180000084618:38681-38763:+	ACA1_080580	t-SNARE coiled-coil homology domain-containing protein [Source:UniProtKB/TrEMBL;Acc:L8HBP6]	0.002353053	0.2039772	0.201624147	8.84E-08
3h	ACA1_023870/clean/scf7180000084436:28036-28142:-	ACA1_023870	M20_dimer domain-containing protein [Source:UniProtKB/TrEMBL;Acc:L8GSF7]	0.026736233	0.228244333	0.2015081	0.000192959
3h	ACA1_036530/clean/scf7180000084565:132445-132538:+	ACA1_036530	CRT-like domain-containing protein [Source:UniProtKB/TrEMBL;Acc:L8HCN5]	0.0111695	0.212646767	0.201477267	7.33E-06
3h	ACA1_173960/clean/scf7180000084741:560758-560883:+	ACA1_173960	AB hydrolase-1 domain-containing protein [Source:UniProtKB/TrEMBL;Acc:L8HJ69]	0.007467687	0.208590267	0.20112258	5.86E-05
3h	ACA1_181830/clean/scf7180000084745:54159-54245:-	ACA1_181830	PIPK domain-containing protein [Source:UniProtKB/TrEMBL;Acc:L8H972]	0	0.200965267	0.200965267	0.001245761
3h	ACA1_158170/clean/scf7180000084724:202227-202379:-	ACA1_158170	Pescadillo homolog [Source:UniProtKB/TrEMBL;Acc:L8HBG6]	0.002927486	0.203704433	0.200776947	1.09E-05
3h	ACA1_360100/clean/scf7180000084622:49539-49607:+	ACA1_360100	hypothetical protein	0	0.200604333	0.200604333	8.05E-05
3h	ACA1_383710/clean/scf7180000084721:250297-250361:+	ACA1_383710	Glucose-6-phosphate 1-dehydrogenase [Source:UniProtKB/TrEMBL;Acc:L8GW58]	0	0.200576667	0.200576667	7.77E-09
3h	ACA1_141950/clean/scf7180000084708:57803-57899:-	ACA1_141950	unspecified product	0.001885556	0.202450933	0.200565378	7.57E-09
3h	ACA1_013340/clean/scf7180000083750:1829-1917:+	ACA1_013340	PH domain-containing protein [Source:UniProtKB/TrEMBL;Acc:L8HGJ7]	0.001934893	0.202441167	0.200506273	1.48E-06
3h	ACA1_361100/clean/scf7180000084622:241835-241925:-	ACA1_361100	TLDc domain-containing protein [Source:UniProtKB/TrEMBL;Acc:L8HE93]	0.00389767	0.204353267	0.200455597	6.70E-05
3h	ACA1_194960/clean/scf7180000084751:41331-41412:+	ACA1_194960	Myotubularin phosphatase domain-containing protein [Source:UniProtKB/TrEMBL;Acc:L8H4F9]	0.088533533	0.288983	0.200449467	0.009307185
3h	ACA1_258270/clean/scf7180000084769:219317-219437:+	ACA1_258270	Ras subfamily protein [Source:UniProtKB/TrEMBL;Acc:L8GHB6]	0.018886467	0.2193314	0.200444933	6.95E-06
3h	ACA1_269320/clean/scf7180000084770:971642-971721:-	ACA1_269320	eIF2 gamma, putative [Source:UniProtKB/TrEMBL;Acc:L8H4Y2]	0.004429263	0.204864133	0.20043487	1.33E-07
3h	ACA1_199970/clean/scf7180000084753:362016-362122:-	ACA1_199970	Glycoside hydrolase family protein [Source:UniProtKB/TrEMBL;Acc:L8H5H5]	0.000729429	0.2011087	0.200379271	3.81E-08
3h	ACA1_078990/clean/scf7180000084615:125843-125973:-	ACA1_078990	AcylCoA synthetase, putative [Source:UniProtKB/TrEMBL;Acc:L8GRK9]	0.000705423	0.2008953	0.200189877	1.77E-08
3h	ACA1_063050/clean/scf7180000084599:1318057-1318181:+	ACA1_063050	hypothetical protein	0.016241247	0.2163972	0.200155953	0.001339565
3h	ACA1_153010/clean/scf7180000084720:53403-53516:-	ACA1_153010	hypothetical protein	0.002211843	0.202346733	0.20013489	2.13E-07
3h	ACA1_113780/clean/scf7180000084669:371922-372012:+	ACA1_113780	COP9 signalosome complex subunit 6 [Source:UniProtKB/TrEMBL;Acc:L8H4A1]	0.010639237	0.210722867	0.20008363	2.47E-05
3h	ACA1_387910/clean/scf7180000084733:88145-88247:-	ACA1_387910	P/Homo B domain-containing protein [Source:UniProtKB/TrEMBL;Acc:L8GG58]	0.003137413	0.203194867	0.200057453	0.000117846
3h	ACA1_362820/clean/scf7180000084624:189010-189094:+	ACA1_362820	hypothetical protein	0.022097933	0.222	0.199902067	5.92E-05
3h	ACA1_166070/clean/scf7180000084732:110986-111081:-	ACA1_166070	Malate dehydrogenase [Source:UniProtKB/TrEMBL;Acc:L8HIY2]	0.00472806	0.204598333	0.199870273	9.69E-07
3h	ACA1_060380/clean/scf7180000084599:663405-663481:-	ACA1_060380	ATG13 domain-containing protein [Source:UniProtKB/TrEMBL;Acc:L8GYW8]	0.0221305	0.221988467	0.199857967	0.000239879
3h	ACA1_219480/clean/scf7180000084758:745535-745616:+	ACA1_219480	hypothetical protein	0	0.199484867	0.199484867	6.68E-07
3h	ACA1_238590/clean/scf7180000084763:103376-103483:+	ACA1_238590	CBS domain containing protein [Source:UniProtKB/TrEMBL;Acc:L8GKE6]	0.0320291	0.2314719	0.1994428	0.000925248
3h	ACA1_358630/clean/scf7180000084742:15864-15957:-	ACA1_358630	hypothetical protein	0.0351923	0.234555867	0.199363567	0.003741226
3h	ACA1_398190/clean/scf7180000084759:212146-212234:-	ACA1_398190	6-phosphogluconate dehydrogenase, decarboxylating [Source:UniProtKB/TrEMBL;Acc:L8HDP8]	0.079724933	0.278948333	0.1992234	0.017612786
3h	ACA1_059540/clean/scf7180000084599:463534-463649:+	ACA1_059540	AMP_N domain-containing protein [Source:UniProtKB/TrEMBL;Acc:L8GWD9]	0.002633177	0.201551333	0.198918156	5.18E-08
3h	ACA1_113530/clean/scf7180000084669:310396-310455:+	ACA1_113530	Isopentenyltransferase [Source:UniProtKB/TrEMBL;Acc:L8H5K6]	0.103699733	0.302591033	0.1988913	0.023569876
3h	ACA1_276370/clean/scf7180000084774:156557-156680:-	ACA1_276370	unspecified product	0.024416567	0.223059233	0.198642667	0.003795816
3h	ACA1_292880/clean/scf7180000084777:923265-923348:-	ACA1_292880	RhoGEF domain containing protein [Source:UniProtKB/TrEMBL;Acc:L8HJL4]	0.026667267	0.225215533	0.198548267	0.001376322
3h	ACA1_229190/clean/scf7180000084761:240650-240754:+	ACA1_229190	MYND-type domain-containing protein [Source:UniProtKB/TrEMBL;Acc:L8HA97]	0.000353418	0.198880867	0.198527448	2.08E-07
3h	ACA1_009890/clean/scf7180000083468:2256-2622:-	ACA1_009890	TPR_REGION domain-containing protein [Source:UniProtKB/TrEMBL;Acc:L8GDE6]	0.0071188	0.2055928	0.198474	2.31E-05
3h	ACA1_277780/clean/scf7180000084776:99500-99598:+	ACA1_277780	Ca2+dependent endoplasmic reticulum nucleoside diphosphatase isoform 3, putative [Source:UniProtKB/TrEMBL;Acc:L8H7Y3]	0.02039759	0.218748533	0.198350943	0.001395077
3h	ACA1_210800/clean/scf7180000084758:68104-68174:+	ACA1_210800	DPBB_1 domain-containing protein [Source:UniProtKB/TrEMBL;Acc:L8GQQ9]	0.006628387	0.204950667	0.19832228	1.21E-05
3h	ACA1_268920/clean/scf7180000084770:939702-939858:-	ACA1_268920	Transket_pyr domain-containing protein [Source:UniProtKB/TrEMBL;Acc:L8H4W8]	0.014259933	0.212574333	0.1983144	7.40E-05
3h	ACA1_360190/clean/scf7180000084622:68033-68102:+	ACA1_360190	Amino_oxidase domain-containing protein [Source:UniProtKB/TrEMBL;Acc:L8HBU7]	0.005037717	0.203334667	0.19829695	9.23E-08
3h	ACA1_150560/clean/scf7180000084716:12630-12695:-	ACA1_150560	hypothetical protein	0.040972133	0.239258767	0.198286633	0.004344916
3h	ACA1_040760/clean/scf7180000084572:76175-76268:-	ACA1_040760	SNF7 family protein [Source:UniProtKB/TrEMBL;Acc:L8H0J8]	0.00515613	0.203197	0.19804087	2.22E-07
3h	ACA1_019010/clean/scf7180000083912:46811-46906:-	ACA1_019010	hypothetical protein	0.044779667	0.2427352	0.197955533	0.000592662
3h	ACA1_291510/clean/scf7180000084777:765531-765647:+	ACA1_291510	Ubiquitin carboxylterminal hydrolase 15, putative [Source:UniProtKB/TrEMBL;Acc:L8HKZ1]	0.006884233	0.204542933	0.1976587	1.03E-05
3h	ACA1_380930/clean/scf7180000084712:117716-117848:-	ACA1_380930	hypothetical protein	0.041847533	0.239360033	0.1975125	0.003775189
3h	ACA1_037900/clean/scf7180000084568:54138-54210:-	ACA1_037900	Nudix hydrolase domain-containing protein [Source:UniProtKB/TrEMBL;Acc:L8GKU1]	0.022222233	0.2197306	0.197508367	0.041106018
3h	ACA1_194910/clean/scf7180000084751:28018-28080:-	ACA1_194910	Alkaline phosphatase [Source:UniProtKB/TrEMBL;Acc:L8H4F3]	0.0136613	0.211045333	0.197384033	1.58E-06
3h	ACA1_278760/clean/scf7180000084776:238031-238115:+	ACA1_278760	Protein kinase domain-containing protein [Source:UniProtKB/TrEMBL;Acc:L8H6Y1]	0.015609337	0.2128166	0.197207263	0.000510937
3h	ACA1_347020/clean/scf7180000084665:2837-2922:-	ACA1_347020	VWFA domain-containing protein [Source:UniProtKB/TrEMBL;Acc:L8GVX0]	0.011574897	0.2087633	0.197188403	7.36E-06
3h	ACA1_286170/clean/scf7180000084776:1051931-1052019:+	ACA1_286170	Peptidase_S9 domain-containing protein [Source:UniProtKB/TrEMBL;Acc:L8H7U8]	0.0416559	0.238838	0.1971821	0.002164149
3h	ACA1_394860/clean/scf7180000084756:138890-138967:+	ACA1_394860	PA domain containing protein [Source:UniProtKB/TrEMBL;Acc:L8GZN2]	0.009431267	0.206602133	0.197170867	5.85E-06
3h	ACA1_392960/clean/scf7180000084756:14970-15040:-	ACA1_392960	Usp domain-containing protein [Source:UniProtKB/TrEMBL;Acc:L8H097]	0.011085333	0.208238633	0.1971533	0.011952049
3h	ACA1_146630/clean/scf7180000084713:80566-80685:+	ACA1_146630	hypothetical protein	0.082523433	0.279667667	0.197144233	0.009692436
3h	ACA1_367940/clean/scf7180000084664:17646-17792:+	ACA1_367940	ATP synthase subunit beta [Source:UniProtKB/TrEMBL;Acc:L8GXZ0]	0.00091992	0.197980367	0.197060447	2.30E-11
3h	ACA1_109770/clean/scf7180000084663:22620-22713:+	ACA1_109770	WD40 repeat-containing protein [Source:UniProtKB/TrEMBL;Acc:L8HKT6]	0.004090213	0.201115	0.197024787	6.38E-06
3h	ACA1_221050/clean/scf7180000084758:986533-986598:-	ACA1_221050	hypothetical protein	0.00046555	0.1974843	0.19701875	0.000141333
3h	ACA1_132870/clean/scf7180000084700:89831-89932:+	ACA1_132870	Chromatin modifying protein 1b, putative [Source:UniProtKB/TrEMBL;Acc:L8GY90]	0.00203252	0.1988391	0.19680658	5.82E-06
3h	ACA1_165210/clean/scf7180000084732:7959-8053:-	ACA1_165210	Ras subfamily protein [Source:UniProtKB/TrEMBL;Acc:L8HGF1]	0.003284407	0.2000884	0.196803993	3.49E-08

3h	ACA1_266770/clean/scf7180000084770:708640-708720:+	ACA1_266770	Fe2OG dioxygenase domain-containing protein [Source:UniProtKB/TrEMBL;Acc:L8H2F4]	0.016000547	0.212627	0.196626453	4.38E-05
3h	ACA1_237850/clean/scf7180000084763:29903-30035:+	ACA1_237850	BTB/POZ domain containing protein [Source:UniProtKB/TrEMBL;Acc:L8GJT8]	0	0.196577833	0.196577833	1.61E-08
3h	ACA1_341070/clean/scf7180000084620:45102-45181:-	ACA1_341070	Peroxisomal membrane 22 kDa family protein [Source:UniProtKB/TrEMBL;Acc:L8GRB3]	0.007171173	0.203642567	0.196471393	1.31E-05
3h	ACA1_007740/clean/scf7180000083166:6777-6864:-	ACA1_007740	RasGEF domain containing protein [Source:UniProtKB/TrEMBL;Acc:L8HBV5]	0.003174603	0.199636433	0.19646183	0.000279386
3h	ACA1_098160/clean/scf7180000084645:1410853-1410914:-	ACA1_098160	ABC transporter, ATPbinding domain containing protein [Source:UniProtKB/TrEMBL;Acc:L8GJY4]	0.00125786	0.197718967	0.196461107	1.99E-06
3h	ACA1_301570/clean/scf7180000083222:10779-10887:-	ACA1_301570	FCP1 homology domain-containing protein [Source:UniProtKB/TrEMBL;Acc:L8HKQ0]	0.010829833	0.207260667	0.196430833	1.71E-06
3h	ACA1_091340/clean/scf7180000084645:92744-92876:+	ACA1_091340	UDENN domain-containing protein [Source:UniProtKB/TrEMBL;Acc:L8GIG9]	0.011633333	0.208022333	0.196389	0.010385404
3h	ACA1_278150/clean/scf7180000084776:180648-180721:-	ACA1_278150	hypothetical protein	0.003324337	0.199594333	0.196269997	5.05E-07
3h	ACA1_334660/clean/scf7180000084586:3012-3090:+	ACA1_334660	hypothetical protein	0.005165197	0.201256167	0.19609097	7.78E-07
3h	ACA1_208580/clean/scf7180000084757:228036-228141:-	ACA1_208580	GPI anchored protein [Source:UniProtKB/TrEMBL;Acc:L8GZM6]	0.000161137	0.1962154	0.196054263	1.68E-08
3h	ACA1_143810/clean/scf7180000084709:102701-102790:-	ACA1_143810	hypothetical protein	0.009422103	0.205445833	0.19602373	2.47E-07
3h	ACA1_381110/clean/scf7180000084712:146638-146711:+	ACA1_381110	PKS_ER domain-containing protein [Source:UniProtKB/TrEMBL;Acc:L8GNM8]	0.008777423	0.204783	0.196005577	3.80E-06
3h	ACA1_287540/clean/scf7180000084777:32340-32429:-	ACA1_287540	Snurportin1 domain-containing protein [Source:UniProtKB/TrEMBL;Acc:L8HIM9]	0.022419467	0.218385667	0.1959662	0.001284563
3h	ACA1_238390/clean/scf7180000084763:82261-82398:-	ACA1_238390	Coronin [Source:UniProtKB/TrEMBL;Acc:L8GJQ2]	0.001195162	0.1971235	0.195928338	1.69E-07
3h	ACA1_158910/clean/scf7180000084724:296040-296193:-	ACA1_158910	hypothetical protein	0.058259267	0.254084933	0.195825667	0.00749327
3h	ACA1_192080/clean/scf7180000084749:95810-95958:-	ACA1_192080	hypothetical protein	0.008289553	0.2041123	0.195822747	4.35E-06
3h	ACA1_113520/clean/scf7180000084669:308322-308394:-	ACA1_113520	Oraov1 protein [Source:UniProtKB/TrEMBL;Acc:L8H472]	0.007287573	0.202908467	0.195620893	3.90E-06
3h	ACA1_119410/clean/scf7180000084672:64439-64535:+	ACA1_119410	PCI domain-containing protein [Source:UniProtKB/TrEMBL;Acc:L8HLX4]	0.003896097	0.199475367	0.19557927	1.83E-07
3h	ACA1_165210/clean/scf7180000084732:7703-7779:-	ACA1_165210	Ras subfamily protein [Source:UniProtKB/TrEMBL;Acc:L8HGF1]	0.009472143	0.205017433	0.19554529	6.81E-06
3h	ACA1_012020/clean/scf7180000083652:4590-4683:-	ACA1_012020	hypothetical protein	0	0.195496567	0.195496567	4.28E-07
3h	ACA1_188290/clean/scf7180000084747:146825-146901:+	ACA1_188290	IPT/TIG domain-containing protein [Source:UniProtKB/TrEMBL;Acc:L8GSG4]	0.005836877	0.201276833	0.195439957	5.04E-06
3h	ACA1_210670/clean/scf7180000084758:62506-62617:-	ACA1_210670	Usp domain-containing protein [Source:UniProtKB/TrEMBL;Acc:L8GPP6]	0.041516867	0.236948467	0.1954316	0.023909503
3h	ACA1_040840/clean/scf7180000084572:92518-92609:-	ACA1_040840	BRO1 domain-containing protein [Source:UniProtKB/TrEMBL;Acc:L8H1X9]	0.00775461	0.203172133	0.195417523	6.11E-05
3h	ACA1_149420/clean/scf7180000084715:149243-149369:-	ACA1_149420	Ribosome assembly factor mrt4 [Source:UniProtKB/TrEMBL;Acc:L8HB77]	0.00263433	0.198050867	0.195416537	2.98E-07
3h	ACA1_384600/clean/scf7180000084730:61769-61847:-	ACA1_384600	hypothetical protein	0.00593132	0.201323433	0.195392113	0.000169231
3h	ACA1_142680/clean/scf7180000084708:219402-219562:+	ACA1_142680	unspecified product	0.016951567	0.212292	0.195340433	1.14E-05
3h	ACA1_094130/clean/scf7180000084645:696553-696660:-	ACA1_094130	Cytochrome b561 domain-containing protein [Source:UniProtKB/TrEMBL;Acc:L8GKY0]	0.01382768	0.2090731	0.19524542	0.000378869
3h	ACA1_325420/clean/scf7180000084495:4408-4531:+	ACA1_325420	Nicotinate-nucleotide pyrophosphorylase [carboxylating] [Source:UniProtKB/TrEMBL;Acc:L8H9V7]	0.000561965	0.195714533	0.195152568	3.99E-08
3h	ACA1_117320/clean/scf7180000084669:974148-974221:-	ACA1_117320	FAD binding domain containing protein [Source:UniProtKB/TrEMBL;Acc:L8H415]	0.0315172	0.226596633	0.195079433	0.003715121
3h	ACA1_051530/clean/scf7180000084592:30114-30200:-	ACA1_051530	MFS domain-containing protein [Source:UniProtKB/TrEMBL;Acc:L8GPJ8]	0	0.195056233	0.195056233	0.000112308
3h	ACA1_322390/clean/scf7180000084463:13841-13933:+	ACA1_322390	DIL domain containing protein [Source:UniProtKB/TrEMBL;Acc:L8HH10]	0.00085597	0.195906	0.19505003	1.58E-05
3h	ACA1_216320/clean/scf7180000084758:467616-467725:+	ACA1_216320	Homogentisate 1,2dioxygenase [Source:UniProtKB/TrEMBL;Acc:L8GQ83]	7.94E-05	0.194973333	0.194893942	1.64E-08
3h	ACA1_073390/clean/scf7180000084603:1408426-1408520:+	ACA1_073390	hypothetical protein	0.003197685	0.1980729	0.194875215	1.42E-05
3h	ACA1_002450/clean/scf7180000082931:3780-3844:+	ACA1_002450	eIF-5a domain-containing protein [Source:UniProtKB/TrEMBL;Acc:L8GWN9]	0.118922467	0.313755333	0.194832867	0.042838554
3h	ACA1_153070/clean/scf7180000084720:77243-77349:+	ACA1_153070	J domain-containing protein [Source:UniProtKB/TrEMBL;Acc:L8HHE7]	0.00620763	0.201023933	0.194816303	3.45E-05
3h	ACA1_057100/clean/scf7180000084599:42449-42534:-	ACA1_057100	unspecified product	0.004993611	0.1997271	0.194733489	0.00043704
3h	ACA1_385700/clean/scf7180000084730:240963-241036:+	ACA1_385700	F-box domain-containing protein [Source:UniProtKB/TrEMBL;Acc:L8HAP4]	0.007546333	0.202240533	0.1946942	0.025038213
3h	ACA1_232810/clean/scf7180000084762:74570-74648:+	ACA1_232810	CHY-type domain-containing protein [Source:UniProtKB/TrEMBL;Acc:L8H073]	0.07746	0.272127933	0.194667933	0.030423512
3h	ACA1_399200/clean/scf7180000084759:269871-269967:-	ACA1_399200	hypothetical protein	0.001097621	0.195537033	0.194439412	1.15E-07
3h	ACA1_113870/clean/scf7180000084669:393544-393624:+	ACA1_113870	CUB domain containing protein [Source:UniProtKB/TrEMBL;Acc:L8H6F7]	0.009796845	0.204126067	0.194329221	0.000823913
3h	ACA1_204010/clean/scf7180000084754:246778-246852:-	ACA1_204010	RAC GTPase [Source:UniProtKB/TrEMBL;Acc:L8GW76]	0.00496976	0.1992735	0.19430374	3.48E-09
3h	ACA1_306840/clean/scf7180000083668:18793-18876:+	ACA1_306840	RTP1_C1 domain-containing protein [Source:UniProtKB/TrEMBL;Acc:L8GQF6]	0	0.1942892	0.1942892	0.001008711
3h	ACA1_358630/clean/scf7180000084742:16414-16499:-	ACA1_358630	hypothetical protein	0.00068236	0.1949392	0.19425684	1.79E-05
3h	ACA1_377400/clean/scf7180000084711:52324-52425:+	ACA1_377400	Plexin repeat domain containing protein [Source:UniProtKB/TrEMBL;Acc:L8GU69]	0.00284396	0.197080367	0.194236407	5.42E-05
3h	ACA1_012970/clean/scf7180000083742:13880-13974:+	ACA1_012970	CBS domain containing protein [Source:UniProtKB/TrEMBL;Acc:L8H720]	0.096357733	0.290400333	0.1940426	0.042663784
3h	ACA1_036520/clean/scf7180000084565:130104-130205:+	ACA1_036520	WD domain, G-beta repeat-containing protein [Source:UniProtKB/TrEMBL;Acc:L8HBN8]	0.019193117	0.213143067	0.19394995	0.000181796
3h	ACA1_165830/clean/scf7180000084732:97987-98105:-	ACA1_165830	Glutathione transferase family protein [Source:UniProtKB/TrEMBL;Acc:L8HGI5]	0.004206397	0.198126833	0.193920437	1.91E-05
3h	ACA1_144390/clean/scf7180000084709:228077-228367:-	ACA1_144390	Leucine rich repeat domain containing protein [Source:UniProtKB/TrEMBL;Acc:L8HH87]	0.00553056	0.199425067	0.193894507	1.67E-05
3h	ACA1_106000/clean/scf7180000084660:50405-50478:+	ACA1_106000	HEAT repeat domain containing protein [Source:UniProtKB/TrEMBL;Acc:L8GN71]	0.002150537	0.196038667	0.19388813	1.08E-05
3h	ACA1_255500/clean/scf7180000084768:516757-516872:+	ACA1_255500	Peptidase C83 domain-containing protein [Source:UniProtKB/TrEMBL;Acc:L8HB52]	0.0193405	0.213218367	0.193877867	0.002769286
3h	ACA1_268930/clean/scf7180000084770:945260-945362:+	ACA1_268930	PWI domain-containing protein [Source:UniProtKB/TrEMBL;Acc:L8H2N5]	0.004658011	0.198225667	0.193567656	3.06E-05
3h	ACA1_285200/clean/scf7180000084776:957273-957359:-	ACA1_285200	Na_H_Exchanger domain-containing protein [Source:UniProtKB/TrEMBL;Acc:L8H9U2]	0.006318463	0.199867333	0.19354887	1.37E-05
3h	ACA1_382580/clean/scf7180000084721:76768-76857:+	ACA1_382580	LBP / BPI / CETP family, Cterminal domain containing protein [Source:UniProtKB/TrEMBL;Acc:L8GVZ1]	0.050346033	0.243846033	0.1935	0.001046001
3h	ACA1_000580/clean/scf7180000082784:8614-8771:-	ACA1_000580	hypothetical protein	0.00981994	0.203289433	0.193469493	6.59E-05
3h	ACA1_186060/clean/scf7180000084746:161033-161111:-	ACA1_186060	Dual specificity phosphatase, catalytic domain containing protein [Source:UniProtKB/TrEMBL;Acc:L8H5A1]	0.029474267	0.2228094	0.193335133	0.006477511
3h	ACA1_077300/clean/scf7180000084612:292009-292081:+	ACA1_077300	Serine/threonine kinase [Source:UniProtKB/TrEMBL;Acc:L8GP03]	0.010847977	0.2039039	0.193055923	0.003856432
3h	ACA1_282720/clean/scf7180000084776:724464-724531:-	ACA1_282720	Leucine rich repeat domain containing protein [Source:UniProtKB/TrEMBL;Acc:L8H9L8]	0.008130067	0.201064033	0.192933967	0.004435025
3h	ACA1_201400/clean/scf7180000084753:543440-543518:+	ACA1_201400	CBS domain containing protein [Source:UniProtKB/TrEMBL;Acc:L8H3G1]	0.00763383	0.2004656	0.19283177	3.18E-06
3h	ACA1_309360/clean/scf7180000083758:12173-12249:-	ACA1_309360	hypothetical protein	0.008130067	0.2009266	0.192796533	0.008909087
3h	ACA1_224580/clean/scf7180000084760:236398-236487:-	ACA1_224580	hypothetical protein	0.043130733	0.235919567	0.192788833	0.001769585
3h	ACA1_279530/clean/scf7180000084776:410046-410125:+	ACA1_279530	Rab4, putative [Source:UniProtKB/TrEMBL;Acc:L8H946]	0.00413135	0.196798667	0.192667316	6.18E-06
3h	ACA1_292880/clean/scf7180000084777:924075-924183:-	ACA1_292880	RhoGEF domain containing protein [Source:UniProtKB/TrEMBL;Acc:L8HJL4]	0.009434047	0.2020997	0.192665653	0.001156053
3h	ACA1_050330/clean/scf7180000084589:64634-64717:-	ACA1_050330	DUF3825 domain-containing protein [Source:UniProtKB/TrEMBL;Acc:L8H3U0]	4.61E-05	0.192655867	0.19260975	2.96E-06
3h	ACA1_075030/clean/scf7180000084608:130520-130604:+	ACA1_075030	Serine carboxypeptidase [Source:UniProtKB/TrEMBL;Acc:L8HFJ6]	0.000774833	0.1933005	0.192525667	2.67E-06
3h	ACA1_271500/clean/scf7180000084771:45198-45365:+	ACA1_271500	Leucine rich repeat domain containing protein [Source:UniProtKB/TrEMBL;Acc:L8GNI4]	0.009923223	0.202394833	0.19247161	0.001014356
3h	ACA1_382280/clean/scf7180000084721:20453-20572:-	ACA1_382280	Aminotran_1_2 domain-containing protein [Source:UniProtKB/TrEMBL;Acc:L8GUT4]	0.011086467	0.203475367	0.1923889	0.004895466
3h	ACA1_220200/clean/scf7180000084758:896119-896189:+	ACA1_220200	Aminopeptidase [Source:UniProtKB/TrEMBL;Acc:L8GT26]	0.001108347	0.1934181	0.192309753	1.67E-07
3h	ACA1_107540/clean/scf7180000084660:218088-218181:+	ACA1_107540	hypothetical protein	0.037835833	0.229954667	0.192118833	0.026506413
3h	ACA1_270200/clean/scf7180000084770:1076690-1076775:-	ACA1_270200	FYVE-type domain-containing protein [Source:UniProtKB/TrEMBL;Acc:L8H522]	0	0.1920539	0.1920539	0.016563107
3h	ACA1_024630/clean/scf7180000084444:2719-2783:+	ACA1_024630	hypothetical protein	0.031583633	0.2236053	0.192021667	0.001967919
3h	ACA1_355980/clean/scf7180000084728:40807-40921:+	ACA1_355980	Aa_trans domain-containing protein [Source:UniProtKB/TrEMBL;Acc:L8H7L7]	0.046366117	0.2379891	0.191622983	0.011514628
3h	ACA1_240830/clean/scf7180000084763:243241-243315:-	ACA1_240830	EFG_C domain-containing protein [Source:UniProtKB/TrEMBL;Acc:L8GMB2]	0.0308827	0.2223809	0.1914982	0.012866524
3h	ACA1_116020/clean/scf7180000084669:706837-706933:+	ACA1_116020	Rho-GAP domain-containing protein [Source:UniProtKB/TrEMBL;Acc:L8H6T0]	0.00141243	0.192896733	0.191484303	9.29E-06
3h	ACA1_261370/clean/scf7180000084769:653328-653449:+	ACA1_261370	Regulator of chromosome condensation (RCC1) repeat domain containing protein [Source:UniProtKB/TrEMBL;Acc:L8GFF1]	0.012516987	0.203974233	0.191457247	8.17E-05
3h	ACA1_224400/clean/scf7180000084760:193198-193298:+	ACA1_224400	F_actin_bund_C domain-containing protein [Source:UniProtKB/TrEMBL;Acc:L8GVG0]	0.001717367	0.193130333	0.191412967	5.83E-10
3h	ACA1_282270/clean/scf7180000084776:641701-641774:-	ACA1_282270	hypothetical protein	0.00294193	0.1943395	0.19139757	0.000391929
3h	ACA1_278900/clean/scf7180000084776:273223-273301:+	ACA1_278900	ANK_REP_REGION domain-containing protein [Source:UniProtKB/TrEMBL;Acc:L8H5W6]	0.016990533	0.2083469	0.191356367	9.84E-05
3h	ACA1_269310/clean/scf7180000084770:969460-969556:-	ACA1_269310	RING-type domain-containing protein [Source:UniProtKB/TrEMBL;Acc:L8H2R5]	0	0.191344667	0.191344667	6.06E-07
3h	ACA1_035470/clean/scf7180000084564:58982-59064:+	ACA1_035470	Cation exchanger, putative [Source:UniProtKB/TrEMBL;Acc:L8HAF4]	0	0.191320233	0.191320233	1.20E-06
3h	ACA1_060350/clean/scf7180000084599:653703-653853:+	ACA1_060350	Rho-GAP domain-containing protein [Source:UniProtKB/TrEMBL;Acc:L8GXL3]	0.009938867	0.201253933	0.191315067	0.000584493
3h	ACA1_277720/clean/scf7180000084776:87286-87350:+	ACA1_277720	CBS domain containing protein [Source:UniProtKB/TrEMBL;Acc:L8H7X6]	0	0.191285833	0.191285833	0.000780979
3h	ACA1_275570/clean/scf7180000084774:44084-44190:-	ACA1_275570	unspecified product	0	0.191250133	0.191250133	1.15E-05
3h	ACA1_033610/clean/scf7180000084559:57895-57985:+	ACA1_033610	Sortilin [Source:UniProtKB/TrEMBL;Acc:L8GHJ0]	0.001216547	0.192465633	0.191249087	1.99E-06
3h	ACA1_273770/clean/scf7180000084773:53224-53289:-	ACA1_273770	hypothetical protein	0.001036686	0.192254633	0.191217947	1.28E-16
3h	ACA1_097490/clean/scf7180000084645:1253825-1253922:-	ACA1_097490	hypothetical protein	0.0144438	0.205512667	0.191068867	8.38E-06
3h	ACA1_061250/clean/scf7180000084599:866886-866960:+	ACA1_061250	Peptidyl-prolyl cis-trans isomerase [Source:UniProtKB/TrEMBL;Acc:L8GWR2]	0.00521212	0.196263667	0.191051547	2.79E-07
3h	ACA1_252210/clean/scf7180000084768:146383-146465:+	ACA1_252210	Protein kinase domain-containing protein [Source:UniProtKB/TrEMBL;Acc:L8HAM0]	0	0.191006633	0.191006633	0.000628218
3h	ACA1_136070/clean/scf7180000084702:124561-124671:-	ACA1_136070	Ribonuclease H1/H2 small subunit [Source:UniProtKB/TrEMBL;Acc:L8GEE5]	0.01694932	0.207798433	0.190849113	0.001383718
3h	ACA1_183240/clean/scf7180000084745:361472-361559:+	ACA1_183240	LysM domain-containing protein [Source:UniProtKB/TrEMBL;Acc:L8H898]	0.000363107	0.1911644	0.190801293	1.79E-06
3h	ACA1_322570/clean/scf7180000084466:21627-21768:+	ACA1_322570	Hsp40, putative [Source:UniProtKB/TrEMBL;Acc:L8HCJ2]	0.000843213	0.191604273	0.19076106	4.71E-07
3h	ACA1_300880/clean/scf7180000083147:14634-14856:-	ACA1_300880	Valine-tRNA ligase [Source:UniProtKB/TrEMBL;Acc:L8HGI8]	0.00640866	0.197127667	0.190719007	2.23E-06
3h	ACA1_248670/clean/scf7180000084766:106904-106975:-	ACA1_248670	CBS domain containing protein [Source:UniProtKB/TrEMBL;Acc:L8GY23]	0.006958193	0.197647333	0.19068914	2.36E-06
3h	ACA1_217760/clean/scf7180000084758:605754-605877:+	ACA1_217760	hypothetical protein	4.27E-05	0.190588733	0.190545987	1.14E-05
3h	ACA1_074750/clean/scf7180000084608:76422-76526:-	ACA1_074750	5formyltetrahydrofolate cyclo-ligase subfamily protein [Source:UniProtKB/TrEMBL;Acc:L8HGR6]	0.00846557	0.198986333	0.190520763	1.32E-07
3h	ACA1_228520/clean/scf7180000084761:179814-179889:-	ACA1_228520	ANK_REP_REGION domain-containing protein [Source:UniProtKB/TrEMBL;Acc:L8HB48]	0.010730533	0.201240333	0.1905098	9.46E-06
3h	ACA1_087390/clean/scf7180000084639:22901-22967:+	ACA1_087390	ANK_REP_REGION domain-containing protein [Source:UniProtKB/TrEMBL;Acc:L8GUE4]	0.000752823	0.191051533	0.19029871	3.93E-05
3h	ACA1_237820/clean/scf7180000084763:24036-24122:-	ACA1_237820	NUDE_C domain-containing protein [Source:UniProtKB/TrEMBL;Acc:L8GKB8]	0.01048928	0.2007442	0.19025492	0.000332762
3h	ACA1_114650/clean/scf7180000084669:531548-531644:-	ACA1_114650	hypothetical protein	0.0139594	0.2042102	0.1902508	9.34E-07
3h	ACA1_095670/clean/scf7180000084645:876554-876612:-	ACA1_095670	FAD-binding PCMH-type domain-containing protein [Source:UniProtKB/TrEMBL;Acc:L8GIT7]	0.039874533	0.229987067	0.190112533	0.003394664
3h	ACA1_296120/clean/scf7180000084777:1300020-1300387:+	ACA1_296120	hypothetical protein	0.020590767	0.210643733	0.190052967	0.033813561
3h	ACA1_164750/clean/scf7180000084729:265699-265771:-	ACA1_164750	Carbonic anhydrase [Source:UniProtKB/TrEMBL;Acc:L8GR38]	0.002380213	0.192430167	0.190049953	1.70E-06
3h	ACA1_234770/clean/scf7180000084762:314972-315058:-	ACA1_234770	SGF29 C-terminal domain-containing protein [Source:UniProtKB/TrEMBL;Acc:L8H0I6]	0.00690026	0.196939133	0.190038873	3.54E-07
3h	ACA1_389150/clean/scf7180000084733:212916-212987:+	ACA1_389150	Tasp1 protein [Source:UniProtKB/TrEMBL;Acc:L8GEC7]	0.013979633	0.2038608	0.189881167	0.000575152
3h	ACA1_282440/clean/scf7180000084776:681213-681311:+	ACA1_282440	hypothetical protein	0.009170933	0.198998633	0.1898277	0.001197652
3h	ACA1_054030/clean/scf7180000084594:376842-376925:-	ACA1_054030	PH domain-containing protein [Source:UniProtKB/TrEMBL;Acc:L8H587]	0.004678467	0.194504933	0.189826467	0.000117575
3h	ACA1_218470/clean/scf7180000084758:664226-664343:-	ACA1_218470	Rasrelated protein Rab-21, putative [Source:UniProtKB/TrEMBL;Acc:L8GRC8]	0.000255853	0.189984733	0.189728881	4.75E-07
3h	ACA1_349660/clean/scf7180000084690:30548-30650:+	ACA1_349660	BAT1 protein [Source:UniProtKB/TrEMBL;Acc:L8GJC9]	0.00316353	0.192778867	0.189615337	3.61E-09
3h	ACA1_358650/clean/scf7180000084742:20199-20259:+	ACA1_358650	Ubiquitin carboxyl-terminal hydrolase [Source:UniProtKB/TrEMBL;Acc:L8GKM0]	0.002663513	0.192240633	0.18957712	2.69E-06
3h	ACA1_108270/clean/scf7180000084661:26977-27056:+	ACA1_108270	Transferrin receptor family dimerization domain containing protein [Source:UniProtKB/TrEMBL;Acc:L8GC39]	0.033407733	0.222955767	0.189548033	0.00044678
3h	ACA1_279580/clean/scf7180000084776:419727-419814:+	ACA1_279580	Interferonrelated developmental regulator 1, putative [Source:UniProtKB/TrEMBL;Acc:L8H954]	0.031570333	0.2209786	0.189408267	0.038117966
3h	ACA1_106370/clean/scf7180000084660:94672-94792:+	ACA1_106370	Glyoxylate reductase [Source:UniProtKB/TrEMBL;Acc:L8GQ89]	0.029026937	0.218392167	0.18936523	0.003493107
3h	ACA1_289330/clean/scf7180000084777:416565-416644:+	ACA1_289330	U-box domain-containing protein [Source:UniProtKB/TrEMBL;Acc:L8HJ41]	0.000832658	0.1899663	0.189133642	2.42E-05
3h	ACA1_255230/clean/scf7180000084768:470724-470921:-	ACA1_255230	U6 snRNA-associated Sm-like protein LSm3 [Source:UniProtKB/TrEMBL;Acc:L8HA54]	0.00628841	0.1952464	0.18895799	3.90E-07
3h	ACA1_116200/clean/scf7180000084669:724244-724319:+	ACA1_116200	WASH_WAHD domain-containing protein [Source:UniProtKB/TrEMBL;Acc:L8H3U1]	0.04338	0.232321967	0.188941967	0.014075871
3h	ACA1_352400/clean/scf7180000084704:8804-8923:-	ACA1_352400	Fn3_like domain-containing protein [Source:UniProtKB/TrEMBL;Acc:L8GTX5]	0.010117357	0.199030667	0.18891331	1.58E-06
3h	ACA1_321740/clean/scf7180000084455:10132-10198:-	ACA1_321740	Peptidase S53 domain-containing protein [Source:UniProtKB/TrEMBL;Acc:L8GSL3]	0.005496313	0.194355833	0.18885952	2.76E-07
3h	ACA1_117320/clean/scf7180000084669:975172-975259:-	ACA1_117320	FAD binding domain containing protein [Source:UniProtKB/TrEMBL;Acc:L8H415]	0.004535133	0.193379767	0.188844633	0.000315515
3h	ACA1_309500/clean/scf7180000083762:11093-11172:+	ACA1_309500	Pyridine nucleotidedisulfide oxidoreductase domain containing protein [Source:UniProtKB/TrEMBL;Acc:L8GI23]	0.0253671	0.214208	0.1888409	3.66E-05
3h	ACA1_062260/clean/scf7180000084599:1153589-1153657:+	ACA1_062260	C2 domain containing protein [Source:UniProtKB/TrEMBL;Acc:L8GW88]	0.008838147	0.197647733	0.188809587	1.80E-05
3h	ACA1_174780/clean/scf7180000084741:795423-795515:-	ACA1_174780	VanY domain-containing protein [Source:UniProtKB/TrEMBL;Acc:L8HK80]	0.0402911	0.229071233	0.188780133	0.001040921
3h	ACA1_189360/clean/scf7180000084748:17577-17659:-	ACA1_189360	Proline dehydrogenase [Source:UniProtKB/TrEMBL;Acc:L8GGF7]	0	0.188656667	0.188656667	0.013502856
3h	ACA1_313730/clean/scf7180000083824:2560-2646:-	ACA1_313730	Glyco_trans_2-like domain-containing protein [Source:UniProtKB/TrEMBL;Acc:L8GN52]	0.005031543	0.193465267	0.188433723	2.89E-09
3h	ACA1_073440/clean/scf7180000084603:1419037-1419128:+	ACA1_073440	MFS domain-containing protein [Source:UniProtKB/TrEMBL;Acc:L8HEW4]	0	0.188345267	0.188345267	0.000349621
3h	ACA1_199710/clean/scf7180000084753:305487-305713:+	ACA1_199710	Phosphate transporter [Source:UniProtKB/TrEMBL;Acc:L8H377]	0.0473913	0.2356438	0.1882525	0.020284816
3h	ACA1_323150/clean/scf7180000084467:19869-19969:-	ACA1_323150	Cytochrome b5 heme-binding domain-containing protein [Source:UniProtKB/TrEMBL;Acc:L8GFA2]	0.000320974	0.1885298	0.188208826	2.90E-14
3h	ACA1_066150/clean/scf7180000084599:2013827-2013927:+	ACA1_066150	Myosin head (Motor domain) domain containing protein [Source:UniProtKB/TrEMBL;Acc:L8GX00]	0.013759433	0.201947267	0.188187833	0.010329793
3h	ACA1_209860/clean/scf7180000084757:341645-341771:-	ACA1_209860	Protein kinase domain-containing protein [Source:UniProtKB/TrEMBL;Acc:L8GYD8]	0.000328709	0.188505667	0.188176957	5.33E-07
3h	ACA1_068700/clean/scf7180000084603:258915-258992:+	ACA1_068700	Purple acid phosphatase [Source:UniProtKB/TrEMBL;Acc:L8HFR9]	0.005654853	0.193659	0.188004147	3.68E-07
3h	ACA1_367360/clean/scf7180000084641:613134-613234:-	ACA1_367360	TPR_REGION domain-containing protein [Source:UniProtKB/TrEMBL;Acc:L8GMB3]	1.00E-05	0.187961367	0.18795133	1.19E-07
3h	ACA1_014870/clean/scf7180000083772:14082-14154:+	ACA1_014870	tRNA phosphotransferase 1 isoform 1, putative [Source:UniProtKB/TrEMBL;Acc:L8HI60]	0.00494856	0.192861267	0.187912707	0.000618697
3h	ACA1_279500/clean/scf7180000084776:403195-403294:-	ACA1_279500	hypothetical protein	0.000725937	0.188633967	0.18790803	1.28E-06
3h	ACA1_189820/clean/scf7180000084748:95580-95687:-	ACA1_189820	Protein bcp1, putative [Source:UniProtKB/TrEMBL;Acc:L8GGI0]	0.007182853	0.1949679	0.187785047	0.001766504
3h	ACA1_066150/clean/scf7180000084599:2014911-2014982:+	ACA1_066150	Myosin head (Motor domain) domain containing protein [Source:UniProtKB/TrEMBL;Acc:L8GX00]	0.012893967	0.2006678	0.187773833	0.007156281
3h	ACA1_170100/clean/scf7180000084739:72123-72187:+	ACA1_170100	DEAD (AspGlu-Ala-Asp) box polypeptide 47 isoform 3, putative [Source:UniProtKB/TrEMBL;Acc:L8HGA0]	0.005372713	0.193112033	0.18773932	7.34E-05
3h	ACA1_282720/clean/scf7180000084776:725452-725540:-	ACA1_282720	Leucine rich repeat domain containing protein [Source:UniProtKB/TrEMBL;Acc:L8H9L8]	0.005640427	0.193304767	0.18766434	0.000485614
3h	ACA1_232260/clean/scf7180000084762:10468-10544:+	ACA1_232260	hypothetical protein	0.0199908	0.207645333	0.187654533	0.001519596
3h	ACA1_279560/clean/scf7180000084776:413936-414003:-	ACA1_279560	Iron hydrogenase assembly protein [Source:UniProtKB/TrEMBL;Acc:L8H633]	0.0305158	0.218038433	0.187522633	0.010767803
3h	ACA1_011270/clean/scf7180000083623:6469-6571:-	ACA1_011270	FYVE-type domain-containing protein [Source:UniProtKB/TrEMBL;Acc:L8GQN9]	0	0.1874324	0.1874324	0.048265125

3h	ACA1_198450/clean/scf7180000084753:115393-115503:-	ACA1_198450	OTU domain-containing protein [Source:UniProtKB/TrEMBL;Acc:L8H2C9]	0	0.187305167	0.187305167	1.32E-07
3h	ACA1_288340/clean/scf7180000084777:172025-172111:+	ACA1_288340	Conditioned medium factor, putative [Source:UniProtKB/TrEMBL;Acc:L8HKD6]	0	0.187241067	0.187241067	9.62E-09
3h	ACA1_359270/clean/scf7180000084742:70286-70438:-	ACA1_359270	RAB family member (Rab7), putative [Source:UniProtKB/TrEMBL;Acc:L8GLL4]	0.00857366	0.195793933	0.187220273	1.92E-05
3h	ACA1_063890/clean/scf7180000084599:1527483-1527538:+	ACA1_063890	DAO domain-containing protein [Source:UniProtKB/TrEMBL;Acc:L8GXC2]	0.006958657	0.194176	0.187217343	5.65E-05
3h	ACA1_211500/clean/scf7180000084758:88977-89036:+	ACA1_211500	RAB5B protein, putative [Source:UniProtKB/TrEMBL;Acc:L8GS95]	0	0.1871207	0.1871207	0.000552541
3h	ACA1_377530/clean/scf7180000084711:83819-83887:-	ACA1_377530	Protein kinase domain-containing protein [Source:UniProtKB/TrEMBL;Acc:L8GR81]	0.013757933	0.200865633	0.1871077	0.000536254
3h	ACA1_254430/clean/scf7180000084768:391138-391227:+	ACA1_254430	Macro domain-containing protein [Source:UniProtKB/TrEMBL;Acc:L8HDC4]	0.011780767	0.198860233	0.187079467	7.10E-05
3h	ACA1_183510/clean/scf7180000084745:435741-435866:+	ACA1_183510	Cyclic nucleotide-binding domain-containing protein [Source:UniProtKB/TrEMBL;Acc:L8HAF9]	0.01294282	0.1999998	0.18705698	0.000303194
3h	ACA1_012970/clean/scf7180000083742:14123-14193:+	ACA1_012970	CBS domain containing protein [Source:UniProtKB/TrEMBL;Acc:L8H720]	0.015238267	0.202051333	0.186813067	0.001963003
3h	ACA1_263870/clean/scf7180000084770:123582-123680:-	ACA1_263870	MAGE domain-containing protein [Source:UniProtKB/TrEMBL;Acc:L8H1Y1]	0.092415933	0.279025333	0.1866094	0.016099553
3h	ACA1_385120/clean/scf7180000084730:137611-137715:+	ACA1_385120	Non-specific serine/threonine protein kinase [Source:UniProtKB/TrEMBL;Acc:L8HBH8]	0.001398633	0.188002633	0.186604	1.50E-07
3h	ACA1_182950/clean/scf7180000084745:297158-297250:+	ACA1_182950	Farnesyl pyrophosphate synthetase [Source:UniProtKB/TrEMBL;Acc:L8HA89]	0.002321933	0.188656033	0.1863341	8.82E-10
3h	ACA1_114500/clean/scf7180000084669:487925-487991:-	ACA1_114500	Macro domain-containing protein [Source:UniProtKB/TrEMBL;Acc:L8H6J5]	0.001711763	0.187986167	0.186274403	3.52E-06
3h	ACA1_290650/clean/scf7180000084777:650108-650226:+	ACA1_290650	DENN domain-containing protein [Source:UniProtKB/TrEMBL;Acc:L8HI74]	0.03718911	0.223406	0.18621689	0.009445906
3h	ACA1_243850/clean/scf7180000084763:361061-361128:+	ACA1_243850	Ras-GAP domain-containing protein [Source:UniProtKB/TrEMBL;Acc:L8GJZ5]	0.00263713	0.1886955	0.18605837	0.005185932
3h	ACA1_012980/clean/scf7180000083742:15384-15475:-	ACA1_012980	Endo/exonuclease/phosphatase domain-containing protein [Source:UniProtKB/TrEMBL;Acc:L8H4W5]	0.023365633	0.209343867	0.185978233	0.002030111
3h	ACA1_138800/clean/scf7180000084705:13540-13730:+	ACA1_138800	hypothetical protein	0.018422167	0.2043663	0.185944133	4.05E-05
3h	ACA1_378170/clean/scf7180000084711:200681-200766:-	ACA1_378170	G_PROTEIN_RECEP_F1_2 domain-containing protein [Source:UniProtKB/TrEMBL;Acc:L8GRC2]	0.00071675	0.1864381	0.18572135	9.21E-06
3h	ACA1_199970/clean/scf7180000084753:362444-362591:-	ACA1_199970	Glycoside hydrolase family protein [Source:UniProtKB/TrEMBL;Acc:L8H5H5]	0.000241393	0.185933567	0.185692174	5.44E-10
3h	ACA1_182120/clean/scf7180000084745:133133-133197:+	ACA1_182120	WD repeat domain 5B, putative [Source:UniProtKB/TrEMBL;Acc:L8HA46]	0.004866166	0.190534667	0.185668501	1.13E-05
3h	ACA1_061930/clean/scf7180000084599:1081298-1081365:-	ACA1_061930	Transporter, major intrinsic protein (MIP) superfamily protein [Source:UniProtKB/TrEMBL;Acc:L8GZB9]	0.004030568	0.189660767	0.185630199	1.31E-05
3h	ACA1_201390/clean/scf7180000084753:541291-541368:-	ACA1_201390	Transmembrane 9 superfamily member [Source:UniProtKB/TrEMBL;Acc:L8H5N1]	0.002022642	0.187609633	0.185586991	6.59E-08
3h	ACA1_135110/clean/scf7180000084702:65426-65562:-	ACA1_135110	Hypothetical protein	0.00182065	0.187402667	0.185582017	1.71E-08
3h	ACA1_027490/clean/scf7180000084469:1774-1866:-	ACA1_027490	Calcium-transporting ATPase [Source:UniProtKB/TrEMBL;Acc:L8H4Z2]	0.001541168	0.187076033	0.185534865	3.15E-06
3h	ACA1_063800/clean/scf7180000084599:1505946-1506036:-	ACA1_063800	B30.2/SPRY domain-containing protein [Source:UniProtKB/TrEMBL;Acc:L8GZV1]	0.032178067	0.217664367	0.1854863	0.001540687
3h	ACA1_270200/clean/scf7180000084770:1076475-1076593:-	ACA1_270200	FYVE-type domain-containing protein [Source:UniProtKB/TrEMBL;Acc:L8H522]	0	0.1852675	0.1852675	0.017762023
3h	ACA1_373600/clean/scf7180000084679:111690-111784:-	ACA1_373600	G domain-containing protein [Source:UniProtKB/TrEMBL;Acc:L8GJC7]	0.025244907	0.210431133	0.185186227	0.009262232
3h	ACA1_095580/clean/scf7180000084645:851667-851859:+	ACA1_095580	Serine/threonine kinase [Source:UniProtKB/TrEMBL;Acc:L8GJI9]	0.031443233	0.216504867	0.185061633	0.002078929
3h	ACA1_252200/clean/scf7180000084768:142883-142954:+	ACA1_252200	Saposin, putative [Source:UniProtKB/TrEMBL;Acc:L8HD22]	0.002421629	0.1874533	0.185031671	1.38E-05
3h	ACA1_340680/clean/scf7180000084620:14786-14857:-	ACA1_340680	hypothetical protein	0.00995414	0.194961167	0.185007027	2.92E-06
3h	ACA1_279560/clean/scf7180000084776:414376-414438:-	ACA1_279560	Iron hydrogenase assembly protein [Source:UniProtKB/TrEMBL;Acc:L8H633]	0.007320193	0.192268933	0.18494874	0.002901393
3h	ACA1_175060/clean/scf7180000084741:840464-840734:+	ACA1_175060	V-type proton ATPase subunit a [Source:UniProtKB/TrEMBL;Acc:L8HHZ1]	0.00316168	0.1880433	0.18488162	3.57E-09
3h	ACA1_198700/clean/scf7180000084753:155594-155713:-	ACA1_198700	ABC transporter domain-containing protein [Source:UniProtKB/TrEMBL;Acc:L8H2D9]	0.000320862	0.185041367	0.184720505	4.24E-08
3h	ACA1_057100/clean/scf7180000084599:43523-43604:-	ACA1_057100	unspecified product	0.009914227	0.1946292	0.184714973	0.003572094
3h	ACA1_261410/clean/scf7180000084769:667988-668075:+	ACA1_261410	UBA_e1_C domain-containing protein [Source:UniProtKB/TrEMBL;Acc:L8GHQ6]	0.004067491	0.1887273	0.184659809	2.36E-07
3h	ACA1_390800/clean/scf7180000084740:97209-97290:+	ACA1_390800	Glycosyltransferase family 8 protein [Source:UniProtKB/TrEMBL;Acc:L8GQ18]	0.011398517	0.1960135	0.184614983	0.000131123
3h	ACA1_227080/clean/scf7180000084761:95156-95255:+	ACA1_227080	Uracil-DNA glycosylase [Source:UniProtKB/TrEMBL;Acc:L8HA23]	0.00330033	0.1878931	0.18459277	0.000363689
3h	ACA1_149410/clean/scf7180000084715:144654-144756:+	ACA1_149410	Lectin_legB domain-containing protein [Source:UniProtKB/TrEMBL;Acc:L8HDD6]	0.002253767	0.1867531	0.184499333	5.64E-05
3h	ACA1_307520/clean/scf7180000083680:24434-24514:-	ACA1_307520	B5 domain-containing protein [Source:UniProtKB/TrEMBL;Acc:L8H4Y1]	0.001486748	0.185985467	0.184498718	8.31E-08
3h	ACA1_277780/clean/scf7180000084776:99148-99256:+	ACA1_277780	Ca2+dependent endoplasmic reticulum nucleoside diphosphatase isoform 3, putative [Source:UniProtKB/TrEMBL;Acc:L8H7Y3]	0.011439527	0.1958649	0.184425373	0.000200773
3h	ACA1_002490/clean/scf7180000082931:7378-7451:-	ACA1_002490	AA_TRNA_LIGASE_II_ALA domain-containing protein [Source:UniProtKB/TrEMBL;Acc:L8GVN5]	0.0068696	0.191292367	0.184422767	0.000259015
3h	ACA1_355360/clean/scf7180000084728:7141-7268:-	ACA1_355360	MFS domain-containing protein [Source:UniProtKB/TrEMBL;Acc:L8H7K9]	0.00702837	0.191400667	0.184372297	0.000896325
3h	ACA1_316750/clean/scf7180000083984:1384-1464:+	ACA1_316750	Peptidase, S8/S53 subfamily protein [Source:UniProtKB/TrEMBL;Acc:L8GLP0]	0.0154003	0.199741333	0.184341033	2.27E-05
3h	ACA1_187780/clean/scf7180000084747:94058-94122:-	ACA1_187780	Corrinoid adenosyltransferase [Source:UniProtKB/TrEMBL;Acc:L8GUU3]	0	0.184334633	0.184334633	4.68E-06
3h	ACA1_264280/clean/scf7180000084770:228394-228480:-	ACA1_264280	hypothetical protein	0.00551107	0.1898223	0.18431123	5.86E-07
3h	ACA1_031960/clean/scf7180000084523:42352-42428:+	ACA1_031960	Inositol oxygenase [Source:UniProtKB/TrEMBL;Acc:L8H1U0]	0.0590419	0.243352667	0.184310767	0.010995707
3h	ACA1_091880/clean/scf7180000084645:220522-220628:+	ACA1_091880	Ubiquitin-like domain-containing protein [Source:UniProtKB/TrEMBL;Acc:L8GIV5]	0.003439127	0.1875921	0.184152973	1.50E-05
3h	ACA1_140190/clean/scf7180000084707:39646-39720:+	ACA1_140190	Cysteine protease [Source:UniProtKB/TrEMBL;Acc:L8GGF1]	0.009472467	0.193506467	0.184034	0.000134265
3h	ACA1_014360/clean/scf7180000083761:4694-4774:+	ACA1_014360	Protein kinase domain-containing protein [Source:UniProtKB/TrEMBL;Acc:L8GL10]	0.000773397	0.184607667	0.18383427	1.20E-06
3h	ACA1_113880/clean/scf7180000084669:397824-397930:-	ACA1_113880	hypothetical protein	0.017528247	0.201346	0.183817753	0.001392423
3h	ACA1_109750/clean/scf7180000084663:17929-18027:-	ACA1_109750	Lightoid family protein [Source:UniProtKB/TrEMBL;Acc:L8HHC7]	0.000886523	0.184555467	0.183668943	1.98E-06
3h	ACA1_399880/clean/scf7180000084775:57894-57992:+	ACA1_399880	Rab-GAP TBC domain-containing protein [Source:UniProtKB/TrEMBL;Acc:L8GGF5]	0	0.1835731	0.1835731	3.56E-05
3h	ACA1_117130/clean/scf7180000084669:922265-922354:+	ACA1_117130	Eukaryotic initiation factor 4e, putative [Source:UniProtKB/TrEMBL;Acc:L8H4Z1]	0.008796606	0.192351067	0.183554461	4.12E-06
3h	ACA1_100080/clean/scf7180000084648:31531-31607:-	ACA1_100080	Epimerase domain-containing protein [Source:UniProtKB/TrEMBL;Acc:L8GKE8]	0.005034157	0.188480733	0.183446577	1.89E-07
3h	ACA1_221330/clean/scf7180000084758:1016699-1016881:-	ACA1_221330	hypothetical protein	0.00456433	0.187919367	0.183355037	0.000397557
3h	ACA1_065030/clean/scf7180000084599:1756782-1756858:+	ACA1_065030	eIF2B_5 domain-containing protein [Source:UniProtKB/TrEMBL;Acc:L8H018]	0.01962667	0.202946333	0.183319663	0.003182801
3h	ACA1_237870/clean/scf7180000084763:36305-36453:+	ACA1_237870	Methyltransferase [Source:UniProtKB/TrEMBL;Acc:L8GKC5]	2.42E-05	0.183199633	0.183175466	5.58E-11
3h	ACA1_235050/clean/scf7180000084762:328522-328620:+	ACA1_235050	RNA ligase, putative [Source:UniProtKB/TrEMBL;Acc:L8H1C3]	0.005579211	0.188682967	0.183103755	0.001417403
3h	ACA1_383470/clean/scf7180000084721:175421-175494:+	ACA1_383470	ANK_REP_REGION domain-containing protein [Source:UniProtKB/TrEMBL;Acc:L8GXK5]	0.000411243	0.1834287	0.183017457	3.87E-06
3h	ACA1_109750/clean/scf7180000084663:18483-18554:-	ACA1_109750	Lightoid family protein [Source:UniProtKB/TrEMBL;Acc:L8HHC7]	0.003741503	0.186736467	0.182994963	1.25E-05
3h	ACA1_229170/clean/scf7180000084761:237562-237654:+	ACA1_229170	hypothetical protein	0.023119053	0.206075333	0.18295628	0.000280875
3h	ACA1_295880/clean/scf7180000084777:1251170-1251265:+	ACA1_295880	Malic enzyme [Source:UniProtKB/TrEMBL;Acc:L8HM80]	0.000171773	0.183121667	0.182949894	5.64E-07
3h	ACA1_181630/clean/scf7180000084745:31077-31156:+	ACA1_181630	hypothetical protein	0.0722367	0.254939667	0.182702967	0.022645946
3h	ACA1_164100/clean/scf7180000084729:161890-161962:-	ACA1_164100	Succinyl-CoA:3-ketoacid-coenzyme A transferase [Source:UniProtKB/TrEMBL;Acc:L8GTX7]	0.002464163	0.185056433	0.18259227	2.96E-10
3h	ACA1_307530/clean/scf7180000083680:27713-27801:+	ACA1_307530	Acyltransferase [Source:UniProtKB/TrEMBL;Acc:L8H6B3]	0.003913817	0.186442533	0.182528717	1.54E-05
3h	ACA1_063500/clean/scf7180000084599:1434109-1434178:+	ACA1_063500	U-box domain-containing protein [Source:UniProtKB/TrEMBL;Acc:L8GZS8]	0.001455603	0.183849533	0.18239393	8.75E-06
3h	ACA1_382630/clean/scf7180000084721:88603-88697:-	ACA1_382630	LBP / BPI / CETP family, Cterminal domain containing protein [Source:UniProtKB/TrEMBL;Acc:L8GVZ7]	0.001238673	0.1833131	0.182074427	3.62E-07
3h	ACA1_237770/clean/scf7180000084763:12266-12387:+	ACA1_237770	PH domain-containing protein [Source:UniProtKB/TrEMBL;Acc:L8GKB5]	0.015801737	0.197864733	0.182062997	0.004950453
3h	ACA1_033720/clean/scf7180000084559:114752-114855:+	ACA1_033720	hypothetical protein	0.014282333	0.196330333	0.182048	5.62E-06
3h	ACA1_325260/clean/scf7180000084475:43058-43158:+	ACA1_325260	Peptidylprolyl isomerase [Source:UniProtKB/TrEMBL;Acc:L8GPH9]	0.010411863	0.192407233	0.18199537	2.49E-06
3h	ACA1_144210/clean/scf7180000084709:172679-172775:-	ACA1_144210	hypothetical protein	0.000571654	0.1825334	0.181961746	8.68E-12
3h	ACA1_017740/clean/scf7180000083876:3086-3196:+	ACA1_017740	5'-3' exoribonuclease 1 [Source:UniProtKB/TrEMBL;Acc:L8GMR4]	0.002083333	0.184003833	0.1819205	3.12E-06
3h	ACA1_091850/clean/scf7180000084645:215660-215740:-	ACA1_091850	hypothetical protein	0.047021433	0.228891667	0.181870233	0.010770517
3h	ACA1_165210/clean/scf7180000084732:8287-8432:-	ACA1_165210	Ras subfamily protein [Source:UniProtKB/TrEMBL;Acc:L8HGF1]	0.004226053	0.186086067	0.181860013	6.30E-07
3h	ACA1_253180/clean/scf7180000084768:266947-267032:-	ACA1_253180	Fes1 domain-containing protein [Source:UniProtKB/TrEMBL;Acc:L8HAR0]	0.016840137	0.1986785	0.181838363	0.00078963
3h	ACA1_359710/clean/scf7180000084622:8336-8407:-	ACA1_359710	WD_REPEATS_REGION domain-containing protein [Source:UniProtKB/TrEMBL;Acc:L8HEZ3]	0.003111193	0.184937733	0.18182654	3.68E-08
3h	ACA1_376270/clean/scf7180000084685:68625-68743:+	ACA1_376270	Phospho-2-dehydro-3-deoxyheptonate aldolase [Source:UniProtKB/TrEMBL;Acc:L8HFZ7]	0.018159763	0.199956667	0.181796903	0.000650335
3h	ACA1_362250/clean/scf7180000084624:39004-39077:+	ACA1_362250	hypothetical protein	0.0042657	0.1860182	0.1817525	0.000189027
3h	ACA1_313610/clean/scf7180000083822:11595-11687:+	ACA1_313610	Calcium-transporting ATPase [Source:UniProtKB/TrEMBL;Acc:L8GL52]	0.004025143	0.185557733	0.18153259	1.06E-06
3h	ACA1_330340/clean/scf7180000084566:42711-42803:-	ACA1_330340	PAPA-1 domain-containing protein [Source:UniProtKB/TrEMBL;Acc:L8GHA1]	0.011322287	0.192776	0.181453713	0.001289762
3h	ACA1_394600/clean/scf7180000084756:116809-116891:-	ACA1_394600	GRIP domain-containing protein [Source:UniProtKB/TrEMBL;Acc:L8H1Q7]	0	0.181439333	0.181439333	4.26E-05
3h	ACA1_061570/clean/scf7180000084599:964176-964243:-	ACA1_061570	hypothetical protein	0.007381967	0.188699033	0.181317067	0.000112009
3h	ACA1_034300/clean/scf7180000084563:5542-5603:+	ACA1_034300	DUF1084 domain-containing protein [Source:UniProtKB/TrEMBL;Acc:L8HCY8]	0.000602227	0.1819172	0.181314973	4.68E-05
3h	ACA1_215600/clean/scf7180000084758:346329-346455:-	ACA1_215600	hypothetical protein	0.008242667	0.189553533	0.181310867	0.025521074
3h	ACA1_287660/clean/scf7180000084777:57518-57720:+	ACA1_287660	Pentapeptide repeatcontaining protein [Source:UniProtKB/TrEMBL;Acc:L8HIP0]	0.044910733	0.2261546	0.181243867	0.005973401
3h	ACA1_111900/clean/scf7180000084669:64489-64599:+	ACA1_111900	Global transcription factor, putative [Source:UniProtKB/TrEMBL;Acc:L8H3V5]	0.0125028	0.193725467	0.181222667	1.18E-05
3h	ACA1_255510/clean/scf7180000084768:518187-518280:-	ACA1_255510	Ephrin_rec_like domain-containing protein [Source:UniProtKB/TrEMBL;Acc:L8HDG9]	0.010886397	0.192019933	0.181133537	1.40E-06
3h	ACA1_113680/clean/scf7180000084669:349628-349794:-	ACA1_113680	M20_dimer domain-containing protein [Source:UniProtKB/TrEMBL;Acc:L8H5M1]	0.011006713	0.192078	0.181071287	6.51E-07
3h	ACA1_200900/clean/scf7180000084753:460470-460558:+	ACA1_200900	Abhydrolase domain containing protein 3, putative [Source:UniProtKB/TrEMBL;Acc:L8H4T5]	0.0797031	0.260620967	0.180917867	0.029076457
3h	ACA1_276370/clean/scf7180000084774:158338-158518:-	ACA1_276370	unspecified product	0.000527041	0.181409133	0.180882092	2.50E-06
3h	ACA1_214850/clean/scf7180000084758:245487-245560:+	ACA1_214850	LIM zinc-binding domain-containing protein [Source:UniProtKB/TrEMBL;Acc:L8GSG1]	2.89E-05	0.180821233	0.180792323	1.15E-06
3h	ACA1_033610/clean/scf7180000084559:59644-59713:+	ACA1_033610	Sortilin [Source:UniProtKB/TrEMBL;Acc:L8GHJ0]	0	0.180729633	0.180729633	1.99E-07
3h	ACA1_117320/clean/scf7180000084669:975026-975094:-	ACA1_117320	FAD binding domain containing protein [Source:UniProtKB/TrEMBL;Acc:L8H415]	0.00243309	0.1830984	0.18066531	0.000223481
3h	ACA1_321020/clean/scf7180000084435:18042-18112:+	ACA1_321020	hypothetical protein	0.00962867	0.190241267	0.180612597	4.44E-06
3h	ACA1_171880/clean/scf7180000084741:184242-184381:-	ACA1_171880	F-box domain-containing protein [Source:UniProtKB/TrEMBL;Acc:L8HGG2]	0.0043808	0.184972833	0.180592033	0.004802879
3h	ACA1_025410/clean/scf7180000084446:14072-14140:+	ACA1_025410	Serine/threonine protein kinase [Source:UniProtKB/TrEMBL;Acc:L8HGY2]	0.0113926	0.191869967	0.180477367	0.00043037
3h	ACA1_051450/clean/scf7180000084592:16584-16651:-	ACA1_051450	TMEM135_C_rich domain-containing protein [Source:UniProtKB/TrEMBL;Acc:L8GQJ0]	0.01717149	0.197484533	0.180313043	0.012434444
3h	ACA1_101560/clean/scf7180000084649:95055-95160:+	ACA1_101560	WD_REPEATS_REGION domain-containing protein [Source:UniProtKB/TrEMBL;Acc:L8GGC0]	0.006315313	0.186513367	0.180198053	1.59E-06
3h	ACA1_106740/clean/scf7180000084660:113292-113428:+	ACA1_106740	Nas2_N domain-containing protein [Source:UniProtKB/TrEMBL;Acc:L8GNH7]	0.00997209	0.1901466	0.18017451	0.000130868
3h	ACA1_002770/clean/scf7180000082937:7296-7425:+	ACA1_002770	Fbox domain containing protein [Source:UniProtKB/TrEMBL;Acc:L8GJ90]	0.00451732	0.184600733	0.180083413	1.56E-05
3h	ACA1_074130/clean/scf7180000084605:33120-33184:+	ACA1_074130	PlsC domain-containing protein [Source:UniProtKB/TrEMBL;Acc:L8HMM5]	0.023247819	0.203229833	0.179982014	0.003701946
3h	ACA1_283300/clean/scf7180000084776:812966-813214:-	ACA1_283300	DEP domain-containing protein [Source:UniProtKB/TrEMBL;Acc:L8H8T3]	0.018594733	0.198445667	0.179850933	2.56E-05
3h	ACA1_040520/clean/scf7180000084572:26898-26980:+	ACA1_040520	Golgi family protein, putative [Source:UniProtKB/TrEMBL;Acc:L8H2W6]	0.00450293	0.184149033	0.179646103	1.29E-08
3h	ACA1_377980/clean/scf7180000084711:156503-156593:+	ACA1_377980	Ubiquitin-like domain-containing protein [Source:UniProtKB/TrEMBL;Acc:L8GRZ4]	2.54E-05	0.1796141	0.179588695	3.66E-07
3h	ACA1_384710/clean/scf7180000084730:91416-91489:+	ACA1_384710	Transmembrane receptor family protein [Source:UniProtKB/TrEMBL;Acc:L8HBG4]	0.000302734	0.179765433	0.179462699	4.43E-08
3h	ACA1_395220/clean/scf7180000084756:157296-157400:+	ACA1_395220	Eukaryotic initiation factor 4E [Source:UniProtKB/TrEMBL;Acc:L8H0E9]	0.027000933	0.2063208	0.179319867	0.000493479
3h	ACA1_267340/clean/scf7180000084770:859511-859580:-	ACA1_267340	CBS domain containing protein [Source:UniProtKB/TrEMBL;Acc:L8H2L9]	0.015856967	0.195123167	0.1792662	0.008508319
3h	ACA1_208580/clean/scf7180000084757:227240-227330:-	ACA1_208580	GPI anchored protein [Source:UniProtKB/TrEMBL;Acc:L8GZM6]	0.00287317	0.181910933	0.179037763	5.20E-07
3h	ACA1_113860/clean/scf7180000084669:389376-389469:+	ACA1_113860	Saposin B-type domain-containing protein [Source:UniProtKB/TrEMBL;Acc:L8H4D7]	0.002531351	0.181503167	0.178971816	8.98E-07
3h	ACA1_195050/clean/scf7180000084751:61450-61529:+	ACA1_195050	Cyclin N-terminal domain-containing protein [Source:UniProtKB/TrEMBL;Acc:L8H6R3]	0.023456867	0.202428267	0.1789714	0.002342979
3h	ACA1_164910/clean/scf7180000084729:310487-310555:-	ACA1_164910	hypothetical protein	0.002469683	0.181292567	0.178822883	4.58E-07
3h	ACA1_208580/clean/scf7180000084757:227435-227526:-	ACA1_208580	GPI anchored protein [Source:UniProtKB/TrEMBL;Acc:L8GZM6]	0.020083467	0.1988364	0.178752933	9.21E-05
3h	ACA1_040830/clean/scf7180000084572:89718-89789:-	ACA1_040830	UBIQUITIN_CONJUGAT_2 domain-containing protein [Source:UniProtKB/TrEMBL;Acc:L8H0M3]	0.0050243	0.1835739	0.1785496	4.72E-07
3h	ACA1_073350/clean/scf7180000084603:1398691-1398782:+	ACA1_073350	AAR2 protein [Source:UniProtKB/TrEMBL;Acc:L8HGY7]	0.013198737	0.191614467	0.17841573	0.000185156
3h	ACA1_360430/clean/scf7180000084622:128751-128810:+	ACA1_360430	hypothetical protein	0	0.178218667	0.178218667	0.001089982
3h	ACA1_275720/clean/scf7180000084774:93175-93261:+	ACA1_275720	Enolase, Cterminal TIM barrel domain containing protein [Source:UniProtKB/TrEMBL;Acc:L8GRZ9]	0.002216333	0.180432667	0.178216333	5.04E-09
3h	ACA1_143070/clean/scf7180000084709:17753-17811:+	ACA1_143070	ATPase, AAA domain containing protein [Source:UniProtKB/TrEMBL;Acc:L8HFD9]	0.003651533	0.181564667	0.177913133	9.99E-06
3h	ACA1_335940/clean/scf7180000084593:9509-9645:-	ACA1_335940	RNA recognition motif domain containing protein [Source:UniProtKB/TrEMBL;Acc:L8H3Y6]	0.006985333	0.184870467	0.177885133	0.000189027
3h	ACA1_382580/clean/scf7180000084721:76219-76425:+	ACA1_382580	LBP / BPI / CETP family, Cterminal domain containing protein [Source:UniProtKB/TrEMBL;Acc:L8GVZ1]	0.005286563	0.183145	0.177858437	8.42E-08
3h	ACA1_062630/clean/scf7180000084599:1258021-1258094:-	ACA1_062630	PX domain-containing protein [Source:UniProtKB/TrEMBL;Acc:L8GWC4]	0.001303993	0.179153033	0.17784904	7.09E-06
3h	ACA1_400360/clean/scf7180000084775:171362-171479:-	ACA1_400360	ATP/GTP nucleotidebinding protein [Source:UniProtKB/TrEMBL;Acc:L8GGI8]	0.009415813	0.1872508	0.177834987	1.23E-05
3h	ACA1_092600/clean/scf7180000084645:363642-363712:-	ACA1_092600	NLPC_P60 domain-containing protein [Source:UniProtKB/TrEMBL;Acc:L8GIC1]	0.0389823	0.216512167	0.177529867	0.00221747
3h	ACA1_189830/clean/scf7180000084748:98448-98512:+	ACA1_189830	Partner of sld five, psf2 protein [Source:UniProtKB/TrEMBL;Acc:L8GE47]	0.000560223	0.178082367	0.177522143	0.001244639
3h	ACA1_173160/clean/scf7180000084741:414663-414794:+	ACA1_173160	hypothetical protein	0.015537893	0.192758367	0.177220473	0.001088377
3h	ACA1_208110/clean/scf7180000084757:105734-105831:+	ACA1_208110	Phosphoinositide 3kinase family, accessory domain (Pik domain) domain containing protein [Source:UniProtKB/TrEMBL;Acc:L8GXD2]	0.002123143	0.179320333	0.17719719	0.000130409
3h	ACA1_310480/clean/scf7180000083773:6492-6580:-	ACA1_310480	hypothetical protein	0.0514763	0.2286097	0.1771334	0.012066135
3h	ACA1_380970/clean/scf7180000084712:132603-132688:+	ACA1_380970	ANK_REP_REGION domain-containing protein [Source:UniProtKB/TrEMBL;Acc:L8GMX1]	0.0161837	0.1932758	0.1770921	0.016804681
3h	ACA1_091850/clean/scf7180000084645:216199-216297:-	ACA1_091850	hypothetical protein	0.0074074	0.184401033	0.176993633	0.011971864
3h	ACA1_369260/clean/scf7180000084664:285218-285318:+	ACA1_369260	hypothetical protein	0.002244907	0.179126833	0.176881927	1.48E-07
3h	ACA1_369850/clean/scf7180000084664:394244-394337:-	ACA1_369850	ATPase with chaperone activity, putative [Source:UniProtKB/TrEMBL;Acc:L8GZ67]	0.002671287	0.179492467	0.176821179	7.28E-05
3h	ACA1_201480/clean/scf7180000084753:561034-561099:+	ACA1_201480	PKS_ER domain-containing protein [Source:UniProtKB/TrEMBL;Acc:L8H3J6]	0.012563667	0.189314567	0.1767509	0.000172262
3h	ACA1_229570/clean/scf7180000084761:280061-280129:-	ACA1_229570	Oxidoreductase, short chain dehydrogenase/reductase superfamily protein [Source:UniProtKB/TrEMBL;Acc:L8H814]	0.0733743	0.250067833	0.176693533	0.029693985
3h	ACA1_098160/clean/scf7180000084645:1410366-1410448:-	ACA1_098160	ABC transporter, ATPbinding domain containing protein [Source:UniProtKB/TrEMBL;Acc:L8GJY4]	0.012003787	0.188602267	0.17659848	8.05E-05
3h	ACA1_193110/clean/scf7180000084749:186202-186290:-	ACA1_193110	hypothetical protein	0.003126153	0.1796596	0.176533447	7.76E-07
3h	ACA1_000550/clean/scf7180000082784:4334-4456:+	ACA1_000550	Eukaryotic translation initiation factor 4E family [Source:UniProtKB/TrEMBL;Acc:L8GRC5]	0.011626163	0.188103733	0.17647757	0.000944482
3h	ACA1_358630/clean/scf7180000084742:16072-16141:-	ACA1_358630	hypothetical protein	0.007133067	0.183512467	0.1763794	0.002094601
3h	ACA1_279600/clean/scf7180000084776:423552-423655:-	ACA1_279600	hypothetical protein	0.00114682	0.177484667	0.176337847	1.82E-05
3h	ACA1_037120/clean/scf7180000084567:64431-64498:+	ACA1_037120	COMM domain-containing protein [Source:UniProtKB/TrEMBL;Acc:L8H290]	0.009995237	0.186264033	0.176268797	2.70E-05

3h	ACA1_201650/clean/scf7180000084753:609590-609683:-	ACA1_201650	DNA repair protein RAD51 homolog [Source:UniProtKB/TrEMBL;Acc:L8H3I3]	0.00213595	0.1783315	0.17619555	1.08E-06
3h	ACA1_017740/clean/scf7180000083876:6912-7000:+	ACA1_017740	5'-3' exoribonuclease 1 [Source:UniProtKB/TrEMBL;Acc:L8GMR4]	0.003282853	0.179454333	0.17617148	1.40E-08
3h	ACA1_220750/clean/scf7180000084758:961082-961228:-	ACA1_220750	Urease accessory protein UreF, putative [Source:UniProtKB/TrEMBL;Acc:L8GQ53]	0.012987399	0.189140567	0.176153167	0.002391687
3h	ACA1_275750/clean/scf7180000084774:99069-99161:-	ACA1_275750	unspecified product	0.001760143	0.177899867	0.176139723	0.000505025
3h	ACA1_157950/clean/scf7180000084724:158230-158353:+	ACA1_157950	hypothetical protein	0.014196833	0.190309133	0.1761123	7.58E-05
3h	ACA1_392990/clean/scf7180000084756:23113-23198:+	ACA1_392990	ANK_REP_REGION domain-containing protein [Source:UniProtKB/TrEMBL;Acc:L8H1M2]	0.100956633	0.277018333	0.1760617	0.032161154
3h	ACA1_116890/clean/scf7180000084669:867360-867444:-	ACA1_116890	RPOL9 domain-containing protein [Source:UniProtKB/TrEMBL;Acc:L8H6X7]	0.002032536	0.178007133	0.175974598	1.43E-06
3h	ACA1_070460/clean/scf7180000084603:687656-687716:-	ACA1_070460	J domain-containing protein [Source:UniProtKB/TrEMBL;Acc:L8HFA5]	0.013690553	0.189486333	0.17579578	0.00036579
3h	ACA1_236110/clean/scf7180000084762:406447-406542:-	ACA1_236110	PPM-type phosphatase domain-containing protein [Source:UniProtKB/TrEMBL;Acc:L8H2W4]	0.00350197	0.179257667	0.175755697	2.88E-05
3h	ACA1_181960/clean/scf7180000084745:91173-91259:+	ACA1_181960	WD_REPEATS_REGION domain-containing protein [Source:UniProtKB/TrEMBL;Acc:L8H808]	0.002136447	0.177880167	0.17574372	9.49E-06
3h	ACA1_057940/clean/scf7180000084599:216037-216103:+	ACA1_057940	Alpha-mannosidase [Source:UniProtKB/TrEMBL;Acc:L8GX61]	0.002282853	0.177779933	0.17549708	3.11E-06
3h	ACA1_378280/clean/scf7180000084711:228984-229049:+	ACA1_378280	hypothetical protein	0.014198583	0.189676933	0.17547835	0.000661478
3h	ACA1_369860/clean/scf7180000084664:397360-397435:+	ACA1_369860	GTPbinding protein SARA, putative [Source:UniProtKB/TrEMBL;Acc:L8H0F0]	0.003633947	0.179028133	0.175394187	5.02E-05
3h	ACA1_238390/clean/scf7180000084763:81863-81966:-	ACA1_238390	Coronin [Source:UniProtKB/TrEMBL;Acc:L8GJQ2]	0.004945246	0.180284733	0.175339488	1.15E-06
3h	ACA1_065910/clean/scf7180000084599:1945204-1945277:+	ACA1_065910	hypothetical protein	0.0038041	0.179113167	0.175309067	0.018462895
3h	ACA1_002750/clean/scf7180000082937:5164-5232:-	ACA1_002750	F-box domain-containing protein [Source:UniProtKB/TrEMBL;Acc:L8GHD2]	0.083086867	0.2581712	0.175084333	0.03599393
3h	ACA1_267240/clean/scf7180000084770:835859-836043:-	ACA1_267240	UBIQUITIN_CONJUGAT_2 domain-containing protein [Source:UniProtKB/TrEMBL;Acc:L8H2K7]	0.022621833	0.197691933	0.1750701	8.09E-05
3h	ACA1_220760/clean/scf7180000084758:961970-962049:-	ACA1_220760	Methyltransf_11 domain-containing protein [Source:UniProtKB/TrEMBL;Acc:L8GQP4]	0.001970055	0.176784833	0.174814778	8.36E-08
3h	ACA1_370080/clean/scf7180000084664:432803-432881:-	ACA1_370080	ATPdependent RNA helicase Ddx1, putative [Source:UniProtKB/TrEMBL;Acc:L8GZ36]	0.00249501	0.177303267	0.174808257	0.000440391
3h	ACA1_364570/clean/scf7180000084641:148577-148644:+	ACA1_364570	hypothetical protein	0.005747133	0.180515433	0.1747683	0.000149362
3h	ACA1_010180/clean/scf7180000083486:7166-7254:+	ACA1_010180	DUF410 family protein [Source:UniProtKB/TrEMBL;Acc:L8HD09]	0.004986297	0.1795523	0.174566003	7.51E-05
3h	ACA1_176050/clean/scf7180000084741:1114231-1114292:-	ACA1_176050	Poly [ADP-ribose] polymerase [Source:UniProtKB/TrEMBL;Acc:L8HI82]	0.001748757	0.176074967	0.17432621	1.22E-06
3h	ACA1_182410/clean/scf7180000084745:207195-207346:-	ACA1_182410	Homeobox prox 1, putative [Source:UniProtKB/TrEMBL;Acc:L8H731]	0.002735443	0.176951167	0.174215723	0.000373331
3h	ACA1_264100/clean/scf7180000084770:189710-189800:+	ACA1_264100	Calponin (CH) domain containing protein [Source:UniProtKB/TrEMBL;Acc:L8H165]	0.00760549	0.181733167	0.174127677	7.66E-06
3h	ACA1_192020/clean/scf7180000084749:75264-75417:+	ACA1_192020	Cystathionine gammasynthase [Source:UniProtKB/TrEMBL;Acc:L8GNB3]	0.001409057	0.1755235	0.174114443	6.36E-08
3h	ACA1_335940/clean/scf7180000084593:10005-10108:-	ACA1_335940	RNA recognition motif domain containing protein [Source:UniProtKB/TrEMBL;Acc:L8H3Y6]	0.001016407	0.174984267	0.17396786	2.49E-05
3h	ACA1_095060/clean/scf7180000084645:802789-802896:-	ACA1_095060	Sidoreflexin [Source:UniProtKB/TrEMBL;Acc:L8GL14]	0.043779	0.217711333	0.173932333	0.006285457
3h	ACA1_174230/clean/scf7180000084741:652526-652596:+	ACA1_174230	Rab5interacting protein (Rab5ip) [Source:UniProtKB/TrEMBL;Acc:L8HJ90]	0.000270487	0.1740167	0.173746213	1.05E-09
3h	ACA1_112010/clean/scf7180000084669:82878-82993:-	ACA1_112010	EF hand domain containing protein [Source:UniProtKB/TrEMBL;Acc:L8H5B3]	0.026881533	0.2005243	0.173642767	0.004385212
3h	ACA1_045380/clean/scf7180000084576:127090-127172:+	ACA1_045380	hypothetical protein	0.002878762	0.176456767	0.173578004	6.67E-07
3h	ACA1_002750/clean/scf7180000082937:5307-5393:-	ACA1_002750	F-box domain-containing protein [Source:UniProtKB/TrEMBL;Acc:L8GHD2]	0.02214752	0.195688067	0.173540547	0.001072582
3h	ACA1_385810/clean/scf7180000084730:274878-274964:+	ACA1_385810	Rap-GAP domain-containing protein [Source:UniProtKB/TrEMBL;Acc:L8HAQ1]	0.011866224	0.185235433	0.173369209	0.000382098
3h	ACA1_151110/clean/scf7180000084716:62053-62169:+	ACA1_151110	Sec23/Sec24 trunk domain containing protein [Source:UniProtKB/TrEMBL;Acc:L8GZW6]	0.00978357	0.18314	0.17335643	1.41E-06
3h	ACA1_366150/clean/scf7180000084641:362916-362983:-	ACA1_366150	tRNA methyltransferase, putative [Source:UniProtKB/TrEMBL;Acc:L8GMF0]	0.010272723	0.183562333	0.17328961	0.000348507
3h	ACA1_123880/clean/scf7180000084686:77196-77296:-	ACA1_123880	hypothetical protein	0.016191903	0.189135267	0.172943363	0.000568137
3h	ACA1_222720/clean/scf7180000084760:63648-63704:-	ACA1_222720	Shortchain dehydrogenase/reductase SDR, putative [Source:UniProtKB/TrEMBL;Acc:L8GV93]	0.01015226	0.183083667	0.172931407	2.46E-06
3h	ACA1_151330/clean/scf7180000084716:104062-104152:-	ACA1_151330	Purple acid phosphatase [Source:UniProtKB/TrEMBL;Acc:L8H0Q3]	0.00350116	0.176406467	0.172905307	9.65E-06
3h	ACA1_292830/clean/scf7180000084777:913434-913544:+	ACA1_292830	CULLIN_2 domain-containing protein [Source:UniProtKB/TrEMBL;Acc:L8HJK9]	0.004195073	0.177084633	0.17288956	1.79E-05
3h	ACA1_199640/clean/scf7180000084753:286810-286897:-	ACA1_199640	RFX-type winged-helix domain-containing protein [Source:UniProtKB/TrEMBL;Acc:L8H5G1]	0.003584233	0.176385133	0.1728009	0.00023427
3h	ACA1_387910/clean/scf7180000084733:88346-88509:-	ACA1_387910	P/Homo B domain-containing protein [Source:UniProtKB/TrEMBL;Acc:L8GG58]	0.002764047	0.175215967	0.17245192	2.44E-05
3h	ACA1_283010/clean/scf7180000084776:763677-763754:-	ACA1_283010	hypothetical protein	0.050594933	0.222988333	0.1723934	0.013814585
3h	ACA1_252530/clean/scf7180000084768:175248-175339:+	ACA1_252530	Protein kinase domain-containing protein [Source:UniProtKB/TrEMBL;Acc:L8HC35]	0.004487936	0.176854033	0.172366098	0.000273434
3h	ACA1_368690/clean/scf7180000084664:139566-139627:-	ACA1_368690	EF hand domain containing protein [Source:UniProtKB/TrEMBL;Acc:L8GYX7]	0.004698333	0.176992633	0.1722943	6.85E-07
3h	ACA1_398190/clean/scf7180000084759:212372-212444:-	ACA1_398190	6-phosphogluconate dehydrogenase, decarboxylating [Source:UniProtKB/TrEMBL;Acc:L8HDP8]	0.02289029	0.195151867	0.172261577	0.00917227
3h	ACA1_368430/clean/scf7180000084664:121755-121870:-	ACA1_368430	Carnitine/acylcarnitine translocase, putative [Source:UniProtKB/TrEMBL;Acc:L8GYX3]	0.00091888	0.1731661	0.17224722	7.61E-07
3h	ACA1_399900/clean/scf7180000084775:59540-59613:-	ACA1_399900	Usp domain-containing protein [Source:UniProtKB/TrEMBL;Acc:L8GID1]	0.0316269	0.203807667	0.172180767	0.000847641
3h	ACA1_073350/clean/scf7180000084603:1397461-1397571:+	ACA1_073350	AAR2 protein [Source:UniProtKB/TrEMBL;Acc:L8HGY7]	0	0.172098667	0.172098667	8.03E-06
3h	ACA1_189450/clean/scf7180000084748:39105-39209:-	ACA1_189450	Chloride channel protein [Source:UniProtKB/TrEMBL;Acc:L8GEJ2]	0.004901967	0.176986833	0.172084867	0.000222801
3h	ACA1_307520/clean/scf7180000083680:24963-25074:-	ACA1_307520	B5 domain-containing protein [Source:UniProtKB/TrEMBL;Acc:L8H4Y1]	0.000259213	0.172267	0.172007787	5.88E-09
3h	ACA1_040720/clean/scf7180000084572:63472-63558:+	ACA1_040720	Adaptin_N domain-containing protein [Source:UniProtKB/TrEMBL;Acc:L8H2X2]	0.022969233	0.194745667	0.171776433	0.000458657
3h	ACA1_176050/clean/scf7180000084741:1113757-1113831:-	ACA1_176050	Poly [ADP-ribose] polymerase [Source:UniProtKB/TrEMBL;Acc:L8HI82]	0.002654727	0.174300633	0.171645907	1.93E-07
3h	ACA1_091330/clean/scf7180000084645:89209-89285:-	ACA1_091330	Pyruvate kinase [Source:UniProtKB/TrEMBL;Acc:L8GIR3]	0.035153033	0.206795333	0.1716423	0.010765404
3h	ACA1_251580/clean/scf7180000084768:88682-88767:-	ACA1_251580	Vesicle-fusing ATPase [Source:UniProtKB/TrEMBL;Acc:L8HCZ8]	0.0070922	0.178734	0.1716418	0.001704231
3h	ACA1_276490/clean/scf7180000084774:192734-192843:+	ACA1_276490	Vesicle-associated membrane protein, putative [Source:UniProtKB/TrEMBL;Acc:L8GS45]	0.007078887	0.178686867	0.17160798	0.033216739
3h	ACA1_072330/clean/scf7180000084603:1202654-1202787:-	ACA1_072330	UBA_e1_C domain-containing protein [Source:UniProtKB/TrEMBL;Acc:L8HGS4]	0.00307746	0.174660067	0.171582607	1.10E-05
3h	ACA1_264630/clean/scf7180000084770:341467-341560:+	ACA1_264630	hypothetical protein	0.002593217	0.174166	0.171572783	7.95E-08
3h	ACA1_180780/clean/scf7180000084744:79046-79151:-	ACA1_180780	RasGEF domain containing protein [Source:UniProtKB/TrEMBL;Acc:L8GF96]	0.026381133	0.1979032	0.171522067	0.002341889
3h	ACA1_095680/clean/scf7180000084645:880247-880358:+	ACA1_095680	hypothetical protein	0.017498233	0.188886667	0.171388433	0.000274312
3h	ACA1_068120/clean/scf7180000084603:111667-111757:+	ACA1_068120	Endo/exonuclease/phosphatase domain-containing protein [Source:UniProtKB/TrEMBL;Acc:L8HEN2]	0.022959771	0.194347467	0.171387696	0.001176182
3h	ACA1_174820/clean/scf7180000084741:805288-805395:+	ACA1_174820	hypothetical protein	0.000314634	0.171693433	0.171378799	1.73E-07
3h	ACA1_237820/clean/scf7180000084763:23740-23896:-	ACA1_237820	NUDE_C domain-containing protein [Source:UniProtKB/TrEMBL;Acc:L8GKB8]	0.005657967	0.177023467	0.1713655	0.000364655
3h	ACA1_110860/clean/scf7180000084666:3063-3130:+	ACA1_110860	SID1 transmembrane family, member 1, putative [Source:UniProtKB/TrEMBL;Acc:L8H6V2]	0.012313377	0.183663367	0.17134999	0.015867071
3h	ACA1_080750/clean/scf7180000084618:76508-76641:-	ACA1_080750	ANK_REP_REGION domain-containing protein [Source:UniProtKB/TrEMBL;Acc:L8HE67]	0.006730447	0.178054367	0.17132392	7.31E-08
3h	ACA1_143780/clean/scf7180000084709:97637-97720:+	ACA1_143780	FGGAP repeat domain containing protein [Source:UniProtKB/TrEMBL;Acc:L8HH27]	0.005577117	0.1768434	0.171266283	8.84E-08
3h	ACA1_214910/clean/scf7180000084758:265369-265433:-	ACA1_214910	DPBB_1 domain-containing protein [Source:UniProtKB/TrEMBL;Acc:L8GQ91]	0.023187897	0.1944512	0.171263303	0.005807769
3h	ACA1_058710/clean/scf7180000084599:362421-362537:-	ACA1_058710	Sphase kinase-associated protein, putative [Source:UniProtKB/TrEMBL;Acc:L8GW23]	0.001969757	0.173198533	0.171228777	1.40E-12
3h	ACA1_248670/clean/scf7180000084766:107639-107748:-	ACA1_248670	CBS domain containing protein [Source:UniProtKB/TrEMBL;Acc:L8GY23]	0.006891667	0.177957633	0.171065967	5.10E-06
3h	ACA1_323120/clean/scf7180000084467:7603-7713:-	ACA1_323120	BHLH domain-containing protein [Source:UniProtKB/TrEMBL;Acc:L8GE91]	0	0.1709538	0.1709538	0.002358066
3h	ACA1_009930/clean/scf7180000083468:13249-13346:+	ACA1_009930	Acyl-CoA_dh_1 domain-containing protein [Source:UniProtKB/TrEMBL;Acc:L8GFW8]	0	0.170900833	0.170900833	1.07E-06
3h	ACA1_077090/clean/scf7180000084612:232796-232860:-	ACA1_077090	Transmembrane 9 superfamily member [Source:UniProtKB/TrEMBL;Acc:L8GLA1]	0.00367788	0.1745032	0.17082532	2.64E-05
3h	ACA1_313610/clean/scf7180000083822:10724-10831:+	ACA1_313610	Calcium-transporting ATPase [Source:UniProtKB/TrEMBL;Acc:L8GL52]	0.00810378	0.178890833	0.170787053	1.01E-05
3h	ACA1_291700/clean/scf7180000084777:812088-812182:+	ACA1_291700	hypothetical protein	0.005676367	0.176414533	0.170738167	0.001396598
3h	ACA1_140180/clean/scf7180000084707:38386-38488:-	ACA1_140180	hypothetical protein	0.009416103	0.180138367	0.170722263	9.91E-06
3h	ACA1_296890/clean/scf7180000084777:1426754-1426844:+	ACA1_296890	Dynamin domain containing protein [Source:UniProtKB/TrEMBL;Acc:L8HLM2]	0.009304363	0.179863633	0.17055927	2.89E-06
3h	ACA1_131570/clean/scf7180000084699:90127-90243:-	ACA1_131570	Transmembrane proteins 14c protein [Source:UniProtKB/TrEMBL;Acc:L8H2Q4]	0.001647418	0.172140067	0.170492649	4.64E-06
3h	ACA1_277780/clean/scf7180000084776:98598-98681:+	ACA1_277780	Ca2+dependent endoplasmic reticulum nucleoside diphosphatase isoform 3, putative [Source:UniProtKB/TrEMBL;Acc:L8H7Y3]	0.001632943	0.171914333	0.17028139	5.67E-05
3h	ACA1_396550/clean/scf7180000084759:90150-90218:-	ACA1_396550	hypothetical protein	0.009010297	0.179289867	0.17027957	6.18E-05
3h	ACA1_298850/clean/scf7180000082992:40874-40985:+	ACA1_298850	Protein transport protein SEC23 [Source:UniProtKB/TrEMBL;Acc:L8H0B4]	0.000410054	0.1705172	0.170107146	3.23E-09
3h	ACA1_298650/clean/scf7180000082992:11902-12040:+	ACA1_298650	hypothetical protein	0.001384797	0.1714731	0.170088303	1.25E-05
3h	ACA1_164750/clean/scf7180000084729:265837-265905:-	ACA1_164750	Carbonic anhydrase [Source:UniProtKB/TrEMBL;Acc:L8GR38]	0.003558733	0.173527367	0.169968633	5.47E-08
3h	ACA1_037480/clean/scf7180000084567:100048-100120:-	ACA1_037480	AB hydrolase-1 domain-containing protein [Source:UniProtKB/TrEMBL;Acc:L8H038]	0.073620833	0.243561333	0.1699405	0.016991802
3h	ACA1_277830/clean/scf7180000084776:110192-110262:+	ACA1_277830	LIM domain containing protein [Source:UniProtKB/TrEMBL;Acc:L8H6H8]	0.030932567	0.2006999	0.169767333	0.006133363
3h	ACA1_097290/clean/scf7180000084645:1210554-1210629:+	ACA1_097290	hypothetical protein	0.0275052	0.1971899	0.1696847	0.001181435
3h	ACA1_091560/clean/scf7180000084645:149513-149602:+	ACA1_091560	UBP-type domain-containing protein [Source:UniProtKB/TrEMBL;Acc:L8GKE3]	0.010496137	0.180100267	0.16960413	0.013712378
3h	ACA1_164340/clean/scf7180000084729:212656-212731:+	ACA1_164340	Fe2OG dioxygenase domain-containing protein [Source:UniProtKB/TrEMBL;Acc:L8GR13]	0.030598033	0.200016067	0.169418033	0.034335647
3h	ACA1_292830/clean/scf7180000084777:913130-913236:+	ACA1_292830	CULLIN_2 domain-containing protein [Source:UniProtKB/TrEMBL;Acc:L8HJK9]	0.00637628	0.175736	0.16935972	2.83E-05
3h	ACA1_189830/clean/scf7180000084748:98108-98180:+	ACA1_189830	Partner of sld five, psf2 protein [Source:UniProtKB/TrEMBL;Acc:L8GE47]	0	0.169295133	0.169295133	0.005709867
3h	ACA1_341030/clean/scf7180000084620:38575-38669:+	ACA1_341030	CRAL/TRIO domain containing protein [Source:UniProtKB/TrEMBL;Acc:L8GS92]	0.002871213	0.1721638	0.169292587	8.83E-08
3h	ACA1_163820/clean/scf7180000084729:74629-74698:-	ACA1_163820	MFS domain-containing protein [Source:UniProtKB/TrEMBL;Acc:L8GQW1]	0.001802373	0.171071333	0.16926896	9.35E-05
3h	ACA1_112170/clean/scf7180000084669:135547-135637:+	ACA1_112170	PPM-type phosphatase domain-containing protein [Source:UniProtKB/TrEMBL;Acc:L8H322]	0.01781046	0.186995067	0.169184607	0.002188449
3h	ACA1_033590/clean/scf7180000084559:48497-48561:-	ACA1_033590	Carn_acyltransf domain-containing protein [Source:UniProtKB/TrEMBL;Acc:L8GJ57]	0.017581267	0.186707	0.169125733	3.84E-05
3h	ACA1_235050/clean/scf7180000084762:328288-328411:+	ACA1_235050	RNA ligase, putative [Source:UniProtKB/TrEMBL;Acc:L8H1C3]	0.0047619	0.1736196	0.1688577	0.001947722
3h	ACA1_365720/clean/scf7180000084641:305213-305280:+	ACA1_365720	AAA domain-containing protein [Source:UniProtKB/TrEMBL;Acc:L8GLT4]	1.16E-05	0.1688639	0.168852274	1.16E-08
3h	ACA1_279000/clean/scf7180000084776:291746-291832:+	ACA1_279000	hypothetical protein	0.01193964	0.1807162	0.16877656	0.000725
3h	ACA1_267690/clean/scf7180000084770:869899-870022:-	ACA1_267690	Cytochrome b-c1 complex subunit 6 [Source:UniProtKB/TrEMBL;Acc:L8H1V3]	0.00353992	0.1722807	0.16874078	4.83E-09
3h	ACA1_086070/clean/scf7180000084636:27449-27666:-	ACA1_086070	Serine/threonine kinase [Source:UniProtKB/TrEMBL;Acc:L8HJ13]	0.002075603	0.170815767	0.168740163	5.33E-05
3h	ACA1_058560/clean/scf7180000084599:350338-350439:-	ACA1_058560	PPM-type phosphatase domain-containing protein [Source:UniProtKB/TrEMBL;Acc:L8GW17]	0.067335733	0.23605591	0.168720177	0.010411326
3h	ACA1_112170/clean/scf7180000084669:135162-135249:+	ACA1_112170	PPM-type phosphatase domain-containing protein [Source:UniProtKB/TrEMBL;Acc:L8H322]	0.002441219	0.171100733	0.168659514	3.77E-05
3h	ACA1_201490/clean/scf7180000084753:572184-572362:+	ACA1_201490	hypothetical protein	0.10483	0.273489	0.168659	0.022378238
3h	ACA1_031650/clean/scf7180000084522:38953-39039:-	ACA1_031650	Endo/exonuclease/phosphatase domain-containing protein [Source:UniProtKB/TrEMBL;Acc:L8GSN6]	0.0103622	0.178907367	0.168545167	0.013212516
3h	ACA1_385120/clean/scf7180000084730:137817-137905:+	ACA1_385120	Non-specific serine/threonine protein kinase [Source:UniProtKB/TrEMBL;Acc:L8HBH8]	0.003583447	0.1721165	0.168533053	4.74E-07
3h	ACA1_051530/clean/scf7180000084592:28179-28240:-	ACA1_051530	MFS domain-containing protein [Source:UniProtKB/TrEMBL;Acc:L8GPJ8]	0.005649733	0.174159267	0.168509533	0.003796618
3h	ACA1_054820/clean/scf7180000084594:579274-579351:-	ACA1_054820	Tetratricopeptide repeat domain containing protein [Source:UniProtKB/TrEMBL;Acc:L8H8E7]	0	0.168404833	0.168404833	0.000104687
3h	ACA1_181830/clean/scf7180000084745:52451-52564:-	ACA1_181830	PIPK domain-containing protein [Source:UniProtKB/TrEMBL;Acc:L8H972]	0.004641733	0.173036767	0.168395033	0.008888027
3h	ACA1_036990/clean/scf7180000084567:30729-30811:-	ACA1_036990	M20_dimer domain-containing protein [Source:UniProtKB/TrEMBL;Acc:L8H0T8]	0.015093867	0.183483733	0.168389867	1.96E-06
3h	ACA1_174180/clean/scf7180000084741:634176-634269:+	ACA1_174180	hypothetical protein	0.798349667	0.966597	0.168247333	0.002756804
3h	ACA1_234780/clean/scf7180000084762:316227-316339:-	ACA1_234780	hypothetical protein	0.006072653	0.174182533	0.16810988	9.51E-06
3h	ACA1_178210/clean/scf7180000084743:180321-180597:-	ACA1_178210	Eukaryotic translation initiation factor 6 [Source:UniProtKB/TrEMBL;Acc:L8GTE0]	0.023941067	0.191951867	0.1680108	0.007255759
3h	ACA1_201170/clean/scf7180000084753:498888-499010:+	ACA1_201170	RWD domain-containing protein [Source:UniProtKB/TrEMBL;Acc:L8H2L8]	0.027274943	0.195083333	0.16780839	0.00705992
3h	ACA1_040470/clean/scf7180000084572:11824-11910:+	ACA1_040470	Leuk-A4-hydro_C domain-containing protein [Source:UniProtKB/TrEMBL;Acc:L8H0K2]	0.002655578	0.170438967	0.167783389	2.01E-08
3h	ACA1_296390/clean/scf7180000084777:1333278-1333379:+	ACA1_296390	hypothetical protein	0.003663	0.1713748	0.1677118	0.000161991
3h	ACA1_121830/clean/scf7180000084682:104007-104118:-	ACA1_121830	COesterase domain-containing protein [Source:UniProtKB/TrEMBL;Acc:L8GEL0]	0.00950959	0.1770777	0.16756811	0.000587525
3h	ACA1_340680/clean/scf7180000084620:14430-14517:-	ACA1_340680	hypothetical protein	0.006900523	0.174371333	0.16747081	2.27E-07
3h	ACA1_072960/clean/scf7180000084603:1347071-1347199:+	ACA1_072960	F-box domain-containing protein [Source:UniProtKB/TrEMBL;Acc:L8HEK9]	0.094159367	0.261564333	0.167404967	0.044386937
3h	ACA1_182310/clean/scf7180000084745:180934-181011:-	ACA1_182310	Adenylosuccinate lyase [Source:UniProtKB/TrEMBL;Acc:L8H721]	0.042293167	0.209696767	0.1674036	0.002241879
3h	ACA1_174560/clean/scf7180000084741:738048-738242:-	ACA1_174560	RRM domain-containing protein [Source:UniProtKB/TrEMBL;Acc:L8HHX6]	0.013452367	0.180709333	0.167256967	0.002059657
3h	ACA1_049530/clean/scf7180000084589:1218-1285:-	ACA1_049530	RNA_ligase domain-containing protein [Source:UniProtKB/TrEMBL;Acc:L8H546]	0	0.167256133	0.167256133	0.01293906
3h	ACA1_169070/clean/scf7180000084739:14716-14815:+	ACA1_169070	Integral membrane protein 1 isoform 5, putative [Source:UniProtKB/TrEMBL;Acc:L8HFG1]	0.000215559	0.167310533	0.167094974	1.67E-08
3h	ACA1_389320/clean/scf7180000084733:229178-229256:+	ACA1_389320	BTB domain-containing protein [Source:UniProtKB/TrEMBL;Acc:M0QSM2]	0.002564103	0.1696411	0.167076997	0.000286745
3h	ACA1_287820/clean/scf7180000084777:88526-88598:+	ACA1_287820	TRANSKETOLASE_1 domain-containing protein [Source:UniProtKB/TrEMBL;Acc:L8HL41]	0.000977927	0.167865667	0.16688774	6.66E-08
3h	ACA1_384590/clean/scf7180000084730:60468-60572:-	ACA1_384590	RAB1B, member RAS oncogene family [Source:UniProtKB/TrEMBL;Acc:L8H896]	0.000609804	0.167373967	0.166764163	1.88E-07
3h	ACA1_274880/clean/scf7180000084773:132440-132516:+	ACA1_274880	AB hydrolase-1 domain-containing protein [Source:UniProtKB/TrEMBL;Acc:L8GI88]	0.004329	0.171031433	0.166702433	0.000540659
3h	ACA1_131800/clean/scf7180000084699:133958-134072:+	ACA1_131800	hypothetical protein	0.017101907	0.183799033	0.166697127	0.000135558
3h	ACA1_017740/clean/scf7180000083876:3645-3741:+	ACA1_017740	5'-3' exoribonuclease 1 [Source:UniProtKB/TrEMBL;Acc:L8GMR4]	0	0.1666608	0.1666608	5.17E-07
3h	ACA1_360080/clean/scf7180000084622:43799-43894:+	ACA1_360080	hypothetical protein	0	0.166653067	0.166653067	4.95E-05
3h	ACA1_216320/clean/scf7180000084758:468514-468622:+	ACA1_216320	Homogentisate 1,2dioxygenase [Source:UniProtKB/TrEMBL;Acc:L8GQ83]	0.006077567	0.172660733	0.166583167	1.30E-06
3h	ACA1_270180/clean/scf7180000084770:1069322-1069401:-	ACA1_270180	Mannosyltransferase [Source:UniProtKB/TrEMBL;Acc:L8H230]	0.018993583	0.185545633	0.16655205	0.005499115
3h	ACA1_131910/clean/scf7180000084699:171029-171113:-	ACA1_131910	EF hand domain containing protein [Source:UniProtKB/TrEMBL;Acc:L8GMI5]	0.001236324	0.167776333	0.16654001	3.50E-07
3h	ACA1_388770/clean/scf7180000084733:164111-164196:+	ACA1_388770	Poly(A) polymerase central domain containing protein [Source:UniProtKB/TrEMBL;Acc:L8GG84]	0	0.166426933	0.166426933	4.07E-05
3h	ACA1_043610/clean/scf7180000084573:184596-184718:-	ACA1_043610	FYVE-type domain-containing protein [Source:UniProtKB/TrEMBL;Acc:L8GV86]	0	0.1663775	0.1663775	0.003246401
3h	ACA1_009930/clean/scf7180000083468:12428-12510:+	ACA1_009930	Acyl-CoA_dh_1 domain-containing protein [Source:UniProtKB/TrEMBL;Acc:L8GFW8]	0.000729851	0.167077433	0.166347583	4.11E-07
3h	ACA1_184420/clean/scf7180000084745:640355-640461:+	ACA1_184420	Cathepsin Llike proteinase [Source:UniProtKB/TrEMBL;Acc:L8H8J9]	0.001689587	0.1679393	0.166249713	9.01E-08
3h	ACA1_070620/clean/scf7180000084603:749729-749797:+	ACA1_070620	Myotubularin phosphatase domain-containing protein [Source:UniProtKB/TrEMBL;Acc:L8HFC4]	0.000691683	0.166879333	0.16618765	3.91E-07
3h	ACA1_252210/clean/scf7180000084768:143683-143759:+	ACA1_252210	Protein kinase domain-containing protein [Source:UniProtKB/TrEMBL;Acc:L8HAM0]	0	0.166054133	0.166054133	0.00286636
3h	ACA1_087870/clean/scf7180000084639:119651-119748:-	ACA1_087870	Peptidase S53 domain-containing protein [Source:UniProtKB/TrEMBL;Acc:L8GUK2]	0	0.166027267	0.166027267	1.68E-07
3h	ACA1_195000/clean/scf7180000084751:53109-53191:+	ACA1_195000	CAP-Gly domain-containing protein [Source:UniProtKB/TrEMBL;Acc:L8H6Q6]	0.00579474	0.1718191	0.16602436	4.24E-05
3h	ACA1_142680/clean/scf7180000084708:219988-220205:+	ACA1_142680	unspecified product	0.01435884	0.180271433	0.165912593	0.000520012
3h	ACA1_220770/clean/scf7180000084758:963845-963938:-	ACA1_220770	cobW domain-containing protein [Source:UniProtKB/TrEMBL;Acc:L8GT55]	0.026620633	0.192405333	0.1657847	0.000177698

3h	ACA1_230240/clean/scf7180000084761:332157-332222:-	ACA1_230240	Calponin-homology (CH) domain-containing protein [Source:UniProtKB/TrEMBL;Acc:L8H830]	0.020664667	0.186254033	0.165589367	0.000106138
3h	ACA1_320840/clean/scf7180000084416:33624-33801:+	ACA1_320840	HSP91 (Heat shock protein 91), putative [Source:UniProtKB/TrEMBL;Acc:L8GTS5]	0.000339343	0.1659264	0.165587057	1.82E-10
3h	ACA1_174420/clean/scf7180000084741:706041-706113:+	ACA1_174420	Diacylglycerol kinase [Source:UniProtKB/TrEMBL;Acc:L8HK54]	0.00889972	0.174476633	0.165576913	0.000192804
3h	ACA1_020770/clean/scf7180000084095:2158-2243:+	ACA1_020770	Multisensor hybrid histidine kinase [Source:UniProtKB/TrEMBL;Acc:L8GWG6]	0.009043627	0.174539	0.165495373	0.000461069
3h	ACA1_050340/clean/scf7180000084589:68371-68446:-	ACA1_050340	BTB domain-containing protein [Source:UniProtKB/TrEMBL;Acc:M0QSG1]	0.023840567	0.189311667	0.1654711	0.000561554
3h	ACA1_328790/clean/scf7180000084560:29939-30022:-	ACA1_328790	Aryl-alcohol dehydrogenase (NADP+), putative [Source:UniProtKB/TrEMBL;Acc:L8GQ90]	0.000805268	0.166173167	0.165367899	2.76E-07
3h	ACA1_164750/clean/scf7180000084729:264700-264797:-	ACA1_164750	Carbonic anhydrase [Source:UniProtKB/TrEMBL;Acc:L8GR38]	0.008918617	0.1741818	0.165263183	6.29E-06
3h	ACA1_206050/clean/scf7180000084755:70428-70490:+	ACA1_206050	ANK_REP_REGION domain-containing protein [Source:UniProtKB/TrEMBL;Acc:L8H7K6]	0	0.165259167	0.165259167	3.29E-05
3h	ACA1_095680/clean/scf7180000084645:881189-881256:+	ACA1_095680	hypothetical protein	0.015489567	0.180747133	0.165257567	0.000767924
3h	ACA1_206870/clean/scf7180000084757:36258-36355:-	ACA1_206870	hypothetical protein	0.002548983	0.167687633	0.16513865	5.53E-09
3h	ACA1_133860/clean/scf7180000084701:100687-100760:-	ACA1_133860	Aa_trans domain-containing protein [Source:UniProtKB/TrEMBL;Acc:L8H539]	0.033173733	0.198284867	0.165111133	0.009074143
3h	ACA1_198300/clean/scf7180000084753:75387-75504:-	ACA1_198300	hypothetical protein	0.00104606	0.1660354	0.16498934	1.49E-05
3h	ACA1_059490/clean/scf7180000084599:454156-454266:+	ACA1_059490	Saposin B domain containing protein [Source:UniProtKB/TrEMBL;Acc:L8GWD6]	0.000478152	0.165416567	0.164938415	3.83E-12
3h	ACA1_093350/clean/scf7180000084645:523539-523621:+	ACA1_093350	Fbox domain containing protein [Source:UniProtKB/TrEMBL;Acc:L8GIV6]	0	0.164916867	0.164916867	0.00016402
3h	ACA1_140730/clean/scf7180000084707:109142-109262:-	ACA1_140730	Kelch repeat protein [Source:UniProtKB/TrEMBL;Acc:L8GGS1]	0.00637075	0.1712839	0.16491315	0.0005219
3h	ACA1_166910/clean/scf7180000084736:27215-27313:-	ACA1_166910	Amine oxidase family protein [Source:UniProtKB/TrEMBL;Acc:L8H242]	0.0138889	0.178779633	0.164890733	0.00499852
3h	ACA1_062690/clean/scf7180000084599:1274501-1274617:-	ACA1_062690	DEAD/DEAH box helicase domain containing protein [Source:UniProtKB/TrEMBL;Acc:L8GWZ4]	0.004694833	0.169319633	0.1646248	0.000510937
3h	ACA1_199010/clean/scf7180000084753:191514-191621:+	ACA1_199010	JmjC domain-containing protein [Source:UniProtKB/TrEMBL;Acc:L8H2E5]	0.001811593	0.166261	0.164449407	1.67E-05
3h	ACA1_358790/clean/scf7180000084742:36329-36399:-	ACA1_358790	hypothetical protein	0	0.164416767	0.164416767	2.23E-05
3h	ACA1_376490/clean/scf7180000084685:90232-90349:-	ACA1_376490	TIP41like family protein [Source:UniProtKB/TrEMBL;Acc:L8HIG8]	0.0333246	0.197688133	0.164363533	0.005931243
3h	ACA1_375810/clean/scf7180000084685:334-445:+	ACA1_375810	hypothetical protein	0.0104718	0.1748124	0.1643406	0.0002007
3h	ACA1_244750/clean/scf7180000084763:407279-407390:-	ACA1_244750	Oxysterol binding protein [Source:UniProtKB/TrEMBL;Acc:L8GK15]	0.005764268	0.170046767	0.164282498	1.17E-05
3h	ACA1_307530/clean/scf7180000083680:28579-28707:+	ACA1_307530	Acyltransferase [Source:UniProtKB/TrEMBL;Acc:L8H6B3]	0.0507481	0.2149017	0.1641536	0.024439035
3h	ACA1_274880/clean/scf7180000084773:132286-132364:+	ACA1_274880	AB hydrolase-1 domain-containing protein [Source:UniProtKB/TrEMBL;Acc:L8GI88]	0.00777209	0.171903167	0.164131077	0.00047882
3h	ACA1_044940/clean/scf7180000084576:66405-66558:-	ACA1_044940	BTB domain-containing protein [Source:UniProtKB/TrEMBL;Acc:L8GYY6]	0.0372289	0.201358933	0.164130033	0.028676678
3h	ACA1_027050/clean/scf7180000084464:32526-32633:-	ACA1_027050	hypothetical protein	0.007876283	0.171938567	0.164062283	3.32E-06
3h	ACA1_063500/clean/scf7180000084599:1432406-1432484:+	ACA1_063500	U-box domain-containing protein [Source:UniProtKB/TrEMBL;Acc:L8GZS8]	0.000298632	0.164305533	0.164006901	8.03E-06
3h	ACA1_164040/clean/scf7180000084729:145458-145531:-	ACA1_164040	Signal peptide binding domain containing protein [Source:UniProtKB/TrEMBL;Acc:L8GRG0]	0.008000267	0.171873233	0.163872967	1.26E-05
3h	ACA1_195050/clean/scf7180000084751:61677-61794:+	ACA1_195050	Cyclin N-terminal domain-containing protein [Source:UniProtKB/TrEMBL;Acc:L8H6R3]	0.018216567	0.182069033	0.163852467	0.002647045
3h	ACA1_295580/clean/scf7180000084777:1177892-1178010:+	ACA1_295580	Solute carrier family protein [Source:UniProtKB/TrEMBL;Acc:L8HJT6]	0.035082967	0.198814467	0.1637315	0.008795014
3h	ACA1_248600/clean/scf7180000084766:93525-93625:+	ACA1_248600	DUF4114 domain-containing protein [Source:UniProtKB/TrEMBL;Acc:L8GXW7]	0.755409667	0.919127	0.163717333	0.010358348
3h	ACA1_146600/clean/scf7180000084713:65941-66011:-	ACA1_146600	Protein kinase domain containing protein [Source:UniProtKB/TrEMBL;Acc:L8GWQ5]	0.00685293	0.170327233	0.163474303	3.39E-06
3h	ACA1_199060/clean/scf7180000084753:210841-210936:-	ACA1_199060	HECT domain-containing protein [Source:UniProtKB/TrEMBL;Acc:L8H2E7]	0	0.163395	0.163395	0.000420792
3h	ACA1_144250/clean/scf7180000084709:183524-183608:-	ACA1_144250	SH2 domain containing protein [Source:UniProtKB/TrEMBL;Acc:L8HEU2]	0.002343386	0.165731233	0.163387847	5.04E-06
3h	ACA1_291470/clean/scf7180000084777:753534-753615:-	ACA1_291470	20S proteasome subunit beta 3, putative [Source:UniProtKB/TrEMBL;Acc:L8HIC0]	0.00685445	0.1701422	0.16328775	5.55E-07
3h	ACA1_075030/clean/scf7180000084608:129588-129690:+	ACA1_075030	Serine carboxypeptidase [Source:UniProtKB/TrEMBL;Acc:L8HFJ6]	0.00167188	0.164912833	0.163240953	5.45E-06
3h	ACA1_382630/clean/scf7180000084721:88162-88235:-	ACA1_382630	LBP / BPI / CETP family, Cterminal domain containing protein [Source:UniProtKB/TrEMBL;Acc:L8GVZ7]	0.00295411	0.166154033	0.163199923	3.81E-07
3h	ACA1_322560/clean/scf7180000084466:19665-19736:+	ACA1_322560	Tr-type G domain-containing protein [Source:UniProtKB/TrEMBL;Acc:L8HB34]	0.000296718	0.163490333	0.163193615	4.93E-06
3h	ACA1_369260/clean/scf7180000084664:285444-285509:+	ACA1_369260	hypothetical protein	0.00573924	0.168815933	0.163076693	4.40E-07
3h	ACA1_309840/clean/scf7180000083767:16985-17055:+	ACA1_309840	1-aminocyclopropane-1-carboxylate deaminase [Source:UniProtKB/TrEMBL;Acc:L8HH90]	0.005325217	0.168374767	0.16304955	1.50E-09
3h	ACA1_091140/clean/scf7180000084645:65620-65691:-	ACA1_091140	BTB/POZ domain containing protein [Source:UniProtKB/TrEMBL;Acc:L8GIG3]	0.003578763	0.1665632	0.162984437	0.00011364
3h	ACA1_064340/clean/scf7180000084599:1594023-1594085:-	ACA1_064340	VASt domain-containing protein [Source:UniProtKB/TrEMBL;Acc:L8GWR3]	0.003076313	0.165956833	0.16288052	0.000519557
3h	ACA1_163840/clean/scf7180000084729:89332-89428:+	ACA1_163840	Ras family protein [Source:UniProtKB/TrEMBL;Acc:L8GTU6]	0.0371543	0.199942	0.1627877	0.007975126
3h	ACA1_058320/clean/scf7180000084599:299449-299534:+	ACA1_058320	GPR1/FUN34/yaaH family protein [Source:UniProtKB/TrEMBL;Acc:L8GYJ8]	0.019719333	0.182395033	0.1626757	0.015934651
3h	ACA1_181960/clean/scf7180000084745:92143-92210:+	ACA1_181960	WD_REPEATS_REGION domain-containing protein [Source:UniProtKB/TrEMBL;Acc:L8H808]	0.00700067	0.169625433	0.162624763	1.31E-05
3h	ACA1_184610/clean/scf7180000084746:9779-9858:+	ACA1_184610	Protein kinase domain containing protein [Source:UniProtKB/TrEMBL;Acc:L8H456]	0.057801233	0.220399567	0.162598333	0.021986331
3h	ACA1_187340/clean/scf7180000084747:39349-39465:-	ACA1_187340	MFS domain-containing protein [Source:UniProtKB/TrEMBL;Acc:L8GTA9]	0.011667553	0.174179467	0.162511913	0.000731591
3h	ACA1_283210/clean/scf7180000084776:788531-788641:-	ACA1_283210	Leucine rich repeat domain containing protein [Source:UniProtKB/TrEMBL;Acc:L8H6K8]	0.009192667	0.171616267	0.1624236	0.002560169
3h	ACA1_140190/clean/scf7180000084707:40333-40423:+	ACA1_140190	Cysteine protease [Source:UniProtKB/TrEMBL;Acc:L8GGF1]	0.006211277	0.1685549	0.162343623	0.000226755
3h	ACA1_382270/clean/scf7180000084721:14827-14892:-	ACA1_382270	ATPase/histidine kinase/DNA gyrase B/HSP90 domain containing protein [Source:UniProtKB/TrEMBL;Acc:L8GU96]	0.0221982	0.1844454	0.1622472	0.001801706
3h	ACA1_138060/clean/scf7180000084703:84282-84358:-	ACA1_138060	hypothetical protein	0.00178253	0.163987533	0.162205003	5.25E-05
3h	ACA1_189420/clean/scf7180000084748:32507-32602:+	ACA1_189420	Protein disulfideisomerase domain containing protein [Source:UniProtKB/TrEMBL;Acc:L8GE28]	0.000977517	0.163151267	0.16217375	7.04E-07
3h	ACA1_043250/clean/scf7180000084573:146620-146705:+	ACA1_043250	Ubiquitin-like domain-containing protein [Source:UniProtKB/TrEMBL;Acc:L8GUP8]	0.00170068	0.1637131	0.16201242	2.07E-05
3h	ACA1_065240/clean/scf7180000084599:1831760-1831858:+	ACA1_065240	Glutaredoxin domain-containing protein [Source:UniProtKB/TrEMBL;Acc:L8GXM1]	0.001125806	0.163066033	0.161940227	9.78E-12
3h	ACA1_158980/clean/scf7180000084724:306739-307046:-	ACA1_158980	GATA-type domain-containing protein [Source:UniProtKB/TrEMBL;Acc:L8HA60]	0.040149433	0.201959	0.161809567	0.04045936
3h	ACA1_289050/clean/scf7180000084777:344506-344578:+	ACA1_289050	Protein kinase domain-containing protein [Source:UniProtKB/TrEMBL;Acc:L8HKI3]	0.003407554	0.1651956	0.161788046	2.86E-06
3h	ACA1_203980/clean/scf7180000084754:243060-243158:+	ACA1_203980	hypothetical protein	0.002889805	0.164660067	0.161770262	2.15E-06
3h	ACA1_383450/clean/scf7180000084721:168645-168722:-	ACA1_383450	G protein coupled receptor, putative [Source:UniProtKB/TrEMBL;Acc:L8GUE6]	0.011352293	0.173103667	0.161751373	3.12E-07
3h	ACA1_296560/clean/scf7180000084777:1383740-1383822:-	ACA1_296560	CitMHS domain-containing protein [Source:UniProtKB/TrEMBL;Acc:L8HLL2]	0.001112843	0.162831467	0.161718624	2.26E-06
3h	ACA1_128450/clean/scf7180000084697:59944-60044:-	ACA1_128450	Adenine nucleotide translocator, putative [Source:UniProtKB/TrEMBL;Acc:L8GUU6]	0.000382133	0.1620353	0.161653167	1.27E-29
3h	ACA1_264610/clean/scf7180000084770:336797-336936:-	ACA1_264610	Peptidase_M28 domain-containing protein [Source:UniProtKB/TrEMBL;Acc:L8H243]	0	0.1613419	0.1613419	6.71E-08
3h	ACA1_289990/clean/scf7180000084777:522410-522502:+	ACA1_289990	hypothetical protein	0.004719157	0.1660569	0.161337743	0.000396193
3h	ACA1_175820/clean/scf7180000084741:1068389-1068494:-	ACA1_175820	F-box domain-containing protein [Source:UniProtKB/TrEMBL;Acc:L8HI73]	0.024100467	0.185409667	0.1613092	0.003979072
3h	ACA1_309360/clean/scf7180000083758:11456-11535:-	ACA1_309360	hypothetical protein	0.021259567	0.182445433	0.161185867	0.031890608
3h	ACA1_091850/clean/scf7180000084645:216915-217022:-	ACA1_091850	hypothetical protein	0.00148743	0.1625891	0.16110167	0.002952886
3h	ACA1_142090/clean/scf7180000084708:85159-85246:+	ACA1_142090	rRNA metabolism protein, SBDS family [Source:UniProtKB/TrEMBL;Acc:L8HCP3]	0.00212842	0.1631127	0.16098428	3.73E-05
3h	ACA1_067410/clean/scf7180000084601:17458-17541:-	ACA1_067410	F-box domain-containing protein [Source:UniProtKB/TrEMBL;Acc:L8GTP9]	0.045733532	0.2067166	0.160983068	0.017575778
3h	ACA1_233420/clean/scf7180000084762:137341-137460:+	ACA1_233420	hypothetical protein	0.002003842	0.162831133	0.160827291	9.43E-08
3h	ACA1_193200/clean/scf7180000084749:209503-209581:+	ACA1_193200	hypothetical protein	0.018526767	0.179315267	0.1607885	0.001095949
3h	ACA1_238390/clean/scf7180000084763:83714-83806:-	ACA1_238390	Coronin [Source:UniProtKB/TrEMBL;Acc:L8GJQ2]	0	0.1607869	0.1607869	3.80E-08
3h	ACA1_073460/clean/scf7180000084603:1423073-1423177:-	ACA1_073460	ABC exporter, ATPbinding protein [Source:UniProtKB/TrEMBL;Acc:L8HEP2]	0.002521307	0.1633006	0.160779293	2.01E-06
3h	ACA1_065000/clean/scf7180000084599:1749287-1749369:+	ACA1_065000	hypothetical protein	0	0.160738367	0.160738367	0.000753155
3h	ACA1_087450/clean/scf7180000084639:32430-32501:-	ACA1_087450	Ras subfamily protein [Source:UniProtKB/TrEMBL;Acc:L8GVE7]	0.025568167	0.1860844	0.160516233	0.018944189
3h	ACA1_096520/clean/scf7180000084645:1033555-1033632:-	ACA1_096520	Cytochrome p450 superfamily protein [Source:UniProtKB/TrEMBL;Acc:L8GLA2]	0	0.160407667	0.160407667	0.041684777
3h	ACA1_119220/clean/scf7180000084672:44936-45015:+	ACA1_119220	Peptidylprolyl isomerase [Source:UniProtKB/TrEMBL;Acc:L8HKC1]	0.003161069	0.163541	0.160379931	6.93E-05
3h	ACA1_395230/clean/scf7180000084756:159323-159712:+	ACA1_395230	WWE domain-containing protein [Source:UniProtKB/TrEMBL;Acc:L8H2U0]	0.013177833	0.173545333	0.1603675	0.007551484
3h	ACA1_341030/clean/scf7180000084620:38157-38274:+	ACA1_341030	CRAL/TRIO domain containing protein [Source:UniProtKB/TrEMBL;Acc:L8GS92]	0.000278746	0.160606367	0.160327621	3.30E-10
3h	ACA1_068350/clean/scf7180000084603:177206-177291:+	ACA1_068350	hypothetical protein	0.00090965	0.1612001	0.16029045	2.13E-06
3h	ACA1_228760/clean/scf7180000084761:206817-206942:+	ACA1_228760	Mitochondrial carrier protein [Source:UniProtKB/TrEMBL;Acc:L8H8X8]	0.032188	0.192444	0.160256	0.004344916
3h	ACA1_257560/clean/scf7180000084769:157626-157711:-	ACA1_257560	zf-3CxxC domain-containing protein [Source:UniProtKB/TrEMBL;Acc:L8GH91]	0.00794145	0.1678938	0.15995235	2.10E-06
3h	ACA1_165510/clean/scf7180000084732:35063-35166:+	ACA1_165510	WD_REPEATS_REGION domain-containing protein [Source:UniProtKB/TrEMBL;Acc:L8HIV6]	0.006569413	0.166465833	0.15989642	3.68E-06
3h	ACA1_315060/clean/scf7180000083887:26351-26440:-	ACA1_315060	AAA domain-containing protein [Source:UniProtKB/TrEMBL;Acc:L8GZM9]	0.001591552	0.1614503	0.159858748	1.82E-08
3h	ACA1_053890/clean/scf7180000084594:344423-344550:-	ACA1_053890	Aconitate hydratase, mitochondrial [Source:UniProtKB/TrEMBL;Acc:L8H690]	0	0.159732267	0.159732267	1.06E-08
3h	ACA1_110060/clean/scf7180000084663:49091-49166:-	ACA1_110060	Initiation factor, subunit 2 family protein [Source:UniProtKB/TrEMBL;Acc:L8HHD7]	0.008281847	0.167949833	0.159667987	0.000145465
3h	ACA1_183960/clean/scf7180000084745:528585-528682:-	ACA1_183960	Phosphoesterase family protein [Source:UniProtKB/TrEMBL;Acc:L8H8F6]	0.005333333	0.16482	0.159486667	6.87E-05
3h	ACA1_201650/clean/scf7180000084753:609141-609217:-	ACA1_201650	DNA repair protein RAD51 homolog [Source:UniProtKB/TrEMBL;Acc:L8H3I3]	0.003687237	0.162998	0.159310763	3.68E-07
3h	ACA1_195000/clean/scf7180000084751:51140-51214:+	ACA1_195000	CAP-Gly domain-containing protein [Source:UniProtKB/TrEMBL;Acc:L8H6Q6]	0	0.159294667	0.159294667	9.84E-07
3h	ACA1_175510/clean/scf7180000084741:973602-973687:+	ACA1_175510	Xylose isomerase [Source:UniProtKB/TrEMBL;Acc:L8HKE1]	0.000485403	0.1595887	0.159103297	1.06E-07
3h	ACA1_101510/clean/scf7180000084649:80750-80837:+	ACA1_101510	Leucine-tRNA ligase [Source:UniProtKB/TrEMBL;Acc:L8GGB5]	0	0.159088167	0.159088167	6.21E-08
3h	ACA1_382280/clean/scf7180000084721:21020-21083:-	ACA1_382280	Aminotran_1_2 domain-containing protein [Source:UniProtKB/TrEMBL;Acc:L8GUT4]	0	0.159078967	0.159078967	0.000451962
3h	ACA1_236110/clean/scf7180000084762:406081-406194:-	ACA1_236110	PPM-type phosphatase domain-containing protein [Source:UniProtKB/TrEMBL;Acc:L8H2W4]	0.024315373	0.1832345	0.158919127	0.000829885
3h	ACA1_040640/clean/scf7180000084572:34263-34339:+	ACA1_040640	MFS domain-containing protein [Source:UniProtKB/TrEMBL;Acc:L8H1W1]	0.04057057	0.199428667	0.158858097	0.015525961
3h	ACA1_325290/clean/scf7180000084475:50161-50247:-	ACA1_325290	hypothetical protein	0.071354467	0.230175633	0.158821167	0.040125469
3h	ACA1_230490/clean/scf7180000084761:359071-359371:+	ACA1_230490	RFX-type winged-helix domain-containing protein [Source:UniProtKB/TrEMBL;Acc:L8HAD4]	0.0242759	0.1829036	0.1586277	0.002735745
3h	ACA1_064550/clean/scf7180000084599:1640354-1640419:-	ACA1_064550	60S ribosomal export protein NMD3 [Source:UniProtKB/TrEMBL;Acc:L8GYR2]	0.001596593	0.1600914	0.158494807	1.09E-05
3h	ACA1_009930/clean/scf7180000083468:11298-11383:+	ACA1_009930	Acyl-CoA_dh_1 domain-containing protein [Source:UniProtKB/TrEMBL;Acc:L8GFW8]	0	0.158135	0.158135	7.31E-07
3h	ACA1_176050/clean/scf7180000084741:1113531-1113598:-	ACA1_176050	Poly [ADP-ribose] polymerase [Source:UniProtKB/TrEMBL;Acc:L8HI82]	0.006257857	0.164347267	0.15808941	2.50E-05
3h	ACA1_043250/clean/scf7180000084573:145712-145808:+	ACA1_043250	Ubiquitin-like domain-containing protein [Source:UniProtKB/TrEMBL;Acc:L8GUP8]	0.016137167	0.174207367	0.1580702	0.001522078
3h	ACA1_040840/clean/scf7180000084572:92233-92311:-	ACA1_040840	BRO1 domain-containing protein [Source:UniProtKB/TrEMBL;Acc:L8H1X9]	0	0.157886033	0.157886033	3.19E-07
3h	ACA1_271500/clean/scf7180000084771:44731-44891:+	ACA1_271500	Leucine rich repeat domain containing protein [Source:UniProtKB/TrEMBL;Acc:L8GNI4]	0.009370053	0.167249267	0.157879213	0.002756907
3h	ACA1_140730/clean/scf7180000084707:108224-108333:-	ACA1_140730	Kelch repeat protein [Source:UniProtKB/TrEMBL;Acc:L8GGS1]	0.085615233	0.243442333	0.1578271	0.029267007
3h	ACA1_033610/clean/scf7180000084559:57690-57820:+	ACA1_033610	Sortilin [Source:UniProtKB/TrEMBL;Acc:L8GHJ0]	0.00043802	0.158104833	0.157666813	2.26E-06
3h	ACA1_050200/clean/scf7180000084589:53420-53518:+	ACA1_050200	Mammalian PTEN tumor suppressorlike, putative [Source:UniProtKB/TrEMBL;Acc:L8H3Q5]	0	0.157443133	0.157443133	0.004548798
3h	ACA1_366310/clean/scf7180000084641:389932-390050:+	ACA1_366310	Reticulon-like protein [Source:UniProtKB/TrEMBL;Acc:L8GMG7]	0.007685593	0.165103233	0.15741764	9.14E-08
3h	ACA1_285540/clean/scf7180000084776:994031-994108:-	ACA1_285540	GPS domain-containing protein [Source:UniProtKB/TrEMBL;Acc:L8H7T4]	0.009690463	0.166898767	0.157208303	0.004632305
3h	ACA1_339700/clean/scf7180000084619:9930-10003:+	ACA1_339700	Serine/threonine protein kinase, putative [Source:UniProtKB/TrEMBL;Acc:L8GJN7]	0	0.157192133	0.157192133	0.000560325
3h	ACA1_092600/clean/scf7180000084645:364257-364331:-	ACA1_092600	NLPC_P60 domain-containing protein [Source:UniProtKB/TrEMBL;Acc:L8GIC1]	0.003868233	0.160913867	0.157045633	3.56E-05
3h	ACA1_111990/clean/scf7180000084669:78900-79046:+	ACA1_111990	hypothetical protein	0.018469833	0.175500367	0.157030533	0.001085697
3h	ACA1_075010/clean/scf7180000084608:126803-126875:-	ACA1_075010	Ras family protein [Source:UniProtKB/TrEMBL;Acc:L8HGU8]	0.01696618	0.1739838	0.15701762	0.000531531
3h	ACA1_174170/clean/scf7180000084741:632679-632834:-	ACA1_174170	Glutathione Stransferase, C-terminal domain containing protein [Source:UniProtKB/TrEMBL;Acc:L8HHR9]	0.00223581	0.159113167	0.156877357	7.16E-11
3h	ACA1_098410/clean/scf7180000084646:4754-4846:+	ACA1_098410	MFS domain-containing protein [Source:UniProtKB/TrEMBL;Acc:L8H7P3]	0	0.156863533	0.156863533	3.32E-05
3h	ACA1_369850/clean/scf7180000084664:394993-395105:-	ACA1_369850	ATPase with chaperone activity, putative [Source:UniProtKB/TrEMBL;Acc:L8GZ67]	0.002347417	0.1591696	0.156822183	0.000202844
3h	ACA1_209890/clean/scf7180000084757:348704-348791:+	ACA1_209890	ROCO family protein kinase, putative [Source:UniProtKB/TrEMBL;Acc:L8GYH9]	0.064349233	0.22114	0.156790767	0.007169067
3h	ACA1_365390/clean/scf7180000084641:269781-269887:+	ACA1_365390	Ubiquitin domain containing protein [Source:UniProtKB/TrEMBL;Acc:L8GPA8]	0.007200737	0.163845067	0.15664433	1.46E-05
3h	ACA1_026980/clean/scf7180000084464:7515-7601:+	ACA1_026980	hypothetical protein	0.015506043	0.1721391	0.156633057	0.000850212
3h	ACA1_031850/clean/scf7180000084523:20561-20619:-	ACA1_031850	Proteintyrosine phosphatase [Source:UniProtKB/TrEMBL;Acc:L8H408]	0.051508833	0.208127333	0.1566185	0.013845952
3h	ACA1_065290/clean/scf7180000084599:1842940-1843006:-	ACA1_065290	GATA-type domain-containing protein [Source:UniProtKB/TrEMBL;Acc:L8GXM6]	0.001884543	0.158487667	0.156603123	2.17E-05
3h	ACA1_054460/clean/scf7180000084594:499007-499088:-	ACA1_054460	Cytochrome b5 heme-binding domain-containing protein [Source:UniProtKB/TrEMBL;Acc:L8H6D5]	0.01419532	0.1707692	0.15657388	7.11E-06
3h	ACA1_257950/clean/scf7180000084769:185291-185394:-	ACA1_257950	hypothetical protein	0.0459872	0.202546033	0.156558833	0.030961499
3h	ACA1_290250/clean/scf7180000084777:569554-569648:+	ACA1_290250	hypothetical protein	0.003611233	0.160161533	0.1565503	0.002647045
3h	ACA1_335940/clean/scf7180000084593:8321-8409:-	ACA1_335940	RNA recognition motif domain containing protein [Source:UniProtKB/TrEMBL;Acc:L8H3Y6]	0.01536491	0.171754833	0.156389923	0.002152593
3h	ACA1_391200/clean/scf7180000084740:147082-147294:-	ACA1_391200	LmbE family protein [Source:UniProtKB/TrEMBL;Acc:L8GPC9]	0.009340763	0.165712667	0.156371903	2.69E-06
3h	ACA1_012020/clean/scf7180000083652:4352-4455:-	ACA1_012020	hypothetical protein	0.003229082	0.159584433	0.156355351	3.10E-05
3h	ACA1_009930/clean/scf7180000083468:12586-12688:+	ACA1_009930	Acyl-CoA_dh_1 domain-containing protein [Source:UniProtKB/TrEMBL;Acc:L8GFW8]	0.000471103	0.1567705	0.156299397	2.33E-06
3h	ACA1_066780/clean/scf7180000084600:43110-43194:-	ACA1_066780	Transcription initiation factor tfiid subunit 13, putative [Source:UniProtKB/TrEMBL;Acc:L8GP29]	0.012712457	0.168938333	0.156225877	2.05E-05
3h	ACA1_185040/clean/scf7180000084746:75933-76068:-	ACA1_185040	hypothetical protein	0.013758047	0.1697608	0.156002753	0.002679314
3h	ACA1_155670/clean/scf7180000084722:211866-211977:+	ACA1_155670	SPRY domain containing protein [Source:UniProtKB/TrEMBL;Acc:L8H063]	0.01727174	0.173248333	0.155976593	0.000755861
3h	ACA1_071580/clean/scf7180000084603:998186-998269:-	ACA1_071580	GOLD domain-containing protein [Source:UniProtKB/TrEMBL;Acc:L8HGH0]	0	0.155821	0.155821	1.01E-05
3h	ACA1_187480/clean/scf7180000084747:73908-74027:+	ACA1_187480	hypothetical protein	0.001491313	0.157048067	0.155556753	9.75E-11
3h	ACA1_274580/clean/scf7180000084773:117107-117271:-	ACA1_274580	Carrier superfamily protein [Source:UniProtKB/TrEMBL;Acc:L8GGL3]	0.010718957	0.1662723	0.155553343	7.01E-06
3h	ACA1_217750/clean/scf7180000084758:601322-601401:+	ACA1_217750	Phosphoenolpyruvate carboxykinase (GTP), putative [Source:UniProtKB/TrEMBL;Acc:L8GQL5]	0.00864193	0.164130033	0.155488103	0.000319255
3h	ACA1_063500/clean/scf7180000084599:1433323-1433415:+	ACA1_063500	U-box domain-containing protein [Source:UniProtKB/TrEMBL;Acc:L8GZS8]	0.001349663	0.156792233	0.15544257	4.33E-05
3h	ACA1_216320/clean/scf7180000084758:468730-468892:+	ACA1_216320	Homogentisate 1,2dioxygenase [Source:UniProtKB/TrEMBL;Acc:L8GQ83]	0.017803767	0.173164667	0.1553609	2.03E-05
3h	ACA1_396550/clean/scf7180000084759:89756-89852:-	ACA1_396550	hypothetical protein	0.0119937	0.167207167	0.155213467	0.000228826
3h	ACA1_112460/clean/scf7180000084669:158341-158404:-	ACA1_112460	Protective antigen 4D8, putative [Source:UniProtKB/TrEMBL;Acc:L8H3Z9]	0.008518333	0.1636484	0.155130067	2.05E-06
3h	ACA1_015330/clean/scf7180000083794:7098-7157:-	ACA1_015330	CBS domain containing protein [Source:UniProtKB/TrEMBL;Acc:L8GGC6]	0.000494537	0.155583567	0.15508903	1.44E-05
3h	ACA1_028150/clean/scf7180000084472:9069-9156:-	ACA1_028150	hypothetical protein	0	0.155088267	0.155088267	5.83E-07
3h	ACA1_383470/clean/scf7180000084721:177108-177206:+	ACA1_383470	ANK_REP_REGION domain-containing protein [Source:UniProtKB/TrEMBL;Acc:L8GXK5]	0.00010446	0.155049733	0.154945273	7.89E-06

3h	ACA1_383560/clean/scf7180000084721:210250-210320:-	ACA1_383560	TBC domain containing protein [Source:UniProtKB/TrEMBL;Acc:L8GW44]	0	0.154937933	0.154937933	0.000529588
3h	ACA1_144350/clean/scf7180000084709:212889-212979:+	ACA1_144350	CN hydrolase domain-containing protein [Source:UniProtKB/TrEMBL;Acc:L8HEV4]	0.046420567	0.2013226	0.154902033	0.008682486
3h	ACA1_197300/clean/scf7180000084753:1069-1153:-	ACA1_197300	hypothetical protein	0.029761467	0.184517033	0.154755567	0.020658401
3h	ACA1_189570/clean/scf7180000084748:47921-48042:-	ACA1_189570	Zinc finger (CCCHtype) family protein [Source:UniProtKB/TrEMBL;Acc:L8GGG5]	0.005794017	0.160288167	0.15449415	1.18E-05
3h	ACA1_218620/clean/scf7180000084758:678676-678777:-	ACA1_218620	Carboxylic ester hydrolase [Source:UniProtKB/TrEMBL;Acc:L8GRD3]	0.002713927	0.157194533	0.154480607	0.000128371
3h	ACA1_012990/clean/scf7180000083742:19015-19072:+	ACA1_012990	ATP-dependent DNA helicase [Source:UniProtKB/TrEMBL;Acc:L8H692]	0	0.1544143	0.1544143	0.002713547
3h	ACA1_174560/clean/scf7180000084741:738363-738441:-	ACA1_174560	RRM domain-containing protein [Source:UniProtKB/TrEMBL;Acc:L8HHX6]	0.014226633	0.168559033	0.1543324	0.004200664
3h	ACA1_223300/clean/scf7180000084760:122859-122996:+	ACA1_223300	Coatomer subunit beta' [Source:UniProtKB/TrEMBL;Acc:L8GSX3]	0.000410693	0.154740433	0.15432974	1.04E-07
3h	ACA1_050350/clean/scf7180000084589:74120-74183:-	ACA1_050350	MFS domain-containing protein [Source:UniProtKB/TrEMBL;Acc:L8H3R1]	0.002381767	0.156566967	0.1541852	2.01E-06
3h	ACA1_181830/clean/scf7180000084745:52678-52778:-	ACA1_181830	PIPK domain-containing protein [Source:UniProtKB/TrEMBL;Acc:L8H972]	0.019625397	0.173716733	0.154091337	0.03083885
3h	ACA1_278070/clean/scf7180000084776:167671-167787:-	ACA1_278070	RBR-type E3 ubiquitin transferase [Source:UniProtKB/TrEMBL;Acc:L8H8S7]	0.000469797	0.154557633	0.154087837	1.08E-05
3h	ACA1_198570/clean/scf7180000084753:146488-146600:+	ACA1_198570	PCI domain-containing protein [Source:UniProtKB/TrEMBL;Acc:L8H5C2]	0.004344807	0.158364967	0.15402016	1.91E-05
3h	ACA1_259500/clean/scf7180000084769:357822-357913:-	ACA1_259500	hypothetical protein	0.0823464	0.236355333	0.154008933	0.044340388
3h	ACA1_265970/clean/scf7180000084770:560679-560759:+	ACA1_265970	SAM domain (Sterile alpha motif) domain containing protein [Source:UniProtKB/TrEMBL;Acc:L8H2A8]	0.022180967	0.176109433	0.153928467	0.004711264
3h	ACA1_389100/clean/scf7180000084733:196746-197030:+	ACA1_389100	DiacylglycerylN,N,N-trimethylhomoserine synthesis protein [Source:UniProtKB/TrEMBL;Acc:L8GEC4]	0.006698777	0.160524067	0.15382529	8.86E-09
3h	ACA1_251350/clean/scf7180000084768:61544-61626:-	ACA1_251350	Phosphomannomutase [Source:UniProtKB/TrEMBL;Acc:L8H9R8]	0.00209525	0.155686667	0.153591417	3.46E-07
3h	ACA1_108250/clean/scf7180000084661:21715-21784:+	ACA1_108250	hypothetical protein	0.017165153	0.170642667	0.153477513	0.001320386
3h	ACA1_152940/clean/scf7180000084720:39602-39700:+	ACA1_152940	hypothetical protein	0.001424806	0.154858633	0.153433827	2.05E-08
3h	ACA1_279800/clean/scf7180000084776:451198-451263:+	ACA1_279800	Peroxisomal biogenesis factor 11c, putative	0.000581077	0.1539319	0.153350823	9.36E-07
3h	ACA1_263890/clean/scf7180000084770:126878-126974:-	ACA1_263890	Methyltransf_11 domain-containing protein [Source:UniProtKB/TrEMBL;Acc:L8H151]	0.001799496	0.1549709	0.153171404	0.000174785
3h	ACA1_074080/clean/scf7180000084605:18299-18377:-	ACA1_074080	hypothetical protein	0.0070582	0.1601171	0.1530589	0.000390991
3h	ACA1_151330/clean/scf7180000084716:106580-106705:-	ACA1_151330	Purple acid phosphatase [Source:UniProtKB/TrEMBL;Acc:L8H0Q3]	0.001335339	0.154390567	0.153055227	1.06E-05
3h	ACA1_261440/clean/scf7180000084769:679608-679681:-	ACA1_261440	Poly [ADP-ribose] polymerase [Source:UniProtKB/TrEMBL;Acc:L8GFU2]	0.026284933	0.179339867	0.153054933	0.001377714
3h	ACA1_025540/clean/scf7180000084448:8199-8287:+	ACA1_025540	hypothetical protein	0.000973947	0.154008333	0.153034387	4.97E-05
3h	ACA1_267000/clean/scf7180000084770:773895-773984:+	ACA1_267000	Camp-dependent protein kinase catalytic subunit family protein [Source:UniProtKB/TrEMBL;Acc:L8H3Y8]	0.005473657	0.1585076	0.153033943	0.000262497
3h	ACA1_396740/clean/scf7180000084759:112032-112142:-	ACA1_396740	hypothetical protein	0.011834687	0.164842267	0.15300758	0.000183952
3h	ACA1_199000/clean/scf7180000084753:189196-189327:-	ACA1_199000	hypothetical protein	0.001679393	0.154583867	0.152904473	2.23E-05
3h	ACA1_267250/clean/scf7180000084770:838048-838152:+	ACA1_267250	Oxidoreductase, short chain dehydrogenase/reductase superfamily protein [Source:UniProtKB/TrEMBL;Acc:L8H4S7]	0.004930747	0.157831367	0.15290062	0.000173749
3h	ACA1_296110/clean/scf7180000084777:1298924-1299009:-	ACA1_296110	MORN repeat variantcontaining protein [Source:UniProtKB/TrEMBL;Acc:L8HLH8]	0.000393092	0.153233033	0.152839941	8.68E-12
3h	ACA1_264380/clean/scf7180000084770:255225-255321:-	ACA1_264380	S-formylglutathione hydrolase [Source:UniProtKB/TrEMBL;Acc:L8H1Z7]	0.008176213	0.1609034	0.152727187	1.91E-06
3h	ACA1_025410/clean/scf7180000084446:13378-13476:+	ACA1_025410	Serine/threonine protein kinase [Source:UniProtKB/TrEMBL;Acc:L8HGY2]	0.004142493	0.156701567	0.152559073	0.00037392
3h	ACA1_054820/clean/scf7180000084594:580061-580183:-	ACA1_054820	Tetratricopeptide repeat domain containing protein [Source:UniProtKB/TrEMBL;Acc:L8H8E7]	0.010040167	0.1624655	0.152425333	0.004031579
3h	ACA1_035260/clean/scf7180000084564:8219-8434:-	ACA1_035260	Guanine nucleotidebinding protein G(O), alpha subunit, putative [Source:UniProtKB/TrEMBL;Acc:L8HCU4]	0.00143099	0.153751	0.15232001	1.47E-06
3h	ACA1_071970/clean/scf7180000084603:1120154-1120210:+	ACA1_071970	Carn_acyltransf domain-containing protein [Source:UniProtKB/TrEMBL;Acc:L8HEI6]	0.007873923	0.160090833	0.15221691	9.58E-05
3h	ACA1_223790/clean/scf7180000084760:148719-148811:-	ACA1_223790	Saccharopine dehydrogenase (NAD+, Llysine-forming), putative protein	0.00218006	0.154261667	0.152081607	3.32E-06
3h	ACA1_071580/clean/scf7180000084603:996942-997035:-	ACA1_071580	GOLD domain-containing protein [Source:UniProtKB/TrEMBL;Acc:L8HGH0]	0.011241967	0.163312667	0.1520707	0.000411848
3h	ACA1_062680/clean/scf7180000084599:1271604-1271745:+	ACA1_062680	hypothetical protein	0.001911357	0.1539355	0.152024143	2.97E-05
3h	ACA1_054100/clean/scf7180000084594:403192-403269:+	ACA1_054100	Diacylglycerol kinase [Source:UniProtKB/TrEMBL;Acc:L8H6A6]	0	0.151982767	0.151982767	0.000232857
3h	ACA1_385700/clean/scf7180000084730:242117-242204:+	ACA1_385700	F-box domain-containing protein [Source:UniProtKB/TrEMBL;Acc:L8HAP4]	0.001986097	0.1537874	0.151801303	0.025349724
3h	ACA1_244080/clean/scf7180000084763:371800-371896:+	ACA1_244080	PCI domain-containing protein [Source:UniProtKB/TrEMBL;Acc:L8GL06]	0.005516023	0.157244767	0.151728743	8.27E-07
3h	ACA1_276830/clean/scf7180000084774:226921-227001:-	ACA1_276830	Peptidyl-prolyl cis-trans isomerase [Source:UniProtKB/TrEMBL;Acc:L8GR77]	0.00178499	0.153507867	0.151722877	5.07E-09
3h	ACA1_172880/clean/scf7180000084741:348621-348744:+	ACA1_172880	Protein kinase domain-containing protein [Source:UniProtKB/TrEMBL;Acc:L8HJV9]	0.045460167	0.197148	0.151687833	0.018602776
3h	ACA1_133480/clean/scf7180000084701:64483-64564:+	ACA1_133480	hypothetical protein	0.005161887	0.156836233	0.151674347	7.88E-05
3h	ACA1_116740/clean/scf7180000084669:840166-840254:+	ACA1_116740	Methyltransf_2 domain-containing protein [Source:UniProtKB/TrEMBL;Acc:L8H4R1]	0.043497267	0.195002533	0.151505267	0.017172593
3h	ACA1_313860/clean/scf7180000083835:1919-1998:-	ACA1_313860	STAS domain-containing protein [Source:UniProtKB/TrEMBL;Acc:L8H2N6]	0.025699733	0.177168	0.151468267	0.000695965
3h	ACA1_171860/clean/scf7180000084741:180478-180569:+	ACA1_171860	POT family protein [Source:UniProtKB/TrEMBL;Acc:L8HHD5]	0.0270359	0.178187967	0.151152067	0.002660541
3h	ACA1_400550/clean/scf7180000084775:188762-188839:-	ACA1_400550	SAM-dependent MTase TRM10-type domain-containing protein [Source:UniProtKB/TrEMBL;Acc:L8GIH1]	0.00218969	0.153119433	0.150929743	0.001006792
3h	ACA1_146440/clean/scf7180000084713:26238-26307:+	ACA1_146440	Methyltransf_11 domain-containing protein [Source:UniProtKB/TrEMBL;Acc:L8GU12]	0.006276113	0.157193433	0.15091732	6.54E-05
3h	ACA1_103860/clean/scf7180000084653:53957-54016:-	ACA1_103860	Kinase [Source:UniProtKB/TrEMBL;Acc:L8GE29]	0	0.150823533	0.150823533	6.34E-05
3h	ACA1_095620/clean/scf7180000084645:858228-858336:-	ACA1_095620	Mediator of RNA polymerase II transcription subunit 13 [Source:UniProtKB/TrEMBL;Acc:L8GIT2]	0.00595314	0.156592033	0.150638893	0.00240861
3h	ACA1_053670/clean/scf7180000084594:313216-313411:+	ACA1_053670	CYP, putative [Source:UniProtKB/TrEMBL;Acc:L8H7F1]	0.043798333	0.1941038	0.150305467	0.021450737
3h	ACA1_096350/clean/scf7180000084645:992199-992274:+	ACA1_096350	Cystatin domain-containing protein [Source:UniProtKB/TrEMBL;Acc:L8GJE3]	0.002907187	0.153185167	0.150277979	4.91E-05
3h	ACA1_142350/clean/scf7180000084708:143761-143869:+	ACA1_142350	Calreticulin [Source:UniProtKB/TrEMBL;Acc:L8HAK1]	0.000519579	0.1507901	0.150270521	7.50E-16
3h	ACA1_135960/clean/scf7180000084702:122305-122410:-	ACA1_135960	V-ATPase_H_C domain-containing protein [Source:UniProtKB/TrEMBL;Acc:L8GGR7]	0.00027346	0.150298467	0.150025007	2.81E-08
3h	ACA1_320840/clean/scf7180000084416:36294-36598:+	ACA1_320840	HSP91 (Heat shock protein 91), putative [Source:UniProtKB/TrEMBL;Acc:L8GTS5]	0.00490931	0.154845467	0.149936157	2.96E-10
3h	ACA1_235670/clean/scf7180000084762:351708-351841:-	ACA1_235670	Glyco_transf_64 domain-containing protein [Source:UniProtKB/TrEMBL;Acc:L8H0L2]	0.010247756	0.160084567	0.149836811	0.003607493
3h	ACA1_187740/clean/scf7180000084747:87201-87305:-	ACA1_187740	hypothetical protein	0.059480733	0.209081333	0.1496006	0.014696104
3h	ACA1_173790/clean/scf7180000084741:520856-520944:+	ACA1_173790	Nicotinamide-nucleotide adenylyltransferase [Source:UniProtKB/TrEMBL;Acc:L8HK06]	0.00474528	0.154317233	0.149571953	9.44E-05
3h	ACA1_384690/clean/scf7180000084730:85209-85300:-	ACA1_384690	Alpha-1,4 glucan phosphorylase [Source:UniProtKB/TrEMBL;Acc:L8H8A5]	9.09E-05	0.149624333	0.149533414	2.19E-09
3h	ACA1_063300/clean/scf7180000084599:1374006-1374087:-	ACA1_063300	Transporter, small conductance mechanosensitive ion channel (MscS) family protein [Source:UniProtKB/TrEMBL;Acc:L8GZP0]	0.035347333	0.184870433	0.1495231	0.013322332
3h	ACA1_117320/clean/scf7180000084669:975697-975773:-	ACA1_117320	FAD binding domain containing protein [Source:UniProtKB/TrEMBL;Acc:L8H415]	0.00338729	0.1528679	0.14948061	0.000169339
3h	ACA1_362650/clean/scf7180000084624:122973-123093:+	ACA1_362650	G-patch domain-containing protein [Source:UniProtKB/TrEMBL;Acc:L8GFY2]	0.843361	0.992681333	0.149320333	0.000695997
3h	ACA1_273500/clean/scf7180000084773:30646-30763:+	ACA1_273500	CBS domain containing protein [Source:UniProtKB/TrEMBL;Acc:L8GG64]	0.00492235	0.154138	0.14921565	1.14E-06
3h	ACA1_074520/clean/scf7180000084608:29376-29454:+	ACA1_074520	SH3 domain containing protein [Source:UniProtKB/TrEMBL;Acc:L8HFD8]	0.002380953	0.1515212	0.149140247	1.91E-05
3h	ACA1_182910/clean/scf7180000084745:289508-289612:+	ACA1_182910	RRM domain-containing protein [Source:UniProtKB/TrEMBL;Acc:L8H7Y4]	0.000372945	0.149485833	0.149112889	9.36E-10
3h	ACA1_234000/clean/scf7180000084762:181714-181804:+	ACA1_234000	Purple acid phosphatase [Source:UniProtKB/TrEMBL;Acc:L8H162]	0	0.148837767	0.148837767	5.53E-09
3h	ACA1_158450/clean/scf7180000084724:220288-220414:-	ACA1_158450	UBIQUITIN_CONJUGAT_2 domain-containing protein [Source:UniProtKB/TrEMBL;Acc:L8HCH6]	0.003968873	0.1527109	0.148742027	1.71E-07
3h	ACA1_293730/clean/scf7180000084777:969082-969174:+	ACA1_293730	PB1 domain containing protein [Source:UniProtKB/TrEMBL;Acc:L8HJM7]	0.020315433	0.1689892	0.148673767	0.000150797
3h	ACA1_177500/clean/scf7180000084743:83279-83360:+	ACA1_177500	hypothetical protein	0	0.148646033	0.148646033	5.56E-07
3h	ACA1_098930/clean/scf7180000084646:67801-67867:-	ACA1_098930	ABC transporter domain-containing protein [Source:UniProtKB/TrEMBL;Acc:L8H7R6]	0.016014943	0.164654933	0.14863999	0.002507167
3h	ACA1_271290/clean/scf7180000084771:11827-11963:-	ACA1_271290	hypothetical protein	0.002824833	0.151368467	0.148543633	1.23E-06
3h	ACA1_359710/clean/scf7180000084622:6566-6716:-	ACA1_359710	WD_REPEATS_REGION domain-containing protein [Source:UniProtKB/TrEMBL;Acc:L8HEZ3]	0.002571713	0.151064033	0.148492321	4.49E-07
3h	ACA1_024250/clean/scf7180000084441:25613-25678:-	ACA1_024250	hypothetical protein	0.733067	0.881552	0.148485	0.037353737
3h	ACA1_268910/clean/scf7180000084770:938629-938749:-	ACA1_268910	Elongation factor 1-gamma family protein [Source:UniProtKB/TrEMBL;Acc:L8H2Q1]	0.000359086	0.1488404	0.148481314	9.94E-15
3h	ACA1_164710/clean/scf7180000084729:257838-257991:-	ACA1_164710	Ribosomal_L23eN domain-containing protein [Source:UniProtKB/TrEMBL;Acc:L8GRL5]	0.002059087	0.150399067	0.14833998	2.61E-12
3h	ACA1_362770/clean/scf7180000084624:170910-171002:-	ACA1_362770	PH domain containing protein [Source:UniProtKB/TrEMBL;Acc:L8GFC9]	0.011879467	0.1601112	0.148231733	0.000115857
3h	ACA1_062230/clean/scf7180000084599:1143503-1143575:-	ACA1_062230	C2 domain-containing protein [Source:UniProtKB/TrEMBL;Acc:L8GZE6]	0.0101066	0.1583378	0.1482312	0.000687253
3h	ACA1_057240/clean/scf7180000084599:93987-94041:-	ACA1_057240	unspecified product	0	0.147976167	0.147976167	0.000191087
3h	ACA1_033610/clean/scf7180000084559:60232-60294:+	ACA1_033610	Sortilin [Source:UniProtKB/TrEMBL;Acc:L8GHJ0]	0.004108833	0.151825933	0.1477171	1.44E-06
3h	ACA1_100440/clean/scf7180000084648:77556-77629:-	ACA1_100440	Cytochrome p450 superfamily protein [Source:UniProtKB/TrEMBL;Acc:L8GKG1]	0.045793067	0.193294633	0.147501567	0.025586803
3h	ACA1_395520/clean/scf7180000084756:198347-198471:+	ACA1_395520	hypothetical protein	0.0769029	0.224338433	0.147435533	0.025545633
3h	ACA1_287790/clean/scf7180000084777:80419-80537:-	ACA1_287790	hypothetical protein	0.002252697	0.149612333	0.147359637	3.68E-05
3h	ACA1_144440/clean/scf7180000084709:243553-243628:+	ACA1_144440	SNF2 family Nterminal domain containing protein [Source:UniProtKB/TrEMBL;Acc:L8HFN1]	0.006269297	0.153545733	0.147276437	6.67E-09
3h	ACA1_264070/clean/scf7180000084770:173572-173662:+	ACA1_264070	COPI_C domain-containing protein [Source:UniProtKB/TrEMBL;Acc:L8H460]	0.008645233	0.155901333	0.1472561	0.000705284
3h	ACA1_063300/clean/scf7180000084599:1374215-1374295:-	ACA1_063300	Transporter, small conductance mechanosensitive ion channel (MscS) family protein [Source:UniProtKB/TrEMBL;Acc:L8GZP0]	0.015184567	0.1623908	0.147206233	0.005532264
3h	ACA1_199600/clean/scf7180000084753:272451-272561:+	ACA1_199600	PH domain-containing protein [Source:UniProtKB/TrEMBL;Acc:L8H366]	0.003622045	0.150708067	0.147086022	0.000206771
3h	ACA1_234340/clean/scf7180000084762:279358-279539:+	ACA1_234340	Ubiquitin carboxylterminal hydrolase domain containing protein [Source:UniProtKB/TrEMBL;Acc:L8H3J5]	0.002786308	0.149643167	0.146856859	8.02E-07
3h	ACA1_006150/clean/scf7180000083029:58853-58933:+	ACA1_006150	PH domain-containing protein [Source:UniProtKB/TrEMBL;Acc:L8HJA0]	0.005665443	0.152498767	0.146833323	6.01E-07
3h	ACA1_368080/clean/scf7180000084664:48424-48546:-	ACA1_368080	hypothetical protein	0.0053248	0.151979	0.1466542	0.01113389
3h	ACA1_054710/clean/scf7180000084594:559211-559295:-	ACA1_054710	Ras family protein [Source:UniProtKB/TrEMBL;Acc:L8H6F4]	0.003088077	0.1495193	0.146431223	1.05E-05
3h	ACA1_392150/clean/scf7180000084740:261633-261718:-	ACA1_392150	AAA domain-containing protein [Source:UniProtKB/TrEMBL;Acc:L8GQ70]	0.003617096	0.149968467	0.146351371	3.49E-05
3h	ACA1_293890/clean/scf7180000084777:985237-985437:+	ACA1_293890	hypothetical protein	0.003343363	0.1495868	0.146243437	5.29E-05
3h	ACA1_285220/clean/scf7180000084776:963136-963222:+	ACA1_285220	Protein prenyltransferase alpha subunit repeat-containing protein [Source:UniProtKB/TrEMBL;Acc:L8H8X1]	0.007229483	0.153451333	0.14622185	0.001229214
3h	ACA1_183740/clean/scf7180000084745:494187-494265:+	ACA1_183740	hypothetical protein	0.021825367	0.168032833	0.146207467	0.011758741
3h	ACA1_376170/clean/scf7180000084685:40723-40808:-	ACA1_376170	Aa_trans domain-containing protein [Source:UniProtKB/TrEMBL;Acc:L8HFY8]	0.0093378	0.155531167	0.146193367	0.002970646
3h	ACA1_323310/clean/scf7180000084468:1842-1927:+	ACA1_323310	hypothetical protein	0.0298358	0.176001833	0.146166033	0.018420065
3h	ACA1_395220/clean/scf7180000084756:157505-157584:+	ACA1_395220	Eukaryotic initiation factor 4E [Source:UniProtKB/TrEMBL;Acc:L8H0E9]	0.003504043	0.149600467	0.146096423	8.01E-05
3h	ACA1_261600/clean/scf7180000084769:721167-721254:-	ACA1_261600	RING-type domain-containing protein [Source:UniProtKB/TrEMBL;Acc:L8GG78]	0.007154507	0.153239533	0.146085027	0.000329314
3h	ACA1_089650/clean/scf7180000084639:375350-375445:+	ACA1_089650	NACHT domain containing protein [Source:UniProtKB/TrEMBL;Acc:L8GUT6]	0.000508517	0.146589267	0.14608075	0.00010985
3h	ACA1_323520/clean/scf7180000084468:40480-40593:-	ACA1_323520	Inosineuridine preferring nucleoside hydrolase [Source:UniProtKB/TrEMBL;Acc:L8H334]	0.0391377	0.185060767	0.145923067	0.013943394
3h	ACA1_265400/clean/scf7180000084770:461892-461985:+	ACA1_265400	Vacuolar protein sorting-associated protein 35 [Source:UniProtKB/TrEMBL;Acc:L8H265]	0.006632987	0.152486967	0.14585398	2.54E-05
3h	ACA1_277460/clean/scf7180000084776:49191-49257:+	ACA1_277460	Ipi1_N domain-containing protein [Source:UniProtKB/TrEMBL;Acc:L8H6F9]	0.02902581	0.174809867	0.145784057	0.00665993
3h	ACA1_119220/clean/scf7180000084672:44660-44855:+	ACA1_119220	Peptidylprolyl isomerase [Source:UniProtKB/TrEMBL;Acc:L8HKC1]	0.027123167	0.172857667	0.1457345	0.000899211
3h	ACA1_320950/clean/scf7180000084435:978-1051:-	ACA1_320950	SET domain containing protein [Source:UniProtKB/TrEMBL;Acc:L8GPN6]	0	0.145657533	0.145657533	0.000628354
3h	ACA1_175510/clean/scf7180000084741:973424-973513:+	ACA1_175510	Xylose isomerase [Source:UniProtKB/TrEMBL;Acc:L8HKE1]	0.00902499	0.154668933	0.145643943	4.56E-05
3h	ACA1_368780/clean/scf7180000084664:160237-160296:-	ACA1_368780	CMP/dCMP-type deaminase domain-containing protein [Source:UniProtKB/TrEMBL;Acc:L8H1B4]	0.0070922	0.152644433	0.145552233	0.023151026
3h	ACA1_042210/clean/scf7180000084573:59645-59759:+	ACA1_042210	Signal transducer and activator of transcription [Source:UniProtKB/TrEMBL;Acc:L8GXQ7]	0.0282306	0.173567	0.1453364	0.002764643
3h	ACA1_181960/clean/scf7180000084745:90943-91008:+	ACA1_181960	WD_REPEATS_REGION domain-containing protein [Source:UniProtKB/TrEMBL;Acc:L8H808]	0.006851457	0.1520798	0.145228343	4.72E-05
3h	ACA1_097200/clean/scf7180000084645:1183305-1183407:+	ACA1_097200	MIF4G domain-containing protein [Source:UniProtKB/TrEMBL;Acc:L8GLG0]	0.0219966	0.1670087	0.1450121	0.03506891
3h	ACA1_107440/clean/scf7180000084660:198191-198292:+	ACA1_107440	Purple acid phosphatase [Source:UniProtKB/TrEMBL;Acc:L8GNJ7]	0.005357543	0.150214667	0.144857123	2.43E-06
3h	ACA1_292880/clean/scf7180000084777:923927-924015:-	ACA1_292880	RhoGEF domain containing protein [Source:UniProtKB/TrEMBL;Acc:L8HJL4]	0.0074074	0.152262867	0.144855467	0.003039078
3h	ACA1_038540/clean/scf7180000084568:174837-174897:+	ACA1_038540	Ubiquitinconjugating enzyme subfamily protein [Source:UniProtKB/TrEMBL;Acc:L8GLG9]	0.010598843	0.155352333	0.14475349	0.000336355
3h	ACA1_044940/clean/scf7180000084576:69032-69149:-	ACA1_044940	BTB domain-containing protein [Source:UniProtKB/TrEMBL;Acc:L8GYY6]	0.0012507	0.145980567	0.144729867	6.26E-05
3h	ACA1_042250/clean/scf7180000084573:70694-70775:-	ACA1_042250	Phosphoglucomutase, putative [Source:UniProtKB/TrEMBL;Acc:L8GV41]	0	0.144646933	0.144646933	1.03E-05
3h	ACA1_219480/clean/scf7180000084758:748332-748410:+	ACA1_219480	hypothetical protein	0	0.144485067	0.144485067	4.57E-06
3h	ACA1_067450/clean/scf7180000084601:32883-32963:-	ACA1_067450	hypothetical protein	0.009242827	0.153663133	0.144420307	6.13E-06
3h	ACA1_321440/clean/scf7180000084439:30964-31106:+	ACA1_321440	hypothetical protein	0.002485777	0.1464317	0.143945923	3.05E-05
3h	ACA1_180600/clean/scf7180000084744:62822-62891:+	ACA1_180600	hypothetical protein	0.0065656	0.1504633	0.1438977	5.60E-07
3h	ACA1_074990/clean/scf7180000084608:124727-124811:+	ACA1_074990	Protein kinase domain-containing protein [Source:UniProtKB/TrEMBL;Acc:L8HHK8]	0.024525133	0.168277433	0.1437523	0.01705976
3h	ACA1_191500/clean/scf7180000084749:5039-5167:-	ACA1_191500	Phosphoenolpyruvate carboxykinase (GTP), putative [Source:UniProtKB/TrEMBL;Acc:L8GN92]	0.001635317	0.145246633	0.143611317	1.18E-08
3h	ACA1_002320/clean/scf7180000082924:9599-9663:+	ACA1_002320	Fbox domain containing protein [Source:UniProtKB/TrEMBL;Acc:L8GU55]	0.006917897	0.150527	0.143609103	0.000144288
3h	ACA1_293880/clean/scf7180000084777:983068-983152:-	ACA1_293880	hypothetical protein	0.006996093	0.150550667	0.143554573	9.61E-06
3h	ACA1_111960/clean/scf7180000084669:73541-73644:-	ACA1_111960	Ornithine cyclodeaminase [Source:UniProtKB/TrEMBL;Acc:L8H5A8]	0.042774767	0.186249	0.143474233	0.003461507
3h	ACA1_087950/clean/scf7180000084639:139728-139841:+	ACA1_087950	Thg1 domain-containing protein [Source:UniProtKB/TrEMBL;Acc:L8GUE5]	0.010096763	0.1535307	0.143433937	0.000589323
3h	ACA1_234770/clean/scf7180000084762:314544-314625:-	ACA1_234770	SGF29 C-terminal domain-containing protein [Source:UniProtKB/TrEMBL;Acc:L8H0I6]	0.007113593	0.150521933	0.14340834	0.000176356
3h	ACA1_199970/clean/scf7180000084753:362195-362301:-	ACA1_199970	Glycoside hydrolase family protein [Source:UniProtKB/TrEMBL;Acc:L8H5H5]	0	0.143260967	0.143260967	2.43E-09
3h	ACA1_296250/clean/scf7180000084777:1306762-1306843:+	ACA1_296250	Rasrelated protein Rab-32, putative [Source:UniProtKB/TrEMBL;Acc:L8HJZ6]	0.012870233	0.156047333	0.1431771	0.002674448
3h	ACA1_289860/clean/scf7180000084777:478746-478840:+	ACA1_289860	Raslike protein 1, putative [Source:UniProtKB/TrEMBL;Acc:L8HKN5]	0.005899343	0.149015067	0.143115723	2.38E-05
3h	ACA1_322090/clean/scf7180000084459:3981-4124:-	ACA1_322090	Myosin head (Motor domain) domain containing protein [Source:UniProtKB/TrEMBL;Acc:L8GN06]	0.0061884	0.149216467	0.143028067	5.31E-05
3h	ACA1_382630/clean/scf7180000084721:87972-88096:-	ACA1_382630	LBP / BPI / CETP family, Cterminal domain containing protein [Source:UniProtKB/TrEMBL;Acc:L8GVZ7]	0.00287294	0.1457172	0.14284426	3.94E-06
3h	ACA1_132870/clean/scf7180000084700:90181-90261:+	ACA1_132870	Chromatin modifying protein 1b, putative [Source:UniProtKB/TrEMBL;Acc:L8GY90]	0.006912483	0.149753667	0.142841183	5.39E-05
3h	ACA1_089960/clean/scf7180000084639:404271-404373:-	ACA1_089960	Dual specificity protein kinase [Source:UniProtKB/TrEMBL;Acc:L8GVS6]	0.006535933	0.149338967	0.142803033	0.000381699
3h	ACA1_173150/clean/scf7180000084741:412216-412293:+	ACA1_173150	PH domain-containing protein [Source:UniProtKB/TrEMBL;Acc:L8HHL2]	0.000140085	0.142905733	0.142765648	0.000215223
3h	ACA1_149540/clean/scf7180000084715:177600-177671:+	ACA1_149540	VPS37 C-terminal domain-containing protein [Source:UniProtKB/TrEMBL;Acc:L8HEF0]	0.037355933	0.179989367	0.142633433	0.009877557
3h	ACA1_057800/clean/scf7180000084599:181668-181787:-	ACA1_057800	BZIP domain-containing protein [Source:UniProtKB/TrEMBL;Acc:L8GVB0]	0.00323773	0.145765333	0.142527603	1.72E-05
3h	ACA1_175370/clean/scf7180000084741:940048-940116:-	ACA1_175370	PKD_channel domain-containing protein [Source:UniProtKB/TrEMBL;Acc:L8HI19]	0.0227748	0.1652763	0.1425015	0.004789583
3h	ACA1_280720/clean/scf7180000084776:530591-530718:+	ACA1_280720	Calmodulin, putative [Source:UniProtKB/TrEMBL;Acc:L8H8H2]	0.00377023	0.146175033	0.142404803	3.49E-09
3h	ACA1_385700/clean/scf7180000084730:241441-241526:+	ACA1_385700	F-box domain-containing protein [Source:UniProtKB/TrEMBL;Acc:L8HAP4]	0.0050826	0.147388333	0.142305733	0.031581059

3h	ACA1_073230/clean/scf7180000084603:1376835-1376901:-	ACA1_073230	EHN domain-containing protein [Source:UniProtKB/TrEMBL;Acc:L8HDT9]	0	0.1422832	0.1422832	5.23E-05
3h	ACA1_113860/clean/scf7180000084669:389601-389663:+	ACA1_113860	Saposin B-type domain-containing protein [Source:UniProtKB/TrEMBL;Acc:L8H4D7]	0.000971817	0.14306	0.142088183	3.46E-06
3h	ACA1_027030/clean/scf7180000084464:26193-26269:-	ACA1_027030	C2 domain-containing protein [Source:UniProtKB/TrEMBL;Acc:L8GJ80]	0.039155733	0.181180533	0.1420248	0.016012605
3h	ACA1_169710/clean/scf7180000084739:56576-56666:+	ACA1_169710	SHSP domain-containing protein [Source:UniProtKB/TrEMBL;Acc:L8HHU0]	0.014930333	0.156795333	0.141865	0.000820058
3h	ACA1_064190/clean/scf7180000084599:1552152-1552245:+	ACA1_064190	hypothetical protein	0.000946674	0.1427541	0.141807426	4.65E-10
3h	ACA1_096830/clean/scf7180000084645:1119142-1119250:+	ACA1_096830	Serine/threonine-protein kinase TOR [Source:UniProtKB/TrEMBL;Acc:L8GIV8]	0.0443224	0.185981533	0.141659133	0.01769641
3h	ACA1_145850/clean/scf7180000084710:167677-167763:-	ACA1_145850	O-methyltransferase, putative [Source:UniProtKB/TrEMBL;Acc:L8GFG9]	0.00042014	0.141972667	0.141552527	4.23E-05
3h	ACA1_313860/clean/scf7180000083835:2645-2714:-	ACA1_313860	STAS domain-containing protein [Source:UniProtKB/TrEMBL;Acc:L8H2N6]	0.001928363	0.143089667	0.141161303	1.42E-06
3h	ACA1_290560/clean/scf7180000084777:625280-625362:+	ACA1_290560	Peptidase_M14 domain-containing protein [Source:UniProtKB/TrEMBL;Acc:L8HLJ4]	0	0.141131167	0.141131167	6.26E-08
3h	ACA1_223340/clean/scf7180000084760:134032-134115:-	ACA1_223340	hypothetical protein	0.014910332	0.1560129	0.141102568	0.011948963
3h	ACA1_203710/clean/scf7180000084754:191117-191234:-	ACA1_203710	hypothetical protein	0.00090529	0.1419947	0.14108941	9.68E-17
3h	ACA1_215000/clean/scf7180000084758:290519-290615:-	ACA1_215000	GATA-type domain-containing protein [Source:UniProtKB/TrEMBL;Acc:L8GSI2]	0.005069077	0.146145233	0.141076157	0.000675395
3h	ACA1_035590/clean/scf7180000084564:94723-94852:-	ACA1_035590	ANK_REP_REGION domain-containing protein [Source:UniProtKB/TrEMBL;Acc:L8H9N8]	0.019835383	0.160848167	0.141012783	0.031688934
3h	ACA1_217370/clean/scf7180000084758:580075-580173:+	ACA1_217370	Adaptorrelated protein complex 1, beta 1 subunit, isoform 2, putative [Source:UniProtKB/TrEMBL;Acc:L8GPS7]	0.002244095	0.142870333	0.140626238	8.99E-07
3h	ACA1_061080/clean/scf7180000084599:814805-814888:-	ACA1_061080	OBG-type G domain-containing protein [Source:UniProtKB/TrEMBL;Acc:L8GWJ2]	0.000135219	0.140576133	0.140440914	1.17E-07
3h	ACA1_109780/clean/scf7180000084663:23817-23958:-	ACA1_109780	AAA domain-containing protein [Source:UniProtKB/TrEMBL;Acc:L8HIF9]	0.014524667	0.15493	0.140405333	0.00123534
3h	ACA1_373690/clean/scf7180000084679:141375-141437:+	ACA1_373690	ADPribosylglycohydrolase superfamily protein [Source:UniProtKB/TrEMBL;Acc:L8GGT7]	0.00293255	0.143265167	0.140332617	0.003344379
3h	ACA1_328770/clean/scf7180000084560:25604-25687:-	ACA1_328770	Aldo_ket_red domain-containing protein [Source:UniProtKB/TrEMBL;Acc:L8GQZ7]	0.00445151	0.144539367	0.140087857	1.23E-08
3h	ACA1_169580/clean/scf7180000084739:44417-44529:+	ACA1_169580	hypothetical protein	0.002175245	0.142259833	0.140084588	0.000221592
3h	ACA1_367990/clean/scf7180000084664:33003-33098:+	ACA1_367990	UBIQUITIN_CONJUGAT_2 domain-containing protein [Source:UniProtKB/TrEMBL;Acc:L8GXZ4]	0.01186296	0.151921933	0.140058973	3.48E-05
3h	ACA1_074420/clean/scf7180000084608:6608-6697:-	ACA1_074420	Putative N6adenosine-methyltransferase [Source:UniProtKB/TrEMBL;Acc:L8HFC7]	0.008058277	0.148098433	0.140040157	6.63E-05
3h	ACA1_206060/clean/scf7180000084755:71787-71868:+	ACA1_206060	Ras subfamily protein [Source:UniProtKB/TrEMBL;Acc:L8H5G9]	0.071398933	0.211269333	0.1398704	0.04130286
3h	ACA1_273850/clean/scf7180000084773:68215-68318:+	ACA1_273850	Membrane protein, putative [Source:UniProtKB/TrEMBL;Acc:L8GGJ5]	0.004105613	0.143869633	0.13976402	2.57E-05
3h	ACA1_042250/clean/scf7180000084573:69959-70028:-	ACA1_042250	Phosphoglucomutase, putative [Source:UniProtKB/TrEMBL;Acc:L8GV41]	0	0.1397167	0.1397167	1.56E-06
3h	ACA1_074110/clean/scf7180000084605:27135-27239:-	ACA1_074110	Peptidase [Source:UniProtKB/TrEMBL;Acc:L8HJ70]	0.002918382	0.142519767	0.139601384	3.13E-05
3h	ACA1_370080/clean/scf7180000084664:433014-433102:-	ACA1_370080	ATPdependent RNA helicase Ddx1, putative [Source:UniProtKB/TrEMBL;Acc:L8GZ36]	0	0.1394657	0.1394657	0.000682942
3h	ACA1_185590/clean/scf7180000084746:112555-112687:+	ACA1_185590	EF hand domain containing protein [Source:UniProtKB/TrEMBL;Acc:L8H583]	4.26E-05	0.139327433	0.139284881	6.84E-07
3h	ACA1_287850/clean/scf7180000084777:94593-94721:-	ACA1_287850	RING-type domain-containing protein [Source:UniProtKB/TrEMBL;Acc:L8HHQ7]	0.014342997	0.153596633	0.139253637	0.001772179
3h	ACA1_175510/clean/scf7180000084741:974171-974269:+	ACA1_175510	Xylose isomerase [Source:UniProtKB/TrEMBL;Acc:L8HKE1]	0.001771275	0.140974267	0.139202991	1.25E-05
3h	ACA1_313600/clean/scf7180000083822:9274-9331:-	ACA1_313600	Peptidyl-prolyl cis-trans isomerase [Source:UniProtKB/TrEMBL;Acc:L8GNF0]	0.00168049	0.140730133	0.139049643	4.26E-05
3h	ACA1_000550/clean/scf7180000082784:4094-4199:+	ACA1_000550	Eukaryotic translation initiation factor 4E family [Source:UniProtKB/TrEMBL;Acc:L8GRC5]	0.001319004	0.140367	0.139047996	7.72E-05
3h	ACA1_117260/clean/scf7180000084669:964811-964901:+	ACA1_117260	hypothetical protein	0.038741533	0.177482867	0.138741333	0.046726743
3h	ACA1_164660/clean/scf7180000084729:245671-245740:-	ACA1_164660	Protein tyrosine phosphatase, dual specificity, putative [Source:UniProtKB/TrEMBL;Acc:L8GU25]	0.000790821	0.139482433	0.138691612	1.15E-06
3h	ACA1_043330/clean/scf7180000084573:165280-165357:-	ACA1_043330	hypothetical protein	0	0.138324967	0.138324967	1.78E-06
3h	ACA1_359270/clean/scf7180000084742:69998-70101:-	ACA1_359270	RAB family member (Rab7), putative [Source:UniProtKB/TrEMBL;Acc:L8GLL4]	0.020417033	0.158658	0.138240967	0.000109085
3h	ACA1_033720/clean/scf7180000084559:114329-114430:+	ACA1_033720	hypothetical protein	0.025611767	0.163824667	0.1382129	0.000260291
3h	ACA1_071540/clean/scf7180000084603:985627-985729:+	ACA1_071540	Dual specificity protein kinase [Source:UniProtKB/TrEMBL;Acc:L8HE77]	0.0041408	0.142323033	0.138182233	0.000558365
3h	ACA1_091040/clean/scf7180000084645:46758-46850:-	ACA1_091040	Amidohydro-rel domain-containing protein [Source:UniProtKB/TrEMBL;Acc:L8GIF1]	0.0035461	0.1416575	0.1381114	0.000657531
3h	ACA1_225920/clean/scf7180000084761:21489-21586:-	ACA1_225920	Carboxylic ester hydrolase [Source:UniProtKB/TrEMBL;Acc:L8H8H3]	0.006206473	0.144224133	0.13801766	4.45E-06
3h	ACA1_383450/clean/scf7180000084721:170122-170194:-	ACA1_383450	G protein coupled receptor, putative [Source:UniProtKB/TrEMBL;Acc:L8GUE6]	0.001583577	0.1395542	0.137970623	1.41E-06
3h	ACA1_171880/clean/scf7180000084741:184041-184122:-	ACA1_171880	F-box domain-containing protein [Source:UniProtKB/TrEMBL;Acc:L8HGG2]	0	0.137954367	0.137954367	0.001460998
3h	ACA1_002320/clean/scf7180000082924:9130-9235:+	ACA1_002320	Fbox domain containing protein [Source:UniProtKB/TrEMBL;Acc:L8GU55]	0.006825543	0.144438033	0.13761249	0.000312557
3h	ACA1_074420/clean/scf7180000084608:5114-5207:-	ACA1_074420	Putative N6adenosine-methyltransferase [Source:UniProtKB/TrEMBL;Acc:L8HFC7]	0.001829308	0.139392533	0.137563226	1.45E-05
3h	ACA1_368250/clean/scf7180000084664:89166-89243:-	ACA1_368250	BPI2 domain-containing protein [Source:UniProtKB/TrEMBL;Acc:L8GY12]	0.010469607	0.1479699	0.137500293	0.000350633
3h	ACA1_076960/clean/scf7180000084612:202108-202570:-	ACA1_076960	hypothetical protein	0.0102027	0.147615133	0.137412433	0.000240872
3h	ACA1_382900/clean/scf7180000084721:112026-112110:-	ACA1_382900	ADPribosylation factor, putative [Source:UniProtKB/TrEMBL;Acc:L8GUW5]	0.063113533	0.200463667	0.137350133	0.027602275
3h	ACA1_088720/clean/scf7180000084639:213382-213456:+	ACA1_088720	AmpGrelated permease [Source:UniProtKB/TrEMBL;Acc:L8GUN5]	0.0263158	0.1636205	0.1373047	0.047078199
3h	ACA1_265330/clean/scf7180000084770:439842-439916:+	ACA1_265330	F-actin-capping protein subunit alpha [Source:UniProtKB/TrEMBL;Acc:L8H1E5]	0.000524541	0.137812033	0.137287492	8.93E-09
3h	ACA1_061200/clean/scf7180000084599:853352-853441:+	ACA1_061200	5' nucleotidase family protein [Source:UniProtKB/TrEMBL;Acc:L8GWQ7]	0.030320567	0.167335633	0.137015067	0.002143776
3h	ACA1_200360/clean/scf7180000084753:428430-428589:+	ACA1_200360	Leucine rich repeat domain containing protein [Source:UniProtKB/TrEMBL;Acc:L8H2J7]	0.00963975	0.146554767	0.136915017	8.05E-05
3h	ACA1_037700/clean/scf7180000084568:4486-4564:+	ACA1_037700	Ras-GAP domain-containing protein [Source:UniProtKB/TrEMBL;Acc:L8GLC0]	0.020338933	0.157229233	0.1368903	0.000355523
3h	ACA1_171090/clean/scf7180000084741:38756-38842:-	ACA1_171090	Cytochrome-c oxidase [Source:UniProtKB/TrEMBL;Acc:L8HJI0]	0.00589185	0.142745967	0.136854117	3.24E-10
3h	ACA1_264070/clean/scf7180000084770:173181-173276:+	ACA1_264070	COPI_C domain-containing protein [Source:UniProtKB/TrEMBL;Acc:L8H460]	4.78E-05	0.1368445	0.136796683	0.000127594
3h	ACA1_058590/clean/scf7180000084599:357797-357897:+	ACA1_058590	Copine VIII, putative [Source:UniProtKB/TrEMBL;Acc:L8GXA8]	0.00137741	0.1380221	0.13664469	3.95E-06
3h	ACA1_339730/clean/scf7180000084619:14310-14417:+	ACA1_339730	PBP_domain domain-containing protein [Source:UniProtKB/TrEMBL;Acc:L8GJJ3]	0.000509317	0.137134067	0.13662475	5.47E-05
3h	ACA1_058790/clean/scf7180000084599:383108-383201:-	ACA1_058790	Rab GDP dissociation inhibitor [Source:UniProtKB/TrEMBL;Acc:L8GXB4]	0.000607949	0.137095267	0.136487318	1.44E-08
3h	ACA1_379350/clean/scf7180000084711:337834-337918:+	ACA1_379350	hypothetical protein	0.011779747	0.148239133	0.136459387	0.010561416
3h	ACA1_320550/clean/scf7180000084416:9600-9707:-	ACA1_320550	Proline iminopeptidase [Source:UniProtKB/TrEMBL;Acc:L8GWD8]	0.013080033	0.1495044	0.136424367	9.11E-06
3h	ACA1_109770/clean/scf7180000084663:21593-21769:+	ACA1_109770	WD40 repeat-containing protein [Source:UniProtKB/TrEMBL;Acc:L8HKT6]	0.002182377	0.1385398	0.136357423	7.58E-05
3h	ACA1_198570/clean/scf7180000084753:146688-146778:+	ACA1_198570	PCI domain-containing protein [Source:UniProtKB/TrEMBL;Acc:L8H5C2]	0.01314404	0.149482	0.13633796	7.11E-05
3h	ACA1_383470/clean/scf7180000084721:177553-177625:+	ACA1_383470	ANK_REP_REGION domain-containing protein [Source:UniProtKB/TrEMBL;Acc:L8GXK5]	0	0.136138	0.136138	2.27E-06
3h	ACA1_144270/clean/scf7180000084709:192472-192589:+	ACA1_144270	SAC domain-containing protein [Source:UniProtKB/TrEMBL;Acc:L8HI07]	0.01674967	0.152823867	0.136074197	0.003375907
3h	ACA1_017740/clean/scf7180000083876:4876-4989:+	ACA1_017740	5'-3' exoribonuclease 1 [Source:UniProtKB/TrEMBL;Acc:L8GMR4]	0.000317836	0.136335833	0.136017997	8.57E-07
3h	ACA1_398950/clean/scf7180000084759:255875-256007:+	ACA1_398950	START domain containing protein [Source:UniProtKB/TrEMBL;Acc:L8HBI4]	0.01265701	0.148610967	0.135953957	0.000273075
3h	ACA1_271380/clean/scf7180000084771:38105-38221:+	ACA1_271380	RL10P_insert domain-containing protein [Source:UniProtKB/TrEMBL;Acc:L8GPQ3]	0.00227586	0.138229133	0.135953273	5.79E-11
3h	ACA1_071540/clean/scf7180000084603:984728-984924:+	ACA1_071540	Dual specificity protein kinase [Source:UniProtKB/TrEMBL;Acc:L8HE77]	0.002627833	0.138444633	0.1358168	0.000450624
3h	ACA1_199500/clean/scf7180000084753:249243-249331:+	ACA1_199500	1acylglycerophosphocholine O-acyltransferase 1, putative [Source:UniProtKB/TrEMBL;Acc:L8H4L3]	0.000852513	0.136597667	0.135745153	0.019239273
3h	ACA1_301700/clean/scf7180000083222:21077-21157:-	ACA1_301700	hypothetical protein	0.00157695	0.1371847	0.13560775	2.56E-05
3h	ACA1_166910/clean/scf7180000084736:26933-27002:-	ACA1_166910	Amine oxidase family protein [Source:UniProtKB/TrEMBL;Acc:L8H242]	0	0.135267467	0.135267467	0.001632071
3h	ACA1_216020/clean/scf7180000084758:432652-432756:-	ACA1_216020	CudA family protein [Source:UniProtKB/TrEMBL;Acc:L8GQ76]	0.00843457	0.143670633	0.135236063	5.90E-05
3h	ACA1_025570/clean/scf7180000084448:16957-17038:+	ACA1_025570	hypothetical protein	0.008620227	0.1438176	0.135197373	2.12E-07
3h	ACA1_367260/clean/scf7180000084641:581366-581445:+	ACA1_367260	tRNA binding domain containing protein [Source:UniProtKB/TrEMBL;Acc:L8GMY8]	0.00124844	0.136436333	0.135187893	3.94E-06
3h	ACA1_074150/clean/scf7180000084605:37031-37114:+	ACA1_074150	Usp domain-containing protein [Source:UniProtKB/TrEMBL;Acc:L8HLV3]	0.008040482	0.1431519	0.135111418	3.40E-05
3h	ACA1_017740/clean/scf7180000083876:1127-1221:+	ACA1_017740	5'-3' exoribonuclease 1 [Source:UniProtKB/TrEMBL;Acc:L8GMR4]	0.0004529	0.135267633	0.134814733	4.75E-06
3h	ACA1_175870/clean/scf7180000084741:1079805-1079895:-	ACA1_175870	Zinc finger, C3HC4 type (RING finger) domain containing protein [Source:UniProtKB/TrEMBL;Acc:L8HI76]	0.0233097	0.158067	0.1347573	0.027466019
3h	ACA1_062680/clean/scf7180000084599:1272111-1272200:+	ACA1_062680	hypothetical protein	0.005846847	0.1405872	0.134740353	2.97E-05
3h	ACA1_116200/clean/scf7180000084669:724842-724919:+	ACA1_116200	WASH_WAHD domain-containing protein [Source:UniProtKB/TrEMBL;Acc:L8H3U1]	0.01048078	0.145190867	0.134710087	0.003785762
3h	ACA1_020770/clean/scf7180000084095:4068-4137:+	ACA1_020770	Multisensor hybrid histidine kinase [Source:UniProtKB/TrEMBL;Acc:L8GWG6]	0.040827243	0.175131667	0.134304423	0.037179075
3h	ACA1_292880/clean/scf7180000084777:926116-926185:-	ACA1_292880	RhoGEF domain containing protein [Source:UniProtKB/TrEMBL;Acc:L8HJL4]	0.009259267	0.1432183	0.133959033	0.00543975
3h	ACA1_189420/clean/scf7180000084748:33586-33667:+	ACA1_189420	Protein disulfideisomerase domain containing protein [Source:UniProtKB/TrEMBL;Acc:L8GE28]	0.00837209	0.1423256	0.13395351	0.00018876
3h	ACA1_385760/clean/scf7180000084730:257708-257833:-	ACA1_385760	MSP domain-containing protein [Source:UniProtKB/TrEMBL;Acc:L8H8G8]	0.020178073	0.154095633	0.13391756	0.010646795
3h	ACA1_178750/clean/scf7180000084743:276241-276321:-	ACA1_178750	PCI domain-containing protein [Source:UniProtKB/TrEMBL;Acc:L8GVV1]	0.00098531	0.134779767	0.133794457	9.69E-07
3h	ACA1_220120/clean/scf7180000084758:867294-867415:+	ACA1_220120	Uricase [Source:UniProtKB/TrEMBL;Acc:L8GQ21]	0.006013843	0.139789567	0.133775723	5.18E-07
3h	ACA1_238390/clean/scf7180000084763:82034-82159:-	ACA1_238390	Coronin [Source:UniProtKB/TrEMBL;Acc:L8GJQ2]	0.001774208	0.1355458	0.133771592	1.56E-06
3h	ACA1_222780/clean/scf7180000084760:81426-81553:-	ACA1_222780	Metal cation transporter, ZIP subfamily protein [Source:UniProtKB/TrEMBL;Acc:L8GSU8]	0.001181872	0.134933633	0.133751761	3.90E-06
3h	ACA1_057960/clean/scf7180000084599:222422-222502:+	ACA1_057960	BTB/POZ domain containing protein [Source:UniProtKB/TrEMBL;Acc:L8GVX1]	0.004774013	0.138405667	0.133631653	0.001136281
3h	ACA1_255760/clean/scf7180000084768:564743-564856:+	ACA1_255760	Acid phosphatase PHOa, putative [Source:UniProtKB/TrEMBL;Acc:L8HB67]	0.003746017	0.137117667	0.13337165	2.15E-08
3h	ACA1_271320/clean/scf7180000084771:19635-19952:-	ACA1_271320	CudAlike protein, putative [Source:UniProtKB/TrEMBL;Acc:L8GP00]	0.036002167	0.169361233	0.133359067	0.019586645
3h	ACA1_024150/clean/scf7180000084441:1949-2029:+	ACA1_024150	WH2 domain-containing protein [Source:UniProtKB/TrEMBL;Acc:L8GKR7]	0.011223767	0.144191667	0.1329679	0.000168282
3h	ACA1_197300/clean/scf7180000084753:1400-1497:-	ACA1_197300	hypothetical protein	0.010786543	0.143710467	0.132923923	0.009492133
3h	ACA1_034160/clean/scf7180000084561:38129-38198:+	ACA1_034160	FERM domain-containing protein [Source:UniProtKB/TrEMBL;Acc:L8GV43]	0.0291628	0.161950433	0.132787633	0.008666268
3h	ACA1_199710/clean/scf7180000084753:304132-304241:+	ACA1_199710	Phosphate transporter [Source:UniProtKB/TrEMBL;Acc:L8H377]	0	0.132745533	0.132745533	0.000187664
3h	ACA1_178260/clean/scf7180000084743:193606-193696:+	ACA1_178260	Cytochrome P450, putative [Source:UniProtKB/TrEMBL;Acc:L8GTE4]	0.007621563	0.1402218	0.132600237	1.19E-06
3h	ACA1_243590/clean/scf7180000084763:350786-350903:+	ACA1_243590	hypothetical protein	0.005163093	0.137735133	0.13257204	0.000637004
3h	ACA1_113590/clean/scf7180000084669:327129-327278:+	ACA1_113590	Kinase [Source:UniProtKB/TrEMBL;Acc:L8H3B8]	0.01088216	0.143430833	0.132548673	9.50E-05
3h	ACA1_294890/clean/scf7180000084777:1057642-1057761:+	ACA1_294890	UDENN domain-containing protein [Source:UniProtKB/TrEMBL;Acc:L8HIK8]	0.0305908	0.1630692	0.1324784	0.008736441
3h	ACA1_040830/clean/scf7180000084572:89562-89620:-	ACA1_040830	UBIQUITIN_CONJUGAT_2 domain-containing protein [Source:UniProtKB/TrEMBL;Acc:L8H0M3]	0.013153203	0.145545033	0.13239183	0.000284442
3h	ACA1_255410/clean/scf7180000084768:490326-490385:-	ACA1_255410	serine/threonine protein kinase, putative	0.004775367	0.137069967	0.1322946	0.000218557
3h	ACA1_140730/clean/scf7180000084707:107648-107707:-	ACA1_140730	Kelch repeat protein [Source:UniProtKB/TrEMBL;Acc:L8GGS1]	0.023519933	0.155807733	0.1322878	0.002898819
3h	ACA1_384700/clean/scf7180000084730:88944-89033:+	ACA1_384700	MFS domain-containing protein [Source:UniProtKB/TrEMBL;Acc:L8H981]	0.02382905	0.156112333	0.132283283	0.013238909
3h	ACA1_296920/clean/scf7180000084777:1434525-1434614:+	ACA1_296920	Adenylyl cyclase-associated protein [Source:UniProtKB/TrEMBL;Acc:L8HMG4]	0.004629465	0.136798667	0.132169202	5.16E-07
3h	ACA1_158800/clean/scf7180000084724:258271-258337:+	ACA1_158800	Fe-S_biosyn domain-containing protein [Source:UniProtKB/TrEMBL;Acc:L8HA83]	0.055256067	0.187408467	0.1321524	0.016269708
3h	ACA1_061140/clean/scf7180000084599:833025-833352:-	ACA1_061140	WD_REPEATS_REGION domain-containing protein [Source:UniProtKB/TrEMBL;Acc:L8GZ38]	0.00301333	0.1350502	0.13203687	0.000137597
3h	ACA1_035260/clean/scf7180000084564:7397-7470:-	ACA1_035260	Guanine nucleotidebinding protein G(O), alpha subunit, putative [Source:UniProtKB/TrEMBL;Acc:L8HCU4]	0.009175703	0.141194533	0.13201883	5.89E-05
3h	ACA1_131560/clean/scf7180000084699:89224-89359:-	ACA1_131560	hypothetical protein	0.002751307	0.134525233	0.131773927	6.47E-06
3h	ACA1_169100/clean/scf7180000084739:21846-21917:+	ACA1_169100	Dual specificity phosphatase, catalytic domain containing protein [Source:UniProtKB/TrEMBL;Acc:L8HG79]	0.000212905	0.131913333	0.131700429	7.00E-08
3h	ACA1_116720/clean/scf7180000084669:834015-834096:+	ACA1_116720	J domain-containing protein [Source:UniProtKB/TrEMBL;Acc:L8H4V8]	0.005569312	0.1367318	0.131162488	0.002132466
3h	ACA1_248630/clean/scf7180000084766:99506-99581:+	ACA1_248630	Thioredoxin domain-containing protein [Source:UniProtKB/TrEMBL;Acc:L8GZ87]	0.004069653	0.1351996	0.131129947	6.48E-09
3h	ACA1_216020/clean/scf7180000084758:433936-434050:-	ACA1_216020	CudA family protein [Source:UniProtKB/TrEMBL;Acc:L8GQ76]	0.003245357	0.1342726	0.131027243	0.000277947
3h	ACA1_164680/clean/scf7180000084729:247701-247778:-	ACA1_164680	ANK_REP_REGION domain-containing protein [Source:UniProtKB/TrEMBL;Acc:L8GSN8]	0.008770587	0.139657567	0.13088698	0.000532223
3h	ACA1_325560/clean/scf7180000084495:34064-34181:+	ACA1_325560	Solute carrier family protein [Source:UniProtKB/TrEMBL;Acc:L8H8Z4]	0.002093307	0.1329379	0.130844593	8.09E-06
3h	ACA1_325730/clean/scf7180000084495:60793-60884:+	ACA1_325730	Kelch repeat protein [Source:UniProtKB/TrEMBL;Acc:L8HC73]	0.002594787	0.133061733	0.130466947	2.17E-05
3h	ACA1_179190/clean/scf7180000084743:319213-319285:+	ACA1_179190	hypothetical protein	0.003524204	0.133634533	0.130110329	3.16E-06
3h	ACA1_152960/clean/scf7180000084720:41237-41304:+	ACA1_152960	Malate dehydrogenase [Source:UniProtKB/TrEMBL;Acc:L8HFT9]	0.000144108	0.130108967	0.129964858	3.05E-11
3h	ACA1_341100/clean/scf7180000084620:51173-51239:-	ACA1_341100	MFS domain-containing protein [Source:UniProtKB/TrEMBL;Acc:L8GR49]	0.00548629	0.1354164	0.12993011	0.00151685
3h	ACA1_217750/clean/scf7180000084758:602708-602777:+	ACA1_217750	Phosphoenolpyruvate carboxykinase (GTP), putative [Source:UniProtKB/TrEMBL;Acc:L8GQL5]	0.011744107	0.1416698	0.129925693	0.00065299
3h	ACA1_381510/clean/scf7180000084712:168193-168308:+	ACA1_381510	Pyr_redox_2 domain-containing protein [Source:UniProtKB/TrEMBL;Acc:L8GNN7]	0.020344133	0.150146567	0.129802433	0.003287328
3h	ACA1_039660/clean/scf7180000084570:17295-17384:+	ACA1_039660	NAD-specific glutamate dehydrogenase [Source:UniProtKB/TrEMBL;Acc:L8H6Y5]	0.001034923	0.130668467	0.129633543	1.72E-06
3h	ACA1_027490/clean/scf7180000084469:1472-1540:-	ACA1_027490	Calcium-transporting ATPase [Source:UniProtKB/TrEMBL;Acc:L8H4Z2]	0.002016827	0.131464933	0.129448107	5.37E-06
3h	ACA1_400280/clean/scf7180000084775:137528-137609:+	ACA1_400280	Mitogen-activated protein kinase [Source:UniProtKB/TrEMBL;Acc:L8GG28]	0.0268401	0.156137833	0.129297733	0.01379508
3h	ACA1_189600/clean/scf7180000084748:63272-63368:-	ACA1_189600	hypothetical protein	0.006252453	0.1355316	0.129279147	8.38E-05
3h	ACA1_318540/clean/scf7180000084158:19312-19457:+	ACA1_318540	SPX domain containing protein [Source:UniProtKB/TrEMBL;Acc:L8GH73]	0.00383207	0.132733833	0.128901763	0.000335657
3h	ACA1_258660/clean/scf7180000084769:297857-297933:-	ACA1_258660	PNP_UDP_1 domain-containing protein [Source:UniProtKB/TrEMBL;Acc:L8GFX7]	0.038953	0.1676679	0.1287149	0.010967357
3h	ACA1_293890/clean/scf7180000084777:984603-984732:+	ACA1_293890	hypothetical protein	0.009563943	0.138236167	0.128672223	0.004832036
3h	ACA1_212580/clean/scf7180000084758:153597-153678:+	ACA1_212580	hypothetical protein	0.00398959	0.1324482	0.12845861	0.000702028
3h	ACA1_227700/clean/scf7180000084761:120218-120353:-	ACA1_227700	Iso_dh domain-containing protein [Source:UniProtKB/TrEMBL;Acc:L8H7T8]	0.015505133	0.143673667	0.128168533	0.000776944
3h	ACA1_289380/clean/scf7180000084777:424678-424741:+	ACA1_289380	Npa1 domain-containing protein [Source:UniProtKB/TrEMBL;Acc:L8HJ45]	0.067597267	0.195761967	0.1281647	0.040470898
3h	ACA1_292880/clean/scf7180000084777:924291-924388:-	ACA1_292880	RhoGEF domain containing protein [Source:UniProtKB/TrEMBL;Acc:L8HJL4]	0	0.128051233	0.128051233	0.000619427
3h	ACA1_099100/clean/scf7180000084646:92401-92471:+	ACA1_099100	PH domain-containing protein [Source:UniProtKB/TrEMBL;Acc:L8H5M3]	0.004826263	0.132701767	0.127875503	0.000624009
3h	ACA1_274920/clean/scf7180000084773:141376-141469:+	ACA1_274920	PB1 domain containing protein [Source:UniProtKB/TrEMBL;Acc:L8GGA9]	0.019843333	0.147638567	0.127795233	0.000197504
3h	ACA1_097590/clean/scf7180000084645:1291251-1291384:-	ACA1_097590	Amine oxidase [Source:UniProtKB/TrEMBL;Acc:L8GK33]	0.009824503	0.137618733	0.12779423	1.35E-05
3h	ACA1_201480/clean/scf7180000084753:561472-561542:+	ACA1_201480	PKS_ER domain-containing protein [Source:UniProtKB/TrEMBL;Acc:L8H3J6]	0.012352787	0.139903567	0.12755078	0.000669242
3h	ACA1_233090/clean/scf7180000084762:124962-125064:+	ACA1_233090	Eukaryotic translation initiation factor 3 subunit B [Source:UniProtKB/TrEMBL;Acc:L8H3E1]	0.000418404	0.127968433	0.127550029	1.18E-07
3h	ACA1_055050/clean/scf7180000084594:640473-640552:+	ACA1_055050	SH3 domain containing protein [Source:UniProtKB/TrEMBL;Acc:L8H5H2]	0.003500885	0.130747333	0.127246448	2.05E-07
3h	ACA1_198970/clean/scf7180000084753:179514-179606:-	ACA1_198970	Viral Atype inclusion protein, putative [Source:UniProtKB/TrEMBL;Acc:L8H376]	0	0.1269347	0.1269347	3.75E-05
3h	ACA1_061530/clean/scf7180000084599:954853-954995:-	ACA1_061530	Ribosomal_L16 domain-containing protein [Source:UniProtKB/TrEMBL;Acc:L8GXX4]	0.001983523	0.12888	0.126896477	4.90E-16
3h	ACA1_036330/clean/scf7180000084565:80022-80106:-	ACA1_036330	Centractin, putative [Source:UniProtKB/TrEMBL;Acc:L8HDV5]	0.000603987	0.127347733	0.126743747	6.92E-07
3h	ACA1_061200/clean/scf7180000084599:853548-853676:+	ACA1_061200	5' nucleotidase family protein [Source:UniProtKB/TrEMBL;Acc:L8GWQ7]	0.037828433	0.164241867	0.126413433	0.003989056

3h	ACA1_040460/clean/scf7180000084572:7984-8073:-	ACA1_040460	Protein disulfideisomerase, putative [Source:UniProtKB/TrEMBL;Acc:L8H2W3]	0.0036186	0.129949733	0.126331133	5.37E-09
3h	ACA1_169710/clean/scf7180000084739:57664-57754:+	ACA1_169710	SHSP domain-containing protein [Source:UniProtKB/TrEMBL;Acc:L8HHU0]	0.0539727	0.1802086	0.1262359	0.041818369
3h	ACA1_195000/clean/scf7180000084751:50904-51022:+	ACA1_195000	CAP-Gly domain-containing protein [Source:UniProtKB/TrEMBL;Acc:L8H6Q6]	0	0.1261832	0.1261832	9.00E-06
3h	ACA1_106370/clean/scf7180000084660:94497-94571:+	ACA1_106370	Glyoxylate reductase [Source:UniProtKB/TrEMBL;Acc:L8GQ89]	0.010292117	0.136468333	0.126176217	0.002647045
3h	ACA1_378000/clean/scf7180000084711:161421-161502:+	ACA1_378000	Hydrolase, NUDIX domain containing protein [Source:UniProtKB/TrEMBL;Acc:L8GRB0]	0.011801867	0.137844533	0.126042667	0.011234692
3h	ACA1_190020/clean/scf7180000084748:113472-113570:+	ACA1_190020	ACPS domain-containing protein [Source:UniProtKB/TrEMBL;Acc:L8GGI7]	0.028413333	0.154331867	0.125918533	0.003173127
3h	ACA1_298640/clean/scf7180000082992:7645-7776:+	ACA1_298640	V-type proton ATPase subunit C [Source:UniProtKB/TrEMBL;Acc:L8H2N9]	0.000448976	0.1262941	0.125845124	3.99E-08
3h	ACA1_229520/clean/scf7180000084761:264383-264505:+	ACA1_229520	Protein serine/threonine kinase, putative [Source:UniProtKB/TrEMBL;Acc:L8H813]	0.0015873	0.1273462	0.1257589	2.46E-05
3h	ACA1_320830/clean/scf7180000084416:31631-31753:+	ACA1_320830	Lipoyl-binding domain-containing protein [Source:UniProtKB/TrEMBL;Acc:L8GT97]	0.007500347	0.133187367	0.12568702	2.28E-06
3h	ACA1_382280/clean/scf7180000084721:21587-21654:-	ACA1_382280	Aminotran_1_2 domain-containing protein [Source:UniProtKB/TrEMBL;Acc:L8GUT4]	0.007854213	0.133376467	0.125522253	0.002228292
3h	ACA1_230310/clean/scf7180000084761:342958-343055:+	ACA1_230310	PAS domain-containing protein [Source:UniProtKB/TrEMBL;Acc:L8HBA3]	0.003163323	0.1285892	0.125425877	0.000351558
3h	ACA1_208580/clean/scf7180000084757:227842-227904:-	ACA1_208580	GPI anchored protein [Source:UniProtKB/TrEMBL;Acc:L8GZM6]	0.00467207	0.129739633	0.125067563	8.41E-07
3h	ACA1_325740/clean/scf7180000084495:63596-63695:-	ACA1_325740	VDE domain-containing protein [Source:UniProtKB/TrEMBL;Acc:L8H9S3]	0.017102367	0.1421321	0.125029733	0.000644936
3h	ACA1_037930/clean/scf7180000084568:58532-58669:+	ACA1_037930	hypothetical protein	0.002781338	0.127790533	0.125009196	7.90E-06
3h	ACA1_395210/clean/scf7180000084756:156206-156286:-	ACA1_395210	Nonspecific lipidtransfer protein [Source:UniProtKB/TrEMBL;Acc:L8GZN6]	0.042394657	0.167403167	0.12500851	0.035900473
3h	ACA1_243590/clean/scf7180000084763:350993-351053:+	ACA1_243590	hypothetical protein	0	0.1245676	0.1245676	2.23E-05
3h	ACA1_232870/clean/scf7180000084762:90531-90634:+	ACA1_232870	Zn-finger in Ran binding protein and others domain containing protein [Source:UniProtKB/TrEMBL;Acc:L8H0Z7]	0.006821503	0.131286967	0.124465463	0.00024998
3h	ACA1_063740/clean/scf7180000084599:1493458-1493553:-	ACA1_063740	Transmembrane protein [Source:UniProtKB/TrEMBL;Acc:L8GXB1]	0.002941293	0.127400433	0.12445914	0.001394064
3h	ACA1_183320/clean/scf7180000084745:379045-379155:+	ACA1_183320	hypothetical protein	0.0225658	0.146920967	0.124355167	0.011251245
3h	ACA1_020160/clean/scf7180000084037:20052-20126:-	ACA1_020160	Sec16_C domain-containing protein [Source:UniProtKB/TrEMBL;Acc:L8GLH3]	0.000895643	0.125249333	0.12435369	5.76E-07
3h	ACA1_057240/clean/scf7180000084599:94130-94240:-	ACA1_057240	unspecified product	0.004412367	0.1287549	0.124342533	0.001163991
3h	ACA1_260070/clean/scf7180000084769:417660-417808:+	ACA1_260070	Vacuolar protein sorting-associated protein 29 [Source:UniProtKB/TrEMBL;Acc:L8GG04]	0.002331903	0.126674133	0.12434223	3.69E-08
3h	ACA1_035470/clean/scf7180000084564:58258-58407:+	ACA1_035470	Cation exchanger, putative [Source:UniProtKB/TrEMBL;Acc:L8HAF4]	0.053265367	0.177385	0.124119633	0.044875139
3h	ACA1_278190/clean/scf7180000084776:187446-187527:-	ACA1_278190	MAT1 domain-containing protein [Source:UniProtKB/TrEMBL;Acc:L8H822]	0.010447283	0.134551033	0.12410375	9.50E-05
3h	ACA1_265570/clean/scf7180000084770:513779-513913:-	ACA1_265570	Ribosomal_L2_C domain-containing protein [Source:UniProtKB/TrEMBL;Acc:L8H292]	0.00051339	0.1245617	0.12404831	1.80E-16
3h	ACA1_058270/clean/scf7180000084599:285779-285852:+	ACA1_058270	U-box domain-containing protein [Source:UniProtKB/TrEMBL;Acc:L8GYJ6]	0.008333333	0.132331733	0.1239984	0.009517897
3h	ACA1_232630/clean/scf7180000084762:50586-50722:+	ACA1_232630	FYVE-type domain-containing protein [Source:UniProtKB/TrEMBL;Acc:L8H3C0]	0.027762333	0.151740767	0.123978433	0.004866389
3h	ACA1_265500/clean/scf7180000084770:494988-495070:-	ACA1_265500	F-box domain-containing protein [Source:UniProtKB/TrEMBL;Acc:L8H275]	0.001862165	0.1257246	0.123862435	3.20E-09
3h	ACA1_248630/clean/scf7180000084766:99219-99324:+	ACA1_248630	Thioredoxin domain-containing protein [Source:UniProtKB/TrEMBL;Acc:L8GZ87]	0.00359443	0.127376233	0.123781803	1.43E-08
3h	ACA1_245940/clean/scf7180000084763:530591-530668:+	ACA1_245940	hypothetical protein	0.024973437	0.148677267	0.12370383	0.008525865
3h	ACA1_381620/clean/scf7180000084712:198373-198472:+	ACA1_381620	Alpha-galactosidase [Source:UniProtKB/TrEMBL;Acc:L8GMY9]	0.00158298	0.125277167	0.123694187	1.69E-09
3h	ACA1_050510/clean/scf7180000084589:109363-109437:+	ACA1_050510	4HBT domain-containing protein [Source:UniProtKB/TrEMBL;Acc:L8H578]	0.009648037	0.133326	0.123677963	0.000174365
3h	ACA1_123390/clean/scf7180000084686:28739-28830:-	ACA1_123390	hypothetical protein	0.015503867	0.138894267	0.1233904	0.012555297
3h	ACA1_163870/clean/scf7180000084729:96026-96125:+	ACA1_163870	hypothetical protein	0.1148369	0.238217667	0.123380767	0.031472881
3h	ACA1_061880/clean/scf7180000084599:1069882-1069967:-	ACA1_061880	UMP-CMP kinase [Source:UniProtKB/TrEMBL;Acc:L8GZB3]	0.005495687	0.1288733	0.123377613	3.68E-08
3h	ACA1_051550/clean/scf7180000084592:36158-36242:-	ACA1_051550	Phenylalanine hydroxylase [Source:UniProtKB/TrEMBL;Acc:L8GPS8]	0.003990772	0.1273136	0.123322828	1.14E-05
3h	ACA1_236110/clean/scf7180000084762:406745-406831:-	ACA1_236110	PPM-type phosphatase domain-containing protein [Source:UniProtKB/TrEMBL;Acc:L8H2W4]	0	0.123235533	0.123235533	1.61E-05
3h	ACA1_260100/clean/scf7180000084769:423402-423476:-	ACA1_260100	Ras-GAP domain-containing protein [Source:UniProtKB/TrEMBL;Acc:L8GHJ9]	0.016585767	0.139789	0.123203233	0.000148905
3h	ACA1_040840/clean/scf7180000084572:91473-91534:-	ACA1_040840	BRO1 domain-containing protein [Source:UniProtKB/TrEMBL;Acc:L8H1X9]	0.000685933	0.1236902	0.123004267	9.53E-08
3h	ACA1_382920/clean/scf7180000084721:119112-119198:-	ACA1_382920	Ras subfamily protein [Source:UniProtKB/TrEMBL;Acc:L8GV31]	0	0.122927	0.122927	0.001058697
3h	ACA1_385810/clean/scf7180000084730:274303-274407:+	ACA1_385810	Rap-GAP domain-containing protein [Source:UniProtKB/TrEMBL;Acc:L8HAQ1]	0.049138733	0.1717731	0.122634367	0.037167542
3h	ACA1_352410/clean/scf7180000084704:14306-14418:+	ACA1_352410	FYVE-type domain-containing protein [Source:UniProtKB/TrEMBL;Acc:L8GUU5]	0.0260973	0.148661567	0.122564267	0.004338999
3h	ACA1_385390/clean/scf7180000084730:161704-161803:+	ACA1_385390	Ubiquitinconjugating enzyme subfamily protein [Source:UniProtKB/TrEMBL;Acc:L8HAL6]	0.00842277	0.1308877	0.12246493	4.88E-05
3h	ACA1_395180/clean/scf7180000084756:149710-149814:+	ACA1_395180	Protein ei24 family protein [Source:UniProtKB/TrEMBL;Acc:L8H2T6]	0.053267067	0.175567933	0.122300867	0.023825246
3h	ACA1_381510/clean/scf7180000084712:166732-166810:+	ACA1_381510	Pyr_redox_2 domain-containing protein [Source:UniProtKB/TrEMBL;Acc:L8GNN7]	4.54E-05	0.122246167	0.122200784	3.47E-06
3h	ACA1_174420/clean/scf7180000084741:706944-707019:+	ACA1_174420	Diacylglycerol kinase [Source:UniProtKB/TrEMBL;Acc:L8HK54]	0.01652429	0.1386794	0.12215511	0.00091236
3h	ACA1_371680/clean/scf7180000084664:576882-576959:-	ACA1_371680	BAR domain containing protein [Source:UniProtKB/TrEMBL;Acc:L8GZ90]	0.004610833	0.126638233	0.1220274	1.03E-06
3h	ACA1_088880/clean/scf7180000084639:253003-253072:-	ACA1_088880	OGFr_N domain-containing protein [Source:UniProtKB/TrEMBL;Acc:L8GUP7]	0.0049795	0.126904633	0.121925133	0.007246119
3h	ACA1_164790/clean/scf7180000084729:284759-284879:+	ACA1_164790	Ras-related protein Rab-2A, putative [Source:UniProtKB/TrEMBL;Acc:L8GSP6]	0.00136259	0.123205933	0.121843343	1.13E-07
3h	ACA1_335940/clean/scf7180000084593:8675-8805:-	ACA1_335940	RNA recognition motif domain containing protein [Source:UniProtKB/TrEMBL;Acc:L8H3Y6]	0.019690667	0.1414197	0.121729033	0.002919185
3h	ACA1_158900/clean/scf7180000084724:294888-295055:+	ACA1_158900	hypothetical protein	0.007743267	0.129342533	0.121599267	0.011126242
3h	ACA1_387720/clean/scf7180000084733:66557-66615:+	ACA1_387720	Usp domain-containing protein [Source:UniProtKB/TrEMBL;Acc:L8GDP5]	0.004399947	0.1256712	0.121271253	0.000199599
3h	ACA1_040830/clean/scf7180000084572:89216-89311:-	ACA1_040830	UBIQUITIN_CONJUGAT_2 domain-containing protein [Source:UniProtKB/TrEMBL;Acc:L8H0M3]	0.020936933	0.142159067	0.121222133	0.00154091
3h	ACA1_380860/clean/scf7180000084712:102077-102189:+	ACA1_380860	Pantoate-beta-alanine ligase [Source:UniProtKB/TrEMBL;Acc:L8GNL4]	0.0139994	0.134976	0.1209766	5.62E-05
3h	ACA1_091040/clean/scf7180000084645:45380-45466:-	ACA1_091040	Amidohydro-rel domain-containing protein [Source:UniProtKB/TrEMBL;Acc:L8GIF1]	0.006053843	0.126774233	0.12072039	0.001165017
3h	ACA1_069320/clean/scf7180000084603:409362-409454:+	ACA1_069320	Eukaryotic initiation factor, putative [Source:UniProtKB/TrEMBL;Acc:L8HF00]	0.012506433	0.133166733	0.1206603	0.000102389
3h	ACA1_208110/clean/scf7180000084757:106926-107021:+	ACA1_208110	Phosphoinositide 3kinase family, accessory domain (Pik domain) domain containing protein [Source:UniProtKB/TrEMBL;Acc:L8GXD2]	0.009390205	0.130041967	0.120651761	0.000708513
3h	ACA1_232690/clean/scf7180000084762:68636-68737:-	ACA1_232690	Vacuolar proton pump d subunit, putative [Source:UniProtKB/TrEMBL;Acc:L8H122]	0.002564697	0.123202633	0.120637937	1.13E-08
3h	ACA1_110500/clean/scf7180000084663:140017-140099:+	ACA1_110500	TMEM189_B_dmain domain-containing protein [Source:UniProtKB/TrEMBL;Acc:L8HKY1]	0.016027067	0.136514567	0.1204875	0.002265846
3h	ACA1_054850/clean/scf7180000084594:591747-591851:-	ACA1_054850	Usp domain-containing protein [Source:UniProtKB/TrEMBL;Acc:L8H5F4]	0.009390427	0.129649967	0.12025954	0.001100154
3h	ACA1_399900/clean/scf7180000084775:59344-59418:-	ACA1_399900	Usp domain-containing protein [Source:UniProtKB/TrEMBL;Acc:L8GID1]	0.018733833	0.138952067	0.120218233	0.000377463
3h	ACA1_026710/clean/scf7180000084458:8776-8902:+	ACA1_026710	Formin domain containing protein [Source:UniProtKB/TrEMBL;Acc:L8GRW6]	0.013620933	0.133601133	0.1199802	0.000462382
3h	ACA1_142400/clean/scf7180000084708:159959-160034:+	ACA1_142400	Plus3 domain-containing protein [Source:UniProtKB/TrEMBL;Acc:L8HAK6]	0	0.119941267	0.119941267	7.59E-05
3h	ACA1_296560/clean/scf7180000084777:1384581-1384737:-	ACA1_296560	CitMHS domain-containing protein [Source:UniProtKB/TrEMBL;Acc:L8HLL2]	0.001669047	0.121492833	0.119823787	0.000281287
3h	ACA1_144200/clean/scf7180000084709:171252-171335:+	ACA1_144200	PH domain-containing protein [Source:UniProtKB/TrEMBL;Acc:L8HET7]	0.00881679	0.1286117	0.11979491	6.06E-07
3h	ACA1_283290/clean/scf7180000084776:809397-809490:-	ACA1_283290	Rho-GAP domain-containing protein [Source:UniProtKB/TrEMBL;Acc:L8H7D8]	0.014836033	0.134482033	0.119646	0.000482036
3h	ACA1_097540/clean/scf7180000084645:1274788-1274908:-	ACA1_097540	Serine/threonine kinase [Source:UniProtKB/TrEMBL;Acc:L8GK27]	0.030998533	0.1505638	0.119565267	0.014166512
3h	ACA1_082010/clean/scf7180000084618:158488-158576:+	ACA1_082010	Protein kinase domain-containing protein [Source:UniProtKB/TrEMBL;Acc:L8HBY6]	0	0.119544567	0.119544567	0.024040507
3h	ACA1_184040/clean/scf7180000084745:547943-548019:-	ACA1_184040	Ubiquitin-like domain-containing protein [Source:UniProtKB/TrEMBL;Acc:L8H9Q4]	0.003867757	0.123389433	0.119521677	2.80E-11
3h	ACA1_048110/clean/scf7180000084582:22456-22526:-	ACA1_048110	Serine carboxypeptidase s28 protein [Source:UniProtKB/TrEMBL;Acc:L8GD90]	0.002524387	0.1218679	0.119343513	1.46E-06
3h	ACA1_387720/clean/scf7180000084733:66945-67033:+	ACA1_387720	Usp domain-containing protein [Source:UniProtKB/TrEMBL;Acc:L8GDP5]	0.043381733	0.162721	0.119339267	0.016103892
3h	ACA1_091710/clean/scf7180000084645:187013-187098:-	ACA1_091710	PAS domain Sbox domain containing protein [Source:UniProtKB/TrEMBL;Acc:L8GKF5]	0.000165037	0.119320633	0.119155596	7.05E-07
3h	ACA1_299380/clean/scf7180000083027:12070-12141:+	ACA1_299380	AAA_11 domain-containing protein [Source:UniProtKB/TrEMBL;Acc:L8GS61]	0.000765822	0.1198926	0.119126778	8.77E-07
3h	ACA1_396210/clean/scf7180000084759:54500-54630:+	ACA1_396210	WD_REPEATS_REGION domain-containing protein [Source:UniProtKB/TrEMBL;Acc:L8HCB3]	0.018086883	0.137206067	0.119119183	0.000467026
3h	ACA1_014100/clean/scf7180000083757:8165-8244:+	ACA1_014100	ATP-grasp_2 domain-containing protein [Source:UniProtKB/TrEMBL;Acc:L8GIY7]	0.00117911	0.1202551	0.11907599	5.17E-08
3h	ACA1_166980/clean/scf7180000084736:43793-43896:-	ACA1_166980	hypothetical protein	0.0519931	0.170919167	0.118926067	0.032799279
3h	ACA1_329470/clean/scf7180000084562:19153-19225:-	ACA1_329470	Oxysterolbinding protein-like protein 8 isoform b, putative [Source:UniProtKB/TrEMBL;Acc:L8GK41]	0.001110197	0.120007033	0.118896836	1.83E-06
3h	ACA1_074930/clean/scf7180000084608:115913-115986:+	ACA1_074930	Calponin domain containing protein [Source:UniProtKB/TrEMBL;Acc:L8HHK0]	0.020080167	0.138777233	0.118697067	0.00245807
3h	ACA1_291040/clean/scf7180000084777:724812-724915:+	ACA1_291040	WRKY DNA-binding domain containing protein [Source:UniProtKB/TrEMBL;Acc:L8HLM9]	0.004261627	0.1228622	0.118600573	0.000454667
3h	ACA1_001520/clean/scf7180000082863:1311-1399:-	ACA1_001520	Corrinoid adenosyltransferase [Source:UniProtKB/TrEMBL;Acc:L8GGP9]	0.014818	0.133392633	0.118574633	0.000176143
3h	ACA1_091980/clean/scf7180000084645:240590-240659:+	ACA1_091980	Synaptobrevin, putative [Source:UniProtKB/TrEMBL;Acc:L8GIW2]	0.011876107	0.130341333	0.118465227	0.001094274
3h	ACA1_246780/clean/scf7180000084763:580226-580315:-	ACA1_246780	Ras subfamily protein [Source:UniProtKB/TrEMBL;Acc:L8GK82]	0.002856183	0.121049333	0.11819315	3.35E-05
3h	ACA1_142840/clean/scf7180000084708:246375-246486:-	ACA1_142840	hypothetical protein	0.0023174	0.1205086	0.1181912	0.000717033
3h	ACA1_041940/clean/scf7180000084573:22881-22969:+	ACA1_041940	Deoxyribonuclease II, putative [Source:UniProtKB/TrEMBL;Acc:L8GV83]	0.019710213	0.137596267	0.117886053	0.003581513
3h	ACA1_228720/clean/scf7180000084761:196361-196426:+	ACA1_228720	BRO1 domain-containing protein [Source:UniProtKB/TrEMBL;Acc:L8HB55]	0	0.1178666	0.1178666	2.12E-06
3h	ACA1_261320/clean/scf7180000084769:636551-636630:-	ACA1_261320	Aminotran_1_2 domain-containing protein [Source:UniProtKB/TrEMBL;Acc:L8GFF0]	0.00891954	0.126486	0.11756646	1.18E-05
3h	ACA1_392150/clean/scf7180000084740:262546-262655:-	ACA1_392150	AAA domain-containing protein [Source:UniProtKB/TrEMBL;Acc:L8GQ70]	0.000118099	0.117020533	0.116902435	5.77E-06
3h	ACA1_067410/clean/scf7180000084601:18019-18106:-	ACA1_067410	F-box domain-containing protein [Source:UniProtKB/TrEMBL;Acc:L8GTP9]	0.00017307	0.1169432	0.11677013	0.000431968
3h	ACA1_274580/clean/scf7180000084773:116788-116866:-	ACA1_274580	Carrier superfamily protein [Source:UniProtKB/TrEMBL;Acc:L8GGL3]	0.001452279	0.1180007	0.116548421	7.44E-06
3h	ACA1_018760/clean/scf7180000083912:10367-10438:+	ACA1_018760	Cell cycle control protein [Source:UniProtKB/TrEMBL;Acc:L8HJG5]	0.003270497	0.119814	0.116543503	2.63E-05
3h	ACA1_117270/clean/scf7180000084669:966954-967042:+	ACA1_117270	hypothetical protein	0.002949853	0.119487767	0.116537913	0.000135021
3h	ACA1_220760/clean/scf7180000084758:962771-962869:-	ACA1_220760	Methyltransf_11 domain-containing protein [Source:UniProtKB/TrEMBL;Acc:L8GQP4]	0.000525002	0.116986233	0.116461232	1.40E-06
3h	ACA1_189450/clean/scf7180000084748:42810-42892:-	ACA1_189450	Chloride channel protein [Source:UniProtKB/TrEMBL;Acc:L8GEJ2]	0.003061997	0.119466133	0.116404137	3.92E-06
3h	ACA1_199060/clean/scf7180000084753:210254-210339:-	ACA1_199060	HECT domain-containing protein [Source:UniProtKB/TrEMBL;Acc:L8H2E7]	0.010101	0.126225533	0.116124533	0.009179934
3h	ACA1_355860/clean/scf7180000084728:35421-35494:+	ACA1_355860	hypothetical protein	0.007216993	0.1232138	0.115996807	2.23E-05
3h	ACA1_301700/clean/scf7180000083222:20427-20542:-	ACA1_301700	hypothetical protein	0.035897933	0.151852733	0.1159548	0.012131463
3h	ACA1_074970/clean/scf7180000084608:120897-120962:+	ACA1_074970	TPR_REGION domain-containing protein [Source:UniProtKB/TrEMBL;Acc:L8HEG7]	0.002043457	0.1177416	0.115698143	3.01E-13
3h	ACA1_088970/clean/scf7180000084639:276326-276410:-	ACA1_088970	hypothetical protein	0.004592197	0.120208567	0.11561637	3.01E-06
3h	ACA1_125120/clean/scf7180000084691:33696-33774:-	ACA1_125120	UBP-type domain-containing protein [Source:UniProtKB/TrEMBL;Acc:L8GNN3]	0.020013183	0.135550133	0.11553695	0.004090198
3h	ACA1_347020/clean/scf7180000084665:3371-3441:-	ACA1_347020	VWFA domain-containing protein [Source:UniProtKB/TrEMBL;Acc:L8GVX0]	0.00162511	0.1171222	0.11549709	3.27E-06
3h	ACA1_121830/clean/scf7180000084682:105033-105125:-	ACA1_121830	COesterase domain-containing protein [Source:UniProtKB/TrEMBL;Acc:L8GEL0]	0.001213263	0.1166898	0.115476537	5.34E-05
3h	ACA1_088960/clean/scf7180000084639:274948-275008:-	ACA1_088960	Beta-lactamase domain-containing protein [Source:UniProtKB/TrEMBL;Acc:L8GU04]	0	0.114890167	0.114890167	0.002079373
3h	ACA1_087450/clean/scf7180000084639:31755-31812:-	ACA1_087450	Ras subfamily protein [Source:UniProtKB/TrEMBL;Acc:L8GVE7]	0.041035367	0.1559154	0.114880033	0.041458288
3h	ACA1_062590/clean/scf7180000084599:1250660-1250743:+	ACA1_062590	TOG domain-containing protein [Source:UniProtKB/TrEMBL;Acc:L8GWY7]	0.016907367	0.131549067	0.1146417	0.001049668
3h	ACA1_323510/clean/scf7180000084468:39259-39355:+	ACA1_323510	hypothetical protein	0.011530207	0.1258686	0.114338393	0.000294831
3h	ACA1_143980/clean/scf7180000084709:119373-119468:-	ACA1_143980	hypothetical protein	0	0.1140795	0.1140795	0.047024349
3h	ACA1_392150/clean/scf7180000084740:261234-261335:-	ACA1_392150	AAA domain-containing protein [Source:UniProtKB/TrEMBL;Acc:L8GQ70]	0.00868891	0.122645967	0.113957057	0.00022798
3h	ACA1_239550/clean/scf7180000084763:165990-166091:-	ACA1_239550	Cytidine and deoxycytidylate deaminase zincbinding region subfamily protein [Source:UniProtKB/TrEMBL;Acc:L8GKS6]	0.001718596	0.1154853	0.113766704	1.81E-05
3h	ACA1_173160/clean/scf7180000084741:414278-414403:+	ACA1_173160	hypothetical protein	0.00494639	0.1186497	0.11370331	0.001963
3h	ACA1_196090/clean/scf7180000084752:50988-51223:+	ACA1_196090	SAC domain-containing protein [Source:UniProtKB/TrEMBL;Acc:L8HEU4]	0.021502033	0.135152267	0.113650233	0.001016328
3h	ACA1_381620/clean/scf7180000084712:197791-197870:+	ACA1_381620	Alpha-galactosidase [Source:UniProtKB/TrEMBL;Acc:L8GMY9]	0.001880768	0.115498933	0.113618166	1.19E-07
3h	ACA1_074150/clean/scf7180000084605:37233-37331:+	ACA1_074150	Usp domain-containing protein [Source:UniProtKB/TrEMBL;Acc:L8HLV3]	0.02095824	0.134566767	0.113608527	0.001024957
3h	ACA1_395220/clean/scf7180000084756:157662-157757:+	ACA1_395220	Eukaryotic initiation factor 4E [Source:UniProtKB/TrEMBL;Acc:L8H0E9]	0.020193887	0.133786967	0.11359308	0.004248862
3h	ACA1_044710/clean/scf7180000084576:34793-34880:+	ACA1_044710	Protein kinase domain-containing protein [Source:UniProtKB/TrEMBL;Acc:L8H263]	0.010147833	0.123678033	0.1135302	4.26E-06
3h	ACA1_244750/clean/scf7180000084763:407699-407776:-	ACA1_244750	Oxysterol binding protein [Source:UniProtKB/TrEMBL;Acc:L8GK15]	0.002065057	0.1152784	0.113213343	6.29E-06
3h	ACA1_109780/clean/scf7180000084663:25203-25303:-	ACA1_109780	AAA domain-containing protein [Source:UniProtKB/TrEMBL;Acc:L8HIF9]	0	0.113203133	0.113203133	1.29E-05
3h	ACA1_261530/clean/scf7180000084769:700149-700210:+	ACA1_261530	hypothetical protein	0	0.113106267	0.113106267	0.00432038
3h	ACA1_057100/clean/scf7180000084599:44807-44903:-	ACA1_057100	unspecified product	0.00852799	0.121080533	0.112552543	0.005138046
3h	ACA1_322390/clean/scf7180000084463:17145-17239:+	ACA1_322390	DIL domain containing protein [Source:UniProtKB/TrEMBL;Acc:L8HH10]	0.005654942	0.118101067	0.112446125	0.003358614
3h	ACA1_054330/clean/scf7180000084594:469736-469837:-	ACA1_054330	Hbxip, putative [Source:UniProtKB/TrEMBL;Acc:L8H639]	0.00486079	0.1172539	0.11239311	1.61E-05
3h	ACA1_290420/clean/scf7180000084777:589777-589844:-	ACA1_290420	hypothetical protein	0.018148453	0.130449033	0.11230058	0.002105383
3h	ACA1_193120/clean/scf7180000084749:191511-191651:+	ACA1_193120	KxDL domain-containing protein [Source:UniProtKB/TrEMBL;Acc:L8GNQ6]	0.046991333	0.159130567	0.112139233	0.024507989
3h	ACA1_105760/clean/scf7180000084660:7715-7794:-	ACA1_105760	Proteasome/cyclosome repeat containing protein [Source:UniProtKB/TrEMBL;Acc:L8GMH4]	0.0141835	0.126187733	0.112004233	6.35E-05
3h	ACA1_182380/clean/scf7180000084745:201014-201096:+	ACA1_182380	TFIIS central domain-containing protein [Source:UniProtKB/TrEMBL;Acc:L8HA65]	0.021841167	0.133515967	0.1116748	0.004125774
3h	ACA1_220200/clean/scf7180000084758:897342-897416:+	ACA1_220200	Aminopeptidase [Source:UniProtKB/TrEMBL;Acc:L8GT26]	0.000931283	0.112592867	0.111661584	2.58E-06
3h	ACA1_055050/clean/scf7180000084594:640926-641000:+	ACA1_055050	SH3 domain containing protein [Source:UniProtKB/TrEMBL;Acc:L8H5H2]	0.000862589	0.1121805	0.111317911	1.04E-07
3h	ACA1_167330/clean/scf7180000084737:6308-6472:+	ACA1_167330	hypothetical protein	0.000621133	0.111835767	0.111214633	0.000243425
3h	ACA1_260070/clean/scf7180000084769:418299-418397:+	ACA1_260070	Vacuolar protein sorting-associated protein 29 [Source:UniProtKB/TrEMBL;Acc:L8GG04]	0.00123298	0.112130567	0.110897587	1.46E-07
3h	ACA1_110310/clean/scf7180000084663:88331-88404:-	ACA1_110310	11beta-hydroxysteroid dehydrogenase-like, putative [Source:UniProtKB/TrEMBL;Acc:L8HIJ0]	0.005748203	0.116608267	0.110860063	4.38E-05
3h	ACA1_176150/clean/scf7180000084741:1134872-1134955:+	ACA1_176150	PhytanoylCoA dioxygenase (PhyH) superfamily protein [Source:UniProtKB/TrEMBL;Acc:L8HI91]	0.00272513	0.1134915	0.11076637	0.000329668
3h	ACA1_336130/clean/scf7180000084593:30413-30743:-	ACA1_336130	Filamin repeat domain containing protein [Source:UniProtKB/TrEMBL;Acc:L8H1N2]	0.012953227	0.122857833	0.109904607	2.73E-07
3h	ACA1_201390/clean/scf7180000084753:539728-539814:-	ACA1_201390	Transmembrane 9 superfamily member [Source:UniProtKB/TrEMBL;Acc:L8H5N1]	0.00596504	0.1155725	0.10960746	1.58E-05
3h	ACA1_108230/clean/scf7180000084661:19764-19966:-	ACA1_108230	hypothetical protein	0.00358695	0.113123933	0.109536983	1.30E-05
3h	ACA1_383660/clean/scf7180000084721:240864-240939:+	ACA1_383660	tRNA (guanine-N(7)-)-methyltransferase non-catalytic subunit [Source:UniProtKB/TrEMBL;Acc:L8GW53]	6.03E-05	0.109391167	0.109330846	9.80E-05
3h	ACA1_368770/clean/scf7180000084664:157312-157398:-	ACA1_368770	Glyco_transf_20 domain-containing protein [Source:UniProtKB/TrEMBL;Acc:L8GYU8]	0	0.109258167	0.109258167	0.000542623
3h	ACA1_276210/clean/scf7180000084774:141829-141894:+	ACA1_276210	Aspartate aminotransferase [Source:UniProtKB/TrEMBL;Acc:L8GTG7]	0.011836	0.120782333	0.108946333	1.44E-05
3h	ACA1_390750/clean/scf7180000084740:87560-87633:-	ACA1_390750	zf-C2H2_jaz domain-containing protein [Source:UniProtKB/TrEMBL;Acc:L8GQ13]	0.044222533	0.152532733	0.1083102	0.011810962
3h	ACA1_339890/clean/scf7180000084619:29978-30067:-	ACA1_339890	Plexin repeat domain containing protein [Source:UniProtKB/TrEMBL;Acc:L8GM00]	0.006427697	0.1146453	0.108217603	7.82E-06

3h	ACA1_203020/clean/scf7180000084754:122990-123065:-	ACA1_203020	Protein pns1, putative [Source:UniProtKB/TrEMBL;Acc:L8GUJ1]	0.000677353	0.108879067	0.108201713	1.67E-05
3h	ACA1_107640/clean/scf7180000084660:239087-239158:+	ACA1_107640	Signal transduction histidine kinase [Source:UniProtKB/TrEMBL;Acc:L8GNL7]	0.01532862	0.1234887	0.10816008	0.002793656
3h	ACA1_163390/clean/scf7180000084729:21588-21654:+	ACA1_163390	Defective in cullin neddylation protein [Source:UniProtKB/TrEMBL;Acc:L8GRF5]	0.025069867	0.133181	0.108111133	0.006883812
3h	ACA1_037490/clean/scf7180000084567:103144-103257:-	ACA1_037490	hypothetical protein	0.006788147	0.114806567	0.10801842	0.001316073
3h	ACA1_040750/clean/scf7180000084572:75091-75212:+	ACA1_040750	Transporter, permease [Source:UniProtKB/TrEMBL;Acc:L8GZS4]	0.007246367	0.115038133	0.107791767	0.006898823
3h	ACA1_293890/clean/scf7180000084777:987036-987135:+	ACA1_293890	hypothetical protein	0.010993103	0.118608833	0.10761573	0.003429961
3h	ACA1_279520/clean/scf7180000084776:408725-408818:-	ACA1_279520	hypothetical protein	0.009696367	0.1170368	0.107340433	0.001525552
3h	ACA1_201880/clean/scf7180000084754:6500-6585:+	ACA1_201880	Protein kinase domain-containing protein [Source:UniProtKB/TrEMBL;Acc:L8GVZ2]	0.018991577	0.126258633	0.107267057	0.002997426
3h	ACA1_289610/clean/scf7180000084777:430296-430363:+	ACA1_289610	Copper transporter [Source:UniProtKB/TrEMBL;Acc:L8HKL4]	0.001889803	0.109023267	0.107133463	2.01E-05
3h	ACA1_217370/clean/scf7180000084758:580696-580781:+	ACA1_217370	Adaptorrelated protein complex 1, beta 1 subunit, isoform 2, putative [Source:UniProtKB/TrEMBL;Acc:L8GPS7]	0.001507492	0.108361633	0.106854141	1.17E-05
3h	ACA1_318540/clean/scf7180000084158:21397-21562:+	ACA1_318540	SPX domain containing protein [Source:UniProtKB/TrEMBL;Acc:L8GH73]	0.001678193	0.108328133	0.10664994	0.000124192
3h	ACA1_392150/clean/scf7180000084740:261947-262026:-	ACA1_392150	AAA domain-containing protein [Source:UniProtKB/TrEMBL;Acc:L8GQ70]	0.00381689	0.1104031	0.10658621	0.000311929
3h	ACA1_265510/clean/scf7180000084770:498871-498969:+	ACA1_265510	Palmitoylprotein thioesterase, putative [Source:UniProtKB/TrEMBL;Acc:L8H4E8]	0.024109733	0.129962767	0.105853033	0.041570478
3h	ACA1_380210/clean/scf7180000084712:32050-32185:-	ACA1_380210	DNA-(apurinic or apyrimidinic site) lyase [Source:UniProtKB/TrEMBL;Acc:L8GQH4]	0.001730103	0.1074637	0.105733597	0.001326023
3h	ACA1_087450/clean/scf7180000084639:33020-33115:-	ACA1_087450	Ras subfamily protein [Source:UniProtKB/TrEMBL;Acc:L8GVE7]	0.005437313	0.111145567	0.105708253	0.008492526
3h	ACA1_188340/clean/scf7180000084747:160354-160505:+	ACA1_188340	GLTP domain-containing protein [Source:UniProtKB/TrEMBL;Acc:L8GUX0]	0.003655937	0.109196467	0.10554053	3.58E-05
3h	ACA1_175160/clean/scf7180000084741:878633-878728:-	ACA1_175160	Alpha/beta hydrolase fold domain containing protein [Source:UniProtKB/TrEMBL;Acc:L8HI00]	0.0116328	0.116786733	0.105153933	0.000399858
3h	ACA1_220050/clean/scf7180000084758:850791-850886:+	ACA1_220050	Calponin domain containing protein [Source:UniProtKB/TrEMBL;Acc:L8GRJ6]	0.01777091	0.122507667	0.104736757	0.000673513
3h	ACA1_073230/clean/scf7180000084603:1376309-1376415:-	ACA1_073230	EHN domain-containing protein [Source:UniProtKB/TrEMBL;Acc:L8HDT9]	0.00420419	0.108845933	0.104641743	0.002267325
3h	ACA1_040520/clean/scf7180000084572:27133-27191:+	ACA1_040520	Golgi family protein, putative [Source:UniProtKB/TrEMBL;Acc:L8H2W6]	0.01294178	0.1173747	0.10443292	7.92E-05
3h	ACA1_298640/clean/scf7180000082992:7385-7550:+	ACA1_298640	V-type proton ATPase subunit C [Source:UniProtKB/TrEMBL;Acc:L8H2N9]	0.000277684	0.1046844	0.104406716	3.36E-08
3h	ACA1_389500/clean/scf7180000084733:268798-268977:+	ACA1_389500	hypothetical protein	0.00261459	0.106976633	0.104362043	4.12E-09
3h	ACA1_291040/clean/scf7180000084777:724254-724340:+	ACA1_291040	WRKY DNA-binding domain containing protein [Source:UniProtKB/TrEMBL;Acc:L8HLM9]	0	0.103923033	0.103923033	0.000220512
3h	ACA1_335940/clean/scf7180000084593:10605-10789:-	ACA1_335940	RNA recognition motif domain containing protein [Source:UniProtKB/TrEMBL;Acc:L8H3Y6]	0.012167044	0.115836667	0.103669623	0.002018008
3h	ACA1_175510/clean/scf7180000084741:974652-974745:+	ACA1_175510	Xylose isomerase [Source:UniProtKB/TrEMBL;Acc:L8HKE1]	0.001618111	0.1049259	0.103307789	3.83E-05
3h	ACA1_071840/clean/scf7180000084603:1077016-1077121:+	ACA1_071840	DIL domain containing protein [Source:UniProtKB/TrEMBL;Acc:L8HEB0]	0.002716237	0.105765767	0.10304953	0.000583032
3h	ACA1_193180/clean/scf7180000084749:206836-206929:+	ACA1_193180	Serine incorporator 3, putative [Source:UniProtKB/TrEMBL;Acc:L8GN74]	0.00809503	0.111121967	0.103026937	4.88E-05
3h	ACA1_384850/clean/scf7180000084730:115091-115197:-	ACA1_384850	Arrestin_C domain-containing protein [Source:UniProtKB/TrEMBL;Acc:L8H8B9]	0.012217967	0.114045567	0.1018276	0.003127723
3h	ACA1_392520/clean/scf7180000084740:284540-284636:-	ACA1_392520	hypothetical protein	0.00152633	0.1023514	0.10082507	0.000238241
3h	ACA1_384830/clean/scf7180000084730:111352-111421:-	ACA1_384830	Raslike protein 1, putative [Source:UniProtKB/TrEMBL;Acc:L8H942]	0.00166175	0.102035133	0.100373383	1.01E-07
3h	ACA1_330780/clean/scf7180000084566:67287-67382:-	ACA1_330780	hypothetical protein	0.014492767	0.114569867	0.1000771	0.036417083
3h	ACA1_396680/clean/scf7180000084759:93300-93438:-	ACA1_396680	Phospholipase A1, putative [Source:UniProtKB/TrEMBL;Acc:L8HC88]	0.162776233	0.019991833	-0.1427844	0.048609125
3h	ACA1_087610/clean/scf7180000084639:73807-73942:+	ACA1_087610	Phospholipase D, putative [Source:UniProtKB/TrEMBL;Acc:L8GVI1]	0.249200667	0.0306981	-0.218502567	0.04293732
3h	ACA1_378490/clean/scf7180000084711:231689-231810:-	ACA1_378490	hypothetical protein	0.857872333	0.266296333	-0.591576	1.12E-06
6h	ACA1_229570/clean/scf7180000084761:280946-281030:-	ACA1_229570	Oxidoreductase, short chain dehydrogenase/reductase superfamily protein [Source:UniProtKB/TrEMBL;Acc:L8H814]	0.019550507	0.952399	0.932848493	9.27E-25
6h	ACA1_177720/clean/scf7180000084743:113679-113788:-	ACA1_177720	PARP-type domain-containing protein [Source:UniProtKB/TrEMBL;Acc:L8GU65]	0.011684629	0.930363333	0.918678704	3.35E-24
6h	ACA1_034030/clean/scf7180000084561:8353-8442:+	ACA1_034030	RNA recognition motif domain containing protein [Source:UniProtKB/TrEMBL;Acc:L8GTW3]	0.000149268	0.901455	0.901305732	1.40E-17
6h	ACA1_053850/clean/scf7180000084594:334939-335036:+	ACA1_053850	ATP11 protein [Source:UniProtKB/TrEMBL;Acc:L8H868]	0.1022162	1	0.8977838	7.13E-05
6h	ACA1_129340/clean/scf7180000084698:5810-5876:+	ACA1_129340	Methyltransf_11 domain-containing protein [Source:UniProtKB/TrEMBL;Acc:L8GNW8]	0.001972387	0.892070667	0.89009828	9.46E-16
6h	ACA1_230520/clean/scf7180000084761:371650-371715:-	ACA1_230520	Hypothetical protein	0.006728557	0.887614333	0.880885777	4.76E-18
6h	ACA1_229570/clean/scf7180000084761:280817-280885:-	ACA1_229570	Oxidoreductase, short chain dehydrogenase/reductase superfamily protein [Source:UniProtKB/TrEMBL;Acc:L8H814]	0.003238689	0.882366333	0.879127644	9.57E-15
6h	ACA1_063140/clean/scf7180000084599:1341052-1341140:-	ACA1_063140	MFS domain-containing protein [Source:UniProtKB/TrEMBL;Acc:L8GX17]	0	0.873450333	0.873450333	4.53E-07
6h	ACA1_072500/clean/scf7180000084603:1218019-1218086:-	ACA1_072500	F-box domain-containing protein [Source:UniProtKB/TrEMBL;Acc:L8HFX9]	0	0.872999	0.872999	7.28E-10
6h	ACA1_237890/clean/scf7180000084763:39673-39763:+	ACA1_237890	SCP2 sterol transfer family protein [Source:UniProtKB/TrEMBL;Acc:L8GM48]	0.098897233	0.968738333	0.8698411	2.28E-46
6h	ACA1_072500/clean/scf7180000084603:1217354-1217428:-	ACA1_072500	F-box domain-containing protein [Source:UniProtKB/TrEMBL;Acc:L8HFX9]	0.009165467	0.870984333	0.861818867	3.58E-08
6h	ACA1_261400/clean/scf7180000084769:661523-661597:-	ACA1_261400	Serine/threonine protein kinase, putative [Source:UniProtKB/TrEMBL;Acc:L8GF66]	0	0.861669	0.861669	1.59E-07
6h	ACA1_261400/clean/scf7180000084769:661324-661387:-	ACA1_261400	Serine/threonine protein kinase, putative [Source:UniProtKB/TrEMBL;Acc:L8GF66]	0	0.860816333	0.860816333	2.79E-07
6h	ACA1_159850/clean/scf7180000084724:393288-393380:+	ACA1_159850	hypothetical protein	0.006938217	0.864249667	0.85731145	9.30E-21
6h	ACA1_129340/clean/scf7180000084698:6522-6619:+	ACA1_129340	Methyltransf_11 domain-containing protein [Source:UniProtKB/TrEMBL;Acc:L8GNW8]	0.00280806	0.857914333	0.855106273	2.32E-12
6h	ACA1_065190/clean/scf7180000084599:1813313-1813401:-	ACA1_065190	RBR-type E3 ubiquitin transferase [Source:UniProtKB/TrEMBL;Acc:L8GXL6]	0.000710733	0.850953	0.850242267	5.93E-13
6h	ACA1_000590/clean/scf7180000082784:12407-12499:+	ACA1_000590	hypothetical protein	0	0.850194333	0.850194333	0.001528935
6h	ACA1_175740/clean/scf7180000084741:1047634-1047697:+	ACA1_175740	hypothetical protein	0.0417984	0.889685	0.8478866	1.13E-06
6h	ACA1_323520/clean/scf7180000084468:41224-41298:-	ACA1_323520	Inosineuridine preferring nucleoside hydrolase [Source:UniProtKB/TrEMBL;Acc:L8H334]	0.006535933	0.852868667	0.846332733	3.54E-16
6h	ACA1_320790/clean/scf7180000084416:23487-23555:-	ACA1_320790	RUN domain-containing protein [Source:UniProtKB/TrEMBL;Acc:L8GTR9]	0.025329797	0.871199	0.845869203	2.70E-36
6h	ACA1_383660/clean/scf7180000084721:240604-240676:+	ACA1_383660	tRNA (guanine-N(7)-)-methyltransferase non-catalytic subunit [Source:UniProtKB/TrEMBL;Acc:L8GW53]	0.00970683	0.851338	0.84163117	9.19E-15
6h	ACA1_071170/clean/scf7180000084603:879314-879414:+	ACA1_071170	RhoGAP domain containing protein [Source:UniProtKB/TrEMBL;Acc:L8HGC8]	0	0.839864333	0.839864333	2.38E-14
6h	ACA1_025540/clean/scf7180000084448:7569-7646:+	ACA1_025540	hypothetical protein	0.023029633	0.861903333	0.8388737	4.57E-19
6h	ACA1_012980/clean/scf7180000083742:16382-16462:-	ACA1_012980	Endo/exonuclease/phosphatase domain-containing protein [Source:UniProtKB/TrEMBL;Acc:L8H4W5]	0.010101	0.848635	0.838534	6.82E-11
6h	ACA1_014250/clean/scf7180000083757:21236-21460:-	ACA1_014250	Cytochrome b5 heme-binding domain-containing protein [Source:UniProtKB/TrEMBL;Acc:L8GIZ1]	0.00750605	0.845215	0.83770895	1.22E-33
6h	ACA1_142590/clean/scf7180000084708:191916-192020:+	ACA1_142590	unspecified product	0.034775	0.866729333	0.831954333	2.70E-24
6h	ACA1_382630/clean/scf7180000084721:88438-88554:-	ACA1_382630	LBP / BPI / CETP family, Cterminal domain containing protein [Source:UniProtKB/TrEMBL;Acc:L8GVZ7]	0.0687792	0.900042667	0.831263467	1.38E-40
6h	ACA1_074840/clean/scf7180000084608:99888-99963:+	ACA1_074840	Lecithin:cholesterol acyltransferase [Source:UniProtKB/TrEMBL;Acc:L8HF90]	0.002368267	0.833295667	0.8309274	5.86E-15
6h	ACA1_321160/clean/scf7180000084437:1881-1967:-	ACA1_321160	FAD-binding FR-type domain-containing protein [Source:UniProtKB/TrEMBL;Acc:L8GGN4]	0.127236333	0.957283333	0.830047	4.84E-09
6h	ACA1_071580/clean/scf7180000084603:997734-997799:-	ACA1_071580	GOLD domain-containing protein [Source:UniProtKB/TrEMBL;Acc:L8HGH0]	0.0051176	0.834960333	0.829842733	1.51E-17
6h	ACA1_389110/clean/scf7180000084733:198465-198568:+	ACA1_389110	hypothetical protein	0.014336903	0.842054333	0.82771743	1.57E-14
6h	ACA1_271330/clean/scf7180000084771:21289-21464:+	ACA1_271330	hypothetical protein	0.031654667	0.857497667	0.825843	7.58E-27
6h	ACA1_224860/clean/scf7180000084760:258809-258888:+	ACA1_224860	Myosin head (Motor domain) domain containing protein [Source:UniProtKB/TrEMBL;Acc:L8GSY9]	0	0.825536	0.825536	0.000105921
6h	ACA1_378010/clean/scf7180000084711:164303-164420:+	ACA1_378010	JmjC domain-containing protein [Source:UniProtKB/TrEMBL;Acc:L8GRT4]	0.042546707	0.866166	0.823619293	5.12E-18
6h	ACA1_072500/clean/scf7180000084603:1218524-1218611:-	ACA1_072500	F-box domain-containing protein [Source:UniProtKB/TrEMBL;Acc:L8HFX9]	0.019607833	0.835985	0.816377167	1.59E-10
6h	ACA1_277500/clean/scf7180000084776:65335-65391:-	ACA1_277500	DBR1 domain-containing protein [Source:UniProtKB/TrEMBL;Acc:L8H8N5]	0.025641033	0.841767667	0.816126633	1.77E-07
6h	ACA1_278900/clean/scf7180000084776:271265-271352:+	ACA1_278900	ANK_REP_REGION domain-containing protein [Source:UniProtKB/TrEMBL;Acc:L8H5W6]	0.0040421	0.819626333	0.815584233	1.21E-14
6h	ACA1_267000/clean/scf7180000084770:772955-773031:+	ACA1_267000	Camp-dependent protein kinase catalytic subunit family protein [Source:UniProtKB/TrEMBL;Acc:L8H3Y8]	0.009126407	0.823499333	0.814372927	4.31E-15
6h	ACA1_074100/clean/scf7180000084605:23993-24088:+	ACA1_074100	Fungal Zn(2)-Cys(6) binuclear cluster domain containing protein [Source:UniProtKB/TrEMBL;Acc:L8HLU8]	0.037093567	0.851168667	0.8140751	9.46E-25
6h	ACA1_243850/clean/scf7180000084763:363141-363235:+	ACA1_243850	Ras-GAP domain-containing protein [Source:UniProtKB/TrEMBL;Acc:L8GJZ5]	0	0.813454333	0.813454333	2.59E-06
6h	ACA1_199500/clean/scf7180000084753:247986-248059:+	ACA1_199500	1acylglycerophosphocholine O-acyltransferase 1, putative [Source:UniProtKB/TrEMBL;Acc:L8H4L3]	0.001171647	0.814381667	0.81321002	2.23E-10
6h	ACA1_129340/clean/scf7180000084698:6286-6409:+	ACA1_129340	Methyltransf_11 domain-containing protein [Source:UniProtKB/TrEMBL;Acc:L8GNW8]	0.009933	0.821271667	0.811338667	3.28E-11
6h	ACA1_048880/clean/scf7180000084583:10024-10100:+	ACA1_048880	LIM zinc-binding domain-containing protein [Source:UniProtKB/TrEMBL;Acc:L8HKD0]	0.04851	0.857142	0.808632	4.90E-40
6h	ACA1_127640/clean/scf7180000084696:40081-40159:+	ACA1_127640	GP-PDE domain-containing protein [Source:UniProtKB/TrEMBL;Acc:L8GEW1]	0.0205704	0.828417667	0.807847267	4.22E-12
6h	ACA1_113550/clean/scf7180000084669:315629-315811:-	ACA1_113550	hypothetical protein	0.0776917	0.884806333	0.807114633	2.04E-29
6h	ACA1_191840/clean/scf7180000084749:51292-51356:+	ACA1_191840	Serine/threonine-protein phosphatase [Source:UniProtKB/TrEMBL;Acc:L8GNP7]	0.0335441	0.840630667	0.807086567	1.91E-21
6h	ACA1_289710/clean/scf7180000084777:446308-446373:-	ACA1_289710	hypothetical protein	0.020961667	0.824706667	0.803745	6.41E-10
6h	ACA1_257320/clean/scf7180000084769:150623-150712:-	ACA1_257320	PNP_phzG_C domain-containing protein [Source:UniProtKB/TrEMBL;Acc:L8GEX9]	0.056868367	0.859036	0.802167633	1.75E-17
6h	ACA1_245630/clean/scf7180000084763:504250-504367:+	ACA1_245630	E3 ubiquitin-protein ligase [Source:UniProtKB/TrEMBL;Acc:L8GML6]	0	0.801776	0.801776	6.84E-05
6h	ACA1_045320/clean/scf7180000084576:115861-115935:+	ACA1_045320	hypothetical protein	0	0.798905	0.798905	0.020518652
6h	ACA1_069640/clean/scf7180000084603:498628-498734:-	ACA1_069640	Thioesterase family domain containing protein [Source:UniProtKB/TrEMBL;Acc:L8HCV4]	0	0.798766	0.798766	8.84E-11
6h	ACA1_142610/clean/scf7180000084708:200466-200547:-	ACA1_142610	Myblike DNA-binding domain containing protein [Source:UniProtKB/TrEMBL;Acc:L8HCT3]	0.007529367	0.805535667	0.7980063	5.58E-12
6h	ACA1_129340/clean/scf7180000084698:6027-6113:+	ACA1_129340	Methyltransf_11 domain-containing protein [Source:UniProtKB/TrEMBL;Acc:L8GNW8]	0.00172554	0.796896667	0.795171127	5.47E-13
6h	ACA1_384720/clean/scf7180000084730:92713-92807:+	ACA1_384720	Vault_2 domain-containing protein [Source:UniProtKB/TrEMBL;Acc:L8H933]	0.150511667	0.944748	0.794236333	2.15E-16
6h	ACA1_142610/clean/scf7180000084708:199733-199829:-	ACA1_142610	Myblike DNA-binding domain containing protein [Source:UniProtKB/TrEMBL;Acc:L8HCT3]	0.010579167	0.804637667	0.7940585	3.15E-12
6h	ACA1_261400/clean/scf7180000084769:660250-660345:-	ACA1_261400	Serine/threonine protein kinase, putative [Source:UniProtKB/TrEMBL;Acc:L8GF66]	0.018518533	0.811272667	0.792754133	3.88E-05
6h	ACA1_067470/clean/scf7180000084601:34845-34913:-	ACA1_067470	hypothetical protein	0.0372706	0.829834	0.7925634	3.34E-29
6h	ACA1_048140/clean/scf7180000084582:32257-32373:+	ACA1_048140	Membrane protein (Cytomegalovirus gHreceptor) family [Source:UniProtKB/TrEMBL;Acc:L8GCZ0]	0.183762667	0.975837333	0.792074667	5.68E-07
6h	ACA1_053360/clean/scf7180000084594:268950-269094:+	ACA1_053360	RING-type domain-containing protein [Source:UniProtKB/TrEMBL;Acc:L8H7D5]	0.031099033	0.823040667	0.791941633	1.56E-14
6h	ACA1_101530/clean/scf7180000084649:87651-87736:-	ACA1_101530	6phosphogluconate dehydrogenase NAD-binding domain containing protein [Source:UniProtKB/TrEMBL;Acc:L8GGL6]	0.0631362	0.854695333	0.791559133	3.00E-23
6h	ACA1_383660/clean/scf7180000084721:239911-240002:+	ACA1_383660	tRNA (guanine-N(7)-)-methyltransferase non-catalytic subunit [Source:UniProtKB/TrEMBL;Acc:L8GW53]	0	0.791279333	0.791279333	6.08E-15
6h	ACA1_269670/clean/scf7180000084770:1010966-1011078:-	ACA1_269670	Tetratricopeptide repeat domain containing protein [Source:UniProtKB/TrEMBL;Acc:L8H2T8]	0.013820102	0.804031333	0.790211231	2.03E-11
6h	ACA1_144440/clean/scf7180000084709:244650-244859:+	ACA1_144440	SNF2 family Nterminal domain containing protein [Source:UniProtKB/TrEMBL;Acc:L8HFN1]	0.012925703	0.802554333	0.78962863	5.52E-40
6h	ACA1_014250/clean/scf7180000083757:21059-21207:-	ACA1_014250	Cytochrome b5 heme-binding domain-containing protein [Source:UniProtKB/TrEMBL;Acc:L8GIZ1]	0.01063075	0.799639667	0.789008917	4.72E-33
6h	ACA1_117020/clean/scf7180000084669:894175-894252:+	ACA1_117020	Carboxypeptidase [Source:UniProtKB/TrEMBL;Acc:L8H3Z1]	0.005633423	0.792228	0.786594577	1.59E-20
6h	ACA1_396120/clean/scf7180000084759:23911-24033:-	ACA1_396120	Rasrelated protein Rap-1b, putative [Source:UniProtKB/TrEMBL;Acc:L8HC60]	0.021497467	0.805707	0.784209533	1.71E-19
6h	ACA1_255760/clean/scf7180000084768:563824-563934:+	ACA1_255760	Acid phosphatase PHOa, putative [Source:UniProtKB/TrEMBL;Acc:L8HB67]	0.0647849	0.848800667	0.784015767	1.01E-43
6h	ACA1_020870/clean/scf7180000084095:29634-29720:+	ACA1_020870	Large conductance mechanosensitive channel protein [Source:UniProtKB/TrEMBL;Acc:L8GWH9]	0.102476967	0.885989667	0.7835127	3.51E-28
6h	ACA1_189830/clean/scf7180000084748:97740-97796:+	ACA1_189830	Partner of sld five, psf2 protein [Source:UniProtKB/TrEMBL;Acc:L8GE47]	0.005096833	0.786935667	0.781838833	7.49E-12
6h	ACA1_046600/clean/scf7180000084577:150912-151000:+	ACA1_046600	FSH1 domain-containing protein [Source:UniProtKB/TrEMBL;Acc:L8H9S5]	0.218454333	1	0.781545667	2.46E-08
6h	ACA1_150950/clean/scf7180000084716:32882-33021:+	ACA1_150950	Exocyst complex component Sec6 [Source:UniProtKB/TrEMBL;Acc:L8H215]	0.000221191	0.781396667	0.781175476	4.44E-15
6h	ACA1_265380/clean/scf7180000084770:455635-455721:-	ACA1_265380	Phosphatidylinositol glycan anchor biosynthesis, class S, putative [Source:UniProtKB/TrEMBL;Acc:L8H3M4]	0.0162782	0.797197333	0.780919133	9.86E-15
6h	ACA1_320790/clean/scf7180000084416:23173-23251:-	ACA1_320790	RUN domain-containing protein [Source:UniProtKB/TrEMBL;Acc:L8GTR9]	0.0078314	0.787476333	0.779644933	3.86E-23
6h	ACA1_117020/clean/scf7180000084669:892765-892859:+	ACA1_117020	Carboxypeptidase [Source:UniProtKB/TrEMBL;Acc:L8H3Z1]	0.00406246	0.783632667	0.779570207	1.62E-16
6h	ACA1_359270/clean/scf7180000084742:70646-70701:-	ACA1_359270	RAB family member (Rab7), putative [Source:UniProtKB/TrEMBL;Acc:L8GLL4]	0.044611733	0.823848667	0.779236933	1.10E-22
6h	ACA1_106950/clean/scf7180000084660:157034-157210:+	ACA1_106950	PH domain containing protein [Source:UniProtKB/TrEMBL;Acc:L8GNB1]	0.0799786	0.859213333	0.779234733	8.89E-05
6h	ACA1_092590/clean/scf7180000084645:361504-361580:-	ACA1_092590	Leucine rich repeat domain containing protein [Source:UniProtKB/TrEMBL;Acc:L8GKK8]	0.037037067	0.816114333	0.779077267	2.02E-08
6h	ACA1_390470/clean/scf7180000084740:49070-49149:+	ACA1_390470	Initiator tRNA phosphoribosyl transferase [Source:UniProtKB/TrEMBL;Acc:L8GQ00]	0.000494743	0.778521667	0.778026923	1.75E-11
6h	ACA1_248200/clean/scf7180000084766:24344-24412:+	ACA1_248200	APH domain-containing protein [Source:UniProtKB/TrEMBL;Acc:L8H0B8]	0	0.777331667	0.777331667	0.000341704
6h	ACA1_131460/clean/scf7180000084699:63136-63212:+	ACA1_131460	MFS domain-containing protein [Source:UniProtKB/TrEMBL;Acc:L8GN39]	0	0.776326	0.776326	1.47E-11
6h	ACA1_107530/clean/scf7180000084660:216036-216160:-	ACA1_107530	UBP-type domain-containing protein [Source:UniProtKB/TrEMBL;Acc:L8GMW8]	0.010640683	0.785797333	0.77515665	7.06E-43
6h	ACA1_129350/clean/scf7180000084698:8929-9053:+	ACA1_129350	Fatty acid hydroxylase domain-containing protein [Source:UniProtKB/TrEMBL;Acc:L8GPA5]	0.00452147	0.779371333	0.774849863	1.13E-15
6h	ACA1_108830/clean/scf7180000084661:93343-93434:+	ACA1_108830	Terpene cyclase/mutase family member [Source:UniProtKB/TrEMBL;Acc:L8GD76]	0.006360407	0.781207333	0.774846927	3.95E-22
6h	ACA1_261230/clean/scf7180000084769:609730-609795:-	ACA1_261230	hypothetical protein	0.055555667	0.830220333	0.774664667	2.18E-05
6h	ACA1_103150/clean/scf7180000084653:7967-8040:+	ACA1_103150	hypothetical protein	0.011667893	0.785991	0.774323107	1.15E-27
6h	ACA1_063690/clean/scf7180000084599:1475674-1475729:-	ACA1_063690	Amidase domain-containing protein [Source:UniProtKB/TrEMBL;Acc:L8GXA6]	0.104897367	0.879042	0.774144633	7.94E-16
6h	ACA1_245500/clean/scf7180000084763:496605-496699:+	ACA1_245500	Fe2OG dioxygenase domain-containing protein [Source:UniProtKB/TrEMBL;Acc:L8GL61]	0.000270343	0.773976333	0.77370599	3.84E-13
6h	ACA1_255030/clean/scf7180000084768:453728-453822:-	ACA1_255030	hypothetical protein	0.020944757	0.794377333	0.773432577	9.71E-12
6h	ACA1_177100/clean/scf7180000084743:31020-31132:-	ACA1_177100	Protein kinase domain containing protein [Source:UniProtKB/TrEMBL;Acc:L8GTA4]	0	0.771828	0.771828	5.28E-10
6h	ACA1_282440/clean/scf7180000084776:680878-680960:+	ACA1_282440	hypothetical protein	0.01253596	0.783875333	0.771339373	1.59E-10
6h	ACA1_153240/clean/scf7180000084720:93630-93727:-	ACA1_153240	hypothetical protein	0	0.768319	0.768319	2.47E-06
6h	ACA1_295300/clean/scf7180000084777:1101617-1101773:-	ACA1_295300	Calponin domain containing protein [Source:UniProtKB/TrEMBL;Acc:L8HJQ6]	0.021363127	0.788529667	0.76716654	2.68E-18
6h	ACA1_309340/clean/scf7180000083758:6222-6285:+	ACA1_309340	Protein tyrosine phosphataselike protein, PTPLA [Source:UniProtKB/TrEMBL;Acc:L8GR24]	0.034955233	0.800918	0.765962767	1.41E-22
6h	ACA1_184090/clean/scf7180000084745:556617-556723:-	ACA1_184090	hypothetical protein	0.042957033	0.808469667	0.765512633	7.25E-20
6h	ACA1_027000/clean/scf7180000084464:15453-15542:-	ACA1_027000	SCP family extracellular, putative [Source:UniProtKB/TrEMBL;Acc:L8GHL6]	0.022774303	0.788253333	0.76547903	1.35E-26
6h	ACA1_259480/clean/scf7180000084769:349054-349195:-	ACA1_259480	EnoylCoA hydratase/isomerase [Source:UniProtKB/TrEMBL;Acc:L8GFL3]	0.210394	0.975187333	0.764793333	1.43E-09
6h	ACA1_101080/clean/scf7180000084649:35292-35381:-	ACA1_101080	Purple acid phosphatase [Source:UniProtKB/TrEMBL;Acc:L8GGG9]	0.003849474	0.768558333	0.764708859	2.79E-23
6h	ACA1_255900/clean/scf7180000084768:579178-579284:+	ACA1_255900	DEP domain-containing protein [Source:UniProtKB/TrEMBL;Acc:L8HA90]	0.032050233	0.796543667	0.764493433	1.63E-39
6h	ACA1_167050/clean/scf7180000084736:63243-63304:-	ACA1_167050	Ras subfamily protein [Source:UniProtKB/TrEMBL;Acc:L8H0S4]	0	0.763777667	0.763777667	1.65E-10
6h	ACA1_131460/clean/scf7180000084699:62869-62961:+	ACA1_131460	MFS domain-containing protein [Source:UniProtKB/TrEMBL;Acc:L8GN39]	0.017543867	0.781250333	0.763706467	5.21E-09
6h	ACA1_183740/clean/scf7180000084745:494022-494084:+	ACA1_183740	hypothetical protein	0.01765994	0.779119667	0.761459727	5.01E-11
6h	ACA1_183210/clean/scf7180000084745:357210-357300:-	ACA1_183210	Thioredoxin domain-containing protein [Source:UniProtKB/TrEMBL;Acc:L8H812]	0	0.761247	0.761247	0.007858091
6h	ACA1_106710/clean/scf7180000084660:106517-106629:+	ACA1_106710	TPR_REGION domain-containing protein [Source:UniProtKB/TrEMBL;Acc:L8GMK9]	0.035059633	0.795914	0.760854367	2.31E-25
6h	ACA1_331590/clean/scf7180000084578:8031-8143:+	ACA1_331590	ATPdependent DNA helicase, RecQ subfamily protein [Source:UniProtKB/TrEMBL;Acc:L8HBM3]	0.020136067	0.780107667	0.7599716	6.11E-13

6h	ACA1_390470/clean/scf7180000084740:49705-49783:+	ACA1_390470	Initiator tRNA phosphoribosyl transferase [Source:UniProtKB/TrEMBL;Acc:L8GQ00]	0.018518533	0.778281667	0.759763133	2.42E-09
6h	ACA1_098230/clean/scf7180000084645:1433747-1433832:-	ACA1_098230	Leucine rich repeat domain containing protein [Source:UniProtKB/TrEMBL;Acc:L8GLR4]	0	0.759575	0.759575	7.11E-09
6h	ACA1_185700/clean/scf7180000084746:114487-114556:+	ACA1_185700	EF hand domain containing protein [Source:UniProtKB/TrEMBL;Acc:L8H786]	0.0368736	0.796098667	0.759225067	5.15E-33
6h	ACA1_381100/clean/scf7180000084712:144628-144684:+	ACA1_381100	hypothetical protein	0.160067667	0.918497667	0.75843	3.50E-12
6h	ACA1_043310/clean/scf7180000084573:162450-162563:-	ACA1_043310	Rieske domain-containing protein [Source:UniProtKB/TrEMBL;Acc:L8GV77]	0.056219367	0.814412	0.758192633	1.25E-11
6h	ACA1_150950/clean/scf7180000084716:32541-32641:+	ACA1_150950	Exocyst complex component Sec6 [Source:UniProtKB/TrEMBL;Acc:L8H215]	0.006222017	0.764316	0.758093983	4.12E-12
6h	ACA1_382580/clean/scf7180000084721:77714-77840:+	ACA1_382580	LBP / BPI / CETP family, Cterminal domain containing protein [Source:UniProtKB/TrEMBL;Acc:L8GVZ1]	0.0453612	0.801103667	0.755742467	1.90E-32
6h	ACA1_329650/clean/scf7180000084562:37997-38061:-	ACA1_329650	hypothetical protein	0.046707047	0.801934333	0.755227287	1.63E-24
6h	ACA1_323310/clean/scf7180000084468:853-932:+	ACA1_323310	hypothetical protein	0.173545333	0.927050667	0.753505333	7.78E-20
6h	ACA1_383660/clean/scf7180000084721:239758-239835:+	ACA1_383660	tRNA (guanine-N(7)-)-methyltransferase non-catalytic subunit [Source:UniProtKB/TrEMBL;Acc:L8GW53]	0.0540741	0.807563667	0.753489567	1.53E-19
6h	ACA1_289690/clean/scf7180000084777:444489-444568:-	ACA1_289690	hypothetical protein	0	0.753385667	0.753385667	2.05E-14
6h	ACA1_216320/clean/scf7180000084758:467384-467533:+	ACA1_216320	Homogentisate 1,2dioxygenase [Source:UniProtKB/TrEMBL;Acc:L8GQ83]	0.01504124	0.767207	0.75216576	1.06E-27
6h	ACA1_298630/clean/scf7180000082992:2242-2325:-	ACA1_298630	Vacuolar protein sorting 16, putative [Source:UniProtKB/TrEMBL;Acc:L8H081]	0.016736483	0.768457333	0.75172085	4.79E-23
6h	ACA1_306850/clean/scf7180000083668:20973-21053:+	ACA1_306850	RanBPMrelated, putative [Source:UniProtKB/TrEMBL;Acc:L8GNY2]	0.065399467	0.815672667	0.7502732	3.77E-17
6h	ACA1_359690/clean/scf7180000084622:3799-3915:-	ACA1_359690	Rab-GAP TBC domain-containing protein [Source:UniProtKB/TrEMBL;Acc:L8HBR2]	0.011387483	0.761469333	0.75008185	3.85E-19
6h	ACA1_180600/clean/scf7180000084744:62961-63079:+	ACA1_180600	hypothetical protein	0.021894067	0.770907667	0.7490136	4.13E-46
6h	ACA1_349640/clean/scf7180000084690:22625-22703:+	ACA1_349640	HEAT repeat domain containing protein [Source:UniProtKB/TrEMBL;Acc:L8GKB3]	0.021920533	0.770145667	0.748225133	1.98E-13
6h	ACA1_360980/clean/scf7180000084622:214115-214203:+	ACA1_360980	hypothetical protein	0.035087667	0.78254	0.747452333	2.95E-08
6h	ACA1_167050/clean/scf7180000084736:63077-63163:-	ACA1_167050	Ras subfamily protein [Source:UniProtKB/TrEMBL;Acc:L8H0S4]	0.103868667	0.851036667	0.747168	1.95E-12
6h	ACA1_040210/clean/scf7180000084571:13868-13936:+	ACA1_040210	Mitochondrial uncoupling protein [Source:UniProtKB/TrEMBL;Acc:L8GGI9]	0.016008133	0.762804667	0.746796533	3.66E-25
6h	ACA1_063560/clean/scf7180000084599:1449108-1449242:+	ACA1_063560	hypothetical protein	0.018984073	0.765267	0.746282927	5.20E-33
6h	ACA1_106350/clean/scf7180000084660:92597-92691:+	ACA1_106350	F-box domain-containing protein [Source:UniProtKB/TrEMBL;Acc:L8GN85]	0.169368667	0.914787	0.745418333	1.84E-05
6h	ACA1_131460/clean/scf7180000084699:63399-63507:+	ACA1_131460	MFS domain-containing protein [Source:UniProtKB/TrEMBL;Acc:L8GN39]	0.0111111	0.755773	0.7446619	2.67E-09
6h	ACA1_224400/clean/scf7180000084760:192704-192861:+	ACA1_224400	F_actin_bund_C domain-containing protein [Source:UniProtKB/TrEMBL;Acc:L8GVG0]	0.004988973	0.749204333	0.74421536	8.77E-46
6h	ACA1_020780/clean/scf7180000084095:7835-7936:-	ACA1_020780	AMP-binding domain-containing protein [Source:UniProtKB/TrEMBL;Acc:L8GU07]	0.011449267	0.755469333	0.744020067	7.08E-17
6h	ACA1_372660/clean/scf7180000084679:40513-40594:-	ACA1_372660	hypothetical protein	0	0.743770333	0.743770333	6.54E-08
6h	ACA1_082750/clean/scf7180000084625:9008-9105:+	ACA1_082750	N-acetyltransferase domain-containing protein [Source:UniProtKB/TrEMBL;Acc:L8GSP4]	0.020863923	0.763761	0.742897077	9.95E-21
6h	ACA1_261400/clean/scf7180000084769:659762-659870:-	ACA1_261400	Serine/threonine protein kinase, putative [Source:UniProtKB/TrEMBL;Acc:L8GF66]	0.111111	0.853531333	0.742420333	1.51E-06
6h	ACA1_140730/clean/scf7180000084707:109513-109699:-	ACA1_140730	Kelch repeat protein [Source:UniProtKB/TrEMBL;Acc:L8GGS1]	0.052951833	0.794104	0.741152167	3.87E-17
6h	ACA1_138470/clean/scf7180000084703:146878-146960:+	ACA1_138470	Ribosomal_L11 domain-containing protein [Source:UniProtKB/TrEMBL;Acc:L8GZJ5]	0	0.74091	0.74091	7.37E-11
6h	ACA1_234140/clean/scf7180000084762:206774-206965:+	ACA1_234140	DAO domain-containing protein [Source:UniProtKB/TrEMBL;Acc:L8H3H7]	0.027710067	0.768404333	0.740694267	3.02E-23
6h	ACA1_282700/clean/scf7180000084776:719212-719286:+	ACA1_282700	EF hand domain containing protein [Source:UniProtKB/TrEMBL;Acc:L8H6I5]	0.035398433	0.775735	0.740336567	5.14E-28
6h	ACA1_077020/clean/scf7180000084612:215408-215503:+	ACA1_077020	Propionate-CoA synthetase and ligase [Source:UniProtKB/TrEMBL;Acc:L8GLY7]	0.171013667	0.911276	0.740262333	2.90E-07
6h	ACA1_323370/clean/scf7180000084468:12780-12836:-	ACA1_323370	SHSP domain-containing protein [Source:UniProtKB/TrEMBL;Acc:L8H320]	0.047619	0.787476	0.739857	1.38E-05
6h	ACA1_028670/clean/scf7180000084473:5229-5378:+	ACA1_028670	Serine/threonine-protein phosphatase [Source:UniProtKB/TrEMBL;Acc:L8GWF5]	0.00320494	0.742785667	0.739580727	1.80E-45
6h	ACA1_144930/clean/scf7180000084710:41854-41980:-	ACA1_144930	N-acetyltransferase domain-containing protein [Source:UniProtKB/TrEMBL;Acc:L8GCT6]	0.121189	0.860416333	0.739227333	3.86E-23
6h	ACA1_274900/clean/scf7180000084773:135398-135494:-	ACA1_274900	2,3diketo-5-methylthio-1-phosphopentane phosphatase subfamily protein [Source:UniProtKB/TrEMBL;Acc:L8GFW5]	0.019678733	0.757852667	0.738173933	4.25E-23
6h	ACA1_309630/clean/scf7180000083763:5485-5601:+	ACA1_309630	DUF1768 domain-containing protein [Source:UniProtKB/TrEMBL;Acc:L8GIA7]	0.107483833	0.844606667	0.737122833	5.51E-16
6h	ACA1_034490/clean/scf7180000084563:56739-56819:-	ACA1_034490	hypothetical protein	0.009518187	0.746614333	0.737096147	8.29E-13
6h	ACA1_145180/clean/scf7180000084710:54054-54133:-	ACA1_145180	F-box domain-containing protein [Source:UniProtKB/TrEMBL;Acc:L8GCU3]	0.048651833	0.784624	0.735972167	2.77E-13
6h	ACA1_172250/clean/scf7180000084741:286136-286219:-	ACA1_172250	Leucine rich repeat domain containing protein [Source:UniProtKB/TrEMBL;Acc:L8HHK1]	0.077040667	0.812817667	0.735777	4.36E-06
6h	ACA1_175700/clean/scf7180000084741:1038479-1038550:-	ACA1_175700	Frizzled/Smoothened family membrane region protein [Source:UniProtKB/TrEMBL;Acc:L8HH45]	0.00808425	0.743385667	0.735301417	6.76E-10
6h	ACA1_120250/clean/scf7180000084675:11997-12066:+	ACA1_120250	Doublecortin domain-containing protein [Source:UniProtKB/TrEMBL;Acc:L8GVW9]	0.025953067	0.761232667	0.7352796	6.51E-26
6h	ACA1_287360/clean/scf7180000084777:16846-17004:-	ACA1_287360	Acid phosphatase [Source:UniProtKB/TrEMBL;Acc:L8HIM1]	0.124379167	0.859606333	0.735227167	1.18E-18
6h	ACA1_156720/clean/scf7180000084723:58112-58195:+	ACA1_156720	INTRADIOL_DIOXYGENAS domain-containing protein [Source:UniProtKB/TrEMBL;Acc:L8GI57]	0.00130841	0.736115	0.73480659	1.30E-13
6h	ACA1_264550/clean/scf7180000084770:319403-319497:+	ACA1_264550	Cys/Met metabolism PLPdependent enzyme superfamily protein [Source:UniProtKB/TrEMBL;Acc:L8H496]	0.022680033	0.757292333	0.7346123	5.48E-21
6h	ACA1_117260/clean/scf7180000084669:964542-964615:+	ACA1_117260	hypothetical protein	0.124135293	0.858553667	0.734418373	1.61E-09
6h	ACA1_171870/clean/scf7180000084741:182777-183056:-	ACA1_171870	Leucine rich repeat domain containing protein [Source:UniProtKB/TrEMBL;Acc:L8HIV4]	0.04679618	0.780456667	0.733660487	5.10E-10
6h	ACA1_287590/clean/scf7180000084777:41331-41396:+	ACA1_287590	hypothetical protein	0.0172811	0.749429333	0.732148233	1.22E-13
6h	ACA1_282540/clean/scf7180000084776:706575-706665:-	ACA1_282540	Ribonuclease [Source:UniProtKB/TrEMBL;Acc:L8H8Q0]	0.01874172	0.750154667	0.731412947	9.20E-16
6h	ACA1_093800/clean/scf7180000084645:648068-648195:-	ACA1_093800	PXMP2/4 family protein 3, putative [Source:UniProtKB/TrEMBL;Acc:L8GJ99]	0.002705047	0.734040667	0.73133562	2.23E-27
6h	ACA1_183740/clean/scf7180000084745:493889-493951:+	ACA1_183740	hypothetical protein	0.192657333	0.923731667	0.731074333	1.59E-17
6h	ACA1_105970/clean/scf7180000084660:40538-40615:-	ACA1_105970	PhytanoylCoA dioxygenase family protein [Source:UniProtKB/TrEMBL;Acc:L8GQ69]	0.004071397	0.734577333	0.730505937	2.08E-21
6h	ACA1_103750/clean/scf7180000084653:52237-52308:+	ACA1_103750	Fascin domain-containing protein [Source:UniProtKB/TrEMBL;Acc:L8GD37]	0.015133167	0.745586	0.730452833	3.18E-09
6h	ACA1_271350/clean/scf7180000084771:24780-25011:+	ACA1_271350	hypothetical protein	0.160384	0.890712333	0.730328333	2.06E-18
6h	ACA1_048880/clean/scf7180000084583:9878-9936:+	ACA1_048880	LIM zinc-binding domain-containing protein [Source:UniProtKB/TrEMBL;Acc:L8HKD0]	0.0783754	0.808700667	0.730325267	4.34E-30
6h	ACA1_154690/clean/scf7180000084722:78274-78372:+	ACA1_154690	unspecified product	0.005728343	0.735446667	0.729718323	4.95E-15
6h	ACA1_359020/clean/scf7180000084742:51981-52065:+	ACA1_359020	HpcH/HpaI aldolase/citrate lyase subfamily protein [Source:UniProtKB/TrEMBL;Acc:L8GLJ7]	0.000898473	0.730182333	0.72928386	3.34E-14
6h	ACA1_071430/clean/scf7180000084603:952109-952216:+	ACA1_071430	EngB-type G domain-containing protein [Source:UniProtKB/TrEMBL;Acc:L8HGF2]	0.036807137	0.765496333	0.728689197	2.86E-15
6h	ACA1_199500/clean/scf7180000084753:248169-248278:+	ACA1_199500	1acylglycerophosphocholine O-acyltransferase 1, putative [Source:UniProtKB/TrEMBL;Acc:L8H4L3]	0.035374467	0.762650667	0.7272762	1.40E-07
6h	ACA1_389330/clean/scf7180000084733:231518-231619:+	ACA1_389330	Nicotinamide riboside kinase 1 family protein [Source:UniProtKB/TrEMBL;Acc:L8GDW0]	0.007722113	0.734696	0.726973887	1.47E-16
6h	ACA1_070900/clean/scf7180000084603:797146-797200:+	ACA1_070900	MMtag domain-containing protein [Source:UniProtKB/TrEMBL;Acc:L8HD49]	0.015616533	0.742058667	0.726442133	2.05E-14
6h	ACA1_260060/clean/scf7180000084769:413925-414056:+	ACA1_260060	WD_REPEATS_REGION domain-containing protein [Source:UniProtKB/TrEMBL;Acc:L8GF95]	0.032699367	0.758310333	0.725610967	1.45E-22
6h	ACA1_309360/clean/scf7180000083758:11988-12070:-	ACA1_309360	hypothetical protein	0.011494267	0.736414	0.724919733	1.16E-06
6h	ACA1_174780/clean/scf7180000084741:795639-795802:-	ACA1_174780	VanY domain-containing protein [Source:UniProtKB/TrEMBL;Acc:L8HK80]	0.0415557	0.766407	0.7248513	1.92E-32
6h	ACA1_184330/clean/scf7180000084745:619706-619791:-	ACA1_184330	SAGA-associated factor 11 [Source:UniProtKB/TrEMBL;Acc:L8H8C5]	0.019132937	0.743913333	0.724780397	2.43E-15
6h	ACA1_189640/clean/scf7180000084748:70712-70803:-	ACA1_189640	Bifunctional aspartate kinase/diaminopimelate decarboxylase protein [Source:UniProtKB/TrEMBL;Acc:L8GEY3]	0.011066237	0.735720667	0.72465443	1.28E-25
6h	ACA1_279550/clean/scf7180000084776:412669-412785:-	ACA1_279550	CBS domain containing protein [Source:UniProtKB/TrEMBL;Acc:L8H8C1]	0.014438333	0.739083333	0.724645	6.43E-17
6h	ACA1_260060/clean/scf7180000084769:413592-413689:+	ACA1_260060	WD_REPEATS_REGION domain-containing protein [Source:UniProtKB/TrEMBL;Acc:L8GF95]	0.016722567	0.739804333	0.723081767	2.48E-23
6h	ACA1_036350/clean/scf7180000084565:84116-84193:+	ACA1_036350	hypothetical protein	0.045739667	0.768519	0.722779333	1.91E-18
6h	ACA1_301540/clean/scf7180000083222:2742-2813:-	ACA1_301540	acidPPc domain-containing protein [Source:UniProtKB/TrEMBL;Acc:L8HJV4]	0.0211597	0.743673667	0.722513967	5.88E-16
6h	ACA1_193130/clean/scf7180000084749:193111-193220:-	ACA1_193130	Metallophos domain-containing protein [Source:UniProtKB/TrEMBL;Acc:L8GN68]	0.1171501	0.839654	0.7225039	4.80E-22
6h	ACA1_389180/clean/scf7180000084733:220295-220373:+	ACA1_389180	hypothetical protein	0.056626533	0.779011333	0.7223848	2.56E-09
6h	ACA1_034030/clean/scf7180000084561:7932-8006:+	ACA1_034030	RNA recognition motif domain containing protein [Source:UniProtKB/TrEMBL;Acc:L8GTW3]	0	0.721843333	0.721843333	2.45E-15
6h	ACA1_233470/clean/scf7180000084762:151193-151262:+	ACA1_233470	hypothetical protein	0.000658517	0.722329667	0.72167115	4.88E-16
6h	ACA1_189450/clean/scf7180000084748:43020-43103:-	ACA1_189450	Chloride channel protein [Source:UniProtKB/TrEMBL;Acc:L8GEJ2]	0.021124867	0.742768333	0.721643467	4.98E-25
6h	ACA1_199500/clean/scf7180000084753:248790-248907:+	ACA1_199500	1acylglycerophosphocholine O-acyltransferase 1, putative [Source:UniProtKB/TrEMBL;Acc:L8H4L3]	0.019607833	0.740631	0.721023167	4.86E-07
6h	ACA1_265380/clean/scf7180000084770:455003-455067:-	ACA1_265380	Phosphatidylinositol glycan anchor biosynthesis, class S, putative [Source:UniProtKB/TrEMBL;Acc:L8H3M4]	0.010038333	0.730867667	0.720829333	2.07E-10
6h	ACA1_065700/clean/scf7180000084599:1873717-1873785:+	ACA1_065700	Protein kinase domain-containing protein [Source:UniProtKB/TrEMBL;Acc:L8H039]	0.037292967	0.75808	0.720787033	2.79E-16
6h	ACA1_266800/clean/scf7180000084770:716880-716965:+	ACA1_266800	Protein kinase domain containing protein [Source:UniProtKB/TrEMBL;Acc:L8H3W0]	0.070527267	0.790936333	0.720409067	5.72E-19
6h	ACA1_330400/clean/scf7180000084566:52801-52937:-	ACA1_330400	SURF1-like protein [Source:UniProtKB/TrEMBL;Acc:L8GJV9]	0.00638882	0.726376333	0.719987513	2.85E-12
6h	ACA1_264290/clean/scf7180000084770:229995-230073:-	ACA1_264290	Geranylgeranyl diphosphate synthase 1, putative [Source:UniProtKB/TrEMBL;Acc:L8H3G3]	0.0075261	0.727160667	0.719634567	5.17E-13
6h	ACA1_057400/clean/scf7180000084599:132202-132301:+	ACA1_057400	Leucine rich repeat domain containing protein [Source:UniProtKB/TrEMBL;Acc:L8GVU1]	0.036664033	0.756285333	0.7196213	1.81E-11
6h	ACA1_396940/clean/scf7180000084759:154158-154348:-	ACA1_396940	OST3 / OST6 family protein [Source:UniProtKB/TrEMBL;Acc:L8HDM5]	0.018479867	0.738063667	0.7195838	2.80E-32
6h	ACA1_389110/clean/scf7180000084733:198080-198168:+	ACA1_389110	hypothetical protein	0.00479035	0.723595333	0.718804983	1.10E-26
6h	ACA1_245500/clean/scf7180000084763:496838-496903:+	ACA1_245500	Fe2OG dioxygenase domain-containing protein [Source:UniProtKB/TrEMBL;Acc:L8GL61]	0.0167552	0.735162	0.7184068	1.05E-13
6h	ACA1_237860/clean/scf7180000084763:32994-33094:+	ACA1_237860	Glycerophosphodiester phosphodiesterase family protein [Source:UniProtKB/TrEMBL;Acc:L8GKN2]	0.136383	0.854764667	0.718381667	5.41E-17
6h	ACA1_193230/clean/scf7180000084749:212965-213041:+	ACA1_193230	Macro domain-containing protein [Source:UniProtKB/TrEMBL;Acc:L8GN79]	0.097080167	0.815088333	0.718008167	6.52E-12
6h	ACA1_382920/clean/scf7180000084721:118628-118727:-	ACA1_382920	Ras subfamily protein [Source:UniProtKB/TrEMBL;Acc:L8GV31]	0.055675	0.773149	0.717474	3.06E-15
6h	ACA1_349640/clean/scf7180000084690:21715-21810:+	ACA1_349640	HEAT repeat domain containing protein [Source:UniProtKB/TrEMBL;Acc:L8GKB3]	0	0.716939333	0.716939333	7.80E-14
6h	ACA1_376660/clean/scf7180000084685:138094-138162:-	ACA1_376660	hypothetical protein	0.0738718	0.790561667	0.716689867	1.41E-15
6h	ACA1_070620/clean/scf7180000084603:749045-749133:+	ACA1_070620	Myotubularin phosphatase domain-containing protein [Source:UniProtKB/TrEMBL;Acc:L8HFC4]	0.009560167	0.726009667	0.7164495	1.40E-23
6h	ACA1_388480/clean/scf7180000084733:123002-123087:-	ACA1_388480	Cofilin/tropomyosin-type actin-binding protein [Source:UniProtKB/TrEMBL;Acc:L8GEM8]	0	0.716300667	0.716300667	3.14E-12
6h	ACA1_261400/clean/scf7180000084769:661113-661173:-	ACA1_261400	Serine/threonine protein kinase, putative [Source:UniProtKB/TrEMBL;Acc:L8GF66]	0	0.716157667	0.716157667	4.66E-06
6h	ACA1_171970/clean/scf7180000084741:211462-211523:+	ACA1_171970	APH domain-containing protein [Source:UniProtKB/TrEMBL;Acc:L8HIW1]	0.083075333	0.799013333	0.715938	3.82E-09
6h	ACA1_075030/clean/scf7180000084608:131508-131570:+	ACA1_075030	Serine carboxypeptidase [Source:UniProtKB/TrEMBL;Acc:L8HFJ6]	0.052937967	0.768705	0.715767033	2.78E-33
6h	ACA1_141960/clean/scf7180000084708:61745-61814:-	ACA1_141960	unspecified product	0.000403063	0.715799333	0.71539627	4.54E-11
6h	ACA1_245940/clean/scf7180000084763:529382-529479:+	ACA1_245940	hypothetical protein	0.010031493	0.725407333	0.71537584	3.05E-14
6h	ACA1_273110/clean/scf7180000084773:16644-16718:+	ACA1_273110	hypothetical protein	0.169894667	0.885129667	0.715235	8.03E-15
6h	ACA1_261270/clean/scf7180000084769:625859-625926:+	ACA1_261270	Methyltransferase domain containing protein [Source:UniProtKB/TrEMBL;Acc:L8GG55]	0.0305152	0.745605333	0.715090133	2.26E-24
6h	ACA1_156030/clean/scf7180000084723:9117-9204:+	ACA1_156030	hypothetical protein	0.0335861	0.748031	0.7144449	2.39E-11
6h	ACA1_063140/clean/scf7180000084599:1340544-1340629:-	ACA1_063140	MFS domain-containing protein [Source:UniProtKB/TrEMBL;Acc:L8GX17]	0	0.714402333	0.714402333	8.93E-07
6h	ACA1_014620/clean/scf7180000083772:1382-1486:-	ACA1_014620	hypothetical protein	0.006938647	0.720087	0.713148353	6.48E-17
6h	ACA1_199000/clean/scf7180000084753:189380-189457:-	ACA1_199000	hypothetical protein	0.011317174	0.724429	0.713111826	6.47E-20
6h	ACA1_108580/clean/scf7180000084661:50794-50882:-	ACA1_108580	hypothetical protein	0.201149333	0.914199667	0.713050333	8.44E-06
6h	ACA1_230520/clean/scf7180000084761:371251-371345:-	ACA1_230520	Hypothetical protein	0.004016067	0.716967333	0.712951267	5.44E-12
6h	ACA1_095540/clean/scf7180000084645:841615-841689:-	ACA1_095540	F-box domain-containing protein [Source:UniProtKB/TrEMBL;Acc:L8GJ76]	0.204709	0.917651333	0.712942333	9.88E-10
6h	ACA1_145180/clean/scf7180000084710:54542-54614:-	ACA1_145180	F-box domain-containing protein [Source:UniProtKB/TrEMBL;Acc:L8GCU3]	0.022996933	0.735860333	0.7128634	3.62E-12
6h	ACA1_138470/clean/scf7180000084703:147254-147322:+	ACA1_138470	Ribosomal_L11 domain-containing protein [Source:UniProtKB/TrEMBL;Acc:L8GZJ5]	0.03879725	0.751200333	0.712403083	3.39E-09
6h	ACA1_139560/clean/scf7180000084705:99659-99801:-	ACA1_139560	YDG domain-containing protein [Source:UniProtKB/TrEMBL;Acc:L8GN24]	0.093800333	0.805405333	0.711605	1.79E-05
6h	ACA1_024200/clean/scf7180000084441:15696-15765:-	ACA1_024200	WD_REPEATS_REGION domain-containing protein [Source:UniProtKB/TrEMBL;Acc:L8GKS0]	0	0.711359	0.711359	3.51E-10
6h	ACA1_330410/clean/scf7180000084566:53858-53947:+	ACA1_330410	hypothetical protein	0	0.711235667	0.711235667	4.48E-15
6h	ACA1_014250/clean/scf7180000083757:21506-21642:-	ACA1_014250	Cytochrome b5 heme-binding domain-containing protein [Source:UniProtKB/TrEMBL;Acc:L8GIZ1]	0.00423577	0.715298667	0.711062897	1.85E-23
6h	ACA1_156030/clean/scf7180000084723:6173-6256:+	ACA1_156030	hypothetical protein	0.006574333	0.716764667	0.710190333	6.48E-10
6h	ACA1_131580/clean/scf7180000084699:90988-91066:-	ACA1_131580	Glycerol-3-phosphate dehydrogenase [NAD(+)] [Source:UniProtKB/TrEMBL;Acc:L8GM84]	0.017995067	0.728014	0.710018933	3.37E-20
6h	ACA1_024240/clean/scf7180000084441:23674-23764:-	ACA1_024240	Yip1 domain containing protein [Source:UniProtKB/TrEMBL;Acc:L8GN09]	0.001357298	0.711369333	0.710012035	8.30E-18
6h	ACA1_186860/clean/scf7180000084746:211879-211967:+	ACA1_186860	GRASP55_65 domain-containing protein [Source:UniProtKB/TrEMBL;Acc:L8H559]	0.010328407	0.720331667	0.710003259	3.50E-14
6h	ACA1_203980/clean/scf7180000084754:243272-243361:+	ACA1_203980	hypothetical protein	0.013868183	0.723834333	0.70996615	4.01E-27
6h	ACA1_390470/clean/scf7180000084740:50342-50440:+	ACA1_390470	Initiator tRNA phosphoribosyl transferase [Source:UniProtKB/TrEMBL;Acc:L8GQ00]	0.0290117	0.738549	0.7095373	6.60E-08
6h	ACA1_175300/clean/scf7180000084741:916822-916909:+	ACA1_175300	Zinc knuckle domain containing protein [Source:UniProtKB/TrEMBL;Acc:L8HKB8]	0.019161933	0.728601667	0.709439733	5.16E-08
6h	ACA1_251640/clean/scf7180000084768:98382-98495:+	ACA1_251640	CBS domain-containing protein [Source:UniProtKB/TrEMBL;Acc:L8HAQ8]	0	0.709283333	0.709283333	3.47E-13
6h	ACA1_246780/clean/scf7180000084763:580698-580795:-	ACA1_246780	Ras subfamily protein [Source:UniProtKB/TrEMBL;Acc:L8GK82]	0.000146671	0.709312333	0.709165663	1.97E-18
6h	ACA1_064860/clean/scf7180000084599:1714844-1714936:+	ACA1_064860	OTU domain-containing protein [Source:UniProtKB/TrEMBL;Acc:L8GWU4]	0.006893783	0.715697	0.708803217	9.43E-11
6h	ACA1_252170/clean/scf7180000084768:137864-137931:-	ACA1_252170	Ras subfamily protein [Source:UniProtKB/TrEMBL;Acc:L8HC26]	0.034882633	0.743568	0.708685367	5.40E-11
6h	ACA1_249910/clean/scf7180000084767:22886-22983:-	ACA1_249910	EGF family domain containing protein [Source:UniProtKB/TrEMBL;Acc:L8HD29]	0	0.708135333	0.708135333	0.005521642
6h	ACA1_035640/clean/scf7180000084564:103091-103162:-	ACA1_035640	Beta-lactamase domain-containing protein [Source:UniProtKB/TrEMBL;Acc:L8H9P2]	0	0.708072	0.708072	3.29E-05
6h	ACA1_383660/clean/scf7180000084721:240089-240200:+	ACA1_383660	tRNA (guanine-N(7)-)-methyltransferase non-catalytic subunit [Source:UniProtKB/TrEMBL;Acc:L8GW53]	0.001741033	0.709333333	0.7075923	5.07E-14
6h	ACA1_400570/clean/scf7180000084775:198113-198199:-	ACA1_400570	Ubiquitin domain containing protein [Source:UniProtKB/TrEMBL;Acc:L8GGV7]	0.00389649	0.711424667	0.707528177	2.58E-22
6h	ACA1_131460/clean/scf7180000084699:64094-64197:+	ACA1_131460	MFS domain-containing protein [Source:UniProtKB/TrEMBL;Acc:L8GN39]	0.044139	0.751436667	0.707297667	4.32E-11
6h	ACA1_154840/clean/scf7180000084722:94082-94159:+	ACA1_154840	Protein kinase domain-containing protein [Source:UniProtKB/TrEMBL;Acc:L8H1C2]	0.063092767	0.770277333	0.707184567	2.27E-14
6h	ACA1_290650/clean/scf7180000084777:646494-646568:+	ACA1_290650	DENN domain-containing protein [Source:UniProtKB/TrEMBL;Acc:L8HI74]	0.029420367	0.736509333	0.707088967	2.52E-09
6h	ACA1_221070/clean/scf7180000084758:990772-990904:+	ACA1_221070	Carrier superfamily protein [Source:UniProtKB/TrEMBL;Acc:L8GT63]	0.005550318	0.710736	0.705185682	4.65E-13
6h	ACA1_307820/clean/scf7180000083687:20646-20742:-	ACA1_307820	BSD domain-containing protein [Source:UniProtKB/TrEMBL;Acc:L8HBM7]	0.017633667	0.722748	0.705114333	1.80E-24
6h	ACA1_371710/clean/scf7180000084664:586749-586829:-	ACA1_371710	Fbox domain containing protein [Source:UniProtKB/TrEMBL;Acc:L8GZD1]	0.0245807	0.729425667	0.704844967	9.31E-13
6h	ACA1_031910/clean/scf7180000084523:35061-35162:-	ACA1_031910	hypothetical protein	0.0555839	0.760281667	0.704697767	8.38E-15
6h	ACA1_154550/clean/scf7180000084722:64577-64674:+	ACA1_154550	unspecified product	0.000814143	0.705217	0.704402857	9.93E-19
6h	ACA1_131000/clean/scf7180000084698:147342-147416:-	ACA1_131000	Nudix hydrolase domain-containing protein [Source:UniProtKB/TrEMBL;Acc:L8GPF6]	0.029624967	0.733790333	0.704165367	2.02E-15
6h	ACA1_264460/clean/scf7180000084770:293954-294030:-	ACA1_264460	GTPbinding protein [Source:UniProtKB/TrEMBL;Acc:L8H231]	0.004866093	0.708970667	0.704104573	1.40E-13

6h	ACA1_125790/clean/scf7180000084691:73280-73354:-	ACA1_125790	Nuclear protein E33 isoform a isoform 3, putative [Source:UniProtKB/TrEMBL;Acc:L8GNY7]	0.029037833	0.732954667	0.703916833	1.08E-20
6h	ACA1_384360/clean/scf7180000084730:32826-32913:-	ACA1_384360	Signal recognition particle, putative [Source:UniProtKB/TrEMBL;Acc:L8HAH5]	0.001236717	0.703406	0.702169283	5.74E-15
6h	ACA1_175760/clean/scf7180000084741:1053649-1053722:-	ACA1_175760	Phosphoglycerate mutase family domain containing protein [Source:UniProtKB/TrEMBL;Acc:L8HH51]	0.0860026	0.786833333	0.700830733	1.12E-16
6h	ACA1_287660/clean/scf7180000084777:56714-56803:+	ACA1_287660	Pentapeptide repeatcontaining protein [Source:UniProtKB/TrEMBL;Acc:L8HIP0]	0.0552258	0.755784667	0.700558867	7.33E-22
6h	ACA1_149540/clean/scf7180000084715:176960-177057:+	ACA1_149540	VPS37 C-terminal domain-containing protein [Source:UniProtKB/TrEMBL;Acc:L8HEF0]	0.020345233	0.720872333	0.7005271	4.61E-21
6h	ACA1_071970/clean/scf7180000084603:1118900-1118980:+	ACA1_071970	Carn_acyltransf domain-containing protein [Source:UniProtKB/TrEMBL;Acc:L8HEI6]	0	0.70033	0.70033	3.23E-12
6h	ACA1_070980/clean/scf7180000084603:821585-821647:-	ACA1_070980	hypothetical protein	0.039872933	0.740129667	0.700256733	9.84E-22
6h	ACA1_388690/clean/scf7180000084733:148853-148950:+	ACA1_388690	hypothetical protein	0.00836394	0.708337	0.69997306	1.50E-25
6h	ACA1_173350/clean/scf7180000084741:467739-467828:-	ACA1_173350	Carrier superfamily protein [Source:UniProtKB/TrEMBL;Acc:L8HHM8]	0.005784587	0.705128333	0.699343747	9.50E-12
6h	ACA1_279360/clean/scf7180000084776:371693-371765:-	ACA1_279360	hypothetical protein	0.002997003	0.702323	0.699325997	5.39E-12
6h	ACA1_287570/clean/scf7180000084777:38466-38543:+	ACA1_287570	hypothetical protein	0.035179267	0.734315	0.699135733	1.40E-16
6h	ACA1_301860/clean/scf7180000083222:43938-44030:+	ACA1_301860	Phosphatidylinositol 3-and 4-kinase [Source:UniProtKB/TrEMBL;Acc:L8HHB0]	0.0200679	0.718978333	0.698910433	5.19E-24
6h	ACA1_219060/clean/scf7180000084758:708330-708474:-	ACA1_219060	DWNN domain containing protein [Source:UniProtKB/TrEMBL;Acc:L8GSX0]	0.108319033	0.807052667	0.698733633	7.47E-08
6h	ACA1_165580/clean/scf7180000084732:55681-55784:-	ACA1_165580	BTB/POZ domain containing protein [Source:UniProtKB/TrEMBL;Acc:L8HI24]	0	0.698696333	0.698696333	5.69E-08
6h	ACA1_349640/clean/scf7180000084690:23912-23996:+	ACA1_349640	HEAT repeat domain containing protein [Source:UniProtKB/TrEMBL;Acc:L8GKB3]	0.0051406	0.703461667	0.698321067	1.27E-10
6h	ACA1_230310/clean/scf7180000084761:343386-343487:+	ACA1_230310	PAS domain-containing protein [Source:UniProtKB/TrEMBL;Acc:L8HBA3]	0.01739217	0.715547333	0.698155163	1.44E-12
6h	ACA1_144100/clean/scf7180000084709:150917-151010:-	ACA1_144100	hypothetical protein	0.032176333	0.729692	0.697515667	2.10E-30
6h	ACA1_282190/clean/scf7180000084776:622680-622783:-	ACA1_282190	Synapsin, ATP binding domain containing protein [Source:UniProtKB/TrEMBL;Acc:L8H7E1]	0.005508893	0.702565667	0.697056773	1.98E-13
6h	ACA1_142140/clean/scf7180000084708:94875-94970:+	ACA1_142140	unspecified product	0.100267333	0.796985333	0.696718	4.25E-15
6h	ACA1_295880/clean/scf7180000084777:1250622-1250693:+	ACA1_295880	Malic enzyme [Source:UniProtKB/TrEMBL;Acc:L8HM80]	0.02989119	0.726181	0.69628981	3.63E-24
6h	ACA1_063580/clean/scf7180000084599:1453309-1453372:+	ACA1_063580	hypothetical protein	0.012086983	0.707671333	0.69558435	8.66E-17
6h	ACA1_261470/clean/scf7180000084769:688960-689043:+	ACA1_261470	hypothetical protein	0.093164333	0.788235	0.695070667	9.89E-20
6h	ACA1_360570/clean/scf7180000084622:164642-164703:+	ACA1_360570	Asparaginase [Source:UniProtKB/TrEMBL;Acc:L8HF54]	0.088373033	0.783361	0.694987967	2.73E-12
6h	ACA1_034520/clean/scf7180000084563:61413-61544:+	ACA1_034520	G_PROTEIN_RECEP_F2_4 domain-containing protein [Source:UniProtKB/TrEMBL;Acc:L8HBK6]	0	0.694672333	0.694672333	2.07E-09
6h	ACA1_263870/clean/scf7180000084770:123783-123880:-	ACA1_263870	MAGE domain-containing protein [Source:UniProtKB/TrEMBL;Acc:L8H1Y1]	0.042328067	0.736721333	0.694393267	4.87E-09
6h	ACA1_060710/clean/scf7180000084599:735176-735284:-	ACA1_060710	Alcohol dehydrogenase [Source:UniProtKB/TrEMBL;Acc:L8GVS2]	0.037763007	0.731974333	0.694211327	4.48E-07
6h	ACA1_343290/clean/scf7180000084638:15395-15453:+	ACA1_343290	hypothetical protein	0.092075433	0.78609	0.694014567	1.91E-18
6h	ACA1_198480/clean/scf7180000084753:124168-124308:+	ACA1_198480	Transcription factor III family protein [Source:UniProtKB/TrEMBL;Acc:L8H324]	0.004066257	0.697887667	0.69382141	2.31E-11
6h	ACA1_199500/clean/scf7180000084753:248627-248734:+	ACA1_199500	1acylglycerophosphocholine O-acyltransferase 1, putative [Source:UniProtKB/TrEMBL;Acc:L8H4L3]	0.042130167	0.735871333	0.693741167	2.34E-08
6h	ACA1_088960/clean/scf7180000084639:274764-274836:-	ACA1_088960	Beta-lactamase domain-containing protein [Source:UniProtKB/TrEMBL;Acc:L8GU04]	0.015637567	0.708912	0.693274433	5.54E-09
6h	ACA1_362550/clean/scf7180000084624:92352-92416:+	ACA1_362550	Aldedh domain-containing protein [Source:UniProtKB/TrEMBL;Acc:L8GHV5]	0.000500632	0.693448333	0.692947701	3.63E-23
6h	ACA1_068040/clean/scf7180000084603:85068-85143:+	ACA1_068040	START domain-containing protein [Source:UniProtKB/TrEMBL;Acc:L8HDG1]	0.031877905	0.724820667	0.692942762	2.87E-11
6h	ACA1_230310/clean/scf7180000084761:342605-342687:+	ACA1_230310	PAS domain-containing protein [Source:UniProtKB/TrEMBL;Acc:L8HBA3]	0.01329864	0.705969	0.69267036	1.78E-10
6h	ACA1_146440/clean/scf7180000084713:25533-25631:+	ACA1_146440	Methyltransf_11 domain-containing protein [Source:UniProtKB/TrEMBL;Acc:L8GU12]	0.053952433	0.746569333	0.6926169	8.45E-16
6h	ACA1_206370/clean/scf7180000084757:17262-17334:+	ACA1_206370	MOSC domain-containing protein [Source:UniProtKB/TrEMBL;Acc:L8GY21]	0.00868972	0.700777	0.69208728	2.02E-22
6h	ACA1_237840/clean/scf7180000084763:27251-27322:-	ACA1_237840	hypothetical protein	0.050167433	0.741515	0.691347567	5.62E-24
6h	ACA1_384800/clean/scf7180000084730:107650-107733:+	ACA1_384800	hypothetical protein	0.0060606	0.696914333	0.690853733	2.66E-10
6h	ACA1_113560/clean/scf7180000084669:321431-321591:-	ACA1_113560	PH domain containing protein [Source:UniProtKB/TrEMBL;Acc:L8H6B7]	0.011086333	0.701607333	0.690521	1.29E-14
6h	ACA1_287570/clean/scf7180000084777:38724-38821:+	ACA1_287570	hypothetical protein	0.019201317	0.709312667	0.69011135	5.75E-22
6h	ACA1_252170/clean/scf7180000084768:136887-136950:-	ACA1_252170	Ras subfamily protein [Source:UniProtKB/TrEMBL;Acc:L8HC26]	0.104043367	0.793944333	0.689900967	1.09E-14
6h	ACA1_175700/clean/scf7180000084741:1038084-1038176:-	ACA1_175700	Frizzled/Smoothened family membrane region protein [Source:UniProtKB/TrEMBL;Acc:L8HH45]	0	0.689109	0.689109	5.32E-13
6h	ACA1_379080/clean/scf7180000084711:275891-275963:+	ACA1_379080	GATA-type domain-containing protein [Source:UniProtKB/TrEMBL;Acc:L8GS28]	0.239021	0.926401	0.68738	8.73E-25
6h	ACA1_230530/clean/scf7180000084761:372175-372254:+	ACA1_230530	S(Hydroxymethyl)glutathione dehydrogenase/class III alcohol dehydrogenase [Source:UniProtKB/TrEMBL;Acc:L8H8X2]	0.001711637	0.688500333	0.686788697	3.40E-34
6h	ACA1_199500/clean/scf7180000084753:248443-248513:+	ACA1_199500	1acylglycerophosphocholine O-acyltransferase 1, putative [Source:UniProtKB/TrEMBL;Acc:L8H4L3]	0.004635467	0.690767667	0.6861322	8.95E-08
6h	ACA1_101490/clean/scf7180000084649:73433-73537:-	ACA1_101490	Methyltransf_11 domain-containing protein [Source:UniProtKB/TrEMBL;Acc:L8GH92]	0.250854333	0.936981667	0.686127333	0.000115938
6h	ACA1_036890/clean/scf7180000084567:6275-6340:+	ACA1_036890	Ras subfamily protein [Source:UniProtKB/TrEMBL;Acc:L8H0T2]	0.048561267	0.73455	0.685988733	1.80E-18
6h	ACA1_100470/clean/scf7180000084648:91887-91949:-	ACA1_100470	ABC transmembrane type-1 domain-containing protein [Source:UniProtKB/TrEMBL;Acc:L8GJH4]	0.0179479	0.703312	0.6853641	1.33E-06
6h	ACA1_264180/clean/scf7180000084770:206798-206889:+	ACA1_264180	Oxidoreductase, short chain dehydrogenase/reductase superfamily protein [Source:UniProtKB/TrEMBL;Acc:L8H208]	0.137198333	0.822147333	0.684949	1.25E-10
6h	ACA1_198340/clean/scf7180000084753:83417-83511:-	ACA1_198340	Pept_C1 domain-containing protein [Source:UniProtKB/TrEMBL;Acc:L8H2C3]	0.037014433	0.721681667	0.684667233	1.18E-22
6h	ACA1_247260/clean/scf7180000084763:657894-658013:+	ACA1_247260	SET domain-containing protein [Source:UniProtKB/TrEMBL;Acc:L8GKH1]	0.005291397	0.689564667	0.68427327	7.43E-22
6h	ACA1_396130/clean/scf7180000084759:24941-25060:-	ACA1_396130	Prefoldin, alpha subunit [Source:UniProtKB/TrEMBL;Acc:L8HDI9]	0.009020933	0.693182333	0.6841614	4.09E-36
6h	ACA1_182880/clean/scf7180000084745:283141-283293:-	ACA1_182880	E3 ubiquitin-protein ligase [Source:UniProtKB/TrEMBL;Acc:L8H743]	0.008598333	0.692409667	0.683811333	6.64E-14
6h	ACA1_116720/clean/scf7180000084669:833657-833739:+	ACA1_116720	J domain-containing protein [Source:UniProtKB/TrEMBL;Acc:L8H4V8]	0.015009	0.698395333	0.683386333	4.08E-24
6h	ACA1_310770/clean/scf7180000083780:8815-8928:+	ACA1_310770	TAP Cterminal subfamily protein [Source:UniProtKB/TrEMBL;Acc:L8GUF1]	0	0.682919667	0.682919667	8.97E-13
6h	ACA1_255530/clean/scf7180000084768:534087-534180:-	ACA1_255530	Carrier superfamily protein [Source:UniProtKB/TrEMBL;Acc:L8HB08]	0.027947383	0.710586	0.682638617	7.49E-15
6h	ACA1_275600/clean/scf7180000084774:49944-50015:+	ACA1_275600	unspecified product	0.1065927	0.789024667	0.682431967	3.98E-09
6h	ACA1_163990/clean/scf7180000084729:126982-127047:-	ACA1_163990	Glycosyl hydrolases family 32 superfamily protein [Source:UniProtKB/TrEMBL;Acc:L8GRF2]	0.0346286	0.715728667	0.681100067	1.03E-08
6h	ACA1_378010/clean/scf7180000084711:163311-163380:+	ACA1_378010	JmjC domain-containing protein [Source:UniProtKB/TrEMBL;Acc:L8GRT4]	0.315293333	0.996384667	0.681091333	8.74E-11
6h	ACA1_265440/clean/scf7180000084770:473375-473449:+	ACA1_265440	Myosin VIIa, putative [Source:UniProtKB/TrEMBL;Acc:L8H1F4]	0.002911863	0.683881667	0.680969803	2.74E-13
6h	ACA1_271330/clean/scf7180000084771:21570-21663:+	ACA1_271330	hypothetical protein	0.014590493	0.695558	0.680967507	6.86E-18
6h	ACA1_069190/clean/scf7180000084603:378149-378217:+	ACA1_069190	PfkB domain-containing protein [Source:UniProtKB/TrEMBL;Acc:L8HDR4]	0	0.680632333	0.680632333	3.54E-13
6h	ACA1_378130/clean/scf7180000084711:193436-193554:-	ACA1_378130	hypothetical protein	0.014563233	0.695143333	0.6805801	7.28E-12
6h	ACA1_291340/clean/scf7180000084777:749586-749657:-	ACA1_291340	Ubiquitin-like domain-containing protein [Source:UniProtKB/TrEMBL;Acc:L8HLN7]	0.00852256	0.688826333	0.680303773	4.28E-37
6h	ACA1_289870/clean/scf7180000084777:484343-484438:+	ACA1_289870	Helicase ATP-binding domain-containing protein [Source:UniProtKB/TrEMBL;Acc:L8HI29]	0.0050606	0.684794333	0.679733733	1.37E-09
6h	ACA1_150950/clean/scf7180000084716:33128-33270:+	ACA1_150950	Exocyst complex component Sec6 [Source:UniProtKB/TrEMBL;Acc:L8H215]	0.0245249	0.703896	0.6793711	1.06E-11
6h	ACA1_034150/clean/scf7180000084561:35650-35723:-	ACA1_034150	hypothetical protein	0.025341567	0.704149333	0.678807767	1.83E-15
6h	ACA1_069910/clean/scf7180000084603:597875-597981:+	ACA1_069910	Citrin, putative [Source:UniProtKB/TrEMBL;Acc:L8HG30]	0.0277261	0.706399667	0.678673567	1.66E-15
6h	ACA1_136780/clean/scf7180000084703:7077-7152:+	ACA1_136780	hypothetical protein	0.130990333	0.809637667	0.678647333	1.88E-28
6h	ACA1_396200/clean/scf7180000084759:51005-51106:+	ACA1_396200	Epimerase domain-containing protein [Source:UniProtKB/TrEMBL;Acc:L8HBC7]	0.00566835	0.684107667	0.678439317	6.88E-15
6h	ACA1_097960/clean/scf7180000084645:1363284-1363366:-	ACA1_097960	Ribosomal protein L33, putative [Source:UniProtKB/TrEMBL;Acc:L8GJX1]	0.016719127	0.694613333	0.677894207	1.34E-21
6h	ACA1_089240/clean/scf7180000084639:346398-346461:+	ACA1_089240	Ubiquitin_4 domain-containing protein [Source:UniProtKB/TrEMBL;Acc:L8GX75]	0.200155667	0.877439333	0.677283667	3.04E-09
6h	ACA1_002780/clean/scf7180000082937:9820-9886:-	ACA1_002780	RRM domain-containing protein [Source:UniProtKB/TrEMBL;Acc:L8GGZ7]	0.050164033	0.726528	0.676363967	4.13E-09
6h	ACA1_323490/clean/scf7180000084468:36325-36414:+	ACA1_323490	DH domain-containing protein [Source:UniProtKB/TrEMBL;Acc:L8H1L1]	0.059355	0.735272667	0.675917667	1.10E-15
6h	ACA1_071160/clean/scf7180000084603:876423-876562:+	ACA1_071160	Letm1 RBD domain-containing protein [Source:UniProtKB/TrEMBL;Acc:L8HE82]	0.014560667	0.690248667	0.675688	7.38E-12
6h	ACA1_058220/clean/scf7180000084599:273589-273651:+	ACA1_058220	hypothetical protein	0.030698833	0.706077	0.675378167	1.54E-14
6h	ACA1_173350/clean/scf7180000084741:468908-469026:-	ACA1_173350	Carrier superfamily protein [Source:UniProtKB/TrEMBL;Acc:L8HHM8]	0.000310945	0.675677	0.675366055	2.99E-15
6h	ACA1_012300/clean/scf7180000083740:10522-10589:-	ACA1_012300	hypothetical protein	0.00620518	0.681212667	0.675007487	4.68E-11
6h	ACA1_277990/clean/scf7180000084776:147560-147651:+	ACA1_277990	Calponin domain containing protein [Source:UniProtKB/TrEMBL;Acc:L8H800]	0	0.674715	0.674715	1.61E-06
6h	ACA1_235410/clean/scf7180000084762:341013-341161:+	ACA1_235410	hypothetical protein	0	0.674672333	0.674672333	8.22E-13
6h	ACA1_091900/clean/scf7180000084645:223935-224032:-	ACA1_091900	Acylphosphatase [Source:UniProtKB/TrEMBL;Acc:L8GHY8]	0.034128833	0.708674667	0.674545833	2.14E-11
6h	ACA1_271330/clean/scf7180000084771:20879-20960:+	ACA1_271330	hypothetical protein	0.036683733	0.710698667	0.674014933	5.38E-19
6h	ACA1_175470/clean/scf7180000084741:966321-966380:-	ACA1_175470	Endo/exonuclease/phosphatase domain-containing protein [Source:UniProtKB/TrEMBL;Acc:L8HI27]	0.139062667	0.812958333	0.673895667	8.59E-11
6h	ACA1_253360/clean/scf7180000084768:284669-284796:+	ACA1_253360	TP_methylase domain-containing protein [Source:UniProtKB/TrEMBL;Acc:L8HAX0]	0.01592528	0.689716667	0.673791387	3.96E-11
6h	ACA1_136170/clean/scf7180000084702:144381-144480:+	ACA1_136170	Cofilin/tropomyosin-type actin-binding protein [Source:UniProtKB/TrEMBL;Acc:L8GGT0]	0.015073333	0.688853	0.673779667	1.22E-39
6h	ACA1_094060/clean/scf7180000084645:688210-688399:+	ACA1_094060	START domain-containing protein [Source:UniProtKB/TrEMBL;Acc:L8GJ20]	0.012755223	0.686471	0.673715777	1.13E-07
6h	ACA1_167050/clean/scf7180000084736:61752-61810:-	ACA1_167050	Ras subfamily protein [Source:UniProtKB/TrEMBL;Acc:L8H0S4]	0.033910133	0.707526667	0.673616533	4.00E-12
6h	ACA1_198340/clean/scf7180000084753:82983-83064:-	ACA1_198340	Pept_C1 domain-containing protein [Source:UniProtKB/TrEMBL;Acc:L8H2C3]	0.002162424	0.675711	0.673548576	1.25E-17
6h	ACA1_033610/clean/scf7180000084559:58270-58352:+	ACA1_033610	Sortilin [Source:UniProtKB/TrEMBL;Acc:L8GHJ0]	0.005732557	0.679024667	0.67329211	1.99E-17
6h	ACA1_396260/clean/scf7180000084759:65295-65402:+	ACA1_396260	hypothetical protein	0.016488267	0.68892	0.672431733	4.24E-15
6h	ACA1_136140/clean/scf7180000084702:137628-137729:+	ACA1_136140	Homeobox domain-containing protein [Source:UniProtKB/TrEMBL;Acc:L8GF88]	0.0216166	0.693959667	0.672343067	2.32E-29
6h	ACA1_247140/clean/scf7180000084763:629097-629192:+	ACA1_247140	hypothetical protein	0.00850344	0.680731	0.67222756	1.72E-09
6h	ACA1_042040/clean/scf7180000084573:44498-44576:-	ACA1_042040	RAI1 domain-containing protein [Source:UniProtKB/TrEMBL;Acc:L8GV91]	0.032930167	0.704522333	0.671592167	3.83E-11
6h	ACA1_215600/clean/scf7180000084758:347256-347381:-	ACA1_215600	hypothetical protein	0.0214432	0.693027	0.6715838	3.17E-07
6h	ACA1_110060/clean/scf7180000084663:50405-50517:-	ACA1_110060	Initiation factor, subunit 2 family protein [Source:UniProtKB/TrEMBL;Acc:L8HHD7]	8.27E-05	0.671536	0.671453287	1.75E-16
6h	ACA1_174080/clean/scf7180000084741:602739-602887:-	ACA1_174080	t-SNARE coiled-coil homology domain-containing protein [Source:UniProtKB/TrEMBL;Acc:L8HGR7]	0.202952333	0.874346333	0.671394	2.34E-20
6h	ACA1_157930/clean/scf7180000084724:152815-152896:-	ACA1_157930	Protein SEY1 homolog [Source:UniProtKB/TrEMBL;Acc:L8HCF0]	0.0057278	0.676969667	0.671241867	2.56E-16
6h	ACA1_332590/clean/scf7180000084579:42865-42943:-	ACA1_332590	GH16 domain-containing protein [Source:UniProtKB/TrEMBL;Acc:L8GKV3]	0.003928711	0.674584333	0.670655622	7.82E-26
6h	ACA1_315790/clean/scf7180000083943:8870-8950:-	ACA1_315790	Copper transport accessory protein [Source:UniProtKB/TrEMBL;Acc:L8HDX3]	0.0277271	0.697673	0.6699459	1.20E-22
6h	ACA1_384710/clean/scf7180000084730:90624-90741:+	ACA1_384710	Transmembrane receptor family protein [Source:UniProtKB/TrEMBL;Acc:L8HBG4]	0.018627267	0.688526333	0.669899067	1.71E-25
6h	ACA1_223310/clean/scf7180000084760:127825-127902:-	ACA1_223310	Amidohydro_3 domain-containing protein [Source:UniProtKB/TrEMBL;Acc:L8GTU8]	0.01784614	0.687636667	0.669790527	5.38E-09
6h	ACA1_087650/clean/scf7180000084639:90740-90831:-	ACA1_087650	CULLIN_2 domain-containing protein [Source:UniProtKB/TrEMBL;Acc:L8GUJ4]	0.040669733	0.710375667	0.669705933	6.80E-13
6h	ACA1_096430/clean/scf7180000084645:1009673-1009757:-	ACA1_096430	Aminotran_1_2 domain-containing protein [Source:UniProtKB/TrEMBL;Acc:L8GIY6]	0.035164627	0.704791333	0.669626707	3.56E-10
6h	ACA1_264850/clean/scf7180000084770:389582-389654:+	ACA1_264850	MaoC-like domain-containing protein [Source:UniProtKB/TrEMBL;Acc:L8H4C0]	0.053124	0.722495333	0.669371333	2.21E-14
6h	ACA1_037450/clean/scf7180000084567:93782-93845:+	ACA1_037450	NTPase_I-T domain-containing protein [Source:UniProtKB/TrEMBL;Acc:L8H395]	0.159161667	0.828469	0.669307333	4.76E-12
6h	ACA1_083640/clean/scf7180000084629:7780-7874:-	ACA1_083640	Elongation factor 1alpha [Source:UniProtKB/TrEMBL;Acc:L8GYH2]	0.004578433	0.673472333	0.6688939	7.73E-27
6h	ACA1_140210/clean/scf7180000084707:44277-44414:-	ACA1_140210	hypothetical protein	0.196891667	0.865558	0.668666333	3.92E-05
6h	ACA1_237860/clean/scf7180000084763:33222-33358:+	ACA1_237860	Glycerophosphodiester phosphodiesterase family protein [Source:UniProtKB/TrEMBL;Acc:L8GKN2]	0.096327333	0.764981	0.668653667	4.79E-17
6h	ACA1_181210/clean/scf7180000084744:148021-148108:-	ACA1_181210	PHDfinger domain containing protein [Source:UniProtKB/TrEMBL;Acc:L8GDS2]	0.021617733	0.690140333	0.6685226	9.41E-07
6h	ACA1_091560/clean/scf7180000084645:149699-149763:+	ACA1_091560	UBP-type domain-containing protein [Source:UniProtKB/TrEMBL;Acc:L8GKE3]	0.146912667	0.815349667	0.668437	1.15E-09
6h	ACA1_051550/clean/scf7180000084592:37118-37210:-	ACA1_051550	Phenylalanine hydroxylase [Source:UniProtKB/TrEMBL;Acc:L8GPS8]	0.008146583	0.676477667	0.668331083	5.71E-34
6h	ACA1_107530/clean/scf7180000084660:215836-215931:-	ACA1_107530	UBP-type domain-containing protein [Source:UniProtKB/TrEMBL;Acc:L8GMW8]	0.178114333	0.846380667	0.668266333	1.93E-25
6h	ACA1_034030/clean/scf7180000084561:9347-9470:+	ACA1_034030	RNA recognition motif domain containing protein [Source:UniProtKB/TrEMBL;Acc:L8GTW3]	0.0292182	0.697135	0.6679168	7.30E-15
6h	ACA1_342290/clean/scf7180000084633:13782-13878:+	ACA1_342290	hypothetical protein	0.0803793	0.748247	0.6678677	1.16E-16
6h	ACA1_039340/clean/scf7180000084569:29189-29258:-	ACA1_039340	hypothetical protein	0	0.667561	0.667561	2.24E-11
6h	ACA1_243850/clean/scf7180000084763:362810-362936:+	ACA1_243850	Ras-GAP domain-containing protein [Source:UniProtKB/TrEMBL;Acc:L8GJZ5]	0	0.667492667	0.667492667	5.60E-05
6h	ACA1_221370/clean/scf7180000084758:1025298-1025411:+	ACA1_221370	hypothetical protein	0.007478627	0.674793333	0.667314707	1.23E-11
6h	ACA1_154690/clean/scf7180000084722:77713-77806:+	ACA1_154690	unspecified product	0.0560526	0.723119333	0.667066733	9.44E-18
6h	ACA1_017540/clean/scf7180000083862:3516-3615:-	ACA1_017540	hypothetical protein	0.006784318	0.673428333	0.666644015	1.53E-11
6h	ACA1_364510/clean/scf7180000084641:126333-126389:-	ACA1_364510	Kelch repeatcontaining protein [Source:UniProtKB/TrEMBL;Acc:L8GM67]	0.171800667	0.838427	0.666626333	6.61E-06
6h	ACA1_383610/clean/scf7180000084721:225990-226087:+	ACA1_383610	PAKA subfamily protein kinase [Source:UniProtKB/TrEMBL;Acc:L8GW48]	0.003267973	0.669618	0.666350027	8.20E-15
6h	ACA1_289590/clean/scf7180000084777:427931-428013:+	ACA1_289590	hypothetical protein	0	0.666013333	0.666013333	2.26E-07
6h	ACA1_234260/clean/scf7180000084762:255364-255470:+	ACA1_234260	DH domain-containing protein [Source:UniProtKB/TrEMBL;Acc:L8H2M0]	0.015868693	0.681437	0.665568307	1.95E-23
6h	ACA1_295340/clean/scf7180000084777:1108539-1108622:-	ACA1_295340	Arl5family small GTPase [Source:UniProtKB/TrEMBL;Acc:L8HIM0]	0.00955623	0.675003667	0.665447437	5.61E-29
6h	ACA1_295320/clean/scf7180000084777:1104376-1104491:+	ACA1_295320	hypothetical protein	0.027977567	0.69324	0.665262433	2.50E-25
6h	ACA1_132840/clean/scf7180000084700:84240-84322:+	ACA1_132840	DHR10 domain-containing protein [Source:UniProtKB/TrEMBL;Acc:L8GWV1]	0	0.665206667	0.665206667	0.028810018
6h	ACA1_381120/clean/scf7180000084712:147842-147924:+	ACA1_381120	Maleylacetoacetate isomerase [Source:UniProtKB/TrEMBL;Acc:L8GMX6]	0.01279174	0.677958	0.66516626	3.18E-24
6h	ACA1_068500/clean/scf7180000084603:217515-217601:+	ACA1_068500	Eukaryotic family protein [Source:UniProtKB/TrEMBL;Acc:L8HFQ5]	0.0259262	0.691000333	0.665074133	7.49E-15
6h	ACA1_071890/clean/scf7180000084603:1095783-1095870:-	ACA1_071890	FHA domain containing protein [Source:UniProtKB/TrEMBL;Acc:L8HEB4]	0	0.664658	0.664658	9.59E-07
6h	ACA1_123630/clean/scf7180000084686:46489-46581:-	ACA1_123630	hypothetical protein	0.183955333	0.848607667	0.664652333	1.11E-12
6h	ACA1_197610/clean/scf7180000084753:30084-30206:-	ACA1_197610	hypothetical protein	0.124280533	0.788824667	0.664544133	1.11E-08
6h	ACA1_290250/clean/scf7180000084777:569198-569268:+	ACA1_290250	hypothetical protein	0.031633427	0.696106333	0.664472907	2.99E-08
6h	ACA1_271750/clean/scf7180000084771:100680-100769:-	ACA1_271750	Metal cation transporter, ZIP subfamily protein [Source:UniProtKB/TrEMBL;Acc:L8GNS8]	0.015555567	0.679969333	0.664413767	3.67E-09
6h	ACA1_050360/clean/scf7180000084589:77953-78031:-	ACA1_050360	Metalloenzyme domain-containing protein [Source:UniProtKB/TrEMBL;Acc:L8H565]	0.0085019	0.672790667	0.664288767	5.92E-19
6h	ACA1_282210/clean/scf7180000084776:628219-628286:+	ACA1_282210	Betacarotene 15,15'-monooxygenase 1, putative [Source:UniProtKB/TrEMBL;Acc:L8H762]	0.01679742	0.680217333	0.663419913	1.10E-15
6h	ACA1_089330/clean/scf7180000084639:370548-370655:-	ACA1_089330	Glyco_hydro_2_C domain-containing protein [Source:UniProtKB/TrEMBL;Acc:L8GUL5]	0.001798883	0.664979333	0.66318045	4.86E-07
6h	ACA1_369740/clean/scf7180000084664:372227-372302:-	ACA1_369740	Cyclin, Nterminal domain containing protein [Source:UniProtKB/TrEMBL;Acc:L8H1H3]	0.001022493	0.6642	0.663177507	2.23E-10
6h	ACA1_060650/clean/scf7180000084599:724928-725019:+	ACA1_060650	Protein kinase domain-containing protein [Source:UniProtKB/TrEMBL;Acc:L8GXP5]	0.03582112	0.698885667	0.663064547	2.49E-09
6h	ACA1_071170/clean/scf7180000084603:881893-881972:+	ACA1_071170	RhoGAP domain containing protein [Source:UniProtKB/TrEMBL;Acc:L8HGC8]	0	0.662990667	0.662990667	3.89E-10

6h	ACA1_165600/clean/scf7180000084732:61708-61785:-	ACA1_165600	VWFA domain-containing protein [Source:UniProtKB/TrEMBL;Acc:L8HGQ3]	0.039760933	0.702749333	0.6629884	2.49E-12
6h	ACA1_382670/clean/scf7180000084721:108239-108304:-	ACA1_382670	DEAD/DEAH box helicase domain containing protein [Source:UniProtKB/TrEMBL;Acc:L8GV26]	0	0.662891333	0.662891333	4.75E-09
6h	ACA1_252450/clean/scf7180000084768:153620-153701:+	ACA1_252450	L1P family protein [Source:UniProtKB/TrEMBL;Acc:L8HAS4]	0.013641367	0.676198667	0.6625573	2.04E-15
6h	ACA1_088960/clean/scf7180000084639:274567-274634:-	ACA1_088960	Beta-lactamase domain-containing protein [Source:UniProtKB/TrEMBL;Acc:L8GU04]	0.055555667	0.718026	0.662470333	1.23E-05
6h	ACA1_094260/clean/scf7180000084645:736982-737079:+	ACA1_094260	RhoGEF domain containing protein [Source:UniProtKB/TrEMBL;Acc:L8GIN9]	0.016418057	0.678468	0.662049943	1.78E-15
6h	ACA1_189640/clean/scf7180000084748:70409-70499:-	ACA1_189640	Bifunctional aspartate kinase/diaminopimelate decarboxylase protein [Source:UniProtKB/TrEMBL;Acc:L8GEY3]	0.007138616	0.668674333	0.661535718	1.92E-21
6h	ACA1_073230/clean/scf7180000084603:1376453-1376536:-	ACA1_073230	EHN domain-containing protein [Source:UniProtKB/TrEMBL;Acc:L8HDT9]	0.014590293	0.675444667	0.660854373	1.23E-12
6h	ACA1_349640/clean/scf7180000084690:21914-22010:+	ACA1_349640	HEAT repeat domain containing protein [Source:UniProtKB/TrEMBL;Acc:L8GKB3]	0	0.660822667	0.660822667	1.32E-12
6h	ACA1_331240/clean/scf7180000084575:9460-9532:-	ACA1_331240	Fe2OG dioxygenase domain-containing protein [Source:UniProtKB/TrEMBL;Acc:L8GVM3]	0.084982233	0.745611667	0.660629433	1.29E-10
6h	ACA1_287660/clean/scf7180000084777:57007-57073:+	ACA1_287660	Pentapeptide repeatcontaining protein [Source:UniProtKB/TrEMBL;Acc:L8HIP0]	0.008549213	0.668885333	0.66033612	1.68E-15
6h	ACA1_184260/clean/scf7180000084745:596192-596270:-	ACA1_184260	Phytanoyl-CoA dioxygenase [Source:UniProtKB/TrEMBL;Acc:L8H8I4]	0.003745333	0.663820667	0.660075333	2.37E-09
6h	ACA1_246780/clean/scf7180000084763:580874-580955:-	ACA1_246780	Ras subfamily protein [Source:UniProtKB/TrEMBL;Acc:L8GK82]	0.01503092	0.675015667	0.659984747	5.91E-23
6h	ACA1_384070/clean/scf7180000084730:14167-14272:+	ACA1_384070	Sel1 repeatcontaining protein [Source:UniProtKB/TrEMBL;Acc:L8H878]	0.014283217	0.674179	0.659895783	5.19E-09
6h	ACA1_204520/clean/scf7180000084754:303203-303287:-	ACA1_204520	hypothetical protein	0	0.659825	0.659825	0.012683586
6h	ACA1_061610/clean/scf7180000084599:983012-983108:-	ACA1_061610	Rap-GAP domain-containing protein [Source:UniProtKB/TrEMBL;Acc:L8GZ88]	0.034278133	0.693960667	0.659682533	3.70E-12
6h	ACA1_095070/clean/scf7180000084645:806721-806844:+	ACA1_095070	hypothetical protein	0.013993887	0.673537	0.659543113	3.08E-15
6h	ACA1_343310/clean/scf7180000084638:23565-23680:-	ACA1_343310	EF-hand domain-containing protein [Source:UniProtKB/TrEMBL;Acc:L8H9D8]	0	0.659442333	0.659442333	7.55E-09
6h	ACA1_221650/clean/scf7180000084760:13550-13639:+	ACA1_221650	R3H domain containing protein [Source:UniProtKB/TrEMBL;Acc:L8GS35]	0.004679073	0.663706667	0.659027593	3.88E-10
6h	ACA1_123220/clean/scf7180000084686:6285-6394:-	ACA1_123220	hypothetical protein	0.006172833	0.664861	0.658688167	2.48E-09
6h	ACA1_287350/clean/scf7180000084777:12717-12815:-	ACA1_287350	His Kinase A (Phosphoacceptor) domain containing protein [Source:UniProtKB/TrEMBL;Acc:L8HKZ2]	0	0.658516667	0.658516667	4.85E-07
6h	ACA1_123220/clean/scf7180000084686:6076-6188:-	ACA1_123220	hypothetical protein	0.005437437	0.663597667	0.65816023	3.05E-11
6h	ACA1_178060/clean/scf7180000084743:162467-162555:-	ACA1_178060	Dipeptidylpeptidase 9, putative [Source:UniProtKB/TrEMBL;Acc:L8GTD8]	0.00051894	0.658618333	0.658099393	7.73E-13
6h	ACA1_175300/clean/scf7180000084741:918255-918344:+	ACA1_175300	Zinc knuckle domain containing protein [Source:UniProtKB/TrEMBL;Acc:L8HKB8]	0.0206527	0.678673333	0.658020633	8.87E-08
6h	ACA1_383700/clean/scf7180000084721:249139-249217:+	ACA1_383700	Endo/exonuclease/phosphatase domain-containing protein [Source:UniProtKB/TrEMBL;Acc:L8GV68]	0.032457267	0.690398667	0.6579414	9.98E-15
6h	ACA1_111960/clean/scf7180000084669:74591-74682:-	ACA1_111960	Ornithine cyclodeaminase [Source:UniProtKB/TrEMBL;Acc:L8H5A8]	0.006179231	0.663731	0.657551769	2.36E-13
6h	ACA1_279550/clean/scf7180000084776:412850-412950:-	ACA1_279550	CBS domain containing protein [Source:UniProtKB/TrEMBL;Acc:L8H8C1]	0.0301074	0.687585333	0.657477933	1.29E-18
6h	ACA1_040210/clean/scf7180000084571:14350-14437:+	ACA1_040210	Mitochondrial uncoupling protein [Source:UniProtKB/TrEMBL;Acc:L8GGI9]	0.019370233	0.676386667	0.657016433	6.85E-14
6h	ACA1_248200/clean/scf7180000084766:24176-24245:+	ACA1_248200	APH domain-containing protein [Source:UniProtKB/TrEMBL;Acc:L8H0B8]	0.111111	0.768050667	0.656939667	0.001804459
6h	ACA1_359690/clean/scf7180000084622:1607-1753:-	ACA1_359690	Rab-GAP TBC domain-containing protein [Source:UniProtKB/TrEMBL;Acc:L8HBR2]	0.034522533	0.691114	0.656591467	1.62E-18
6h	ACA1_192360/clean/scf7180000084749:128129-128217:+	ACA1_192360	hypothetical protein	0.036444633	0.693001	0.656556367	4.18E-17
6h	ACA1_172910/clean/scf7180000084741:354932-355040:-	ACA1_172910	Chloride channel protein [Source:UniProtKB/TrEMBL;Acc:L8HGK6]	0.0050505	0.661386	0.6563355	6.06E-10
6h	ACA1_170770/clean/scf7180000084741:556-670:-	ACA1_170770	UBP-type domain-containing protein [Source:UniProtKB/TrEMBL;Acc:L8HJG8]	0.000402553	0.656690667	0.656288113	5.88E-16
6h	ACA1_264270/clean/scf7180000084770:226686-226775:-	ACA1_264270	Oxidoreductase, short chain dehydrogenase/reductase family protein [Source:UniProtKB/TrEMBL;Acc:L8H474]	0.005318563	0.661487667	0.656169103	9.53E-13
6h	ACA1_058140/clean/scf7180000084599:259084-259173:+	ACA1_058140	Sarp-3, putative [Source:UniProtKB/TrEMBL;Acc:L8GX72]	0.0371116	0.693155	0.6560434	7.09E-12
6h	ACA1_063580/clean/scf7180000084599:1453099-1453168:+	ACA1_063580	hypothetical protein	0	0.655813	0.655813	8.35E-13
6h	ACA1_193180/clean/scf7180000084749:206163-206277:+	ACA1_193180	Serine incorporator 3, putative [Source:UniProtKB/TrEMBL;Acc:L8GN74]	0.003027707	0.658634333	0.655606627	3.83E-19
6h	ACA1_039450/clean/scf7180000084569:31765-31840:-	ACA1_039450	SAP domain-containing protein [Source:UniProtKB/TrEMBL;Acc:L8HI99]	0.007790767	0.663390333	0.655599567	6.82E-08
6h	ACA1_169220/clean/scf7180000084739:31092-31182:+	ACA1_169220	ANK_REP_REGION domain-containing protein [Source:UniProtKB/TrEMBL;Acc:L8HFG6]	0.002456077	0.657990667	0.65553459	4.14E-11
6h	ACA1_252800/clean/scf7180000084768:220260-220346:+	ACA1_252800	Dual specificity phosphatase, catalytic domain containing protein [Source:UniProtKB/TrEMBL;Acc:L8H9X0]	0.017751467	0.673237667	0.6554862	2.35E-07
6h	ACA1_279010/clean/scf7180000084776:293222-293302:+	ACA1_279010	LRAT domain-containing protein [Source:UniProtKB/TrEMBL;Acc:L8H710]	0	0.655277667	0.655277667	0.000576022
6h	ACA1_396170/clean/scf7180000084759:44228-44310:-	ACA1_396170	Phosphatidylserine decarboxylase proenzyme 2 [Source:UniProtKB/TrEMBL;Acc:L8HEJ4]	0.034394833	0.689003333	0.6546085	1.59E-35
6h	ACA1_289660/clean/scf7180000084777:439305-439384:+	ACA1_289660	Churchill domain containing 1, putative [Source:UniProtKB/TrEMBL;Acc:L8HKL9]	0.074428333	0.728703667	0.654275333	2.21E-19
6h	ACA1_062280/clean/scf7180000084599:1156724-1156817:-	ACA1_062280	hypothetical protein	0.060492133	0.714755333	0.6542632	3.89E-10
6h	ACA1_025560/clean/scf7180000084448:14754-14846:-	ACA1_025560	hypothetical protein	0	0.653431333	0.653431333	0.000207692
6h	ACA1_163490/clean/scf7180000084729:48425-48479:-	ACA1_163490	hypothetical protein	0.011562067	0.664626667	0.6530646	2.22E-13
6h	ACA1_282500/clean/scf7180000084776:694970-695050:-	ACA1_282500	ANK_REP_REGION domain-containing protein [Source:UniProtKB/TrEMBL;Acc:L8H6H9]	0.00169618	0.654683	0.65298682	1.51E-07
6h	ACA1_382900/clean/scf7180000084721:112168-112290:-	ACA1_382900	ADPribosylation factor, putative [Source:UniProtKB/TrEMBL;Acc:L8GUW5]	0.01523151	0.668166667	0.652935157	4.39E-25
6h	ACA1_376140/clean/scf7180000084685:34335-34541:-	ACA1_376140	AcetylCoA acetyltransferase, mitochondrial, putative [Source:UniProtKB/TrEMBL;Acc:L8HG98]	0.000505817	0.653347667	0.65284185	5.47E-10
6h	ACA1_278900/clean/scf7180000084776:272601-272705:+	ACA1_278900	ANK_REP_REGION domain-containing protein [Source:UniProtKB/TrEMBL;Acc:L8H5W6]	0.00214731	0.654863	0.65271569	3.71E-12
6h	ACA1_072990/clean/scf7180000084603:1350925-1350996:-	ACA1_072990	hypothetical protein	0.042929267	0.695207333	0.652278067	6.57E-08
6h	ACA1_380210/clean/scf7180000084712:32261-32346:-	ACA1_380210	DNA-(apurinic or apyrimidinic site) lyase [Source:UniProtKB/TrEMBL;Acc:L8GQH4]	0.0060606	0.658265667	0.652205067	1.36E-08
6h	ACA1_247950/clean/scf7180000084766:1669-1749:-	ACA1_247950	hypothetical protein	0.0536693	0.705564667	0.651895367	1.54E-18
6h	ACA1_367890/clean/scf7180000084664:9160-9276:-	ACA1_367890	hypothetical protein	0.056893833	0.708618667	0.651724833	3.57E-14
6h	ACA1_322160/clean/scf7180000084459:21575-21642:+	ACA1_322160	AB hydrolase-1 domain-containing protein [Source:UniProtKB/TrEMBL;Acc:L8GMT9]	0.210638333	0.862323333	0.651685	1.40E-15
6h	ACA1_157220/clean/scf7180000084724:11786-11881:+	ACA1_157220	Pro-kuma_activ domain-containing protein [Source:UniProtKB/TrEMBL;Acc:L8H9Y5]	0.0096119	0.661239333	0.651627433	3.99E-08
6h	ACA1_295880/clean/scf7180000084777:1250924-1251027:+	ACA1_295880	Malic enzyme [Source:UniProtKB/TrEMBL;Acc:L8HM80]	0.005563433	0.656967667	0.651404233	2.34E-14
6h	ACA1_191700/clean/scf7180000084749:34786-34872:-	ACA1_191700	Aldo_ket_red domain-containing protein [Source:UniProtKB/TrEMBL;Acc:L8GNA1]	0.080456333	0.730959667	0.650503333	2.40E-12
6h	ACA1_291600/clean/scf7180000084777:784568-784671:-	ACA1_291600	Macro domain-containing protein [Source:UniProtKB/TrEMBL;Acc:L8HJG2]	0.009742173	0.660226667	0.650484493	3.38E-11
6h	ACA1_069120/clean/scf7180000084603:361014-361103:-	ACA1_069120	hypothetical protein	0.004503203	0.654875667	0.650372463	3.86E-09
6h	ACA1_233000/clean/scf7180000084762:98659-98763:-	ACA1_233000	Fatty acid betaoxidation-related protein [Source:UniProtKB/TrEMBL;Acc:L8H2F3]	0.006825513	0.656842667	0.650017153	4.33E-54
6h	ACA1_146620/clean/scf7180000084713:76879-76983:-	ACA1_146620	Membrane protein [Source:UniProtKB/TrEMBL;Acc:L8GVA1]	0.230253667	0.880257	0.650003333	2.20E-09
6h	ACA1_378010/clean/scf7180000084711:163722-163847:+	ACA1_378010	JmjC domain-containing protein [Source:UniProtKB/TrEMBL;Acc:L8GRT4]	0.012482533	0.662476333	0.6499938	3.88E-08
6h	ACA1_006200/clean/scf7180000083029:69118-69193:-	ACA1_006200	hypothetical protein	0.029384533	0.679322333	0.6499378	7.67E-15
6h	ACA1_071170/clean/scf7180000084603:880211-880318:+	ACA1_071170	RhoGAP domain containing protein [Source:UniProtKB/TrEMBL;Acc:L8HGC8]	0	0.649710333	0.649710333	2.39E-12
6h	ACA1_167050/clean/scf7180000084736:61957-62045:-	ACA1_167050	Ras subfamily protein [Source:UniProtKB/TrEMBL;Acc:L8H0S4]	0.0403254	0.689768333	0.649442933	3.83E-11
6h	ACA1_195850/clean/scf7180000084752:35524-35609:+	ACA1_195850	BRO1 domain-containing protein [Source:UniProtKB/TrEMBL;Acc:L8HG99]	0.008519427	0.657935667	0.64941624	1.55E-09
6h	ACA1_323430/clean/scf7180000084468:21313-21415:+	ACA1_323430	SnoaL family polyketide cyclase [Source:UniProtKB/TrEMBL;Acc:L8H0W6]	0.003624157	0.652744	0.649119843	1.39E-30
6h	ACA1_332520/clean/scf7180000084579:35216-35302:+	ACA1_332520	ANK_REP_REGION domain-containing protein [Source:UniProtKB/TrEMBL;Acc:L8GLU6]	0.015437867	0.664330667	0.6488928	7.84E-14
6h	ACA1_365090/clean/scf7180000084641:234290-234365:+	ACA1_365090	ADPribosylglycohydrolase superfamily protein [Source:UniProtKB/TrEMBL;Acc:L8GP97]	0.201504333	0.849647667	0.648143333	1.13E-19
6h	ACA1_062330/clean/scf7180000084599:1166502-1166593:+	ACA1_062330	U-box domain-containing protein [Source:UniProtKB/TrEMBL;Acc:L8GZF7]	0.000241896	0.648348333	0.648106437	3.03E-06
6h	ACA1_385700/clean/scf7180000084730:241285-241343:+	ACA1_385700	F-box domain-containing protein [Source:UniProtKB/TrEMBL;Acc:L8HAP4]	0.047619	0.69548	0.647861	0.000314405
6h	ACA1_131910/clean/scf7180000084699:171925-172008:-	ACA1_131910	EF hand domain containing protein [Source:UniProtKB/TrEMBL;Acc:L8GMI5]	0.00688509	0.654697	0.64781191	4.09E-23
6h	ACA1_251640/clean/scf7180000084768:98620-98709:+	ACA1_251640	CBS domain-containing protein [Source:UniProtKB/TrEMBL;Acc:L8HAQ8]	0.000133896	0.647449	0.647315104	4.21E-13
6h	ACA1_012330/clean/scf7180000083740:16392-16483:+	ACA1_012330	WD_REPEATS_REGION domain-containing protein [Source:UniProtKB/TrEMBL;Acc:L8HCD7]	0.001222033	0.648482	0.647259967	6.41E-14
6h	ACA1_287660/clean/scf7180000084777:57258-57335:+	ACA1_287660	Pentapeptide repeatcontaining protein [Source:UniProtKB/TrEMBL;Acc:L8HIP0]	0.00579397	0.652561	0.64676703	1.59E-12
6h	ACA1_069730/clean/scf7180000084603:538907-538984:+	ACA1_069730	Lysozyme, putative [Source:UniProtKB/TrEMBL;Acc:L8HF46]	0.01174178	0.658499	0.64675722	2.02E-30
6h	ACA1_274930/clean/scf7180000084773:142635-142725:-	ACA1_274930	Histone deacetylaserelated, putative [Source:UniProtKB/TrEMBL;Acc:L8GFN9]	0.018081833	0.664633667	0.646551833	4.20E-21
6h	ACA1_343290/clean/scf7180000084638:15622-15699:+	ACA1_343290	hypothetical protein	0.0158115	0.661865667	0.646054167	1.49E-31
6h	ACA1_156050/clean/scf7180000084723:14447-14504:+	ACA1_156050	Armadillo/betacatenin-like repeat domain containing protein [Source:UniProtKB/TrEMBL;Acc:L8GIE3]	0	0.645637667	0.645637667	0.002711651
6h	ACA1_069350/clean/scf7180000084603:419723-419807:-	ACA1_069350	Mannosyltransferase [Source:UniProtKB/TrEMBL;Acc:L8HFX6]	0.0042735	0.649874667	0.645601167	9.57E-10
6h	ACA1_266230/clean/scf7180000084770:638959-639034:+	ACA1_266230	Dilute domain-containing protein [Source:UniProtKB/TrEMBL;Acc:L8H2D0]	0.077319333	0.722805333	0.645486	1.17E-06
6h	ACA1_064490/clean/scf7180000084599:1628218-1628296:+	ACA1_064490	TYR_PHOSPHATASE_2 domain-containing protein [Source:UniProtKB/TrEMBL;Acc:L8GXK2]	0.0174377	0.662922333	0.645484633	6.12E-09
6h	ACA1_158030/clean/scf7180000084724:186383-186453:-	ACA1_158030	Doublecortin domain-containing protein [Source:UniProtKB/TrEMBL;Acc:L8H983]	0.01634994	0.661627	0.64527706	1.40E-33
6h	ACA1_275770/clean/scf7180000084774:105627-105692:-	ACA1_275770	Twinfilin, putative [Source:UniProtKB/TrEMBL;Acc:L8GS07]	0	0.644860667	0.644860667	1.37E-14
6h	ACA1_127640/clean/scf7180000084696:39029-39136:+	ACA1_127640	GP-PDE domain-containing protein [Source:UniProtKB/TrEMBL;Acc:L8GEW1]	0	0.644498333	0.644498333	7.58E-12
6h	ACA1_216380/clean/scf7180000084758:483140-483253:+	ACA1_216380	Magnesium transporter [Source:UniProtKB/TrEMBL;Acc:L8GSQ2]	0.005442637	0.649904667	0.64446203	1.84E-12
6h	ACA1_186900/clean/scf7180000084746:219860-219973:-	ACA1_186900	Synapsin, ATP binding domain containing protein [Source:UniProtKB/TrEMBL;Acc:L8H7B4]	0.011400367	0.655754	0.644353633	1.78E-07
6h	ACA1_216300/clean/scf7180000084758:461860-462045:+	ACA1_216300	Ras family protein [Source:UniProtKB/TrEMBL;Acc:L8GR60]	0.022222233	0.666497	0.644274767	9.07E-10
6h	ACA1_163630/clean/scf7180000084729:58702-58809:-	ACA1_163630	RNA_lig_T4_1 domain-containing protein [Source:UniProtKB/TrEMBL;Acc:L8GRB2]	0.008066676	0.652240667	0.64417399	1.77E-07
6h	ACA1_289740/clean/scf7180000084777:453772-453865:+	ACA1_289740	Rho-GAP domain-containing protein [Source:UniProtKB/TrEMBL;Acc:L8HLF4]	0.0840064	0.728031	0.6440246	1.73E-11
6h	ACA1_396180/clean/scf7180000084759:46996-47065:-	ACA1_396180	C2 domain-containing protein [Source:UniProtKB/TrEMBL;Acc:L8HC65]	0.000394401	0.644154667	0.643760265	2.49E-18
6h	ACA1_264440/clean/scf7180000084770:287523-287589:+	ACA1_264440	Sulfurtransferase [Source:UniProtKB/TrEMBL;Acc:L8H202]	0.0189378	0.662609	0.6436712	1.18E-17
6h	ACA1_341100/clean/scf7180000084620:51340-51404:-	ACA1_341100	MFS domain-containing protein [Source:UniProtKB/TrEMBL;Acc:L8GR49]	0.172777	0.815829667	0.643052667	9.87E-18
6h	ACA1_176150/clean/scf7180000084741:1135023-1135115:+	ACA1_176150	PhytanoylCoA dioxygenase (PhyH) superfamily protein [Source:UniProtKB/TrEMBL;Acc:L8HI91]	0.0285017	0.671480667	0.642978967	3.18E-16
6h	ACA1_077120/clean/scf7180000084612:240145-240249:-	ACA1_077120	DH domain-containing protein [Source:UniProtKB/TrEMBL;Acc:L8GM77]	0.046495467	0.689455	0.642959533	4.37E-20
6h	ACA1_230510/clean/scf7180000084761:367743-367834:-	ACA1_230510	hypothetical protein	0.003894063	0.646820333	0.64292627	3.32E-12
6h	ACA1_109740/clean/scf7180000084663:15415-15485:-	ACA1_109740	Myblike DNA-binding domain containing protein [Source:UniProtKB/TrEMBL;Acc:L8HJY9]	0	0.642743	0.642743	4.64E-11
6h	ACA1_296250/clean/scf7180000084777:1306313-1306402:+	ACA1_296250	Rasrelated protein Rab-32, putative [Source:UniProtKB/TrEMBL;Acc:L8HJZ6]	0.0681629	0.710835667	0.642672767	7.66E-19
6h	ACA1_110100/clean/scf7180000084663:63653-63772:-	ACA1_110100	Protein kinase [Source:UniProtKB/TrEMBL;Acc:L8HK05]	0	0.642623333	0.642623333	2.27E-11
6h	ACA1_127640/clean/scf7180000084696:39639-39757:+	ACA1_127640	GP-PDE domain-containing protein [Source:UniProtKB/TrEMBL;Acc:L8GEW1]	0.023464933	0.665989	0.642524067	2.66E-12
6h	ACA1_058500/clean/scf7180000084599:334235-334349:-	ACA1_058500	RNA_ligase domain-containing protein [Source:UniProtKB/TrEMBL;Acc:L8GVF6]	0.023333133	0.665639667	0.642306533	6.49E-07
6h	ACA1_277790/clean/scf7180000084776:102377-102471:-	ACA1_277790	Protease, serine, 16 (Thymus), putative [Source:UniProtKB/TrEMBL;Acc:L8H5Q8]	0.001206977	0.642700667	0.64149369	2.21E-14
6h	ACA1_153200/clean/scf7180000084720:84587-84756:+	ACA1_153200	RBR-type E3 ubiquitin transferase [Source:UniProtKB/TrEMBL;Acc:L8HIB3]	0.187242	0.828656333	0.641414333	8.49E-07
6h	ACA1_089980/clean/scf7180000084639:410662-410733:-	ACA1_089980	hypothetical protein	0	0.641412667	0.641412667	9.66E-10
6h	ACA1_266230/clean/scf7180000084770:638288-638370:+	ACA1_266230	Dilute domain-containing protein [Source:UniProtKB/TrEMBL;Acc:L8H2D0]	0.0139457	0.655302667	0.641356967	7.60E-07
6h	ACA1_083640/clean/scf7180000084629:7550-7683:-	ACA1_083640	Elongation factor 1alpha [Source:UniProtKB/TrEMBL;Acc:L8GYH2]	0.008075484	0.649229	0.641153516	2.04E-22
6h	ACA1_228910/clean/scf7180000084761:221754-221849:+	ACA1_228910	hypothetical protein	0.002106373	0.643152333	0.64104596	1.96E-12
6h	ACA1_252460/clean/scf7180000084768:156364-156440:-	ACA1_252460	Isovaleryl Coenzyme A dehydrogenase [Source:UniProtKB/TrEMBL;Acc:L8HD26]	0.002355713	0.643335	0.640979287	3.65E-14
6h	ACA1_295350/clean/scf7180000084777:1110414-1110529:-	ACA1_295350	RNA_bind domain-containing protein [Source:UniProtKB/TrEMBL;Acc:L8HJR1]	0.0459326	0.686810667	0.640878067	1.40E-18
6h	ACA1_219750/clean/scf7180000084758:799849-799936:+	ACA1_219750	FAD_binding_3 domain-containing protein [Source:UniProtKB/TrEMBL;Acc:L8GPZ6]	0.004385967	0.645157333	0.640771367	2.27E-10
6h	ACA1_297090/clean/scf7180000084777:1454490-1454573:-	ACA1_297090	Kelch repeatcontaining protein [Source:UniProtKB/TrEMBL;Acc:L8HK57]	0.042592333	0.683136333	0.640544	3.39E-07
6h	ACA1_061250/clean/scf7180000084599:866748-866812:+	ACA1_061250	Peptidyl-prolyl cis-trans isomerase [Source:UniProtKB/TrEMBL;Acc:L8GWR2]	0.006295453	0.646498333	0.64020288	4.26E-22
6h	ACA1_229570/clean/scf7180000084761:280212-280297:-	ACA1_229570	Oxidoreductase, short chain dehydrogenase/reductase superfamily protein [Source:UniProtKB/TrEMBL;Acc:L8H814]	0.001678632	0.641864333	0.640185701	2.54E-12
6h	ACA1_236680/clean/scf7180000084762:472553-472639:+	ACA1_236680	Membrane protein, putative [Source:UniProtKB/TrEMBL;Acc:L8H1H4]	0	0.640163333	0.640163333	0.005760546
6h	ACA1_360070/clean/scf7180000084622:41510-41584:-	ACA1_360070	Thr_synth_N domain-containing protein [Source:UniProtKB/TrEMBL;Acc:L8HCM4]	0	0.639801667	0.639801667	1.83E-12
6h	ACA1_273780/clean/scf7180000084773:56205-56295:-	ACA1_273780	CP-type G domain-containing protein [Source:UniProtKB/TrEMBL;Acc:L8GI50]	0.003197083	0.642956	0.639758917	7.37E-12
6h	ACA1_214850/clean/scf7180000084758:245642-245741:+	ACA1_214850	LIM zinc-binding domain-containing protein [Source:UniProtKB/TrEMBL;Acc:L8GSG1]	0.029425733	0.669178333	0.6397526	9.59E-19
6h	ACA1_095680/clean/scf7180000084645:878735-878801:+	ACA1_095680	hypothetical protein	0.0381868	0.677906667	0.639719867	6.25E-16
6h	ACA1_066880/clean/scf7180000084600:69990-70100:+	ACA1_066880	hypothetical protein	0.019607833	0.659276667	0.639668833	2.02E-08
6h	ACA1_027030/clean/scf7180000084464:27743-27840:-	ACA1_027030	C2 domain-containing protein [Source:UniProtKB/TrEMBL;Acc:L8GJ80]	0.007995633	0.647011333	0.6390157	2.61E-18
6h	ACA1_140730/clean/scf7180000084707:109354-109458:-	ACA1_140730	Kelch repeat protein [Source:UniProtKB/TrEMBL;Acc:L8GGS1]	0.013534	0.65222	0.638686	1.27E-11
6h	ACA1_385810/clean/scf7180000084730:270939-271053:+	ACA1_385810	Rap-GAP domain-containing protein [Source:UniProtKB/TrEMBL;Acc:L8HAQ1]	0.00506239	0.643653333	0.638590943	1.09E-20
6h	ACA1_218690/clean/scf7180000084758:691064-691176:+	ACA1_218690	1,2-dihydroxy-3-keto-5-methylthiopentene dioxygenase [Source:UniProtKB/TrEMBL;Acc:L8GPW7]	0.005354983	0.643856333	0.63850135	7.77E-22
6h	ACA1_323530/clean/scf7180000084468:42919-42985:+	ACA1_323530	Fbox domain containing protein [Source:UniProtKB/TrEMBL;Acc:L8H0X5]	0.0462745	0.684739	0.6384645	7.75E-07
6h	ACA1_189390/clean/scf7180000084748:24502-24592:-	ACA1_189390	hypothetical protein	0.002070393	0.640455667	0.638385273	1.17E-09
6h	ACA1_103160/clean/scf7180000084653:9834-9913:+	ACA1_103160	PALP domain-containing protein [Source:UniProtKB/TrEMBL;Acc:L8GDL9]	0.006384613	0.644740333	0.63835572	6.61E-12
6h	ACA1_236130/clean/scf7180000084762:412143-412249:+	ACA1_236130	MRG domain-containing protein [Source:UniProtKB/TrEMBL;Acc:L8H1D7]	0.022579533	0.660821667	0.638242133	2.06E-11
6h	ACA1_326880/clean/scf7180000084510:63541-63624:-	ACA1_326880	hypothetical protein	0.012695367	0.650770667	0.6380753	2.75E-13
6h	ACA1_013630/clean/scf7180000083751:14940-15013:-	ACA1_013630	Serine/threonine-protein phosphatase [Source:UniProtKB/TrEMBL;Acc:L8HEX2]	0.003872755	0.641582667	0.637709912	4.17E-18
6h	ACA1_012020/clean/scf7180000083652:3751-3838:-	ACA1_012020	hypothetical protein	0.009854257	0.647379667	0.63752541	1.75E-17
6h	ACA1_355980/clean/scf7180000084728:39846-39939:+	ACA1_355980	Aa_trans domain-containing protein [Source:UniProtKB/TrEMBL;Acc:L8H7L7]	0.006666667	0.644154333	0.637487667	6.47E-10
6h	ACA1_113490/clean/scf7180000084669:294158-294362:-	ACA1_113490	SAM domain-containing protein [Source:UniProtKB/TrEMBL;Acc:L8H3A5]	0.109635333	0.747118333	0.637483	1.01E-12
6h	ACA1_087900/clean/scf7180000084639:127577-127668:-	ACA1_087900	hypothetical protein	0.008771933	0.646015	0.637243067	4.76E-09
6h	ACA1_070970/clean/scf7180000084603:820037-820286:-	ACA1_070970	BTB domain-containing protein [Source:UniProtKB/TrEMBL;Acc:L8HGA8]	0.177426	0.814499333	0.637073333	1.23E-05
6h	ACA1_248600/clean/scf7180000084766:93354-93479:+	ACA1_248600	DUF4114 domain-containing protein [Source:UniProtKB/TrEMBL;Acc:L8GXW7]	0.01891588	0.655987333	0.637071453	1.46E-17
6h	ACA1_175830/clean/scf7180000084741:1070881-1070965:-	ACA1_175830	Mago nashi protein [Source:UniProtKB/TrEMBL;Acc:L8HKH2]	0.00834927	0.645255333	0.636906063	6.26E-29
6h	ACA1_300860/clean/scf7180000083147:9958-10040:+	ACA1_300860	TFIIB-type domain-containing protein [Source:UniProtKB/TrEMBL;Acc:L8HHA5]	0.072407867	0.709199333	0.636791467	1.39E-08
6h	ACA1_108220/clean/scf7180000084661:17262-17336:+	ACA1_108220	hypothetical protein	0.01811	0.654658333	0.636548333	1.29E-08

6h	ACA1_219740/clean/scf7180000084758:797938-798020:+	ACA1_219740	Glucosamine 6-phosphate N-acetyltransferase [Source:UniProtKB/TrEMBL;Acc:L8GRH7]	0.0141893	0.650620667	0.636431367	3.41E-15
6h	ACA1_095060/clean/scf7180000084645:804428-804665:-	ACA1_095060	Sidoreflexin [Source:UniProtKB/TrEMBL;Acc:L8GL14]	0.00837559	0.644526333	0.636150743	7.02E-11
6h	ACA1_068620/clean/scf7180000084603:241137-241216:-	ACA1_068620	hypothetical protein	0	0.635628	0.635628	3.23E-12
6h	ACA1_296570/clean/scf7180000084777:1386553-1386632:+	ACA1_296570	TRANSMEMBRANE PROTEIN, putative [Source:UniProtKB/TrEMBL;Acc:L8HIX9]	0.013529367	0.649022333	0.635492967	8.85E-11
6h	ACA1_252770/clean/scf7180000084768:206448-206539:-	ACA1_252770	Peripla_BP_6 domain-containing protein [Source:UniProtKB/TrEMBL;Acc:L8HAP5]	0	0.635296333	0.635296333	4.34E-13
6h	ACA1_275720/clean/scf7180000084774:92727-92792:+	ACA1_275720	Enolase, Cterminal TIM barrel domain containing protein [Source:UniProtKB/TrEMBL;Acc:L8GRZ9]	0.006655303	0.641931	0.635275697	8.46E-30
6h	ACA1_264180/clean/scf7180000084770:206973-207072:+	ACA1_264180	Oxidoreductase, short chain dehydrogenase/reductase superfamily protein [Source:UniProtKB/TrEMBL;Acc:L8H208]	0.0139228	0.648998333	0.635075533	1.71E-14
6h	ACA1_394730/clean/scf7180000084756:131130-131192:-	ACA1_394730	Putative tyrosineprotein phosphatase oca1, putative [Source:UniProtKB/TrEMBL;Acc:L8H2T3]	0.028714073	0.663744333	0.63503026	1.15E-15
6h	ACA1_358270/clean/scf7180000084742:2246-2350:-	ACA1_358270	Pyr_redox_2 domain-containing protein [Source:UniProtKB/TrEMBL;Acc:L8GL60]	0	0.634788333	0.634788333	8.08E-06
6h	ACA1_213080/clean/scf7180000084758:173868-174005:+	ACA1_213080	hypothetical protein	0.0449166	0.679700667	0.634784067	2.32E-13
6h	ACA1_084920/clean/scf7180000084632:35230-35297:+	ACA1_084920	Phospholipase, putative [Source:UniProtKB/TrEMBL;Acc:L8HJC3]	0.0228926	0.657618667	0.634726067	5.62E-24
6h	ACA1_383660/clean/scf7180000084721:240313-240397:+	ACA1_383660	tRNA (guanine-N(7)-)-methyltransferase non-catalytic subunit [Source:UniProtKB/TrEMBL;Acc:L8GW53]	0.006973697	0.641502	0.634528303	5.08E-11
6h	ACA1_025980/clean/scf7180000084456:1499-1568:-	ACA1_025980	hypothetical protein	0.040991733	0.675508667	0.634516933	1.20E-14
6h	ACA1_199500/clean/scf7180000084753:247190-247293:+	ACA1_199500	1acylglycerophosphocholine O-acyltransferase 1, putative [Source:UniProtKB/TrEMBL;Acc:L8H4L3]	0.2	0.834197	0.634197	7.06E-07
6h	ACA1_007740/clean/scf7180000083166:5829-5922:-	ACA1_007740	RasGEF domain containing protein [Source:UniProtKB/TrEMBL;Acc:L8HBV5]	0.168597667	0.802749	0.634151333	3.58E-11
6h	ACA1_108260/clean/scf7180000084661:22710-22796:-	ACA1_108260	Prenylcys_lyase domain-containing protein [Source:UniProtKB/TrEMBL;Acc:L8GD56]	0.028752423	0.662727333	0.63397491	2.79E-11
6h	ACA1_272530/clean/scf7180000084772:54571-54739:+	ACA1_272530	VWFA domain-containing protein [Source:UniProtKB/TrEMBL;Acc:L8HIL5]	0.0299856	0.663895	0.6339094	6.42E-11
6h	ACA1_025560/clean/scf7180000084448:13829-13904:-	ACA1_025560	hypothetical protein	0.134563867	0.768146	0.633582133	3.09E-06
6h	ACA1_035610/clean/scf7180000084564:98516-98665:+	ACA1_035610	hypothetical protein	0	0.633247	0.633247	2.61E-05
6h	ACA1_209860/clean/scf7180000084757:341281-341374:-	ACA1_209860	Protein kinase domain-containing protein [Source:UniProtKB/TrEMBL;Acc:L8GYD8]	0.005607897	0.638798333	0.633190437	1.62E-10
6h	ACA1_096520/clean/scf7180000084645:1033373-1033467:-	ACA1_096520	Cytochrome p450 superfamily protein [Source:UniProtKB/TrEMBL;Acc:L8GLA2]	0	0.632872667	0.632872667	1.32E-05
6h	ACA1_066420/clean/scf7180000084600:1002-1072:+	ACA1_066420	hypothetical protein	0.272639667	0.905352333	0.632712667	1.29E-15
6h	ACA1_131000/clean/scf7180000084698:147497-147588:-	ACA1_131000	Nudix hydrolase domain-containing protein [Source:UniProtKB/TrEMBL;Acc:L8GPF6]	0.0040681	0.636375	0.6323069	3.43E-09
6h	ACA1_258970/clean/scf7180000084769:330568-330717:-	ACA1_258970	hypothetical protein	0.207627667	0.839486	0.631858333	4.34E-08
6h	ACA1_142370/clean/scf7180000084708:153137-153244:-	ACA1_142370	Serine hydroxymethyltransferase [Source:UniProtKB/TrEMBL;Acc:L8HDT1]	0.000649417	0.632248667	0.63159925	1.32E-18
6h	ACA1_243160/clean/scf7180000084763:324384-324442:+	ACA1_243160	Glyco_trans_2-like domain-containing protein [Source:UniProtKB/TrEMBL;Acc:L8GMD7]	0.244714667	0.875201333	0.630486667	5.04E-20
6h	ACA1_063140/clean/scf7180000084599:1339479-1339561:-	ACA1_063140	MFS domain-containing protein [Source:UniProtKB/TrEMBL;Acc:L8GX17]	0.047619	0.677869333	0.630250333	0.000203315
6h	ACA1_037190/clean/scf7180000084567:80442-80529:+	ACA1_037190	Aminotran_5 domain-containing protein [Source:UniProtKB/TrEMBL;Acc:L8H0V4]	0.046153267	0.676311	0.630157733	2.93E-19
6h	ACA1_072370/clean/scf7180000084603:1212767-1212852:-	ACA1_072370	Leucine rich repeat domain containing protein [Source:UniProtKB/TrEMBL;Acc:L8HEP0]	0.012345667	0.642204667	0.629859	1.92E-06
6h	ACA1_296490/clean/scf7180000084777:1360507-1360647:-	ACA1_296490	Succinate--CoA ligase [ADP-forming] subunit beta, mitochondrial [Source:UniProtKB/TrEMBL;Acc:L8HMD9]	0	0.629837333	0.629837333	8.37E-17
6h	ACA1_264230/clean/scf7180000084770:216003-216090:+	ACA1_264230	ATP/GTPbinding-like protein [Source:UniProtKB/TrEMBL;Acc:L8H212]	0.0089411	0.638638	0.6296969	1.49E-10
6h	ACA1_174050/clean/scf7180000084741:588559-588720:-	ACA1_174050	hypothetical protein	0.009338307	0.63892	0.629581693	2.45E-10
6h	ACA1_264360/clean/scf7180000084770:250014-250079:+	ACA1_264360	hypothetical protein	0.041972767	0.671491667	0.6295189	3.17E-08
6h	ACA1_261480/clean/scf7180000084769:691021-691098:+	ACA1_261480	ATP-dependent (S)-NAD(P)H-hydrate dehydratase [Source:UniProtKB/TrEMBL;Acc:L8GG71]	0.054219533	0.683638667	0.629419133	1.61E-14
6h	ACA1_011490/clean/scf7180000083641:7396-7465:+	ACA1_011490	DH domain-containing protein [Source:UniProtKB/TrEMBL;Acc:L8GZK7]	0.001139387	0.630466667	0.62932728	5.08E-12
6h	ACA1_070500/clean/scf7180000084603:702737-702888:-	ACA1_070500	Ras subfamily protein [Source:UniProtKB/TrEMBL;Acc:L8HDY4]	0.00237017	0.631595667	0.629225497	5.14E-13
6h	ACA1_223350/clean/scf7180000084760:138588-138666:+	ACA1_223350	F-box domain-containing protein [Source:UniProtKB/TrEMBL;Acc:L8GSX8]	0.009505743	0.638418	0.628912257	1.17E-09
6h	ACA1_278900/clean/scf7180000084776:271851-271938:+	ACA1_278900	ANK_REP_REGION domain-containing protein [Source:UniProtKB/TrEMBL;Acc:L8H5W6]	0.000480307	0.629353333	0.628873027	4.40E-13
6h	ACA1_185180/clean/scf7180000084746:81927-82011:-	ACA1_185180	LIM zinc-binding domain-containing protein [Source:UniProtKB/TrEMBL;Acc:L8H575]	0.003584233	0.632204	0.628619767	1.34E-15
6h	ACA1_127620/clean/scf7180000084696:36745-36966:+	ACA1_127620	Fe2OG dioxygenase domain-containing protein [Source:UniProtKB/TrEMBL;Acc:L8GGE8]	0.038494833	0.666983333	0.6284885	2.67E-09
6h	ACA1_018780/clean/scf7180000083912:15861-15963:-	ACA1_018780	AAA domain-containing protein [Source:UniProtKB/TrEMBL;Acc:L8HMV6]	0.011538067	0.639916667	0.6283786	3.78E-13
6h	ACA1_367410/clean/scf7180000084641:627723-627805:-	ACA1_367410	Phosphodiesterase [Source:UniProtKB/TrEMBL;Acc:L8GMB7]	0.007575767	0.635836333	0.628260567	6.60E-09
6h	ACA1_092000/clean/scf7180000084645:243958-244035:-	ACA1_092000	hypothetical protein	0.0573783	0.685223667	0.627845367	1.26E-14
6h	ACA1_164920/clean/scf7180000084729:312405-312467:-	ACA1_164920	DJ-1_PfpI domain-containing protein [Source:UniProtKB/TrEMBL;Acc:L8GRN3]	0	0.627821667	0.627821667	6.71E-17
6h	ACA1_298660/clean/scf7180000082992:14321-14412:+	ACA1_298660	Bromo domain-containing protein [Source:UniProtKB/TrEMBL;Acc:L8H1K3]	0.011651673	0.639455	0.627803327	3.15E-09
6h	ACA1_264270/clean/scf7180000084770:226332-226432:-	ACA1_264270	Oxidoreductase, short chain dehydrogenase/reductase family protein [Source:UniProtKB/TrEMBL;Acc:L8H474]	0.023915333	0.651650667	0.627735333	1.97E-16
6h	ACA1_382380/clean/scf7180000084721:45368-45448:+	ACA1_382380	WD_REPEATS_REGION domain-containing protein [Source:UniProtKB/TrEMBL;Acc:L8GUA6]	0.0047619	0.631664667	0.626902767	3.98E-10
6h	ACA1_203010/clean/scf7180000084754:117069-117184:+	ACA1_203010	RhoGEF domain containing protein [Source:UniProtKB/TrEMBL;Acc:L8GTP3]	0	0.626764333	0.626764333	5.35E-10
6h	ACA1_129920/clean/scf7180000084698:49667-49722:+	ACA1_129920	SET domain-containing protein [Source:UniProtKB/TrEMBL;Acc:L8GPI9]	0.218362667	0.845101333	0.626738667	2.52E-09
6h	ACA1_197880/clean/scf7180000084753:46052-46119:-	ACA1_197880	NTP_transf_2 domain-containing protein [Source:UniProtKB/TrEMBL;Acc:L8H4G5]	0.220819667	0.847441	0.626621333	3.97E-14
6h	ACA1_252040/clean/scf7180000084768:131484-131563:-	ACA1_252040	Maflike protein [Source:UniProtKB/TrEMBL;Acc:L8HAR6]	0.014914922	0.641398667	0.626483744	8.75E-11
6h	ACA1_061010/clean/scf7180000084599:798761-798970:-	ACA1_061010	zf-Tim10_DDP domain-containing protein [Source:UniProtKB/TrEMBL;Acc:L8GXS4]	0.00734742	0.633813333	0.626465913	9.25E-12
6h	ACA1_236690/clean/scf7180000084762:473755-473831:+	ACA1_236690	Peptidyl-prolyl cis-trans isomerase [Source:UniProtKB/TrEMBL;Acc:L8H3U5]	0.002288157	0.628716667	0.62642851	2.85E-15
6h	ACA1_156480/clean/scf7180000084723:44699-44791:+	ACA1_156480	PHB domain-containing protein [Source:UniProtKB/TrEMBL;Acc:L8GK25]	0.003364187	0.629523	0.626158813	1.50E-23
6h	ACA1_180160/clean/scf7180000084744:34572-34703:+	ACA1_180160	hypothetical protein	0.00993325	0.63609	0.62615675	8.02E-11
6h	ACA1_295270/clean/scf7180000084777:1088796-1088877:-	ACA1_295270	Rhodanese domain-containing protein [Source:UniProtKB/TrEMBL;Acc:L8HJQ3]	0.1154595	0.741304333	0.625844833	1.12E-14
6h	ACA1_279800/clean/scf7180000084776:450441-450513:+	ACA1_279800	Peroxisomal biogenesis factor 11c, putative	0	0.625699667	0.625699667	8.80E-14
6h	ACA1_070000/clean/scf7180000084603:617418-617515:+	ACA1_070000	hypothetical protein	0.00883279	0.63435	0.62551721	5.15E-11
6h	ACA1_208280/clean/scf7180000084757:151184-151273:+	ACA1_208280	hypothetical protein	0.0074885	0.632816333	0.625327833	1.24E-12
6h	ACA1_288440/clean/scf7180000084777:207268-207333:-	ACA1_288440	DH domain-containing protein [Source:UniProtKB/TrEMBL;Acc:L8HKE3]	0.009156963	0.634388333	0.62523137	9.99E-09
6h	ACA1_077100/clean/scf7180000084612:235899-235985:-	ACA1_077100	Rab1/RabDfamily small GTPase [Source:UniProtKB/TrEMBL;Acc:L8GNN9]	0.0308913	0.656042667	0.625151367	4.13E-13
6h	ACA1_097540/clean/scf7180000084645:1277344-1277416:-	ACA1_097540	Serine/threonine kinase [Source:UniProtKB/TrEMBL;Acc:L8GK27]	0.0100621	0.635156	0.6250939	1.95E-11
6h	ACA1_193130/clean/scf7180000084749:192717-192794:-	ACA1_193130	Metallophos domain-containing protein [Source:UniProtKB/TrEMBL;Acc:L8GN68]	0.218590667	0.843426	0.624835333	8.30E-16
6h	ACA1_154240/clean/scf7180000084722:29860-29948:-	ACA1_154240	unspecified product	0.012283067	0.636805333	0.624522267	1.74E-08
6h	ACA1_368230/clean/scf7180000084664:85448-85533:-	ACA1_368230	C2 domain-containing protein [Source:UniProtKB/TrEMBL;Acc:L8GYW6]	0.00036996	0.624738333	0.624368373	1.11E-06
6h	ACA1_380750/clean/scf7180000084712:80815-80899:-	ACA1_380750	hypothetical protein	0.011401807	0.635717333	0.624315527	1.75E-13
6h	ACA1_070910/clean/scf7180000084603:799130-799275:-	ACA1_070910	HD domain containing protein [Source:UniProtKB/TrEMBL;Acc:L8HE54]	0.005109067	0.629336	0.624226933	3.39E-13
6h	ACA1_237860/clean/scf7180000084763:33904-34103:+	ACA1_237860	Glycerophosphodiester phosphodiesterase family protein [Source:UniProtKB/TrEMBL;Acc:L8GKN2]	0.0141023	0.638249333	0.624147033	4.60E-23
6h	ACA1_048110/clean/scf7180000084582:24254-24512:-	ACA1_048110	Serine carboxypeptidase s28 protein [Source:UniProtKB/TrEMBL;Acc:L8GD90]	0.015239033	0.639315667	0.624076633	2.89E-24
6h	ACA1_323520/clean/scf7180000084468:41386-41442:-	ACA1_323520	Inosineuridine preferring nucleoside hydrolase [Source:UniProtKB/TrEMBL;Acc:L8H334]	0.0331957	0.656980333	0.623784633	1.81E-14
6h	ACA1_133170/clean/scf7180000084701:14570-14642:+	ACA1_133170	Threonine synthase [Source:UniProtKB/TrEMBL;Acc:L8H3M8]	0.000206654	0.623544	0.623337346	2.17E-12
6h	ACA1_065910/clean/scf7180000084599:1945841-1945919:+	ACA1_065910	hypothetical protein	0.0291841	0.652454	0.6232699	1.32E-09
6h	ACA1_036890/clean/scf7180000084567:7021-7111:+	ACA1_036890	Ras subfamily protein [Source:UniProtKB/TrEMBL;Acc:L8H0T2]	0.0679363	0.690914333	0.622978033	3.79E-13
6h	ACA1_306840/clean/scf7180000083668:17533-17628:+	ACA1_306840	RTP1_C1 domain-containing protein [Source:UniProtKB/TrEMBL;Acc:L8GQF6]	0	0.622913333	0.622913333	1.99E-09
6h	ACA1_379630/clean/scf7180000084712:11567-11662:-	ACA1_379630	Fbox domain containing protein [Source:UniProtKB/TrEMBL;Acc:L8GMS8]	0.002791517	0.625601667	0.62281015	2.59E-11
6h	ACA1_060250/clean/scf7180000084599:626783-626906:-	ACA1_060250	Alpha-carbonic anhydrase domain-containing protein [Source:UniProtKB/TrEMBL;Acc:L8GXK3]	0.018149767	0.640635667	0.6224859	1.97E-30
6h	ACA1_366090/clean/scf7180000084641:346853-346946:-	ACA1_366090	RNA polymerase III subunit C3 [Source:UniProtKB/TrEMBL;Acc:L8GML8]	0.0216129	0.643994667	0.622381767	2.56E-10
6h	ACA1_027030/clean/scf7180000084464:27235-27352:-	ACA1_027030	C2 domain-containing protein [Source:UniProtKB/TrEMBL;Acc:L8GJ80]	0.052472967	0.674823	0.622350033	2.92E-19
6h	ACA1_300850/clean/scf7180000083147:7438-7517:+	ACA1_300850	WD_REPEATS_REGION domain-containing protein [Source:UniProtKB/TrEMBL;Acc:L8HF88]	0.003813157	0.626116667	0.62230351	5.47E-10
6h	ACA1_053360/clean/scf7180000084594:269298-269383:+	ACA1_053360	RING-type domain-containing protein [Source:UniProtKB/TrEMBL;Acc:L8H7D5]	0.016791863	0.639077	0.622285137	4.47E-09
6h	ACA1_193130/clean/scf7180000084749:192862-192958:-	ACA1_193130	Metallophos domain-containing protein [Source:UniProtKB/TrEMBL;Acc:L8GN68]	0.0213181	0.643311	0.6219929	1.44E-12
6h	ACA1_287790/clean/scf7180000084777:79994-80085:-	ACA1_287790	hypothetical protein	0.001988523	0.622894	0.620905477	1.12E-12
6h	ACA1_353190/clean/scf7180000084717:13097-13169:-	ACA1_353190	Cytochrome p450 superfamily protein [Source:UniProtKB/TrEMBL;Acc:L8GGB2]	0.012443547	0.633270333	0.620826787	3.52E-19
6h	ACA1_063530/clean/scf7180000084599:1445944-1446018:+	ACA1_063530	hypothetical protein	0.0075694	0.628064667	0.620495267	2.00E-12
6h	ACA1_293860/clean/scf7180000084777:980224-980374:+	ACA1_293860	Ubiquitin carboxyl-terminal hydrolase [Source:UniProtKB/TrEMBL;Acc:L8HJN0]	0.036106767	0.656404667	0.6202979	1.54E-10
6h	ACA1_385640/clean/scf7180000084730:221950-222051:+	ACA1_385640	hypothetical protein	0.004480247	0.624768667	0.62028842	1.49E-10
6h	ACA1_369880/clean/scf7180000084664:400264-400333:+	ACA1_369880	hypothetical protein	0	0.619817	0.619817	4.48E-13
6h	ACA1_142140/clean/scf7180000084708:94197-94282:+	ACA1_142140	unspecified product	0.005181593	0.624854333	0.61967274	3.71E-10
6h	ACA1_174110/clean/scf7180000084741:614339-614452:+	ACA1_174110	hypothetical protein	0.061361633	0.680906	0.619544367	5.61E-19
6h	ACA1_295300/clean/scf7180000084777:1097247-1097363:-	ACA1_295300	Calponin domain containing protein [Source:UniProtKB/TrEMBL;Acc:L8HJQ6]	0.048256	0.667792667	0.619536667	9.58E-18
6h	ACA1_170940/clean/scf7180000084741:19208-19275:+	ACA1_170940	Proteasome subunit alpha type [Source:UniProtKB/TrEMBL;Acc:L8HH61]	0.003386863	0.622772667	0.619385803	8.74E-41
6h	ACA1_091690/clean/scf7180000084645:181591-181680:+	ACA1_091690	PolyA_pol domain-containing protein [Source:UniProtKB/TrEMBL;Acc:L8GIJ9]	0.162156	0.78134	0.619184	1.99E-07
6h	ACA1_027040/clean/scf7180000084464:30637-30720:-	ACA1_027040	Protein kinase domain-containing protein [Source:UniProtKB/TrEMBL;Acc:L8GGY9]	0.01905325	0.638154333	0.619101083	4.18E-08
6h	ACA1_018120/clean/scf7180000083885:10839-10910:+	ACA1_018120	hypothetical protein	0	0.619096667	0.619096667	6.44E-09
6h	ACA1_237880/clean/scf7180000084763:38355-38479:-	ACA1_237880	ICAT domain-containing protein [Source:UniProtKB/TrEMBL;Acc:L8GJP4]	0.005657033	0.624709	0.619051967	2.44E-26
6h	ACA1_396220/clean/scf7180000084759:56800-56883:+	ACA1_396220	Acyl-coenzyme A oxidase [Source:UniProtKB/TrEMBL;Acc:L8HEJ7]	0.004099333	0.623077	0.618977667	3.05E-14
6h	ACA1_016360/clean/scf7180000083820:5823-5920:+	ACA1_016360	DUF4392 domain-containing protein [Source:UniProtKB/TrEMBL;Acc:L8GQ34]	0.013277017	0.632103667	0.61882665	1.18E-18
6h	ACA1_073410/clean/scf7180000084603:1412452-1412531:-	ACA1_073410	UBX domain-containing protein [Source:UniProtKB/TrEMBL;Acc:L8HEN7]	0	0.618748	0.618748	1.09E-08
6h	ACA1_263870/clean/scf7180000084770:124823-124911:-	ACA1_263870	MAGE domain-containing protein [Source:UniProtKB/TrEMBL;Acc:L8H1Y1]	0.031746033	0.650245333	0.6184993	1.88E-06
6h	ACA1_230570/clean/scf7180000084761:380667-380763:-	ACA1_230570	DUF4139 domain-containing protein [Source:UniProtKB/TrEMBL;Acc:L8H928]	0.002057613	0.620535667	0.618478053	3.96E-12
6h	ACA1_024240/clean/scf7180000084441:23869-23950:-	ACA1_024240	Yip1 domain containing protein [Source:UniProtKB/TrEMBL;Acc:L8GN09]	0.005636167	0.623744667	0.6181085	3.82E-16
6h	ACA1_185180/clean/scf7180000084746:81730-81836:-	ACA1_185180	LIM zinc-binding domain-containing protein [Source:UniProtKB/TrEMBL;Acc:L8H575]	0.001471307	0.619528	0.618056693	2.78E-15
6h	ACA1_031540/clean/scf7180000084522:6395-6502:+	ACA1_031540	CBS domain containing protein [Source:UniProtKB/TrEMBL;Acc:L8GSM4]	0.047125767	0.665011	0.617885233	3.22E-10
6h	ACA1_144350/clean/scf7180000084709:212705-212787:+	ACA1_144350	CN hydrolase domain-containing protein [Source:UniProtKB/TrEMBL;Acc:L8HEV4]	0.0836836	0.701331333	0.617647733	1.09E-14
6h	ACA1_155710/clean/scf7180000084722:223275-223352:-	ACA1_155710	TAF6_C domain-containing protein [Source:UniProtKB/TrEMBL;Acc:L8GZG8]	0.00106813	0.618581	0.61751287	6.57E-12
6h	ACA1_236130/clean/scf7180000084762:411969-412058:+	ACA1_236130	MRG domain-containing protein [Source:UniProtKB/TrEMBL;Acc:L8H1D7]	0.01231616	0.629604333	0.617288173	3.62E-11
6h	ACA1_174820/clean/scf7180000084741:805494-805556:+	ACA1_174820	hypothetical protein	0.0246151	0.641789333	0.617174233	2.13E-20
6h	ACA1_025560/clean/scf7180000084448:14048-14122:-	ACA1_025560	hypothetical protein	0.00103842	0.618176333	0.617137913	0.000193326
6h	ACA1_087720/clean/scf7180000084639:105672-105743:+	ACA1_087720	Dihydrodipicolinate synthetase [Source:UniProtKB/TrEMBL;Acc:L8GVJ3]	0.003344167	0.620464333	0.617120167	3.15E-09
6h	ACA1_266370/clean/scf7180000084770:671565-671664:+	ACA1_266370	Methyltransf_11 domain-containing protein [Source:UniProtKB/TrEMBL;Acc:L8H4L5]	0.093659933	0.710654667	0.616994733	1.29E-05
6h	ACA1_369270/clean/scf7180000084664:286599-286678:+	ACA1_369270	Rasrelated protein Rab-18-like, putative [Source:UniProtKB/TrEMBL;Acc:L8GYY7]	0	0.616780333	0.616780333	5.67E-15
6h	ACA1_118750/clean/scf7180000084671:18638-18714:-	ACA1_118750	Threonylcarbamoyl-AMP synthase [Source:UniProtKB/TrEMBL;Acc:L8GLU7]	0	0.616742	0.616742	8.36E-09
6h	ACA1_058340/clean/scf7180000084599:302546-302629:-	ACA1_058340	DBR1 domain-containing protein [Source:UniProtKB/TrEMBL;Acc:L8GX89]	0	0.616648333	0.616648333	8.96E-11
6h	ACA1_050150/clean/scf7180000084589:42556-42627:+	ACA1_050150	Glutathione Stransferase [Source:UniProtKB/TrEMBL;Acc:L8H557]	0.314478	0.930911667	0.616433667	0.002168449
6h	ACA1_122710/clean/scf7180000084682:151823-151897:-	ACA1_122710	hypothetical protein	0.0273664	0.643706	0.6163396	5.49E-21
6h	ACA1_193130/clean/scf7180000084749:192351-192441:-	ACA1_193130	Metallophos domain-containing protein [Source:UniProtKB/TrEMBL;Acc:L8GN68]	0.091052833	0.707338	0.616285167	2.15E-14
6h	ACA1_392570/clean/scf7180000084740:297087-297206:-	ACA1_392570	hypothetical protein	0	0.616285	0.616285	3.38E-11
6h	ACA1_382360/clean/scf7180000084721:37875-37972:+	ACA1_382360	F-box domain-containing protein [Source:UniProtKB/TrEMBL;Acc:L8GV11]	0.039214667	0.655396333	0.616181667	9.89E-20
6h	ACA1_152970/clean/scf7180000084720:43703-43833:-	ACA1_152970	SET family protein [Source:UniProtKB/TrEMBL;Acc:L8HHE2]	0.0234288	0.639526333	0.616097533	7.14E-24
6h	ACA1_063560/clean/scf7180000084599:1449774-1449836:+	ACA1_063560	hypothetical protein	0.008298034	0.624372333	0.616074299	2.30E-17
6h	ACA1_203000/clean/scf7180000084754:115272-115368:+	ACA1_203000	PGM1_C domain-containing protein [Source:UniProtKB/TrEMBL;Acc:L8GW29]	0.0159447	0.631994333	0.616049633	2.48E-08
6h	ACA1_103160/clean/scf7180000084653:10002-10093:+	ACA1_103160	PALP domain-containing protein [Source:UniProtKB/TrEMBL;Acc:L8GDL9]	0.01108613	0.627117	0.61603087	6.70E-16
6h	ACA1_201010/clean/scf7180000084753:462387-462451:+	ACA1_201010	Fe2OG dioxygenase domain-containing protein [Source:UniProtKB/TrEMBL;Acc:L8H2K6]	0.1396341	0.755473667	0.615839567	3.39E-11
6h	ACA1_154200/clean/scf7180000084722:21233-21309:-	ACA1_154200	Peptidase S53 domain-containing protein [Source:UniProtKB/TrEMBL;Acc:L8GZY7]	0.000379307	0.616072333	0.615693027	2.24E-14
6h	ACA1_208320/clean/scf7180000084757:161025-161086:-	ACA1_208320	ADPribosylglycohydrolase superfamily protein [Source:UniProtKB/TrEMBL;Acc:L8GXF3]	0	0.615613	0.615613	1.71E-08
6h	ACA1_376490/clean/scf7180000084685:90956-91024:-	ACA1_376490	TIP41like family protein [Source:UniProtKB/TrEMBL;Acc:L8HIG8]	0.0447791	0.660242333	0.615463233	2.94E-17
6h	ACA1_140520/clean/scf7180000084707:79757-79833:+	ACA1_140520	hypothetical protein	0.153397	0.768842667	0.615445667	0.000312872
6h	ACA1_106330/clean/scf7180000084660:86277-86363:-	ACA1_106330	Methyltransf_11 domain-containing protein [Source:UniProtKB/TrEMBL;Acc:L8GMT5]	0.01467455	0.629911	0.61523645	2.36E-18
6h	ACA1_012980/clean/scf7180000083742:15602-15666:-	ACA1_012980	Endo/exonuclease/phosphatase domain-containing protein [Source:UniProtKB/TrEMBL;Acc:L8H4W5]	0.009009	0.623890333	0.614881333	2.50E-08
6h	ACA1_061890/clean/scf7180000084599:1071854-1071943:-	ACA1_061890	DHO_dh domain-containing protein [Source:UniProtKB/TrEMBL;Acc:L8GWX2]	0.004068667	0.618709	0.614640333	1.50E-08
6h	ACA1_376490/clean/scf7180000084685:90440-90523:-	ACA1_376490	TIP41like family protein [Source:UniProtKB/TrEMBL;Acc:L8HIG8]	0.028527067	0.642952	0.614424933	5.47E-16
6h	ACA1_070900/clean/scf7180000084603:797496-797640:+	ACA1_070900	MMtag domain-containing protein [Source:UniProtKB/TrEMBL;Acc:L8HD49]	0.006500443	0.620825333	0.61432489	3.03E-10
6h	ACA1_012040/clean/scf7180000083652:9387-9539:+	ACA1_012040	LIM domain containing protein [Source:UniProtKB/TrEMBL;Acc:L8GI34]	0.041672267	0.655468333	0.613796067	1.04E-17
6h	ACA1_363780/clean/scf7180000084641:13637-13723:+	ACA1_363780	Vtype ATPase, D subunit [Source:UniProtKB/TrEMBL;Acc:L8GM99]	0.004465467	0.617924	0.613458533	1.03E-33
6h	ACA1_388680/clean/scf7180000084733:147129-147210:-	ACA1_388680	Frizzled/Smoothened family membrane region protein [Source:UniProtKB/TrEMBL;Acc:L8GEN4]	0.109019033	0.722443	0.613423967	7.53E-12
6h	ACA1_250310/clean/scf7180000084767:47140-47204:-	ACA1_250310	Serine/threonine protein kinase [Source:UniProtKB/TrEMBL;Acc:L8HD37]	0.037928	0.651293667	0.613365667	3.46E-05
6h	ACA1_264460/clean/scf7180000084770:294313-294427:-	ACA1_264460	GTPbinding protein [Source:UniProtKB/TrEMBL;Acc:L8H231]	0.076561067	0.689910667	0.6133496	3.30E-15
6h	ACA1_177720/clean/scf7180000084743:114016-114083:-	ACA1_177720	PARP-type domain-containing protein [Source:UniProtKB/TrEMBL;Acc:L8GU65]	0.007535133	0.620661333	0.6131262	1.18E-11

6h	ACA1_267310/clean/scf7180000084770:854160-854312:-	ACA1_267310	PHDfinger domain containing protein [Source:UniProtKB/TrEMBL;Acc:L8H2K2]	0	0.612827	0.612827	1.02E-08
6h	ACA1_382640/clean/scf7180000084721:94859-95090:+	ACA1_382640	von Willebrand factor type A domain containing protein [Source:UniProtKB/TrEMBL;Acc:L8GUB9]	0.046901333	0.659681333	0.61278	0.011233192
6h	ACA1_043320/clean/scf7180000084573:164658-164730:+	ACA1_043320	Ubiquitin-like domain-containing protein [Source:UniProtKB/TrEMBL;Acc:L8GXV7]	0	0.612623	0.612623	0.000157905
6h	ACA1_131240/clean/scf7180000084699:37234-37314:+	ACA1_131240	Alpha-galactosidase [Source:UniProtKB/TrEMBL;Acc:L8GN31]	0.0498103	0.662378	0.6125677	8.17E-17
6h	ACA1_093770/clean/scf7180000084645:642070-642184:+	ACA1_093770	hypothetical protein	0.225714	0.838048	0.612334	1.19E-14
6h	ACA1_068270/clean/scf7180000084603:153550-153651:-	ACA1_068270	Phospholipid-transporting ATPase [Source:UniProtKB/TrEMBL;Acc:L8HEP8]	0.007246367	0.619557667	0.6123113	1.78E-08
6h	ACA1_397190/clean/scf7180000084759:167925-168044:+	ACA1_397190	Metallophos domain-containing protein [Source:UniProtKB/TrEMBL;Acc:L8HDM9]	0.002583786	0.614457	0.611873214	7.99E-20
6h	ACA1_182990/clean/scf7180000084745:306895-306986:-	ACA1_182990	Alkaline phosphatase D domain containing protein [Source:UniProtKB/TrEMBL;Acc:L8H874]	0.018392613	0.630200333	0.61180772	3.33E-18
6h	ACA1_265560/clean/scf7180000084770:510918-511000:+	ACA1_265560	RFX-type winged-helix domain-containing protein [Source:UniProtKB/TrEMBL;Acc:L8H4F0]	0.012888497	0.624539667	0.61165117	8.57E-12
6h	ACA1_212240/clean/scf7180000084758:140808-140882:+	ACA1_212240	Esterase/lipase ( ), putative [Source:UniProtKB/TrEMBL;Acc:L8GQU1]	0.002380953	0.613906333	0.61152538	5.73E-12
6h	ACA1_298630/clean/scf7180000082992:5660-5755:-	ACA1_298630	Vacuolar protein sorting 16, putative [Source:UniProtKB/TrEMBL;Acc:L8H081]	0	0.611392	0.611392	6.32E-14
6h	ACA1_123630/clean/scf7180000084686:46279-46384:-	ACA1_123630	hypothetical protein	0.113738933	0.725035	0.611296067	2.15E-10
6h	ACA1_024020/clean/scf7180000084440:7262-7342:-	ACA1_024020	RhoGAP domain containing protein [Source:UniProtKB/TrEMBL;Acc:L8GJP9]	0	0.610758667	0.610758667	3.43E-12
6h	ACA1_360090/clean/scf7180000084622:48555-48653:-	ACA1_360090	hypothetical protein	0.039508967	0.650178667	0.6106697	4.10E-13
6h	ACA1_331240/clean/scf7180000084575:10130-10195:-	ACA1_331240	Fe2OG dioxygenase domain-containing protein [Source:UniProtKB/TrEMBL;Acc:L8GVM3]	0.147063667	0.757595333	0.610531667	1.10E-08
6h	ACA1_265420/clean/scf7180000084770:465875-465937:+	ACA1_265420	hypothetical protein	0.076990637	0.687474	0.610483363	6.32E-11
6h	ACA1_170960/clean/scf7180000084741:22768-22892:+	ACA1_170960	hypothetical protein	0.005044877	0.615409667	0.61036479	5.84E-09
6h	ACA1_182530/clean/scf7180000084745:210813-210892:+	ACA1_182530	PUM-HD domain-containing protein [Source:UniProtKB/TrEMBL;Acc:L8H849]	0	0.610243667	0.610243667	6.42E-17
6h	ACA1_146620/clean/scf7180000084713:76617-76692:-	ACA1_146620	Membrane protein [Source:UniProtKB/TrEMBL;Acc:L8GVA1]	0.00389188	0.613611	0.60971912	3.43E-11
6h	ACA1_376270/clean/scf7180000084685:67757-67830:+	ACA1_376270	Phospho-2-dehydro-3-deoxyheptonate aldolase [Source:UniProtKB/TrEMBL;Acc:L8HFZ7]	0.00613785	0.615827333	0.609689483	6.25E-12
6h	ACA1_210540/clean/scf7180000084758:52571-52660:-	ACA1_210540	ABC transporter, ATPbinding domain containing protein [Source:UniProtKB/TrEMBL;Acc:L8GPZ1]	0.0897839	0.699429667	0.609645767	2.07E-12
6h	ACA1_394750/clean/scf7180000084756:135738-135804:-	ACA1_394750	PH domain containing protein [Source:UniProtKB/TrEMBL;Acc:L8H1R2]	0.030012333	0.639562	0.609549667	1.24E-12
6h	ACA1_265380/clean/scf7180000084770:454661-454742:-	ACA1_265380	Phosphatidylinositol glycan anchor biosynthesis, class S, putative [Source:UniProtKB/TrEMBL;Acc:L8H3M4]	0.018720534	0.628257333	0.6095368	9.31E-13
6h	ACA1_153600/clean/scf7180000084720:136063-136179:-	ACA1_153600	tRNA pseudouridine synthase [Source:UniProtKB/TrEMBL;Acc:L8HG78]	0.175605	0.784806	0.609201	1.16E-08
6h	ACA1_388780/clean/scf7180000084733:169440-169528:+	ACA1_388780	EGFlike domain containing protein [Source:UniProtKB/TrEMBL;Acc:L8GDT8]	0.002896039	0.611646	0.608749961	8.69E-14
6h	ACA1_156720/clean/scf7180000084723:58346-58452:+	ACA1_156720	INTRADIOL_DIOXYGENAS domain-containing protein [Source:UniProtKB/TrEMBL;Acc:L8GI57]	0.02394988	0.632620333	0.608670453	7.41E-13
6h	ACA1_280690/clean/scf7180000084776:525576-525659:+	ACA1_280690	AP_endonuc_2 domain-containing protein [Source:UniProtKB/TrEMBL;Acc:L8H794]	0.022645733	0.631093333	0.6084476	6.33E-07
6h	ACA1_287790/clean/scf7180000084777:80215-80303:-	ACA1_287790	hypothetical protein	0.017542533	0.625724	0.608181467	2.69E-16
6h	ACA1_274930/clean/scf7180000084773:142362-142465:-	ACA1_274930	Histone deacetylaserelated, putative [Source:UniProtKB/TrEMBL;Acc:L8GFN9]	0.118861333	0.726909333	0.608048	6.26E-16
6h	ACA1_199750/clean/scf7180000084753:319007-319075:-	ACA1_199750	hypothetical protein	0.005330067	0.613313667	0.6079836	6.67E-11
6h	ACA1_204620/clean/scf7180000084754:322549-322759:+	ACA1_204620	hypothetical protein	0.100007433	0.707981333	0.6079739	3.43E-11
6h	ACA1_039980/clean/scf7180000084571:7590-7679:+	ACA1_039980	DSBA domain-containing protein [Source:UniProtKB/TrEMBL;Acc:L8GGU1]	0	0.607920333	0.607920333	6.92E-15
6h	ACA1_089980/clean/scf7180000084639:410886-410951:-	ACA1_089980	hypothetical protein	0	0.607918333	0.607918333	4.25E-09
6h	ACA1_112080/clean/scf7180000084669:101717-102431:+	ACA1_112080	hypothetical protein	0.009511983	0.617305667	0.607793683	1.28E-12
6h	ACA1_390570/clean/scf7180000084740:74970-75038:-	ACA1_390570	Acetyl/propionyl CoA carboxylase, beta subunit, putative [Source:UniProtKB/TrEMBL;Acc:L8GRH2]	0.0946855	0.702086333	0.607400833	2.84E-13
6h	ACA1_182190/clean/scf7180000084745:150598-150674:-	ACA1_182190	hypothetical protein	0.0560573	0.663397333	0.607340033	3.49E-15
6h	ACA1_264550/clean/scf7180000084770:319649-319732:+	ACA1_264550	Cys/Met metabolism PLPdependent enzyme superfamily protein [Source:UniProtKB/TrEMBL;Acc:L8H496]	0.012814747	0.61974	0.606925253	1.29E-13
6h	ACA1_057790/clean/scf7180000084599:179391-179504:+	ACA1_057790	Proteasome subunit beta [Source:UniProtKB/TrEMBL;Acc:L8GX50]	0.005728017	0.61255	0.606821983	1.93E-28
6h	ACA1_097190/clean/scf7180000084645:1182307-1182396:-	ACA1_097190	JmjC domain-containing protein [Source:UniProtKB/TrEMBL;Acc:L8GIZ2]	0.068291567	0.6751	0.606808433	6.29E-08
6h	ACA1_252580/clean/scf7180000084768:184710-184800:+	ACA1_252580	AAA domain-containing protein [Source:UniProtKB/TrEMBL;Acc:L8HC38]	0	0.606790333	0.606790333	2.66E-09
6h	ACA1_259480/clean/scf7180000084769:348383-348468:-	ACA1_259480	EnoylCoA hydratase/isomerase [Source:UniProtKB/TrEMBL;Acc:L8GFL3]	0.098535067	0.705145333	0.606610267	4.16E-11
6h	ACA1_122360/clean/scf7180000084682:131950-132030:+	ACA1_122360	PH domain-containing protein [Source:UniProtKB/TrEMBL;Acc:L8GF24]	0	0.606243333	0.606243333	5.58E-10
6h	ACA1_325170/clean/scf7180000084475:15371-15565:-	ACA1_325170	MHD domain-containing protein [Source:UniProtKB/TrEMBL;Acc:L8GPP2]	0.0015015	0.607161667	0.605660167	1.80E-13
6h	ACA1_129350/clean/scf7180000084698:9563-9631:+	ACA1_129350	Fatty acid hydroxylase domain-containing protein [Source:UniProtKB/TrEMBL;Acc:L8GPA5]	0.014620877	0.620223667	0.60560279	4.35E-17
6h	ACA1_133550/clean/scf7180000084701:79089-79146:+	ACA1_133550	Lactamase_B domain-containing protein [Source:UniProtKB/TrEMBL;Acc:L8H3M7]	0.056332733	0.661914	0.605581267	2.00E-12
6h	ACA1_048110/clean/scf7180000084582:24054-24142:-	ACA1_048110	Serine carboxypeptidase s28 protein [Source:UniProtKB/TrEMBL;Acc:L8GD90]	0.003939683	0.609492667	0.605552983	1.61E-14
6h	ACA1_369880/clean/scf7180000084664:400671-400766:+	ACA1_369880	hypothetical protein	0.005555567	0.611087667	0.6055321	6.71E-10
6h	ACA1_341080/clean/scf7180000084620:46511-46581:+	ACA1_341080	RING-type domain-containing protein [Source:UniProtKB/TrEMBL;Acc:L8GS99]	0.0751052	0.68039	0.6052848	7.93E-16
6h	ACA1_088880/clean/scf7180000084639:253988-254051:-	ACA1_088880	OGFr_N domain-containing protein [Source:UniProtKB/TrEMBL;Acc:L8GUP7]	0.082277933	0.687525667	0.605247733	5.12E-06
6h	ACA1_295560/clean/scf7180000084777:1172523-1172637:-	ACA1_295560	hypothetical protein	0.033402867	0.638640667	0.6052378	1.48E-25
6h	ACA1_184200/clean/scf7180000084745:582319-582381:-	ACA1_184200	hypothetical protein	0	0.604736333	0.604736333	0.000829279
6h	ACA1_157940/clean/scf7180000084724:155453-155550:-	ACA1_157940	Histidine acid phosphatase superfamily protein [Source:UniProtKB/TrEMBL;Acc:L8H9Z4]	0.003653893	0.608301667	0.604647773	3.57E-22
6h	ACA1_097640/clean/scf7180000084645:1304418-1304521:+	ACA1_097640	Fbox domain containing protein [Source:UniProtKB/TrEMBL;Acc:L8GK52]	0.052659267	0.657055333	0.604396067	1.08E-15
6h	ACA1_070380/clean/scf7180000084603:660541-660606:+	ACA1_070380	RhoGEF domain containing protein [Source:UniProtKB/TrEMBL;Acc:L8HE08]	0.093950033	0.698302333	0.6043523	0.014342983
6h	ACA1_118770/clean/scf7180000084671:29500-29586:-	ACA1_118770	Transmembrane protein 144 family protein [Source:UniProtKB/TrEMBL;Acc:L8GKD1]	0	0.604138	0.604138	0.026477291
6h	ACA1_376200/clean/scf7180000084685:50220-50304:+	ACA1_376200	Kelch repeat protein [Source:UniProtKB/TrEMBL;Acc:L8HGA3]	0.093734233	0.697802667	0.604068433	4.33E-08
6h	ACA1_200220/clean/scf7180000084753:416908-416993:+	ACA1_200220	FBPase domain-containing protein [Source:UniProtKB/TrEMBL;Acc:L8H3E2]	0.008624227	0.612619	0.603994773	1.42E-15
6h	ACA1_182860/clean/scf7180000084745:267911-267998:-	ACA1_182860	Ankyrin repeat protein, putative [Source:UniProtKB/TrEMBL;Acc:L8H7X7]	0.0081339	0.612107333	0.603973433	2.79E-07
6h	ACA1_130470/clean/scf7180000084698:82042-82104:+	ACA1_130470	Carbonic anhydrase [Source:UniProtKB/TrEMBL;Acc:L8GPJ9]	0	0.603923667	0.603923667	4.11E-06
6h	ACA1_275770/clean/scf7180000084774:105292-105429:-	ACA1_275770	Twinfilin, putative [Source:UniProtKB/TrEMBL;Acc:L8GS07]	0.011491467	0.615392667	0.6039012	6.83E-16
6h	ACA1_271330/clean/scf7180000084771:21073-21211:+	ACA1_271330	hypothetical protein	0.002369053	0.60591	0.603540947	4.20E-11
6h	ACA1_138500/clean/scf7180000084703:153574-153798:-	ACA1_138500	UBIQUITIN_CONJUGAT_2 domain-containing protein [Source:UniProtKB/TrEMBL;Acc:L8H0X9]	0.025021467	0.628556	0.603534533	7.00E-15
6h	ACA1_287350/clean/scf7180000084777:13931-14004:-	ACA1_287350	His Kinase A (Phosphoacceptor) domain containing protein [Source:UniProtKB/TrEMBL;Acc:L8HKZ2]	0.041666667	0.645107	0.603440333	0.000103568
6h	ACA1_072660/clean/scf7180000084603:1247629-1247710:+	ACA1_072660	hypothetical protein	0	0.603414333	0.603414333	0.000829421
6h	ACA1_114600/clean/scf7180000084669:519964-520122:+	ACA1_114600	hypothetical protein	0.006724967	0.610074667	0.6033497	4.24E-10
6h	ACA1_173880/clean/scf7180000084741:540111-540182:+	ACA1_173880	hypothetical protein	0.057489567	0.660780333	0.603290767	1.12E-15
6h	ACA1_156050/clean/scf7180000084723:13246-13325:+	ACA1_156050	Armadillo/betacatenin-like repeat domain containing protein [Source:UniProtKB/TrEMBL;Acc:L8GIE3]	0	0.603276	0.603276	0.005035725
6h	ACA1_172940/clean/scf7180000084741:366868-367025:+	ACA1_172940	Protein kinase domain-containing protein [Source:UniProtKB/TrEMBL;Acc:L8HHJ2]	0.006029047	0.609027333	0.602998287	6.47E-07
6h	ACA1_325610/clean/scf7180000084495:50432-50509:-	ACA1_325610	Protein kinase domain containing protein [Source:UniProtKB/TrEMBL;Acc:L8H900]	0.035807167	0.638645	0.602837833	1.96E-14
6h	ACA1_174410/clean/scf7180000084741:700522-700584:-	ACA1_174410	Nucleoside diphosphate kinase [Source:UniProtKB/TrEMBL;Acc:L8HHW4]	0.018939567	0.621714	0.602774433	4.29E-11
6h	ACA1_119430/clean/scf7180000084672:66464-66597:+	ACA1_119430	RRM domain-containing protein [Source:UniProtKB/TrEMBL;Acc:L8HKC8]	0.000497868	0.603201	0.602703132	1.91E-15
6h	ACA1_098910/clean/scf7180000084646:61019-61121:-	ACA1_098910	OGFr_N domain-containing protein [Source:UniProtKB/TrEMBL;Acc:L8H4R8]	0.010103398	0.612802667	0.602699269	1.09E-14
6h	ACA1_191520/clean/scf7180000084749:10693-10860:-	ACA1_191520	GDP-Man:Man(3)GlcNAc(2)-PP-Dol alpha-1,2-mannosyltransferase [Source:UniProtKB/TrEMBL;Acc:L8GNP5]	0.0190736	0.621482333	0.602408733	6.87E-08
6h	ACA1_199520/clean/scf7180000084753:254433-254519:+	ACA1_199520	hypothetical protein	0.021738567	0.624133333	0.602394767	2.07E-15
6h	ACA1_255500/clean/scf7180000084768:515873-516003:+	ACA1_255500	Peptidase C83 domain-containing protein [Source:UniProtKB/TrEMBL;Acc:L8HB52]	0.02663658	0.629019333	0.602382753	1.55E-13
6h	ACA1_396920/clean/scf7180000084759:149436-149523:-	ACA1_396920	Guanylate kinase-like domain-containing protein [Source:UniProtKB/TrEMBL;Acc:L8HEM5]	0.005217427	0.607545	0.602327573	1.15E-33
6h	ACA1_063750/clean/scf7180000084599:1496469-1496536:+	ACA1_063750	TauD domain-containing protein [Source:UniProtKB/TrEMBL;Acc:L8GZU6]	0.119301767	0.721521667	0.6022199	2.70E-06
6h	ACA1_070540/clean/scf7180000084603:717753-717904:+	ACA1_070540	Rab-GAP TBC domain-containing protein [Source:UniProtKB/TrEMBL;Acc:L8HG76]	0.0128205	0.614794	0.6019735	6.36E-07
6h	ACA1_027030/clean/scf7180000084464:26788-26853:-	ACA1_027030	C2 domain-containing protein [Source:UniProtKB/TrEMBL;Acc:L8GJ80]	0.014514503	0.616462333	0.60194783	1.28E-21
6h	ACA1_061150/clean/scf7180000084599:837026-837153:-	ACA1_061150	UBA/TSN domain containing protein [Source:UniProtKB/TrEMBL;Acc:L8GWQ2]	0.019479533	0.621395	0.601915467	1.40E-11
6h	ACA1_360980/clean/scf7180000084622:214564-214645:+	ACA1_360980	hypothetical protein	0	0.60189	0.60189	9.81E-09
6h	ACA1_368080/clean/scf7180000084664:49179-49280:-	ACA1_368080	hypothetical protein	0.020833333	0.622201	0.601367667	2.92E-05
6h	ACA1_081100/clean/scf7180000084618:90457-90538:-	ACA1_081100	Prephenate dehydratase [Source:UniProtKB/TrEMBL;Acc:L8HE69]	0.009018767	0.610369333	0.601350567	3.46E-08
6h	ACA1_183740/clean/scf7180000084745:493341-493409:+	ACA1_183740	hypothetical protein	0.013816833	0.615149667	0.601332833	7.33E-07
6h	ACA1_277990/clean/scf7180000084776:149339-149419:+	ACA1_277990	Calponin domain containing protein [Source:UniProtKB/TrEMBL;Acc:L8H800]	0.071161	0.672239	0.601078	0.001361366
6h	ACA1_015480/clean/scf7180000083799:1937-2030:+	ACA1_015480	hypothetical protein	0.058727233	0.659748333	0.6010211	2.01E-13
6h	ACA1_051660/clean/scf7180000084592:44893-44955:-	ACA1_051660	DNA repair and recombination protein RAD54B, putative [Source:UniProtKB/TrEMBL;Acc:L8GQK0]	0.009803933	0.610675333	0.6008714	3.51E-07
6h	ACA1_342400/clean/scf7180000084633:17539-17635:+	ACA1_342400	Tubulin domain-containing protein [Source:UniProtKB/TrEMBL;Acc:L8HFG0]	0	0.600830333	0.600830333	5.49E-08
6h	ACA1_260520/clean/scf7180000084769:492333-492443:-	ACA1_260520	Bromodomain containing protein [Source:UniProtKB/TrEMBL;Acc:L8GG22]	0.0060293	0.606839	0.6008097	1.65E-10
6h	ACA1_114590/clean/scf7180000084669:517662-517781:-	ACA1_114590	Proteasome activator protein [Source:UniProtKB/TrEMBL;Acc:L8H6L8]	0.0263453	0.627152667	0.600807367	1.31E-17
6h	ACA1_230440/clean/scf7180000084761:349024-349125:+	ACA1_230440	WD_REPEATS_REGION domain-containing protein [Source:UniProtKB/TrEMBL;Acc:L8H839]	0	0.600638	0.600638	1.97E-09
6h	ACA1_321440/clean/scf7180000084439:30739-30833:+	ACA1_321440	hypothetical protein	0.00364035	0.604165333	0.600524983	1.12E-11
6h	ACA1_367180/clean/scf7180000084641:562268-562320:-	ACA1_367180	Profilin [Source:UniProtKB/TrEMBL;Acc:L8GM22]	0.013044253	0.613386333	0.60034208	1.37E-08
6h	ACA1_321060/clean/scf7180000084435:30853-31235:-	ACA1_321060	hypothetical protein	0.215622667	0.815878333	0.600255667	1.50E-12
6h	ACA1_046590/clean/scf7180000084577:149263-149343:+	ACA1_046590	EnoylCoA hydratase/isomerase, putative [Source:UniProtKB/TrEMBL;Acc:L8H8U0]	0.002175698	0.602392	0.600216302	1.51E-12
6h	ACA1_389150/clean/scf7180000084733:212333-212451:+	ACA1_389150	Tasp1 protein [Source:UniProtKB/TrEMBL;Acc:L8GEC7]	0.005890123	0.605813333	0.59992321	1.94E-09
6h	ACA1_279050/clean/scf7180000084776:301937-302029:-	ACA1_279050	PCI domain-containing protein [Source:UniProtKB/TrEMBL;Acc:L8H5Y4]	0.00682781	0.606680667	0.599852857	1.89E-25
6h	ACA1_111560/clean/scf7180000084669:19557-19640:-	ACA1_111560	hypothetical protein	0	0.599759333	0.599759333	5.25E-08
6h	ACA1_164500/clean/scf7180000084729:229331-229528:+	ACA1_164500	SH3 domain-containing protein [Source:UniProtKB/TrEMBL;Acc:L8GRK1]	0.02501701	0.624282667	0.599265657	5.82E-11
6h	ACA1_150950/clean/scf7180000084716:34574-34656:+	ACA1_150950	Exocyst complex component Sec6 [Source:UniProtKB/TrEMBL;Acc:L8H215]	0.009275367	0.608319	0.599043633	2.73E-10
6h	ACA1_065460/clean/scf7180000084599:1859341-1859421:-	ACA1_065460	RhoGAP domain containing protein [Source:UniProtKB/TrEMBL;Acc:L8GXS7]	0.015604733	0.614596333	0.5989916	1.54E-10
6h	ACA1_045960/clean/scf7180000084577:14211-14312:-	ACA1_045960	hypothetical protein	0.096324133	0.695197	0.598872867	4.85E-07
6h	ACA1_154230/clean/scf7180000084722:28173-28267:+	ACA1_154230	hypothetical protein	0.01140631	0.609822333	0.598416023	3.88E-28
6h	ACA1_068780/clean/scf7180000084603:275019-275103:-	ACA1_068780	ANK_REP_REGION domain-containing protein [Source:UniProtKB/TrEMBL;Acc:L8HCM2]	0.002439023	0.600850667	0.598411643	3.40E-09
6h	ACA1_389480/clean/scf7180000084733:265304-265404:+	ACA1_389480	Fe2OG dioxygenase domain-containing protein [Source:UniProtKB/TrEMBL;Acc:L8GDX3]	0.098175433	0.696492333	0.5983169	4.07E-12
6h	ACA1_389110/clean/scf7180000084733:197830-197923:+	ACA1_389110	hypothetical protein	0.000973659	0.599175	0.598201341	3.05E-15
6h	ACA1_174540/clean/scf7180000084741:732266-732350:-	ACA1_174540	Fucosylgalactoside 3-alpha-galactosyltransferase [Source:UniProtKB/TrEMBL;Acc:L8HJC5]	0.032163733	0.630301	0.598137267	5.42E-13
6h	ACA1_123880/clean/scf7180000084686:78302-78433:-	ACA1_123880	hypothetical protein	0.0500911	0.648166333	0.598075233	5.12E-18
6h	ACA1_059530/clean/scf7180000084599:462217-462292:-	ACA1_059530	hypothetical protein	0.001233263	0.599112	0.597878737	1.95E-13
6h	ACA1_323330/clean/scf7180000084468:6454-6526:-	ACA1_323330	CULLIN_2 domain-containing protein [Source:UniProtKB/TrEMBL;Acc:L8H0W0]	0.000214915	0.598024667	0.597809752	1.12E-08
6h	ACA1_307510/clean/scf7180000083680:22393-22498:-	ACA1_307510	hypothetical protein	0.0266133	0.624265667	0.597652367	2.09E-11
6h	ACA1_341070/clean/scf7180000084620:45270-45348:-	ACA1_341070	Peroxisomal membrane 22 kDa family protein [Source:UniProtKB/TrEMBL;Acc:L8GRB3]	0.011640677	0.608994333	0.597353657	1.41E-17
6h	ACA1_252190/clean/scf7180000084768:141517-141589:-	ACA1_252190	hypothetical protein	0	0.596295	0.596295	9.96E-07
6h	ACA1_060240/clean/scf7180000084599:626066-626270:-	ACA1_060240	hypothetical protein	0.053262033	0.649528	0.596265967	2.21E-16
6h	ACA1_133120/clean/scf7180000084701:2967-3036:-	ACA1_133120	Methyltransferase, putatative [Source:UniProtKB/TrEMBL;Acc:L8H3M1]	0.089317433	0.685521	0.596203567	6.50E-10
6h	ACA1_218620/clean/scf7180000084758:679392-679454:-	ACA1_218620	Carboxylic ester hydrolase [Source:UniProtKB/TrEMBL;Acc:L8GRD3]	0.008274333	0.603953	0.595678667	2.73E-12
6h	ACA1_130580/clean/scf7180000084698:115749-115836:-	ACA1_130580	CID domain-containing protein [Source:UniProtKB/TrEMBL;Acc:L8GQC8]	0.01574391	0.611365667	0.595621757	3.21E-07
6h	ACA1_103860/clean/scf7180000084653:54119-54193:-	ACA1_103860	Kinase [Source:UniProtKB/TrEMBL;Acc:L8GE29]	0.013051277	0.608513	0.595461723	7.79E-09
6h	ACA1_054840/clean/scf7180000084594:589274-589344:-	ACA1_054840	Cytochrome p450 superfamily protein [Source:UniProtKB/TrEMBL;Acc:L8H7M8]	0.132531	0.727966333	0.595435333	0.000108983
6h	ACA1_069380/clean/scf7180000084603:424419-424538:+	ACA1_069380	LNS2 domain-containing protein [Source:UniProtKB/TrEMBL;Acc:L8HCT1]	0.128399333	0.723499	0.595099667	1.17E-10
6h	ACA1_164660/clean/scf7180000084729:245424-245533:-	ACA1_164660	Protein tyrosine phosphatase, dual specificity, putative [Source:UniProtKB/TrEMBL;Acc:L8GU25]	0.004118577	0.599063	0.594944423	2.77E-21
6h	ACA1_117020/clean/scf7180000084669:893977-894081:+	ACA1_117020	Carboxypeptidase [Source:UniProtKB/TrEMBL;Acc:L8H3Z1]	0.001718704	0.596638	0.594919296	2.55E-14
6h	ACA1_037190/clean/scf7180000084567:79804-79879:+	ACA1_037190	Aminotran_5 domain-containing protein [Source:UniProtKB/TrEMBL;Acc:L8H0V4]	0.037324733	0.632152333	0.5948276	8.32E-15
6h	ACA1_276410/clean/scf7180000084774:168233-168364:+	ACA1_276410	Phosphate transporter [Source:UniProtKB/TrEMBL;Acc:L8GTH7]	0.011380317	0.605882	0.594501683	3.17E-09
6h	ACA1_113440/clean/scf7180000084669:277101-277221:+	ACA1_113440	hypothetical protein	0.00322946	0.597633333	0.594403873	8.17E-15
6h	ACA1_172080/clean/scf7180000084741:242125-242231:-	ACA1_172080	GRAM domain-containing protein [Source:UniProtKB/TrEMBL;Acc:L8HIX0]	0.179991333	0.774115333	0.594124	0.001781081
6h	ACA1_178310/clean/scf7180000084743:209503-209626:-	ACA1_178310	Ubiquitin-like domain-containing protein [Source:UniProtKB/TrEMBL;Acc:L8GTE8]	0.330925333	0.92504	0.594114667	0.000290806
6h	ACA1_300860/clean/scf7180000083147:9476-9599:+	ACA1_300860	TFIIB-type domain-containing protein [Source:UniProtKB/TrEMBL;Acc:L8HHA5]	0.316842333	0.910438333	0.593596	1.96E-10
6h	ACA1_370300/clean/scf7180000084664:490213-490274:-	ACA1_370300	hypothetical protein	0.008501193	0.601760333	0.59325914	3.53E-21
6h	ACA1_307560/clean/scf7180000083680:38070-38177:-	ACA1_307560	WD domain, G-beta repeat-containing protein [Source:UniProtKB/TrEMBL;Acc:L8H741]	0.008130067	0.601335	0.593204933	1.50E-08
6h	ACA1_138310/clean/scf7180000084703:99739-99863:+	ACA1_138310	Rap-GAP domain-containing protein [Source:UniProtKB/TrEMBL;Acc:L8GYP5]	0	0.592862667	0.592862667	2.98E-09
6h	ACA1_146530/clean/scf7180000084713:42538-42627:+	ACA1_146530	Mak10 subunit, NatC N(Alpha)terminal acetyltransferase [Source:UniProtKB/TrEMBL;Acc:L8GTL7]	0	0.592842	0.592842	3.08E-11
6h	ACA1_189820/clean/scf7180000084748:95840-95933:-	ACA1_189820	Protein bcp1, putative [Source:UniProtKB/TrEMBL;Acc:L8GGI0]	0.00895779	0.601454667	0.592496877	2.02E-10
6h	ACA1_071170/clean/scf7180000084603:879775-879862:+	ACA1_071170	RhoGAP domain containing protein [Source:UniProtKB/TrEMBL;Acc:L8HGC8]	0	0.592364	0.592364	2.22E-10
6h	ACA1_281250/clean/scf7180000084776:571898-571963:+	ACA1_281250	Zinc finger, zz type domain containing protein [Source:UniProtKB/TrEMBL;Acc:L8H9B9]	0	0.59216	0.59216	1.52E-05
6h	ACA1_248650/clean/scf7180000084766:103047-103124:+	ACA1_248650	Dehydrogenase/reductase SDR family member 12, putative [Source:UniProtKB/TrEMBL;Acc:L8GXX2]	0.003516167	0.595672	0.592155833	1.08E-07
6h	ACA1_217170/clean/scf7180000084758:534596-534678:-	ACA1_217170	MFAP1 domain-containing protein [Source:UniProtKB/TrEMBL;Acc:L8GQA3]	0	0.591896	0.591896	1.30E-08
6h	ACA1_386950/clean/scf7180000084730:381406-381485:-	ACA1_386950	hypothetical protein	0.009285467	0.601118667	0.5918332	2.14E-07

6h	ACA1_141960/clean/scf7180000084708:61214-61304:-	ACA1_141960	unspecified product	0.006181933	0.597968333	0.5917864	1.65E-08
6h	ACA1_275430/clean/scf7180000084774:17039-17149:-	ACA1_275430	unspecified product	0.011477733	0.603127	0.591649267	1.33E-11
6h	ACA1_031650/clean/scf7180000084522:39142-39243:-	ACA1_031650	Endo/exonuclease/phosphatase domain-containing protein [Source:UniProtKB/TrEMBL;Acc:L8GSN6]	0.023896767	0.615382667	0.5914859	1.01E-09
6h	ACA1_071600/clean/scf7180000084603:1001778-1001854:+	ACA1_071600	Oxidoreductase, short chain dehydrogenase/reductase superfamily protein [Source:UniProtKB/TrEMBL;Acc:L8HFN0]	0	0.591407333	0.591407333	2.33E-09
6h	ACA1_373540/clean/scf7180000084679:96177-96301:-	ACA1_373540	hypothetical protein	0.061992167	0.653073667	0.5910815	6.70E-14
6h	ACA1_250180/clean/scf7180000084767:34616-34692:-	ACA1_250180	RhoGEF domain containing protein [Source:UniProtKB/TrEMBL;Acc:L8HC96]	0.017157247	0.607905667	0.59074842	2.46E-14
6h	ACA1_382920/clean/scf7180000084721:120592-120664:-	ACA1_382920	Ras subfamily protein [Source:UniProtKB/TrEMBL;Acc:L8GV31]	0	0.590729667	0.590729667	7.98E-08
6h	ACA1_063340/clean/scf7180000084599:1391167-1391249:+	ACA1_063340	Dynein intermediate chain, cytosolic, putative [Source:UniProtKB/TrEMBL;Acc:L8GX48]	0.01446433	0.605174	0.59070967	1.06E-12
6h	ACA1_288740/clean/scf7180000084777:275902-275997:-	ACA1_288740	EnoylCoA hydratase/isomerase family domain containing protein [Source:UniProtKB/TrEMBL;Acc:L8HKG6]	0.0017814	0.592311	0.5905296	1.40E-11
6h	ACA1_031400/clean/scf7180000084506:20052-20122:-	ACA1_031400	hypothetical protein	0.037037	0.627432333	0.590395333	0.000102265
6h	ACA1_064340/clean/scf7180000084599:1595826-1595930:-	ACA1_064340	VASt domain-containing protein [Source:UniProtKB/TrEMBL;Acc:L8GWR3]	0.006803023	0.596882333	0.59007931	1.27E-07
6h	ACA1_117270/clean/scf7180000084669:966766-966849:+	ACA1_117270	hypothetical protein	0.004016067	0.593935	0.589918933	1.57E-10
6h	ACA1_199500/clean/scf7180000084753:249005-249140:+	ACA1_199500	1acylglycerophosphocholine O-acyltransferase 1, putative [Source:UniProtKB/TrEMBL;Acc:L8H4L3]	0	0.589673333	0.589673333	1.26E-07
6h	ACA1_186870/clean/scf7180000084746:214665-214801:+	ACA1_186870	Protein kinase domain-containing protein [Source:UniProtKB/TrEMBL;Acc:L8H6J9]	0.284792	0.874237667	0.589445667	2.69E-14
6h	ACA1_174640/clean/scf7180000084741:755282-755358:-	ACA1_174640	hypothetical protein	0.032286407	0.62156	0.589273593	6.49E-07
6h	ACA1_126460/clean/scf7180000084693:72453-72526:+	ACA1_126460	hypothetical protein	0.001827083	0.591011667	0.589184583	1.66E-11
6h	ACA1_068660/clean/scf7180000084603:250291-250364:+	ACA1_068660	WD_REPEATS_REGION domain-containing protein [Source:UniProtKB/TrEMBL;Acc:L8HDH3]	0	0.58914	0.58914	1.96E-06
6h	ACA1_394600/clean/scf7180000084756:117156-117236:-	ACA1_394600	GRIP domain-containing protein [Source:UniProtKB/TrEMBL;Acc:L8H1Q7]	0.239803333	0.828889	0.589085667	2.37E-12
6h	ACA1_151250/clean/scf7180000084716:81709-81861:+	ACA1_151250	Glycine cleavage Tprotein (Aminomethyl transferase) domain containing protein [Source:UniProtKB/TrEMBL;Acc:L8H0R3]	0.0123727	0.601451667	0.589078967	2.05E-09
6h	ACA1_169580/clean/scf7180000084739:44606-44690:+	ACA1_169580	hypothetical protein	0.001759023	0.590789333	0.58903031	2.32E-12
6h	ACA1_290520/clean/scf7180000084777:613366-613471:+	ACA1_290520	ABC transporter domain-containing protein [Source:UniProtKB/TrEMBL;Acc:L8HJ82]	0.002328101	0.591037333	0.588709232	5.61E-11
6h	ACA1_266230/clean/scf7180000084770:636975-637061:+	ACA1_266230	Dilute domain-containing protein [Source:UniProtKB/TrEMBL;Acc:L8H2D0]	0	0.588631667	0.588631667	1.22E-07
6h	ACA1_378010/clean/scf7180000084711:164964-165035:+	ACA1_378010	JmjC domain-containing protein [Source:UniProtKB/TrEMBL;Acc:L8GRT4]	0.019194767	0.607793667	0.5885989	0.000119841
6h	ACA1_128500/clean/scf7180000084697:73280-73366:-	ACA1_128500	UBIQUITIN_CONJUGAT_2 domain-containing protein [Source:UniProtKB/TrEMBL;Acc:L8GWJ5]	0.004329	0.592379	0.58805	3.73E-10
6h	ACA1_015490/clean/scf7180000083799:3872-3948:+	ACA1_015490	hypothetical protein	0.0255404	0.613570333	0.588029933	2.22E-14
6h	ACA1_146600/clean/scf7180000084713:68166-68264:-	ACA1_146600	Protein kinase domain containing protein [Source:UniProtKB/TrEMBL;Acc:L8GWQ5]	0.002147001	0.590017667	0.587870665	4.04E-16
6h	ACA1_287760/clean/scf7180000084777:72663-72737:-	ACA1_287760	JmjC domain-containing protein [Source:UniProtKB/TrEMBL;Acc:L8HIQ1]	0.091733567	0.679596333	0.587862767	9.33E-10
6h	ACA1_037730/clean/scf7180000084568:9408-9492:+	ACA1_037730	Transmembrane protein, putative [Source:UniProtKB/TrEMBL;Acc:L8GN13]	0.046341867	0.634151	0.587809133	0.00026509
6h	ACA1_012040/clean/scf7180000083652:9146-9265:+	ACA1_012040	LIM domain containing protein [Source:UniProtKB/TrEMBL;Acc:L8GI34]	0.009549847	0.597291667	0.58774182	1.02E-14
6h	ACA1_053390/clean/scf7180000084594:274392-274454:-	ACA1_053390	Prefoldin, alpha subunit [Source:UniProtKB/TrEMBL;Acc:L8H843]	0.132711933	0.72043	0.587718067	0.036625622
6h	ACA1_289830/clean/scf7180000084777:474997-475067:+	ACA1_289830	WD_REPEATS_REGION domain-containing protein [Source:UniProtKB/TrEMBL;Acc:L8HJ65]	0.003401367	0.590931667	0.5875303	2.43E-10
6h	ACA1_027030/clean/scf7180000084464:26564-26656:-	ACA1_027030	C2 domain-containing protein [Source:UniProtKB/TrEMBL;Acc:L8GJ80]	0.106488333	0.693749333	0.587261	4.97E-13
6h	ACA1_070820/clean/scf7180000084603:769723-769888:+	ACA1_070820	hypothetical protein	0.222738667	0.809887333	0.587148667	1.15E-08
6h	ACA1_143760/clean/scf7180000084709:90343-90408:-	ACA1_143760	hypothetical protein	0.010582	0.597664667	0.587082667	1.82E-09
6h	ACA1_063310/clean/scf7180000084599:1380387-1380452:+	ACA1_063310	Phospholipid/glycerol acyltransferase, putative [Source:UniProtKB/TrEMBL;Acc:L8GX94]	0.054958767	0.641929667	0.5869709	5.51E-12
6h	ACA1_061890/clean/scf7180000084599:1071001-1071063:-	ACA1_061890	DHO_dh domain-containing protein [Source:UniProtKB/TrEMBL;Acc:L8GWX2]	0	0.586948333	0.586948333	5.87E-10
6h	ACA1_174620/clean/scf7180000084741:750292-750421:-	ACA1_174620	Late embryogenesis abundant protein [Source:UniProtKB/TrEMBL;Acc:L8HK68]	0.023583247	0.610218667	0.58663542	2.33E-10
6h	ACA1_215970/clean/scf7180000084758:418277-418362:-	ACA1_215970	EGFlike domain containing protein [Source:UniProtKB/TrEMBL;Acc:L8GQ73]	0.003929733	0.590508	0.586578267	9.24E-08
6h	ACA1_217140/clean/scf7180000084758:525585-525697:+	ACA1_217140	hypothetical protein	0.00194875	0.588492667	0.586543917	4.41E-14
6h	ACA1_382920/clean/scf7180000084721:119288-119370:-	ACA1_382920	Ras subfamily protein [Source:UniProtKB/TrEMBL;Acc:L8GV31]	0.115924	0.702345667	0.586421667	9.89E-08
6h	ACA1_199500/clean/scf7180000084753:247552-247646:+	ACA1_199500	1acylglycerophosphocholine O-acyltransferase 1, putative [Source:UniProtKB/TrEMBL;Acc:L8H4L3]	0	0.586355	0.586355	5.05E-06
6h	ACA1_260180/clean/scf7180000084769:452536-452630:+	ACA1_260180	Nitric oxide synthase-interacting protein homolog [Source:UniProtKB/TrEMBL;Acc:L8GFN7]	0.003656	0.589945	0.586289	1.74E-11
6h	ACA1_087900/clean/scf7180000084639:127387-127464:-	ACA1_087900	hypothetical protein	0	0.586284	0.586284	5.60E-10
6h	ACA1_282880/clean/scf7180000084776:740849-740946:+	ACA1_282880	DUF667 domain-containing protein [Source:UniProtKB/TrEMBL;Acc:L8H7B1]	0.019289633	0.605220333	0.5859307	7.66E-09
6h	ACA1_076410/clean/scf7180000084612:66157-66239:-	ACA1_076410	hypothetical protein	0.026737033	0.612252333	0.5855153	6.72E-29
6h	ACA1_078020/clean/scf7180000084614:73985-74101:-	ACA1_078020	AlbA_2 domain-containing protein [Source:UniProtKB/TrEMBL;Acc:L8GR16]	0.0074928	0.592575	0.5850822	4.24E-07
6h	ACA1_152960/clean/scf7180000084720:41676-41745:+	ACA1_152960	Malate dehydrogenase [Source:UniProtKB/TrEMBL;Acc:L8HFT9]	0.001442019	0.586374333	0.584932315	2.64E-46
6h	ACA1_158020/clean/scf7180000084724:184779-184901:+	ACA1_158020	hypothetical protein	0.01161823	0.596536333	0.584918103	2.67E-14
6h	ACA1_183740/clean/scf7180000084745:493681-493758:+	ACA1_183740	hypothetical protein	0.099257667	0.684081	0.584823333	2.35E-08
6h	ACA1_309310/clean/scf7180000083758:3382-3457:+	ACA1_309310	hypothetical protein	0.019744467	0.604449333	0.584704867	2.21E-06
6h	ACA1_173330/clean/scf7180000084741:465043-465136:+	ACA1_173330	Fbox domain containing protein [Source:UniProtKB/TrEMBL;Acc:L8HHQ0]	0.007773523	0.592405333	0.58463181	8.21E-10
6h	ACA1_296550/clean/scf7180000084777:1380790-1380880:+	ACA1_296550	hypothetical protein	0	0.584507	0.584507	3.16E-11
6h	ACA1_091560/clean/scf7180000084645:149389-149458:+	ACA1_091560	UBP-type domain-containing protein [Source:UniProtKB/TrEMBL;Acc:L8GKE3]	0.135811	0.719883667	0.584072667	3.45E-07
6h	ACA1_325150/clean/scf7180000084475:10629-10700:+	ACA1_325150	Rab7/RabGfamily small GTPase [Source:UniProtKB/TrEMBL;Acc:L8GPH2]	0.002035763	0.585648	0.583612237	3.78E-14
6h	ACA1_106720/clean/scf7180000084660:109896-110061:-	ACA1_106720	CID domain-containing protein [Source:UniProtKB/TrEMBL;Acc:L8GQ94]	0.0534467	0.637025667	0.583578967	1.54E-13
6h	ACA1_103600/clean/scf7180000084653:42540-42629:+	ACA1_103600	BTB domain-containing protein [Source:UniProtKB/TrEMBL;Acc:L8GD31]	0.014713223	0.598139333	0.58342611	4.13E-10
6h	ACA1_319100/clean/scf7180000084239:14464-14523:+	ACA1_319100	hypothetical protein	0.033769067	0.617192	0.583422933	1.75E-09
6h	ACA1_182530/clean/scf7180000084745:213252-213348:+	ACA1_182530	PUM-HD domain-containing protein [Source:UniProtKB/TrEMBL;Acc:L8H849]	0.007507187	0.590667667	0.58316048	8.46E-13
6h	ACA1_065210/clean/scf7180000084599:1816570-1816657:+	ACA1_065210	3-hydroxyisobutyryl-CoA hydrolase, mitochondrial [Source:UniProtKB/TrEMBL;Acc:L8GXR1]	0.01302504	0.596173667	0.583148627	6.28E-14
6h	ACA1_088900/clean/scf7180000084639:256770-256856:-	ACA1_088900	hypothetical protein	0.0252381	0.608361	0.5831229	5.73E-07
6h	ACA1_333230/clean/scf7180000084581:23705-23764:-	ACA1_333230	ANK_REP_REGION domain-containing protein [Source:UniProtKB/TrEMBL;Acc:L8HIB9]	0.385124	0.968008333	0.582884333	2.96E-12
6h	ACA1_217160/clean/scf7180000084758:532181-532298:+	ACA1_217160	hypothetical protein	0.016123933	0.598988333	0.5828644	9.52E-34
6h	ACA1_208430/clean/scf7180000084757:189819-189937:+	ACA1_208430	hypothetical protein	0.013795233	0.596365667	0.582570433	4.54E-11
6h	ACA1_282720/clean/scf7180000084776:724598-724666:-	ACA1_282720	Leucine rich repeat domain containing protein [Source:UniProtKB/TrEMBL;Acc:L8H9L8]	0.0734317	0.655956333	0.582524633	1.26E-14
6h	ACA1_149380/clean/scf7180000084715:139069-139180:+	ACA1_149380	Ribose 5phosphate isomerase A, putative [Source:UniProtKB/TrEMBL;Acc:L8HC48]	0.0303705	0.612881333	0.582510833	1.49E-16
6h	ACA1_133480/clean/scf7180000084701:64308-64391:+	ACA1_133480	hypothetical protein	0.004607183	0.586716333	0.58210915	1.41E-11
6h	ACA1_088860/clean/scf7180000084639:249275-249387:+	ACA1_088860	Leucine rich repeat domain containing protein [Source:UniProtKB/TrEMBL;Acc:L8GUH8]	0.0042404	0.585688	0.5814476	7.89E-09
6h	ACA1_155810/clean/scf7180000084722:246604-246668:+	ACA1_155810	Fructose6-phosphate-2-kinase, putative [Source:UniProtKB/TrEMBL;Acc:L8H1J5]	0.017605133	0.598943667	0.581338533	2.13E-20
6h	ACA1_246100/clean/scf7180000084763:536023-536097:-	ACA1_246100	ANK_REP_REGION domain-containing protein [Source:UniProtKB/TrEMBL;Acc:L8GKB9]	0.252102	0.833201333	0.581099333	1.24E-05
6h	ACA1_134400/clean/scf7180000084702:29591-29670:+	ACA1_134400	C2 NT-type domain-containing protein [Source:UniProtKB/TrEMBL;Acc:L8GF46]	0.00866334	0.589660667	0.580997327	1.12E-13
6h	ACA1_116460/clean/scf7180000084669:759601-759666:-	ACA1_116460	Queuosine salvage protein [Source:UniProtKB/TrEMBL;Acc:L8H4T6]	0.024966437	0.605875	0.580908563	2.86E-08
6h	ACA1_042040/clean/scf7180000084573:44825-44929:-	ACA1_042040	RAI1 domain-containing protein [Source:UniProtKB/TrEMBL;Acc:L8GV91]	0.011791899	0.592306	0.580514101	1.09E-07
6h	ACA1_053270/clean/scf7180000084594:240196-240304:+	ACA1_053270	Nudix hydrolase domain-containing protein [Source:UniProtKB/TrEMBL;Acc:L8H547]	0.005952367	0.586449	0.580496633	1.44E-09
6h	ACA1_097210/clean/scf7180000084645:1184782-1184948:-	ACA1_097210	Ubiquitin-like domain-containing protein [Source:UniProtKB/TrEMBL;Acc:L8GJ53]	0.034391667	0.614863	0.580471333	4.08E-18
6h	ACA1_175420/clean/scf7180000084741:951239-951332:+	ACA1_175420	hypothetical protein	0.072885233	0.653061	0.580175767	8.46E-06
6h	ACA1_246520/clean/scf7180000084763:567380-567436:+	ACA1_246520	Tetratricopeptide repeat domain containing protein [Source:UniProtKB/TrEMBL;Acc:L8GKX6]	0.306858	0.887005667	0.580147667	0.000992356
6h	ACA1_101470/clean/scf7180000084649:69416-69477:+	ACA1_101470	Dynactin 4 isoform 2, putative [Source:UniProtKB/TrEMBL;Acc:L8GIW5]	0.008959877	0.589001	0.580041123	3.46E-12
6h	ACA1_398890/clean/scf7180000084759:240075-240161:+	ACA1_398890	hypothetical protein	0.01090454	0.590760667	0.579856127	5.37E-11
6h	ACA1_002760/clean/scf7180000082937:5713-5798:+	ACA1_002760	Membrane protein, putative [Source:UniProtKB/TrEMBL;Acc:L8GGP6]	0.0176072	0.597262667	0.579655467	4.51E-11
6h	ACA1_071570/clean/scf7180000084603:995279-995382:-	ACA1_071570	hypothetical protein	0	0.579640667	0.579640667	3.22E-05
6h	ACA1_182190/clean/scf7180000084745:150193-150310:-	ACA1_182190	hypothetical protein	0.0884492	0.668024667	0.579575467	4.54E-20
6h	ACA1_001280/clean/scf7180000082832:18593-18690:-	ACA1_001280	Peptidase C83 domain-containing protein [Source:UniProtKB/TrEMBL;Acc:L8HEW3]	0.02587796	0.605316333	0.579438373	1.13E-07
6h	ACA1_011280/clean/scf7180000083623:7698-7768:+	ACA1_011280	AAA domain-containing protein [Source:UniProtKB/TrEMBL;Acc:L8GP61]	0.004620403	0.583774	0.579153597	2.82E-12
6h	ACA1_363760/clean/scf7180000084641:8925-8996:-	ACA1_363760	Adenosine specific kinase [Source:UniProtKB/TrEMBL;Acc:L8GP09]	0.221845233	0.800815	0.578969767	4.24E-07
6h	ACA1_082750/clean/scf7180000084625:9212-9308:+	ACA1_082750	N-acetyltransferase domain-containing protein [Source:UniProtKB/TrEMBL;Acc:L8GSP4]	0.0395919	0.618547333	0.578955433	1.72E-17
6h	ACA1_144300/clean/scf7180000084709:198671-198801:-	ACA1_144300	RRM domain-containing protein [Source:UniProtKB/TrEMBL;Acc:L8HEU9]	0.003727637	0.582672667	0.57894503	2.75E-12
6h	ACA1_321790/clean/scf7180000084455:25628-25709:+	ACA1_321790	Protein phosphatase 2C domain containing protein [Source:UniProtKB/TrEMBL;Acc:L8GSL9]	0.038158767	0.617012667	0.5788539	3.00E-06
6h	ACA1_114840/clean/scf7180000084669:559497-559603:+	ACA1_114840	Serine/threonine-protein phosphatase [Source:UniProtKB/TrEMBL;Acc:L8H3P4]	0.044915633	0.623582	0.578666367	5.32E-14
6h	ACA1_127640/clean/scf7180000084696:40270-40344:+	ACA1_127640	GP-PDE domain-containing protein [Source:UniProtKB/TrEMBL;Acc:L8GEW1]	0.003058103	0.581629	0.578570897	2.09E-10
6h	ACA1_261400/clean/scf7180000084769:660786-660856:-	ACA1_261400	Serine/threonine protein kinase, putative [Source:UniProtKB/TrEMBL;Acc:L8GF66]	0	0.578530333	0.578530333	0.000670118
6h	ACA1_060920/clean/scf7180000084599:781530-781651:-	ACA1_060920	MFS domain-containing protein [Source:UniProtKB/TrEMBL;Acc:L8GVT5]	0.041287833	0.619805	0.578517167	9.25E-11
6h	ACA1_221050/clean/scf7180000084758:987080-987154:-	ACA1_221050	hypothetical protein	0	0.578513	0.578513	2.03E-10
6h	ACA1_212240/clean/scf7180000084758:142783-142883:+	ACA1_212240	Esterase/lipase ( ), putative [Source:UniProtKB/TrEMBL;Acc:L8GQU1]	0.016666667	0.595152333	0.578485667	4.28E-06
6h	ACA1_280570/clean/scf7180000084776:498567-498649:+	ACA1_280570	Znfinger in Ran binding protein and others domain containing protein [Source:UniProtKB/TrEMBL;Acc:L8H8F5]	0	0.578351667	0.578351667	5.87E-09
6h	ACA1_273100/clean/scf7180000084773:15170-15262:-	ACA1_273100	EF-hand domain-containing protein [Source:UniProtKB/TrEMBL;Acc:L8GG54]	0.001422163	0.579520333	0.57809817	4.42E-08
6h	ACA1_289320/clean/scf7180000084777:415041-415102:-	ACA1_289320	Myblike DNA-binding domain containing protein [Source:UniProtKB/TrEMBL;Acc:L8HI02]	0.031746033	0.609743333	0.5779973	1.73E-06
6h	ACA1_125040/clean/scf7180000084691:21879-21937:-	ACA1_125040	hypothetical protein	0.007719427	0.585679	0.577959573	1.22E-25
6h	ACA1_061120/clean/scf7180000084599:822640-822737:-	ACA1_061120	Leucine rich repeat domain containing protein [Source:UniProtKB/TrEMBL;Acc:L8GVW2]	0.044838843	0.622723667	0.577884823	1.76E-06
6h	ACA1_076920/clean/scf7180000084612:185887-185980:-	ACA1_076920	B30.2/SPRY domain-containing protein [Source:UniProtKB/TrEMBL;Acc:L8GLX5]	0	0.577851667	0.577851667	3.92E-09
6h	ACA1_310760/clean/scf7180000083780:7543-7608:+	ACA1_310760	hypothetical protein	0.080136533	0.657813333	0.5776768	8.10E-11
6h	ACA1_071440/clean/scf7180000084603:953480-953643:-	ACA1_071440	Nascent polypeptide-associated complex subunit beta [Source:UniProtKB/TrEMBL;Acc:L8HE68]	0.00506226	0.582669333	0.577607073	2.30E-66
6h	ACA1_389080/clean/scf7180000084733:193049-193150:-	ACA1_389080	Fbox domain containing protein [Source:UniProtKB/TrEMBL;Acc:L8GDU7]	0.002146323	0.579731	0.577584677	6.36E-09
6h	ACA1_058700/clean/scf7180000084599:360866-360937:+	ACA1_058700	hypothetical protein	0.005773607	0.583192	0.577418393	7.43E-16
6h	ACA1_112100/clean/scf7180000084669:110220-110322:+	ACA1_112100	BTB/POZ domain containing protein [Source:UniProtKB/TrEMBL;Acc:L8H3Y0]	0.0079365	0.58532	0.5773835	4.59E-08
6h	ACA1_071130/clean/scf7180000084603:867717-867865:-	ACA1_071130	hypothetical protein	0.157891067	0.73523	0.577338933	3.76E-10
6h	ACA1_088800/clean/scf7180000084639:230582-230655:-	ACA1_088800	hypothetical protein	0.005208333	0.582282333	0.577074	3.23E-06
6h	ACA1_158430/clean/scf7180000084724:216856-216980:+	ACA1_158430	hypothetical protein	0.0913817	0.668205667	0.576823967	2.24E-08
6h	ACA1_074110/clean/scf7180000084605:26973-27054:-	ACA1_074110	Peptidase [Source:UniProtKB/TrEMBL;Acc:L8HJ70]	0.00467077	0.581459667	0.576788897	8.63E-13
6h	ACA1_291660/clean/scf7180000084777:798633-798737:-	ACA1_291660	ABC transporter domain-containing protein [Source:UniProtKB/TrEMBL;Acc:L8HL07]	0.0233489	0.600063667	0.576714767	5.55E-08
6h	ACA1_263020/clean/scf7180000084770:80634-80735:-	ACA1_263020	Molybdenum cofactor sulfurase [Source:UniProtKB/TrEMBL;Acc:L8H3B2]	0.007168467	0.583855	0.576686533	1.11E-06
6h	ACA1_251300/clean/scf7180000084768:54521-54618:-	ACA1_251300	J domain-containing protein [Source:UniProtKB/TrEMBL;Acc:L8H9R3]	0.011875183	0.588476333	0.57660115	1.63E-16
6h	ACA1_146460/clean/scf7180000084713:28760-28847:-	ACA1_146460	RING-type domain-containing protein [Source:UniProtKB/TrEMBL;Acc:L8GU89]	0.00521884	0.581625667	0.576406827	2.64E-13
6h	ACA1_174040/clean/scf7180000084741:585390-585506:-	ACA1_174040	Protein kinase domain-containing protein [Source:UniProtKB/TrEMBL;Acc:L8HK27]	0.016913556	0.593091667	0.576178111	3.94E-15
6h	ACA1_230530/clean/scf7180000084761:373234-373308:+	ACA1_230530	S(Hydroxymethyl)glutathione dehydrogenase/class III alcohol dehydrogenase [Source:UniProtKB/TrEMBL;Acc:L8H8X2]	0.004023567	0.580042667	0.5760191	1.46E-34
6h	ACA1_331800/clean/scf7180000084578:33444-33574:-	ACA1_331800	hypothetical protein	0.0064807	0.582420667	0.575939967	1.51E-07
6h	ACA1_301860/clean/scf7180000083222:44083-44178:+	ACA1_301860	Phosphatidylinositol 3-and 4-kinase [Source:UniProtKB/TrEMBL;Acc:L8HHB0]	0.002721973	0.578634667	0.575912693	6.07E-14
6h	ACA1_258230/clean/scf7180000084769:203429-203488:-	ACA1_258230	DNA ligase [Source:UniProtKB/TrEMBL;Acc:L8GF13]	0.0193442	0.595254333	0.575910133	1.52E-11
6h	ACA1_252770/clean/scf7180000084768:205098-205181:-	ACA1_252770	Peripla_BP_6 domain-containing protein [Source:UniProtKB/TrEMBL;Acc:L8HAP5]	0.024233733	0.600123	0.575889267	7.45E-14
6h	ACA1_044720/clean/scf7180000084576:37692-37786:-	ACA1_044720	F-box domain-containing protein [Source:UniProtKB/TrEMBL;Acc:L8GZR9]	0.049243933	0.624937667	0.575693733	5.11E-13
6h	ACA1_100470/clean/scf7180000084648:92073-92158:-	ACA1_100470	ABC transmembrane type-1 domain-containing protein [Source:UniProtKB/TrEMBL;Acc:L8GJH4]	0.034145467	0.609823667	0.5756782	2.12E-05
6h	ACA1_129370/clean/scf7180000084698:11892-11992:+	ACA1_129370	GRAM domain-containing protein [Source:UniProtKB/TrEMBL;Acc:L8GPH7]	0.010650147	0.586297667	0.57564752	1.17E-08
6h	ACA1_173010/clean/scf7180000084741:386169-386233:-	ACA1_173010	Vtype ATPase, A subunit [Source:UniProtKB/TrEMBL;Acc:L8HGL3]	0	0.575474333	0.575474333	1.68E-07
6h	ACA1_267340/clean/scf7180000084770:859690-859749:-	ACA1_267340	CBS domain containing protein [Source:UniProtKB/TrEMBL;Acc:L8H2L9]	0.035610467	0.611015667	0.5754052	1.41E-16
6h	ACA1_107540/clean/scf7180000084660:217615-217705:+	ACA1_107540	hypothetical protein	0.044443427	0.619821333	0.575377907	1.54E-08
6h	ACA1_294840/clean/scf7180000084777:1046068-1046186:+	ACA1_294840	Transcription elongation factor SII, hS-II-T1 isoform 8, putative [Source:UniProtKB/TrEMBL;Acc:L8HIK4]	0.085292367	0.660616667	0.5753243	5.48E-14
6h	ACA1_072710/clean/scf7180000084603:1272012-1272093:+	ACA1_072710	ABC transporter domain-containing protein [Source:UniProtKB/TrEMBL;Acc:L8HFZ6]	0.049367413	0.624464667	0.575097253	1.20E-09
6h	ACA1_096150/clean/scf7180000084645:942614-942780:+	ACA1_096150	RhoGEF domain containing protein [Source:UniProtKB/TrEMBL;Acc:L8GIN2]	0	0.574882667	0.574882667	0.002669405
6h	ACA1_103160/clean/scf7180000084653:9668-9738:+	ACA1_103160	PALP domain-containing protein [Source:UniProtKB/TrEMBL;Acc:L8GDL9]	0.020537233	0.595247333	0.5747101	2.74E-13
6h	ACA1_264450/clean/scf7180000084770:292348-292432:+	ACA1_264450	Rho-GAP domain-containing protein [Source:UniProtKB/TrEMBL;Acc:L8H487]	0.018469867	0.593173667	0.5747038	4.40E-05
6h	ACA1_391330/clean/scf7180000084740:153902-154145:-	ACA1_391330	hypothetical protein	0.015524	0.590099	0.574575	5.14E-15
6h	ACA1_061900/clean/scf7180000084599:1074877-1074960:-	ACA1_061900	GTP_EFTU_D3 domain-containing protein [Source:UniProtKB/TrEMBL;Acc:L8GY16]	0.006951203	0.581277667	0.574326463	1.24E-12
6h	ACA1_271720/clean/scf7180000084771:92521-92594:-	ACA1_271720	Ras-GAP domain-containing protein [Source:UniProtKB/TrEMBL;Acc:L8GR76]	0.000835147	0.574994	0.574158853	1.64E-11
6h	ACA1_368780/clean/scf7180000084664:160564-160633:-	ACA1_368780	CMP/dCMP-type deaminase domain-containing protein [Source:UniProtKB/TrEMBL;Acc:L8H1B4]	0.032822567	0.606756667	0.5739341	5.48E-10
6h	ACA1_170970/clean/scf7180000084741:30730-30855:-	ACA1_170970	Protein kinase domain-containing protein [Source:UniProtKB/TrEMBL;Acc:L8HH98]	0.004246257	0.578162333	0.573916077	7.63E-29
6h	ACA1_252610/clean/scf7180000084768:189232-189315:-	ACA1_252610	ADPribosylglycohydrolase superfamily protein [Source:UniProtKB/TrEMBL;Acc:L8HD40]	0.010814263	0.584723667	0.573909403	3.08E-14
6h	ACA1_246510/clean/scf7180000084763:563756-563857:+	ACA1_246510	PPM-type phosphatase domain-containing protein [Source:UniProtKB/TrEMBL;Acc:L8GL94]	0.007363267	0.581172667	0.5738094	4.67E-13
6h	ACA1_012020/clean/scf7180000083652:3504-3611:-	ACA1_012020	hypothetical protein	0.00659521	0.580129667	0.573534457	2.80E-15
6h	ACA1_167050/clean/scf7180000084736:63402-63471:-	ACA1_167050	Ras subfamily protein [Source:UniProtKB/TrEMBL;Acc:L8H0S4]	0	0.573112667	0.573112667	6.34E-09

6h	ACA1_002620/clean/scf7180000082935:5438-5505:+	ACA1_002620	Fbox domain containing protein [Source:UniProtKB/TrEMBL;Acc:L8HLR9]	0.057660633	0.630746	0.573085367	7.50E-15
6h	ACA1_072690/clean/scf7180000084603:1254859-1254947:+	ACA1_072690	Signal peptide peptidase [Source:UniProtKB/TrEMBL;Acc:L8HGU4]	0	0.573071667	0.573071667	3.33E-11
6h	ACA1_101530/clean/scf7180000084649:86623-86676:-	ACA1_101530	6phosphogluconate dehydrogenase NAD-binding domain containing protein [Source:UniProtKB/TrEMBL;Acc:L8GGL6]	0.039838233	0.612830667	0.572992433	1.34E-15
6h	ACA1_055570/clean/scf7180000084594:731044-731168:-	ACA1_055570	hypothetical protein	0.008	0.580659333	0.572659333	9.05E-10
6h	ACA1_237810/clean/scf7180000084763:21450-21610:-	ACA1_237810	hypothetical protein	0.016145933	0.588584	0.572438067	1.27E-13
6h	ACA1_071550/clean/scf7180000084603:992184-992305:-	ACA1_071550	F-box domain-containing protein [Source:UniProtKB/TrEMBL;Acc:L8HFM3]	0	0.572362	0.572362	1.33E-07
6h	ACA1_343310/clean/scf7180000084638:23405-23505:-	ACA1_343310	EF-hand domain-containing protein [Source:UniProtKB/TrEMBL;Acc:L8H9D8]	0.004097123	0.576423667	0.572326543	1.65E-07
6h	ACA1_088860/clean/scf7180000084639:249725-249829:+	ACA1_088860	Leucine rich repeat domain containing protein [Source:UniProtKB/TrEMBL;Acc:L8GUH8]	0.00310652	0.575399667	0.572293147	3.26E-08
6h	ACA1_260100/clean/scf7180000084769:431999-432139:-	ACA1_260100	Ras-GAP domain-containing protein [Source:UniProtKB/TrEMBL;Acc:L8GHJ9]	0.00066108	0.57291	0.57224892	4.29E-15
6h	ACA1_087570/clean/scf7180000084639:61533-61619:-	ACA1_087570	hypothetical protein	0	0.572037333	0.572037333	1.28E-07
6h	ACA1_271290/clean/scf7180000084771:12743-12808:-	ACA1_271290	hypothetical protein	0.010620693	0.582578	0.571957307	4.79E-23
6h	ACA1_073240/clean/scf7180000084603:1378734-1378834:-	ACA1_073240	hypothetical protein	0.0107527	0.582609667	0.571856967	1.11E-06
6h	ACA1_372580/clean/scf7180000084679:16554-16631:-	ACA1_372580	Lipase [Source:UniProtKB/TrEMBL;Acc:L8GHE3]	0.032038167	0.603887667	0.5718495	1.72E-21
6h	ACA1_044920/clean/scf7180000084576:59578-59775:-	ACA1_044920	hypothetical protein	0.016497643	0.588289333	0.57179169	1.38E-19
6h	ACA1_216440/clean/scf7180000084758:507618-507870:+	ACA1_216440	PPM-type phosphatase domain-containing protein [Source:UniProtKB/TrEMBL;Acc:L8GQI6]	0.038664733	0.610346	0.571681267	1.19E-15
6h	ACA1_037090/clean/scf7180000084567:55401-55520:+	ACA1_037090	Ankyrin repeat containing protein [Source:UniProtKB/TrEMBL;Acc:L8H0U5]	0.000125628	0.571597333	0.571471705	2.17E-11
6h	ACA1_098000/clean/scf7180000084645:1372584-1372691:+	ACA1_098000	SAM domain-containing protein [Source:UniProtKB/TrEMBL;Acc:L8GK75]	0.001186833	0.572391	0.571204167	2.85E-08
6h	ACA1_257950/clean/scf7180000084769:185729-185812:-	ACA1_257950	hypothetical protein	0.00717727	0.578006667	0.570829397	8.50E-10
6h	ACA1_208170/clean/scf7180000084757:117608-117690:+	ACA1_208170	hypothetical protein	0.000411017	0.571220667	0.57080965	6.28E-10
6h	ACA1_152930/clean/scf7180000084720:36638-36735:-	ACA1_152930	PG_binding_1 domain-containing protein [Source:UniProtKB/TrEMBL;Acc:L8HF34]	0.03242858	0.603192333	0.570763753	6.69E-13
6h	ACA1_204620/clean/scf7180000084754:323173-323245:+	ACA1_204620	hypothetical protein	0.000141423	0.57084	0.570698577	1.29E-09
6h	ACA1_234250/clean/scf7180000084762:249378-249492:-	ACA1_234250	Protein kinase domain-containing protein [Source:UniProtKB/TrEMBL;Acc:L8H190]	0.056433867	0.627063	0.570629133	1.66E-11
6h	ACA1_269300/clean/scf7180000084770:967672-967832:-	ACA1_269300	hypothetical protein	0.00510998	0.575616667	0.570506687	5.50E-24
6h	ACA1_152950/clean/scf7180000084720:40695-40788:-	ACA1_152950	UBIQUITIN_CONJUGAT_2 domain-containing protein [Source:UniProtKB/TrEMBL;Acc:L8HI96]	0.005130957	0.575591	0.570460043	3.60E-44
6h	ACA1_036290/clean/scf7180000084565:71313-71416:-	ACA1_036290	Phospholipid:sterol acyl transferase [Source:UniProtKB/TrEMBL;Acc:L8HBL0]	0.041293133	0.611596667	0.570303533	1.08E-08
6h	ACA1_062090/clean/scf7180000084599:1119120-1119195:+	ACA1_062090	Dihydrolipoamide dehydrogenase [Source:UniProtKB/TrEMBL;Acc:L8GWY6]	0	0.570203	0.570203	0.000773133
6h	ACA1_360020/clean/scf7180000084622:35106-35195:-	ACA1_360020	Mannosyltransferase [Source:UniProtKB/TrEMBL;Acc:L8HCL9]	0.003342827	0.573526	0.570183173	3.23E-10
6h	ACA1_360960/clean/scf7180000084622:208722-208799:-	ACA1_360960	Aminotran_1_2 domain-containing protein [Source:UniProtKB/TrEMBL;Acc:L8HCZ9]	0.005120567	0.575287	0.570166433	2.03E-15
6h	ACA1_261080/clean/scf7180000084769:596122-596226:-	ACA1_261080	MIT domain-containing protein [Source:UniProtKB/TrEMBL;Acc:L8GF47]	0.174711	0.744821667	0.570110667	4.62E-10
6h	ACA1_260830/clean/scf7180000084769:534492-534561:+	ACA1_260830	hypothetical protein	0.022040067	0.592018	0.569977933	1.36E-10
6h	ACA1_288630/clean/scf7180000084777:253003-253094:-	ACA1_288630	Mediator of RNA polymerase II transcription subunit 20 [Source:UniProtKB/TrEMBL;Acc:L8HIV7]	0.053679333	0.623596667	0.569917333	4.54E-11
6h	ACA1_227080/clean/scf7180000084761:94749-94826:+	ACA1_227080	Uracil-DNA glycosylase [Source:UniProtKB/TrEMBL;Acc:L8HA23]	0.007575767	0.577449	0.569873233	1.63E-08
6h	ACA1_069380/clean/scf7180000084603:425300-425412:+	ACA1_069380	LNS2 domain-containing protein [Source:UniProtKB/TrEMBL;Acc:L8HCT1]	0.0051458	0.574908	0.5697622	2.55E-10
6h	ACA1_315630/clean/scf7180000083939:8298-8439:-	ACA1_315630	PKS_ER domain-containing protein [Source:UniProtKB/TrEMBL;Acc:L8GYF9]	0.0186647	0.58831	0.5696453	2.45E-05
6h	ACA1_283060/clean/scf7180000084776:774800-774859:+	ACA1_283060	BolA like protein [Source:UniProtKB/TrEMBL;Acc:L8H6K5]	0.011494267	0.581065	0.569570733	1.65E-06
6h	ACA1_073650/clean/scf7180000084603:1465455-1465522:-	ACA1_073650	hypothetical protein	0.123491167	0.693042333	0.569551167	2.59E-05
6h	ACA1_051660/clean/scf7180000084592:44331-44396:-	ACA1_051660	DNA repair and recombination protein RAD54B, putative [Source:UniProtKB/TrEMBL;Acc:L8GQK0]	0.02362524	0.593160667	0.569535427	2.90E-06
6h	ACA1_038160/clean/scf7180000084568:85780-85892:-	ACA1_038160	Protein kinase domain-containing protein [Source:UniProtKB/TrEMBL;Acc:L8GN38]	0.153649	0.723031333	0.569382333	1.11E-13
6h	ACA1_259490/clean/scf7180000084769:353911-354003:+	ACA1_259490	TPR_REGION domain-containing protein [Source:UniProtKB/TrEMBL;Acc:L8GEZ4]	0.018084633	0.587451667	0.569367033	6.12E-10
6h	ACA1_336100/clean/scf7180000084593:24873-24958:-	ACA1_336100	Ribosomal protein L12, putative [Source:UniProtKB/TrEMBL;Acc:L8H1R0]	0.004089967	0.573429667	0.5693397	1.62E-08
6h	ACA1_015490/clean/scf7180000083799:4080-4159:+	ACA1_015490	hypothetical protein	0.0566425	0.625509	0.5688665	3.71E-13
6h	ACA1_068950/clean/scf7180000084603:317063-317141:+	ACA1_068950	hypothetical protein	0.004509567	0.573322333	0.568812767	7.21E-12
6h	ACA1_129340/clean/scf7180000084698:6133-6209:+	ACA1_129340	Methyltransf_11 domain-containing protein [Source:UniProtKB/TrEMBL;Acc:L8GNW8]	0.00549233	0.574191667	0.568699337	3.48E-08
6h	ACA1_391480/clean/scf7180000084740:186497-186566:+	ACA1_391480	F-box domain-containing protein [Source:UniProtKB/TrEMBL;Acc:L8GPE0]	0.0224443	0.590972333	0.568528033	1.65E-09
6h	ACA1_042070/clean/scf7180000084573:53726-53833:+	ACA1_042070	Dual specificity protein kinase [Source:UniProtKB/TrEMBL;Acc:L8GW91]	0.0834678	0.651703667	0.568235867	1.42E-12
6h	ACA1_143680/clean/scf7180000084709:69453-69543:+	ACA1_143680	hypothetical protein	0.00778244	0.575864	0.56808156	4.70E-13
6h	ACA1_167020/clean/scf7180000084736:57765-57856:+	ACA1_167020	EF-hand domain-containing protein [Source:UniProtKB/TrEMBL;Acc:L8H0U0]	0.011249393	0.578745667	0.567496273	5.66E-16
6h	ACA1_247250/clean/scf7180000084763:656124-656212:-	ACA1_247250	MBD domain-containing protein [Source:UniProtKB/TrEMBL;Acc:L8GMS1]	0.008333333	0.575798667	0.567465333	7.89E-09
6h	ACA1_013340/clean/scf7180000083750:1388-1489:+	ACA1_013340	PH domain-containing protein [Source:UniProtKB/TrEMBL;Acc:L8HGJ7]	0.00414202	0.571306667	0.567164647	4.36E-15
6h	ACA1_332070/clean/scf7180000084579:1244-1332:+	ACA1_332070	Nucleolar gtpbinding protein 1, putative [Source:UniProtKB/TrEMBL;Acc:L8GLH2]	0.010837933	0.577911667	0.567073733	1.19E-21
6h	ACA1_036890/clean/scf7180000084567:6541-6603:+	ACA1_036890	Ras subfamily protein [Source:UniProtKB/TrEMBL;Acc:L8H0T2]	0.006881437	0.573855667	0.56697423	1.20E-14
6h	ACA1_088860/clean/scf7180000084639:250079-250161:+	ACA1_088860	Leucine rich repeat domain containing protein [Source:UniProtKB/TrEMBL;Acc:L8GUH8]	0.013370529	0.580314	0.566943471	6.41E-07
6h	ACA1_253350/clean/scf7180000084768:280355-280427:-	ACA1_253350	Protein kinase domain containing protein [Source:UniProtKB/TrEMBL;Acc:L8H9Z2]	0	0.566845333	0.566845333	8.87E-10
6h	ACA1_097620/clean/scf7180000084645:1298532-1298633:-	ACA1_097620	hypothetical protein	0.010078437	0.576896	0.566817563	3.13E-09
6h	ACA1_132840/clean/scf7180000084700:84887-84959:+	ACA1_132840	DHR10 domain-containing protein [Source:UniProtKB/TrEMBL;Acc:L8GWV1]	0	0.566568	0.566568	0.034800555
6h	ACA1_388690/clean/scf7180000084733:148660-148720:+	ACA1_388690	hypothetical protein	0.010169308	0.576716333	0.566547025	4.37E-19
6h	ACA1_182860/clean/scf7180000084745:267307-267418:-	ACA1_182860	Ankyrin repeat protein, putative [Source:UniProtKB/TrEMBL;Acc:L8H7X7]	0.0928778	0.65939	0.5665122	2.81E-12
6h	ACA1_345230/clean/scf7180000084655:13425-13540:-	ACA1_345230	hypothetical protein	0.008828467	0.575230333	0.566401867	2.47E-08
6h	ACA1_012980/clean/scf7180000083742:15968-16052:-	ACA1_012980	Endo/exonuclease/phosphatase domain-containing protein [Source:UniProtKB/TrEMBL;Acc:L8H4W5]	0.005464467	0.571864	0.566399533	4.92E-10
6h	ACA1_208430/clean/scf7180000084757:190256-190365:+	ACA1_208430	hypothetical protein	0.017070133	0.583406333	0.5663362	2.03E-09
6h	ACA1_309340/clean/scf7180000083758:6371-6447:+	ACA1_309340	Protein tyrosine phosphataselike protein, PTPLA [Source:UniProtKB/TrEMBL;Acc:L8GR24]	0.004859343	0.571148667	0.566289323	1.21E-11
6h	ACA1_204520/clean/scf7180000084754:303501-303604:-	ACA1_204520	hypothetical protein	0	0.565894333	0.565894333	0.018484144
6h	ACA1_252210/clean/scf7180000084768:146713-146801:+	ACA1_252210	Protein kinase domain-containing protein [Source:UniProtKB/TrEMBL;Acc:L8HAM0]	0.0079281	0.573675	0.5657469	1.89E-08
6h	ACA1_377500/clean/scf7180000084711:76511-76598:+	ACA1_377500	Peptidase family c78 protein [Source:UniProtKB/TrEMBL;Acc:L8GU78]	0.022812837	0.588478667	0.56566583	4.76E-12
6h	ACA1_002770/clean/scf7180000082937:7507-7597:+	ACA1_002770	Fbox domain containing protein [Source:UniProtKB/TrEMBL;Acc:L8GJ90]	0.009821373	0.574962	0.565140627	3.85E-09
6h	ACA1_372060/clean/scf7180000084664:624290-624376:-	ACA1_372060	Aldo_ket_red domain-containing protein [Source:UniProtKB/TrEMBL;Acc:L8GYH3]	0.004329	0.569251333	0.564922333	1.84E-06
6h	ACA1_108210/clean/scf7180000084661:13246-13334:-	ACA1_108210	PH domain containing protein [Source:UniProtKB/TrEMBL;Acc:L8GD50]	0.026252757	0.591130333	0.564877577	1.86E-09
6h	ACA1_146440/clean/scf7180000084713:25343-25426:+	ACA1_146440	Methyltransf_11 domain-containing protein [Source:UniProtKB/TrEMBL;Acc:L8GU12]	0.039606667	0.604454333	0.564847667	9.44E-14
6h	ACA1_024220/clean/scf7180000084441:20702-20769:+	ACA1_024220	Purinergic receptor, putative [Source:UniProtKB/TrEMBL;Acc:L8GLB7]	0.00326118	0.567994	0.56473282	1.47E-23
6h	ACA1_191150/clean/scf7180000084748:186959-187031:+	ACA1_191150	Triosephosphate isomerase [Source:UniProtKB/TrEMBL;Acc:L8GE90]	0.000423213	0.565130333	0.56470712	1.73E-15
6h	ACA1_352370/clean/scf7180000084704:3070-3179:+	ACA1_352370	Oxidoreductase, short chain dehydrogenase/reductase superfamily protein [Source:UniProtKB/TrEMBL;Acc:L8GTQ2]	0.050496333	0.615121667	0.564625333	1.80E-12
6h	ACA1_164920/clean/scf7180000084729:311774-311845:-	ACA1_164920	DJ-1_PfpI domain-containing protein [Source:UniProtKB/TrEMBL;Acc:L8GRN3]	0.013201127	0.577638	0.564436873	3.37E-20
6h	ACA1_184270/clean/scf7180000084745:600306-600377:+	ACA1_184270	Ankyrin repeatcontaining protein [Source:UniProtKB/TrEMBL;Acc:L8HAN7]	0.064879433	0.629147	0.564267567	4.91E-12
6h	ACA1_396730/clean/scf7180000084759:110114-110211:+	ACA1_396730	Conserved oligomeric Golgi complex subunit 6 [Source:UniProtKB/TrEMBL;Acc:L8HC92]	0.128217	0.692252667	0.564035667	6.38E-12
6h	ACA1_014990/clean/scf7180000083783:2697-2793:+	ACA1_014990	hypothetical protein	0.0190036	0.582786667	0.563783067	1.39E-06
6h	ACA1_323490/clean/scf7180000084468:35894-35977:+	ACA1_323490	DH domain-containing protein [Source:UniProtKB/TrEMBL;Acc:L8H1L1]	0.0096694	0.573448	0.5637786	2.15E-10
6h	ACA1_055560/clean/scf7180000084594:728891-728955:-	ACA1_055560	Protein C16orf58 family protein [Source:UniProtKB/TrEMBL;Acc:L8H6L0]	0.020833333	0.584590667	0.563757333	2.50E-05
6h	ACA1_033590/clean/scf7180000084559:48303-48395:-	ACA1_033590	Carn_acyltransf domain-containing protein [Source:UniProtKB/TrEMBL;Acc:L8GJ57]	0.0099252	0.573638333	0.563713133	7.92E-17
6h	ACA1_184600/clean/scf7180000084746:3175-3235:-	ACA1_184600	Alpha-N-acetylglucosaminidase family protein [Source:UniProtKB/TrEMBL;Acc:L8H6D3]	0.032258067	0.595752667	0.5634946	3.77E-07
6h	ACA1_094910/clean/scf7180000084645:789055-789264:-	ACA1_094910	Deoxyhypusine synthase [Source:UniProtKB/TrEMBL;Acc:L8GL08]	0.01637769	0.57961	0.56323231	3.74E-15
6h	ACA1_314110/clean/scf7180000083842:3057-3128:-	ACA1_314110	DH domain-containing protein [Source:UniProtKB/TrEMBL;Acc:L8GGY1]	0.0074074	0.570627333	0.563219933	4.10E-09
6h	ACA1_264680/clean/scf7180000084770:356495-356592:+	ACA1_264680	Protein kinase domain-containing protein [Source:UniProtKB/TrEMBL;Acc:L8H1B3]	0.004814367	0.567932	0.563117633	2.06E-11
6h	ACA1_066600/clean/scf7180000084600:26067-26143:-	ACA1_066600	Myotubularin phosphatase domain-containing protein [Source:UniProtKB/TrEMBL;Acc:L8GNT0]	0.00270644	0.565746333	0.563039893	7.76E-10
6h	ACA1_106290/clean/scf7180000084660:76899-76971:+	ACA1_106290	WD_REPEATS_REGION domain-containing protein [Source:UniProtKB/TrEMBL;Acc:L8GNF9]	0.097266233	0.660251667	0.562985433	2.26E-07
6h	ACA1_209820/clean/scf7180000084757:333216-333335:-	ACA1_209820	hypothetical protein	0.048391267	0.611335333	0.562944067	7.19E-13
6h	ACA1_066040/clean/scf7180000084599:1980510-1980578:-	ACA1_066040	hypothetical protein	0	0.562758333	0.562758333	0.000245687
6h	ACA1_268930/clean/scf7180000084770:944267-944367:+	ACA1_268930	PWI domain-containing protein [Source:UniProtKB/TrEMBL;Acc:L8H2N5]	0.007211493	0.569931	0.562719507	6.23E-10
6h	ACA1_263890/clean/scf7180000084770:127106-127201:-	ACA1_263890	Methyltransf_11 domain-containing protein [Source:UniProtKB/TrEMBL;Acc:L8H151]	0.410762667	0.973277333	0.562514667	9.71E-23
6h	ACA1_389180/clean/scf7180000084733:220082-220163:+	ACA1_389180	hypothetical protein	0.009803933	0.572156	0.562352067	2.80E-07
6h	ACA1_072210/clean/scf7180000084603:1177179-1177279:+	ACA1_072210	FeS_assembly_P domain-containing protein [Source:UniProtKB/TrEMBL;Acc:L8HDM7]	0.018518533	0.580658667	0.562140133	1.14E-05
6h	ACA1_298630/clean/scf7180000082992:3177-3286:-	ACA1_298630	Vacuolar protein sorting 16, putative [Source:UniProtKB/TrEMBL;Acc:L8H081]	0.004290348	0.566335	0.562044653	1.05E-12
6h	ACA1_171940/clean/scf7180000084741:205726-205791:-	ACA1_171940	hypothetical protein	0.00332002	0.565325333	0.562005313	1.30E-06
6h	ACA1_090230/clean/scf7180000084639:462541-462641:+	ACA1_090230	Chromatin associated protein [Source:UniProtKB/TrEMBL;Acc:L8GUQ1]	0.002873563	0.564875	0.562001437	4.18E-11
6h	ACA1_011270/clean/scf7180000083623:4881-4950:-	ACA1_011270	FYVE-type domain-containing protein [Source:UniProtKB/TrEMBL;Acc:L8GQN9]	0.0078161	0.569634	0.5618179	2.84E-05
6h	ACA1_292810/clean/scf7180000084777:908276-908391:-	ACA1_292810	hypothetical protein	0.002487563	0.564225	0.561737437	1.29E-06
6h	ACA1_142130/clean/scf7180000084708:90404-90467:+	ACA1_142130	unspecified product	0.000386587	0.562109333	0.561722747	1.79E-13
6h	ACA1_272260/clean/scf7180000084772:42979-43079:-	ACA1_272260	M20_dimer domain-containing protein [Source:UniProtKB/TrEMBL;Acc:L8HGE8]	0.003792197	0.565424667	0.56163247	5.68E-12
6h	ACA1_208100/clean/scf7180000084757:101353-101427:+	ACA1_208100	hypothetical protein	0.004582783	0.566008667	0.561425884	1.90E-10
6h	ACA1_098170/clean/scf7180000084645:1414823-1414880:+	ACA1_098170	BTB domain-containing protein [Source:UniProtKB/TrEMBL;Acc:L8GJA5]	0.054817163	0.615987667	0.561170503	2.73E-06
6h	ACA1_296570/clean/scf7180000084777:1386317-1386385:+	ACA1_296570	TRANSMEMBRANE PROTEIN, putative [Source:UniProtKB/TrEMBL;Acc:L8HIX9]	0.052741733	0.613670333	0.5609286	1.86E-11
6h	ACA1_275600/clean/scf7180000084774:50134-50207:+	ACA1_275600	unspecified product	0.058874467	0.619646	0.560771533	3.12E-09
6h	ACA1_061500/clean/scf7180000084599:944837-944916:+	ACA1_061500	Ras-GAP domain-containing protein [Source:UniProtKB/TrEMBL;Acc:L8GWM5]	0.005208333	0.565853	0.560644667	9.67E-12
6h	ACA1_278900/clean/scf7180000084776:272443-272532:+	ACA1_278900	ANK_REP_REGION domain-containing protein [Source:UniProtKB/TrEMBL;Acc:L8H5W6]	0.008024663	0.568618333	0.56059367	6.88E-11
6h	ACA1_024630/clean/scf7180000084444:1644-1733:+	ACA1_024630	hypothetical protein	0.00066992	0.561131333	0.560461413	3.17E-12
6h	ACA1_096440/clean/scf7180000084645:1011284-1011382:+	ACA1_096440	RhoGAP domain containing protein [Source:UniProtKB/TrEMBL;Acc:L8GJR5]	0.015136667	0.575540667	0.560404	1.71E-11
6h	ACA1_061900/clean/scf7180000084599:1073764-1073854:-	ACA1_061900	GTP_EFTU_D3 domain-containing protein [Source:UniProtKB/TrEMBL;Acc:L8GY16]	0.012548777	0.572931	0.560382223	4.60E-13
6h	ACA1_388780/clean/scf7180000084733:169656-169730:+	ACA1_388780	EGFlike domain containing protein [Source:UniProtKB/TrEMBL;Acc:L8GDT8]	0.001205325	0.56153	0.560324675	7.08E-12
6h	ACA1_175590/clean/scf7180000084741:1009855-1009970:+	ACA1_175590	hypothetical protein	0.034777633	0.595030333	0.5602527	1.29E-08
6h	ACA1_109780/clean/scf7180000084663:24917-25076:-	ACA1_109780	AAA domain-containing protein [Source:UniProtKB/TrEMBL;Acc:L8HIF9]	0.022420433	0.582554667	0.560134233	8.38E-13
6h	ACA1_218390/clean/scf7180000084758:651851-651986:-	ACA1_218390	Glycosyl hydrolase family 20, catalytic domain containing protein [Source:UniProtKB/TrEMBL;Acc:L8GQD2]	0	0.560124667	0.560124667	1.20E-09
6h	ACA1_377260/clean/scf7180000084711:15650-15739:+	ACA1_377260	PH domain containing protein [Source:UniProtKB/TrEMBL;Acc:L8GRU5]	0.017260093	0.577337667	0.560077573	8.74E-12
6h	ACA1_208140/clean/scf7180000084757:113957-114035:-	ACA1_208140	ER lumen proteinretaining receptor-like protein [Source:UniProtKB/TrEMBL;Acc:L8GYA7]	0.038671067	0.59835	0.559678933	2.89E-15
6h	ACA1_255750/clean/scf7180000084768:561071-561239:+	ACA1_255750	hypothetical protein	0.082894533	0.642427333	0.5595328	3.99E-10
6h	ACA1_142680/clean/scf7180000084708:218869-219110:+	ACA1_142680	unspecified product	0.0319097	0.5912	0.5592903	8.68E-18
6h	ACA1_384800/clean/scf7180000084730:106158-106257:+	ACA1_384800	hypothetical protein	0.000117	0.559360667	0.559243667	4.97E-10
6h	ACA1_051680/clean/scf7180000084592:48175-48250:+	ACA1_051680	Ras subfamily protein [Source:UniProtKB/TrEMBL;Acc:L8GPK3]	0	0.558903	0.558903	1.61E-07
6h	ACA1_253360/clean/scf7180000084768:284886-284969:+	ACA1_253360	TP_methylase domain-containing protein [Source:UniProtKB/TrEMBL;Acc:L8HAX0]	0	0.558875	0.558875	7.37E-11
6h	ACA1_149300/clean/scf7180000084715:123972-124099:-	ACA1_149300	Cyclin N-terminal domain-containing protein [Source:UniProtKB/TrEMBL;Acc:L8HBZ1]	0.012479307	0.570791333	0.558312027	3.71E-12
6h	ACA1_174200/clean/scf7180000084741:638941-639014:-	ACA1_174200	Alpha-galactosidase [Source:UniProtKB/TrEMBL;Acc:L8HHU7]	0.02051645	0.578683	0.55816655	1.29E-08
6h	ACA1_171960/clean/scf7180000084741:209021-209110:-	ACA1_171960	F-box domain-containing protein [Source:UniProtKB/TrEMBL;Acc:L8HHE3]	0.001663497	0.559757333	0.558093837	4.20E-10
6h	ACA1_380350/clean/scf7180000084712:46445-46508:+	ACA1_380350	Membrane protein, putative [Source:UniProtKB/TrEMBL;Acc:L8GMT8]	0.039587733	0.597253333	0.5576656	2.25E-15
6h	ACA1_289690/clean/scf7180000084777:443645-443723:-	ACA1_289690	hypothetical protein	0.006043267	0.56348	0.557436733	4.41E-09
6h	ACA1_269260/clean/scf7180000084770:960397-960509:-	ACA1_269260	Ras family protein [Source:UniProtKB/TrEMBL;Acc:L8H2Q9]	0.010702838	0.567771333	0.557068495	1.33E-09
6h	ACA1_100560/clean/scf7180000084648:114503-114581:+	ACA1_100560	hypothetical protein	0.0572869	0.614325333	0.557038433	1.26E-12
6h	ACA1_173790/clean/scf7180000084741:520699-520797:+	ACA1_173790	Nicotinamide-nucleotide adenylyltransferase [Source:UniProtKB/TrEMBL;Acc:L8HK06]	0.00844396	0.565402333	0.556958373	2.17E-13
6h	ACA1_299810/clean/scf7180000083079:11487-11570:+	ACA1_299810	hypothetical protein	0.017177367	0.574109333	0.556931967	8.88E-08
6h	ACA1_199490/clean/scf7180000084753:243507-243587:+	ACA1_199490	ATPdependent RNA helicase DBP5, putative [Source:UniProtKB/TrEMBL;Acc:L8H357]	0.0085102	0.565426667	0.556916467	1.81E-15
6h	ACA1_125100/clean/scf7180000084691:31212-31290:-	ACA1_125100	hypothetical protein	0	0.556555667	0.556555667	3.40E-09
6h	ACA1_119240/clean/scf7180000084672:49968-50078:+	ACA1_119240	Aspartate aminotransferase [Source:UniProtKB/TrEMBL;Acc:L8HKA6]	0.00487724	0.561365667	0.556488427	1.42E-27
6h	ACA1_063690/clean/scf7180000084599:1476053-1476157:-	ACA1_063690	Amidase domain-containing protein [Source:UniProtKB/TrEMBL;Acc:L8GXA6]	0.003831433	0.560305333	0.5564739	3.57E-10
6h	ACA1_153630/clean/scf7180000084720:142691-142766:+	ACA1_153630	OTU domain-containing protein [Source:UniProtKB/TrEMBL;Acc:L8HHG7]	0.006499093	0.562846333	0.55634724	9.42E-10
6h	ACA1_180150/clean/scf7180000084744:32787-32927:-	ACA1_180150	Protein kinase domain-containing protein [Source:UniProtKB/TrEMBL;Acc:L8GDP6]	0.011268267	0.567556333	0.556288067	4.57E-14
6h	ACA1_278920/clean/scf7180000084776:275183-275245:+	ACA1_278920	ABC1 domain-containing protein [Source:UniProtKB/TrEMBL;Acc:L8H8Y2]	0.0281501	0.584262667	0.556112567	2.13E-08
6h	ACA1_367390/clean/scf7180000084641:619598-619664:+	ACA1_367390	FAD-binding PCMH-type domain-containing protein [Source:UniProtKB/TrEMBL;Acc:L8GM36]	0.115472233	0.671499667	0.556027433	3.99E-06
6h	ACA1_126140/clean/scf7180000084693:30481-30602:-	ACA1_126140	HA domain-containing protein [Source:UniProtKB/TrEMBL;Acc:L8HAG8]	0.01870596	0.574725667	0.556019706	1.28E-07
6h	ACA1_313850/clean/scf7180000083835:964-1035:+	ACA1_313850	PAS, putative [Source:UniProtKB/TrEMBL;Acc:L8H077]	0.025252533	0.581145333	0.5558928	0.001626346
6h	ACA1_096820/clean/scf7180000084645:1108374-1108494:-	ACA1_096820	hypothetical protein	0.005545087	0.561362667	0.55581758	2.49E-16
6h	ACA1_385490/clean/scf7180000084730:186508-186591:+	ACA1_385490	SH3 domain-containing protein [Source:UniProtKB/TrEMBL;Acc:L8H963]	0.00151515	0.557255333	0.555740183	6.50E-10

6h	ACA1_144320/clean/scf7180000084709:203655-203749:+	ACA1_144320	ANK_REP_REGION domain-containing protein [Source:UniProtKB/TrEMBL;Acc:L8HI15]	0.027777767	0.583355333	0.555577567	1.09E-05
6h	ACA1_072180/clean/scf7180000084603:1169997-1170069:-	ACA1_072180	ABM domain-containing protein [Source:UniProtKB/TrEMBL;Acc:L8HGR1]	0	0.555515	0.555515	0.000155965
6h	ACA1_112830/clean/scf7180000084669:227776-227917:-	ACA1_112830	RFX-type winged-helix domain-containing protein [Source:UniProtKB/TrEMBL;Acc:L8H5H1]	0.011172154	0.566678	0.555505846	6.04E-08
6h	ACA1_170210/clean/scf7180000084739:72847-72936:-	ACA1_170210	hypothetical protein	0.022952633	0.578394667	0.555442033	2.85E-05
6h	ACA1_101340/clean/scf7180000084649:49273-49353:-	ACA1_101340	Leucine rich repeat domain containing protein [Source:UniProtKB/TrEMBL;Acc:L8GH62]	0.0263158	0.581725333	0.555409533	2.73E-05
6h	ACA1_099740/clean/scf7180000084647:19101-19234:-	ACA1_099740	COesterase domain-containing protein [Source:UniProtKB/TrEMBL;Acc:L8HMJ7]	0.017087383	0.572494333	0.55540695	4.58E-10
6h	ACA1_175780/clean/scf7180000084741:1056519-1056610:-	ACA1_175780	Methyltransf_25 domain-containing protein [Source:UniProtKB/TrEMBL;Acc:L8HKG8]	0.014743477	0.569951667	0.55520819	4.29E-14
6h	ACA1_035350/clean/scf7180000084564:32749-32839:-	ACA1_035350	Protein kinase domain-containing protein [Source:UniProtKB/TrEMBL;Acc:L8HAJ4]	0.032822167	0.587890333	0.555068167	1.77E-09
6h	ACA1_282440/clean/scf7180000084776:681534-681599:+	ACA1_282440	hypothetical protein	0.012992143	0.568049333	0.555057191	7.10E-08
6h	ACA1_260100/clean/scf7180000084769:424379-424488:-	ACA1_260100	Ras-GAP domain-containing protein [Source:UniProtKB/TrEMBL;Acc:L8GHJ9]	0.005826023	0.560806667	0.554980643	8.13E-18
6h	ACA1_338180/clean/scf7180000084604:48884-48941:-	ACA1_338180	hypothetical protein	0.005225823	0.559741	0.554515177	6.21E-24
6h	ACA1_123360/clean/scf7180000084686:18603-18681:+	ACA1_123360	Zn(2)-C6 fungal-type domain-containing protein [Source:UniProtKB/TrEMBL;Acc:L8HF21]	0.004830933	0.559084667	0.554253733	7.99E-09
6h	ACA1_038110/clean/scf7180000084568:74645-74704:-	ACA1_038110	Glycosyltransferase family 8 protein [Source:UniProtKB/TrEMBL;Acc:L8GN34]	0.009926167	0.564127333	0.554201167	3.62E-12
6h	ACA1_291330/clean/scf7180000084777:747832-747920:-	ACA1_291330	Putative tRNA (cytidine(32)/guanosine(34)-2'-O)-methyltransferase [Source:UniProtKB/TrEMBL;Acc:L8HJG0]	0.029455033	0.583517667	0.554062633	2.97E-08
6h	ACA1_071520/clean/scf7180000084603:977450-977545:+	ACA1_071520	Piwi domain containing protein [Source:UniProtKB/TrEMBL;Acc:L8HEC1]	0.004790137	0.558483667	0.55369353	7.19E-16
6h	ACA1_109740/clean/scf7180000084663:16399-16579:-	ACA1_109740	Myblike DNA-binding domain containing protein [Source:UniProtKB/TrEMBL;Acc:L8HJY9]	0.003322507	0.556710333	0.553387827	3.22E-08
6h	ACA1_396910/clean/scf7180000084759:145836-145957:-	ACA1_396910	hypothetical protein	0.117737667	0.671114333	0.553376667	3.26E-05
6h	ACA1_282210/clean/scf7180000084776:627570-627638:+	ACA1_282210	Betacarotene 15,15'-monooxygenase 1, putative [Source:UniProtKB/TrEMBL;Acc:L8H762]	0.000948903	0.554127	0.553178097	1.02E-11
6h	ACA1_061890/clean/scf7180000084599:1072162-1072244:-	ACA1_061890	DHO_dh domain-containing protein [Source:UniProtKB/TrEMBL;Acc:L8GWX2]	0.00275482	0.555930333	0.553175513	1.33E-08
6h	ACA1_063930/clean/scf7180000084599:1538248-1538320:-	ACA1_063930	THUMP domain-containing protein [Source:UniProtKB/TrEMBL;Acc:L8GWP9]	0.001425413	0.554233333	0.55280792	3.71E-10
6h	ACA1_115040/clean/scf7180000084669:594335-594423:-	ACA1_115040	hypothetical protein	0.148571	0.701275	0.552704	7.48E-07
6h	ACA1_392390/clean/scf7180000084740:269368-269474:+	ACA1_392390	Rfwd1prov protein [Source:UniProtKB/TrEMBL;Acc:L8GPG4]	0.00287794	0.555580667	0.552702727	4.46E-09
6h	ACA1_399880/clean/scf7180000084775:55725-55835:+	ACA1_399880	Rab-GAP TBC domain-containing protein [Source:UniProtKB/TrEMBL;Acc:L8GGF5]	0.0082933	0.560953333	0.552660033	1.54E-09
6h	ACA1_329650/clean/scf7180000084562:37527-37589:-	ACA1_329650	hypothetical protein	0.321049	0.873699	0.55265	1.05E-17
6h	ACA1_400300/clean/scf7180000084775:144626-144713:+	ACA1_400300	UBIQUITIN_CONJUGAT_2 domain-containing protein [Source:UniProtKB/TrEMBL;Acc:L8GGI3]	0.0161978	0.568841667	0.552643867	3.41E-06
6h	ACA1_246780/clean/scf7180000084763:580522-580609:-	ACA1_246780	Ras subfamily protein [Source:UniProtKB/TrEMBL;Acc:L8GK82]	0.0222863	0.57478	0.5524937	1.68E-24
6h	ACA1_323370/clean/scf7180000084468:11999-12065:-	ACA1_323370	SHSP domain-containing protein [Source:UniProtKB/TrEMBL;Acc:L8H320]	0.075091533	0.627548333	0.5524568	2.25E-05
6h	ACA1_389330/clean/scf7180000084733:231682-231815:+	ACA1_389330	Nicotinamide riboside kinase 1 family protein [Source:UniProtKB/TrEMBL;Acc:L8GDW0]	0.00553396	0.557668	0.55213404	4.21E-10
6h	ACA1_192960/clean/scf7180000084749:172629-172757:-	ACA1_192960	Multiple Ankyrin repeat domain containing protein [Source:UniProtKB/TrEMBL;Acc:L8GPD9]	0.0557471	0.607791667	0.552044567	2.29E-09
6h	ACA1_296560/clean/scf7180000084777:1382605-1382716:-	ACA1_296560	CitMHS domain-containing protein [Source:UniProtKB/TrEMBL;Acc:L8HLL2]	0.0252208	0.577114	0.5518932	7.41E-16
6h	ACA1_164120/clean/scf7180000084729:166172-166278:+	ACA1_164120	Mitochondrial helicase twinkle, putative [Source:UniProtKB/TrEMBL;Acc:L8GSJ2]	0	0.551861	0.551861	2.83E-06
6h	ACA1_331590/clean/scf7180000084578:2911-2986:+	ACA1_331590	ATPdependent DNA helicase, RecQ subfamily protein [Source:UniProtKB/TrEMBL;Acc:L8HBM3]	0	0.551482333	0.551482333	1.54E-09
6h	ACA1_277560/clean/scf7180000084776:74362-74440:-	ACA1_277560	F-box domain-containing protein [Source:UniProtKB/TrEMBL;Acc:L8H6G6]	0.063677067	0.615060333	0.551383267	1.56E-09
6h	ACA1_087570/clean/scf7180000084639:62204-62347:-	ACA1_087570	hypothetical protein	0.035711333	0.587019333	0.551308	2.33E-05
6h	ACA1_279020/clean/scf7180000084776:294363-294449:-	ACA1_279020	EF hand domain containing protein [Source:UniProtKB/TrEMBL;Acc:L8H8Z6]	0.0735361	0.624677667	0.551141567	9.64E-05
6h	ACA1_272600/clean/scf7180000084772:75686-75789:-	ACA1_272600	SAM domain (Sterile alpha motif) domain containing protein [Source:UniProtKB/TrEMBL;Acc:L8HG53]	0.404546667	0.955657	0.551110333	2.61E-09
6h	ACA1_378130/clean/scf7180000084711:192570-192662:-	ACA1_378130	hypothetical protein	0.056447677	0.607503333	0.551055657	2.46E-08
6h	ACA1_098900/clean/scf7180000084646:58872-58937:-	ACA1_098900	Putative transcriptional regulator cudA [Source:UniProtKB/TrEMBL;Acc:L8H713]	0.221777	0.772655	0.550878	6.06E-10
6h	ACA1_195570/clean/scf7180000084752:17604-17718:+	ACA1_195570	hypothetical protein	0.105691	0.65653	0.550839	7.91E-05
6h	ACA1_037780/clean/scf7180000084568:21302-21398:+	ACA1_037780	RhoGEF domain containing protein [Source:UniProtKB/TrEMBL;Acc:L8GN17]	0	0.550835	0.550835	2.96E-09
6h	ACA1_069380/clean/scf7180000084603:425840-425951:+	ACA1_069380	LNS2 domain-containing protein [Source:UniProtKB/TrEMBL;Acc:L8HCT1]	0.00422634	0.554806667	0.550580327	5.77E-10
6h	ACA1_271580/clean/scf7180000084771:78428-78551:-	ACA1_271580	Protein phosphatase 1, regulatory subunit 7, putative [Source:UniProtKB/TrEMBL;Acc:L8GPQ8]	0.024358733	0.574874	0.550515267	1.36E-13
6h	ACA1_171470/clean/scf7180000084741:111648-111714:+	ACA1_171470	Cystinosis, nephropathic, putative [Source:UniProtKB/TrEMBL;Acc:L8HIQ6]	0	0.550501	0.550501	2.01E-09
6h	ACA1_097280/clean/scf7180000084645:1208545-1208609:-	ACA1_097280	hypothetical protein	0	0.550310333	0.550310333	1.44E-10
6h	ACA1_230520/clean/scf7180000084761:371518-371591:-	ACA1_230520	Hypothetical protein	0	0.550119333	0.550119333	7.89E-13
6h	ACA1_216330/clean/scf7180000084758:470543-470701:+	ACA1_216330	hypothetical protein	0.03224486	0.582103	0.54985814	1.11E-09
6h	ACA1_096600/clean/scf7180000084645:1058449-1058537:+	ACA1_096600	CUE domain containing protein [Source:UniProtKB/TrEMBL;Acc:L8GJT0]	2.95E-05	0.549823667	0.549794197	6.19E-12
6h	ACA1_062850/clean/scf7180000084599:1286377-1286467:+	ACA1_062850	WD_REPEATS_REGION domain-containing protein [Source:UniProtKB/TrEMBL;Acc:L8GZK3]	0.004771646	0.554549333	0.549777687	1.91E-14
6h	ACA1_153240/clean/scf7180000084720:94781-94904:-	ACA1_153240	hypothetical protein	0	0.549607	0.549607	0.001430828
6h	ACA1_380820/clean/scf7180000084712:93466-93535:+	ACA1_380820	tRNA-binding domain-containing protein [Source:UniProtKB/TrEMBL;Acc:L8GMV0]	0.005790493	0.555294	0.549503507	2.89E-20
6h	ACA1_275510/clean/scf7180000084774:34500-34581:+	ACA1_275510	unspecified product	0.0427812	0.592167333	0.549386133	6.80E-11
6h	ACA1_321160/clean/scf7180000084437:565-657:-	ACA1_321160	FAD-binding FR-type domain-containing protein [Source:UniProtKB/TrEMBL;Acc:L8GGN4]	0.01417422	0.563457333	0.549283113	1.87E-14
6h	ACA1_183970/clean/scf7180000084745:533533-533601:-	ACA1_183970	hypothetical protein	0.1332005	0.682242667	0.549042167	2.80E-05
6h	ACA1_035640/clean/scf7180000084564:101470-101553:-	ACA1_035640	Beta-lactamase domain-containing protein [Source:UniProtKB/TrEMBL;Acc:L8H9P2]	0	0.548778	0.548778	6.27E-05
6h	ACA1_061890/clean/scf7180000084599:1071680-1071766:-	ACA1_061890	DHO_dh domain-containing protein [Source:UniProtKB/TrEMBL;Acc:L8GWX2]	0	0.548523667	0.548523667	1.33E-09
6h	ACA1_309500/clean/scf7180000083762:8213-8284:+	ACA1_309500	Pyridine nucleotidedisulfide oxidoreductase domain containing protein [Source:UniProtKB/TrEMBL;Acc:L8GI23]	0	0.548522	0.548522	2.23E-14
6h	ACA1_040740/clean/scf7180000084572:72323-72412:+	ACA1_040740	SAM-dependent MTase TRM10-type domain-containing protein [Source:UniProtKB/TrEMBL;Acc:L8H1W9]	0.001049016	0.549505333	0.548456318	1.08E-08
6h	ACA1_054080/clean/scf7180000084594:395339-395422:-	ACA1_054080	hypothetical protein	0	0.548435667	0.548435667	0.000972737
6h	ACA1_227800/clean/scf7180000084761:139645-139715:+	ACA1_227800	hypothetical protein	0.021182633	0.569574667	0.548392033	4.56E-14
6h	ACA1_062680/clean/scf7180000084599:1272290-1272416:+	ACA1_062680	hypothetical protein	0.032385467	0.580775333	0.548389867	9.50E-20
6h	ACA1_193230/clean/scf7180000084749:213281-213365:+	ACA1_193230	Macro domain-containing protein [Source:UniProtKB/TrEMBL;Acc:L8GN79]	0.0331888	0.581569667	0.548380867	4.17E-13
6h	ACA1_064270/clean/scf7180000084599:1574123-1574231:-	ACA1_064270	Dihydroxybiphenyl dioxygenase, putative [Source:UniProtKB/TrEMBL;Acc:L8GYP3]	0.024851167	0.573194	0.548342833	6.02E-16
6h	ACA1_173790/clean/scf7180000084741:520286-520366:+	ACA1_173790	Nicotinamide-nucleotide adenylyltransferase [Source:UniProtKB/TrEMBL;Acc:L8HK06]	0.002276877	0.550522667	0.54824579	8.80E-11
6h	ACA1_283190/clean/scf7180000084776:783803-783869:+	ACA1_283190	hypothetical protein	0	0.548242333	0.548242333	1.45E-06
6h	ACA1_096400/clean/scf7180000084645:1005006-1005063:+	ACA1_096400	Methyltransf_11 domain-containing protein [Source:UniProtKB/TrEMBL;Acc:L8GJF1]	0	0.547926333	0.547926333	1.08E-12
6h	ACA1_383380/clean/scf7180000084721:159102-159169:+	ACA1_383380	Lysosomal ProX carboxypeptidase [Source:UniProtKB/TrEMBL;Acc:L8GV42]	0.006016313	0.553880333	0.54786402	9.27E-18
6h	ACA1_066150/clean/scf7180000084599:2010567-2010667:+	ACA1_066150	Myosin head (Motor domain) domain containing protein [Source:UniProtKB/TrEMBL;Acc:L8GX00]	0.004791233	0.552375	0.547583767	1.68E-05
6h	ACA1_298780/clean/scf7180000082992:22619-22727:+	ACA1_298780	LIM domain containing protein [Source:UniProtKB/TrEMBL;Acc:L8H085]	0.000678887	0.54824	0.547561113	3.07E-12
6h	ACA1_057820/clean/scf7180000084599:186668-186780:-	ACA1_057820	Dual specificity phosphatase, putative [Source:UniProtKB/TrEMBL;Acc:L8GYG8]	0.018168167	0.565650333	0.547482167	2.87E-09
6h	ACA1_216390/clean/scf7180000084758:489868-490049:-	ACA1_216390	MI domain-containing protein [Source:UniProtKB/TrEMBL;Acc:L8GQI1]	0.025090133	0.572433667	0.547343533	1.16E-13
6h	ACA1_301860/clean/scf7180000083222:44246-44344:+	ACA1_301860	Phosphatidylinositol 3-and 4-kinase [Source:UniProtKB/TrEMBL;Acc:L8HHB0]	0.002754787	0.549935	0.547180213	2.47E-13
6h	ACA1_017740/clean/scf7180000083876:7199-7289:+	ACA1_017740	5'-3' exoribonuclease 1 [Source:UniProtKB/TrEMBL;Acc:L8GMR4]	0.002955201	0.549948	0.546992799	8.20E-15
6h	ACA1_245510/clean/scf7180000084763:498860-499095:+	ACA1_245510	SAM domain (Sterile alpha motif) domain containing protein [Source:UniProtKB/TrEMBL;Acc:L8GKU3]	0.064696163	0.611610667	0.546914503	3.65E-07
6h	ACA1_189640/clean/scf7180000084748:71362-71558:-	ACA1_189640	Bifunctional aspartate kinase/diaminopimelate decarboxylase protein [Source:UniProtKB/TrEMBL;Acc:L8GEY3]	0.01775906	0.564663	0.54690394	2.74E-16
6h	ACA1_264270/clean/scf7180000084770:227768-227864:-	ACA1_264270	Oxidoreductase, short chain dehydrogenase/reductase family protein [Source:UniProtKB/TrEMBL;Acc:L8H474]	0.003450377	0.550176333	0.546725957	5.23E-10
6h	ACA1_114910/clean/scf7180000084669:582408-582519:-	ACA1_114910	C2 domain containing protein [Source:UniProtKB/TrEMBL;Acc:L8H6P3]	0.025441533	0.572129	0.546687467	4.47E-06
6h	ACA1_181950/clean/scf7180000084745:90330-90428:+	ACA1_181950	hypothetical protein	0.0596847	0.606189333	0.546504633	1.52E-07
6h	ACA1_220200/clean/scf7180000084758:895601-895675:+	ACA1_220200	Aminopeptidase [Source:UniProtKB/TrEMBL;Acc:L8GT26]	0	0.546342333	0.546342333	9.92E-16
6h	ACA1_360290/clean/scf7180000084622:83456-83534:+	ACA1_360290	Singlestrand binding protein family [Source:UniProtKB/TrEMBL;Acc:L8HBV6]	0.005260403	0.551553	0.546292597	3.07E-17
6h	ACA1_215970/clean/scf7180000084758:418506-418598:-	ACA1_215970	EGFlike domain containing protein [Source:UniProtKB/TrEMBL;Acc:L8GQ73]	0.011132933	0.557347667	0.546214733	8.51E-06
6h	ACA1_206620/clean/scf7180000084757:25811-25922:+	ACA1_206620	MCM domain-containing protein [Source:UniProtKB/TrEMBL;Acc:L8GY26]	0.030617123	0.576586	0.545968877	3.12E-10
6h	ACA1_276410/clean/scf7180000084774:168454-168580:+	ACA1_276410	Phosphate transporter [Source:UniProtKB/TrEMBL;Acc:L8GTH7]	0.023841933	0.569810333	0.5459684	1.86E-10
6h	ACA1_198700/clean/scf7180000084753:154317-154378:-	ACA1_198700	ABC transporter domain-containing protein [Source:UniProtKB/TrEMBL;Acc:L8H2D9]	0.0318228	0.577779667	0.545956867	6.13E-16
6h	ACA1_063230/clean/scf7180000084599:1362150-1362242:-	ACA1_063230	Flagellar associated protein [Source:UniProtKB/TrEMBL;Acc:L8GWG8]	0.002372137	0.548320333	0.545948197	2.51E-12
6h	ACA1_287720/clean/scf7180000084777:67808-67863:-	ACA1_287720	Pyrid_ox_like domain-containing protein [Source:UniProtKB/TrEMBL;Acc:L8HL21]	0.454257667	1	0.545742333	8.15E-06
6h	ACA1_114500/clean/scf7180000084669:486959-487042:-	ACA1_114500	Macro domain-containing protein [Source:UniProtKB/TrEMBL;Acc:L8H6J5]	0.017652467	0.563349667	0.5456972	1.93E-18
6h	ACA1_123350/clean/scf7180000084686:16397-16486:-	ACA1_123350	hypothetical protein	0.013350967	0.558873333	0.545522367	2.83E-13
6h	ACA1_189390/clean/scf7180000084748:24698-24786:-	ACA1_189390	hypothetical protein	0	0.545055	0.545055	1.91E-08
6h	ACA1_048550/clean/scf7180000084582:143037-143141:-	ACA1_048550	TPR_REGION domain-containing protein [Source:UniProtKB/TrEMBL;Acc:L8GDY1]	0.012329193	0.557334	0.545004807	2.73E-10
6h	ACA1_037740/clean/scf7180000084568:13020-13114:-	ACA1_037740	hypothetical protein	0	0.544978667	0.544978667	3.34E-12
6h	ACA1_200030/clean/scf7180000084753:376008-376104:+	ACA1_200030	hypothetical protein	0.033198467	0.57808	0.544881533	0.000679849
6h	ACA1_253450/clean/scf7180000084768:305304-305369:+	ACA1_253450	C2 domain containing protein [Source:UniProtKB/TrEMBL;Acc:L8HC75]	0	0.544864	0.544864	2.44E-06
6h	ACA1_203940/clean/scf7180000084754:227645-227726:+	ACA1_203940	hypothetical protein	0.167097633	0.711605333	0.5445077	6.15E-09
6h	ACA1_063210/clean/scf7180000084599:1353683-1353740:-	ACA1_063210	Rab-GAP TBC domain-containing protein [Source:UniProtKB/TrEMBL;Acc:L8GX80]	0.0246416	0.569132333	0.544490733	2.97E-07
6h	ACA1_187260/clean/scf7180000084747:13517-13621:-	ACA1_187260	hypothetical protein	0.024582867	0.568942333	0.544359467	1.49E-10
6h	ACA1_170960/clean/scf7180000084741:23403-23513:+	ACA1_170960	hypothetical protein	0	0.544212	0.544212	2.98E-11
6h	ACA1_002780/clean/scf7180000082937:9977-10056:-	ACA1_002780	RRM domain-containing protein [Source:UniProtKB/TrEMBL;Acc:L8GGZ7]	0.1153824	0.659204	0.5438216	1.13E-06
6h	ACA1_069730/clean/scf7180000084603:538676-538805:+	ACA1_069730	Lysozyme, putative [Source:UniProtKB/TrEMBL;Acc:L8HF46]	0.01354853	0.557113667	0.543565137	2.35E-25
6h	ACA1_297530/clean/scf7180000082850:1045-1126:-	ACA1_297530	hypothetical protein	0.0272578	0.570433667	0.543175867	2.32E-14
6h	ACA1_393070/clean/scf7180000084756:40059-40138:+	ACA1_393070	Vault protein interalpha-trypsin [Source:UniProtKB/TrEMBL;Acc:L8H2R0]	0.00327291	0.546323667	0.543050757	5.87E-08
6h	ACA1_329480/clean/scf7180000084562:20383-20443:-	ACA1_329480	hypothetical protein	0.100804267	0.643841333	0.543037067	2.63E-10
6h	ACA1_048870/clean/scf7180000084583:6450-6544:-	ACA1_048870	hypothetical protein	0.020279773	0.563247	0.542967227	1.92E-17
6h	ACA1_112000/clean/scf7180000084669:80448-80542:+	ACA1_112000	Rho-GAP domain-containing protein [Source:UniProtKB/TrEMBL;Acc:L8H3W7]	0.004115233	0.547071	0.542955767	2.26E-09
6h	ACA1_091390/clean/scf7180000084645:106149-106243:+	ACA1_091390	Ras family GTPase [Source:UniProtKB/TrEMBL;Acc:L8GIH3]	0.006513187	0.549183667	0.54267048	2.73E-42
6h	ACA1_385700/clean/scf7180000084730:241652-241741:+	ACA1_385700	F-box domain-containing protein [Source:UniProtKB/TrEMBL;Acc:L8HAP4]	0.097474733	0.640055333	0.5425806	0.001236324
6h	ACA1_252610/clean/scf7180000084768:190102-190198:-	ACA1_252610	ADPribosylglycohydrolase superfamily protein [Source:UniProtKB/TrEMBL;Acc:L8HD40]	0.001415157	0.543988	0.542572843	1.57E-12
6h	ACA1_213340/clean/scf7180000084758:180473-180586:-	ACA1_213340	Vacuolar proteinsorting-associated protein 25, putative [Source:UniProtKB/TrEMBL;Acc:L8GQ55]	0.018062333	0.560531	0.542468667	5.63E-12
6h	ACA1_097620/clean/scf7180000084645:1299116-1299202:-	ACA1_097620	hypothetical protein	0.0088889	0.551307	0.5424181	9.40E-09
6h	ACA1_071580/clean/scf7180000084603:998552-998623:-	ACA1_071580	GOLD domain-containing protein [Source:UniProtKB/TrEMBL;Acc:L8HGH0]	0	0.542271333	0.542271333	2.55E-10
6h	ACA1_322160/clean/scf7180000084459:22615-22689:+	ACA1_322160	AB hydrolase-1 domain-containing protein [Source:UniProtKB/TrEMBL;Acc:L8GMT9]	0.002220293	0.544473	0.542252707	3.72E-12
6h	ACA1_200200/clean/scf7180000084753:413201-413324:-	ACA1_200200	Asp_Arg_Hydrox domain-containing protein [Source:UniProtKB/TrEMBL;Acc:L8H4R6]	0.021920183	0.564131333	0.54221115	3.60E-10
6h	ACA1_088860/clean/scf7180000084639:249535-249615:+	ACA1_088860	Leucine rich repeat domain containing protein [Source:UniProtKB/TrEMBL;Acc:L8GUH8]	0.0355684	0.577528667	0.541960267	3.40E-08
6h	ACA1_062680/clean/scf7180000084599:1271935-1272031:+	ACA1_062680	hypothetical protein	0.016253933	0.558176333	0.5419224	1.17E-23
6h	ACA1_358610/clean/scf7180000084742:12864-12929:-	ACA1_358610	hypothetical protein	0.118798333	0.660709	0.541910667	2.10E-07
6h	ACA1_024020/clean/scf7180000084440:5771-5842:-	ACA1_024020	RhoGAP domain containing protein [Source:UniProtKB/TrEMBL;Acc:L8GJP9]	0.003557587	0.545357	0.541799413	3.17E-11
6h	ACA1_164040/clean/scf7180000084729:145632-145724:-	ACA1_164040	Signal peptide binding domain containing protein [Source:UniProtKB/TrEMBL;Acc:L8GRG0]	0.314493333	0.856179	0.541685667	1.31E-06
6h	ACA1_136770/clean/scf7180000084703:5790-5874:+	ACA1_136770	hypothetical protein	0.269475	0.811005	0.54153	3.52E-14
6h	ACA1_392150/clean/scf7180000084740:262748-262807:-	ACA1_392150	AAA domain-containing protein [Source:UniProtKB/TrEMBL;Acc:L8GQ70]	0.002020203	0.543548333	0.54152813	1.75E-10
6h	ACA1_386950/clean/scf7180000084730:381619-381724:-	ACA1_386950	hypothetical protein	0.020916267	0.56244	0.541523733	6.44E-06
6h	ACA1_048870/clean/scf7180000084583:6870-6969:-	ACA1_048870	hypothetical protein	0.0314934	0.572937333	0.541443933	2.21E-17
6h	ACA1_094110/clean/scf7180000084645:694650-694712:+	ACA1_094110	hypothetical protein	0.00880071	0.550240333	0.541439623	4.26E-23
6h	ACA1_066890/clean/scf7180000084600:71023-71321:+	ACA1_066890	RFX-type winged-helix domain-containing protein [Source:UniProtKB/TrEMBL;Acc:L8GPT8]	0.01082374	0.552103667	0.541279927	6.51E-07
6h	ACA1_172880/clean/scf7180000084741:348146-348217:+	ACA1_172880	Protein kinase domain-containing protein [Source:UniProtKB/TrEMBL;Acc:L8HJV9]	0.0630598	0.604116	0.5410562	9.45E-11
6h	ACA1_092140/clean/scf7180000084645:285331-285419:+	ACA1_092140	PseudoU_synth_2 domain-containing protein [Source:UniProtKB/TrEMBL;Acc:L8GIN1]	0.005358197	0.546358333	0.541000137	2.10E-09
6h	ACA1_155810/clean/scf7180000084722:245570-245665:+	ACA1_155810	Fructose6-phosphate-2-kinase, putative [Source:UniProtKB/TrEMBL;Acc:L8H1J5]	0.062607	0.60359	0.540983	1.58E-13
6h	ACA1_217220/clean/scf7180000084758:543524-543651:-	ACA1_217220	VPS9 domain-containing protein [Source:UniProtKB/TrEMBL;Acc:L8GQA7]	0.032812633	0.573655333	0.5408427	6.91E-08
6h	ACA1_182110/clean/scf7180000084745:128244-128342:+	ACA1_182110	MethylcrotonoylCoA carboxylase beta chain, mitochondrial, putative [Source:UniProtKB/TrEMBL;Acc:L8H820]	0.002418963	0.543134	0.540715037	6.49E-12
6h	ACA1_237790/clean/scf7180000084763:15275-15477:+	ACA1_237790	Protein kinase domain containing protein [Source:UniProtKB/TrEMBL;Acc:L8GM38]	0.00249133	0.543025	0.54053367	4.72E-11
6h	ACA1_389450/clean/scf7180000084733:258084-258149:+	ACA1_389450	hypothetical protein	0.001683503	0.542019	0.540335497	4.65E-12
6h	ACA1_296280/clean/scf7180000084777:1313753-1313833:+	ACA1_296280	RhoGEF domain containing protein [Source:UniProtKB/TrEMBL;Acc:L8HJZ8]	0.013007233	0.553331333	0.5403241	2.01E-07
6h	ACA1_214220/clean/scf7180000084758:199152-199233:-	ACA1_214220	hypothetical protein	0	0.540061	0.540061	0.000253585
6h	ACA1_084820/clean/scf7180000084632:6508-6676:+	ACA1_084820	RhoGEF domain containing protein [Source:UniProtKB/TrEMBL;Acc:L8HMR3]	0.000701753	0.540619667	0.539917913	1.89E-09
6h	ACA1_192960/clean/scf7180000084749:172383-172480:-	ACA1_192960	Multiple Ankyrin repeat domain containing protein [Source:UniProtKB/TrEMBL;Acc:L8GPD9]	0.000596037	0.540444	0.539847963	1.90E-10
6h	ACA1_149000/clean/scf7180000084715:48078-48159:+	ACA1_149000	Deacetylase sirtuin-type domain-containing protein [Source:UniProtKB/TrEMBL;Acc:L8HD96]	0.401277333	0.941075667	0.539798333	6.38E-09
6h	ACA1_061980/clean/scf7180000084599:1093017-1093086:+	ACA1_061980	F-box domain-containing protein [Source:UniProtKB/TrEMBL;Acc:L8GZC3]	0	0.539731	0.539731	2.72E-07
6h	ACA1_287920/clean/scf7180000084777:125484-125555:-	ACA1_287920	Leucinerich repeat protein lrrA, putative [Source:UniProtKB/TrEMBL;Acc:L8HL59]	0.02392337	0.563578667	0.539655297	3.51E-16
6h	ACA1_060250/clean/scf7180000084599:626597-626697:-	ACA1_060250	Alpha-carbonic anhydrase domain-containing protein [Source:UniProtKB/TrEMBL;Acc:L8GXK3]	0.034381467	0.574022	0.539640533	3.35E-23
6h	ACA1_176000/clean/scf7180000084741:1100444-1100515:-	ACA1_176000	CN hydrolase domain-containing protein [Source:UniProtKB/TrEMBL;Acc:L8HI77]	0.020935897	0.560444333	0.539508437	5.25E-14

6h	ACA1_372640/clean/scf7180000084679:36233-36342:+	ACA1_372640	Mediator of RNA polymerase II transcription subunit 10 [Source:UniProtKB/TrEMBL;Acc:L8GGR4]	0.014329123	0.553619333	0.53929021	2.40E-07
6h	ACA1_276440/clean/scf7180000084774:178813-178943:+	ACA1_276440	unspecified product	0.096347567	0.635536333	0.539188767	4.38E-09
6h	ACA1_236680/clean/scf7180000084762:472824-472907:+	ACA1_236680	Membrane protein, putative [Source:UniProtKB/TrEMBL;Acc:L8H1H4]	0	0.539105333	0.539105333	0.023859454
6h	ACA1_230500/clean/scf7180000084761:364851-364958:+	ACA1_230500	Ammonium transporter [Source:UniProtKB/TrEMBL;Acc:L8H844]	0.0231433	0.562190667	0.539047367	7.06E-11
6h	ACA1_067410/clean/scf7180000084601:20340-20437:-	ACA1_067410	F-box domain-containing protein [Source:UniProtKB/TrEMBL;Acc:L8GTP9]	0.045861107	0.584835667	0.53897456	1.28E-07
6h	ACA1_320950/clean/scf7180000084435:1169-1253:-	ACA1_320950	SET domain containing protein [Source:UniProtKB/TrEMBL;Acc:L8GPN6]	0.10344	0.642342	0.538902	2.64E-07
6h	ACA1_203060/clean/scf7180000084754:134008-134130:+	ACA1_203060	hypothetical protein	0.074891433	0.61373	0.538838567	3.52E-05
6h	ACA1_050200/clean/scf7180000084589:54929-55015:+	ACA1_050200	Mammalian PTEN tumor suppressorlike, putative [Source:UniProtKB/TrEMBL;Acc:L8H3Q5]	0.017694667	0.556419333	0.538724667	3.63E-14
6h	ACA1_035420/clean/scf7180000084564:45465-45575:-	ACA1_035420	Carrier superfamily protein [Source:UniProtKB/TrEMBL;Acc:L8HAE9]	0.000545083	0.539255333	0.53871025	4.40E-12
6h	ACA1_277440/clean/scf7180000084776:32185-32296:-	ACA1_277440	Clu domain-containing protein [Source:UniProtKB/TrEMBL;Acc:L8H8N0]	0.0757856	0.614449	0.5386634	4.22E-10
6h	ACA1_012020/clean/scf7180000083652:3336-3399:-	ACA1_012020	hypothetical protein	0.01375398	0.552375333	0.538621353	1.39E-17
6h	ACA1_185180/clean/scf7180000084746:82109-82187:-	ACA1_185180	LIM zinc-binding domain-containing protein [Source:UniProtKB/TrEMBL;Acc:L8H575]	0.002724333	0.541315667	0.538591333	9.88E-14
6h	ACA1_259490/clean/scf7180000084769:354404-354629:+	ACA1_259490	TPR_REGION domain-containing protein [Source:UniProtKB/TrEMBL;Acc:L8GEZ4]	0.0549673	0.593354333	0.538387033	1.40E-08
6h	ACA1_072340/clean/scf7180000084603:1204709-1204781:+	ACA1_072340	Serine/threonine-protein phosphatase [Source:UniProtKB/TrEMBL;Acc:L8HEH2]	0	0.538376333	0.538376333	3.62E-10
6h	ACA1_333220/clean/scf7180000084581:22910-22983:+	ACA1_333220	hypothetical protein	0.02212627	0.560233333	0.538107063	5.14E-15
6h	ACA1_203070/clean/scf7180000084754:135341-135436:+	ACA1_203070	Fbox domain containing protein [Source:UniProtKB/TrEMBL;Acc:L8GUJ5]	0.020899487	0.558982333	0.538082847	9.66E-15
6h	ACA1_113440/clean/scf7180000084669:275010-275200:+	ACA1_113440	hypothetical protein	0.005312087	0.543361333	0.538049247	1.40E-15
6h	ACA1_009910/clean/scf7180000083468:7688-7767:+	ACA1_009910	tRNA-synt_1c domain-containing protein [Source:UniProtKB/TrEMBL;Acc:L8GDZ9]	0.000153292	0.538124333	0.537971041	6.14E-10
6h	ACA1_235700/clean/scf7180000084762:359324-359468:-	ACA1_235700	TLDc domain-containing protein [Source:UniProtKB/TrEMBL;Acc:L8H1D8]	0.0766765	0.614379333	0.537702833	4.74E-07
6h	ACA1_048880/clean/scf7180000084583:10197-10268:+	ACA1_048880	LIM zinc-binding domain-containing protein [Source:UniProtKB/TrEMBL;Acc:L8HKD0]	0.044758367	0.582301	0.537542633	5.38E-18
6h	ACA1_383600/clean/scf7180000084721:223030-223102:-	ACA1_383600	hypothetical protein	0.021506533	0.559037	0.537530467	3.93E-12
6h	ACA1_279760/clean/scf7180000084776:441821-441902:-	ACA1_279760	PWI domain containing protein [Source:UniProtKB/TrEMBL;Acc:L8H642]	0	0.537493	0.537493	1.05E-11
6h	ACA1_137290/clean/scf7180000084703:30965-31082:+	ACA1_137290	hypothetical protein	0	0.537218	0.537218	0.002081547
6h	ACA1_264270/clean/scf7180000084770:227336-227517:-	ACA1_264270	Oxidoreductase, short chain dehydrogenase/reductase family protein [Source:UniProtKB/TrEMBL;Acc:L8H474]	0.001109494	0.538213	0.537103506	1.16E-11
6h	ACA1_293740/clean/scf7180000084777:971321-971434:-	ACA1_293740	hypothetical protein	0.0269426	0.564038667	0.537096067	2.54E-07
6h	ACA1_373930/clean/scf7180000084679:192749-192884:-	ACA1_373930	hypothetical protein	0.014811773	0.551794333	0.53698256	2.95E-07
6h	ACA1_264440/clean/scf7180000084770:287226-287290:+	ACA1_264440	Sulfurtransferase [Source:UniProtKB/TrEMBL;Acc:L8H202]	0.185601	0.72254	0.536939	1.56E-11
6h	ACA1_156050/clean/scf7180000084723:14255-14318:+	ACA1_156050	Armadillo/betacatenin-like repeat domain containing protein [Source:UniProtKB/TrEMBL;Acc:L8GIE3]	0	0.536824	0.536824	0.005583397
6h	ACA1_103170/clean/scf7180000084653:11803-11882:+	ACA1_103170	CBS domain containing protein [Source:UniProtKB/TrEMBL;Acc:L8GCP7]	0	0.536823667	0.536823667	5.43E-07
6h	ACA1_221030/clean/scf7180000084758:982682-982774:-	ACA1_221030	DUF1992 domain-containing protein [Source:UniProtKB/TrEMBL;Acc:L8GQX4]	0.003411167	0.540233	0.536821833	1.11E-09
6h	ACA1_034030/clean/scf7180000084561:8537-8638:+	ACA1_034030	RNA recognition motif domain containing protein [Source:UniProtKB/TrEMBL;Acc:L8GTW3]	0.00207338	0.538759333	0.536685953	5.16E-11
6h	ACA1_060200/clean/scf7180000084599:612384-612495:-	ACA1_060200	hypothetical protein	0.010698967	0.547330333	0.536631367	2.65E-08
6h	ACA1_068620/clean/scf7180000084603:241745-241843:-	ACA1_068620	hypothetical protein	0	0.536602	0.536602	1.42E-09
6h	ACA1_237840/clean/scf7180000084763:27490-27600:-	ACA1_237840	hypothetical protein	0.001220607	0.537808	0.536587393	1.82E-14
6h	ACA1_251050/clean/scf7180000084768:43384-43458:-	ACA1_251050	SER_THR_PHOSPHATASE domain-containing protein [Source:UniProtKB/TrEMBL;Acc:L8H9Q8]	0.062358267	0.598939667	0.5365814	6.48E-05
6h	ACA1_088880/clean/scf7180000084639:253579-253653:-	ACA1_088880	OGFr_N domain-containing protein [Source:UniProtKB/TrEMBL;Acc:L8GUP7]	0.064814767	0.601339333	0.536524567	0.000102468
6h	ACA1_309500/clean/scf7180000083762:8572-8655:+	ACA1_309500	Pyridine nucleotidedisulfide oxidoreductase domain containing protein [Source:UniProtKB/TrEMBL;Acc:L8GI23]	0.002834897	0.539303333	0.536468437	3.91E-13
6h	ACA1_037730/clean/scf7180000084568:10361-10446:+	ACA1_037730	Transmembrane protein, putative [Source:UniProtKB/TrEMBL;Acc:L8GN13]	0.088128933	0.624562667	0.536433733	0.001032028
6h	ACA1_187260/clean/scf7180000084747:13707-13794:-	ACA1_187260	hypothetical protein	0	0.536229	0.536229	3.51E-11
6h	ACA1_272530/clean/scf7180000084772:55234-55314:+	ACA1_272530	VWFA domain-containing protein [Source:UniProtKB/TrEMBL;Acc:L8HIL5]	0	0.536211667	0.536211667	8.34E-12
6h	ACA1_252030/clean/scf7180000084768:126962-127264:+	ACA1_252030	Nucleolar complex associated protein, putative [Source:UniProtKB/TrEMBL;Acc:L8H9T8]	0.215365333	0.751483333	0.536118	7.19E-07
6h	ACA1_367290/clean/scf7180000084641:590324-590420:-	ACA1_367290	hypothetical protein	0.00247431	0.538373	0.53589869	4.12E-13
6h	ACA1_396970/clean/scf7180000084759:157730-157801:-	ACA1_396970	mRNA cap 0 methyltransferase domain-containing protein [Source:UniProtKB/TrEMBL;Acc:L8HEN0]	0.002552323	0.538046	0.535493677	7.60E-11
6h	ACA1_261080/clean/scf7180000084769:597263-597345:-	ACA1_261080	MIT domain-containing protein [Source:UniProtKB/TrEMBL;Acc:L8GF47]	0.00042587	0.535820667	0.535394797	2.76E-11
6h	ACA1_069940/clean/scf7180000084603:608086-608181:+	ACA1_069940	hypothetical protein	0	0.535324333	0.535324333	7.87E-08
6h	ACA1_230510/clean/scf7180000084761:368092-368152:-	ACA1_230510	hypothetical protein	0.036092267	0.571403667	0.5353114	5.54E-12
6h	ACA1_266100/clean/scf7180000084770:595136-595230:+	ACA1_266100	Protein kinase domain-containing protein [Source:UniProtKB/TrEMBL;Acc:L8H1J1]	0.024911993	0.560137667	0.535225673	2.38E-09
6h	ACA1_174150/clean/scf7180000084741:629625-629692:-	ACA1_174150	RRP7 domain-containing protein [Source:UniProtKB/TrEMBL;Acc:L8HHU1]	0.0127812	0.547948667	0.535167467	1.59E-06
6h	ACA1_051940/clean/scf7180000084592:89741-89824:-	ACA1_051940	Str_synth domain-containing protein [Source:UniProtKB/TrEMBL;Acc:L8GS41]	0.00114083	0.536251333	0.535110503	1.47E-11
6h	ACA1_062060/clean/scf7180000084599:1112997-1113072:+	ACA1_062060	PPM-type phosphatase domain-containing protein [Source:UniProtKB/TrEMBL;Acc:L8GW64]	0	0.535089333	0.535089333	3.98E-10
6h	ACA1_137620/clean/scf7180000084703:61906-61962:+	ACA1_137620	hypothetical protein	0.067435467	0.602490333	0.535054867	0.000213265
6h	ACA1_028680/clean/scf7180000084473:9343-9451:+	ACA1_028680	Protein kinase domain-containing protein [Source:UniProtKB/TrEMBL;Acc:L8GU00]	0.016718067	0.551771333	0.535053267	5.16E-09
6h	ACA1_253110/clean/scf7180000084768:244300-244387:+	ACA1_253110	hypothetical protein	0.02807122	0.563074333	0.535003113	6.37E-10
6h	ACA1_171960/clean/scf7180000084741:209672-209738:-	ACA1_171960	F-box domain-containing protein [Source:UniProtKB/TrEMBL;Acc:L8HHE3]	0	0.534824667	0.534824667	1.81E-08
6h	ACA1_012260/clean/scf7180000083740:2627-2698:+	ACA1_012260	hypothetical protein	0.017819433	0.552260333	0.5344409	3.75E-20
6h	ACA1_385640/clean/scf7180000084730:221743-221864:+	ACA1_385640	hypothetical protein	0.008832217	0.543041	0.534208783	9.40E-08
6h	ACA1_187290/clean/scf7180000084747:21887-21953:-	ACA1_187290	hypothetical protein	0	0.534188	0.534188	9.22E-08
6h	ACA1_070820/clean/scf7180000084603:769554-769636:+	ACA1_070820	hypothetical protein	0.063463667	0.597512667	0.534049	6.20E-07
6h	ACA1_195280/clean/scf7180000084751:88448-88517:+	ACA1_195280	Ras-GAP domain-containing protein [Source:UniProtKB/TrEMBL;Acc:L8H7I4]	0	0.533955333	0.533955333	7.76E-09
6h	ACA1_073280/clean/scf7180000084603:1387447-1387525:+	ACA1_073280	Thg1 domain-containing protein [Source:UniProtKB/TrEMBL;Acc:L8HDU3]	0	0.533882667	0.533882667	1.64E-10
6h	ACA1_111680/clean/scf7180000084669:30535-30618:+	ACA1_111680	RhoGAP domain containing protein [Source:UniProtKB/TrEMBL;Acc:L8H5Z0]	0.007945317	0.541799	0.533853683	1.96E-12
6h	ACA1_162120/clean/scf7180000084726:33650-33739:+	ACA1_162120	hypothetical protein	0.025973157	0.559693333	0.533720177	3.20E-06
6h	ACA1_305550/clean/scf7180000083609:19660-19739:+	ACA1_305550	Folate gamma-glutamyl hydrolase [Source:UniProtKB/TrEMBL;Acc:L8GKY1]	0.005498607	0.538838	0.533339393	1.56E-29
6h	ACA1_217360/clean/scf7180000084758:576296-576457:-	ACA1_217360	Thioredoxin domain-containing protein [Source:UniProtKB/TrEMBL;Acc:L8GRA1]	0.00046027	0.533754	0.53329373	1.39E-11
6h	ACA1_251790/clean/scf7180000084768:120698-120769:-	ACA1_251790	Cellulase (Glycosyl hydrolase family 5) subfamily protein [Source:UniProtKB/TrEMBL;Acc:L8HAR2]	0.00245098	0.535602	0.53315102	1.12E-10
6h	ACA1_183210/clean/scf7180000084745:357657-357771:-	ACA1_183210	Thioredoxin domain-containing protein [Source:UniProtKB/TrEMBL;Acc:L8H812]	0	0.532999333	0.532999333	0.00645949
6h	ACA1_264070/clean/scf7180000084770:171249-171356:+	ACA1_264070	COPI_C domain-containing protein [Source:UniProtKB/TrEMBL;Acc:L8H460]	0.005846303	0.538822667	0.532976363	9.85E-09
6h	ACA1_064400/clean/scf7180000084599:1613130-1613210:-	ACA1_064400	Geranylgeranyl transferase type-2 subunit beta [Source:UniProtKB/TrEMBL;Acc:L8GWR5]	0.035944133	0.568906	0.532961867	1.01E-10
6h	ACA1_250030/clean/scf7180000084767:26975-27098:-	ACA1_250030	C2 NT-type domain-containing protein [Source:UniProtKB/TrEMBL;Acc:L8HC94]	0.011258467	0.544092	0.532833533	7.61E-08
6h	ACA1_045250/clean/scf7180000084576:93674-93744:+	ACA1_045250	hypothetical protein	0.258725667	0.791549333	0.532823667	1.72E-09
6h	ACA1_035640/clean/scf7180000084564:102553-102618:-	ACA1_035640	Beta-lactamase domain-containing protein [Source:UniProtKB/TrEMBL;Acc:L8H9P2]	0	0.532812333	0.532812333	0.000136051
6h	ACA1_267810/clean/scf7180000084770:872973-873051:+	ACA1_267810	hypothetical protein	0.038775333	0.571350333	0.532575	1.38E-10
6h	ACA1_171110/clean/scf7180000084741:43057-43134:-	ACA1_171110	Carrier superfamily protein [Source:UniProtKB/TrEMBL;Acc:L8HIN2]	0.044491967	0.576795	0.532303033	6.31E-11
6h	ACA1_038180/clean/scf7180000084568:91146-91232:+	ACA1_038180	FHA domain containing protein [Source:UniProtKB/TrEMBL;Acc:L8GLQ3]	0.02481072	0.557053	0.53224228	4.39E-12
6h	ACA1_217710/clean/scf7180000084758:592975-593087:-	ACA1_217710	F-box domain-containing protein [Source:UniProtKB/TrEMBL;Acc:L8GRA5]	0.348111	0.880342	0.532231	0.032494808
6h	ACA1_074770/clean/scf7180000084608:82846-82935:-	ACA1_074770	Vps52 / Sac2 family protein [Source:UniProtKB/TrEMBL;Acc:L8HFG7]	0	0.532229	0.532229	8.13E-11
6h	ACA1_141880/clean/scf7180000084708:40670-40762:+	ACA1_141880	RNA editing ligase [Source:UniProtKB/TrEMBL;Acc:L8HCM1]	0.05199847	0.584214	0.53221553	1.04E-06
6h	ACA1_065220/clean/scf7180000084599:1820932-1821040:+	ACA1_065220	F-box domain-containing protein [Source:UniProtKB/TrEMBL;Acc:L8GYX0]	0.00527646	0.537423667	0.532147207	2.61E-09
6h	ACA1_095220/clean/scf7180000084645:816680-816762:-	ACA1_095220	Protein kinase domain-containing protein [Source:UniProtKB/TrEMBL;Acc:L8GIR4]	0.0329439	0.565003	0.5320591	6.11E-14
6h	ACA1_174300/clean/scf7180000084741:664726-664785:+	ACA1_174300	hypothetical protein	0.030181533	0.562238667	0.532057133	4.35E-10
6h	ACA1_252580/clean/scf7180000084768:185628-185737:+	ACA1_252580	AAA domain-containing protein [Source:UniProtKB/TrEMBL;Acc:L8HC38]	0.024711133	0.556555	0.531843867	4.22E-09
6h	ACA1_217210/clean/scf7180000084758:541683-541784:-	ACA1_217210	Mitochondrial import receptor subunit tom40, putative [Source:UniProtKB/TrEMBL;Acc:L8GPR5]	0.009080403	0.540778	0.531697597	1.38E-21
6h	ACA1_174110/clean/scf7180000084741:614875-614964:+	ACA1_174110	hypothetical protein	0.018311567	0.549828333	0.531516767	2.97E-15
6h	ACA1_035370/clean/scf7180000084564:36712-36831:-	ACA1_035370	Protein kinase domain-containing protein [Source:UniProtKB/TrEMBL;Acc:L8HAE6]	0.014785167	0.546117	0.531331833	5.30E-10
6h	ACA1_236050/clean/scf7180000084762:390192-390305:+	ACA1_236050	hypothetical protein	0.039244333	0.570552667	0.531308333	1.05E-06
6h	ACA1_170960/clean/scf7180000084741:23197-23344:+	ACA1_170960	hypothetical protein	0.003497767	0.534767	0.531269233	6.77E-08
6h	ACA1_025520/clean/scf7180000084448:4005-4079:-	ACA1_025520	FERM domain-containing protein [Source:UniProtKB/TrEMBL;Acc:L8GVM5]	0.002201003	0.533330333	0.53112933	3.28E-17
6h	ACA1_391200/clean/scf7180000084740:147426-147508:-	ACA1_391200	LmbE family protein [Source:UniProtKB/TrEMBL;Acc:L8GPC9]	0.006294563	0.537415	0.531120437	6.67E-18
6h	ACA1_279550/clean/scf7180000084776:412272-412359:-	ACA1_279550	CBS domain containing protein [Source:UniProtKB/TrEMBL;Acc:L8H8C1]	0.00311496	0.534185333	0.531070373	3.49E-11
6h	ACA1_206620/clean/scf7180000084757:25192-25300:+	ACA1_206620	MCM domain-containing protein [Source:UniProtKB/TrEMBL;Acc:L8GY26]	0.0104021	0.541368667	0.530966567	6.95E-08
6h	ACA1_296390/clean/scf7180000084777:1333866-1333968:+	ACA1_296390	hypothetical protein	0.00828967	0.539025	0.53073533	2.93E-09
6h	ACA1_124920/clean/scf7180000084691:12892-13001:-	ACA1_124920	hypothetical protein	0.003367	0.534004333	0.530637333	5.97E-08
6h	ACA1_389460/clean/scf7180000084733:260501-260585:-	ACA1_389460	hypothetical protein	0.012664957	0.543286667	0.53062171	6.34E-11
6h	ACA1_377980/clean/scf7180000084711:155850-155930:+	ACA1_377980	Ubiquitin-like domain-containing protein [Source:UniProtKB/TrEMBL;Acc:L8GRZ4]	0.004499547	0.534815667	0.53031612	7.64E-15
6h	ACA1_064810/clean/scf7180000084599:1704648-1704718:-	ACA1_064810	Transporter, major facilitator subfamily protein [Source:UniProtKB/TrEMBL;Acc:L8GWU0]	0.000205634	0.530510667	0.530305032	1.27E-07
6h	ACA1_069620/clean/scf7180000084603:486125-486207:+	ACA1_069620	Poly [ADP-ribose] polymerase [Source:UniProtKB/TrEMBL;Acc:L8HDR5]	0	0.530036	0.530036	7.52E-08
6h	ACA1_060930/clean/scf7180000084599:782725-782872:+	ACA1_060930	RFX-type winged-helix domain-containing protein [Source:UniProtKB/TrEMBL;Acc:L8GWH8]	0.035378667	0.565414	0.530035333	4.63E-16
6h	ACA1_215970/clean/scf7180000084758:419689-419788:-	ACA1_215970	EGFlike domain containing protein [Source:UniProtKB/TrEMBL;Acc:L8GQ73]	0.016599733	0.546630333	0.5300306	5.53E-06
6h	ACA1_385370/clean/scf7180000084730:150991-151075:-	ACA1_385370	Drf_GBD domain-containing protein [Source:UniProtKB/TrEMBL;Acc:L8HBI2]	0.0086506	0.538575667	0.529925067	1.71E-08
6h	ACA1_103150/clean/scf7180000084653:7755-7852:+	ACA1_103150	hypothetical protein	0.013252447	0.543001	0.529748553	1.75E-20
6h	ACA1_045680/clean/scf7180000084576:174319-174407:-	ACA1_045680	hypothetical protein	0	0.52962	0.52962	1.87E-06
6h	ACA1_040640/clean/scf7180000084572:33164-33262:+	ACA1_040640	MFS domain-containing protein [Source:UniProtKB/TrEMBL;Acc:L8H1W1]	0.0128205	0.542341667	0.529521167	9.92E-07
6h	ACA1_392550/clean/scf7180000084740:292133-292241:+	ACA1_392550	Helicase C-terminal domain-containing protein [Source:UniProtKB/TrEMBL;Acc:L8GRN9]	0.303877333	0.833383	0.529505667	0.000124254
6h	ACA1_174040/clean/scf7180000084741:586167-586249:-	ACA1_174040	Protein kinase domain-containing protein [Source:UniProtKB/TrEMBL;Acc:L8HK27]	0.013779557	0.543229333	0.529449777	1.10E-10
6h	ACA1_043160/clean/scf7180000084573:133683-133793:+	ACA1_043160	ANK_REP_REGION domain-containing protein [Source:UniProtKB/TrEMBL;Acc:L8GV67]	0.005546463	0.534832333	0.52928587	6.03E-08
6h	ACA1_035640/clean/scf7180000084564:101627-101695:-	ACA1_035640	Beta-lactamase domain-containing protein [Source:UniProtKB/TrEMBL;Acc:L8H9P2]	0.034055667	0.563318	0.529262333	0.000298288
6h	ACA1_201180/clean/scf7180000084753:501386-501465:-	ACA1_201180	hypothetical protein	0.009320933	0.538571333	0.5292504	3.22E-11
6h	ACA1_352370/clean/scf7180000084704:2699-2777:+	ACA1_352370	Oxidoreductase, short chain dehydrogenase/reductase superfamily protein [Source:UniProtKB/TrEMBL;Acc:L8GTQ2]	0.0789691	0.608059	0.5290899	4.54E-10
6h	ACA1_230490/clean/scf7180000084761:359460-359580:+	ACA1_230490	RFX-type winged-helix domain-containing protein [Source:UniProtKB/TrEMBL;Acc:L8HAD4]	0.009491112	0.538508333	0.529017221	1.02E-13
6h	ACA1_034030/clean/scf7180000084561:8712-8834:+	ACA1_034030	RNA recognition motif domain containing protein [Source:UniProtKB/TrEMBL;Acc:L8GTW3]	0.002381583	0.531263333	0.52888175	2.86E-09
6h	ACA1_046730/clean/scf7180000084577:175120-175236:-	ACA1_046730	SPRY domain containing protein [Source:UniProtKB/TrEMBL;Acc:L8HB40]	0.010611687	0.539459	0.528847313	1.79E-11
6h	ACA1_069640/clean/scf7180000084603:498390-498517:-	ACA1_069640	Thioesterase family domain containing protein [Source:UniProtKB/TrEMBL;Acc:L8HCV4]	0.001632303	0.530323667	0.528691363	4.33E-09
6h	ACA1_055390/clean/scf7180000084594:693398-693471:-	ACA1_055390	hypothetical protein	0.048731633	0.577404667	0.528673033	6.61E-08
6h	ACA1_158440/clean/scf7180000084724:218990-219268:+	ACA1_158440	Coiled-coil domain containing 53, putative [Source:UniProtKB/TrEMBL;Acc:L8HA48]	0.028614033	0.557215333	0.5286013	1.87E-12
6h	ACA1_391680/clean/scf7180000084740:242626-242740:-	ACA1_391680	Squalene and phytoene synthases superfamily protein [Source:UniProtKB/TrEMBL;Acc:L8GRM8]	0.022933	0.551390667	0.528457667	1.91E-10
6h	ACA1_258230/clean/scf7180000084769:203614-203716:-	ACA1_258230	DNA ligase [Source:UniProtKB/TrEMBL;Acc:L8GF13]	0.084271567	0.612721667	0.5284501	5.03E-07
6h	ACA1_171370/clean/scf7180000084741:88541-88616:+	ACA1_171370	hypothetical protein	0.0282547	0.556522667	0.528267967	2.95E-14
6h	ACA1_036240/clean/scf7180000084565:47890-47951:-	ACA1_036240	Serine/threonine kinase [Source:UniProtKB/TrEMBL;Acc:L8HCL1]	0.005158967	0.533407667	0.5282487	4.27E-08
6h	ACA1_063970/clean/scf7180000084599:1547310-1547404:-	ACA1_063970	SH3 domain containing protein [Source:UniProtKB/TrEMBL;Acc:L8GYN6]	0.000724637	0.528908	0.528183363	3.83E-14
6h	ACA1_191490/clean/scf7180000084749:2272-2373:-	ACA1_191490	RTR1-type domain-containing protein [Source:UniProtKB/TrEMBL;Acc:L8GQP2]	0.009917183	0.537646333	0.52772915	2.53E-13
6h	ACA1_320550/clean/scf7180000084416:9471-9549:-	ACA1_320550	Proline iminopeptidase [Source:UniProtKB/TrEMBL;Acc:L8GWD8]	0.018179833	0.545858	0.527678167	1.08E-30
6h	ACA1_252540/clean/scf7180000084768:176270-176389:-	ACA1_252540	hypothetical protein	0.027015267	0.554686333	0.527671067	1.27E-21
6h	ACA1_264550/clean/scf7180000084770:318285-318375:+	ACA1_264550	Cys/Met metabolism PLPdependent enzyme superfamily protein [Source:UniProtKB/TrEMBL;Acc:L8H496]	0.066351233	0.593961	0.527609767	1.35E-11
6h	ACA1_357160/clean/scf7180000084735:10478-10574:-	ACA1_357160	Actin subfamily protein [Source:UniProtKB/TrEMBL;Acc:L8HDB7]	0.185205	0.712811	0.527606	3.34E-10
6h	ACA1_054430/clean/scf7180000084594:492841-492953:-	ACA1_054430	PHB domain-containing protein [Source:UniProtKB/TrEMBL;Acc:L8H647]	0	0.527594667	0.527594667	0.001189641
6h	ACA1_307840/clean/scf7180000083687:22174-22248:+	ACA1_307840	TehB domain-containing protein [Source:UniProtKB/TrEMBL;Acc:L8HBJ4]	0.005649733	0.533084333	0.5274346	2.84E-08
6h	ACA1_174440/clean/scf7180000084741:709874-709977:-	ACA1_174440	Zinc knuckle domain containing protein [Source:UniProtKB/TrEMBL;Acc:L8HJB4]	0.207017	0.734448333	0.527431333	6.28E-09
6h	ACA1_277590/clean/scf7180000084776:82748-82829:-	ACA1_277590	Peptidase_S66 domain-containing protein [Source:UniProtKB/TrEMBL;Acc:L8H6R2]	0.00112376	0.528469	0.52734524	5.02E-14
6h	ACA1_269280/clean/scf7180000084770:964523-964604:-	ACA1_269280	RNA methyltransferase protein [Source:UniProtKB/TrEMBL;Acc:L8H2P1]	0.00297619	0.530186	0.52720981	3.30E-10
6h	ACA1_031270/clean/scf7180000084506:13798-13878:+	ACA1_031270	hypothetical protein	0.021901837	0.549088	0.527186163	1.02E-18
6h	ACA1_106940/clean/scf7180000084660:151955-152106:-	ACA1_106940	TPT domain-containing protein [Source:UniProtKB/TrEMBL;Acc:L8GNI5]	0.001414395	0.5286	0.527185605	3.22E-12
6h	ACA1_234280/clean/scf7180000084762:258164-258229:+	ACA1_234280	hypothetical protein	0.001792113	0.528942	0.527149887	1.92E-11
6h	ACA1_341100/clean/scf7180000084620:50566-50665:-	ACA1_341100	MFS domain-containing protein [Source:UniProtKB/TrEMBL;Acc:L8GR49]	0.010812723	0.537768667	0.526955943	1.53E-14
6h	ACA1_283310/clean/scf7180000084776:814168-814262:-	ACA1_283310	SAM domain-containing protein [Source:UniProtKB/TrEMBL;Acc:L8H6L5]	0.0574784	0.584151667	0.526673267	8.84E-11
6h	ACA1_313620/clean/scf7180000083822:14470-14562:-	ACA1_313620	DUF4461 domain-containing protein [Source:UniProtKB/TrEMBL;Acc:L8GLZ5]	0.006802733	0.533378	0.526575267	3.68E-08
6h	ACA1_074140/clean/scf7180000084605:34786-34885:+	ACA1_074140	Transporter, major facilitator subfamily protein [Source:UniProtKB/TrEMBL;Acc:L8HK94]	0.000625867	0.527190667	0.5265648	2.35E-15
6h	ACA1_043190/clean/scf7180000084573:136795-136870:-	ACA1_043190	hypothetical protein	0.075077667	0.601572	0.526494333	2.48E-11
6h	ACA1_074110/clean/scf7180000084605:25398-25493:-	ACA1_074110	Peptidase [Source:UniProtKB/TrEMBL;Acc:L8HJ70]	0.023758333	0.550178	0.526419667	5.41E-19

6h	ACA1_389400/clean/scf7180000084733:243972-244054:+	ACA1_389400	Transporter, major facilitator subfamily protein [Source:UniProtKB/TrEMBL;Acc:L8GED9]	0	0.526410667	0.526410667	1.52E-06
6h	ACA1_199700/clean/scf7180000084753:301171-301408:+	ACA1_199700	RFX DNAbinding domain containing protein [Source:UniProtKB/TrEMBL;Acc:L8H5G4]	0.021776867	0.548141	0.526364133	9.88E-08
6h	ACA1_258610/clean/scf7180000084769:280967-281061:+	ACA1_258610	hypothetical protein	0.048994267	0.575161667	0.5261674	1.57E-06
6h	ACA1_216300/clean/scf7180000084758:464725-464883:+	ACA1_216300	Ras family protein [Source:UniProtKB/TrEMBL;Acc:L8GR60]	0.036957667	0.563019667	0.526062	1.22E-09
6h	ACA1_113870/clean/scf7180000084669:392445-392548:+	ACA1_113870	CUB domain containing protein [Source:UniProtKB/TrEMBL;Acc:L8H6F7]	0.0195104	0.545538	0.5260276	2.44E-14
6h	ACA1_251760/clean/scf7180000084768:109657-109774:+	ACA1_251760	Protein kinase domain-containing protein [Source:UniProtKB/TrEMBL;Acc:L8HAK7]	0.017986233	0.544012667	0.526026433	1.35E-11
6h	ACA1_224860/clean/scf7180000084760:257532-257630:+	ACA1_224860	Myosin head (Motor domain) domain containing protein [Source:UniProtKB/TrEMBL;Acc:L8GSY9]	0.051282	0.577275333	0.525993333	0.017343388
6h	ACA1_197890/clean/scf7180000084753:47808-47914:+	ACA1_197890	hypothetical protein	0.011928327	0.537857333	0.525929007	5.81E-11
6h	ACA1_072330/clean/scf7180000084603:1199537-1199608:-	ACA1_072330	UBA_e1_C domain-containing protein [Source:UniProtKB/TrEMBL;Acc:L8HGS4]	0.009200857	0.534849667	0.52564881	4.01E-16
6h	ACA1_116740/clean/scf7180000084669:838858-838946:+	ACA1_116740	Methyltransf_2 domain-containing protein [Source:UniProtKB/TrEMBL;Acc:L8H4R1]	0.024330193	0.549856	0.525525807	5.14E-08
6h	ACA1_063620/clean/scf7180000084599:1462782-1462872:-	ACA1_063620	Hydroxysteroid dehydrogenase, putative [Source:UniProtKB/TrEMBL;Acc:L8GYL0]	0.00540447	0.530921	0.52551653	1.51E-17
6h	ACA1_391320/clean/scf7180000084740:152440-152522:-	ACA1_391320	Rab-GAP TBC domain-containing protein [Source:UniProtKB/TrEMBL;Acc:L8GNV7]	0.010264667	0.535698	0.525433333	2.46E-10
6h	ACA1_096400/clean/scf7180000084645:1005120-1005220:+	ACA1_096400	Methyltransf_11 domain-containing protein [Source:UniProtKB/TrEMBL;Acc:L8GJF1]	0.009283233	0.534434333	0.5251511	1.78E-11
6h	ACA1_109130/clean/scf7180000084661:125288-125381:+	ACA1_109130	Serine/threonine kinase [Source:UniProtKB/TrEMBL;Acc:L8GDM5]	0.008143267	0.533253	0.525109733	5.06E-10
6h	ACA1_216440/clean/scf7180000084758:507929-508049:+	ACA1_216440	PPM-type phosphatase domain-containing protein [Source:UniProtKB/TrEMBL;Acc:L8GQI6]	0.010747627	0.535856667	0.52510904	2.83E-19
6h	ACA1_383610/clean/scf7180000084721:228305-228408:+	ACA1_383610	PAKA subfamily protein kinase [Source:UniProtKB/TrEMBL;Acc:L8GW48]	0.020168333	0.545143	0.524974667	1.21E-17
6h	ACA1_349660/clean/scf7180000084690:31077-31713:+	ACA1_349660	BAT1 protein [Source:UniProtKB/TrEMBL;Acc:L8GJC9]	0.1017343	0.626660333	0.524926033	2.32E-25
6h	ACA1_220570/clean/scf7180000084758:924151-924223:+	ACA1_220570	Cyclopropyl isomeraselike protein [Source:UniProtKB/TrEMBL;Acc:L8GT38]	0.001876193	0.526799	0.524922807	9.21E-13
6h	ACA1_155740/clean/scf7180000084722:233024-233111:+	ACA1_155740	Ras subfamily protein [Source:UniProtKB/TrEMBL;Acc:L8H2N0]	0	0.524878667	0.524878667	1.08E-09
6h	ACA1_131540/clean/scf7180000084699:80895-80981:-	ACA1_131540	Pentatricopeptide repeat domain containing protein [Source:UniProtKB/TrEMBL;Acc:L8GPX6]	0.052483167	0.577265333	0.524782167	9.06E-11
6h	ACA1_391420/clean/scf7180000084740:169174-169273:+	ACA1_391420	hypothetical protein	0.006712335	0.531454	0.524741665	5.16E-10
6h	ACA1_091900/clean/scf7180000084645:224221-224305:-	ACA1_091900	Acylphosphatase [Source:UniProtKB/TrEMBL;Acc:L8GHY8]	0.169933333	0.694624	0.524690667	1.49E-07
6h	ACA1_271570/clean/scf7180000084771:75525-75627:-	ACA1_271570	Protein kinase domain-containing protein [Source:UniProtKB/TrEMBL;Acc:L8GP11]	0.009346873	0.533968333	0.52462146	6.37E-20
6h	ACA1_061680/clean/scf7180000084599:1010082-1010167:+	ACA1_061680	PH domain containing protein [Source:UniProtKB/TrEMBL;Acc:L8GXZ1]	0.01398854	0.538592667	0.524604127	1.96E-07
6h	ACA1_051700/clean/scf7180000084592:51591-51693:+	ACA1_051700	FGGAP repeat domain containing protein [Source:UniProtKB/TrEMBL;Acc:L8GPT4]	0.005308553	0.529877333	0.52456878	8.53E-09
6h	ACA1_164910/clean/scf7180000084729:310627-310734:-	ACA1_164910	hypothetical protein	0.001573978	0.526074667	0.524500688	6.23E-16
6h	ACA1_023890/clean/scf7180000084436:31989-32101:-	ACA1_023890	SAM domain-containing protein [Source:UniProtKB/TrEMBL;Acc:L8GSK7]	0.010225653	0.534624	0.524398347	2.43E-13
6h	ACA1_216040/clean/scf7180000084758:442804-442903:-	ACA1_216040	Protein kinase domain-containing protein [Source:UniProtKB/TrEMBL;Acc:L8GQH1]	0.00477163	0.529132	0.52436037	7.81E-09
6h	ACA1_265560/clean/scf7180000084770:510245-510315:+	ACA1_265560	RFX-type winged-helix domain-containing protein [Source:UniProtKB/TrEMBL;Acc:L8H4F0]	0.012138097	0.536472333	0.524334237	1.43E-08
6h	ACA1_376200/clean/scf7180000084685:49470-49533:+	ACA1_376200	Kelch repeat protein [Source:UniProtKB/TrEMBL;Acc:L8HGA3]	0.102920567	0.626942	0.524021433	2.27E-07
6h	ACA1_063340/clean/scf7180000084599:1390889-1390971:+	ACA1_063340	Dynein intermediate chain, cytosolic, putative [Source:UniProtKB/TrEMBL;Acc:L8GX48]	0.004961663	0.528920333	0.52395867	6.30E-10
6h	ACA1_101530/clean/scf7180000084649:86478-86546:-	ACA1_101530	6phosphogluconate dehydrogenase NAD-binding domain containing protein [Source:UniProtKB/TrEMBL;Acc:L8GGL6]	0.010388453	0.534303	0.523914547	4.06E-18
6h	ACA1_106290/clean/scf7180000084660:72437-72509:+	ACA1_106290	WD_REPEATS_REGION domain-containing protein [Source:UniProtKB/TrEMBL;Acc:L8GNF9]	0	0.523840667	0.523840667	1.43E-08
6h	ACA1_263870/clean/scf7180000084770:124597-124697:-	ACA1_263870	MAGE domain-containing protein [Source:UniProtKB/TrEMBL;Acc:L8H1Y1]	0.008321567	0.532095333	0.523773767	2.48E-06
6h	ACA1_369060/clean/scf7180000084664:230440-230527:+	ACA1_369060	Ubiquitin-related modifier 1 homolog [Source:UniProtKB/TrEMBL;Acc:L8GY50]	0.0554254	0.579132	0.5237066	4.03E-08
6h	ACA1_244760/clean/scf7180000084763:411555-411655:-	ACA1_244760	CitMHS domain-containing protein [Source:UniProtKB/TrEMBL;Acc:L8GMI1]	0.001655439	0.525280333	0.523624895	1.73E-16
6h	ACA1_325100/clean/scf7180000084475:966-1052:+	ACA1_325100	Calponin domain containing protein [Source:UniProtKB/TrEMBL;Acc:L8GPG8]	0.00268821	0.526192333	0.523504123	8.68E-18
6h	ACA1_042090/clean/scf7180000084573:55521-55587:+	ACA1_042090	hypothetical protein	0	0.523423	0.523423	0.000744481
6h	ACA1_098950/clean/scf7180000084646:73861-73927:+	ACA1_098950	hypothetical protein	0.020560043	0.543872333	0.52331229	3.84E-16
6h	ACA1_063740/clean/scf7180000084599:1493710-1493799:-	ACA1_063740	Transmembrane protein [Source:UniProtKB/TrEMBL;Acc:L8GXB1]	0.00049154	0.523787	0.52329546	3.06E-11
6h	ACA1_154230/clean/scf7180000084722:28525-28653:+	ACA1_154230	hypothetical protein	0.00691485	0.530164667	0.523249817	1.62E-28
6h	ACA1_291600/clean/scf7180000084777:783503-783632:-	ACA1_291600	Macro domain-containing protein [Source:UniProtKB/TrEMBL;Acc:L8HJG2]	0.008951763	0.532096667	0.523144903	1.57E-11
6h	ACA1_219830/clean/scf7180000084758:822151-822269:-	ACA1_219830	Fe2OG dioxygenase domain-containing protein [Source:UniProtKB/TrEMBL;Acc:L8GQS5]	0.031056467	0.554150333	0.523093867	6.48E-06
6h	ACA1_115420/clean/scf7180000084669:630252-630349:-	ACA1_115420	SMI1_KNR4 domain-containing protein [Source:UniProtKB/TrEMBL;Acc:L8H4J8]	0.0243855	0.547331	0.5229455	8.53E-08
6h	ACA1_393390/clean/scf7180000084756:54963-55084:+	ACA1_393390	Exostosin domain-containing protein [Source:UniProtKB/TrEMBL;Acc:L8H1N1]	0	0.522708333	0.522708333	1.25E-09
6h	ACA1_174480/clean/scf7180000084741:717073-717227:-	ACA1_174480	Usp domain-containing protein [Source:UniProtKB/TrEMBL;Acc:L8HHU8]	0.266985	0.789682333	0.522697333	2.32E-05
6h	ACA1_100480/clean/scf7180000084648:94586-94659:-	ACA1_100480	Fbox/LRR-repeat protein [Source:UniProtKB/TrEMBL;Acc:L8GLX9]	0.011247433	0.533898333	0.5226509	3.63E-06
6h	ACA1_234790/clean/scf7180000084762:317398-317491:-	ACA1_234790	PPM-type phosphatase domain-containing protein [Source:UniProtKB/TrEMBL;Acc:L8H3L1]	0.025024337	0.547610667	0.52258633	6.97E-13
6h	ACA1_225960/clean/scf7180000084761:31320-31402:+	ACA1_225960	EF hand domain containing protein [Source:UniProtKB/TrEMBL;Acc:L8H8N3]	0.003674453	0.525882	0.522207547	4.72E-11
6h	ACA1_031600/clean/scf7180000084522:23098-23182:-	ACA1_031600	Leucine rich repeat-containing protein [Source:UniProtKB/TrEMBL;Acc:L8GSM8]	0.0497884	0.571916333	0.522127933	9.37E-12
6h	ACA1_156050/clean/scf7180000084723:13874-13934:+	ACA1_156050	Armadillo/betacatenin-like repeat domain containing protein [Source:UniProtKB/TrEMBL;Acc:L8GIE3]	0	0.521891667	0.521891667	0.010847518
6h	ACA1_343300/clean/scf7180000084638:17201-17284:-	ACA1_343300	PHD-type domain-containing protein [Source:UniProtKB/TrEMBL;Acc:L8HBT2]	0.007984033	0.52947	0.521485967	2.08E-08
6h	ACA1_215970/clean/scf7180000084758:420997-421118:-	ACA1_215970	EGFlike domain containing protein [Source:UniProtKB/TrEMBL;Acc:L8GQ73]	0.0139255	0.535297	0.5213715	2.63E-05
6h	ACA1_080550/clean/scf7180000084618:32435-32524:+	ACA1_080550	FAD binding domain containing protein [Source:UniProtKB/TrEMBL;Acc:L8HBU6]	0.059774333	0.581016	0.521241667	5.36E-13
6h	ACA1_103150/clean/scf7180000084653:8166-8250:+	ACA1_103150	hypothetical protein	0.015877987	0.536791667	0.52091368	3.19E-17
6h	ACA1_234110/clean/scf7180000084762:203060-203129:+	ACA1_234110	Sulfurtransferase [Source:UniProtKB/TrEMBL;Acc:L8H2J9]	0.010293553	0.531050333	0.52075678	2.08E-19
6h	ACA1_072960/clean/scf7180000084603:1346124-1346195:+	ACA1_072960	F-box domain-containing protein [Source:UniProtKB/TrEMBL;Acc:L8HEK9]	0.013306967	0.533995333	0.520688367	1.23E-07
6h	ACA1_279800/clean/scf7180000084776:449696-449950:+	ACA1_279800	Peroxisomal biogenesis factor 11c, putative	0.036811567	0.557452333	0.520640767	9.01E-12
6h	ACA1_200100/clean/scf7180000084753:390262-390353:-	ACA1_200100	hypothetical protein	0.018299037	0.538463333	0.520164297	1.29E-11
6h	ACA1_107160/clean/scf7180000084660:161645-161783:+	ACA1_107160	hypothetical protein	0.009524433	0.529655	0.520130567	3.46E-11
6h	ACA1_144860/clean/scf7180000084710:27645-27722:+	ACA1_144860	PH domain containing protein [Source:UniProtKB/TrEMBL;Acc:L8GCF4]	0	0.520094667	0.520094667	4.16E-08
6h	ACA1_204520/clean/scf7180000084754:303775-303886:-	ACA1_204520	hypothetical protein	0	0.520067	0.520067	0.000516343
6h	ACA1_072730/clean/scf7180000084603:1278745-1278848:+	ACA1_072730	hypothetical protein	0.004975133	0.524746667	0.519771533	6.07E-09
6h	ACA1_135130/clean/scf7180000084702:68253-68315:-	ACA1_135130	SWIRM domain-containing protein [Source:UniProtKB/TrEMBL;Acc:L8GE50]	0.014419367	0.534058333	0.519638967	4.04E-07
6h	ACA1_176030/clean/scf7180000084741:1109438-1109561:-	ACA1_176030	ATPase, AAA domain containing protein [Source:UniProtKB/TrEMBL;Acc:L8HI90]	0.023034257	0.542635333	0.519601077	1.76E-06
6h	ACA1_037090/clean/scf7180000084567:51871-51953:+	ACA1_037090	Ankyrin repeat containing protein [Source:UniProtKB/TrEMBL;Acc:L8H0U5]	0.007302397	0.526849	0.519546603	2.28E-08
6h	ACA1_095220/clean/scf7180000084645:815769-815891:-	ACA1_095220	Protein kinase domain-containing protein [Source:UniProtKB/TrEMBL;Acc:L8GIR4]	0.026595767	0.546050667	0.5194549	1.38E-12
6h	ACA1_399930/clean/scf7180000084775:67294-67422:+	ACA1_399930	Ras subfamily protein [Source:UniProtKB/TrEMBL;Acc:L8GGR5]	0.00299663	0.522398667	0.519402037	2.53E-11
6h	ACA1_061900/clean/scf7180000084599:1074373-1074465:-	ACA1_061900	GTP_EFTU_D3 domain-containing protein [Source:UniProtKB/TrEMBL;Acc:L8GY16]	0.00069541	0.519921	0.51922559	1.07E-11
6h	ACA1_164900/clean/scf7180000084729:305191-305285:-	ACA1_164900	Bromo domain-containing protein [Source:UniProtKB/TrEMBL;Acc:L8GSQ5]	0.00887713	0.528014667	0.519137537	5.00E-10
6h	ACA1_002750/clean/scf7180000082937:4567-4630:-	ACA1_002750	F-box domain-containing protein [Source:UniProtKB/TrEMBL;Acc:L8GHD2]	0.017186633	0.536145	0.518958367	4.36E-10
6h	ACA1_259490/clean/scf7180000084769:354857-354932:+	ACA1_259490	TPR_REGION domain-containing protein [Source:UniProtKB/TrEMBL;Acc:L8GEZ4]	0	0.518918667	0.518918667	7.26E-10
6h	ACA1_103630/clean/scf7180000084653:47179-47296:+	ACA1_103630	Peroxidase [Source:UniProtKB/TrEMBL;Acc:L8GCR8]	0.002691551	0.521401	0.518709449	1.24E-28
6h	ACA1_109730/clean/scf7180000084663:11630-11733:-	ACA1_109730	Replication factor C subunit 1 [Source:UniProtKB/TrEMBL;Acc:L8HIF4]	0.0035138	0.522069	0.5185552	1.47E-11
6h	ACA1_143760/clean/scf7180000084709:91761-91824:-	ACA1_143760	hypothetical protein	0	0.518410667	0.518410667	7.00E-11
6h	ACA1_093570/clean/scf7180000084645:582645-582733:-	ACA1_093570	hypothetical protein	0.089444233	0.607749667	0.518305433	8.31E-10
6h	ACA1_235090/clean/scf7180000084762:336006-336100:-	ACA1_235090	Lysophospholipaselike protein [Source:UniProtKB/TrEMBL;Acc:L8H3L9]	0.01006165	0.528308333	0.518246683	2.69E-14
6h	ACA1_287820/clean/scf7180000084777:87080-87184:+	ACA1_287820	TRANSKETOLASE_1 domain-containing protein [Source:UniProtKB/TrEMBL;Acc:L8HL41]	0	0.518170667	0.518170667	4.22E-15
6h	ACA1_325610/clean/scf7180000084495:50077-50181:-	ACA1_325610	Protein kinase domain containing protein [Source:UniProtKB/TrEMBL;Acc:L8H900]	0.013648197	0.531818	0.518169803	3.73E-12
6h	ACA1_063950/clean/scf7180000084599:1542815-1542906:+	ACA1_063950	Fbox domain containing protein [Source:UniProtKB/TrEMBL;Acc:L8GZW1]	0	0.51797	0.51797	1.52E-07
6h	ACA1_256450/clean/scf7180000084769:33137-33225:+	ACA1_256450	Poly [ADP-ribose] polymerase [Source:UniProtKB/TrEMBL;Acc:L8GH34]	0	0.517913	0.517913	1.28E-07
6h	ACA1_063830/clean/scf7180000084599:1514650-1514755:+	ACA1_063830	hypothetical protein	0	0.517906	0.517906	1.00E-09
6h	ACA1_174680/clean/scf7180000084741:761015-761076:-	ACA1_174680	Acid phosphatase, putative [Source:UniProtKB/TrEMBL;Acc:L8HK72]	0.018428537	0.536181	0.517752463	1.35E-09
6h	ACA1_063870/clean/scf7180000084599:1522068-1522158:+	ACA1_063870	Metallophos domain-containing protein [Source:UniProtKB/TrEMBL;Acc:L8GYM9]	0.0306841	0.548361	0.5176769	2.49E-14
6h	ACA1_020770/clean/scf7180000084095:2994-3071:+	ACA1_020770	Multisensor hybrid histidine kinase [Source:UniProtKB/TrEMBL;Acc:L8GWG6]	0.050651433	0.568113667	0.517462233	1.33E-13
6h	ACA1_123220/clean/scf7180000084686:5779-5875:-	ACA1_123220	hypothetical protein	0.018999437	0.536215	0.517215563	4.49E-08
6h	ACA1_245510/clean/scf7180000084763:498620-498736:+	ACA1_245510	SAM domain (Sterile alpha motif) domain containing protein [Source:UniProtKB/TrEMBL;Acc:L8GKU3]	0.00203832	0.519249667	0.517211347	1.29E-07
6h	ACA1_087720/clean/scf7180000084639:105307-105395:+	ACA1_087720	Dihydrodipicolinate synthetase [Source:UniProtKB/TrEMBL;Acc:L8GVJ3]	0.001536097	0.518706667	0.51717057	7.99E-10
6h	ACA1_097130/clean/scf7180000084645:1166625-1166721:+	ACA1_097130	Serine/threonine kinase [Source:UniProtKB/TrEMBL;Acc:L8GIY5]	0.071373733	0.588492	0.517118267	1.97E-05
6h	ACA1_112100/clean/scf7180000084669:109259-109345:+	ACA1_112100	BTB/POZ domain containing protein [Source:UniProtKB/TrEMBL;Acc:L8H3Y0]	0.000709723	0.517698333	0.51698861	3.35E-08
6h	ACA1_108260/clean/scf7180000084661:23165-23238:-	ACA1_108260	Prenylcys_lyase domain-containing protein [Source:UniProtKB/TrEMBL;Acc:L8GD56]	0.195444	0.712382333	0.516938333	4.08E-06
6h	ACA1_383540/clean/scf7180000084721:205823-205891:-	ACA1_383540	hypothetical protein	0.01382954	0.530659333	0.516829793	6.31E-08
6h	ACA1_320090/clean/scf7180000084352:1706-1820:+	ACA1_320090	hypothetical protein	0.012330567	0.529002	0.516671433	5.32E-07
6h	ACA1_301240/clean/scf7180000083203:7657-7747:+	ACA1_301240	Usp domain-containing protein [Source:UniProtKB/TrEMBL;Acc:L8H1P7]	0.00420571	0.520713667	0.516507957	4.55E-12
6h	ACA1_178250/clean/scf7180000084743:190407-190567:-	ACA1_178250	hypothetical protein	0.005649733	0.522094333	0.5164446	1.22E-07
6h	ACA1_189600/clean/scf7180000084748:62792-62864:-	ACA1_189600	hypothetical protein	0.01225177	0.528666667	0.516414897	1.03E-18
6h	ACA1_396260/clean/scf7180000084759:66490-66584:+	ACA1_396260	hypothetical protein	0.012461093	0.528674333	0.51621324	1.23E-10
6h	ACA1_077300/clean/scf7180000084612:287216-287304:+	ACA1_077300	Serine/threonine kinase [Source:UniProtKB/TrEMBL;Acc:L8GP03]	0.001931247	0.518133	0.516201753	4.17E-06
6h	ACA1_214970/clean/scf7180000084758:283058-283130:-	ACA1_214970	SNF2 family Nterminal domain containing protein [Source:UniProtKB/TrEMBL;Acc:L8GR02]	0.004329	0.520423667	0.516094667	7.99E-08
6h	ACA1_376220/clean/scf7180000084685:57232-57317:-	ACA1_376220	Breast carcinoma amplified sequence 2 (Bcas2) protein [Source:UniProtKB/TrEMBL;Acc:L8HFZ3]	0.008975133	0.525020333	0.5160452	5.43E-17
6h	ACA1_326090/clean/scf7180000084504:19588-19712:-	ACA1_326090	hypothetical protein	0.00049766	0.516385333	0.515887673	7.68E-12
6h	ACA1_068620/clean/scf7180000084603:240766-240843:-	ACA1_068620	hypothetical protein	0.000408413	0.515984333	0.51557592	2.49E-11
6h	ACA1_067410/clean/scf7180000084601:17627-17716:-	ACA1_067410	F-box domain-containing protein [Source:UniProtKB/TrEMBL;Acc:L8GTP9]	0.018263567	0.533775667	0.5155121	5.94E-08
6h	ACA1_093490/clean/scf7180000084645:558740-558823:+	ACA1_093490	Arf-GAP domain-containing protein [Source:UniProtKB/TrEMBL;Acc:L8GJ65]	9.82E-05	0.515528	0.515429831	2.01E-13
6h	ACA1_349640/clean/scf7180000084690:23441-23527:+	ACA1_349640	HEAT repeat domain containing protein [Source:UniProtKB/TrEMBL;Acc:L8GKB3]	0	0.515416333	0.515416333	2.64E-11
6h	ACA1_018780/clean/scf7180000083912:16536-16630:-	ACA1_018780	AAA domain-containing protein [Source:UniProtKB/TrEMBL;Acc:L8HMV6]	0.010675267	0.525877	0.515201733	1.16E-10
6h	ACA1_195240/clean/scf7180000084751:82355-82410:+	ACA1_195240	CBS domain containing protein [Source:UniProtKB/TrEMBL;Acc:L8H5D8]	0.0660312	0.580791333	0.514760133	3.98E-16
6h	ACA1_197300/clean/scf7180000084753:918-994:-	ACA1_197300	hypothetical protein	0.0889095	0.603574	0.5146645	5.11E-11
6h	ACA1_380970/clean/scf7180000084712:130020-130098:+	ACA1_380970	ANK_REP_REGION domain-containing protein [Source:UniProtKB/TrEMBL;Acc:L8GMX1]	0.010928967	0.525532	0.514603033	1.13E-07
6h	ACA1_234250/clean/scf7180000084762:252254-252401:-	ACA1_234250	Protein kinase domain-containing protein [Source:UniProtKB/TrEMBL;Acc:L8H190]	0.001459707	0.516018	0.514558293	3.01E-11
6h	ACA1_265960/clean/scf7180000084770:554280-554391:-	ACA1_265960	Mac domain-containing protein [Source:UniProtKB/TrEMBL;Acc:L8H4H0]	0.016797667	0.531333333	0.514535667	2.08E-16
6h	ACA1_279550/clean/scf7180000084776:413027-413129:-	ACA1_279550	CBS domain containing protein [Source:UniProtKB/TrEMBL;Acc:L8H8C1]	0.00205264	0.516421333	0.514368693	1.60E-11
6h	ACA1_096760/clean/scf7180000084645:1097981-1098124:+	ACA1_096760	NIF3 (NGG1p interacting factor 3) domain containing protein [Source:UniProtKB/TrEMBL;Acc:L8GJJ9]	0	0.514357	0.514357	1.25E-10
6h	ACA1_265940/clean/scf7180000084770:551456-551543:+	ACA1_265940	hypothetical protein	0.0328266	0.547157667	0.514331067	5.13E-14
6h	ACA1_232420/clean/scf7180000084762:22374-22498:+	ACA1_232420	TPR_REGION domain-containing protein [Source:UniProtKB/TrEMBL;Acc:L8H3B0]	0.053622867	0.567878333	0.514255467	1.24E-13
6h	ACA1_367890/clean/scf7180000084664:9478-9562:-	ACA1_367890	hypothetical protein	0.0050505	0.519289667	0.514239167	1.95E-07
6h	ACA1_139830/clean/scf7180000084707:6998-7101:-	ACA1_139830	Eukaryotic translation initiation factor 3 subunit L [Source:UniProtKB/TrEMBL;Acc:L8GIZ4]	0.00112027	0.514789667	0.513669397	2.09E-12
6h	ACA1_198390/clean/scf7180000084753:101759-101836:-	ACA1_198390	hypothetical protein	0.00498763	0.518640333	0.513652703	1.22E-15
6h	ACA1_385460/clean/scf7180000084730:177306-177382:-	ACA1_385460	PH domain containing protein [Source:UniProtKB/TrEMBL;Acc:L8H8E0]	0.045175767	0.558788333	0.513612567	7.06E-08
6h	ACA1_069100/clean/scf7180000084603:355575-355666:+	ACA1_069100	Lactamase_B domain-containing protein [Source:UniProtKB/TrEMBL;Acc:L8HFU9]	0.0895108	0.603094333	0.513583533	2.86E-08
6h	ACA1_232260/clean/scf7180000084762:9428-9542:+	ACA1_232260	hypothetical protein	0.00988084	0.523441	0.51356016	7.57E-07
6h	ACA1_053890/clean/scf7180000084594:345261-345378:-	ACA1_053890	Aconitate hydratase, mitochondrial [Source:UniProtKB/TrEMBL;Acc:L8H690]	0.000452951	0.513677	0.513224049	9.79E-18
6h	ACA1_011280/clean/scf7180000083623:8206-8281:+	ACA1_011280	AAA domain-containing protein [Source:UniProtKB/TrEMBL;Acc:L8GP61]	0.002222223	0.515389	0.513166777	4.38E-11
6h	ACA1_153210/clean/scf7180000084720:90197-90290:-	ACA1_153210	Solute carrier family protein [Source:UniProtKB/TrEMBL;Acc:L8HFV0]	0.0179089	0.530953	0.5130441	1.31E-06
6h	ACA1_183660/clean/scf7180000084745:476556-476620:+	ACA1_183660	Phospholipase B-like [Source:UniProtKB/TrEMBL;Acc:L8H8D7]	0.012715663	0.525701667	0.512986003	4.67E-21
6h	ACA1_016690/clean/scf7180000083833:6343-6408:-	ACA1_016690	hypothetical protein	0	0.512958667	0.512958667	2.45E-07
6h	ACA1_109100/clean/scf7180000084661:116146-116223:-	ACA1_109100	Sodium symporter, putative [Source:UniProtKB/TrEMBL;Acc:L8GC73]	0	0.512887333	0.512887333	2.83E-07
6h	ACA1_306840/clean/scf7180000083668:16847-16972:+	ACA1_306840	RTP1_C1 domain-containing protein [Source:UniProtKB/TrEMBL;Acc:L8GQF6]	0.004668	0.517444333	0.512776333	2.73E-08
6h	ACA1_352370/clean/scf7180000084704:3253-3320:+	ACA1_352370	Oxidoreductase, short chain dehydrogenase/reductase superfamily protein [Source:UniProtKB/TrEMBL;Acc:L8GTQ2]	0.00559609	0.518268	0.51267191	1.39E-08
6h	ACA1_266230/clean/scf7180000084770:635053-635149:+	ACA1_266230	Dilute domain-containing protein [Source:UniProtKB/TrEMBL;Acc:L8H2D0]	0	0.512585	0.512585	0.001223887
6h	ACA1_211500/clean/scf7180000084758:89745-89807:+	ACA1_211500	RAB5B protein, putative [Source:UniProtKB/TrEMBL;Acc:L8GS95]	0.055944067	0.568528667	0.5125846	0.000112114
6h	ACA1_349090/clean/scf7180000084689:27172-27287:-	ACA1_349090	3'-5' exonuclease domain-containing protein [Source:UniProtKB/TrEMBL;Acc:L8GV60]	0.009677433	0.522176667	0.512499233	4.63E-07
6h	ACA1_062850/clean/scf7180000084599:1286012-1286095:+	ACA1_062850	WD_REPEATS_REGION domain-containing protein [Source:UniProtKB/TrEMBL;Acc:L8GZK3]	0	0.51243	0.51243	1.22E-12
6h	ACA1_076720/clean/scf7180000084612:121950-122024:-	ACA1_076720	Fbox domain containing protein [Source:UniProtKB/TrEMBL;Acc:L8GLV4]	0.01048928	0.522895667	0.512406387	9.40E-09
6h	ACA1_195200/clean/scf7180000084751:72092-72174:+	ACA1_195200	F-box domain-containing protein [Source:UniProtKB/TrEMBL;Acc:L8H6S1]	0.004901967	0.517228333	0.512326367	7.65E-08
6h	ACA1_053470/clean/scf7180000084594:286488-286567:-	ACA1_053470	hypothetical protein	0.009652837	0.521898	0.512245163	1.26E-07
6h	ACA1_024200/clean/scf7180000084441:15525-15618:-	ACA1_024200	WD_REPEATS_REGION domain-containing protein [Source:UniProtKB/TrEMBL;Acc:L8GKS0]	0	0.512131667	0.512131667	1.07E-07
6h	ACA1_229570/clean/scf7180000084761:280569-280672:-	ACA1_229570	Oxidoreductase, short chain dehydrogenase/reductase superfamily protein [Source:UniProtKB/TrEMBL;Acc:L8H814]	0	0.512077667	0.512077667	6.96E-10

6h	ACA1_197890/clean/scf7180000084753:48477-48554:+	ACA1_197890	hypothetical protein	0.00461479	0.516597	0.51198221	2.73E-11
6h	ACA1_171960/clean/scf7180000084741:210738-210846:-	ACA1_171960	F-box domain-containing protein [Source:UniProtKB/TrEMBL;Acc:L8HHE3]	0.0155039	0.527351667	0.511847767	3.77E-06
6h	ACA1_020880/clean/scf7180000084095:30828-30926:+	ACA1_020880	Protozoan/cyanobacterial globin family subfamily protein [Source:UniProtKB/TrEMBL;Acc:L8GU16]	0.036448767	0.548190333	0.511741567	5.27E-22
6h	ACA1_223310/clean/scf7180000084760:128282-128363:-	ACA1_223310	Amidohydro_3 domain-containing protein [Source:UniProtKB/TrEMBL;Acc:L8GTU8]	0	0.511700667	0.511700667	1.50E-08
6h	ACA1_298660/clean/scf7180000082992:15906-16011:+	ACA1_298660	Bromo domain-containing protein [Source:UniProtKB/TrEMBL;Acc:L8H1K3]	0.000278242	0.511968	0.511689758	2.25E-10
6h	ACA1_108830/clean/scf7180000084661:90816-90901:+	ACA1_108830	Terpene cyclase/mutase family member [Source:UniProtKB/TrEMBL;Acc:L8GD76]	0	0.51154	0.51154	6.39E-14
6h	ACA1_095700/clean/scf7180000084645:889709-889816:+	ACA1_095700	hypothetical protein	0	0.511488	0.511488	3.66E-07
6h	ACA1_061780/clean/scf7180000084599:1042427-1042517:+	ACA1_061780	Serine/threonine kinase [Source:UniProtKB/TrEMBL;Acc:L8GWW2]	0.010582	0.521976667	0.511394667	5.45E-08
6h	ACA1_228760/clean/scf7180000084761:208247-208349:+	ACA1_228760	Mitochondrial carrier protein [Source:UniProtKB/TrEMBL;Acc:L8H8X8]	0.021611733	0.532918667	0.511306933	4.74E-13
6h	ACA1_140730/clean/scf7180000084707:108943-109027:-	ACA1_140730	Kelch repeat protein [Source:UniProtKB/TrEMBL;Acc:L8GGS1]	0.006833003	0.518082333	0.51124933	8.11E-10
6h	ACA1_064810/clean/scf7180000084599:1704201-1704283:-	ACA1_064810	Transporter, major facilitator subfamily protein [Source:UniProtKB/TrEMBL;Acc:L8GWU0]	0	0.511209	0.511209	3.89E-09
6h	ACA1_140770/clean/scf7180000084707:117224-117327:+	ACA1_140770	ATPase, AAA domain containing protein [Source:UniProtKB/TrEMBL;Acc:L8GJ22]	0.015077933	0.525967	0.510889067	2.54E-10
6h	ACA1_153010/clean/scf7180000084720:53815-53877:-	ACA1_153010	hypothetical protein	0.044908967	0.555719667	0.5108107	4.09E-14
6h	ACA1_377740/clean/scf7180000084711:113771-113874:-	ACA1_377740	2Fe-2S ferredoxin-type domain-containing protein [Source:UniProtKB/TrEMBL;Acc:L8GR90]	0.007965197	0.518765333	0.510800137	5.73E-24
6h	ACA1_189390/clean/scf7180000084748:24252-24325:-	ACA1_189390	hypothetical protein	0	0.510673667	0.510673667	3.46E-08
6h	ACA1_244800/clean/scf7180000084763:426306-426389:-	ACA1_244800	Usp domain-containing protein [Source:UniProtKB/TrEMBL;Acc:L8GK21]	0.030303033	0.540968333	0.5106653	9.97E-05
6h	ACA1_233420/clean/scf7180000084762:138350-138427:+	ACA1_233420	hypothetical protein	0.00832397	0.518858333	0.510534363	5.98E-42
6h	ACA1_281340/clean/scf7180000084776:593104-593186:-	ACA1_281340	Ydr279_N domain-containing protein [Source:UniProtKB/TrEMBL;Acc:L8H7C2]	0.095841467	0.60632	0.510478533	1.33E-07
6h	ACA1_352370/clean/scf7180000084704:2343-2421:+	ACA1_352370	Oxidoreductase, short chain dehydrogenase/reductase superfamily protein [Source:UniProtKB/TrEMBL;Acc:L8GTQ2]	0.00640864	0.516766333	0.510357693	2.09E-08
6h	ACA1_082180/clean/scf7180000084623:19916-20024:+	ACA1_082180	N-acetyltransferase domain-containing protein [Source:UniProtKB/TrEMBL;Acc:L8GMF4]	0.011845337	0.522099333	0.510253997	2.71E-13
6h	ACA1_369220/clean/scf7180000084664:274857-274921:-	ACA1_369220	RBR-type E3 ubiquitin transferase [Source:UniProtKB/TrEMBL;Acc:L8GYY2]	0.001539123	0.511741	0.510201877	7.51E-10
6h	ACA1_296280/clean/scf7180000084777:1313574-1313673:+	ACA1_296280	RhoGEF domain containing protein [Source:UniProtKB/TrEMBL;Acc:L8HJZ8]	0.0269132	0.537113333	0.510200133	1.58E-07
6h	ACA1_383560/clean/scf7180000084721:208395-208461:-	ACA1_383560	TBC domain containing protein [Source:UniProtKB/TrEMBL;Acc:L8GW44]	0.039437867	0.549578333	0.510140467	4.90E-09
6h	ACA1_234060/clean/scf7180000084762:191723-191789:-	ACA1_234060	SPRY domain containing protein [Source:UniProtKB/TrEMBL;Acc:L8H2J5]	0.0389228	0.549044	0.5101212	1.42E-10
6h	ACA1_181910/clean/scf7180000084745:72043-72122:-	ACA1_181910	CrcB family protein [Source:UniProtKB/TrEMBL;Acc:L8H804]	0.004282233	0.514287333	0.5100051	5.10E-08
6h	ACA1_065890/clean/scf7180000084599:1941150-1941246:+	ACA1_065890	Beta-lactamase domain-containing protein [Source:UniProtKB/TrEMBL;Acc:L8GZ04]	0.0206642	0.530573	0.5099088	9.15E-09
6h	ACA1_321930/clean/scf7180000084457:17839-17945:+	ACA1_321930	Transporter, major intrinsic protein (MIP) superfamily protein [Source:UniProtKB/TrEMBL;Acc:L8HCR2]	0.00276967	0.512556667	0.509786997	2.81E-09
6h	ACA1_015490/clean/scf7180000083799:3028-3128:+	ACA1_015490	hypothetical protein	0.01686516	0.526642667	0.509777507	2.53E-11
6h	ACA1_276410/clean/scf7180000084774:169469-169552:+	ACA1_276410	Phosphate transporter [Source:UniProtKB/TrEMBL;Acc:L8GTH7]	0.011553613	0.521304667	0.509751053	1.15E-09
6h	ACA1_178230/clean/scf7180000084743:183875-183989:+	ACA1_178230	Transducin family protein [Source:UniProtKB/TrEMBL;Acc:L8GSK8]	0.056729467	0.566383667	0.5096542	1.32E-10
6h	ACA1_295270/clean/scf7180000084777:1088137-1088304:-	ACA1_295270	Rhodanese domain-containing protein [Source:UniProtKB/TrEMBL;Acc:L8HJQ3]	0.020202653	0.529852	0.509649347	1.09E-12
6h	ACA1_108830/clean/scf7180000084661:93198-93283:+	ACA1_108830	Terpene cyclase/mutase family member [Source:UniProtKB/TrEMBL;Acc:L8GD76]	0.01050282	0.520147333	0.509644513	3.98E-19
6h	ACA1_187290/clean/scf7180000084747:21323-21434:-	ACA1_187290	hypothetical protein	0.01176454	0.521389667	0.509625127	1.87E-07
6h	ACA1_184160/clean/scf7180000084745:573081-573213:+	ACA1_184160	AAA domain-containing protein [Source:UniProtKB/TrEMBL;Acc:L8H8H4]	0.002275297	0.511785667	0.50951037	6.71E-18
6h	ACA1_152960/clean/scf7180000084720:41370-41458:+	ACA1_152960	Malate dehydrogenase [Source:UniProtKB/TrEMBL;Acc:L8HFT9]	0.000759762	0.510202333	0.509442571	1.83E-33
6h	ACA1_321380/clean/scf7180000084439:16194-16281:+	ACA1_321380	RasGEF domain containing protein [Source:UniProtKB/TrEMBL;Acc:L8HKN6]	0	0.509376667	0.509376667	3.20E-07
6h	ACA1_272530/clean/scf7180000084772:55711-55787:+	ACA1_272530	VWFA domain-containing protein [Source:UniProtKB/TrEMBL;Acc:L8HIL5]	0.003968267	0.513308	0.509339733	1.52E-09
6h	ACA1_069480/clean/scf7180000084603:450852-450944:-	ACA1_069480	MFS domain-containing protein [Source:UniProtKB/TrEMBL;Acc:L8HCU0]	0.010416667	0.519736333	0.509319667	9.05E-07
6h	ACA1_153650/clean/scf7180000084720:149191-149261:+	ACA1_153650	Pterin4-alpha-carbinolamine dehydratase [Source:UniProtKB/TrEMBL;Acc:L8HG82]	0.037687367	0.546984	0.509296633	7.45E-18
6h	ACA1_178530/clean/scf7180000084743:254362-254496:-	ACA1_178530	Dual specificity phosphatase, catalytic domain containing protein [Source:UniProtKB/TrEMBL;Acc:L8GSL7]	0.011464433	0.520635667	0.509171233	0.000100408
6h	ACA1_173330/clean/scf7180000084741:465227-465294:+	ACA1_173330	Fbox domain containing protein [Source:UniProtKB/TrEMBL;Acc:L8HHQ0]	0.196866333	0.705827	0.508960667	7.93E-07
6h	ACA1_129970/clean/scf7180000084698:60113-60219:+	ACA1_129970	Cation transport domain containing protein [Source:UniProtKB/TrEMBL;Acc:L8GPJ3]	0.0398249	0.548781	0.5089561	1.80E-09
6h	ACA1_219070/clean/scf7180000084758:710751-710831:+	ACA1_219070	Rieske domain-containing protein [Source:UniProtKB/TrEMBL;Acc:L8GQP6]	0	0.508903333	0.508903333	5.47E-13
6h	ACA1_187900/clean/scf7180000084747:96152-96235:-	ACA1_187900	F-box domain-containing protein [Source:UniProtKB/TrEMBL;Acc:L8GTD5]	0.038813867	0.547275	0.508461133	1.24E-12
6h	ACA1_326860/clean/scf7180000084510:55930-56018:-	ACA1_326860	Lipase_3 domain-containing protein [Source:UniProtKB/TrEMBL;Acc:L8HKH4]	0.028218667	0.536678333	0.508459667	1.88E-06
6h	ACA1_282930/clean/scf7180000084776:749553-749654:-	ACA1_282930	Aminotran_1_2 domain-containing protein [Source:UniProtKB/TrEMBL;Acc:L8H7B5]	0.021189823	0.52952	0.508330177	1.59E-06
6h	ACA1_096520/clean/scf7180000084645:1032991-1033146:-	ACA1_096520	Cytochrome p450 superfamily protein [Source:UniProtKB/TrEMBL;Acc:L8GLA2]	0.086023897	0.594282	0.508258103	0.001130274
6h	ACA1_200560/clean/scf7180000084753:448510-448602:-	ACA1_200560	Calcium binding atopyrelated autoantigen 1, putative [Source:UniProtKB/TrEMBL;Acc:L8H3D2]	0.013071763	0.521307	0.508235237	7.69E-13
6h	ACA1_279270/clean/scf7180000084776:344789-344860:+	ACA1_279270	RNA_ligase domain-containing protein [Source:UniProtKB/TrEMBL;Acc:L8H728]	0.000120852	0.508331	0.508210148	2.55E-15
6h	ACA1_054420/clean/scf7180000084594:489284-489375:+	ACA1_054420	N-formylglutamate amidohydrolase [Source:UniProtKB/TrEMBL;Acc:L8H8B3]	0	0.508193	0.508193	9.12E-09
6h	ACA1_384780/clean/scf7180000084730:101879-101987:+	ACA1_384780	MIT domain containing protein [Source:UniProtKB/TrEMBL;Acc:L8H937]	0.132293933	0.640345333	0.5080514	1.14E-12
6h	ACA1_319980/clean/scf7180000084259:22116-22235:-	ACA1_319980	PDEase domain-containing protein [Source:UniProtKB/TrEMBL;Acc:L8HLY7]	0.030132867	0.53818	0.508047133	3.93E-18
6h	ACA1_201360/clean/scf7180000084753:530840-530910:-	ACA1_201360	Regulator of chromosome condensation (RCC1) repeat domain containing protein [Source:UniProtKB/TrEMBL;Acc:L8H4W1]	0.0280956	0.535989	0.5078934	1.04E-09
6h	ACA1_243850/clean/scf7180000084763:360846-360971:+	ACA1_243850	Ras-GAP domain-containing protein [Source:UniProtKB/TrEMBL;Acc:L8GJZ5]	0.141576233	0.649457667	0.507881433	0.006738418
6h	ACA1_027610/clean/scf7180000084469:11042-11116:+	ACA1_027610	hypothetical protein	0.0297633	0.537563	0.5077997	5.15E-06
6h	ACA1_063220/clean/scf7180000084599:1360115-1360189:+	ACA1_063220	PDZ domain-containing protein [Source:UniProtKB/TrEMBL;Acc:L8GYE7]	0.006944433	0.514678333	0.5077339	3.68E-08
6h	ACA1_062650/clean/scf7180000084599:1263319-1263414:+	ACA1_062650	hypothetical protein	0.0148465	0.522504	0.5076575	1.30E-06
6h	ACA1_277990/clean/scf7180000084776:148268-148342:+	ACA1_277990	Calponin domain containing protein [Source:UniProtKB/TrEMBL;Acc:L8H800]	0	0.507511	0.507511	1.52E-06
6h	ACA1_210400/clean/scf7180000084758:39920-40009:-	ACA1_210400	PPM-type phosphatase domain-containing protein [Source:UniProtKB/TrEMBL;Acc:L8GQQ0]	0.0099049	0.517235	0.5073301	8.90E-13
6h	ACA1_052770/clean/scf7180000084594:176437-176538:-	ACA1_052770	Ubiquitinconjugating enzyme subfamily protein [Source:UniProtKB/TrEMBL;Acc:L8H635]	0	0.50729	0.50729	5.00E-08
6h	ACA1_378010/clean/scf7180000084711:163514-163603:+	ACA1_378010	JmjC domain-containing protein [Source:UniProtKB/TrEMBL;Acc:L8GRT4]	0.461475667	0.968679	0.507203333	4.00E-09
6h	ACA1_362250/clean/scf7180000084624:36792-36861:+	ACA1_362250	hypothetical protein	0.064612533	0.571779	0.507166467	9.40E-08
6h	ACA1_216320/clean/scf7180000084758:466814-466955:+	ACA1_216320	Homogentisate 1,2dioxygenase [Source:UniProtKB/TrEMBL;Acc:L8GQ83]	0.000259489	0.507279	0.507019511	9.67E-15
6h	ACA1_368670/clean/scf7180000084664:134366-134428:-	ACA1_368670	Mitochondrial GTPase 1 [Source:UniProtKB/TrEMBL;Acc:L8GYT9]	0.013798833	0.520783667	0.506984833	4.35E-06
6h	ACA1_173140/clean/scf7180000084741:410239-410321:+	ACA1_173140	Deacetylase sirtuin-type domain-containing protein [Source:UniProtKB/TrEMBL;Acc:L8HJX9]	0.0265148	0.533495667	0.506980867	1.43E-10
6h	ACA1_321380/clean/scf7180000084439:15988-16103:+	ACA1_321380	RasGEF domain containing protein [Source:UniProtKB/TrEMBL;Acc:L8HKN6]	0.038697167	0.545658	0.506960833	4.93E-06
6h	ACA1_016010/clean/scf7180000083800:13409-13521:-	ACA1_016010	hypothetical protein	0.0523773	0.559278	0.5069007	1.31E-08
6h	ACA1_328770/clean/scf7180000084560:25823-25892:-	ACA1_328770	Aldo_ket_red domain-containing protein [Source:UniProtKB/TrEMBL;Acc:L8GQZ7]	0.001279358	0.508174	0.506894642	4.26E-23
6h	ACA1_145180/clean/scf7180000084710:53681-53750:-	ACA1_145180	F-box domain-containing protein [Source:UniProtKB/TrEMBL;Acc:L8GCU3]	0.001710653	0.508563	0.506852347	2.14E-08
6h	ACA1_276080/clean/scf7180000084774:131407-131493:+	ACA1_276080	Serine 3-dehydrogenase [Source:UniProtKB/TrEMBL;Acc:L8GS19]	0.0885002	0.595321667	0.506821467	1.24E-07
6h	ACA1_201510/clean/scf7180000084753:576658-576747:+	ACA1_201510	Ubiquitin fusion degradation protein, putative [Source:UniProtKB/TrEMBL;Acc:L8H4X5]	0.004901967	0.511579333	0.506677367	9.60E-08
6h	ACA1_106940/clean/scf7180000084660:151224-151352:-	ACA1_106940	TPT domain-containing protein [Source:UniProtKB/TrEMBL;Acc:L8GNI5]	0.011646313	0.518288	0.506641687	7.00E-14
6h	ACA1_024410/clean/scf7180000084442:17621-17719:+	ACA1_024410	Serine/threonineprotein kinase [Source:UniProtKB/TrEMBL;Acc:L8H7W5]	0.003173949	0.509799	0.506625051	2.60E-12
6h	ACA1_019140/clean/scf7180000083937:2662-2754:-	ACA1_019140	Regulator of chromosome condensation (RCC1) repeat domain containing protein [Source:UniProtKB/TrEMBL;Acc:L8HI50]	0.008667533	0.515257667	0.506590133	5.66E-08
6h	ACA1_123630/clean/scf7180000084686:46779-46850:-	ACA1_123630	hypothetical protein	0.170136667	0.676603333	0.506466667	3.01E-08
6h	ACA1_238410/clean/scf7180000084763:86954-87061:+	ACA1_238410	hypothetical protein	0.009354863	0.515640333	0.50628547	1.16E-18
6h	ACA1_224950/clean/scf7180000084760:290473-290588:-	ACA1_224950	ANK_REP_REGION domain-containing protein [Source:UniProtKB/TrEMBL;Acc:L8GSF6]	0.0107527	0.516999667	0.506246967	1.19E-06
6h	ACA1_323430/clean/scf7180000084468:21892-21996:+	ACA1_323430	SnoaL family polyketide cyclase [Source:UniProtKB/TrEMBL;Acc:L8H0W6]	0.003929313	0.510162333	0.50623302	2.44E-23
6h	ACA1_174000/clean/scf7180000084741:569158-569235:-	ACA1_174000	hypothetical protein	0.010990837	0.517082	0.506091163	1.71E-25
6h	ACA1_095290/clean/scf7180000084645:829918-830018:+	ACA1_095290	DUF3533 domain-containing protein [Source:UniProtKB/TrEMBL;Acc:L8GJ71]	0.050919133	0.556875667	0.505956533	4.49E-06
6h	ACA1_060270/clean/scf7180000084599:632676-632780:-	ACA1_060270	TYR_PHOSPHATASE_2 domain-containing protein [Source:UniProtKB/TrEMBL;Acc:L8GWC1]	0.012682023	0.518547	0.505864977	4.02E-11
6h	ACA1_178710/clean/scf7180000084743:267426-267491:-	ACA1_178710	ADPribosylation factor related protein 1, putative [Source:UniProtKB/TrEMBL;Acc:L8GTG3]	0.005208333	0.511068	0.505859667	4.65E-09
6h	ACA1_096520/clean/scf7180000084645:1034256-1034328:-	ACA1_096520	Cytochrome p450 superfamily protein [Source:UniProtKB/TrEMBL;Acc:L8GLA2]	0	0.505797667	0.505797667	0.000351084
6h	ACA1_071970/clean/scf7180000084603:1118651-1118742:+	ACA1_071970	Carn_acyltransf domain-containing protein [Source:UniProtKB/TrEMBL;Acc:L8HEI6]	0.0036232	0.509385667	0.505762467	9.72E-10
6h	ACA1_077090/clean/scf7180000084612:235055-235248:-	ACA1_077090	Transmembrane 9 superfamily member [Source:UniProtKB/TrEMBL;Acc:L8GLA1]	0.002282104	0.508025667	0.505743562	9.30E-12
6h	ACA1_035460/clean/scf7180000084564:56282-56378:+	ACA1_035460	Sugar (GlycosidePentoside-hexuronide) transporter, putative [Source:UniProtKB/TrEMBL;Acc:L8HCV9]	0.0371963	0.542931667	0.505735367	3.48E-08
6h	ACA1_052510/clean/scf7180000084594:92523-92612:+	ACA1_052510	PQ loop repeat family protein [Source:UniProtKB/TrEMBL;Acc:L8H5Y5]	0.005972477	0.511662667	0.50569019	2.31E-12
6h	ACA1_087880/clean/scf7180000084639:122870-122957:-	ACA1_087880	Beta-amylase [Source:UniProtKB/TrEMBL;Acc:L8GVJ7]	0	0.505542	0.505542	3.38E-10
6h	ACA1_088960/clean/scf7180000084639:275276-275368:-	ACA1_088960	Beta-lactamase domain-containing protein [Source:UniProtKB/TrEMBL;Acc:L8GU04]	0.00347722	0.509004667	0.505527447	1.26E-07
6h	ACA1_400220/clean/scf7180000084775:122387-122477:-	ACA1_400220	Abhydrolase_2 domain-containing protein [Source:UniProtKB/TrEMBL;Acc:L8GG24]	0.0158064	0.521332667	0.505526267	9.86E-15
6h	ACA1_220170/clean/scf7180000084758:881997-882104:+	ACA1_220170	RSL1D1 protein [Source:UniProtKB/TrEMBL;Acc:L8GRK5]	0	0.505373333	0.505373333	1.13E-07
6h	ACA1_393070/clean/scf7180000084756:39791-39886:+	ACA1_393070	Vault protein interalpha-trypsin [Source:UniProtKB/TrEMBL;Acc:L8H2R0]	0	0.505371667	0.505371667	1.02E-09
6h	ACA1_098000/clean/scf7180000084645:1372836-1372919:+	ACA1_098000	SAM domain-containing protein [Source:UniProtKB/TrEMBL;Acc:L8GK75]	0	0.505248	0.505248	9.23E-08
6h	ACA1_034150/clean/scf7180000084561:36366-36471:-	ACA1_034150	hypothetical protein	0.001825307	0.507028	0.505202693	3.93E-11
6h	ACA1_072990/clean/scf7180000084603:1350480-1350572:-	ACA1_072990	hypothetical protein	0.062223533	0.567340333	0.5051168	2.50E-06
6h	ACA1_053290/clean/scf7180000084594:249958-250104:+	ACA1_053290	V-type proton ATPase proteolipid subunit [Source:UniProtKB/TrEMBL;Acc:L8H836]	0.003658513	0.508513	0.504854487	3.22E-43
6h	ACA1_310750/clean/scf7180000083780:5265-5743:+	ACA1_310750	RFX-type winged-helix domain-containing protein [Source:UniProtKB/TrEMBL;Acc:L8GVY4]	0.0943432	0.599172333	0.504829133	5.77E-10
6h	ACA1_229570/clean/scf7180000084761:281105-281197:-	ACA1_229570	Oxidoreductase, short chain dehydrogenase/reductase superfamily protein [Source:UniProtKB/TrEMBL;Acc:L8H814]	0.009126151	0.513931333	0.504805182	1.78E-09
6h	ACA1_068740/clean/scf7180000084603:268724-268797:+	ACA1_068740	tRNAdihydrouridine synthase A, putative [Source:UniProtKB/TrEMBL;Acc:L8HDM1]	0.049591387	0.554298333	0.504706947	0.000386757
6h	ACA1_251550/clean/scf7180000084768:82104-82171:-	ACA1_251550	5' nucleotidase family protein [Source:UniProtKB/TrEMBL;Acc:L8HC09]	0.0033841	0.508059333	0.504675233	3.62E-08
6h	ACA1_052440/clean/scf7180000084594:75891-75961:-	ACA1_052440	GPI mannosyltransferase 1 [Source:UniProtKB/TrEMBL;Acc:L8H760]	0.0999213	0.604432667	0.504511367	4.06E-07
6h	ACA1_366300/clean/scf7180000084641:388036-388173:+	ACA1_366300	RRM domain-containing protein [Source:UniProtKB/TrEMBL;Acc:L8GMN9]	0.003570733	0.508005333	0.5044346	9.50E-10
6h	ACA1_091480/clean/scf7180000084645:130881-130949:-	ACA1_091480	TBPIP domain-containing protein [Source:UniProtKB/TrEMBL;Acc:L8GIS6]	0.005688267	0.510099333	0.504411067	1.12E-15
6h	ACA1_282880/clean/scf7180000084776:740461-740523:+	ACA1_282880	DUF667 domain-containing protein [Source:UniProtKB/TrEMBL;Acc:L8H7B1]	0.02391942	0.528215333	0.504295913	2.15E-06
6h	ACA1_042070/clean/scf7180000084573:53936-54010:+	ACA1_042070	Dual specificity protein kinase [Source:UniProtKB/TrEMBL;Acc:L8GW91]	0.123962667	0.62775	0.503787333	2.77E-11
6h	ACA1_235940/clean/scf7180000084762:388892-389005:+	ACA1_235940	hypothetical protein	0.030452833	0.53424	0.503787167	1.02E-06
6h	ACA1_209110/clean/scf7180000084757:272560-272628:+	ACA1_209110	hypothetical protein	0	0.503752333	0.503752333	9.39E-08
6h	ACA1_091840/clean/scf7180000084645:213128-213217:+	ACA1_091840	Ras subfamily protein [Source:UniProtKB/TrEMBL;Acc:L8GIK8]	0.0355004	0.539186333	0.503685933	9.96E-07
6h	ACA1_209790/clean/scf7180000084757:323666-323730:-	ACA1_209790	AMPbinding enzyme domain containing protein [Source:UniProtKB/TrEMBL;Acc:L8GXL7]	0.02	0.523634333	0.503634333	5.90E-07
6h	ACA1_217210/clean/scf7180000084758:541415-541521:-	ACA1_217210	Mitochondrial import receptor subunit tom40, putative [Source:UniProtKB/TrEMBL;Acc:L8GPR5]	0.007597867	0.511192333	0.503594467	1.79E-22
6h	ACA1_175510/clean/scf7180000084741:974008-974097:+	ACA1_175510	Xylose isomerase [Source:UniProtKB/TrEMBL;Acc:L8HKE1]	0.0200256	0.523483667	0.503458067	2.22E-22
6h	ACA1_335940/clean/scf7180000084593:8135-8206:-	ACA1_335940	RNA recognition motif domain containing protein [Source:UniProtKB/TrEMBL;Acc:L8H3Y6]	0.018803867	0.522164333	0.503360467	2.33E-16
6h	ACA1_048090/clean/scf7180000084582:18556-18635:+	ACA1_048090	hypothetical protein	0.011227833	0.514543333	0.5033155	6.03E-08
6h	ACA1_188690/clean/scf7180000084747:192460-192537:-	ACA1_188690	PhytanoylCoA dioxygenase domain containing 1 (Predicted), putative [Source:UniProtKB/TrEMBL;Acc:L8GUY3]	0.055471133	0.558774	0.503302867	6.39E-07
6h	ACA1_322380/clean/scf7180000084463:10543-10619:-	ACA1_322380	hypothetical protein	0.017248697	0.520474333	0.503225637	2.08E-08
6h	ACA1_095270/clean/scf7180000084645:825557-825678:-	ACA1_095270	Thioredoxin-like_fold domain-containing protein [Source:UniProtKB/TrEMBL;Acc:L8GIR9]	0.00200129	0.505005333	0.503004043	1.72E-09
6h	ACA1_363800/clean/scf7180000084641:20540-20630:-	ACA1_363800	ABC transporter domain-containing protein [Source:UniProtKB/TrEMBL;Acc:L8GM21]	0.0521631	0.555095333	0.502932233	3.81E-14
6h	ACA1_031950/clean/scf7180000084523:39720-39829:+	ACA1_031950	CBS domain containing protein [Source:UniProtKB/TrEMBL;Acc:L8H414]	0.096911	0.599815	0.502904	8.34E-09
6h	ACA1_366090/clean/scf7180000084641:345652-345716:-	ACA1_366090	RNA polymerase III subunit C3 [Source:UniProtKB/TrEMBL;Acc:L8GML8]	0	0.502904	0.502904	1.87E-09
6h	ACA1_388680/clean/scf7180000084733:146953-147052:-	ACA1_388680	Frizzled/Smoothened family membrane region protein [Source:UniProtKB/TrEMBL;Acc:L8GEN4]	0.012746627	0.51555	0.502803373	2.26E-07
6h	ACA1_294850/clean/scf7180000084777:1048390-1048471:-	ACA1_294850	hypothetical protein	0.157539	0.660317667	0.502778667	4.09E-05
6h	ACA1_077120/clean/scf7180000084612:240326-240404:-	ACA1_077120	DH domain-containing protein [Source:UniProtKB/TrEMBL;Acc:L8GM77]	0.00081069	0.503587	0.50277631	9.60E-13
6h	ACA1_173060/clean/scf7180000084741:394438-394536:-	ACA1_173060	SNARE associated Golgi protein [Source:UniProtKB/TrEMBL;Acc:L8HGL7]	0	0.502652333	0.502652333	1.78E-08
6h	ACA1_140610/clean/scf7180000084707:102916-102996:-	ACA1_140610	Poly [ADP-ribose] polymerase [Source:UniProtKB/TrEMBL;Acc:L8GGG7]	0	0.502637	0.502637	1.27E-05
6h	ACA1_174040/clean/scf7180000084741:585983-586095:-	ACA1_174040	Protein kinase domain-containing protein [Source:UniProtKB/TrEMBL;Acc:L8HK27]	0.018018033	0.520630667	0.502612633	6.52E-07
6h	ACA1_395500/clean/scf7180000084756:195233-195321:-	ACA1_395500	Rho-GAP domain-containing protein [Source:UniProtKB/TrEMBL;Acc:L8GZP8]	0.003875967	0.506487667	0.5026117	2.57E-09
6h	ACA1_151330/clean/scf7180000084716:106350-106466:-	ACA1_151330	Purple acid phosphatase [Source:UniProtKB/TrEMBL;Acc:L8H0Q3]	0.001851853	0.504255	0.502403147	2.03E-11
6h	ACA1_297430/clean/scf7180000082849:7907-7999:-	ACA1_297430	PH domain containing protein [Source:UniProtKB/TrEMBL;Acc:L8GK59]	0	0.502333667	0.502333667	7.87E-10
6h	ACA1_089040/clean/scf7180000084639:304817-304890:+	ACA1_089040	UBA/TS-N domain containing protein [Source:UniProtKB/TrEMBL;Acc:L8GUR2]	0.005518767	0.50761	0.502091233	1.29E-09
6h	ACA1_069440/clean/scf7180000084603:440160-440291:+	ACA1_069440	PPM-type phosphatase domain-containing protein [Source:UniProtKB/TrEMBL;Acc:L8HDU2]	0.001318803	0.503395333	0.502076531	7.86E-12
6h	ACA1_054710/clean/scf7180000084594:559063-559161:-	ACA1_054710	Ras family protein [Source:UniProtKB/TrEMBL;Acc:L8H6F4]	0.002065177	0.504016333	0.501951157	5.30E-12
6h	ACA1_208120/clean/scf7180000084757:108039-108131:+	ACA1_208120	hypothetical protein	0.003626949	0.505479333	0.501852385	8.54E-32
6h	ACA1_062020/clean/scf7180000084599:1103533-1103653:+	ACA1_062020	Major vault protein alpha, putative [Source:UniProtKB/TrEMBL;Acc:L8GWT3]	0.005727367	0.507081	0.501353633	1.93E-06
6h	ACA1_264550/clean/scf7180000084770:319147-319252:+	ACA1_264550	Cys/Met metabolism PLPdependent enzyme superfamily protein [Source:UniProtKB/TrEMBL;Acc:L8H496]	0.000653967	0.501999	0.501345033	6.14E-13
6h	ACA1_298780/clean/scf7180000082992:23912-23988:+	ACA1_298780	LIM domain containing protein [Source:UniProtKB/TrEMBL;Acc:L8H085]	0.039500633	0.540838333	0.5013377	2.34E-15
6h	ACA1_250180/clean/scf7180000084767:36436-36575:-	ACA1_250180	RhoGEF domain containing protein [Source:UniProtKB/TrEMBL;Acc:L8HC96]	0.034208933	0.535221667	0.501012733	7.55E-11
6h	ACA1_234600/clean/scf7180000084762:303604-303693:+	ACA1_234600	ANK_REP_REGION domain-containing protein [Source:UniProtKB/TrEMBL;Acc:L8H1A6]	0.027010257	0.527937333	0.500927077	1.74E-14
6h	ACA1_337990/clean/scf7180000084604:22182-22278:-	ACA1_337990	DJ-1_PfpI domain-containing protein [Source:UniProtKB/TrEMBL;Acc:L8GCZ7]	0.00275795	0.50343	0.50067205	2.28E-12

6h	ACA1_025980/clean/scf7180000084456:1823-1924:-	ACA1_025980	hypothetical protein	0.002057613	0.502707667	0.500650053	2.35E-09
6h	ACA1_277880/clean/scf7180000084776:118607-118720:+	ACA1_277880	RBR-type E3 ubiquitin transferase [Source:UniProtKB/TrEMBL;Acc:L8H6I3]	0.00445575	0.505061	0.50060525	1.67E-12
6h	ACA1_296280/clean/scf7180000084777:1313917-1314017:+	ACA1_296280	RhoGEF domain containing protein [Source:UniProtKB/TrEMBL;Acc:L8HJZ8]	0.002893709	0.503382	0.500488291	6.29E-09
6h	ACA1_113870/clean/scf7180000084669:392641-392775:+	ACA1_113870	CUB domain containing protein [Source:UniProtKB/TrEMBL;Acc:L8H6F7]	0.009452533	0.509870667	0.500418133	2.23E-11
6h	ACA1_037090/clean/scf7180000084567:56654-56718:+	ACA1_037090	Ankyrin repeat containing protein [Source:UniProtKB/TrEMBL;Acc:L8H0U5]	0.018624333	0.518954333	0.50033	2.78E-08
6h	ACA1_268750/clean/scf7180000084770:911892-912048:+	ACA1_268750	Thioredoxin domain-containing protein [Source:UniProtKB/TrEMBL;Acc:L8H1X3]	0.004576267	0.504898667	0.5003224	7.17E-10
6h	ACA1_365090/clean/scf7180000084641:234844-234924:+	ACA1_365090	ADPribosylglycohydrolase superfamily protein [Source:UniProtKB/TrEMBL;Acc:L8GP97]	0.143423	0.643710667	0.500287667	1.31E-10
6h	ACA1_039450/clean/scf7180000084569:30872-30956:-	ACA1_039450	SAP domain-containing protein [Source:UniProtKB/TrEMBL;Acc:L8HI99]	0.010101	0.510199333	0.500098333	2.29E-07
6h	ACA1_216420/clean/scf7180000084758:497448-497572:-	ACA1_216420	C2 domain-containing protein [Source:UniProtKB/TrEMBL;Acc:L8GQ92]	0.01505442	0.515089667	0.500035247	1.95E-13
6h	ACA1_293710/clean/scf7180000084777:965419-965511:+	ACA1_293710	F-box domain-containing protein [Source:UniProtKB/TrEMBL;Acc:L8HJM5]	0.003874341	0.503809667	0.499935326	6.37E-09
6h	ACA1_264000/clean/scf7180000084770:149917-150017:-	ACA1_264000	Glutamine amidotransferase type-1 domain-containing protein [Source:UniProtKB/TrEMBL;Acc:L8H1W5]	0.030082067	0.529912667	0.4998306	5.42E-12
6h	ACA1_323400/clean/scf7180000084468:17902-17990:-	ACA1_323400	MFS domain-containing protein [Source:UniProtKB/TrEMBL;Acc:L8H3W6]	0.002857627	0.502523667	0.49966604	3.78E-10
6h	ACA1_018950/clean/scf7180000083912:33190-33281:-	ACA1_018950	Lipoprotein, putative [Source:UniProtKB/TrEMBL;Acc:L8HM57]	0.009009	0.508586	0.499577	2.61E-07
6h	ACA1_281250/clean/scf7180000084776:572635-572715:+	ACA1_281250	Zinc finger, zz type domain containing protein [Source:UniProtKB/TrEMBL;Acc:L8H9B9]	0	0.499477	0.499477	0.000836551
6h	ACA1_381520/clean/scf7180000084712:169819-169896:+	ACA1_381520	WH2 domain-containing protein [Source:UniProtKB/TrEMBL;Acc:L8GMY2]	0	0.499353	0.499353	3.49E-11
6h	ACA1_068390/clean/scf7180000084603:186577-186659:-	ACA1_068390	hypothetical protein	0.0146408	0.513982333	0.499341533	0.000410362
6h	ACA1_050370/clean/scf7180000084589:81891-81978:+	ACA1_050370	Fbox domain containing protein [Source:UniProtKB/TrEMBL;Acc:L8H2W7]	0.020702583	0.519980333	0.49927775	1.46E-11
6h	ACA1_081440/clean/scf7180000084618:110361-110456:-	ACA1_081440	Alg14 family oligosaccharide biosynthesis protein [Source:UniProtKB/TrEMBL;Acc:L8HD51]	0.029777233	0.528918333	0.4991411	4.00E-13
6h	ACA1_264360/clean/scf7180000084770:248365-248433:+	ACA1_264360	hypothetical protein	0.002141327	0.501191333	0.499050007	4.43E-08
6h	ACA1_024220/clean/scf7180000084441:21145-21228:+	ACA1_024220	Purinergic receptor, putative [Source:UniProtKB/TrEMBL;Acc:L8GLB7]	0.007072093	0.506108667	0.499036573	4.43E-27
6h	ACA1_290910/clean/scf7180000084777:699306-699418:+	ACA1_290910	ATPase ASNA1 homolog [Source:UniProtKB/TrEMBL;Acc:L8HKV9]	0.002589084	0.501598	0.499008916	4.96E-11
6h	ACA1_060200/clean/scf7180000084599:611251-611369:-	ACA1_060200	hypothetical protein	0.00950592	0.508472667	0.498966747	1.07E-07
6h	ACA1_088790/clean/scf7180000084639:229172-229240:-	ACA1_088790	hypothetical protein	0	0.498902	0.498902	8.60E-05
6h	ACA1_245690/clean/scf7180000084763:521198-521278:-	ACA1_245690	Pribosyltran domain-containing protein [Source:UniProtKB/TrEMBL;Acc:L8GMM1]	0.0468419	0.545688	0.4988461	2.86E-11
6h	ACA1_118750/clean/scf7180000084671:18436-18544:-	ACA1_118750	Threonylcarbamoyl-AMP synthase [Source:UniProtKB/TrEMBL;Acc:L8GLU7]	0.022624433	0.521439333	0.4988149	4.33E-06
6h	ACA1_175520/clean/scf7180000084741:984104-984175:-	ACA1_175520	PH domain-containing protein [Source:UniProtKB/TrEMBL;Acc:L8HI32]	0	0.498769667	0.498769667	1.31E-06
6h	ACA1_346610/clean/scf7180000084658:29753-29845:+	ACA1_346610	DH domain-containing protein [Source:UniProtKB/TrEMBL;Acc:L8GHN5]	0.128625333	0.627270333	0.498645	7.04E-07
6h	ACA1_065270/clean/scf7180000084599:1835406-1835500:+	ACA1_065270	Ubiquitin ligase, putative [Source:UniProtKB/TrEMBL;Acc:L8GYX5]	0.035648733	0.534198	0.498549267	0.002168449
6h	ACA1_064490/clean/scf7180000084599:1628383-1628451:+	ACA1_064490	TYR_PHOSPHATASE_2 domain-containing protein [Source:UniProtKB/TrEMBL;Acc:L8GXK2]	0.0572481	0.555787	0.4985389	2.01E-08
6h	ACA1_376690/clean/scf7180000084685:141469-141621:-	ACA1_376690	Ribosomal_L18e/L15P domain-containing protein [Source:UniProtKB/TrEMBL;Acc:L8HGC7]	0.010290367	0.508815667	0.4985253	3.51E-68
6h	ACA1_054250/clean/scf7180000084594:443992-444080:+	ACA1_054250	G_PROTEIN_RECEP_F2_4 domain-containing protein [Source:UniProtKB/TrEMBL;Acc:L8H5A3]	0.1051502	0.603615667	0.498465467	7.58E-08
6h	ACA1_116760/clean/scf7180000084669:842665-842761:+	ACA1_116760	hypothetical protein	0.03199458	0.530406667	0.498412087	2.06E-12
6h	ACA1_320790/clean/scf7180000084416:22658-22828:-	ACA1_320790	RUN domain-containing protein [Source:UniProtKB/TrEMBL;Acc:L8GTR9]	0.00354931	0.501934667	0.498385357	2.48E-11
6h	ACA1_068080/clean/scf7180000084603:100227-100300:+	ACA1_068080	PlsC domain-containing protein [Source:UniProtKB/TrEMBL;Acc:L8HCG7]	0.006707287	0.504966667	0.49825938	4.07E-22
6h	ACA1_034520/clean/scf7180000084563:62420-62502:+	ACA1_034520	G_PROTEIN_RECEP_F2_4 domain-containing protein [Source:UniProtKB/TrEMBL;Acc:L8HBK6]	0.011329767	0.509511	0.498181233	6.28E-05
6h	ACA1_144350/clean/scf7180000084709:213275-213361:+	ACA1_144350	CN hydrolase domain-containing protein [Source:UniProtKB/TrEMBL;Acc:L8HEV4]	0.0117148	0.509886333	0.498171533	3.84E-20
6h	ACA1_096810/clean/scf7180000084645:1105820-1105924:+	ACA1_096810	IU_nuc_hydro domain-containing protein [Source:UniProtKB/TrEMBL;Acc:L8GJV1]	0.056461333	0.554407667	0.497946333	5.04E-10
6h	ACA1_035580/clean/scf7180000084564:93699-93769:+	ACA1_035580	hypothetical protein	0.00778661	0.505566667	0.497780057	1.24E-23
6h	ACA1_320100/clean/scf7180000084352:7150-7238:+	ACA1_320100	VWFA domain-containing protein [Source:UniProtKB/TrEMBL;Acc:L8H729]	0.01	0.507770333	0.497770333	2.04E-08
6h	ACA1_366460/clean/scf7180000084641:433689-433754:+	ACA1_366460	Dynactin subunit 6, putative [Source:UniProtKB/TrEMBL;Acc:L8GMI4]	0.016312513	0.514065	0.497752487	1.58E-08
6h	ACA1_138930/clean/scf7180000084705:26756-26823:+	ACA1_138930	Protein kinase domain containing protein [Source:UniProtKB/TrEMBL;Acc:L8GQ12]	0.089734633	0.587236	0.497501367	5.58E-10
6h	ACA1_338290/clean/scf7180000084604:52417-52493:-	ACA1_338290	hypothetical protein	0.001508297	0.498886333	0.497378037	5.01E-07
6h	ACA1_369190/clean/scf7180000084664:268491-268582:+	ACA1_369190	PCI domain-containing protein [Source:UniProtKB/TrEMBL;Acc:L8GZ19]	0.001718213	0.499029333	0.49731112	1.87E-11
6h	ACA1_181910/clean/scf7180000084745:71362-71422:-	ACA1_181910	CrcB family protein [Source:UniProtKB/TrEMBL;Acc:L8H804]	0.005952367	0.503256667	0.4973043	6.38E-08
6h	ACA1_155460/clean/scf7180000084722:162605-162681:-	ACA1_155460	unspecified product	0.038126367	0.535356	0.497229633	7.41E-07
6h	ACA1_392690/clean/scf7180000084756:135-207:+	ACA1_392690	PAS domain-containing protein [Source:UniProtKB/TrEMBL;Acc:L8GZJ0]	0.016677833	0.513896667	0.497218833	1.24E-09
6h	ACA1_390060/clean/scf7180000084740:14521-14588:+	ACA1_390060	Carbamoylphosphate synthase L chain, ATP binding domain containing protein [Source:UniProtKB/TrEMBL;Acc:L8GP92]	0.030587053	0.52772	0.497132947	5.21E-10
6h	ACA1_255710/clean/scf7180000084768:547719-547841:+	ACA1_255710	Rho-GAP domain-containing protein [Source:UniProtKB/TrEMBL;Acc:L8HB62]	0.086175767	0.583304333	0.497128567	1.17E-07
6h	ACA1_057960/clean/scf7180000084599:220116-220196:+	ACA1_057960	BTB/POZ domain containing protein [Source:UniProtKB/TrEMBL;Acc:L8GVX1]	0	0.497093667	0.497093667	1.45E-08
6h	ACA1_277590/clean/scf7180000084776:83167-83265:-	ACA1_277590	Peptidase_S66 domain-containing protein [Source:UniProtKB/TrEMBL;Acc:L8H6R2]	0.001875944	0.498919667	0.497043723	1.96E-14
6h	ACA1_171280/clean/scf7180000084741:62616-63006:+	ACA1_171280	Nicotinate phosphoribosyltransferase [Source:UniProtKB/TrEMBL;Acc:L8HHA8]	0.068138267	0.565146333	0.497008067	1.39E-06
6h	ACA1_138310/clean/scf7180000084703:99951-100138:+	ACA1_138310	Rap-GAP domain-containing protein [Source:UniProtKB/TrEMBL;Acc:L8GYP5]	0.00254842	0.499314	0.49676558	2.01E-09
6h	ACA1_161750/clean/scf7180000084726:20030-20183:-	ACA1_161750	hypothetical protein	0.004155687	0.500889	0.496733313	3.14E-10
6h	ACA1_350480/clean/scf7180000084692:13014-13109:+	ACA1_350480	ABC2_membrane domain-containing protein [Source:UniProtKB/TrEMBL;Acc:L8HLS4]	0.034860433	0.531573667	0.496713233	1.20E-07
6h	ACA1_305680/clean/scf7180000083609:29380-29471:+	ACA1_305680	hypothetical protein	0.014709057	0.511419333	0.496710277	7.58E-33
6h	ACA1_326190/clean/scf7180000084504:49979-50064:+	ACA1_326190	F-box domain-containing protein [Source:UniProtKB/TrEMBL;Acc:L8HH77]	0	0.496579667	0.496579667	7.02E-05
6h	ACA1_352400/clean/scf7180000084704:9409-9548:-	ACA1_352400	Fn3_like domain-containing protein [Source:UniProtKB/TrEMBL;Acc:L8GTX5]	0.0215111	0.517964667	0.496453567	8.31E-21
6h	ACA1_251790/clean/scf7180000084768:119067-119139:-	ACA1_251790	Cellulase (Glycosyl hydrolase family 5) subfamily protein [Source:UniProtKB/TrEMBL;Acc:L8HAR2]	0.037018883	0.533463333	0.49644445	1.28E-12
6h	ACA1_189390/clean/scf7180000084748:23617-23691:-	ACA1_189390	hypothetical protein	0.0488516	0.545218667	0.496367067	3.39E-06
6h	ACA1_278320/clean/scf7180000084776:196179-196318:+	ACA1_278320	N-acetyltransferase domain-containing protein [Source:UniProtKB/TrEMBL;Acc:L8H8U2]	0.05305558	0.549266667	0.496211087	3.72E-07
6h	ACA1_130560/clean/scf7180000084698:108441-108510:+	ACA1_130560	Glucosyl hydrolase family protein [Source:UniProtKB/TrEMBL;Acc:L8GRU3]	0.00138874	0.497582	0.49619326	3.22E-11
6h	ACA1_279560/clean/scf7180000084776:415307-415370:-	ACA1_279560	Iron hydrogenase assembly protein [Source:UniProtKB/TrEMBL;Acc:L8H633]	0.150412667	0.64623	0.495817333	9.93E-09
6h	ACA1_278860/clean/scf7180000084776:259282-259393:-	ACA1_278860	Glycosylphosphatidylinositol anchor attachment 1 protein [Source:UniProtKB/TrEMBL;Acc:L8H6Z3]	0	0.495710667	0.495710667	3.16E-09
6h	ACA1_145850/clean/scf7180000084710:168067-168142:-	ACA1_145850	O-methyltransferase, putative [Source:UniProtKB/TrEMBL;Acc:L8GFG9]	0.008694407	0.504326333	0.495631927	3.60E-12
6h	ACA1_385390/clean/scf7180000084730:162929-163029:+	ACA1_385390	Ubiquitinconjugating enzyme subfamily protein [Source:UniProtKB/TrEMBL;Acc:L8HAL6]	0.015784313	0.511384	0.495599687	2.55E-05
6h	ACA1_372060/clean/scf7180000084664:624148-624217:-	ACA1_372060	Aldo_ket_red domain-containing protein [Source:UniProtKB/TrEMBL;Acc:L8GYH3]	0.01038811	0.50597	0.49558189	2.64E-05
6h	ACA1_179960/clean/scf7180000084744:9775-9950:-	ACA1_179960	Protein kinase domain/MORN repeatcontaining protein [Source:UniProtKB/TrEMBL;Acc:L8GC88]	0.002763407	0.498313333	0.495549927	2.56E-11
6h	ACA1_266230/clean/scf7180000084770:638678-638768:+	ACA1_266230	Dilute domain-containing protein [Source:UniProtKB/TrEMBL;Acc:L8H2D0]	0	0.495528667	0.495528667	2.66E-05
6h	ACA1_298630/clean/scf7180000082992:2946-3045:-	ACA1_298630	Vacuolar protein sorting 16, putative [Source:UniProtKB/TrEMBL;Acc:L8H081]	0.0018775	0.497366	0.4954885	1.96E-09
6h	ACA1_380760/clean/scf7180000084712:83365-83483:+	ACA1_380760	Aminotran_5 domain-containing protein [Source:UniProtKB/TrEMBL;Acc:L8GNK4]	0.014394053	0.50986	0.495465947	5.65E-14
6h	ACA1_251790/clean/scf7180000084768:119786-119868:-	ACA1_251790	Cellulase (Glycosyl hydrolase family 5) subfamily protein [Source:UniProtKB/TrEMBL;Acc:L8HAR2]	0.009041223	0.504316	0.495274777	6.22E-14
6h	ACA1_359700/clean/scf7180000084622:5468-5539:-	ACA1_359700	Thioredoxin domain-containing protein [Source:UniProtKB/TrEMBL;Acc:L8HCQ3]	0.006544947	0.501588667	0.49504372	1.37E-32
6h	ACA1_070980/clean/scf7180000084603:821233-821347:-	ACA1_070980	hypothetical protein	0.019133533	0.513932	0.494798467	1.89E-15
6h	ACA1_400300/clean/scf7180000084775:142648-142729:+	ACA1_400300	UBIQUITIN_CONJUGAT_2 domain-containing protein [Source:UniProtKB/TrEMBL;Acc:L8GGI3]	0	0.494792	0.494792	2.74E-08
6h	ACA1_064430/clean/scf7180000084599:1618145-1618232:+	ACA1_064430	hypothetical protein	0.00183952	0.496575667	0.494736147	5.78E-10
6h	ACA1_400220/clean/scf7180000084775:121956-122032:-	ACA1_400220	Abhydrolase_2 domain-containing protein [Source:UniProtKB/TrEMBL;Acc:L8GG24]	0.031589567	0.526281	0.494691433	4.60E-13
6h	ACA1_351920/clean/scf7180000084695:35007-35076:+	ACA1_351920	VWFA domain-containing protein [Source:UniProtKB/TrEMBL;Acc:L8GE58]	0.0568873	0.551465	0.4945777	4.42E-09
6h	ACA1_119010/clean/scf7180000084672:10792-10870:+	ACA1_119010	Ras-GEF domain-containing protein [Source:UniProtKB/TrEMBL;Acc:L8HJ80]	0.1210583	0.615598	0.4945397	8.57E-06
6h	ACA1_172940/clean/scf7180000084741:367963-368045:+	ACA1_172940	Protein kinase domain-containing protein [Source:UniProtKB/TrEMBL;Acc:L8HHJ2]	0.047619	0.542121	0.494502	0.002404038
6h	ACA1_230440/clean/scf7180000084761:348679-348785:+	ACA1_230440	WD_REPEATS_REGION domain-containing protein [Source:UniProtKB/TrEMBL;Acc:L8H839]	0	0.494463333	0.494463333	1.38E-07
6h	ACA1_387360/clean/scf7180000084733:52962-53050:-	ACA1_387360	hypothetical protein	0.009087653	0.503506333	0.49441868	9.20E-08
6h	ACA1_368950/clean/scf7180000084664:203224-203297:-	ACA1_368950	Methylglyoxal synthase [Source:UniProtKB/TrEMBL;Acc:L8H078]	0.023818153	0.518128	0.494309847	7.61E-08
6h	ACA1_102790/clean/scf7180000084652:28296-28369:-	ACA1_102790	hypothetical protein	0.002073613	0.496297667	0.494224053	0.0001335
6h	ACA1_399930/clean/scf7180000084775:68133-68216:+	ACA1_399930	Ras subfamily protein [Source:UniProtKB/TrEMBL;Acc:L8GGR5]	0.005927367	0.499896	0.493968633	1.78E-09
6h	ACA1_389450/clean/scf7180000084733:259021-259117:+	ACA1_389450	hypothetical protein	0.018713467	0.512659	0.493945533	1.76E-20
6h	ACA1_060920/clean/scf7180000084599:780517-780569:-	ACA1_060920	MFS domain-containing protein [Source:UniProtKB/TrEMBL;Acc:L8GVT5]	0.1422562	0.636152667	0.493896467	2.25E-09
6h	ACA1_063390/clean/scf7180000084599:1399113-1399332:+	ACA1_063390	Ribosomal_L7Ae domain-containing protein [Source:UniProtKB/TrEMBL;Acc:L8GX53]	0.022086633	0.515975333	0.4938887	1.05E-52
6h	ACA1_271290/clean/scf7180000084771:12075-12196:-	ACA1_271290	hypothetical protein	0.00639255	0.500133333	0.493740783	1.15E-19
6h	ACA1_107530/clean/scf7180000084660:215648-215770:-	ACA1_107530	UBP-type domain-containing protein [Source:UniProtKB/TrEMBL;Acc:L8GMW8]	0.028394567	0.521976667	0.4935821	3.58E-21
6h	ACA1_071520/clean/scf7180000084603:977304-977374:+	ACA1_071520	Piwi domain containing protein [Source:UniProtKB/TrEMBL;Acc:L8HEC1]	0.00494489	0.498511	0.49356611	1.03E-15
6h	ACA1_333170/clean/scf7180000084581:9655-9714:-	ACA1_333170	Ras-GEF domain-containing protein [Source:UniProtKB/TrEMBL;Acc:L8HGS6]	0	0.493075	0.493075	7.53E-05
6h	ACA1_152950/clean/scf7180000084720:40493-40555:-	ACA1_152950	UBIQUITIN_CONJUGAT_2 domain-containing protein [Source:UniProtKB/TrEMBL;Acc:L8HI96]	0.0155581	0.508429333	0.492871233	2.05E-43
6h	ACA1_309650/clean/scf7180000083763:8949-9015:+	ACA1_309650	hypothetical protein	0	0.492745	0.492745	0.017420269
6h	ACA1_109140/clean/scf7180000084661:130568-130668:+	ACA1_109140	F-box domain-containing protein [Source:UniProtKB/TrEMBL;Acc:L8GD84]	0.0045662	0.497239333	0.492673133	1.50E-08
6h	ACA1_163660/clean/scf7180000084729:65575-65663:-	ACA1_163660	hypothetical protein	0.008407337	0.500891333	0.492483997	1.95E-19
6h	ACA1_112100/clean/scf7180000084669:111897-111958:+	ACA1_112100	BTB/POZ domain containing protein [Source:UniProtKB/TrEMBL;Acc:L8H3Y0]	0.047512133	0.539932	0.492419867	1.27E-08
6h	ACA1_143650/clean/scf7180000084709:58895-59033:+	ACA1_143650	Nudix hydrolase domain-containing protein [Source:UniProtKB/TrEMBL;Acc:L8HFQ7]	0	0.492147333	0.492147333	1.39E-06
6h	ACA1_151240/clean/scf7180000084716:78904-79098:-	ACA1_151240	OTU domain-containing protein [Source:UniProtKB/TrEMBL;Acc:L8H309]	0.005221443	0.497325333	0.49210389	1.32E-09
6h	ACA1_035640/clean/scf7180000084564:102936-103008:-	ACA1_035640	Beta-lactamase domain-containing protein [Source:UniProtKB/TrEMBL;Acc:L8H9P2]	0	0.491937	0.491937	0.007545856
6h	ACA1_232860/clean/scf7180000084762:88446-88580:+	ACA1_232860	hypothetical protein	0.0608728	0.552771333	0.491898533	8.69E-08
6h	ACA1_198480/clean/scf7180000084753:125119-125235:+	ACA1_198480	Transcription factor III family protein [Source:UniProtKB/TrEMBL;Acc:L8H324]	0.068562533	0.560426667	0.491864133	2.65E-10
6h	ACA1_397180/clean/scf7180000084759:165084-165169:-	ACA1_397180	hypothetical protein	0.005294667	0.497157	0.491862333	3.35E-07
6h	ACA1_068230/clean/scf7180000084603:141492-141580:-	ACA1_068230	DAGKc domain-containing protein [Source:UniProtKB/TrEMBL;Acc:L8HCH8]	0.010442813	0.502177333	0.49173452	1.29E-08
6h	ACA1_064810/clean/scf7180000084599:1704388-1704469:-	ACA1_064810	Transporter, major facilitator subfamily protein [Source:UniProtKB/TrEMBL;Acc:L8GWU0]	0	0.491612667	0.491612667	6.65E-09
6h	ACA1_057940/clean/scf7180000084599:214671-214751:+	ACA1_057940	Alpha-mannosidase [Source:UniProtKB/TrEMBL;Acc:L8GX61]	0.001919612	0.493524333	0.491604722	3.80E-12
6h	ACA1_279420/clean/scf7180000084776:381866-381972:+	ACA1_279420	BAR domain containing protein [Source:UniProtKB/TrEMBL;Acc:L8H745]	0.007528083	0.499124667	0.491596584	2.39E-17
6h	ACA1_054330/clean/scf7180000084594:469621-469674:-	ACA1_054330	Hbxip, putative [Source:UniProtKB/TrEMBL;Acc:L8H639]	0.011682263	0.503268	0.491585737	1.83E-22
6h	ACA1_202770/clean/scf7180000084754:106110-106195:-	ACA1_202770	TPR_REGION domain-containing protein [Source:UniProtKB/TrEMBL;Acc:L8GSW6]	0.063632167	0.555171	0.491538833	7.54E-12
6h	ACA1_230470/clean/scf7180000084761:356650-356728:+	ACA1_230470	hypothetical protein	0	0.491530333	0.491530333	4.31E-06
6h	ACA1_055410/clean/scf7180000084594:698739-698859:+	ACA1_055410	Protein kinase domain-containing protein [Source:UniProtKB/TrEMBL;Acc:L8H6J6]	0.00409857	0.495576	0.49147743	2.81E-10
6h	ACA1_220700/clean/scf7180000084758:947835-947909:+	ACA1_220700	3-oxoacyl-[acyl-carrier-protein] synthase [Source:UniProtKB/TrEMBL;Acc:L8GQ49]	0	0.491456667	0.491456667	1.97E-08
6h	ACA1_367290/clean/scf7180000084641:591222-591322:-	ACA1_367290	hypothetical protein	0.001206299	0.492548333	0.491342035	8.90E-11
6h	ACA1_116990/clean/scf7180000084669:885639-885731:+	ACA1_116990	Carboxypeptidase [Source:UniProtKB/TrEMBL;Acc:L8H6Y6]	0.00054984	0.491675333	0.491125493	4.90E-13
6h	ACA1_063800/clean/scf7180000084599:1507318-1507419:-	ACA1_063800	B30.2/SPRY domain-containing protein [Source:UniProtKB/TrEMBL;Acc:L8GZV1]	0.002306807	0.493419	0.491112193	1.46E-09
6h	ACA1_209860/clean/scf7180000084757:339630-339718:-	ACA1_209860	Protein kinase domain-containing protein [Source:UniProtKB/TrEMBL;Acc:L8GYD8]	0.0102564	0.501164	0.4909076	2.17E-11
6h	ACA1_367360/clean/scf7180000084641:613564-613660:-	ACA1_367360	TPR_REGION domain-containing protein [Source:UniProtKB/TrEMBL;Acc:L8GMB3]	0.000663953	0.491534667	0.490870713	9.00E-13
6h	ACA1_254920/clean/scf7180000084768:447370-447468:+	ACA1_254920	von Willebrand factor type A domain containing protein [Source:UniProtKB/TrEMBL;Acc:L8HA47]	0.00846518	0.499242	0.49077682	1.02E-14
6h	ACA1_266230/clean/scf7180000084770:637429-637519:+	ACA1_266230	Dilute domain-containing protein [Source:UniProtKB/TrEMBL;Acc:L8H2D0]	0.0051282	0.495697333	0.490569133	2.33E-05
6h	ACA1_037070/clean/scf7180000084567:47522-47626:-	ACA1_037070	DLH domain-containing protein [Source:UniProtKB/TrEMBL;Acc:L8H284]	0.002231387	0.492767333	0.490535947	2.02E-11
6h	ACA1_396240/clean/scf7180000084759:61669-61776:+	ACA1_396240	Transcription factor e2f/dimerization partner (Tdp) domain containing protein [Source:UniProtKB/TrEMBL;Acc:L8HDJ6]	0.032610633	0.523116	0.490505367	1.35E-09
6h	ACA1_048870/clean/scf7180000084583:6274-6347:-	ACA1_048870	hypothetical protein	0.0258753	0.516356333	0.490481033	1.70E-14
6h	ACA1_178260/clean/scf7180000084743:193114-193265:+	ACA1_178260	Cytochrome P450, putative [Source:UniProtKB/TrEMBL;Acc:L8GTE4]	0.030861057	0.521305333	0.490444277	3.12E-16
6h	ACA1_069380/clean/scf7180000084603:424176-424254:+	ACA1_069380	LNS2 domain-containing protein [Source:UniProtKB/TrEMBL;Acc:L8HCT1]	0.004498433	0.494939	0.490440567	1.45E-07
6h	ACA1_360060/clean/scf7180000084622:40272-40346:-	ACA1_360060	GHMP_kinases_C domain-containing protein [Source:UniProtKB/TrEMBL;Acc:L8HF07]	0.009299043	0.499733667	0.490434623	1.14E-11
6h	ACA1_039450/clean/scf7180000084569:31058-31129:-	ACA1_039450	SAP domain-containing protein [Source:UniProtKB/TrEMBL;Acc:L8HI99]	0	0.490432	0.490432	9.26E-09
6h	ACA1_282190/clean/scf7180000084776:623109-623222:-	ACA1_282190	Synapsin, ATP binding domain containing protein [Source:UniProtKB/TrEMBL;Acc:L8H7E1]	0	0.490428333	0.490428333	5.08E-12
6h	ACA1_252560/clean/scf7180000084768:181722-181799:+	ACA1_252560	hypothetical protein	0.021018643	0.511388333	0.49036969	8.35E-07
6h	ACA1_096590/clean/scf7180000084645:1054694-1054778:-	ACA1_096590	Usp domain-containing protein [Source:UniProtKB/TrEMBL;Acc:L8GJ01]	0.1526991	0.643044333	0.490345233	0.000115684
6h	ACA1_383070/clean/scf7180000084721:135091-135159:+	ACA1_383070	CBS domain containing protein [Source:UniProtKB/TrEMBL;Acc:L8GXI5]	0.034415	0.524692	0.490277	4.12E-09
6h	ACA1_265300/clean/scf7180000084770:429774-429849:+	ACA1_265300	Cog4 domain-containing protein [Source:UniProtKB/TrEMBL;Acc:L8H4D2]	0	0.490148667	0.490148667	3.85E-08
6h	ACA1_072740/clean/scf7180000084603:1282481-1282595:-	ACA1_072740	BTB domain-containing protein [Source:UniProtKB/TrEMBL;Acc:L8HGU7]	0.012722633	0.502815667	0.490093033	4.25E-09
6h	ACA1_183970/clean/scf7180000084745:533357-533440:-	ACA1_183970	hypothetical protein	0	0.489871333	0.489871333	0.000207479
6h	ACA1_174040/clean/scf7180000084741:585590-585728:-	ACA1_174040	Protein kinase domain-containing protein [Source:UniProtKB/TrEMBL;Acc:L8HK27]	0.00186016	0.491601	0.48974084	2.88E-10
6h	ACA1_189820/clean/scf7180000084748:96180-96262:-	ACA1_189820	Protein bcp1, putative [Source:UniProtKB/TrEMBL;Acc:L8GGI0]	0.00330033	0.492984667	0.489684337	5.04E-09
6h	ACA1_063800/clean/scf7180000084599:1506142-1506202:-	ACA1_063800	B30.2/SPRY domain-containing protein [Source:UniProtKB/TrEMBL;Acc:L8GZV1]	0.027416	0.517048333	0.489632333	9.91E-12
6h	ACA1_064570/clean/scf7180000084599:1645492-1645560:+	ACA1_064570	Ras subfamily protein [Source:UniProtKB/TrEMBL;Acc:L8GXF9]	0.004471973	0.493843	0.489371027	5.80E-10
6h	ACA1_181590/clean/scf7180000084745:22859-22933:+	ACA1_181590	AcylCoenzyme A dehydrogenase family, member 10 isoform 7, putative [Source:UniProtKB/TrEMBL;Acc:L8H7Y6]	0	0.489195667	0.489195667	2.28E-10
6h	ACA1_043650/clean/scf7180000084573:193636-193766:-	ACA1_043650	Poly(Adpribose) glycohydrolase [Source:UniProtKB/TrEMBL;Acc:L8GUS0]	0.007278033	0.496414333	0.4891363	7.46E-06

6h	ACA1_069070/clean/scf7180000084603:348205-348267:-	ACA1_069070	Animal heme peroxidase family protein [Source:UniProtKB/TrEMBL;Acc:L8HEX5]	0.039500533	0.528502	0.489001467	4.75E-05
6h	ACA1_182880/clean/scf7180000084745:276011-276107:-	ACA1_182880	E3 ubiquitin-protein ligase [Source:UniProtKB/TrEMBL;Acc:L8H743]	0.015660247	0.504451333	0.488791087	1.16E-07
6h	ACA1_138060/clean/scf7180000084703:83840-83979:-	ACA1_138060	hypothetical protein	0.018217956	0.506920333	0.488702377	1.49E-09
6h	ACA1_189640/clean/scf7180000084748:70191-70262:-	ACA1_189640	Bifunctional aspartate kinase/diaminopimelate decarboxylase protein [Source:UniProtKB/TrEMBL;Acc:L8GEY3]	0.000854973	0.489475667	0.488620693	1.77E-11
6h	ACA1_203620/clean/scf7180000084754:172093-172181:+	ACA1_203620	Cullin domain-containing protein [Source:UniProtKB/TrEMBL;Acc:L8GUK9]	0.0476463	0.536258333	0.488612033	8.22E-08
6h	ACA1_397180/clean/scf7180000084759:165490-165573:-	ACA1_397180	hypothetical protein	0	0.488548333	0.488548333	9.61E-09
6h	ACA1_031600/clean/scf7180000084522:21493-21557:-	ACA1_031600	Leucine rich repeat-containing protein [Source:UniProtKB/TrEMBL;Acc:L8GSM8]	0.0043516	0.492691	0.4883394	2.02E-07
6h	ACA1_204550/clean/scf7180000084754:310011-310261:+	ACA1_204550	VPS9 domain-containing protein [Source:UniProtKB/TrEMBL;Acc:L8GTV1]	0.003210957	0.491460667	0.48824971	2.67E-09
6h	ACA1_320840/clean/scf7180000084416:33068-33130:+	ACA1_320840	HSP91 (Heat shock protein 91), putative [Source:UniProtKB/TrEMBL;Acc:L8GTS5]	0.001481239	0.489725	0.488243761	9.96E-35
6h	ACA1_347660/clean/scf7180000084667:6260-6317:+	ACA1_347660	Fructose-2,6-bisphosphate 2-phosphatase [Source:UniProtKB/TrEMBL;Acc:L8GM28]	0.003994297	0.492169333	0.488175037	5.48E-10
6h	ACA1_220510/clean/scf7180000084758:910862-910973:+	ACA1_220510	WD_REPEATS_REGION domain-containing protein [Source:UniProtKB/TrEMBL;Acc:L8GQV1]	0.004649	0.492286333	0.487637333	3.19E-08
6h	ACA1_315780/clean/scf7180000083943:7650-7760:+	ACA1_315780	hypothetical protein	0.012532283	0.500030333	0.48749805	1.70E-09
6h	ACA1_290190/clean/scf7180000084777:548007-548067:+	ACA1_290190	SCF ubiquitin ligase complex protein SKP1a, putative [Source:UniProtKB/TrEMBL;Acc:L8HJ88]	0.037751667	0.525165667	0.487414	4.46E-13
6h	ACA1_110930/clean/scf7180000084666:16863-16947:-	ACA1_110930	Nudix hydrolase domain-containing protein [Source:UniProtKB/TrEMBL;Acc:L8H9Z8]	0.002466853	0.489878	0.487411147	1.83E-08
6h	ACA1_236660/clean/scf7180000084762:470425-470488:-	ACA1_236660	Fbox domain containing protein [Source:UniProtKB/TrEMBL;Acc:L8H304]	0.29082	0.778134667	0.487314667	4.49E-08
6h	ACA1_002610/clean/scf7180000082935:2862-2923:+	ACA1_002610	hypothetical protein	0.037479933	0.524765333	0.4872854	1.13E-16
6h	ACA1_129350/clean/scf7180000084698:9343-9407:+	ACA1_129350	Fatty acid hydroxylase domain-containing protein [Source:UniProtKB/TrEMBL;Acc:L8GPA5]	0.001799513	0.489083667	0.487284153	1.01E-10
6h	ACA1_182840/clean/scf7180000084745:262855-263104:-	ACA1_182840	MATE efflux family subfamily protein [Source:UniProtKB/TrEMBL;Acc:L8H859]	0.00595673	0.493210333	0.487253603	1.96E-09
6h	ACA1_276500/clean/scf7180000084774:194235-194312:+	ACA1_276500	hypothetical protein	0.019523033	0.506646667	0.487123633	8.67E-08
6h	ACA1_286170/clean/scf7180000084776:1051777-1051856:+	ACA1_286170	Peptidase_S9 domain-containing protein [Source:UniProtKB/TrEMBL;Acc:L8H7U8]	0	0.487110333	0.487110333	6.40E-11
6h	ACA1_252770/clean/scf7180000084768:205342-205434:-	ACA1_252770	Peripla_BP_6 domain-containing protein [Source:UniProtKB/TrEMBL;Acc:L8HAP5]	0.013311233	0.500255333	0.4869441	2.31E-09
6h	ACA1_022330/clean/scf7180000084293:9476-9582:+	ACA1_022330	Carbamoylphosphate synthase L chain, ATP-binding, putative [Source:UniProtKB/TrEMBL;Acc:L8GNZ7]	0.001098464	0.487789667	0.486691203	4.29E-16
6h	ACA1_198360/clean/scf7180000084753:89805-89893:-	ACA1_198360	Epithiospecifier protein [Source:UniProtKB/TrEMBL;Acc:L8H5A7]	0.026444593	0.512920333	0.48647574	8.58E-10
6h	ACA1_098880/clean/scf7180000084646:52313-52437:-	ACA1_098880	UDENN domain-containing protein [Source:UniProtKB/TrEMBL;Acc:L8H7R0]	0.012228817	0.498666	0.486437183	4.97E-10
6h	ACA1_183960/clean/scf7180000084745:528043-528144:-	ACA1_183960	Phosphoesterase family protein [Source:UniProtKB/TrEMBL;Acc:L8H8F6]	0.017882167	0.504194	0.486311833	1.71E-15
6h	ACA1_167020/clean/scf7180000084736:56119-56216:+	ACA1_167020	EF-hand domain-containing protein [Source:UniProtKB/TrEMBL;Acc:L8H0U0]	0.016914353	0.503204667	0.486290313	1.20E-14
6h	ACA1_092640/clean/scf7180000084645:373459-373558:-	ACA1_092640	Amidase domain-containing protein [Source:UniProtKB/TrEMBL;Acc:L8GKL1]	0.003643737	0.489827333	0.486183597	2.59E-10
6h	ACA1_193170/clean/scf7180000084749:204307-204453:-	ACA1_193170	JmjC domain-containing protein [Source:UniProtKB/TrEMBL;Acc:L8GNQ7]	0.013406473	0.499250667	0.485844193	2.37E-12
6h	ACA1_228930/clean/scf7180000084761:227992-228096:-	ACA1_228930	Vta1 domain-containing protein [Source:UniProtKB/TrEMBL;Acc:L8H8T2]	0	0.485837333	0.485837333	1.42E-15
6h	ACA1_264230/clean/scf7180000084770:215636-215713:+	ACA1_264230	ATP/GTPbinding-like protein [Source:UniProtKB/TrEMBL;Acc:L8H212]	0	0.485781667	0.485781667	6.96E-10
6h	ACA1_221030/clean/scf7180000084758:982040-982107:-	ACA1_221030	DUF1992 domain-containing protein [Source:UniProtKB/TrEMBL;Acc:L8GQX4]	0.039967567	0.525559333	0.485591767	1.88E-07
6h	ACA1_172920/clean/scf7180000084741:360953-361041:+	ACA1_172920	hypothetical protein	0	0.485479667	0.485479667	7.98E-07
6h	ACA1_279570/clean/scf7180000084776:417259-417335:+	ACA1_279570	Elp3 domain-containing protein [Source:UniProtKB/TrEMBL;Acc:L8H759]	0.168208733	0.653538667	0.485329933	4.46E-08
6h	ACA1_192970/clean/scf7180000084749:173824-173907:-	ACA1_192970	Basic helixloop-helix (BHLH) family protein [Source:UniProtKB/TrEMBL;Acc:L8GNQ5]	0.093366	0.578508667	0.485142667	2.12E-05
6h	ACA1_182370/clean/scf7180000084745:198115-198265:-	ACA1_182370	ANK_REP_REGION domain-containing protein [Source:UniProtKB/TrEMBL;Acc:L8H846]	0.024437167	0.509481333	0.485044167	5.35E-22
6h	ACA1_277480/clean/scf7180000084776:61383-61451:-	ACA1_277480	DegPtype protease [Source:UniProtKB/TrEMBL;Acc:L8H5P1]	0.1097669	0.594540667	0.484773767	1.06E-05
6h	ACA1_343180/clean/scf7180000084638:12545-12625:-	ACA1_343180	RPOLD domain-containing protein [Source:UniProtKB/TrEMBL;Acc:L8H8M4]	0.020041343	0.504811333	0.48476999	7.77E-11
6h	ACA1_053660/clean/scf7180000084594:310694-310809:-	ACA1_053660	TNF receptor-associated protein 1, putative [Source:UniProtKB/TrEMBL;Acc:L8H601]	0.00049343	0.485101667	0.484608237	4.72E-14
6h	ACA1_068390/clean/scf7180000084603:186759-186845:-	ACA1_068390	hypothetical protein	0	0.484567667	0.484567667	4.08E-06
6h	ACA1_289750/clean/scf7180000084777:456537-456620:+	ACA1_289750	Antioxidant, AhpC/TSA superfamily protein [Source:UniProtKB/TrEMBL;Acc:L8HJ25]	0.078157767	0.562670667	0.4845129	8.95E-10
6h	ACA1_068620/clean/scf7180000084603:241992-242158:-	ACA1_068620	hypothetical protein	0.012759957	0.497264	0.484504043	3.75E-07
6h	ACA1_279570/clean/scf7180000084776:416486-416547:+	ACA1_279570	Elp3 domain-containing protein [Source:UniProtKB/TrEMBL;Acc:L8H759]	0.167636167	0.651995	0.484358833	2.73E-07
6h	ACA1_329700/clean/scf7180000084562:53182-53258:+	ACA1_329700	Electron transfer flavoprotein subunit alpha [Source:UniProtKB/TrEMBL;Acc:L8GI91]	0.00264451	0.486933333	0.484288823	4.35E-13
6h	ACA1_156030/clean/scf7180000084723:5781-5867:+	ACA1_156030	hypothetical protein	0	0.484239667	0.484239667	1.61E-08
6h	ACA1_281330/clean/scf7180000084776:590958-591045:-	ACA1_281330	Protein kinase domain containing protein [Source:UniProtKB/TrEMBL;Acc:L8H6A8]	0	0.484056667	0.484056667	2.95E-08
6h	ACA1_238630/clean/scf7180000084763:109469-109558:-	ACA1_238630	Phosphogluconate dehydrogenase (Decarboxylating), NAD binding domain containing protein [Source:UniProtKB/TrEMBL;Acc:L8GKQ5]	0.093786733	0.577834333	0.4840476	0.001025018
6h	ACA1_396130/clean/scf7180000084759:25092-25192:-	ACA1_396130	Prefoldin, alpha subunit [Source:UniProtKB/TrEMBL;Acc:L8HDI9]	0.028762333	0.512756	0.483993667	1.10E-24
6h	ACA1_095220/clean/scf7180000084645:816295-816399:-	ACA1_095220	Protein kinase domain-containing protein [Source:UniProtKB/TrEMBL;Acc:L8GIR4]	0.00015507	0.484121667	0.483966597	1.18E-11
6h	ACA1_054230/clean/scf7180000084594:437846-437925:+	ACA1_054230	CDPalcohol phosphatidyltransferase superfamily protein [Source:UniProtKB/TrEMBL;Acc:L8H630]	0.00745064	0.491406667	0.483956027	1.81E-14
6h	ACA1_176170/clean/scf7180000084741:1139229-1139311:+	ACA1_176170	Bin3-type SAM domain-containing protein [Source:UniProtKB/TrEMBL;Acc:L8HH82]	0.00527459	0.489202333	0.483927743	2.72E-07
6h	ACA1_088710/clean/scf7180000084639:210149-210225:+	ACA1_088710	hypothetical protein	0.00636817	0.490145333	0.483777163	5.06E-08
6h	ACA1_171670/clean/scf7180000084741:139744-139818:+	ACA1_171670	DAO domain-containing protein [Source:UniProtKB/TrEMBL;Acc:L8HIU0]	0	0.483749667	0.483749667	4.43E-10
6h	ACA1_324040/clean/scf7180000084470:12975-13048:-	ACA1_324040	Carrier superfamily protein [Source:UniProtKB/TrEMBL;Acc:L8HE29]	0.008385733	0.492104333	0.4837186	1.05E-13
6h	ACA1_295600/clean/scf7180000084777:1181563-1181652:-	ACA1_295600	6-phosphogluconolactonase [Source:UniProtKB/TrEMBL;Acc:L8HIP4]	0.01341672	0.496997333	0.483580613	4.48E-11
6h	ACA1_064810/clean/scf7180000084599:1704086-1704147:-	ACA1_064810	Transporter, major facilitator subfamily protein [Source:UniProtKB/TrEMBL;Acc:L8GWU0]	0.021225067	0.504748667	0.4835236	3.88E-08
6h	ACA1_388760/clean/scf7180000084733:160383-160457:+	ACA1_388760	hypothetical protein	0.000542593	0.484050333	0.48350774	1.10E-10
6h	ACA1_062850/clean/scf7180000084599:1287408-1287498:+	ACA1_062850	WD_REPEATS_REGION domain-containing protein [Source:UniProtKB/TrEMBL;Acc:L8GZK3]	0.013755067	0.497119	0.483363933	1.82E-12
6h	ACA1_173790/clean/scf7180000084741:519853-519992:+	ACA1_173790	Nicotinamide-nucleotide adenylyltransferase [Source:UniProtKB/TrEMBL;Acc:L8HK06]	0.00496314	0.488318667	0.483355527	4.96E-10
6h	ACA1_024220/clean/scf7180000084441:21653-21736:+	ACA1_024220	Purinergic receptor, putative [Source:UniProtKB/TrEMBL;Acc:L8GLB7]	0.010425457	0.493758333	0.483332877	1.35E-26
6h	ACA1_045090/clean/scf7180000084576:77398-77513:-	ACA1_045090	F-box domain-containing protein [Source:UniProtKB/TrEMBL;Acc:L8GYZ3]	0.023561833	0.506846333	0.4832845	6.96E-10
6h	ACA1_095560/clean/scf7180000084645:848678-848775:+	ACA1_095560	Protein pelota homolog [Source:UniProtKB/TrEMBL;Acc:L8GL32]	0.025438767	0.508617	0.483178233	2.27E-07
6h	ACA1_230440/clean/scf7180000084761:349253-349327:+	ACA1_230440	WD_REPEATS_REGION domain-containing protein [Source:UniProtKB/TrEMBL;Acc:L8H839]	0	0.483162333	0.483162333	4.08E-08
6h	ACA1_266230/clean/scf7180000084770:637129-637290:+	ACA1_266230	Dilute domain-containing protein [Source:UniProtKB/TrEMBL;Acc:L8H2D0]	0.0318272	0.514901667	0.483074467	0.000119683
6h	ACA1_133350/clean/scf7180000084701:37041-37138:-	ACA1_133350	PlsC domain-containing protein [Source:UniProtKB/TrEMBL;Acc:L8H3L0]	0.004086403	0.486981	0.482894597	3.75E-09
6h	ACA1_287760/clean/scf7180000084777:72896-72958:-	ACA1_287760	JmjC domain-containing protein [Source:UniProtKB/TrEMBL;Acc:L8HIQ1]	0.017555433	0.500438	0.482882567	7.68E-07
6h	ACA1_097190/clean/scf7180000084645:1182101-1182239:-	ACA1_097190	JmjC domain-containing protein [Source:UniProtKB/TrEMBL;Acc:L8GIZ2]	0.010562327	0.493383667	0.48282134	4.26E-06
6h	ACA1_081870/clean/scf7180000084618:151975-152069:+	ACA1_081870	HIV1 Tat interactive protein 2, 30kDa, putative [Source:UniProtKB/TrEMBL;Acc:L8HE84]	0.00702051	0.489636333	0.482615823	9.28E-12
6h	ACA1_021690/clean/scf7180000084219:2216-2305:-	ACA1_021690	Uso1_p115_head domain-containing protein [Source:UniProtKB/TrEMBL;Acc:L8GJF5]	0.00267872	0.485051667	0.482372947	2.87E-08
6h	ACA1_230440/clean/scf7180000084761:349473-349564:+	ACA1_230440	WD_REPEATS_REGION domain-containing protein [Source:UniProtKB/TrEMBL;Acc:L8H839]	0	0.482251	0.482251	9.22E-08
6h	ACA1_093760/clean/scf7180000084645:639871-640020:+	ACA1_093760	ADPdependent glucokinase [Source:UniProtKB/TrEMBL;Acc:L8GIZ6]	0.001081327	0.483295333	0.482214007	3.15E-09
6h	ACA1_031790/clean/scf7180000084523:3111-3199:+	ACA1_031790	Rap-GAP domain-containing protein [Source:UniProtKB/TrEMBL;Acc:L8H1P5]	0.004385967	0.486474	0.482088033	1.38E-08
6h	ACA1_057090/clean/scf7180000084599:40383-40455:-	ACA1_057090	unspecified product	0.012473853	0.494517333	0.48204348	4.56E-09
6h	ACA1_271720/clean/scf7180000084771:91574-91677:-	ACA1_271720	Ras-GAP domain-containing protein [Source:UniProtKB/TrEMBL;Acc:L8GR76]	0	0.482017	0.482017	1.47E-09
6h	ACA1_277880/clean/scf7180000084776:118255-118323:+	ACA1_277880	RBR-type E3 ubiquitin transferase [Source:UniProtKB/TrEMBL;Acc:L8H6I3]	0.005716813	0.487603	0.481886187	1.39E-14
6h	ACA1_139150/clean/scf7180000084705:61307-61431:+	ACA1_139150	ENT domain-containing protein [Source:UniProtKB/TrEMBL;Acc:L8GN95]	0.0375938	0.519300333	0.481706533	1.75E-13
6h	ACA1_369600/clean/scf7180000084664:343551-343618:+	ACA1_369600	PH domain containing protein [Source:UniProtKB/TrEMBL;Acc:L8GZ46]	0.021672433	0.503248333	0.4815759	5.89E-16
6h	ACA1_053310/clean/scf7180000084594:261120-261214:+	ACA1_053310	Serine/threonine kinase [Source:UniProtKB/TrEMBL;Acc:L8H7D1]	0	0.481195	0.481195	2.74E-08
6h	ACA1_264360/clean/scf7180000084770:249206-249296:+	ACA1_264360	hypothetical protein	0.044718633	0.525795667	0.481077033	4.33E-08
6h	ACA1_285450/clean/scf7180000084776:968477-968563:-	ACA1_285450	TYR_PHOSPHATASE_2 domain-containing protein [Source:UniProtKB/TrEMBL;Acc:L8H9U5]	0.003144653	0.484107667	0.480963013	4.97E-09
6h	ACA1_025410/clean/scf7180000084446:14306-14377:+	ACA1_025410	Serine/threonine protein kinase [Source:UniProtKB/TrEMBL;Acc:L8HGY2]	0.025608933	0.506556	0.480947067	5.68E-18
6h	ACA1_220900/clean/scf7180000084758:972138-972231:-	ACA1_220900	MYND finger domain containing protein [Source:UniProtKB/TrEMBL;Acc:L8GQ59]	0	0.480926333	0.480926333	0.00014572
6h	ACA1_007760/clean/scf7180000083166:21352-21463:+	ACA1_007760	PH domain containing protein [Source:UniProtKB/TrEMBL;Acc:L8HAY1]	0	0.480859667	0.480859667	1.08E-09
6h	ACA1_150720/clean/scf7180000084716:26078-26203:-	ACA1_150720	TPR_REGION domain-containing protein [Source:UniProtKB/TrEMBL;Acc:L8H0N3]	0.161056667	0.641500667	0.480444	3.02E-09
6h	ACA1_376600/clean/scf7180000084685:115786-115883:-	ACA1_376600	Choline/Carnitine oacyltransferase superfamily protein [Source:UniProtKB/TrEMBL;Acc:L8HG13]	0	0.480392	0.480392	6.65E-12
6h	ACA1_315060/clean/scf7180000083887:26780-26897:-	ACA1_315060	AAA domain-containing protein [Source:UniProtKB/TrEMBL;Acc:L8GZM9]	0.002788083	0.483132667	0.480344584	1.21E-22
6h	ACA1_263610/clean/scf7180000084770:104388-104476:+	ACA1_263610	hypothetical protein	0.0092127	0.489541	0.4803283	4.18E-12
6h	ACA1_100130/clean/scf7180000084648:42508-42582:+	ACA1_100130	Metal cation transporter, ZIP subfamily protein [Source:UniProtKB/TrEMBL;Acc:L8GKF2]	0	0.480260667	0.480260667	0.007750119
6h	ACA1_128840/clean/scf7180000084697:108026-108092:+	ACA1_128840	CMP/dCMP-type deaminase domain-containing protein [Source:UniProtKB/TrEMBL;Acc:L8GVK0]	0.011514967	0.491662667	0.4801477	3.55E-07
6h	ACA1_233020/clean/scf7180000084762:105959-106078:-	ACA1_233020	hypothetical protein	0.00811103	0.488131333	0.480020303	1.20E-21
6h	ACA1_046640/clean/scf7180000084577:156885-156968:-	ACA1_046640	ANK_REP_REGION domain-containing protein [Source:UniProtKB/TrEMBL;Acc:L8H8U5]	0.0214744	0.501481667	0.480007267	0.000253166
6h	ACA1_255750/clean/scf7180000084768:561918-562089:+	ACA1_255750	hypothetical protein	0.179018667	0.658911	0.479892333	2.14E-06
6h	ACA1_097670/clean/scf7180000084645:1314191-1314306:+	ACA1_097670	hypothetical protein	0.01058422	0.490437	0.47985278	1.01E-06
6h	ACA1_264270/clean/scf7180000084770:227620-227701:-	ACA1_264270	Oxidoreductase, short chain dehydrogenase/reductase family protein [Source:UniProtKB/TrEMBL;Acc:L8H474]	0.00572341	0.485548	0.47982459	1.08E-09
6h	ACA1_028720/clean/scf7180000084473:22637-22740:-	ACA1_028720	RING-type domain-containing protein [Source:UniProtKB/TrEMBL;Acc:L8GWG0]	0.01727872	0.497101333	0.479822613	2.82E-06
6h	ACA1_136190/clean/scf7180000084702:150505-150600:+	ACA1_136190	SER_THR_PHOSPHATASE domain-containing protein [Source:UniProtKB/TrEMBL;Acc:L8GF93]	0.01920702	0.499023333	0.479816313	1.25E-09
6h	ACA1_048880/clean/scf7180000084583:10471-10596:+	ACA1_048880	LIM zinc-binding domain-containing protein [Source:UniProtKB/TrEMBL;Acc:L8HKD0]	0.1208421	0.600518667	0.479676567	9.63E-11
6h	ACA1_155470/clean/scf7180000084722:163461-163564:-	ACA1_155470	unspecified product	0.068217833	0.547852333	0.4796345	5.21E-08
6h	ACA1_063230/clean/scf7180000084599:1361746-1361840:-	ACA1_063230	Flagellar associated protein [Source:UniProtKB/TrEMBL;Acc:L8GWG8]	0.019121107	0.498702333	0.479581227	1.38E-09
6h	ACA1_145180/clean/scf7180000084710:53858-53921:-	ACA1_145180	F-box domain-containing protein [Source:UniProtKB/TrEMBL;Acc:L8GCU3]	0.065831533	0.545401667	0.479570133	1.00E-09
6h	ACA1_033500/clean/scf7180000084559:27975-28066:+	ACA1_033500	SAM domain-containing protein [Source:UniProtKB/TrEMBL;Acc:L8GGV5]	0.004385967	0.483934333	0.479548367	1.36E-07
6h	ACA1_176050/clean/scf7180000084741:1115030-1115110:-	ACA1_176050	Poly [ADP-ribose] polymerase [Source:UniProtKB/TrEMBL;Acc:L8HI82]	0.016132323	0.495438333	0.47930601	6.18E-17
6h	ACA1_110060/clean/scf7180000084663:50654-50726:-	ACA1_110060	Initiation factor, subunit 2 family protein [Source:UniProtKB/TrEMBL;Acc:L8HHD7]	0.000729873	0.480029333	0.47929946	1.77E-12
6h	ACA1_101520/clean/scf7180000084649:84188-84292:-	ACA1_101520	ANK_REP_REGION domain-containing protein [Source:UniProtKB/TrEMBL;Acc:L8GIW9]	0.002166476	0.481434667	0.479268191	5.96E-11
6h	ACA1_371710/clean/scf7180000084664:587403-587473:-	ACA1_371710	Fbox domain containing protein [Source:UniProtKB/TrEMBL;Acc:L8GZD1]	0.0185328	0.497763667	0.479230867	1.51E-09
6h	ACA1_080550/clean/scf7180000084618:31513-31572:+	ACA1_080550	FAD binding domain containing protein [Source:UniProtKB/TrEMBL;Acc:L8HBU6]	0.254557133	0.733589333	0.4790322	6.26E-07
6h	ACA1_215730/clean/scf7180000084758:384498-384583:+	ACA1_215730	Endo/exonuclease/phosphatase domain-containing protein [Source:UniProtKB/TrEMBL;Acc:L8GSM7]	0.0149393	0.493963	0.4790237	6.03E-05
6h	ACA1_040720/clean/scf7180000084572:61279-61361:+	ACA1_040720	Adaptin_N domain-containing protein [Source:UniProtKB/TrEMBL;Acc:L8H2X2]	0.00388685	0.482816333	0.478929484	1.59E-10
6h	ACA1_383090/clean/scf7180000084721:137751-137825:-	ACA1_383090	Ras subfamily protein [Source:UniProtKB/TrEMBL;Acc:L8GW16]	0.018488357	0.497386333	0.478897977	1.61E-05
6h	ACA1_315050/clean/scf7180000083887:23635-23738:-	ACA1_315050	Chain a, 4ank: a designed ankyrin repeat protein [Source:UniProtKB/TrEMBL;Acc:L8GYU2]	0.042058733	0.520933333	0.4788746	2.37E-12
6h	ACA1_060320/clean/scf7180000084599:645729-645930:-	ACA1_060320	ABC transporter, ATPbinding domain containing protein [Source:UniProtKB/TrEMBL;Acc:L8GWC7]	0.411620333	0.890313333	0.478693	0.015844228
6h	ACA1_048490/clean/scf7180000084582:123238-123310:+	ACA1_048490	Coatomer subunit alpha [Source:UniProtKB/TrEMBL;Acc:L8GEB3]	0.0043573	0.483028667	0.478671367	2.06E-11
6h	ACA1_182400/clean/scf7180000084745:205223-205407:+	ACA1_182400	Integral membrane protein [Source:UniProtKB/TrEMBL;Acc:L8H9C3]	0.007505213	0.486134667	0.478629453	5.21E-14
6h	ACA1_089960/clean/scf7180000084639:403854-403923:-	ACA1_089960	Dual specificity protein kinase [Source:UniProtKB/TrEMBL;Acc:L8GVS6]	0.030222833	0.508810333	0.4785875	1.50E-12
6h	ACA1_182120/clean/scf7180000084745:133465-133567:+	ACA1_182120	WD repeat domain 5B, putative [Source:UniProtKB/TrEMBL;Acc:L8HA46]	0.009174773	0.487749667	0.478574893	1.52E-12
6h	ACA1_061500/clean/scf7180000084599:945591-945680:+	ACA1_061500	Ras-GAP domain-containing protein [Source:UniProtKB/TrEMBL;Acc:L8GWM5]	0.010064163	0.48857	0.478505837	3.27E-12
6h	ACA1_290180/clean/scf7180000084777:547112-547176:+	ACA1_290180	hypothetical protein	0.188322	0.666666667	0.478344667	0.000117712
6h	ACA1_389490/clean/scf7180000084733:268098-268238:-	ACA1_389490	RRM domain-containing protein [Source:UniProtKB/TrEMBL;Acc:L8GES5]	0.0246041	0.502852	0.4782479	4.82E-11
6h	ACA1_000820/clean/scf7180000082828:9531-9606:+	ACA1_000820	RING-type domain-containing protein [Source:UniProtKB/TrEMBL;Acc:L8H7M3]	0.004065033	0.482297333	0.4782323	5.41E-08
6h	ACA1_072530/clean/scf7180000084603:1220959-1221038:+	ACA1_072530	Cytochrome c oxidase assembly protein, putative [Source:UniProtKB/TrEMBL;Acc:L8HGT1]	0.020067143	0.498093	0.478025857	2.02E-12
6h	ACA1_054480/clean/scf7180000084594:509797-509869:+	ACA1_054480	Pribosyltran domain-containing protein [Source:UniProtKB/TrEMBL;Acc:L8H652]	0.00904999	0.487038667	0.477988677	2.58E-06
6h	ACA1_189300/clean/scf7180000084748:823-910:+	ACA1_189300	hypothetical protein	0.030767133	0.508613	0.477845867	1.16E-13
6h	ACA1_066020/clean/scf7180000084599:1974254-1974352:+	ACA1_066020	CBS domain containing protein [Source:UniProtKB/TrEMBL;Acc:L8H067]	0.033333333	0.511178667	0.477845333	0.001881832
6h	ACA1_237770/clean/scf7180000084763:10256-10371:+	ACA1_237770	PH domain-containing protein [Source:UniProtKB/TrEMBL;Acc:L8GKB5]	0.003472233	0.481226333	0.4777541	1.43E-08
6h	ACA1_035640/clean/scf7180000084564:102338-102416:-	ACA1_035640	Beta-lactamase domain-containing protein [Source:UniProtKB/TrEMBL;Acc:L8H9P2]	0	0.477640667	0.477640667	0.000260815
6h	ACA1_159190/clean/scf7180000084724:357570-357632:-	ACA1_159190	hypothetical protein	0.0387279	0.516302	0.4775741	3.05E-08
6h	ACA1_262100/clean/scf7180000084770:14286-14357:+	ACA1_262100	JmjC domain-containing protein [Source:UniProtKB/TrEMBL;Acc:L8H1V0]	0.003893153	0.481466667	0.477573513	9.26E-10
6h	ACA1_106270/clean/scf7180000084660:67312-67407:-	ACA1_106270	ZT_dimer domain-containing protein [Source:UniProtKB/TrEMBL;Acc:L8GQ80]	0.018614423	0.496185333	0.47757091	7.84E-07
6h	ACA1_063760/clean/scf7180000084599:1497266-1497380:+	ACA1_063760	PH domain-containing protein [Source:UniProtKB/TrEMBL;Acc:L8GXG1]	0.149265	0.626828	0.477563	7.12E-07
6h	ACA1_074110/clean/scf7180000084605:26556-26636:-	ACA1_074110	Peptidase [Source:UniProtKB/TrEMBL;Acc:L8HJ70]	0.0093027	0.486789333	0.477486633	2.08E-14
6h	ACA1_062550/clean/scf7180000084599:1228360-1228443:+	ACA1_062550	TPR_REGION domain-containing protein [Source:UniProtKB/TrEMBL;Acc:L8GZH6]	0.00208896	0.479346333	0.477257373	3.45E-10
6h	ACA1_150950/clean/scf7180000084716:31777-31849:+	ACA1_150950	Exocyst complex component Sec6 [Source:UniProtKB/TrEMBL;Acc:L8H215]	0.001719197	0.478713	0.476993803	6.51E-11
6h	ACA1_121840/clean/scf7180000084682:109160-109230:+	ACA1_121840	Protein kinase domain-containing protein [Source:UniProtKB/TrEMBL;Acc:L8GFF3]	0.0138889	0.490844333	0.476955433	7.20E-11
6h	ACA1_012020/clean/scf7180000083652:5780-5918:-	ACA1_012020	hypothetical protein	0.009409117	0.486349333	0.476940217	3.66E-19
6h	ACA1_326070/clean/scf7180000084504:13184-13265:+	ACA1_326070	START domain-containing protein [Source:UniProtKB/TrEMBL;Acc:L8HG48]	0.027739933	0.504510667	0.476770733	4.35E-09
6h	ACA1_150720/clean/scf7180000084716:24813-24943:-	ACA1_150720	TPR_REGION domain-containing protein [Source:UniProtKB/TrEMBL;Acc:L8H0N3]	0.00656618	0.48328	0.47671382	9.75E-15
6h	ACA1_121540/clean/scf7180000084682:57609-57685:+	ACA1_121540	Peptidase_M1 domain-containing protein [Source:UniProtKB/TrEMBL;Acc:L8GGX0]	0	0.476590333	0.476590333	3.67E-11
6h	ACA1_052590/clean/scf7180000084594:117536-117595:+	ACA1_052590	PeptidyltRNA hydrolase PTH2 domain containing protein [Source:UniProtKB/TrEMBL;Acc:L8H778]	0.007129453	0.483637	0.476507547	3.97E-12
6h	ACA1_372700/clean/scf7180000084679:59067-59150:-	ACA1_372700	hypothetical protein	0.003333333	0.479797	0.476463667	8.93E-09

6h	ACA1_088960/clean/scf7180000084639:275091-275161:-	ACA1_088960	Beta-lactamase domain-containing protein [Source:UniProtKB/TrEMBL;Acc:L8GU04]	0	0.476309333	0.476309333	3.45E-07
6h	ACA1_298630/clean/scf7180000082992:2726-2837:-	ACA1_298630	Vacuolar protein sorting 16, putative [Source:UniProtKB/TrEMBL;Acc:L8H081]	0.01019659	0.486023	0.47582641	5.88E-12
6h	ACA1_117020/clean/scf7180000084669:893185-893263:+	ACA1_117020	Carboxypeptidase [Source:UniProtKB/TrEMBL;Acc:L8H3Z1]	0.0036358	0.479159	0.4755232	1.08E-11
6h	ACA1_362670/clean/scf7180000084624:135081-135169:+	ACA1_362670	Rieske domain-containing protein [Source:UniProtKB/TrEMBL;Acc:L8GHW3]	0	0.475468	0.475468	2.60E-13
6h	ACA1_095260/clean/scf7180000084645:824660-824750:-	ACA1_095260	F-box domain-containing protein [Source:UniProtKB/TrEMBL;Acc:L8GL23]	0.008883207	0.484282667	0.47539946	7.96E-07
6h	ACA1_151320/clean/scf7180000084716:103003-103108:-	ACA1_151320	PPM-type phosphatase domain-containing protein [Source:UniProtKB/TrEMBL;Acc:L8GZX5]	0.00245098	0.477777	0.47532602	4.35E-10
6h	ACA1_210530/clean/scf7180000084758:47739-47843:-	ACA1_210530	4Fe-4S ferredoxin-type domain-containing protein [Source:UniProtKB/TrEMBL;Acc:L8GS75]	0	0.475304667	0.475304667	4.08E-10
6h	ACA1_240060/clean/scf7180000084763:191820-191899:-	ACA1_240060	COesterase domain-containing protein [Source:UniProtKB/TrEMBL;Acc:L8GKI1]	0.00131329	0.476529	0.47521571	1.57E-11
6h	ACA1_024200/clean/scf7180000084441:14633-14719:-	ACA1_024200	WD_REPEATS_REGION domain-containing protein [Source:UniProtKB/TrEMBL;Acc:L8GKS0]	0.030725506	0.505437333	0.474711827	1.80E-06
6h	ACA1_351930/clean/scf7180000084695:37199-37294:-	ACA1_351930	hypothetical protein	0.009360667	0.484017333	0.474656667	2.52E-09
6h	ACA1_290440/clean/scf7180000084777:592407-592509:-	ACA1_290440	hypothetical protein	0.007564097	0.482160333	0.474596237	1.83E-07
6h	ACA1_173980/clean/scf7180000084741:565611-565737:-	ACA1_173980	OTU domain-containing protein [Source:UniProtKB/TrEMBL;Acc:L8HHS8]	0	0.474514333	0.474514333	1.35E-10
6h	ACA1_062650/clean/scf7180000084599:1264261-1264341:+	ACA1_062650	hypothetical protein	0	0.474331667	0.474331667	1.07E-08
6h	ACA1_264460/clean/scf7180000084770:294092-294171:-	ACA1_264460	GTPbinding protein [Source:UniProtKB/TrEMBL;Acc:L8H231]	0.005509633	0.479703	0.474193367	3.14E-10
6h	ACA1_137810/clean/scf7180000084703:75859-75969:-	ACA1_137810	PPR_long domain-containing protein [Source:UniProtKB/TrEMBL;Acc:L8GYN4]	0.009294907	0.48343	0.474135093	4.91E-06
6h	ACA1_150950/clean/scf7180000084716:34364-34467:+	ACA1_150950	Exocyst complex component Sec6 [Source:UniProtKB/TrEMBL;Acc:L8H215]	0.007246367	0.481319667	0.4740733	5.47E-09
6h	ACA1_095220/clean/scf7180000084645:815066-815222:-	ACA1_095220	Protein kinase domain-containing protein [Source:UniProtKB/TrEMBL;Acc:L8GIR4]	0.1038276	0.577759333	0.473931733	1.37E-09
6h	ACA1_377320/clean/scf7180000084711:25648-25716:+	ACA1_377320	SPRY domain containing protein [Source:UniProtKB/TrEMBL;Acc:L8GSS3]	0.008207563	0.482031	0.473823437	2.36E-13
6h	ACA1_116740/clean/scf7180000084669:839044-839133:+	ACA1_116740	Methyltransf_2 domain-containing protein [Source:UniProtKB/TrEMBL;Acc:L8H4R1]	0.004885133	0.478540667	0.473655533	3.87E-07
6h	ACA1_062080/clean/scf7180000084599:1117584-1117650:-	ACA1_062080	DUF2263 domain-containing protein [Source:UniProtKB/TrEMBL;Acc:L8GZD2]	0.001670463	0.475291667	0.473621203	9.33E-09
6h	ACA1_371760/clean/scf7180000084664:600814-600921:-	ACA1_371760	PH domain-containing protein [Source:UniProtKB/TrEMBL;Acc:L8H1R1]	0.01350036	0.487067	0.47356664	3.55E-07
6h	ACA1_034600/clean/scf7180000084563:85402-85477:+	ACA1_034600	hypothetical protein	0.0546801	0.528062667	0.473382567	2.60E-07
6h	ACA1_264360/clean/scf7180000084770:247246-247338:+	ACA1_264360	hypothetical protein	0.005952367	0.479303	0.473350633	9.24E-08
6h	ACA1_321160/clean/scf7180000084437:1283-1383:-	ACA1_321160	FAD-binding FR-type domain-containing protein [Source:UniProtKB/TrEMBL;Acc:L8GGN4]	0.006246052	0.479526333	0.473280281	1.14E-07
6h	ACA1_230540/clean/scf7180000084761:374608-374681:+	ACA1_230540	hypothetical protein	0.0162164	0.489491	0.4732746	8.34E-17
6h	ACA1_061670/clean/scf7180000084599:999525-999601:-	ACA1_061670	SSD domain-containing protein [Source:UniProtKB/TrEMBL;Acc:L8GWV2]	0	0.473239	0.473239	1.70E-08
6h	ACA1_203970/clean/scf7180000084754:241877-241966:-	ACA1_203970	HECTdomain (Ubiquitin-transferase) domain containing protein [Source:UniProtKB/TrEMBL;Acc:L8GTT3]	0.005782867	0.478998333	0.473215467	9.86E-07
6h	ACA1_230510/clean/scf7180000084761:368478-368553:-	ACA1_230510	hypothetical protein	0.00230947	0.475401667	0.473092197	3.10E-08
6h	ACA1_038180/clean/scf7180000084568:90704-90794:+	ACA1_038180	FHA domain containing protein [Source:UniProtKB/TrEMBL;Acc:L8GLQ3]	0.013925607	0.486970667	0.47304506	2.32E-09
6h	ACA1_037780/clean/scf7180000084568:24668-24779:+	ACA1_037780	RhoGEF domain containing protein [Source:UniProtKB/TrEMBL;Acc:L8GN17]	0.009549663	0.482588	0.473038337	7.97E-08
6h	ACA1_256610/clean/scf7180000084769:64624-64695:-	ACA1_256610	hypothetical protein	0	0.473018	0.473018	0.002689845
6h	ACA1_002780/clean/scf7180000082937:9646-9732:-	ACA1_002780	RRM domain-containing protein [Source:UniProtKB/TrEMBL;Acc:L8GGZ7]	0.0479374	0.520887	0.4729496	1.06E-06
6h	ACA1_278900/clean/scf7180000084776:272854-272964:+	ACA1_278900	ANK_REP_REGION domain-containing protein [Source:UniProtKB/TrEMBL;Acc:L8H5W6]	0.012860767	0.485706	0.472845233	2.89E-12
6h	ACA1_012310/clean/scf7180000083740:11493-11577:+	ACA1_012310	Biogenesis of lysosome related family protein [Source:UniProtKB/TrEMBL;Acc:L8HEJ0]	0.017336506	0.490112333	0.472775827	1.32E-15
6h	ACA1_097600/clean/scf7180000084645:1292967-1293075:+	ACA1_097600	Peroxidase [Source:UniProtKB/TrEMBL;Acc:L8GJT1]	0.000560254	0.473032333	0.472472079	7.73E-25
6h	ACA1_321790/clean/scf7180000084455:24845-24919:+	ACA1_321790	Protein phosphatase 2C domain containing protein [Source:UniProtKB/TrEMBL;Acc:L8GSL9]	0.015873	0.488334333	0.472461333	2.05E-05
6h	ACA1_278000/clean/scf7180000084776:150636-150704:+	ACA1_278000	Carrier superfamily protein [Source:UniProtKB/TrEMBL;Acc:L8H5S3]	0.021560233	0.493986	0.472425767	2.84E-08
6h	ACA1_097520/clean/scf7180000084645:1266668-1266744:-	ACA1_097520	hypothetical protein	0.00336615	0.475785333	0.472419183	2.18E-26
6h	ACA1_174880/clean/scf7180000084741:820094-820177:-	ACA1_174880	Cation transport domain containing protein [Source:UniProtKB/TrEMBL;Acc:L8HI03]	0.072294367	0.544482	0.472187633	4.86E-05
6h	ACA1_061500/clean/scf7180000084599:943300-943400:+	ACA1_061500	Ras-GAP domain-containing protein [Source:UniProtKB/TrEMBL;Acc:L8GWM5]	0.000376168	0.472388667	0.472012498	2.05E-12
6h	ACA1_026390/clean/scf7180000084456:9941-10035:-	ACA1_026390	EFhand domain containing protein [Source:UniProtKB/TrEMBL;Acc:L8GMZ4]	0.009321177	0.481313	0.471991823	8.26E-17
6h	ACA1_114870/clean/scf7180000084669:570774-570884:+	ACA1_114870	TAF4 domain-containing protein [Source:UniProtKB/TrEMBL;Acc:L8H4I0]	0.009572633	0.481512667	0.471940033	8.30E-08
6h	ACA1_149030/clean/scf7180000084715:53559-53640:+	ACA1_149030	Thioredoxin domain-containing protein [Source:UniProtKB/TrEMBL;Acc:L8HEB1]	0.000475607	0.472402667	0.47192706	1.96E-09
6h	ACA1_174810/clean/scf7180000084741:803732-803809:+	ACA1_174810	Cytochrome p450 superfamily protein [Source:UniProtKB/TrEMBL;Acc:L8HGX6]	0.021829967	0.493707	0.471877033	1.35E-14
6h	ACA1_247140/clean/scf7180000084763:629505-629573:+	ACA1_247140	hypothetical protein	0.0095238	0.481361	0.4718372	2.39E-07
6h	ACA1_051900/clean/scf7180000084592:79506-79583:+	ACA1_051900	DOCKER domain-containing protein [Source:UniProtKB/TrEMBL;Acc:L8GPU7]	0.0760271	0.547394667	0.471367567	0.000878696
6h	ACA1_289740/clean/scf7180000084777:453935-454019:+	ACA1_289740	Rho-GAP domain-containing protein [Source:UniProtKB/TrEMBL;Acc:L8HLF4]	0.001533923	0.472853333	0.47131941	1.07E-08
6h	ACA1_175240/clean/scf7180000084741:899197-899283:+	ACA1_175240	Rab32 protein, putative [Source:UniProtKB/TrEMBL;Acc:L8HI25]	0.019829083	0.491036333	0.47120725	2.85E-12
6h	ACA1_184770/clean/scf7180000084746:58348-58429:-	ACA1_184770	Aminotran_1_2 domain-containing protein [Source:UniProtKB/TrEMBL;Acc:L8H468]	0.03356218	0.504654	0.47109182	1.51E-09
6h	ACA1_061670/clean/scf7180000084599:1002349-1002427:-	ACA1_061670	SSD domain-containing protein [Source:UniProtKB/TrEMBL;Acc:L8GWV2]	0.017543867	0.488503667	0.4709598	6.55E-06
6h	ACA1_228670/clean/scf7180000084761:188026-188136:+	ACA1_228670	hypothetical protein	0.020768467	0.491673	0.470904533	5.78E-14
6h	ACA1_264380/clean/scf7180000084770:255988-256087:-	ACA1_264380	S-formylglutathione hydrolase [Source:UniProtKB/TrEMBL;Acc:L8H1Z7]	0	0.470743333	0.470743333	1.00E-13
6h	ACA1_074140/clean/scf7180000084605:36353-36439:+	ACA1_074140	Transporter, major facilitator subfamily protein [Source:UniProtKB/TrEMBL;Acc:L8HK94]	0.031226167	0.501935667	0.4707095	8.94E-11
6h	ACA1_284210/clean/scf7180000084776:891613-891897:-	ACA1_284210	Oxoglutarate carrier, putative [Source:UniProtKB/TrEMBL;Acc:L8H8V6]	0.0222596	0.492948333	0.470688733	8.63E-12
6h	ACA1_024200/clean/scf7180000084441:15012-15094:-	ACA1_024200	WD_REPEATS_REGION domain-containing protein [Source:UniProtKB/TrEMBL;Acc:L8GKS0]	0.001317523	0.471785	0.470467477	2.84E-08
6h	ACA1_074140/clean/scf7180000084605:35972-36043:+	ACA1_074140	Transporter, major facilitator subfamily protein [Source:UniProtKB/TrEMBL;Acc:L8HK94]	0.01216606	0.482555333	0.470389273	2.22E-19
6h	ACA1_215970/clean/scf7180000084758:418669-418750:-	ACA1_215970	EGFlike domain containing protein [Source:UniProtKB/TrEMBL;Acc:L8GQ73]	0	0.470334	0.470334	1.65E-05
6h	ACA1_068620/clean/scf7180000084603:240917-241034:-	ACA1_068620	hypothetical protein	0	0.470234333	0.470234333	6.87E-10
6h	ACA1_133510/clean/scf7180000084701:72695-72772:+	ACA1_133510	Glutathione peroxidase [Source:UniProtKB/TrEMBL;Acc:L8H527]	0	0.470232667	0.470232667	7.42E-15
6h	ACA1_306850/clean/scf7180000083668:20589-20677:+	ACA1_306850	RanBPMrelated, putative [Source:UniProtKB/TrEMBL;Acc:L8GNY2]	0.012410547	0.482635667	0.47022512	9.46E-12
6h	ACA1_175460/clean/scf7180000084741:964122-964194:-	ACA1_175460	hypothetical protein	0.044544067	0.514680333	0.470136267	5.57E-05
6h	ACA1_103170/clean/scf7180000084653:13209-13280:+	ACA1_103170	CBS domain containing protein [Source:UniProtKB/TrEMBL;Acc:L8GCP7]	0.123003933	0.593075667	0.470071733	1.64E-06
6h	ACA1_288660/clean/scf7180000084777:255931-255996:+	ACA1_288660	hypothetical protein	0.0076336	0.477678667	0.470045067	9.76E-11
6h	ACA1_011490/clean/scf7180000083641:7562-7646:+	ACA1_011490	DH domain-containing protein [Source:UniProtKB/TrEMBL;Acc:L8GZK7]	0.003290197	0.473287	0.469996803	6.01E-08
6h	ACA1_390600/clean/scf7180000084740:79999-80082:-	ACA1_390600	Metallophos domain-containing protein [Source:UniProtKB/TrEMBL;Acc:L8GNV1]	0.05044508	0.520403333	0.469958253	4.94E-07
6h	ACA1_223350/clean/scf7180000084760:138324-138433:+	ACA1_223350	F-box domain-containing protein [Source:UniProtKB/TrEMBL;Acc:L8GSX8]	0.017877733	0.487815333	0.4699376	8.81E-08
6h	ACA1_116560/clean/scf7180000084669:781449-781520:-	ACA1_116560	PINc domain-containing protein [Source:UniProtKB/TrEMBL;Acc:L8H4U3]	0	0.469872667	0.469872667	3.01E-05
6h	ACA1_256450/clean/scf7180000084769:34236-34297:+	ACA1_256450	Poly [ADP-ribose] polymerase [Source:UniProtKB/TrEMBL;Acc:L8GH34]	0.005007167	0.474727333	0.469720167	6.53E-08
6h	ACA1_239220/clean/scf7180000084763:139205-139339:-	ACA1_239220	ApaG domain-containing protein [Source:UniProtKB/TrEMBL;Acc:L8GJW5]	0.0601659	0.529880667	0.469714767	2.66E-07
6h	ACA1_288290/clean/scf7180000084777:161048-161128:+	ACA1_288290	SH3 domain-containing protein [Source:UniProtKB/TrEMBL;Acc:L8HKD1]	0.007222983	0.476838333	0.46961535	3.59E-08
6h	ACA1_097640/clean/scf7180000084645:1303348-1303443:+	ACA1_097640	Fbox domain containing protein [Source:UniProtKB/TrEMBL;Acc:L8GK52]	0	0.4696	0.4696	6.30E-10
6h	ACA1_131570/clean/scf7180000084699:89928-90029:-	ACA1_131570	Transmembrane proteins 14c protein [Source:UniProtKB/TrEMBL;Acc:L8H2Q4]	0.007279297	0.476840333	0.469561037	1.49E-12
6h	ACA1_325440/clean/scf7180000084495:9064-9161:+	ACA1_325440	hypothetical protein	0.00075255	0.470278333	0.469525784	3.66E-12
6h	ACA1_184420/clean/scf7180000084745:640192-640285:+	ACA1_184420	Cathepsin Llike proteinase [Source:UniProtKB/TrEMBL;Acc:L8H8J9]	0.001605612	0.471110333	0.469504722	4.36E-14
6h	ACA1_183130/clean/scf7180000084745:338433-338503:-	ACA1_183130	PNP_UDP_1 domain-containing protein [Source:UniProtKB/TrEMBL;Acc:L8H767]	0.160293	0.629714333	0.469421333	2.44E-08
6h	ACA1_203070/clean/scf7180000084754:134949-135029:+	ACA1_203070	Fbox domain containing protein [Source:UniProtKB/TrEMBL;Acc:L8GUJ5]	0.006278323	0.475655667	0.469377343	8.64E-09
6h	ACA1_155670/clean/scf7180000084722:212265-212357:+	ACA1_155670	SPRY domain containing protein [Source:UniProtKB/TrEMBL;Acc:L8H063]	0.000564613	0.469929333	0.46936472	4.26E-12
6h	ACA1_053340/clean/scf7180000084594:265973-266111:-	ACA1_053340	Dynein light intermediate chain [Source:UniProtKB/TrEMBL;Acc:L8H840]	0.051041133	0.520347333	0.4693062	5.01E-12
6h	ACA1_070890/clean/scf7180000084603:796399-796459:-	ACA1_070890	hypothetical protein	0.088166567	0.557448	0.469281433	1.49E-11
6h	ACA1_071240/clean/scf7180000084603:895983-896129:-	ACA1_071240	Transporter, major facilitator subfamily protein [Source:UniProtKB/TrEMBL;Acc:L8HFI7]	0.016438567	0.485614333	0.469175767	3.13E-08
6h	ACA1_137790/clean/scf7180000084703:72675-72755:-	ACA1_137790	IBR domain-containing protein [Source:UniProtKB/TrEMBL;Acc:L8GZJ6]	0.071859467	0.540998667	0.4691392	0.000177299
6h	ACA1_199010/clean/scf7180000084753:192086-192182:+	ACA1_199010	JmjC domain-containing protein [Source:UniProtKB/TrEMBL;Acc:L8H2E5]	0.011461733	0.480594667	0.469132933	1.13E-16
6h	ACA1_188010/clean/scf7180000084747:103555-103652:+	ACA1_188010	Importin 13, putative [Source:UniProtKB/TrEMBL;Acc:L8GRR2]	0.001053603	0.470176667	0.469123063	1.76E-08
6h	ACA1_348980/clean/scf7180000084689:23268-23352:+	ACA1_348980	3hydroxyanthranilate 3,4-dioxygenase [Source:UniProtKB/TrEMBL;Acc:L8GU67]	0.021961233	0.491082	0.469120767	3.99E-15
6h	ACA1_057460/clean/scf7180000084599:147412-147512:-	ACA1_057460	hypothetical protein	0.023007933	0.492038	0.469030067	7.08E-07
6h	ACA1_107410/clean/scf7180000084660:186158-186226:+	ACA1_107410	UBIQUITIN_CONJUGAT_2 domain-containing protein [Source:UniProtKB/TrEMBL;Acc:L8GMM6]	0.000661813	0.469571667	0.468909853	1.51E-10
6h	ACA1_191700/clean/scf7180000084749:33276-33365:-	ACA1_191700	Aldo_ket_red domain-containing protein [Source:UniProtKB/TrEMBL;Acc:L8GNA1]	0.040726633	0.509617	0.468890367	9.12E-12
6h	ACA1_180770/clean/scf7180000084744:76507-76598:+	ACA1_180770	Rasrelated C3 botulinum toxin substrate 1, putative [Source:UniProtKB/TrEMBL;Acc:L8GCB8]	0	0.468684	0.468684	1.83E-08
6h	ACA1_035560/clean/scf7180000084564:90049-90117:-	ACA1_035560	Short chain dehydrogenase/reductase family oxidoreductase [Source:UniProtKB/TrEMBL;Acc:L8HCW5]	0.00289855	0.471568333	0.468669783	2.39E-12
6h	ACA1_373610/clean/scf7180000084679:114855-114958:-	ACA1_373610	hypothetical protein	0.012861733	0.481443	0.468581267	1.47E-06
6h	ACA1_323130/clean/scf7180000084467:11228-11311:-	ACA1_323130	HemolysinIII related subfamily protein [Source:UniProtKB/TrEMBL;Acc:L8GGU0]	0	0.468568	0.468568	4.12E-08
6h	ACA1_365710/clean/scf7180000084641:299364-299447:-	ACA1_365710	Protein kinase domain-containing protein [Source:UniProtKB/TrEMBL;Acc:L8GPB8]	0.021778697	0.490215	0.468436303	1.39E-08
6h	ACA1_198250/clean/scf7180000084753:65183-65367:+	ACA1_198250	hypothetical protein	0.041661925	0.510037	0.468375075	0.000252292
6h	ACA1_042070/clean/scf7180000084573:53114-53201:+	ACA1_042070	Dual specificity protein kinase [Source:UniProtKB/TrEMBL;Acc:L8GW91]	0.029984397	0.498308	0.468323603	4.66E-12
6h	ACA1_077050/clean/scf7180000084612:223896-223994:-	ACA1_077050	hypothetical protein	0.047619	0.51594	0.468321	0.046397276
6h	ACA1_067810/clean/scf7180000084603:25920-26015:+	ACA1_067810	hypothetical protein	0.003015077	0.471274	0.468258923	7.28E-07
6h	ACA1_252450/clean/scf7180000084768:154080-154183:+	ACA1_252450	L1P family protein [Source:UniProtKB/TrEMBL;Acc:L8HAS4]	0.013696423	0.481811333	0.46811491	3.07E-11
6h	ACA1_013630/clean/scf7180000083751:15141-15206:-	ACA1_013630	Serine/threonine-protein phosphatase [Source:UniProtKB/TrEMBL;Acc:L8HEX2]	0.00768401	0.475680667	0.467996657	1.36E-18
6h	ACA1_068490/clean/scf7180000084603:214283-214349:-	ACA1_068490	Amino acid permease superfamily protein [Source:UniProtKB/TrEMBL;Acc:L8HDJ9]	0.035087733	0.502956667	0.467868933	4.62E-05
6h	ACA1_287900/clean/scf7180000084777:121656-121719:-	ACA1_287900	ATPase/histidine kinase/DNA gyrase B/HSP90 domain containing protein [Source:UniProtKB/TrEMBL;Acc:L8HHR0]	0.014074567	0.481886667	0.4678121	3.06E-05
6h	ACA1_069880/clean/scf7180000084603:585889-585970:-	ACA1_069880	hypothetical protein	0.005464467	0.473204	0.467739533	2.50E-08
6h	ACA1_356140/clean/scf7180000084731:14470-14560:-	ACA1_356140	RasGEF domain containing protein [Source:UniProtKB/TrEMBL;Acc:L8HHY3]	0	0.467642	0.467642	1.09E-07
6h	ACA1_116990/clean/scf7180000084669:885859-885973:+	ACA1_116990	Carboxypeptidase [Source:UniProtKB/TrEMBL;Acc:L8H6Y6]	0.000101184	0.467700667	0.467599483	5.23E-12
6h	ACA1_320790/clean/scf7180000084416:21487-21767:-	ACA1_320790	RUN domain-containing protein [Source:UniProtKB/TrEMBL;Acc:L8GTR9]	0.128266333	0.595843	0.467576667	9.22E-11
6h	ACA1_027500/clean/scf7180000084469:6883-6956:-	ACA1_027500	hypothetical protein	3.83E-05	0.467222333	0.467183997	1.57E-11
6h	ACA1_197850/clean/scf7180000084753:40430-40513:-	ACA1_197850	HAD hydrolase, family IIA subfamily protein [Source:UniProtKB/TrEMBL;Acc:L8H335]	0.0177711	0.484928	0.4671569	1.97E-08
6h	ACA1_083530/clean/scf7180000084629:657-759:-	ACA1_083530	hypothetical protein	0.008846127	0.475994	0.467147873	3.23E-08
6h	ACA1_179130/clean/scf7180000084743:304298-304411:+	ACA1_179130	Protein kinase domain containing protein [Source:UniProtKB/TrEMBL;Acc:L8GTH0]	0	0.467065667	0.467065667	2.26E-07
6h	ACA1_234110/clean/scf7180000084762:203365-203463:+	ACA1_234110	Sulfurtransferase [Source:UniProtKB/TrEMBL;Acc:L8H2J9]	0.0208664	0.487912333	0.467045933	1.57E-16
6h	ACA1_193230/clean/scf7180000084749:212585-212645:+	ACA1_193230	Macro domain-containing protein [Source:UniProtKB/TrEMBL;Acc:L8GN79]	0.012771467	0.479640667	0.4668692	1.55E-11
6h	ACA1_074850/clean/scf7180000084608:103944-104027:-	ACA1_074850	S5 DRBM domain-containing protein [Source:UniProtKB/TrEMBL;Acc:L8HGT0]	0	0.466785	0.466785	1.28E-07
6h	ACA1_135130/clean/scf7180000084702:68024-68094:-	ACA1_135130	SWIRM domain-containing protein [Source:UniProtKB/TrEMBL;Acc:L8GE50]	0.0130719	0.479831	0.4667591	2.14E-07
6h	ACA1_143770/clean/scf7180000084709:94219-94280:+	ACA1_143770	WD repeatcontaining protein 40A, putative [Source:UniProtKB/TrEMBL;Acc:L8HFH4]	0.424981	0.891699333	0.466718333	1.78E-09
6h	ACA1_072130/clean/scf7180000084603:1152690-1152798:+	ACA1_072130	hypothetical protein	0.014642367	0.481343667	0.4667013	4.41E-08
6h	ACA1_243850/clean/scf7180000084763:362555-362616:+	ACA1_243850	Ras-GAP domain-containing protein [Source:UniProtKB/TrEMBL;Acc:L8GJZ5]	0	0.466547	0.466547	0.013069794
6h	ACA1_140610/clean/scf7180000084707:100951-101047:-	ACA1_140610	Poly [ADP-ribose] polymerase [Source:UniProtKB/TrEMBL;Acc:L8GGG7]	0	0.466439333	0.466439333	1.90E-05
6h	ACA1_061140/clean/scf7180000084599:833415-833692:-	ACA1_061140	WD_REPEATS_REGION domain-containing protein [Source:UniProtKB/TrEMBL;Acc:L8GZ38]	0.0401757	0.506561333	0.466385633	2.53E-11
6h	ACA1_296510/clean/scf7180000084777:1368199-1368265:+	ACA1_296510	Phosphate ABC transporter, phosphatebinding protein PstS, putative [Source:UniProtKB/TrEMBL;Acc:L8HLK8]	0	0.466320667	0.466320667	0.000236914
6h	ACA1_063740/clean/scf7180000084599:1492697-1492779:-	ACA1_063740	Transmembrane protein [Source:UniProtKB/TrEMBL;Acc:L8GXB1]	0.016947567	0.483263	0.466315433	8.95E-13
6h	ACA1_127420/clean/scf7180000084696:18737-19121:-	ACA1_127420	Aconitase domain-containing protein [Source:UniProtKB/TrEMBL;Acc:L8GGD8]	0.010480467	0.476629	0.466148533	2.09E-25
6h	ACA1_137810/clean/scf7180000084703:74334-74460:-	ACA1_137810	PPR_long domain-containing protein [Source:UniProtKB/TrEMBL;Acc:L8GYN4]	0.016491367	0.482593	0.466101633	3.04E-05
6h	ACA1_172100/clean/scf7180000084741:247283-247378:+	ACA1_172100	PIPK domain-containing protein [Source:UniProtKB/TrEMBL;Acc:L8HHI9]	0.031560267	0.497627	0.466066733	1.34E-12
6h	ACA1_279280/clean/scf7180000084776:346662-346742:-	ACA1_279280	Lcarnitine dehydratase/bile acid-inducible protein F, putative [Source:UniProtKB/TrEMBL;Acc:L8H915]	0.022178333	0.488211667	0.466033333	9.49E-09
6h	ACA1_197850/clean/scf7180000084753:39614-39697:-	ACA1_197850	HAD hydrolase, family IIA subfamily protein [Source:UniProtKB/TrEMBL;Acc:L8H335]	0.006831277	0.472777667	0.46594639	1.84E-10
6h	ACA1_172230/clean/scf7180000084741:280114-280212:+	ACA1_172230	Serine/threonine-protein phosphatase 2A activator [Source:UniProtKB/TrEMBL;Acc:L8HIY6]	0.00330014	0.469170667	0.465870527	1.06E-09
6h	ACA1_204490/clean/scf7180000084754:291375-291451:+	ACA1_204490	Inosine triphosphate pyrophosphatase [Source:UniProtKB/TrEMBL;Acc:L8GTU7]	0.000119496	0.465823333	0.465703838	8.09E-12
6h	ACA1_385720/clean/scf7180000084730:250366-250427:+	ACA1_385720	Protein kinase domain containing protein [Source:UniProtKB/TrEMBL;Acc:L8H9D4]	0.012990107	0.478529	0.465538893	1.21E-07
6h	ACA1_189890/clean/scf7180000084748:107335-107408:-	ACA1_189890	Usp domain-containing protein [Source:UniProtKB/TrEMBL;Acc:L8GEZ9]	0.007697853	0.473215	0.465517147	2.44E-17
6h	ACA1_223310/clean/scf7180000084760:127174-127284:-	ACA1_223310	Amidohydro_3 domain-containing protein [Source:UniProtKB/TrEMBL;Acc:L8GTU8]	0.018231023	0.483557667	0.465326643	8.55E-07
6h	ACA1_295580/clean/scf7180000084777:1177076-1177238:+	ACA1_295580	Solute carrier family protein [Source:UniProtKB/TrEMBL;Acc:L8HJT6]	0	0.465205	0.465205	3.29E-07
6h	ACA1_024040/clean/scf7180000084440:15786-15919:-	ACA1_024040	WD_REPEATS_REGION domain-containing protein [Source:UniProtKB/TrEMBL;Acc:L8GI27]	0.00211908	0.467062667	0.464943586	1.18E-08
6h	ACA1_237860/clean/scf7180000084763:33441-33566:+	ACA1_237860	Glycerophosphodiester phosphodiesterase family protein [Source:UniProtKB/TrEMBL;Acc:L8GKN2]	0.001550387	0.466324333	0.464773947	3.34E-11
6h	ACA1_310500/clean/scf7180000083773:11761-11822:+	ACA1_310500	hypothetical protein	0.004901967	0.469477	0.464575033	3.01E-08
6h	ACA1_054930/clean/scf7180000084594:607466-607531:+	ACA1_054930	Zinc finger, zz type domain containing protein [Source:UniProtKB/TrEMBL;Acc:L8H689]	0.002313897	0.466531667	0.46421777	6.11E-12
6h	ACA1_368700/clean/scf7180000084664:140703-140789:-	ACA1_368700	hypothetical protein	0	0.464212	0.464212	7.70E-10
6h	ACA1_090110/clean/scf7180000084639:437754-437863:+	ACA1_090110	TBC domain containing protein [Source:UniProtKB/TrEMBL;Acc:L8GVT7]	0.00825957	0.472443333	0.464183763	1.63E-06
6h	ACA1_262360/clean/scf7180000084770:36839-36919:-	ACA1_262360	Methyltransf_11 domain-containing protein [Source:UniProtKB/TrEMBL;Acc:L8H399]	0.002092082	0.466222	0.464129918	1.97E-18
6h	ACA1_203750/clean/scf7180000084754:204756-204852:-	ACA1_203750	PH domain-containing protein [Source:UniProtKB/TrEMBL;Acc:L8GTJ3]	0.00552185	0.469623667	0.464101817	8.85E-10

6h	ACA1_288510/clean/scf7180000084777:228020-228088:+	ACA1_288510	SNF2 family Nterminal domain containing protein [Source:UniProtKB/TrEMBL;Acc:L8HIW8]	0.001824153	0.465884	0.464059847	1.37E-08
6h	ACA1_234290/clean/scf7180000084762:261484-261542:-	ACA1_234290	Deacetylase sirtuin-type domain-containing protein [Source:UniProtKB/TrEMBL;Acc:L8H3J1]	0.276730333	0.740725	0.463994667	0.00118458
6h	ACA1_109710/clean/scf7180000084663:5198-5319:+	ACA1_109710	FYVE zinc finger domain containing protein [Source:UniProtKB/TrEMBL;Acc:L8HIF7]	0.022632453	0.486472333	0.46383988	1.42E-09
6h	ACA1_271540/clean/scf7180000084771:64133-64351:+	ACA1_271540	Leucine rich repeat domain containing protein [Source:UniProtKB/TrEMBL;Acc:L8GNS6]	0.004689067	0.468435	0.463745933	2.22E-07
6h	ACA1_331370/clean/scf7180000084575:18605-18688:-	ACA1_331370	Succinylglutamate desuccinylase/aspartoacylase subfamily protein [Source:UniProtKB/TrEMBL;Acc:L8GSG2]	0.020045833	0.483685	0.463639167	1.87E-08
6h	ACA1_385760/clean/scf7180000084730:257493-257632:-	ACA1_385760	MSP domain-containing protein [Source:UniProtKB/TrEMBL;Acc:L8H8G8]	0.1186734	0.581979667	0.463306267	1.26E-07
6h	ACA1_257300/clean/scf7180000084769:148293-148395:-	ACA1_257300	CTP_transf_like domain-containing protein [Source:UniProtKB/TrEMBL;Acc:L8GEN2]	0	0.463263	0.463263	0.000104928
6h	ACA1_091690/clean/scf7180000084645:181380-181457:+	ACA1_091690	PolyA_pol domain-containing protein [Source:UniProtKB/TrEMBL;Acc:L8GIJ9]	0.000340413	0.463574333	0.46323392	1.11E-09
6h	ACA1_182870/clean/scf7180000084745:272977-273123:+	ACA1_182870	G-patch domain-containing protein [Source:UniProtKB/TrEMBL;Acc:L8H9D6]	0.0115361	0.474762667	0.463226567	2.43E-08
6h	ACA1_293730/clean/scf7180000084777:968202-968295:+	ACA1_293730	PB1 domain containing protein [Source:UniProtKB/TrEMBL;Acc:L8HJM7]	0.00679404	0.470019333	0.463225293	1.04E-38
6h	ACA1_066870/clean/scf7180000084600:67221-67435:-	ACA1_066870	Glucose-6-phosphate isomerase [Source:UniProtKB/TrEMBL;Acc:L8GRA3]	0.001084773	0.464293333	0.46320856	3.71E-13
6h	ACA1_071550/clean/scf7180000084603:989497-989584:-	ACA1_071550	F-box domain-containing protein [Source:UniProtKB/TrEMBL;Acc:L8HFM3]	0.056763267	0.519922333	0.463159067	3.68E-06
6h	ACA1_220060/clean/scf7180000084758:852998-853072:+	ACA1_220060	SAP domain-containing protein [Source:UniProtKB/TrEMBL;Acc:L8GQ17]	0.006745653	0.469867333	0.46312168	1.41E-17
6h	ACA1_146510/clean/scf7180000084713:37409-37488:-	ACA1_146510	hypothetical protein	0.001637447	0.464688333	0.463050887	1.18E-10
6h	ACA1_037730/clean/scf7180000084568:9598-9669:+	ACA1_037730	Transmembrane protein, putative [Source:UniProtKB/TrEMBL;Acc:L8GN13]	0	0.462950333	0.462950333	0.001211303
6h	ACA1_376300/clean/scf7180000084685:73528-73638:+	ACA1_376300	Phos_pyr_kin domain-containing protein [Source:UniProtKB/TrEMBL;Acc:L8HF92]	0.022650867	0.485529333	0.462878467	3.28E-13
6h	ACA1_106720/clean/scf7180000084660:110842-110956:-	ACA1_106720	CID domain-containing protein [Source:UniProtKB/TrEMBL;Acc:L8GQ94]	0.211513667	0.674372	0.462858333	9.58E-07
6h	ACA1_106600/clean/scf7180000084660:103308-103427:-	ACA1_106600	Protein kinase domain-containing protein [Source:UniProtKB/TrEMBL;Acc:L8GN97]	0.228578667	0.691406333	0.462827667	0.01765934
6h	ACA1_164720/clean/scf7180000084729:258583-258663:+	ACA1_164720	Autophagy-related protein 9 [Source:UniProtKB/TrEMBL;Acc:L8GU30]	0.014620957	0.477366	0.462745043	7.71E-09
6h	ACA1_091560/clean/scf7180000084645:151025-151111:+	ACA1_091560	UBP-type domain-containing protein [Source:UniProtKB/TrEMBL;Acc:L8GKE3]	0.0275064	0.490181667	0.462675267	2.24E-05
6h	ACA1_068500/clean/scf7180000084603:217752-217825:+	ACA1_068500	Eukaryotic family protein [Source:UniProtKB/TrEMBL;Acc:L8HFQ5]	0.036138767	0.498799667	0.4626609	1.48E-08
6h	ACA1_285650/clean/scf7180000084776:1019584-1019664:-	ACA1_285650	G-patch domain-containing protein [Source:UniProtKB/TrEMBL;Acc:L8H9W2]	0.0141332	0.476781667	0.462648467	3.89E-06
6h	ACA1_159160/clean/scf7180000084724:349201-349286:-	ACA1_159160	hypothetical protein	0.016959067	0.479564	0.462604933	2.19E-08
6h	ACA1_175470/clean/scf7180000084741:966160-966235:-	ACA1_175470	Endo/exonuclease/phosphatase domain-containing protein [Source:UniProtKB/TrEMBL;Acc:L8HI27]	0	0.46255	0.46255	1.87E-09
6h	ACA1_171500/clean/scf7180000084741:121490-121558:-	ACA1_171500	Uridine-cytidine kinase [Source:UniProtKB/TrEMBL;Acc:L8HJM4]	0.00452868	0.467005	0.46247632	1.68E-09
6h	ACA1_323430/clean/scf7180000084468:21742-21820:+	ACA1_323430	SnoaL family polyketide cyclase [Source:UniProtKB/TrEMBL;Acc:L8H0W6]	0.002795747	0.465226667	0.46243092	9.79E-20
6h	ACA1_107600/clean/scf7180000084660:227603-227689:+	ACA1_107600	Mitochondrial chaperone bcs1, putative [Source:UniProtKB/TrEMBL;Acc:L8GND8]	0.000135593	0.462561	0.462425407	7.33E-11
6h	ACA1_060530/clean/scf7180000084599:702702-702838:+	ACA1_060530	TPR_REGION domain-containing protein [Source:UniProtKB/TrEMBL;Acc:L8GYY8]	0.119304333	0.581599667	0.462295333	6.21E-06
6h	ACA1_216300/clean/scf7180000084758:461309-461450:+	ACA1_216300	Ras family protein [Source:UniProtKB/TrEMBL;Acc:L8GR60]	0.002865357	0.465045333	0.462179977	9.46E-08
6h	ACA1_178270/clean/scf7180000084743:194715-194799:+	ACA1_178270	Rasrelated protein O-RAL, putative [Source:UniProtKB/TrEMBL;Acc:L8GU86]	0.029834033	0.491985667	0.462151633	6.19E-14
6h	ACA1_203990/clean/scf7180000084754:244146-244224:-	ACA1_203990	hypothetical protein	0.007803933	0.469908	0.462104067	1.50E-08
6h	ACA1_218470/clean/scf7180000084758:663155-663259:-	ACA1_218470	Rasrelated protein Rab-21, putative [Source:UniProtKB/TrEMBL;Acc:L8GRC8]	0.011800033	0.473853333	0.4620533	1.47E-16
6h	ACA1_071890/clean/scf7180000084603:1096037-1096133:-	ACA1_071890	FHA domain containing protein [Source:UniProtKB/TrEMBL;Acc:L8HEB4]	0	0.462039667	0.462039667	3.40E-06
6h	ACA1_369090/clean/scf7180000084664:240241-240305:-	ACA1_369090	2hydroxyacyl-CoA lyase 1, putative [Source:UniProtKB/TrEMBL;Acc:L8GZ11]	0.005067987	0.467032333	0.461964347	3.61E-08
6h	ACA1_063560/clean/scf7180000084599:1449613-1449716:+	ACA1_063560	hypothetical protein	0.001200033	0.463098333	0.4618983	1.25E-11
6h	ACA1_063970/clean/scf7180000084599:1548446-1548534:-	ACA1_063970	SH3 domain containing protein [Source:UniProtKB/TrEMBL;Acc:L8GYN6]	0	0.461869333	0.461869333	2.96E-14
6h	ACA1_185170/clean/scf7180000084746:80720-80790:+	ACA1_185170	Imidazoleglycerol-phosphate dehydratase [Source:UniProtKB/TrEMBL;Acc:L8H477]	0.005182027	0.466981667	0.46179964	1.02E-14
6h	ACA1_163160/clean/scf7180000084726:93400-93498:-	ACA1_163160	Ankyrin repeat-containing protein [Source:UniProtKB/TrEMBL;Acc:L8GXX1]	0	0.461786333	0.461786333	0.000978096
6h	ACA1_378010/clean/scf7180000084711:163036-163122:+	ACA1_378010	JmjC domain-containing protein [Source:UniProtKB/TrEMBL;Acc:L8GRT4]	0.0045662	0.466105667	0.461539467	7.57E-09
6h	ACA1_072290/clean/scf7180000084603:1191686-1191771:-	ACA1_072290	Endo/exonuclease/phosphatase domain-containing protein [Source:UniProtKB/TrEMBL;Acc:L8HEG9]	0.018518533	0.479980333	0.4614618	0.000174875
6h	ACA1_023890/clean/scf7180000084436:33024-33091:-	ACA1_023890	SAM domain-containing protein [Source:UniProtKB/TrEMBL;Acc:L8GSK7]	0.001808407	0.463194	0.461385593	2.11E-10
6h	ACA1_282510/clean/scf7180000084776:697014-697084:+	ACA1_282510	hypothetical protein	0	0.461346333	0.461346333	1.76E-07
6h	ACA1_125950/clean/scf7180000084693:6024-6098:+	ACA1_125950	AB hydrolase-1 domain-containing protein [Source:UniProtKB/TrEMBL;Acc:L8HCR9]	0.003708781	0.464886333	0.461177552	5.81E-09
6h	ACA1_145180/clean/scf7180000084710:53470-53557:-	ACA1_145180	F-box domain-containing protein [Source:UniProtKB/TrEMBL;Acc:L8GCU3]	0.00926137	0.470426333	0.461164963	6.41E-06
6h	ACA1_366090/clean/scf7180000084641:346529-346595:-	ACA1_366090	RNA polymerase III subunit C3 [Source:UniProtKB/TrEMBL;Acc:L8GML8]	0.005291	0.466454333	0.461163333	5.55E-07
6h	ACA1_173790/clean/scf7180000084741:520498-520597:+	ACA1_173790	Nicotinamide-nucleotide adenylyltransferase [Source:UniProtKB/TrEMBL;Acc:L8HK06]	0.01071031	0.471836	0.46112569	4.71E-11
6h	ACA1_184420/clean/scf7180000084745:639596-639680:+	ACA1_184420	Cathepsin Llike proteinase [Source:UniProtKB/TrEMBL;Acc:L8H8J9]	0.001041515	0.462141	0.461099485	1.82E-13
6h	ACA1_321060/clean/scf7180000084435:31332-31425:-	ACA1_321060	hypothetical protein	0.0128518	0.473909333	0.461057533	1.43E-11
6h	ACA1_012980/clean/scf7180000083742:15172-15248:-	ACA1_012980	Endo/exonuclease/phosphatase domain-containing protein [Source:UniProtKB/TrEMBL;Acc:L8H4W5]	0.040109567	0.501065667	0.4609561	8.52E-10
6h	ACA1_060260/clean/scf7180000084599:630467-630638:-	ACA1_060260	RFX-type winged-helix domain-containing protein [Source:UniProtKB/TrEMBL;Acc:L8GVN2]	0.322204333	0.783148667	0.460944333	2.20E-05
6h	ACA1_315060/clean/scf7180000083887:26134-26237:-	ACA1_315060	AAA domain-containing protein [Source:UniProtKB/TrEMBL;Acc:L8GZM9]	0.004400887	0.465199333	0.460798447	2.17E-27
6h	ACA1_204500/clean/scf7180000084754:293440-293535:+	ACA1_204500	hypothetical protein	0.00136692	0.462029	0.46066208	3.07E-11
6h	ACA1_142580/clean/scf7180000084708:186949-187071:-	ACA1_142580	unspecified product	0.097358233	0.557871333	0.4605131	0.000257442
6h	ACA1_364750/clean/scf7180000084641:190220-190277:-	ACA1_364750	UBP-type domain-containing protein [Source:UniProtKB/TrEMBL;Acc:L8GLN3]	0.0419421	0.502286667	0.460344567	7.52E-13
6h	ACA1_190770/clean/scf7180000084748:171322-171412:-	ACA1_190770	Rho-GAP domain-containing protein [Source:UniProtKB/TrEMBL;Acc:L8GE16]	0.006582637	0.466880333	0.460297697	4.79E-10
6h	ACA1_258290/clean/scf7180000084769:221792-221855:+	ACA1_258290	hypothetical protein	0.055434333	0.515561	0.460126667	4.99E-07
6h	ACA1_255670/clean/scf7180000084768:542882-543003:+	ACA1_255670	Aminotran_1_2 domain-containing protein [Source:UniProtKB/TrEMBL;Acc:L8HDH4]	0.012004373	0.472121333	0.46011696	7.05E-22
6h	ACA1_172250/clean/scf7180000084741:285905-285972:-	ACA1_172250	Leucine rich repeat domain containing protein [Source:UniProtKB/TrEMBL;Acc:L8HHK1]	0.025641033	0.485742667	0.460101633	0.000162139
6h	ACA1_179130/clean/scf7180000084743:306584-306661:+	ACA1_179130	Protein kinase domain containing protein [Source:UniProtKB/TrEMBL;Acc:L8GTH0]	0.009803933	0.469744333	0.4599404	4.16E-06
6h	ACA1_237810/clean/scf7180000084763:20822-20921:-	ACA1_237810	hypothetical protein	0.02054784	0.480416667	0.459868827	5.90E-12
6h	ACA1_171940/clean/scf7180000084741:205428-205505:-	ACA1_171940	hypothetical protein	0.043729333	0.503526667	0.459797333	0.000141072
6h	ACA1_322160/clean/scf7180000084459:22404-22485:+	ACA1_322160	AB hydrolase-1 domain-containing protein [Source:UniProtKB/TrEMBL;Acc:L8GMT9]	0.006243947	0.466029333	0.459785387	1.11E-12
6h	ACA1_370080/clean/scf7180000084664:435113-435201:-	ACA1_370080	ATPdependent RNA helicase Ddx1, putative [Source:UniProtKB/TrEMBL;Acc:L8GZ36]	0.0065177	0.466107667	0.459589967	1.61E-06
6h	ACA1_301690/clean/scf7180000083222:18938-19055:-	ACA1_301690	MoCF_biosynth domain-containing protein [Source:UniProtKB/TrEMBL;Acc:L8HJW0]	0.004657013	0.464190333	0.45953332	2.18E-18
6h	ACA1_069770/clean/scf7180000084603:553152-553260:+	ACA1_069770	Tyrosine--tRNA ligase [Source:UniProtKB/TrEMBL;Acc:L8HDT0]	0.002193937	0.461700333	0.459506397	1.86E-11
6h	ACA1_384570/clean/scf7180000084730:53394-53484:-	ACA1_384570	Gelsolin repeat-containing protein [Source:UniProtKB/TrEMBL;Acc:L8H918]	0.002949853	0.462317	0.459367147	7.66E-09
6h	ACA1_073290/clean/scf7180000084603:1389263-1389338:-	ACA1_073290	EF hand domain containing protein [Source:UniProtKB/TrEMBL;Acc:L8HEV1]	0	0.459330667	0.459330667	4.83E-11
6h	ACA1_151100/clean/scf7180000084716:56953-57080:-	ACA1_151100	NFACT-R_1 domain-containing protein [Source:UniProtKB/TrEMBL;Acc:L8H219]	0.002326167	0.461633667	0.4593075	4.83E-09
6h	ACA1_278060/clean/scf7180000084776:164731-164790:-	ACA1_278060	hypothetical protein	0.023940333	0.483246	0.459305667	8.52E-13
6h	ACA1_234790/clean/scf7180000084762:318697-318794:-	ACA1_234790	PPM-type phosphatase domain-containing protein [Source:UniProtKB/TrEMBL;Acc:L8H3L1]	0.1022398	0.561542667	0.459302867	1.35E-10
6h	ACA1_315780/clean/scf7180000083943:7371-7450:+	ACA1_315780	hypothetical protein	0.021666653	0.480524	0.458857347	1.82E-11
6h	ACA1_331960/clean/scf7180000084578:52210-52297:-	ACA1_331960	Zinc finger, c2h2 type domain containing protein [Source:UniProtKB/TrEMBL;Acc:L8HD24]	0.0564089	0.515079333	0.458670433	0.00017144
6h	ACA1_372600/clean/scf7180000084679:23425-23510:-	ACA1_372600	F-box domain-containing protein [Source:UniProtKB/TrEMBL;Acc:L8GJA4]	0	0.458489	0.458489	5.46E-10
6h	ACA1_211500/clean/scf7180000084758:89959-90037:+	ACA1_211500	RAB5B protein, putative [Source:UniProtKB/TrEMBL;Acc:L8GS95]	0.020833333	0.479203667	0.458370333	0.000440073
6h	ACA1_070540/clean/scf7180000084603:719726-719828:+	ACA1_070540	Rab-GAP TBC domain-containing protein [Source:UniProtKB/TrEMBL;Acc:L8HG76]	0	0.458359	0.458359	2.46E-06
6h	ACA1_074450/clean/scf7180000084608:15069-15154:-	ACA1_074450	Galactokinase [Source:UniProtKB/TrEMBL;Acc:L8HGN2]	0.006068167	0.464339667	0.4582715	9.18E-14
6h	ACA1_389130/clean/scf7180000084733:207304-207388:+	ACA1_389130	BTB/POZ domain containing protein [Source:UniProtKB/TrEMBL;Acc:L8GDV1]	0.005726973	0.463922667	0.458195693	3.98E-13
6h	ACA1_199520/clean/scf7180000084753:252733-252844:+	ACA1_199520	hypothetical protein	0	0.458168	0.458168	9.98E-11
6h	ACA1_129510/clean/scf7180000084698:18998-19072:+	ACA1_129510	Hydrolase_4 domain-containing protein [Source:UniProtKB/TrEMBL;Acc:L8GRQ5]	0.002923977	0.460995	0.458071023	1.71E-09
6h	ACA1_058210/clean/scf7180000084599:272304-272396:-	ACA1_058210	F-box domain-containing protein [Source:UniProtKB/TrEMBL;Acc:L8GVZ0]	0.013865067	0.471822333	0.457957267	2.94E-07
6h	ACA1_398930/clean/scf7180000084759:253623-253717:+	ACA1_398930	hypothetical protein	0.025694	0.483546333	0.457852333	1.01E-09
6h	ACA1_061460/clean/scf7180000084599:933776-933853:-	ACA1_061460	Leucine rich repeat domain containing protein [Source:UniProtKB/TrEMBL;Acc:L8GZ73]	0.02094193	0.478792333	0.457850403	3.71E-06
6h	ACA1_116630/clean/scf7180000084669:815788-815895:+	ACA1_116630	BPL/LPL catalytic domain-containing protein [Source:UniProtKB/TrEMBL;Acc:L8H6V8]	0.0514301	0.509246	0.4578159	3.29E-08
6h	ACA1_264460/clean/scf7180000084770:294506-294622:-	ACA1_264460	GTPbinding protein [Source:UniProtKB/TrEMBL;Acc:L8H231]	0	0.457728	0.457728	3.05E-11
6h	ACA1_369850/clean/scf7180000084664:395768-395843:-	ACA1_369850	ATPase with chaperone activity, putative [Source:UniProtKB/TrEMBL;Acc:L8GZ67]	0.002440727	0.460141333	0.457700607	2.46E-08
6h	ACA1_368760/clean/scf7180000084664:155315-155383:-	ACA1_368760	hypothetical protein	0.063086167	0.520785333	0.457699167	0.000155074
6h	ACA1_368170/clean/scf7180000084664:71184-71347:+	ACA1_368170	Actin-related protein 2/3 complex subunit 5 [Source:UniProtKB/TrEMBL;Acc:L8H192]	0.00359329	0.461266667	0.457673377	8.44E-33
6h	ACA1_161750/clean/scf7180000084726:19838-19940:-	ACA1_161750	hypothetical protein	0.02146599	0.479019333	0.457553343	8.01E-09
6h	ACA1_063800/clean/scf7180000084599:1506577-1506664:-	ACA1_063800	B30.2/SPRY domain-containing protein [Source:UniProtKB/TrEMBL;Acc:L8GZV1]	0.015390167	0.472865333	0.457475167	1.74E-10
6h	ACA1_103990/clean/scf7180000084653:63287-63375:-	ACA1_103990	Ras-GAP domain-containing protein [Source:UniProtKB/TrEMBL;Acc:L8GFL9]	0.009784193	0.467173	0.457388807	6.97E-12
6h	ACA1_248570/clean/scf7180000084766:85768-85851:-	ACA1_248570	Phenylalanine-tRNA ligase [Source:UniProtKB/TrEMBL;Acc:L8GY13]	0.005782187	0.463126333	0.457344147	4.54E-08
6h	ACA1_287950/clean/scf7180000084777:131726-131837:-	ACA1_287950	hypothetical protein	0.013759523	0.471098	0.457338477	3.91E-08
6h	ACA1_053270/clean/scf7180000084594:240479-240550:+	ACA1_053270	Nudix hydrolase domain-containing protein [Source:UniProtKB/TrEMBL;Acc:L8H547]	0.006545013	0.463863333	0.45731832	1.21E-07
6h	ACA1_218090/clean/scf7180000084758:623504-623626:-	ACA1_218090	AB hydrolase-1 domain-containing protein [Source:UniProtKB/TrEMBL;Acc:L8GSU3]	0.0105263	0.467825333	0.457299033	1.68E-07
6h	ACA1_298660/clean/scf7180000082992:16141-16268:+	ACA1_298660	Bromo domain-containing protein [Source:UniProtKB/TrEMBL;Acc:L8H1K3]	0.002383677	0.459437	0.457053323	2.83E-08
6h	ACA1_279380/clean/scf7180000084776:374375-374432:+	ACA1_279380	4HBT domain-containing protein [Source:UniProtKB/TrEMBL;Acc:L8H926]	0.206000333	0.663029667	0.457029333	0.000102327
6h	ACA1_095670/clean/scf7180000084645:877336-877423:-	ACA1_095670	FAD-binding PCMH-type domain-containing protein [Source:UniProtKB/TrEMBL;Acc:L8GIT7]	0.007584567	0.464596333	0.457011767	1.71E-08
6h	ACA1_127620/clean/scf7180000084696:36475-36564:+	ACA1_127620	Fe2OG dioxygenase domain-containing protein [Source:UniProtKB/TrEMBL;Acc:L8GGE8]	0.018464716	0.475434	0.456969284	1.17E-05
6h	ACA1_365420/clean/scf7180000084641:277162-277216:+	ACA1_365420	HEAT repeat domain containing protein [Source:UniProtKB/TrEMBL;Acc:L8GMB6]	0.253264333	0.710229333	0.456965	0.000245132
6h	ACA1_174020/clean/scf7180000084741:578701-578845:+	ACA1_174020	ANK_REP_REGION domain-containing protein [Source:UniProtKB/TrEMBL;Acc:L8HGR2]	0.040150033	0.497050333	0.4569003	2.14E-10
6h	ACA1_070410/clean/scf7180000084603:669575-669737:-	ACA1_070410	L-type lectin-like domain-containing protein [Source:UniProtKB/TrEMBL;Acc:L8HFA0]	0.096782767	0.553681333	0.456898567	5.04E-08
6h	ACA1_193140/clean/scf7180000084749:195982-196056:+	ACA1_193140	Metallophosphoesterase [Source:UniProtKB/TrEMBL;Acc:L8GQV5]	0.016468233	0.473344667	0.456876433	4.67E-07
6h	ACA1_264330/clean/scf7180000084770:240681-240784:+	ACA1_264330	Abhydrolase_3 domain-containing protein [Source:UniProtKB/TrEMBL;Acc:L8H479]	0.002654124	0.459253	0.456598876	8.14E-10
6h	ACA1_250030/clean/scf7180000084767:27299-27396:-	ACA1_250030	C2 NT-type domain-containing protein [Source:UniProtKB/TrEMBL;Acc:L8HC94]	0.002668607	0.459225333	0.456556727	2.32E-08
6h	ACA1_233870/clean/scf7180000084762:173852-173957:-	ACA1_233870	hypothetical protein	0.024170127	0.480673667	0.45650354	6.54E-08
6h	ACA1_201590/clean/scf7180000084753:596202-596275:-	ACA1_201590	ANK_REP_REGION domain-containing protein [Source:UniProtKB/TrEMBL;Acc:L8H5P8]	0	0.456288333	0.456288333	3.60E-05
6h	ACA1_091400/clean/scf7180000084645:107462-107540:+	ACA1_091400	hypothetical protein	0.018448767	0.474691	0.456242233	2.00E-09
6h	ACA1_171240/clean/scf7180000084741:51471-51565:-	ACA1_171240	Ribosomal_L7Ae domain-containing protein [Source:UniProtKB/TrEMBL;Acc:L8HJI5]	0.017329117	0.473531667	0.45620255	1.11E-06
6h	ACA1_220110/clean/scf7180000084758:865091-865175:-	ACA1_220110	hypothetical protein	0.007778003	0.463942333	0.45616433	1.71E-07
6h	ACA1_062120/clean/scf7180000084599:1124119-1124212:+	ACA1_062120	RING-type domain-containing protein [Source:UniProtKB/TrEMBL;Acc:L8GWU5]	0.019478733	0.475564667	0.456085933	2.02E-12
6h	ACA1_127620/clean/scf7180000084696:36254-36326:+	ACA1_127620	Fe2OG dioxygenase domain-containing protein [Source:UniProtKB/TrEMBL;Acc:L8GGE8]	0.045921053	0.501962333	0.456041281	8.85E-07
6h	ACA1_163990/clean/scf7180000084729:126499-126571:-	ACA1_163990	Glycosyl hydrolases family 32 superfamily protein [Source:UniProtKB/TrEMBL;Acc:L8GRF2]	0.020354367	0.476245333	0.455890967	4.38E-06
6h	ACA1_039640/clean/scf7180000084570:10128-10275:+	ACA1_039640	hypothetical protein	0.0148549	0.470614333	0.455759433	5.22E-09
6h	ACA1_278160/clean/scf7180000084776:181450-181523:-	ACA1_278160	hypothetical protein	0.040994767	0.496587	0.455592233	1.20E-21
6h	ACA1_224370/clean/scf7180000084760:181943-182039:-	ACA1_224370	Exportin 1like protein [Source:UniProtKB/TrEMBL;Acc:L8GTX4]	0.00187204	0.457464	0.45559196	2.06E-08
6h	ACA1_188500/clean/scf7180000084747:173095-173201:+	ACA1_188500	CRAL/TRIO domain containing protein [Source:UniProtKB/TrEMBL;Acc:L8GSH3]	0.001532157	0.457075333	0.455543177	1.78E-09
6h	ACA1_221090/clean/scf7180000084758:997413-997496:-	ACA1_221090	Peptidyl-prolyl cis-trans isomerase [Source:UniProtKB/TrEMBL;Acc:L8GRR0]	0.002341011	0.457804	0.455462989	3.16E-10
6h	ACA1_095220/clean/scf7180000084645:816828-816928:-	ACA1_095220	Protein kinase domain-containing protein [Source:UniProtKB/TrEMBL;Acc:L8GIR4]	0.00344401	0.458849667	0.455405657	2.65E-08
6h	ACA1_115860/clean/scf7180000084669:690605-690703:-	ACA1_115860	NOT2 / NOT3 / NOT5 family protein [Source:UniProtKB/TrEMBL;Acc:L8H4R3]	0.001086629	0.456334667	0.455248038	1.73E-11
6h	ACA1_175110/clean/scf7180000084741:867082-867169:-	ACA1_175110	hypothetical protein	0.001530237	0.456777333	0.455247097	1.54E-07
6h	ACA1_109740/clean/scf7180000084663:15113-15200:-	ACA1_109740	Myblike DNA-binding domain containing protein [Source:UniProtKB/TrEMBL;Acc:L8HJY9]	0	0.45519	0.45519	1.20E-09
6h	ACA1_247260/clean/scf7180000084763:659906-659981:+	ACA1_247260	SET domain-containing protein [Source:UniProtKB/TrEMBL;Acc:L8GKH1]	0.018054157	0.47319	0.455135843	2.69E-13
6h	ACA1_320550/clean/scf7180000084416:10044-10177:-	ACA1_320550	Proline iminopeptidase [Source:UniProtKB/TrEMBL;Acc:L8GWD8]	0.001869128	0.456746667	0.454877538	4.42E-27
6h	ACA1_106800/clean/scf7180000084660:134786-134866:+	ACA1_106800	EF hand domain containing protein [Source:UniProtKB/TrEMBL;Acc:L8GNA6]	0.013479293	0.468288	0.454808707	1.32E-12
6h	ACA1_091750/clean/scf7180000084645:196695-196827:-	ACA1_091750	hypothetical protein	0.006714363	0.461515667	0.454801303	9.61E-09
6h	ACA1_364750/clean/scf7180000084641:189597-189670:-	ACA1_364750	UBP-type domain-containing protein [Source:UniProtKB/TrEMBL;Acc:L8GLN3]	0.0077448	0.462535333	0.454790533	1.95E-16
6h	ACA1_153680/clean/scf7180000084720:153573-153655:-	ACA1_153680	Amz2 protein [Source:UniProtKB/TrEMBL;Acc:L8HHH0]	0	0.454783	0.454783	0.018381406
6h	ACA1_266530/clean/scf7180000084770:688265-688362:-	ACA1_266530	UBA/TSN domain containing protein [Source:UniProtKB/TrEMBL;Acc:L8H2F1]	0.002634987	0.457416	0.454781013	3.37E-09
6h	ACA1_306690/clean/scf7180000083668:5730-5853:-	ACA1_306690	Gammasecretase subunit APH-1B, putative [Source:UniProtKB/TrEMBL;Acc:L8GQE8]	0.00518359	0.459794667	0.454611077	1.67E-08
6h	ACA1_338910/clean/scf7180000084607:14148-14235:-	ACA1_338910	hypothetical protein	0.004174602	0.458759667	0.454585065	1.01E-08
6h	ACA1_036210/clean/scf7180000084565:41915-42014:-	ACA1_036210	WD_REPEATS_REGION domain-containing protein [Source:UniProtKB/TrEMBL;Acc:L8HCG6]	0	0.454516667	0.454516667	7.44E-07
6h	ACA1_273770/clean/scf7180000084773:52999-53074:-	ACA1_273770	hypothetical protein	0.009111723	0.463355	0.454243277	1.03E-47
6h	ACA1_101310/clean/scf7180000084649:44350-44425:+	ACA1_101310	VIT domain-containing protein [Source:UniProtKB/TrEMBL;Acc:L8GG72]	0	0.454221	0.454221	6.88E-08
6h	ACA1_069160/clean/scf7180000084603:369418-369491:+	ACA1_069160	Serine/threonineprotein kinase SNF1-like protein [Source:UniProtKB/TrEMBL;Acc:L8HDL9]	0.022685023	0.476888	0.454202977	9.25E-06
6h	ACA1_309360/clean/scf7180000083758:12370-12449:-	ACA1_309360	hypothetical protein	0	0.45411	0.45411	0.000109312
6h	ACA1_389150/clean/scf7180000084733:212916-212987:+	ACA1_389150	Tasp1 protein [Source:UniProtKB/TrEMBL;Acc:L8GEC7]	0	0.454098333	0.454098333	3.46E-10
6h	ACA1_331780/clean/scf7180000084578:26527-26614:-	ACA1_331780	BRCA1 C Terminus (BRCT) domain containing protein [Source:UniProtKB/TrEMBL;Acc:L8HE43]	0.03150268	0.485278	0.45377532	1.49E-09
6h	ACA1_306840/clean/scf7180000083668:19565-19636:+	ACA1_306840	RTP1_C1 domain-containing protein [Source:UniProtKB/TrEMBL;Acc:L8GQF6]	0.0282738	0.482032667	0.453758867	3.47E-05

6h	ACA1_362670/clean/scf7180000084624:135269-135367:+	ACA1_362670	Rieske domain-containing protein [Source:UniProtKB/TrEMBL;Acc:L8GHW3]	0.000554903	0.454270667	0.453715763	1.26E-12
6h	ACA1_166970/clean/scf7180000084736:43145-43234:+	ACA1_166970	Poly(ADP-ribose) polymerase catalytic domain containing protein [Source:UniProtKB/TrEMBL;Acc:L8H0T4]	0.0421343	0.495812667	0.453678367	1.84E-06
6h	ACA1_200050/clean/scf7180000084753:381969-382055:-	ACA1_200050	Poly [ADP-ribose] polymerase [Source:UniProtKB/TrEMBL;Acc:L8H4Q2]	0.00289855	0.456539	0.45364045	3.60E-08
6h	ACA1_400300/clean/scf7180000084775:142790-142876:+	ACA1_400300	UBIQUITIN_CONJUGAT_2 domain-containing protein [Source:UniProtKB/TrEMBL;Acc:L8GGI3]	0	0.453551333	0.453551333	1.26E-06
6h	ACA1_074770/clean/scf7180000084608:83126-83215:-	ACA1_074770	Vps52 / Sac2 family protein [Source:UniProtKB/TrEMBL;Acc:L8HFG7]	0	0.453548667	0.453548667	2.68E-10
6h	ACA1_127880/clean/scf7180000084697:8424-8483:+	ACA1_127880	Bis(5'-adenosyl)-triphosphatase [Source:UniProtKB/TrEMBL;Acc:L8GUT1]	0	0.453504	0.453504	1.55E-07
6h	ACA1_326760/clean/scf7180000084510:30181-30276:+	ACA1_326760	hypothetical protein	0.02883816	0.482262333	0.453424173	4.78E-05
6h	ACA1_042010/clean/scf7180000084573:34147-34218:-	ACA1_042010	Endoalpha-1,2-mannosidase-like protein [Source:UniProtKB/TrEMBL;Acc:L8GUJ3]	0.0451645	0.498554667	0.453390167	3.82E-07
6h	ACA1_173980/clean/scf7180000084741:565443-565557:-	ACA1_173980	OTU domain-containing protein [Source:UniProtKB/TrEMBL;Acc:L8HHS8]	0	0.453376	0.453376	3.29E-10
6h	ACA1_127880/clean/scf7180000084697:9029-9132:+	ACA1_127880	Bis(5'-adenosyl)-triphosphatase [Source:UniProtKB/TrEMBL;Acc:L8GUT1]	0.017704033	0.471050333	0.4533463	1.74E-05
6h	ACA1_325610/clean/scf7180000084495:50712-50792:-	ACA1_325610	Protein kinase domain containing protein [Source:UniProtKB/TrEMBL;Acc:L8H900]	0	0.453327667	0.453327667	1.82E-11
6h	ACA1_282440/clean/scf7180000084776:680488-680556:+	ACA1_282440	hypothetical protein	0.0299013	0.483221667	0.453320367	1.58E-07
6h	ACA1_382290/clean/scf7180000084721:23618-23680:+	ACA1_382290	hypothetical protein	0.004961667	0.458253333	0.453291667	0.000832726
6h	ACA1_331590/clean/scf7180000084578:6452-6527:+	ACA1_331590	ATPdependent DNA helicase, RecQ subfamily protein [Source:UniProtKB/TrEMBL;Acc:L8HBM3]	0.0196708	0.472949	0.4532782	9.32E-08
6h	ACA1_336100/clean/scf7180000084593:24490-24579:-	ACA1_336100	Ribosomal protein L12, putative [Source:UniProtKB/TrEMBL;Acc:L8H1R0]	0.0380823	0.491318333	0.453236033	1.64E-07
6h	ACA1_184890/clean/scf7180000084746:64236-64321:-	ACA1_184890	hypothetical protein	0.120071	0.573264667	0.453193667	1.18E-05
6h	ACA1_157940/clean/scf7180000084724:155695-155765:-	ACA1_157940	Histidine acid phosphatase superfamily protein [Source:UniProtKB/TrEMBL;Acc:L8H9Z4]	0.00419715	0.457331333	0.453134183	1.82E-18
6h	ACA1_172970/clean/scf7180000084741:373844-373903:-	ACA1_172970	Chloride channel protein [Source:UniProtKB/TrEMBL;Acc:L8HHM6]	0.017170913	0.470029667	0.452858753	4.22E-09
6h	ACA1_389490/clean/scf7180000084733:267243-267337:-	ACA1_389490	RRM domain-containing protein [Source:UniProtKB/TrEMBL;Acc:L8GES5]	0.007580657	0.460379333	0.452798677	1.55E-07
6h	ACA1_184390/clean/scf7180000084745:633021-633123:+	ACA1_184390	hypothetical protein	0.017552867	0.470172333	0.452619467	5.23E-06
6h	ACA1_058140/clean/scf7180000084599:258784-258863:+	ACA1_058140	Sarp-3, putative [Source:UniProtKB/TrEMBL;Acc:L8GX72]	0.00300385	0.455468667	0.452464817	1.37E-06
6h	ACA1_203000/clean/scf7180000084754:114207-114285:+	ACA1_203000	PGM1_C domain-containing protein [Source:UniProtKB/TrEMBL;Acc:L8GW29]	0	0.452462333	0.452462333	2.01E-06
6h	ACA1_092000/clean/scf7180000084645:244529-244654:-	ACA1_092000	hypothetical protein	0.007767437	0.460200333	0.452432897	1.29E-08
6h	ACA1_264400/clean/scf7180000084770:258966-259061:+	ACA1_264400	hypothetical protein	0.0017069	0.454126333	0.452419433	2.81E-07
6h	ACA1_110100/clean/scf7180000084663:62398-62534:-	ACA1_110100	Protein kinase [Source:UniProtKB/TrEMBL;Acc:L8HK05]	0	0.452369667	0.452369667	1.22E-08
6h	ACA1_216320/clean/scf7180000084758:467825-467908:+	ACA1_216320	Homogentisate 1,2dioxygenase [Source:UniProtKB/TrEMBL;Acc:L8GQ83]	0.00274869	0.455093667	0.452344977	2.07E-14
6h	ACA1_190080/clean/scf7180000084748:122576-122659:-	ACA1_190080	CN hydrolase domain-containing protein [Source:UniProtKB/TrEMBL;Acc:L8GE63]	0.001428943	0.453748667	0.452319723	4.69E-09
6h	ACA1_173640/clean/scf7180000084741:500503-500596:+	ACA1_173640	ZnF_Rad18 domain-containing protein [Source:UniProtKB/TrEMBL;Acc:L8HK04]	0.034537333	0.486788	0.452250667	1.49E-06
6h	ACA1_065700/clean/scf7180000084599:1875275-1875408:+	ACA1_065700	Protein kinase domain-containing protein [Source:UniProtKB/TrEMBL;Acc:L8H039]	0.049017633	0.501220333	0.4522027	1.39E-11
6h	ACA1_296120/clean/scf7180000084777:1300741-1300872:+	ACA1_296120	hypothetical protein	0.002951813	0.45496	0.452008187	3.85E-08
6h	ACA1_301230/clean/scf7180000083203:5881-6002:-	ACA1_301230	hypothetical protein	0.0094467	0.461437667	0.451990967	3.83E-07
6h	ACA1_288770/clean/scf7180000084777:285800-285877:-	ACA1_288770	Leucine rich repeat domain containing protein [Source:UniProtKB/TrEMBL;Acc:L8HLA9]	0	0.45195	0.45195	1.25E-09
6h	ACA1_069190/clean/scf7180000084603:377964-378034:+	ACA1_069190	PfkB domain-containing protein [Source:UniProtKB/TrEMBL;Acc:L8HDR4]	0.003943002	0.455802333	0.451859331	4.55E-07
6h	ACA1_306870/clean/scf7180000083668:23219-23346:+	ACA1_306870	hypothetical protein	0.017328173	0.469181	0.451852827	1.61E-08
6h	ACA1_123360/clean/scf7180000084686:18125-18421:+	ACA1_123360	Zn(2)-C6 fungal-type domain-containing protein [Source:UniProtKB/TrEMBL;Acc:L8HF21]	0.019072813	0.470838667	0.451765853	6.66E-10
6h	ACA1_252200/clean/scf7180000084768:142514-142612:+	ACA1_252200	Saposin, putative [Source:UniProtKB/TrEMBL;Acc:L8HD22]	0.003857883	0.455612333	0.45175445	4.50E-11
6h	ACA1_236650/clean/scf7180000084762:466578-466803:+	ACA1_236650	Calciumbinding mitochondrial carrier protein [Source:UniProtKB/TrEMBL;Acc:L8H1J4]	0.005430603	0.45705	0.451619397	6.71E-17
6h	ACA1_140380/clean/scf7180000084707:68291-68376:+	ACA1_140380	Clu domain-containing protein [Source:UniProtKB/TrEMBL;Acc:L8GGQ7]	0.005521033	0.456947667	0.451426633	1.20E-05
6h	ACA1_034030/clean/scf7180000084561:7755-7856:+	ACA1_034030	RNA recognition motif domain containing protein [Source:UniProtKB/TrEMBL;Acc:L8GTW3]	0	0.451402	0.451402	1.32E-10
6h	ACA1_179970/clean/scf7180000084744:11779-11912:+	ACA1_179970	PPM-type phosphatase domain-containing protein [Source:UniProtKB/TrEMBL;Acc:L8GF73]	0.238789	0.690071333	0.451282333	2.88E-08
6h	ACA1_074100/clean/scf7180000084605:24405-24516:+	ACA1_074100	Fungal Zn(2)-Cys(6) binuclear cluster domain containing protein [Source:UniProtKB/TrEMBL;Acc:L8HLU8]	0.019877933	0.471093	0.451215067	1.60E-11
6h	ACA1_095690/clean/scf7180000084645:885411-885502:+	ACA1_095690	hypothetical protein	0.0062893	0.457469667	0.451180367	9.66E-08
6h	ACA1_259960/clean/scf7180000084769:388729-388913:+	ACA1_259960	Cystathionine beta-synthase [Source:UniProtKB/TrEMBL;Acc:L8GF87]	0.041636533	0.492769333	0.4511328	2.43E-13
6h	ACA1_110130/clean/scf7180000084663:70570-70717:-	ACA1_110130	MOSC domain-containing protein [Source:UniProtKB/TrEMBL;Acc:L8HKV4]	0.004697077	0.455735333	0.451038257	9.51E-21
6h	ACA1_165790/clean/scf7180000084732:83464-83565:-	ACA1_165790	hypothetical protein	0.015881803	0.466914	0.451032197	7.52E-13
6h	ACA1_144300/clean/scf7180000084709:198894-199012:-	ACA1_144300	RRM domain-containing protein [Source:UniProtKB/TrEMBL;Acc:L8HEU9]	0.00258398	0.453566667	0.450982687	2.51E-09
6h	ACA1_068670/clean/scf7180000084603:254788-254891:-	ACA1_068670	Dihydrolipoyl dehydrogenase [Source:UniProtKB/TrEMBL;Acc:L8HET6]	0.000327978	0.451242	0.450914022	3.13E-14
6h	ACA1_036340/clean/scf7180000084565:81580-81680:+	ACA1_036340	Serine hydroxymethyltransferase [Source:UniProtKB/TrEMBL;Acc:L8HBL5]	0.002727371	0.453634667	0.450907296	3.09E-12
6h	ACA1_321760/clean/scf7180000084455:14414-14492:-	ACA1_321760	Haloacid dehalogenaselike hydrolase domain containing protein [Source:UniProtKB/TrEMBL;Acc:L8GRZ2]	0.201333067	0.652084333	0.450751267	2.32E-05
6h	ACA1_089120/clean/scf7180000084639:324858-324951:-	ACA1_089120	hypothetical protein	0	0.450741667	0.450741667	2.82E-08
6h	ACA1_040880/clean/scf7180000084572:99099-99190:-	ACA1_040880	BAH domain-containing protein [Source:UniProtKB/TrEMBL;Acc:L8H0M7]	0.002349577	0.45309	0.450740423	8.67E-09
6h	ACA1_057410/clean/scf7180000084599:136140-136205:-	ACA1_057410	hypothetical protein	0	0.450716	0.450716	0.000474162
6h	ACA1_237270/clean/scf7180000084762:500332-500412:+	ACA1_237270	RNA 2'phosphotransferase, Tpt1/KptA domain containing protein [Source:UniProtKB/TrEMBL;Acc:L8H0V2]	0	0.450706667	0.450706667	1.69E-08
6h	ACA1_065190/clean/scf7180000084599:1811641-1811729:-	ACA1_065190	RBR-type E3 ubiquitin transferase [Source:UniProtKB/TrEMBL;Acc:L8GXL6]	0	0.450631	0.450631	3.03E-08
6h	ACA1_135150/clean/scf7180000084702:74989-75060:-	ACA1_135150	hypothetical protein	0.147222233	0.597764667	0.450542433	0.001812749
6h	ACA1_149500/clean/scf7180000084715:161323-161460:-	ACA1_149500	Palmitoyltransferase [Source:UniProtKB/TrEMBL;Acc:L8HC12]	0.129311333	0.579842333	0.450531	8.03E-05
6h	ACA1_150430/clean/scf7180000084716:8225-8304:+	ACA1_150430	Protein kinase domain-containing protein [Source:UniProtKB/TrEMBL;Acc:L8H2Z5]	0	0.450432667	0.450432667	0.000868529
6h	ACA1_257430/clean/scf7180000084769:152223-152293:-	ACA1_257430	Pyridoxamine 5'phosphate oxidase family superfamily protein [Source:UniProtKB/TrEMBL;Acc:L8GFR0]	0.027580133	0.477923667	0.450343533	3.92E-08
6h	ACA1_146440/clean/scf7180000084713:25683-25753:+	ACA1_146440	Methyltransf_11 domain-containing protein [Source:UniProtKB/TrEMBL;Acc:L8GU12]	0.001269777	0.451577	0.450307223	9.22E-11
6h	ACA1_090030/clean/scf7180000084639:418935-419041:-	ACA1_090030	Tcomplex protein 1, theta subunit [Source:UniProtKB/TrEMBL;Acc:L8GUN4]	0.001768347	0.451990333	0.450221987	7.63E-13
6h	ACA1_359690/clean/scf7180000084622:4827-4900:-	ACA1_359690	Rab-GAP TBC domain-containing protein [Source:UniProtKB/TrEMBL;Acc:L8HBR2]	0.00529493	0.455444	0.45014907	2.54E-12
6h	ACA1_376170/clean/scf7180000084685:40580-40657:-	ACA1_376170	Aa_trans domain-containing protein [Source:UniProtKB/TrEMBL;Acc:L8HFY8]	0.008322797	0.458284333	0.449961537	5.21E-09
6h	ACA1_264830/clean/scf7180000084770:384034-384157:-	ACA1_264830	hypothetical protein	0.0348628	0.484786	0.4499232	1.87E-06
6h	ACA1_040880/clean/scf7180000084572:100810-100890:-	ACA1_040880	BAH domain-containing protein [Source:UniProtKB/TrEMBL;Acc:L8H0M7]	0.0263158	0.476159333	0.449843533	1.59E-06
6h	ACA1_381580/clean/scf7180000084712:186079-186136:+	ACA1_381580	hypothetical protein	0.077140167	0.526938	0.449797833	2.87E-05
6h	ACA1_236320/clean/scf7180000084762:439077-439163:+	ACA1_236320	Cytochrome p450 superfamily protein [Source:UniProtKB/TrEMBL;Acc:L8H0Q4]	0.00583947	0.455374667	0.449535197	4.73E-14
6h	ACA1_201510/clean/scf7180000084753:577863-577923:+	ACA1_201510	Ubiquitin fusion degradation protein, putative [Source:UniProtKB/TrEMBL;Acc:L8H4X5]	0.057551733	0.507047667	0.449495933	4.95E-09
6h	ACA1_270200/clean/scf7180000084770:1076880-1076967:-	ACA1_270200	FYVE-type domain-containing protein [Source:UniProtKB/TrEMBL;Acc:L8H522]	0.166666667	0.615994333	0.449327667	0.000168667
6h	ACA1_301690/clean/scf7180000083222:19137-19205:-	ACA1_301690	MoCF_biosynth domain-containing protein [Source:UniProtKB/TrEMBL;Acc:L8HJW0]	0.00328979	0.452588667	0.449298877	9.05E-14
6h	ACA1_183690/clean/scf7180000084745:483195-483327:-	ACA1_183690	hypothetical protein	0.027123367	0.476412333	0.449288967	0.002346932
6h	ACA1_057520/clean/scf7180000084599:158722-158807:+	ACA1_057520	NTP_transf_2 domain-containing protein [Source:UniProtKB/TrEMBL;Acc:L8GYG1]	0.0375	0.486528	0.449028	3.68E-05
6h	ACA1_392270/clean/scf7180000084740:266569-266636:-	ACA1_392270	Carrier superfamily protein [Source:UniProtKB/TrEMBL;Acc:L8GP94]	0.039369267	0.488391333	0.449022067	7.14E-12
6h	ACA1_059470/clean/scf7180000084599:452241-452329:+	ACA1_059470	G-patch domain-containing protein [Source:UniProtKB/TrEMBL;Acc:L8GW60]	0.014428167	0.463415333	0.448987167	3.90E-06
6h	ACA1_059980/clean/scf7180000084599:552285-552366:-	ACA1_059980	von Willebrand factor type A domain containing protein [Source:UniProtKB/TrEMBL;Acc:L8GYS2]	0.002649963	0.451559333	0.44890937	1.89E-08
6h	ACA1_214970/clean/scf7180000084758:280717-280794:-	ACA1_214970	SNF2 family Nterminal domain containing protein [Source:UniProtKB/TrEMBL;Acc:L8GR02]	0.037037067	0.485928667	0.4488916	0.000473012
6h	ACA1_069350/clean/scf7180000084603:417014-417073:-	ACA1_069350	Mannosyltransferase [Source:UniProtKB/TrEMBL;Acc:L8HFX6]	0.010639847	0.459499667	0.44885982	2.39E-09
6h	ACA1_380350/clean/scf7180000084712:43172-43252:+	ACA1_380350	Membrane protein, putative [Source:UniProtKB/TrEMBL;Acc:L8GMT8]	0.00273224	0.451536	0.44880376	1.04E-09
6h	ACA1_093110/clean/scf7180000084645:481958-482028:+	ACA1_093110	GON domain-containing protein [Source:UniProtKB/TrEMBL;Acc:L8GKP7]	0.0662113	0.515006333	0.448795033	2.60E-06
6h	ACA1_196310/clean/scf7180000084752:56942-57152:+	ACA1_196310	SET domain-containing protein [Source:UniProtKB/TrEMBL;Acc:L8HDZ8]	0.531911333	0.980683333	0.448772	1.34E-06
6h	ACA1_253100/clean/scf7180000084768:240633-240703:-	ACA1_253100	MORN repeatcontaining protein [Source:UniProtKB/TrEMBL;Acc:L8H9X8]	0.003549767	0.452214	0.448664233	3.17E-06
6h	ACA1_175300/clean/scf7180000084741:914599-914661:+	ACA1_175300	Zinc knuckle domain containing protein [Source:UniProtKB/TrEMBL;Acc:L8HKB8]	0.063990533	0.512455333	0.4484648	0.000420548
6h	ACA1_218650/clean/scf7180000084758:684567-684689:-	ACA1_218650	RIBOSOMAL_L9 domain-containing protein [Source:UniProtKB/TrEMBL;Acc:L8GSW4]	0.018782117	0.467240667	0.44845855	9.37E-12
6h	ACA1_230520/clean/scf7180000084761:371059-371162:-	ACA1_230520	Hypothetical protein	0.013007867	0.461434333	0.448426467	1.92E-12
6h	ACA1_243590/clean/scf7180000084763:351163-351245:+	ACA1_243590	hypothetical protein	0.00525285	0.453465	0.44821215	3.86E-10
6h	ACA1_144000/clean/scf7180000084709:123119-123192:+	ACA1_144000	RhoGEF domain containing protein [Source:UniProtKB/TrEMBL;Acc:L8HFT1]	0	0.448057333	0.448057333	6.42E-10
6h	ACA1_175480/clean/scf7180000084741:966948-967026:-	ACA1_175480	PQ loop repeat-containing protein [Source:UniProtKB/TrEMBL;Acc:L8HJK6]	0.181901667	0.629906333	0.448004667	1.63E-05
6h	ACA1_264360/clean/scf7180000084770:247574-247662:+	ACA1_264360	hypothetical protein	0	0.447937667	0.447937667	1.60E-07
6h	ACA1_383060/clean/scf7180000084721:134017-134123:+	ACA1_383060	hypothetical protein	0.012049673	0.459937333	0.44788766	2.29E-18
6h	ACA1_258270/clean/scf7180000084769:216391-216534:+	ACA1_258270	Ras subfamily protein [Source:UniProtKB/TrEMBL;Acc:L8GHB6]	0.002252253	0.450064	0.447811747	1.04E-08
6h	ACA1_144350/clean/scf7180000084709:213861-213936:+	ACA1_144350	CN hydrolase domain-containing protein [Source:UniProtKB/TrEMBL;Acc:L8HEV4]	0.008646357	0.456425	0.447778643	5.25E-17
6h	ACA1_171880/clean/scf7180000084741:183873-183962:-	ACA1_171880	F-box domain-containing protein [Source:UniProtKB/TrEMBL;Acc:L8HGG2]	0.0564495	0.503777	0.4473275	6.74E-06
6h	ACA1_066870/clean/scf7180000084600:67042-67112:-	ACA1_066870	Glucose-6-phosphate isomerase [Source:UniProtKB/TrEMBL;Acc:L8GRA3]	0.001910477	0.449188333	0.447277857	3.02E-12
6h	ACA1_043650/clean/scf7180000084573:193413-193506:-	ACA1_043650	Poly(Adpribose) glycohydrolase [Source:UniProtKB/TrEMBL;Acc:L8GUS0]	0.000801283	0.448072333	0.44727105	9.84E-08
6h	ACA1_043340/clean/scf7180000084573:167436-167527:+	ACA1_043340	hypothetical protein	0.0198435	0.467112333	0.447268833	2.00E-08
6h	ACA1_054460/clean/scf7180000084594:500282-500388:-	ACA1_054460	Cytochrome b5 heme-binding domain-containing protein [Source:UniProtKB/TrEMBL;Acc:L8H6D5]	0.000631236	0.447884333	0.447253098	4.94E-16
6h	ACA1_106330/clean/scf7180000084660:87229-87413:-	ACA1_106330	Methyltransf_11 domain-containing protein [Source:UniProtKB/TrEMBL;Acc:L8GMT5]	0.006431735	0.453608333	0.447176598	2.02E-09
6h	ACA1_133850/clean/scf7180000084701:99651-99718:-	ACA1_133850	hypothetical protein	0.0116373	0.458777667	0.447140367	1.79E-07
6h	ACA1_269750/clean/scf7180000084770:1032178-1032260:+	ACA1_269750	DUF953 domain-containing protein [Source:UniProtKB/TrEMBL;Acc:L8H4A0]	0	0.447088667	0.447088667	1.84E-13
6h	ACA1_175530/clean/scf7180000084741:986908-987012:+	ACA1_175530	Nitrogen permease, putative [Source:UniProtKB/TrEMBL;Acc:L8HJL1]	0.00091683	0.447954333	0.447037503	8.19E-07
6h	ACA1_271750/clean/scf7180000084771:100342-100416:-	ACA1_271750	Metal cation transporter, ZIP subfamily protein [Source:UniProtKB/TrEMBL;Acc:L8GNS8]	0.000365097	0.447383667	0.44701857	4.44E-09
6h	ACA1_109100/clean/scf7180000084661:115761-115851:-	ACA1_109100	Sodium symporter, putative [Source:UniProtKB/TrEMBL;Acc:L8GC73]	0	0.446849333	0.446849333	7.37E-07
6h	ACA1_280570/clean/scf7180000084776:497657-497750:+	ACA1_280570	Znfinger in Ran binding protein and others domain containing protein [Source:UniProtKB/TrEMBL;Acc:L8H8F5]	0.009259267	0.456095	0.446835733	4.55E-06
6h	ACA1_371900/clean/scf7180000084664:609040-609125:-	ACA1_371900	EF hand domain containing protein [Source:UniProtKB/TrEMBL;Acc:L8GYH0]	0.049820467	0.496494333	0.446673867	2.35E-07
6h	ACA1_382270/clean/scf7180000084721:19199-19296:-	ACA1_382270	ATPase/histidine kinase/DNA gyrase B/HSP90 domain containing protein [Source:UniProtKB/TrEMBL;Acc:L8GU96]	0	0.446630667	0.446630667	5.41E-11
6h	ACA1_156720/clean/scf7180000084723:58738-58837:+	ACA1_156720	INTRADIOL_DIOXYGENAS domain-containing protein [Source:UniProtKB/TrEMBL;Acc:L8GI57]	0.004941517	0.451515333	0.446573817	2.37E-07
6h	ACA1_251650/clean/scf7180000084768:104067-104145:+	ACA1_251650	Protein kinase domain-containing protein [Source:UniProtKB/TrEMBL;Acc:L8HD04]	0	0.446559333	0.446559333	1.76E-10
6h	ACA1_275670/clean/scf7180000084774:82349-82491:-	ACA1_275670	unspecified product	0.045069383	0.491488	0.446418617	5.00E-08
6h	ACA1_391460/clean/scf7180000084740:177806-177905:+	ACA1_391460	Ubiquitin carboxyl-terminal hydrolase [Source:UniProtKB/TrEMBL;Acc:L8GP69]	8.82E-05	0.446505667	0.446417504	2.61E-10
6h	ACA1_057110/clean/scf7180000084599:48663-48731:-	ACA1_057110	unspecified product	0	0.446416	0.446416	3.92E-05
6h	ACA1_095060/clean/scf7180000084645:804206-804358:-	ACA1_095060	Sidoreflexin [Source:UniProtKB/TrEMBL;Acc:L8GL14]	0.020670167	0.467058	0.446387833	1.29E-09
6h	ACA1_293710/clean/scf7180000084777:965243-965355:+	ACA1_293710	F-box domain-containing protein [Source:UniProtKB/TrEMBL;Acc:L8HJM5]	0.018928447	0.46528	0.446351553	2.21E-11
6h	ACA1_067770/clean/scf7180000084603:12864-12944:+	ACA1_067770	CBS domain containing protein [Source:UniProtKB/TrEMBL;Acc:L8HEJ6]	0.071670267	0.517931	0.446260733	1.14E-11
6h	ACA1_089050/clean/scf7180000084639:309752-309829:+	ACA1_089050	Serine/threonine kinase [Source:UniProtKB/TrEMBL;Acc:L8GVP4]	0	0.446148333	0.446148333	1.44E-06
6h	ACA1_119430/clean/scf7180000084672:66699-66799:+	ACA1_119430	RRM domain-containing protein [Source:UniProtKB/TrEMBL;Acc:L8HKC8]	0	0.445956333	0.445956333	6.19E-12
6h	ACA1_173810/clean/scf7180000084741:522815-522900:+	ACA1_173810	Carrier superfamily protein [Source:UniProtKB/TrEMBL;Acc:L8HJ56]	0.000501037	0.446430667	0.44592963	5.83E-13
6h	ACA1_380780/clean/scf7180000084712:88004-88081:-	ACA1_380780	hypothetical protein	0.001097427	0.446926333	0.445828907	1.12E-11
6h	ACA1_193320/clean/scf7180000084749:231312-231402:-	ACA1_193320	hypothetical protein	0	0.445800667	0.445800667	0.000123365
6h	ACA1_191150/clean/scf7180000084748:186806-186874:+	ACA1_191150	Triosephosphate isomerase [Source:UniProtKB/TrEMBL;Acc:L8GE90]	0.0027894	0.448564333	0.445774933	2.45E-12
6h	ACA1_175700/clean/scf7180000084741:1039598-1039761:-	ACA1_175700	Frizzled/Smoothened family membrane region protein [Source:UniProtKB/TrEMBL;Acc:L8HH45]	0.058022433	0.503670667	0.445648233	4.04E-08
6h	ACA1_111900/clean/scf7180000084669:61857-61940:+	ACA1_111900	Global transcription factor, putative [Source:UniProtKB/TrEMBL;Acc:L8H3V5]	0	0.445642333	0.445642333	8.25E-09
6h	ACA1_367310/clean/scf7180000084641:595398-595498:-	ACA1_367310	CCHC-type domain-containing protein [Source:UniProtKB/TrEMBL;Acc:L8GMZ2]	0.083288867	0.528761	0.445472133	5.57E-08
6h	ACA1_070820/clean/scf7180000084603:769327-769472:+	ACA1_070820	hypothetical protein	0.021840687	0.467308667	0.44546798	4.83E-06
6h	ACA1_253160/clean/scf7180000084768:255534-255626:+	ACA1_253160	Leucine rich repeat domain containing protein [Source:UniProtKB/TrEMBL;Acc:L8HAV9]	0.005641733	0.450867667	0.445225933	8.72E-06
6h	ACA1_171240/clean/scf7180000084741:51894-51993:-	ACA1_171240	Ribosomal_L7Ae domain-containing protein [Source:UniProtKB/TrEMBL;Acc:L8HJI5]	0.031274333	0.476448	0.445173667	1.35E-06
6h	ACA1_042250/clean/scf7180000084573:69303-69401:-	ACA1_042250	Phosphoglucomutase, putative [Source:UniProtKB/TrEMBL;Acc:L8GV41]	0.003552303	0.448724667	0.445172363	3.03E-10
6h	ACA1_319080/clean/scf7180000084239:7937-8008:+	ACA1_319080	hypothetical protein	0	0.445104667	0.445104667	1.44E-09
6h	ACA1_174040/clean/scf7180000084741:586408-586513:-	ACA1_174040	Protein kinase domain-containing protein [Source:UniProtKB/TrEMBL;Acc:L8HK27]	0.000288143	0.445384	0.445095857	4.49E-10
6h	ACA1_250030/clean/scf7180000084767:27167-27233:-	ACA1_250030	C2 NT-type domain-containing protein [Source:UniProtKB/TrEMBL;Acc:L8HC94]	0.026579933	0.471648333	0.4450684	5.21E-09
6h	ACA1_378630/clean/scf7180000084711:242888-242977:-	ACA1_378630	AAA-ATPase_like domain-containing protein [Source:UniProtKB/TrEMBL;Acc:L8GSY2]	0.006172833	0.451202667	0.445029833	1.71E-07
6h	ACA1_065270/clean/scf7180000084599:1839053-1839144:+	ACA1_065270	Ubiquitin ligase, putative [Source:UniProtKB/TrEMBL;Acc:L8GYX5]	0	0.444956	0.444956	2.03E-05
6h	ACA1_039190/clean/scf7180000084569:18227-18322:+	ACA1_039190	ANK_REP_REGION domain-containing protein [Source:UniProtKB/TrEMBL;Acc:L8HGQ0]	0.016972303	0.461784	0.444811697	2.87E-06
6h	ACA1_097130/clean/scf7180000084645:1167071-1167154:+	ACA1_097130	Serine/threonine kinase [Source:UniProtKB/TrEMBL;Acc:L8GIY5]	0.0148342	0.459589	0.4447548	0.000357868
6h	ACA1_178230/clean/scf7180000084743:185278-185343:+	ACA1_178230	Transducin family protein [Source:UniProtKB/TrEMBL;Acc:L8GSK8]	0.013356604	0.45795	0.444593396	1.03E-11
6h	ACA1_277560/clean/scf7180000084776:74553-74614:-	ACA1_277560	F-box domain-containing protein [Source:UniProtKB/TrEMBL;Acc:L8H6G6]	0.140561833	0.585136	0.444574167	1.52E-06
6h	ACA1_063800/clean/scf7180000084599:1506266-1506367:-	ACA1_063800	B30.2/SPRY domain-containing protein [Source:UniProtKB/TrEMBL;Acc:L8GZV1]	0.020877317	0.465429	0.444551683	2.22E-11
6h	ACA1_115650/clean/scf7180000084669:637106-637175:+	ACA1_115650	hypothetical protein	0.025601623	0.470121333	0.44451971	3.72E-07
6h	ACA1_229490/clean/scf7180000084761:259611-259691:+	ACA1_229490	hypothetical protein	0.00255726	0.446975333	0.444418073	1.48E-08
6h	ACA1_189360/clean/scf7180000084748:17086-17158:-	ACA1_189360	Proline dehydrogenase [Source:UniProtKB/TrEMBL;Acc:L8GGF7]	0	0.444309667	0.444309667	0.000413155

6h	ACA1_368080/clean/scf7180000084664:49417-49488:-	ACA1_368080	hypothetical protein	0.023809533	0.468063333	0.4442538	0.000512642
6h	ACA1_064290/clean/scf7180000084599:1576110-1576188:-	ACA1_064290	hypothetical protein	0.023710753	0.467848667	0.444137913	2.14E-08
6h	ACA1_163110/clean/scf7180000084726:84011-84099:-	ACA1_163110	hypothetical protein	0	0.443857333	0.443857333	1.06E-08
6h	ACA1_200210/clean/scf7180000084753:414913-414999:+	ACA1_200210	hypothetical protein	0.011755267	0.455497	0.443741733	1.80E-09
6h	ACA1_091960/clean/scf7180000084645:236987-237087:-	ACA1_091960	hypothetical protein	0.0179645	0.461519667	0.443555167	3.29E-07
6h	ACA1_323310/clean/scf7180000084468:1691-1771:+	ACA1_323310	hypothetical protein	0.005617967	0.449088	0.443470033	1.48E-06
6h	ACA1_141880/clean/scf7180000084708:42168-42260:+	ACA1_141880	RNA editing ligase [Source:UniProtKB/TrEMBL;Acc:L8HCM1]	0.015137267	0.458586	0.443448733	1.96E-06
6h	ACA1_286600/clean/scf7180000084776:1072354-1072431:-	ACA1_286600	VWFA domain-containing protein [Source:UniProtKB/TrEMBL;Acc:L8H904]	0.004157233	0.447382333	0.4432251	1.23E-05
6h	ACA1_040450/clean/scf7180000084572:6990-7080:+	ACA1_040450	Carrier superfamily protein [Source:UniProtKB/TrEMBL;Acc:L8H0I1]	0.033562333	0.476696	0.443133667	3.92E-08
6h	ACA1_262400/clean/scf7180000084770:43217-43302:+	ACA1_262400	Probable tRNA N6-adenosine threonylcarbamoyltransferase [Source:UniProtKB/TrEMBL;Acc:L8H1V9]	0.001430617	0.444494667	0.44306405	1.38E-11
6h	ACA1_290510/clean/scf7180000084777:609159-609232:-	ACA1_290510	ABC transporter domain-containing protein [Source:UniProtKB/TrEMBL;Acc:L8HLJ2]	0.002783337	0.445843667	0.44306033	5.54E-11
6h	ACA1_320820/clean/scf7180000084416:29287-29395:+	ACA1_320820	hypothetical protein	0.0133225	0.456351	0.4430285	9.24E-45
6h	ACA1_003640/clean/scf7180000082964:23566-23672:+	ACA1_003640	hypothetical protein	0	0.443011667	0.443011667	0.003933588
6h	ACA1_037450/clean/scf7180000084567:93445-93506:+	ACA1_037450	NTPase_I-T domain-containing protein [Source:UniProtKB/TrEMBL;Acc:L8H395]	0.021470753	0.464389333	0.44291858	6.69E-16
6h	ACA1_218390/clean/scf7180000084758:650712-650826:-	ACA1_218390	Glycosyl hydrolase family 20, catalytic domain containing protein [Source:UniProtKB/TrEMBL;Acc:L8GQD2]	0.022022867	0.464843333	0.442820467	1.16E-08
6h	ACA1_306840/clean/scf7180000083668:18316-18415:+	ACA1_306840	RTP1_C1 domain-containing protein [Source:UniProtKB/TrEMBL;Acc:L8GQF6]	0.007416613	0.450181667	0.442765053	2.17E-06
6h	ACA1_195560/clean/scf7180000084752:15901-15995:+	ACA1_195560	RIO domain-containing protein [Source:UniProtKB/TrEMBL;Acc:L8HDY7]	0.009260491	0.451959333	0.442698842	5.24E-07
6h	ACA1_320100/clean/scf7180000084352:7362-7452:+	ACA1_320100	VWFA domain-containing protein [Source:UniProtKB/TrEMBL;Acc:L8H729]	0.01174122	0.454320667	0.442579447	1.78E-08
6h	ACA1_071190/clean/scf7180000084603:886799-886897:+	ACA1_071190	RNA_ligase domain-containing protein [Source:UniProtKB/TrEMBL;Acc:L8HFI2]	0	0.442474667	0.442474667	0.003337376
6h	ACA1_400560/clean/scf7180000084775:194180-194258:+	ACA1_400560	hypothetical protein	0.00061162	0.443016333	0.442404713	1.10E-09
6h	ACA1_200240/clean/scf7180000084753:419661-419755:-	ACA1_200240	Phospholipase D active site domain containing protein [Source:UniProtKB/TrEMBL;Acc:L8H3B9]	0.005847967	0.448193333	0.442345367	9.48E-08
6h	ACA1_158180/clean/scf7180000084724:204624-204933:+	ACA1_158180	eIF2A domain-containing protein [Source:UniProtKB/TrEMBL;Acc:L8H989]	0.014410267	0.456695667	0.4422854	4.31E-18
6h	ACA1_276410/clean/scf7180000084774:167647-167841:+	ACA1_276410	Phosphate transporter [Source:UniProtKB/TrEMBL;Acc:L8GTH7]	0.001861527	0.444143	0.442281473	1.45E-08
6h	ACA1_038210/clean/scf7180000084568:97245-97329:-	ACA1_038210	hypothetical protein	0.030120072	0.472401	0.442280928	4.74E-07
6h	ACA1_125990/clean/scf7180000084693:16902-17000:-	ACA1_125990	hypothetical protein	0.014609667	0.456802667	0.442193	2.74E-09
6h	ACA1_144440/clean/scf7180000084709:244330-244447:+	ACA1_144440	SNF2 family Nterminal domain containing protein [Source:UniProtKB/TrEMBL;Acc:L8HFN1]	0.007186517	0.449258	0.442071483	2.69E-17
6h	ACA1_082180/clean/scf7180000084623:19725-19815:+	ACA1_082180	N-acetyltransferase domain-containing protein [Source:UniProtKB/TrEMBL;Acc:L8GMF4]	0.005433097	0.44742	0.441986903	8.38E-09
6h	ACA1_189450/clean/scf7180000084748:41176-41260:-	ACA1_189450	Chloride channel protein [Source:UniProtKB/TrEMBL;Acc:L8GEJ2]	0.003551733	0.445497667	0.441945933	1.24E-09
6h	ACA1_150950/clean/scf7180000084716:33380-33489:+	ACA1_150950	Exocyst complex component Sec6 [Source:UniProtKB/TrEMBL;Acc:L8H215]	0.006172833	0.447993	0.441820167	4.52E-08
6h	ACA1_277790/clean/scf7180000084776:102207-102299:-	ACA1_277790	Protease, serine, 16 (Thymus), putative [Source:UniProtKB/TrEMBL;Acc:L8H5Q8]	0.000270783	0.441881	0.441610217	5.30E-11
6h	ACA1_109740/clean/scf7180000084663:15776-15847:-	ACA1_109740	Myblike DNA-binding domain containing protein [Source:UniProtKB/TrEMBL;Acc:L8HJY9]	0	0.441604333	0.441604333	4.91E-09
6h	ACA1_027030/clean/scf7180000084464:27590-27664:-	ACA1_027030	C2 domain-containing protein [Source:UniProtKB/TrEMBL;Acc:L8GJ80]	0.008248873	0.449590333	0.44134146	2.29E-15
6h	ACA1_383380/clean/scf7180000084721:159237-159298:+	ACA1_383380	Lysosomal ProX carboxypeptidase [Source:UniProtKB/TrEMBL;Acc:L8GV42]	0.005662123	0.446704	0.441041877	1.91E-12
6h	ACA1_266140/clean/scf7180000084770:612202-612275:-	ACA1_266140	CBAH domain-containing protein [Source:UniProtKB/TrEMBL;Acc:L8H3S3]	0.015524167	0.45655	0.441025833	5.42E-15
6h	ACA1_172870/clean/scf7180000084741:343494-343576:-	ACA1_172870	Nadph-dependent carbonyl reductase family protein [Source:UniProtKB/TrEMBL;Acc:L8HHL8]	0.002087903	0.442951667	0.440863763	1.67E-10
6h	ACA1_381570/clean/scf7180000084712:183636-183712:+	ACA1_381570	Ras-GAP domain-containing protein [Source:UniProtKB/TrEMBL;Acc:L8GMY5]	0.006239997	0.447039	0.440799003	5.29E-12
6h	ACA1_323200/clean/scf7180000084467:29736-29809:-	ACA1_323200	ShKT domain-containing protein [Source:UniProtKB/TrEMBL;Acc:L8GFA8]	0	0.440792	0.440792	9.25E-09
6h	ACA1_264290/clean/scf7180000084770:230333-230425:-	ACA1_264290	Geranylgeranyl diphosphate synthase 1, putative [Source:UniProtKB/TrEMBL;Acc:L8H3G3]	0.004172933	0.444768667	0.440595733	3.73E-09
6h	ACA1_377830/clean/scf7180000084711:127178-127253:+	ACA1_377830	UBIQUITIN_CONJUGAT_2 domain-containing protein [Source:UniProtKB/TrEMBL;Acc:L8GSV2]	0.037370233	0.477917667	0.440547433	4.91E-13
6h	ACA1_184600/clean/scf7180000084746:4633-4704:-	ACA1_184600	Alpha-N-acetylglucosaminidase family protein [Source:UniProtKB/TrEMBL;Acc:L8H6D3]	0.010101	0.450591667	0.440490667	2.99E-06
6h	ACA1_282990/clean/scf7180000084776:759373-759441:+	ACA1_282990	hypothetical protein	0.056256333	0.496546333	0.44029	1.68E-08
6h	ACA1_116270/clean/scf7180000084669:743933-744008:+	ACA1_116270	G_PROTEIN_RECEP_F1_2 domain-containing protein [Source:UniProtKB/TrEMBL;Acc:L8H6T9]	0.005952367	0.446233667	0.4402813	8.15E-08
6h	ACA1_186700/clean/scf7180000084746:194534-194603:+	ACA1_186700	ADPribosylation factor subfamily protein [Source:UniProtKB/TrEMBL;Acc:L8H553]	0.020451767	0.460585667	0.4401339	6.41E-07
6h	ACA1_257980/clean/scf7180000084769:190719-190795:+	ACA1_257980	5oxoprolinase [Source:UniProtKB/TrEMBL;Acc:L8GF06]	0	0.440012	0.440012	3.38E-11
6h	ACA1_053110/clean/scf7180000084594:216562-216663:-	ACA1_053110	HEAT repeat domain containing protein [Source:UniProtKB/TrEMBL;Acc:L8H7C3]	0	0.439914	0.439914	4.58E-08
6h	ACA1_176030/clean/scf7180000084741:1110789-1110905:-	ACA1_176030	ATPase, AAA domain containing protein [Source:UniProtKB/TrEMBL;Acc:L8HI90]	0.0216126	0.461431333	0.439818733	4.80E-06
6h	ACA1_266900/clean/scf7180000084770:749028-749168:+	ACA1_266900	hypothetical protein	0.184495667	0.624272	0.439776333	0.000473205
6h	ACA1_243160/clean/scf7180000084763:323902-323967:+	ACA1_243160	Glyco_trans_2-like domain-containing protein [Source:UniProtKB/TrEMBL;Acc:L8GMD7]	0.0131471	0.452904	0.4397569	1.25E-17
6h	ACA1_364280/clean/scf7180000084641:70499-70610:+	ACA1_364280	Sporulation proteinrelated, putative [Source:UniProtKB/TrEMBL;Acc:L8GLJ0]	0.002293859	0.442009	0.439715141	3.16E-13
6h	ACA1_203010/clean/scf7180000084754:117347-117424:+	ACA1_203010	RhoGEF domain containing protein [Source:UniProtKB/TrEMBL;Acc:L8GTP3]	0	0.439705	0.439705	8.86E-08
6h	ACA1_320810/clean/scf7180000084416:25573-25730:+	ACA1_320810	PGM1_C domain-containing protein [Source:UniProtKB/TrEMBL;Acc:L8GTZ1]	0.00393445	0.443584	0.43964955	3.16E-07
6h	ACA1_074520/clean/scf7180000084608:30026-30107:+	ACA1_074520	SH3 domain containing protein [Source:UniProtKB/TrEMBL;Acc:L8HFD8]	0.0414707	0.481031667	0.439560967	1.00E-12
6h	ACA1_073270/clean/scf7180000084603:1385579-1385638:+	ACA1_073270	WD_REPEATS_REGION domain-containing protein [Source:UniProtKB/TrEMBL;Acc:L8HG33]	0	0.439458667	0.439458667	1.06E-09
6h	ACA1_278320/clean/scf7180000084776:195840-195922:+	ACA1_278320	N-acetyltransferase domain-containing protein [Source:UniProtKB/TrEMBL;Acc:L8H8U2]	0.002294963	0.441723	0.439428037	1.70E-09
6h	ACA1_062880/clean/scf7180000084599:1299475-1299554:+	ACA1_062880	hypothetical protein	0	0.439153667	0.439153667	2.57E-09
6h	ACA1_037780/clean/scf7180000084568:22777-22859:+	ACA1_037780	RhoGEF domain containing protein [Source:UniProtKB/TrEMBL;Acc:L8GN17]	0.018470233	0.457517333	0.4390471	2.73E-07
6h	ACA1_155670/clean/scf7180000084722:212842-212956:+	ACA1_155670	SPRY domain containing protein [Source:UniProtKB/TrEMBL;Acc:L8H063]	0.013483103	0.452436667	0.438953563	4.35E-10
6h	ACA1_392390/clean/scf7180000084740:269099-269224:+	ACA1_392390	Rfwd1prov protein [Source:UniProtKB/TrEMBL;Acc:L8GPG4]	0.01002025	0.448949	0.43892875	2.41E-07
6h	ACA1_191490/clean/scf7180000084749:2550-2612:-	ACA1_191490	RTR1-type domain-containing protein [Source:UniProtKB/TrEMBL;Acc:L8GQP2]	0.00307318	0.442	0.43892682	4.31E-10
6h	ACA1_232860/clean/scf7180000084762:87864-87958:+	ACA1_232860	hypothetical protein	0.003053127	0.441968333	0.438915207	8.00E-09
6h	ACA1_116660/clean/scf7180000084669:823444-823541:-	ACA1_116660	Zinc finger domain containing protein [Source:UniProtKB/TrEMBL;Acc:L8H3W5]	0.007709433	0.446587	0.438877567	4.59E-08
6h	ACA1_252540/clean/scf7180000084768:176023-176105:-	ACA1_252540	hypothetical protein	0.00983622	0.448701667	0.438865447	2.15E-22
6h	ACA1_057400/clean/scf7180000084599:134922-135003:+	ACA1_057400	Leucine rich repeat domain containing protein [Source:UniProtKB/TrEMBL;Acc:L8GVU1]	0.004325431	0.442868333	0.438542902	4.17E-07
6h	ACA1_365680/clean/scf7180000084641:292311-292409:-	ACA1_365680	NADPH-dependent diflavin oxidoreductase 1 [Source:UniProtKB/TrEMBL;Acc:L8GMJ6]	0.001855287	0.440311333	0.438456047	1.40E-08
6h	ACA1_061500/clean/scf7180000084599:942490-942596:+	ACA1_061500	Ras-GAP domain-containing protein [Source:UniProtKB/TrEMBL;Acc:L8GWM5]	0.0136963	0.45211	0.4384137	2.92E-09
6h	ACA1_000820/clean/scf7180000082828:8859-8939:+	ACA1_000820	RING-type domain-containing protein [Source:UniProtKB/TrEMBL;Acc:L8H7M3]	0	0.438355	0.438355	5.49E-09
6h	ACA1_191030/clean/scf7180000084748:183919-184032:+	ACA1_191030	CUE domain-containing protein [Source:UniProtKB/TrEMBL;Acc:L8GE21]	0.011485867	0.44984	0.438354133	1.48E-06
6h	ACA1_005320/clean/scf7180000083021:2093-2164:+	ACA1_005320	DEAD/DEAH box helicase [Source:UniProtKB/TrEMBL;Acc:L8GS15]	0.009191033	0.447532667	0.438341633	5.33E-09
6h	ACA1_202770/clean/scf7180000084754:106342-106433:-	ACA1_202770	TPR_REGION domain-containing protein [Source:UniProtKB/TrEMBL;Acc:L8GSW6]	0.004292497	0.442597667	0.43830517	2.28E-08
6h	ACA1_111870/clean/scf7180000084669:50339-50419:-	ACA1_111870	SNF2 family Nterminal domain containing protein [Source:UniProtKB/TrEMBL;Acc:L8H2Z3]	0.01029138	0.448503667	0.438212287	1.65E-09
6h	ACA1_356140/clean/scf7180000084731:17434-17528:-	ACA1_356140	RasGEF domain containing protein [Source:UniProtKB/TrEMBL;Acc:L8HHY3]	0	0.438209	0.438209	9.41E-09
6h	ACA1_116730/clean/scf7180000084669:837932-838017:+	ACA1_116730	PH domain-containing protein [Source:UniProtKB/TrEMBL;Acc:L8H6W6]	0.042170533	0.480341333	0.4381708	4.48E-07
6h	ACA1_245470/clean/scf7180000084763:493953-494009:+	ACA1_245470	4F5 domain-containing protein [Source:UniProtKB/TrEMBL;Acc:L8GK44]	0.002396063	0.440421667	0.438025603	3.53E-36
6h	ACA1_256610/clean/scf7180000084769:64179-64258:-	ACA1_256610	hypothetical protein	0	0.437834	0.437834	0.000674938
6h	ACA1_052260/clean/scf7180000084594:27136-27237:-	ACA1_052260	Leucine rich repeat domain containing protein [Source:UniProtKB/TrEMBL;Acc:L8H5N7]	0.023524733	0.461239333	0.4377146	3.21E-05
6h	ACA1_065770/clean/scf7180000084599:1893327-1893416:-	ACA1_065770	Phosphatidylinositol 3kinase [Source:UniProtKB/TrEMBL;Acc:L8GXT7]	0.00064085	0.438305	0.43766415	9.25E-11
6h	ACA1_209530/clean/scf7180000084757:303079-303231:-	ACA1_209530	Purple acid phosphatase [Source:UniProtKB/TrEMBL;Acc:L8GZQ1]	0.0245371	0.461926	0.4373889	5.58E-09
6h	ACA1_215970/clean/scf7180000084758:421221-421292:-	ACA1_215970	EGFlike domain containing protein [Source:UniProtKB/TrEMBL;Acc:L8GQ73]	0.018518533	0.455842	0.437323467	0.000910428
6h	ACA1_091950/clean/scf7180000084645:235130-235224:-	ACA1_091950	Peptidase_M13_N domain-containing protein [Source:UniProtKB/TrEMBL;Acc:L8GHZ1]	0.00125786	0.438521333	0.437263473	1.18E-08
6h	ACA1_108290/clean/scf7180000084661:30497-30581:-	ACA1_108290	Golgi SNAP receptor complex member 1 [Source:UniProtKB/TrEMBL;Acc:L8GCJ1]	0.011265503	0.448487333	0.43722183	1.96E-10
6h	ACA1_323460/clean/scf7180000084468:25874-25972:-	ACA1_323460	DEP domain-containing protein [Source:UniProtKB/TrEMBL;Acc:L8H1P0]	0.006489633	0.443622667	0.437133033	5.09E-08
6h	ACA1_279050/clean/scf7180000084776:301443-301573:-	ACA1_279050	PCI domain-containing protein [Source:UniProtKB/TrEMBL;Acc:L8H5Y4]	0.006160727	0.443267	0.437106273	1.38E-19
6h	ACA1_095270/clean/scf7180000084645:825850-825963:-	ACA1_095270	Thioredoxin-like_fold domain-containing protein [Source:UniProtKB/TrEMBL;Acc:L8GIR9]	0.004721703	0.441724333	0.43700263	1.87E-09
6h	ACA1_148550/clean/scf7180000084715:32478-32558:+	ACA1_148550	AMPbinding enzyme domain containing protein [Source:UniProtKB/TrEMBL;Acc:L8HD91]	0.006661363	0.443588333	0.43692697	1.21E-07
6h	ACA1_399880/clean/scf7180000084775:55516-55596:+	ACA1_399880	Rab-GAP TBC domain-containing protein [Source:UniProtKB/TrEMBL;Acc:L8GGF5]	0.015860367	0.452785333	0.436924967	4.48E-09
6h	ACA1_380340/clean/scf7180000084712:41224-41301:-	ACA1_380340	Leucine rich repeat-containing protein [Source:UniProtKB/TrEMBL;Acc:L8GNJ3]	0.028669867	0.465543667	0.4368738	1.73E-07
6h	ACA1_165280/clean/scf7180000084732:25341-25446:-	ACA1_165280	PH domain containing protein [Source:UniProtKB/TrEMBL;Acc:L8HI11]	0.055749687	0.49261	0.436860313	1.20E-07
6h	ACA1_391090/clean/scf7180000084740:126852-126962:-	ACA1_391090	Inorganic diphosphatase [Source:UniProtKB/TrEMBL;Acc:L8GPC3]	0.014037087	0.450667667	0.43663058	7.93E-07
6h	ACA1_345670/clean/scf7180000084657:2098-2172:-	ACA1_345670	hypothetical protein	0	0.436568667	0.436568667	3.97E-05
6h	ACA1_046390/clean/scf7180000084577:84544-84655:-	ACA1_046390	hypothetical protein	0.017543867	0.454111333	0.436567467	0.00040942
6h	ACA1_214850/clean/scf7180000084758:245772-245864:+	ACA1_214850	LIM zinc-binding domain-containing protein [Source:UniProtKB/TrEMBL;Acc:L8GSG1]	0.024551363	0.461088	0.436536637	5.36E-13
6h	ACA1_165210/clean/scf7180000084732:8585-8677:-	ACA1_165210	Ras subfamily protein [Source:UniProtKB/TrEMBL;Acc:L8HGF1]	0.002190143	0.438673667	0.436483523	1.25E-16
6h	ACA1_075020/clean/scf7180000084608:128325-128392:-	ACA1_075020	Rab7, putative [Source:UniProtKB/TrEMBL;Acc:L8HEH5]	0.017725333	0.454178	0.436452667	3.90E-22
6h	ACA1_215900/clean/scf7180000084758:400405-400524:+	ACA1_215900	RAP1A, member of RAS oncogene family [Source:UniProtKB/TrEMBL;Acc:L8GR43]	0.005600087	0.442035667	0.43643558	3.73E-39
6h	ACA1_163930/clean/scf7180000084729:115954-116010:+	ACA1_163930	Peptidase_M14 domain-containing protein [Source:UniProtKB/TrEMBL;Acc:L8GQX3]	0.002168856	0.438469	0.436300144	3.58E-10
6h	ACA1_026960/clean/scf7180000084464:4996-5073:-	ACA1_026960	FtsX domain-containing protein [Source:UniProtKB/TrEMBL;Acc:L8GHC2]	0	0.436026	0.436026	2.91E-12
6h	ACA1_183530/clean/scf7180000084745:440996-441100:-	ACA1_183530	Clu domain-containing protein [Source:UniProtKB/TrEMBL;Acc:L8H9K5]	0.013460067	0.449449667	0.4359896	1.39E-08
6h	ACA1_084820/clean/scf7180000084632:8597-8702:+	ACA1_084820	RhoGEF domain containing protein [Source:UniProtKB/TrEMBL;Acc:L8HMR3]	0	0.435940667	0.435940667	4.80E-09
6h	ACA1_183970/clean/scf7180000084745:532391-532483:-	ACA1_183970	hypothetical protein	0.082278333	0.518195333	0.435917	0.001041629
6h	ACA1_002620/clean/scf7180000082935:5797-5865:+	ACA1_002620	Fbox domain containing protein [Source:UniProtKB/TrEMBL;Acc:L8HLR9]	0.00660207	0.442329667	0.435727597	6.44E-10
6h	ACA1_073280/clean/scf7180000084603:1387092-1387153:+	ACA1_073280	Thg1 domain-containing protein [Source:UniProtKB/TrEMBL;Acc:L8HDU3]	0.0060094	0.441704667	0.435695267	2.62E-08
6h	ACA1_057360/clean/scf7180000084599:123236-123301:+	ACA1_057360	Mitochondrial inner membrane protease subunit [Source:UniProtKB/TrEMBL;Acc:L8GYF2]	0.011976033	0.447669333	0.4356933	1.56E-06
6h	ACA1_114470/clean/scf7180000084669:482446-482528:+	ACA1_114470	C2 domain containing protein [Source:UniProtKB/TrEMBL;Acc:L8H5S6]	0.230987333	0.666666667	0.435679333	0.002268635
6h	ACA1_038160/clean/scf7180000084568:86387-86636:-	ACA1_038160	Protein kinase domain-containing protein [Source:UniProtKB/TrEMBL;Acc:L8GN38]	0.030728	0.466292	0.435564	3.36E-15
6h	ACA1_295580/clean/scf7180000084777:1177642-1177749:+	ACA1_295580	Solute carrier family protein [Source:UniProtKB/TrEMBL;Acc:L8HJT6]	0	0.435331	0.435331	1.43E-09
6h	ACA1_126940/clean/scf7180000084696:5030-5098:+	ACA1_126940	hypothetical protein	0.010755467	0.446030333	0.435274867	1.42E-09
6h	ACA1_225950/clean/scf7180000084761:29711-29772:-	ACA1_225950	TPK_B1_binding domain-containing protein [Source:UniProtKB/TrEMBL;Acc:L8H7N9]	0.210611	0.645807	0.435196	0.004547935
6h	ACA1_252210/clean/scf7180000084768:144673-144770:+	ACA1_252210	Protein kinase domain-containing protein [Source:UniProtKB/TrEMBL;Acc:L8HAM0]	0.011184373	0.446337	0.435152627	6.57E-07
6h	ACA1_141880/clean/scf7180000084708:41471-41568:+	ACA1_141880	RNA editing ligase [Source:UniProtKB/TrEMBL;Acc:L8HCM1]	0.0018224	0.436972667	0.435150267	6.30E-09
6h	ACA1_174690/clean/scf7180000084741:764862-764944:+	ACA1_174690	Deacetylase sirtuin-type domain-containing protein [Source:UniProtKB/TrEMBL;Acc:L8HHW7]	0.00142418	0.436564333	0.435140153	1.64E-09
6h	ACA1_265500/clean/scf7180000084770:495229-495291:-	ACA1_265500	F-box domain-containing protein [Source:UniProtKB/TrEMBL;Acc:L8H275]	0.218698	0.653787	0.435089	2.34E-06
6h	ACA1_142600/clean/scf7180000084708:198585-198652:+	ACA1_142600	unspecified product	0.01670515	0.451757667	0.435052517	2.80E-15
6h	ACA1_381620/clean/scf7180000084712:198143-198217:+	ACA1_381620	Alpha-galactosidase [Source:UniProtKB/TrEMBL;Acc:L8GMY9]	0.001519843	0.436528	0.435008157	5.17E-39
6h	ACA1_177570/clean/scf7180000084743:104051-104110:+	ACA1_177570	hypothetical protein	0.005006337	0.440002333	0.434995997	3.62E-18
6h	ACA1_142370/clean/scf7180000084708:151634-151726:-	ACA1_142370	Serine hydroxymethyltransferase [Source:UniProtKB/TrEMBL;Acc:L8HDT1]	0.001396799	0.436288333	0.434891534	2.84E-19
6h	ACA1_320810/clean/scf7180000084416:26650-26800:+	ACA1_320810	PGM1_C domain-containing protein [Source:UniProtKB/TrEMBL;Acc:L8GTZ1]	0.00589457	0.440771333	0.434876763	5.00E-08
6h	ACA1_109740/clean/scf7180000084663:14679-14797:-	ACA1_109740	Myblike DNA-binding domain containing protein [Source:UniProtKB/TrEMBL;Acc:L8HJY9]	0.009885233	0.444389	0.434503767	5.33E-09
6h	ACA1_385400/clean/scf7180000084730:164152-164234:-	ACA1_385400	Rieske domain-containing protein [Source:UniProtKB/TrEMBL;Acc:L8H8D5]	0.236476667	0.670961	0.434484333	0.000413452
6h	ACA1_114910/clean/scf7180000084669:585661-585813:-	ACA1_114910	C2 domain containing protein [Source:UniProtKB/TrEMBL;Acc:L8H6P3]	0.00713669	0.441582	0.43444531	8.49E-07
6h	ACA1_114840/clean/scf7180000084669:557459-557608:+	ACA1_114840	Serine/threonine-protein phosphatase [Source:UniProtKB/TrEMBL;Acc:L8H3P4]	0.001144433	0.435575333	0.4344309	3.85E-10
6h	ACA1_182400/clean/scf7180000084745:204872-204961:+	ACA1_182400	Integral membrane protein [Source:UniProtKB/TrEMBL;Acc:L8H9C3]	0.000138608	0.434561667	0.434423058	1.06E-12
6h	ACA1_143970/clean/scf7180000084709:118089-118163:+	ACA1_143970	CyclicAMP phosphodiesterase [Source:UniProtKB/TrEMBL;Acc:L8HFI5]	0.236676667	0.671097	0.434420333	0.015286732
6h	ACA1_055640/clean/scf7180000084594:760067-760133:+	ACA1_055640	Tetratricopeptide repeat-containing protein [Source:UniProtKB/TrEMBL;Acc:L8H7T9]	0.0177859	0.452161	0.4343751	3.57E-07
6h	ACA1_173870/clean/scf7180000084741:537810-537913:-	ACA1_173870	AP complex subunit beta [Source:UniProtKB/TrEMBL;Acc:L8HGQ2]	0	0.434323	0.434323	1.79E-09
6h	ACA1_209790/clean/scf7180000084757:321834-321906:-	ACA1_209790	AMPbinding enzyme domain containing protein [Source:UniProtKB/TrEMBL;Acc:L8GXL7]	0	0.434269333	0.434269333	1.07E-07
6h	ACA1_037730/clean/scf7180000084568:9257-9331:+	ACA1_037730	Transmembrane protein, putative [Source:UniProtKB/TrEMBL;Acc:L8GN13]	0.166666667	0.600884333	0.434217667	0.002957484
6h	ACA1_024200/clean/scf7180000084441:15319-15408:-	ACA1_024200	WD_REPEATS_REGION domain-containing protein [Source:UniProtKB/TrEMBL;Acc:L8GKS0]	0.009073233	0.443123667	0.434050433	5.48E-06
6h	ACA1_082130/clean/scf7180000084623:2925-3013:+	ACA1_082130	hypothetical protein	0	0.434047333	0.434047333	6.72E-07
6h	ACA1_073230/clean/scf7180000084603:1376077-1376161:-	ACA1_073230	EHN domain-containing protein [Source:UniProtKB/TrEMBL;Acc:L8HDT9]	0.012045983	0.445986667	0.433940683	6.96E-09
6h	ACA1_174900/clean/scf7180000084741:826211-826287:-	ACA1_174900	Rasrelated protein Rab-32, putative [Source:UniProtKB/TrEMBL;Acc:L8HHY7]	0.00426679	0.438204667	0.433937877	5.96E-27
6h	ACA1_360220/clean/scf7180000084622:70950-71021:+	ACA1_360220	Ribonucloprotein [Source:UniProtKB/TrEMBL;Acc:L8HCP6]	0.001443471	0.435373	0.433929529	1.90E-28
6h	ACA1_030750/clean/scf7180000084505:19510-19576:+	ACA1_030750	Ribonuclease P protein subunit p20 [Source:UniProtKB/TrEMBL;Acc:L8HKS0]	0.008734327	0.442641	0.433906673	2.08E-06
6h	ACA1_114500/clean/scf7180000084669:487740-487839:-	ACA1_114500	Macro domain-containing protein [Source:UniProtKB/TrEMBL;Acc:L8H6J5]	0.001397043	0.435249	0.433851957	1.17E-11
6h	ACA1_158550/clean/scf7180000084724:248957-249036:+	ACA1_158550	Enoyl-CoA hydratase, putative [Source:UniProtKB/TrEMBL;Acc:L8HA73]	0.000900537	0.434739333	0.433838797	4.23E-11
6h	ACA1_362720/clean/scf7180000084624:159751-159841:-	ACA1_362720	Neutral ceramidase [Source:UniProtKB/TrEMBL;Acc:L8GFC5]	0.000768577	0.434412333	0.433643756	6.75E-15
6h	ACA1_191850/clean/scf7180000084749:59090-59170:-	ACA1_191850	Protein kinase domain-containing protein [Source:UniProtKB/TrEMBL;Acc:L8GN20]	0.137960333	0.571581667	0.433621333	9.66E-10
6h	ACA1_117300/clean/scf7180000084669:971884-971959:-	ACA1_117300	hypothetical protein	0.008255493	0.441860667	0.433605173	1.20E-07
6h	ACA1_109790/clean/scf7180000084663:28021-28187:+	ACA1_109790	Delta-1-pyrroline-5-carboxylate synthase [Source:UniProtKB/TrEMBL;Acc:L8HJZ5]	0.01056127	0.444162333	0.433601063	3.75E-10

6h	ACA1_191700/clean/scf7180000084749:33707-33798:-	ACA1_191700	Aldo_ket_red domain-containing protein [Source:UniProtKB/TrEMBL;Acc:L8GNA1]	0.010419563	0.443920333	0.43350077	2.65E-09
6h	ACA1_112000/clean/scf7180000084669:80725-80858:+	ACA1_112000	Rho-GAP domain-containing protein [Source:UniProtKB/TrEMBL;Acc:L8H3W7]	0.004524253	0.437989667	0.433465413	4.40E-09
6h	ACA1_244850/clean/scf7180000084763:438630-438712:+	ACA1_244850	EF-hand domain-containing protein [Source:UniProtKB/TrEMBL;Acc:L8GK26]	0.034132033	0.467588667	0.433456633	4.49E-09
6h	ACA1_128500/clean/scf7180000084697:73459-73584:-	ACA1_128500	UBIQUITIN_CONJUGAT_2 domain-containing protein [Source:UniProtKB/TrEMBL;Acc:L8GWJ5]	0.01447955	0.447935667	0.433456117	9.08E-09
6h	ACA1_084390/clean/scf7180000084631:17573-17664:-	ACA1_084390	hypothetical protein	0	0.433441667	0.433441667	0.015313755
6h	ACA1_158500/clean/scf7180000084724:239620-239729:+	ACA1_158500	Nudix hydrolase domain-containing protein [Source:UniProtKB/TrEMBL;Acc:L8HA53]	0.008644767	0.442079667	0.4334349	2.77E-06
6h	ACA1_289770/clean/scf7180000084777:461570-461672:-	ACA1_289770	Type I phosphodiesterase/nucleotide pyrophosphatase family protein [Source:UniProtKB/TrEMBL;Acc:L8HI22]	0.020903347	0.454133	0.433229653	1.12E-09
6h	ACA1_188290/clean/scf7180000084747:146175-146239:+	ACA1_188290	IPT/TIG domain-containing protein [Source:UniProtKB/TrEMBL;Acc:L8GSG4]	0.00568811	0.438830667	0.433142557	1.61E-14
6h	ACA1_024630/clean/scf7180000084444:1859-1932:+	ACA1_024630	hypothetical protein	0.005777743	0.4389	0.433122257	4.70E-09
6h	ACA1_315070/clean/scf7180000083887:27826-27897:+	ACA1_315070	Isobutyryl-CoA dehydrogenase, putative [Source:UniProtKB/TrEMBL;Acc:L8H248]	0	0.433107	0.433107	8.41E-13
6h	ACA1_182250/clean/scf7180000084745:164720-164798:-	ACA1_182250	hypothetical protein	0.0170799	0.450006333	0.432926433	2.11E-06
6h	ACA1_230510/clean/scf7180000084761:369220-369307:-	ACA1_230510	hypothetical protein	0.015873	0.448599667	0.432726667	5.97E-07
6h	ACA1_229230/clean/scf7180000084761:247497-247594:-	ACA1_229230	Mitochondrial carrier protein [Source:UniProtKB/TrEMBL;Acc:L8HB72]	0.006660153	0.439162667	0.432502513	2.22E-19
6h	ACA1_398880/clean/scf7180000084759:238990-239064:-	ACA1_398880	RNA_ligase domain-containing protein [Source:UniProtKB/TrEMBL;Acc:L8HCD5]	0.0008658	0.433214667	0.432348867	8.15E-08
6h	ACA1_243850/clean/scf7180000084763:361546-361624:+	ACA1_243850	Ras-GAP domain-containing protein [Source:UniProtKB/TrEMBL;Acc:L8GJZ5]	0.055555667	0.487802333	0.432246667	0.01174874
6h	ACA1_263870/clean/scf7180000084770:124317-124453:-	ACA1_263870	MAGE domain-containing protein [Source:UniProtKB/TrEMBL;Acc:L8H1Y1]	0	0.432229	0.432229	2.15E-06
6h	ACA1_321180/clean/scf7180000084437:7343-7447:+	ACA1_321180	PH domain containing protein [Source:UniProtKB/TrEMBL;Acc:L8GIK9]	0.01052996	0.442547667	0.432017707	1.09E-06
6h	ACA1_399930/clean/scf7180000084775:70212-70322:+	ACA1_399930	Ras subfamily protein [Source:UniProtKB/TrEMBL;Acc:L8GGR5]	0.0276089	0.459508333	0.431899433	7.97E-11
6h	ACA1_133170/clean/scf7180000084701:15608-15701:+	ACA1_133170	Threonine synthase [Source:UniProtKB/TrEMBL;Acc:L8H3M8]	0.01011526	0.441933333	0.431818073	1.16E-10
6h	ACA1_191150/clean/scf7180000084748:186629-186685:+	ACA1_191150	Triosephosphate isomerase [Source:UniProtKB/TrEMBL;Acc:L8GE90]	0.001296613	0.433113	0.431816387	7.59E-12
6h	ACA1_090140/clean/scf7180000084639:446432-446509:-	ACA1_090140	hypothetical protein	0.052520967	0.484318333	0.431797367	0.001323654
6h	ACA1_042850/clean/scf7180000084573:106349-106462:+	ACA1_042850	hypothetical protein	0.000795433	0.432579	0.431783567	1.80E-08
6h	ACA1_085170/clean/scf7180000084632:63626-63722:+	ACA1_085170	TPR_REGION domain-containing protein [Source:UniProtKB/TrEMBL;Acc:L8HM05]	0.001047197	0.432531	0.431483803	9.62E-12
6h	ACA1_100920/clean/scf7180000084649:18083-18170:-	ACA1_100920	WD_REPEATS_REGION domain-containing protein [Source:UniProtKB/TrEMBL;Acc:L8GIR5]	0	0.431444667	0.431444667	2.00E-09
6h	ACA1_323110/clean/scf7180000084467:4257-4360:+	ACA1_323110	hypothetical protein	0.0076524	0.439058	0.4314056	3.23E-06
6h	ACA1_114300/clean/scf7180000084669:456367-456534:+	ACA1_114300	Poly(ADPribose) polymerase catalytic domain containing protein [Source:UniProtKB/TrEMBL;Acc:L8H6I0]	0.005036583	0.436422333	0.43138575	1.07E-07
6h	ACA1_109100/clean/scf7180000084661:115886-115959:-	ACA1_109100	Sodium symporter, putative [Source:UniProtKB/TrEMBL;Acc:L8GC73]	0.005653567	0.436858	0.431204433	2.22E-05
6h	ACA1_203970/clean/scf7180000084754:241232-241314:-	ACA1_203970	HECTdomain (Ubiquitin-transferase) domain containing protein [Source:UniProtKB/TrEMBL;Acc:L8GTT3]	0.0070922	0.438289	0.4311968	6.80E-07
6h	ACA1_287950/clean/scf7180000084777:132024-132103:-	ACA1_287950	hypothetical protein	0.005016949	0.436091333	0.431074384	3.12E-08
6h	ACA1_184110/clean/scf7180000084745:561552-561644:+	ACA1_184110	hypothetical protein	0.010101	0.441152	0.431051	5.80E-06
6h	ACA1_266510/clean/scf7180000084770:684211-684320:-	ACA1_266510	RhoGEF domain containing protein [Source:UniProtKB/TrEMBL;Acc:L8H2E9]	0.081123533	0.512093333	0.4309698	2.64E-07
6h	ACA1_371700/clean/scf7180000084664:586002-586110:-	ACA1_371700	Ubiquitin carboxyl-terminal hydrolase [Source:UniProtKB/TrEMBL;Acc:L8H1Q6]	0	0.430888667	0.430888667	6.22E-07
6h	ACA1_164040/clean/scf7180000084729:145873-145971:-	ACA1_164040	Signal peptide binding domain containing protein [Source:UniProtKB/TrEMBL;Acc:L8GRG0]	0.0061031	0.436889333	0.430786233	4.33E-08
6h	ACA1_360290/clean/scf7180000084622:83775-83991:+	ACA1_360290	Singlestrand binding protein family [Source:UniProtKB/TrEMBL;Acc:L8HBV6]	0.015296653	0.446078	0.430781347	7.05E-18
6h	ACA1_183530/clean/scf7180000084745:446232-446333:-	ACA1_183530	Clu domain-containing protein [Source:UniProtKB/TrEMBL;Acc:L8H9K5]	0.0095419	0.440258333	0.430716433	6.54E-06
6h	ACA1_189600/clean/scf7180000084748:62669-62743:-	ACA1_189600	hypothetical protein	0.001689933	0.432398	0.430708067	1.61E-13
6h	ACA1_287810/clean/scf7180000084777:85064-85137:+	ACA1_287810	HemolysinIII related subfamily protein [Source:UniProtKB/TrEMBL;Acc:L8HIR3]	0.006158133	0.436819333	0.4306612	8.09E-08
6h	ACA1_129510/clean/scf7180000084698:18795-18888:+	ACA1_129510	Hydrolase_4 domain-containing protein [Source:UniProtKB/TrEMBL;Acc:L8GRQ5]	0.013790773	0.444413	0.430622227	8.60E-09
6h	ACA1_171980/clean/scf7180000084741:213092-213166:-	ACA1_171980	Ras subfamily protein [Source:UniProtKB/TrEMBL;Acc:L8HGG9]	0.029042033	0.459606667	0.430564633	2.40E-05
6h	ACA1_143580/clean/scf7180000084709:40489-40576:-	ACA1_143580	Med12 domain-containing protein [Source:UniProtKB/TrEMBL;Acc:L8HH02]	0.017287291	0.447643667	0.430356376	4.54E-12
6h	ACA1_200010/clean/scf7180000084753:368354-368532:+	ACA1_200010	Protein kinase domain containing protein [Source:UniProtKB/TrEMBL;Acc:L8H3C5]	0.00676138	0.437059333	0.430297953	2.10E-09
6h	ACA1_173060/clean/scf7180000084741:394159-394234:-	ACA1_173060	SNARE associated Golgi protein [Source:UniProtKB/TrEMBL;Acc:L8HGL7]	0	0.430294	0.430294	1.53E-07
6h	ACA1_369100/clean/scf7180000084664:244326-244390:+	ACA1_369100	IPPc domain-containing protein [Source:UniProtKB/TrEMBL;Acc:L8H092]	0	0.430235	0.430235	2.16E-07
6h	ACA1_257990/clean/scf7180000084769:197272-197394:+	ACA1_257990	hypothetical protein	0	0.430187	0.430187	0.007697215
6h	ACA1_260890/clean/scf7180000084769:548509-548616:+	ACA1_260890	F-box domain-containing protein [Source:UniProtKB/TrEMBL;Acc:L8GFC7]	0.006781899	0.436850333	0.430068435	1.90E-07
6h	ACA1_376180/clean/scf7180000084685:44633-44803:-	ACA1_376180	hypothetical protein	0.191937	0.621971333	0.430034333	1.76E-07
6h	ACA1_385410/clean/scf7180000084730:165251-165371:+	ACA1_385410	TRANSKETOLASE_1 domain-containing protein [Source:UniProtKB/TrEMBL;Acc:L8H9A8]	0.003729587	0.433562	0.429832413	1.15E-20
6h	ACA1_179970/clean/scf7180000084744:12494-12615:+	ACA1_179970	PPM-type phosphatase domain-containing protein [Source:UniProtKB/TrEMBL;Acc:L8GF73]	0.007872093	0.437691333	0.42981924	3.10E-14
6h	ACA1_276900/clean/scf7180000084774:249299-249383:-	ACA1_276900	unspecified product	0	0.429816333	0.429816333	5.52E-08
6h	ACA1_019960/clean/scf7180000084033:1032-1099:+	ACA1_019960	SWIRM domain containing protein [Source:UniProtKB/TrEMBL;Acc:L8GUS1]	0.006468607	0.436267333	0.429798727	5.48E-13
6h	ACA1_256380/clean/scf7180000084769:14878-14950:-	ACA1_256380	UBA/TS-N domain containing protein [Source:UniProtKB/TrEMBL;Acc:L8GF49]	0.00049554	0.430251	0.42975546	5.06E-10
6h	ACA1_215620/clean/scf7180000084758:350379-350487:-	ACA1_215620	Rasrelated GTPase [Source:UniProtKB/TrEMBL;Acc:L8GSM1]	0.00965641	0.439240667	0.429584257	1.61E-14
6h	ACA1_389080/clean/scf7180000084733:192798-192908:-	ACA1_389080	Fbox domain containing protein [Source:UniProtKB/TrEMBL;Acc:L8GDU7]	0.004718567	0.434297	0.429578433	5.74E-06
6h	ACA1_310750/clean/scf7180000083780:6473-6541:+	ACA1_310750	RFX-type winged-helix domain-containing protein [Source:UniProtKB/TrEMBL;Acc:L8GVY4]	0.012411822	0.441969333	0.429557511	5.26E-07
6h	ACA1_227780/clean/scf7180000084761:135439-135518:+	ACA1_227780	NAD(P)-bd_dom domain-containing protein [Source:UniProtKB/TrEMBL;Acc:L8H8P3]	0.010168533	0.439656	0.429487467	3.00E-09
6h	ACA1_272730/clean/scf7180000084772:108098-108184:-	ACA1_272730	Ubiquitin carboxyl-terminal hydrolase [Source:UniProtKB/TrEMBL;Acc:L8HFF3]	0.004985717	0.434398667	0.42941295	1.84E-08
6h	ACA1_376730/clean/scf7180000084685:147951-148022:-	ACA1_376730	Glutamate dehydrogenase [Source:UniProtKB/TrEMBL;Acc:L8HFB5]	0.001567335	0.430968	0.429400665	4.67E-21
6h	ACA1_173980/clean/scf7180000084741:564374-564461:-	ACA1_173980	OTU domain-containing protein [Source:UniProtKB/TrEMBL;Acc:L8HHS8]	0.003144653	0.432451667	0.429307013	1.29E-08
6h	ACA1_096600/clean/scf7180000084645:1059470-1059550:+	ACA1_096600	CUE domain containing protein [Source:UniProtKB/TrEMBL;Acc:L8GJT0]	0.007237693	0.436438333	0.42920064	9.66E-14
6h	ACA1_106800/clean/scf7180000084660:132767-132880:+	ACA1_106800	EF hand domain containing protein [Source:UniProtKB/TrEMBL;Acc:L8GNA6]	0.003384188	0.432542667	0.429158479	2.39E-09
6h	ACA1_372660/clean/scf7180000084679:39894-39985:-	ACA1_372660	hypothetical protein	0.00045662	0.429593	0.42913638	0.000112546
6h	ACA1_110140/clean/scf7180000084663:73668-73797:-	ACA1_110140	hypothetical protein	0.005383203	0.434505667	0.429122463	3.97E-12
6h	ACA1_100930/clean/scf7180000084649:23543-23628:+	ACA1_100930	DNA topoisomerase [Source:UniProtKB/TrEMBL;Acc:L8GGF9]	0	0.429008667	0.429008667	2.43E-09
6h	ACA1_286130/clean/scf7180000084776:1036910-1036979:-	ACA1_286130	Clu domain-containing protein [Source:UniProtKB/TrEMBL;Acc:L8H7K1]	0	0.428934	0.428934	5.55E-08
6h	ACA1_343140/clean/scf7180000084638:1909-1996:-	ACA1_343140	CN hydrolase domain-containing protein [Source:UniProtKB/TrEMBL;Acc:L8H9J3]	0.0088408	0.437751667	0.428910867	7.48E-11
6h	ACA1_085210/clean/scf7180000084632:74539-74613:+	ACA1_085210	ThiF domain-containing protein [Source:UniProtKB/TrEMBL;Acc:L8HKE8]	0.005543083	0.434368	0.428824917	2.01E-09
6h	ACA1_123360/clean/scf7180000084686:20295-20388:+	ACA1_123360	Zn(2)-C6 fungal-type domain-containing protein [Source:UniProtKB/TrEMBL;Acc:L8HF21]	0	0.428802	0.428802	4.58E-10
6h	ACA1_078930/clean/scf7180000084615:97902-98060:-	ACA1_078930	RNA recognition motif domain containing protein [Source:UniProtKB/TrEMBL;Acc:L8GT65]	0.015841653	0.444593	0.428751347	6.44E-08
6h	ACA1_308770/clean/scf7180000083743:16896-16961:-	ACA1_308770	Fumarylacetoacetase [Source:UniProtKB/TrEMBL;Acc:L8HKF9]	0.001785485	0.430508	0.428722515	9.46E-23
6h	ACA1_252780/clean/scf7180000084768:208290-208539:-	ACA1_252780	Sel1 repeatcontaining protein [Source:UniProtKB/TrEMBL;Acc:L8HC46]	0.0751295	0.503810333	0.428680833	7.16E-11
6h	ACA1_172150/clean/scf7180000084741:257238-257310:+	ACA1_172150	Outer membrane autotransporter, putative [Source:UniProtKB/TrEMBL;Acc:L8HHJ4]	0.06083538	0.489399667	0.428564287	1.55E-08
6h	ACA1_215750/clean/scf7180000084758:387576-387695:+	ACA1_215750	CBS domain containing protein [Source:UniProtKB/TrEMBL;Acc:L8GR40]	0.0224585	0.450916333	0.428457833	5.37E-21
6h	ACA1_229540/clean/scf7180000084761:271858-271980:-	ACA1_229540	HD phosphohydrolase, putative [Source:UniProtKB/TrEMBL;Acc:L8HB82]	0.134404333	0.562860667	0.428456333	1.47E-09
6h	ACA1_341560/clean/scf7180000084628:4069-4138:+	ACA1_341560	CBS domain containing protein [Source:UniProtKB/TrEMBL;Acc:L8GL09]	0.149759	0.578133333	0.428374333	8.11E-07
6h	ACA1_037730/clean/scf7180000084568:10181-10253:+	ACA1_037730	Transmembrane protein, putative [Source:UniProtKB/TrEMBL;Acc:L8GN13]	0.149152433	0.577355	0.428202567	0.004361491
6h	ACA1_151300/clean/scf7180000084716:95958-96130:+	ACA1_151300	TIM21 protein [Source:UniProtKB/TrEMBL;Acc:L8H0R8]	0.005946027	0.434101	0.428154973	3.14E-08
6h	ACA1_372660/clean/scf7180000084679:41225-41299:-	ACA1_372660	hypothetical protein	0.050653947	0.478808333	0.428154387	0.001801503
6h	ACA1_234820/clean/scf7180000084762:324776-324861:-	ACA1_234820	Serine/threonine-protein phosphatase [Source:UniProtKB/TrEMBL;Acc:L8H0J1]	0.010178133	0.438315333	0.4281372	6.49E-12
6h	ACA1_267280/clean/scf7180000084770:842495-842635:+	ACA1_267280	Coatomer subunit gamma [Source:UniProtKB/TrEMBL;Acc:L8H1U2]	0.00244314	0.430547333	0.428104193	1.24E-09
6h	ACA1_078990/clean/scf7180000084615:125424-125539:-	ACA1_078990	AcylCoA synthetase, putative [Source:UniProtKB/TrEMBL;Acc:L8GRK9]	0	0.428019667	0.428019667	1.42E-15
6h	ACA1_088960/clean/scf7180000084639:274349-274431:-	ACA1_088960	Beta-lactamase domain-containing protein [Source:UniProtKB/TrEMBL;Acc:L8GU04]	0	0.427933333	0.427933333	2.15E-07
6h	ACA1_087730/clean/scf7180000084639:110278-110349:+	ACA1_087730	Armadillo/betacatenin-like repeat domain containing protein [Source:UniProtKB/TrEMBL;Acc:L8GTW4]	0	0.427733	0.427733	7.29E-08
6h	ACA1_290650/clean/scf7180000084777:646292-646368:+	ACA1_290650	DENN domain-containing protein [Source:UniProtKB/TrEMBL;Acc:L8HI74]	0.013698633	0.441394667	0.427696033	4.00E-05
6h	ACA1_171690/clean/scf7180000084741:144691-144805:+	ACA1_171690	L51_S25_CI-B8 domain-containing protein [Source:UniProtKB/TrEMBL;Acc:L8HHF4]	0.002449669	0.4301	0.427650331	1.75E-10
6h	ACA1_369100/clean/scf7180000084664:242876-242964:+	ACA1_369100	IPPc domain-containing protein [Source:UniProtKB/TrEMBL;Acc:L8H092]	0.005841533	0.433473333	0.4276318	4.93E-07
6h	ACA1_158810/clean/scf7180000084724:258771-258859:-	ACA1_158810	Coatomer subunit epsilon [Source:UniProtKB/TrEMBL;Acc:L8HCK7]	0.007491387	0.435122333	0.427630947	5.89E-17
6h	ACA1_209530/clean/scf7180000084757:302907-302981:-	ACA1_209530	Purple acid phosphatase [Source:UniProtKB/TrEMBL;Acc:L8GZQ1]	0.031618227	0.459146667	0.42752844	1.15E-08
6h	ACA1_111310/clean/scf7180000084669:2486-2561:+	ACA1_111310	hypothetical protein	0.019854133	0.447330333	0.4274762	2.57E-06
6h	ACA1_224390/clean/scf7180000084760:191867-192021:-	ACA1_224390	hypothetical protein	0.003436433	0.430868	0.427431567	1.63E-08
6h	ACA1_024390/clean/scf7180000084442:10495-10567:+	ACA1_024390	hypothetical protein	0	0.42742	0.42742	6.68E-12
6h	ACA1_045630/clean/scf7180000084576:165810-165880:-	ACA1_045630	Deacetylase sirtuin-type domain-containing protein [Source:UniProtKB/TrEMBL;Acc:L8GZU7]	0.012407833	0.439739	0.427331167	1.13E-06
6h	ACA1_199050/clean/scf7180000084753:206369-206497:-	ACA1_199050	Pentatricopeptide repeat domain containing protein [Source:UniProtKB/TrEMBL;Acc:L8H4K3]	0.034146767	0.461266333	0.427119567	3.91E-09
6h	ACA1_183130/clean/scf7180000084745:338850-338939:-	ACA1_183130	PNP_UDP_1 domain-containing protein [Source:UniProtKB/TrEMBL;Acc:L8H767]	0.004046854	0.431057	0.427010146	5.48E-14
6h	ACA1_114870/clean/scf7180000084669:569913-570047:+	ACA1_114870	TAF4 domain-containing protein [Source:UniProtKB/TrEMBL;Acc:L8H4I0]	0	0.426953	0.426953	5.73E-08
6h	ACA1_321030/clean/scf7180000084435:22889-22989:-	ACA1_321030	hypothetical protein	0.001424261	0.428369333	0.426945072	5.13E-26
6h	ACA1_175520/clean/scf7180000084741:984256-984334:-	ACA1_175520	PH domain-containing protein [Source:UniProtKB/TrEMBL;Acc:L8HI32]	0.030303033	0.457218667	0.426915633	8.84E-05
6h	ACA1_155100/clean/scf7180000084722:105294-105350:+	ACA1_155100	EF-hand domain-containing protein [Source:UniProtKB/TrEMBL;Acc:L8GZ97]	0.001761443	0.428575333	0.42681389	4.77E-19
6h	ACA1_126180/clean/scf7180000084693:46167-46237:+	ACA1_126180	BTB domain-containing protein [Source:UniProtKB/TrEMBL;Acc:L8HBT3]	0	0.426795333	0.426795333	2.41E-11
6h	ACA1_211150/clean/scf7180000084758:78679-78738:+	ACA1_211150	Metallophos domain-containing protein [Source:UniProtKB/TrEMBL;Acc:L8GQ03]	0.01892964	0.445619333	0.426689693	6.19E-12
6h	ACA1_272960/clean/scf7180000084773:4513-4572:-	ACA1_272960	Ras-GAP domain-containing protein [Source:UniProtKB/TrEMBL;Acc:L8GFH7]	0	0.426520667	0.426520667	2.62E-12
6h	ACA1_061670/clean/scf7180000084599:1001738-1001828:-	ACA1_061670	SSD domain-containing protein [Source:UniProtKB/TrEMBL;Acc:L8GWV2]	0	0.42652	0.42652	2.75E-08
6h	ACA1_127880/clean/scf7180000084697:8703-8768:+	ACA1_127880	Bis(5'-adenosyl)-triphosphatase [Source:UniProtKB/TrEMBL;Acc:L8GUT1]	0.027222233	0.453737667	0.426515433	6.11E-06
6h	ACA1_138510/clean/scf7180000084703:155595-155698:+	ACA1_138510	TGc domain-containing protein [Source:UniProtKB/TrEMBL;Acc:L8GYR5]	0	0.426439333	0.426439333	3.48E-08
6h	ACA1_288440/clean/scf7180000084777:206799-206860:-	ACA1_288440	DH domain-containing protein [Source:UniProtKB/TrEMBL;Acc:L8HKE3]	0.055185233	0.481585667	0.426400433	3.42E-06
6h	ACA1_256450/clean/scf7180000084769:36279-36374:+	ACA1_256450	Poly [ADP-ribose] polymerase [Source:UniProtKB/TrEMBL;Acc:L8GH34]	0.0309457	0.457338333	0.426392633	3.69E-06
6h	ACA1_185320/clean/scf7180000084746:93897-93963:+	ACA1_185320	hypothetical protein	0.005339447	0.431603667	0.42626422	4.64E-15
6h	ACA1_365380/clean/scf7180000084641:257430-257515:+	ACA1_365380	PHB depolymerase [Source:UniProtKB/TrEMBL;Acc:L8GLL3]	0.009127077	0.435322333	0.426195257	2.25E-06
6h	ACA1_038390/clean/scf7180000084568:138656-138759:+	ACA1_038390	R3H domain-containing protein [Source:UniProtKB/TrEMBL;Acc:L8GLF9]	0.004257807	0.430414333	0.426156527	6.81E-08
6h	ACA1_117020/clean/scf7180000084669:892992-893071:+	ACA1_117020	Carboxypeptidase [Source:UniProtKB/TrEMBL;Acc:L8H3Z1]	0	0.426120333	0.426120333	1.21E-12
6h	ACA1_187470/clean/scf7180000084747:73317-73370:-	ACA1_187470	hypothetical protein	0.03908114	0.465142	0.42606086	2.24E-07
6h	ACA1_068120/clean/scf7180000084603:111854-111934:+	ACA1_068120	Endo/exonuclease/phosphatase domain-containing protein [Source:UniProtKB/TrEMBL;Acc:L8HEN2]	0.012494693	0.438524	0.426029307	2.08E-08
6h	ACA1_040710/clean/scf7180000084572:56753-56854:+	ACA1_040710	AAA domain-containing protein [Source:UniProtKB/TrEMBL;Acc:L8H0J4]	0.007756497	0.433762667	0.42600617	2.15E-09
6h	ACA1_320790/clean/scf7180000084416:22475-22607:-	ACA1_320790	RUN domain-containing protein [Source:UniProtKB/TrEMBL;Acc:L8GTR9]	0	0.425997333	0.425997333	1.11E-11
6h	ACA1_331590/clean/scf7180000084578:7020-7162:+	ACA1_331590	ATPdependent DNA helicase, RecQ subfamily protein [Source:UniProtKB/TrEMBL;Acc:L8HBM3]	0.028912767	0.454780333	0.425867567	1.46E-05
6h	ACA1_050990/clean/scf7180000084592:3034-3105:+	ACA1_050990	Protein phosphatase [Source:UniProtKB/TrEMBL;Acc:L8GQI4]	0.007928993	0.433756667	0.425827673	1.50E-07
6h	ACA1_171690/clean/scf7180000084741:144351-144449:+	ACA1_171690	L51_S25_CI-B8 domain-containing protein [Source:UniProtKB/TrEMBL;Acc:L8HHF4]	0.001718213	0.427486333	0.42576812	1.07E-09
6h	ACA1_292880/clean/scf7180000084777:924577-924698:-	ACA1_292880	RhoGEF domain containing protein [Source:UniProtKB/TrEMBL;Acc:L8HJL4]	0.022560867	0.448308667	0.4257478	1.30E-07
6h	ACA1_227100/clean/scf7180000084761:98617-98696:-	ACA1_227100	hypothetical protein	0.025641033	0.451244	0.425602967	0.002337919
6h	ACA1_251330/clean/scf7180000084768:57419-57497:+	ACA1_251330	CHHC U11-48K-type domain-containing protein [Source:UniProtKB/TrEMBL;Acc:L8HAJ2]	0.0473356	0.472857	0.4255214	0.000900319
6h	ACA1_150720/clean/scf7180000084716:23953-24031:-	ACA1_150720	TPR_REGION domain-containing protein [Source:UniProtKB/TrEMBL;Acc:L8H0N3]	0	0.425462	0.425462	2.30E-11
6h	ACA1_288310/clean/scf7180000084777:166872-166972:-	ACA1_288310	hypothetical protein	0.0187606	0.444132333	0.425371733	1.31E-07
6h	ACA1_400560/clean/scf7180000084775:193886-193980:+	ACA1_400560	hypothetical protein	0.004551233	0.429813	0.425261767	4.51E-06
6h	ACA1_326150/clean/scf7180000084504:37268-37374:+	ACA1_326150	PI3K/PI4K domain-containing protein [Source:UniProtKB/TrEMBL;Acc:L8HJE7]	0.002931803	0.428128	0.425196197	7.47E-09
6h	ACA1_301230/clean/scf7180000083203:5071-5169:-	ACA1_301230	hypothetical protein	0	0.424903333	0.424903333	3.94E-09
6h	ACA1_054710/clean/scf7180000084594:559354-559408:-	ACA1_054710	Ras family protein [Source:UniProtKB/TrEMBL;Acc:L8H6F4]	0.009223967	0.433887333	0.424663367	7.05E-16
6h	ACA1_037190/clean/scf7180000084567:79244-79301:+	ACA1_037190	Aminotran_5 domain-containing protein [Source:UniProtKB/TrEMBL;Acc:L8H0V4]	0.000526851	0.425171	0.424644149	1.43E-12
6h	ACA1_295690/clean/scf7180000084777:1195605-1195700:-	ACA1_295690	NTP_transferase domain-containing protein [Source:UniProtKB/TrEMBL;Acc:L8HLD2]	0.003174603	0.427809667	0.424635063	9.60E-11
6h	ACA1_088650/clean/scf7180000084639:189445-189511:-	ACA1_088650	Myotubularin phosphatase domain-containing protein [Source:UniProtKB/TrEMBL;Acc:L8GUG2]	0.0124883	0.436934333	0.424446033	3.46E-06
6h	ACA1_219720/clean/scf7180000084758:787470-787552:+	ACA1_219720	Bromodomain domain containing protein [Source:UniProtKB/TrEMBL;Acc:L8GSZ1]	0.012667043	0.437102667	0.424435623	9.57E-07
6h	ACA1_189340/clean/scf7180000084748:11515-11605:-	ACA1_189340	Dyptype peroxidase superfamily protein [Source:UniProtKB/TrEMBL;Acc:L8GDU2]	0.000686466	0.425117333	0.424430868	5.29E-16
6h	ACA1_208170/clean/scf7180000084757:117964-118044:+	ACA1_208170	hypothetical protein	0.0038108	0.428161667	0.424350867	7.74E-09
6h	ACA1_237860/clean/scf7180000084763:34198-34290:+	ACA1_237860	Glycerophosphodiester phosphodiesterase family protein [Source:UniProtKB/TrEMBL;Acc:L8GKN2]	0.000649927	0.424906333	0.424256407	2.45E-12
6h	ACA1_252770/clean/scf7180000084768:206652-206753:-	ACA1_252770	Peripla_BP_6 domain-containing protein [Source:UniProtKB/TrEMBL;Acc:L8HAP5]	0	0.424225333	0.424225333	5.82E-11
6h	ACA1_234610/clean/scf7180000084762:304780-304857:-	ACA1_234610	Peptidyl-prolyl cis-trans isomerase [Source:UniProtKB/TrEMBL;Acc:L8H2P2]	0.004888843	0.429035333	0.42414649	1.54E-26
6h	ACA1_204000/clean/scf7180000084754:245238-245313:-	ACA1_204000	hypothetical protein	0.005129933	0.429116	0.423986067	3.97E-09
6h	ACA1_058590/clean/scf7180000084599:357646-357714:+	ACA1_058590	Copine VIII, putative [Source:UniProtKB/TrEMBL;Acc:L8GXA8]	0	0.423985333	0.423985333	4.43E-14
6h	ACA1_209790/clean/scf7180000084757:323051-323205:-	ACA1_209790	AMPbinding enzyme domain containing protein [Source:UniProtKB/TrEMBL;Acc:L8GXL7]	0.083049167	0.507028667	0.4239795	7.30E-07
6h	ACA1_099100/clean/scf7180000084646:91129-91197:+	ACA1_099100	PH domain-containing protein [Source:UniProtKB/TrEMBL;Acc:L8H5M3]	0.008333333	0.432292	0.423958667	2.88E-06

6h	ACA1_172250/clean/scf7180000084741:283363-283457:-	ACA1_172250	Leucine rich repeat domain containing protein [Source:UniProtKB/TrEMBL;Acc:L8HHK1]	0.256044	0.679673667	0.423629667	2.66E-05
6h	ACA1_002430/clean/scf7180000082931:156-243:-	ACA1_002430	G domain-containing protein [Source:UniProtKB/TrEMBL;Acc:L8GY39]	0.0070922	0.430692667	0.423600467	7.10E-07
6h	ACA1_333220/clean/scf7180000084581:21044-21173:+	ACA1_333220	hypothetical protein	0.006693743	0.430257	0.423563257	1.37E-09
6h	ACA1_069810/clean/scf7180000084603:565098-565179:+	ACA1_069810	hypothetical protein	0.001611083	0.425162	0.423550917	9.60E-09
6h	ACA1_287960/clean/scf7180000084777:134226-134309:-	ACA1_287960	SET domain-containing protein [Source:UniProtKB/TrEMBL;Acc:L8HIS3]	0	0.423547333	0.423547333	0.000135797
6h	ACA1_232420/clean/scf7180000084762:22625-22739:+	ACA1_232420	TPR_REGION domain-containing protein [Source:UniProtKB/TrEMBL;Acc:L8H3B0]	0.025789067	0.449315333	0.423526267	1.65E-12
6h	ACA1_260100/clean/scf7180000084769:428101-428230:-	ACA1_260100	Ras-GAP domain-containing protein [Source:UniProtKB/TrEMBL;Acc:L8GHJ9]	0.000258365	0.423771667	0.423513302	3.82E-16
6h	ACA1_396760/clean/scf7180000084759:128741-129027:+	ACA1_396760	Clu domain-containing protein [Source:UniProtKB/TrEMBL;Acc:L8HCD9]	0.052453567	0.475952667	0.4234991	2.49E-09
6h	ACA1_248410/clean/scf7180000084766:67266-67339:-	ACA1_248410	Alpha2-macroglobulin domain containing protein [Source:UniProtKB/TrEMBL;Acc:L8GY08]	0.006269717	0.429652667	0.42338295	2.22E-08
6h	ACA1_158530/clean/scf7180000084724:245827-245913:+	ACA1_158530	Glucose-6-phosphate 1-dehydrogenase [Source:UniProtKB/TrEMBL;Acc:L8HBJ1]	0.068929743	0.492283667	0.423353923	4.27E-05
6h	ACA1_250030/clean/scf7180000084767:26831-26922:-	ACA1_250030	C2 NT-type domain-containing protein [Source:UniProtKB/TrEMBL;Acc:L8HC94]	0.002141743	0.425421667	0.423279923	2.35E-08
6h	ACA1_142090/clean/scf7180000084708:85673-85755:+	ACA1_142090	rRNA metabolism protein, SBDS family [Source:UniProtKB/TrEMBL;Acc:L8HCP3]	0.0058801	0.428819667	0.422939567	3.96E-12
6h	ACA1_388780/clean/scf7180000084733:170483-170573:+	ACA1_388780	EGFlike domain containing protein [Source:UniProtKB/TrEMBL;Acc:L8GDT8]	0.001774279	0.424611	0.422836721	1.46E-13
6h	ACA1_158170/clean/scf7180000084724:201936-202068:-	ACA1_158170	Pescadillo homolog [Source:UniProtKB/TrEMBL;Acc:L8HBG6]	0.02122189	0.443966333	0.422744443	1.84E-13
6h	ACA1_260270/clean/scf7180000084769:469224-469318:+	ACA1_260270	Proteasome subunit alpha type [Source:UniProtKB/TrEMBL;Acc:L8GG17]	0.00454033	0.427282	0.42274167	5.56E-30
6h	ACA1_400260/clean/scf7180000084775:131653-131733:+	ACA1_400260	Ranlike small GTPase [Source:UniProtKB/TrEMBL;Acc:L8GIF4]	0.011000267	0.433586667	0.4225864	0.000313428
6h	ACA1_157770/clean/scf7180000084724:113451-113578:-	ACA1_157770	Uracil-DNA glycosylase [Source:UniProtKB/TrEMBL;Acc:L8HA19]	0.005698	0.428102333	0.422404333	2.20E-08
6h	ACA1_368150/clean/scf7180000084664:67691-67881:-	ACA1_368150	Synaptobrevin protein [Source:UniProtKB/TrEMBL;Acc:L8GY05]	0.017520667	0.439842333	0.422321667	9.64E-11
6h	ACA1_027780/clean/scf7180000084471:8790-8881:+	ACA1_027780	RasGEF domain containing protein [Source:UniProtKB/TrEMBL;Acc:L8HMK7]	0.000879507	0.423009	0.422129493	6.85E-11
6h	ACA1_289800/clean/scf7180000084777:466602-466662:-	ACA1_289800	J domain-containing protein [Source:UniProtKB/TrEMBL;Acc:L8HJ30]	0.041476067	0.463568	0.422091933	2.12E-05
6h	ACA1_174540/clean/scf7180000084741:731377-731485:-	ACA1_174540	Fucosylgalactoside 3-alpha-galactosyltransferase [Source:UniProtKB/TrEMBL;Acc:L8HJC5]	0.032875533	0.454689	0.421813467	1.79E-10
6h	ACA1_336380/clean/scf7180000084593:56143-56247:-	ACA1_336380	RasGEF domain containing protein [Source:UniProtKB/TrEMBL;Acc:L8H1N6]	0.00886883	0.430666	0.42179717	8.81E-16
6h	ACA1_172710/clean/scf7180000084741:298315-298392:-	ACA1_172710	Foie-gras_1 domain-containing protein [Source:UniProtKB/TrEMBL;Acc:L8HHK5]	0	0.421706667	0.421706667	2.61E-08
6h	ACA1_170640/clean/scf7180000084739:99812-99895:+	ACA1_170640	Protein kinase domain-containing protein [Source:UniProtKB/TrEMBL;Acc:L8HIR2]	0.031735923	0.453347333	0.42161141	2.84E-06
6h	ACA1_320840/clean/scf7180000084416:34222-34326:+	ACA1_320840	HSP91 (Heat shock protein 91), putative [Source:UniProtKB/TrEMBL;Acc:L8GTS5]	0.001204729	0.422768333	0.421563605	5.82E-29
6h	ACA1_059450/clean/scf7180000084599:449818-449920:+	ACA1_059450	Palmitoyltransferase [Source:UniProtKB/TrEMBL;Acc:L8GXD6]	0.032221463	0.453766	0.421544537	6.74E-08
6h	ACA1_025670/clean/scf7180000084451:2912-2969:-	ACA1_025670	DOMON domain containing protein [Source:UniProtKB/TrEMBL;Acc:L8GGT9]	0	0.421290333	0.421290333	3.52E-11
6h	ACA1_230510/clean/scf7180000084761:369005-369094:-	ACA1_230510	hypothetical protein	0.00426561	0.425481	0.42121539	6.65E-09
6h	ACA1_220070/clean/scf7180000084758:855121-855222:+	ACA1_220070	Ergosterol biosynthesis erg4/erg24 family protein [Source:UniProtKB/TrEMBL;Acc:L8GQK3]	0	0.421139333	0.421139333	1.28E-07
6h	ACA1_090960/clean/scf7180000084645:24753-24842:-	ACA1_090960	Protein kinase domain-containing protein [Source:UniProtKB/TrEMBL;Acc:L8GI25]	0.038081	0.459211667	0.421130667	4.01E-05
6h	ACA1_107160/clean/scf7180000084660:161296-161454:+	ACA1_107160	hypothetical protein	0.010222533	0.431335333	0.4211128	5.79E-11
6h	ACA1_265560/clean/scf7180000084770:509388-509464:+	ACA1_265560	RFX-type winged-helix domain-containing protein [Source:UniProtKB/TrEMBL;Acc:L8H4F0]	0.007135567	0.428169	0.421033433	1.39E-08
6h	ACA1_252880/clean/scf7180000084768:233963-234086:-	ACA1_252880	hypothetical protein	0.03600225	0.457000333	0.420998083	1.23E-09
6h	ACA1_379300/clean/scf7180000084711:327843-327910:-	ACA1_379300	hypothetical protein	0.006172833	0.427105667	0.420932833	6.58E-08
6h	ACA1_116270/clean/scf7180000084669:744118-744229:+	ACA1_116270	G_PROTEIN_RECEP_F1_2 domain-containing protein [Source:UniProtKB/TrEMBL;Acc:L8H6T9]	0.03894794	0.459862	0.42091406	1.55E-08
6h	ACA1_349670/clean/scf7180000084690:34494-34588:-	ACA1_349670	hypothetical protein	0.0057127	0.426604667	0.420891967	5.77E-10
6h	ACA1_112640/clean/scf7180000084669:203111-203196:+	ACA1_112640	O-acyltransferase [Source:UniProtKB/TrEMBL;Acc:L8H355]	0	0.420878333	0.420878333	1.56E-06
6h	ACA1_255900/clean/scf7180000084768:576990-577198:+	ACA1_255900	DEP domain-containing protein [Source:UniProtKB/TrEMBL;Acc:L8HA90]	0.001333652	0.422021333	0.420687682	1.40E-14
6h	ACA1_023890/clean/scf7180000084436:31824-31923:-	ACA1_023890	SAM domain-containing protein [Source:UniProtKB/TrEMBL;Acc:L8GSK7]	0.015161247	0.435835333	0.420674087	1.01E-12
6h	ACA1_372600/clean/scf7180000084679:23249-23331:-	ACA1_372600	F-box domain-containing protein [Source:UniProtKB/TrEMBL;Acc:L8GJA4]	0.0060606	0.426547333	0.420486733	9.69E-08
6h	ACA1_037740/clean/scf7180000084568:13444-13539:-	ACA1_037740	hypothetical protein	0.006745873	0.427063333	0.42031746	2.19E-10
6h	ACA1_392940/clean/scf7180000084756:10912-10993:-	ACA1_392940	hypothetical protein	0	0.420084333	0.420084333	3.01E-07
6h	ACA1_138520/clean/scf7180000084703:160269-160390:+	ACA1_138520	Phospholipase B-like [Source:UniProtKB/TrEMBL;Acc:L8GZK2]	0.004409033	0.424336667	0.419927633	1.32E-12
6h	ACA1_095060/clean/scf7180000084645:803777-803882:-	ACA1_095060	Sidoreflexin [Source:UniProtKB/TrEMBL;Acc:L8GL14]	0.008001413	0.427924667	0.419923253	4.08E-07
6h	ACA1_068620/clean/scf7180000084603:238624-238715:-	ACA1_068620	hypothetical protein	0.02484748	0.444769333	0.419921853	4.84E-09
6h	ACA1_018770/clean/scf7180000083912:14324-14394:+	ACA1_018770	Signal recognition particle subunit SRP68 [Source:UniProtKB/TrEMBL;Acc:L8HKJ5]	0.01571969	0.435593	0.41987331	8.35E-17
6h	ACA1_117270/clean/scf7180000084669:967092-967189:+	ACA1_117270	hypothetical protein	0.011208733	0.430997333	0.4197886	6.64E-11
6h	ACA1_173130/clean/scf7180000084741:408927-409049:-	ACA1_173130	hypothetical protein	0.155606667	0.575301667	0.419695	0.000344923
6h	ACA1_234220/clean/scf7180000084762:229096-229177:-	ACA1_234220	hypothetical protein	0.150936333	0.570585667	0.419649333	8.48E-07
6h	ACA1_065900/clean/scf7180000084599:1943053-1943142:-	ACA1_065900	Inositol-1-monophosphatase [Source:UniProtKB/TrEMBL;Acc:L8GWY8]	0.028310833	0.447780667	0.419469833	1.89E-10
6h	ACA1_154690/clean/scf7180000084722:78498-78651:+	ACA1_154690	unspecified product	0.005350623	0.424778	0.419427377	6.28E-10
6h	ACA1_053890/clean/scf7180000084594:344928-344996:-	ACA1_053890	Aconitate hydratase, mitochondrial [Source:UniProtKB/TrEMBL;Acc:L8H690]	0	0.419375667	0.419375667	4.99E-14
6h	ACA1_278460/clean/scf7180000084776:206742-206808:+	ACA1_278460	Peptidase_S8 domain-containing protein [Source:UniProtKB/TrEMBL;Acc:L8H6X0]	0.005747133	0.424999	0.419251867	1.49E-07
6h	ACA1_069190/clean/scf7180000084603:379154-379241:+	ACA1_069190	PfkB domain-containing protein [Source:UniProtKB/TrEMBL;Acc:L8HDR4]	0.065579167	0.484779	0.419199833	3.43E-09
6h	ACA1_191700/clean/scf7180000084749:34242-34378:-	ACA1_191700	Aldo_ket_red domain-containing protein [Source:UniProtKB/TrEMBL;Acc:L8GNA1]	0.00522	0.424263	0.419043	4.33E-09
6h	ACA1_295300/clean/scf7180000084777:1096068-1096174:-	ACA1_295300	Calponin domain containing protein [Source:UniProtKB/TrEMBL;Acc:L8HJQ6]	0.00081728	0.419804333	0.418987053	8.90E-11
6h	ACA1_182260/clean/scf7180000084745:165588-165718:-	ACA1_182260	SAM domain (Sterile alpha motif) domain containing protein [Source:UniProtKB/TrEMBL;Acc:L8H717]	0.003178217	0.422092333	0.418914117	7.28E-07
6h	ACA1_022080/clean/scf7180000084264:12378-12499:+	ACA1_022080	Ankyrin repeat RhoGAP domain containing protein [Source:UniProtKB/TrEMBL;Acc:L8GC83]	0.016666667	0.435510667	0.418844	9.96E-05
6h	ACA1_060380/clean/scf7180000084599:663950-664033:-	ACA1_060380	ATG13 domain-containing protein [Source:UniProtKB/TrEMBL;Acc:L8GYW8]	0.026904467	0.445688333	0.418783867	2.72E-06
6h	ACA1_107440/clean/scf7180000084660:197117-197262:+	ACA1_107440	Purple acid phosphatase [Source:UniProtKB/TrEMBL;Acc:L8GNJ7]	0.00263401	0.421225333	0.418591323	1.71E-18
6h	ACA1_174690/clean/scf7180000084741:763572-763645:+	ACA1_174690	Deacetylase sirtuin-type domain-containing protein [Source:UniProtKB/TrEMBL;Acc:L8HHW7]	0.004487167	0.423016	0.418528833	2.75E-08
6h	ACA1_208500/clean/scf7180000084757:210179-210290:+	ACA1_208500	Aldedh domain-containing protein [Source:UniProtKB/TrEMBL;Acc:L8GYD7]	0.00450127	0.422985333	0.418484064	1.18E-08
6h	ACA1_106330/clean/scf7180000084660:85538-85649:-	ACA1_106330	Methyltransf_11 domain-containing protein [Source:UniProtKB/TrEMBL;Acc:L8GMT5]	0.009139063	0.427447667	0.418308603	3.17E-14
6h	ACA1_060350/clean/scf7180000084599:650935-651104:+	ACA1_060350	Rho-GAP domain-containing protein [Source:UniProtKB/TrEMBL;Acc:L8GXL3]	0.001476683	0.419640667	0.418163983	6.09E-09
6h	ACA1_068360/clean/scf7180000084603:181458-181558:+	ACA1_068360	Dual specificity protein kinase [Source:UniProtKB/TrEMBL;Acc:L8HDE4]	0.011033937	0.429102333	0.418068397	4.79E-23
6h	ACA1_074080/clean/scf7180000084605:18489-18575:-	ACA1_074080	hypothetical protein	0.016618403	0.434664667	0.418046263	5.99E-11
6h	ACA1_382640/clean/scf7180000084721:93099-93253:+	ACA1_382640	von Willebrand factor type A domain containing protein [Source:UniProtKB/TrEMBL;Acc:L8GUB9]	0.008887233	0.426911333	0.4180241	5.12E-07
6h	ACA1_201400/clean/scf7180000084753:543628-543713:+	ACA1_201400	CBS domain containing protein [Source:UniProtKB/TrEMBL;Acc:L8H3G1]	0.0211548	0.439063333	0.417908533	3.45E-16
6h	ACA1_339910/clean/scf7180000084619:32479-32585:-	ACA1_339910	hypothetical protein	0.004925253	0.422661	0.417735747	2.51E-08
6h	ACA1_232830/clean/scf7180000084762:84122-84216:-	ACA1_232830	Surfeit locus protein 5 [Source:UniProtKB/TrEMBL;Acc:L8H3C8]	0.000445287	0.418059667	0.41761438	1.21E-11
6h	ACA1_106000/clean/scf7180000084660:52967-53058:+	ACA1_106000	HEAT repeat domain containing protein [Source:UniProtKB/TrEMBL;Acc:L8GN71]	0.007984477	0.425597333	0.417612857	9.41E-09
6h	ACA1_257280/clean/scf7180000084769:142460-142547:-	ACA1_257280	Chloride transporter, chloride channel (ClC) subfamily protein [Source:UniProtKB/TrEMBL;Acc:L8GFQ3]	0	0.417582	0.417582	3.85E-08
6h	ACA1_331590/clean/scf7180000084578:7864-7965:+	ACA1_331590	ATPdependent DNA helicase, RecQ subfamily protein [Source:UniProtKB/TrEMBL;Acc:L8HBM3]	0.0125786	0.430142667	0.417564067	1.75E-06
6h	ACA1_060330/clean/scf7180000084599:648316-648463:-	ACA1_060330	Leucine rich repeat domain containing protein [Source:UniProtKB/TrEMBL;Acc:L8GYW2]	0.082792767	0.500339	0.417546233	0.000162115
6h	ACA1_156480/clean/scf7180000084723:44890-45000:+	ACA1_156480	PHB domain-containing protein [Source:UniProtKB/TrEMBL;Acc:L8GK25]	0.002838877	0.420342	0.417503123	1.09E-15
6h	ACA1_332500/clean/scf7180000084579:30639-30752:-	ACA1_332500	hypothetical protein	0.001230753	0.418419667	0.417188913	8.73E-10
6h	ACA1_107440/clean/scf7180000084660:198377-198487:+	ACA1_107440	Purple acid phosphatase [Source:UniProtKB/TrEMBL;Acc:L8GNJ7]	0.008560367	0.425659333	0.417098967	1.30E-25
6h	ACA1_369270/clean/scf7180000084664:286464-286533:+	ACA1_369270	Rasrelated protein Rab-18-like, putative [Source:UniProtKB/TrEMBL;Acc:L8GYY7]	0	0.417095	0.417095	5.62E-12
6h	ACA1_381750/clean/scf7180000084712:203995-204088:-	ACA1_381750	hypothetical protein	0.001926299	0.419005667	0.417079368	4.41E-10
6h	ACA1_072350/clean/scf7180000084603:1206844-1206921:+	ACA1_072350	USP domain-containing protein [Source:UniProtKB/TrEMBL;Acc:L8HFX4]	0.012345667	0.429210333	0.416864667	3.46E-05
6h	ACA1_156040/clean/scf7180000084723:9936-10061:+	ACA1_156040	Fungal Zn(2)-Cys(6) binuclear cluster domain containing protein [Source:UniProtKB/TrEMBL;Acc:L8GHR8]	0.008547	0.425241333	0.416694333	6.65E-06
6h	ACA1_193140/clean/scf7180000084749:195334-195457:+	ACA1_193140	Metallophosphoesterase [Source:UniProtKB/TrEMBL;Acc:L8GQV5]	0.012464867	0.429139	0.416674133	2.13E-07
6h	ACA1_037870/clean/scf7180000084568:48974-49056:+	ACA1_037870	Vacuolar protein sorting-associated protein 11 homolog [Source:UniProtKB/TrEMBL;Acc:L8GKP2]	0.00087489	0.417423667	0.416548777	1.85E-10
6h	ACA1_114300/clean/scf7180000084669:456606-456707:+	ACA1_114300	Poly(ADPribose) polymerase catalytic domain containing protein [Source:UniProtKB/TrEMBL;Acc:L8H6I0]	0.008771933	0.425160333	0.4163884	4.93E-06
6h	ACA1_369650/clean/scf7180000084664:349834-349906:+	ACA1_369650	S5A_REDUCTASE domain-containing protein [Source:UniProtKB/TrEMBL;Acc:L8GZ51]	0.039264887	0.455605667	0.41634078	2.17E-06
6h	ACA1_210530/clean/scf7180000084758:48860-48958:-	ACA1_210530	4Fe-4S ferredoxin-type domain-containing protein [Source:UniProtKB/TrEMBL;Acc:L8GS75]	0.0097635	0.426102333	0.416338833	3.62E-10
6h	ACA1_035520/clean/scf7180000084564:79128-79212:-	ACA1_035520	Fatty acid hydroxylase domain-containing protein [Source:UniProtKB/TrEMBL;Acc:L8HAF7]	0.016736547	0.43297	0.416233453	5.62E-25
6h	ACA1_096440/clean/scf7180000084645:1011822-1011893:+	ACA1_096440	RhoGAP domain containing protein [Source:UniProtKB/TrEMBL;Acc:L8GJR5]	0.004469973	0.420631333	0.416161361	8.15E-09
6h	ACA1_374010/clean/scf7180000084679:203888-203956:-	ACA1_374010	Peptidase M15D, vanX Dala-D-ala dipeptidase [Source:UniProtKB/TrEMBL;Acc:L8GHK1]	0	0.416108333	0.416108333	2.03E-06
6h	ACA1_024200/clean/scf7180000084441:14171-14278:-	ACA1_024200	WD_REPEATS_REGION domain-containing protein [Source:UniProtKB/TrEMBL;Acc:L8GKS0]	0.0237607	0.439777333	0.416016633	1.04E-06
6h	ACA1_181600/clean/scf7180000084745:25457-25524:+	ACA1_181600	ACB domain-containing protein [Source:UniProtKB/TrEMBL;Acc:L8HA17]	0.027701	0.44361	0.415909	1.56E-10
6h	ACA1_287960/clean/scf7180000084777:133903-133970:-	ACA1_287960	SET domain-containing protein [Source:UniProtKB/TrEMBL;Acc:L8HIS3]	0	0.415844	0.415844	4.48E-05
6h	ACA1_178530/clean/scf7180000084743:254623-254733:-	ACA1_178530	Dual specificity phosphatase, catalytic domain containing protein [Source:UniProtKB/TrEMBL;Acc:L8GSL7]	0.093338267	0.509128667	0.4157904	0.001881718
6h	ACA1_081440/clean/scf7180000084618:110143-110229:-	ACA1_081440	Alg14 family oligosaccharide biosynthesis protein [Source:UniProtKB/TrEMBL;Acc:L8HD51]	0.004696853	0.420411333	0.41571448	8.23E-10
6h	ACA1_232460/clean/scf7180000084762:31294-31396:+	ACA1_232460	Protein kinase domain-containing protein [Source:UniProtKB/TrEMBL;Acc:L8H0X8]	0.026399033	0.442064667	0.415665633	3.79E-08
6h	ACA1_182400/clean/scf7180000084745:204326-204539:+	ACA1_182400	Integral membrane protein [Source:UniProtKB/TrEMBL;Acc:L8H9C3]	0.197557	0.613167333	0.415610333	7.61E-05
6h	ACA1_015480/clean/scf7180000083799:2123-2230:+	ACA1_015480	hypothetical protein	0.046044	0.461582333	0.415538333	8.57E-11
6h	ACA1_034300/clean/scf7180000084563:6500-6560:+	ACA1_034300	DUF1084 domain-containing protein [Source:UniProtKB/TrEMBL;Acc:L8HCY8]	0	0.415481	0.415481	5.61E-08
6h	ACA1_170640/clean/scf7180000084739:98781-98870:+	ACA1_170640	Protein kinase domain-containing protein [Source:UniProtKB/TrEMBL;Acc:L8HIR2]	0.014445549	0.429914333	0.415468784	1.23E-05
6h	ACA1_071160/clean/scf7180000084603:876700-876840:+	ACA1_071160	Letm1 RBD domain-containing protein [Source:UniProtKB/TrEMBL;Acc:L8HE82]	0.011135887	0.426475333	0.415339447	4.40E-07
6h	ACA1_239550/clean/scf7180000084763:165718-165828:-	ACA1_239550	Cytidine and deoxycytidylate deaminase zincbinding region subfamily protein [Source:UniProtKB/TrEMBL;Acc:L8GKS6]	0.0075715	0.422836333	0.415264833	1.83E-14
6h	ACA1_069340/clean/scf7180000084603:413563-413630:-	ACA1_069340	hypothetical protein	0.050369367	0.465599667	0.4152303	9.76E-11
6h	ACA1_080580/clean/scf7180000084618:39260-39370:+	ACA1_080580	t-SNARE coiled-coil homology domain-containing protein [Source:UniProtKB/TrEMBL;Acc:L8HBP6]	0.0461161	0.461338333	0.415222233	7.93E-16
6h	ACA1_012330/clean/scf7180000083740:16931-17020:+	ACA1_012330	WD_REPEATS_REGION domain-containing protein [Source:UniProtKB/TrEMBL;Acc:L8HCD7]	0.00298866	0.418174	0.41518534	2.58E-08
6h	ACA1_045710/clean/scf7180000084576:181997-182084:-	ACA1_045710	PH domain-containing protein [Source:UniProtKB/TrEMBL;Acc:L8H189]	0	0.415064667	0.415064667	8.29E-11
6h	ACA1_342510/clean/scf7180000084633:24583-24659:-	ACA1_342510	Glucuronidase, beta, putative [Source:UniProtKB/TrEMBL;Acc:L8HD44]	0	0.415039333	0.415039333	0.00031607
6h	ACA1_288290/clean/scf7180000084777:160883-160972:+	ACA1_288290	SH3 domain-containing protein [Source:UniProtKB/TrEMBL;Acc:L8HKD1]	0.0211199	0.436065	0.4149451	7.28E-07
6h	ACA1_144380/clean/scf7180000084709:220085-220293:+	ACA1_144380	Transcription elongation factor spt6 [Source:UniProtKB/TrEMBL;Acc:L8HFM7]	0.00270486	0.417611333	0.414906473	3.28E-10
6h	ACA1_192020/clean/scf7180000084749:74755-74851:+	ACA1_192020	Cystathionine gammasynthase [Source:UniProtKB/TrEMBL;Acc:L8GNB3]	0.001337027	0.416224333	0.414887307	5.22E-20
6h	ACA1_037900/clean/scf7180000084568:53753-53841:-	ACA1_037900	Nudix hydrolase domain-containing protein [Source:UniProtKB/TrEMBL;Acc:L8GKU1]	0.019932913	0.434733	0.414800087	1.32E-05
6h	ACA1_200000/clean/scf7180000084753:365781-365870:-	ACA1_200000	LAO/AO transport system ATPase [Source:UniProtKB/TrEMBL;Acc:L8H2I2]	0.008119593	0.422753	0.414633407	5.09E-08
6h	ACA1_199640/clean/scf7180000084753:286627-286768:-	ACA1_199640	RFX-type winged-helix domain-containing protein [Source:UniProtKB/TrEMBL;Acc:L8H5G1]	0.0342639	0.448856333	0.414592433	1.08E-10
6h	ACA1_377540/clean/scf7180000084711:87785-87883:-	ACA1_377540	PNPLA domain-containing protein [Source:UniProtKB/TrEMBL;Acc:L8GRQ7]	0.007446507	0.422018	0.414571493	4.82E-07
6h	ACA1_323310/clean/scf7180000084468:572-652:+	ACA1_323310	hypothetical protein	0.002414	0.416839667	0.414425667	2.82E-08
6h	ACA1_360210/clean/scf7180000084622:69456-69542:+	ACA1_360210	Fbox domain containing protein [Source:UniProtKB/TrEMBL;Acc:L8HF19]	0.022244093	0.436603333	0.41435924	2.28E-06
6h	ACA1_214940/clean/scf7180000084758:269745-269843:-	ACA1_214940	Leucine rich repeat domain containing protein [Source:UniProtKB/TrEMBL;Acc:L8GQ02]	0.0128266	0.427157667	0.414331067	3.48E-06
6h	ACA1_089960/clean/scf7180000084639:405072-405145:-	ACA1_089960	Dual specificity protein kinase [Source:UniProtKB/TrEMBL;Acc:L8GVS6]	0.003263453	0.417568333	0.414304881	4.58E-09
6h	ACA1_337970/clean/scf7180000084604:15039-15122:-	ACA1_337970	JmjC domain-containing protein [Source:UniProtKB/TrEMBL;Acc:L8GCM4]	0.0066237	0.420906667	0.414282967	2.16E-05
6h	ACA1_326190/clean/scf7180000084504:50397-50502:+	ACA1_326190	F-box domain-containing protein [Source:UniProtKB/TrEMBL;Acc:L8HH77]	0.007751933	0.422008	0.414256067	5.04E-05
6h	ACA1_037180/clean/scf7180000084567:78528-78617:-	ACA1_037180	hypothetical protein	0.013601113	0.427837667	0.414236553	1.33E-06
6h	ACA1_322230/clean/scf7180000084459:36794-36854:-	ACA1_322230	UvrD/REP helicase subfamily protein [Source:UniProtKB/TrEMBL;Acc:L8GM54]	0.395389	0.809602667	0.414213667	0.000647388
6h	ACA1_381510/clean/scf7180000084712:167822-167918:+	ACA1_381510	Pyr_redox_2 domain-containing protein [Source:UniProtKB/TrEMBL;Acc:L8GNN7]	0.0196076	0.433801667	0.414194067	2.78E-19
6h	ACA1_082180/clean/scf7180000084623:20111-20193:+	ACA1_082180	N-acetyltransferase domain-containing protein [Source:UniProtKB/TrEMBL;Acc:L8GMF4]	0.000685563	0.414851333	0.41416577	6.30E-11
6h	ACA1_329650/clean/scf7180000084562:38124-38216:-	ACA1_329650	hypothetical protein	0.039933867	0.45402	0.414086133	6.79E-11
6h	ACA1_396130/clean/scf7180000084759:24761-24866:-	ACA1_396130	Prefoldin, alpha subunit [Source:UniProtKB/TrEMBL;Acc:L8HDI9]	0.019136433	0.433057	0.413920567	1.25E-20
6h	ACA1_394730/clean/scf7180000084756:130776-130857:-	ACA1_394730	Putative tyrosineprotein phosphatase oca1, putative [Source:UniProtKB/TrEMBL;Acc:L8H2T3]	0.00710118	0.420976667	0.413875487	1.36E-08
6h	ACA1_325600/clean/scf7180000084495:42202-42303:-	ACA1_325600	Protein kinase domain containing protein [Source:UniProtKB/TrEMBL;Acc:L8HB78]	0.002194592	0.416069667	0.413875074	4.58E-11
6h	ACA1_107160/clean/scf7180000084660:158503-158640:+	ACA1_107160	hypothetical protein	0	0.413852	0.413852	1.78E-11
6h	ACA1_097390/clean/scf7180000084645:1231725-1231792:+	ACA1_097390	Phosphatidylinositol glycan [Source:UniProtKB/TrEMBL;Acc:L8GJ19]	0.036040217	0.449881333	0.413841117	2.62E-05
6h	ACA1_268930/clean/scf7180000084770:944457-944617:+	ACA1_268930	PWI domain-containing protein [Source:UniProtKB/TrEMBL;Acc:L8H2N5]	0.081677867	0.495399333	0.413721467	9.80E-08
6h	ACA1_266120/clean/scf7180000084770:606223-606428:+	ACA1_266120	SAM domain-containing protein [Source:UniProtKB/TrEMBL;Acc:L8H4I6]	0.338165	0.751690667	0.413525667	7.41E-05
6h	ACA1_084820/clean/scf7180000084632:9172-9260:+	ACA1_084820	RhoGEF domain containing protein [Source:UniProtKB/TrEMBL;Acc:L8HMR3]	0.010214367	0.423679667	0.4134653	9.15E-07
6h	ACA1_297530/clean/scf7180000082850:1426-1494:-	ACA1_297530	hypothetical protein	0.005227285	0.418643333	0.413416048	1.59E-08
6h	ACA1_267340/clean/scf7180000084770:859813-859892:-	ACA1_267340	CBS domain containing protein [Source:UniProtKB/TrEMBL;Acc:L8H2L9]	0.031787967	0.4452	0.413412033	2.34E-11
6h	ACA1_228520/clean/scf7180000084761:180223-180303:-	ACA1_228520	ANK_REP_REGION domain-containing protein [Source:UniProtKB/TrEMBL;Acc:L8HB48]	0.00217865	0.415454333	0.413275683	6.77E-10
6h	ACA1_325170/clean/scf7180000084475:14416-14537:-	ACA1_325170	MHD domain-containing protein [Source:UniProtKB/TrEMBL;Acc:L8GPP2]	0.002535747	0.415772667	0.41323692	2.04E-12
6h	ACA1_261400/clean/scf7180000084769:660462-660526:-	ACA1_261400	Serine/threonine protein kinase, putative [Source:UniProtKB/TrEMBL;Acc:L8GF66]	0	0.413177333	0.413177333	0.008037297
6h	ACA1_175590/clean/scf7180000084741:1009263-1009406:+	ACA1_175590	hypothetical protein	0.003506067	0.416581	0.413074933	9.77E-08

6h	ACA1_116850/clean/scf7180000084669:861119-861213:+	ACA1_116850	JmjC domain-containing protein [Source:UniProtKB/TrEMBL;Acc:L8H4R9]	0.022393233	0.435464333	0.4130711	1.96E-07
6h	ACA1_184420/clean/scf7180000084745:638985-639079:+	ACA1_184420	Cathepsin Llike proteinase [Source:UniProtKB/TrEMBL;Acc:L8H8J9]	0.00159951	0.414664	0.41306449	5.72E-12
6h	ACA1_042070/clean/scf7180000084573:52710-52803:+	ACA1_042070	Dual specificity protein kinase [Source:UniProtKB/TrEMBL;Acc:L8GW91]	0.028589467	0.44155	0.412960533	3.31E-12
6h	ACA1_389490/clean/scf7180000084733:266985-267069:-	ACA1_389490	RRM domain-containing protein [Source:UniProtKB/TrEMBL;Acc:L8GES5]	0.009581099	0.422528667	0.412947567	1.50E-08
6h	ACA1_063780/clean/scf7180000084599:1500678-1500777:+	ACA1_063780	Nucleotidyltransferase domain containing protein [Source:UniProtKB/TrEMBL;Acc:L8GWN7]	0.0177714	0.430697333	0.412925933	3.53E-08
6h	ACA1_093350/clean/scf7180000084645:525501-525579:+	ACA1_093350	Fbox domain containing protein [Source:UniProtKB/TrEMBL;Acc:L8GIV6]	0.006618997	0.419516333	0.412897337	8.78E-07
6h	ACA1_096400/clean/scf7180000084645:1004286-1004403:+	ACA1_096400	Methyltransf_11 domain-containing protein [Source:UniProtKB/TrEMBL;Acc:L8GJF1]	0.001254623	0.414142333	0.41288771	1.97E-10
6h	ACA1_096950/clean/scf7180000084645:1123502-1123575:+	ACA1_096950	RNA recognition motif domain containing protein [Source:UniProtKB/TrEMBL;Acc:L8GJ25]	0.005037317	0.417912333	0.412875017	1.21E-11
6h	ACA1_283050/clean/scf7180000084776:773943-774023:+	ACA1_283050	Eukaryotic translation initiation factor 4e1a-binding protein [Source:UniProtKB/TrEMBL;Acc:L8H8S1]	0.001686383	0.414466333	0.41277995	2.31E-28
6h	ACA1_110100/clean/scf7180000084663:63149-63339:-	ACA1_110100	Protein kinase [Source:UniProtKB/TrEMBL;Acc:L8HK05]	0.02998132	0.442756667	0.412775347	9.43E-07
6h	ACA1_115400/clean/scf7180000084669:624959-625077:+	ACA1_115400	Protoporphyrinogen oxidase [Source:UniProtKB/TrEMBL;Acc:L8H4N9]	0.073592267	0.486237	0.412644733	1.81E-07
6h	ACA1_061680/clean/scf7180000084599:1009569-1009660:+	ACA1_061680	PH domain containing protein [Source:UniProtKB/TrEMBL;Acc:L8GXZ1]	0.023171567	0.435796667	0.4126251	3.58E-06
6h	ACA1_051700/clean/scf7180000084592:53094-53190:+	ACA1_051700	FGGAP repeat domain containing protein [Source:UniProtKB/TrEMBL;Acc:L8GPT4]	0.002961183	0.415581	0.412619817	2.02E-08
6h	ACA1_100560/clean/scf7180000084648:113230-113316:+	ACA1_100560	hypothetical protein	0.001734033	0.414233	0.412498967	6.21E-10
6h	ACA1_288810/clean/scf7180000084777:292516-292614:-	ACA1_288810	Leucine rich repeat domain containing protein [Source:UniProtKB/TrEMBL;Acc:L8HIZ3]	0.0838386	0.496179333	0.412340733	0.000173101
6h	ACA1_176120/clean/scf7180000084741:1127027-1127092:+	ACA1_176120	Armadillo repeat containing 7 isoform 1 family protein [Source:UniProtKB/TrEMBL;Acc:L8HH78]	0.011494267	0.423829	0.412334733	3.20E-07
6h	ACA1_011270/clean/scf7180000083623:6645-6751:-	ACA1_011270	FYVE-type domain-containing protein [Source:UniProtKB/TrEMBL;Acc:L8GQN9]	0.04434558	0.456633667	0.412288087	0.000496625
6h	ACA1_322400/clean/scf7180000084463:19292-19385:-	ACA1_322400	Zinc finger, C3HC4 type (RING finger) domain containing protein [Source:UniProtKB/TrEMBL;Acc:L8HII3]	0.002535647	0.414775667	0.41224002	1.22E-09
6h	ACA1_109730/clean/scf7180000084663:13183-13260:-	ACA1_109730	Replication factor C subunit 1 [Source:UniProtKB/TrEMBL;Acc:L8HIF4]	0.006250633	0.418477667	0.412227033	9.10E-10
6h	ACA1_068440/clean/scf7180000084603:195541-195605:+	ACA1_068440	F-box domain-containing protein [Source:UniProtKB/TrEMBL;Acc:L8HDJ5]	0.099890667	0.512088	0.412197333	0.003209558
6h	ACA1_329450/clean/scf7180000084562:13493-13567:-	ACA1_329450	hypothetical protein	0	0.412127333	0.412127333	4.41E-09
6h	ACA1_091930/clean/scf7180000084645:231444-231532:-	ACA1_091930	hypothetical protein	0.050562067	0.462493333	0.411931267	7.19E-09
6h	ACA1_057520/clean/scf7180000084599:158538-158606:+	ACA1_057520	NTP_transf_2 domain-containing protein [Source:UniProtKB/TrEMBL;Acc:L8GYG1]	0.002849003	0.414642667	0.411793663	3.61E-06
6h	ACA1_269380/clean/scf7180000084770:991502-991635:+	ACA1_269380	hypothetical protein	0.056724033	0.468506333	0.4117823	3.83E-08
6h	ACA1_015210/clean/scf7180000083783:10085-10194:-	ACA1_015210	MYND-type domain-containing protein [Source:UniProtKB/TrEMBL;Acc:L8GGA6]	0.0260682	0.437810333	0.411742133	6.34E-07
6h	ACA1_364680/clean/scf7180000084641:176138-176258:+	ACA1_364680	BTB domain-containing protein [Source:UniProtKB/TrEMBL;Acc:L8GLI9]	0.041228787	0.452929	0.411700213	5.08E-07
6h	ACA1_091440/clean/scf7180000084645:124093-124166:+	ACA1_091440	hypothetical protein	0.035087667	0.446675	0.411587333	0.047753614
6h	ACA1_171860/clean/scf7180000084741:179773-179861:+	ACA1_171860	POT family protein [Source:UniProtKB/TrEMBL;Acc:L8HHD5]	0	0.411569	0.411569	3.56E-08
6h	ACA1_258670/clean/scf7180000084769:300251-300336:-	ACA1_258670	DUF1084 domain-containing protein [Source:UniProtKB/TrEMBL;Acc:L8GFJ7]	0.016310493	0.427843	0.411532507	6.73E-10
6h	ACA1_201360/clean/scf7180000084753:531245-531351:-	ACA1_201360	Regulator of chromosome condensation (RCC1) repeat domain containing protein [Source:UniProtKB/TrEMBL;Acc:L8H4W1]	0.004901967	0.41643	0.411528033	2.47E-07
6h	ACA1_264180/clean/scf7180000084770:206427-206505:+	ACA1_264180	Oxidoreductase, short chain dehydrogenase/reductase superfamily protein [Source:UniProtKB/TrEMBL;Acc:L8H208]	0.001959307	0.41346	0.411500693	1.49E-08
6h	ACA1_044720/clean/scf7180000084576:36793-36894:-	ACA1_044720	F-box domain-containing protein [Source:UniProtKB/TrEMBL;Acc:L8GZR9]	0.01464465	0.425966667	0.411322017	5.04E-07
6h	ACA1_116460/clean/scf7180000084669:759356-759458:-	ACA1_116460	Queuosine salvage protein [Source:UniProtKB/TrEMBL;Acc:L8H4T6]	0.014051863	0.425162667	0.411110803	2.09E-06
6h	ACA1_022040/clean/scf7180000084264:1839-1928:+	ACA1_022040	hypothetical protein	0	0.411094667	0.411094667	0.00419345
6h	ACA1_019350/clean/scf7180000083937:29143-29229:-	ACA1_019350	FGGAP repeat domain containing protein [Source:UniProtKB/TrEMBL;Acc:L8HFR8]	0.003121122	0.414182333	0.411061212	3.24E-08
6h	ACA1_169100/clean/scf7180000084739:22012-22080:+	ACA1_169100	Dual specificity phosphatase, catalytic domain containing protein [Source:UniProtKB/TrEMBL;Acc:L8HG79]	0.013881107	0.424903333	0.411022227	2.21E-14
6h	ACA1_164900/clean/scf7180000084729:304911-305007:-	ACA1_164900	Bromo domain-containing protein [Source:UniProtKB/TrEMBL;Acc:L8GSQ5]	0.023416423	0.434303	0.410886577	6.61E-11
6h	ACA1_017770/clean/scf7180000083876:12600-12668:+	ACA1_017770	ABC transporter domain-containing protein [Source:UniProtKB/TrEMBL;Acc:L8GNP0]	0	0.410830667	0.410830667	4.16E-07
6h	ACA1_139840/clean/scf7180000084707:9627-9741:+	ACA1_139840	Receptor mediated endocytosis family member (Rme1), putative [Source:UniProtKB/TrEMBL;Acc:L8GGP3]	0.004997473	0.415815333	0.41081786	7.49E-17
6h	ACA1_400560/clean/scf7180000084775:195585-195664:+	ACA1_400560	hypothetical protein	0.042788067	0.453591	0.410802933	4.66E-08
6h	ACA1_369200/clean/scf7180000084664:271537-271620:-	ACA1_369200	Mannosyltransferase [Source:UniProtKB/TrEMBL;Acc:L8H0A1]	0.015873	0.426586333	0.410713333	0.000299223
6h	ACA1_298630/clean/scf7180000082992:5235-5346:-	ACA1_298630	Vacuolar protein sorting 16, putative [Source:UniProtKB/TrEMBL;Acc:L8H081]	0.004309867	0.415019667	0.4107098	6.16E-10
6h	ACA1_061500/clean/scf7180000084599:943535-943652:+	ACA1_061500	Ras-GAP domain-containing protein [Source:UniProtKB/TrEMBL;Acc:L8GWM5]	0.002990246	0.413599333	0.410609088	9.26E-10
6h	ACA1_261400/clean/scf7180000084769:662931-663023:-	ACA1_261400	Serine/threonine protein kinase, putative [Source:UniProtKB/TrEMBL;Acc:L8GF66]	0	0.410455	0.410455	0.041947335
6h	ACA1_252570/clean/scf7180000084768:183489-183558:+	ACA1_252570	CBS domain containing protein [Source:UniProtKB/TrEMBL;Acc:L8HAN6]	0.106915633	0.517353667	0.410438033	8.00E-06
6h	ACA1_119170/clean/scf7180000084672:31357-31436:+	ACA1_119170	hypothetical protein	0.056656067	0.467042667	0.4103866	5.74E-07
6h	ACA1_252590/clean/scf7180000084768:186735-186821:-	ACA1_252590	Fe2OG dioxygenase domain-containing protein [Source:UniProtKB/TrEMBL;Acc:L8H9W1]	0.046480933	0.456718	0.410237067	1.22E-05
6h	ACA1_156700/clean/scf7180000084723:55202-55267:+	ACA1_156700	Proteintyrosine phosphatase [Source:UniProtKB/TrEMBL;Acc:L8GIF7]	0.011714517	0.421907	0.410192483	4.43E-10
6h	ACA1_041920/clean/scf7180000084573:20123-20225:+	ACA1_041920	Deoxyribonuclease II, putative [Source:UniProtKB/TrEMBL;Acc:L8GV23]	0.001890016	0.411987	0.410096984	3.45E-11
6h	ACA1_280530/clean/scf7180000084776:490147-490220:-	ACA1_280530	hypothetical protein	0.050685167	0.460717667	0.4100325	5.06E-08
6h	ACA1_296970/clean/scf7180000084777:1451620-1451693:-	ACA1_296970	Kelch domain containing 1, putative [Source:UniProtKB/TrEMBL;Acc:L8HK47]	0	0.409979	0.409979	1.12E-05
6h	ACA1_388770/clean/scf7180000084733:165843-165909:+	ACA1_388770	Poly(A) polymerase central domain containing protein [Source:UniProtKB/TrEMBL;Acc:L8GG84]	0.247942667	0.657847	0.409904333	3.32E-08
6h	ACA1_074740/clean/scf7180000084608:74663-74757:-	ACA1_074740	Thioredoxin domain-containing protein [Source:UniProtKB/TrEMBL;Acc:L8HF80]	0.032879233	0.442693333	0.4098141	7.19E-10
6h	ACA1_175110/clean/scf7180000084741:866353-866453:-	ACA1_175110	hypothetical protein	0.00026434	0.410062	0.40979766	2.25E-06
6h	ACA1_144080/clean/scf7180000084709:147575-147640:+	ACA1_144080	hypothetical protein	0.003508767	0.413244667	0.4097359	1.58E-08
6h	ACA1_040690/clean/scf7180000084572:48081-48160:+	ACA1_040690	Multisensor hybrid histidine kinase [Source:UniProtKB/TrEMBL;Acc:L8H1W6]	0.1414696	0.551205	0.4097354	2.70E-05
6h	ACA1_096400/clean/scf7180000084645:1004534-1004608:+	ACA1_096400	Methyltransf_11 domain-containing protein [Source:UniProtKB/TrEMBL;Acc:L8GJF1]	0.003236247	0.412954333	0.409718087	5.41E-09
6h	ACA1_093910/clean/scf7180000084645:676989-677084:+	ACA1_093910	WD_REPEATS_REGION domain-containing protein [Source:UniProtKB/TrEMBL;Acc:L8GJ15]	0.005345717	0.415031333	0.409685617	7.54E-08
6h	ACA1_240070/clean/scf7180000084763:196311-196389:-	ACA1_240070	Carboxylic ester hydrolase [Source:UniProtKB/TrEMBL;Acc:L8GJU3]	0.001666667	0.411306333	0.409639667	3.63E-11
6h	ACA1_038180/clean/scf7180000084568:90337-90406:+	ACA1_038180	FHA domain containing protein [Source:UniProtKB/TrEMBL;Acc:L8GLQ3]	0.00588122	0.415519667	0.409638447	2.75E-08
6h	ACA1_183960/clean/scf7180000084745:528936-529022:-	ACA1_183960	Phosphoesterase family protein [Source:UniProtKB/TrEMBL;Acc:L8H8F6]	0.00316361	0.412801667	0.409638057	1.71E-08
6h	ACA1_295350/clean/scf7180000084777:1112407-1112487:-	ACA1_295350	RNA_bind domain-containing protein [Source:UniProtKB/TrEMBL;Acc:L8HJR1]	0.021861067	0.431467	0.409605933	6.50E-09
6h	ACA1_368290/clean/scf7180000084664:113185-113255:+	ACA1_368290	RWP-RK domain-containing protein [Source:UniProtKB/TrEMBL;Acc:L8H051]	0	0.409436	0.409436	0.000135917
6h	ACA1_006130/clean/scf7180000083029:49066-49161:+	ACA1_006130	Tyrosine kinase, putative [Source:UniProtKB/TrEMBL;Acc:L8HG15]	0.004814337	0.414145333	0.409330997	1.89E-07
6h	ACA1_271720/clean/scf7180000084771:89622-89735:-	ACA1_271720	Ras-GAP domain-containing protein [Source:UniProtKB/TrEMBL;Acc:L8GR76]	0.031757033	0.440941667	0.409184633	1.18E-06
6h	ACA1_187780/clean/scf7180000084747:93736-93812:-	ACA1_187780	Corrinoid adenosyltransferase [Source:UniProtKB/TrEMBL;Acc:L8GUU3]	0.011574867	0.420756333	0.409181467	2.09E-09
6h	ACA1_114500/clean/scf7180000084669:487494-487582:-	ACA1_114500	Macro domain-containing protein [Source:UniProtKB/TrEMBL;Acc:L8H6J5]	0.003557682	0.412695333	0.409137652	2.00E-10
6h	ACA1_145170/clean/scf7180000084710:52939-53062:+	ACA1_145170	Carrier superfamily protein [Source:UniProtKB/TrEMBL;Acc:L8GFD9]	0.014374533	0.423154667	0.408780133	2.29E-10
6h	ACA1_291500/clean/scf7180000084777:759663-759783:-	ACA1_291500	Dual specificity protein kinase shkC, putative [Source:UniProtKB/TrEMBL;Acc:L8HJF2]	0.005395367	0.414132667	0.4087373	1.47E-07
6h	ACA1_189830/clean/scf7180000084748:97905-97987:+	ACA1_189830	Partner of sld five, psf2 protein [Source:UniProtKB/TrEMBL;Acc:L8GE47]	0.000971817	0.409674	0.408702183	2.35E-06
6h	ACA1_385410/clean/scf7180000084730:165472-165643:+	ACA1_385410	TRANSKETOLASE_1 domain-containing protein [Source:UniProtKB/TrEMBL;Acc:L8H9A8]	0.009057713	0.417749333	0.40869162	7.20E-23
6h	ACA1_062300/clean/scf7180000084599:1159040-1159109:+	ACA1_062300	Transmembrane protein 93, putative [Source:UniProtKB/TrEMBL;Acc:L8GY61]	0	0.408413	0.408413	2.91E-09
6h	ACA1_113490/clean/scf7180000084669:293914-294054:-	ACA1_113490	SAM domain-containing protein [Source:UniProtKB/TrEMBL;Acc:L8H3A5]	0.055639633	0.463952	0.408312367	5.43E-09
6h	ACA1_369060/clean/scf7180000084664:230677-230745:+	ACA1_369060	Ubiquitin-related modifier 1 homolog [Source:UniProtKB/TrEMBL;Acc:L8GY50]	0.031060383	0.439307	0.408246617	1.37E-07
6h	ACA1_007760/clean/scf7180000083166:23510-23602:+	ACA1_007760	PH domain containing protein [Source:UniProtKB/TrEMBL;Acc:L8HAY1]	0.0070922	0.415271667	0.408179467	1.29E-06
6h	ACA1_184450/clean/scf7180000084745:648814-648883:+	ACA1_184450	3Beta_HSD domain-containing protein [Source:UniProtKB/TrEMBL;Acc:L8H9V0]	0.003139351	0.411213667	0.408074315	1.32E-17
6h	ACA1_063460/clean/scf7180000084599:1414064-1414133:-	ACA1_063460	AAA domain-containing protein [Source:UniProtKB/TrEMBL;Acc:L8GXD1]	0.026656167	0.434714333	0.408058167	9.60E-13
6h	ACA1_072260/clean/scf7180000084603:1181931-1182001:-	ACA1_072260	PNPLA domain-containing protein [Source:UniProtKB/TrEMBL;Acc:L8HDN5]	0.076968733	0.484779667	0.407810933	4.56E-06
6h	ACA1_068720/clean/scf7180000084603:263264-263334:+	ACA1_068720	WD_REPEATS_REGION domain-containing protein [Source:UniProtKB/TrEMBL;Acc:L8HEU1]	0	0.407722333	0.407722333	5.37E-06
6h	ACA1_269320/clean/scf7180000084770:971302-971371:-	ACA1_269320	eIF2 gamma, putative [Source:UniProtKB/TrEMBL;Acc:L8H4Y2]	0.030620967	0.438322667	0.4077017	4.90E-20
6h	ACA1_216300/clean/scf7180000084758:463728-463951:+	ACA1_216300	Ras family protein [Source:UniProtKB/TrEMBL;Acc:L8GR60]	0.015050833	0.422671	0.407620167	2.71E-06
6h	ACA1_223310/clean/scf7180000084760:126296-126381:-	ACA1_223310	Amidohydro_3 domain-containing protein [Source:UniProtKB/TrEMBL;Acc:L8GTU8]	0.037159767	0.444766	0.407606233	4.69E-07
6h	ACA1_045250/clean/scf7180000084576:94242-94324:+	ACA1_045250	hypothetical protein	0.00561474	0.413199667	0.407584927	1.30E-07
6h	ACA1_048180/clean/scf7180000084582:46006-46122:-	ACA1_048180	Dual specificity protein kinase [Source:UniProtKB/TrEMBL;Acc:L8GDV2]	0.013781233	0.421175	0.407393767	0.003525728
6h	ACA1_074850/clean/scf7180000084608:103799-103861:-	ACA1_074850	S5 DRBM domain-containing protein [Source:UniProtKB/TrEMBL;Acc:L8HGT0]	0.009009	0.416183	0.407174	3.70E-06
6h	ACA1_116020/clean/scf7180000084669:706526-706635:+	ACA1_116020	Rho-GAP domain-containing protein [Source:UniProtKB/TrEMBL;Acc:L8H6T0]	0.002406437	0.409268	0.406861563	4.06E-10
6h	ACA1_155470/clean/scf7180000084722:165157-165239:-	ACA1_155470	unspecified product	0	0.406688667	0.406688667	3.73E-06
6h	ACA1_044480/clean/scf7180000084576:26833-26935:-	ACA1_044480	Allergen V5/Tpx1 family protein [Source:UniProtKB/TrEMBL;Acc:L8H123]	0	0.406615333	0.406615333	1.86E-11
6h	ACA1_266100/clean/scf7180000084770:594563-594658:+	ACA1_266100	Protein kinase domain-containing protein [Source:UniProtKB/TrEMBL;Acc:L8H1J1]	0.00063903	0.407235333	0.406596303	3.37E-08
6h	ACA1_393050/clean/scf7180000084756:33186-33287:-	ACA1_393050	RhoGEF domain containing protein [Source:UniProtKB/TrEMBL;Acc:L8GZK0]	0.0379607	0.444529667	0.406568967	3.26E-08
6h	ACA1_189810/clean/scf7180000084748:90804-90905:-	ACA1_189810	GYF domain-containing protein [Source:UniProtKB/TrEMBL;Acc:L8GDX6]	0.018673837	0.425196	0.406522163	5.31E-07
6h	ACA1_360160/clean/scf7180000084622:62363-62438:-	ACA1_360160	Pribosyltran domain-containing protein [Source:UniProtKB/TrEMBL;Acc:L8HF15]	0.003367248	0.409886333	0.406519086	1.38E-10
6h	ACA1_042270/clean/scf7180000084573:74276-74337:-	ACA1_042270	RING-type domain-containing protein [Source:UniProtKB/TrEMBL;Acc:L8GVA2]	0.156689333	0.563136333	0.406447	1.09E-05
6h	ACA1_133450/clean/scf7180000084701:60189-60275:+	ACA1_133450	HECT domain-containing protein [Source:UniProtKB/TrEMBL;Acc:L8H3L8]	0.005046437	0.411435333	0.406388897	1.01E-07
6h	ACA1_278990/clean/scf7180000084776:290850-290980:+	ACA1_278990	hypothetical protein	0.0258353	0.432167333	0.406332033	2.36E-06
6h	ACA1_033500/clean/scf7180000084559:27763-27862:+	ACA1_033500	SAM domain-containing protein [Source:UniProtKB/TrEMBL;Acc:L8GGV5]	0.01043943	0.416701	0.40626157	1.24E-06
6h	ACA1_060020/clean/scf7180000084599:562214-562326:-	ACA1_060020	WD_REPEATS_REGION domain-containing protein [Source:UniProtKB/TrEMBL;Acc:L8GW95]	0.035122867	0.441379333	0.406256467	1.40E-05
6h	ACA1_274880/clean/scf7180000084773:132806-132883:+	ACA1_274880	AB hydrolase-1 domain-containing protein [Source:UniProtKB/TrEMBL;Acc:L8GI88]	0.03539081	0.441633	0.40624219	2.20E-10
6h	ACA1_395410/clean/scf7180000084756:172095-172176:-	ACA1_395410	hypothetical protein	0	0.406103333	0.406103333	2.36E-07
6h	ACA1_239240/clean/scf7180000084763:147302-147388:+	ACA1_239240	DH domain-containing protein [Source:UniProtKB/TrEMBL;Acc:L8GKG2]	0.008427107	0.414492333	0.406065227	4.85E-07
6h	ACA1_295150/clean/scf7180000084777:1084542-1084703:+	ACA1_295150	Phosphatidylserine decarboxylase [Source:UniProtKB/TrEMBL;Acc:L8HJP9]	0.016586343	0.422651	0.406064657	1.61E-11
6h	ACA1_051840/clean/scf7180000084592:60925-61005:-	ACA1_051840	RNA recognition motif domain containing protein [Source:UniProtKB/TrEMBL;Acc:L8GS29]	0.004329	0.410258	0.405929	2.01E-07
6h	ACA1_279740/clean/scf7180000084776:433643-433735:+	ACA1_279740	BATS domain-containing protein [Source:UniProtKB/TrEMBL;Acc:L8H6X4]	0.001978637	0.407779333	0.405800697	2.80E-09
6h	ACA1_068360/clean/scf7180000084603:179230-179333:+	ACA1_068360	Dual specificity protein kinase [Source:UniProtKB/TrEMBL;Acc:L8HDE4]	0.00104507	0.406843667	0.405798597	2.08E-14
6h	ACA1_276780/clean/scf7180000084774:213018-213110:-	ACA1_276780	unspecified product	0.0070922	0.412639	0.4055468	7.84E-08
6h	ACA1_092030/clean/scf7180000084645:251922-252002:-	ACA1_092030	Nudix hydrolase domain-containing protein [Source:UniProtKB/TrEMBL;Acc:L8GIW6]	0	0.405475333	0.405475333	1.33E-08
6h	ACA1_325290/clean/scf7180000084475:50584-50667:-	ACA1_325290	hypothetical protein	0.03063767	0.436062333	0.405424663	2.66E-07
6h	ACA1_397190/clean/scf7180000084759:168448-168531:+	ACA1_397190	Metallophos domain-containing protein [Source:UniProtKB/TrEMBL;Acc:L8HDM9]	0	0.40536	0.40536	2.96E-14
6h	ACA1_015560/clean/scf7180000083799:20968-21049:+	ACA1_015560	hypothetical protein	0.01055775	0.415917667	0.405359917	7.62E-14
6h	ACA1_395210/clean/scf7180000084756:156582-156652:-	ACA1_395210	Nonspecific lipidtransfer protein [Source:UniProtKB/TrEMBL;Acc:L8GZN6]	0	0.405295	0.405295	2.24E-08
6h	ACA1_019190/clean/scf7180000083937:12672-12762:+	ACA1_019190	Abhydrolase_3 domain-containing protein [Source:UniProtKB/TrEMBL;Acc:L8HI58]	0.012197713	0.417379333	0.40518162	1.25E-13
6h	ACA1_144000/clean/scf7180000084709:125968-126052:+	ACA1_144000	RhoGEF domain containing protein [Source:UniProtKB/TrEMBL;Acc:L8HFT1]	0.016877633	0.421828333	0.4049507	1.65E-08
6h	ACA1_178200/clean/scf7180000084743:179106-179226:+	ACA1_178200	B30.2/SPRY domain-containing protein [Source:UniProtKB/TrEMBL;Acc:L8GVS5]	0.006531539	0.411473667	0.404942128	4.12E-13
6h	ACA1_234610/clean/scf7180000084762:304894-304968:-	ACA1_234610	Peptidyl-prolyl cis-trans isomerase [Source:UniProtKB/TrEMBL;Acc:L8H2P2]	0.004253853	0.409161667	0.404907813	2.74E-29
6h	ACA1_164680/clean/scf7180000084729:248155-248375:-	ACA1_164680	ANK_REP_REGION domain-containing protein [Source:UniProtKB/TrEMBL;Acc:L8GSN8]	0.00439098	0.409295667	0.404904687	5.86E-10
6h	ACA1_145850/clean/scf7180000084710:167925-168007:-	ACA1_145850	O-methyltransferase, putative [Source:UniProtKB/TrEMBL;Acc:L8GFG9]	0.001676696	0.406571333	0.404894637	2.35E-09
6h	ACA1_142140/clean/scf7180000084708:92888-92945:+	ACA1_142140	unspecified product	0.024553967	0.429285	0.404731033	2.11E-06
6h	ACA1_218390/clean/scf7180000084758:649638-649732:-	ACA1_218390	Glycosyl hydrolase family 20, catalytic domain containing protein [Source:UniProtKB/TrEMBL;Acc:L8GQD2]	0.028114877	0.432756667	0.40464179	3.72E-07
6h	ACA1_098160/clean/scf7180000084645:1411447-1411532:-	ACA1_098160	ABC transporter, ATPbinding domain containing protein [Source:UniProtKB/TrEMBL;Acc:L8GJY4]	0.000931573	0.405547333	0.40461576	2.40E-11
6h	ACA1_158480/clean/scf7180000084724:234197-234308:+	ACA1_158480	Glutamine amidotransferase type-1 domain-containing protein [Source:UniProtKB/TrEMBL;Acc:L8HBH7]	0.000605083	0.405212333	0.40460725	5.49E-08
6h	ACA1_100610/clean/scf7180000084648:131645-131728:-	ACA1_100610	Serine/threonine kinase [Source:UniProtKB/TrEMBL;Acc:L8GK64]	0	0.404562333	0.404562333	4.26E-12
6h	ACA1_298650/clean/scf7180000082992:12188-12277:+	ACA1_298650	hypothetical protein	0.0193957	0.423948667	0.404552967	2.61E-09
6h	ACA1_035530/clean/scf7180000084564:82165-82296:-	ACA1_035530	Oxidoreductase domain containing protein [Source:UniProtKB/TrEMBL;Acc:L8HBX0]	0.19061	0.595155	0.404545	0.008873898
6h	ACA1_265380/clean/scf7180000084770:455492-455573:-	ACA1_265380	Phosphatidylinositol glycan anchor biosynthesis, class S, putative [Source:UniProtKB/TrEMBL;Acc:L8H3M4]	0.014551233	0.419038667	0.404487433	3.65E-08
6h	ACA1_360980/clean/scf7180000084622:214758-214843:+	ACA1_360980	hypothetical protein	0.037037	0.441511333	0.404474333	0.000853628
6h	ACA1_331930/clean/scf7180000084578:38562-38669:-	ACA1_331930	WWE domain-containing protein [Source:UniProtKB/TrEMBL;Acc:L8HE47]	0.000514667	0.404582333	0.404067667	2.91E-06
6h	ACA1_251790/clean/scf7180000084768:118705-118780:-	ACA1_251790	Cellulase (Glycosyl hydrolase family 5) subfamily protein [Source:UniProtKB/TrEMBL;Acc:L8HAR2]	0.01237897	0.416396	0.40401703	7.05E-11
6h	ACA1_371900/clean/scf7180000084664:609221-609299:-	ACA1_371900	EF hand domain containing protein [Source:UniProtKB/TrEMBL;Acc:L8GYH0]	0.0355156	0.439447667	0.403932067	4.62E-07
6h	ACA1_153550/clean/scf7180000084720:123143-123236:+	ACA1_153550	Aminotran_5 domain-containing protein [Source:UniProtKB/TrEMBL;Acc:L8HG75]	0.003994387	0.407801	0.403806613	3.06E-10
6h	ACA1_318540/clean/scf7180000084158:19585-19665:+	ACA1_318540	SPX domain containing protein [Source:UniProtKB/TrEMBL;Acc:L8GH73]	0.00087825	0.404632	0.40375375	3.01E-11
6h	ACA1_246510/clean/scf7180000084763:563928-564012:+	ACA1_246510	PPM-type phosphatase domain-containing protein [Source:UniProtKB/TrEMBL;Acc:L8GL94]	0.000216422	0.403923	0.403706578	1.01E-10
6h	ACA1_144220/clean/scf7180000084709:174375-174477:-	ACA1_144220	hypothetical protein	0.019263033	0.422838	0.403574967	5.43E-05
6h	ACA1_173970/clean/scf7180000084741:561495-561580:+	ACA1_173970	Aldedh domain-containing protein [Source:UniProtKB/TrEMBL;Acc:L8HGQ9]	0.00115147	0.404717333	0.403565864	1.01E-12
6h	ACA1_133450/clean/scf7180000084701:59613-59703:+	ACA1_133450	HECT domain-containing protein [Source:UniProtKB/TrEMBL;Acc:L8H3L8]	0.007876667	0.411361	0.403484333	2.95E-06
6h	ACA1_399880/clean/scf7180000084775:58072-58207:+	ACA1_399880	Rab-GAP TBC domain-containing protein [Source:UniProtKB/TrEMBL;Acc:L8GGF5]	0.019931967	0.423405333	0.403473367	6.10E-11
6h	ACA1_276410/clean/scf7180000084774:167946-168119:+	ACA1_276410	Phosphate transporter [Source:UniProtKB/TrEMBL;Acc:L8GTH7]	0.175470667	0.578938	0.403467333	5.59E-05
6h	ACA1_015340/clean/scf7180000083794:9024-9099:+	ACA1_015340	RNA_lig_T4_1 domain-containing protein [Source:UniProtKB/TrEMBL;Acc:L8GIY2]	0	0.403464333	0.403464333	1.11E-05
6h	ACA1_109770/clean/scf7180000084663:21117-21357:+	ACA1_109770	WD40 repeat-containing protein [Source:UniProtKB/TrEMBL;Acc:L8HKT6]	0.00466461	0.407931	0.40326639	4.52E-10

6h	ACA1_211500/clean/scf7180000084758:89330-89418:+	ACA1_211500	RAB5B protein, putative [Source:UniProtKB/TrEMBL;Acc:L8GS95]	0.015081267	0.418329333	0.403248067	1.42E-06
6h	ACA1_069510/clean/scf7180000084603:459844-459916:-	ACA1_069510	hypothetical protein	0	0.403100333	0.403100333	3.90E-08
6h	ACA1_251800/clean/scf7180000084768:122196-122338:+	ACA1_251800	WD domain, Gbeta repeat-containing protein [Source:UniProtKB/TrEMBL;Acc:L8HD10]	0.008501133	0.411564	0.403062867	1.73E-05
6h	ACA1_298660/clean/scf7180000082992:13238-13360:+	ACA1_298660	Bromo domain-containing protein [Source:UniProtKB/TrEMBL;Acc:L8H1K3]	0.013358667	0.416385667	0.403027	1.33E-08
6h	ACA1_063740/clean/scf7180000084599:1492988-1493075:-	ACA1_063740	Transmembrane protein [Source:UniProtKB/TrEMBL;Acc:L8GXB1]	0.008680037	0.411701	0.403020963	3.99E-08
6h	ACA1_253360/clean/scf7180000084768:285419-285589:+	ACA1_253360	TP_methylase domain-containing protein [Source:UniProtKB/TrEMBL;Acc:L8HAX0]	0.00118371	0.404195	0.40301129	3.87E-09
6h	ACA1_113490/clean/scf7180000084669:296279-296420:-	ACA1_113490	SAM domain-containing protein [Source:UniProtKB/TrEMBL;Acc:L8H3A5]	0.0109645	0.413887	0.4029225	2.60E-13
6h	ACA1_078010/clean/scf7180000084614:70609-70756:-	ACA1_078010	hypothetical protein	0.00203252	0.404886667	0.402854147	1.19E-10
6h	ACA1_377830/clean/scf7180000084711:127015-127081:+	ACA1_377830	UBIQUITIN_CONJUGAT_2 domain-containing protein [Source:UniProtKB/TrEMBL;Acc:L8GSV2]	0.025255867	0.428086	0.402830133	1.66E-13
6h	ACA1_058840/clean/scf7180000084599:393402-393492:-	ACA1_058840	HATPase_c domain-containing protein [Source:UniProtKB/TrEMBL;Acc:L8GXB7]	0.002644933	0.405387333	0.4027424	1.86E-16
6h	ACA1_267210/clean/scf7180000084770:831062-831152:-	ACA1_267210	FAA_hydrolase domain-containing protein [Source:UniProtKB/TrEMBL;Acc:L8H2J3]	0.009977263	0.412645333	0.40266807	5.41E-15
6h	ACA1_055590/clean/scf7180000084594:739242-739339:-	ACA1_055590	Dual specificity phosphatase, putative [Source:UniProtKB/TrEMBL;Acc:L8H7T5]	0.0220398	0.424663333	0.402623533	0.00015911
6h	ACA1_061870/clean/scf7180000084599:1069341-1069425:+	ACA1_061870	Gluconokinase [Source:UniProtKB/TrEMBL;Acc:L8GWR6]	0.001249717	0.403795333	0.402545617	1.15E-09
6h	ACA1_113490/clean/scf7180000084669:295569-295693:-	ACA1_113490	SAM domain-containing protein [Source:UniProtKB/TrEMBL;Acc:L8H3A5]	0.004636023	0.407097	0.402460977	3.30E-10
6h	ACA1_309500/clean/scf7180000083762:10938-11013:+	ACA1_309500	Pyridine nucleotidedisulfide oxidoreductase domain containing protein [Source:UniProtKB/TrEMBL;Acc:L8GI23]	0.0303153	0.432744667	0.402429367	2.50E-16
6h	ACA1_193130/clean/scf7180000084749:193543-193641:-	ACA1_193130	Metallophos domain-containing protein [Source:UniProtKB/TrEMBL;Acc:L8GN68]	0.175661	0.578087333	0.402426333	1.82E-07
6h	ACA1_206030/clean/scf7180000084755:66654-66730:+	ACA1_206030	Abnormal long morphology protein [Source:UniProtKB/TrEMBL;Acc:L8H4K0]	0	0.402316667	0.402316667	2.48E-08
6h	ACA1_078990/clean/scf7180000084615:123952-124044:-	ACA1_078990	AcylCoA synthetase, putative [Source:UniProtKB/TrEMBL;Acc:L8GRK9]	0.002995653	0.405302667	0.402307014	1.48E-11
6h	ACA1_224390/clean/scf7180000084760:189277-189404:-	ACA1_224390	hypothetical protein	0.069941433	0.472245	0.402303567	1.32E-09
6h	ACA1_272960/clean/scf7180000084773:5189-5354:-	ACA1_272960	Ras-GAP domain-containing protein [Source:UniProtKB/TrEMBL;Acc:L8GFH7]	0.013613967	0.415911333	0.402297367	5.60E-13
6h	ACA1_151100/clean/scf7180000084716:56601-56788:-	ACA1_151100	NFACT-R_1 domain-containing protein [Source:UniProtKB/TrEMBL;Acc:L8H219]	0.006048593	0.408339667	0.402291073	2.32E-08
6h	ACA1_116500/clean/scf7180000084669:766022-766134:-	ACA1_116500	NAD(+)/NADH kinase [Source:UniProtKB/TrEMBL;Acc:L8H3V3]	0.016056747	0.418293	0.402236253	2.53E-06
6h	ACA1_042040/clean/scf7180000084573:44273-44356:-	ACA1_042040	RAI1 domain-containing protein [Source:UniProtKB/TrEMBL;Acc:L8GV91]	0.018734867	0.420810667	0.4020758	1.00E-05
6h	ACA1_253850/clean/scf7180000084768:347791-347913:+	ACA1_253850	HTTM domain-containing protein [Source:UniProtKB/TrEMBL;Acc:L8HC95]	0.01488657	0.416916667	0.402030097	2.64E-11
6h	ACA1_259490/clean/scf7180000084769:351787-351847:+	ACA1_259490	TPR_REGION domain-containing protein [Source:UniProtKB/TrEMBL;Acc:L8GEZ4]	0.015678399	0.417664	0.401985601	1.57E-06
6h	ACA1_131880/clean/scf7180000084699:167255-167327:-	ACA1_131880	N-acetyltransferase domain-containing protein [Source:UniProtKB/TrEMBL;Acc:L8GMY7]	0.000963463	0.402698667	0.401735203	5.09E-10
6h	ACA1_288670/clean/scf7180000084777:257269-257364:-	ACA1_288670	Psf1 family protein [Source:UniProtKB/TrEMBL;Acc:L8HLA2]	0.081180567	0.482881667	0.4017011	0.000145444
6h	ACA1_119410/clean/scf7180000084672:63718-63916:+	ACA1_119410	PCI domain-containing protein [Source:UniProtKB/TrEMBL;Acc:L8HLX4]	0.012928307	0.414620667	0.40169236	1.23E-15
6h	ACA1_073000/clean/scf7180000084603:1352541-1352615:+	ACA1_073000	Mis12 domain containing protein [Source:UniProtKB/TrEMBL;Acc:L8HGW7]	0.017094033	0.418677667	0.401583633	6.74E-06
6h	ACA1_299380/clean/scf7180000083027:14025-14093:+	ACA1_299380	AAA_11 domain-containing protein [Source:UniProtKB/TrEMBL;Acc:L8GS61]	0.010159167	0.411742	0.401582833	1.18E-29
6h	ACA1_097360/clean/scf7180000084645:1226825-1226927:+	ACA1_097360	RINGfinger protein [Source:UniProtKB/TrEMBL;Acc:L8GJ66]	0.02856261	0.430055333	0.401492723	6.79E-05
6h	ACA1_131820/clean/scf7180000084699:136691-136799:+	ACA1_131820	CTCHY-type domain-containing protein [Source:UniProtKB/TrEMBL;Acc:L8GMY3]	0.034504167	0.435868	0.401363833	7.83E-07
6h	ACA1_173090/clean/scf7180000084741:400136-400221:+	ACA1_173090	Arginine-tRNA-protein transferase, putative [Source:UniProtKB/TrEMBL;Acc:L8HHK6]	0.009095853	0.410403	0.401307147	6.58E-09
6h	ACA1_237870/clean/scf7180000084763:36569-36706:+	ACA1_237870	Methyltransferase [Source:UniProtKB/TrEMBL;Acc:L8GKC5]	0.005085987	0.406388667	0.40130268	6.91E-33
6h	ACA1_393010/clean/scf7180000084756:26612-26709:+	ACA1_393010	AA_TRNA_LIGASE_II domain-containing protein [Source:UniProtKB/TrEMBL;Acc:L8H0A5]	0.004239623	0.405435333	0.40119571	1.75E-10
6h	ACA1_331800/clean/scf7180000084578:33219-33350:-	ACA1_331800	hypothetical protein	0	0.401148	0.401148	5.54E-07
6h	ACA1_174200/clean/scf7180000084741:640250-640354:-	ACA1_174200	Alpha-galactosidase [Source:UniProtKB/TrEMBL;Acc:L8HHU7]	0.005952367	0.407038667	0.4010863	4.67E-07
6h	ACA1_255510/clean/scf7180000084768:519043-519130:-	ACA1_255510	Ephrin_rec_like domain-containing protein [Source:UniProtKB/TrEMBL;Acc:L8HDG9]	0.005433629	0.406449667	0.401016037	8.17E-14
6h	ACA1_215990/clean/scf7180000084758:424786-424859:+	ACA1_215990	hypothetical protein	0	0.400961	0.400961	1.00E-10
6h	ACA1_017740/clean/scf7180000083876:1581-1655:+	ACA1_017740	5'-3' exoribonuclease 1 [Source:UniProtKB/TrEMBL;Acc:L8GMR4]	0	0.400837333	0.400837333	1.78E-11
6h	ACA1_054370/clean/scf7180000084594:474673-474777:-	ACA1_054370	Fe2OG dioxygenase domain-containing protein [Source:UniProtKB/TrEMBL;Acc:L8H8A8]	0.233863333	0.634693	0.400829667	1.00E-05
6h	ACA1_104240/clean/scf7180000084653:73974-74052:-	ACA1_104240	hypothetical protein	0.005591113	0.406278333	0.40068722	4.58E-08
6h	ACA1_379850/clean/scf7180000084712:15456-15570:-	ACA1_379850	Isochorismatase domain containing protein 2, mitochondrial, putative [Source:UniProtKB/TrEMBL;Acc:L8GN12]	0.00056225	0.401184667	0.400622417	1.90E-12
6h	ACA1_309650/clean/scf7180000083763:9144-9205:+	ACA1_309650	hypothetical protein	0.075307467	0.475914667	0.4006072	0.002503517
6h	ACA1_173830/clean/scf7180000084741:530355-530464:+	ACA1_173830	Methylisocitrate lyase, putative [Source:UniProtKB/TrEMBL;Acc:L8HHR5]	0.0193275	0.419912	0.4005845	2.25E-10
6h	ACA1_370300/clean/scf7180000084664:489711-489779:-	ACA1_370300	hypothetical protein	0.004549577	0.404964	0.400414423	1.68E-16
6h	ACA1_063910/clean/scf7180000084599:1535400-1535495:+	ACA1_063910	Tho complex subunit 7 protein [Source:UniProtKB/TrEMBL;Acc:L8GXH2]	0.004261114	0.40463	0.400368886	2.15E-08
6h	ACA1_269380/clean/scf7180000084770:991275-991377:+	ACA1_269380	hypothetical protein	0.006598923	0.406956	0.400357077	3.76E-07
6h	ACA1_061900/clean/scf7180000084599:1073918-1073994:-	ACA1_061900	GTP_EFTU_D3 domain-containing protein [Source:UniProtKB/TrEMBL;Acc:L8GY16]	0.009915447	0.410186	0.400270553	2.46E-11
6h	ACA1_068510/clean/scf7180000084603:219358-219482:-	ACA1_068510	Tyrosine phosphatase [Source:UniProtKB/TrEMBL;Acc:L8HDF9]	0.0471666	0.447378333	0.400211733	1.02E-11
6h	ACA1_134730/clean/scf7180000084702:40453-40555:+	ACA1_134730	G_PROTEIN_RECEP_F2_4 domain-containing protein [Source:UniProtKB/TrEMBL;Acc:L8GGN6]	0.00057542	0.400646	0.40007058	3.10E-10
6h	ACA1_282860/clean/scf7180000084776:737017-737087:-	ACA1_282860	3Beta_HSD domain-containing protein [Source:UniProtKB/TrEMBL;Acc:L8H7K3]	0.042928557	0.442971667	0.40004311	3.93E-05
6h	ACA1_233070/clean/scf7180000084762:118783-118908:+	ACA1_233070	hypothetical protein	0.000801867	0.400771333	0.399969467	8.18E-11
6h	ACA1_061900/clean/scf7180000084599:1073541-1073609:-	ACA1_061900	GTP_EFTU_D3 domain-containing protein [Source:UniProtKB/TrEMBL;Acc:L8GY16]	0.000671183	0.400329	0.399657817	9.89E-11
6h	ACA1_212820/clean/scf7180000084758:158781-158861:-	ACA1_212820	hypothetical protein	0.02002516	0.41964	0.39961484	6.07E-12
6h	ACA1_244870/clean/scf7180000084763:447172-447262:+	ACA1_244870	DNA polymerase [Source:UniProtKB/TrEMBL;Acc:L8GK72]	0.006838247	0.406371333	0.399533087	5.98E-07
6h	ACA1_175280/clean/scf7180000084741:907913-908010:+	ACA1_175280	PKS_ER domain-containing protein [Source:UniProtKB/TrEMBL;Acc:L8HH07]	0.0088356	0.408307667	0.399472067	1.57E-06
6h	ACA1_062040/clean/scf7180000084599:1108407-1108514:-	ACA1_062040	PPR repeat-containing protein [Source:UniProtKB/TrEMBL;Acc:L8GWY3]	0	0.399432667	0.399432667	3.02E-06
6h	ACA1_310630/clean/scf7180000083773:19669-19766:-	ACA1_310630	Allergen v5/tpx1 family protein [Source:UniProtKB/TrEMBL;Acc:L8GZF5]	0.002728247	0.402154333	0.399426087	5.11E-08
6h	ACA1_092460/clean/scf7180000084645:354027-354101:+	ACA1_092460	RFX-type winged-helix domain-containing protein [Source:UniProtKB/TrEMBL;Acc:L8GIP9]	0.042842233	0.442211333	0.3993691	1.64E-05
6h	ACA1_323430/clean/scf7180000084468:21543-21624:+	ACA1_323430	SnoaL family polyketide cyclase [Source:UniProtKB/TrEMBL;Acc:L8H0W6]	0.010310253	0.409577333	0.39926708	6.79E-24
6h	ACA1_338400/clean/scf7180000084606:3536-3645:+	ACA1_338400	Endo/exonuclease/phosphatase domain-containing protein [Source:UniProtKB/TrEMBL;Acc:L8HGL4]	0.00253729	0.401659667	0.399122377	2.16E-09
6h	ACA1_396720/clean/scf7180000084759:107324-107395:+	ACA1_396720	Mitochondrial protein 18 kDa, putative [Source:UniProtKB/TrEMBL;Acc:L8HEL5]	0.008255597	0.407298667	0.39904307	2.68E-10
6h	ACA1_258010/clean/scf7180000084769:200813-200886:-	ACA1_258010	BRCA1 C Terminus (BRCT) domain containing protein [Source:UniProtKB/TrEMBL;Acc:L8GHA9]	0.008090773	0.407114	0.399023227	8.90E-07
6h	ACA1_072550/clean/scf7180000084603:1222606-1222728:+	ACA1_072550	Fcf2 domain-containing protein [Source:UniProtKB/TrEMBL;Acc:L8HFY2]	0.021570613	0.420545333	0.39897472	6.11E-06
6h	ACA1_395480/clean/scf7180000084756:188708-188784:-	ACA1_395480	Copine subfamily protein [Source:UniProtKB/TrEMBL;Acc:L8H0H7]	0.01767485	0.416614	0.39893915	1.38E-08
6h	ACA1_200240/clean/scf7180000084753:420288-420429:-	ACA1_200240	Phospholipase D active site domain containing protein [Source:UniProtKB/TrEMBL;Acc:L8H3B9]	0.00516681	0.404079	0.39891219	6.73E-07
6h	ACA1_151250/clean/scf7180000084716:82275-82359:+	ACA1_151250	Glycine cleavage Tprotein (Aminomethyl transferase) domain containing protein [Source:UniProtKB/TrEMBL;Acc:L8H0R3]	0.008981267	0.407885	0.398903733	1.05E-07
6h	ACA1_036340/clean/scf7180000084565:82453-82527:+	ACA1_036340	Serine hydroxymethyltransferase [Source:UniProtKB/TrEMBL;Acc:L8HBL5]	0.000644167	0.399542333	0.398898167	1.33E-11
6h	ACA1_036660/clean/scf7180000084565:166868-166953:-	ACA1_036660	ATPase [Source:UniProtKB/TrEMBL;Acc:L8HCK4]	0.008130067	0.40692	0.398789933	5.83E-06
6h	ACA1_290280/clean/scf7180000084777:577191-577258:-	ACA1_290280	Exportin-T [Source:UniProtKB/TrEMBL;Acc:L8HI54]	0.0138889	0.412656333	0.398767433	4.31E-05
6h	ACA1_052320/clean/scf7180000084594:42980-43068:-	ACA1_052320	Phosphatidylcholinesterol acyltransferase (Lecithin-cholesterol acyltransferase), putative [Source:UniProtKB/TrEMBL;Acc:L8H748]	0.022108133	0.420804333	0.3986962	4.87E-05
6h	ACA1_320550/clean/scf7180000084416:10566-10667:-	ACA1_320550	Proline iminopeptidase [Source:UniProtKB/TrEMBL;Acc:L8GWD8]	0.00108	0.399738667	0.398658667	3.79E-18
6h	ACA1_269260/clean/scf7180000084770:960577-960675:-	ACA1_269260	Ras family protein [Source:UniProtKB/TrEMBL;Acc:L8H2Q9]	0.015073163	0.413631	0.398557837	6.62E-09
6h	ACA1_400210/clean/scf7180000084775:119182-119265:-	ACA1_400210	Non-specific serine/threonine protein kinase [Source:UniProtKB/TrEMBL;Acc:L8GIE6]	0.017716323	0.41625	0.398533677	1.69E-11
6h	ACA1_044480/clean/scf7180000084576:28308-28377:-	ACA1_044480	Allergen V5/Tpx1 family protein [Source:UniProtKB/TrEMBL;Acc:L8H123]	0	0.398515	0.398515	6.97E-09
6h	ACA1_336860/clean/scf7180000084596:6782-6880:-	ACA1_336860	Glutathione reductase [Source:UniProtKB/TrEMBL;Acc:L8GUD1]	0.003769433	0.402254667	0.398485233	2.81E-10
6h	ACA1_191520/clean/scf7180000084749:9344-9455:-	ACA1_191520	GDP-Man:Man(3)GlcNAc(2)-PP-Dol alpha-1,2-mannosyltransferase [Source:UniProtKB/TrEMBL;Acc:L8GNP5]	0.003893443	0.402372667	0.398479223	6.30E-07
6h	ACA1_031790/clean/scf7180000084523:3510-3590:+	ACA1_031790	Rap-GAP domain-containing protein [Source:UniProtKB/TrEMBL;Acc:L8H1P5]	0.007701223	0.406021667	0.398320443	1.00E-07
6h	ACA1_144830/clean/scf7180000084710:21731-21865:-	ACA1_144830	C2 domain containing protein [Source:UniProtKB/TrEMBL;Acc:L8GCS6]	0.00241546	0.40071	0.39829454	1.36E-08
6h	ACA1_037720/clean/scf7180000084568:7586-7687:+	ACA1_037720	F-box domain-containing protein [Source:UniProtKB/TrEMBL;Acc:L8GKN0]	0	0.398224	0.398224	1.32E-06
6h	ACA1_253380/clean/scf7180000084768:288685-288805:+	ACA1_253380	EF hand domain containing protein [Source:UniProtKB/TrEMBL;Acc:L8HAR9]	0.013209423	0.411346	0.398136577	3.45E-19
6h	ACA1_275470/clean/scf7180000084774:25392-25503:+	ACA1_275470	Calmodulin [Source:UniProtKB/TrEMBL;Acc:L8GRW7]	0.000284269	0.39839	0.398105731	7.22E-08
6h	ACA1_129350/clean/scf7180000084698:9764-9826:+	ACA1_129350	Fatty acid hydroxylase domain-containing protein [Source:UniProtKB/TrEMBL;Acc:L8GPA5]	0.00111111	0.399212333	0.398101223	8.52E-12
6h	ACA1_042070/clean/scf7180000084573:51434-51504:+	ACA1_042070	Dual specificity protein kinase [Source:UniProtKB/TrEMBL;Acc:L8GW91]	0.00506172	0.403085	0.39802328	1.25E-08
6h	ACA1_095960/clean/scf7180000084645:920020-920105:-	ACA1_095960	hypothetical protein	3.80E-05	0.398045667	0.398007684	7.73E-10
6h	ACA1_112160/clean/scf7180000084669:130450-130540:+	ACA1_112160	Dual specificity protein kinase [Source:UniProtKB/TrEMBL;Acc:L8H5D1]	0.001009823	0.398986333	0.39797651	6.68E-10
6h	ACA1_176030/clean/scf7180000084741:1110484-1110552:-	ACA1_176030	ATPase, AAA domain containing protein [Source:UniProtKB/TrEMBL;Acc:L8HI90]	0	0.397973333	0.397973333	4.85E-07
6h	ACA1_074900/clean/scf7180000084608:110930-110994:-	ACA1_074900	E3 SUMOprotein ligase NSE2, putative [Source:UniProtKB/TrEMBL;Acc:L8HGT6]	0.008849567	0.406809	0.397959433	1.44E-06
6h	ACA1_332820/clean/scf7180000084579:48411-48501:+	ACA1_332820	Sel1 repeatcontaining protein [Source:UniProtKB/TrEMBL;Acc:L8GLV3]	0.01693413	0.414769	0.39783487	9.97E-12
6h	ACA1_002750/clean/scf7180000082937:5164-5232:-	ACA1_002750	F-box domain-containing protein [Source:UniProtKB/TrEMBL;Acc:L8GHD2]	0.0425942	0.440425333	0.397831133	3.96E-09
6h	ACA1_040470/clean/scf7180000084572:9158-9247:+	ACA1_040470	Leuk-A4-hydro_C domain-containing protein [Source:UniProtKB/TrEMBL;Acc:L8H0K2]	0.00349287	0.401309	0.39781613	8.96E-11
6h	ACA1_295270/clean/scf7180000084777:1087863-1087956:-	ACA1_295270	Rhodanese domain-containing protein [Source:UniProtKB/TrEMBL;Acc:L8HJQ3]	0.049601533	0.447353333	0.3977518	3.90E-10
6h	ACA1_052260/clean/scf7180000084594:24689-24763:-	ACA1_052260	Leucine rich repeat domain containing protein [Source:UniProtKB/TrEMBL;Acc:L8H5N7]	0.091622933	0.489366333	0.3977434	8.93E-05
6h	ACA1_178500/clean/scf7180000084743:245428-245516:-	ACA1_178500	Coppertranslocating P-type ATPase [Source:UniProtKB/TrEMBL;Acc:L8GVU0]	0.039595267	0.437326	0.397730733	3.97E-07
6h	ACA1_126460/clean/scf7180000084693:72672-72751:+	ACA1_126460	hypothetical protein	0	0.397686333	0.397686333	1.31E-09
6h	ACA1_377500/clean/scf7180000084711:76640-76705:+	ACA1_377500	Peptidase family c78 protein [Source:UniProtKB/TrEMBL;Acc:L8GU78]	0.007333007	0.405005	0.397671993	9.13E-08
6h	ACA1_108260/clean/scf7180000084661:23738-23802:-	ACA1_108260	Prenylcys_lyase domain-containing protein [Source:UniProtKB/TrEMBL;Acc:L8GD56]	0.002641167	0.400267333	0.397626167	1.08E-07
6h	ACA1_113440/clean/scf7180000084669:276420-276530:+	ACA1_113440	hypothetical protein	0.006132713	0.403714	0.397581287	2.48E-15
6h	ACA1_045670/clean/scf7180000084576:171239-171309:-	ACA1_045670	Tyrosine-protein phosphatase domain-containing protein [Source:UniProtKB/TrEMBL;Acc:L8GZ41]	0.114196233	0.511756333	0.3975601	2.55E-05
6h	ACA1_329820/clean/scf7180000084566:3372-3461:-	ACA1_329820	Transporter, major facilitator subfamily protein [Source:UniProtKB/TrEMBL;Acc:L8GHX2]	0.01695329	0.414248333	0.397295043	7.05E-16
6h	ACA1_266110/clean/scf7180000084770:605502-605578:+	ACA1_266110	B30.2/SPRY domain-containing protein [Source:UniProtKB/TrEMBL;Acc:L8H2B4]	0.013200367	0.410488333	0.397287967	1.05E-07
6h	ACA1_216040/clean/scf7180000084758:440784-440910:-	ACA1_216040	Protein kinase domain-containing protein [Source:UniProtKB/TrEMBL;Acc:L8GQH1]	0.016837913	0.414044667	0.397206753	4.27E-13
6h	ACA1_237860/clean/scf7180000084763:34391-34510:+	ACA1_237860	Glycerophosphodiester phosphodiesterase family protein [Source:UniProtKB/TrEMBL;Acc:L8GKN2]	0.007643927	0.404741333	0.397097407	5.30E-12
6h	ACA1_228740/clean/scf7180000084761:203777-203876:-	ACA1_228740	hypothetical protein	0.001410607	0.398436333	0.397025727	3.55E-11
6h	ACA1_238440/clean/scf7180000084763:95336-95452:+	ACA1_238440	UDG domain-containing protein [Source:UniProtKB/TrEMBL;Acc:L8GJQ7]	0.006596397	0.403610667	0.39701427	3.98E-07
6h	ACA1_088870/clean/scf7180000084639:252540-252597:-	ACA1_088870	Checkpoint protein [Source:UniProtKB/TrEMBL;Acc:L8GX47]	0	0.396948	0.396948	5.62E-07
6h	ACA1_315050/clean/scf7180000083887:24482-24590:-	ACA1_315050	Chain a, 4ank: a designed ankyrin repeat protein [Source:UniProtKB/TrEMBL;Acc:L8GYU2]	0.012149587	0.409066667	0.39691708	1.62E-11
6h	ACA1_388490/clean/scf7180000084733:125693-125768:+	ACA1_388490	ANK_REP_REGION domain-containing protein [Source:UniProtKB/TrEMBL;Acc:L8GEA1]	0.0285538	0.425467	0.3969132	2.59E-07
6h	ACA1_144350/clean/scf7180000084709:212889-212979:+	ACA1_144350	CN hydrolase domain-containing protein [Source:UniProtKB/TrEMBL;Acc:L8HEV4]	0.036287367	0.433112	0.396824633	5.69E-11
6h	ACA1_128500/clean/scf7180000084697:73108-73227:-	ACA1_128500	UBIQUITIN_CONJUGAT_2 domain-containing protein [Source:UniProtKB/TrEMBL;Acc:L8GWJ5]	0.003918737	0.400667333	0.396748597	1.37E-08
6h	ACA1_209120/clean/scf7180000084757:276405-276512:+	ACA1_209120	Leucine rich repeat domain containing protein [Source:UniProtKB/TrEMBL;Acc:L8GYC0]	0.004166667	0.400904667	0.396738	3.37E-08
6h	ACA1_088900/clean/scf7180000084639:256988-257078:-	ACA1_088900	hypothetical protein	0	0.396732	0.396732	8.58E-08
6h	ACA1_123760/clean/scf7180000084686:55557-55645:+	ACA1_123760	Protein kinase domain-containing protein [Source:UniProtKB/TrEMBL;Acc:L8HF31]	0.171643133	0.568265	0.396621867	9.29E-06
6h	ACA1_264610/clean/scf7180000084770:336586-336677:-	ACA1_264610	Peptidase_M28 domain-containing protein [Source:UniProtKB/TrEMBL;Acc:L8H243]	0	0.396616667	0.396616667	1.78E-12
6h	ACA1_264440/clean/scf7180000084770:287964-288021:+	ACA1_264440	Sulfurtransferase [Source:UniProtKB/TrEMBL;Acc:L8H202]	0.0693797	0.465882667	0.396502967	4.13E-09
6h	ACA1_114590/clean/scf7180000084669:517824-517946:-	ACA1_114590	Proteasome activator protein [Source:UniProtKB/TrEMBL;Acc:L8H6L8]	0.008381483	0.404876667	0.396495183	2.06E-13
6h	ACA1_057520/clean/scf7180000084599:156398-156467:+	ACA1_057520	NTP_transf_2 domain-containing protein [Source:UniProtKB/TrEMBL;Acc:L8GYG1]	0	0.396392333	0.396392333	6.68E-06
6h	ACA1_258290/clean/scf7180000084769:221649-221726:+	ACA1_258290	hypothetical protein	0.213975	0.610296333	0.396321333	0.000171662
6h	ACA1_048540/clean/scf7180000084582:141505-141676:+	ACA1_048540	hypothetical protein	0.028926967	0.425229	0.396302033	0.001692232
6h	ACA1_054820/clean/scf7180000084594:578865-578942:-	ACA1_054820	Tetratricopeptide repeat domain containing protein [Source:UniProtKB/TrEMBL;Acc:L8H8E7]	0.005473423	0.40171	0.396236577	2.42E-07
6h	ACA1_063730/clean/scf7180000084599:1488428-1488504:-	ACA1_063730	Tetratricopeptide repeat domain containing protein [Source:UniProtKB/TrEMBL;Acc:L8GWN4]	0.150437633	0.546545667	0.396108033	7.77E-05
6h	ACA1_385820/clean/scf7180000084730:275715-275809:+	ACA1_385820	SH2 domain/protein kinase domain containing protein [Source:UniProtKB/TrEMBL;Acc:L8H8H1]	0	0.396069333	0.396069333	9.41E-10
6h	ACA1_042070/clean/scf7180000084573:52272-52368:+	ACA1_042070	Dual specificity protein kinase [Source:UniProtKB/TrEMBL;Acc:L8GW91]	0.012617513	0.408616667	0.395999153	6.93E-10
6h	ACA1_098920/clean/scf7180000084646:64080-64186:-	ACA1_098920	DH domain-containing protein [Source:UniProtKB/TrEMBL;Acc:L8H5S9]	0.000388437	0.396310333	0.395921897	1.93E-10
6h	ACA1_276780/clean/scf7180000084774:212241-212370:-	ACA1_276780	unspecified product	0.067836033	0.46348	0.395643967	1.01E-06
6h	ACA1_367310/clean/scf7180000084641:597334-597403:-	ACA1_367310	CCHC-type domain-containing protein [Source:UniProtKB/TrEMBL;Acc:L8GMZ2]	0.0430025	0.438627	0.3956245	2.36E-09
6h	ACA1_176030/clean/scf7180000084741:1108424-1108510:-	ACA1_176030	ATPase, AAA domain containing protein [Source:UniProtKB/TrEMBL;Acc:L8HI90]	0.007083067	0.402651333	0.395568267	9.91E-07
6h	ACA1_106820/clean/scf7180000084660:140689-140833:-	ACA1_106820	AAA domain-containing protein [Source:UniProtKB/TrEMBL;Acc:L8GQA4]	0.096254	0.491793333	0.395539333	8.98E-06
6h	ACA1_358270/clean/scf7180000084742:1842-1914:-	ACA1_358270	Pyr_redox_2 domain-containing protein [Source:UniProtKB/TrEMBL;Acc:L8GL60]	0	0.395492667	0.395492667	2.84E-06
6h	ACA1_306840/clean/scf7180000083668:17866-17944:+	ACA1_306840	RTP1_C1 domain-containing protein [Source:UniProtKB/TrEMBL;Acc:L8GQF6]	0	0.395459333	0.395459333	4.00E-07
6h	ACA1_221030/clean/scf7180000084758:982492-982571:-	ACA1_221030	DUF1992 domain-containing protein [Source:UniProtKB/TrEMBL;Acc:L8GQX4]	0.01413413	0.409537667	0.395403537	1.37E-06
6h	ACA1_259980/clean/scf7180000084769:398355-398534:+	ACA1_259980	DUF4042 domain-containing protein [Source:UniProtKB/TrEMBL;Acc:L8GFM1]	0.274379667	0.669759333	0.395379667	0.003550295
6h	ACA1_183970/clean/scf7180000084745:534067-534158:-	ACA1_183970	hypothetical protein	0	0.395306333	0.395306333	0.000393026
6h	ACA1_278330/clean/scf7180000084776:197150-197238:+	ACA1_278330	WH1 domain containing protein [Source:UniProtKB/TrEMBL;Acc:L8H6L6]	0.039173133	0.434472667	0.395299533	2.61E-10
6h	ACA1_369100/clean/scf7180000084664:245498-245570:+	ACA1_369100	IPPc domain-containing protein [Source:UniProtKB/TrEMBL;Acc:L8H092]	0.0148148	0.410062333	0.395247533	1.26E-05
6h	ACA1_252610/clean/scf7180000084768:188656-188734:-	ACA1_252610	ADPribosylglycohydrolase superfamily protein [Source:UniProtKB/TrEMBL;Acc:L8HD40]	0.0189001	0.414076	0.3951759	4.87E-11
6h	ACA1_365390/clean/scf7180000084641:258387-258485:+	ACA1_365390	Ubiquitin domain containing protein [Source:UniProtKB/TrEMBL;Acc:L8GPA8]	0.000621503	0.395593333	0.39497183	2.27E-08

6h	ACA1_135650/clean/scf7180000084702:99776-99849:-	ACA1_135650	MYND finger domain containing protein [Source:UniProtKB/TrEMBL;Acc:L8GGQ8]	0.232656	0.627465333	0.394809333	0.000323001
6h	ACA1_052260/clean/scf7180000084594:24301-24407:-	ACA1_052260	Leucine rich repeat domain containing protein [Source:UniProtKB/TrEMBL;Acc:L8H5N7]	0.003010753	0.397808333	0.39479758	3.69E-07
6h	ACA1_063400/clean/scf7180000084599:1400743-1400821:+	ACA1_063400	Prepeptidase C-terminal domain containing protein [Source:UniProtKB/TrEMBL;Acc:L8GZQ0]	0.01160976	0.406382	0.39477224	7.27E-12
6h	ACA1_112660/clean/scf7180000084669:210662-210737:+	ACA1_112660	BTB_2 domain-containing protein [Source:UniProtKB/TrEMBL;Acc:M0QSU3]	0	0.394675667	0.394675667	1.37E-08
6h	ACA1_156020/clean/scf7180000084723:3918-4054:-	ACA1_156020	AT hook motif domain containing protein [Source:UniProtKB/TrEMBL;Acc:L8GHF9]	0.0275763	0.422233333	0.394657033	1.49E-05
6h	ACA1_234100/clean/scf7180000084762:199826-199959:+	ACA1_234100	G_PROTEIN_RECEP_F2_4 domain-containing protein [Source:UniProtKB/TrEMBL;Acc:L8H173]	0.007878367	0.402523	0.394644633	3.16E-08
6h	ACA1_113850/clean/scf7180000084669:387282-387362:-	ACA1_113850	PMSR domain-containing protein [Source:UniProtKB/TrEMBL;Acc:L8H3F4]	0.004701739	0.399344333	0.394642594	2.97E-19
6h	ACA1_145740/clean/scf7180000084710:137045-137117:+	ACA1_145740	CBS domain containing protein [Source:UniProtKB/TrEMBL;Acc:L8GFG0]	0.0956881	0.490301	0.3946129	4.23E-07
6h	ACA1_018140/clean/scf7180000083885:16722-16811:-	ACA1_018140	Leucine-tRNA ligase [Source:UniProtKB/TrEMBL;Acc:L8GL00]	0.032584233	0.427175333	0.3945911	9.92E-07
6h	ACA1_200000/clean/scf7180000084753:365602-365700:-	ACA1_200000	LAO/AO transport system ATPase [Source:UniProtKB/TrEMBL;Acc:L8H2I2]	0.011536987	0.406104	0.394567013	2.25E-08
6h	ACA1_101470/clean/scf7180000084649:69592-69696:+	ACA1_101470	Dynactin 4 isoform 2, putative [Source:UniProtKB/TrEMBL;Acc:L8GIW5]	0.037328233	0.431867667	0.394539433	2.66E-10
6h	ACA1_309840/clean/scf7180000083767:18017-18108:+	ACA1_309840	1-aminocyclopropane-1-carboxylate deaminase [Source:UniProtKB/TrEMBL;Acc:L8HH90]	0.1221465	0.516634	0.3944875	8.15E-07
6h	ACA1_171280/clean/scf7180000084741:63075-63177:+	ACA1_171280	Nicotinate phosphoribosyltransferase [Source:UniProtKB/TrEMBL;Acc:L8HHA8]	0.065919533	0.460307333	0.3943878	0.00016646
6h	ACA1_209120/clean/scf7180000084757:276070-276146:+	ACA1_209120	Leucine rich repeat domain containing protein [Source:UniProtKB/TrEMBL;Acc:L8GYC0]	0.000636227	0.395013333	0.394377107	1.63E-09
6h	ACA1_360290/clean/scf7180000084622:83356-83419:+	ACA1_360290	Singlestrand binding protein family [Source:UniProtKB/TrEMBL;Acc:L8HBV6]	0.0100644	0.404333	0.3942686	1.22E-12
6h	ACA1_125100/clean/scf7180000084691:30240-30322:-	ACA1_125100	hypothetical protein	0.014449267	0.408627333	0.394178067	2.06E-07
6h	ACA1_277740/clean/scf7180000084776:90718-90790:+	ACA1_277740	MORN repeat-containing protein [Source:UniProtKB/TrEMBL;Acc:L8H6R9]	0	0.394148	0.394148	2.09E-05
6h	ACA1_298630/clean/scf7180000082992:4522-4647:-	ACA1_298630	Vacuolar protein sorting 16, putative [Source:UniProtKB/TrEMBL;Acc:L8H081]	0	0.393984333	0.393984333	4.65E-11
6h	ACA1_171960/clean/scf7180000084741:208318-208384:-	ACA1_171960	F-box domain-containing protein [Source:UniProtKB/TrEMBL;Acc:L8HHE3]	0.028125233	0.422109333	0.3939841	8.82E-07
6h	ACA1_033770/clean/scf7180000084559:129571-129655:+	ACA1_033770	RING-type domain-containing protein [Source:UniProtKB/TrEMBL;Acc:L8GJ68]	0.0560528	0.450019667	0.393966867	1.49E-07
6h	ACA1_174730/clean/scf7180000084741:783690-783814:-	ACA1_174730	Guanine deaminase [Source:UniProtKB/TrEMBL;Acc:L8HK76]	0.005223333	0.399113667	0.393890333	8.72E-07
6h	ACA1_099100/clean/scf7180000084646:89827-89916:+	ACA1_099100	PH domain-containing protein [Source:UniProtKB/TrEMBL;Acc:L8H5M3]	0.001867597	0.395693333	0.393825737	1.37E-09
6h	ACA1_234250/clean/scf7180000084762:252108-252203:-	ACA1_234250	Protein kinase domain-containing protein [Source:UniProtKB/TrEMBL;Acc:L8H190]	0.007032747	0.400837333	0.393804587	1.86E-10
6h	ACA1_035600/clean/scf7180000084564:97530-97598:+	ACA1_035600	Cupin_2 domain-containing protein [Source:UniProtKB/TrEMBL;Acc:L8HAL8]	0.034210533	0.427963667	0.393753133	0.000153284
6h	ACA1_063300/clean/scf7180000084599:1377784-1377848:-	ACA1_063300	Transporter, small conductance mechanosensitive ion channel (MscS) family protein [Source:UniProtKB/TrEMBL;Acc:L8GZP0]	0.009803933	0.403387667	0.393583733	5.69E-06
6h	ACA1_167040/clean/scf7180000084736:61130-61247:+	ACA1_167040	Elongation of fatty acids protein [Source:UniProtKB/TrEMBL;Acc:L8GZZ6]	0.011538847	0.405047333	0.393508487	4.54E-10
6h	ACA1_011150/clean/scf7180000083576:9030-9092:-	ACA1_011150	NTP_transferase domain-containing protein [Source:UniProtKB/TrEMBL;Acc:L8GG90]	0.005496273	0.398962	0.393465727	4.51E-08
6h	ACA1_061890/clean/scf7180000084599:1070828-1070905:-	ACA1_061890	DHO_dh domain-containing protein [Source:UniProtKB/TrEMBL;Acc:L8GWX2]	0	0.393185333	0.393185333	3.41E-07
6h	ACA1_007760/clean/scf7180000083166:21649-21712:+	ACA1_007760	PH domain containing protein [Source:UniProtKB/TrEMBL;Acc:L8HAY1]	0	0.393070667	0.393070667	4.09E-08
6h	ACA1_273530/clean/scf7180000084773:45176-45273:+	ACA1_273530	hypothetical protein	0.038549067	0.431523	0.392973933	7.35E-08
6h	ACA1_141780/clean/scf7180000084708:21735-21814:+	ACA1_141780	unspecified product	0.005812733	0.398781667	0.392968933	8.21E-10
6h	ACA1_064250/clean/scf7180000084599:1570056-1570133:+	ACA1_064250	AP-2 complex subunit alpha [Source:UniProtKB/TrEMBL;Acc:L8GZW9]	0.000594253	0.393541333	0.39294708	4.93E-11
6h	ACA1_346900/clean/scf7180000084658:41959-42022:+	ACA1_346900	Cnd3 domain-containing protein [Source:UniProtKB/TrEMBL;Acc:L8GHD4]	0.00275602	0.395473	0.39271698	1.07E-08
6h	ACA1_226550/clean/scf7180000084761:50597-50687:+	ACA1_226550	Histidine ammonia-lyase [Source:UniProtKB/TrEMBL;Acc:L8H7Q1]	0	0.392676	0.392676	2.80E-10
6h	ACA1_176030/clean/scf7180000084741:1110009-1110075:-	ACA1_176030	ATPase, AAA domain containing protein [Source:UniProtKB/TrEMBL;Acc:L8HI90]	0	0.392636667	0.392636667	3.82E-06
6h	ACA1_300890/clean/scf7180000083147:17584-17679:-	ACA1_300890	hypothetical protein	0.004194979	0.396776667	0.392581688	3.41E-13
6h	ACA1_231570/clean/scf7180000084761:442917-442972:+	ACA1_231570	hypothetical protein	0.097222333	0.489773667	0.392551333	0.000784492
6h	ACA1_157300/clean/scf7180000084724:35095-35178:+	ACA1_157300	TOPEUc domain-containing protein [Source:UniProtKB/TrEMBL;Acc:L8HB99]	0.067748667	0.460282667	0.392534	3.57E-07
6h	ACA1_231690/clean/scf7180000084761:445154-445228:-	ACA1_231690	hypothetical protein	0.033262633	0.425691	0.392428367	6.37E-07
6h	ACA1_379350/clean/scf7180000084711:339829-339925:+	ACA1_379350	hypothetical protein	0.056525963	0.448881	0.392355037	0.000754387
6h	ACA1_070460/clean/scf7180000084603:687875-687944:-	ACA1_070460	J domain-containing protein [Source:UniProtKB/TrEMBL;Acc:L8HFA5]	0.163113033	0.555365333	0.3922523	0.036174699
6h	ACA1_287660/clean/scf7180000084777:57133-57198:+	ACA1_287660	Pentapeptide repeatcontaining protein [Source:UniProtKB/TrEMBL;Acc:L8HIP0]	0.0064725	0.398583	0.3921105	1.41E-08
6h	ACA1_202430/clean/scf7180000084754:43540-43610:-	ACA1_202430	Clat_adaptor_s domain-containing protein [Source:UniProtKB/TrEMBL;Acc:L8GW07]	0.042523333	0.434507	0.391983667	2.00E-07
6h	ACA1_092600/clean/scf7180000084645:363984-364061:-	ACA1_092600	NLPC_P60 domain-containing protein [Source:UniProtKB/TrEMBL;Acc:L8GIC1]	0.00530958	0.397287333	0.391977753	2.00E-12
6h	ACA1_138490/clean/scf7180000084703:151741-151805:-	ACA1_138490	Tyrosine-protein phosphatase domain-containing protein [Source:UniProtKB/TrEMBL;Acc:L8GZM7]	0.007637467	0.399475667	0.3918382	9.52E-08
6h	ACA1_170290/clean/scf7180000084739:85997-86093:+	ACA1_170290	tRNA_edit domain-containing protein [Source:UniProtKB/TrEMBL;Acc:L8HIQ9]	0.179402	0.571226667	0.391824667	0.00379269
6h	ACA1_378490/clean/scf7180000084711:230233-230313:-	ACA1_378490	hypothetical protein	0.02338755	0.415108333	0.391720783	3.07E-08
6h	ACA1_191490/clean/scf7180000084749:3530-3630:-	ACA1_191490	RTR1-type domain-containing protein [Source:UniProtKB/TrEMBL;Acc:L8GQP2]	0.000106547	0.391788667	0.391682119	6.19E-11
6h	ACA1_077270/clean/scf7180000084612:275002-275145:+	ACA1_077270	RBR-type E3 ubiquitin transferase [Source:UniProtKB/TrEMBL;Acc:L8GM90]	0.0107527	0.402361	0.3916083	7.16E-06
6h	ACA1_144430/clean/scf7180000084709:240217-240384:-	ACA1_144430	CASP_C domain-containing protein [Source:UniProtKB/TrEMBL;Acc:L8HI28]	0.028859067	0.420437333	0.391578267	1.51E-12
6h	ACA1_330410/clean/scf7180000084566:54038-54138:+	ACA1_330410	hypothetical protein	0.00648702	0.397909667	0.391422647	3.17E-10
6h	ACA1_009890/clean/scf7180000083468:804-909:-	ACA1_009890	TPR_REGION domain-containing protein [Source:UniProtKB/TrEMBL;Acc:L8GDE6]	0.006024383	0.397374333	0.39134995	6.84E-14
6h	ACA1_287850/clean/scf7180000084777:96448-96521:-	ACA1_287850	RING-type domain-containing protein [Source:UniProtKB/TrEMBL;Acc:L8HHQ7]	0	0.391213	0.391213	6.06E-10
6h	ACA1_310750/clean/scf7180000083780:6657-6735:+	ACA1_310750	RFX-type winged-helix domain-containing protein [Source:UniProtKB/TrEMBL;Acc:L8GVY4]	0	0.391203333	0.391203333	2.46E-08
6h	ACA1_150950/clean/scf7180000084716:34732-34875:+	ACA1_150950	Exocyst complex component Sec6 [Source:UniProtKB/TrEMBL;Acc:L8H215]	0.004260212	0.395342667	0.391082454	8.35E-08
6h	ACA1_367180/clean/scf7180000084641:563525-563594:-	ACA1_367180	Profilin [Source:UniProtKB/TrEMBL;Acc:L8GM22]	0	0.391062333	0.391062333	0.034866997
6h	ACA1_133510/clean/scf7180000084701:73196-73283:+	ACA1_133510	Glutathione peroxidase [Source:UniProtKB/TrEMBL;Acc:L8H527]	0.000458407	0.391491333	0.391032926	1.92E-14
6h	ACA1_384860/clean/scf7180000084730:117276-117365:+	ACA1_384860	Isochorismatase domain-containing protein [Source:UniProtKB/TrEMBL;Acc:L8H995]	0.001785793	0.392799	0.391013207	6.37E-20
6h	ACA1_116900/clean/scf7180000084669:868835-868951:+	ACA1_116900	UBA/TSN domain containing protein [Source:UniProtKB/TrEMBL;Acc:L8H4S4]	0.059857767	0.450864	0.391006233	1.29E-08
6h	ACA1_072950/clean/scf7180000084603:1342236-1342314:+	ACA1_072950	Peptidase M16 inactive domain containing protein [Source:UniProtKB/TrEMBL;Acc:L8HGW3]	0	0.390992	0.390992	5.63E-06
6h	ACA1_070900/clean/scf7180000084603:797775-797923:+	ACA1_070900	MMtag domain-containing protein [Source:UniProtKB/TrEMBL;Acc:L8HD49]	0.071844733	0.462815667	0.390970933	3.46E-07
6h	ACA1_095220/clean/scf7180000084645:815360-815477:-	ACA1_095220	Protein kinase domain-containing protein [Source:UniProtKB/TrEMBL;Acc:L8GIR4]	0.013289623	0.404227333	0.39093771	2.53E-10
6h	ACA1_281240/clean/scf7180000084776:570117-570226:+	ACA1_281240	Dual specificity protein kinase shka, putative [Source:UniProtKB/TrEMBL;Acc:L8H7B2]	0.013972333	0.404902667	0.390930333	7.18E-19
6h	ACA1_224310/clean/scf7180000084760:166677-166744:+	ACA1_224310	PH domain-containing protein [Source:UniProtKB/TrEMBL;Acc:L8GTW7]	0.001821493	0.392701	0.390879507	2.04E-09
6h	ACA1_037840/clean/scf7180000084568:34677-34741:-	ACA1_037840	hypothetical protein	0.513561333	0.904426333	0.390865	0.046167078
6h	ACA1_268920/clean/scf7180000084770:942148-942262:-	ACA1_268920	Transket_pyr domain-containing protein [Source:UniProtKB/TrEMBL;Acc:L8H4W8]	0.001924325	0.392704667	0.390780341	7.46E-11
6h	ACA1_054820/clean/scf7180000084594:585013-585108:-	ACA1_054820	Tetratricopeptide repeat domain containing protein [Source:UniProtKB/TrEMBL;Acc:L8H8E7]	0.000306091	0.39103	0.390723909	3.43E-09
6h	ACA1_338000/clean/scf7180000084604:23377-23439:-	ACA1_338000	hypothetical protein	0.001175155	0.391863667	0.390688512	1.02E-14
6h	ACA1_297530/clean/scf7180000082850:1266-1335:-	ACA1_297530	hypothetical protein	0.002523317	0.393142667	0.39061935	1.19E-07
6h	ACA1_050200/clean/scf7180000084589:53819-53916:+	ACA1_050200	Mammalian PTEN tumor suppressorlike, putative [Source:UniProtKB/TrEMBL;Acc:L8H3Q5]	0.004432267	0.395035	0.390602733	8.61E-07
6h	ACA1_054220/clean/scf7180000084594:434183-434258:-	ACA1_054220	MHCK/EF2 kinase domain containing protein [Source:UniProtKB/TrEMBL;Acc:L8H897]	0.193490667	0.584081	0.390590333	7.42E-06
6h	ACA1_279550/clean/scf7180000084776:412516-412590:-	ACA1_279550	CBS domain containing protein [Source:UniProtKB/TrEMBL;Acc:L8H8C1]	0.025535933	0.416007667	0.390471733	3.76E-09
6h	ACA1_245070/clean/scf7180000084763:467622-467724:-	ACA1_245070	Aminotransferase class IV, putative [Source:UniProtKB/TrEMBL;Acc:L8GMK1]	0.01328585	0.403707667	0.390421817	1.27E-10
6h	ACA1_369480/clean/scf7180000084664:310081-310164:-	ACA1_369480	SMP-LTD domain-containing protein [Source:UniProtKB/TrEMBL;Acc:L8GYZ6]	0.016375233	0.406777667	0.390402433	6.38E-10
6h	ACA1_054040/clean/scf7180000084594:380163-380256:-	ACA1_054040	F-box domain-containing protein [Source:UniProtKB/TrEMBL;Acc:L8H6A3]	0.003264483	0.39326	0.389995517	6.67E-08
6h	ACA1_203000/clean/scf7180000084754:115511-115576:+	ACA1_203000	PGM1_C domain-containing protein [Source:UniProtKB/TrEMBL;Acc:L8GW29]	0.00480527	0.394761333	0.389956063	2.41E-11
6h	ACA1_116990/clean/scf7180000084669:886931-887010:+	ACA1_116990	Carboxypeptidase [Source:UniProtKB/TrEMBL;Acc:L8H6Y6]	0.00242047	0.392299	0.38987853	2.16E-10
6h	ACA1_306700/clean/scf7180000083668:7515-7665:+	ACA1_306700	F-box domain-containing protein [Source:UniProtKB/TrEMBL;Acc:L8GNY1]	0	0.389845667	0.389845667	5.04E-09
6h	ACA1_218090/clean/scf7180000084758:622886-622990:-	ACA1_218090	AB hydrolase-1 domain-containing protein [Source:UniProtKB/TrEMBL;Acc:L8GSU3]	0.011278023	0.401103667	0.389825643	9.32E-08
6h	ACA1_063340/clean/scf7180000084599:1391990-1392050:+	ACA1_063340	Dynein intermediate chain, cytosolic, putative [Source:UniProtKB/TrEMBL;Acc:L8GX48]	0.01439298	0.404202333	0.389809353	3.09E-10
6h	ACA1_053350/clean/scf7180000084594:267829-267951:+	ACA1_053350	Histone domain-containing protein [Source:UniProtKB/TrEMBL;Acc:L8H5Y7]	0.031514063	0.421269667	0.389755603	3.67E-08
6h	ACA1_074140/clean/scf7180000084605:35012-35116:+	ACA1_074140	Transporter, major facilitator subfamily protein [Source:UniProtKB/TrEMBL;Acc:L8HK94]	0.00196727	0.391449667	0.389482397	2.69E-10
6h	ACA1_076210/clean/scf7180000084612:26292-26379:-	ACA1_076210	hypothetical protein	0.000663633	0.390092	0.389428367	1.36E-10
6h	ACA1_287820/clean/scf7180000084777:86411-86508:+	ACA1_287820	TRANSKETOLASE_1 domain-containing protein [Source:UniProtKB/TrEMBL;Acc:L8HL41]	0.000659283	0.390081667	0.389422383	1.59E-11
6h	ACA1_024000/clean/scf7180000084440:1464-1559:+	ACA1_024000	C2H2-type domain-containing protein [Source:UniProtKB/TrEMBL;Acc:L8GHS6]	0.038896827	0.428229667	0.38933284	0.000734063
6h	ACA1_117080/clean/scf7180000084669:909747-909821:+	ACA1_117080	PALP domain-containing protein [Source:UniProtKB/TrEMBL;Acc:L8H4Y6]	0	0.389259	0.389259	1.11E-11
6h	ACA1_175760/clean/scf7180000084741:1053427-1053544:-	ACA1_175760	Phosphoglycerate mutase family domain containing protein [Source:UniProtKB/TrEMBL;Acc:L8HH51]	0.046168567	0.435399333	0.389230767	4.65E-08
6h	ACA1_227780/clean/scf7180000084761:135981-136077:+	ACA1_227780	NAD(P)-bd_dom domain-containing protein [Source:UniProtKB/TrEMBL;Acc:L8H8P3]	0.012522133	0.401724	0.389201867	2.03E-09
6h	ACA1_290440/clean/scf7180000084777:593300-593391:-	ACA1_290440	hypothetical protein	0.010107433	0.399258667	0.389151233	3.38E-06
6h	ACA1_320830/clean/scf7180000084416:30264-30365:+	ACA1_320830	Lipoyl-binding domain-containing protein [Source:UniProtKB/TrEMBL;Acc:L8GT97]	0.000984584	0.390126	0.389141416	1.77E-24
6h	ACA1_227780/clean/scf7180000084761:135595-135673:+	ACA1_227780	NAD(P)-bd_dom domain-containing protein [Source:UniProtKB/TrEMBL;Acc:L8H8P3]	0.006944433	0.396070667	0.389126233	9.51E-08
6h	ACA1_063490/clean/scf7180000084599:1429894-1429974:-	ACA1_063490	Peptidylprolyl isomerase [Source:UniProtKB/TrEMBL;Acc:L8GX87]	0.051851867	0.440957333	0.389105467	0.006243579
6h	ACA1_234280/clean/scf7180000084762:259084-259148:+	ACA1_234280	hypothetical protein	0.1059255	0.494971333	0.389045833	1.22E-07
6h	ACA1_395210/clean/scf7180000084756:155873-155953:-	ACA1_395210	Nonspecific lipidtransfer protein [Source:UniProtKB/TrEMBL;Acc:L8GZN6]	0	0.388909333	0.388909333	1.05E-07
6h	ACA1_015560/clean/scf7180000083799:20456-20518:+	ACA1_015560	hypothetical protein	0	0.388886	0.388886	5.43E-13
6h	ACA1_315640/clean/scf7180000083939:10508-10582:+	ACA1_315640	Aldedh domain-containing protein [Source:UniProtKB/TrEMBL;Acc:L8H0Y9]	0.0148148	0.403642	0.3888272	5.04E-06
6h	ACA1_135150/clean/scf7180000084702:75201-75253:-	ACA1_135150	hypothetical protein	0.357347333	0.746155	0.388807667	0.004015834
6h	ACA1_374710/clean/scf7180000084679:372778-372862:+	ACA1_374710	WD_REPEATS_REGION domain-containing protein [Source:UniProtKB/TrEMBL;Acc:L8GHC6]	0.0070922	0.395776333	0.388684133	2.53E-06
6h	ACA1_081870/clean/scf7180000084618:152198-152305:+	ACA1_081870	HIV1 Tat interactive protein 2, 30kDa, putative [Source:UniProtKB/TrEMBL;Acc:L8HE84]	0.017905133	0.406547333	0.3886422	1.58E-11
6h	ACA1_218620/clean/scf7180000084758:680745-680883:-	ACA1_218620	Carboxylic ester hydrolase [Source:UniProtKB/TrEMBL;Acc:L8GRD3]	8.54E-05	0.388718	0.388632639	2.87E-09
6h	ACA1_370140/clean/scf7180000084664:448611-448699:+	ACA1_370140	RFX-type winged-helix domain-containing protein [Source:UniProtKB/TrEMBL;Acc:L8GZ40]	0.022544933	0.411169	0.388624067	5.48E-11
6h	ACA1_097270/clean/scf7180000084645:1203414-1203483:-	ACA1_097270	Serine/threonine protein kinase [Source:UniProtKB/TrEMBL;Acc:L8GJZ3]	0	0.388603	0.388603	3.66E-07
6h	ACA1_133350/clean/scf7180000084701:36463-36575:-	ACA1_133350	PlsC domain-containing protein [Source:UniProtKB/TrEMBL;Acc:L8H3L0]	0.00278495	0.391375667	0.388590716	4.00E-09
6h	ACA1_183010/clean/scf7180000084745:310040-310117:+	ACA1_183010	ABC1 domain-containing protein [Source:UniProtKB/TrEMBL;Acc:L8H7Z3]	0	0.388561333	0.388561333	6.03E-07
6h	ACA1_268430/clean/scf7180000084770:905997-906114:-	ACA1_268430	SAM domain (Sterile alpha motif) domain containing protein [Source:UniProtKB/TrEMBL;Acc:L8H2M3]	0.340432333	0.728935	0.388502667	0.000274983
6h	ACA1_115370/clean/scf7180000084669:616951-617018:+	ACA1_115370	hypothetical protein	7.59E-05	0.388497333	0.388421403	2.91E-12
6h	ACA1_378040/clean/scf7180000084711:177186-177287:-	ACA1_378040	RING-CH-type domain-containing protein [Source:UniProtKB/TrEMBL;Acc:L8GRZ8]	0.00258112	0.390963667	0.388382547	1.26E-07
6h	ACA1_054180/clean/scf7180000084594:419404-419581:+	ACA1_054180	hypothetical protein	0.008972427	0.397324333	0.388351907	1.24E-08
6h	ACA1_053660/clean/scf7180000084594:310213-310317:-	ACA1_053660	TNF receptor-associated protein 1, putative [Source:UniProtKB/TrEMBL;Acc:L8H601]	0	0.388192333	0.388192333	4.15E-13
6h	ACA1_339940/clean/scf7180000084619:43733-43837:-	ACA1_339940	Tetratricopeptide repeat domain containing protein [Source:UniProtKB/TrEMBL;Acc:L8GM04]	0	0.388157667	0.388157667	6.83E-08
6h	ACA1_025560/clean/scf7180000084448:14272-14352:-	ACA1_025560	hypothetical protein	0.171717	0.559801333	0.388084333	0.028266684
6h	ACA1_024430/clean/scf7180000084442:25766-25842:+	ACA1_024430	hypothetical protein	0.010065137	0.398102667	0.38803753	1.89E-05
6h	ACA1_369150/clean/scf7180000084664:260165-260245:-	ACA1_369150	Chorein_N domain-containing protein [Source:UniProtKB/TrEMBL;Acc:L8H096]	0	0.387990333	0.387990333	1.36E-09
6h	ACA1_132620/clean/scf7180000084700:46169-46261:-	ACA1_132620	RING-CH-type domain-containing protein [Source:UniProtKB/TrEMBL;Acc:L8GV25]	0	0.387882333	0.387882333	2.37E-06
6h	ACA1_370030/clean/scf7180000084664:410580-410664:+	ACA1_370030	hypothetical protein	0.003333333	0.391213667	0.387880333	3.39E-08
6h	ACA1_201360/clean/scf7180000084753:531001-531080:-	ACA1_201360	Regulator of chromosome condensation (RCC1) repeat domain containing protein [Source:UniProtKB/TrEMBL;Acc:L8H4W1]	0	0.387859	0.387859	1.14E-08
6h	ACA1_331480/clean/scf7180000084575:24194-24267:-	ACA1_331480	WD_REPEATS_REGION domain-containing protein [Source:UniProtKB/TrEMBL;Acc:L8GT05]	4.77E-05	0.387865667	0.387818007	9.24E-10
6h	ACA1_158500/clean/scf7180000084724:239288-239428:+	ACA1_158500	Nudix hydrolase domain-containing protein [Source:UniProtKB/TrEMBL;Acc:L8HA53]	0.024341432	0.412129667	0.387788235	5.60E-06
6h	ACA1_266510/clean/scf7180000084770:684749-684868:-	ACA1_266510	RhoGEF domain containing protein [Source:UniProtKB/TrEMBL;Acc:L8H2E9]	0.00211968	0.389900667	0.387780987	5.46E-09
6h	ACA1_071390/clean/scf7180000084603:946700-946777:+	ACA1_071390	EF hand domain containing protein [Source:UniProtKB/TrEMBL;Acc:L8HE62]	0.003345267	0.39101	0.387664733	3.13E-08
6h	ACA1_187780/clean/scf7180000084747:94180-94277:-	ACA1_187780	Corrinoid adenosyltransferase [Source:UniProtKB/TrEMBL;Acc:L8GUU3]	0.006230533	0.393868	0.387637467	5.93E-09
6h	ACA1_058280/clean/scf7180000084599:286913-287040:-	ACA1_058280	OGFr_N domain-containing protein [Source:UniProtKB/TrEMBL;Acc:L8GW80]	0.017896047	0.405384333	0.387488287	1.32E-09
6h	ACA1_297530/clean/scf7180000082850:931-1004:-	ACA1_297530	hypothetical protein	0.006744313	0.394168	0.387423687	2.27E-10
6h	ACA1_190080/clean/scf7180000084748:122140-122212:-	ACA1_190080	CN hydrolase domain-containing protein [Source:UniProtKB/TrEMBL;Acc:L8GE63]	0.0256811	0.413079333	0.387398233	1.79E-08
6h	ACA1_158790/clean/scf7180000084724:256532-256679:+	ACA1_158790	Peroxiredoxin [Source:UniProtKB/TrEMBL;Acc:L8H9D0]	0	0.387353	0.387353	9.25E-15
6h	ACA1_331950/clean/scf7180000084578:50840-50966:-	ACA1_331950	RRM domain-containing protein [Source:UniProtKB/TrEMBL;Acc:L8HBN4]	0	0.387330667	0.387330667	0.000117668
6h	ACA1_169100/clean/scf7180000084739:22157-22247:+	ACA1_169100	Dual specificity phosphatase, catalytic domain containing protein [Source:UniProtKB/TrEMBL;Acc:L8HG79]	0.0227561	0.410057	0.3873009	4.66E-14
6h	ACA1_268930/clean/scf7180000084770:945260-945362:+	ACA1_268930	PWI domain-containing protein [Source:UniProtKB/TrEMBL;Acc:L8H2N5]	0.005379917	0.392676333	0.387296417	3.41E-08
6h	ACA1_100600/clean/scf7180000084648:127558-127643:+	ACA1_100600	AAA_28 domain-containing protein [Source:UniProtKB/TrEMBL;Acc:L8GKH3]	0.01147417	0.398740333	0.387266163	3.39E-10
6h	ACA1_065910/clean/scf7180000084599:1944900-1945001:+	ACA1_065910	hypothetical protein	0.02376479	0.410886333	0.387121543	9.21E-07
6h	ACA1_109100/clean/scf7180000084661:115591-115667:-	ACA1_109100	Sodium symporter, putative [Source:UniProtKB/TrEMBL;Acc:L8GC73]	0.0092744	0.396296333	0.387021933	0.000581201
6h	ACA1_097190/clean/scf7180000084645:1180754-1180820:-	ACA1_097190	JmjC domain-containing protein [Source:UniProtKB/TrEMBL;Acc:L8GIZ2]	0.051736767	0.438752	0.387015233	6.38E-06
6h	ACA1_151250/clean/scf7180000084716:82026-82159:+	ACA1_151250	Glycine cleavage Tprotein (Aminomethyl transferase) domain containing protein [Source:UniProtKB/TrEMBL;Acc:L8H0R3]	0.001387519	0.388398	0.387010481	4.54E-10
6h	ACA1_165830/clean/scf7180000084732:98918-99008:-	ACA1_165830	Glutathione transferase family protein [Source:UniProtKB/TrEMBL;Acc:L8HGI5]	0.038518967	0.425498333	0.386979367	2.27E-11
6h	ACA1_369710/clean/scf7180000084664:365819-365928:+	ACA1_369710	Hypothetical protein	0.023456767	0.410413	0.386956233	7.38E-05
6h	ACA1_243850/clean/scf7180000084763:360729-360801:+	ACA1_243850	Ras-GAP domain-containing protein [Source:UniProtKB/TrEMBL;Acc:L8GJZ5]	0.034538	0.421490333	0.386952333	0.010252097
6h	ACA1_002430/clean/scf7180000082931:1142-1228:-	ACA1_002430	G domain-containing protein [Source:UniProtKB/TrEMBL;Acc:L8GY39]	0.004372433	0.391245333	0.3868729	9.33E-08
6h	ACA1_167030/clean/scf7180000084736:59624-59738:+	ACA1_167030	hypothetical protein	0.035572567	0.422189333	0.386616767	8.84E-09

6h	ACA1_093170/clean/scf7180000084645:500708-500768:+	ACA1_093170	Kelch repeat-containing protein [Source:UniProtKB/TrEMBL;Acc:L8GKQ1]	0	0.386540667	0.386540667	0.038184272
6h	ACA1_087910/clean/scf7180000084639:128810-128956:+	ACA1_087910	hypothetical protein	0.03797552	0.424503667	0.386528147	2.27E-07
6h	ACA1_295300/clean/scf7180000084777:1096926-1097098:-	ACA1_295300	Calponin domain containing protein [Source:UniProtKB/TrEMBL;Acc:L8HJQ6]	0.001639135	0.388093	0.386453865	8.44E-10
6h	ACA1_051680/clean/scf7180000084592:49001-49082:+	ACA1_051680	Ras subfamily protein [Source:UniProtKB/TrEMBL;Acc:L8GPK3]	0.1088811	0.495286	0.3864049	1.94E-05
6h	ACA1_098210/clean/scf7180000084645:1430260-1430332:+	ACA1_098210	Peroxidase, putative [Source:UniProtKB/TrEMBL;Acc:L8GJY8]	0.004016067	0.390375	0.386358933	7.01E-07
6h	ACA1_175480/clean/scf7180000084741:967654-967753:-	ACA1_175480	PQ loop repeat-containing protein [Source:UniProtKB/TrEMBL;Acc:L8HJK6]	0.00399309	0.390296333	0.386303243	1.03E-07
6h	ACA1_235050/clean/scf7180000084762:328035-328180:+	ACA1_235050	RNA ligase, putative [Source:UniProtKB/TrEMBL;Acc:L8H1C3]	0	0.386293333	0.386293333	1.58E-07
6h	ACA1_048850/clean/scf7180000084583:685-776:-	ACA1_048850	hypothetical protein	0.0246666	0.410872667	0.386206067	1.85E-13
6h	ACA1_113490/clean/scf7180000084669:295920-296037:-	ACA1_113490	SAM domain-containing protein [Source:UniProtKB/TrEMBL;Acc:L8H3A5]	0.000812773	0.38699	0.386177227	5.52E-12
6h	ACA1_054090/clean/scf7180000084594:398950-399051:-	ACA1_054090	Fbox domain containing protein [Source:UniProtKB/TrEMBL;Acc:L8H592]	0.168644	0.554702	0.386058	0.000609399
6h	ACA1_071550/clean/scf7180000084603:990543-990625:-	ACA1_071550	F-box domain-containing protein [Source:UniProtKB/TrEMBL;Acc:L8HFM3]	0	0.386001667	0.386001667	3.68E-07
6h	ACA1_365710/clean/scf7180000084641:301242-301326:-	ACA1_365710	Protein kinase domain-containing protein [Source:UniProtKB/TrEMBL;Acc:L8GPB8]	0	0.385702667	0.385702667	4.01E-08
6h	ACA1_280640/clean/scf7180000084776:516999-517084:-	ACA1_280640	PH domain/RhoGEF domain containing protein [Source:UniProtKB/TrEMBL;Acc:L8H790]	0	0.38569	0.38569	3.93E-12
6h	ACA1_278860/clean/scf7180000084776:259142-259222:-	ACA1_278860	Glycosylphosphatidylinositol anchor attachment 1 protein [Source:UniProtKB/TrEMBL;Acc:L8H6Z3]	0	0.385667333	0.385667333	1.58E-08
6h	ACA1_130480/clean/scf7180000084698:86350-86421:-	ACA1_130480	RhoGEF domain containing protein [Source:UniProtKB/TrEMBL;Acc:L8GQC0]	0	0.385634667	0.385634667	4.41E-09
6h	ACA1_276410/clean/scf7180000084774:167410-167494:+	ACA1_276410	Phosphate transporter [Source:UniProtKB/TrEMBL;Acc:L8GTH7]	0	0.385467333	0.385467333	9.11E-09
6h	ACA1_307820/clean/scf7180000083687:20308-20395:-	ACA1_307820	BSD domain-containing protein [Source:UniProtKB/TrEMBL;Acc:L8HBM7]	0.001727117	0.387153333	0.385426217	2.26E-10
6h	ACA1_094200/clean/scf7180000084645:708399-708500:-	ACA1_094200	Palmitoyltransferase [Source:UniProtKB/TrEMBL;Acc:L8GJD4]	0.011195493	0.396583	0.385387507	1.89E-08
6h	ACA1_080550/clean/scf7180000084618:30788-30879:+	ACA1_080550	FAD binding domain containing protein [Source:UniProtKB/TrEMBL;Acc:L8HBU6]	0.046164667	0.431452667	0.385288	7.52E-10
6h	ACA1_251650/clean/scf7180000084768:105931-106012:+	ACA1_251650	Protein kinase domain-containing protein [Source:UniProtKB/TrEMBL;Acc:L8HD04]	0	0.385242667	0.385242667	1.30E-09
6h	ACA1_058840/clean/scf7180000084599:392263-392344:-	ACA1_058840	HATPase_c domain-containing protein [Source:UniProtKB/TrEMBL;Acc:L8GXB7]	0.002590934	0.38775	0.385159066	7.14E-16
6h	ACA1_322550/clean/scf7180000084466:13257-13355:+	ACA1_322550	UDENN FLCN/SMCR8-type domain-containing protein [Source:UniProtKB/TrEMBL;Acc:L8HDJ4]	0.001862197	0.386852	0.384989803	4.10E-10
6h	ACA1_106270/clean/scf7180000084660:67052-67126:-	ACA1_106270	ZT_dimer domain-containing protein [Source:UniProtKB/TrEMBL;Acc:L8GQ80]	0.049322833	0.434235667	0.384912833	1.89E-06
6h	ACA1_312180/clean/scf7180000083781:14136-14240:-	ACA1_312180	Fn3_like domain-containing protein [Source:UniProtKB/TrEMBL;Acc:L8GRI1]	0.004521851	0.389363667	0.384841815	5.53E-17
6h	ACA1_091560/clean/scf7180000084645:149958-150040:+	ACA1_091560	UBP-type domain-containing protein [Source:UniProtKB/TrEMBL;Acc:L8GKE3]	0	0.38481	0.38481	1.12E-06
6h	ACA1_108220/clean/scf7180000084661:17705-17793:+	ACA1_108220	hypothetical protein	0.010416667	0.395188667	0.384772	1.80E-05
6h	ACA1_292880/clean/scf7180000084777:924787-924890:-	ACA1_292880	RhoGEF domain containing protein [Source:UniProtKB/TrEMBL;Acc:L8HJL4]	0.001582193	0.386314	0.384731807	1.46E-07
6h	ACA1_264730/clean/scf7180000084770:368538-368614:-	ACA1_264730	hypothetical protein	0.0384162	0.423092333	0.384676133	6.84E-05
6h	ACA1_365090/clean/scf7180000084641:235057-235120:+	ACA1_365090	ADPribosylglycohydrolase superfamily protein [Source:UniProtKB/TrEMBL;Acc:L8GP97]	0.045990867	0.430635	0.384644133	1.80E-09
6h	ACA1_275430/clean/scf7180000084774:16807-16903:-	ACA1_275430	unspecified product	0.007248731	0.391823333	0.384574602	4.22E-08
6h	ACA1_034040/clean/scf7180000084561:9926-9992:+	ACA1_034040	Ankyrin repeat-containing protein [Source:UniProtKB/TrEMBL;Acc:L8GWJ4]	0.004385967	0.388960333	0.384574367	2.41E-07
6h	ACA1_116230/clean/scf7180000084669:731451-731542:+	ACA1_116230	Protein kinase domain containing protein [Source:UniProtKB/TrEMBL;Acc:L8H4M9]	0	0.384534	0.384534	1.53E-07
6h	ACA1_138450/clean/scf7180000084703:142002-142149:-	ACA1_138450	S1/P1 Nuclease [Source:UniProtKB/TrEMBL;Acc:L8H0X4]	0.000682672	0.385167333	0.384484662	2.31E-10
6h	ACA1_320420/clean/scf7180000084352:15910-15979:-	ACA1_320420	hypothetical protein	0.003584233	0.388016333	0.3844321	1.04E-07
6h	ACA1_165280/clean/scf7180000084732:26737-26871:-	ACA1_165280	PH domain containing protein [Source:UniProtKB/TrEMBL;Acc:L8HI11]	0.005291	0.389677667	0.384386667	2.02E-05
6h	ACA1_260050/clean/scf7180000084769:412181-412270:+	ACA1_260050	WD_REPEATS_REGION domain-containing protein [Source:UniProtKB/TrEMBL;Acc:L8GHJ4]	0.003943087	0.388326667	0.38438358	6.37E-20
6h	ACA1_329850/clean/scf7180000084566:11472-11581:-	ACA1_329850	hypothetical protein	0.014081853	0.398346333	0.38426448	4.48E-06
6h	ACA1_015510/clean/scf7180000083799:8363-8443:-	ACA1_015510	Replication factora protein 1 (Rpa1) subfamily protein [Source:UniProtKB/TrEMBL;Acc:L8GZQ4]	0.053256133	0.437493667	0.384237533	3.25E-08
6h	ACA1_184780/clean/scf7180000084746:61444-61560:+	ACA1_184780	Protein YIPF [Source:UniProtKB/TrEMBL;Acc:L8H566]	0.00452098	0.388727667	0.384206687	8.41E-12
6h	ACA1_096590/clean/scf7180000084645:1054831-1054920:-	ACA1_096590	Usp domain-containing protein [Source:UniProtKB/TrEMBL;Acc:L8GJ01]	0.362155333	0.74633	0.384174667	0.000264795
6h	ACA1_097990/clean/scf7180000084645:1368246-1368345:-	ACA1_097990	Protein kinase [Source:UniProtKB/TrEMBL;Acc:L8GJD1]	0.054146567	0.438255	0.384108433	1.97E-07
6h	ACA1_201510/clean/scf7180000084753:577999-578090:+	ACA1_201510	Ubiquitin fusion degradation protein, putative [Source:UniProtKB/TrEMBL;Acc:L8H4X5]	0.007641372	0.391713667	0.384072294	1.88E-07
6h	ACA1_085170/clean/scf7180000084632:63406-63495:+	ACA1_085170	TPR_REGION domain-containing protein [Source:UniProtKB/TrEMBL;Acc:L8HM05]	0	0.383969333	0.383969333	7.16E-12
6h	ACA1_072950/clean/scf7180000084603:1344302-1344395:+	ACA1_072950	Peptidase M16 inactive domain containing protein [Source:UniProtKB/TrEMBL;Acc:L8HGW3]	0	0.383945667	0.383945667	1.62E-06
6h	ACA1_400210/clean/scf7180000084775:120658-120909:-	ACA1_400210	Non-specific serine/threonine protein kinase [Source:UniProtKB/TrEMBL;Acc:L8GIE6]	0.005587397	0.389478333	0.383890937	5.51E-10
6h	ACA1_253320/clean/scf7180000084768:272650-272717:+	ACA1_253320	hypothetical protein	0	0.383734667	0.383734667	4.45E-08
6h	ACA1_143940/clean/scf7180000084709:109794-109910:-	ACA1_143940	VWFA domain-containing protein [Source:UniProtKB/TrEMBL;Acc:L8HEQ6]	0.029367603	0.413017333	0.38364973	5.39E-10
6h	ACA1_073050/clean/scf7180000084603:1362378-1362450:+	ACA1_073050	Anti-silencing protein, ASF1 family protein [Source:UniProtKB/TrEMBL;Acc:L8HGX1]	0.00275482	0.386384	0.38362918	3.34E-08
6h	ACA1_193130/clean/scf7180000084749:193343-193451:-	ACA1_193130	Metallophos domain-containing protein [Source:UniProtKB/TrEMBL;Acc:L8GN68]	0.003663	0.387066	0.383403	5.08E-08
6h	ACA1_191150/clean/scf7180000084748:187164-187231:+	ACA1_191150	Triosephosphate isomerase [Source:UniProtKB/TrEMBL;Acc:L8GE90]	0.004587598	0.387928667	0.383341068	2.63E-09
6h	ACA1_208260/clean/scf7180000084757:147033-147092:-	ACA1_208260	Acyl-coenzyme A oxidase [Source:UniProtKB/TrEMBL;Acc:L8GZJ4]	0.000202398	0.383526333	0.383323935	3.56E-09
6h	ACA1_015560/clean/scf7180000083799:20616-20698:+	ACA1_015560	hypothetical protein	0.003396603	0.386689667	0.383293064	4.97E-11
6h	ACA1_384530/clean/scf7180000084730:41638-41784:+	ACA1_384530	hypothetical protein	0	0.383291333	0.383291333	2.06E-09
6h	ACA1_287870/clean/scf7180000084777:112568-112652:+	ACA1_287870	Domain found in dishevelled, egl10, and pleckstrin domain containing protein [Source:UniProtKB/TrEMBL;Acc:L8HL57]	0.007246367	0.390453	0.383206633	1.12E-06
6h	ACA1_321760/clean/scf7180000084455:14116-14197:-	ACA1_321760	Haloacid dehalogenaselike hydrolase domain containing protein [Source:UniProtKB/TrEMBL;Acc:L8GRZ2]	0.110120333	0.493304	0.383183667	5.87E-05
6h	ACA1_377500/clean/scf7180000084711:75890-75963:+	ACA1_377500	Peptidase family c78 protein [Source:UniProtKB/TrEMBL;Acc:L8GU78]	0.000628829	0.383632667	0.383003838	8.29E-10
6h	ACA1_072660/clean/scf7180000084603:1246556-1246616:+	ACA1_072660	hypothetical protein	0	0.383002333	0.383002333	0.000653651
6h	ACA1_064810/clean/scf7180000084599:1703861-1703981:-	ACA1_064810	Transporter, major facilitator subfamily protein [Source:UniProtKB/TrEMBL;Acc:L8GWU0]	0.0047359	0.387560667	0.382824767	8.47E-06
6h	ACA1_368770/clean/scf7180000084664:157495-157572:-	ACA1_368770	Glyco_transf_20 domain-containing protein [Source:UniProtKB/TrEMBL;Acc:L8GYU8]	0.1235422	0.506339	0.3827968	1.41E-05
6h	ACA1_361100/clean/scf7180000084622:242045-242142:-	ACA1_361100	TLDc domain-containing protein [Source:UniProtKB/TrEMBL;Acc:L8HE93]	0.008658	0.391313333	0.382655333	1.81E-07
6h	ACA1_167020/clean/scf7180000084736:56265-56367:+	ACA1_167020	EF-hand domain-containing protein [Source:UniProtKB/TrEMBL;Acc:L8H0U0]	0.002525253	0.385076333	0.38255108	4.70E-09
6h	ACA1_264360/clean/scf7180000084770:249483-249542:+	ACA1_264360	hypothetical protein	0.005111933	0.387636	0.382524067	2.90E-06
6h	ACA1_188320/clean/scf7180000084747:156308-156413:-	ACA1_188320	AcylCoA-binding domain containing protein [Source:UniProtKB/TrEMBL;Acc:L8GRT2]	0.003979337	0.386471	0.382491663	7.18E-08
6h	ACA1_152970/clean/scf7180000084720:43273-43393:-	ACA1_152970	SET family protein [Source:UniProtKB/TrEMBL;Acc:L8HHE2]	0.000682466	0.383171333	0.382488868	5.73E-12
6h	ACA1_009910/clean/scf7180000083468:6058-6159:+	ACA1_009910	tRNA-synt_1c domain-containing protein [Source:UniProtKB/TrEMBL;Acc:L8GDZ9]	0.006339437	0.388661667	0.38232223	8.35E-07
6h	ACA1_170960/clean/scf7180000084741:25616-25727:+	ACA1_170960	hypothetical protein	0.017908533	0.400167	0.382258467	1.70E-09
6h	ACA1_370300/clean/scf7180000084664:491066-491172:-	ACA1_370300	hypothetical protein	0	0.382198	0.382198	1.18E-12
6h	ACA1_072940/clean/scf7180000084603:1338242-1338337:+	ACA1_072940	Protein kinase domain-containing protein [Source:UniProtKB/TrEMBL;Acc:L8HET0]	0.00189231	0.384081	0.38218869	1.13E-08
6h	ACA1_025540/clean/scf7180000084448:7399-7495:+	ACA1_025540	hypothetical protein	0.009983944	0.392087	0.382103056	7.76E-09
6h	ACA1_144070/clean/scf7180000084709:142464-142609:-	ACA1_144070	Methyltrans_SAM domain-containing protein [Source:UniProtKB/TrEMBL;Acc:L8HHX9]	0.012941773	0.394998333	0.38205656	3.98E-08
6h	ACA1_076860/clean/scf7180000084612:168311-168409:+	ACA1_076860	RNA binding motif containing protein [Source:UniProtKB/TrEMBL;Acc:L8GM55]	0.003422865	0.385466333	0.382043468	1.25E-10
6h	ACA1_261300/clean/scf7180000084769:631073-631163:-	ACA1_261300	Fbox domain containing protein [Source:UniProtKB/TrEMBL;Acc:L8GF57]	0.011620097	0.393650333	0.382030237	2.10E-06
6h	ACA1_065930/clean/scf7180000084599:1953599-1953672:-	ACA1_065930	Cytochrome p450 superfamily protein [Source:UniProtKB/TrEMBL;Acc:L8GXV1]	0.002533357	0.384553333	0.382019977	5.06E-07
6h	ACA1_326860/clean/scf7180000084510:57047-57141:-	ACA1_326860	Lipase_3 domain-containing protein [Source:UniProtKB/TrEMBL;Acc:L8HKH4]	0.003436433	0.385450667	0.382014233	2.63E-06
6h	ACA1_290140/clean/scf7180000084777:541328-541418:-	ACA1_290140	Rap-GAP domain-containing protein [Source:UniProtKB/TrEMBL;Acc:L8HJ83]	0	0.381956	0.381956	3.64E-06
6h	ACA1_116020/clean/scf7180000084669:707018-707120:+	ACA1_116020	Rho-GAP domain-containing protein [Source:UniProtKB/TrEMBL;Acc:L8H6T0]	0.010013513	0.391909	0.381895487	2.20E-13
6h	ACA1_264630/clean/scf7180000084770:341467-341560:+	ACA1_264630	hypothetical protein	0.004011737	0.385828333	0.381816597	2.33E-17
6h	ACA1_153680/clean/scf7180000084720:153721-153806:-	ACA1_153680	Amz2 protein [Source:UniProtKB/TrEMBL;Acc:L8HHH0]	0.0284553	0.410228333	0.381773033	0.003078079
6h	ACA1_112570/clean/scf7180000084669:187021-187117:-	ACA1_112570	Protein kinase domain-containing protein [Source:UniProtKB/TrEMBL;Acc:L8H5F2]	0.004067967	0.385760333	0.381692367	8.19E-07
6h	ACA1_362780/clean/scf7180000084624:177242-177331:+	ACA1_362780	Cofilin/tropomyosin-type actin-binding protein [Source:UniProtKB/TrEMBL;Acc:L8GHX4]	0.007017763	0.388564	0.381546237	4.94E-13
6h	ACA1_230510/clean/scf7180000084761:367350-367433:-	ACA1_230510	hypothetical protein	0.021382307	0.402884	0.381501693	2.21E-07
6h	ACA1_117070/clean/scf7180000084669:906917-906980:-	ACA1_117070	Rap-GAP domain-containing protein [Source:UniProtKB/TrEMBL;Acc:L8H3Z5]	0.0037037	0.385141333	0.381437633	2.52E-07
6h	ACA1_355980/clean/scf7180000084728:41075-41175:+	ACA1_355980	Aa_trans domain-containing protein [Source:UniProtKB/TrEMBL;Acc:L8H7L7]	0.038722867	0.419998667	0.3812758	1.68E-08
6h	ACA1_162370/clean/scf7180000084726:43032-43115:-	ACA1_162370	Protein kinase domain-containing protein [Source:UniProtKB/TrEMBL;Acc:L8GYN7]	0.01353856	0.394559	0.38102044	7.37E-14
6h	ACA1_219750/clean/scf7180000084758:800634-800781:+	ACA1_219750	FAD_binding_3 domain-containing protein [Source:UniProtKB/TrEMBL;Acc:L8GPZ6]	0.015873	0.396853333	0.380980333	1.63E-07
6h	ACA1_077080/clean/scf7180000084612:228144-228225:-	ACA1_077080	Ras-GAP domain-containing protein [Source:UniProtKB/TrEMBL;Acc:L8GLZ3]	0.016526637	0.397415667	0.38088903	1.06E-05
6h	ACA1_255530/clean/scf7180000084768:534584-534689:-	ACA1_255530	Carrier superfamily protein [Source:UniProtKB/TrEMBL;Acc:L8HB08]	0.009987923	0.390791	0.380803077	1.16E-06
6h	ACA1_220050/clean/scf7180000084758:849404-849542:+	ACA1_220050	Calponin domain containing protein [Source:UniProtKB/TrEMBL;Acc:L8GRJ6]	0.001087783	0.381890333	0.380802551	9.15E-14
6h	ACA1_175910/clean/scf7180000084741:1086924-1087016:+	ACA1_175910	Eukaryotic translation elongation factor 2, like, putative [Source:UniProtKB/TrEMBL;Acc:L8HH63]	0.0839101	0.464698667	0.380788567	1.80E-05
6h	ACA1_072790/clean/scf7180000084603:1297669-1297749:-	ACA1_072790	AB hydrolase-1 domain-containing protein [Source:UniProtKB/TrEMBL;Acc:L8HGV1]	0.004694833	0.385357667	0.380662833	2.89E-06
6h	ACA1_173170/clean/scf7180000084741:418037-418176:-	ACA1_173170	SNF2 family Nterminal domain containing protein [Source:UniProtKB/TrEMBL;Acc:L8HGM5]	0.005208333	0.385794	0.380585667	1.66E-06
6h	ACA1_174880/clean/scf7180000084741:820843-820915:-	ACA1_174880	Cation transport domain containing protein [Source:UniProtKB/TrEMBL;Acc:L8HI03]	0.030303033	0.410869667	0.380566633	0.000366403
6h	ACA1_027390/clean/scf7180000084465:11019-11106:-	ACA1_027390	hypothetical protein	0.016639103	0.397164333	0.38052523	8.23E-15
6h	ACA1_264550/clean/scf7180000084770:318940-319033:+	ACA1_264550	Cys/Met metabolism PLPdependent enzyme superfamily protein [Source:UniProtKB/TrEMBL;Acc:L8H496]	0	0.380479	0.380479	4.97E-11
6h	ACA1_266230/clean/scf7180000084770:637607-637702:+	ACA1_266230	Dilute domain-containing protein [Source:UniProtKB/TrEMBL;Acc:L8H2D0]	0	0.380370667	0.380370667	0.000456394
6h	ACA1_054180/clean/scf7180000084594:420012-420095:+	ACA1_054180	hypothetical protein	0.02732852	0.407632667	0.380304147	5.11E-11
6h	ACA1_255530/clean/scf7180000084768:534405-534497:-	ACA1_255530	Carrier superfamily protein [Source:UniProtKB/TrEMBL;Acc:L8HB08]	0	0.380245	0.380245	5.85E-08
6h	ACA1_038430/clean/scf7180000084568:147368-147430:+	ACA1_038430	hypothetical protein	0.00114798	0.381323333	0.380175354	4.42E-11
6h	ACA1_182310/clean/scf7180000084745:180934-181011:-	ACA1_182310	Adenylosuccinate lyase [Source:UniProtKB/TrEMBL;Acc:L8H721]	0.047743567	0.427849667	0.3801061	3.68E-09
6h	ACA1_210520/clean/scf7180000084758:44074-44214:+	ACA1_210520	SANT domain-containing protein [Source:UniProtKB/TrEMBL;Acc:L8GPP0]	0.028664167	0.408597333	0.379933167	6.58E-08
6h	ACA1_038180/clean/scf7180000084568:90497-90571:+	ACA1_038180	FHA domain containing protein [Source:UniProtKB/TrEMBL;Acc:L8GLQ3]	0.038878267	0.418761667	0.3798834	1.24E-07
6h	ACA1_369480/clean/scf7180000084664:310357-310435:-	ACA1_369480	SMP-LTD domain-containing protein [Source:UniProtKB/TrEMBL;Acc:L8GYZ6]	0.021921267	0.401781	0.379859733	2.26E-11
6h	ACA1_064240/clean/scf7180000084599:1565362-1565431:+	ACA1_064240	NAD(P)-bd_dom domain-containing protein [Source:UniProtKB/TrEMBL;Acc:L8GXD4]	0.000911073	0.380738	0.379826927	2.49E-11
6h	ACA1_240360/clean/scf7180000084763:227124-227204:+	ACA1_240360	hypothetical protein	0.166666667	0.546433667	0.379767	0.03387161
6h	ACA1_066580/clean/scf7180000084600:18563-18633:+	ACA1_066580	E3 ubiquitin-protein ligase [Source:UniProtKB/TrEMBL;Acc:L8GP23]	0.000396353	0.380156333	0.37975998	1.51E-08
6h	ACA1_096400/clean/scf7180000084645:1005516-1005574:+	ACA1_096400	Methyltransf_11 domain-containing protein [Source:UniProtKB/TrEMBL;Acc:L8GJF1]	0.007404	0.387104667	0.379700667	1.91E-10
6h	ACA1_097590/clean/scf7180000084645:1291900-1291982:-	ACA1_097590	Amine oxidase [Source:UniProtKB/TrEMBL;Acc:L8GK33]	0.001514692	0.380852	0.379337308	7.11E-24
6h	ACA1_277460/clean/scf7180000084776:53692-53774:+	ACA1_277460	Ipi1_N domain-containing protein [Source:UniProtKB/TrEMBL;Acc:L8H6F9]	0	0.379333	0.379333	5.05E-05
6h	ACA1_083640/clean/scf7180000084629:7975-8105:-	ACA1_083640	Elongation factor 1alpha [Source:UniProtKB/TrEMBL;Acc:L8GYH2]	0.00097431	0.380257667	0.379283357	5.87E-11
6h	ACA1_156700/clean/scf7180000084723:55893-55976:+	ACA1_156700	Proteintyrosine phosphatase [Source:UniProtKB/TrEMBL;Acc:L8GIF7]	0.003123767	0.382334333	0.379210567	9.37E-09
6h	ACA1_323370/clean/scf7180000084468:12393-12455:-	ACA1_323370	SHSP domain-containing protein [Source:UniProtKB/TrEMBL;Acc:L8H320]	0.047619	0.426756	0.379137	0.007051236
6h	ACA1_260950/clean/scf7180000084769:559980-560080:-	ACA1_260950	Ribonuclease 25A protein [Source:UniProtKB/TrEMBL;Acc:L8GG37]	0.0360226	0.415158667	0.379136067	1.45E-08
6h	ACA1_289610/clean/scf7180000084777:431001-431070:+	ACA1_289610	Copper transporter [Source:UniProtKB/TrEMBL;Acc:L8HKL4]	0.017365647	0.396365667	0.37900002	1.07E-21
6h	ACA1_136780/clean/scf7180000084703:6818-6911:+	ACA1_136780	hypothetical protein	0.01972178	0.39865	0.37892822	3.62E-11
6h	ACA1_087960/clean/scf7180000084639:142648-142721:+	ACA1_087960	Rho-GAP domain-containing protein [Source:UniProtKB/TrEMBL;Acc:L8GX18]	0.003859833	0.382778	0.378918167	1.29E-09
6h	ACA1_252490/clean/scf7180000084768:161575-161673:-	ACA1_252490	Protein kinase domain-containing protein [Source:UniProtKB/TrEMBL;Acc:L8H9V3]	0.012933133	0.391818	0.378884867	1.97E-08
6h	ACA1_289060/clean/scf7180000084777:346414-346485:+	ACA1_289060	hypothetical protein	0.001568158	0.380341333	0.378773175	2.84E-13
6h	ACA1_264390/clean/scf7180000084770:257107-257204:+	ACA1_264390	GOLD domain-containing protein [Source:UniProtKB/TrEMBL;Acc:L8H484]	0	0.378697	0.378697	4.60E-12
6h	ACA1_248540/clean/scf7180000084766:73235-73409:+	ACA1_248540	Dynein light chain tctextype, putative [Source:UniProtKB/TrEMBL;Acc:L8GXW2]	0.0208958	0.39957	0.3786742	8.68E-12
6h	ACA1_199610/clean/scf7180000084753:273580-273677:+	ACA1_199610	hypothetical protein	0.013418867	0.392083667	0.3786648	4.19E-11
6h	ACA1_175590/clean/scf7180000084741:1009517-1009646:+	ACA1_175590	hypothetical protein	0.001655826	0.380265	0.378609174	2.38E-08
6h	ACA1_373590/clean/scf7180000084679:109405-109488:-	ACA1_373590	hypothetical protein	0.098464	0.47704	0.378576	0.000135302
6h	ACA1_164760/clean/scf7180000084729:267583-267653:+	ACA1_164760	hypothetical protein	0.003512963	0.382063667	0.378550703	4.68E-08
6h	ACA1_151320/clean/scf7180000084716:102512-102607:-	ACA1_151320	PPM-type phosphatase domain-containing protein [Source:UniProtKB/TrEMBL;Acc:L8GZX5]	0.00013611	0.378515333	0.378379223	9.22E-11
6h	ACA1_376720/clean/scf7180000084685:145825-145947:-	ACA1_376720	LIM zinc-binding domain-containing protein [Source:UniProtKB/TrEMBL;Acc:L8HHL9]	0.001875339	0.380238	0.378362661	1.57E-18
6h	ACA1_314970/clean/scf7180000083887:4665-4766:+	ACA1_314970	USP domain-containing protein [Source:UniProtKB/TrEMBL;Acc:L8H239]	0.0307507	0.409034333	0.378283633	6.73E-09
6h	ACA1_119230/clean/scf7180000084672:48362-48431:+	ACA1_119230	Armadillo/betacatenin-like repeat domain containing protein [Source:UniProtKB/TrEMBL;Acc:L8HMP1]	0.014823303	0.393076667	0.378253363	9.31E-17
6h	ACA1_185310/clean/scf7180000084746:89539-89628:-	ACA1_185310	Serine/threonine protein kinase [Source:UniProtKB/TrEMBL;Acc:L8H6G0]	0.002055664	0.380262333	0.37820667	1.37E-08
6h	ACA1_156040/clean/scf7180000084723:11662-11733:+	ACA1_156040	Fungal Zn(2)-Cys(6) binuclear cluster domain containing protein [Source:UniProtKB/TrEMBL;Acc:L8GHR8]	0	0.378041333	0.378041333	1.31E-06
6h	ACA1_264920/clean/scf7180000084770:406613-406698:-	ACA1_264920	Protein kinase domain-containing protein [Source:UniProtKB/TrEMBL;Acc:L8H3L2]	0	0.378035	0.378035	2.46E-06
6h	ACA1_199000/clean/scf7180000084753:189542-189633:-	ACA1_199000	hypothetical protein	0.003390017	0.381413333	0.378023317	4.08E-09
6h	ACA1_326170/clean/scf7180000084504:46457-46581:+	ACA1_326170	SAM domain-containing protein [Source:UniProtKB/TrEMBL;Acc:L8HIK1]	0.036792967	0.41468	0.377887033	5.34E-08
6h	ACA1_054090/clean/scf7180000084594:399508-399597:-	ACA1_054090	Fbox domain containing protein [Source:UniProtKB/TrEMBL;Acc:L8H592]	0.011494267	0.389315	0.377820733	1.26E-05
6h	ACA1_085240/clean/scf7180000084632:80721-80817:-	ACA1_085240	hypothetical protein	0.009222127	0.38704	0.377817873	4.24E-06
6h	ACA1_164780/clean/scf7180000084729:283386-283464:+	ACA1_164780	Ubiquitin domain containing protein [Source:UniProtKB/TrEMBL;Acc:L8GRS0]	0.004270163	0.382044667	0.377774503	1.18E-08
6h	ACA1_096770/clean/scf7180000084645:1098784-1098873:+	ACA1_096770	NIF3 family protein [Source:UniProtKB/TrEMBL;Acc:L8GIV2]	0.012136767	0.389777333	0.377640567	4.29E-08
6h	ACA1_321930/clean/scf7180000084457:17621-17707:+	ACA1_321930	Transporter, major intrinsic protein (MIP) superfamily protein [Source:UniProtKB/TrEMBL;Acc:L8HCR2]	0.006535933	0.384148667	0.377612733	9.65E-07
6h	ACA1_236340/clean/scf7180000084762:441442-441554:-	ACA1_236340	hypothetical protein	0.007456623	0.385059	0.377602377	4.42E-27

6h	ACA1_159090/clean/scf7180000084724:338093-338163:-	ACA1_159090	hypothetical protein	0.003875967	0.381345667	0.3774697	7.91E-08
6h	ACA1_351920/clean/scf7180000084695:34008-34082:+	ACA1_351920	VWFA domain-containing protein [Source:UniProtKB/TrEMBL;Acc:L8GE58]	0.01550826	0.392906333	0.377398073	2.17E-15
6h	ACA1_234310/clean/scf7180000084762:269591-269698:-	ACA1_234310	RRM domain-containing protein [Source:UniProtKB/TrEMBL;Acc:L8H2M7]	0.047204067	0.424562	0.377357933	2.46E-09
6h	ACA1_188710/clean/scf7180000084747:195856-195965:+	ACA1_188710	SNARE associated Golgi protein [Source:UniProtKB/TrEMBL;Acc:L8GTI0]	0.0190996	0.396418333	0.377318733	5.17E-16
6h	ACA1_252460/clean/scf7180000084768:155684-155783:-	ACA1_252460	Isovaleryl Coenzyme A dehydrogenase [Source:UniProtKB/TrEMBL;Acc:L8HD26]	0.002057613	0.379317333	0.37725972	5.81E-10
6h	ACA1_264650/clean/scf7180000084770:350893-350972:+	ACA1_264650	WD domain, G-beta repeat-containing protein [Source:UniProtKB/TrEMBL;Acc:L8H4A3]	0.006410267	0.383629667	0.3772194	9.50E-07
6h	ACA1_395500/clean/scf7180000084756:194703-194776:-	ACA1_395500	Rho-GAP domain-containing protein [Source:UniProtKB/TrEMBL;Acc:L8GZP8]	0.01764481	0.394825333	0.377180523	2.38E-09
6h	ACA1_167020/clean/scf7180000084736:57078-57146:+	ACA1_167020	EF-hand domain-containing protein [Source:UniProtKB/TrEMBL;Acc:L8H0U0]	0.004604167	0.381664	0.377059833	1.22E-08
6h	ACA1_070160/clean/scf7180000084603:631427-631622:+	ACA1_070160	Tetratricopeptide repeat-containing protein [Source:UniProtKB/TrEMBL;Acc:L8HDZ7]	0.022797133	0.399796	0.376998867	0.000103667
6h	ACA1_080550/clean/scf7180000084618:32715-32818:+	ACA1_080550	FAD binding domain containing protein [Source:UniProtKB/TrEMBL;Acc:L8HBU6]	0.2099827	0.586957667	0.376974967	5.15E-06
6h	ACA1_045640/clean/scf7180000084576:167658-167776:+	ACA1_045640	hypothetical protein	0.003387513	0.380157667	0.376770153	1.65E-08
6h	ACA1_269270/clean/scf7180000084770:962462-962615:+	ACA1_269270	Protein phosphatase [Source:UniProtKB/TrEMBL;Acc:L8H4X6]	0.023620417	0.400339	0.376718583	1.66E-07
6h	ACA1_182300/clean/scf7180000084745:176763-176837:+	ACA1_182300	hypothetical protein	0.0120482	0.388766	0.3767178	5.55E-08
6h	ACA1_040690/clean/scf7180000084572:48865-49027:+	ACA1_040690	Multisensor hybrid histidine kinase [Source:UniProtKB/TrEMBL;Acc:L8H1W6]	0.012696943	0.389410667	0.376713723	4.36E-06
6h	ACA1_014490/clean/scf7180000083768:3052-3132:+	ACA1_014490	Protein kinase domain-containing protein [Source:UniProtKB/TrEMBL;Acc:L8GY11]	0.003921567	0.380634	0.376712433	1.39E-07
6h	ACA1_381590/clean/scf7180000084712:187872-187942:+	ACA1_381590	PI3K/PI4K domain-containing protein [Source:UniProtKB/TrEMBL;Acc:L8GN80]	0	0.376670667	0.376670667	8.54E-09
6h	ACA1_288670/clean/scf7180000084777:257118-257191:-	ACA1_288670	Psf1 family protein [Source:UniProtKB/TrEMBL;Acc:L8HLA2]	0.034643233	0.411258333	0.3766151	0.000235906
6h	ACA1_184270/clean/scf7180000084745:598197-598275:+	ACA1_184270	Ankyrin repeatcontaining protein [Source:UniProtKB/TrEMBL;Acc:L8HAN7]	0.006944433	0.383532333	0.3765879	1.58E-06
6h	ACA1_276410/clean/scf7180000084774:167196-167304:+	ACA1_276410	Phosphate transporter [Source:UniProtKB/TrEMBL;Acc:L8GTH7]	0	0.376584	0.376584	1.35E-08
6h	ACA1_064240/clean/scf7180000084599:1567024-1567106:+	ACA1_064240	NAD(P)-bd_dom domain-containing protein [Source:UniProtKB/TrEMBL;Acc:L8GXD4]	0.011161317	0.387617667	0.37645635	6.64E-19
6h	ACA1_063750/clean/scf7180000084599:1495816-1495887:+	ACA1_063750	TauD domain-containing protein [Source:UniProtKB/TrEMBL;Acc:L8GZU6]	0.148726467	0.525179	0.376452533	0.005065439
6h	ACA1_300890/clean/scf7180000083147:17144-17221:-	ACA1_300890	hypothetical protein	0.001503575	0.377952333	0.376448759	5.02E-11
6h	ACA1_063290/clean/scf7180000084599:1372534-1372628:-	ACA1_063290	hypothetical protein	0	0.376440667	0.376440667	2.10E-06
6h	ACA1_196200/clean/scf7180000084752:53301-53402:+	ACA1_196200	Endonuclease V protein, putative [Source:UniProtKB/TrEMBL;Acc:L8HGA4]	0.011641327	0.388081333	0.376440007	1.53E-05
6h	ACA1_067770/clean/scf7180000084603:12696-12777:+	ACA1_067770	CBS domain containing protein [Source:UniProtKB/TrEMBL;Acc:L8HEJ6]	0.045474867	0.421870667	0.3763958	8.52E-13
6h	ACA1_038430/clean/scf7180000084568:146759-146853:+	ACA1_038430	hypothetical protein	0.00132802	0.377639667	0.376311647	1.11E-10
6h	ACA1_101330/clean/scf7180000084649:46882-46979:+	ACA1_101330	t-SNARE coiled-coil homology domain-containing protein [Source:UniProtKB/TrEMBL;Acc:L8GGH6]	0.004721433	0.380967667	0.376246233	4.21E-06
6h	ACA1_157220/clean/scf7180000084724:11626-11717:+	ACA1_157220	Pro-kuma_activ domain-containing protein [Source:UniProtKB/TrEMBL;Acc:L8H9Y5]	0.004341733	0.380446667	0.376104933	3.53E-06
6h	ACA1_096470/clean/scf7180000084645:1022003-1022094:-	ACA1_096470	Dual specificity phosphatase, catalytic domain containing protein [Source:UniProtKB/TrEMBL;Acc:L8GL97]	0	0.376090333	0.376090333	1.33E-06
6h	ACA1_236940/clean/scf7180000084762:486531-486645:-	ACA1_236940	hypothetical protein	0.01361896	0.389705667	0.376086707	2.38E-10
6h	ACA1_208500/clean/scf7180000084757:210829-210928:+	ACA1_208500	Aldedh domain-containing protein [Source:UniProtKB/TrEMBL;Acc:L8GYD7]	0.01975735	0.395821	0.37606365	3.62E-10
6h	ACA1_174110/clean/scf7180000084741:614529-614649:+	ACA1_174110	hypothetical protein	0.009528527	0.385569667	0.37604114	3.00E-17
6h	ACA1_369100/clean/scf7180000084664:244699-244775:+	ACA1_369100	IPPc domain-containing protein [Source:UniProtKB/TrEMBL;Acc:L8H092]	0.012137933	0.388082	0.375944067	2.24E-05
6h	ACA1_254440/clean/scf7180000084768:392335-392466:+	ACA1_254440	Protein kinase domain-containing protein [Source:UniProtKB/TrEMBL;Acc:L8HAW2]	0.009887333	0.385789667	0.375902333	2.19E-05
6h	ACA1_206370/clean/scf7180000084757:17397-17471:+	ACA1_206370	MOSC domain-containing protein [Source:UniProtKB/TrEMBL;Acc:L8GY21]	0.00646356	0.382331667	0.375868107	7.93E-10
6h	ACA1_097790/clean/scf7180000084645:1340064-1340125:-	ACA1_097790	DUF4209 domain-containing protein [Source:UniProtKB/TrEMBL;Acc:L8GJC1]	0.013333333	0.389139	0.375805667	0.000218524
6h	ACA1_059530/clean/scf7180000084599:461942-462117:-	ACA1_059530	hypothetical protein	0.005103337	0.380906333	0.375802997	3.83E-09
6h	ACA1_058840/clean/scf7180000084599:392653-392745:-	ACA1_058840	HATPase_c domain-containing protein [Source:UniProtKB/TrEMBL;Acc:L8GXB7]	0.001241463	0.37695	0.375708537	3.56E-13
6h	ACA1_266510/clean/scf7180000084770:684373-684448:-	ACA1_266510	RhoGEF domain containing protein [Source:UniProtKB/TrEMBL;Acc:L8H2E9]	0.0443689	0.420029667	0.375660767	1.59E-07
6h	ACA1_068350/clean/scf7180000084603:178101-178173:+	ACA1_068350	hypothetical protein	0.003073406	0.378697	0.375623594	1.98E-14
6h	ACA1_174170/clean/scf7180000084741:632983-633100:-	ACA1_174170	Glutathione Stransferase, C-terminal domain containing protein [Source:UniProtKB/TrEMBL;Acc:L8HHR9]	0.00296823	0.378581333	0.375613103	1.66E-34
6h	ACA1_113860/clean/scf7180000084669:390316-390439:+	ACA1_113860	Saposin B-type domain-containing protein [Source:UniProtKB/TrEMBL;Acc:L8H4D7]	0.0022291	0.377795	0.3755659	1.11E-12
6h	ACA1_240070/clean/scf7180000084763:194838-194958:-	ACA1_240070	Carboxylic ester hydrolase [Source:UniProtKB/TrEMBL;Acc:L8GJU3]	0.01497774	0.390465333	0.375487593	5.58E-14
6h	ACA1_264590/clean/scf7180000084770:329262-329327:-	ACA1_264590	Saposin B-type domain-containing protein [Source:UniProtKB/TrEMBL;Acc:L8H216]	0.01937418	0.394856667	0.375482487	4.53E-09
6h	ACA1_230570/clean/scf7180000084761:379563-379648:-	ACA1_230570	DUF4139 domain-containing protein [Source:UniProtKB/TrEMBL;Acc:L8H928]	0.007884667	0.383284	0.375399333	2.30E-11
6h	ACA1_221050/clean/scf7180000084758:986656-986755:-	ACA1_221050	hypothetical protein	0.035634033	0.410934	0.375299967	1.70E-06
6h	ACA1_183620/clean/scf7180000084745:465916-466084:-	ACA1_183620	Citrate synthase [Source:UniProtKB/TrEMBL;Acc:L8HAH1]	0.000585467	0.375574333	0.374988867	3.79E-16
6h	ACA1_109780/clean/scf7180000084663:25393-25562:-	ACA1_109780	AAA domain-containing protein [Source:UniProtKB/TrEMBL;Acc:L8HIF9]	0.004090003	0.379076667	0.374986663	1.57E-08
6h	ACA1_191480/clean/scf7180000084749:673-766:-	ACA1_191480	CS domain containing protein [Source:UniProtKB/TrEMBL;Acc:L8GMZ3]	0.00076805	0.375653333	0.374885283	4.51E-13
6h	ACA1_142570/clean/scf7180000084708:185727-185793:+	ACA1_142570	hypothetical protein	0.020947673	0.395743667	0.374795993	1.16E-08
6h	ACA1_109770/clean/scf7180000084663:21593-21769:+	ACA1_109770	WD40 repeat-containing protein [Source:UniProtKB/TrEMBL;Acc:L8HKT6]	0.005291	0.379531	0.37424	3.88E-09
6h	ACA1_363800/clean/scf7180000084641:20926-21105:-	ACA1_363800	ABC transporter domain-containing protein [Source:UniProtKB/TrEMBL;Acc:L8GM21]	0.074698333	0.448771667	0.374073333	7.61E-08
6h	ACA1_050360/clean/scf7180000084589:78236-78298:-	ACA1_050360	Metalloenzyme domain-containing protein [Source:UniProtKB/TrEMBL;Acc:L8H565]	0.002671977	0.376709333	0.374037357	7.12E-09
6h	ACA1_150560/clean/scf7180000084716:12630-12695:-	ACA1_150560	hypothetical protein	0.032855867	0.406885	0.374029133	3.73E-10
6h	ACA1_146580/clean/scf7180000084713:60298-60420:+	ACA1_146580	Thioredoxin-like_fold domain-containing protein [Source:UniProtKB/TrEMBL;Acc:L8GTM4]	0.00417649	0.378182333	0.374005843	6.83E-16
6h	ACA1_117130/clean/scf7180000084669:922596-922676:+	ACA1_117130	Eukaryotic initiation factor 4e, putative [Source:UniProtKB/TrEMBL;Acc:L8H4Z1]	0.00525956	0.379259333	0.373999773	2.06E-09
6h	ACA1_192060/clean/scf7180000084749:88909-89037:-	ACA1_192060	Initiation factor eif4 gamma, middle, putative [Source:UniProtKB/TrEMBL;Acc:L8GQR7]	0	0.37394	0.37394	1.33E-10
6h	ACA1_199630/clean/scf7180000084753:281181-281351:-	ACA1_199630	PRAI domain-containing protein [Source:UniProtKB/TrEMBL;Acc:L8H3A6]	0.000382065	0.374075667	0.373693601	2.73E-11
6h	ACA1_308240/clean/scf7180000083743:7891-7986:+	ACA1_308240	VPS13_C domain-containing protein [Source:UniProtKB/TrEMBL;Acc:L8HJD8]	0	0.37366	0.37366	7.12E-07
6h	ACA1_063500/clean/scf7180000084599:1432881-1432973:+	ACA1_063500	U-box domain-containing protein [Source:UniProtKB/TrEMBL;Acc:L8GZS8]	0	0.373655333	0.373655333	6.55E-10
6h	ACA1_256390/clean/scf7180000084769:21120-21181:+	ACA1_256390	Glutamine amidotransferase type-1 domain-containing protein [Source:UniProtKB/TrEMBL;Acc:L8GEH2]	0.005882367	0.379418	0.373535633	3.18E-09
6h	ACA1_390520/clean/scf7180000084740:55901-55966:+	ACA1_390520	EnoylCoA hydratase/isomerase family domain containing protein [Source:UniProtKB/TrEMBL;Acc:L8GPA6]	0	0.373480667	0.373480667	1.70E-11
6h	ACA1_111100/clean/scf7180000084666:33325-33426:+	ACA1_111100	hypothetical protein	0.00516192	0.378614667	0.373452747	1.31E-08
6h	ACA1_188190/clean/scf7180000084747:123733-123800:-	ACA1_188190	Calponin-homology (CH) domain-containing protein [Source:UniProtKB/TrEMBL;Acc:L8GSF5]	0.024530733	0.397846333	0.3733156	1.28E-21
6h	ACA1_338290/clean/scf7180000084604:52939-53041:-	ACA1_338290	hypothetical protein	0	0.373301667	0.373301667	7.69E-08
6h	ACA1_186210/clean/scf7180000084746:173443-173527:+	ACA1_186210	Tyrosine phosphataselike protein, putative [Source:UniProtKB/TrEMBL;Acc:L8H5A9]	0.003518633	0.376739667	0.373221033	5.85E-08
6h	ACA1_098210/clean/scf7180000084645:1430759-1430850:+	ACA1_098210	Peroxidase, putative [Source:UniProtKB/TrEMBL;Acc:L8GJY8]	0	0.373121	0.373121	1.00E-07
6h	ACA1_071170/clean/scf7180000084603:878925-879009:+	ACA1_071170	RhoGAP domain containing protein [Source:UniProtKB/TrEMBL;Acc:L8HGC8]	0	0.373069	0.373069	1.69E-07
6h	ACA1_163650/clean/scf7180000084729:64641-64736:+	ACA1_163650	hypothetical protein	0.020227046	0.393208667	0.372981621	6.28E-06
6h	ACA1_055580/clean/scf7180000084594:737411-737523:+	ACA1_055580	RasGEF domain containing protein [Source:UniProtKB/TrEMBL;Acc:L8H6C9]	0.109901567	0.482874667	0.3729731	0.000653056
6h	ACA1_359690/clean/scf7180000084622:444-714:-	ACA1_359690	Rab-GAP TBC domain-containing protein [Source:UniProtKB/TrEMBL;Acc:L8HBR2]	0.0373721	0.410325333	0.372953233	6.81E-07
6h	ACA1_031780/clean/scf7180000084523:1398-1474:-	ACA1_031780	Lactamase_B domain-containing protein [Source:UniProtKB/TrEMBL;Acc:L8H103]	0.000550057	0.373377667	0.37282761	3.14E-08
6h	ACA1_063460/clean/scf7180000084599:1414231-1414296:-	ACA1_063460	AAA domain-containing protein [Source:UniProtKB/TrEMBL;Acc:L8GXD1]	0.035643767	0.408352	0.372708233	2.84E-10
6h	ACA1_365010/clean/scf7180000084641:202533-202597:-	ACA1_365010	hypothetical protein	0.232929667	0.605478333	0.372548667	9.08E-05
6h	ACA1_140530/clean/scf7180000084707:81723-81859:-	ACA1_140530	F-box domain containing protein [Source:UniProtKB/TrEMBL;Acc:L8GGR1]	0.085517533	0.457969333	0.3724518	2.97E-05
6h	ACA1_309330/clean/scf7180000083758:5719-5781:-	ACA1_309330	hypothetical protein	0.040504067	0.412945	0.372440933	2.90E-06
6h	ACA1_151320/clean/scf7180000084716:100415-100522:-	ACA1_151320	PPM-type phosphatase domain-containing protein [Source:UniProtKB/TrEMBL;Acc:L8GZX5]	0.0452659	0.417649	0.3723831	1.99E-10
6h	ACA1_051820/clean/scf7180000084592:58958-59027:+	ACA1_051820	Ubiquitin-like domain-containing protein [Source:UniProtKB/TrEMBL;Acc:L8GP24]	0.004568643	0.376911333	0.37234269	1.44E-37
6h	ACA1_183690/clean/scf7180000084745:483620-483729:-	ACA1_183690	hypothetical protein	0.039013043	0.411309	0.372295957	0.002254567
6h	ACA1_192660/clean/scf7180000084749:149551-149662:+	ACA1_192660	PBP_domain domain-containing protein [Source:UniProtKB/TrEMBL;Acc:L8GPC6]	0.024246273	0.396476333	0.37223006	1.09E-07
6h	ACA1_322420/clean/scf7180000084463:22720-22833:-	ACA1_322420	hypothetical protein	0.0084737	0.380668667	0.372194967	2.79E-07
6h	ACA1_323110/clean/scf7180000084467:4448-4543:+	ACA1_323110	hypothetical protein	0	0.372158333	0.372158333	4.15E-08
6h	ACA1_379080/clean/scf7180000084711:276786-276865:+	ACA1_379080	GATA-type domain-containing protein [Source:UniProtKB/TrEMBL;Acc:L8GS28]	0.001054853	0.373159333	0.37210448	2.41E-10
6h	ACA1_307520/clean/scf7180000083680:25965-26033:-	ACA1_307520	B5 domain-containing protein [Source:UniProtKB/TrEMBL;Acc:L8H4Y1]	0.000786163	0.372883	0.372096837	4.66E-13
6h	ACA1_101080/clean/scf7180000084649:35804-35874:-	ACA1_101080	Purple acid phosphatase [Source:UniProtKB/TrEMBL;Acc:L8GGG9]	0.000949534	0.372988333	0.3720388	2.20E-12
6h	ACA1_273880/clean/scf7180000084773:77862-77977:-	ACA1_273880	Peptidase_S8 domain-containing protein [Source:UniProtKB/TrEMBL;Acc:L8GI59]	0.003043721	0.375055333	0.372011612	7.52E-07
6h	ACA1_362650/clean/scf7180000084624:124114-124224:+	ACA1_362650	G-patch domain-containing protein [Source:UniProtKB/TrEMBL;Acc:L8GFY2]	0.002711103	0.374594333	0.37188323	9.02E-09
6h	ACA1_043610/clean/scf7180000084573:184100-184177:-	ACA1_043610	FYVE-type domain-containing protein [Source:UniProtKB/TrEMBL;Acc:L8GV86]	0.00070922	0.372547667	0.371838447	2.39E-06
6h	ACA1_068440/clean/scf7180000084603:195006-195082:+	ACA1_068440	F-box domain-containing protein [Source:UniProtKB/TrEMBL;Acc:L8HDJ5]	0.009406967	0.381127	0.371720033	0.000784765
6h	ACA1_240060/clean/scf7180000084763:191650-191731:-	ACA1_240060	COesterase domain-containing protein [Source:UniProtKB/TrEMBL;Acc:L8GKI1]	0.0037637	0.375437667	0.371673966	4.55E-10
6h	ACA1_239790/clean/scf7180000084763:176607-176683:-	ACA1_239790	SurE domain-containing protein [Source:UniProtKB/TrEMBL;Acc:L8GJY0]	0.009880833	0.381551667	0.371670833	2.53E-06
6h	ACA1_369720/clean/scf7180000084664:367484-367579:-	ACA1_369720	Ras subfamily protein [Source:UniProtKB/TrEMBL;Acc:L8GY91]	0.01099062	0.382633333	0.371642713	7.74E-05
6h	ACA1_150560/clean/scf7180000084716:12758-12822:-	ACA1_150560	hypothetical protein	0.021168917	0.392750667	0.37158175	6.10E-12
6h	ACA1_112100/clean/scf7180000084669:110452-110563:+	ACA1_112100	BTB/POZ domain containing protein [Source:UniProtKB/TrEMBL;Acc:L8H3Y0]	0.0121524	0.383715	0.3715626	1.01E-06
6h	ACA1_173090/clean/scf7180000084741:399713-399792:+	ACA1_173090	Arginine-tRNA-protein transferase, putative [Source:UniProtKB/TrEMBL;Acc:L8HHK6]	3.93E-05	0.371493667	0.371454396	5.90E-10
6h	ACA1_055580/clean/scf7180000084594:737657-737738:+	ACA1_055580	RasGEF domain containing protein [Source:UniProtKB/TrEMBL;Acc:L8H6C9]	0.217575667	0.588996667	0.371421	0.0007019
6h	ACA1_369660/clean/scf7180000084664:350925-350997:-	ACA1_369660	Signal recognition particle subunit SRP72 [Source:UniProtKB/TrEMBL;Acc:L8H0D1]	0.002420407	0.373820667	0.37140026	1.90E-07
6h	ACA1_221620/clean/scf7180000084760:6445-6539:-	ACA1_221620	Purple acid phosphatase [Source:UniProtKB/TrEMBL;Acc:L8GV66]	0.00982532	0.381201	0.37137568	2.23E-16
6h	ACA1_388680/clean/scf7180000084733:146059-146129:-	ACA1_388680	Frizzled/Smoothened family membrane region protein [Source:UniProtKB/TrEMBL;Acc:L8GEN4]	0.164373233	0.535729667	0.371356433	0.000556619
6h	ACA1_301240/clean/scf7180000083203:7377-7535:+	ACA1_301240	Usp domain-containing protein [Source:UniProtKB/TrEMBL;Acc:L8H1P7]	0.008477167	0.379811333	0.371334167	5.49E-11
6h	ACA1_381590/clean/scf7180000084712:187425-187513:+	ACA1_381590	PI3K/PI4K domain-containing protein [Source:UniProtKB/TrEMBL;Acc:L8GN80]	0.006944433	0.378169667	0.371225233	1.25E-06
6h	ACA1_246770/clean/scf7180000084763:578592-578670:+	ACA1_246770	Metallophos domain-containing protein [Source:UniProtKB/TrEMBL;Acc:L8GKY3]	0.006980283	0.378113	0.371132717	2.05E-12
6h	ACA1_166070/clean/scf7180000084732:112050-112168:-	ACA1_166070	Malate dehydrogenase [Source:UniProtKB/TrEMBL;Acc:L8HIY2]	0.000550237	0.371682333	0.371132097	1.58E-12
6h	ACA1_061860/clean/scf7180000084599:1065715-1065789:+	ACA1_061860	ATP_transf domain-containing protein [Source:UniProtKB/TrEMBL;Acc:L8GW40]	0.00325733	0.374367	0.37110967	7.80E-08
6h	ACA1_107270/clean/scf7180000084660:167346-167442:-	ACA1_107270	AAA domain-containing protein [Source:UniProtKB/TrEMBL;Acc:L8GQA9]	0.00034348	0.371449333	0.371105853	2.12E-09
6h	ACA1_268540/clean/scf7180000084770:908225-908334:+	ACA1_268540	Ras-GEF domain-containing protein [Source:UniProtKB/TrEMBL;Acc:L8H452]	0.028208521	0.399169333	0.370960813	2.26E-06
6h	ACA1_069760/clean/scf7180000084603:548260-548331:-	ACA1_069760	VWFA domain-containing protein [Source:UniProtKB/TrEMBL;Acc:L8HG18]	0	0.370912333	0.370912333	6.12E-05
6h	ACA1_381640/clean/scf7180000084712:202051-202132:+	ACA1_381640	hypothetical protein	0.0483021	0.419178333	0.370876233	0.000312712
6h	ACA1_368230/clean/scf7180000084664:85669-85735:-	ACA1_368230	C2 domain-containing protein [Source:UniProtKB/TrEMBL;Acc:L8GYW6]	0	0.370859333	0.370859333	0.000151578
6h	ACA1_019960/clean/scf7180000084033:2110-2213:+	ACA1_019960	SWIRM domain containing protein [Source:UniProtKB/TrEMBL;Acc:L8GUS1]	0.025318023	0.395916667	0.370598643	1.23E-11
6h	ACA1_077300/clean/scf7180000084612:287557-287643:+	ACA1_077300	Serine/threonine kinase [Source:UniProtKB/TrEMBL;Acc:L8GP03]	0.024390233	0.394794667	0.370404433	0.001217814
6h	ACA1_128200/clean/scf7180000084697:46067-46151:-	ACA1_128200	CBS domain containing protein [Source:UniProtKB/TrEMBL;Acc:L8GUU2]	0.0276651	0.398042	0.3703769	0.000260906
6h	ACA1_390540/clean/scf7180000084740:65784-65852:+	ACA1_390540	Phosphoesterase family protein [Source:UniProtKB/TrEMBL;Acc:L8GNV0]	0.025825923	0.396111333	0.37028541	1.80E-05
6h	ACA1_216320/clean/scf7180000084758:466607-466719:+	ACA1_216320	Homogentisate 1,2dioxygenase [Source:UniProtKB/TrEMBL;Acc:L8GQ83]	0.009266353	0.37941	0.370143647	1.71E-14
6h	ACA1_313620/clean/scf7180000083822:14037-14116:-	ACA1_313620	DUF4461 domain-containing protein [Source:UniProtKB/TrEMBL;Acc:L8GLZ5]	0.005847967	0.375979667	0.3701317	2.82E-07
6h	ACA1_094910/clean/scf7180000084645:788068-788240:-	ACA1_094910	Deoxyhypusine synthase [Source:UniProtKB/TrEMBL;Acc:L8GL08]	0.004807157	0.374916333	0.370109177	2.25E-09
6h	ACA1_060530/clean/scf7180000084599:702515-702621:+	ACA1_060530	TPR_REGION domain-containing protein [Source:UniProtKB/TrEMBL;Acc:L8GYY8]	0.029000133	0.399096333	0.3700962	0.000171675
6h	ACA1_255750/clean/scf7180000084768:560812-560965:+	ACA1_255750	hypothetical protein	0.051705033	0.421783333	0.3700783	8.16E-08
6h	ACA1_397190/clean/scf7180000084759:168159-168237:+	ACA1_397190	Metallophos domain-containing protein [Source:UniProtKB/TrEMBL;Acc:L8HDM9]	0.000209894	0.370262333	0.37005244	2.34E-14
6h	ACA1_015340/clean/scf7180000083794:8651-8724:+	ACA1_015340	RNA_lig_T4_1 domain-containing protein [Source:UniProtKB/TrEMBL;Acc:L8GIY2]	0.052670187	0.422656667	0.36998648	0.002175546
6h	ACA1_382370/clean/scf7180000084721:42250-42320:-	ACA1_382370	P-type domain-containing protein [Source:UniProtKB/TrEMBL;Acc:L8GVY3]	0.070443867	0.440421	0.369977133	9.88E-06
6h	ACA1_171960/clean/scf7180000084741:208480-208555:-	ACA1_171960	F-box domain-containing protein [Source:UniProtKB/TrEMBL;Acc:L8HHE3]	0.029411767	0.399380667	0.3699689	7.87E-06
6h	ACA1_207360/clean/scf7180000084757:61352-61449:-	ACA1_207360	Tr-type G domain-containing protein [Source:UniProtKB/TrEMBL;Acc:L8GXB6]	0	0.369966667	0.369966667	5.22E-07
6h	ACA1_046390/clean/scf7180000084577:84248-84359:-	ACA1_046390	hypothetical protein	0.0061747	0.376101667	0.369926967	3.27E-05
6h	ACA1_025670/clean/scf7180000084451:1888-1976:-	ACA1_025670	DOMON domain containing protein [Source:UniProtKB/TrEMBL;Acc:L8GGT9]	0	0.369858	0.369858	2.79E-09
6h	ACA1_200240/clean/scf7180000084753:418890-418976:-	ACA1_200240	Phospholipase D active site domain containing protein [Source:UniProtKB/TrEMBL;Acc:L8H3B9]	0.028872067	0.398719333	0.369847267	7.63E-10
6h	ACA1_210510/clean/scf7180000084758:42178-42279:+	ACA1_210510	hypothetical protein	0.00274914	0.372522333	0.369773193	1.91E-07
6h	ACA1_012020/clean/scf7180000083652:4590-4683:-	ACA1_012020	hypothetical protein	0.006834651	0.376595667	0.369761016	2.92E-12
6h	ACA1_399880/clean/scf7180000084775:58262-58352:+	ACA1_399880	Rab-GAP TBC domain-containing protein [Source:UniProtKB/TrEMBL;Acc:L8GGF5]	0.010183563	0.379898333	0.36971477	1.72E-07
6h	ACA1_174540/clean/scf7180000084741:731587-731741:-	ACA1_174540	Fucosylgalactoside 3-alpha-galactosyltransferase [Source:UniProtKB/TrEMBL;Acc:L8HJC5]	0.033917367	0.403556	0.369638633	1.48E-08
6h	ACA1_298630/clean/scf7180000082992:5428-5589:-	ACA1_298630	Vacuolar protein sorting 16, putative [Source:UniProtKB/TrEMBL;Acc:L8H081]	0.001579016	0.371207333	0.369628318	9.47E-11
6h	ACA1_269320/clean/scf7180000084770:971980-972071:-	ACA1_269320	eIF2 gamma, putative [Source:UniProtKB/TrEMBL;Acc:L8H4Y2]	0.004235047	0.373805667	0.36957062	2.38E-13
6h	ACA1_034160/clean/scf7180000084561:37460-37550:+	ACA1_034160	FERM domain-containing protein [Source:UniProtKB/TrEMBL;Acc:L8GV43]	0.02578364	0.395117667	0.369334027	1.04E-15
6h	ACA1_366990/clean/scf7180000084641:537575-537668:-	ACA1_366990	UPF0020 domain-containing protein [Source:UniProtKB/TrEMBL;Acc:L8GPM7]	0.00305207	0.372349333	0.369297263	5.79E-08
6h	ACA1_057940/clean/scf7180000084599:214420-214484:+	ACA1_057940	Alpha-mannosidase [Source:UniProtKB/TrEMBL;Acc:L8GX61]	0.003022004	0.372241333	0.369219329	1.46E-09
6h	ACA1_248540/clean/scf7180000084766:73487-73566:+	ACA1_248540	Dynein light chain tctextype, putative [Source:UniProtKB/TrEMBL;Acc:L8GXW2]	0.00195234	0.371127	0.36917466	2.94E-10
6h	ACA1_012280/clean/scf7180000083740:8343-8416:+	ACA1_012280	CobW C-terminal domain-containing protein [Source:UniProtKB/TrEMBL;Acc:L8HDD1]	0.087227533	0.456360667	0.369133133	0.001820015
6h	ACA1_034080/clean/scf7180000084561:18179-18286:-	ACA1_034080	RhoGEF domain containing protein [Source:UniProtKB/TrEMBL;Acc:L8GTW8]	0.00158691	0.370716333	0.369129423	5.38E-09

6h	ACA1_263900/clean/scf7180000084770:128660-128751:-	ACA1_263900	Calponin domain containing protein [Source:UniProtKB/TrEMBL;Acc:L8H1V7]	0.007158	0.376278	0.36912	4.51E-08
6h	ACA1_234590/clean/scf7180000084762:299812-299917:+	ACA1_234590	PH domain-containing protein [Source:UniProtKB/TrEMBL;Acc:L8H3K0]	0.01221379	0.381279333	0.369065543	2.38E-06
6h	ACA1_113520/clean/scf7180000084669:308322-308394:-	ACA1_113520	Oraov1 protein [Source:UniProtKB/TrEMBL;Acc:L8H472]	0.004458773	0.373516333	0.36905756	1.74E-19
6h	ACA1_369480/clean/scf7180000084664:309917-309996:-	ACA1_369480	SMP-LTD domain-containing protein [Source:UniProtKB/TrEMBL;Acc:L8GYZ6]	0.003875967	0.372884333	0.369008367	1.01E-07
6h	ACA1_184600/clean/scf7180000084746:5215-5293:-	ACA1_184600	Alpha-N-acetylglucosaminidase family protein [Source:UniProtKB/TrEMBL;Acc:L8H6D3]	0.005925933	0.374896333	0.3689704	2.44E-06
6h	ACA1_059480/clean/scf7180000084599:453454-453547:+	ACA1_059480	hypothetical protein	0.164464667	0.533355	0.368890333	0.020742088
6h	ACA1_097430/clean/scf7180000084645:1244828-1244909:+	ACA1_097430	AcylCoA binding protein, putative [Source:UniProtKB/TrEMBL;Acc:L8GJR6]	0.005376333	0.374180333	0.368804	4.62E-08
6h	ACA1_377500/clean/scf7180000084711:77169-77247:+	ACA1_377500	Peptidase family c78 protein [Source:UniProtKB/TrEMBL;Acc:L8GU78]	0.003856847	0.372631333	0.368774487	2.03E-08
6h	ACA1_234000/clean/scf7180000084762:183633-183732:+	ACA1_234000	Purple acid phosphatase [Source:UniProtKB/TrEMBL;Acc:L8H162]	0.00443565	0.373186	0.36875035	2.78E-20
6h	ACA1_071160/clean/scf7180000084603:877002-877103:+	ACA1_071160	Letm1 RBD domain-containing protein [Source:UniProtKB/TrEMBL;Acc:L8HE82]	0.016129033	0.384836333	0.3687073	1.93E-06
6h	ACA1_258340/clean/scf7180000084769:239845-239949:+	ACA1_258340	Protein kinase domain-containing protein [Source:UniProtKB/TrEMBL;Acc:L8GFV7]	0.010352997	0.379001667	0.36864867	1.62E-05
6h	ACA1_193110/clean/scf7180000084749:185314-185482:-	ACA1_193110	hypothetical protein	0.094646133	0.463278667	0.368632533	1.77E-09
6h	ACA1_022330/clean/scf7180000084293:8864-8937:+	ACA1_022330	Carbamoylphosphate synthase L chain, ATP-binding, putative [Source:UniProtKB/TrEMBL;Acc:L8GNZ7]	3.00E-05	0.368636333	0.368606359	1.61E-14
6h	ACA1_264360/clean/scf7180000084770:247982-248040:+	ACA1_264360	hypothetical protein	0.088501833	0.457043667	0.368541833	3.61E-05
6h	ACA1_117320/clean/scf7180000084669:976028-976106:-	ACA1_117320	FAD binding domain containing protein [Source:UniProtKB/TrEMBL;Acc:L8H415]	0.0078854	0.376381	0.3684956	1.93E-09
6h	ACA1_384860/clean/scf7180000084730:117424-117503:+	ACA1_384860	Isochorismatase domain-containing protein [Source:UniProtKB/TrEMBL;Acc:L8H995]	0.00562346	0.373946333	0.368322873	4.68E-20
6h	ACA1_065970/clean/scf7180000084599:1963947-1964010:-	ACA1_065970	Usp domain-containing protein [Source:UniProtKB/TrEMBL;Acc:L8H061]	0.051270233	0.419448333	0.3681781	1.17E-10
6h	ACA1_096820/clean/scf7180000084645:1108122-1108225:-	ACA1_096820	hypothetical protein	0.006757513	0.374885667	0.368128153	9.53E-13
6h	ACA1_362790/clean/scf7180000084624:182483-182743:-	ACA1_362790	Protein kinase domain containing protein [Source:UniProtKB/TrEMBL;Acc:L8GFL4]	0.0340722	0.402121333	0.368049133	0.000945691
6h	ACA1_061700/clean/scf7180000084599:1013449-1013522:+	ACA1_061700	Amine oxidase family protein [Source:UniProtKB/TrEMBL;Acc:L8GWP7]	0.00061889	0.368554333	0.367935443	3.17E-10
6h	ACA1_065810/clean/scf7180000084599:1913394-1913455:-	ACA1_065810	SERPIN domain-containing protein [Source:UniProtKB/TrEMBL;Acc:L8H047]	0	0.367918667	0.367918667	0.007453274
6h	ACA1_018120/clean/scf7180000083885:11460-11565:+	ACA1_018120	hypothetical protein	0.00924728	0.377052	0.36780472	7.14E-06
6h	ACA1_074800/clean/scf7180000084608:86967-87038:-	ACA1_074800	Vesicle transport protein [Source:UniProtKB/TrEMBL;Acc:L8HGS3]	0.061262367	0.429046	0.367783633	4.43E-10
6h	ACA1_237870/clean/scf7180000084763:37297-37437:+	ACA1_237870	Methyltransferase [Source:UniProtKB/TrEMBL;Acc:L8GKC5]	0.004098557	0.371865667	0.36776711	1.04E-32
6h	ACA1_035310/clean/scf7180000084564:17774-17825:+	ACA1_035310	AMP-binding domain-containing protein [Source:UniProtKB/TrEMBL;Acc:L8HCU7]	0.012823377	0.380452	0.367628623	2.80E-06
6h	ACA1_031950/clean/scf7180000084523:40789-40866:+	ACA1_031950	CBS domain containing protein [Source:UniProtKB/TrEMBL;Acc:L8H414]	0.1772997	0.544919	0.3676193	0.000410099
6h	ACA1_144350/clean/scf7180000084709:213014-213163:+	ACA1_144350	CN hydrolase domain-containing protein [Source:UniProtKB/TrEMBL;Acc:L8HEV4]	0.003837557	0.371443	0.367605443	4.80E-13
6h	ACA1_306840/clean/scf7180000083668:16589-16680:+	ACA1_306840	RTP1_C1 domain-containing protein [Source:UniProtKB/TrEMBL;Acc:L8GQF6]	0	0.367514667	0.367514667	4.33E-07
6h	ACA1_366990/clean/scf7180000084641:538248-538325:-	ACA1_366990	UPF0020 domain-containing protein [Source:UniProtKB/TrEMBL;Acc:L8GPM7]	0.004660347	0.372143333	0.367482987	6.00E-07
6h	ACA1_201510/clean/scf7180000084753:577414-577494:+	ACA1_201510	Ubiquitin fusion degradation protein, putative [Source:UniProtKB/TrEMBL;Acc:L8H4X5]	0.01094349	0.378379333	0.367435843	1.47E-08
6h	ACA1_314440/clean/scf7180000083843:10994-11075:-	ACA1_314440	Protein kinase domain-containing protein [Source:UniProtKB/TrEMBL;Acc:L8HEY9]	0.002459547	0.369800667	0.36734112	8.01E-07
6h	ACA1_027500/clean/scf7180000084469:6658-6734:-	ACA1_027500	hypothetical protein	0	0.367161667	0.367161667	5.98E-10
6h	ACA1_144430/clean/scf7180000084709:241010-241169:-	ACA1_144430	CASP_C domain-containing protein [Source:UniProtKB/TrEMBL;Acc:L8HI28]	0.012631457	0.379772333	0.367140877	1.90E-14
6h	ACA1_066150/clean/scf7180000084599:2012380-2012448:+	ACA1_066150	Myosin head (Motor domain) domain containing protein [Source:UniProtKB/TrEMBL;Acc:L8GX00]	0	0.367131	0.367131	0.000134806
6h	ACA1_374080/clean/scf7180000084679:215376-215475:-	ACA1_374080	Peptidase family C78 [Source:UniProtKB/TrEMBL;Acc:L8GJH3]	0.0087945	0.375897	0.3671025	3.65E-06
6h	ACA1_123640/clean/scf7180000084686:49453-49539:+	ACA1_123640	hypothetical protein	0.0591807	0.426256333	0.367075633	1.93E-10
6h	ACA1_255530/clean/scf7180000084768:534754-534854:-	ACA1_255530	Carrier superfamily protein [Source:UniProtKB/TrEMBL;Acc:L8HB08]	0	0.367032	0.367032	2.03E-08
6h	ACA1_396150/clean/scf7180000084759:28954-29031:+	ACA1_396150	Dynein heavy chain protein [Source:UniProtKB/TrEMBL;Acc:L8HCA8]	0	0.367025333	0.367025333	5.18E-09
6h	ACA1_262400/clean/scf7180000084770:43571-43671:+	ACA1_262400	Probable tRNA N6-adenosine threonylcarbamoyltransferase [Source:UniProtKB/TrEMBL;Acc:L8H1V9]	0.00457467	0.371576667	0.367001997	5.16E-11
6h	ACA1_164180/clean/scf7180000084729:180252-180340:+	ACA1_164180	Phosphatidylinositol-3,4,5-trisphosphate 3-phosphatase [Source:UniProtKB/TrEMBL;Acc:L8GR06]	0	0.366722667	0.366722667	2.63E-06
6h	ACA1_198380/clean/scf7180000084753:95669-95775:-	ACA1_198380	AMP-binding domain-containing protein [Source:UniProtKB/TrEMBL;Acc:L8H4H9]	0.01317947	0.379781	0.36660153	2.48E-05
6h	ACA1_387910/clean/scf7180000084733:87498-87572:-	ACA1_387910	P/Homo B domain-containing protein [Source:UniProtKB/TrEMBL;Acc:L8GG58]	0.003994933	0.370524	0.366529067	2.99E-09
6h	ACA1_171940/clean/scf7180000084741:204878-204961:-	ACA1_171940	hypothetical protein	0.011037533	0.377530333	0.3664928	0.00019724
6h	ACA1_061900/clean/scf7180000084599:1074126-1074213:-	ACA1_061900	GTP_EFTU_D3 domain-containing protein [Source:UniProtKB/TrEMBL;Acc:L8GY16]	0.003091553	0.369556333	0.36646478	1.07E-08
6h	ACA1_349510/clean/scf7180000084690:16762-16843:-	ACA1_349510	Regulator of chromosome condensation (RCC1) repeat domain containing protein [Source:UniProtKB/TrEMBL;Acc:L8GJC5]	0.158317333	0.524752333	0.366435	0.005503238
6h	ACA1_169220/clean/scf7180000084739:31631-31698:+	ACA1_169220	ANK_REP_REGION domain-containing protein [Source:UniProtKB/TrEMBL;Acc:L8HFG6]	0.004972445	0.371277333	0.366304888	4.15E-09
6h	ACA1_062250/clean/scf7180000084599:1149887-1149953:-	ACA1_062250	C2 and SH3 domain containing protein [Source:UniProtKB/TrEMBL;Acc:L8GY56]	0.004540667	0.370839	0.366298333	1.02E-08
6h	ACA1_066600/clean/scf7180000084600:26404-26511:-	ACA1_066600	Myotubularin phosphatase domain-containing protein [Source:UniProtKB/TrEMBL;Acc:L8GNT0]	0	0.366294333	0.366294333	8.13E-07
6h	ACA1_203780/clean/scf7180000084754:212209-212287:+	ACA1_203780	hypothetical protein	0.002693701	0.368840667	0.366146966	1.04E-08
6h	ACA1_109730/clean/scf7180000084663:10678-10797:-	ACA1_109730	Replication factor C subunit 1 [Source:UniProtKB/TrEMBL;Acc:L8HIF4]	0.00243309	0.368530333	0.366097243	8.55E-09
6h	ACA1_366940/clean/scf7180000084641:518913-519036:-	ACA1_366940	BTB/POZ domain containing protein [Source:UniProtKB/TrEMBL;Acc:L8GPM1]	0.109015033	0.475100667	0.366085633	0.000233672
6h	ACA1_277500/clean/scf7180000084776:64999-65080:-	ACA1_277500	DBR1 domain-containing protein [Source:UniProtKB/TrEMBL;Acc:L8H8N5]	0.1331831	0.499172333	0.365989233	0.002363893
6h	ACA1_095990/clean/scf7180000084645:929530-929583:-	ACA1_095990	GTPaseactivator protein for Ras-like GTPase [Source:UniProtKB/TrEMBL;Acc:L8GJB0]	0.164106333	0.530037	0.365930667	0.000268163
6h	ACA1_060380/clean/scf7180000084599:665803-665878:-	ACA1_060380	ATG13 domain-containing protein [Source:UniProtKB/TrEMBL;Acc:L8GYW8]	0.003921567	0.369716333	0.365794767	1.54E-07
6h	ACA1_261030/clean/scf7180000084769:584175-584277:-	ACA1_261030	hypothetical protein	0.133165333	0.498943	0.365777667	0.015066228
6h	ACA1_035640/clean/scf7180000084564:101858-101972:-	ACA1_035640	Beta-lactamase domain-containing protein [Source:UniProtKB/TrEMBL;Acc:L8H9P2]	0	0.365772333	0.365772333	0.016607538
6h	ACA1_062880/clean/scf7180000084599:1299258-1299350:+	ACA1_062880	hypothetical protein	0.013614847	0.379380333	0.365765487	6.06E-06
6h	ACA1_142840/clean/scf7180000084708:245796-245869:-	ACA1_142840	hypothetical protein	0.038945467	0.404606333	0.365660867	8.11E-08
6h	ACA1_305550/clean/scf7180000083609:19042-19144:+	ACA1_305550	Folate gamma-glutamyl hydrolase [Source:UniProtKB/TrEMBL;Acc:L8GKY1]	0.001510537	0.367134	0.365623463	1.60E-11
6h	ACA1_329650/clean/scf7180000084562:37827-37914:-	ACA1_329650	hypothetical protein	0.027044213	0.392558667	0.365514453	5.17E-12
6h	ACA1_046390/clean/scf7180000084577:84893-85000:-	ACA1_046390	hypothetical protein	0.0219561	0.387386	0.3654299	0.000777949
6h	ACA1_147520/clean/scf7180000084714:30769-30881:+	ACA1_147520	RanBP2-type domain-containing protein [Source:UniProtKB/TrEMBL;Acc:L8GKJ7]	0.050008503	0.415350667	0.365342163	5.06E-05
6h	ACA1_366690/clean/scf7180000084641:481553-481642:+	ACA1_366690	Aspartyl aminopeptidase [Source:UniProtKB/TrEMBL;Acc:L8GM39]	0.0128421	0.378118667	0.365276567	2.55E-17
6h	ACA1_176030/clean/scf7180000084741:1109151-1109222:-	ACA1_176030	ATPase, AAA domain containing protein [Source:UniProtKB/TrEMBL;Acc:L8HI90]	0.00096154	0.366188333	0.365226793	1.47E-05
6h	ACA1_129370/clean/scf7180000084698:11680-11785:+	ACA1_129370	GRAM domain-containing protein [Source:UniProtKB/TrEMBL;Acc:L8GPH7]	0.004390533	0.369544667	0.365154133	1.36E-08
6h	ACA1_331800/clean/scf7180000084578:33940-34061:-	ACA1_331800	hypothetical protein	0.022005767	0.387119333	0.365113567	4.02E-05
6h	ACA1_152930/clean/scf7180000084720:34858-34927:-	ACA1_152930	PG_binding_1 domain-containing protein [Source:UniProtKB/TrEMBL;Acc:L8HF34]	0.011615033	0.376638	0.365022967	1.22E-07
6h	ACA1_381600/clean/scf7180000084712:193812-193897:+	ACA1_381600	Golgi family protein, putative [Source:UniProtKB/TrEMBL;Acc:L8GP66]	0.014728133	0.379739333	0.3650112	1.58E-08
6h	ACA1_088660/clean/scf7180000084639:198771-198881:-	ACA1_088660	RhoGEF domain containing protein [Source:UniProtKB/TrEMBL;Acc:L8GX30]	0	0.364860333	0.364860333	1.23E-07
6h	ACA1_261980/clean/scf7180000084770:12145-12224:+	ACA1_261980	Defective in cullin neddylation protein [Source:UniProtKB/TrEMBL;Acc:L8H1R9]	0.008815427	0.373668	0.364852573	3.89E-09
6h	ACA1_027030/clean/scf7180000084464:27978-28086:-	ACA1_027030	C2 domain-containing protein [Source:UniProtKB/TrEMBL;Acc:L8GJ80]	0.001831503	0.366577	0.364745497	7.47E-10
6h	ACA1_113880/clean/scf7180000084669:397824-397930:-	ACA1_113880	hypothetical protein	0.017393937	0.382086333	0.364692397	5.75E-07
6h	ACA1_087940/clean/scf7180000084639:137047-137107:+	ACA1_087940	ANK_REP_REGION domain-containing protein [Source:UniProtKB/TrEMBL;Acc:L8GTX2]	0.019437213	0.384127667	0.364690453	0.000388451
6h	ACA1_282940/clean/scf7180000084776:752372-752454:-	ACA1_282940	hypothetical protein	0	0.364608667	0.364608667	1.47E-10
6h	ACA1_031780/clean/scf7180000084523:804-904:-	ACA1_031780	Lactamase_B domain-containing protein [Source:UniProtKB/TrEMBL;Acc:L8H103]	0.052660267	0.417240333	0.364580067	4.45E-07
6h	ACA1_062580/clean/scf7180000084599:1238158-1238216:-	ACA1_062580	Phosducin domain-containing protein [Source:UniProtKB/TrEMBL;Acc:L8GWB8]	0.0264777	0.391008667	0.364530967	3.95E-06
6h	ACA1_374430/clean/scf7180000084679:296537-296632:-	ACA1_374430	Cyclin N-terminal domain-containing protein [Source:UniProtKB/TrEMBL;Acc:L8GGZ8]	0.009918717	0.374406333	0.364487617	1.35E-06
6h	ACA1_164900/clean/scf7180000084729:306381-306459:-	ACA1_164900	Bromo domain-containing protein [Source:UniProtKB/TrEMBL;Acc:L8GSQ5]	0.012242857	0.376726333	0.364483477	6.90E-11
6h	ACA1_288630/clean/scf7180000084777:253569-253646:-	ACA1_288630	Mediator of RNA polymerase II transcription subunit 20 [Source:UniProtKB/TrEMBL;Acc:L8HIV7]	0.003092683	0.367492	0.364399317	1.89E-08
6h	ACA1_272270/clean/scf7180000084772:44324-44442:-	ACA1_272270	ANK_REP_REGION domain-containing protein [Source:UniProtKB/TrEMBL;Acc:L8HIL0]	0.0601249	0.424453667	0.364328767	2.11E-05
6h	ACA1_214610/clean/scf7180000084758:234403-234478:-	ACA1_214610	Aminotran_1_2 domain-containing protein [Source:UniProtKB/TrEMBL;Acc:L8GQ78]	0.011393583	0.375616333	0.36422275	3.67E-07
6h	ACA1_127620/clean/scf7180000084696:35972-36040:+	ACA1_127620	Fe2OG dioxygenase domain-containing protein [Source:UniProtKB/TrEMBL;Acc:L8GGE8]	0.013333333	0.377548	0.364214667	5.20E-05
6h	ACA1_276370/clean/scf7180000084774:157610-157783:-	ACA1_276370	unspecified product	0.01070266	0.374849667	0.364147007	2.45E-11
6h	ACA1_158500/clean/scf7180000084724:239124-239230:+	ACA1_158500	Nudix hydrolase domain-containing protein [Source:UniProtKB/TrEMBL;Acc:L8HA53]	0.033215397	0.397321667	0.36410627	5.38E-06
6h	ACA1_201200/clean/scf7180000084753:503819-503898:+	ACA1_201200	Ras subfamily protein [Source:UniProtKB/TrEMBL;Acc:L8H3F2]	0.018518533	0.382558	0.364039467	0.000323969
6h	ACA1_110100/clean/scf7180000084663:60937-61117:-	ACA1_110100	Protein kinase [Source:UniProtKB/TrEMBL;Acc:L8HK05]	0.002082913	0.366068667	0.363985753	7.23E-09
6h	ACA1_184600/clean/scf7180000084746:4218-4291:-	ACA1_184600	Alpha-N-acetylglucosaminidase family protein [Source:UniProtKB/TrEMBL;Acc:L8H6D3]	0.006014827	0.36993	0.363915173	8.34E-06
6h	ACA1_093770/clean/scf7180000084645:642884-643002:+	ACA1_093770	hypothetical protein	0.020736733	0.384439667	0.363702933	7.61E-06
6h	ACA1_372610/clean/scf7180000084679:26211-26320:-	ACA1_372610	Asp_Arg_Hydrox domain-containing protein [Source:UniProtKB/TrEMBL;Acc:L8GH09]	0.000734907	0.364399667	0.36366476	3.50E-08
6h	ACA1_171400/clean/scf7180000084741:95534-95620:-	ACA1_171400	ATPase/histidine kinase/DNA gyrase B/HSP90 domain containing protein [Source:UniProtKB/TrEMBL;Acc:L8HJK8]	0.0069775	0.370619	0.3636415	1.62E-05
6h	ACA1_061670/clean/scf7180000084599:1001264-1001359:-	ACA1_061670	SSD domain-containing protein [Source:UniProtKB/TrEMBL;Acc:L8GWV2]	0.000733677	0.364362667	0.36362899	5.07E-08
6h	ACA1_320790/clean/scf7180000084416:22914-23073:-	ACA1_320790	RUN domain-containing protein [Source:UniProtKB/TrEMBL;Acc:L8GTR9]	8.28E-05	0.363562	0.363479164	6.85E-12
6h	ACA1_210400/clean/scf7180000084758:39748-39833:-	ACA1_210400	PPM-type phosphatase domain-containing protein [Source:UniProtKB/TrEMBL;Acc:L8GQQ0]	0.005172453	0.368585667	0.363413213	1.73E-08
6h	ACA1_071580/clean/scf7180000084603:998403-998482:-	ACA1_071580	GOLD domain-containing protein [Source:UniProtKB/TrEMBL;Acc:L8HGH0]	0	0.363330333	0.363330333	3.28E-08
6h	ACA1_125790/clean/scf7180000084691:73086-73179:-	ACA1_125790	Nuclear protein E33 isoform a isoform 3, putative [Source:UniProtKB/TrEMBL;Acc:L8GNY7]	0.010514422	0.373723667	0.363209244	1.47E-08
6h	ACA1_261080/clean/scf7180000084769:597038-597138:-	ACA1_261080	MIT domain-containing protein [Source:UniProtKB/TrEMBL;Acc:L8GF47]	0.00086077	0.363752667	0.362891897	1.51E-09
6h	ACA1_024220/clean/scf7180000084441:20479-20587:+	ACA1_024220	Purinergic receptor, putative [Source:UniProtKB/TrEMBL;Acc:L8GLB7]	0.0330933	0.39593	0.3628367	8.80E-18
6h	ACA1_063890/clean/scf7180000084599:1527245-1527337:+	ACA1_063890	DAO domain-containing protein [Source:UniProtKB/TrEMBL;Acc:L8GXC2]	0.0100769	0.372870333	0.362793433	5.56E-12
6h	ACA1_289060/clean/scf7180000084777:345688-345762:+	ACA1_289060	hypothetical protein	0.006838407	0.369614667	0.36277626	5.83E-18
6h	ACA1_210510/clean/scf7180000084758:41972-42093:+	ACA1_210510	hypothetical protein	0.00603137	0.368576667	0.362545297	3.20E-07
6h	ACA1_232470/clean/scf7180000084762:35230-35308:-	ACA1_232470	hypothetical protein	0.047921567	0.410377333	0.362455767	2.16E-09
6h	ACA1_076200/clean/scf7180000084612:24668-24744:-	ACA1_076200	PITH domain-containing protein [Source:UniProtKB/TrEMBL;Acc:L8GL73]	0.002687337	0.365036	0.362348663	9.97E-08
6h	ACA1_258610/clean/scf7180000084769:281479-281566:+	ACA1_258610	hypothetical protein	0.0314285	0.393706333	0.362277833	3.29E-05
6h	ACA1_051920/clean/scf7180000084592:86344-86429:+	ACA1_051920	SMP-LTD domain-containing protein [Source:UniProtKB/TrEMBL;Acc:L8GP37]	0	0.362260333	0.362260333	4.73E-09
6h	ACA1_220710/clean/scf7180000084758:950349-950440:+	ACA1_220710	Malate dehydrogenase [Source:UniProtKB/TrEMBL;Acc:L8GQP0]	0.002618797	0.364824333	0.362205536	1.70E-15
6h	ACA1_018110/clean/scf7180000083885:9899-9971:-	ACA1_018110	hypothetical protein	0.031645233	0.393825	0.362179767	8.32E-17
6h	ACA1_234820/clean/scf7180000084762:323620-323700:-	ACA1_234820	Serine/threonine-protein phosphatase [Source:UniProtKB/TrEMBL;Acc:L8H0J1]	0.007906643	0.369927667	0.362021023	4.10E-10
6h	ACA1_053470/clean/scf7180000084594:286750-286801:-	ACA1_053470	hypothetical protein	0.0108876	0.372903333	0.362015733	7.91E-07
6h	ACA1_366370/clean/scf7180000084641:403127-403225:+	ACA1_366370	2OG-FeII_Oxy_2 domain-containing protein [Source:UniProtKB/TrEMBL;Acc:L8GLS4]	0.01750805	0.379467667	0.361959617	2.50E-15
6h	ACA1_189580/clean/scf7180000084748:52472-52550:-	ACA1_189580	Viral life cyclerelated protein [Source:UniProtKB/TrEMBL;Acc:L8GE33]	0.001150799	0.363086333	0.361935534	1.05E-10
6h	ACA1_048870/clean/scf7180000084583:5856-5968:-	ACA1_048870	hypothetical protein	0.066655633	0.428567667	0.361912033	1.19E-09
6h	ACA1_031600/clean/scf7180000084522:22241-22308:-	ACA1_031600	Leucine rich repeat-containing protein [Source:UniProtKB/TrEMBL;Acc:L8GSM8]	0.007575767	0.369437	0.361861233	2.34E-07
6h	ACA1_298630/clean/scf7180000082992:4324-4415:-	ACA1_298630	Vacuolar protein sorting 16, putative [Source:UniProtKB/TrEMBL;Acc:L8H081]	0.001357617	0.363048	0.361690383	8.01E-09
6h	ACA1_142590/clean/scf7180000084708:193123-193240:+	ACA1_142590	unspecified product	0.01457245	0.376179667	0.361607217	5.91E-11
6h	ACA1_263870/clean/scf7180000084770:124121-124238:-	ACA1_263870	MAGE domain-containing protein [Source:UniProtKB/TrEMBL;Acc:L8H1Y1]	0.0069148	0.368451667	0.361536867	0.000102334
6h	ACA1_368180/clean/scf7180000084664:72008-72140:-	ACA1_368180	NTP_transferase domain-containing protein [Source:UniProtKB/TrEMBL;Acc:L8GYW1]	0.01723076	0.378743333	0.361512573	7.09E-06
6h	ACA1_068620/clean/scf7180000084603:241302-241398:-	ACA1_068620	hypothetical protein	0	0.361460667	0.361460667	1.50E-08
6h	ACA1_210400/clean/scf7180000084758:40088-40184:-	ACA1_210400	PPM-type phosphatase domain-containing protein [Source:UniProtKB/TrEMBL;Acc:L8GQQ0]	0.00392601	0.365358667	0.361432657	2.32E-08
6h	ACA1_210540/clean/scf7180000084758:52370-52434:-	ACA1_210540	ABC transporter, ATPbinding domain containing protein [Source:UniProtKB/TrEMBL;Acc:L8GPZ1]	0.0595238	0.420940667	0.361416867	6.46E-06
6h	ACA1_142590/clean/scf7180000084708:193402-193539:+	ACA1_142590	unspecified product	0.002521059	0.363846667	0.361325608	1.51E-08
6h	ACA1_214870/clean/scf7180000084758:251764-251859:+	ACA1_214870	Rho-GAP domain-containing protein [Source:UniProtKB/TrEMBL;Acc:L8GQY9]	0.000600903	0.361921333	0.36132043	6.29E-08
6h	ACA1_100610/clean/scf7180000084648:133838-133937:-	ACA1_100610	Serine/threonine kinase [Source:UniProtKB/TrEMBL;Acc:L8GK64]	0.00037599	0.361693667	0.361317677	3.09E-11
6h	ACA1_091840/clean/scf7180000084645:214192-214254:+	ACA1_091840	Ras subfamily protein [Source:UniProtKB/TrEMBL;Acc:L8GIK8]	0.012824966	0.373972	0.361147034	7.38E-06
6h	ACA1_174180/clean/scf7180000084741:633984-634065:+	ACA1_174180	hypothetical protein	0.033813863	0.394939333	0.36112547	2.66E-07
6h	ACA1_329500/clean/scf7180000084562:25420-25498:+	ACA1_329500	hypothetical protein	0.016882887	0.377797333	0.360914447	7.59E-06
6h	ACA1_331800/clean/scf7180000084578:34207-34302:-	ACA1_331800	hypothetical protein	0.028762563	0.389609333	0.36084677	2.08E-05
6h	ACA1_097600/clean/scf7180000084645:1293231-1293503:+	ACA1_097600	Peroxidase [Source:UniProtKB/TrEMBL;Acc:L8GJT1]	0.00458425	0.365418	0.36083375	3.23E-24
6h	ACA1_156480/clean/scf7180000084723:44530-44606:+	ACA1_156480	PHB domain-containing protein [Source:UniProtKB/TrEMBL;Acc:L8GK25]	0.005282283	0.366026333	0.36074405	1.83E-19
6h	ACA1_053350/clean/scf7180000084594:267998-268097:+	ACA1_053350	Histone domain-containing protein [Source:UniProtKB/TrEMBL;Acc:L8H5Y7]	0.024269133	0.385003667	0.360734533	2.14E-07
6h	ACA1_365090/clean/scf7180000084641:234437-234503:+	ACA1_365090	ADPribosylglycohydrolase superfamily protein [Source:UniProtKB/TrEMBL;Acc:L8GP97]	0.0299433	0.390635	0.3606917	1.40E-11
6h	ACA1_072010/clean/scf7180000084603:1126168-1126249:-	ACA1_072010	hypothetical protein	0	0.360665333	0.360665333	2.83E-07
6h	ACA1_048390/clean/scf7180000084582:75905-75974:+	ACA1_048390	Domain found in dishevelled, egl10, and pleckstrin domain containing protein [Source:UniProtKB/TrEMBL;Acc:L8GDW4]	0.00638189	0.367042667	0.360660777	2.43E-08
6h	ACA1_278100/clean/scf7180000084776:173079-173177:+	ACA1_278100	F-box domain-containing protein [Source:UniProtKB/TrEMBL;Acc:L8H5T0]	0.035560667	0.396073	0.360512333	3.22E-05
6h	ACA1_106820/clean/scf7180000084660:141204-141302:-	ACA1_106820	AAA domain-containing protein [Source:UniProtKB/TrEMBL;Acc:L8GQA4]	0.005555567	0.366025333	0.360469767	2.29E-07
6h	ACA1_216420/clean/scf7180000084758:496886-497134:-	ACA1_216420	C2 domain-containing protein [Source:UniProtKB/TrEMBL;Acc:L8GQ92]	0.047307933	0.407753	0.360445067	5.87E-08
6h	ACA1_253490/clean/scf7180000084768:315380-315439:+	ACA1_253490	hypothetical protein	0.00594174	0.366341667	0.360399927	8.30E-08
6h	ACA1_380750/clean/scf7180000084712:82030-82146:-	ACA1_380750	hypothetical protein	0	0.360332	0.360332	2.99E-09
6h	ACA1_391330/clean/scf7180000084740:153748-153821:-	ACA1_391330	hypothetical protein	0.012923703	0.37324	0.360316297	1.80E-08

6h	ACA1_219480/clean/scf7180000084758:747663-747773:+	ACA1_219480	hypothetical protein	0	0.360228667	0.360228667	1.62E-11
6h	ACA1_158550/clean/scf7180000084724:249111-249213:+	ACA1_158550	Enoyl-CoA hydratase, putative [Source:UniProtKB/TrEMBL;Acc:L8HA73]	0.0888848	0.449096667	0.360211867	2.53E-07
6h	ACA1_321790/clean/scf7180000084455:22047-22145:+	ACA1_321790	Protein phosphatase 2C domain containing protein [Source:UniProtKB/TrEMBL;Acc:L8GSL9]	0	0.360188667	0.360188667	0.000230317
6h	ACA1_145790/clean/scf7180000084710:147713-147805:+	ACA1_145790	hypothetical protein	0.023199467	0.383325333	0.360125867	7.63E-13
6h	ACA1_272530/clean/scf7180000084772:56353-56433:+	ACA1_272530	VWFA domain-containing protein [Source:UniProtKB/TrEMBL;Acc:L8HIL5]	0.00194349	0.361994	0.36005051	2.47E-08
6h	ACA1_172250/clean/scf7180000084741:283940-284074:-	ACA1_172250	Leucine rich repeat domain containing protein [Source:UniProtKB/TrEMBL;Acc:L8HHK1]	0.0596078	0.419609	0.3600012	0.000137232
6h	ACA1_199790/clean/scf7180000084753:329824-329949:+	ACA1_199790	hypothetical protein	0.008199033	0.367818	0.359618967	4.45E-05
6h	ACA1_069340/clean/scf7180000084603:414272-414368:-	ACA1_069340	hypothetical protein	0.000753353	0.360364	0.359610647	2.49E-10
6h	ACA1_061040/clean/scf7180000084599:805649-805716:+	ACA1_061040	Oxidoreductase family, NADbinding Rossmann fold domain containing protein [Source:UniProtKB/TrEMBL;Acc:L8GZ28]	0.118324867	0.477935	0.359610133	8.42E-06
6h	ACA1_024630/clean/scf7180000084444:3113-3184:+	ACA1_024630	hypothetical protein	0.012518517	0.372101	0.359582483	7.20E-08
6h	ACA1_237840/clean/scf7180000084763:26986-27178:-	ACA1_237840	hypothetical protein	0.0152761	0.37485	0.3595739	2.25E-14
6h	ACA1_088790/clean/scf7180000084639:228773-228844:-	ACA1_088790	hypothetical protein	0	0.359558333	0.359558333	0.000112672
6h	ACA1_177570/clean/scf7180000084743:103161-103252:+	ACA1_177570	hypothetical protein	0.0009603	0.360498	0.3595377	6.24E-12
6h	ACA1_277880/clean/scf7180000084776:117787-117885:+	ACA1_277880	RBR-type E3 ubiquitin transferase [Source:UniProtKB/TrEMBL;Acc:L8H6I3]	0.007076393	0.366558333	0.35948194	6.64E-11
6h	ACA1_053270/clean/scf7180000084594:239782-239855:+	ACA1_053270	Nudix hydrolase domain-containing protein [Source:UniProtKB/TrEMBL;Acc:L8H547]	0	0.359440333	0.359440333	3.30E-08
6h	ACA1_199630/clean/scf7180000084753:280964-281071:-	ACA1_199630	PRAI domain-containing protein [Source:UniProtKB/TrEMBL;Acc:L8H3A6]	0.007778621	0.367207333	0.359428712	8.28E-10
6h	ACA1_368860/clean/scf7180000084664:177530-177653:+	ACA1_368860	Usp domain-containing protein [Source:UniProtKB/TrEMBL;Acc:L8GY34]	0.077643767	0.437011667	0.3593679	3.69E-07
6h	ACA1_108210/clean/scf7180000084661:12839-12929:-	ACA1_108210	PH domain containing protein [Source:UniProtKB/TrEMBL;Acc:L8GD50]	0.043520933	0.402868667	0.359347733	2.23E-05
6h	ACA1_051840/clean/scf7180000084592:62620-62695:-	ACA1_051840	RNA recognition motif domain containing protein [Source:UniProtKB/TrEMBL;Acc:L8GS29]	0.004366367	0.363656	0.359289633	6.97E-07
6h	ACA1_053340/clean/scf7180000084594:266805-266875:-	ACA1_053340	Dynein light intermediate chain [Source:UniProtKB/TrEMBL;Acc:L8H840]	0.005236787	0.364475	0.359238213	1.51E-09
6h	ACA1_260140/clean/scf7180000084769:440794-440879:+	ACA1_260140	Endonuclease III, putative [Source:UniProtKB/TrEMBL;Acc:L8GF10]	0.00538573	0.364337667	0.358951937	2.92E-06
6h	ACA1_066100/clean/scf7180000084599:1996906-1997004:-	ACA1_066100	Arf-GAP domain-containing protein [Source:UniProtKB/TrEMBL;Acc:L8GWZ8]	0.00114886	0.359909	0.35876014	2.16E-09
6h	ACA1_051660/clean/scf7180000084592:42945-43015:-	ACA1_051660	DNA repair and recombination protein RAD54B, putative [Source:UniProtKB/TrEMBL;Acc:L8GQK0]	0	0.358734	0.358734	8.16E-07
6h	ACA1_280640/clean/scf7180000084776:514154-514264:-	ACA1_280640	PH domain/RhoGEF domain containing protein [Source:UniProtKB/TrEMBL;Acc:L8H790]	0.01313591	0.371762333	0.358626423	3.13E-11
6h	ACA1_066030/clean/scf7180000084599:1976375-1976451:-	ACA1_066030	hypothetical protein	0.000792573	0.359412	0.358619427	7.01E-09
6h	ACA1_069620/clean/scf7180000084603:484301-484381:+	ACA1_069620	Poly [ADP-ribose] polymerase [Source:UniProtKB/TrEMBL;Acc:L8HDR5]	0	0.358467333	0.358467333	6.86E-06
6h	ACA1_068360/clean/scf7180000084603:180971-181059:+	ACA1_068360	Dual specificity protein kinase [Source:UniProtKB/TrEMBL;Acc:L8HDE4]	0.025093733	0.383418667	0.358324933	2.66E-15
6h	ACA1_228720/clean/scf7180000084761:196202-196282:+	ACA1_228720	BRO1 domain-containing protein [Source:UniProtKB/TrEMBL;Acc:L8HB55]	0.004245278	0.362489333	0.358244055	7.78E-10
6h	ACA1_019960/clean/scf7180000084033:2900-3001:+	ACA1_019960	SWIRM domain containing protein [Source:UniProtKB/TrEMBL;Acc:L8GUS1]	0.092737933	0.450897333	0.3581594	5.22E-08
6h	ACA1_116560/clean/scf7180000084669:781207-781308:-	ACA1_116560	PINc domain-containing protein [Source:UniProtKB/TrEMBL;Acc:L8H4U3]	0	0.358137667	0.358137667	7.23E-05
6h	ACA1_234460/clean/scf7180000084762:289735-289845:+	ACA1_234460	hypothetical protein	0.00908302	0.367197	0.35811398	2.30E-15
6h	ACA1_117070/clean/scf7180000084669:906406-906497:-	ACA1_117070	Rap-GAP domain-containing protein [Source:UniProtKB/TrEMBL;Acc:L8H3Z5]	0.006012547	0.364119333	0.358106787	6.81E-08
6h	ACA1_195840/clean/scf7180000084752:32366-32440:+	ACA1_195840	EGFlike domain containing protein [Source:UniProtKB/TrEMBL;Acc:L8HET8]	0.008124907	0.366131	0.358006093	1.40E-06
6h	ACA1_171960/clean/scf7180000084741:210162-210281:-	ACA1_171960	F-box domain-containing protein [Source:UniProtKB/TrEMBL;Acc:L8HHE3]	0.015775367	0.373777	0.358001633	8.29E-05
6h	ACA1_156050/clean/scf7180000084723:14060-14126:+	ACA1_156050	Armadillo/betacatenin-like repeat domain containing protein [Source:UniProtKB/TrEMBL;Acc:L8GIE3]	0	0.357991	0.357991	0.007801731
6h	ACA1_279530/clean/scf7180000084776:409658-409719:+	ACA1_279530	Rab4, putative [Source:UniProtKB/TrEMBL;Acc:L8H946]	0.007324437	0.365027667	0.35770323	1.67E-12
6h	ACA1_332300/clean/scf7180000084579:9249-9386:+	ACA1_332300	hypothetical protein	0.0769615	0.434663	0.3577015	0.00187794
6h	ACA1_087600/clean/scf7180000084639:67742-67840:-	ACA1_087600	DAHP_synth_1 domain-containing protein [Source:UniProtKB/TrEMBL;Acc:L8GUI0]	0.01141744	0.369094	0.35767656	5.85E-10
6h	ACA1_223060/clean/scf7180000084760:96223-96283:-	ACA1_223060	hypothetical protein	0.0101787	0.367844667	0.357665967	1.92E-06
6h	ACA1_096810/clean/scf7180000084645:1106882-1107046:+	ACA1_096810	IU_nuc_hydro domain-containing protein [Source:UniProtKB/TrEMBL;Acc:L8GJV1]	0.006578553	0.364199	0.357620447	1.10E-09
6h	ACA1_114540/clean/scf7180000084669:503826-503929:-	ACA1_114540	Carrier superfamily protein [Source:UniProtKB/TrEMBL;Acc:L8H4H4]	0.002096773	0.359664	0.357567227	2.06E-09
6h	ACA1_174580/clean/scf7180000084741:742359-742425:-	ACA1_174580	hypothetical protein	0.002027154	0.359461	0.357433846	1.14E-16
6h	ACA1_270940/clean/scf7180000084770:1172125-1172191:-	ACA1_270940	hypothetical protein	0.0905014	0.447924333	0.357422933	5.16E-05
6h	ACA1_230510/clean/scf7180000084761:369567-369657:-	ACA1_230510	hypothetical protein	0.006944433	0.364275333	0.3573309	2.24E-06
6h	ACA1_198700/clean/scf7180000084753:155062-155148:-	ACA1_198700	ABC transporter domain-containing protein [Source:UniProtKB/TrEMBL;Acc:L8H2D9]	0.002809135	0.360126333	0.357317199	1.67E-09
6h	ACA1_064510/clean/scf7180000084599:1629940-1630010:-	ACA1_064510	Small GTPbinding protein domain containing protein [Source:UniProtKB/TrEMBL;Acc:L8GWS3]	0.098887033	0.456097667	0.357210633	0.000181979
6h	ACA1_275720/clean/scf7180000084774:93371-93447:+	ACA1_275720	Enolase, Cterminal TIM barrel domain containing protein [Source:UniProtKB/TrEMBL;Acc:L8GRZ9]	0.005295507	0.362461333	0.357165827	7.25E-22
6h	ACA1_215920/clean/scf7180000084758:408234-408327:+	ACA1_215920	SH3 domain containing protein [Source:UniProtKB/TrEMBL;Acc:L8GQ68]	0.008638647	0.365680333	0.357041687	1.18E-09
6h	ACA1_091910/clean/scf7180000084645:226226-226297:-	ACA1_091910	NIPSNAP family protein [Source:UniProtKB/TrEMBL;Acc:L8GKH2]	0.007090423	0.364082333	0.35699191	9.87E-21
6h	ACA1_362610/clean/scf7180000084624:112422-112519:-	ACA1_362610	Leucine rich repeat domain containing protein [Source:UniProtKB/TrEMBL;Acc:L8GFJ8]	0.00475166	0.361645667	0.356894007	4.76E-08
6h	ACA1_198480/clean/scf7180000084753:123077-123169:+	ACA1_198480	Transcription factor III family protein [Source:UniProtKB/TrEMBL;Acc:L8H324]	0.015873	0.372736	0.356863	3.27E-05
6h	ACA1_364410/clean/scf7180000084641:107096-107173:-	ACA1_364410	INTS5_C domain-containing protein [Source:UniProtKB/TrEMBL;Acc:L8GM57]	0.370802667	0.727585333	0.356782667	0.044857183
6h	ACA1_091340/clean/scf7180000084645:94393-94473:+	ACA1_091340	UDENN domain-containing protein [Source:UniProtKB/TrEMBL;Acc:L8GIG9]	0.0408453	0.397586333	0.356741033	6.32E-05
6h	ACA1_061460/clean/scf7180000084599:932956-933047:-	ACA1_061460	Leucine rich repeat domain containing protein [Source:UniProtKB/TrEMBL;Acc:L8GZ73]	0.022957833	0.379662	0.356704167	2.46E-05
6h	ACA1_062850/clean/scf7180000084599:1285512-1285582:+	ACA1_062850	WD_REPEATS_REGION domain-containing protein [Source:UniProtKB/TrEMBL;Acc:L8GZK3]	0.00184162	0.358545667	0.356704047	3.95E-09
6h	ACA1_116740/clean/scf7180000084669:839211-839288:+	ACA1_116740	Methyltransf_2 domain-containing protein [Source:UniProtKB/TrEMBL;Acc:L8H4R1]	0.142142067	0.498825	0.356682933	3.99E-05
6h	ACA1_171230/clean/scf7180000084741:50562-50638:+	ACA1_171230	Arf-GAP domain-containing protein [Source:UniProtKB/TrEMBL;Acc:L8HHA4]	0.002814431	0.359410667	0.356596236	8.13E-10
6h	ACA1_309640/clean/scf7180000083763:7789-7904:-	ACA1_309640	hypothetical protein	0.007722987	0.364256667	0.35653368	4.79E-07
6h	ACA1_164920/clean/scf7180000084729:312227-312291:-	ACA1_164920	DJ-1_PfpI domain-containing protein [Source:UniProtKB/TrEMBL;Acc:L8GRN3]	0.018763667	0.375293667	0.35653	9.21E-15
6h	ACA1_020160/clean/scf7180000084037:20817-20907:-	ACA1_020160	Sec16_C domain-containing protein [Source:UniProtKB/TrEMBL;Acc:L8GLH3]	0.00087248	0.35734	0.35646752	4.20E-12
6h	ACA1_174050/clean/scf7180000084741:589005-589183:-	ACA1_174050	hypothetical protein	0	0.356438667	0.356438667	1.07E-07
6h	ACA1_096600/clean/scf7180000084645:1060902-1060985:+	ACA1_096600	CUE domain containing protein [Source:UniProtKB/TrEMBL;Acc:L8GJT0]	0.004154693	0.360563667	0.356408973	7.63E-10
6h	ACA1_330350/clean/scf7180000084566:44131-44210:-	ACA1_330350	SEP domain containing protein [Source:UniProtKB/TrEMBL;Acc:L8GJV6]	0.004683433	0.361003333	0.3563199	1.12E-11
6h	ACA1_107630/clean/scf7180000084660:236310-236422:+	ACA1_107630	DNA-(apurinic or apyrimidinic site) lyase [Source:UniProtKB/TrEMBL;Acc:L8GMX8]	0.349365667	0.705662667	0.356297	0.006673862
6h	ACA1_064320/clean/scf7180000084599:1585151-1585313:+	ACA1_064320	RNA_lig_T4_1 domain-containing protein [Source:UniProtKB/TrEMBL;Acc:L8GXI9]	0.003615333	0.359903333	0.356288	4.29E-07
6h	ACA1_114920/clean/scf7180000084669:587100-587180:+	ACA1_114920	Josephin domain-containing protein [Source:UniProtKB/TrEMBL;Acc:L8H4I3]	0.000237925	0.356422667	0.356184741	3.66E-08
6h	ACA1_383710/clean/scf7180000084721:251646-251739:+	ACA1_383710	Glucose-6-phosphate 1-dehydrogenase [Source:UniProtKB/TrEMBL;Acc:L8GW58]	0.003254363	0.359434	0.356179637	1.20E-12
6h	ACA1_121240/clean/scf7180000084682:9279-9362:-	ACA1_121240	Phosphatidylinositol Nacetylglucosaminyltransferase subunit c, putative [Source:UniProtKB/TrEMBL;Acc:L8GEH1]	0.013813967	0.369944	0.356130033	6.51E-06
6h	ACA1_290480/clean/scf7180000084777:599741-599824:+	ACA1_290480	AminoacyltRNA hydrolase [Source:UniProtKB/TrEMBL;Acc:L8HI62]	0.022855667	0.378973333	0.356117667	8.58E-06
6h	ACA1_116740/clean/scf7180000084669:839406-839489:+	ACA1_116740	Methyltransf_2 domain-containing protein [Source:UniProtKB/TrEMBL;Acc:L8H4R1]	0.146204567	0.502281667	0.3560771	1.87E-05
6h	ACA1_237810/clean/scf7180000084763:21205-21326:-	ACA1_237810	hypothetical protein	0.014711203	0.370743	0.356031797	1.45E-08
6h	ACA1_173090/clean/scf7180000084741:399853-399946:+	ACA1_173090	Arginine-tRNA-protein transferase, putative [Source:UniProtKB/TrEMBL;Acc:L8HHK6]	0.058401833	0.414425	0.356023167	4.28E-06
6h	ACA1_366530/clean/scf7180000084641:447336-447447:-	ACA1_366530	hypothetical protein	0	0.356005	0.356005	2.59E-05
6h	ACA1_306850/clean/scf7180000083668:20789-20860:+	ACA1_306850	RanBPMrelated, putative [Source:UniProtKB/TrEMBL;Acc:L8GNY2]	0.028812233	0.384797	0.355984767	2.62E-11
6h	ACA1_108200/clean/scf7180000084661:11050-11111:-	ACA1_108200	hypothetical protein	0	0.355984667	0.355984667	0.000175827
6h	ACA1_295260/clean/scf7180000084777:1086620-1086842:+	ACA1_295260	CTP:ethanolaminephosphate cytidylyltransferase [Source:UniProtKB/TrEMBL;Acc:L8HM00]	0.0660346	0.421966	0.3559314	4.66E-09
6h	ACA1_295530/clean/scf7180000084777:1166746-1166838:+	ACA1_295530	RhoGEF domain containing protein [Source:UniProtKB/TrEMBL;Acc:L8HJT0]	0.006323243	0.362245667	0.355922423	1.44E-09
6h	ACA1_068440/clean/scf7180000084603:195393-195464:+	ACA1_068440	F-box domain-containing protein [Source:UniProtKB/TrEMBL;Acc:L8HDJ5]	0.052662	0.408530667	0.355868667	0.0124378
6h	ACA1_176030/clean/scf7180000084741:1108914-1108997:-	ACA1_176030	ATPase, AAA domain containing protein [Source:UniProtKB/TrEMBL;Acc:L8HI90]	0	0.35581	0.35581	3.72E-06
6h	ACA1_198250/clean/scf7180000084753:65515-65678:+	ACA1_198250	hypothetical protein	0.023809533	0.379586667	0.355777133	0.000241344
6h	ACA1_229560/clean/scf7180000084761:279027-279107:-	ACA1_229560	MORN repeatcontaining protein [Source:UniProtKB/TrEMBL;Acc:L8HAA8]	0.004938267	0.360705667	0.3557674	7.98E-06
6h	ACA1_278960/clean/scf7180000084776:285690-285777:-	ACA1_278960	Cbl_N2 domain-containing protein [Source:UniProtKB/TrEMBL;Acc:L8H705]	0.00296251	0.358616667	0.355654157	2.66E-06
6h	ACA1_136150/clean/scf7180000084702:140023-140118:-	ACA1_136150	MPN domain-containing protein [Source:UniProtKB/TrEMBL;Acc:L8GEU6]	0.001347786	0.356995333	0.355647547	7.56E-11
6h	ACA1_313860/clean/scf7180000083835:2108-2202:-	ACA1_313860	STAS domain-containing protein [Source:UniProtKB/TrEMBL;Acc:L8H2N6]	0.009924077	0.365570667	0.35564659	9.89E-22
6h	ACA1_182200/clean/scf7180000084745:156033-156162:+	ACA1_182200	Aldo_ket_red domain-containing protein [Source:UniProtKB/TrEMBL;Acc:M0QSG2]	0.012692663	0.368323333	0.35563067	8.64E-10
6h	ACA1_257310/clean/scf7180000084769:149672-149746:+	ACA1_257310	CTP_transf_like domain-containing protein [Source:UniProtKB/TrEMBL;Acc:L8GH84]	0.015910133	0.371428	0.355517867	8.56E-06
6h	ACA1_180150/clean/scf7180000084744:33020-33091:-	ACA1_180150	Protein kinase domain-containing protein [Source:UniProtKB/TrEMBL;Acc:L8GDP6]	0.000738707	0.356221	0.355482293	1.20E-10
6h	ACA1_149210/clean/scf7180000084715:98277-98386:-	ACA1_149210	hypothetical protein	0.001826483	0.357297333	0.35547085	6.83E-07
6h	ACA1_062850/clean/scf7180000084599:1285680-1285750:+	ACA1_062850	WD_REPEATS_REGION domain-containing protein [Source:UniProtKB/TrEMBL;Acc:L8GZK3]	0.002983703	0.358426667	0.355442963	1.04E-09
6h	ACA1_323360/clean/scf7180000084468:11198-11284:-	ACA1_323360	SUI1 domain-containing protein [Source:UniProtKB/TrEMBL;Acc:L8H1M7]	0.003371262	0.358664667	0.355293405	2.02E-10
6h	ACA1_144430/clean/scf7180000084709:241994-242159:-	ACA1_144430	CASP_C domain-containing protein [Source:UniProtKB/TrEMBL;Acc:L8HI28]	0.00405364	0.359201333	0.355147693	5.81E-12
6h	ACA1_282370/clean/scf7180000084776:663587-663679:+	ACA1_282370	EXPERA domain-containing protein [Source:UniProtKB/TrEMBL;Acc:L8H780]	0.000100341	0.355239667	0.355139326	9.28E-10
6h	ACA1_266220/clean/scf7180000084770:633308-633416:+	ACA1_266220	Adenylate kinase, putative [Source:UniProtKB/TrEMBL;Acc:L8H4J7]	0.111021733	0.466128	0.355106267	1.66E-05
6h	ACA1_178230/clean/scf7180000084743:184735-184871:+	ACA1_178230	Transducin family protein [Source:UniProtKB/TrEMBL;Acc:L8GSK8]	0.000563585	0.355668	0.355104415	4.39E-10
6h	ACA1_166560/clean/scf7180000084736:3378-3446:-	ACA1_166560	Endoribonuclease L-PSP protein [Source:UniProtKB/TrEMBL;Acc:L8GZY5]	0.00029074	0.355345	0.35505426	1.04E-08
6h	ACA1_057820/clean/scf7180000084599:186866-186950:-	ACA1_057820	Dual specificity phosphatase, putative [Source:UniProtKB/TrEMBL;Acc:L8GYG8]	0.008492203	0.363455	0.354962797	1.45E-06
6h	ACA1_232450/clean/scf7180000084762:28330-28434:-	ACA1_232450	Protein kinase domain-containing protein [Source:UniProtKB/TrEMBL;Acc:L8H053]	0.006944433	0.361846	0.354901567	5.47E-06
6h	ACA1_254400/clean/scf7180000084768:385789-385866:+	ACA1_254400	Golgi family protein, putative [Source:UniProtKB/TrEMBL;Acc:L8HCB2]	0.003792943	0.358675667	0.354882724	6.97E-06
6h	ACA1_009890/clean/scf7180000083468:1586-1695:-	ACA1_009890	TPR_REGION domain-containing protein [Source:UniProtKB/TrEMBL;Acc:L8GDE6]	0.010546387	0.365428667	0.35488228	4.26E-14
6h	ACA1_123840/clean/scf7180000084686:71343-71416:-	ACA1_123840	tRNA_edit domain-containing protein [Source:UniProtKB/TrEMBL;Acc:L8HFB4]	0.008811477	0.363614	0.354802523	0.000120654
6h	ACA1_219070/clean/scf7180000084758:711031-711109:+	ACA1_219070	Rieske domain-containing protein [Source:UniProtKB/TrEMBL;Acc:L8GQP6]	0.001933358	0.356625333	0.354691976	3.73E-09
6h	ACA1_228630/clean/scf7180000084761:181675-181760:+	ACA1_228630	hypothetical protein	0.003572867	0.358209	0.354636133	4.16E-09
6h	ACA1_116610/clean/scf7180000084669:803197-803269:-	ACA1_116610	HECTdomain (Ubiquitin-transferase) domain containing protein [Source:UniProtKB/TrEMBL;Acc:L8H3W1]	0.001057277	0.355533667	0.35447639	1.29E-10
6h	ACA1_149410/clean/scf7180000084715:144991-145103:+	ACA1_149410	Lectin_legB domain-containing protein [Source:UniProtKB/TrEMBL;Acc:L8HDD6]	0.003584233	0.358057333	0.3544731	3.66E-08
6h	ACA1_204500/clean/scf7180000084754:298431-298516:+	ACA1_204500	hypothetical protein	0.013314237	0.367761	0.354446763	1.16E-09
6h	ACA1_037870/clean/scf7180000084568:48733-48836:+	ACA1_037870	Vacuolar protein sorting-associated protein 11 homolog [Source:UniProtKB/TrEMBL;Acc:L8GKP2]	0.002100457	0.356461	0.354360543	5.50E-09
6h	ACA1_389080/clean/scf7180000084733:192498-192641:-	ACA1_389080	Fbox domain containing protein [Source:UniProtKB/TrEMBL;Acc:L8GDU7]	0.00113533	0.355283667	0.354148337	1.92E-07
6h	ACA1_247140/clean/scf7180000084763:630109-630175:+	ACA1_247140	hypothetical protein	0.006827033	0.360921333	0.3540943	1.93E-07
6h	ACA1_235890/clean/scf7180000084762:379526-379614:+	ACA1_235890	hypothetical protein	0.0036232	0.357676	0.3540528	2.30E-08
6h	ACA1_071010/clean/scf7180000084603:831713-831772:-	ACA1_071010	Leucine rich repeat domain containing protein [Source:UniProtKB/TrEMBL;Acc:L8HE65]	0.047489833	0.40139	0.353900167	4.35E-05
6h	ACA1_383700/clean/scf7180000084721:247054-247127:+	ACA1_383700	Endo/exonuclease/phosphatase domain-containing protein [Source:UniProtKB/TrEMBL;Acc:L8GV68]	0	0.353830333	0.353830333	9.04E-09
6h	ACA1_264920/clean/scf7180000084770:401969-402072:-	ACA1_264920	Protein kinase domain-containing protein [Source:UniProtKB/TrEMBL;Acc:L8H3L2]	0.018530167	0.372109667	0.3535795	0.000243511
6h	ACA1_113540/clean/scf7180000084669:314829-314913:-	ACA1_113540	SCP domain-containing protein [Source:UniProtKB/TrEMBL;Acc:L8H3B1]	0.003236247	0.356804333	0.353568087	2.87E-08
6h	ACA1_373550/clean/scf7180000084679:97348-97456:-	ACA1_373550	CBS domain containing protein [Source:UniProtKB/TrEMBL;Acc:L8GH26]	0.064651667	0.418199	0.353547333	0.002510325
6h	ACA1_189360/clean/scf7180000084748:16941-17012:-	ACA1_189360	Proline dehydrogenase [Source:UniProtKB/TrEMBL;Acc:L8GGF7]	0.001961673	0.355273333	0.35331166	0.00058508
6h	ACA1_360060/clean/scf7180000084622:40068-40138:-	ACA1_360060	GHMP_kinases_C domain-containing protein [Source:UniProtKB/TrEMBL;Acc:L8HF07]	0.014953083	0.368192333	0.35323925	1.51E-09
6h	ACA1_182890/clean/scf7180000084745:285759-285866:+	ACA1_182890	hypothetical protein	0	0.353187667	0.353187667	1.76E-10
6h	ACA1_068700/clean/scf7180000084603:258575-258649:+	ACA1_068700	Purple acid phosphatase [Source:UniProtKB/TrEMBL;Acc:L8HFR9]	0.001941982	0.354941	0.352999018	6.93E-12
6h	ACA1_399770/clean/scf7180000084775:31941-32077:+	ACA1_399770	PARP catalytic domain-containing protein [Source:UniProtKB/TrEMBL;Acc:L8GGQ1]	0.0047146	0.357539667	0.352825067	7.78E-06
6h	ACA1_377500/clean/scf7180000084711:75320-75443:+	ACA1_377500	Peptidase family c78 protein [Source:UniProtKB/TrEMBL;Acc:L8GU78]	0	0.352803333	0.352803333	4.11E-08
6h	ACA1_306690/clean/scf7180000083668:5095-5181:-	ACA1_306690	Gammasecretase subunit APH-1B, putative [Source:UniProtKB/TrEMBL;Acc:L8GQE8]	0.008460233	0.361205	0.352744767	2.21E-07
6h	ACA1_048870/clean/scf7180000084583:6674-6807:-	ACA1_048870	hypothetical protein	0.109194367	0.46191	0.352715633	6.64E-07
6h	ACA1_109730/clean/scf7180000084663:12984-13076:-	ACA1_109730	Replication factor C subunit 1 [Source:UniProtKB/TrEMBL;Acc:L8HIF4]	0.015328023	0.367946333	0.35261831	4.12E-09
6h	ACA1_091880/clean/scf7180000084645:220382-220450:+	ACA1_091880	Ubiquitin-like domain-containing protein [Source:UniProtKB/TrEMBL;Acc:L8GIV5]	0	0.352487667	0.352487667	1.12E-11
6h	ACA1_179160/clean/scf7180000084743:313382-313518:+	ACA1_179160	hypothetical protein	0.007847097	0.360232	0.352384903	1.45E-07
6h	ACA1_230470/clean/scf7180000084761:356415-356493:+	ACA1_230470	hypothetical protein	0	0.352311333	0.352311333	4.53E-05
6h	ACA1_052780/clean/scf7180000084594:178097-178173:-	ACA1_052780	F-box domain-containing protein [Source:UniProtKB/TrEMBL;Acc:L8H818]	0.0190476	0.371303	0.3522554	6.61E-05
6h	ACA1_228910/clean/scf7180000084761:222557-222616:+	ACA1_228910	hypothetical protein	0.010235243	0.36245	0.352214757	5.46E-09
6h	ACA1_362610/clean/scf7180000084624:112600-112679:-	ACA1_362610	Leucine rich repeat domain containing protein [Source:UniProtKB/TrEMBL;Acc:L8GFJ8]	0.006390557	0.358488667	0.35209811	4.00E-08
6h	ACA1_020860/clean/scf7180000084095:27963-28052:+	ACA1_020860	Mitochondrial ribosomal protein, L38, putative [Source:UniProtKB/TrEMBL;Acc:L8GTU5]	0.0032597	0.355343667	0.352083967	9.19E-09
6h	ACA1_216040/clean/scf7180000084758:441277-441367:-	ACA1_216040	Protein kinase domain-containing protein [Source:UniProtKB/TrEMBL;Acc:L8GQH1]	0	0.351997333	0.351997333	5.65E-10
6h	ACA1_061350/clean/scf7180000084599:889801-889882:+	ACA1_061350	PlsC domain-containing protein [Source:UniProtKB/TrEMBL;Acc:L8GWS1]	0.0840952	0.436078667	0.351983467	0.000178271
6h	ACA1_298660/clean/scf7180000082992:14957-15026:+	ACA1_298660	Bromo domain-containing protein [Source:UniProtKB/TrEMBL;Acc:L8H1K3]	0.011768543	0.363720333	0.35195179	2.08E-06
6h	ACA1_277700/clean/scf7180000084776:85015-85170:-	ACA1_277700	WD_REPEATS_REGION domain-containing protein [Source:UniProtKB/TrEMBL;Acc:L8H8P1]	0.004438393	0.356325667	0.351887273	9.89E-14
6h	ACA1_054090/clean/scf7180000084594:398387-398478:-	ACA1_054090	Fbox domain containing protein [Source:UniProtKB/TrEMBL;Acc:L8H592]	0.072338567	0.424203	0.351864433	0.000168071
6h	ACA1_024020/clean/scf7180000084440:5901-5983:-	ACA1_024020	RhoGAP domain containing protein [Source:UniProtKB/TrEMBL;Acc:L8GJP9]	0.003718693	0.355436333	0.35171764	1.42E-08
6h	ACA1_292890/clean/scf7180000084777:929125-929280:-	ACA1_292890	Ribosomal_S17_N domain-containing protein [Source:UniProtKB/TrEMBL;Acc:L8HL46]	0.002301863	0.353988	0.351686137	2.02E-60
6h	ACA1_036520/clean/scf7180000084565:128822-128929:+	ACA1_036520	WD domain, G-beta repeat-containing protein [Source:UniProtKB/TrEMBL;Acc:L8HBN8]	0.014966187	0.366634333	0.351668147	3.92E-09

6h	ACA1_283030/clean/scf7180000084776:770511-770595:+	ACA1_283030	Rattus norvegicus clone C53 CDK5 activator-binding family protein [Source:UniProtKB/TrEMBL;Acc:L8H9N6]	0.015743867	0.367298333	0.351554467	1.19E-06
6h	ACA1_114920/clean/scf7180000084669:587222-587330:+	ACA1_114920	Josephin domain-containing protein [Source:UniProtKB/TrEMBL;Acc:L8H4I3]	0.015237633	0.366741333	0.3515037	4.46E-07
6h	ACA1_107160/clean/scf7180000084660:158305-158417:+	ACA1_107160	hypothetical protein	0.00030987	0.351777	0.35146713	9.96E-12
6h	ACA1_380780/clean/scf7180000084712:87267-87363:-	ACA1_380780	hypothetical protein	0.02471923	0.376106667	0.351387437	4.15E-09
6h	ACA1_111550/clean/scf7180000084669:16161-16253:-	ACA1_111550	alpha-1,2-Mannosidase [Source:UniProtKB/TrEMBL;Acc:L8H589]	0.008658	0.360044333	0.351386333	4.47E-07
6h	ACA1_106800/clean/scf7180000084660:134310-134398:+	ACA1_106800	EF hand domain containing protein [Source:UniProtKB/TrEMBL;Acc:L8GNA6]	0.00102148	0.352344333	0.351322854	6.18E-10
6h	ACA1_183960/clean/scf7180000084745:527800-527901:-	ACA1_183960	Phosphoesterase family protein [Source:UniProtKB/TrEMBL;Acc:L8H8F6]	0.0359064	0.387119667	0.351213267	1.20E-09
6h	ACA1_224380/clean/scf7180000084760:188717-188789:+	ACA1_224380	hypothetical protein	0.103748333	0.454923	0.351174667	0.002515854
6h	ACA1_060200/clean/scf7180000084599:612625-612712:-	ACA1_060200	hypothetical protein	0	0.351113667	0.351113667	4.75E-08
6h	ACA1_264320/clean/scf7180000084770:235487-235565:-	ACA1_264320	PH domain-containing protein [Source:UniProtKB/TrEMBL;Acc:L8H1Z3]	0.000746083	0.351856667	0.351110583	3.26E-08
6h	ACA1_321550/clean/scf7180000084454:1954-2049:-	ACA1_321550	F-box domain-containing protein [Source:UniProtKB/TrEMBL;Acc:L8GYJ0]	0	0.351090667	0.351090667	1.29E-09
6h	ACA1_369090/clean/scf7180000084664:240376-240452:-	ACA1_369090	2hydroxyacyl-CoA lyase 1, putative [Source:UniProtKB/TrEMBL;Acc:L8GZ11]	0.011299433	0.362354333	0.3510549	1.65E-06
6h	ACA1_377500/clean/scf7180000084711:76982-77057:+	ACA1_377500	Peptidase family c78 protein [Source:UniProtKB/TrEMBL;Acc:L8GU78]	0.045418167	0.396466333	0.351048167	1.28E-08
6h	ACA1_054860/clean/scf7180000084594:593116-593235:+	ACA1_054860	hypothetical protein	0.01428253	0.365124667	0.350842137	6.08E-05
6h	ACA1_315790/clean/scf7180000083943:8714-8778:-	ACA1_315790	Copper transport accessory protein [Source:UniProtKB/TrEMBL;Acc:L8HDX3]	0.006163737	0.35696	0.350796263	2.43E-15
6h	ACA1_336110/clean/scf7180000084593:26373-26477:-	ACA1_336110	RING-type domain-containing protein [Source:UniProtKB/TrEMBL;Acc:L8H352]	0.007368867	0.358148667	0.3507798	0.000128866
6h	ACA1_366610/clean/scf7180000084641:462725-462820:+	ACA1_366610	DNAbinding protein HU superfamily protein [Source:UniProtKB/TrEMBL;Acc:L8GMK2]	0.003559063	0.354259	0.350699937	3.78E-17
6h	ACA1_230470/clean/scf7180000084761:355828-355890:+	ACA1_230470	hypothetical protein	0	0.350678667	0.350678667	5.18E-05
6h	ACA1_368140/clean/scf7180000084664:66043-66118:-	ACA1_368140	Glutamine amidotransferase type-2 domain-containing protein [Source:UniProtKB/TrEMBL;Acc:L8H040]	0	0.350649667	0.350649667	0.000159434
6h	ACA1_150570/clean/scf7180000084716:13500-13563:-	ACA1_150570	Rhodanese domain-containing protein [Source:UniProtKB/TrEMBL;Acc:L8H0M9]	0.0152828	0.365906	0.3506232	9.31E-18
6h	ACA1_217150/clean/scf7180000084758:527957-528044:-	ACA1_217150	Protein kinase domain-containing protein [Source:UniProtKB/TrEMBL;Acc:L8GR83]	0.0145196	0.365140333	0.350620733	3.68E-06
6h	ACA1_157260/clean/scf7180000084724:24782-24899:+	ACA1_157260	hypothetical protein	0.017996467	0.368415667	0.3504192	7.72E-11
6h	ACA1_015560/clean/scf7180000083799:20788-20875:+	ACA1_015560	hypothetical protein	0.00822501	0.358629	0.35040399	2.56E-13
6h	ACA1_185040/clean/scf7180000084746:74904-74992:-	ACA1_185040	hypothetical protein	0.022730063	0.373125333	0.35039527	1.31E-08
6h	ACA1_384030/clean/scf7180000084730:7451-7540:+	ACA1_384030	hypothetical protein	0.043386567	0.393711	0.350324433	7.63E-07
6h	ACA1_109730/clean/scf7180000084663:12281-12383:-	ACA1_109730	Replication factor C subunit 1 [Source:UniProtKB/TrEMBL;Acc:L8HIF4]	0.00439555	0.354668667	0.350273116	6.11E-08
6h	ACA1_192010/clean/scf7180000084749:73702-73983:+	ACA1_192010	PCI domain-containing protein [Source:UniProtKB/TrEMBL;Acc:L8GQR2]	0.00516291	0.355426333	0.350263423	2.65E-19
6h	ACA1_217150/clean/scf7180000084758:528171-528251:-	ACA1_217150	Protein kinase domain-containing protein [Source:UniProtKB/TrEMBL;Acc:L8GR83]	0.012648253	0.362808333	0.35016008	1.64E-06
6h	ACA1_119270/clean/scf7180000084672:55895-55970:+	ACA1_119270	Raclike protein [Source:UniProtKB/TrEMBL;Acc:L8HJ94]	0.002729033	0.352681	0.349951967	1.05E-26
6h	ACA1_377500/clean/scf7180000084711:75616-75695:+	ACA1_377500	Peptidase family c78 protein [Source:UniProtKB/TrEMBL;Acc:L8GU78]	0	0.349848667	0.349848667	6.59E-10
6h	ACA1_270950/clean/scf7180000084770:1173610-1173689:-	ACA1_270950	BTB/POZ domain containing protein [Source:UniProtKB/TrEMBL;Acc:L8H4F4]	0.007608883	0.357387667	0.349778783	2.14E-07
6h	ACA1_179970/clean/scf7180000084744:12240-12338:+	ACA1_179970	PPM-type phosphatase domain-containing protein [Source:UniProtKB/TrEMBL;Acc:L8GF73]	0.00326292	0.35301	0.34974708	6.76E-10
6h	ACA1_389690/clean/scf7180000084733:309282-309376:-	ACA1_389690	RBR-type E3 ubiquitin transferase [Source:UniProtKB/TrEMBL;Acc:L8GEU0]	0.0169542	0.366698667	0.349744467	3.20E-11
6h	ACA1_396260/clean/scf7180000084759:66675-66813:+	ACA1_396260	hypothetical protein	0.005241303	0.354928	0.349686697	3.84E-08
6h	ACA1_186700/clean/scf7180000084746:195975-196055:+	ACA1_186700	ADPribosylation factor subfamily protein [Source:UniProtKB/TrEMBL;Acc:L8H553]	0.00873244	0.358418	0.34968556	1.70E-06
6h	ACA1_382080/clean/scf7180000084721:1129-1184:-	ACA1_382080	Ubiquitin-like protein ATG12 [Source:UniProtKB/TrEMBL;Acc:L8GUS5]	0	0.349671	0.349671	2.92E-08
6h	ACA1_199520/clean/scf7180000084753:252448-252548:+	ACA1_199520	hypothetical protein	0.00253785	0.352190667	0.349652817	1.58E-06
6h	ACA1_361020/clean/scf7180000084622:223753-223883:-	ACA1_361020	Ribosomal L22e protein family [Source:UniProtKB/TrEMBL;Acc:L8HF78]	0.001500158	0.351146333	0.349646176	3.92E-53
6h	ACA1_306690/clean/scf7180000083668:5555-5647:-	ACA1_306690	Gammasecretase subunit APH-1B, putative [Source:UniProtKB/TrEMBL;Acc:L8GQE8]	0.007370613	0.357006667	0.349636053	3.29E-09
6h	ACA1_219050/clean/scf7180000084758:705438-705531:-	ACA1_219050	hypothetical protein	0.011569893	0.361135	0.349565107	2.97E-10
6h	ACA1_024020/clean/scf7180000084440:7763-7834:-	ACA1_024020	RhoGAP domain containing protein [Source:UniProtKB/TrEMBL;Acc:L8GJP9]	0	0.349521	0.349521	1.90E-09
6h	ACA1_325210/clean/scf7180000084475:30781-30892:+	ACA1_325210	CitMHS domain-containing protein [Source:UniProtKB/TrEMBL;Acc:L8GPH6]	0.01517252	0.364480333	0.349307813	6.10E-12
6h	ACA1_142390/clean/scf7180000084708:157897-157981:-	ACA1_142390	Acetyltransferase component of pyruvate dehydrogenase complex [Source:UniProtKB/TrEMBL;Acc:L8HCS5]	0.000743917	0.350047667	0.34930375	1.71E-15
6h	ACA1_053370/clean/scf7180000084594:270222-270305:-	ACA1_053370	acidPPc domain-containing protein [Source:UniProtKB/TrEMBL;Acc:L8H555]	0.017649733	0.366938	0.349288267	1.36E-08
6h	ACA1_266750/clean/scf7180000084770:704077-704156:-	ACA1_266750	Ubiquitin domain containing protein [Source:UniProtKB/TrEMBL;Acc:L8H3V4]	0.007559067	0.356822	0.349262933	3.01E-11
6h	ACA1_399230/clean/scf7180000084759:277602-277689:+	ACA1_399230	Synaptobrevin protein [Source:UniProtKB/TrEMBL;Acc:L8HCF2]	0.049472233	0.398718	0.349245767	2.48E-10
6h	ACA1_096600/clean/scf7180000084645:1058657-1058759:+	ACA1_096600	CUE domain containing protein [Source:UniProtKB/TrEMBL;Acc:L8GJT0]	0.002690214	0.351930667	0.349240452	1.50E-08
6h	ACA1_078990/clean/scf7180000084615:125843-125973:-	ACA1_078990	AcylCoA synthetase, putative [Source:UniProtKB/TrEMBL;Acc:L8GRK9]	9.43E-05	0.3493	0.349205665	6.62E-15
6h	ACA1_094690/clean/scf7180000084645:784964-785076:+	ACA1_094690	Fanconi anemia complementation group D2 family protein [Source:UniProtKB/TrEMBL;Acc:L8GJ52]	0.286008	0.635170333	0.349162333	0.032117643
6h	ACA1_071630/clean/scf7180000084603:1006127-1006212:+	ACA1_071630	SAM_MT_RSMB_NOP domain-containing protein [Source:UniProtKB/TrEMBL;Acc:L8HGH8]	0.011904767	0.361022667	0.3491179	2.91E-05
6h	ACA1_339760/clean/scf7180000084619:20437-20503:-	ACA1_339760	Alpha/beta hydrolase, putative [Source:UniProtKB/TrEMBL;Acc:L8GKI7]	0.025038167	0.374044333	0.349006167	1.66E-07
6h	ACA1_103170/clean/scf7180000084653:12172-12259:+	ACA1_103170	CBS domain containing protein [Source:UniProtKB/TrEMBL;Acc:L8GCP7]	0.00229292	0.351225333	0.348932413	1.76E-05
6h	ACA1_228910/clean/scf7180000084761:222724-222807:+	ACA1_228910	hypothetical protein	0.0262177	0.375131	0.3489133	9.60E-11
6h	ACA1_072500/clean/scf7180000084603:1217831-1217929:-	ACA1_072500	F-box domain-containing protein [Source:UniProtKB/TrEMBL;Acc:L8HFX9]	0.003215433	0.351985667	0.348770233	0.00240148
6h	ACA1_397190/clean/scf7180000084759:169163-169242:+	ACA1_397190	Metallophos domain-containing protein [Source:UniProtKB/TrEMBL;Acc:L8HDM9]	0.00213047	0.350848	0.34871753	1.36E-11
6h	ACA1_389130/clean/scf7180000084733:208785-208858:+	ACA1_389130	BTB/POZ domain containing protein [Source:UniProtKB/TrEMBL;Acc:L8GDV1]	0.009133237	0.357828667	0.34869543	1.58E-14
6h	ACA1_330350/clean/scf7180000084566:44409-44484:-	ACA1_330350	SEP domain containing protein [Source:UniProtKB/TrEMBL;Acc:L8GJV6]	0.001513344	0.350171333	0.34865799	5.16E-09
6h	ACA1_361220/clean/scf7180000084622:270850-270928:+	ACA1_361220	EF hand domain containing protein [Source:UniProtKB/TrEMBL;Acc:L8HC55]	0.005708367	0.354352667	0.3486443	4.47E-07
6h	ACA1_113610/clean/scf7180000084669:331967-332146:-	ACA1_113610	AAA_16 domain-containing protein [Source:UniProtKB/TrEMBL;Acc:L8H6C2]	0.051333233	0.399948667	0.348615433	4.98E-07
6h	ACA1_174020/clean/scf7180000084741:578459-578559:+	ACA1_174020	ANK_REP_REGION domain-containing protein [Source:UniProtKB/TrEMBL;Acc:L8HGR2]	0.011112003	0.359695333	0.34858333	2.19E-10
6h	ACA1_333220/clean/scf7180000084581:20760-20852:+	ACA1_333220	hypothetical protein	0	0.348577667	0.348577667	8.73E-09
6h	ACA1_399880/clean/scf7180000084775:56003-56114:+	ACA1_399880	Rab-GAP TBC domain-containing protein [Source:UniProtKB/TrEMBL;Acc:L8GGF5]	0	0.348535333	0.348535333	1.99E-08
6h	ACA1_087350/clean/scf7180000084639:12801-12894:+	ACA1_087350	Protein prenyltransferase, putative [Source:UniProtKB/TrEMBL;Acc:L8GVD3]	0	0.348483667	0.348483667	2.36E-05
6h	ACA1_367310/clean/scf7180000084641:596383-596464:-	ACA1_367310	CCHC-type domain-containing protein [Source:UniProtKB/TrEMBL;Acc:L8GMZ2]	0.019070533	0.367538	0.348467467	7.62E-09
6h	ACA1_026960/clean/scf7180000084464:210-308:-	ACA1_026960	FtsX domain-containing protein [Source:UniProtKB/TrEMBL;Acc:L8GHC2]	0.003583927	0.352002333	0.348418407	6.13E-09
6h	ACA1_216430/clean/scf7180000084758:502348-502421:+	ACA1_216430	Ubiquitin carboxyl-terminal hydrolase [Source:UniProtKB/TrEMBL;Acc:L8GSQ7]	0.048093433	0.396459667	0.348366233	2.30E-05
6h	ACA1_074110/clean/scf7180000084605:29861-29969:-	ACA1_074110	Peptidase [Source:UniProtKB/TrEMBL;Acc:L8HJ70]	7.91E-05	0.348411333	0.348332251	2.23E-10
6h	ACA1_209120/clean/scf7180000084757:275829-275933:+	ACA1_209120	Leucine rich repeat domain containing protein [Source:UniProtKB/TrEMBL;Acc:L8GYC0]	0.004016067	0.352303333	0.348287267	1.43E-06
6h	ACA1_195250/clean/scf7180000084751:82816-82885:+	ACA1_195250	hypothetical protein	0.002569493	0.350803	0.348233507	1.50E-07
6h	ACA1_283220/clean/scf7180000084776:791864-791958:+	ACA1_283220	Calponin domain containing protein [Source:UniProtKB/TrEMBL;Acc:L8H7M9]	0.005368467	0.353601667	0.3482332	3.37E-05
6h	ACA1_382920/clean/scf7180000084721:120003-120076:-	ACA1_382920	Ras subfamily protein [Source:UniProtKB/TrEMBL;Acc:L8GV31]	0.003058103	0.351249667	0.348191563	0.000143499
6h	ACA1_067410/clean/scf7180000084601:20097-20177:-	ACA1_067410	F-box domain-containing protein [Source:UniProtKB/TrEMBL;Acc:L8GTP9]	0	0.348167667	0.348167667	3.76E-06
6h	ACA1_320540/clean/scf7180000084416:8426-8517:-	ACA1_320540	Calponin-homology (CH) domain-containing protein [Source:UniProtKB/TrEMBL;Acc:L8GTR4]	0.001406155	0.349535	0.348128845	1.63E-16
6h	ACA1_202730/clean/scf7180000084754:93256-93346:-	ACA1_202730	Serine/threonine protein kinase, putative [Source:UniProtKB/TrEMBL;Acc:L8GTF5]	0.164953667	0.513072333	0.348118667	0.00160581
6h	ACA1_301230/clean/scf7180000083203:5743-5824:-	ACA1_301230	hypothetical protein	0.000537883	0.348642333	0.34810445	1.51E-07
6h	ACA1_058300/clean/scf7180000084599:296463-296686:+	ACA1_058300	DH domain-containing protein [Source:UniProtKB/TrEMBL;Acc:L8GVE4]	0.0543679	0.402407667	0.348039767	4.68E-07
6h	ACA1_035640/clean/scf7180000084564:103277-103356:-	ACA1_035640	Beta-lactamase domain-containing protein [Source:UniProtKB/TrEMBL;Acc:L8H9P2]	0.076696	0.424728667	0.348032667	0.024885899
6h	ACA1_186680/clean/scf7180000084746:186389-186488:+	ACA1_186680	Rho-GAP domain-containing protein [Source:UniProtKB/TrEMBL;Acc:L8H7A6]	0.0611466	0.409079	0.3479324	5.44E-05
6h	ACA1_064190/clean/scf7180000084599:1553016-1553086:+	ACA1_064190	hypothetical protein	0.00501702	0.352864333	0.347847313	2.04E-22
6h	ACA1_381510/clean/scf7180000084712:167057-167139:+	ACA1_381510	Pyr_redox_2 domain-containing protein [Source:UniProtKB/TrEMBL;Acc:L8GNN7]	0.026539487	0.374218667	0.34767918	6.58E-14
6h	ACA1_151100/clean/scf7180000084716:55212-55306:-	ACA1_151100	NFACT-R_1 domain-containing protein [Source:UniProtKB/TrEMBL;Acc:L8H219]	0	0.347508667	0.347508667	5.76E-10
6h	ACA1_072330/clean/scf7180000084603:1200199-1200290:-	ACA1_072330	UBA_e1_C domain-containing protein [Source:UniProtKB/TrEMBL;Acc:L8HGS4]	0.003075673	0.350563	0.347487327	4.15E-10
6h	ACA1_151240/clean/scf7180000084716:79890-80121:-	ACA1_151240	OTU domain-containing protein [Source:UniProtKB/TrEMBL;Acc:L8H309]	0.0274853	0.374927667	0.347442367	3.19E-07
6h	ACA1_068620/clean/scf7180000084603:241536-241635:-	ACA1_068620	hypothetical protein	0	0.347434667	0.347434667	3.42E-08
6h	ACA1_158480/clean/scf7180000084724:233812-233900:+	ACA1_158480	Glutamine amidotransferase type-1 domain-containing protein [Source:UniProtKB/TrEMBL;Acc:L8HBH7]	0	0.347366333	0.347366333	7.61E-07
6h	ACA1_254400/clean/scf7180000084768:385963-386051:+	ACA1_254400	Golgi family protein, putative [Source:UniProtKB/TrEMBL;Acc:L8HCB2]	0.0222777	0.369599333	0.347321633	3.39E-05
6h	ACA1_384350/clean/scf7180000084730:31621-31723:+	ACA1_384350	RNA recognition motif domain containing protein [Source:UniProtKB/TrEMBL;Acc:L8H910]	0.003801061	0.351110333	0.347309272	6.37E-10
6h	ACA1_348970/clean/scf7180000084689:22092-22185:+	ACA1_348970	UBA domain-containing protein [Source:UniProtKB/TrEMBL;Acc:L8GWL7]	0.018135967	0.365436333	0.347300367	3.85E-06
6h	ACA1_172940/clean/scf7180000084741:368975-369075:+	ACA1_172940	Protein kinase domain-containing protein [Source:UniProtKB/TrEMBL;Acc:L8HHJ2]	0.0050505	0.352261667	0.347211167	0.000223449
6h	ACA1_174040/clean/scf7180000084741:582067-582231:-	ACA1_174040	Protein kinase domain-containing protein [Source:UniProtKB/TrEMBL;Acc:L8HK27]	0.073569533	0.420741	0.347171467	2.63E-08
6h	ACA1_211790/clean/scf7180000084758:114621-114717:+	ACA1_211790	RhoGEF domain containing protein [Source:UniProtKB/TrEMBL;Acc:L8GQS6]	0.090796333	0.437854667	0.347058333	5.46E-06
6h	ACA1_103160/clean/scf7180000084653:10249-10339:+	ACA1_103160	PALP domain-containing protein [Source:UniProtKB/TrEMBL;Acc:L8GDL9]	0.00824995	0.355271667	0.347021717	1.63E-09
6h	ACA1_338180/clean/scf7180000084604:49039-49122:-	ACA1_338180	hypothetical protein	0.002340676	0.349284667	0.346943991	2.29E-12
6h	ACA1_156030/clean/scf7180000084723:7948-8031:+	ACA1_156030	hypothetical protein	0	0.346940667	0.346940667	5.52E-07
6h	ACA1_060100/clean/scf7180000084599:583514-583616:+	ACA1_060100	DENN (AEX3) domain containing protein [Source:UniProtKB/TrEMBL;Acc:L8GXI7]	0	0.346827667	0.346827667	3.80E-08
6h	ACA1_313600/clean/scf7180000083822:8657-8767:-	ACA1_313600	Peptidyl-prolyl cis-trans isomerase [Source:UniProtKB/TrEMBL;Acc:L8GNF0]	0.0225338	0.369346667	0.346812867	5.34E-13
6h	ACA1_329660/clean/scf7180000084562:42484-42552:+	ACA1_329660	Putative ubiquitin carboxylterminal hydrolase 11, putative [Source:UniProtKB/TrEMBL;Acc:L8GHL1]	0	0.346780667	0.346780667	1.65E-06
6h	ACA1_060920/clean/scf7180000084599:779077-779184:-	ACA1_060920	MFS domain-containing protein [Source:UniProtKB/TrEMBL;Acc:L8GVT5]	0.066413867	0.413180333	0.346766467	2.24E-05
6h	ACA1_278320/clean/scf7180000084776:195978-196056:+	ACA1_278320	N-acetyltransferase domain-containing protein [Source:UniProtKB/TrEMBL;Acc:L8H8U2]	0.018628833	0.365300333	0.3466715	2.12E-06
6h	ACA1_100480/clean/scf7180000084648:93704-93778:-	ACA1_100480	Fbox/LRR-repeat protein [Source:UniProtKB/TrEMBL;Acc:L8GLX9]	0	0.346637	0.346637	4.97E-06
6h	ACA1_309660/clean/scf7180000083763:12047-12158:+	ACA1_309660	hypothetical protein	0.00306872	0.349702	0.34663328	7.36E-07
6h	ACA1_071580/clean/scf7180000084603:997551-997630:-	ACA1_071580	GOLD domain-containing protein [Source:UniProtKB/TrEMBL;Acc:L8HGH0]	0.010198933	0.356732667	0.346533733	1.12E-05
6h	ACA1_133200/clean/scf7180000084701:20338-20414:-	ACA1_133200	Protein kinase domain containing protein [Source:UniProtKB/TrEMBL;Acc:L8H3K5]	0.0127796	0.359246333	0.346466733	1.35E-06
6h	ACA1_051550/clean/scf7180000084592:35889-35957:-	ACA1_051550	Phenylalanine hydroxylase [Source:UniProtKB/TrEMBL;Acc:L8GPS8]	0.016760733	0.363219	0.346458267	5.56E-16
6h	ACA1_216390/clean/scf7180000084758:489629-489798:-	ACA1_216390	MI domain-containing protein [Source:UniProtKB/TrEMBL;Acc:L8GQI1]	0.004635467	0.351057	0.346421533	2.79E-07
6h	ACA1_253350/clean/scf7180000084768:279778-279877:-	ACA1_253350	Protein kinase domain containing protein [Source:UniProtKB/TrEMBL;Acc:L8H9Z2]	0.004084767	0.350480333	0.346395567	1.23E-05
6h	ACA1_295390/clean/scf7180000084777:1124678-1124794:-	ACA1_295390	Aamy domain-containing protein [Source:UniProtKB/TrEMBL;Acc:L8HIM5]	0.000697749	0.347077	0.346379251	1.09E-11
6h	ACA1_326170/clean/scf7180000084504:46208-46289:+	ACA1_326170	SAM domain-containing protein [Source:UniProtKB/TrEMBL;Acc:L8HIK1]	0.019061233	0.365419	0.346357767	8.24E-10
6h	ACA1_377490/clean/scf7180000084711:74242-74334:+	ACA1_377490	Fe2OG dioxygenase domain-containing protein [Source:UniProtKB/TrEMBL;Acc:L8GRQ4]	0.022146643	0.36847	0.346323357	5.09E-07
6h	ACA1_007780/clean/scf7180000083166:28143-28309:+	ACA1_007780	V-type proton ATPase subunit G [Source:UniProtKB/TrEMBL;Acc:L8H9M3]	0.00264194	0.348936233	0.346294293	8.93E-25
6h	ACA1_175980/clean/scf7180000084741:1097616-1097730:+	ACA1_175980	Trafficking protein particle complex subunit [Source:UniProtKB/TrEMBL;Acc:L8HI86]	0.002562487	0.348850667	0.34628818	4.94E-08
6h	ACA1_382920/clean/scf7180000084721:120334-120416:-	ACA1_382920	Ras subfamily protein [Source:UniProtKB/TrEMBL;Acc:L8GV31]	0	0.34625	0.34625	0.000536049
6h	ACA1_164750/clean/scf7180000084729:265255-265346:-	ACA1_164750	Carbonic anhydrase [Source:UniProtKB/TrEMBL;Acc:L8GR38]	0.00352773	0.349747333	0.346219603	1.41E-10
6h	ACA1_088960/clean/scf7180000084639:273666-273747:-	ACA1_088960	Beta-lactamase domain-containing protein [Source:UniProtKB/TrEMBL;Acc:L8GU04]	0.010977233	0.357182667	0.346205433	4.02E-05
6h	ACA1_112030/clean/scf7180000084669:89103-89186:-	ACA1_112030	COMM domain-containing protein [Source:UniProtKB/TrEMBL;Acc:L8H411]	0	0.346093333	0.346093333	7.74E-12
6h	ACA1_179970/clean/scf7180000084744:11109-11306:+	ACA1_179970	PPM-type phosphatase domain-containing protein [Source:UniProtKB/TrEMBL;Acc:L8GF73]	0.072660833	0.418753	0.346092167	3.03E-09
6h	ACA1_396170/clean/scf7180000084759:44563-44644:-	ACA1_396170	Phosphatidylserine decarboxylase proenzyme 2 [Source:UniProtKB/TrEMBL;Acc:L8HEJ4]	0.00996406	0.355986667	0.346022607	2.22E-16
6h	ACA1_092590/clean/scf7180000084645:361078-361159:-	ACA1_092590	Leucine rich repeat domain containing protein [Source:UniProtKB/TrEMBL;Acc:L8GKK8]	0.1029574	0.448942667	0.345985267	0.001974448
6h	ACA1_262400/clean/scf7180000084770:43369-43469:+	ACA1_262400	Probable tRNA N6-adenosine threonylcarbamoyltransferase [Source:UniProtKB/TrEMBL;Acc:L8H1V9]	0.0161551	0.362111667	0.345956567	1.50E-11
6h	ACA1_281350/clean/scf7180000084776:593786-593875:-	ACA1_281350	hypothetical protein	0.025149877	0.371096	0.345946123	3.75E-09
6h	ACA1_235920/clean/scf7180000084762:385520-385599:-	ACA1_235920	RRM domain-containing protein [Source:UniProtKB/TrEMBL;Acc:L8H0M8]	0.02179163	0.36773	0.34593837	1.15E-11
6h	ACA1_045300/clean/scf7180000084576:110688-110752:-	ACA1_045300	Nicotinamide n-methyltransferase [Source:UniProtKB/TrEMBL;Acc:L8GZ08]	0.417238	0.763124333	0.345886333	0.003142539
6h	ACA1_064870/clean/scf7180000084599:1716198-1716273:-	ACA1_064870	Ubiquitin-like domain-containing protein [Source:UniProtKB/TrEMBL;Acc:L8GXI8]	0.00442646	0.350309667	0.345883207	1.59E-17
6h	ACA1_097180/clean/scf7180000084645:1176805-1177095:+	ACA1_097180	C2 NT-type domain-containing protein [Source:UniProtKB/TrEMBL;Acc:L8GJN6]	0.016137153	0.362019667	0.345882513	0.000294027
6h	ACA1_336860/clean/scf7180000084596:7456-7564:-	ACA1_336860	Glutathione reductase [Source:UniProtKB/TrEMBL;Acc:L8GUD1]	0.0011066	0.346947333	0.345840733	2.53E-09
6h	ACA1_051450/clean/scf7180000084592:17304-17397:-	ACA1_051450	TMEM135_C_rich domain-containing protein [Source:UniProtKB/TrEMBL;Acc:L8GQJ0]	0.037048133	0.382884667	0.345836533	1.03E-06
6h	ACA1_184280/clean/scf7180000084745:605736-605805:+	ACA1_184280	hypothetical protein	0.057763303	0.403599333	0.34583603	1.63E-05
6h	ACA1_183970/clean/scf7180000084745:533676-533762:-	ACA1_183970	hypothetical protein	0.0103218	0.356139333	0.345817533	0.000161469
6h	ACA1_184260/clean/scf7180000084745:594176-594326:-	ACA1_184260	Phytanoyl-CoA dioxygenase [Source:UniProtKB/TrEMBL;Acc:L8H8I4]	0.00180424	0.347602667	0.345798427	9.18E-08
6h	ACA1_218390/clean/scf7180000084758:649877-649960:-	ACA1_218390	Glycosyl hydrolase family 20, catalytic domain containing protein [Source:UniProtKB/TrEMBL;Acc:L8GQD2]	0	0.345777667	0.345777667	6.72E-09
6h	ACA1_182870/clean/scf7180000084745:273240-273362:+	ACA1_182870	G-patch domain-containing protein [Source:UniProtKB/TrEMBL;Acc:L8H9D6]	0.024545347	0.370225	0.345679653	6.66E-07
6h	ACA1_369480/clean/scf7180000084664:310484-310563:-	ACA1_369480	SMP-LTD domain-containing protein [Source:UniProtKB/TrEMBL;Acc:L8GYZ6]	0.011880767	0.357444333	0.345563567	7.15E-10
6h	ACA1_107400/clean/scf7180000084660:184195-184281:-	ACA1_107400	Nucleoside diphosphate, putative [Source:UniProtKB/TrEMBL;Acc:L8GNB6]	0.008850043	0.3544	0.345549957	4.65E-07
6h	ACA1_191570/clean/scf7180000084749:18987-19155:+	ACA1_191570	Cation diffusion facilitator family transporter superfamily protein [Source:UniProtKB/TrEMBL;Acc:L8GNP6]	0.034882633	0.380417667	0.345535033	2.02E-08
6h	ACA1_057110/clean/scf7180000084599:48807-48879:-	ACA1_057110	unspecified product	0.027777767	0.373197667	0.3454199	0.006757695
6h	ACA1_064910/clean/scf7180000084599:1721261-1721367:-	ACA1_064910	Deacetylase sirtuin-type domain-containing protein [Source:UniProtKB/TrEMBL;Acc:L8GWU7]	0.01188394	0.357294667	0.345410727	2.97E-05
6h	ACA1_377350/clean/scf7180000084711:41136-41242:+	ACA1_377350	YDG/SRA domain containing protein [Source:UniProtKB/TrEMBL;Acc:L8GU64]	0.02820752	0.373382	0.34517448	6.22E-06
6h	ACA1_178060/clean/scf7180000084743:161956-162037:-	ACA1_178060	Dipeptidylpeptidase 9, putative [Source:UniProtKB/TrEMBL;Acc:L8GTD8]	0.002729267	0.347817	0.345087733	8.21E-08

6h	ACA1_073500/clean/scf7180000084603:1431819-1431888:+	ACA1_073500	MHD domain-containing protein [Source:UniProtKB/TrEMBL;Acc:L8HGZ9]	0.009129933	0.354196333	0.3450664	7.98E-06
6h	ACA1_011270/clean/scf7180000083623:5055-5144:-	ACA1_011270	FYVE-type domain-containing protein [Source:UniProtKB/TrEMBL;Acc:L8GQN9]	0.0990619	0.444098667	0.345036767	0.000885397
6h	ACA1_109130/clean/scf7180000084661:127105-127197:+	ACA1_109130	Serine/threonine kinase [Source:UniProtKB/TrEMBL;Acc:L8GDM5]	0.0274501	0.372442667	0.344992567	4.18E-05
6h	ACA1_013340/clean/scf7180000083750:2040-2117:+	ACA1_013340	PH domain-containing protein [Source:UniProtKB/TrEMBL;Acc:L8HGJ7]	0.0284574	0.373425	0.3449676	6.64E-14
6h	ACA1_208460/clean/scf7180000084757:197296-197367:-	ACA1_208460	Abhydro_lipase domain-containing protein [Source:UniProtKB/TrEMBL;Acc:L8GZL5]	0.061081233	0.406045333	0.3449641	5.67E-08
6h	ACA1_198970/clean/scf7180000084753:181198-181282:-	ACA1_198970	Viral Atype inclusion protein, putative [Source:UniProtKB/TrEMBL;Acc:L8H376]	0	0.344942	0.344942	5.29E-08
6h	ACA1_013390/clean/scf7180000083750:11683-11776:-	ACA1_013390	Aamy domain-containing protein [Source:UniProtKB/TrEMBL;Acc:L8HGK3]	0.032307293	0.377196	0.344888707	3.47E-10
6h	ACA1_290140/clean/scf7180000084777:542430-542514:-	ACA1_290140	Rap-GAP domain-containing protein [Source:UniProtKB/TrEMBL;Acc:L8HJ83]	0	0.344867333	0.344867333	6.13E-07
6h	ACA1_310800/clean/scf7180000083780:17627-17703:-	ACA1_310800	EamA domain-containing protein [Source:UniProtKB/TrEMBL;Acc:L8GVY7]	0.03660218	0.381435667	0.344833487	7.99E-06
6h	ACA1_073320/clean/scf7180000084603:1393286-1393392:+	ACA1_073320	Tyrosine phosphatase, putative [Source:UniProtKB/TrEMBL;Acc:L8HG36]	0.000144613	0.344973333	0.34482872	7.02E-13
6h	ACA1_211140/clean/scf7180000084758:75843-75920:+	ACA1_211140	Ser/Thr protein phosphatase [Source:UniProtKB/TrEMBL;Acc:L8GS86]	0.004191472	0.349016667	0.344825195	1.76E-07
6h	ACA1_156050/clean/scf7180000084723:13054-13126:+	ACA1_156050	Armadillo/betacatenin-like repeat domain containing protein [Source:UniProtKB/TrEMBL;Acc:L8GIE3]	0	0.344743667	0.344743667	0.027230492
6h	ACA1_041790/clean/scf7180000084573:12909-12980:+	ACA1_041790	Ornithine/lysine/arginine decarboxylase [Source:UniProtKB/TrEMBL;Acc:L8GV78]	0.020624023	0.365173	0.344548977	3.83E-09
6h	ACA1_372600/clean/scf7180000084679:21958-22062:-	ACA1_372600	F-box domain-containing protein [Source:UniProtKB/TrEMBL;Acc:L8GJA4]	0.067726733	0.412224667	0.344497933	9.81E-06
6h	ACA1_037810/clean/scf7180000084568:28553-28619:-	ACA1_037810	Dual specificity phosphatase, putative [Source:UniProtKB/TrEMBL;Acc:L8GLC7]	0.556096667	0.900559667	0.344463	0.005386923
6h	ACA1_074070/clean/scf7180000084605:15812-15919:-	ACA1_074070	PAS domain-containing protein [Source:UniProtKB/TrEMBL;Acc:L8HKA1]	0.00105402	0.345483333	0.344429313	3.43E-09
6h	ACA1_247140/clean/scf7180000084763:629858-630020:+	ACA1_247140	hypothetical protein	0.1362889	0.480693667	0.344404767	2.36E-06
6h	ACA1_184220/clean/scf7180000084745:586207-586308:-	ACA1_184220	RhoGEF domain containing protein [Source:UniProtKB/TrEMBL;Acc:L8HAN1]	0	0.344358333	0.344358333	3.54E-09
6h	ACA1_153200/clean/scf7180000084720:85325-85413:+	ACA1_153200	RBR-type E3 ubiquitin transferase [Source:UniProtKB/TrEMBL;Acc:L8HIB3]	0.004124057	0.348459667	0.34433561	9.77E-08
6h	ACA1_182880/clean/scf7180000084745:274698-274808:-	ACA1_182880	E3 ubiquitin-protein ligase [Source:UniProtKB/TrEMBL;Acc:L8H743]	0.013147697	0.357476333	0.344328637	8.29E-10
6h	ACA1_038420/clean/scf7180000084568:144338-144402:-	ACA1_038420	SPX domain-containing protein [Source:UniProtKB/TrEMBL;Acc:L8GKX4]	0.028646833	0.372912667	0.344265833	1.00E-06
6h	ACA1_039640/clean/scf7180000084570:10422-10497:+	ACA1_039640	hypothetical protein	0.015800897	0.360044	0.344243103	7.24E-09
6h	ACA1_057880/clean/scf7180000084599:199437-199577:-	ACA1_057880	hypothetical protein	0.02454072	0.368689333	0.344148613	2.49E-06
6h	ACA1_053300/clean/scf7180000084594:252494-252585:+	ACA1_053300	Prolyl oligopeptidase [Source:UniProtKB/TrEMBL;Acc:L8H5Y2]	0.001613303	0.345736667	0.344123363	9.24E-11
6h	ACA1_216390/clean/scf7180000084758:487487-487548:-	ACA1_216390	MI domain-containing protein [Source:UniProtKB/TrEMBL;Acc:L8GQI1]	0.054175833	0.398284	0.344108167	6.29E-11
6h	ACA1_226180/clean/scf7180000084761:36205-36297:+	ACA1_226180	5'nucleotidase [Source:UniProtKB/TrEMBL;Acc:L8H8H8]	0	0.344070667	0.344070667	7.15E-08
6h	ACA1_198250/clean/scf7180000084753:66075-66160:+	ACA1_198250	hypothetical protein	0.012103003	0.356163333	0.34406033	2.50E-05
6h	ACA1_096820/clean/scf7180000084645:1108663-1108764:-	ACA1_096820	hypothetical protein	0.0050919	0.349143333	0.344051433	5.69E-12
6h	ACA1_002770/clean/scf7180000082937:8154-8223:+	ACA1_002770	Fbox domain containing protein [Source:UniProtKB/TrEMBL;Acc:L8GJ90]	0.027190367	0.371125667	0.3439353	1.82E-13
6h	ACA1_364320/clean/scf7180000084641:78831-78919:-	ACA1_364320	TFCD_C domain-containing protein [Source:UniProtKB/TrEMBL;Acc:L8GP35]	0	0.343918	0.343918	0.000184217
6h	ACA1_140730/clean/scf7180000084707:108602-108685:-	ACA1_140730	Kelch repeat protein [Source:UniProtKB/TrEMBL;Acc:L8GGS1]	0.004115233	0.347992	0.343876767	8.94E-08
6h	ACA1_176160/clean/scf7180000084741:1136411-1136498:-	ACA1_176160	Elongation factor1 alpha, putative [Source:UniProtKB/TrEMBL;Acc:L8HJS7]	0.081597533	0.425406667	0.343809133	1.64E-06
6h	ACA1_072370/clean/scf7180000084603:1211942-1212047:-	ACA1_072370	Leucine rich repeat domain containing protein [Source:UniProtKB/TrEMBL;Acc:L8HEP0]	0.047469933	0.391199	0.343729067	0.000595201
6h	ACA1_149320/clean/scf7180000084715:128313-128428:+	ACA1_149320	DUF4209 domain-containing protein [Source:UniProtKB/TrEMBL;Acc:L8HB66]	0.014385467	0.35796	0.343574533	2.71E-06
6h	ACA1_255900/clean/scf7180000084768:578188-578307:+	ACA1_255900	DEP domain-containing protein [Source:UniProtKB/TrEMBL;Acc:L8HA90]	0.011916247	0.355465	0.343548753	1.85E-16
6h	ACA1_175700/clean/scf7180000084741:1037382-1037466:-	ACA1_175700	Frizzled/Smoothened family membrane region protein [Source:UniProtKB/TrEMBL;Acc:L8HH45]	0.038325733	0.381799333	0.3434736	2.09E-06
6h	ACA1_074110/clean/scf7180000084605:25988-26082:-	ACA1_074110	Peptidase [Source:UniProtKB/TrEMBL;Acc:L8HJ70]	0.006181643	0.349532	0.343350357	1.78E-09
6h	ACA1_253340/clean/scf7180000084768:276701-276837:-	ACA1_253340	hypothetical protein	0.014207293	0.357556333	0.34334904	1.24E-11
6h	ACA1_110100/clean/scf7180000084663:63473-63572:-	ACA1_110100	Protein kinase [Source:UniProtKB/TrEMBL;Acc:L8HK05]	0.001701093	0.345027667	0.343326573	7.60E-08
6h	ACA1_035420/clean/scf7180000084564:46156-46285:-	ACA1_035420	Carrier superfamily protein [Source:UniProtKB/TrEMBL;Acc:L8HAE9]	0.033964077	0.377221333	0.343257257	2.60E-07
6h	ACA1_261400/clean/scf7180000084769:662506-662617:-	ACA1_261400	Serine/threonine protein kinase, putative [Source:UniProtKB/TrEMBL;Acc:L8GF66]	0	0.343186667	0.343186667	0.007841727
6h	ACA1_203900/clean/scf7180000084754:218065-218158:-	ACA1_203900	Phosphatidylethanolamine Nmethyltransferase [Source:UniProtKB/TrEMBL;Acc:L8GTJ8]	0	0.343168	0.343168	3.76E-10
6h	ACA1_250800/clean/scf7180000084768:30004-30086:+	ACA1_250800	hypothetical protein	0.004863017	0.347889667	0.34302665	3.62E-08
6h	ACA1_297750/clean/scf7180000082853:1384-1500:-	ACA1_297750	14_3_3 domain-containing protein [Source:UniProtKB/TrEMBL;Acc:L8HE45]	0	0.342934333	0.342934333	0.000176514
6h	ACA1_068270/clean/scf7180000084603:148496-148596:-	ACA1_068270	Phospholipid-transporting ATPase [Source:UniProtKB/TrEMBL;Acc:L8HEP8]	0.01994666	0.362873	0.34292634	4.16E-07
6h	ACA1_137270/clean/scf7180000084703:29286-29369:-	ACA1_137270	hypothetical protein	0.018949167	0.361847333	0.342898167	0.000206612
6h	ACA1_020160/clean/scf7180000084037:19922-19986:-	ACA1_020160	Sec16_C domain-containing protein [Source:UniProtKB/TrEMBL;Acc:L8GLH3]	0.00705561	0.349949333	0.342893723	1.38E-13
6h	ACA1_288670/clean/scf7180000084777:257617-257689:-	ACA1_288670	Psf1 family protein [Source:UniProtKB/TrEMBL;Acc:L8HLA2]	0.006207333	0.349081667	0.342874333	2.62E-05
6h	ACA1_027160/clean/scf7180000084465:2956-3045:+	ACA1_027160	SSD domain-containing protein [Source:UniProtKB/TrEMBL;Acc:L8GH18]	0.002461128	0.345155333	0.342694206	1.68E-08
6h	ACA1_054930/clean/scf7180000084594:607614-607675:+	ACA1_054930	Zinc finger, zz type domain containing protein [Source:UniProtKB/TrEMBL;Acc:L8H689]	0.015001133	0.357587	0.342585867	2.40E-14
6h	ACA1_360220/clean/scf7180000084622:71091-71169:+	ACA1_360220	Ribonucloprotein [Source:UniProtKB/TrEMBL;Acc:L8HCP6]	0.003518403	0.346023	0.342504597	2.56E-24
6h	ACA1_088920/clean/scf7180000084639:264622-264700:+	ACA1_088920	Phorbol esters/diacylglycerol binding domain containing protein [Source:UniProtKB/TrEMBL;Acc:L8GUI1]	0.0042859	0.346647333	0.342361433	1.39E-06
6h	ACA1_307470/clean/scf7180000083680:10623-10709:-	ACA1_307470	FHA domain-containing protein [Source:UniProtKB/TrEMBL;Acc:L8H4X7]	0.0249974	0.367321	0.3423236	3.20E-07
6h	ACA1_280520/clean/scf7180000084776:487728-487796:-	ACA1_280520	C2 domain containing protein [Source:UniProtKB/TrEMBL;Acc:L8H660]	0.033280267	0.375588667	0.3423084	1.96E-08
6h	ACA1_358790/clean/scf7180000084742:31721-31800:-	ACA1_358790	hypothetical protein	0.011641897	0.353943333	0.342301437	1.20E-09
6h	ACA1_361070/clean/scf7180000084622:234228-234320:-	ACA1_361070	PUB domain-containing protein [Source:UniProtKB/TrEMBL;Acc:L8HD14]	0.026422217	0.368707	0.342284783	0.000236161
6h	ACA1_359690/clean/scf7180000084622:3965-4158:-	ACA1_359690	Rab-GAP TBC domain-containing protein [Source:UniProtKB/TrEMBL;Acc:L8HBR2]	0.015828093	0.358071667	0.342243573	3.56E-10
6h	ACA1_250800/clean/scf7180000084768:30219-30292:+	ACA1_250800	hypothetical protein	0.024128213	0.366329	0.342200787	2.00E-09
6h	ACA1_062880/clean/scf7180000084599:1298156-1298234:+	ACA1_062880	hypothetical protein	0	0.342195667	0.342195667	1.11E-07
6h	ACA1_185730/clean/scf7180000084746:120097-120185:+	ACA1_185730	Poly (ADPribose) glycohydrolase [Source:UniProtKB/TrEMBL;Acc:L8H490]	0.324499	0.666666667	0.342167667	0.031933865
6h	ACA1_066150/clean/scf7180000084599:2010779-2010890:+	ACA1_066150	Myosin head (Motor domain) domain containing protein [Source:UniProtKB/TrEMBL;Acc:L8GX00]	0	0.342150333	0.342150333	8.11E-05
6h	ACA1_368060/clean/scf7180000084664:44836-44896:+	ACA1_368060	PPM-type phosphatase domain-containing protein [Source:UniProtKB/TrEMBL;Acc:L8GYR6]	0.00132802	0.343339333	0.342011313	2.54E-07
6h	ACA1_360980/clean/scf7180000084622:214412-214483:+	ACA1_360980	hypothetical protein	0	0.341998667	0.341998667	2.66E-06
6h	ACA1_162510/clean/scf7180000084726:52413-52497:+	ACA1_162510	G_PROTEIN_RECEP_F2_4 domain-containing protein [Source:UniProtKB/TrEMBL;Acc:L8H134]	0.002680967	0.344667333	0.341986367	9.06E-05
6h	ACA1_177470/clean/scf7180000084743:73730-73798:+	ACA1_177470	Nudix hydrolase domain-containing protein [Source:UniProtKB/TrEMBL;Acc:L8GSI6]	0.002849003	0.344793667	0.341944663	1.79E-07
6h	ACA1_182860/clean/scf7180000084745:268926-269060:-	ACA1_182860	Ankyrin repeat protein, putative [Source:UniProtKB/TrEMBL;Acc:L8H7X7]	0	0.341917	0.341917	4.77E-06
6h	ACA1_210810/clean/scf7180000084758:70302-70378:+	ACA1_210810	PH domain-containing protein [Source:UniProtKB/TrEMBL;Acc:L8GP83]	0.024721467	0.366608	0.341886533	4.03E-06
6h	ACA1_176120/clean/scf7180000084741:1127266-1127347:+	ACA1_176120	Armadillo repeat containing 7 isoform 1 family protein [Source:UniProtKB/TrEMBL;Acc:L8HH78]	0.0100941	0.351956	0.3418619	1.00E-06
6h	ACA1_323100/clean/scf7180000084467:1895-1979:-	ACA1_323100	Protein kinase domain-containing protein [Source:UniProtKB/TrEMBL;Acc:L8GF97]	0.006636033	0.348379667	0.341743633	6.27E-07
6h	ACA1_055410/clean/scf7180000084594:699600-699677:+	ACA1_055410	Protein kinase domain-containing protein [Source:UniProtKB/TrEMBL;Acc:L8H6J6]	0.413254333	0.754876333	0.341622	0.000180313
6h	ACA1_127610/clean/scf7180000084696:31712-31840:-	ACA1_127610	Histone domain-containing protein [Source:UniProtKB/TrEMBL;Acc:L8GDT0]	0.001450839	0.342988	0.341537161	1.02E-09
6h	ACA1_389130/clean/scf7180000084733:209238-209313:+	ACA1_389130	BTB/POZ domain containing protein [Source:UniProtKB/TrEMBL;Acc:L8GDV1]	0.0224106	0.363945667	0.341535067	2.56E-13
6h	ACA1_325470/clean/scf7180000084495:15007-15185:-	ACA1_325470	BTB domain-containing protein [Source:UniProtKB/TrEMBL;Acc:M0QSM7]	0.085269533	0.4268	0.341530467	1.36E-09
6h	ACA1_070910/clean/scf7180000084603:799920-800030:-	ACA1_070910	HD domain containing protein [Source:UniProtKB/TrEMBL;Acc:L8HE54]	0.00152207	0.342747333	0.341225263	1.62E-09
6h	ACA1_063540/clean/scf7180000084599:1446534-1446604:+	ACA1_063540	hypothetical protein	0.0045662	0.345706667	0.341140467	3.68E-11
6h	ACA1_215010/clean/scf7180000084758:293378-293461:-	ACA1_215010	Inorganic diphosphatase [Source:UniProtKB/TrEMBL;Acc:L8GQA5]	0.00089848	0.342009333	0.341110853	2.81E-15
6h	ACA1_315000/clean/scf7180000083887:9514-9608:-	ACA1_315000	WD_REPEATS_REGION domain-containing protein [Source:UniProtKB/TrEMBL;Acc:L8GYT6]	0.057450333	0.398536667	0.341086333	4.63E-06
6h	ACA1_101340/clean/scf7180000084649:48345-48420:-	ACA1_101340	Leucine rich repeat domain containing protein [Source:UniProtKB/TrEMBL;Acc:L8GH62]	0.082027333	0.422901333	0.340874	1.74E-05
6h	ACA1_292140/clean/scf7180000084777:860346-860436:-	ACA1_292140	hypothetical protein	0.0111111	0.351952333	0.340841233	2.58E-05
6h	ACA1_280630/clean/scf7180000084776:513188-513285:-	ACA1_280630	Palmitoyltransferase [Source:UniProtKB/TrEMBL;Acc:L8H675]	0.005183927	0.345986	0.340802073	5.04E-11
6h	ACA1_057110/clean/scf7180000084599:47217-47277:-	ACA1_057110	unspecified product	0.1274929	0.468234333	0.340741433	0.002900669
6h	ACA1_144880/clean/scf7180000084710:31250-31334:-	ACA1_144880	hypothetical protein	0.005055647	0.3457	0.340644353	3.94E-10
6h	ACA1_353620/clean/scf7180000084717:46741-46845:-	ACA1_353620	RhoGAP domain containing protein [Source:UniProtKB/TrEMBL;Acc:L8GGS6]	0.0509797	0.391574667	0.340594967	5.69E-09
6h	ACA1_048230/clean/scf7180000084582:61311-61425:+	ACA1_048230	Protein kinase domain-containing protein [Source:UniProtKB/TrEMBL;Acc:L8GE93]	0.0442734	0.384866	0.3405926	1.42E-06
6h	ACA1_048650/clean/scf7180000084582:187916-188042:+	ACA1_048650	Hsp40, putative [Source:UniProtKB/TrEMBL;Acc:L8GDD9]	0.030496	0.371042	0.340546	1.12E-11
6h	ACA1_266090/clean/scf7180000084770:592220-592311:+	ACA1_266090	Protein kinase domain-containing protein [Source:UniProtKB/TrEMBL;Acc:L8H3R7]	0	0.34054	0.34054	1.26E-07
6h	ACA1_310750/clean/scf7180000083780:6854-6919:+	ACA1_310750	RFX-type winged-helix domain-containing protein [Source:UniProtKB/TrEMBL;Acc:L8GVY4]	0	0.340531667	0.340531667	2.36E-08
6h	ACA1_183480/clean/scf7180000084745:425959-426075:-	ACA1_183480	EF hand,SPla/RYanodine receptor (SPRY) domain containing protein [Source:UniProtKB/TrEMBL;Acc:L8H9K0]	0.001560664	0.34207	0.340509336	5.53E-09
6h	ACA1_198250/clean/scf7180000084753:63778-63847:+	ACA1_198250	hypothetical protein	0	0.340471	0.340471	7.92E-08
6h	ACA1_061900/clean/scf7180000084599:1074598-1074679:-	ACA1_061900	GTP_EFTU_D3 domain-containing protein [Source:UniProtKB/TrEMBL;Acc:L8GY16]	0.003182593	0.343623	0.340440407	1.89E-08
6h	ACA1_373560/clean/scf7180000084679:100067-100184:-	ACA1_373560	hypothetical protein	0.002473717	0.342854333	0.340380617	2.04E-07
6h	ACA1_096400/clean/scf7180000084645:1005348-1005419:+	ACA1_096400	Methyltransf_11 domain-containing protein [Source:UniProtKB/TrEMBL;Acc:L8GJF1]	0	0.340298333	0.340298333	1.08E-10
6h	ACA1_088880/clean/scf7180000084639:253226-253307:-	ACA1_088880	OGFr_N domain-containing protein [Source:UniProtKB/TrEMBL;Acc:L8GUP7]	0.041666667	0.381950333	0.340283667	0.001308242
6h	ACA1_113540/clean/scf7180000084669:314491-314618:-	ACA1_113540	SCP domain-containing protein [Source:UniProtKB/TrEMBL;Acc:L8H3B1]	0.018007287	0.358254	0.340246713	5.08E-08
6h	ACA1_379850/clean/scf7180000084712:14820-14891:-	ACA1_379850	Isochorismatase domain containing protein 2, mitochondrial, putative [Source:UniProtKB/TrEMBL;Acc:L8GN12]	0.002807075	0.342983333	0.340176259	1.86E-11
6h	ACA1_301230/clean/scf7180000083203:5519-5605:-	ACA1_301230	hypothetical protein	0.004975133	0.345089667	0.340114533	1.98E-06
6h	ACA1_360060/clean/scf7180000084622:39717-39787:-	ACA1_360060	GHMP_kinases_C domain-containing protein [Source:UniProtKB/TrEMBL;Acc:L8HF07]	0.022188433	0.362286	0.340097567	3.12E-08
6h	ACA1_138810/clean/scf7180000084705:14662-14755:-	ACA1_138810	Signal transducer and activator of transcription [Source:UniProtKB/TrEMBL;Acc:L8GN02]	0.022884467	0.362885667	0.3400012	1.74E-14
6h	ACA1_074740/clean/scf7180000084608:75936-76058:-	ACA1_074740	Thioredoxin domain-containing protein [Source:UniProtKB/TrEMBL;Acc:L8HF80]	0	0.339981333	0.339981333	4.11E-11
6h	ACA1_257000/clean/scf7180000084769:121514-121596:+	ACA1_257000	3-hydroxyisobutyryl-CoA hydrolase, mitochondrial [Source:UniProtKB/TrEMBL;Acc:L8GH72]	0.007034054	0.346985667	0.339951612	1.24E-06
6h	ACA1_244850/clean/scf7180000084763:440505-440640:+	ACA1_244850	EF-hand domain-containing protein [Source:UniProtKB/TrEMBL;Acc:L8GK26]	0.014081793	0.353992667	0.339910873	2.93E-07
6h	ACA1_093930/clean/scf7180000084645:680979-681111:+	ACA1_093930	p21Rho-binding domain containing protein [Source:UniProtKB/TrEMBL;Acc:L8GKX1]	0.001532687	0.341422333	0.339889647	1.93E-08
6h	ACA1_215970/clean/scf7180000084758:421425-421589:-	ACA1_215970	EGFlike domain containing protein [Source:UniProtKB/TrEMBL;Acc:L8GQ73]	0.030104467	0.369988333	0.339883867	0.000520955
6h	ACA1_155710/clean/scf7180000084722:223694-223826:-	ACA1_155710	TAF6_C domain-containing protein [Source:UniProtKB/TrEMBL;Acc:L8GZG8]	0.015149487	0.355007667	0.33985818	8.86E-07
6h	ACA1_290520/clean/scf7180000084777:613202-613282:+	ACA1_290520	ABC transporter domain-containing protein [Source:UniProtKB/TrEMBL;Acc:L8HJ82]	0.003243927	0.343020333	0.339776407	1.69E-08
6h	ACA1_178340/clean/scf7180000084743:222348-222490:+	ACA1_178340	RhoGEF domain containing protein [Source:UniProtKB/TrEMBL;Acc:L8GT64]	0.00269847	0.342414333	0.339715863	5.61E-09
6h	ACA1_114860/clean/scf7180000084669:568623-568746:-	ACA1_114860	ANK_REP_REGION domain-containing protein [Source:UniProtKB/TrEMBL;Acc:L8H6N8]	0.003449283	0.343143333	0.33969405	6.73E-08
6h	ACA1_126680/clean/scf7180000084693:76811-76893:+	ACA1_126680	Ras family protein [Source:UniProtKB/TrEMBL;Acc:L8HAC7]	0.002541318	0.342235333	0.339694016	7.54E-18
6h	ACA1_065190/clean/scf7180000084599:1812945-1813028:-	ACA1_065190	RBR-type E3 ubiquitin transferase [Source:UniProtKB/TrEMBL;Acc:L8GXL6]	0.0097111	0.349388667	0.339677567	8.81E-05
6h	ACA1_306840/clean/scf7180000083668:18142-18222:+	ACA1_306840	RTP1_C1 domain-containing protein [Source:UniProtKB/TrEMBL;Acc:L8GQF6]	0.008130067	0.347768	0.339637933	4.62E-05
6h	ACA1_019200/clean/scf7180000083937:13913-13981:-	ACA1_019200	hypothetical protein	0.012891333	0.352479667	0.339588333	0.011608538
6h	ACA1_145790/clean/scf7180000084710:147088-147257:+	ACA1_145790	hypothetical protein	0.005661423	0.345219667	0.339558243	2.46E-14
6h	ACA1_144200/clean/scf7180000084709:170745-170841:+	ACA1_144200	PH domain-containing protein [Source:UniProtKB/TrEMBL;Acc:L8HET7]	0.005088943	0.344613	0.339524057	5.11E-26
6h	ACA1_364720/clean/scf7180000084641:184376-184457:-	ACA1_364720	Sas10/Utp3/C1D family protein [Source:UniProtKB/TrEMBL;Acc:L8GM85]	0.016115417	0.355596	0.339480583	1.10E-08
6h	ACA1_040710/clean/scf7180000084572:57248-57315:+	ACA1_040710	AAA domain-containing protein [Source:UniProtKB/TrEMBL;Acc:L8H0J4]	0.001349527	0.340710667	0.33936114	1.36E-08
6h	ACA1_157530/clean/scf7180000084724:75409-75582:-	ACA1_157530	hypothetical protein	0.015316967	0.354586	0.339269033	5.83E-16
6h	ACA1_006110/clean/scf7180000083029:43274-43520:+	ACA1_006110	GGACT domain-containing protein [Source:UniProtKB/TrEMBL;Acc:L8HGX8]	0.120955333	0.460098333	0.339143	1.67E-06
6h	ACA1_095690/clean/scf7180000084645:885684-885772:+	ACA1_095690	hypothetical protein	0.015313133	0.354433333	0.3391202	8.94E-08
6h	ACA1_282970/clean/scf7180000084776:756337-756415:+	ACA1_282970	DephosphoCoA kinase [Source:UniProtKB/TrEMBL;Acc:L8H9N2]	0.005847967	0.344811667	0.3389637	6.01E-06
6h	ACA1_002620/clean/scf7180000082935:4713-4780:+	ACA1_002620	Fbox domain containing protein [Source:UniProtKB/TrEMBL;Acc:L8HLR9]	0	0.338959	0.338959	2.54E-10
6h	ACA1_245470/clean/scf7180000084763:493760-493847:+	ACA1_245470	4F5 domain-containing protein [Source:UniProtKB/TrEMBL;Acc:L8GK44]	0.003829983	0.342748667	0.338918683	1.51E-27
6h	ACA1_224480/clean/scf7180000084760:209828-209932:+	ACA1_224480	ABC transporter domain-containing protein [Source:UniProtKB/TrEMBL;Acc:L8GSB4]	0.065296133	0.404156667	0.338860533	8.01E-05
6h	ACA1_029390/clean/scf7180000084476:5503-5597:-	ACA1_029390	WD repeat protein [Source:UniProtKB/TrEMBL;Acc:L8GZ57]	0.0669465	0.405770667	0.338824167	5.25E-05
6h	ACA1_201470/clean/scf7180000084753:559589-559680:-	ACA1_201470	Rasrelated protein Rab-2B, putative [Source:UniProtKB/TrEMBL;Acc:L8H2N2]	0.015195417	0.354017667	0.33882225	8.59E-18
6h	ACA1_280510/clean/scf7180000084776:486423-486517:-	ACA1_280510	Peptidase A1 domain-containing protein [Source:UniProtKB/TrEMBL;Acc:L8H8F0]	0.011116537	0.349915	0.338798463	6.00E-07
6h	ACA1_061570/clean/scf7180000084599:963989-964069:-	ACA1_061570	hypothetical protein	0.006130597	0.344829333	0.338698737	2.81E-08
6h	ACA1_325560/clean/scf7180000084495:32979-33123:+	ACA1_325560	Solute carrier family protein [Source:UniProtKB/TrEMBL;Acc:L8H8Z4]	0.002103502	0.340792667	0.338689165	5.86E-10
6h	ACA1_296720/clean/scf7180000084777:1410295-1410452:-	ACA1_296720	Hist_deacetyl domain-containing protein [Source:UniProtKB/TrEMBL;Acc:L8HK34]	0.001092897	0.339735667	0.33864277	9.63E-11
6h	ACA1_377930/clean/scf7180000084711:146238-146345:-	ACA1_377930	hypothetical protein	0.02405456	0.362680333	0.338625773	1.15E-06
6h	ACA1_062430/clean/scf7180000084599:1185515-1185580:-	ACA1_062430	Dual specificity phosphatase, putative [Source:UniProtKB/TrEMBL;Acc:L8GWX5]	0.035837767	0.374366667	0.3385289	4.77E-08
6h	ACA1_126740/clean/scf7180000084693:88147-88241:-	ACA1_126740	hypothetical protein	0.037275824	0.37579	0.338514176	0.000916341
6h	ACA1_392520/clean/scf7180000084740:284330-284448:-	ACA1_392520	hypothetical protein	0.008331064	0.346814667	0.338483603	3.15E-07
6h	ACA1_068500/clean/scf7180000084603:216885-216972:+	ACA1_068500	Eukaryotic family protein [Source:UniProtKB/TrEMBL;Acc:L8HFQ5]	0.007246367	0.345722667	0.3384763	2.49E-07
6h	ACA1_162120/clean/scf7180000084726:34976-35075:+	ACA1_162120	hypothetical protein	0	0.338422667	0.338422667	3.60E-07
6h	ACA1_318550/clean/scf7180000084158:23927-24008:-	ACA1_318550	HECT domain-containing protein [Source:UniProtKB/TrEMBL;Acc:L8GGI5]	0.00303139	0.341314667	0.338283277	1.60E-09
6h	ACA1_201470/clean/scf7180000084753:560005-560114:-	ACA1_201470	Rasrelated protein Rab-2B, putative [Source:UniProtKB/TrEMBL;Acc:L8H2N2]	0.002617879	0.340803	0.338185121	3.00E-11
6h	ACA1_290560/clean/scf7180000084777:626298-626371:+	ACA1_290560	Peptidase_M14 domain-containing protein [Source:UniProtKB/TrEMBL;Acc:L8HLJ4]	0.006003037	0.344018667	0.338015629	1.95E-13
6h	ACA1_309840/clean/scf7180000083767:18264-18347:+	ACA1_309840	1-aminocyclopropane-1-carboxylate deaminase [Source:UniProtKB/TrEMBL;Acc:L8HH90]	0.022682067	0.360575667	0.3378936	4.01E-07

6h	ACA1_368680/clean/scf7180000084664:135825-135919:-	ACA1_368680	PlsC domain-containing protein [Source:UniProtKB/TrEMBL;Acc:L8H1A8]	0.01149593	0.349354667	0.337858737	8.86E-10
6h	ACA1_233420/clean/scf7180000084762:137601-137680:+	ACA1_233420	hypothetical protein	0.001275481	0.339068667	0.337793185	2.56E-25
6h	ACA1_116230/clean/scf7180000084669:731797-731867:+	ACA1_116230	Protein kinase domain containing protein [Source:UniProtKB/TrEMBL;Acc:L8H4M9]	0.007988367	0.345732	0.337743633	1.41E-06
6h	ACA1_052820/clean/scf7180000084594:189025-189126:-	ACA1_052820	hypothetical protein	0.0369781	0.374712667	0.337734567	4.38E-08
6h	ACA1_376490/clean/scf7180000084685:90232-90349:-	ACA1_376490	TIP41like family protein [Source:UniProtKB/TrEMBL;Acc:L8HIG8]	0.027133467	0.364721333	0.337587867	5.01E-10
6h	ACA1_206050/clean/scf7180000084755:71084-71165:+	ACA1_206050	ANK_REP_REGION domain-containing protein [Source:UniProtKB/TrEMBL;Acc:L8H7K6]	0.014473467	0.352031	0.337557533	5.70E-07
6h	ACA1_175470/clean/scf7180000084741:964863-964932:-	ACA1_175470	Endo/exonuclease/phosphatase domain-containing protein [Source:UniProtKB/TrEMBL;Acc:L8HI27]	0	0.337431333	0.337431333	5.37E-08
6h	ACA1_325440/clean/scf7180000084495:8032-8101:+	ACA1_325440	hypothetical protein	0.006930497	0.344354333	0.337423837	3.02E-09
6h	ACA1_035640/clean/scf7180000084564:102748-102824:-	ACA1_035640	Beta-lactamase domain-containing protein [Source:UniProtKB/TrEMBL;Acc:L8H9P2]	0.016025633	0.353355333	0.3373297	0.005179391
6h	ACA1_031800/clean/scf7180000084523:6323-6433:+	ACA1_031800	VWFA domain-containing protein [Source:UniProtKB/TrEMBL;Acc:L8H404]	0	0.337234	0.337234	9.87E-07
6h	ACA1_071690/clean/scf7180000084603:1028965-1029025:+	ACA1_071690	Vacuolar protein sorting factor 4, putative [Source:UniProtKB/TrEMBL;Acc:L8HE96]	0	0.337224667	0.337224667	1.34E-09
6h	ACA1_071130/clean/scf7180000084603:867985-868066:-	ACA1_071130	hypothetical protein	0.014548967	0.351731333	0.337182367	4.95E-05
6h	ACA1_176050/clean/scf7180000084741:1114231-1114292:-	ACA1_176050	Poly [ADP-ribose] polymerase [Source:UniProtKB/TrEMBL;Acc:L8HI82]	0.002089863	0.339269667	0.337179803	1.07E-11
6h	ACA1_377830/clean/scf7180000084711:126742-126846:+	ACA1_377830	UBIQUITIN_CONJUGAT_2 domain-containing protein [Source:UniProtKB/TrEMBL;Acc:L8GSV2]	0.005820583	0.342810667	0.336990083	3.61E-11
6h	ACA1_064540/clean/scf7180000084599:1637914-1638018:-	ACA1_064540	hypothetical protein	0.024206333	0.361185	0.336978667	2.49E-05
6h	ACA1_199970/clean/scf7180000084753:361595-361666:-	ACA1_199970	Glycoside hydrolase family protein [Source:UniProtKB/TrEMBL;Acc:L8H5H5]	0.000647342	0.337446667	0.336799325	1.41E-12
6h	ACA1_002760/clean/scf7180000082937:5959-6025:+	ACA1_002760	Membrane protein, putative [Source:UniProtKB/TrEMBL;Acc:L8GGP6]	0.0537655	0.390483333	0.336717833	2.66E-06
6h	ACA1_371770/clean/scf7180000084664:602527-602618:-	ACA1_371770	Ubiquinone biosynthesis protein COQ4 homolog, mitochondrial [Source:UniProtKB/TrEMBL;Acc:L8GZD5]	0.0091683	0.345839333	0.336671033	1.48E-05
6h	ACA1_296270/clean/scf7180000084777:1308665-1308777:-	ACA1_296270	Deoxyribosephosphate aldolase [Source:UniProtKB/TrEMBL;Acc:L8HIU4]	0.011522797	0.348165	0.336642203	3.65E-14
6h	ACA1_062700/clean/scf7180000084599:1277101-1277164:+	ACA1_062700	ULP_PROTEASE domain-containing protein [Source:UniProtKB/TrEMBL;Acc:L8GZJ7]	0.034674767	0.371287	0.336612233	2.90E-06
6h	ACA1_191530/clean/scf7180000084749:12297-12400:+	ACA1_191530	Pex2_Pex12 domain-containing protein [Source:UniProtKB/TrEMBL;Acc:L8GMZ9]	0.024226387	0.360794667	0.33656828	2.64E-08
6h	ACA1_326070/clean/scf7180000084504:14711-14777:+	ACA1_326070	START domain-containing protein [Source:UniProtKB/TrEMBL;Acc:L8HG48]	0	0.336532667	0.336532667	7.92E-08
6h	ACA1_061150/clean/scf7180000084599:840124-840195:-	ACA1_061150	UBA/TSN domain containing protein [Source:UniProtKB/TrEMBL;Acc:L8GWQ2]	0	0.336506	0.336506	3.22E-08
6h	ACA1_279360/clean/scf7180000084776:371531-371613:-	ACA1_279360	hypothetical protein	0.00207597	0.338409333	0.336333363	3.64E-07
6h	ACA1_031790/clean/scf7180000084523:3273-3410:+	ACA1_031790	Rap-GAP domain-containing protein [Source:UniProtKB/TrEMBL;Acc:L8H1P5]	0.025631667	0.361963	0.336331333	1.33E-07
6h	ACA1_280570/clean/scf7180000084776:499790-499868:+	ACA1_280570	Znfinger in Ran binding protein and others domain containing protein [Source:UniProtKB/TrEMBL;Acc:L8H8F5]	0.017556	0.353843	0.336287	3.03E-05
6h	ACA1_383630/clean/scf7180000084721:234073-234172:+	ACA1_383630	Peptidylprolyl isomerase [Source:UniProtKB/TrEMBL;Acc:L8GV01]	0.00161782	0.337881667	0.336263847	5.10E-16
6h	ACA1_366940/clean/scf7180000084641:519853-519932:-	ACA1_366940	BTB/POZ domain containing protein [Source:UniProtKB/TrEMBL;Acc:L8GPM1]	0	0.336216	0.336216	2.58E-06
6h	ACA1_107160/clean/scf7180000084660:158109-158239:+	ACA1_107160	hypothetical protein	0	0.336177	0.336177	6.59E-11
6h	ACA1_320100/clean/scf7180000084352:6987-7049:+	ACA1_320100	VWFA domain-containing protein [Source:UniProtKB/TrEMBL;Acc:L8H729]	0.00323876	0.339408	0.33616924	3.72E-08
6h	ACA1_140610/clean/scf7180000084707:97399-97496:-	ACA1_140610	Poly [ADP-ribose] polymerase [Source:UniProtKB/TrEMBL;Acc:L8GGG7]	0.024725267	0.360825333	0.336100067	0.000775627
6h	ACA1_043610/clean/scf7180000084573:183936-184024:-	ACA1_043610	FYVE-type domain-containing protein [Source:UniProtKB/TrEMBL;Acc:L8GV86]	0	0.336043	0.336043	1.38E-05
6h	ACA1_175420/clean/scf7180000084741:951379-951465:+	ACA1_175420	hypothetical protein	0.068604533	0.404561	0.335956467	0.003877556
6h	ACA1_107440/clean/scf7180000084660:196719-196824:+	ACA1_107440	Purple acid phosphatase [Source:UniProtKB/TrEMBL;Acc:L8GNJ7]	0.00257488	0.338436333	0.335861454	9.46E-12
6h	ACA1_080670/clean/scf7180000084618:60100-60180:+	ACA1_080670	Non-specific serine/threonine protein kinase [Source:UniProtKB/TrEMBL;Acc:L8HBV7]	0.002554447	0.338363	0.335808553	1.16E-13
6h	ACA1_158530/clean/scf7180000084724:245492-245610:+	ACA1_158530	Glucose-6-phosphate 1-dehydrogenase [Source:UniProtKB/TrEMBL;Acc:L8HBJ1]	0.0923306	0.428115667	0.335785067	0.004021929
6h	ACA1_369160/clean/scf7180000084664:262312-262394:+	ACA1_369160	CYTOSOL_AP domain-containing protein [Source:UniProtKB/TrEMBL;Acc:L8GY57]	0	0.335780333	0.335780333	4.63E-11
6h	ACA1_184430/clean/scf7180000084745:642737-642852:+	ACA1_184430	Solute carrier family 29 (Nucleoside transporters), member 1, putative [Source:UniProtKB/TrEMBL;Acc:L8HAQ2]	0	0.335714	0.335714	1.95E-09
6h	ACA1_238450/clean/scf7180000084763:96471-96564:-	ACA1_238450	TLDc domain-containing protein [Source:UniProtKB/TrEMBL;Acc:L8GM61]	0.018617	0.354266	0.335649	2.49E-09
6h	ACA1_111870/clean/scf7180000084669:52386-52466:-	ACA1_111870	SNF2 family Nterminal domain containing protein [Source:UniProtKB/TrEMBL;Acc:L8H2Z3]	0.000645427	0.336232	0.335586573	6.69E-09
6h	ACA1_056540/clean/scf7180000084597:15291-15388:+	ACA1_056540	B30.2/SPRY domain-containing protein [Source:UniProtKB/TrEMBL;Acc:L8HGP5]	0.139695667	0.475266333	0.335570667	0.006146606
6h	ACA1_063790/clean/scf7180000084599:1504745-1504846:-	ACA1_063790	hypothetical protein	0.0074074	0.342941667	0.335534267	1.17E-05
6h	ACA1_221060/clean/scf7180000084758:989055-989126:-	ACA1_221060	Leucine rich repeat domain containing protein [Source:UniProtKB/TrEMBL;Acc:L8GQQ4]	0.007730533	0.343255667	0.335525133	8.08E-06
6h	ACA1_230440/clean/scf7180000084761:348838-348906:+	ACA1_230440	WD_REPEATS_REGION domain-containing protein [Source:UniProtKB/TrEMBL;Acc:L8H839]	0	0.335520333	0.335520333	2.54E-06
6h	ACA1_151380/clean/scf7180000084716:118354-118413:-	ACA1_151380	hypothetical protein	0.021334667	0.356846333	0.335511667	3.31E-14
6h	ACA1_377380/clean/scf7180000084711:47368-47432:+	ACA1_377380	N-acetyltransferase domain-containing protein [Source:UniProtKB/TrEMBL;Acc:L8GR67]	0.00329102	0.338791333	0.335500313	1.93E-06
6h	ACA1_305380/clean/scf7180000083609:6656-6729:-	ACA1_305380	Myb_DNA-bind_2 domain-containing protein [Source:UniProtKB/TrEMBL;Acc:L8GLW8]	0.00092336	0.336306	0.33538264	7.67E-07
6h	ACA1_184600/clean/scf7180000084746:3319-3394:-	ACA1_184600	Alpha-N-acetylglucosaminidase family protein [Source:UniProtKB/TrEMBL;Acc:L8H6D3]	0	0.335352	0.335352	2.17E-06
6h	ACA1_388780/clean/scf7180000084733:168902-169001:+	ACA1_388780	EGFlike domain containing protein [Source:UniProtKB/TrEMBL;Acc:L8GDT8]	0	0.335339333	0.335339333	4.10E-13
6h	ACA1_221620/clean/scf7180000084760:6813-6911:-	ACA1_221620	Purple acid phosphatase [Source:UniProtKB/TrEMBL;Acc:L8GV66]	0.01008672	0.345402667	0.335315947	4.50E-16
6h	ACA1_355850/clean/scf7180000084728:31588-31649:-	ACA1_355850	Methionine aminopeptidase [Source:UniProtKB/TrEMBL;Acc:L8H6U0]	0.006786813	0.341933333	0.33514652	9.33E-17
6h	ACA1_182120/clean/scf7180000084745:132080-132209:+	ACA1_182120	WD repeat domain 5B, putative [Source:UniProtKB/TrEMBL;Acc:L8HA46]	0.00178597	0.3369	0.33511403	2.10E-07
6h	ACA1_116230/clean/scf7180000084669:732121-732224:+	ACA1_116230	Protein kinase domain containing protein [Source:UniProtKB/TrEMBL;Acc:L8H4M9]	0	0.335079	0.335079	8.87E-08
6h	ACA1_251560/clean/scf7180000084768:84923-85036:+	ACA1_251560	hypothetical protein	0.000551617	0.335616333	0.335064717	1.13E-06
6h	ACA1_320980/clean/scf7180000084435:6131-6280:+	ACA1_320980	RNA recognition motif domain containing protein [Source:UniProtKB/TrEMBL;Acc:L8GR35]	0	0.335001	0.335001	9.30E-07
6h	ACA1_290650/clean/scf7180000084777:647611-647721:+	ACA1_290650	DENN domain-containing protein [Source:UniProtKB/TrEMBL;Acc:L8HI74]	0.089311333	0.424286667	0.334975333	0.002558414
6h	ACA1_037870/clean/scf7180000084568:46898-46989:+	ACA1_037870	Vacuolar protein sorting-associated protein 11 homolog [Source:UniProtKB/TrEMBL;Acc:L8GKP2]	0.000574257	0.335510333	0.334936077	3.42E-08
6h	ACA1_293890/clean/scf7180000084777:987232-987321:+	ACA1_293890	hypothetical protein	0.026124837	0.361053667	0.33492883	3.33E-09
6h	ACA1_268750/clean/scf7180000084770:912240-912428:+	ACA1_268750	Thioredoxin domain-containing protein [Source:UniProtKB/TrEMBL;Acc:L8H1X3]	0.008918093	0.343721	0.334802907	2.31E-10
6h	ACA1_375990/clean/scf7180000084685:23065-23280:-	ACA1_375990	AcetylCoA acetyltransferase, mitochondrial, putative [Source:UniProtKB/TrEMBL;Acc:L8HFY3]	0.003150247	0.337944333	0.334794087	2.34E-08
6h	ACA1_107450/clean/scf7180000084660:199711-199784:-	ACA1_107450	PB1 domain-containing protein [Source:UniProtKB/TrEMBL;Acc:L8GNC1]	0.011627997	0.346401667	0.33477367	2.39E-09
6h	ACA1_233410/clean/scf7180000084762:136540-136622:+	ACA1_233410	NFX1type zinc finger-containing protein 1, putative [Source:UniProtKB/TrEMBL;Acc:L8H2G5]	0.0143713	0.34903	0.3346587	2.86E-25
6h	ACA1_290520/clean/scf7180000084777:612444-612542:+	ACA1_290520	ABC transporter domain-containing protein [Source:UniProtKB/TrEMBL;Acc:L8HJ82]	0	0.334636333	0.334636333	5.71E-10
6h	ACA1_055340/clean/scf7180000084594:686980-687052:+	ACA1_055340	Autophagy related protein 2 (ATG2), putative [Source:UniProtKB/TrEMBL;Acc:L8H7Q9]	0.038549567	0.373178333	0.334628767	7.12E-07
6h	ACA1_220740/clean/scf7180000084758:959233-959410:+	ACA1_220740	Mitochondrial fission 1 protein [Source:UniProtKB/TrEMBL;Acc:L8GRP2]	0.0318089	0.366436333	0.334627433	1.22E-08
6h	ACA1_006190/clean/scf7180000083029:67656-67745:-	ACA1_006190	eIF-5a domain-containing protein [Source:UniProtKB/TrEMBL;Acc:L8HH38]	0.062624367	0.397165333	0.334540967	0.000311907
6h	ACA1_299380/clean/scf7180000083027:11354-11419:+	ACA1_299380	AAA_11 domain-containing protein [Source:UniProtKB/TrEMBL;Acc:L8GS61]	0.0127455	0.347285667	0.334540167	5.60E-27
6h	ACA1_036010/clean/scf7180000084565:17507-17599:+	ACA1_036010	XPG_I_2 domain-containing protein [Source:UniProtKB/TrEMBL;Acc:L8HDT4]	0	0.334501667	0.334501667	3.03E-05
6h	ACA1_288340/clean/scf7180000084777:173904-173984:+	ACA1_288340	Conditioned medium factor, putative [Source:UniProtKB/TrEMBL;Acc:L8HKD6]	0.003553163	0.338024333	0.33447117	1.25E-16
6h	ACA1_123350/clean/scf7180000084686:16563-16641:-	ACA1_123350	hypothetical protein	0.0076687	0.342127333	0.334458633	1.03E-07
6h	ACA1_054860/clean/scf7180000084594:593314-593390:+	ACA1_054860	hypothetical protein	0.0136672	0.348112333	0.334445133	6.77E-05
6h	ACA1_200140/clean/scf7180000084753:404135-404216:+	ACA1_200140	SH3 domain-containing protein [Source:UniProtKB/TrEMBL;Acc:L8H3A8]	0.03013747	0.364575667	0.334438197	3.38E-07
6h	ACA1_071570/clean/scf7180000084603:994874-994944:-	ACA1_071570	hypothetical protein	0.071895333	0.406313	0.334417667	0.009783532
6h	ACA1_295310/clean/scf7180000084777:1103144-1103275:-	ACA1_295310	AP complex subunit sigma [Source:UniProtKB/TrEMBL;Acc:L8HM08]	0.00988822	0.34423	0.33434178	1.37E-14
6h	ACA1_278150/clean/scf7180000084776:180073-180150:-	ACA1_278150	hypothetical protein	0.011130207	0.345435667	0.33430546	1.18E-16
6h	ACA1_327240/clean/scf7180000084518:8806-8876:+	ACA1_327240	TOP4c domain-containing protein [Source:UniProtKB/TrEMBL;Acc:L8GYI5]	0.0229913	0.357229667	0.334238367	2.29E-08
6h	ACA1_006680/clean/scf7180000083091:815-885:+	ACA1_006680	Arginine deiminase [Source:UniProtKB/TrEMBL;Acc:L8H3W4]	0.230783333	0.56497	0.334186667	0.006646374
6h	ACA1_069710/clean/scf7180000084603:533620-533695:+	ACA1_069710	Serine/threonine kinase [Source:UniProtKB/TrEMBL;Acc:L8HG11]	0.016091033	0.350275667	0.334184633	2.25E-08
6h	ACA1_062260/clean/scf7180000084599:1152029-1152106:+	ACA1_062260	C2 domain containing protein [Source:UniProtKB/TrEMBL;Acc:L8GW88]	0.025096507	0.359261667	0.33416516	1.97E-05
6h	ACA1_389630/clean/scf7180000084733:299426-299505:+	ACA1_389630	F-box domain-containing protein [Source:UniProtKB/TrEMBL;Acc:L8GDY9]	0.019879667	0.353843667	0.333964	2.36E-08
6h	ACA1_078530/clean/scf7180000084615:39057-39142:+	ACA1_078530	START domain-containing protein [Source:UniProtKB/TrEMBL;Acc:L8GUK7]	0	0.333884	0.333884	2.27E-13
6h	ACA1_264380/clean/scf7180000084770:255387-255477:-	ACA1_264380	S-formylglutathione hydrolase [Source:UniProtKB/TrEMBL;Acc:L8H1Z7]	1.97E-05	0.333879	0.333859254	7.39E-12
6h	ACA1_263890/clean/scf7180000084770:126878-126974:-	ACA1_263890	Methyltransf_11 domain-containing protein [Source:UniProtKB/TrEMBL;Acc:L8H151]	0.00223547	0.336027	0.33379153	2.53E-07
6h	ACA1_363760/clean/scf7180000084641:8623-8721:-	ACA1_363760	Adenosine specific kinase [Source:UniProtKB/TrEMBL;Acc:L8GP09]	0.297237467	0.631008667	0.3337712	0.0016462
6h	ACA1_279550/clean/scf7180000084776:413156-413262:-	ACA1_279550	CBS domain containing protein [Source:UniProtKB/TrEMBL;Acc:L8H8C1]	0.01340196	0.347113	0.33371104	1.14E-07
6h	ACA1_379300/clean/scf7180000084711:326598-326706:-	ACA1_379300	hypothetical protein	0.008461423	0.342165	0.333703577	4.08E-08
6h	ACA1_086300/clean/scf7180000084636:35201-35286:-	ACA1_086300	Acyltransferase [Source:UniProtKB/TrEMBL;Acc:L8HFT3]	0.013131677	0.346683667	0.33355199	2.35E-06
6h	ACA1_031640/clean/scf7180000084522:34712-34805:-	ACA1_031640	PH domain-containing protein [Source:UniProtKB/TrEMBL;Acc:L8GV44]	0.0062893	0.339684667	0.333395367	1.49E-06
6h	ACA1_279740/clean/scf7180000084776:433856-433934:+	ACA1_279740	BATS domain-containing protein [Source:UniProtKB/TrEMBL;Acc:L8H6X4]	0.00866278	0.342032333	0.333369553	8.80E-07
6h	ACA1_139400/clean/scf7180000084705:73757-73922:+	ACA1_139400	WD domain, Gbeta repeat-containing protein [Source:UniProtKB/TrEMBL;Acc:L8GNA0]	0.012191533	0.345529667	0.333338133	1.14E-05
6h	ACA1_164150/clean/scf7180000084729:171866-171957:-	ACA1_164150	Alpha/beta hydrolase fold domain containing protein [Source:UniProtKB/TrEMBL;Acc:L8GTY4]	0.015805433	0.349096333	0.3332909	4.96E-05
6h	ACA1_298630/clean/scf7180000082992:3419-3543:-	ACA1_298630	Vacuolar protein sorting 16, putative [Source:UniProtKB/TrEMBL;Acc:L8H081]	0.004848	0.338023667	0.333175667	2.45E-09
6h	ACA1_264380/clean/scf7180000084770:256205-256288:-	ACA1_264380	S-formylglutathione hydrolase [Source:UniProtKB/TrEMBL;Acc:L8H1Z7]	0.003801497	0.336911333	0.333109837	1.72E-09
6h	ACA1_364570/clean/scf7180000084641:145145-145293:+	ACA1_364570	hypothetical protein	0.00699997	0.340077333	0.333077363	1.38E-07
6h	ACA1_204530/clean/scf7180000084754:305340-305423:-	ACA1_204530	F-box domain-containing protein [Source:UniProtKB/TrEMBL;Acc:L8GTM3]	0.366666667	0.699740333	0.333073667	0.003730995
6h	ACA1_131500/clean/scf7180000084699:70720-70797:+	ACA1_131500	Mediator of RNA polymerase II transcription subunit 31 [Source:UniProtKB/TrEMBL;Acc:L8GMF8]	0.0233971	0.356348	0.3329509	1.17E-07
6h	ACA1_251640/clean/scf7180000084768:98848-98950:+	ACA1_251640	CBS domain-containing protein [Source:UniProtKB/TrEMBL;Acc:L8HAQ8]	0	0.332913667	0.332913667	2.21E-08
6h	ACA1_051530/clean/scf7180000084592:29744-29840:-	ACA1_051530	MFS domain-containing protein [Source:UniProtKB/TrEMBL;Acc:L8GPJ8]	0	0.33291	0.33291	1.45E-08
6h	ACA1_115740/clean/scf7180000084669:659434-659533:+	ACA1_115740	3Beta_HSD domain-containing protein [Source:UniProtKB/TrEMBL;Acc:L8H3S6]	0.0002772	0.333147	0.3328698	9.52E-12
6h	ACA1_164720/clean/scf7180000084729:259081-259180:+	ACA1_164720	Autophagy-related protein 9 [Source:UniProtKB/TrEMBL;Acc:L8GU30]	0.000506407	0.333313333	0.332806927	2.45E-09
6h	ACA1_381570/clean/scf7180000084712:184501-184559:+	ACA1_381570	Ras-GAP domain-containing protein [Source:UniProtKB/TrEMBL;Acc:L8GMY5]	0.054537667	0.387270333	0.332732667	1.45E-09
6h	ACA1_295300/clean/scf7180000084777:1100782-1100965:-	ACA1_295300	Calponin domain containing protein [Source:UniProtKB/TrEMBL;Acc:L8HJQ6]	0.01727085	0.349973	0.33270215	1.00E-07
6h	ACA1_123500/clean/scf7180000084686:32796-32893:+	ACA1_123500	hypothetical protein	0.019325767	0.352024667	0.3326989	0.000132552
6h	ACA1_378170/clean/scf7180000084711:201471-201558:-	ACA1_378170	G_PROTEIN_RECEP_F1_2 domain-containing protein [Source:UniProtKB/TrEMBL;Acc:L8GRC2]	0.00979314	0.342487	0.33269386	7.08E-08
6h	ACA1_214970/clean/scf7180000084758:282850-282959:-	ACA1_214970	SNF2 family Nterminal domain containing protein [Source:UniProtKB/TrEMBL;Acc:L8GR02]	0.020111443	0.352597667	0.332486223	0.000152118
6h	ACA1_069380/clean/scf7180000084603:423907-424011:+	ACA1_069380	LNS2 domain-containing protein [Source:UniProtKB/TrEMBL;Acc:L8HCT1]	0.000133761	0.332616333	0.332482572	5.34E-07
6h	ACA1_245660/clean/scf7180000084763:516351-516489:-	ACA1_245660	hypothetical protein	0.112504	0.444952333	0.332448333	6.03E-06
6h	ACA1_025570/clean/scf7180000084448:16070-16149:+	ACA1_025570	hypothetical protein	0.000735786	0.333164667	0.332428881	1.40E-12
6h	ACA1_068740/clean/scf7180000084603:267386-267468:+	ACA1_068740	tRNAdihydrouridine synthase A, putative [Source:UniProtKB/TrEMBL;Acc:L8HDM1]	0.030303033	0.362676333	0.3323733	0.002053967
6h	ACA1_109770/clean/scf7180000084663:22620-22713:+	ACA1_109770	WD40 repeat-containing protein [Source:UniProtKB/TrEMBL;Acc:L8HKT6]	0.007810763	0.340126	0.332315237	2.68E-13
6h	ACA1_143670/clean/scf7180000084709:67636-67770:-	ACA1_143670	Ribosomal_S10 domain-containing protein [Source:UniProtKB/TrEMBL;Acc:L8HFG4]	0.004876177	0.337186667	0.33231049	4.66E-14
6h	ACA1_144220/clean/scf7180000084709:174191-174322:-	ACA1_144220	hypothetical protein	0.001123173	0.33341	0.332286827	8.49E-06
6h	ACA1_315820/clean/scf7180000083943:14459-14606:+	ACA1_315820	hypothetical protein	0.00022273	0.332482	0.33225927	9.83E-10
6h	ACA1_013340/clean/scf7180000083750:2258-2321:+	ACA1_013340	PH domain-containing protein [Source:UniProtKB/TrEMBL;Acc:L8HGJ7]	0.021873133	0.354112	0.332238867	1.08E-11
6h	ACA1_006200/clean/scf7180000083029:68953-69040:-	ACA1_006200	hypothetical protein	0.004870353	0.337085333	0.33221498	8.50E-07
6h	ACA1_210530/clean/scf7180000084758:49657-49753:-	ACA1_210530	4Fe-4S ferredoxin-type domain-containing protein [Source:UniProtKB/TrEMBL;Acc:L8GS75]	0.013019803	0.345219333	0.33219953	1.08E-09
6h	ACA1_072810/clean/scf7180000084603:1302045-1302194:+	ACA1_072810	Aha1_N domain-containing protein [Source:UniProtKB/TrEMBL;Acc:L8HG06]	0.004038337	0.336137333	0.332098997	1.66E-23
6h	ACA1_174630/clean/scf7180000084741:751856-751961:+	ACA1_174630	Aminotran_1_2 domain-containing protein [Source:UniProtKB/TrEMBL;Acc:L8HHW2]	0.000330688	0.332293	0.331962312	9.40E-10
6h	ACA1_202460/clean/scf7180000084754:53442-53528:+	ACA1_202460	MADS-box domain-containing protein [Source:UniProtKB/TrEMBL;Acc:L8GSU5]	0.013669833	0.345605	0.331935167	0.000880151
6h	ACA1_207490/clean/scf7180000084757:69893-69965:-	ACA1_207490	WD_REPEATS_REGION domain-containing protein [Source:UniProtKB/TrEMBL;Acc:L8GY92]	0	0.331930667	0.331930667	3.96E-07
6h	ACA1_264380/clean/scf7180000084770:255714-255791:-	ACA1_264380	S-formylglutathione hydrolase [Source:UniProtKB/TrEMBL;Acc:L8H1Z7]	0.002222617	0.334044333	0.331821717	5.00E-11
6h	ACA1_166080/clean/scf7180000084732:114580-114659:+	ACA1_166080	hypothetical protein	0	0.331813	0.331813	9.68E-11
6h	ACA1_109740/clean/scf7180000084663:14195-14268:-	ACA1_109740	Myblike DNA-binding domain containing protein [Source:UniProtKB/TrEMBL;Acc:L8HJY9]	0.0134966	0.345289333	0.331792733	3.56E-07
6h	ACA1_278070/clean/scf7180000084776:166103-166171:-	ACA1_278070	RBR-type E3 ubiquitin transferase [Source:UniProtKB/TrEMBL;Acc:L8H8S7]	0.1050624	0.436840333	0.331777933	4.93E-06
6h	ACA1_385120/clean/scf7180000084730:138677-138748:+	ACA1_385120	Non-specific serine/threonine protein kinase [Source:UniProtKB/TrEMBL;Acc:L8HBH8]	0.000420995	0.332184667	0.331763672	5.04E-13
6h	ACA1_189410/clean/scf7180000084748:31340-31423:+	ACA1_189410	BTB/POZ domain containing protein [Source:UniProtKB/TrEMBL;Acc:L8GGG2]	0.014432163	0.346127667	0.331695503	4.62E-05
6h	ACA1_176060/clean/scf7180000084741:1116941-1117028:+	ACA1_176060	DAGKc domain-containing protein [Source:UniProtKB/TrEMBL;Acc:L8HJR7]	0.050912633	0.382532667	0.331620033	1.35E-07
6h	ACA1_117240/clean/scf7180000084669:959269-959342:-	ACA1_117240	Non-specific serine/threonine protein kinase [Source:UniProtKB/TrEMBL;Acc:L8H707]	0.002470036	0.334010333	0.331540298	4.18E-09
6h	ACA1_087950/clean/scf7180000084639:140095-140174:+	ACA1_087950	Thg1 domain-containing protein [Source:UniProtKB/TrEMBL;Acc:L8GUE5]	0.000947643	0.332456	0.331508357	1.73E-08
6h	ACA1_367310/clean/scf7180000084641:597531-597605:-	ACA1_367310	CCHC-type domain-containing protein [Source:UniProtKB/TrEMBL;Acc:L8GMZ2]	0.013047833	0.344483333	0.3314355	5.70E-07
6h	ACA1_041970/clean/scf7180000084573:28614-28699:+	ACA1_041970	hypothetical protein	0.020954133	0.352325	0.331370867	0.003831691
6h	ACA1_248410/clean/scf7180000084766:62200-62278:-	ACA1_248410	Alpha2-macroglobulin domain containing protein [Source:UniProtKB/TrEMBL;Acc:L8GY08]	0	0.331344333	0.331344333	2.14E-09
6h	ACA1_369750/clean/scf7180000084664:373493-373566:+	ACA1_369750	RRM domain-containing protein [Source:UniProtKB/TrEMBL;Acc:L8GZ60]	0.019705853	0.350932333	0.33122648	1.58E-07
6h	ACA1_222650/clean/scf7180000084760:44553-44681:-	ACA1_222650	hypothetical protein	0.016767047	0.347987	0.331219953	2.80E-06
6h	ACA1_162510/clean/scf7180000084726:51018-51118:+	ACA1_162510	G_PROTEIN_RECEP_F2_4 domain-containing protein [Source:UniProtKB/TrEMBL;Acc:L8H134]	0.011444933	0.342607	0.331162067	0.0006732
6h	ACA1_245660/clean/scf7180000084763:515695-515807:-	ACA1_245660	hypothetical protein	0.0940226	0.424963	0.3309404	4.81E-05
6h	ACA1_069920/clean/scf7180000084603:600765-600853:-	ACA1_069920	Phospholipid-transporting ATPase [Source:UniProtKB/TrEMBL;Acc:L8HDU8]	0	0.330897667	0.330897667	3.47E-07
6h	ACA1_201490/clean/scf7180000084753:565305-565399:+	ACA1_201490	hypothetical protein	0.008890467	0.339762667	0.3308722	3.33E-07

6h	ACA1_215600/clean/scf7180000084758:345624-345694:-	ACA1_215600	hypothetical protein	0.06043692	0.391303667	0.330866747	0.002727348
6h	ACA1_186050/clean/scf7180000084746:156675-156743:+	ACA1_186050	GAF domain containing protein [Source:UniProtKB/TrEMBL;Acc:L8H498]	0.000281091	0.331144	0.330862909	5.74E-11
6h	ACA1_128820/clean/scf7180000084697:102961-103019:-	ACA1_128820	Leucine rich repeat domain containing protein [Source:UniProtKB/TrEMBL;Acc:L8GVD0]	0	0.330750333	0.330750333	9.35E-07
6h	ACA1_380930/clean/scf7180000084712:118788-118863:-	ACA1_380930	hypothetical protein	0.008107433	0.338854667	0.330747233	1.98E-07
6h	ACA1_341060/clean/scf7180000084620:41362-41440:-	ACA1_341060	RhoGAP domain containing protein [Source:UniProtKB/TrEMBL;Acc:L8GTM9]	0.047932933	0.378672333	0.3307394	3.60E-09
6h	ACA1_182150/clean/scf7180000084745:141640-141726:+	ACA1_182150	hypothetical protein	0.0644225	0.395079333	0.330656833	2.27E-05
6h	ACA1_341080/clean/scf7180000084620:46699-46785:+	ACA1_341080	RING-type domain-containing protein [Source:UniProtKB/TrEMBL;Acc:L8GS99]	0.0147995	0.345383	0.3305835	4.46E-09
6h	ACA1_297760/clean/scf7180000082853:3740-3815:+	ACA1_297760	hypothetical protein	0.003585965	0.334154667	0.330568701	1.03E-11
6h	ACA1_265380/clean/scf7180000084770:455824-455907:-	ACA1_265380	Phosphatidylinositol glycan anchor biosynthesis, class S, putative [Source:UniProtKB/TrEMBL;Acc:L8H3M4]	0.011657623	0.342204333	0.33054671	1.90E-06
6h	ACA1_186870/clean/scf7180000084746:214400-214497:+	ACA1_186870	Protein kinase domain-containing protein [Source:UniProtKB/TrEMBL;Acc:L8H6J9]	0.012845243	0.343379	0.330533757	3.82E-08
6h	ACA1_109770/clean/scf7180000084663:20833-20987:+	ACA1_109770	WD40 repeat-containing protein [Source:UniProtKB/TrEMBL;Acc:L8HKT6]	0.001218803	0.331707	0.330488197	3.79E-10
6h	ACA1_264870/clean/scf7180000084770:393143-393260:+	ACA1_264870	Peptide deformylase [Source:UniProtKB/TrEMBL;Acc:L8H3K8]	0.087663233	0.418050667	0.330387433	4.73E-06
6h	ACA1_087350/clean/scf7180000084639:12629-12707:+	ACA1_087350	Protein prenyltransferase, putative [Source:UniProtKB/TrEMBL;Acc:L8GVD3]	0	0.330343333	0.330343333	4.96E-05
6h	ACA1_315600/clean/scf7180000083939:3645-3749:-	ACA1_315600	hypothetical protein	0.006941047	0.337095333	0.330154287	2.71E-17
6h	ACA1_119140/clean/scf7180000084672:19998-20114:-	ACA1_119140	hypothetical protein	0.008615733	0.338769333	0.3301536	2.57E-05
6h	ACA1_020880/clean/scf7180000084095:31114-31212:+	ACA1_020880	Protozoan/cyanobacterial globin family subfamily protein [Source:UniProtKB/TrEMBL;Acc:L8GU16]	0.050786167	0.380920667	0.3301345	5.44E-12
6h	ACA1_066920/clean/scf7180000084600:83127-83204:+	ACA1_066920	Protein kinase domain-containing protein [Source:UniProtKB/TrEMBL;Acc:L8GRA7]	0	0.330086667	0.330086667	5.97E-09
6h	ACA1_228650/clean/scf7180000084761:185158-185241:-	ACA1_228650	hypothetical protein	0.000416393	0.33044	0.330023607	4.41E-10
6h	ACA1_228910/clean/scf7180000084761:221604-221704:+	ACA1_228910	hypothetical protein	0	0.329995	0.329995	4.68E-09
6h	ACA1_162500/clean/scf7180000084726:49146-49243:-	ACA1_162500	31.5 kDa midgut protein [Source:UniProtKB/TrEMBL;Acc:L8GYK7]	0.009790067	0.339759333	0.329969267	1.94E-06
6h	ACA1_155500/clean/scf7180000084722:176554-176676:-	ACA1_155500	unspecified product	0	0.329964333	0.329964333	1.74E-07
6h	ACA1_201480/clean/scf7180000084753:560737-560807:+	ACA1_201480	PKS_ER domain-containing protein [Source:UniProtKB/TrEMBL;Acc:L8H3J6]	0.057052267	0.386967333	0.329915067	4.83E-11
6h	ACA1_279560/clean/scf7180000084776:414745-414813:-	ACA1_279560	Iron hydrogenase assembly protein [Source:UniProtKB/TrEMBL;Acc:L8H633]	0.300024	0.629852	0.329828	6.63E-05
6h	ACA1_060930/clean/scf7180000084599:782291-782460:+	ACA1_060930	RFX-type winged-helix domain-containing protein [Source:UniProtKB/TrEMBL;Acc:L8GWH8]	0.031188767	0.360792667	0.3296039	7.32E-10
6h	ACA1_366690/clean/scf7180000084641:481294-481391:+	ACA1_366690	Aspartyl aminopeptidase [Source:UniProtKB/TrEMBL;Acc:L8GM39]	0.002973686	0.332547	0.329573314	1.12E-10
6h	ACA1_106280/clean/scf7180000084660:68813-68940:-	ACA1_106280	hypothetical protein	0.004341763	0.333870667	0.329528903	3.01E-18
6h	ACA1_092590/clean/scf7180000084645:361941-362013:-	ACA1_092590	Leucine rich repeat domain containing protein [Source:UniProtKB/TrEMBL;Acc:L8GKK8]	0.017543867	0.347061667	0.3295178	0.000147188
6h	ACA1_264710/clean/scf7180000084770:363623-363766:-	ACA1_264710	Peptidase_S8 domain-containing protein [Source:UniProtKB/TrEMBL;Acc:L8H251]	0.006083897	0.335591	0.329507103	1.10E-07
6h	ACA1_052610/clean/scf7180000084594:123358-123437:+	ACA1_052610	hypothetical protein	0.0308345	0.360312667	0.329478167	2.29E-12
6h	ACA1_074160/clean/scf7180000084605:39260-39355:+	ACA1_074160	Aamy domain-containing protein [Source:UniProtKB/TrEMBL;Acc:L8HJ75]	0.00304991	0.332356	0.32930609	2.80E-10
6h	ACA1_376500/clean/scf7180000084685:92929-93011:+	ACA1_376500	Iso_dh domain-containing protein [Source:UniProtKB/TrEMBL;Acc:L8HG04]	0.017012903	0.346311333	0.32929843	4.50E-13
6h	ACA1_224500/clean/scf7180000084760:218475-218568:-	ACA1_224500	Phospholipid-transporting ATPase [Source:UniProtKB/TrEMBL;Acc:L8GVH2]	0.000891267	0.330176667	0.3292854	5.29E-10
6h	ACA1_244810/clean/scf7180000084763:427580-427662:-	ACA1_244810	tRNA pseudouridine synthase [Source:UniProtKB/TrEMBL;Acc:L8GMI6]	0.01707813	0.346208333	0.329130203	6.67E-05
6h	ACA1_017740/clean/scf7180000083876:1127-1221:+	ACA1_017740	5'-3' exoribonuclease 1 [Source:UniProtKB/TrEMBL;Acc:L8GMR4]	0	0.329020333	0.329020333	2.40E-10
6h	ACA1_092000/clean/scf7180000084645:244151-244261:-	ACA1_092000	hypothetical protein	0.015585517	0.344602	0.329016483	4.47E-07
6h	ACA1_321440/clean/scf7180000084439:31178-31266:+	ACA1_321440	hypothetical protein	0.008320565	0.337242	0.328921435	6.41E-12
6h	ACA1_285340/clean/scf7180000084776:967531-967606:-	ACA1_285340	J domain-containing protein [Source:UniProtKB/TrEMBL;Acc:L8H7S6]	0.011284133	0.340194333	0.3289102	3.42E-07
6h	ACA1_400210/clean/scf7180000084775:120248-120333:-	ACA1_400210	Non-specific serine/threonine protein kinase [Source:UniProtKB/TrEMBL;Acc:L8GIE6]	0.004694833	0.333603	0.328908167	1.53E-08
6h	ACA1_296110/clean/scf7180000084777:1298314-1298423:-	ACA1_296110	MORN repeat variantcontaining protein [Source:UniProtKB/TrEMBL;Acc:L8HLH8]	0.004467277	0.333331	0.328863723	1.39E-28
6h	ACA1_309340/clean/scf7180000083758:6495-6559:+	ACA1_309340	Protein tyrosine phosphataselike protein, PTPLA [Source:UniProtKB/TrEMBL;Acc:L8GR24]	0	0.328823333	0.328823333	9.45E-10
6h	ACA1_074700/clean/scf7180000084608:65750-65851:-	ACA1_074700	hypothetical protein	0.00437882	0.333127667	0.328748847	6.80E-07
6h	ACA1_183350/clean/scf7180000084745:383835-383945:+	ACA1_183350	Short chain dehydrogenase/reductase family [Source:UniProtKB/TrEMBL;Acc:L8HAD9]	0.001831503	0.330571	0.328739497	1.18E-08
6h	ACA1_165280/clean/scf7180000084732:25500-25580:-	ACA1_165280	PH domain containing protein [Source:UniProtKB/TrEMBL;Acc:L8HI11]	0.013323133	0.342061667	0.328738533	1.08E-06
6h	ACA1_107160/clean/scf7180000084660:157873-157970:+	ACA1_107160	hypothetical protein	0	0.328530333	0.328530333	5.61E-11
6h	ACA1_265460/clean/scf7180000084770:485167-485239:-	ACA1_265460	Dolichyl-diphosphooligosaccharide--protein glycosyltransferase subunit 2 [Source:UniProtKB/TrEMBL;Acc:L8H4E5]	0.002100661	0.330623667	0.328523006	2.11E-11
6h	ACA1_174230/clean/scf7180000084741:652526-652596:+	ACA1_174230	Rab5interacting protein (Rab5ip) [Source:UniProtKB/TrEMBL;Acc:L8HJ90]	0.000561167	0.328921667	0.3283605	1.51E-13
6h	ACA1_261390/clean/scf7180000084769:657038-657158:+	ACA1_261390	BTB domain-containing protein [Source:UniProtKB/TrEMBL;Acc:L8GFT7]	0.013011248	0.341238667	0.328227419	9.35E-08
6h	ACA1_077100/clean/scf7180000084612:236568-236660:-	ACA1_077100	Rab1/RabDfamily small GTPase [Source:UniProtKB/TrEMBL;Acc:L8GNN9]	0.013464267	0.341631333	0.328167067	1.82E-07
6h	ACA1_173160/clean/scf7180000084741:414877-414975:+	ACA1_173160	hypothetical protein	0.041277067	0.369442667	0.3281656	1.47E-07
6h	ACA1_253380/clean/scf7180000084768:289028-289095:+	ACA1_253380	EF hand domain containing protein [Source:UniProtKB/TrEMBL;Acc:L8HAR9]	0.0211862	0.349314333	0.328128133	1.08E-16
6h	ACA1_121560/clean/scf7180000084682:60018-60159:+	ACA1_121560	Aminopeptidase N, putative [Source:UniProtKB/TrEMBL;Acc:L8GFD6]	0.005970817	0.334024	0.328053183	2.57E-07
6h	ACA1_369160/clean/scf7180000084664:263263-263328:+	ACA1_369160	CYTOSOL_AP domain-containing protein [Source:UniProtKB/TrEMBL;Acc:L8GY57]	6.56E-05	0.328117	0.328051375	2.34E-12
6h	ACA1_113540/clean/scf7180000084669:313580-313658:-	ACA1_113540	SCP domain-containing protein [Source:UniProtKB/TrEMBL;Acc:L8H3B1]	0.016810167	0.344829333	0.328019167	4.91E-11
6h	ACA1_012970/clean/scf7180000083742:13707-13796:+	ACA1_012970	CBS domain containing protein [Source:UniProtKB/TrEMBL;Acc:L8H720]	0.002393777	0.330406667	0.32801289	1.48E-05
6h	ACA1_058300/clean/scf7180000084599:292124-292211:+	ACA1_058300	DH domain-containing protein [Source:UniProtKB/TrEMBL;Acc:L8GVE4]	0.00318147	0.331076667	0.327895197	3.20E-08
6h	ACA1_259540/clean/scf7180000084769:368402-368499:-	ACA1_259540	SNF2 family Nterminal domain containing protein [Source:UniProtKB/TrEMBL;Acc:L8GEZ6]	0.004444433	0.332274	0.327829567	1.07E-06
6h	ACA1_313740/clean/scf7180000083824:6465-6557:-	ACA1_313740	F-box domain-containing protein [Source:UniProtKB/TrEMBL;Acc:L8GMG9]	0.004891003	0.332716	0.327824997	1.91E-08
6h	ACA1_276460/clean/scf7180000084774:185222-185330:+	ACA1_276460	unspecified product	0.01177308	0.339507333	0.327734253	2.12E-09
6h	ACA1_152930/clean/scf7180000084720:38286-38375:-	ACA1_152930	PG_binding_1 domain-containing protein [Source:UniProtKB/TrEMBL;Acc:L8HF34]	0.0034678	0.331166333	0.327698533	6.69E-06
6h	ACA1_260220/clean/scf7180000084769:460002-460060:+	ACA1_260220	Cytochrome b561 domain-containing protein [Source:UniProtKB/TrEMBL;Acc:L8GG14]	0.054998233	0.382666	0.327667767	4.60E-07
6h	ACA1_039340/clean/scf7180000084569:28955-29070:-	ACA1_039340	hypothetical protein	0.033528667	0.361127	0.327598333	1.15E-05
6h	ACA1_256410/clean/scf7180000084769:27394-27464:+	ACA1_256410	ATPase, AAA domain containing protein [Source:UniProtKB/TrEMBL;Acc:L8GER0]	0.001383127	0.328979333	0.327596207	2.55E-05
6h	ACA1_182940/clean/scf7180000084745:293616-293687:+	ACA1_182940	Myblike DNA-binding domain containing protein [Source:UniProtKB/TrEMBL;Acc:L8H867]	0	0.327594333	0.327594333	5.04E-09
6h	ACA1_164660/clean/scf7180000084729:245014-245087:-	ACA1_164660	Protein tyrosine phosphatase, dual specificity, putative [Source:UniProtKB/TrEMBL;Acc:L8GU25]	0.01214547	0.339739333	0.327593863	1.28E-16
6h	ACA1_282190/clean/scf7180000084776:622904-623031:-	ACA1_282190	Synapsin, ATP binding domain containing protein [Source:UniProtKB/TrEMBL;Acc:L8H7E1]	0.004115233	0.331581	0.327465767	1.08E-07
6h	ACA1_064540/clean/scf7180000084599:1638097-1638240:-	ACA1_064540	hypothetical protein	0.0055453	0.332974667	0.327429367	6.05E-05
6h	ACA1_389080/clean/scf7180000084733:192146-192245:-	ACA1_389080	Fbox domain containing protein [Source:UniProtKB/TrEMBL;Acc:L8GDU7]	0.0115942	0.338956333	0.327362133	2.52E-05
6h	ACA1_307250/clean/scf7180000083680:1413-1548:-	ACA1_307250	Mss4 protein [Source:UniProtKB/TrEMBL;Acc:L8H520]	0.089167333	0.416513333	0.327346	1.05E-06
6h	ACA1_033610/clean/scf7180000084559:58769-58849:+	ACA1_033610	Sortilin [Source:UniProtKB/TrEMBL;Acc:L8GHJ0]	0.000226808	0.327565	0.327338192	1.56E-12
6h	ACA1_289740/clean/scf7180000084777:454108-454202:+	ACA1_289740	Rho-GAP domain-containing protein [Source:UniProtKB/TrEMBL;Acc:L8HLF4]	0.010621467	0.337953333	0.327331867	4.69E-06
6h	ACA1_089980/clean/scf7180000084639:410147-410215:-	ACA1_089980	hypothetical protein	0	0.327220667	0.327220667	3.34E-06
6h	ACA1_377950/clean/scf7180000084711:150463-150540:-	ACA1_377950	hypothetical protein	0.001713307	0.328873667	0.32716036	1.40E-07
6h	ACA1_352050/clean/scf7180000084695:42556-42645:+	ACA1_352050	Succinate dehydrogenase [ubiquinone] iron-sulfur subunit, mitochondrial [Source:UniProtKB/TrEMBL;Acc:L8GDM2]	0.020166067	0.347310333	0.327144267	0.001154876
6h	ACA1_215970/clean/scf7180000084758:418028-418157:-	ACA1_215970	EGFlike domain containing protein [Source:UniProtKB/TrEMBL;Acc:L8GQ73]	0.006904333	0.333993333	0.327089	5.80E-05
6h	ACA1_109800/clean/scf7180000084663:32079-32196:-	ACA1_109800	WD_REPEATS_REGION domain-containing protein [Source:UniProtKB/TrEMBL;Acc:L8HHD3]	0.007343993	0.334417	0.327073007	2.47E-08
6h	ACA1_198970/clean/scf7180000084753:180606-180664:-	ACA1_198970	Viral Atype inclusion protein, putative [Source:UniProtKB/TrEMBL;Acc:L8H376]	0	0.327022667	0.327022667	3.67E-07
6h	ACA1_184670/clean/scf7180000084746:30388-30467:+	ACA1_184670	B30.2/SPRY domain-containing protein [Source:UniProtKB/TrEMBL;Acc:L8H461]	0.007542933	0.334520333	0.3269774	1.28E-06
6h	ACA1_140580/clean/scf7180000084707:90027-90160:-	ACA1_140580	SAM domain (Sterile alpha motif) domain containing protein [Source:UniProtKB/TrEMBL;Acc:L8GGR6]	0.005854027	0.332797333	0.326943307	6.14E-07
6h	ACA1_288370/clean/scf7180000084777:179314-179407:+	ACA1_288370	RasGEF domain containing protein [Source:UniProtKB/TrEMBL;Acc:L8HL79]	0.023331533	0.350267	0.326935467	1.95E-13
6h	ACA1_240070/clean/scf7180000084763:196478-196549:-	ACA1_240070	Carboxylic ester hydrolase [Source:UniProtKB/TrEMBL;Acc:L8GJU3]	0.000231385	0.327166333	0.326934948	8.77E-11
6h	ACA1_028650/clean/scf7180000084473:1125-1224:-	ACA1_028650	Peroxisomal biogenesis protein Pex19, putative [Source:UniProtKB/TrEMBL;Acc:L8GTT0]	0.003745333	0.330643333	0.326898	2.33E-07
6h	ACA1_277750/clean/scf7180000084776:92166-92253:-	ACA1_277750	Raslike protein 1, putative [Source:UniProtKB/TrEMBL;Acc:L8H8P6]	0.00330014	0.330146	0.32684586	1.07E-24
6h	ACA1_144300/clean/scf7180000084709:197578-197695:-	ACA1_144300	RRM domain-containing protein [Source:UniProtKB/TrEMBL;Acc:L8HEU9]	0.000450567	0.327261333	0.326810767	1.44E-10
6h	ACA1_192020/clean/scf7180000084749:76837-76939:+	ACA1_192020	Cystathionine gammasynthase [Source:UniProtKB/TrEMBL;Acc:L8GNB3]	0.00504096	0.33179	0.32674904	6.71E-22
6h	ACA1_063800/clean/scf7180000084599:1507134-1507219:-	ACA1_063800	B30.2/SPRY domain-containing protein [Source:UniProtKB/TrEMBL;Acc:L8GZV1]	0.004853117	0.331590333	0.326737217	1.52E-07
6h	ACA1_263870/clean/scf7180000084770:123350-123468:-	ACA1_263870	MAGE domain-containing protein [Source:UniProtKB/TrEMBL;Acc:L8H1Y1]	0.053609033	0.380342667	0.326733633	7.36E-07
6h	ACA1_091560/clean/scf7180000084645:150319-150426:+	ACA1_091560	UBP-type domain-containing protein [Source:UniProtKB/TrEMBL;Acc:L8GKE3]	0.0138889	0.340601667	0.326712767	0.000111086
6h	ACA1_175870/clean/scf7180000084741:1080567-1080666:-	ACA1_175870	Zinc finger, C3HC4 type (RING finger) domain containing protein [Source:UniProtKB/TrEMBL;Acc:L8HI76]	0.00212653	0.328826333	0.326699803	1.34E-06
6h	ACA1_396260/clean/scf7180000084759:66015-66111:+	ACA1_396260	hypothetical protein	0.00389839	0.330500333	0.326601943	9.60E-08
6h	ACA1_216320/clean/scf7180000084758:468241-468353:+	ACA1_216320	Homogentisate 1,2dioxygenase [Source:UniProtKB/TrEMBL;Acc:L8GQ83]	0.003940257	0.330495667	0.32655541	6.29E-13
6h	ACA1_295630/clean/scf7180000084777:1184529-1184623:-	ACA1_295630	RanBP2-type domain-containing protein [Source:UniProtKB/TrEMBL;Acc:L8HJU1]	0.175373	0.501923333	0.326550333	0.002906752
6h	ACA1_371740/clean/scf7180000084664:598375-598488:-	ACA1_371740	Cir_N domain-containing protein [Source:UniProtKB/TrEMBL;Acc:L8GYG4]	0.005046467	0.331593	0.326546533	1.11E-05
6h	ACA1_095560/clean/scf7180000084645:847586-847717:+	ACA1_095560	Protein pelota homolog [Source:UniProtKB/TrEMBL;Acc:L8GL32]	0.000675253	0.327142333	0.32646708	2.24E-07
6h	ACA1_313720/clean/scf7180000083824:1094-1166:+	ACA1_313720	VWFA domain-containing protein [Source:UniProtKB/TrEMBL;Acc:L8GND4]	0.033163367	0.359621667	0.3264583	4.47E-11
6h	ACA1_369270/clean/scf7180000084664:286917-286969:+	ACA1_369270	Rasrelated protein Rab-18-like, putative [Source:UniProtKB/TrEMBL;Acc:L8GYY7]	0.006704133	0.333135333	0.3264312	3.69E-10
6h	ACA1_048880/clean/scf7180000084583:10720-10818:+	ACA1_048880	LIM zinc-binding domain-containing protein [Source:UniProtKB/TrEMBL;Acc:L8HKD0]	0.01621497	0.342615	0.32640003	0.000845677
6h	ACA1_352400/clean/scf7180000084704:9684-9767:-	ACA1_352400	Fn3_like domain-containing protein [Source:UniProtKB/TrEMBL;Acc:L8GTX5]	0.003678437	0.330071	0.326392563	3.91E-12
6h	ACA1_050510/clean/scf7180000084589:109512-109598:+	ACA1_050510	4HBT domain-containing protein [Source:UniProtKB/TrEMBL;Acc:L8H578]	0.002138589	0.328434333	0.326295745	1.48E-09
6h	ACA1_188160/clean/scf7180000084747:114118-114217:-	ACA1_188160	RBR-type E3 ubiquitin transferase [Source:UniProtKB/TrEMBL;Acc:L8GRR7]	0.002352763	0.328378	0.326025237	3.77E-07
6h	ACA1_035310/clean/scf7180000084564:17353-17444:+	ACA1_035310	AMP-binding domain-containing protein [Source:UniProtKB/TrEMBL;Acc:L8HCU7]	0.011524833	0.337508333	0.3259835	4.86E-05
6h	ACA1_290130/clean/scf7180000084777:539039-539123:+	ACA1_290130	GST C-terminal domain-containing protein [Source:UniProtKB/TrEMBL;Acc:L8HI41]	0.01307765	0.339010333	0.325932683	7.64E-11
6h	ACA1_142840/clean/scf7180000084708:246031-246136:-	ACA1_142840	hypothetical protein	0.071483533	0.397399333	0.3259158	2.11E-06
6h	ACA1_200010/clean/scf7180000084753:367930-368028:+	ACA1_200010	Protein kinase domain containing protein [Source:UniProtKB/TrEMBL;Acc:L8H3C5]	0.03859158	0.364463667	0.325872087	5.51E-07
6h	ACA1_398440/clean/scf7180000084759:226213-226330:-	ACA1_398440	hypothetical protein	0	0.325787	0.325787	0.000449458
6h	ACA1_258470/clean/scf7180000084769:245773-245860:+	ACA1_258470	N-acetyltransferase domain-containing protein [Source:UniProtKB/TrEMBL;Acc:L8GHE7]	0.021549933	0.347327	0.325777067	7.66E-05
6h	ACA1_298630/clean/scf7180000082992:4993-5129:-	ACA1_298630	Vacuolar protein sorting 16, putative [Source:UniProtKB/TrEMBL;Acc:L8H081]	0.003714457	0.32947	0.325755543	2.91E-10
6h	ACA1_360270/clean/scf7180000084622:80565-80632:+	ACA1_360270	hypothetical protein	0.001526863	0.327261333	0.32573447	4.01E-08
6h	ACA1_216440/clean/scf7180000084758:505206-505330:+	ACA1_216440	PPM-type phosphatase domain-containing protein [Source:UniProtKB/TrEMBL;Acc:L8GQI6]	0.280173667	0.605895667	0.325722	0.000546715
6h	ACA1_248870/clean/scf7180000084766:139373-139461:-	ACA1_248870	GOLD domain-containing protein [Source:UniProtKB/TrEMBL;Acc:L8GY33]	0.006721857	0.332267333	0.325545477	1.89E-16
6h	ACA1_057240/clean/scf7180000084599:95296-95387:-	ACA1_057240	unspecified product	0.0012945	0.326777667	0.325483167	3.95E-07
6h	ACA1_156040/clean/scf7180000084723:11284-11380:+	ACA1_156040	Fungal Zn(2)-Cys(6) binuclear cluster domain containing protein [Source:UniProtKB/TrEMBL;Acc:L8GHR8]	0.018267133	0.343732	0.325464867	8.91E-05
6h	ACA1_138800/clean/scf7180000084705:12684-12782:+	ACA1_138800	hypothetical protein	0.029177367	0.354625	0.325447633	4.41E-15
6h	ACA1_289870/clean/scf7180000084777:481625-481739:+	ACA1_289870	Helicase ATP-binding domain-containing protein [Source:UniProtKB/TrEMBL;Acc:L8HI29]	0.00136799	0.326776667	0.325408677	1.97E-05
6h	ACA1_112030/clean/scf7180000084669:88904-89063:-	ACA1_112030	COMM domain-containing protein [Source:UniProtKB/TrEMBL;Acc:L8H411]	0.00369618	0.329097667	0.325401487	3.77E-09
6h	ACA1_329820/clean/scf7180000084566:5717-5824:-	ACA1_329820	Transporter, major facilitator subfamily protein [Source:UniProtKB/TrEMBL;Acc:L8GHX2]	0.000552887	0.325951	0.325398113	2.20E-12
6h	ACA1_369260/clean/scf7180000084664:285218-285318:+	ACA1_369260	hypothetical protein	0.003044783	0.328423333	0.325378551	8.49E-14
6h	ACA1_252570/clean/scf7180000084768:184026-184101:+	ACA1_252570	CBS domain containing protein [Source:UniProtKB/TrEMBL;Acc:L8HAN6]	0.256292333	0.581628333	0.325336	0.00064041
6h	ACA1_061700/clean/scf7180000084599:1013822-1013921:+	ACA1_061700	Amine oxidase family protein [Source:UniProtKB/TrEMBL;Acc:L8GWP7]	0.010972107	0.336261333	0.325289227	2.85E-08
6h	ACA1_181260/clean/scf7180000084744:158446-158517:-	ACA1_181260	hypothetical protein	0.028752433	0.353917333	0.3251649	0.000476348
6h	ACA1_076230/clean/scf7180000084612:37849-37951:+	ACA1_076230	Multisensor hybrid histidine kinase [Source:UniProtKB/TrEMBL;Acc:L8GL34]	0.009336067	0.334403667	0.3250676	2.40E-05
6h	ACA1_255750/clean/scf7180000084768:562737-562888:+	ACA1_255750	hypothetical protein	0.141592667	0.466590667	0.324998	0.000244567
6h	ACA1_063340/clean/scf7180000084599:1391374-1391478:+	ACA1_063340	Dynein intermediate chain, cytosolic, putative [Source:UniProtKB/TrEMBL;Acc:L8GX48]	0.000206943	0.325203333	0.32499639	1.62E-10
6h	ACA1_142400/clean/scf7180000084708:161044-161182:+	ACA1_142400	Plus3 domain-containing protein [Source:UniProtKB/TrEMBL;Acc:L8HAK6]	0.026468807	0.351444	0.324975193	3.39E-07
6h	ACA1_044940/clean/scf7180000084576:66966-67063:-	ACA1_044940	BTB domain-containing protein [Source:UniProtKB/TrEMBL;Acc:L8GYY6]	0.0095238	0.334475667	0.324951867	8.78E-06
6h	ACA1_391080/clean/scf7180000084740:123866-123940:+	ACA1_391080	PIG-P domain-containing protein [Source:UniProtKB/TrEMBL;Acc:L8GRI9]	0.01726885	0.342217667	0.324948817	4.10E-07
6h	ACA1_234340/clean/scf7180000084762:280161-280266:+	ACA1_234340	Ubiquitin carboxylterminal hydrolase domain containing protein [Source:UniProtKB/TrEMBL;Acc:L8H3J5]	0.001586968	0.326046	0.324459032	2.30E-11
6h	ACA1_277560/clean/scf7180000084776:74974-75066:-	ACA1_277560	F-box domain-containing protein [Source:UniProtKB/TrEMBL;Acc:L8H6G6]	0.0151515	0.339607667	0.324456167	0.000144136
6h	ACA1_034530/clean/scf7180000084563:64713-64799:+	ACA1_034530	hypothetical protein	0.001423033	0.325811	0.324387967	6.09E-09
6h	ACA1_264710/clean/scf7180000084770:363441-363511:-	ACA1_264710	Peptidase_S8 domain-containing protein [Source:UniProtKB/TrEMBL;Acc:L8H251]	0.00546207	0.329798	0.32433593	2.79E-08
6h	ACA1_366690/clean/scf7180000084641:480521-480599:+	ACA1_366690	Aspartyl aminopeptidase [Source:UniProtKB/TrEMBL;Acc:L8GM39]	7.26E-05	0.324391333	0.324318728	8.31E-11
6h	ACA1_183190/clean/scf7180000084745:352781-352887:-	ACA1_183190	TYR_PHOSPHATASE_2 domain-containing protein [Source:UniProtKB/TrEMBL;Acc:L8H893]	0.020730633	0.345021667	0.324291033	8.54E-23
6h	ACA1_234780/clean/scf7180000084762:315778-315882:-	ACA1_234780	hypothetical protein	0.010857337	0.335127333	0.324269997	2.24E-29
6h	ACA1_125420/clean/scf7180000084691:56832-56918:-	ACA1_125420	hypothetical protein	0.033333333	0.357536	0.324202667	0.000220672
6h	ACA1_167040/clean/scf7180000084736:60858-60982:+	ACA1_167040	Elongation of fatty acids protein [Source:UniProtKB/TrEMBL;Acc:L8GZZ6]	0.007764067	0.331901	0.324136933	4.03E-08
6h	ACA1_352370/clean/scf7180000084704:3388-3488:+	ACA1_352370	Oxidoreductase, short chain dehydrogenase/reductase superfamily protein [Source:UniProtKB/TrEMBL;Acc:L8GTQ2]	0.060764633	0.384887	0.324122367	3.24E-06
6h	ACA1_066530/clean/scf7180000084600:6667-6730:-	ACA1_066530	Bac_rhamnosid_C domain-containing protein [Source:UniProtKB/TrEMBL;Acc:L8GPR7]	0.0865906	0.410635	0.3240444	0.000100614
6h	ACA1_230230/clean/scf7180000084761:331434-331499:+	ACA1_230230	hypothetical protein	0.006013053	0.330010333	0.32399728	3.68E-11
6h	ACA1_201160/clean/scf7180000084753:495733-495824:-	ACA1_201160	RasGEF domain containing protein [Source:UniProtKB/TrEMBL;Acc:L8H4V2]	0.003734233	0.327727	0.323992767	2.68E-08

6h	ACA1_252570/clean/scf7180000084768:183159-183221:+	ACA1_252570	CBS domain containing protein [Source:UniProtKB/TrEMBL;Acc:L8HAN6]	0.0128205	0.336769333	0.323948833	4.34E-05
6h	ACA1_378160/clean/scf7180000084711:199652-199767:-	ACA1_378160	Ubiquitin carboxyl-terminal hydrolase [Source:UniProtKB/TrEMBL;Acc:L8GSX2]	0.005902797	0.329814667	0.32391187	1.11E-14
6h	ACA1_285450/clean/scf7180000084776:967989-968114:-	ACA1_285450	TYR_PHOSPHATASE_2 domain-containing protein [Source:UniProtKB/TrEMBL;Acc:L8H9U5]	0.008974513	0.332871	0.323896487	1.65E-08
6h	ACA1_189360/clean/scf7180000084748:17319-17409:-	ACA1_189360	Proline dehydrogenase [Source:UniProtKB/TrEMBL;Acc:L8GGF7]	0	0.323893667	0.323893667	0.007970775
6h	ACA1_189590/clean/scf7180000084748:56075-56133:+	ACA1_189590	Transcriptional regulatorrelated, putative [Source:UniProtKB/TrEMBL;Acc:L8GEX7]	0.005698	0.329563667	0.323865667	4.50E-08
6h	ACA1_320950/clean/scf7180000084435:756-846:-	ACA1_320950	SET domain containing protein [Source:UniProtKB/TrEMBL;Acc:L8GPN6]	0.030303033	0.353896667	0.323593633	0.000252607
6h	ACA1_174090/clean/scf7180000084741:604092-604228:+	ACA1_174090	AB hydrolase-1 domain-containing protein [Source:UniProtKB/TrEMBL;Acc:L8HHT7]	0.00055279	0.324032333	0.323479543	1.19E-09
6h	ACA1_061900/clean/scf7180000084599:1072934-1073042:-	ACA1_061900	GTP_EFTU_D3 domain-containing protein [Source:UniProtKB/TrEMBL;Acc:L8GY16]	0.012656973	0.336112333	0.32345536	1.12E-09
6h	ACA1_064240/clean/scf7180000084599:1566300-1566368:+	ACA1_064240	NAD(P)-bd_dom domain-containing protein [Source:UniProtKB/TrEMBL;Acc:L8GXD4]	0.002679811	0.326126667	0.323446855	1.05E-11
6h	ACA1_045470/clean/scf7180000084576:154254-154337:+	ACA1_045470	PH domain containing protein [Source:UniProtKB/TrEMBL;Acc:L8GZ29]	0.013947513	0.337392	0.323444487	3.61E-10
6h	ACA1_208290/clean/scf7180000084757:152591-152678:-	ACA1_208290	PPM-type phosphatase domain-containing protein [Source:UniProtKB/TrEMBL;Acc:L8H0Q6]	0.0021252	0.325568	0.3234428	6.27E-08
6h	ACA1_174050/clean/scf7180000084741:588256-588403:-	ACA1_174050	hypothetical protein	0.014535243	0.337956667	0.323421423	4.42E-06
6h	ACA1_063630/clean/scf7180000084599:1463812-1463872:-	ACA1_063630	Singlestranded DNA binding protein, putative [Source:UniProtKB/TrEMBL;Acc:L8GWM4]	0.002959473	0.326375333	0.32341586	1.17E-09
6h	ACA1_309660/clean/scf7180000083763:14617-14698:+	ACA1_309660	hypothetical protein	0.013486233	0.336749333	0.3232631	1.68E-07
6h	ACA1_063810/clean/scf7180000084599:1510224-1510314:+	ACA1_063810	DHHC zinc finger domain containing protein [Source:UniProtKB/TrEMBL;Acc:L8GXG4]	0.005217117	0.328451333	0.323234217	5.40E-07
6h	ACA1_024040/clean/scf7180000084440:13938-14014:-	ACA1_024040	WD_REPEATS_REGION domain-containing protein [Source:UniProtKB/TrEMBL;Acc:L8GI27]	0.00043111	0.323655333	0.323224223	8.00E-10
6h	ACA1_383100/clean/scf7180000084721:141138-141211:+	ACA1_383100	Glutamine amidotransferase type-2 domain-containing protein [Source:UniProtKB/TrEMBL;Acc:L8GUD3]	0.0267943	0.350009333	0.323215033	1.70E-06
6h	ACA1_277560/clean/scf7180000084776:76332-76405:-	ACA1_277560	F-box domain-containing protein [Source:UniProtKB/TrEMBL;Acc:L8H6G6]	0.1335065	0.456712667	0.323206167	0.001511495
6h	ACA1_377350/clean/scf7180000084711:40994-41075:+	ACA1_377350	YDG/SRA domain containing protein [Source:UniProtKB/TrEMBL;Acc:L8GU64]	0.010500667	0.333655	0.323154333	4.32E-05
6h	ACA1_113650/clean/scf7180000084669:343300-343384:+	ACA1_113650	hypothetical protein	0.001500548	0.324604667	0.323104118	2.04E-08
6h	ACA1_060270/clean/scf7180000084599:632963-633120:-	ACA1_060270	TYR_PHOSPHATASE_2 domain-containing protein [Source:UniProtKB/TrEMBL;Acc:L8GWC1]	0.007931633	0.331013667	0.323082033	1.22E-06
6h	ACA1_379220/clean/scf7180000084711:303859-303974:-	ACA1_379220	FYVE-type domain-containing protein [Source:UniProtKB/TrEMBL;Acc:L8GUF8]	0	0.323044667	0.323044667	1.21E-08
6h	ACA1_174230/clean/scf7180000084741:652700-652761:+	ACA1_174230	Rab5interacting protein (Rab5ip) [Source:UniProtKB/TrEMBL;Acc:L8HJ90]	0.006887643	0.329926	0.323038357	1.22E-21
6h	ACA1_065460/clean/scf7180000084599:1859119-1859196:-	ACA1_065460	RhoGAP domain containing protein [Source:UniProtKB/TrEMBL;Acc:L8GXS7]	0.001502503	0.324528667	0.323026163	6.74E-08
6h	ACA1_076230/clean/scf7180000084612:38062-38170:+	ACA1_076230	Multisensor hybrid histidine kinase [Source:UniProtKB/TrEMBL;Acc:L8GL34]	0.0689691	0.391968	0.3229989	7.52E-05
6h	ACA1_171660/clean/scf7180000084741:137144-137221:+	ACA1_171660	Serine/threonine-protein phosphatase 2A 55 kDa regulatory subunit B [Source:UniProtKB/TrEMBL;Acc:L8HHC4]	0.01573426	0.338579333	0.322845073	1.99E-18
6h	ACA1_395480/clean/scf7180000084756:188514-188622:-	ACA1_395480	Copine subfamily protein [Source:UniProtKB/TrEMBL;Acc:L8H0H7]	0	0.322723333	0.322723333	5.91E-08
6h	ACA1_384590/clean/scf7180000084730:59739-59809:-	ACA1_384590	RAB1B, member RAS oncogene family [Source:UniProtKB/TrEMBL;Acc:L8H896]	0.008482033	0.331169333	0.3226873	1.18E-14
6h	ACA1_087970/clean/scf7180000084639:146642-146710:+	ACA1_087970	NACHT domain containing protein [Source:UniProtKB/TrEMBL;Acc:L8GUL2]	0.0040802	0.326754667	0.322674467	3.15E-07
6h	ACA1_200240/clean/scf7180000084753:423406-423505:-	ACA1_200240	Phospholipase D active site domain containing protein [Source:UniProtKB/TrEMBL;Acc:L8H3B9]	0	0.322638333	0.322638333	5.72E-08
6h	ACA1_060920/clean/scf7180000084599:780888-780993:-	ACA1_060920	MFS domain-containing protein [Source:UniProtKB/TrEMBL;Acc:L8GVT5]	0.0050505	0.327682333	0.322631833	3.00E-06
6h	ACA1_015510/clean/scf7180000083799:8968-9051:-	ACA1_015510	Replication factora protein 1 (Rpa1) subfamily protein [Source:UniProtKB/TrEMBL;Acc:L8GZQ4]	0.006706053	0.329249	0.322542947	2.35E-06
6h	ACA1_189590/clean/scf7180000084748:58748-58833:+	ACA1_189590	Transcriptional regulatorrelated, putative [Source:UniProtKB/TrEMBL;Acc:L8GEX7]	0.003945973	0.326465333	0.32251936	1.14E-08
6h	ACA1_331110/clean/scf7180000084575:3232-3336:-	ACA1_331110	RAP1A, member of RAS oncogene family [Source:UniProtKB/TrEMBL;Acc:L8GU20]	0.009503673	0.331973667	0.322469993	1.39E-14
6h	ACA1_275770/clean/scf7180000084774:105002-105129:-	ACA1_275770	Twinfilin, putative [Source:UniProtKB/TrEMBL;Acc:L8GS07]	0.011970703	0.334375667	0.322404963	1.74E-09
6h	ACA1_121260/clean/scf7180000084682:17542-17609:+	ACA1_121260	Ankyrin repeat containing serine/threonine kinase [Source:UniProtKB/TrEMBL;Acc:L8GEX5]	0	0.322382667	0.322382667	9.70E-09
6h	ACA1_117240/clean/scf7180000084669:956730-956816:-	ACA1_117240	Non-specific serine/threonine protein kinase [Source:UniProtKB/TrEMBL;Acc:L8H707]	0.00064375	0.322812	0.32216825	8.74E-10
6h	ACA1_307250/clean/scf7180000083680:1829-1943:-	ACA1_307250	Mss4 protein [Source:UniProtKB/TrEMBL;Acc:L8H520]	0.0076841	0.329787	0.3221029	2.00E-09
6h	ACA1_097960/clean/scf7180000084645:1363072-1363152:-	ACA1_097960	Ribosomal protein L33, putative [Source:UniProtKB/TrEMBL;Acc:L8GJX1]	0.013758173	0.33578	0.322021827	2.98E-11
6h	ACA1_116960/clean/scf7180000084669:882717-882794:+	ACA1_116960	hypothetical protein	0.011795303	0.333719	0.321923697	3.10E-09
6h	ACA1_055410/clean/scf7180000084594:699765-699866:+	ACA1_055410	Protein kinase domain-containing protein [Source:UniProtKB/TrEMBL;Acc:L8H6J6]	0.083299167	0.405078667	0.3217795	6.44E-06
6h	ACA1_116140/clean/scf7180000084669:711352-711420:+	ACA1_116140	Dipeptidyl-peptidase family protein [Source:UniProtKB/TrEMBL;Acc:L8H5Z5]	0.001013684	0.32275	0.321736316	1.12E-10
6h	ACA1_085170/clean/scf7180000084632:62727-62809:+	ACA1_085170	TPR_REGION domain-containing protein [Source:UniProtKB/TrEMBL;Acc:L8HM05]	0.00055564	0.322287333	0.321731693	2.70E-10
6h	ACA1_245670/clean/scf7180000084763:517937-518040:-	ACA1_245670	hypothetical protein	0.00419699	0.325772	0.32157501	7.48E-07
6h	ACA1_128240/clean/scf7180000084697:57125-57273:+	ACA1_128240	RNA_pol_Rpb1_5 domain-containing protein [Source:UniProtKB/TrEMBL;Acc:L8GWI9]	0.003628	0.325189	0.321561	6.04E-06
6h	ACA1_149410/clean/scf7180000084715:147065-147173:+	ACA1_149410	Lectin_legB domain-containing protein [Source:UniProtKB/TrEMBL;Acc:L8HDD6]	0.014105687	0.335598667	0.32149298	2.32E-08
6h	ACA1_322220/clean/scf7180000084459:33690-33832:+	ACA1_322220	hypothetical protein	0.0684562	0.389936333	0.321480133	1.04E-06
6h	ACA1_368060/clean/scf7180000084664:46053-46174:+	ACA1_368060	PPM-type phosphatase domain-containing protein [Source:UniProtKB/TrEMBL;Acc:L8GYR6]	0.004975133	0.326428333	0.3214532	6.47E-07
6h	ACA1_073390/clean/scf7180000084603:1406652-1406760:+	ACA1_073390	hypothetical protein	0	0.32141	0.32141	4.92E-10
6h	ACA1_178340/clean/scf7180000084743:218036-218165:+	ACA1_178340	RhoGEF domain containing protein [Source:UniProtKB/TrEMBL;Acc:L8GT64]	0	0.321314667	0.321314667	7.59E-08
6h	ACA1_164910/clean/scf7180000084729:309590-309661:-	ACA1_164910	hypothetical protein	0.02737	0.348648333	0.321278333	4.65E-14
6h	ACA1_190280/clean/scf7180000084748:146909-146991:+	ACA1_190280	Ubiquitinyl hydrolase 1 [Source:UniProtKB/TrEMBL;Acc:L8GGJ9]	0.003542657	0.324764	0.321221343	7.42E-09
6h	ACA1_065700/clean/scf7180000084599:1875010-1875081:+	ACA1_065700	Protein kinase domain-containing protein [Source:UniProtKB/TrEMBL;Acc:L8H039]	0.010559787	0.331771667	0.32121188	2.73E-06
6h	ACA1_289320/clean/scf7180000084777:415181-415259:-	ACA1_289320	Myblike DNA-binding domain containing protein [Source:UniProtKB/TrEMBL;Acc:L8HI02]	0.0111111	0.332312	0.3212009	8.15E-05
6h	ACA1_140770/clean/scf7180000084707:116793-116863:+	ACA1_140770	ATPase, AAA domain containing protein [Source:UniProtKB/TrEMBL;Acc:L8GJ22]	0.019190967	0.340329667	0.3211387	5.58E-07
6h	ACA1_164510/clean/scf7180000084729:234870-234939:+	ACA1_164510	Leucine rich repeat domain containing protein [Source:UniProtKB/TrEMBL;Acc:L8GU14]	0.0345377	0.355651	0.3211133	1.08E-09
6h	ACA1_367890/clean/scf7180000084664:8887-8973:-	ACA1_367890	hypothetical protein	0	0.321054667	0.321054667	1.15E-07
6h	ACA1_098210/clean/scf7180000084645:1429951-1430017:+	ACA1_098210	Peroxidase, putative [Source:UniProtKB/TrEMBL;Acc:L8GJY8]	0.008547	0.329521667	0.320974667	8.72E-06
6h	ACA1_057960/clean/scf7180000084599:218209-218281:+	ACA1_057960	BTB/POZ domain containing protein [Source:UniProtKB/TrEMBL;Acc:L8GVX1]	0	0.320876	0.320876	5.03E-07
6h	ACA1_226760/clean/scf7180000084761:73349-73460:+	ACA1_226760	hypothetical protein	0.032452067	0.353152	0.320699933	1.03E-05
6h	ACA1_095690/clean/scf7180000084645:882589-882688:+	ACA1_095690	hypothetical protein	0.0070922	0.327733	0.3206408	2.59E-05
6h	ACA1_116740/clean/scf7180000084669:839696-839776:+	ACA1_116740	Methyltransf_2 domain-containing protein [Source:UniProtKB/TrEMBL;Acc:L8H4R1]	0.072105033	0.392743	0.320637967	5.97E-05
6h	ACA1_252180/clean/scf7180000084768:139838-139926:+	ACA1_252180	Leucine rich repeat domain containing protein [Source:UniProtKB/TrEMBL;Acc:L8H9U3]	0.036801667	0.357416333	0.320614667	5.20E-06
6h	ACA1_086070/clean/scf7180000084636:21556-21640:-	ACA1_086070	Serine/threonine kinase [Source:UniProtKB/TrEMBL;Acc:L8HJ13]	0.040016267	0.360625	0.320608733	2.32E-12
6h	ACA1_072130/clean/scf7180000084603:1157588-1157677:+	ACA1_072130	hypothetical protein	0.009931705	0.330533	0.320601295	1.75E-06
6h	ACA1_070990/clean/scf7180000084603:822851-822953:+	ACA1_070990	hypothetical protein	0	0.320527667	0.320527667	1.11E-09
6h	ACA1_131570/clean/scf7180000084699:90127-90243:-	ACA1_131570	Transmembrane proteins 14c protein [Source:UniProtKB/TrEMBL;Acc:L8H2Q4]	0.001751482	0.322156	0.320404518	5.93E-09
6h	ACA1_144360/clean/scf7180000084709:215192-215302:+	ACA1_144360	Repressor of RNA polymerase III transcription [Source:UniProtKB/TrEMBL;Acc:L8HFX0]	0.009436423	0.329818	0.320381577	1.71E-21
6h	ACA1_074070/clean/scf7180000084605:16230-16468:-	ACA1_074070	PAS domain-containing protein [Source:UniProtKB/TrEMBL;Acc:L8HKA1]	0.017925667	0.338277667	0.320352	2.78E-07
6h	ACA1_025670/clean/scf7180000084451:591-689:-	ACA1_025670	DOMON domain containing protein [Source:UniProtKB/TrEMBL;Acc:L8GGT9]	0.007852897	0.328204667	0.32035177	2.89E-06
6h	ACA1_372600/clean/scf7180000084679:24410-24543:-	ACA1_372600	F-box domain-containing protein [Source:UniProtKB/TrEMBL;Acc:L8GJA4]	0.00076412	0.320912667	0.320148547	3.50E-07
6h	ACA1_024040/clean/scf7180000084440:15364-15448:-	ACA1_024040	WD_REPEATS_REGION domain-containing protein [Source:UniProtKB/TrEMBL;Acc:L8GI27]	0	0.320031333	0.320031333	2.08E-09
6h	ACA1_195840/clean/scf7180000084752:33617-33721:+	ACA1_195840	EGFlike domain containing protein [Source:UniProtKB/TrEMBL;Acc:L8HET8]	0.018047663	0.338025333	0.31997767	1.28E-08
6h	ACA1_326170/clean/scf7180000084504:45178-45256:+	ACA1_326170	SAM domain-containing protein [Source:UniProtKB/TrEMBL;Acc:L8HIK1]	0.05201641	0.371947667	0.319931257	9.88E-06
6h	ACA1_000820/clean/scf7180000082828:9107-9182:+	ACA1_000820	RING-type domain-containing protein [Source:UniProtKB/TrEMBL;Acc:L8H7M3]	0	0.319898	0.319898	3.32E-08
6h	ACA1_290650/clean/scf7180000084777:646014-646124:+	ACA1_290650	DENN domain-containing protein [Source:UniProtKB/TrEMBL;Acc:L8HI74]	0	0.319866	0.319866	1.94E-05
6h	ACA1_045280/clean/scf7180000084576:105411-105505:+	ACA1_045280	Protein kinase domain-containing protein [Source:UniProtKB/TrEMBL;Acc:L8GZU5]	0.0316963	0.351475667	0.319779367	5.79E-05
6h	ACA1_062880/clean/scf7180000084599:1297823-1297889:+	ACA1_062880	hypothetical protein	0	0.319686333	0.319686333	2.07E-07
6h	ACA1_183010/clean/scf7180000084745:311394-311469:+	ACA1_183010	ABC1 domain-containing protein [Source:UniProtKB/TrEMBL;Acc:L8H7Z3]	0.018229167	0.337898667	0.3196695	0.00024696
6h	ACA1_013340/clean/scf7180000083750:376-456:+	ACA1_013340	PH domain-containing protein [Source:UniProtKB/TrEMBL;Acc:L8HGJ7]	0.005830993	0.325465667	0.319634673	1.69E-08
6h	ACA1_266220/clean/scf7180000084770:633065-633158:+	ACA1_266220	Adenylate kinase, putative [Source:UniProtKB/TrEMBL;Acc:L8H4J7]	0.036417657	0.356050333	0.319632677	9.86E-07
6h	ACA1_218390/clean/scf7180000084758:651633-651713:-	ACA1_218390	Glycosyl hydrolase family 20, catalytic domain containing protein [Source:UniProtKB/TrEMBL;Acc:L8GQD2]	0.0173867	0.336947	0.3195603	4.71E-07
6h	ACA1_209850/clean/scf7180000084757:338282-338428:-	ACA1_209850	Rho-GAP domain-containing protein [Source:UniProtKB/TrEMBL;Acc:L8GXM2]	0.005887413	0.325334333	0.31944692	3.18E-07
6h	ACA1_057160/clean/scf7180000084599:62755-62850:-	ACA1_057160	unspecified product	0.030546033	0.349979667	0.319433633	0.001928065
6h	ACA1_068270/clean/scf7180000084603:154134-154202:-	ACA1_068270	Phospholipid-transporting ATPase [Source:UniProtKB/TrEMBL;Acc:L8HEP8]	0	0.319318667	0.319318667	2.13E-07
6h	ACA1_260950/clean/scf7180000084769:563933-564031:-	ACA1_260950	Ribonuclease 25A protein [Source:UniProtKB/TrEMBL;Acc:L8GG37]	0.004286367	0.323585667	0.3192993	1.21E-05
6h	ACA1_310750/clean/scf7180000083780:5895-5971:+	ACA1_310750	RFX-type winged-helix domain-containing protein [Source:UniProtKB/TrEMBL;Acc:L8GVY4]	0.008775237	0.327931667	0.31915643	2.45E-06
6h	ACA1_366420/clean/scf7180000084641:418952-419055:+	ACA1_366420	Translation factor GUF1 homolog, mitochondrial [Source:UniProtKB/TrEMBL;Acc:L8GLT1]	0.031681083	0.350698	0.319016917	1.49E-05
6h	ACA1_384590/clean/scf7180000084730:59547-59635:-	ACA1_384590	RAB1B, member RAS oncogene family [Source:UniProtKB/TrEMBL;Acc:L8H896]	0.00613867	0.325148	0.31900933	7.83E-10
6h	ACA1_121250/clean/scf7180000084682:12197-12301:-	ACA1_121250	hypothetical protein	0.039380267	0.358348333	0.318968067	8.99E-05
6h	ACA1_269770/clean/scf7180000084770:1035923-1036009:+	ACA1_269770	hypothetical protein	0.046075867	0.364974667	0.3188988	0.01017161
6h	ACA1_158440/clean/scf7180000084724:219616-219782:+	ACA1_158440	Coiled-coil domain containing 53, putative [Source:UniProtKB/TrEMBL;Acc:L8HA48]	0.005102737	0.323955333	0.318852597	2.24E-11
6h	ACA1_158490/clean/scf7180000084724:236630-236709:+	ACA1_158490	Mur_ligase_M domain-containing protein [Source:UniProtKB/TrEMBL;Acc:L8H9A2]	0.019802767	0.33861	0.318807233	4.57E-05
6h	ACA1_072110/clean/scf7180000084603:1150136-1150240:+	ACA1_072110	MYND-type domain-containing protein [Source:UniProtKB/TrEMBL;Acc:L8HDL8]	0.035622533	0.354421667	0.318799133	0.001912039
6h	ACA1_215970/clean/scf7180000084758:420598-420690:-	ACA1_215970	EGFlike domain containing protein [Source:UniProtKB/TrEMBL;Acc:L8GQ73]	0	0.318785667	0.318785667	0.002446581
6h	ACA1_066000/clean/scf7180000084599:1971115-1971228:+	ACA1_066000	Arp2/3 complex 34 kDa subunit [Source:UniProtKB/TrEMBL;Acc:L8GWZ2]	0.009381097	0.328164	0.318782903	2.09E-25
6h	ACA1_400260/clean/scf7180000084775:131525-131607:+	ACA1_400260	Ranlike small GTPase [Source:UniProtKB/TrEMBL;Acc:L8GIF4]	0	0.318739667	0.318739667	0.000197129
6h	ACA1_181640/clean/scf7180000084745:31798-31889:+	ACA1_181640	hypothetical protein	0.006172833	0.324889	0.318716167	8.74E-06
6h	ACA1_138520/clean/scf7180000084703:160596-160691:+	ACA1_138520	Phospholipase B-like [Source:UniProtKB/TrEMBL;Acc:L8GZK2]	0.000465697	0.319065333	0.318599637	3.34E-11
6h	ACA1_310760/clean/scf7180000083780:7379-7449:+	ACA1_310760	hypothetical protein	0.042892033	0.361435	0.318542967	7.29E-07
6h	ACA1_175110/clean/scf7180000084741:866763-866825:-	ACA1_175110	hypothetical protein	0	0.318505667	0.318505667	2.83E-06
6h	ACA1_337970/clean/scf7180000084604:14378-14482:-	ACA1_337970	JmjC domain-containing protein [Source:UniProtKB/TrEMBL;Acc:L8GCM4]	0.0246416	0.343069	0.3184274	0.000212608
6h	ACA1_237440/clean/scf7180000084762:511003-511057:-	ACA1_237440	Peptidyl-prolyl cis-trans isomerase [Source:UniProtKB/TrEMBL;Acc:L8H0V6]	0.005394298	0.323535667	0.318141369	1.17E-07
6h	ACA1_289690/clean/scf7180000084777:443984-444052:-	ACA1_289690	hypothetical protein	0	0.318136333	0.318136333	3.73E-09
6h	ACA1_366940/clean/scf7180000084641:520165-520263:-	ACA1_366940	BTB/POZ domain containing protein [Source:UniProtKB/TrEMBL;Acc:L8GPM1]	0	0.318132667	0.318132667	1.31E-05
6h	ACA1_155500/clean/scf7180000084722:174742-174857:-	ACA1_155500	unspecified product	0.023061133	0.341021	0.317959867	1.85E-06
6h	ACA1_328630/clean/scf7180000084560:12479-12567:+	ACA1_328630	EF hand domain containing protein [Source:UniProtKB/TrEMBL;Acc:L8GRT5]	0.006777317	0.324731	0.317953683	4.95E-08
6h	ACA1_181590/clean/scf7180000084745:24029-24118:+	ACA1_181590	AcylCoenzyme A dehydrogenase family, member 10 isoform 7, putative [Source:UniProtKB/TrEMBL;Acc:L8H7Y6]	0.005143933	0.322977	0.317833067	3.20E-07
6h	ACA1_248410/clean/scf7180000084766:59382-59451:-	ACA1_248410	Alpha2-macroglobulin domain containing protein [Source:UniProtKB/TrEMBL;Acc:L8GY08]	0.026435033	0.344246333	0.3178113	2.92E-08
6h	ACA1_272530/clean/scf7180000084772:55901-55976:+	ACA1_272530	VWFA domain-containing protein [Source:UniProtKB/TrEMBL;Acc:L8HIL5]	0	0.317755	0.317755	1.45E-08
6h	ACA1_208460/clean/scf7180000084757:198318-198447:-	ACA1_208460	Abhydro_lipase domain-containing protein [Source:UniProtKB/TrEMBL;Acc:L8GZL5]	0.02318023	0.340863333	0.317683103	3.02E-07
6h	ACA1_369880/clean/scf7180000084664:401681-401757:+	ACA1_369880	hypothetical protein	0.007673717	0.325355	0.317681283	1.20E-07
6h	ACA1_064570/clean/scf7180000084599:1646653-1646730:+	ACA1_064570	Ras subfamily protein [Source:UniProtKB/TrEMBL;Acc:L8GXF9]	0.021045593	0.338705	0.317659407	9.94E-12
6h	ACA1_258680/clean/scf7180000084769:307502-307613:-	ACA1_258680	hypothetical protein	0.013224267	0.330791	0.317566733	1.64E-08
6h	ACA1_278150/clean/scf7180000084776:180508-180568:-	ACA1_278150	hypothetical protein	0.001938379	0.319486	0.317547621	1.17E-11
6h	ACA1_230510/clean/scf7180000084761:367949-368020:-	ACA1_230510	hypothetical protein	0	0.317453333	0.317453333	3.19E-08
6h	ACA1_388780/clean/scf7180000084733:169874-169988:+	ACA1_388780	EGFlike domain containing protein [Source:UniProtKB/TrEMBL;Acc:L8GDT8]	0.002838227	0.320223	0.317384773	2.93E-08
6h	ACA1_162370/clean/scf7180000084726:43208-43287:-	ACA1_162370	Protein kinase domain-containing protein [Source:UniProtKB/TrEMBL;Acc:L8GYN7]	0.002715636	0.320077333	0.317361698	3.46E-09
6h	ACA1_385400/clean/scf7180000084730:163891-163989:-	ACA1_385400	Rieske domain-containing protein [Source:UniProtKB/TrEMBL;Acc:L8H8D5]	0.034131963	0.351455667	0.317323703	1.87E-05
6h	ACA1_323130/clean/scf7180000084467:10677-10747:-	ACA1_323130	HemolysinIII related subfamily protein [Source:UniProtKB/TrEMBL;Acc:L8GGU0]	0.103979933	0.421213	0.317233067	0.000109969
6h	ACA1_074700/clean/scf7180000084608:66156-66250:-	ACA1_074700	hypothetical protein	0.000913243	0.318103667	0.317190423	1.04E-07
6h	ACA1_215990/clean/scf7180000084758:425792-425869:+	ACA1_215990	hypothetical protein	0	0.317103	0.317103	1.11E-08
6h	ACA1_377500/clean/scf7180000084711:77350-77475:+	ACA1_377500	Peptidase family c78 protein [Source:UniProtKB/TrEMBL;Acc:L8GU78]	0.01771907	0.334731333	0.317012263	5.67E-10
6h	ACA1_153200/clean/scf7180000084720:85478-85613:+	ACA1_153200	RBR-type E3 ubiquitin transferase [Source:UniProtKB/TrEMBL;Acc:L8HIB3]	0.003414579	0.320333333	0.316918754	2.27E-07
6h	ACA1_216300/clean/scf7180000084758:462686-462827:+	ACA1_216300	Ras family protein [Source:UniProtKB/TrEMBL;Acc:L8GR60]	0.080431167	0.397335	0.316903833	3.44E-05
6h	ACA1_076210/clean/scf7180000084612:25919-26023:-	ACA1_076210	hypothetical protein	0.008913017	0.325784667	0.31687165	1.37E-14
6h	ACA1_379130/clean/scf7180000084711:283158-283247:-	ACA1_379130	hypothetical protein	0	0.316839667	0.316839667	3.03E-05
6h	ACA1_369860/clean/scf7180000084664:397360-397435:+	ACA1_369860	GTPbinding protein SARA, putative [Source:UniProtKB/TrEMBL;Acc:L8H0F0]	0	0.316811	0.316811	1.16E-10
6h	ACA1_199630/clean/scf7180000084753:281475-281552:-	ACA1_199630	PRAI domain-containing protein [Source:UniProtKB/TrEMBL;Acc:L8H3A6]	0.007083913	0.323859333	0.31677542	3.60E-08
6h	ACA1_395480/clean/scf7180000084756:189470-189562:-	ACA1_395480	Copine subfamily protein [Source:UniProtKB/TrEMBL;Acc:L8H0H7]	0	0.316753	0.316753	7.07E-08
6h	ACA1_151110/clean/scf7180000084716:63070-63174:+	ACA1_151110	Sec23/Sec24 trunk domain containing protein [Source:UniProtKB/TrEMBL;Acc:L8GZW6]	0.01071909	0.327303333	0.316584243	1.28E-16
6h	ACA1_019350/clean/scf7180000083937:31255-31317:-	ACA1_019350	FGGAP repeat domain containing protein [Source:UniProtKB/TrEMBL;Acc:L8HFR8]	0.003467087	0.320019	0.316551913	1.33E-07
6h	ACA1_400300/clean/scf7180000084775:144005-144087:+	ACA1_400300	UBIQUITIN_CONJUGAT_2 domain-containing protein [Source:UniProtKB/TrEMBL;Acc:L8GGI3]	0	0.316479333	0.316479333	0.000231454
6h	ACA1_297750/clean/scf7180000082853:1671-1830:-	ACA1_297750	14_3_3 domain-containing protein [Source:UniProtKB/TrEMBL;Acc:L8HE45]	0.036336433	0.352804	0.316467567	0.004111086
6h	ACA1_053280/clean/scf7180000084594:244606-244683:+	ACA1_053280	Eukaryotic translation initiation factor 4 gamma 3 (EIF4-gamma 3), putative [Source:UniProtKB/TrEMBL;Acc:L8H653]	0	0.316449667	0.316449667	3.91E-12
6h	ACA1_071170/clean/scf7180000084603:879108-879221:+	ACA1_071170	RhoGAP domain containing protein [Source:UniProtKB/TrEMBL;Acc:L8HGC8]	0	0.316361667	0.316361667	1.35E-07
6h	ACA1_076800/clean/scf7180000084612:151448-151520:+	ACA1_076800	ABC transporter domain-containing protein [Source:UniProtKB/TrEMBL;Acc:L8GLB2]	0.0367199	0.353001667	0.316281767	0.003020324
6h	ACA1_170820/clean/scf7180000084741:13828-13934:-	ACA1_170820	hypothetical protein	0.010366893	0.326638667	0.316271773	1.79E-13

6h	ACA1_182860/clean/scf7180000084745:268271-268365:-	ACA1_182860	Ankyrin repeat protein, putative [Source:UniProtKB/TrEMBL;Acc:L8H7X7]	0	0.316215667	0.316215667	4.16E-06
6h	ACA1_065770/clean/scf7180000084599:1899509-1899603:-	ACA1_065770	Phosphatidylinositol 3kinase [Source:UniProtKB/TrEMBL;Acc:L8GXT7]	0.000451907	0.316647	0.316195093	3.85E-08
6h	ACA1_275790/clean/scf7180000084774:109983-110108:-	ACA1_275790	RNA recognition motif domain containing protein [Source:UniProtKB/TrEMBL;Acc:L8GQX9]	0.065333667	0.381493	0.316159333	2.33E-05
6h	ACA1_203090/clean/scf7180000084754:141553-141661:+	ACA1_203090	Glycosyl hydrolase, family 31 protein [Source:UniProtKB/TrEMBL;Acc:L8GTG9]	0	0.316156	0.316156	2.59E-10
6h	ACA1_019350/clean/scf7180000083937:28549-28604:-	ACA1_019350	FGGAP repeat domain containing protein [Source:UniProtKB/TrEMBL;Acc:L8HFR8]	0.0193937	0.335532667	0.316138967	2.19E-10
6h	ACA1_165790/clean/scf7180000084732:83168-83274:-	ACA1_165790	hypothetical protein	0.014187107	0.330316667	0.31612956	5.51E-09
6h	ACA1_228760/clean/scf7180000084761:207487-207604:+	ACA1_228760	Mitochondrial carrier protein [Source:UniProtKB/TrEMBL;Acc:L8H8X8]	0.006043217	0.322152333	0.316109117	6.03E-06
6h	ACA1_051460/clean/scf7180000084592:18415-18494:-	ACA1_051460	hypothetical protein	0	0.315959333	0.315959333	0.000264222
6h	ACA1_014210/clean/scf7180000083757:12588-12960:-	ACA1_014210	hypothetical protein	0.092398633	0.40821	0.315811367	0.000576617
6h	ACA1_116230/clean/scf7180000084669:730421-730513:+	ACA1_116230	Protein kinase domain containing protein [Source:UniProtKB/TrEMBL;Acc:L8H4M9]	0	0.315772667	0.315772667	4.91E-06
6h	ACA1_074160/clean/scf7180000084605:39009-39090:+	ACA1_074160	Aamy domain-containing protein [Source:UniProtKB/TrEMBL;Acc:L8HJ75]	0.002574003	0.318272667	0.315698663	4.50E-10
6h	ACA1_144880/clean/scf7180000084710:32797-32889:-	ACA1_144880	hypothetical protein	0.00066737	0.316351	0.31568363	3.04E-12
6h	ACA1_092820/clean/scf7180000084645:414756-414853:-	ACA1_092820	Dipeptidyl peptidase [Source:UniProtKB/TrEMBL;Acc:L8GJ18]	0	0.315432	0.315432	1.45E-06
6h	ACA1_363770/clean/scf7180000084641:11365-11442:+	ACA1_363770	TPR_REGION domain-containing protein [Source:UniProtKB/TrEMBL;Acc:L8GLH0]	0.0035461	0.318962	0.3154159	1.11E-07
6h	ACA1_095670/clean/scf7180000084645:876126-876212:-	ACA1_095670	FAD-binding PCMH-type domain-containing protein [Source:UniProtKB/TrEMBL;Acc:L8GIT7]	0.009192167	0.324591667	0.3153995	2.09E-08
6h	ACA1_174170/clean/scf7180000084741:632361-632467:-	ACA1_174170	Glutathione Stransferase, C-terminal domain containing protein [Source:UniProtKB/TrEMBL;Acc:L8HHR9]	0.001492317	0.316842667	0.31535035	1.18E-30
6h	ACA1_034090/clean/scf7180000084561:21671-21749:-	ACA1_034090	Protein kinase domain containing protein [Source:UniProtKB/TrEMBL;Acc:L8GWJ8]	0.00996433	0.325308	0.31534367	7.06E-11
6h	ACA1_370300/clean/scf7180000084664:490379-490456:-	ACA1_370300	hypothetical protein	0	0.315291	0.315291	3.15E-12
6h	ACA1_326250/clean/scf7180000084504:65072-65149:-	ACA1_326250	Methyltranfer_dom domain-containing protein [Source:UniProtKB/TrEMBL;Acc:L8HJF9]	0	0.315205667	0.315205667	7.31E-10
6h	ACA1_309500/clean/scf7180000083762:8738-8873:+	ACA1_309500	Pyridine nucleotidedisulfide oxidoreductase domain containing protein [Source:UniProtKB/TrEMBL;Acc:L8GI23]	0.000229727	0.315374	0.315144273	4.17E-12
6h	ACA1_038420/clean/scf7180000084568:144930-145010:-	ACA1_038420	SPX domain-containing protein [Source:UniProtKB/TrEMBL;Acc:L8GKX4]	0.0038168	0.318945333	0.315128533	4.29E-05
6h	ACA1_269730/clean/scf7180000084770:1027056-1027219:+	ACA1_269730	Metallophos domain-containing protein [Source:UniProtKB/TrEMBL;Acc:L8H506]	0.0215187	0.336550667	0.315031967	0.000356521
6h	ACA1_399850/clean/scf7180000084775:47377-47456:-	ACA1_399850	Ras family protein [Source:UniProtKB/TrEMBL;Acc:L8GIC5]	0.005418123	0.320422667	0.315004543	5.27E-10
6h	ACA1_184000/clean/scf7180000084745:542997-543123:-	ACA1_184000	hypothetical protein	0.0175567	0.332555333	0.314998633	4.99E-10
6h	ACA1_188180/clean/scf7180000084747:121324-121412:-	ACA1_188180	IU_nuc_hydro domain-containing protein [Source:UniProtKB/TrEMBL;Acc:L8GUV3]	0	0.31481	0.31481	0.018109156
6h	ACA1_314440/clean/scf7180000083843:10769-10877:-	ACA1_314440	Protein kinase domain-containing protein [Source:UniProtKB/TrEMBL;Acc:L8HEY9]	0.009910536	0.324667667	0.31475713	7.08E-06
6h	ACA1_233070/clean/scf7180000084762:118616-118695:+	ACA1_233070	hypothetical protein	0.025620233	0.340344333	0.3147241	3.95E-09
6h	ACA1_146570/clean/scf7180000084713:59139-59226:+	ACA1_146570	Ceramidase [Source:UniProtKB/TrEMBL;Acc:L8GV94]	0.009500913	0.324221333	0.31472042	4.62E-06
6h	ACA1_074110/clean/scf7180000084605:29242-29324:-	ACA1_074110	Peptidase [Source:UniProtKB/TrEMBL;Acc:L8HJ70]	0.000620552	0.315267	0.314646448	7.13E-11
6h	ACA1_287970/clean/scf7180000084777:135486-135651:+	ACA1_287970	Ras-GEF domain-containing protein [Source:UniProtKB/TrEMBL;Acc:L8HL64]	0.030285	0.344830667	0.314545667	1.04E-08
6h	ACA1_335940/clean/scf7180000084593:9194-9295:-	ACA1_335940	RNA recognition motif domain containing protein [Source:UniProtKB/TrEMBL;Acc:L8H3Y6]	0.00177624	0.316306	0.31452976	1.10E-09
6h	ACA1_095660/clean/scf7180000084645:872223-872345:+	ACA1_095660	Poly [ADP-ribose] polymerase [Source:UniProtKB/TrEMBL;Acc:L8GL42]	0.001459467	0.315982333	0.314522867	3.65E-08
6h	ACA1_400560/clean/scf7180000084775:194346-194436:+	ACA1_400560	hypothetical protein	0.005376333	0.319878	0.314501667	1.70E-06
6h	ACA1_282710/clean/scf7180000084776:721287-721373:+	ACA1_282710	Cyclic nucleotide-binding domain-containing protein [Source:UniProtKB/TrEMBL;Acc:L8H7K2]	0.012432567	0.326806	0.314373433	0.000692103
6h	ACA1_225890/clean/scf7180000084761:14059-14132:+	ACA1_225890	hypothetical protein	0.011750827	0.326109333	0.314358507	2.04E-11
6h	ACA1_100610/clean/scf7180000084648:129276-129364:-	ACA1_100610	Serine/threonine kinase [Source:UniProtKB/TrEMBL;Acc:L8GK64]	0.017272213	0.331432333	0.31416012	3.06E-11
6h	ACA1_195570/clean/scf7180000084752:18293-18409:+	ACA1_195570	hypothetical protein	0.050463333	0.364609667	0.314146333	0.000118328
6h	ACA1_257430/clean/scf7180000084769:151870-151949:-	ACA1_257430	Pyridoxamine 5'phosphate oxidase family superfamily protein [Source:UniProtKB/TrEMBL;Acc:L8GFR0]	0.02118094	0.335323	0.31414206	0.000142652
6h	ACA1_074550/clean/scf7180000084608:42050-42122:-	ACA1_074550	zf-C2HC5 domain-containing protein [Source:UniProtKB/TrEMBL;Acc:L8HGP3]	0.041782634	0.355884333	0.314101699	0.000965683
6h	ACA1_131740/clean/scf7180000084699:112916-112993:+	ACA1_131740	Carrier domain-containing protein [Source:UniProtKB/TrEMBL;Acc:L8GPY2]	0.000870183	0.314861333	0.31399115	7.83E-10
6h	ACA1_043230/clean/scf7180000084573:141988-142055:-	ACA1_043230	hypothetical protein	0.100198533	0.414066667	0.313868133	0.00786872
6h	ACA1_153010/clean/scf7180000084720:53403-53516:-	ACA1_153010	hypothetical protein	0.00285535	0.316715333	0.313859983	1.06E-08
6h	ACA1_309310/clean/scf7180000083758:3806-3883:+	ACA1_309310	hypothetical protein	0.0203312	0.334029667	0.313698467	0.00025555
6h	ACA1_206060/clean/scf7180000084755:71787-71868:+	ACA1_206060	Ras subfamily protein [Source:UniProtKB/TrEMBL;Acc:L8H5G9]	0.015336667	0.329	0.313663333	1.84E-07
6h	ACA1_253360/clean/scf7180000084768:284541-284623:+	ACA1_253360	TP_methylase domain-containing protein [Source:UniProtKB/TrEMBL;Acc:L8HAX0]	0	0.313637	0.313637	6.99E-08
6h	ACA1_382380/clean/scf7180000084721:46296-46394:+	ACA1_382380	WD_REPEATS_REGION domain-containing protein [Source:UniProtKB/TrEMBL;Acc:L8GUA6]	0.03474628	0.348343	0.31359672	1.99E-06
6h	ACA1_289320/clean/scf7180000084777:414850-414944:-	ACA1_289320	Myblike DNA-binding domain containing protein [Source:UniProtKB/TrEMBL;Acc:L8HI02]	0.026132693	0.339685333	0.31355264	0.00040356
6h	ACA1_277940/clean/scf7180000084776:133596-133680:-	ACA1_277940	Cysteine protease 5, putative [Source:UniProtKB/TrEMBL;Acc:L8H7Z6]	0.00145494	0.314993	0.31353806	8.18E-12
6h	ACA1_240180/clean/scf7180000084763:201250-201329:+	ACA1_240180	WD_REPEATS_REGION domain-containing protein [Source:UniProtKB/TrEMBL;Acc:L8GM97]	0	0.313526	0.313526	1.21E-07
6h	ACA1_198520/clean/scf7180000084753:132850-132950:-	ACA1_198520	Carrier superfamily protein [Source:UniProtKB/TrEMBL;Acc:L8H5C1]	0.013100633	0.326562333	0.3134617	5.21E-08
6h	ACA1_068040/clean/scf7180000084603:85721-85801:+	ACA1_068040	START domain-containing protein [Source:UniProtKB/TrEMBL;Acc:L8HDG1]	0.008771933	0.322200333	0.3134284	3.63E-05
6h	ACA1_230430/clean/scf7180000084761:347673-347754:-	ACA1_230430	RNA recognition motif domain containing protein [Source:UniProtKB/TrEMBL;Acc:L8HAC9]	0	0.313407333	0.313407333	5.15E-07
6h	ACA1_173090/clean/scf7180000084741:399021-399092:+	ACA1_173090	Arginine-tRNA-protein transferase, putative [Source:UniProtKB/TrEMBL;Acc:L8HHK6]	0.004344663	0.317647	0.313302337	2.60E-07
6h	ACA1_258270/clean/scf7180000084769:219009-219104:+	ACA1_258270	Ras subfamily protein [Source:UniProtKB/TrEMBL;Acc:L8GHB6]	0.007089837	0.320383667	0.31329383	1.57E-10
6h	ACA1_289980/clean/scf7180000084777:519909-520012:-	ACA1_289980	RNA polymerase II subunit A C-terminal domain phosphatase [Source:UniProtKB/TrEMBL;Acc:L8HI37]	0	0.313199667	0.313199667	4.72E-05
6h	ACA1_091710/clean/scf7180000084645:187300-187383:-	ACA1_091710	PAS domain Sbox domain containing protein [Source:UniProtKB/TrEMBL;Acc:L8GKF5]	0.008488573	0.321656667	0.313168093	2.75E-12
6h	ACA1_210530/clean/scf7180000084758:50540-50608:-	ACA1_210530	4Fe-4S ferredoxin-type domain-containing protein [Source:UniProtKB/TrEMBL;Acc:L8GS75]	0.0087975	0.321931333	0.313133833	1.07E-06
6h	ACA1_313610/clean/scf7180000083822:11595-11687:+	ACA1_313610	Calcium-transporting ATPase [Source:UniProtKB/TrEMBL;Acc:L8GL52]	0.003528507	0.316557667	0.31302916	1.48E-23
6h	ACA1_172210/clean/scf7180000084741:276289-276388:-	ACA1_172210	DUF3402 domain-containing protein [Source:UniProtKB/TrEMBL;Acc:L8HJU0]	0.023813853	0.336830333	0.31301648	2.45E-05
6h	ACA1_178060/clean/scf7180000084743:159942-160032:-	ACA1_178060	Dipeptidylpeptidase 9, putative [Source:UniProtKB/TrEMBL;Acc:L8GTD8]	0.0147334	0.327748	0.3130146	7.12E-08
6h	ACA1_170940/clean/scf7180000084741:19596-19670:+	ACA1_170940	Proteasome subunit alpha type [Source:UniProtKB/TrEMBL;Acc:L8HH61]	0.006216413	0.319224333	0.31300792	3.76E-26
6h	ACA1_000820/clean/scf7180000082828:9880-9953:+	ACA1_000820	RING-type domain-containing protein [Source:UniProtKB/TrEMBL;Acc:L8H7M3]	0	0.312837333	0.312837333	2.80E-07
6h	ACA1_193520/clean/scf7180000084749:250499-250569:-	ACA1_193520	hypothetical protein	0	0.31282	0.31282	0.000330302
6h	ACA1_325610/clean/scf7180000084495:52119-52237:-	ACA1_325610	Protein kinase domain containing protein [Source:UniProtKB/TrEMBL;Acc:L8H900]	0.008036	0.320855667	0.312819667	2.86E-07
6h	ACA1_264220/clean/scf7180000084770:212956-213018:+	ACA1_264220	hypothetical protein	0.01337337	0.326139333	0.312765963	2.57E-09
6h	ACA1_171430/clean/scf7180000084741:103211-103296:+	ACA1_171430	hypothetical protein	0.018518533	0.331177667	0.312659133	0.00046173
6h	ACA1_285650/clean/scf7180000084776:1018945-1019051:-	ACA1_285650	G-patch domain-containing protein [Source:UniProtKB/TrEMBL;Acc:L8H9W2]	0.040132	0.352739333	0.312607333	0.000218183
6h	ACA1_399200/clean/scf7180000084759:271147-271248:-	ACA1_399200	hypothetical protein	0.001574424	0.314036	0.312461576	2.48E-09
6h	ACA1_180160/clean/scf7180000084744:34838-35013:+	ACA1_180160	hypothetical protein	0.030344833	0.342725667	0.312380833	1.23E-08
6h	ACA1_369250/clean/scf7180000084664:284903-285015:-	ACA1_369250	Dynactin 50 kDa subunit [Source:UniProtKB/TrEMBL;Acc:L8H0A3]	0.008943467	0.321319333	0.312375867	2.47E-06
6h	ACA1_074670/clean/scf7180000084608:61534-61607:-	ACA1_074670	Arginase [Source:UniProtKB/TrEMBL;Acc:L8HFF6]	0.01385898	0.326206667	0.312347687	3.21E-08
6h	ACA1_107610/clean/scf7180000084660:229994-230110:+	ACA1_107610	Aminotransferase, class III superfamily protein [Source:UniProtKB/TrEMBL;Acc:L8GMP2]	0.02662269	0.338896333	0.312273643	4.10E-05
6h	ACA1_031620/clean/scf7180000084522:26885-26972:-	ACA1_031620	Prenylcysteine lyase, putative [Source:UniProtKB/TrEMBL;Acc:L8GS08]	0.134055233	0.446256	0.312200767	0.000104928
6h	ACA1_116200/clean/scf7180000084669:725438-725519:+	ACA1_116200	WASH_WAHD domain-containing protein [Source:UniProtKB/TrEMBL;Acc:L8H3U1]	0.068466433	0.380510333	0.3120439	1.40E-05
6h	ACA1_163660/clean/scf7180000084729:65265-65343:-	ACA1_163660	hypothetical protein	0.009482153	0.321373	0.311890847	2.32E-12
6h	ACA1_158530/clean/scf7180000084724:245652-245735:+	ACA1_158530	Glucose-6-phosphate 1-dehydrogenase [Source:UniProtKB/TrEMBL;Acc:L8HBJ1]	0.0260223	0.33777	0.3117477	0.001063246
6h	ACA1_116180/clean/scf7180000084669:718831-718915:+	ACA1_116180	NFACT-R_1 domain-containing protein [Source:UniProtKB/TrEMBL;Acc:L8H4M4]	0.013940833	0.325594333	0.3116535	5.30E-12
6h	ACA1_325260/clean/scf7180000084475:40178-40271:+	ACA1_325260	Peptidylprolyl isomerase [Source:UniProtKB/TrEMBL;Acc:L8GPH9]	0.001727657	0.313317333	0.311589676	9.61E-10
6h	ACA1_070490/clean/scf7180000084603:701074-701178:+	ACA1_070490	Phospholipase D, putative [Source:UniProtKB/TrEMBL;Acc:L8HG70]	0.0187261	0.330315667	0.311589567	1.22E-06
6h	ACA1_257170/clean/scf7180000084769:139618-139712:+	ACA1_257170	hypothetical protein	0.013430092	0.325008333	0.311578241	0.000113241
6h	ACA1_027500/clean/scf7180000084469:5711-5791:-	ACA1_027500	hypothetical protein	0.00373578	0.315293333	0.311557553	1.37E-07
6h	ACA1_203020/clean/scf7180000084754:123698-123792:-	ACA1_203020	Protein pns1, putative [Source:UniProtKB/TrEMBL;Acc:L8GUJ1]	0.00086954	0.312401	0.31153146	2.56E-10
6h	ACA1_186060/clean/scf7180000084746:160727-160849:-	ACA1_186060	Dual specificity phosphatase, catalytic domain containing protein [Source:UniProtKB/TrEMBL;Acc:L8H5A1]	0.00915717	0.320663	0.31150583	3.88E-07
6h	ACA1_296250/clean/scf7180000084777:1306540-1306676:+	ACA1_296250	Rasrelated protein Rab-32, putative [Source:UniProtKB/TrEMBL;Acc:L8HJZ6]	0.024594299	0.335982	0.311387701	4.14E-09
6h	ACA1_058730/clean/scf7180000084599:365546-365626:+	ACA1_058730	Tetratricopeptide repeat domain containing protein [Source:UniProtKB/TrEMBL;Acc:L8GWA7]	0.115719367	0.427107	0.311387633	3.50E-06
6h	ACA1_040880/clean/scf7180000084572:99555-99627:-	ACA1_040880	BAH domain-containing protein [Source:UniProtKB/TrEMBL;Acc:L8H0M7]	0.001814117	0.313123333	0.311309217	3.17E-07
6h	ACA1_198820/clean/scf7180000084753:161488-161572:+	ACA1_198820	Sec34 family protein [Source:UniProtKB/TrEMBL;Acc:L8H5C8]	0	0.311237	0.311237	9.83E-07
6h	ACA1_072950/clean/scf7180000084603:1343818-1343894:+	ACA1_072950	Peptidase M16 inactive domain containing protein [Source:UniProtKB/TrEMBL;Acc:L8HGW3]	0	0.311222	0.311222	7.30E-05
6h	ACA1_029510/clean/scf7180000084477:871-950:-	ACA1_029510	Leucine rich repeat containing protein [Source:UniProtKB/TrEMBL;Acc:L8H9E3]	0	0.311187667	0.311187667	4.39E-08
6h	ACA1_133480/clean/scf7180000084701:64015-64075:+	ACA1_133480	hypothetical protein	0.011751373	0.322865333	0.31111396	1.97E-10
6h	ACA1_086290/clean/scf7180000084636:32991-33073:+	ACA1_086290	hypothetical protein	0.005435567	0.316522333	0.311086767	0.000105921
6h	ACA1_089040/clean/scf7180000084639:304449-304525:+	ACA1_089040	UBA/TS-N domain containing protein [Source:UniProtKB/TrEMBL;Acc:L8GUR2]	0.012955157	0.323977667	0.31102251	1.60E-07
6h	ACA1_050510/clean/scf7180000084589:109705-109774:+	ACA1_050510	4HBT domain-containing protein [Source:UniProtKB/TrEMBL;Acc:L8H578]	0.006861459	0.317878333	0.311016874	5.76E-11
6h	ACA1_368140/clean/scf7180000084664:66573-66650:-	ACA1_368140	Glutamine amidotransferase type-2 domain-containing protein [Source:UniProtKB/TrEMBL;Acc:L8H040]	0	0.311001667	0.311001667	0.001263198
6h	ACA1_396940/clean/scf7180000084759:153575-153750:-	ACA1_396940	OST3 / OST6 family protein [Source:UniProtKB/TrEMBL;Acc:L8HDM5]	0.011651773	0.32261	0.310958227	6.69E-13
6h	ACA1_130550/clean/scf7180000084698:106137-106229:-	ACA1_130550	WH2 domain-containing protein [Source:UniProtKB/TrEMBL;Acc:L8GPE2]	0.017244933	0.32818	0.310935067	2.23E-07
6h	ACA1_072810/clean/scf7180000084603:1301123-1301213:+	ACA1_072810	Aha1_N domain-containing protein [Source:UniProtKB/TrEMBL;Acc:L8HG06]	0.000806288	0.311712333	0.310906045	5.90E-14
6h	ACA1_216930/clean/scf7180000084758:525067-525192:-	ACA1_216930	Rab11/RabAfamily small GTPase [Source:UniProtKB/TrEMBL;Acc:L8GSR3]	0.003424465	0.314322333	0.310897868	7.72E-10
6h	ACA1_184310/clean/scf7180000084745:616024-616134:+	ACA1_184310	DEAD/DEAH box helicase domain containing protein [Source:UniProtKB/TrEMBL;Acc:L8H8J0]	0.385017667	0.695914333	0.310896667	0.03047813
6h	ACA1_385810/clean/scf7180000084730:270731-270804:+	ACA1_385810	Rap-GAP domain-containing protein [Source:UniProtKB/TrEMBL;Acc:L8HAQ1]	0.00617025	0.317031	0.31086075	7.20E-12
6h	ACA1_115860/clean/scf7180000084669:689277-689367:-	ACA1_115860	NOT2 / NOT3 / NOT5 family protein [Source:UniProtKB/TrEMBL;Acc:L8H4R3]	0.0304048	0.341264667	0.310859867	5.67E-08
6h	ACA1_038980/clean/scf7180000084569:12015-12115:-	ACA1_038980	Palmitoyl protein thioesterase, putative [Source:UniProtKB/TrEMBL;Acc:L8HJ32]	0.010208007	0.321024	0.310815993	2.92E-11
6h	ACA1_022330/clean/scf7180000084293:8675-8788:+	ACA1_022330	Carbamoylphosphate synthase L chain, ATP-binding, putative [Source:UniProtKB/TrEMBL;Acc:L8GNZ7]	1.72E-05	0.310706333	0.31068912	8.37E-14
6h	ACA1_236350/clean/scf7180000084762:443297-443380:+	ACA1_236350	Peptidase_M28 domain-containing protein [Source:UniProtKB/TrEMBL;Acc:L8H1H1]	0.002881775	0.313472333	0.310590559	1.14E-10
6h	ACA1_035310/clean/scf7180000084564:19335-19433:+	ACA1_035310	AMP-binding domain-containing protein [Source:UniProtKB/TrEMBL;Acc:L8HCU7]	0.010405467	0.320945	0.310539533	1.12E-05
6h	ACA1_219070/clean/scf7180000084758:711442-711524:+	ACA1_219070	Rieske domain-containing protein [Source:UniProtKB/TrEMBL;Acc:L8GQP6]	0.000236038	0.310723667	0.310487628	1.92E-11
6h	ACA1_382620/clean/scf7180000084721:86613-86695:+	ACA1_382620	SERPIN domain-containing protein [Source:UniProtKB/TrEMBL;Acc:L8GV22]	0.02573385	0.336137333	0.310403483	6.67E-09
6h	ACA1_063490/clean/scf7180000084599:1429650-1429747:-	ACA1_063490	Peptidylprolyl isomerase [Source:UniProtKB/TrEMBL;Acc:L8GX87]	0.028528533	0.338739	0.310210467	0.040566892
6h	ACA1_093150/clean/scf7180000084645:496119-496206:+	ACA1_093150	PAS domain Sbox domain containing protein [Source:UniProtKB/TrEMBL;Acc:L8GIU7]	0.113099133	0.423279667	0.310180533	0.000669842
6h	ACA1_252210/clean/scf7180000084768:145823-145907:+	ACA1_252210	Protein kinase domain-containing protein [Source:UniProtKB/TrEMBL;Acc:L8HAM0]	0.00101044	0.311130667	0.310120227	2.56E-07
6h	ACA1_229530/clean/scf7180000084761:269073-269175:+	ACA1_229530	Diacylglycerol kinase [Source:UniProtKB/TrEMBL;Acc:L8H8Z8]	0.00327096	0.313359667	0.310088707	2.41E-08
6h	ACA1_107270/clean/scf7180000084660:167627-167730:-	ACA1_107270	AAA domain-containing protein [Source:UniProtKB/TrEMBL;Acc:L8GQA9]	0.000120435	0.310165	0.310044565	5.50E-09
6h	ACA1_034530/clean/scf7180000084563:64912-64984:+	ACA1_034530	hypothetical protein	0.005376333	0.315411333	0.310035	2.21E-06
6h	ACA1_089130/clean/scf7180000084639:327898-327974:+	ACA1_089130	Beta-lactamase domain-containing protein [Source:UniProtKB/TrEMBL;Acc:L8GX66]	0.006184173	0.316154667	0.309970493	1.36E-07
6h	ACA1_321190/clean/scf7180000084437:9774-9860:-	ACA1_321190	DUF1681 domain-containing protein [Source:UniProtKB/TrEMBL;Acc:L8GG94]	8.27E-05	0.309979	0.309896266	1.40E-12
6h	ACA1_018760/clean/scf7180000083912:11268-11348:+	ACA1_018760	Cell cycle control protein [Source:UniProtKB/TrEMBL;Acc:L8HJG5]	0.012604667	0.322483667	0.309879	5.49E-19
6h	ACA1_055410/clean/scf7180000084594:699015-699086:+	ACA1_055410	Protein kinase domain-containing protein [Source:UniProtKB/TrEMBL;Acc:L8H6J6]	0.0266423	0.336316	0.3096737	8.83E-08
6h	ACA1_027830/clean/scf7180000084471:31003-31074:-	ACA1_027830	DH domain-containing protein [Source:UniProtKB/TrEMBL;Acc:L8HML0]	0.000784703	0.310385	0.309600297	6.16E-10
6h	ACA1_012020/clean/scf7180000083652:5181-5290:-	ACA1_012020	hypothetical protein	0.0024976	0.312049	0.3095514	5.98E-09
6h	ACA1_012990/clean/scf7180000083742:17999-18072:+	ACA1_012990	ATP-dependent DNA helicase [Source:UniProtKB/TrEMBL;Acc:L8H692]	0	0.309522	0.309522	1.17E-05
6h	ACA1_135140/clean/scf7180000084702:73263-73337:+	ACA1_135140	hypothetical protein	0.137724	0.447034	0.30931	0.000394523
6h	ACA1_296850/clean/scf7180000084777:1416411-1416507:+	ACA1_296850	UBIQUITIN_CONJUGAT_2 domain-containing protein [Source:UniProtKB/TrEMBL;Acc:L8HK35]	0.026582567	0.335877333	0.309294767	1.70E-12
6h	ACA1_246490/clean/scf7180000084763:555005-555103:-	ACA1_246490	Aldo_ket_red domain-containing protein [Source:UniProtKB/TrEMBL;Acc:L8GMP1]	0.066209363	0.375466333	0.30925697	0.000716195
6h	ACA1_061120/clean/scf7180000084599:822855-822978:-	ACA1_061120	Leucine rich repeat domain containing protein [Source:UniProtKB/TrEMBL;Acc:L8GVW2]	0.024580067	0.333813	0.309232933	0.000143073
6h	ACA1_277400/clean/scf7180000084776:23241-23309:-	ACA1_277400	RFX-type winged-helix domain-containing protein [Source:UniProtKB/TrEMBL;Acc:L8H6F5]	0.045563967	0.354750667	0.3091867	0.000752455
6h	ACA1_103330/clean/scf7180000084653:28712-28798:-	ACA1_103330	hypothetical protein	0	0.309153333	0.309153333	1.22E-05
6h	ACA1_252610/clean/scf7180000084768:188867-188966:-	ACA1_252610	ADPribosylglycohydrolase superfamily protein [Source:UniProtKB/TrEMBL;Acc:L8HD40]	0.003440583	0.312567333	0.30912675	7.41E-07
6h	ACA1_013390/clean/scf7180000083750:12035-12124:-	ACA1_013390	Aamy domain-containing protein [Source:UniProtKB/TrEMBL;Acc:L8HGK3]	0.001164007	0.310132333	0.308968327	1.30E-09
6h	ACA1_130620/clean/scf7180000084698:123820-123918:-	ACA1_130620	Rho-GAP domain-containing protein [Source:UniProtKB/TrEMBL;Acc:L8GPL1]	0.052382033	0.361336333	0.3089543	0.000193622
6h	ACA1_264730/clean/scf7180000084770:369210-369298:-	ACA1_264730	hypothetical protein	0.028146067	0.337014667	0.3088686	3.79E-05
6h	ACA1_164710/clean/scf7180000084729:257838-257991:-	ACA1_164710	Ribosomal_L23eN domain-containing protein [Source:UniProtKB/TrEMBL;Acc:L8GRL5]	0.001637442	0.310406333	0.308768891	1.91E-39
6h	ACA1_057960/clean/scf7180000084599:217683-217738:+	ACA1_057960	BTB/POZ domain containing protein [Source:UniProtKB/TrEMBL;Acc:L8GVX1]	0	0.3087565	0.3087565	1.27E-06
6h	ACA1_144250/clean/scf7180000084709:185569-185706:-	ACA1_144250	SH2 domain containing protein [Source:UniProtKB/TrEMBL;Acc:L8HEU2]	0.001626017	0.310256333	0.308630317	6.29E-10
6h	ACA1_012330/clean/scf7180000083740:19290-19375:+	ACA1_012330	WD_REPEATS_REGION domain-containing protein [Source:UniProtKB/TrEMBL;Acc:L8HCD7]	0.010018613	0.318615667	0.308597053	1.64E-08
6h	ACA1_141800/clean/scf7180000084708:25029-25143:-	ACA1_141800	hypothetical protein	0.026102733	0.334619	0.308516267	4.99E-10
6h	ACA1_214940/clean/scf7180000084758:269962-270064:-	ACA1_214940	Leucine rich repeat domain containing protein [Source:UniProtKB/TrEMBL;Acc:L8GQ02]	0.0138889	0.322367667	0.308478767	7.55E-05
6h	ACA1_045070/clean/scf7180000084576:73165-73266:-	ACA1_045070	SAYSvFN domain-containing protein [Source:UniProtKB/TrEMBL;Acc:L8GZT3]	0.01280684	0.321197333	0.308390493	1.58E-09
6h	ACA1_158530/clean/scf7180000084724:245296-245369:+	ACA1_158530	Glucose-6-phosphate 1-dehydrogenase [Source:UniProtKB/TrEMBL;Acc:L8HBJ1]	0.0247619	0.333022333	0.308260433	0.001311957

6h	ACA1_362640/clean/scf7180000084624:121647-121868:+	ACA1_362640	H/ACA ribonucleoprotein complex subunit [Source:UniProtKB/TrEMBL;Acc:L8GGA8]	0.002149227	0.310227667	0.30807844	1.61E-14
6h	ACA1_205990/clean/scf7180000084755:59856-59931:-	ACA1_205990	Histidine acid phosphatase superfamily protein [Source:UniProtKB/TrEMBL;Acc:L8H5L5]	0	0.308019333	0.308019333	6.14E-09
6h	ACA1_260040/clean/scf7180000084769:410220-410329:+	ACA1_260040	Membrane magnesium transporter [Source:UniProtKB/TrEMBL;Acc:L8GF04]	0.053920733	0.361872667	0.307951933	3.94E-06
6h	ACA1_301570/clean/scf7180000083222:10779-10887:-	ACA1_301570	FCP1 homology domain-containing protein [Source:UniProtKB/TrEMBL;Acc:L8HKQ0]	0.009103482	0.317005	0.307901518	2.43E-13
6h	ACA1_396170/clean/scf7180000084759:46000-46127:-	ACA1_396170	Phosphatidylserine decarboxylase proenzyme 2 [Source:UniProtKB/TrEMBL;Acc:L8HEJ4]	0.003031852	0.310883333	0.307851481	5.38E-19
6h	ACA1_309500/clean/scf7180000083762:9901-9988:+	ACA1_309500	Pyridine nucleotidedisulfide oxidoreductase domain containing protein [Source:UniProtKB/TrEMBL;Acc:L8GI23]	0.018779033	0.326599333	0.3078203	1.82E-12
6h	ACA1_002490/clean/scf7180000082931:7570-7674:-	ACA1_002490	AA_TRNA_LIGASE_II_ALA domain-containing protein [Source:UniProtKB/TrEMBL;Acc:L8GVN5]	0	0.307758667	0.307758667	2.96E-08
6h	ACA1_087400/clean/scf7180000084639:24026-24114:+	ACA1_087400	Ras subfamily protein [Source:UniProtKB/TrEMBL;Acc:L8GVE2]	0.037204433	0.344945	0.307740567	0.006365262
6h	ACA1_376560/clean/scf7180000084685:107515-107600:+	ACA1_376560	Ankyrin repeatcontaining protein [Source:UniProtKB/TrEMBL;Acc:L8HHK7]	0.040802167	0.348533667	0.3077315	1.03E-07
6h	ACA1_285210/clean/scf7180000084776:961182-961325:-	ACA1_285210	Protein kinase domain containing protein [Source:UniProtKB/TrEMBL;Acc:L8H7H7]	0.010667262	0.318396333	0.307729071	2.05E-08
6h	ACA1_070640/clean/scf7180000084603:752528-752690:+	ACA1_070640	RNA recognition motif domain containing protein [Source:UniProtKB/TrEMBL;Acc:L8HE36]	0.00951736	0.317143	0.30762564	3.81E-16
6h	ACA1_234000/clean/scf7180000084762:181511-181600:+	ACA1_234000	Purple acid phosphatase [Source:UniProtKB/TrEMBL;Acc:L8H162]	0	0.307572333	0.307572333	1.42E-15
6h	ACA1_287540/clean/scf7180000084777:32340-32429:-	ACA1_287540	Snurportin1 domain-containing protein [Source:UniProtKB/TrEMBL;Acc:L8HIM9]	0.0347155	0.342224667	0.307509167	4.91E-06
6h	ACA1_175550/clean/scf7180000084741:992901-992973:+	ACA1_175550	AP4E_app_platf domain-containing protein [Source:UniProtKB/TrEMBL;Acc:L8HI53]	0.00110323	0.308545	0.30744177	5.06E-09
6h	ACA1_199500/clean/scf7180000084753:249429-249541:+	ACA1_199500	1acylglycerophosphocholine O-acyltransferase 1, putative [Source:UniProtKB/TrEMBL;Acc:L8H4L3]	0.0552084	0.362629333	0.307420933	0.002302032
6h	ACA1_106810/clean/scf7180000084660:135709-135786:-	ACA1_106810	FHA domain containing protein [Source:UniProtKB/TrEMBL;Acc:L8GML7]	0.015550527	0.322969	0.307418473	3.00E-11
6h	ACA1_158940/clean/scf7180000084724:301716-301854:+	ACA1_158940	Oxidoreductase [Source:UniProtKB/TrEMBL;Acc:L8HBL7]	0.003743899	0.311144333	0.307400434	3.64E-07
6h	ACA1_071830/clean/scf7180000084603:1075724-1075837:+	ACA1_071830	Beige/BEACH domain containing protein [Source:UniProtKB/TrEMBL;Acc:L8HGK4]	0.024061577	0.33142	0.307358423	4.58E-06
6h	ACA1_142670/clean/scf7180000084708:216978-217353:-	ACA1_142670	Zinc finger, c2h2 type domain containing protein [Source:UniProtKB/TrEMBL;Acc:L8HAM8]	0.1269383	0.434270667	0.307332367	0.001031118
6h	ACA1_325160/clean/scf7180000084475:13430-13504:+	ACA1_325160	Rab7/RabGfamily small GTPase [Source:UniProtKB/TrEMBL;Acc:L8GRY8]	0.029795767	0.33712	0.307324233	2.59E-09
6h	ACA1_031950/clean/scf7180000084523:40094-40180:+	ACA1_031950	CBS domain containing protein [Source:UniProtKB/TrEMBL;Acc:L8H414]	0.0390388	0.346354	0.3073152	1.52E-05
6h	ACA1_314440/clean/scf7180000083843:13552-13914:-	ACA1_314440	Protein kinase domain-containing protein [Source:UniProtKB/TrEMBL;Acc:L8HEY9]	0.00414312	0.311418	0.30727488	3.04E-07
6h	ACA1_073230/clean/scf7180000084603:1377131-1377188:-	ACA1_073230	EHN domain-containing protein [Source:UniProtKB/TrEMBL;Acc:L8HDT9]	0	0.307199333	0.307199333	1.45E-08
6h	ACA1_219870/clean/scf7180000084758:830046-830154:-	ACA1_219870	Lysophospholipase 3 (Lysosomal phospholipase A2) isoform 7, putative [Source:UniProtKB/TrEMBL;Acc:L8GT04]	0.000393893	0.307572667	0.307178773	6.50E-09
6h	ACA1_372720/clean/scf7180000084679:62815-62908:+	ACA1_372720	Obtusifoliol 14alphademethylase, putative [Source:UniProtKB/TrEMBL;Acc:L8GJB3]	0.007404343	0.314575667	0.307171323	1.11E-14
6h	ACA1_208170/clean/scf7180000084757:117779-117868:+	ACA1_208170	hypothetical protein	0.006944433	0.314049	0.307104567	9.11E-06
6h	ACA1_133320/clean/scf7180000084701:31451-31523:+	ACA1_133320	Homeobox domain-containing protein [Source:UniProtKB/TrEMBL;Acc:L8H2R6]	0.005537166	0.312626	0.307088834	1.50E-07
6h	ACA1_070800/clean/scf7180000084603:762930-763046:+	ACA1_070800	Transmembrane protein 33 family protein, putative [Source:UniProtKB/TrEMBL;Acc:L8HG91]	0.005365407	0.31239	0.307024593	2.81E-05
6h	ACA1_282720/clean/scf7180000084776:725452-725540:-	ACA1_282720	Leucine rich repeat domain containing protein [Source:UniProtKB/TrEMBL;Acc:L8H9L8]	0.00670107	0.313702667	0.307001597	1.33E-06
6h	ACA1_101530/clean/scf7180000084649:87522-87585:-	ACA1_101530	6phosphogluconate dehydrogenase NAD-binding domain containing protein [Source:UniProtKB/TrEMBL;Acc:L8GGL6]	0	0.306950667	0.306950667	6.30E-12
6h	ACA1_369710/clean/scf7180000084664:366147-366252:+	ACA1_369710	Hypothetical protein	0.017951533	0.324859033	0.3069075	0.000653724
6h	ACA1_370080/clean/scf7180000084664:433686-433787:-	ACA1_370080	ATPdependent RNA helicase Ddx1, putative [Source:UniProtKB/TrEMBL;Acc:L8GZ36]	0.014062	0.320965	0.306903	0.000181279
6h	ACA1_244870/clean/scf7180000084763:446739-446804:+	ACA1_244870	DNA polymerase [Source:UniProtKB/TrEMBL;Acc:L8GK72]	0.028330933	0.335202333	0.3068714	1.41E-05
6h	ACA1_189420/clean/scf7180000084748:34009-34110:+	ACA1_189420	Protein disulfideisomerase domain containing protein [Source:UniProtKB/TrEMBL;Acc:L8GE28]	0.0292835	0.336117667	0.306834167	3.48E-13
6h	ACA1_323530/clean/scf7180000084468:43087-43186:+	ACA1_323530	Fbox domain containing protein [Source:UniProtKB/TrEMBL;Acc:L8H0X5]	0.008192963	0.315022333	0.30682937	0.000332911
6h	ACA1_070350/clean/scf7180000084603:649556-649679:-	ACA1_070350	PKS_ER domain-containing protein [Source:UniProtKB/TrEMBL;Acc:L8HDW9]	0.00317329	0.309993667	0.306820377	9.00E-15
6h	ACA1_292830/clean/scf7180000084777:910756-910822:+	ACA1_292830	CULLIN_2 domain-containing protein [Source:UniProtKB/TrEMBL;Acc:L8HJK9]	0.00124409	0.308004667	0.306760577	2.41E-08
6h	ACA1_095680/clean/scf7180000084645:880496-880579:+	ACA1_095680	hypothetical protein	0	0.306729333	0.306729333	1.21E-08
6h	ACA1_151330/clean/scf7180000084716:105769-105873:-	ACA1_151330	Purple acid phosphatase [Source:UniProtKB/TrEMBL;Acc:L8H0Q3]	0.008098207	0.314795	0.306696793	3.15E-13
6h	ACA1_055340/clean/scf7180000084594:686288-686355:+	ACA1_055340	Autophagy related protein 2 (ATG2), putative [Source:UniProtKB/TrEMBL;Acc:L8H7Q9]	0.022399467	0.329026667	0.3066272	1.95E-06
6h	ACA1_280650/clean/scf7180000084776:518363-518452:-	ACA1_280650	Aldedh domain-containing protein [Source:UniProtKB/TrEMBL;Acc:L8H998]	0.002731433	0.309274333	0.3065429	2.50E-16
6h	ACA1_301720/clean/scf7180000083222:28463-28525:+	ACA1_301720	Amidohydro-rel domain-containing protein [Source:UniProtKB/TrEMBL;Acc:L8HKQ5]	0.014474677	0.320946667	0.30647199	4.02E-13
6h	ACA1_173140/clean/scf7180000084741:410426-410498:+	ACA1_173140	Deacetylase sirtuin-type domain-containing protein [Source:UniProtKB/TrEMBL;Acc:L8HJX9]	0	0.306399	0.306399	3.92E-08
6h	ACA1_199830/clean/scf7180000084753:345039-345137:-	ACA1_199830	Glycine cleavage system Pprotein [Source:UniProtKB/TrEMBL;Acc:L8H387]	0.001526824	0.307907333	0.30638051	1.45E-11
6h	ACA1_368560/clean/scf7180000084664:128461-128572:-	ACA1_368560	Protein kinase domain-containing protein [Source:UniProtKB/TrEMBL;Acc:L8GY18]	0.029713033	0.336062	0.306348967	2.01E-09
6h	ACA1_387910/clean/scf7180000084733:86416-86560:-	ACA1_387910	P/Homo B domain-containing protein [Source:UniProtKB/TrEMBL;Acc:L8GG58]	0.003787867	0.310116333	0.306328467	4.41E-07
6h	ACA1_236630/clean/scf7180000084762:462944-463028:-	ACA1_236630	PX domain-containing protein [Source:UniProtKB/TrEMBL;Acc:L8H1G0]	0.053974283	0.360220333	0.30624605	0.000194608
6h	ACA1_229510/clean/scf7180000084761:262333-262409:+	ACA1_229510	Methyltranfer_dom domain-containing protein [Source:UniProtKB/TrEMBL;Acc:L8HAA4]	0.016472922	0.322624333	0.306151412	7.69E-05
6h	ACA1_325130/clean/scf7180000084475:7524-7604:-	ACA1_325130	hypothetical protein	0.001321869	0.307433667	0.306111797	1.35E-10
6h	ACA1_382270/clean/scf7180000084721:15803-15892:-	ACA1_382270	ATPase/histidine kinase/DNA gyrase B/HSP90 domain containing protein [Source:UniProtKB/TrEMBL;Acc:L8GU96]	0.034137133	0.340239	0.306101867	0.000463933
6h	ACA1_288730/clean/scf7180000084777:273290-273390:-	ACA1_288730	Esterase, PHB depolymerase subfamily protein [Source:UniProtKB/TrEMBL;Acc:L8HIW4]	0	0.306074	0.306074	5.45E-09
6h	ACA1_362670/clean/scf7180000084624:135657-135740:+	ACA1_362670	Rieske domain-containing protein [Source:UniProtKB/TrEMBL;Acc:L8GHW3]	0.004727283	0.310765333	0.30603805	4.41E-09
6h	ACA1_393050/clean/scf7180000084756:32961-33101:-	ACA1_393050	RhoGEF domain containing protein [Source:UniProtKB/TrEMBL;Acc:L8GZK0]	0.012277503	0.318242667	0.305965163	2.24E-06
6h	ACA1_287570/clean/scf7180000084777:38251-38327:+	ACA1_287570	hypothetical protein	0.003745753	0.309707333	0.30596158	3.32E-08
6h	ACA1_388490/clean/scf7180000084733:125549-125622:+	ACA1_388490	ANK_REP_REGION domain-containing protein [Source:UniProtKB/TrEMBL;Acc:L8GEA1]	0.039715833	0.345585333	0.3058695	6.43E-05
6h	ACA1_054840/clean/scf7180000084594:591203-591285:-	ACA1_054840	Cytochrome p450 superfamily protein [Source:UniProtKB/TrEMBL;Acc:L8H7M8]	0.240636667	0.546458333	0.305821667	0.025462386
6h	ACA1_064710/clean/scf7180000084599:1676510-1676571:-	ACA1_064710	hypothetical protein	0.018242767	0.323990667	0.3057479	1.57E-05
6h	ACA1_074930/clean/scf7180000084608:115506-115588:+	ACA1_074930	Calponin domain containing protein [Source:UniProtKB/TrEMBL;Acc:L8HHK0]	0.011384767	0.317079667	0.3056949	1.10E-06
6h	ACA1_230570/clean/scf7180000084761:379321-379410:-	ACA1_230570	DUF4139 domain-containing protein [Source:UniProtKB/TrEMBL;Acc:L8H928]	0.0065497	0.312207333	0.305657633	6.87E-09
6h	ACA1_264550/clean/scf7180000084770:318678-318759:+	ACA1_264550	Cys/Met metabolism PLPdependent enzyme superfamily protein [Source:UniProtKB/TrEMBL;Acc:L8H496]	0.001302073	0.306954	0.305651927	1.22E-08
6h	ACA1_117320/clean/scf7180000084669:975172-975259:-	ACA1_117320	FAD binding domain containing protein [Source:UniProtKB/TrEMBL;Acc:L8H415]	0.015266478	0.320875333	0.305608856	3.83E-10
6h	ACA1_307510/clean/scf7180000083680:21664-21741:-	ACA1_307510	hypothetical protein	0.008196733	0.313802667	0.305605933	2.79E-08
6h	ACA1_277990/clean/scf7180000084776:148063-148141:+	ACA1_277990	Calponin domain containing protein [Source:UniProtKB/TrEMBL;Acc:L8H800]	0	0.3056	0.3056	0.000517947
6h	ACA1_048110/clean/scf7180000084582:21937-22110:-	ACA1_048110	Serine carboxypeptidase s28 protein [Source:UniProtKB/TrEMBL;Acc:L8GD90]	0.007764377	0.313290333	0.305525957	1.72E-16
6h	ACA1_279570/clean/scf7180000084776:416051-416118:+	ACA1_279570	Elp3 domain-containing protein [Source:UniProtKB/TrEMBL;Acc:L8H759]	0.274396	0.579889667	0.305493667	0.008073469
6h	ACA1_054220/clean/scf7180000084594:433735-433820:-	ACA1_054220	MHCK/EF2 kinase domain containing protein [Source:UniProtKB/TrEMBL;Acc:L8H897]	0.078057433	0.383523333	0.3054659	5.80E-07
6h	ACA1_169060/clean/scf7180000084739:13168-13262:-	ACA1_169060	Derlin [Source:UniProtKB/TrEMBL;Acc:L8HHS6]	0.012366263	0.317830667	0.305464403	3.99E-10
6h	ACA1_031600/clean/scf7180000084522:23623-23685:-	ACA1_031600	Leucine rich repeat-containing protein [Source:UniProtKB/TrEMBL;Acc:L8GSM8]	0.001684717	0.307105	0.305420283	2.90E-07
6h	ACA1_088090/clean/scf7180000084639:156656-156714:-	ACA1_088090	Retinol dehydrogenase 14 (All-trans and 9-cis) family protein [Source:UniProtKB/TrEMBL;Acc:L8GTX6]	0.038566433	0.343858333	0.3052919	5.33E-05
6h	ACA1_192070/clean/scf7180000084749:93937-94059:+	ACA1_192070	hypothetical protein	0.014526767	0.3198	0.305273233	7.37E-14
6h	ACA1_172910/clean/scf7180000084741:354580-354676:-	ACA1_172910	Chloride channel protein [Source:UniProtKB/TrEMBL;Acc:L8HGK6]	0.013960407	0.319133333	0.305172927	1.22E-07
6h	ACA1_232800/clean/scf7180000084762:70416-70514:-	ACA1_232800	M20_dimer domain-containing protein [Source:UniProtKB/TrEMBL;Acc:L8H2E1]	0.033515867	0.3386834	0.305167533	8.52E-07
6h	ACA1_163650/clean/scf7180000084729:63783-63891:+	ACA1_163650	hypothetical protein	0.000133333	0.305290333	0.305157	4.18E-07
6h	ACA1_261980/clean/scf7180000084770:12567-12661:+	ACA1_261980	Defective in cullin neddylation protein [Source:UniProtKB/TrEMBL;Acc:L8H1R9]	0.049198467	0.354349667	0.3051512	5.68E-08
6h	ACA1_309500/clean/scf7180000083762:11093-11172:+	ACA1_309500	Pyridine nucleotidedisulfide oxidoreductase domain containing protein [Source:UniProtKB/TrEMBL;Acc:L8GI23]	0.01133141	0.316349	0.30501759	4.48E-14
6h	ACA1_158530/clean/scf7180000084724:243842-243956:+	ACA1_158530	Glucose-6-phosphate 1-dehydrogenase [Source:UniProtKB/TrEMBL;Acc:L8HBJ1]	0	0.305007	0.305007	6.37E-05
6h	ACA1_028680/clean/scf7180000084473:8610-8710:+	ACA1_028680	Protein kinase domain-containing protein [Source:UniProtKB/TrEMBL;Acc:L8GU00]	0.021169367	0.326163	0.304993633	0.000366338
6h	ACA1_325420/clean/scf7180000084495:4408-4531:+	ACA1_325420	Nicotinate-nucleotide pyrophosphorylase [carboxylating] [Source:UniProtKB/TrEMBL;Acc:L8H9V7]	0.006460313	0.311357333	0.30489702	1.35E-13
6h	ACA1_268430/clean/scf7180000084770:905691-905838:-	ACA1_268430	SAM domain (Sterile alpha motif) domain containing protein [Source:UniProtKB/TrEMBL;Acc:L8H2M3]	0.066429633	0.371279333	0.3048497	0.000215949
6h	ACA1_285200/clean/scf7180000084776:956203-956288:-	ACA1_285200	Na_H_Exchanger domain-containing protein [Source:UniProtKB/TrEMBL;Acc:L8H9U2]	0.010408647	0.315093333	0.304684687	1.15E-09
6h	ACA1_184120/clean/scf7180000084745:565534-565605:-	ACA1_184120	hypothetical protein	0.000672343	0.305342333	0.30466999	9.46E-10
6h	ACA1_201180/clean/scf7180000084753:500184-500268:-	ACA1_201180	hypothetical protein	0.011697957	0.316356667	0.30465871	5.08E-10
6h	ACA1_368250/clean/scf7180000084664:89375-89466:-	ACA1_368250	BPI2 domain-containing protein [Source:UniProtKB/TrEMBL;Acc:L8GY12]	0	0.304632667	0.304632667	5.86E-09
6h	ACA1_158840/clean/scf7180000084724:275704-275767:-	ACA1_158840	HD domain-containing protein [Source:UniProtKB/TrEMBL;Acc:L8H9D5]	0	0.304621667	0.304621667	0.017614292
6h	ACA1_285540/clean/scf7180000084776:995351-995423:-	ACA1_285540	GPS domain-containing protein [Source:UniProtKB/TrEMBL;Acc:L8H7T4]	0	0.30458	0.30458	3.01E-05
6h	ACA1_191880/clean/scf7180000084749:63112-63222:+	ACA1_191880	Calponin domain containing protein [Source:UniProtKB/TrEMBL;Acc:L8GP96]	0.000700918	0.305133	0.304432082	3.19E-12
6h	ACA1_040760/clean/scf7180000084572:76175-76268:-	ACA1_040760	SNF7 family protein [Source:UniProtKB/TrEMBL;Acc:L8H0J8]	0.00347524	0.307846333	0.304371093	1.70E-09
6h	ACA1_306840/clean/scf7180000083668:19063-19166:+	ACA1_306840	RTP1_C1 domain-containing protein [Source:UniProtKB/TrEMBL;Acc:L8GQF6]	0.00304414	0.307394333	0.304350193	5.92E-06
6h	ACA1_068620/clean/scf7180000084603:240525-240638:-	ACA1_068620	hypothetical protein	0	0.304299667	0.304299667	8.31E-08
6h	ACA1_146500/clean/scf7180000084713:35021-35116:-	ACA1_146500	DH domain-containing protein [Source:UniProtKB/TrEMBL;Acc:L8GWP3]	0.001277804	0.305563667	0.304285862	6.94E-09
6h	ACA1_171430/clean/scf7180000084741:102473-102603:+	ACA1_171430	hypothetical protein	0.010995167	0.315258667	0.3042635	3.14E-05
6h	ACA1_185580/clean/scf7180000084746:110623-110715:+	ACA1_185580	FYVE-type domain-containing protein [Source:UniProtKB/TrEMBL;Acc:L8H486]	0.01814876	0.322407667	0.304258907	1.19E-07
6h	ACA1_071930/clean/scf7180000084603:1111870-1111938:+	ACA1_071930	hypothetical protein	0.0098771	0.314098333	0.304221233	4.73E-06
6h	ACA1_011280/clean/scf7180000083623:9478-9593:+	ACA1_011280	AAA domain-containing protein [Source:UniProtKB/TrEMBL;Acc:L8GP61]	0.0015873	0.305784667	0.304197367	7.08E-09
6h	ACA1_322580/clean/scf7180000084466:23648-23757:+	ACA1_322580	Calmodulin, putative [Source:UniProtKB/TrEMBL;Acc:L8HAA1]	0.0094034	0.313597333	0.304193933	1.44E-10
6h	ACA1_315790/clean/scf7180000083943:8548-8618:-	ACA1_315790	Copper transport accessory protein [Source:UniProtKB/TrEMBL;Acc:L8HDX3]	0.004973073	0.309103	0.304129927	5.51E-13
6h	ACA1_325500/clean/scf7180000084495:21736-21872:+	ACA1_325500	Usp domain-containing protein [Source:UniProtKB/TrEMBL;Acc:L8HB68]	0.013344263	0.317464667	0.304120403	1.71E-12
6h	ACA1_184600/clean/scf7180000084746:5438-5520:-	ACA1_184600	Alpha-N-acetylglucosaminidase family protein [Source:UniProtKB/TrEMBL;Acc:L8H6D3]	0	0.304021	0.304021	2.62E-05
6h	ACA1_272960/clean/scf7180000084773:3498-3584:-	ACA1_272960	Ras-GAP domain-containing protein [Source:UniProtKB/TrEMBL;Acc:L8GFH7]	0.004915923	0.308922333	0.30400641	1.70E-10
6h	ACA1_215970/clean/scf7180000084758:417444-417528:-	ACA1_215970	EGFlike domain containing protein [Source:UniProtKB/TrEMBL;Acc:L8GQ73]	0.014178767	0.318131333	0.303952567	7.16E-05
6h	ACA1_253100/clean/scf7180000084768:242489-242584:-	ACA1_253100	MORN repeatcontaining protein [Source:UniProtKB/TrEMBL;Acc:L8H9X8]	0.021088633	0.324983	0.303894367	0.000303007
6h	ACA1_260140/clean/scf7180000084769:441968-442057:+	ACA1_260140	Endonuclease III, putative [Source:UniProtKB/TrEMBL;Acc:L8GF10]	0.052978133	0.356758667	0.303780533	2.36E-05
6h	ACA1_211790/clean/scf7180000084758:114935-115029:+	ACA1_211790	RhoGEF domain containing protein [Source:UniProtKB/TrEMBL;Acc:L8GQS6]	0.061100133	0.364802333	0.3037022	3.12E-05
6h	ACA1_256610/clean/scf7180000084769:64009-64092:-	ACA1_256610	hypothetical protein	0	0.303663333	0.303663333	0.00956395
6h	ACA1_253970/clean/scf7180000084768:354638-354691:-	ACA1_253970	EGFlike domain containing protein [Source:UniProtKB/TrEMBL;Acc:L8HAZ9]	0.500982	0.804530333	0.303548333	1.34E-05
6h	ACA1_228520/clean/scf7180000084761:179814-179889:-	ACA1_228520	ANK_REP_REGION domain-containing protein [Source:UniProtKB/TrEMBL;Acc:L8HB48]	0.00353695	0.307047333	0.303510383	5.79E-13
6h	ACA1_269260/clean/scf7180000084770:959544-959684:-	ACA1_269260	Ras family protein [Source:UniProtKB/TrEMBL;Acc:L8H2Q9]	0.0221523	0.325563667	0.303411367	2.60E-05
6h	ACA1_046170/clean/scf7180000084577:74141-74213:-	ACA1_046170	Kelch repeat-containing protein [Source:UniProtKB/TrEMBL;Acc:L8HB10]	0.007874	0.31128	0.303406	0.000289934
6h	ACA1_177730/clean/scf7180000084743:116284-116373:-	ACA1_177730	Protein phosphatase 4 regulatory subunit 1, putative [Source:UniProtKB/TrEMBL;Acc:L8GSJ6]	0.006208963	0.309456	0.303247037	1.25E-05
6h	ACA1_111060/clean/scf7180000084666:20960-21046:-	ACA1_111060	Xtp3transactivated protein, putative [Source:UniProtKB/TrEMBL;Acc:L8H6W1]	0.005433553	0.308680333	0.30324678	9.92E-10
6h	ACA1_148480/clean/scf7180000084715:10674-10768:-	ACA1_148480	I/LWEQ domain containing protein [Source:UniProtKB/TrEMBL;Acc:L8HEA2]	0.001070821	0.304309667	0.303238845	1.27E-11
6h	ACA1_191550/clean/scf7180000084749:14829-14972:+	ACA1_191550	Ribosomal protein L13, putative [Source:UniProtKB/TrEMBL;Acc:L8GN96]	0.004886857	0.308119333	0.303232477	1.69E-11
6h	ACA1_088660/clean/scf7180000084639:197664-197741:-	ACA1_088660	RhoGEF domain containing protein [Source:UniProtKB/TrEMBL;Acc:L8GX30]	0.016203233	0.319347	0.303143767	4.55E-05
6h	ACA1_208460/clean/scf7180000084757:197801-197910:-	ACA1_208460	Abhydro_lipase domain-containing protein [Source:UniProtKB/TrEMBL;Acc:L8GZL5]	0.027716667	0.330855	0.303138333	7.19E-08
6h	ACA1_166080/clean/scf7180000084732:115005-115088:+	ACA1_166080	hypothetical protein	0.00139909	0.304504	0.30310491	1.21E-07
6h	ACA1_114450/clean/scf7180000084669:473707-473816:-	ACA1_114450	SH3 domain containing protein [Source:UniProtKB/TrEMBL;Acc:L8H6I9]	0.005770647	0.308864333	0.303093687	3.09E-19
6h	ACA1_174100/clean/scf7180000084741:612490-612574:-	ACA1_174100	Protein kinase domain containing protein [Source:UniProtKB/TrEMBL;Acc:L8HK30]	0.01220982	0.315259667	0.303049847	1.76E-11
6h	ACA1_173860/clean/scf7180000084741:534951-535166:-	ACA1_173860	Mob4B protein isoform 3, putative [Source:UniProtKB/TrEMBL;Acc:L8HJ60]	0.006618153	0.309596	0.302977847	4.52E-18
6h	ACA1_369320/clean/scf7180000084664:301580-301661:+	ACA1_369320	Coronin [Source:UniProtKB/TrEMBL;Acc:L8GYZ1]	0.006973795	0.309933333	0.302959538	4.48E-07
6h	ACA1_063740/clean/scf7180000084599:1492463-1492533:-	ACA1_063740	Transmembrane protein [Source:UniProtKB/TrEMBL;Acc:L8GXB1]	0.027108533	0.330045	0.302936467	4.80E-10
6h	ACA1_036660/clean/scf7180000084565:166315-166396:-	ACA1_036660	ATPase [Source:UniProtKB/TrEMBL;Acc:L8HCK4]	0.0307998	0.333727333	0.302927533	7.82E-05
6h	ACA1_133410/clean/scf7180000084701:48569-48649:-	ACA1_133410	EF hand domain containing protein [Source:UniProtKB/TrEMBL;Acc:L8H515]	0.00421899	0.307134	0.30291501	1.40E-07
6h	ACA1_218620/clean/scf7180000084758:679705-679786:-	ACA1_218620	Carboxylic ester hydrolase [Source:UniProtKB/TrEMBL;Acc:L8GRD3]	0.019104403	0.322009667	0.302905263	1.56E-09
6h	ACA1_000820/clean/scf7180000082828:10098-10168:+	ACA1_000820	RING-type domain-containing protein [Source:UniProtKB/TrEMBL;Acc:L8H7M3]	0	0.302872667	0.302872667	3.02E-07
6h	ACA1_135120/clean/scf7180000084702:66041-66141:-	ACA1_135120	SRI domain-containing protein [Source:UniProtKB/TrEMBL;Acc:L8GER9]	0.032941733	0.335677667	0.302735933	5.79E-06
6h	ACA1_068360/clean/scf7180000084603:181269-181348:+	ACA1_068360	Dual specificity protein kinase [Source:UniProtKB/TrEMBL;Acc:L8HDE4]	0.007634093	0.310356333	0.30272224	3.00E-18
6h	ACA1_074750/clean/scf7180000084608:76422-76526:-	ACA1_074750	5formyltetrahydrofolate cyclo-ligase subfamily protein [Source:UniProtKB/TrEMBL;Acc:L8HGR6]	0.003331283	0.305924667	0.302593383	1.07E-12
6h	ACA1_173170/clean/scf7180000084741:421408-421487:-	ACA1_173170	SNF2 family Nterminal domain containing protein [Source:UniProtKB/TrEMBL;Acc:L8HGM5]	0.00584517	0.308422333	0.302577163	9.51E-06
6h	ACA1_100110/clean/scf7180000084648:38446-38525:+	ACA1_100110	hypothetical protein	0	0.302554667	0.302554667	0.000299538
6h	ACA1_019210/clean/scf7180000083937:15720-15822:-	ACA1_019210	PX domain-containing protein [Source:UniProtKB/TrEMBL;Acc:L8HHB5]	0	0.302447	0.302447	0.040520979
6h	ACA1_396220/clean/scf7180000084759:58945-59024:+	ACA1_396220	Acyl-coenzyme A oxidase [Source:UniProtKB/TrEMBL;Acc:L8HEJ7]	0.0494013	0.351845	0.3024437	2.38E-10
6h	ACA1_112820/clean/scf7180000084669:223584-223643:-	ACA1_112820	PeptidyltRNA hydrolase [Source:UniProtKB/TrEMBL;Acc:L8H433]	0.025951933	0.328394	0.302442067	1.60E-11
6h	ACA1_012330/clean/scf7180000083740:15837-15919:+	ACA1_012330	WD_REPEATS_REGION domain-containing protein [Source:UniProtKB/TrEMBL;Acc:L8HCD7]	0.013204197	0.315528	0.302323803	3.51E-06
6h	ACA1_163990/clean/scf7180000084729:125047-125106:-	ACA1_163990	Glycosyl hydrolases family 32 superfamily protein [Source:UniProtKB/TrEMBL;Acc:L8GRF2]	0.05759887	0.359801667	0.302202797	0.000160058
6h	ACA1_036200/clean/scf7180000084565:38125-38217:-	ACA1_036200	hypothetical protein	0.005830637	0.308006	0.302175363	2.04E-07
6h	ACA1_000580/clean/scf7180000082784:8876-9025:-	ACA1_000580	hypothetical protein	0.000942545	0.303058	0.302115455	7.78E-10
6h	ACA1_164720/clean/scf7180000084729:259879-259973:+	ACA1_164720	Autophagy-related protein 9 [Source:UniProtKB/TrEMBL;Acc:L8GU30]	0.011161203	0.313269333	0.30210813	3.35E-06
6h	ACA1_117320/clean/scf7180000084669:974148-974221:-	ACA1_117320	FAD binding domain containing protein [Source:UniProtKB/TrEMBL;Acc:L8H415]	0.039983967	0.342052	0.302068033	3.49E-08

6h	ACA1_054430/clean/scf7180000084594:492338-492402:-	ACA1_054430	PHB domain-containing protein [Source:UniProtKB/TrEMBL;Acc:L8H647]	0	0.302017333	0.302017333	0.004212588
6h	ACA1_061760/clean/scf7180000084599:1034013-1034119:+	ACA1_061760	Serine protease, putative [Source:UniProtKB/TrEMBL;Acc:L8GWQ3]	0.002890763	0.304905667	0.302014903	3.37E-09
6h	ACA1_149320/clean/scf7180000084715:129202-129291:+	ACA1_149320	DUF4209 domain-containing protein [Source:UniProtKB/TrEMBL;Acc:L8HB66]	0.024978867	0.326979333	0.302000467	2.63E-05
6h	ACA1_063910/clean/scf7180000084599:1535012-1535091:+	ACA1_063910	Tho complex subunit 7 protein [Source:UniProtKB/TrEMBL;Acc:L8GXH2]	0.005060763	0.307049333	0.30198857	6.05E-07
6h	ACA1_101080/clean/scf7180000084649:35507-35581:-	ACA1_101080	Purple acid phosphatase [Source:UniProtKB/TrEMBL;Acc:L8GGG9]	0.002658473	0.304555333	0.30189686	2.09E-12
6h	ACA1_040470/clean/scf7180000084572:10378-10459:+	ACA1_040470	Leuk-A4-hydro_C domain-containing protein [Source:UniProtKB/TrEMBL;Acc:L8H0K2]	0.001046422	0.302925667	0.301879245	5.12E-09
6h	ACA1_200210/clean/scf7180000084753:414446-414510:+	ACA1_200210	hypothetical protein	0.01089518	0.312731333	0.301836153	5.76E-07
6h	ACA1_277880/clean/scf7180000084776:119301-119364:+	ACA1_277880	RBR-type E3 ubiquitin transferase [Source:UniProtKB/TrEMBL;Acc:L8H6I3]	0.013470327	0.315273	0.301802673	9.88E-14
6h	ACA1_149150/clean/scf7180000084715:84401-84672:-	ACA1_149150	GATA-type domain-containing protein [Source:UniProtKB/TrEMBL;Acc:L8HBX6]	0.017720267	0.319456333	0.301736067	1.16E-10
6h	ACA1_362820/clean/scf7180000084624:188858-188981:+	ACA1_362820	hypothetical protein	0.00806697	0.309800667	0.301733697	1.60E-11
6h	ACA1_174010/clean/scf7180000084741:569758-569859:-	ACA1_174010	Rho-GAP domain-containing protein [Source:UniProtKB/TrEMBL;Acc:L8HJ74]	0.032487407	0.334206333	0.301718927	7.21E-06
6h	ACA1_365810/clean/scf7180000084641:329187-329256:+	ACA1_365810	Golgi apparatus membrane protein TVP23 homolog [Source:UniProtKB/TrEMBL;Acc:L8GLN2]	0.0136999	0.315385	0.3016851	9.90E-10
6h	ACA1_279570/clean/scf7180000084776:416623-416704:+	ACA1_279570	Elp3 domain-containing protein [Source:UniProtKB/TrEMBL;Acc:L8H759]	0.0665887	0.368158333	0.301569633	8.95E-05
6h	ACA1_157770/clean/scf7180000084724:114098-114202:-	ACA1_157770	Uracil-DNA glycosylase [Source:UniProtKB/TrEMBL;Acc:L8HA19]	0.00119021	0.302587333	0.301397123	3.40E-08
6h	ACA1_035450/clean/scf7180000084564:51986-52093:-	ACA1_035450	Opposite strand transcription unit to Stag3, putative [Source:UniProtKB/TrEMBL;Acc:L8HAK5]	0.0041968	0.305463667	0.301266867	8.35E-07
6h	ACA1_372610/clean/scf7180000084679:25970-26071:-	ACA1_372610	Asp_Arg_Hydrox domain-containing protein [Source:UniProtKB/TrEMBL;Acc:L8GH09]	0	0.301254667	0.301254667	7.36E-07
6h	ACA1_389330/clean/scf7180000084733:232236-232365:+	ACA1_389330	Nicotinamide riboside kinase 1 family protein [Source:UniProtKB/TrEMBL;Acc:L8GDW0]	0.023885767	0.325096	0.301210233	1.39E-08
6h	ACA1_034080/clean/scf7180000084561:17049-17170:-	ACA1_034080	RhoGEF domain containing protein [Source:UniProtKB/TrEMBL;Acc:L8GTW8]	0.00535276	0.306444333	0.301091573	4.32E-07
6h	ACA1_322160/clean/scf7180000084459:22002-22077:+	ACA1_322160	AB hydrolase-1 domain-containing protein [Source:UniProtKB/TrEMBL;Acc:L8GMT9]	0.014993653	0.315990333	0.30099668	5.71E-11
6h	ACA1_322160/clean/scf7180000084459:22758-22833:+	ACA1_322160	AB hydrolase-1 domain-containing protein [Source:UniProtKB/TrEMBL;Acc:L8GMT9]	0.004692367	0.305687667	0.3009953	2.18E-07
6h	ACA1_106750/clean/scf7180000084660:115907-115995:+	ACA1_106750	Ubiquinone biosynthesis protein AarF family protein, putative [Source:UniProtKB/TrEMBL;Acc:L8GNA2]	0.033127333	0.334042333	0.300915	1.31E-08
6h	ACA1_270330/clean/scf7180000084770:1113474-1113555:-	ACA1_270330	hypothetical protein	0.072277333	0.373164	0.300886667	0.017108994
6h	ACA1_057400/clean/scf7180000084599:133871-133944:+	ACA1_057400	Leucine rich repeat domain containing protein [Source:UniProtKB/TrEMBL;Acc:L8GVU1]	0	0.300878	0.300878	6.03E-07
6h	ACA1_369720/clean/scf7180000084664:368301-368379:-	ACA1_369720	Ras subfamily protein [Source:UniProtKB/TrEMBL;Acc:L8GY91]	0.002560163	0.303422	0.300861837	7.51E-05
6h	ACA1_116230/clean/scf7180000084669:730170-730242:+	ACA1_116230	Protein kinase domain containing protein [Source:UniProtKB/TrEMBL;Acc:L8H4M9]	0	0.300859667	0.300859667	3.12E-06
6h	ACA1_321910/clean/scf7180000084457:11302-11453:-	ACA1_321910	Importin N-terminal domain-containing protein [Source:UniProtKB/TrEMBL;Acc:L8H9I3]	0.0047226	0.305518333	0.300795733	3.25E-07
6h	ACA1_224370/clean/scf7180000084760:180961-181096:-	ACA1_224370	Exportin 1like protein [Source:UniProtKB/TrEMBL;Acc:L8GTX4]	0	0.300751667	0.300751667	9.38E-06
6h	ACA1_292920/clean/scf7180000084777:934178-934306:-	ACA1_292920	Protein kinase domain-containing protein [Source:UniProtKB/TrEMBL;Acc:L8HLV1]	0.005102553	0.305834667	0.300732113	2.86E-11
6h	ACA1_323460/clean/scf7180000084468:25142-25256:-	ACA1_323460	DEP domain-containing protein [Source:UniProtKB/TrEMBL;Acc:L8H1P0]	0.0308816	0.331606333	0.300724733	1.76E-08
6h	ACA1_201200/clean/scf7180000084753:503623-503706:+	ACA1_201200	Ras subfamily protein [Source:UniProtKB/TrEMBL;Acc:L8H3F2]	0.180752667	0.481324333	0.300571667	0.009031381
6h	ACA1_221050/clean/scf7180000084758:986157-986242:-	ACA1_221050	hypothetical protein	0.015159333	0.315730333	0.300571	2.50E-05
6h	ACA1_193330/clean/scf7180000084749:235407-235486:+	ACA1_193330	6,7-dimethyl-8-ribityllumazine synthase [Source:UniProtKB/TrEMBL;Acc:L8GN89]	0.002530297	0.303035333	0.300505037	1.20E-06
6h	ACA1_399930/clean/scf7180000084775:70426-70537:+	ACA1_399930	Ras subfamily protein [Source:UniProtKB/TrEMBL;Acc:L8GGR5]	0.020837633	0.321317333	0.3004797	1.38E-07
6h	ACA1_360010/clean/scf7180000084622:32902-32965:+	ACA1_360010	Tubulin-specific chaperone A [Source:UniProtKB/TrEMBL;Acc:L8HF03]	0.009805447	0.310261333	0.300455887	2.28E-07
6h	ACA1_170960/clean/scf7180000084741:22542-22654:+	ACA1_170960	hypothetical protein	0	0.300453	0.300453	3.49E-07
6h	ACA1_100440/clean/scf7180000084648:77937-78021:-	ACA1_100440	Cytochrome p450 superfamily protein [Source:UniProtKB/TrEMBL;Acc:L8GKG1]	0.011533763	0.311885333	0.30035157	1.25E-10
6h	ACA1_184040/clean/scf7180000084745:547943-548019:-	ACA1_184040	Ubiquitin-like domain-containing protein [Source:UniProtKB/TrEMBL;Acc:L8H9Q4]	0.002928893	0.303258667	0.300329773	1.21E-50
6h	ACA1_109100/clean/scf7180000084661:115282-115349:-	ACA1_109100	Sodium symporter, putative [Source:UniProtKB/TrEMBL;Acc:L8GC73]	0	0.300255333	0.300255333	0.000298982
6h	ACA1_396170/clean/scf7180000084759:45748-45838:-	ACA1_396170	Phosphatidylserine decarboxylase proenzyme 2 [Source:UniProtKB/TrEMBL;Acc:L8HEJ4]	0.000193882	0.300339667	0.300145785	1.01E-14
6h	ACA1_158790/clean/scf7180000084724:257299-257398:+	ACA1_158790	Peroxiredoxin [Source:UniProtKB/TrEMBL;Acc:L8H9D0]	0.002022863	0.302014	0.299991137	1.31E-11
6h	ACA1_151110/clean/scf7180000084716:62053-62169:+	ACA1_151110	Sec23/Sec24 trunk domain containing protein [Source:UniProtKB/TrEMBL;Acc:L8GZW6]	0.00561479	0.305603333	0.299988543	1.16E-14
6h	ACA1_264730/clean/scf7180000084770:368997-369082:-	ACA1_264730	hypothetical protein	0.057704467	0.357659667	0.2999552	0.00021932
6h	ACA1_091560/clean/scf7180000084645:150152-150225:+	ACA1_091560	UBP-type domain-containing protein [Source:UniProtKB/TrEMBL;Acc:L8GKE3]	0	0.299951	0.299951	1.64E-05
6h	ACA1_325480/clean/scf7180000084495:17775-17866:+	ACA1_325480	RRM domain-containing protein [Source:UniProtKB/TrEMBL;Acc:L8HC56]	0.0554267	0.355268	0.2998413	1.73E-07
6h	ACA1_071550/clean/scf7180000084603:990739-990880:-	ACA1_071550	F-box domain-containing protein [Source:UniProtKB/TrEMBL;Acc:L8HFM3]	0.002814167	0.30255	0.299735833	4.96E-06
6h	ACA1_067460/clean/scf7180000084601:33815-33893:-	ACA1_067460	F-box domain-containing protein [Source:UniProtKB/TrEMBL;Acc:L8GTQ5]	0.049976013	0.34965	0.299673987	3.60E-06
6h	ACA1_061980/clean/scf7180000084599:1092694-1092764:+	ACA1_061980	F-box domain-containing protein [Source:UniProtKB/TrEMBL;Acc:L8GZC3]	0	0.2993285	0.2993285	0.000241006
6h	ACA1_110860/clean/scf7180000084666:3841-3908:+	ACA1_110860	SID1 transmembrane family, member 1, putative [Source:UniProtKB/TrEMBL;Acc:L8H6V2]	0.004458433	0.303778333	0.2993199	4.04E-06
6h	ACA1_020090/clean/scf7180000084037:350-476:-	ACA1_020090	RRM domain-containing protein [Source:UniProtKB/TrEMBL;Acc:L8GKJ8]	0.01962276	0.318876333	0.299253573	0.000187385
6h	ACA1_313850/clean/scf7180000083835:1187-1275:+	ACA1_313850	PAS, putative [Source:UniProtKB/TrEMBL;Acc:L8H077]	0	0.299219667	0.299219667	0.047779266
6h	ACA1_199750/clean/scf7180000084753:317340-317480:-	ACA1_199750	hypothetical protein	0.031431233	0.330493333	0.2990621	6.93E-08
6h	ACA1_350480/clean/scf7180000084692:12237-12362:+	ACA1_350480	ABC2_membrane domain-containing protein [Source:UniProtKB/TrEMBL;Acc:L8HLS4]	0.00966012	0.308624667	0.298964547	1.79E-05
6h	ACA1_131580/clean/scf7180000084699:91174-91241:-	ACA1_131580	Glycerol-3-phosphate dehydrogenase [NAD(+)] [Source:UniProtKB/TrEMBL;Acc:L8GM84]	0.011693883	0.310637667	0.298943783	1.17E-08
6h	ACA1_071250/clean/scf7180000084603:898573-898647:-	ACA1_071250	DEAD box RNA helicase, putative [Source:UniProtKB/TrEMBL;Acc:L8HD92]	0.0872465	0.386149	0.2989025	4.81E-06
6h	ACA1_109100/clean/scf7180000084661:114327-114417:-	ACA1_109100	Sodium symporter, putative [Source:UniProtKB/TrEMBL;Acc:L8GC73]	0.013333333	0.312048667	0.298715333	0.000132963
6h	ACA1_096000/clean/scf7180000084645:935841-935951:-	ACA1_096000	RNA_lig_T4_1 domain-containing protein [Source:UniProtKB/TrEMBL;Acc:L8GIM7]	0.0151515	0.313842333	0.298690833	0.001001111
6h	ACA1_360020/clean/scf7180000084622:34006-34082:-	ACA1_360020	Mannosyltransferase [Source:UniProtKB/TrEMBL;Acc:L8HCL9]	0.000462	0.299150333	0.298688333	1.48E-09
6h	ACA1_256650/clean/scf7180000084769:75244-75390:+	ACA1_256650	von Willebrand factor type A domain containing protein [Source:UniProtKB/TrEMBL;Acc:L8GH56]	0.00507586	0.303759	0.29868314	4.36E-05
6h	ACA1_289280/clean/scf7180000084777:405528-405609:-	ACA1_289280	RasGEF domain containing protein [Source:UniProtKB/TrEMBL;Acc:L8HJ36]	0.0288714	0.327494333	0.298622933	0.001337621
6h	ACA1_193320/clean/scf7180000084749:232384-232463:-	ACA1_193320	hypothetical protein	0	0.298592333	0.298592333	0.013788274
6h	ACA1_091930/clean/scf7180000084645:230656-230729:-	ACA1_091930	hypothetical protein	0.392546	0.691124333	0.298578333	0.003725397
6h	ACA1_061150/clean/scf7180000084599:840710-840784:-	ACA1_061150	UBA/TSN domain containing protein [Source:UniProtKB/TrEMBL;Acc:L8GWQ2]	0.018744829	0.317305333	0.298560504	4.63E-05
6h	ACA1_271370/clean/scf7180000084771:37477-37569:+	ACA1_271370	PIPK domain-containing protein [Source:UniProtKB/TrEMBL;Acc:L8GP04]	0.138065667	0.436622	0.298556333	0.000105225
6h	ACA1_269790/clean/scf7180000084770:1043333-1043445:-	ACA1_269790	CRT-like domain-containing protein [Source:UniProtKB/TrEMBL;Acc:L8H2S4]	0.002315666	0.300861333	0.298545667	1.92E-08
6h	ACA1_209110/clean/scf7180000084757:273278-273360:+	ACA1_209110	hypothetical protein	0.00925882	0.307798667	0.298539847	1.22E-05
6h	ACA1_178200/clean/scf7180000084743:178324-178506:+	ACA1_178200	B30.2/SPRY domain-containing protein [Source:UniProtKB/TrEMBL;Acc:L8GVS5]	0.00602393	0.304562667	0.298538737	1.49E-09
6h	ACA1_210540/clean/scf7180000084758:51911-51998:-	ACA1_210540	ABC transporter, ATPbinding domain containing protein [Source:UniProtKB/TrEMBL;Acc:L8GPZ1]	0.014492767	0.312964	0.298471233	0.000512261
6h	ACA1_147710/clean/scf7180000084714:52594-52658:+	ACA1_147710	Zn_dep_PLPC domain-containing protein [Source:UniProtKB/TrEMBL;Acc:L8GJR2]	0	0.298417	0.298417	9.36E-09
6h	ACA1_199720/clean/scf7180000084753:308968-309079:-	ACA1_199720	Pentatricopeptide repeat domain containing protein [Source:UniProtKB/TrEMBL;Acc:L8H4N1]	0.022024207	0.320380667	0.29835646	5.88E-10
6h	ACA1_183530/clean/scf7180000084745:444481-444548:-	ACA1_183530	Clu domain-containing protein [Source:UniProtKB/TrEMBL;Acc:L8H9K5]	0.002062243	0.300329333	0.29826709	2.15E-07
6h	ACA1_068780/clean/scf7180000084603:275366-275452:-	ACA1_068780	ANK_REP_REGION domain-containing protein [Source:UniProtKB/TrEMBL;Acc:L8HCM2]	0	0.298085667	0.298085667	2.68E-05
6h	ACA1_133450/clean/scf7180000084701:55591-55716:+	ACA1_133450	HECT domain-containing protein [Source:UniProtKB/TrEMBL;Acc:L8H3L8]	0	0.298077	0.298077	6.30E-07
6h	ACA1_375930/clean/scf7180000084685:5713-5834:-	ACA1_375930	SUN domain-containing protein [Source:UniProtKB/TrEMBL;Acc:L8HIE1]	0	0.298055	0.298055	4.43E-05
6h	ACA1_261480/clean/scf7180000084769:690827-690912:+	ACA1_261480	ATP-dependent (S)-NAD(P)H-hydrate dehydratase [Source:UniProtKB/TrEMBL;Acc:L8GG71]	0	0.298029667	0.298029667	3.90E-09
6h	ACA1_158440/clean/scf7180000084724:219391-219486:+	ACA1_158440	Coiled-coil domain containing 53, putative [Source:UniProtKB/TrEMBL;Acc:L8HA48]	0.004801337	0.302827	0.298025663	4.18E-08
6h	ACA1_380820/clean/scf7180000084712:93901-93958:+	ACA1_380820	tRNA-binding domain-containing protein [Source:UniProtKB/TrEMBL;Acc:L8GMV0]	0.0294841	0.327473667	0.297989567	2.54E-12
6h	ACA1_376710/clean/scf7180000084685:145072-145135:+	ACA1_376710	PHD-type domain-containing protein [Source:UniProtKB/TrEMBL;Acc:L8HG20]	0.04065055	0.338607333	0.297956783	0.000257791
6h	ACA1_297750/clean/scf7180000082853:780-880:-	ACA1_297750	14_3_3 domain-containing protein [Source:UniProtKB/TrEMBL;Acc:L8HE45]	0.042693467	0.340531	0.297837533	0.001560393
6h	ACA1_289870/clean/scf7180000084777:481833-481924:+	ACA1_289870	Helicase ATP-binding domain-containing protein [Source:UniProtKB/TrEMBL;Acc:L8HI29]	0.019607833	0.317382333	0.2977745	0.001098778
6h	ACA1_075160/clean/scf7180000084608:143116-143193:-	ACA1_075160	RRM domain-containing protein [Source:UniProtKB/TrEMBL;Acc:L8HGV3]	0.001461277	0.299132667	0.29767139	4.36E-22
6h	ACA1_333280/clean/scf7180000084581:30055-30249:-	ACA1_333280	Gpatch domain containing protein [Source:UniProtKB/TrEMBL;Acc:L8HIC3]	0.299286667	0.596924667	0.297638	0.000381727
6h	ACA1_191530/clean/scf7180000084749:11293-11447:+	ACA1_191530	Pex2_Pex12 domain-containing protein [Source:UniProtKB/TrEMBL;Acc:L8GMZ9]	0.004769277	0.302393667	0.29762439	5.08E-07
6h	ACA1_002620/clean/scf7180000082935:4435-4653:+	ACA1_002620	Fbox domain containing protein [Source:UniProtKB/TrEMBL;Acc:L8HLR9]	0.004784647	0.302405333	0.297620687	3.77E-07
6h	ACA1_063620/clean/scf7180000084599:1463003-1463063:-	ACA1_063620	Hydroxysteroid dehydrogenase, putative [Source:UniProtKB/TrEMBL;Acc:L8GYL0]	0.003472233	0.301040667	0.297568433	1.61E-09
6h	ACA1_144300/clean/scf7180000084709:198282-198430:-	ACA1_144300	RRM domain-containing protein [Source:UniProtKB/TrEMBL;Acc:L8HEU9]	0.004574191	0.302126333	0.297552142	2.18E-09
6h	ACA1_144430/clean/scf7180000084709:240637-240732:-	ACA1_144430	CASP_C domain-containing protein [Source:UniProtKB/TrEMBL;Acc:L8HI28]	0.0223163	0.319833	0.2975167	2.66E-09
6h	ACA1_164720/clean/scf7180000084729:258777-258842:+	ACA1_164720	Autophagy-related protein 9 [Source:UniProtKB/TrEMBL;Acc:L8GU30]	0.002590433	0.300059333	0.2974689	2.21E-06
6h	ACA1_107870/clean/scf7180000084661:2230-2315:-	ACA1_107870	UBA domain-containing protein [Source:UniProtKB/TrEMBL;Acc:L8GC31]	0	0.297386	0.297386	1.62E-08
6h	ACA1_272960/clean/scf7180000084773:2315-2374:-	ACA1_272960	Ras-GAP domain-containing protein [Source:UniProtKB/TrEMBL;Acc:L8GFH7]	0.040215833	0.337581	0.297365167	1.26E-10
6h	ACA1_195280/clean/scf7180000084751:89698-89805:+	ACA1_195280	Ras-GAP domain-containing protein [Source:UniProtKB/TrEMBL;Acc:L8H7I4]	0.014351833	0.311696	0.297344167	4.67E-05
6h	ACA1_258340/clean/scf7180000084769:239241-239315:+	ACA1_258340	Protein kinase domain-containing protein [Source:UniProtKB/TrEMBL;Acc:L8GFV7]	0	0.297258667	0.297258667	8.94E-06
6h	ACA1_240180/clean/scf7180000084763:203096-203202:+	ACA1_240180	WD_REPEATS_REGION domain-containing protein [Source:UniProtKB/TrEMBL;Acc:L8GM97]	0.028716783	0.325959	0.297242217	3.65E-06
6h	ACA1_278150/clean/scf7180000084776:180648-180721:-	ACA1_278150	hypothetical protein	0.00250164	0.299733667	0.297232027	7.04E-11
6h	ACA1_065450/clean/scf7180000084599:1854162-1854227:-	ACA1_065450	Cation_ATPase_N domain-containing protein [Source:UniProtKB/TrEMBL;Acc:L8H037]	0.044630333	0.341861	0.297230667	0.003974437
6h	ACA1_112470/clean/scf7180000084669:159387-159469:-	ACA1_112470	KH domain-containing protein [Source:UniProtKB/TrEMBL;Acc:L8H5E2]	0.014690063	0.311856333	0.29716627	4.11E-07
6h	ACA1_275630/clean/scf7180000084774:68332-68411:-	ACA1_275630	unspecified product	0.003802244	0.300953333	0.29715109	1.51E-07
6h	ACA1_332590/clean/scf7180000084579:43190-43267:-	ACA1_332590	GH16 domain-containing protein [Source:UniProtKB/TrEMBL;Acc:L8GKV3]	0.00546913	0.302597333	0.297128203	1.30E-14
6h	ACA1_387600/clean/scf7180000084733:60773-60849:-	ACA1_387600	Ubiquitin domain containing protein [Source:UniProtKB/TrEMBL;Acc:L8GG51]	0.010263867	0.307260333	0.296996467	2.50E-05
6h	ACA1_265960/clean/scf7180000084770:554873-554942:-	ACA1_265960	Mac domain-containing protein [Source:UniProtKB/TrEMBL;Acc:L8H4H0]	0.010325073	0.307049	0.296723927	2.48E-14
6h	ACA1_060100/clean/scf7180000084599:585188-585260:+	ACA1_060100	DENN (AEX3) domain containing protein [Source:UniProtKB/TrEMBL;Acc:L8GXI7]	0.0082893	0.305001	0.2967117	1.58E-07
6h	ACA1_191840/clean/scf7180000084749:51568-51634:+	ACA1_191840	Serine/threonine-protein phosphatase [Source:UniProtKB/TrEMBL;Acc:L8GNP7]	0.00275482	0.299446333	0.296691513	8.50E-08
6h	ACA1_230310/clean/scf7180000084761:343197-343294:+	ACA1_230310	PAS domain-containing protein [Source:UniProtKB/TrEMBL;Acc:L8HBA3]	0.0222493	0.318873	0.2966237	1.93E-05
6h	ACA1_089970/clean/scf7180000084639:406694-406801:-	ACA1_089970	Macro domain-containing protein [Source:UniProtKB/TrEMBL;Acc:L8GU43]	0.026554733	0.323172667	0.296617933	1.14E-08
6h	ACA1_061120/clean/scf7180000084599:825449-825535:-	ACA1_061120	Leucine rich repeat domain containing protein [Source:UniProtKB/TrEMBL;Acc:L8GVW2]	0.00117667	0.297777667	0.296600997	5.53E-05
6h	ACA1_178260/clean/scf7180000084743:192177-192342:+	ACA1_178260	Cytochrome P450, putative [Source:UniProtKB/TrEMBL;Acc:L8GTE4]	0.00140647	0.297951333	0.296544863	2.85E-10
6h	ACA1_362650/clean/scf7180000084624:123843-123955:+	ACA1_362650	G-patch domain-containing protein [Source:UniProtKB/TrEMBL;Acc:L8GFY2]	0.004252543	0.300717333	0.29646479	2.24E-05
6h	ACA1_058720/clean/scf7180000084599:363582-363681:-	ACA1_058720	hypothetical protein	0.003567283	0.300029	0.296461717	4.04E-09
6h	ACA1_144880/clean/scf7180000084710:31420-31538:-	ACA1_144880	hypothetical protein	0.008047903	0.304492	0.296444097	7.97E-11
6h	ACA1_037490/clean/scf7180000084567:102610-102718:-	ACA1_037490	hypothetical protein	0.0104896	0.306914	0.2964244	1.35E-05
6h	ACA1_074670/clean/scf7180000084608:61886-61957:-	ACA1_074670	Arginase [Source:UniProtKB/TrEMBL;Acc:L8HFF6]	0	0.296406667	0.296406667	1.93E-08
6h	ACA1_100560/clean/scf7180000084648:114016-114093:+	ACA1_100560	hypothetical protein	0.0106049	0.306994333	0.296389433	6.19E-07
6h	ACA1_173350/clean/scf7180000084741:467553-467682:-	ACA1_173350	Carrier superfamily protein [Source:UniProtKB/TrEMBL;Acc:L8HHM8]	0.004305337	0.300693	0.296387663	3.49E-07
6h	ACA1_037900/clean/scf7180000084568:54489-54553:-	ACA1_037900	Nudix hydrolase domain-containing protein [Source:UniProtKB/TrEMBL;Acc:L8GKU1]	0	0.296371333	0.296371333	0.000148943
6h	ACA1_093710/clean/scf7180000084645:620530-620611:-	ACA1_093710	DH domain-containing protein [Source:UniProtKB/TrEMBL;Acc:L8GIB0]	0.000419903	0.296724667	0.296304763	8.09E-07
6h	ACA1_263000/clean/scf7180000084770:71981-72053:-	ACA1_263000	Molybdenum cofactor sulfurase, putative [Source:UniProtKB/TrEMBL;Acc:L8H432]	0	0.296261667	0.296261667	2.29E-08
6h	ACA1_068040/clean/scf7180000084603:85203-85282:+	ACA1_068040	START domain-containing protein [Source:UniProtKB/TrEMBL;Acc:L8HDG1]	0.010598833	0.306804667	0.296205833	2.38E-05
6h	ACA1_107540/clean/scf7180000084660:218088-218181:+	ACA1_107540	hypothetical protein	0.107159833	0.403330667	0.296170833	2.84E-05
6h	ACA1_151330/clean/scf7180000084716:104444-104535:-	ACA1_151330	Purple acid phosphatase [Source:UniProtKB/TrEMBL;Acc:L8H0Q3]	0.00973483	0.305876	0.29614117	8.73E-15
6h	ACA1_215990/clean/scf7180000084758:427226-427293:+	ACA1_215990	hypothetical protein	0.0195941	0.3157	0.2961059	5.80E-07
6h	ACA1_144200/clean/scf7180000084709:169867-170034:+	ACA1_144200	PH domain-containing protein [Source:UniProtKB/TrEMBL;Acc:L8HET7]	0.0047526	0.300812667	0.296060067	8.21E-25
6h	ACA1_185590/clean/scf7180000084746:112338-112485:+	ACA1_185590	EF hand domain containing protein [Source:UniProtKB/TrEMBL;Acc:L8H583]	0.00094008	0.296992	0.29605192	1.60E-10
6h	ACA1_310730/clean/scf7180000083780:163-233:+	ACA1_310730	PRKCSH_1 domain-containing protein [Source:UniProtKB/TrEMBL;Acc:L8GSR5]	0.00606053	0.302043667	0.295983137	1.87E-09
6h	ACA1_163160/clean/scf7180000084726:94030-94142:-	ACA1_163160	Ankyrin repeat-containing protein [Source:UniProtKB/TrEMBL;Acc:L8GXX1]	0.333333333	0.629284333	0.295951	0.002914021
6h	ACA1_050400/clean/scf7180000084589:86032-86100:+	ACA1_050400	Usp domain-containing protein [Source:UniProtKB/TrEMBL;Acc:L8H3R6]	0.049928	0.345863	0.295935	3.29E-07
6h	ACA1_130580/clean/scf7180000084698:114581-114694:-	ACA1_130580	CID domain-containing protein [Source:UniProtKB/TrEMBL;Acc:L8GQC8]	0.008056467	0.303990667	0.2959342	1.04E-06
6h	ACA1_043190/clean/scf7180000084573:137070-137138:-	ACA1_043190	hypothetical protein	0.185329433	0.481218667	0.295889233	0.00093199
6h	ACA1_276210/clean/scf7180000084774:140860-140967:+	ACA1_276210	Aspartate aminotransferase [Source:UniProtKB/TrEMBL;Acc:L8GTG7]	0.0028721	0.298754667	0.295882567	1.52E-17
6h	ACA1_289660/clean/scf7180000084777:439502-439570:+	ACA1_289660	Churchill domain containing 1, putative [Source:UniProtKB/TrEMBL;Acc:L8HKL9]	0.01221828	0.308047	0.29582872	1.25E-11
6h	ACA1_075170/clean/scf7180000084608:145693-145784:+	ACA1_075170	hypothetical protein	0.021680583	0.317479	0.295798417	5.45E-06
6h	ACA1_220750/clean/scf7180000084758:960718-960834:-	ACA1_220750	Urease accessory protein UreF, putative [Source:UniProtKB/TrEMBL;Acc:L8GQ53]	0.026572267	0.322260667	0.2956884	4.53E-06
6h	ACA1_146600/clean/scf7180000084713:64470-64559:-	ACA1_146600	Protein kinase domain containing protein [Source:UniProtKB/TrEMBL;Acc:L8GWQ5]	0.000764236	0.296185667	0.295421431	2.90E-12
6h	ACA1_396200/clean/scf7180000084759:51394-51489:+	ACA1_396200	Epimerase domain-containing protein [Source:UniProtKB/TrEMBL;Acc:L8HBC7]	0.033175133	0.328541333	0.2953662	1.71E-07
6h	ACA1_116920/clean/scf7180000084669:873673-873763:-	ACA1_116920	hypothetical protein	0.0014907	0.296826	0.2953353	5.10E-08
6h	ACA1_174330/clean/scf7180000084741:677827-677919:+	ACA1_174330	MHD domain-containing protein [Source:UniProtKB/TrEMBL;Acc:L8HHT4]	0.0295342	0.324753333	0.295219133	3.26E-08
6h	ACA1_368190/clean/scf7180000084664:74697-74764:+	ACA1_368190	hypothetical protein	0.003663	0.298879333	0.295216333	2.29E-12
6h	ACA1_358660/clean/scf7180000084742:25344-25435:+	ACA1_358660	SH3 domain containing protein [Source:UniProtKB/TrEMBL;Acc:L8GLJ2]	0.030316233	0.325503667	0.295187433	6.07E-07
6h	ACA1_236110/clean/scf7180000084762:408617-408728:-	ACA1_236110	PPM-type phosphatase domain-containing protein [Source:UniProtKB/TrEMBL;Acc:L8H2W4]	0	0.295145667	0.295145667	3.43E-09
6h	ACA1_012980/clean/scf7180000083742:15384-15475:-	ACA1_012980	Endo/exonuclease/phosphatase domain-containing protein [Source:UniProtKB/TrEMBL;Acc:L8H4W5]	0.024018733	0.319108333	0.2950896	3.44E-06

6h	ACA1_174600/clean/scf7180000084741:747619-747726:+	ACA1_174600	VIT domain-containing protein [Source:UniProtKB/TrEMBL;Acc:L8HGW0]	0.01085951	0.305877333	0.295017823	0.000186712
6h	ACA1_062280/clean/scf7180000084599:1156358-1156428:-	ACA1_062280	hypothetical protein	0.013369827	0.308319	0.294949173	7.36E-05
6h	ACA1_191850/clean/scf7180000084749:57923-57993:-	ACA1_191850	Protein kinase domain-containing protein [Source:UniProtKB/TrEMBL;Acc:L8GN20]	0.006937653	0.301832333	0.29489468	9.38E-12
6h	ACA1_228790/clean/scf7180000084761:214543-214629:+	ACA1_228790	IgA-specific serine endopeptidase [Source:UniProtKB/TrEMBL;Acc:L8HA86]	0	0.294877	0.294877	4.83E-08
6h	ACA1_100090/clean/scf7180000084648:35188-35282:-	ACA1_100090	Fbox domain containing protein [Source:UniProtKB/TrEMBL;Acc:L8GK42]	0.00460075	0.299457333	0.294856583	8.71E-07
6h	ACA1_229170/clean/scf7180000084761:237562-237654:+	ACA1_229170	hypothetical protein	0.0251678	0.32	0.2948322	1.62E-10
6h	ACA1_114600/clean/scf7180000084669:519829-519920:+	ACA1_114600	hypothetical protein	0.0116112	0.306438	0.2948268	4.86E-06
6h	ACA1_383700/clean/scf7180000084721:246889-246976:+	ACA1_383700	Endo/exonuclease/phosphatase domain-containing protein [Source:UniProtKB/TrEMBL;Acc:L8GV68]	0.000204073	0.294925	0.294720927	1.43E-08
6h	ACA1_006130/clean/scf7180000083029:49885-50000:+	ACA1_006130	Tyrosine kinase, putative [Source:UniProtKB/TrEMBL;Acc:L8HG15]	0.0020909	0.296714	0.2946231	8.01E-07
6h	ACA1_068260/clean/scf7180000084603:147536-147629:+	ACA1_068260	hypothetical protein	0.02700347	0.321626333	0.294622863	9.43E-07
6h	ACA1_289280/clean/scf7180000084777:406200-406267:-	ACA1_289280	RasGEF domain containing protein [Source:UniProtKB/TrEMBL;Acc:L8HJ36]	0.010416667	0.304976333	0.294559667	0.000520752
6h	ACA1_114530/clean/scf7180000084669:499409-499485:+	ACA1_114530	NB-ARC domain containing protein [Source:UniProtKB/TrEMBL;Acc:L8H3K3]	0.000483593	0.294983333	0.29449974	8.49E-09
6h	ACA1_282270/clean/scf7180000084776:641318-641382:-	ACA1_282270	hypothetical protein	0	0.29442	0.29442	1.14E-06
6h	ACA1_261210/clean/scf7180000084769:605381-605461:+	ACA1_261210	F-box domain containing protein [Source:UniProtKB/TrEMBL;Acc:L8GG50]	0.017979233	0.312366667	0.294387433	0.000355659
6h	ACA1_378280/clean/scf7180000084711:229167-229294:+	ACA1_378280	hypothetical protein	0.087419533	0.381654333	0.2942348	7.18E-05
6h	ACA1_069430/clean/scf7180000084603:436504-436613:+	ACA1_069430	CULLIN_2 domain-containing protein [Source:UniProtKB/TrEMBL;Acc:L8HCT6]	0	0.294088	0.294088	2.63E-07
6h	ACA1_343730/clean/scf7180000084638:30718-30800:+	ACA1_343730	hypothetical protein	0.083059433	0.377090333	0.2940309	0.000339681
6h	ACA1_074160/clean/scf7180000084605:40254-40321:+	ACA1_074160	Aamy domain-containing protein [Source:UniProtKB/TrEMBL;Acc:L8HJ75]	0.023170867	0.317136667	0.2939658	2.07E-12
6h	ACA1_310740/clean/scf7180000083780:1209-1271:-	ACA1_310740	COesterase domain-containing protein [Source:UniProtKB/TrEMBL;Acc:L8GTB6]	0.0128205	0.306773333	0.293952833	0.000180639
6h	ACA1_206330/clean/scf7180000084757:10107-10190:+	ACA1_206330	RBR-type E3 ubiquitin transferase [Source:UniProtKB/TrEMBL;Acc:L8H0J7]	0.028359733	0.322276333	0.2939166	5.61E-07
6h	ACA1_279440/clean/scf7180000084776:384455-384538:+	ACA1_279440	Hypothetical protein	0	0.293857333	0.293857333	2.37E-06
6h	ACA1_012020/clean/scf7180000083652:5417-5545:-	ACA1_012020	hypothetical protein	0.001876887	0.295722	0.293845113	7.31E-11
6h	ACA1_114570/clean/scf7180000084669:514095-514166:-	ACA1_114570	Mediator of RNA polymerase II transcription subunit 4 [Source:UniProtKB/TrEMBL;Acc:L8H5U5]	0.0017452	0.295565667	0.293820467	6.58E-08
6h	ACA1_201010/clean/scf7180000084753:462694-462776:+	ACA1_201010	Fe2OG dioxygenase domain-containing protein [Source:UniProtKB/TrEMBL;Acc:L8H2K6]	0.082813667	0.376471667	0.293658	0.00031111
6h	ACA1_296490/clean/scf7180000084777:1361099-1361182:-	ACA1_296490	Succinate--CoA ligase [ADP-forming] subunit beta, mitochondrial [Source:UniProtKB/TrEMBL;Acc:L8HMD9]	0.003058103	0.296714	0.293655897	2.10E-09
6h	ACA1_385940/clean/scf7180000084730:313318-313433:+	ACA1_385940	N-acetyltransferase domain-containing protein [Source:UniProtKB/TrEMBL;Acc:L8HBM5]	0.003000793	0.296534667	0.293533873	1.97E-10
6h	ACA1_250030/clean/scf7180000084767:26625-26710:-	ACA1_250030	C2 NT-type domain-containing protein [Source:UniProtKB/TrEMBL;Acc:L8HC94]	0.00346937	0.296955	0.29348563	3.85E-08
6h	ACA1_278980/clean/scf7180000084776:289721-289823:-	ACA1_278980	Extracellular ligandbinding receptor, putative [Source:UniProtKB/TrEMBL;Acc:L8H6Q3]	0.009390833	0.302875333	0.2934845	0.000112008
6h	ACA1_126940/clean/scf7180000084696:5229-5308:+	ACA1_126940	hypothetical protein	0.018094133	0.311536667	0.293442533	5.65E-07
6h	ACA1_059990/clean/scf7180000084599:557665-557746:+	ACA1_059990	Actine, putative [Source:UniProtKB/TrEMBL;Acc:L8GWG1]	0	0.293398333	0.293398333	3.70E-07
6h	ACA1_308240/clean/scf7180000083743:7731-7798:+	ACA1_308240	VPS13_C domain-containing protein [Source:UniProtKB/TrEMBL;Acc:L8HJD8]	0.000907207	0.294298667	0.29339146	1.28E-06
6h	ACA1_058010/clean/scf7180000084599:231275-231347:+	ACA1_058010	WD_REPEATS_REGION domain-containing protein [Source:UniProtKB/TrEMBL;Acc:L8GVX5]	0.013984867	0.307320667	0.2933358	8.27E-08
6h	ACA1_370300/clean/scf7180000084664:490668-490743:-	ACA1_370300	hypothetical protein	0.000884687	0.294145667	0.29326098	7.35E-11
6h	ACA1_339770/clean/scf7180000084619:22973-23125:+	ACA1_339770	Metallophos domain-containing protein [Source:UniProtKB/TrEMBL;Acc:L8GK77]	0.197516	0.490713667	0.293197667	0.002572705
6h	ACA1_174040/clean/scf7180000084741:586577-586704:-	ACA1_174040	Protein kinase domain-containing protein [Source:UniProtKB/TrEMBL;Acc:L8HK27]	0.000703264	0.2938575	0.293154236	2.83E-08
6h	ACA1_217280/clean/scf7180000084758:556192-556295:+	ACA1_217280	Protein kinase domain containing protein [Source:UniProtKB/TrEMBL;Acc:L8GQB1]	0.005261957	0.298368333	0.293106377	7.27E-12
6h	ACA1_143300/clean/scf7180000084709:24232-24373:-	ACA1_143300	mRNA_triPase domain-containing protein [Source:UniProtKB/TrEMBL;Acc:L8HFN9]	0.006300203	0.299401	0.293100797	3.45E-06
6h	ACA1_178530/clean/scf7180000084743:254103-254234:-	ACA1_178530	Dual specificity phosphatase, catalytic domain containing protein [Source:UniProtKB/TrEMBL;Acc:L8GSL7]	0.0204973	0.313513	0.2930157	0.002275868
6h	ACA1_380870/clean/scf7180000084712:104372-104447:+	ACA1_380870	Transporter, major facilitator subfamily protein [Source:UniProtKB/TrEMBL;Acc:L8GMV6]	0.036759067	0.329724633	0.292965567	1.28E-05
6h	ACA1_191150/clean/scf7180000084748:186494-186559:+	ACA1_191150	Triosephosphate isomerase [Source:UniProtKB/TrEMBL;Acc:L8GE90]	0.002949151	0.295886	0.292936849	1.68E-09
6h	ACA1_170810/clean/scf7180000084741:13234-13348:+	ACA1_170810	CDPalcohol phosphatidyltransferase superfamily protein [Source:UniProtKB/TrEMBL;Acc:L8HH92]	0.0282743	0.321117667	0.292843367	9.10E-10
6h	ACA1_027780/clean/scf7180000084471:14664-14740:+	ACA1_027780	RasGEF domain containing protein [Source:UniProtKB/TrEMBL;Acc:L8HMK7]	0.0035461	0.296257333	0.292711233	4.68E-07
6h	ACA1_172250/clean/scf7180000084741:286322-286431:-	ACA1_172250	Leucine rich repeat domain containing protein [Source:UniProtKB/TrEMBL;Acc:L8HHK1]	0.276910033	0.569559333	0.2926493	0.023638945
6h	ACA1_156480/clean/scf7180000084723:43942-44042:+	ACA1_156480	PHB domain-containing protein [Source:UniProtKB/TrEMBL;Acc:L8GK25]	0.000323584	0.292963	0.292639416	1.34E-12
6h	ACA1_366940/clean/scf7180000084641:520374-520490:-	ACA1_366940	BTB/POZ domain containing protein [Source:UniProtKB/TrEMBL;Acc:L8GPM1]	0.142190667	0.434762333	0.292571667	0.016008547
6h	ACA1_071210/clean/scf7180000084603:889848-889923:-	ACA1_071210	AAC-rich mRNA clone AAC4 protein [Source:UniProtKB/TrEMBL;Acc:L8HE90]	0.031005197	0.323575667	0.29257047	1.11E-10
6h	ACA1_140220/clean/scf7180000084707:45898-46032:-	ACA1_140220	hypothetical protein	0.009874403	0.302275333	0.29240093	2.04E-07
6h	ACA1_377320/clean/scf7180000084711:27201-27316:+	ACA1_377320	SPRY domain containing protein [Source:UniProtKB/TrEMBL;Acc:L8GSS3]	0.017895307	0.310235	0.292339693	3.06E-09
6h	ACA1_071180/clean/scf7180000084603:884784-884890:-	ACA1_071180	hypothetical protein	0.0193664	0.311531	0.2921646	6.84E-07
6h	ACA1_251640/clean/scf7180000084768:99134-99194:+	ACA1_251640	CBS domain-containing protein [Source:UniProtKB/TrEMBL;Acc:L8HAQ8]	0	0.292136	0.292136	3.89E-07
6h	ACA1_170940/clean/scf7180000084741:19010-19079:+	ACA1_170940	Proteasome subunit alpha type [Source:UniProtKB/TrEMBL;Acc:L8HH61]	0.001185727	0.293271667	0.292085939	9.50E-20
6h	ACA1_277790/clean/scf7180000084776:101990-102107:-	ACA1_277790	Protease, serine, 16 (Thymus), putative [Source:UniProtKB/TrEMBL;Acc:L8H5Q8]	0.001465983	0.293521333	0.29205535	7.87E-09
6h	ACA1_072980/clean/scf7180000084603:1349916-1350024:-	ACA1_072980	Proteasome subunit alpha type [Source:UniProtKB/TrEMBL;Acc:L8HDS9]	0.0011105	0.293162	0.2920515	1.54E-12
6h	ACA1_073230/clean/scf7180000084603:1375556-1375653:-	ACA1_073230	EHN domain-containing protein [Source:UniProtKB/TrEMBL;Acc:L8HDT9]	0.000566708	0.292592	0.292025292	3.35E-08
6h	ACA1_369270/clean/scf7180000084664:286334-286395:+	ACA1_369270	Rasrelated protein Rab-18-like, putative [Source:UniProtKB/TrEMBL;Acc:L8GYY7]	0.00275482	0.294765333	0.292010513	7.14E-08
6h	ACA1_164660/clean/scf7180000084729:244788-244867:-	ACA1_164660	Protein tyrosine phosphatase, dual specificity, putative [Source:UniProtKB/TrEMBL;Acc:L8GU25]	0.006984967	0.298916667	0.2919317	3.32E-14
6h	ACA1_164150/clean/scf7180000084729:173921-173992:-	ACA1_164150	Alpha/beta hydrolase fold domain containing protein [Source:UniProtKB/TrEMBL;Acc:L8GTY4]	0.025252533	0.317091	0.291838467	0.001613155
6h	ACA1_218440/clean/scf7180000084758:659643-659765:+	ACA1_218440	RNA_pol_L_2 domain-containing protein [Source:UniProtKB/TrEMBL;Acc:L8GQD6]	0.0477056	0.339523333	0.291817733	1.25E-06
6h	ACA1_278170/clean/scf7180000084776:185080-185225:-	ACA1_278170	RFX-type winged-helix domain-containing protein [Source:UniProtKB/TrEMBL;Acc:L8H8T8]	0	0.29181	0.29181	5.26E-06
6h	ACA1_144070/clean/scf7180000084709:142267-142358:-	ACA1_144070	Methyltrans_SAM domain-containing protein [Source:UniProtKB/TrEMBL;Acc:L8HHX9]	0.019354933	0.311115667	0.291760733	1.39E-06
6h	ACA1_192990/clean/scf7180000084749:176784-176908:-	ACA1_192990	PH domain-containing protein [Source:UniProtKB/TrEMBL;Acc:L8GQU9]	0.060011467	0.351743667	0.2917322	0.000122866
6h	ACA1_379300/clean/scf7180000084711:326415-326507:-	ACA1_379300	hypothetical protein	0.010169153	0.301846667	0.291677513	1.52E-06
6h	ACA1_057940/clean/scf7180000084599:212853-212941:+	ACA1_057940	Alpha-mannosidase [Source:UniProtKB/TrEMBL;Acc:L8GX61]	0.000965636	0.292554667	0.29158903	7.88E-11
6h	ACA1_389130/clean/scf7180000084733:207141-207241:+	ACA1_389130	BTB/POZ domain containing protein [Source:UniProtKB/TrEMBL;Acc:L8GDV1]	0.000290234	0.291741333	0.2914511	3.37E-10
6h	ACA1_333170/clean/scf7180000084581:9495-9594:-	ACA1_333170	Ras-GEF domain-containing protein [Source:UniProtKB/TrEMBL;Acc:L8HGS6]	0.121047467	0.412485333	0.291437867	0.005611955
6h	ACA1_146530/clean/scf7180000084713:44532-44618:+	ACA1_146530	Mak10 subunit, NatC N(Alpha)terminal acetyltransferase [Source:UniProtKB/TrEMBL;Acc:L8GTL7]	0	0.291368	0.291368	6.00E-08
6h	ACA1_057520/clean/scf7180000084599:157412-157494:+	ACA1_057520	NTP_transf_2 domain-containing protein [Source:UniProtKB/TrEMBL;Acc:L8GYG1]	0	0.291299	0.291299	0.000288563
6h	ACA1_068620/clean/scf7180000084603:239817-239920:-	ACA1_068620	hypothetical protein	0.005163573	0.296380667	0.291217093	1.08E-05
6h	ACA1_203500/clean/scf7180000084754:168662-168763:-	ACA1_203500	Coatomer subunit delta [Source:UniProtKB/TrEMBL;Acc:L8GW47]	0.000804587	0.292011	0.291206413	3.03E-09
6h	ACA1_121830/clean/scf7180000084682:105394-105524:-	ACA1_121830	COesterase domain-containing protein [Source:UniProtKB/TrEMBL;Acc:L8GEL0]	0.000137118	0.291303	0.291165882	3.15E-08
6h	ACA1_236090/clean/scf7180000084762:402247-402314:-	ACA1_236090	PUL domain-containing protein [Source:UniProtKB/TrEMBL;Acc:L8H3Q0]	0.139441	0.430566333	0.291125333	0.000393361
6h	ACA1_023850/clean/scf7180000084436:21467-21548:-	ACA1_023850	Protein kinase domain-containing protein [Source:UniProtKB/TrEMBL;Acc:L8GTK1]	0.031473767	0.322559667	0.2910859	7.33E-06
6h	ACA1_113870/clean/scf7180000084669:393189-393268:+	ACA1_113870	CUB domain containing protein [Source:UniProtKB/TrEMBL;Acc:L8H6F7]	0.010247937	0.301319333	0.291071397	1.12E-08
6h	ACA1_366940/clean/scf7180000084641:520029-520114:-	ACA1_366940	BTB/POZ domain containing protein [Source:UniProtKB/TrEMBL;Acc:L8GPM1]	0.03	0.320942667	0.290942667	0.000208459
6h	ACA1_202400/clean/scf7180000084754:37803-37926:+	ACA1_202400	K+ channel protein [Source:UniProtKB/TrEMBL;Acc:M0QSM5]	0.005138073	0.296041667	0.290903593	1.37E-06
6h	ACA1_019190/clean/scf7180000083937:12876-12966:+	ACA1_019190	Abhydrolase_3 domain-containing protein [Source:UniProtKB/TrEMBL;Acc:L8HI58]	0.004473547	0.295327667	0.29085412	1.04E-09
6h	ACA1_250180/clean/scf7180000084767:35782-35885:-	ACA1_250180	RhoGEF domain containing protein [Source:UniProtKB/TrEMBL;Acc:L8HC96]	0	0.290818667	0.290818667	4.46E-08
6h	ACA1_098010/clean/scf7180000084645:1376697-1376809:+	ACA1_098010	Lactamase_B domain-containing protein [Source:UniProtKB/TrEMBL;Acc:L8GJX3]	0.041666667	0.332385667	0.290719	0.005880908
6h	ACA1_272600/clean/scf7180000084772:73163-73252:-	ACA1_272600	SAM domain (Sterile alpha motif) domain containing protein [Source:UniProtKB/TrEMBL;Acc:L8HG53]	0.001413377	0.292066	0.290652623	5.86E-08
6h	ACA1_257950/clean/scf7180000084769:186516-186604:-	ACA1_257950	hypothetical protein	0.008217067	0.298861333	0.290644267	3.24E-08
6h	ACA1_237850/clean/scf7180000084763:31919-32056:+	ACA1_237850	BTB/POZ domain containing protein [Source:UniProtKB/TrEMBL;Acc:L8GJT8]	0.0277375	0.318348333	0.290610833	2.68E-11
6h	ACA1_389320/clean/scf7180000084733:229801-229875:+	ACA1_389320	BTB domain-containing protein [Source:UniProtKB/TrEMBL;Acc:M0QSM2]	0.0087963	0.299355333	0.290559033	1.12E-06
6h	ACA1_114860/clean/scf7180000084669:567689-567813:-	ACA1_114860	ANK_REP_REGION domain-containing protein [Source:UniProtKB/TrEMBL;Acc:L8H6N8]	0.0085354	0.298952	0.2904166	2.88E-08
6h	ACA1_372600/clean/scf7180000084679:24075-24169:-	ACA1_372600	F-box domain-containing protein [Source:UniProtKB/TrEMBL;Acc:L8GJA4]	0	0.290388	0.290388	1.21E-07
6h	ACA1_339740/clean/scf7180000084619:15983-16085:+	ACA1_339740	Serine/threonine protein kinase [Source:UniProtKB/TrEMBL;Acc:L8GLZ6]	0.002521007	0.292813333	0.290292327	6.04E-08
6h	ACA1_395480/clean/scf7180000084756:189615-189726:-	ACA1_395480	Copine subfamily protein [Source:UniProtKB/TrEMBL;Acc:L8H0H7]	0.030238167	0.320404333	0.290166167	2.65E-06
6h	ACA1_260230/clean/scf7180000084769:460625-460846:+	ACA1_260230	Peptidylprolyl isomerase [Source:UniProtKB/TrEMBL;Acc:L8GFP0]	0.004818788	0.294964	0.290145212	2.10E-10
6h	ACA1_349640/clean/scf7180000084690:22395-22516:+	ACA1_349640	HEAT repeat domain containing protein [Source:UniProtKB/TrEMBL;Acc:L8GKB3]	0.00075959	0.290904667	0.290145077	8.80E-09
6h	ACA1_219420/clean/scf7180000084758:732284-732384:+	ACA1_219420	Fbox domain containing protein [Source:UniProtKB/TrEMBL;Acc:L8GQP8]	0.0595424	0.349656667	0.290114267	0.000109203
6h	ACA1_089980/clean/scf7180000084639:409728-409800:-	ACA1_089980	hypothetical protein	0	0.290061	0.290061	9.09E-06
6h	ACA1_248620/clean/scf7180000084766:97747-97823:+	ACA1_248620	DEAD/DEAH box helicase domain containing protein [Source:UniProtKB/TrEMBL;Acc:L8GY19]	0.004881563	0.294893333	0.29001177	1.18E-06
6h	ACA1_149410/clean/scf7180000084715:145848-145989:+	ACA1_149410	Lectin_legB domain-containing protein [Source:UniProtKB/TrEMBL;Acc:L8HDD6]	0.00151194	0.291432667	0.289920727	7.12E-08
6h	ACA1_385390/clean/scf7180000084730:163120-163287:+	ACA1_385390	Ubiquitinconjugating enzyme subfamily protein [Source:UniProtKB/TrEMBL;Acc:L8HAL6]	0.088975433	0.378862	0.289886567	0.004085077
6h	ACA1_000820/clean/scf7180000082828:11325-11405:+	ACA1_000820	RING-type domain-containing protein [Source:UniProtKB/TrEMBL;Acc:L8H7M3]	0.00330844	0.293058333	0.289749893	1.27E-06
6h	ACA1_361100/clean/scf7180000084622:241585-241688:-	ACA1_361100	TLDc domain-containing protein [Source:UniProtKB/TrEMBL;Acc:L8HE93]	0.00196335	0.291689333	0.289725983	2.12E-07
6h	ACA1_068700/clean/scf7180000084603:257668-257768:+	ACA1_068700	Purple acid phosphatase [Source:UniProtKB/TrEMBL;Acc:L8HFR9]	0.00157477	0.291255333	0.289680563	1.56E-10
6h	ACA1_183460/clean/scf7180000084745:418586-418677:-	ACA1_183460	BTB/POZ domain containing protein [Source:UniProtKB/TrEMBL;Acc:L8HAF2]	0.003117507	0.29271	0.289592493	5.73E-05
6h	ACA1_384040/clean/scf7180000084730:9909-9991:-	ACA1_384040	hypothetical protein	0.009919707	0.299495667	0.28957596	1.70E-08
6h	ACA1_091960/clean/scf7180000084645:237349-237463:-	ACA1_091960	hypothetical protein	0.00598451	0.295488667	0.289504157	4.67E-06
6h	ACA1_230530/clean/scf7180000084761:373502-373586:+	ACA1_230530	S(Hydroxymethyl)glutathione dehydrogenase/class III alcohol dehydrogenase [Source:UniProtKB/TrEMBL;Acc:L8H8X2]	0.005754683	0.295239	0.289484317	3.00E-23
6h	ACA1_140600/clean/scf7180000084707:95129-95256:-	ACA1_140600	Transmembrane protein 34 family protein [Source:UniProtKB/TrEMBL;Acc:L8GH57]	0.018966567	0.308417	0.289450433	6.47E-10
6h	ACA1_096520/clean/scf7180000084645:1033555-1033632:-	ACA1_096520	Cytochrome p450 superfamily protein [Source:UniProtKB/TrEMBL;Acc:L8GLA2]	0	0.289404333	0.289404333	0.019708635
6h	ACA1_173670/clean/scf7180000084741:511391-511490:+	ACA1_173670	DEP domain-containing protein [Source:UniProtKB/TrEMBL;Acc:L8HGP4]	0.333333333	0.622650333	0.289317	0.000432009
6h	ACA1_068460/clean/scf7180000084603:206309-206423:-	ACA1_068460	Leucinerich repeat kinase [Source:UniProtKB/TrEMBL;Acc:L8HDF4]	2.69E-05	0.289324	0.289297068	3.54E-09
6h	ACA1_089970/clean/scf7180000084639:407210-407286:-	ACA1_089970	Macro domain-containing protein [Source:UniProtKB/TrEMBL;Acc:L8GU43]	0.000446913	0.289672667	0.289225753	1.43E-08
6h	ACA1_068460/clean/scf7180000084603:205883-205968:-	ACA1_068460	Leucinerich repeat kinase [Source:UniProtKB/TrEMBL;Acc:L8HDF4]	0.00684957	0.296057667	0.289208097	4.06E-08
6h	ACA1_077300/clean/scf7180000084612:285504-285697:+	ACA1_077300	Serine/threonine kinase [Source:UniProtKB/TrEMBL;Acc:L8GP03]	0.036458333	0.325653667	0.289195333	0.003493346
6h	ACA1_251640/clean/scf7180000084768:101379-101457:+	ACA1_251640	CBS domain-containing protein [Source:UniProtKB/TrEMBL;Acc:L8HAQ8]	0.008187667	0.297229333	0.289041667	5.42E-07
6h	ACA1_260180/clean/scf7180000084769:453080-453182:+	ACA1_260180	Nitric oxide synthase-interacting protein homolog [Source:UniProtKB/TrEMBL;Acc:L8GFN7]	0.018192067	0.307231	0.289038933	3.29E-08
6h	ACA1_068110/clean/scf7180000084603:110672-110731:+	ACA1_068110	Peptidylprolyl isomerase [Source:UniProtKB/TrEMBL;Acc:L8HDB8]	0.01012245	0.299157667	0.289035217	6.37E-17
6h	ACA1_371760/clean/scf7180000084664:601229-601303:-	ACA1_371760	PH domain-containing protein [Source:UniProtKB/TrEMBL;Acc:L8H1R1]	0.005747133	0.294665333	0.2889182	7.81E-06
6h	ACA1_285200/clean/scf7180000084776:957273-957359:-	ACA1_285200	Na_H_Exchanger domain-containing protein [Source:UniProtKB/TrEMBL;Acc:L8H9U2]	0.019676133	0.308591333	0.2889152	3.61E-12
6h	ACA1_014870/clean/scf7180000083772:13193-13276:+	ACA1_014870	tRNA phosphotransferase 1 isoform 1, putative [Source:UniProtKB/TrEMBL;Acc:L8HI60]	0.000418393	0.289303667	0.288885273	1.26E-09
6h	ACA1_145850/clean/scf7180000084710:168824-168900:-	ACA1_145850	O-methyltransferase, putative [Source:UniProtKB/TrEMBL;Acc:L8GFG9]	0	0.288848	0.288848	2.50E-08
6h	ACA1_113710/clean/scf7180000084669:359070-359209:+	ACA1_113710	SH3 domain-containing protein [Source:UniProtKB/TrEMBL;Acc:L8H4B9]	0.00224525	0.291082667	0.288837416	1.57E-09
6h	ACA1_028690/clean/scf7180000084473:11198-11259:-	ACA1_028690	Lipase [Source:UniProtKB/TrEMBL;Acc:L8GUX6]	0.005636447	0.294313333	0.288676886	3.04E-09
6h	ACA1_006060/clean/scf7180000083029:25532-25679:+	ACA1_006060	hypothetical protein	0.020596167	0.309152333	0.288556167	9.59E-07
6h	ACA1_366310/clean/scf7180000084641:389531-389642:+	ACA1_366310	Reticulon-like protein [Source:UniProtKB/TrEMBL;Acc:L8GMG7]	0.012335357	0.300887667	0.28855231	5.68E-18
6h	ACA1_266210/clean/scf7180000084770:631215-631287:-	ACA1_266210	hypothetical protein	0.01133839	0.299847	0.28850861	2.44E-10
6h	ACA1_171630/clean/scf7180000084741:129176-129257:-	ACA1_171630	TFIIE beta domain-containing protein [Source:UniProtKB/TrEMBL;Acc:L8HGD3]	0	0.288478	0.288478	6.04E-09
6h	ACA1_247070/clean/scf7180000084763:614953-615050:+	ACA1_247070	hypothetical protein	0.024119933	0.312577	0.288457067	6.47E-08
6h	ACA1_164680/clean/scf7180000084729:247960-248059:-	ACA1_164680	ANK_REP_REGION domain-containing protein [Source:UniProtKB/TrEMBL;Acc:L8GSN8]	0.0049422	0.293352667	0.288410467	5.42E-07
6h	ACA1_237790/clean/scf7180000084763:18597-18760:+	ACA1_237790	Protein kinase domain containing protein [Source:UniProtKB/TrEMBL;Acc:L8GM38]	0.003207177	0.291597667	0.28839049	1.69E-07
6h	ACA1_122370/clean/scf7180000084682:134875-134966:-	ACA1_122370	Short chain oxidoreductase [Source:UniProtKB/TrEMBL;Acc:L8GEE8]	0.009885193	0.298271333	0.28838614	1.14E-05
6h	ACA1_091140/clean/scf7180000084645:66200-66314:-	ACA1_091140	BTB/POZ domain containing protein [Source:UniProtKB/TrEMBL;Acc:L8GIG3]	0.000516157	0.288849667	0.28833351	1.40E-07
6h	ACA1_156490/clean/scf7180000084723:50535-50619:+	ACA1_156490	RasGEF domain containing protein [Source:UniProtKB/TrEMBL;Acc:L8GHT4]	0.005517917	0.293804333	0.288286417	9.87E-08
6h	ACA1_011280/clean/scf7180000083623:8404-8499:+	ACA1_011280	AAA domain-containing protein [Source:UniProtKB/TrEMBL;Acc:L8GP61]	0.009787597	0.298071333	0.288283737	4.62E-07
6h	ACA1_038350/clean/scf7180000084568:132294-132363:-	ACA1_038350	Fbox domain containing protein [Source:UniProtKB/TrEMBL;Acc:L8GKR6]	0.003197443	0.291438	0.288240557	3.60E-05
6h	ACA1_046130/clean/scf7180000084577:58140-58233:+	ACA1_046130	Cytochrome p450 superfamily protein [Source:UniProtKB/TrEMBL;Acc:L8H8R3]	0.00579972	0.294037	0.28823728	3.00E-06
6h	ACA1_133590/clean/scf7180000084701:90285-90358:-	ACA1_133590	hypothetical protein	0.043478333	0.331668333	0.28819	0.000481369
6h	ACA1_091040/clean/scf7180000084645:46974-47036:-	ACA1_091040	Amidohydro-rel domain-containing protein [Source:UniProtKB/TrEMBL;Acc:L8GIF1]	0	0.288124333	0.288124333	2.25E-08
6h	ACA1_035640/clean/scf7180000084564:103520-103627:-	ACA1_035640	Beta-lactamase domain-containing protein [Source:UniProtKB/TrEMBL;Acc:L8H9P2]	0	0.288122667	0.288122667	0.018047671
6h	ACA1_389490/clean/scf7180000084733:267751-267852:-	ACA1_389490	RRM domain-containing protein [Source:UniProtKB/TrEMBL;Acc:L8GES5]	0.000147438	0.288242667	0.288095228	1.64E-09
6h	ACA1_097590/clean/scf7180000084645:1290908-1290980:-	ACA1_097590	Amine oxidase [Source:UniProtKB/TrEMBL;Acc:L8GK33]	0.00559401	0.293635667	0.288041657	6.09E-30
6h	ACA1_192610/clean/scf7180000084749:139452-139594:+	ACA1_192610	Hypothetical protein	0.097357267	0.385312	0.287954733	0.000248588
6h	ACA1_141800/clean/scf7180000084708:24818-24926:-	ACA1_141800	hypothetical protein	0.01245671	0.300394	0.28793729	7.71E-11
6h	ACA1_035470/clean/scf7180000084564:59220-59289:+	ACA1_035470	Cation exchanger, putative [Source:UniProtKB/TrEMBL;Acc:L8HAF4]	0.002123143	0.290058333	0.28793519	1.28E-08
6h	ACA1_377770/clean/scf7180000084711:116415-116530:+	ACA1_377770	WDrepeat protein pop3, putative [Source:UniProtKB/TrEMBL;Acc:L8GRX7]	0.005166593	0.293096667	0.287930073	2.00E-09

6h	ACA1_087870/clean/scf7180000084639:120678-120758:-	ACA1_087870	Peptidase S53 domain-containing protein [Source:UniProtKB/TrEMBL;Acc:L8GUK2]	0.000914227	0.288780333	0.287866106	1.48E-10
6h	ACA1_274410/clean/scf7180000084773:103464-103584:+	ACA1_274410	Trafficking protein particle complex subunit [Source:UniProtKB/TrEMBL;Acc:L8GFV3]	0.01840498	0.306248	0.28784302	1.28E-10
6h	ACA1_002750/clean/scf7180000082937:5307-5393:-	ACA1_002750	F-box domain-containing protein [Source:UniProtKB/TrEMBL;Acc:L8GHD2]	0.007324827	0.295124	0.287799173	5.17E-06
6h	ACA1_367420/clean/scf7180000084641:629907-629989:-	ACA1_367420	Bifunctional protein FolC subfamily protein [Source:UniProtKB/TrEMBL;Acc:L8GN01]	0	0.287780667	0.287780667	1.22E-06
6h	ACA1_309500/clean/scf7180000083762:8353-8429:+	ACA1_309500	Pyridine nucleotidedisulfide oxidoreductase domain containing protein [Source:UniProtKB/TrEMBL;Acc:L8GI23]	0	0.287730333	0.287730333	2.56E-11
6h	ACA1_121840/clean/scf7180000084682:110630-110689:+	ACA1_121840	Protein kinase domain-containing protein [Source:UniProtKB/TrEMBL;Acc:L8GFF3]	0.266934333	0.554604333	0.28767	7.90E-05
6h	ACA1_052720/clean/scf7180000084594:161730-161823:+	ACA1_052720	hypothetical protein	0.018087643	0.305750667	0.287663023	1.16E-06
6h	ACA1_186210/clean/scf7180000084746:172909-173013:+	ACA1_186210	Tyrosine phosphataselike protein, putative [Source:UniProtKB/TrEMBL;Acc:L8H5A9]	0.002331913	0.289992667	0.287660753	9.00E-07
6h	ACA1_031780/clean/scf7180000084523:1694-1844:-	ACA1_031780	Lactamase_B domain-containing protein [Source:UniProtKB/TrEMBL;Acc:L8H103]	0	0.287633667	0.287633667	3.74E-08
6h	ACA1_220750/clean/scf7180000084758:961082-961228:-	ACA1_220750	Urease accessory protein UreF, putative [Source:UniProtKB/TrEMBL;Acc:L8GQ53]	0.00200787	0.2896351	0.28762723	1.30E-05
6h	ACA1_074110/clean/scf7180000084605:30152-30317:-	ACA1_074110	Peptidase [Source:UniProtKB/TrEMBL;Acc:L8HJ70]	0.00859822	0.296225333	0.287627113	7.08E-08
6h	ACA1_106000/clean/scf7180000084660:47459-47592:+	ACA1_106000	HEAT repeat domain containing protein [Source:UniProtKB/TrEMBL;Acc:L8GN71]	0	0.287562667	0.287562667	2.15E-08
6h	ACA1_024220/clean/scf7180000084441:19818-19927:+	ACA1_024220	Purinergic receptor, putative [Source:UniProtKB/TrEMBL;Acc:L8GLB7]	0	0.287517667	0.287517667	2.37E-13
6h	ACA1_175570/clean/scf7180000084741:998587-998681:-	ACA1_175570	DH domain-containing protein [Source:UniProtKB/TrEMBL;Acc:L8HI39]	0.00555839	0.292963333	0.287404943	1.15E-06
6h	ACA1_252180/clean/scf7180000084768:138852-138971:+	ACA1_252180	Leucine rich repeat domain containing protein [Source:UniProtKB/TrEMBL;Acc:L8H9U3]	0.017431067	0.304803333	0.287372267	9.13E-06
6h	ACA1_068620/clean/scf7180000084603:240313-240407:-	ACA1_068620	hypothetical protein	0	0.287371	0.287371	2.09E-07
6h	ACA1_062680/clean/scf7180000084599:1272561-1272678:+	ACA1_062680	hypothetical protein	0.028579467	0.315949333	0.287369867	3.96E-09
6h	ACA1_276370/clean/scf7180000084774:158338-158518:-	ACA1_276370	unspecified product	0.014572857	0.301933	0.287360143	1.14E-12
6h	ACA1_170640/clean/scf7180000084739:98356-98416:+	ACA1_170640	Protein kinase domain-containing protein [Source:UniProtKB/TrEMBL;Acc:L8HIR2]	0.074098733	0.361455	0.287356267	0.000394821
6h	ACA1_331960/clean/scf7180000084578:52652-52746:-	ACA1_331960	Zinc finger, c2h2 type domain containing protein [Source:UniProtKB/TrEMBL;Acc:L8HD24]	0	0.287339333	0.287339333	0.000122764
6h	ACA1_121560/clean/scf7180000084682:59474-59554:+	ACA1_121560	Aminopeptidase N, putative [Source:UniProtKB/TrEMBL;Acc:L8GFD6]	0.000652404	0.287964667	0.287312262	3.23E-10
6h	ACA1_216320/clean/scf7180000084758:468030-468136:+	ACA1_216320	Homogentisate 1,2dioxygenase [Source:UniProtKB/TrEMBL;Acc:L8GQ83]	0.002359727	0.289636333	0.287276607	6.19E-10
6h	ACA1_269260/clean/scf7180000084770:960749-960855:-	ACA1_269260	Ras family protein [Source:UniProtKB/TrEMBL;Acc:L8H2Q9]	0.010517147	0.297733333	0.287216187	2.32E-06
6h	ACA1_288840/clean/scf7180000084777:307197-307277:-	ACA1_288840	Saposin family type B, region 1 protein [Source:UniProtKB/TrEMBL;Acc:L8HKH6]	0.014804467	0.302018	0.287213533	1.23E-11
6h	ACA1_074110/clean/scf7180000084605:26757-26862:-	ACA1_074110	Peptidase [Source:UniProtKB/TrEMBL;Acc:L8HJ70]	0.004112377	0.291301333	0.287188957	1.23E-08
6h	ACA1_071610/clean/scf7180000084603:1004023-1004096:+	ACA1_071610	Short-chain dehydrogenase [Source:UniProtKB/TrEMBL;Acc:L8HDD9]	0.004496003	0.291652333	0.28715633	8.58E-15
6h	ACA1_012300/clean/scf7180000083740:10372-10445:-	ACA1_012300	hypothetical protein	0.007246367	0.294374333	0.287127967	7.54E-05
6h	ACA1_291470/clean/scf7180000084777:753793-753891:-	ACA1_291470	20S proteasome subunit beta 3, putative [Source:UniProtKB/TrEMBL;Acc:L8HIC0]	0.008226283	0.295309	0.287082717	7.38E-23
6h	ACA1_130480/clean/scf7180000084698:86177-86269:-	ACA1_130480	RhoGEF domain containing protein [Source:UniProtKB/TrEMBL;Acc:L8GQC0]	0.002901353	0.289958	0.287056647	5.85E-07
6h	ACA1_093740/clean/scf7180000084645:628418-628563:-	ACA1_093740	Serine/threonine protein kinase [Source:UniProtKB/TrEMBL;Acc:L8GJ93]	0.00224631	0.289199	0.28695269	3.61E-08
6h	ACA1_315810/clean/scf7180000083943:13287-13363:-	ACA1_315810	hypothetical protein	0	0.286932	0.286932	2.59E-09
6h	ACA1_065270/clean/scf7180000084599:1840905-1840998:+	ACA1_065270	Ubiquitin ligase, putative [Source:UniProtKB/TrEMBL;Acc:L8GYX5]	0.1135901	0.400368333	0.286778233	0.003164373
6h	ACA1_325440/clean/scf7180000084495:7936-8010:+	ACA1_325440	hypothetical protein	0.0293952	0.316094667	0.286699467	3.13E-08
6h	ACA1_374010/clean/scf7180000084679:203649-203727:-	ACA1_374010	Peptidase M15D, vanX Dala-D-ala dipeptidase [Source:UniProtKB/TrEMBL;Acc:L8GHK1]	0	0.286549667	0.286549667	1.67E-05
6h	ACA1_191490/clean/scf7180000084749:3123-3197:-	ACA1_191490	RTR1-type domain-containing protein [Source:UniProtKB/TrEMBL;Acc:L8GQP2]	0.00025048	0.286757	0.28650652	2.24E-10
6h	ACA1_054740/clean/scf7180000084594:563568-563683:+	ACA1_054740	hypothetical protein	0.0627639	0.349153667	0.286389767	3.64E-06
6h	ACA1_322090/clean/scf7180000084459:3302-3408:-	ACA1_322090	Myosin head (Motor domain) domain containing protein [Source:UniProtKB/TrEMBL;Acc:L8GN06]	0.01358697	0.299968333	0.286381363	1.17E-09
6h	ACA1_271600/clean/scf7180000084771:81521-81609:-	ACA1_271600	Cation diffusion facilitator family transporter superfamily protein [Source:UniProtKB/TrEMBL;Acc:L8GNJ1]	0.0279918	0.314352667	0.286360867	2.03E-05
6h	ACA1_116230/clean/scf7180000084669:730680-730783:+	ACA1_116230	Protein kinase domain containing protein [Source:UniProtKB/TrEMBL;Acc:L8H4M9]	0.038961033	0.325292667	0.286331633	0.000296878
6h	ACA1_101340/clean/scf7180000084649:49093-49203:-	ACA1_101340	Leucine rich repeat domain containing protein [Source:UniProtKB/TrEMBL;Acc:L8GH62]	0.01014376	0.296459667	0.286315907	5.14E-06
6h	ACA1_063800/clean/scf7180000084599:1505946-1506036:-	ACA1_063800	B30.2/SPRY domain-containing protein [Source:UniProtKB/TrEMBL;Acc:L8GZV1]	0.046177367	0.332407667	0.2862303	7.09E-06
6h	ACA1_164760/clean/scf7180000084729:269004-269067:+	ACA1_164760	hypothetical protein	0.064779367	0.350994667	0.2862153	1.29E-06
6h	ACA1_074740/clean/scf7180000084608:75041-75159:-	ACA1_074740	Thioredoxin domain-containing protein [Source:UniProtKB/TrEMBL;Acc:L8HF80]	0.005328079	0.291464467	0.286136388	4.11E-09
6h	ACA1_060260/clean/scf7180000084599:630262-630400:-	ACA1_060260	RFX-type winged-helix domain-containing protein [Source:UniProtKB/TrEMBL;Acc:L8GVN2]	0.052914267	0.339024	0.286109733	8.56E-05
6h	ACA1_119410/clean/scf7180000084672:64174-64236:+	ACA1_119410	PCI domain-containing protein [Source:UniProtKB/TrEMBL;Acc:L8HLX4]	0.007717364	0.293811	0.286093636	8.07E-13
6h	ACA1_092040/clean/scf7180000084645:253501-253575:+	ACA1_092040	Pyr_redox_2 domain-containing protein [Source:UniProtKB/TrEMBL;Acc:L8GIM3]	0.004975133	0.291050667	0.286075533	1.04E-05
6h	ACA1_107640/clean/scf7180000084660:238691-238901:+	ACA1_107640	Signal transduction histidine kinase [Source:UniProtKB/TrEMBL;Acc:L8GNL7]	0.01405476	0.300103	0.28604824	2.38E-10
6h	ACA1_266210/clean/scf7180000084770:630932-631007:-	ACA1_266210	hypothetical protein	0.020140067	0.306147667	0.2860076	4.36E-08
6h	ACA1_183970/clean/scf7180000084745:532902-532979:-	ACA1_183970	hypothetical protein	0	0.285952	0.285952	0.000695339
6h	ACA1_220110/clean/scf7180000084758:864747-864824:-	ACA1_220110	hypothetical protein	0.003690033	0.289613667	0.285923633	1.46E-05
6h	ACA1_221070/clean/scf7180000084758:991801-991869:+	ACA1_221070	Carrier superfamily protein [Source:UniProtKB/TrEMBL;Acc:L8GT63]	0.000372773	0.286177333	0.28580456	1.03E-07
6h	ACA1_256610/clean/scf7180000084769:63847-63922:-	ACA1_256610	hypothetical protein	0.026729567	0.312508667	0.2857791	0.006160379
6h	ACA1_060250/clean/scf7180000084599:627094-627214:-	ACA1_060250	Alpha-carbonic anhydrase domain-containing protein [Source:UniProtKB/TrEMBL;Acc:L8GXK3]	0.00717746	0.292855	0.28567754	1.90E-15
6h	ACA1_339740/clean/scf7180000084619:17500-17590:+	ACA1_339740	Serine/threonine protein kinase [Source:UniProtKB/TrEMBL;Acc:L8GLZ6]	0.00052002	0.286188	0.28566798	2.89E-08
6h	ACA1_382540/clean/scf7180000084721:67630-67727:-	ACA1_382540	SET domain-containing protein [Source:UniProtKB/TrEMBL;Acc:L8GUV0]	0.00200441	0.287654	0.28564959	6.31E-07
6h	ACA1_244930/clean/scf7180000084763:459325-459399:+	ACA1_244930	hypothetical protein	0	0.285562667	0.285562667	6.36E-09
6h	ACA1_191880/clean/scf7180000084749:63946-64068:+	ACA1_191880	Calponin domain containing protein [Source:UniProtKB/TrEMBL;Acc:L8GP96]	0.007044273	0.292510333	0.28546606	1.64E-14
6h	ACA1_235680/clean/scf7180000084762:353996-354109:-	ACA1_235680	Rap-GAP domain-containing protein [Source:UniProtKB/TrEMBL;Acc:L8H1A5]	0.028700633	0.314079	0.285378367	6.86E-08
6h	ACA1_024250/clean/scf7180000084441:25613-25678:-	ACA1_024250	hypothetical protein	0.689015333	0.974338667	0.285323333	0.000510951
6h	ACA1_366920/clean/scf7180000084641:514796-514869:-	ACA1_366920	Protein kinase domain-containing protein [Source:UniProtKB/TrEMBL;Acc:L8GMM5]	0.038861487	0.324131	0.285269513	9.66E-06
6h	ACA1_164910/clean/scf7180000084729:308905-308976:-	ACA1_164910	hypothetical protein	0.043777433	0.328965	0.285187567	4.82E-11
6h	ACA1_147710/clean/scf7180000084714:54370-54476:+	ACA1_147710	Zn_dep_PLPC domain-containing protein [Source:UniProtKB/TrEMBL;Acc:L8GJR2]	0.029902233	0.315083	0.285180767	7.54E-07
6h	ACA1_062350/clean/scf7180000084599:1169339-1169434:+	ACA1_062350	ADF-H domain-containing protein [Source:UniProtKB/TrEMBL;Acc:L8GY68]	0.012010933	0.297148667	0.285137733	5.03E-13
6h	ACA1_133320/clean/scf7180000084701:31138-31230:+	ACA1_133320	Homeobox domain-containing protein [Source:UniProtKB/TrEMBL;Acc:L8H2R6]	0.00699123	0.292054	0.28506277	5.07E-07
6h	ACA1_364270/clean/scf7180000084641:69003-69079:+	ACA1_364270	DTW domain-containing protein [Source:UniProtKB/TrEMBL;Acc:L8GP32]	0.011351577	0.296403667	0.28505209	1.78E-06
6h	ACA1_068520/clean/scf7180000084603:221704-221788:+	ACA1_068520	hypothetical protein	0	0.285014	0.285014	6.75E-05
6h	ACA1_031950/clean/scf7180000084523:40636-40713:+	ACA1_031950	CBS domain containing protein [Source:UniProtKB/TrEMBL;Acc:L8H414]	0.281251333	0.566137333	0.284886	0.030504885
6h	ACA1_276830/clean/scf7180000084774:226921-227001:-	ACA1_276830	Peptidyl-prolyl cis-trans isomerase [Source:UniProtKB/TrEMBL;Acc:L8GR77]	0.000948334	0.285712667	0.284764332	9.23E-17
6h	ACA1_033540/clean/scf7180000084559:34754-34861:+	ACA1_033540	hypothetical protein	0.051743367	0.336453	0.284709633	8.83E-06
6h	ACA1_358630/clean/scf7180000084742:16414-16499:-	ACA1_358630	hypothetical protein	0	0.284683	0.284683	3.75E-07
6h	ACA1_261480/clean/scf7180000084769:690246-690359:+	ACA1_261480	ATP-dependent (S)-NAD(P)H-hydrate dehydratase [Source:UniProtKB/TrEMBL;Acc:L8GG71]	0.005316077	0.289956	0.284639923	1.81E-06
6h	ACA1_153240/clean/scf7180000084720:94064-94161:-	ACA1_153240	hypothetical protein	0	0.284572333	0.284572333	0.005947583
6h	ACA1_057810/clean/scf7180000084599:184127-184233:+	ACA1_057810	unspecified product	0.0202218	0.304789	0.2845672	0.002738398
6h	ACA1_123760/clean/scf7180000084686:56024-56116:+	ACA1_123760	Protein kinase domain-containing protein [Source:UniProtKB/TrEMBL;Acc:L8HF31]	0.00541507	0.289800333	0.284385263	2.42E-05
6h	ACA1_230520/clean/scf7180000084761:370842-370924:-	ACA1_230520	Hypothetical protein	0.001413883	0.28571	0.284296117	2.04E-10
6h	ACA1_062080/clean/scf7180000084599:1116765-1116848:-	ACA1_062080	DUF2263 domain-containing protein [Source:UniProtKB/TrEMBL;Acc:L8GZD2]	0.013436717	0.297725	0.284288283	3.31E-05
6h	ACA1_207340/clean/scf7180000084757:56213-56330:+	ACA1_207340	t-SNARE coiled-coil homology domain-containing protein [Source:UniProtKB/TrEMBL;Acc:L8GY87]	0.009999167	0.294280667	0.2842815	1.08E-06
6h	ACA1_393010/clean/scf7180000084756:26923-27009:+	ACA1_393010	AA_TRNA_LIGASE_II domain-containing protein [Source:UniProtKB/TrEMBL;Acc:L8H0A5]	0.012427777	0.296572667	0.28414489	1.61E-12
6h	ACA1_383070/clean/scf7180000084721:134923-134997:+	ACA1_383070	CBS domain containing protein [Source:UniProtKB/TrEMBL;Acc:L8GXI5]	0.000636537	0.284776667	0.28414013	4.30E-07
6h	ACA1_379260/clean/scf7180000084711:313424-313505:+	ACA1_379260	Coatomer subunit beta [Source:UniProtKB/TrEMBL;Acc:L8GRY6]	0.005479436	0.289522	0.284042564	1.19E-12
6h	ACA1_372070/clean/scf7180000084664:625094-625203:-	ACA1_372070	Hepatocellular carcinomaassociated antigen 59, putative [Source:UniProtKB/TrEMBL;Acc:L8GZA6]	0.007060653	0.291055333	0.28399468	7.07E-07
6h	ACA1_182120/clean/scf7180000084745:131412-131473:+	ACA1_182120	WD repeat domain 5B, putative [Source:UniProtKB/TrEMBL;Acc:L8HA46]	0	0.283968667	0.283968667	7.99E-09
6h	ACA1_380870/clean/scf7180000084712:103329-103487:+	ACA1_380870	Transporter, major facilitator subfamily protein [Source:UniProtKB/TrEMBL;Acc:L8GMV6]	0.0654027	0.349344	0.2839413	0.00057667
6h	ACA1_296720/clean/scf7180000084777:1409945-1410064:-	ACA1_296720	Hist_deacetyl domain-containing protein [Source:UniProtKB/TrEMBL;Acc:L8HK34]	0.001196637	0.285122	0.283925363	1.63E-08
6h	ACA1_355850/clean/scf7180000084728:32484-32571:-	ACA1_355850	Methionine aminopeptidase [Source:UniProtKB/TrEMBL;Acc:L8H6U0]	0.002849449	0.286748	0.283898551	6.34E-14
6h	ACA1_274880/clean/scf7180000084773:131972-132132:+	ACA1_274880	AB hydrolase-1 domain-containing protein [Source:UniProtKB/TrEMBL;Acc:L8GI88]	0.313653	0.597546	0.283893	0.002533432
6h	ACA1_290100/clean/scf7180000084777:524496-524576:+	ACA1_290100	Kri1_C domain-containing protein [Source:UniProtKB/TrEMBL;Acc:L8HLH0]	0.00932708	0.293140667	0.283813587	3.02E-07
6h	ACA1_298650/clean/scf7180000082992:10581-10681:+	ACA1_298650	hypothetical protein	0	0.283808667	0.283808667	1.43E-08
6h	ACA1_276370/clean/scf7180000084774:156557-156680:-	ACA1_276370	unspecified product	0.044936933	0.328708667	0.283771733	4.61E-10
6h	ACA1_366590/clean/scf7180000084641:459139-459217:-	ACA1_366590	Regulator of chromosome condensation (RCC1) repeat domain containing protein [Source:UniProtKB/TrEMBL;Acc:L8GM24]	0	0.283743333	0.283743333	0.000586653
6h	ACA1_072330/clean/scf7180000084603:1202654-1202787:-	ACA1_072330	UBA_e1_C domain-containing protein [Source:UniProtKB/TrEMBL;Acc:L8HGS4]	0	0.283717	0.283717	1.99E-10
6h	ACA1_377500/clean/scf7180000084711:76083-76178:+	ACA1_377500	Peptidase family c78 protein [Source:UniProtKB/TrEMBL;Acc:L8GU78]	0.00137576	0.285037	0.28366124	5.77E-08
6h	ACA1_058860/clean/scf7180000084599:397495-397620:-	ACA1_058860	Ribosomal_L18e/L15P domain-containing protein [Source:UniProtKB/TrEMBL;Acc:L8GW37]	0.00557594	0.289224667	0.283648727	2.07E-11
6h	ACA1_095690/clean/scf7180000084645:882871-882945:+	ACA1_095690	hypothetical protein	0	0.283607	0.283607	1.81E-06
6h	ACA1_035350/clean/scf7180000084564:29634-29748:-	ACA1_035350	Protein kinase domain-containing protein [Source:UniProtKB/TrEMBL;Acc:L8HAJ4]	0.021699433	0.305249333	0.2835499	2.18E-06
6h	ACA1_285540/clean/scf7180000084776:994717-994808:-	ACA1_285540	GPS domain-containing protein [Source:UniProtKB/TrEMBL;Acc:L8H7T4]	0.005208333	0.288664333	0.283456	8.14E-06
6h	ACA1_383690/clean/scf7180000084721:246125-246194:-	ACA1_383690	Methyltransf_11 domain-containing protein [Source:UniProtKB/TrEMBL;Acc:L8GXM4]	0	0.283361333	0.283361333	4.47E-07
6h	ACA1_117020/clean/scf7180000084669:893437-893505:+	ACA1_117020	Carboxypeptidase [Source:UniProtKB/TrEMBL;Acc:L8H3Z1]	0	0.283360333	0.283360333	1.61E-10
6h	ACA1_398190/clean/scf7180000084759:213373-213445:-	ACA1_398190	6-phosphogluconate dehydrogenase, decarboxylating [Source:UniProtKB/TrEMBL;Acc:L8HDP8]	0.045891867	0.329232667	0.2833408	0.001686523
6h	ACA1_035460/clean/scf7180000084564:55408-55555:+	ACA1_035460	Sugar (GlycosidePentoside-hexuronide) transporter, putative [Source:UniProtKB/TrEMBL;Acc:L8HCV9]	0.064228273	0.347517333	0.28328906	0.0005514
6h	ACA1_071550/clean/scf7180000084603:991035-991156:-	ACA1_071550	F-box domain-containing protein [Source:UniProtKB/TrEMBL;Acc:L8HFM3]	0	0.283273333	0.283273333	0.000125835
6h	ACA1_076200/clean/scf7180000084612:24507-24590:-	ACA1_076200	PITH domain-containing protein [Source:UniProtKB/TrEMBL;Acc:L8GL73]	0.03970593	0.322969	0.28326307	8.28E-06
6h	ACA1_000820/clean/scf7180000082828:10871-10965:+	ACA1_000820	RING-type domain-containing protein [Source:UniProtKB/TrEMBL;Acc:L8H7M3]	0.00961598	0.292792	0.28317602	1.99E-06
6h	ACA1_035370/clean/scf7180000084564:36175-36277:-	ACA1_035370	Protein kinase domain-containing protein [Source:UniProtKB/TrEMBL;Acc:L8HAE6]	8.23E-05	0.283210667	0.283128342	2.47E-07
6h	ACA1_366920/clean/scf7180000084641:514989-515083:-	ACA1_366920	Protein kinase domain-containing protein [Source:UniProtKB/TrEMBL;Acc:L8GMM5]	0	0.283031667	0.283031667	4.03E-07
6h	ACA1_253150/clean/scf7180000084768:252136-252208:+	ACA1_253150	RAB8D, putative [Source:UniProtKB/TrEMBL;Acc:L8H9Y3]	0.011771373	0.294778667	0.283007293	1.33E-07
6h	ACA1_055450/clean/scf7180000084594:708264-708348:+	ACA1_055450	SBF2 domain-containing protein [Source:UniProtKB/TrEMBL;Acc:L8H5J1]	0.0380544	0.320920667	0.282866267	3.59E-08
6h	ACA1_072330/clean/scf7180000084603:1201929-1201998:-	ACA1_072330	UBA_e1_C domain-containing protein [Source:UniProtKB/TrEMBL;Acc:L8HGS4]	0.00204499	0.284889333	0.282844343	6.97E-09
6h	ACA1_038350/clean/scf7180000084568:131015-131095:-	ACA1_038350	Fbox domain containing protein [Source:UniProtKB/TrEMBL;Acc:L8GKR6]	0.01004229	0.292835667	0.282793377	1.13E-05
6h	ACA1_247070/clean/scf7180000084763:615116-615189:+	ACA1_247070	hypothetical protein	0.045200646	0.327977667	0.28277702	0.006542008
6h	ACA1_068590/clean/scf7180000084603:234519-234592:-	ACA1_068590	SpoU_methylase domain-containing protein [Source:UniProtKB/TrEMBL;Acc:L8HDK4]	0	0.282757333	0.282757333	5.13E-05
6h	ACA1_230440/clean/scf7180000084761:348462-348569:+	ACA1_230440	WD_REPEATS_REGION domain-containing protein [Source:UniProtKB/TrEMBL;Acc:L8H839]	0	0.282717333	0.282717333	8.19E-06
6h	ACA1_035560/clean/scf7180000084564:88757-88837:-	ACA1_035560	Short chain dehydrogenase/reductase family oxidoreductase [Source:UniProtKB/TrEMBL;Acc:L8HCW5]	0.0082292	0.290906	0.2826768	1.42E-09
6h	ACA1_129940/clean/scf7180000084698:52867-52973:+	ACA1_129940	LisH domain-containing protein [Source:UniProtKB/TrEMBL;Acc:L8GNX2]	0.026065467	0.308736333	0.282670867	0.000172121
[truncated: 1,585,563 more chars]
